# Retrospective phenology in western Mediterranean plants: revealing climate change patterns through herbarium specimens

**DOI:** 10.1093/aobpla/plaf064

**Published:** 2025-11-03

**Authors:** Andros Solakis-Tena, Federico Casimiro-Soriguer Solanas, Noelia Hidalgo-Triana

**Affiliations:** Department of Botany and Plant Physiology (Botany Area), University of Malaga, Blvr. Louis Pasteur, 31, 29010 Málaga, Spain; Department of Botany and Plant Physiology (Botany Area), University of Malaga, Blvr. Louis Pasteur, 31, 29010 Málaga, Spain; Department of Botany and Plant Physiology (Botany Area), University of Malaga, Blvr. Louis Pasteur, 31, 29010 Málaga, Spain; Plants, Ecosystems & Climate

**Keywords:** day of year (DOY), flowering, fruiting, global warming, growth, preserved specimen, thermotypes

## Abstract

Herbarium specimens have proven useful for assessing phenological responses to climate change. Using preserved specimens, we analysed the changes in day of year (DOY) for four phenophases: three reproductive (preflowering, flowering, fruiting) and one vegetative (growth). We conducted phenological analysis across bioclimatic belts (thermotypes) from the Rivas–Martinez classification and across 77 taxa present in the Baetic Ranges of the southern Iberian Peninsula. Taxa were characteristic, common, or endemic species from Habitats of Community Interest (HCI) under the European Directive 92/43/EEC. Phenological shifts were assessed using two approaches: long-term trends in DOY with time and relationships with historical climate variables related to temperature and precipitation. At the thermotypes level, flowering advanced consistently over time and with increasing temperatures, showing homogeneous responses and suggesting a weakening of altitudinal differentiation. In contrast, growth exhibited thermotype-specific trends, with stronger advances at high elevations, while preflowering and fruiting showed little or no sensitivity to time or climate variables. At the species level, 31% of taxa showed phenological changes over time in the Baetic Ranges (−3.6 days/decade for reproductive and −5.6 days/decade for vegetative phenophases). However, 97% of taxa showed significant relationships with increasing temperatures and decreasing precipitation, particularly with mean annual temperature (−12.7 days for reproductive and −14.3 days for vegetative phenophases per increased °C). These phenological changes could hinder reproductive and vegetative success by causing mismatches with other ecosystem role-players. As the Mediterranean is expected to become warmer and drier, our findings indicate a potential threat to HCI in the southern Mediterranean.

## Teaser

Herbarium records reveal widespread earlier timing of Mediterranean plant life cycles under warming. Flowering shifted earlier by ~3.6 days per decade (~12.7 days per °C), and vegetative growth by ~5.6 days per decade (~14.3 days per °C). Responses were largely consistent across elevational thermotypes, suggesting emerging phenological homogenization. Such advances threaten the timing with pollinators and seed dispersers, and highlight the power of herbaria to track climate impacts.

## Introduction

Phenology is the study of the seasonal timing of life-history events (e.g. flowering or growing in plants; Orshan 1989), which occur at specific times each year (Willis et al. 2017) and depend on seasonal environmental conditions (Schwartz 1994, 2003). Phenology depends directly on climatic variables, especially temperature and precipitation (Sparks and Carey 1995, Peñuelas et al. 2002, Pau et al. 2011). Phases of reproductive and vegetative plant phenology, such as growing, flowering, or fruiting, are highly related to warmer months due to the temperatures of spring that induce plants to develop reproductive and vegetative structures by biochemical responses (chilling and forcing) after winter periods (Lieth 1974, Schwartz 1990, Luedeling et al. 2021). In some cases, growth (as leaf onset), flowering (as bud-break), or fruiting phenophases are also related to moisture and precipitation variables present in the environment, being driven by drought periods or rainy seasons throughout the year (De Oliveira et al. 2015, Mazer et al. 2015, Armstrong-Herniman and Greenwood 2021, Wang et al. 2022). Timing in reproductive phenology is important for plants because it determines the best period for an individual to invest efforts and resources in: (1) developing flowers, creating pollen, and making it available to other components of the ecosystem (e.g. insect pollinators) (Rathcke and Lacey 1985, Dunnell and Travers 2011) or (2) developing fruits, which contribute to the maintenance of the ecosystem; e.g. as a source of food or as an assurance to preserve the continuity of the species’ population (Rathcke and Lacey 1985, Mendes et al. 2023). Instead, vegetative phenology can be related to growing phases (e.g. leaf production), granting properties for the individual, such as increasing its lifespan, improving its capacity to take nourishment and light, or its ability to withstand predation (herbivory), but also contributing to other environmental interactions like carbon sequestration (Westman 1981, Estiarte and Peñuelas 2015, Morellato et al. 2016). Besides, in many cases, vegetative phenophases precede reproductive phenophases. For example, sometimes preflowering buds cannot develop unless there has been prior growth in the dolichoblasts (Navarro et al. 1993). Dolichoblast vegetative growth (DVG) functions to increase the size of the crown and explore favourable microenvironments adjacent to it, mainly growing in the spring and usually ending their growth with an inflorescence (Orshan 1989).

In the context of recent climate change, the global surface temperature has increased by 1.1°C, causing severe alterations in climatic conditions worldwide (IPCC 2023), especially in Spain (Brunet et al. 2006, Luna et al. 2012a, 2012b, Mestre et al. 2015). In response to this situation, plant phenology has received increased attention in the past few decades due to its direct relationship with climate (Tang et al. 2016). Earlier studies that linked climate change with phenology confirmed the fundamental role that it plays as a bioindicator of climate change (Beaubien and Johnson 1994, Schwartz 1994, Keeling et al. 1996, Parmesan and Yohe 2003, Menzel et al. 2006a). Studies conducted through various approaches have demonstrated that global warming is causing shifts in plant phenology (Parmesan and Yohe 2003, Cleland et al. 2007), from studies based on satellite observations (Myneni et al. 1997, Jin et al. 2019) to *in situ* field studies (Inouye 2008, Primack and Miller-Rushing 2009), or research conducted with herbarium specimens (Davis et al. 2015, Jones and Daehler 2018). Several studies have demonstrated that herbarium specimens are a good tool for tracking climate change by studying the phenological patterns over time (Primack et al. 2004, Panchen et al. 2012, Calinger et al. 2013, Willis et al. 2017). Consequently, the study of retrospective phenology has become an essential tool, both for its relationship with field-based phenology studies and for predicting how species are responding to climate change, tracking it across time (Love et al. 2019). Recent climate may have left their print on the phenological behaviour of some Mediterranean plants that have not yet been studied.

Among these, some of the most ecologically relevant are key species that define habitat types protected under European legislation (European Commission DG-ENV 2013). According to the European Directive 92/43/CEE, a Habitat of Community Interest (HCI) refers to a natural habitat type that is endangered, has a restricted natural range, or represents an outstanding example of a biogeographical region in the EU (Council Directive 92/43/EEC 1992). These habitats encompass characteristic vegetation species, which can be considered a combination of diagnostic species and species with higher constancy that together define vegetation units in each type of the natural habitats (Braun-Blanquet 1979, Chytrý et al. 2020). Phenological changes in these characteristic species could lead to changes in the habitat itself, an aspect that, despite its importance, has yet to be studied. Recent research using modelling and measurement of functional traits has indicated the vulnerability of the Mediterranean HCI (Del Río et al. 2018, Hidalgo-Triana et al. 2023a , 2023b) and the anthropogenic threats they face (Prisco et al. 2013, Hidalgo-Triana et al. 2023a, 2023b). However, their phenology remains understudied.

The use of herbarium specimens to track phenological responses to climate change is not a novel methodology (Primack et al. 2004, Miller-Rushing et al. 2006, Davis et al. 2015, Willis et al. 2017). Nevertheless, this approach has not yet been applied to Mediterranean key species from the European HCI, although some taxa studied in Rondinel-Mendoza et al. (2024) and Pareja-Bonilla et al. (2025) belonged to some regional HCI. Furthermore, phenological changes have not been assessed from the perspective of the different bioclimatic belts present in the region, nor have trends been compared across thermophilous, mid-mountain and high-mountain taxa. Although Rondinel-Mendoza et al. (2024) compared alpine and non-alpine taxa in the Sierra Nevada massif, species from lower belts remained unstudied, leaving their phenological behaviour unknown. These bioclimatic belts are known as thermotypes and refer to thermoclimatic ranges based on the sum of the yearly average temperature, the average of minimum temperature of the coldest month, and the average of maximum temperature of the coldest month (Rivas-Martínez et al. 2007, 2011). Thermotypes are associated with altitudinal gradients, which are key determinants of phenological timing (e.g. the ‘Hopkins’ bioclimatic law; Hopkins 1920) and are therefore likely to exhibit different patterns (Recio et al. 2009, Legave et al. 2015). Based on this evidence, we expected that phenological changes might differ among thermotypes, with earlier timing in warmer, low-elevation thermotypes and delayed timing in colder, high-elevation ones. However, more recent research, suggests that such altitudinal differences may be diminishing under the influence of climate change (Chen et al. 2018, Vitasse et al. 2018). In light of this, we also considered the alternative possibility that this pattern has been weakened by ongoing climate change and thus we designed our study with both scenarios in mind. In response to this knowledge gap, our study aims to examine the phenological responses across different bioclimatic belts in greater detail and to assess whether thermotypic differences persist or have been attenuated under ongoing climate change. Therefore, we have conducted a study across the western Mediterranean area of the Baetic Ranges in the Iberian Peninsula, using retrospective phenological data from herbarium specimens, in order to assess the impacts of climate change on the phenology of taxa belonging to the HCI groups: 4—temperate heath and scrub (HCI4); 5—sclerophyllous scrub-matorral (HCI5); 6—natural and semi-natural grassland formations (HCI6); 8—rocky habitats and caves (HCI8); and 9—forests (HCI9). Since phenology could behave differently depending on the bioclimatic belts, in addition, we focused on plant phenology trends at different altitudes using the classification of the thermotypes.

The purpose of this study was to analyse phenological trends over the past centuries in the western Baetic Ranges (southern Iberian Peninsula) for three reproductive phenophases (preflowering, flowering, and fruiting) and one vegetative phenophase (growth), based on characteristic plant taxa from HCI and across different thermotypes. Therefore, this study will focus on the following research questions:

1. Is the day of year (DOY) of the phenophases changing over time?
  1. Is plant phenology advancing/delaying and responding in a different way depending on the thermotype?
  2. Are plant taxa, belonging to different HCI, showing significant changes in phenology across the Baetic Range territory?
2. What is the relationship between the climatic variables and the occurrence of each phenophase across thermotypes and taxa?

Moreover, the phenological results will provide valuable insights for the management and conservation of the studied HCIs, which are also present in other Mediterranean regions of Europe and considered threatened habitats under the European Directive.

## Materials and methods

### Study area

**Figure 1.**
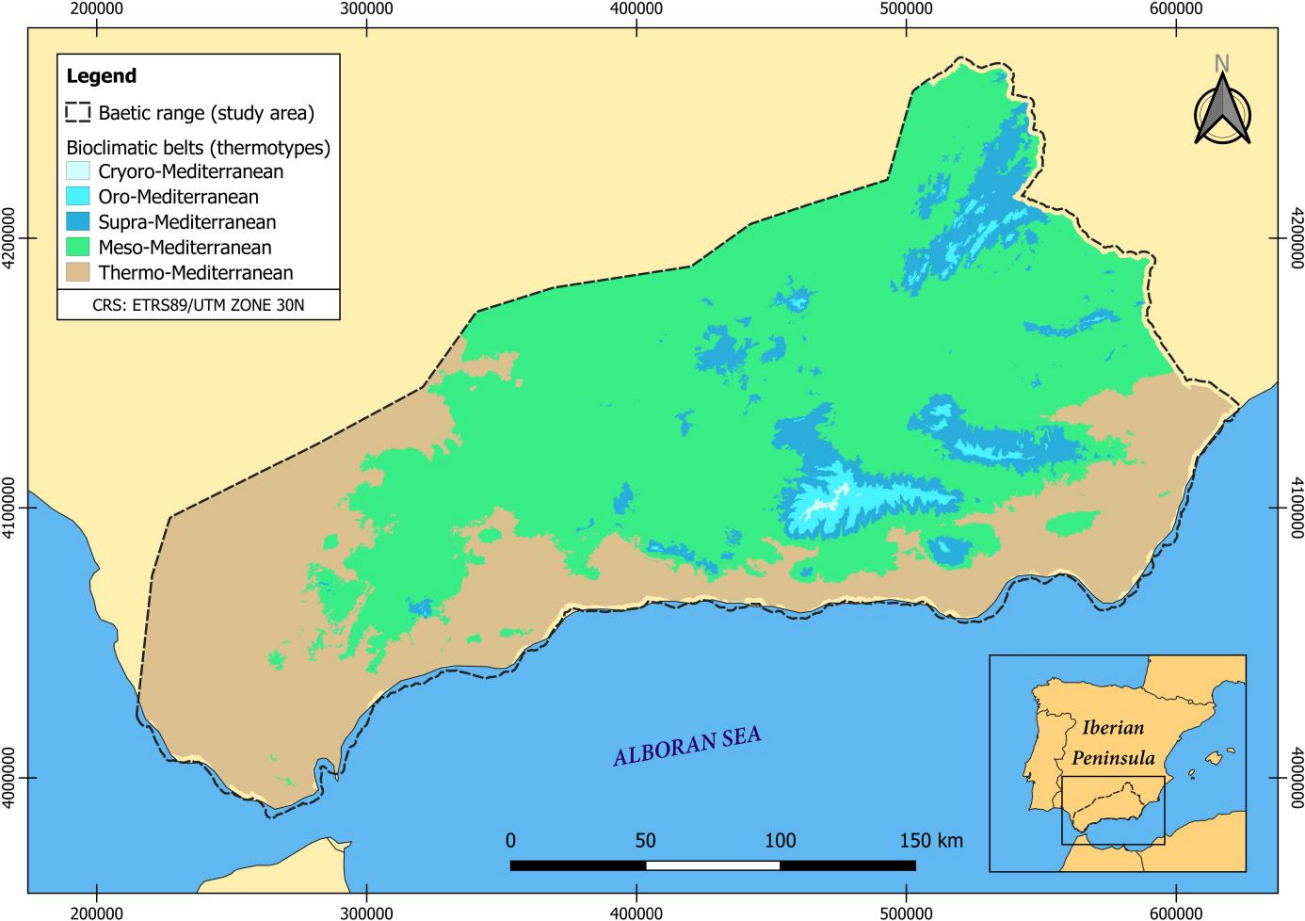
Study area delimitation (dashed line) and map of the thermotypes distribution (CRS: ETRS89/UTM zone 30N). The bioclimatic map is based on the division of thermotypes according to Rivas-Martínez et al. (2007) and developed by the Environmental Information Network of Andalusia (REDIAM 2023). Map lines delineate study areas and do not necessarily depict accepted national boundaries.

The study was conducted for the major area of the Baetic mountain ranges (∼90%), present in southern Iberian Peninsula and covering an extension of 49 669.71 km^2^ (Fig. 1). According to the Köppen–Geiger classification, the Baetic Range has a hot-summer Mediterranean climate (Csa), with cold semi-arid (steppe) climate (BSk) in the region of Almeria (Kottek et al. 2006; Peel et al. 2007). There is a rapid and high contrast of altitudes in these mountain ranges due to their abrupted topography caused by the Alpine orogeny (Maldonado et al. 1999): elevations range from 0 to 3479 m.a.s.l., and mean annual temperatures range from 8.35°C in the coldest month (January) to 27.1°C in the hottest month (July) (REDIAM 2024), encompassing the hottest and driest (Almeria), but also one of wettest areas of the Iberian Peninsula (Grazalema, in Cadiz), with annual precipitations of 206–2223 mm (Rivas-Martínez et al. 1997, Rodrigo et al. 1999, Giménez et al. 2004). This complexity mirrors the bioclimatic diversity of the study area (Fig. 1) showcasing five recognized thermotypes, all of which are stages of the Mediterranean bioclimate (Rivas-Martínez et al. 2007, 2011): thermo-Mediterranean (< 800 m.a.s.l.), meso-Mediterranean (800–1400 m.a.s.l.), supra-Mediterranean (1400–1900 m.a.s.l.), oro-Mediterranean (1900–2900 m.a.s.l.), and cryoro-Mediterranean (>2900 m.a.s.l.). Typically, different types of vegetation are identified within each thermotype class. This bioclimatic heterogeneity has resulted in a high level of plant endemism in the Baetic Ranges, making them one of the highest areas of endemicity in the Iberian Peninsula (Medail and Quezel 1997, 1999, Casimiro-Soriguer et al. 2021).

### Studied taxa and phenological data collection

We examined 128 taxa that were characteristic of habitats included in the Annex I of the European Directive 92/43/EEC, known as Habitats of Community Interest (HCI): (1) HCI4, containing seven different habitats; (2) HCI5, including 12 different habitats; (3) HCI6, including seven different habitats; (4) HCI8, including four different habitats; and (5) HCI9, including 12 different habitats. Several taxa were selected for this study due to their endemic character to the Baetic Ranges (Blanca et al. 2011). The composition of these characteristic taxa was identified according to the regional habitats guide (REDIAM 2020), the national habitats guide (VV.AA 2009) and the ‘Interpretation Manual of European Union Habitats’ (European Commission DG-ENV 2013). The characteristic taxa list (Supplementary Table S1) was reviewed including three types of taxa: (1) diagnostic taxa, which are present in a particular habitat but absent or rare in others; (2) constant taxa, which are frequent but not exclusive to a habitat; and (3) majority cover taxa, which often reach high cover in a particular habitat determining its physiognomy (Chytrý and Tichý 2003). From the total selected taxa, 31 corresponded to the HCI4, 55 to the HCI5, 10 to the HCI6, 14 to the HCI8, and 18 to the HCI9. At the same time, 50 out of 128 taxa were Baetic endemics, and half of those were narrow endemic, mostly found in biodiversity hotspots of the area (Cañadas et al. 2014). The phenological calendars (i.e. graphical representations by taxon which detail the timing of its phenophases occurring from January to December) of the taxa were also developed.

**Figure 2.**
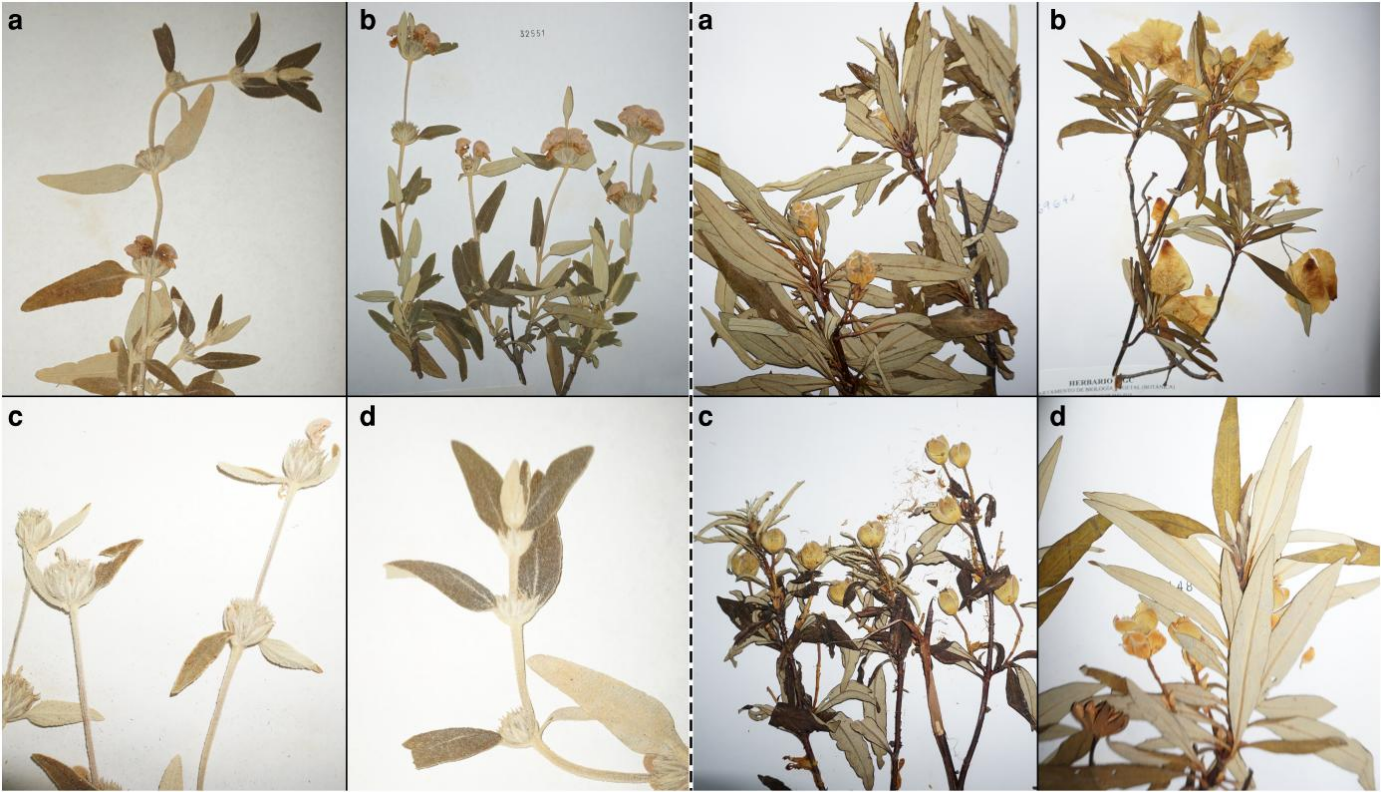
Examples of two different species of the observed phenophases: (a) preflowering (FBF); (b) flowering (F); (c) fruiting (FS); (d) growth (DVG). *Phlomis purpurea* on the left and *Cistus ladanifer* on the right.

We used stored data from the herbaria of the University of Malaga (MGC), University of Granada (GDA), University of Sevilla (SEV), and from the Royal Botanical Garden of Madrid (MA) to examine over 10 000 preserved specimens, collected between 1822 and 2020 (Supplementary Tables S1 and S2). Of the total observed preserved specimens, 6805 showed at least one of the phenophases under study. Each phenophase was determined by visual analysis of the preserved specimens, observing both the presence/absence of different reproductive and vegetative structures (Fig. 2). The reproductive phenophases were preflowering (flowering bud formation = FBF), flowering (F) and fruiting (fruit set = FS), while the vegetative phenophase was growth activity in the dolichoblast branches (Navarro and Cabezudo 1998), known as dolichoblast vegetative growth (DVG). The phenophase identification was conducted according to Primack et al. (2004), Miller-Rushing et al. (2006) and Solakis-Tena et al. (2025). We also observed if trends across phenophases were consistent within the same taxon; that is, if FBF advanced, then F, FS, and/or DVG did as well, and vice versa. This effect is known as sequencing and has been reported in previous studies (Milla et al. 2010, Navarro and Hidalgo-Triana 2021, Solakis-Tena et al. 2025). Location data collection was obtained from the labels of the observed herbarium specimens, and each register was assigned to a thermotype based on its locality and altitude at which it was collected, following the classification of Rivas-Martínez et al. (2007).

### Climatic data

A total of 1224 weather stations were selected throughout the Baetic mountain ranges, containing meteorological data spanning from 1805 to 2019 (Supplementary Figure S1) and using the Environmental Information Network of Andalusia (REDIAM 2023), which in turn was received from the State Meteorological Agency (AEMET). Climate data prior to 1911 were excluded from the analyses due to their basis on a limited number of meteorological stations (<30) scattered across the study area. Subsequently, two distinct climatic datasets were generated, the first dataset included the computation of monthly and annual climatic data for the entire Baetic territory and the second involved calculations partitioned by thermotypes. To zone the territory based on its various thermotypes, we utilized a bioclimatic map of the southern Iberian Peninsula (Fig. 1; REDIAM 2023). Due to the limited data available for some phenophases in specific thermotypes, and in order to make possible a more concise analysis of the phenological patterns in low, middle and high-mountain ecological requirements, we opted to regroup the thermotypes into three broad bioclimatic groups: bioclimatic belts with more thermophilous taxa (thermo-Mediterranean thermotype = TM), bioclimatic belts with mesophilous or mid-mountain taxa (meso-Mediterranean and supra-Mediterranean thermotypes = MS), and bioclimatic belts with orophilous or high-mountain taxa (oro-Mediterranean and cryoro-Mediterranean thermotypes = OC). After running correlation analysis (Supplementary Figures S2 and S3), we selected the following climatic variables as potential predictors: annual T and P, and spring T and P. Temperature-related variables (T) were expressed in °C, while precipitation-related variables (P) were expressed in mm. Spring variables were considered as the average of the months from March to May. To assess the effect of climate change on these variables, we fitted separate linear models for each of the four long-term variables using date as a continuous predictor. This step aimed to quantify the magnitude and direction of climatic change over time, in order to test whether phenological advances or delays in the Baetic Ranges are linked to ongoing warming and drying in the region.

### Statistical analysis

For each register, the DOY was collected from the date of sampling recorded on the herbarium specimen's label and subsequently converted to an integer number. We established a minimum sample size of 15 specimens’ records from different dates and locations that were in a specific phenophase, increasing the minimum sample size of similar previous studies (Gallagher et al. 2009, Calinger et al. 2013, Solakis-Tena et al. 2025). Taxa included in our analysis presented sample sizes up to 161 specimens in a phenophase (see Supplementary Table S2). To avoid problems related with outliers, we applied a conservative data correction method that uses the median absolute deviation (MAD) following Leys et al. (2013). DOY outside the range of the median ± 3.5 × MAD were discarded. The threshold of 3.5 was selected since it only excludes the extreme values (Menzel et al. 2020, Picornell et al. 2023; Solakis-Tena et al. 2025). Altogether, these requirements reduced the number of taxa with sufficient data for analysis from 128 to 77.

To address the first question (Q1), we run time-based models comparing the DOY with its corresponding decimal year, in which decimals of a year refer to a specific day and month of the same year. For Q1.1, the DOY was modelled with the time variable (decimal years) across bioclimatic groups. In these models we analysed the fixed effect of the bioclimatic groups and added taxa as a random effect variable to account for potential non-independence of observations within species, acknowledging that phenological responses may vary among taxa. To specifically test whether phenological responses differed among thermotypes, we incorporated the interaction term between time (decimal year) and bioclimatic group. To cope with this, we used R software (R Core Team 2021) and package ‘lme4’ (Bates et al. 2015) to fit linear mixed effects models (LMM). To obtain order-invariant tests robust to the unbalanced design, we computed Type II sums of squares with ‘lmerTest’ (Kuznetsova et al. 2017) using Satterthwaite degrees of freedom (anova, type II) and contrasts were set to sum-to-zero. When the interaction was not significant, we reported the assemblage-wide slope. When the interaction was significant, we reported simple slopes by thermotype and Tukey-adjusted pairwise contrasts of slopes. We considered effects significant at α = 0.05 (*p* < 0.05). Results with 0.05 ≤ *p* < 0.06 were reported as marginally significant and interpreted with caution. We also checked for overdispersion by using the R package ‘performance’ (Lüdecke et al. 2021). The formula implemented in the time-based model for the bioclimatic groups was:


DOY∼Years*BioclimaticGroup+(1|Taxon)

For Q1.2, the DOY was modelled with the time variable by taxon. Initially, we used LM; however, when assumptions for these models were not met, we used GLM of Gaussian family and logarithmic link function (Nelder and Wedderburn 1972) or GAM (Wood 2017) of the Gaussian type with an identity link function, including five bases (*k* = 5) smoothed with the shrinkage modified version of the thin plate regression spline (bs = ‘ts’), as indicated in Marra and Wood (2011). To test for normality in LM, the Shapiro-Wilk test was applied when *n* < 50, while the Kolmogorov–Smirnoff test was used for cases where *n* ≥ 50. To verify if the influence of the independent variable on the dependent variable of each model was significant, the Student's *t*-test was applied (α = 0.05).

Prior to addressing the second question (Q2), we conducted a regression analysis using simple linear models to provide context on climate change in the study area, regressing the main long-term climatic variables (annual and spring temperature, and annual and spring precipitation) against time. To respond Q2, we run climate-based models comparing the DOY with the aforementioned corresponding climatic variables independently. When analysing the climatic variables relationship with phenophases across the bioclimatic groups, the DOY was modelled following the same approach as for Q1.1. The formula implemented in the climate-based model for the bioclimatic groups was:


$$\begin{array}{rl} & DOY∼\mathrm{climatic}\;\mathrm{variable}\;*\mathrm{Bioclimatic}\;\mathrm{Group}\;+\;(1|\mathrm{Taxon});\\ & \mathrm{where}\;\mathrm{the}\;\mathrm{climatic}\;\mathrm{variable}\;\mathrm{is}\;\mathrm{one}\;\mathrm{of}\;\mathrm{the}\;\mathrm{four}\;\mathrm{aforementioned}. \end{array}$$

When the DOY was modelled with the climatic variables by taxon, we followed the same approach as for Q1.2, using LM, GLM or GAM for the models and the same tests for normality and influence of the variables. Here, a set of models with different variables were evaluated in each case (phenophase and taxon), based on the Akaike Information Criterion (AIC; Aho et al. 2014). The models to be evaluated followed a hierarchical sequence starting with annual temperature and then, additional variables were incorporated one by one in a predefined order: monthly temperature, spring temperature, annual precipitation, monthly precipitation, and spring precipitation. Thus, the model that minimized information loss (lowest AIC) was selected. The climate variables were selected based on the existing correlation among them (Supplementary Figures S2 and S3), eliminating those that were beforehand highly correlated (>0.7), using the ‘corrplot’ function in R. The variance inflation factor (VIF) for variables’ collinearity was also obtained for the climate-based models.

## RESULTS

### Phenological trends over years

#### Phenological trends by thermotypes over years

While the fixed effects of years and thermotypes explained only a modest proportion of variance (*R*² marginal ≈ 0.05–0.15), the higher values in the conditional R² (≈0.31–0.61) and the substantial intra-class correlation coefficient (ICC ≈ 0.19–0.57) indicated that much of the phenological variability is attributable to differences among taxa. This indicated taxa played a major role in shaping phenological responses, beyond the shared influence of climate or thermotype.

**Figure 3.**
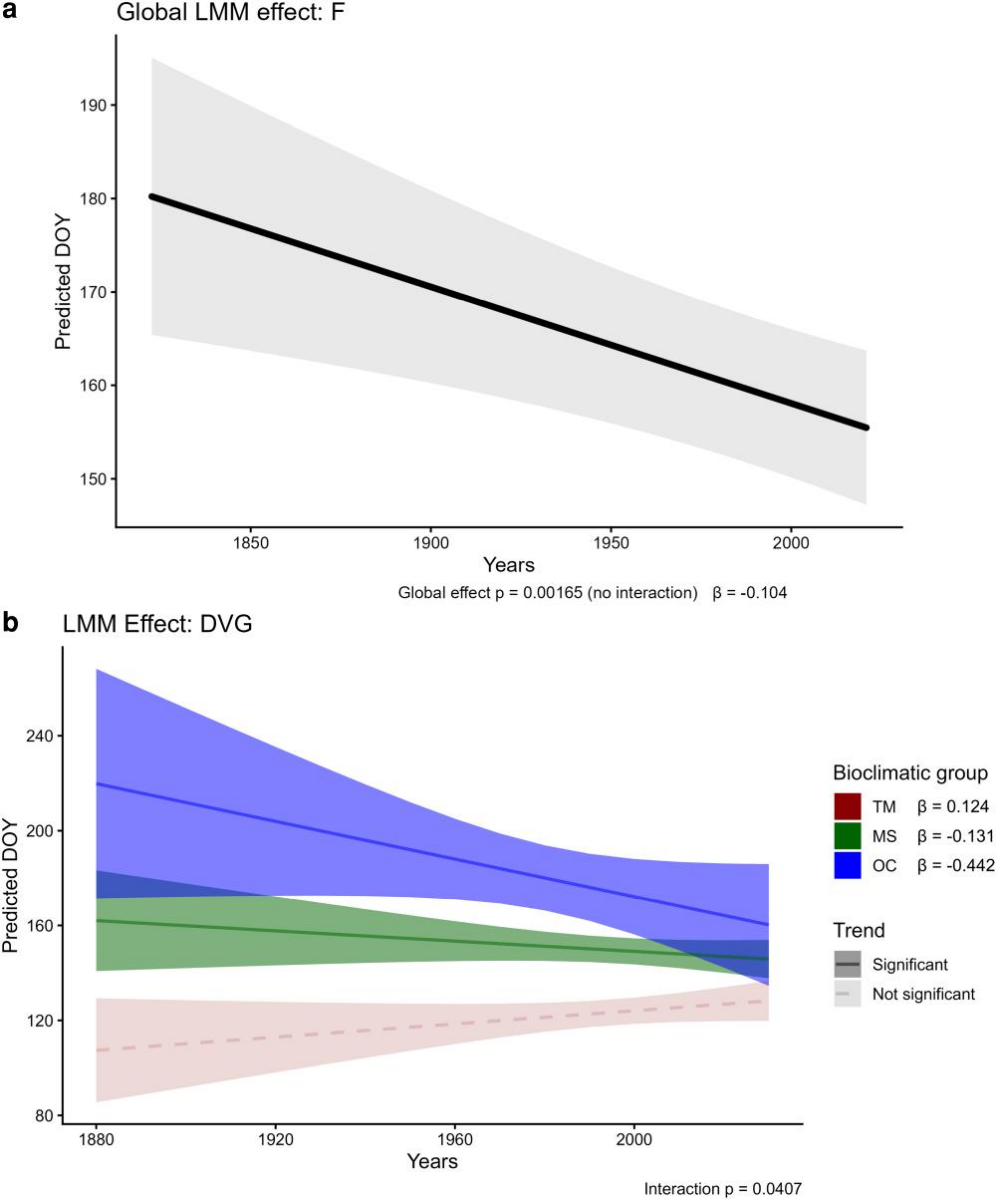
Global trend of DOY over the years in (a) F (flowering) and trends in (b) of DVG (growing) by bioclimatic group (TM , thermo-Mediterranean; MS, meso-supra-Mediterranean; OC, oro-cryoro-Mediterranean) with taxa as a random variable effect. β = slope, denoting the change in DOY per year.

When observing the phenophases, FBF (preflowering) and FS (fruiting) showed no effect of the variable years and thermotypes, and neither an interaction effect, in consequence, their global slopes were not different from zero and the DOY means among thermotypes were not different between them (Table 1). In contrast, a significant effect of years was found in F (flowering; Fig. 3a), exhibiting a significant global advance of 0.87 days/decade (Table 1). However, no effect of the thermotypes and interaction with years was found in F, showing no statistical difference in the means between thermotypes. The DVG (vegetative growth; Fig. 3b) phenophase presented a significant effect of the thermotypes, showing differences in their means, and a significant interaction between years and thermotypes. The post-hoc analysis showed that DVG was advancing 1.31 days/decade in the MS, and 4.42 days/decade in the OC thermotypes (Table 2), while the slope was not different from zero in the TM thermotype. This divergence was captured in the pairwise comparisons, where TM showed statistically different slopes with MS (Δslope = + 2.55 day/decade; *P* = 0.024) and OC (Δslope = + 5.66 day/decade; *P* = 0.007), indicating that the slope in TM advances less (or delays more) than in MS or OC. However, no differences were found between MS and OC.

**Table 1.** Results of LMMs for the years variable and thermotypes by phenophase with taxa as a random variable effect.

Ph^a^	Eff^a^	F	*p* value eff.^a^	R^2^ m^a^	R^2^ con^a^	ICC^a^ Taxon	Glob^a^ β (days/year)	SE glob^a^	Lower. CL glob^a^	Upper. CL glob^a^	*t*.ratio glob^a^	*P* value glob^a^ β
FBF	Years	0.215	0.643	0.052	0.513	0.487	−0.004	0.053	−0.108	0.099	−0.085	0.932
	Tt^a^	1.526	0.218									
	Years: Tt^a^	1.336	0.263									
F	Years	15.026	**0.0001**	0.090	0.610	0.571	**−0.087**	0.027	−0.140	−0.034	−3.207	**0.001**
	Tt^a^	2.208	0.110									
	Years: Tt^a^	1.613	0.199									
FS	Years	1.818	0.178	0.115	0.466	0.396	−0.061	0.052	−0.163	0.041	−1.175	0.24
	Tt^a^	0.384	0.682									
	Years: Tt^a^	0.309	0.734									
DVG	Years	0.832	0.362	0.147	0.310	0.191	—	—	—	—	—	—
	Tt^a^	6.891	**0.001**									
	Years: Tt^a^	6.265	**0.002**									

^a^Ph, Phenophase, Eff, Effect; Tt, Thermotypes; R2 m, Marginal R^2^; R^2^ con, Conditional R^2^; Glob, Global; β, Slope; ICC, Intra-class correlation coefficient. Values are in bold when *P* < 0.06.

**Table 2.** LMMs for phenophases with significant interaction between years and thermotypes.

Ph^a^	Tt^a^	β (days/year)	SE	Df	Lower.CL	Upper.CL	T.ratio	*P* value
DVG	TM	0.124	0.071	1629.740	−0.015	0.263	1.743	0.081
	MS	**−0**.**131**	0.067	1651.825	−0.263	0.0003	−1.956	**0**.051*
	OC	**−0**.**442**	0.172	1653.998	−0.780	−0.104	−2.563	**0**.**010**

^a^Ph, Phenophase; Tt, Thermotypes; Glob, Global; β, Slope. Values are in bold when *P* < 0.06. *Marginally significant (0.05 ≤ *P* < 0.06).

Extended information regarding model selection, model validation, and results of the phenological trends over time by thermotypes are in Supplementary Appendix S1.

#### Phenological trends by taxon over years

**Figure 4.**
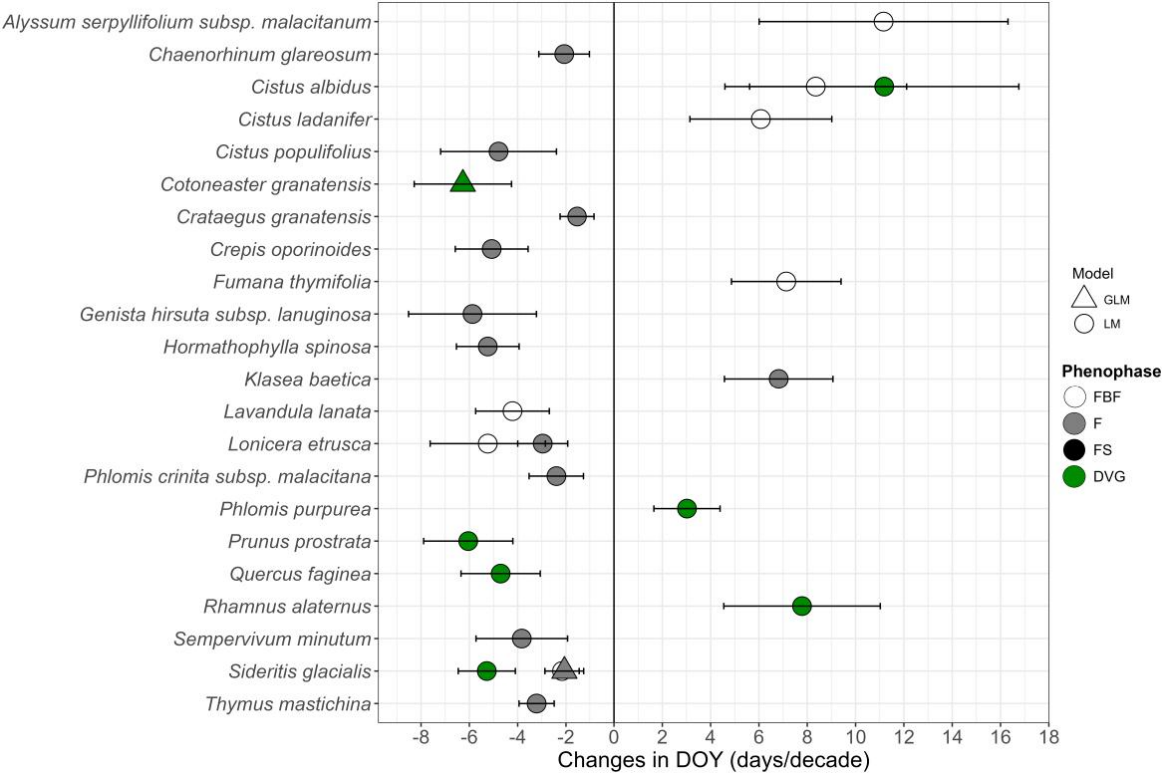
Significant tendencies of the day of year (DOY) over time models, conducted for every taxon and phenophase. LM refers to the linear models and GLM to the generalized linear models. Regarding phenophases, FBF corresponds to preflowering, F to flowering, FS to fruiting, and DVG to growth. The results show the slopes of the DOY with years, which can be expressed in days of advance or delay per decade. It should be noted that *Sideritis glacialis* showed advances in both FBF and F (FBF = −2.16 ± 0.7 days/decade; *F* = −2.06 ± 0.81 days/decade) despite not being clearly visible in the plot. To facilitate understanding of the results, all slopes shown are from LM results. However, for species marked with triangles, the interpretation is based on GLM models and readers should refer to the Supplementary Appendix S2 for the accurate model results.

Phenological calendars were constructed for each taxon based on the observed data (Supplementary Figures S4–S7). Over time, 22 taxa exhibited phenological shifts in one or more of their phenophases (Fig. 4). In phenophase FBF, three taxa showed advances (*Lavandula stoechas*, *Lonicera etrusca* and *Sideritis glacials*) and four delays (*Alyssum serpyllifolium* subsp. *arundanum*, *Cistus albidus*, *Cistus ladanifer* and *Fumana thymifolia*) over time, in phenophase F, 11 showed advances (*Chaenorhinum glareosum*, *Cistus populifolius*, *Crataegus granatensis*, *Crepis oporinoides*, *Genista hirsuta* subsp. *lanuginosa*, *Hormathophylla spinosa*, *L. etrusca*, *Phlomis crinita* subsp. *malacitana*, *Sempervivum minutum*, *S. glacialis* and *Thymus mastichina*) and one (*Klasea baetica*) showed a delay over time, in phenophase FS no taxa showed trends with time, and in phenophase DVG four showed advances (*Cotoneaster granatensis*, *Prunus prostrata*, *Quercus faginea* and *S. glacialis*) while three showed delays (*C. albidus*, *Phlomis purpurea* and *Rhamnus alaternus*) over time. All the taxa with delays pertained to the HCI5, with the exception of *C. ladanifer* (HCI4). The number of taxa showing advances was higher and more diverse across the HCI groups, affecting all of them. Among the affected taxa, 10 were endemic, four of which were considered narrow endemics (Supplementary Table S1). It is noteworthy that trends across phenophases are consistent within the same taxon, showing phenophase sequencing. No cases exhibited opposing outcomes over years (e.g. FBF advancing and F delaying). The models presented an R^2^ range of ∼0 (low explained variance)—0.6 (high explained variance). Additional information on the time-based models is provided in Supplementary Appendix S2.

### Phenological trends through climate variables

#### Climatic variables trends in the baetic ranges

**Figure 5.**
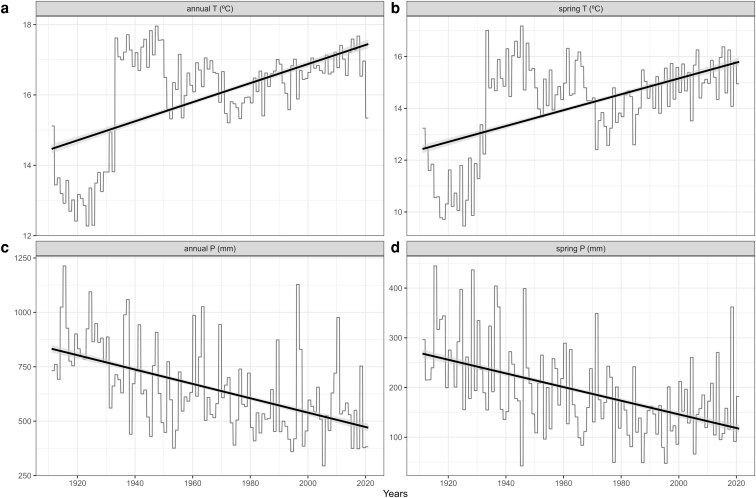
Long-term significant trends (all *P* < 0.01) of the climatic variables over the years in the Baetic Ranges. β = slope.

Linear trend analyses conducted on long-term climatic records across the Baetic region revealed consistent and statistically significant signals of recent climate change (Fig. 5). Specifically, annual mean temperature increased at a rate of ∼0.27°C/decade (Fig. 5a), while spring mean temperature (Fig. 5b) rose by 0.31°C/decade (both *P* ∼ 0). This indicates a warming of ∼2.7°C in the mean annual T and 3.1°C in the spring T in the last century. In contrast, precipitation exhibited a declining trend, with annual precipitation decreasing by 33 mm per decade (Fig. 5c) and spring precipitation by 14 mm per decade (Fig. 5d), also with strong statistical support (*P* ∼ 0). This is translated as a loss of approximately 330 mm in annual precipitation and 140 mm in spring precipitation over the last century. These trends were associated with moderately high coefficients of determination (R² values between 0.24 and 0.35). Extended model results and residual analyses are provided in Supplementary Appendix S3.

***Phenological relationships of thermotypes through climate variables***

### Effect of annual and spring temperatures

The effect of temperature on DOY varied across phenophases and bioclimatic groups (thermotypes), showing slight differences in the results regarding annual or spring temperature (annual T and spring T, respectively, Table 3). Across phenophases, marginal R² values ranged from 0.049 to 0.133, indicating that annual and spring T and bioclimatic group (fixed effects) explained a modest proportion of the variance in DOY. In contrast, conditional R² ranged from 0.3 to 0.579, and ICC values from 0.195 to 0.535, suggesting that a substantial portion of the explained variance was attributable to the taxa (random effect). Extended details on model selection, validation, and phenological trends with climatic variables by thermotypes are provided in Supplementary Appendix S4.

**Table 3.** Results LMMs of the temperature variables by phenophase.

Ph^a^	Var^a^	*P* value var^a^	*P* value Tt^a^	*P* value inter^a^	R^2^ m^a^	R^2^ con^a^	ICC^a^ taxon	Glob^a^β (day/°C)	Lower.CL	Upper.CL	*P* value glob^a^ β
FBF	annual T (°C)	0.728	0.883	0.946	0.049	0.511	0.486	−0.541	−3.386	2.304	0.709
F	annual T (°C)	**3.756** **×** **10⁻^11^**	0.759	0.835	0.093	0.578	0.535	**−5**.**144**	−6.702	−3.587	**1.100** **×** **10⁻^10^**
FS	annual T (°C)	**0**.**035**	0.299	0.286	0.115	0.461	0.391	−2.379	−6.399	1.642	0.246
DVG	annual T (°C)	*0.**053**	0.149	0.319	0.132	0.301	0.195	−2.018	−4.665	0.630	0.135
FBF	spring T (°C)	*0.**052**	0.418	0.884	0.050	0.513	0.487	−1.528	−3.281	0.224	0.087
F	spring T (°C)	**4.852** **×** **10⁻^10^**	0.292	0.631	0.092	0.579	0.536	**−2**.**862**	−3.841	−1.882	**1.132** **×** **10⁻^8^**
FS	spring T (°C)	**0**.**039**	*0.**056**	0.118	0.116	0.463	0.393	−1.358	−3.927	1.210	0.299
DVG	spring T (°C)	0.653	**0**.**001**	0.105	0.133	0.300	0.193	0.156	−1.494	1.807	0.853

^a^Ph, Phenophase; Var, Variables; Tt, Thermotypes; Inter, Interaction; R^2^ m, Marginal R^2^; R^2^ con, Conditional R^2^; Glob, Global; β, Slope; ICC, Intra-class correlation coefficient. Values are in bold when *P* < 0.06.

*Marginally significant (0.05 ≤ *P* < 0.06).

No interaction between bioclimatic group and temperature was detected in any phenophase (Table 3), indicating that phenological responses to temperature were consistent across thermotypes. This suggests that elevational differences no longer modulate temperature sensitivity, pointing to a homogenization of phenological responses along the gradient. For preflowering (FBF), fruiting (FS), and growing (DVG), global slopes were no significantly different from zero in relation to annual and spring T. In contrast, F showed a significant main effect without interaction with spring T (global slope = −2.86 days/°C) and annual T (global slope = −5.14 days/°C), indicating an earlier DOY occurrence with the increase of temperatures in all thermotypes homogeneously. The means of DOY differed among thermotypes in DVG (*P* = 0.001) and FS (but marginally significant: *P* = 0.056) regarding spring T; however, no changes were found in their slopes.

### Effect of annual and spring precipitation

The effect of annual and spring precipitation (annual P and spring P, respectively) on DOY was generally weak across phenophases and bioclimatic groups (Table 4). Across models, marginal R² values ranged from 0.048 to 0.134, indicating that precipitation and bioclimatic group accounted for a limited proportion of the variance in DOY. In contrast, conditional R² values ranged from 0.301 to 0.614, and ICC values from 0.193 to 0.587, suggesting that a substantial portion of the explained variance was attributable to the taxa (Table 5).

**Table 4.** Results LMMs of the precipitation variables by phenophase.

Ph^a^	Var^a^	*P* value var^a^	*P* value Tt^a^	*P* value inter^a^	R^2^ m^a^	R^2^ con^a^	ICC^a^ taxon	Glob^a^ β (day/°C)	Lower.CL	Upper.CL	*P* value glob^a^ β
FBF	annual P (mm)	0.878	**6.231** **×** **10⁻^6^**	**0**.054*	0.048	0.513	0.488	—	—	—	—
F	annual P (mm)	**0**.**023**	**9.428** **×** **10⁻^8^**	0.995	0.072	0.614	0.584	**0**.**007**	0.001	0.014	**0**.**024**
FS	annual P (mm)	0.664	0.375	0.074	0.105	0.450	0.385	−0.001	−0.017	0.015	0.928
DVG	annual P (mm)	0.164	**1.178** **×** **10⁻^5^**	0.982	0.134	0.302	0.193	0.007	−0.003	0.018	0.162
FBF	spring P (mm)	**0**.**018**	**2.464** **×** **10⁻^5^**	0.817	0.049	0.512	0.487	**0**.**033**	0.006	0.060	**0**.**015**
F	spring P (mm)	**8.372** **×** **10⁻^7^**	**3.248** **×** **10⁻^11^**	0.434	0.076	0.618	0.587	**0**.**034**	0.019	0.049	**1.236** **×** **10⁻^5^**
FS	spring P (mm)	0.252	*0.059	0.157	0.105	0.452	0.387	0.010	−0.030	0.050	0.626
DVG	spring P (mm)	0.775	**3.029** **×** **10⁻^10^**	0.924	0.134	0.301	0.193	0.004	−0.022	0.029	0.782

^a^Ph, Phenophase; Var, Variables; Tt, Thermotypes; Inter, Interaction; R^2^ m, Marginal R^2^; R^2^ con, Conditional R^2^; Glob, Global; β, Slope; ICC, Intra-class correlation coefficient. Values are in bold when *P* < 0.06.

*Marginally significant (0.05 ≤ *P* < 0.06).

**Table 5.** LMMs by phenophases with significant interaction between precipitation variables and thermotypes.

Ph^a^	Var^a^	Tt^a^	β (days/mm)	SE	Df	Lower CL	Upper CL	t.ratio	*P* value
FBF	annual P (mm)	TM	0.011	0.008	1278.799	−0.005	0.026	1.298	0.194
		MS	−0.006	0.009	1310.441	−0.023	0.011	−0.704	0.482
		OC	**−0**.**083**	0.043	1351.816	−0.167	0.001	−1.928	**0**.054*

^a^Ph, Phenophase; Var, Variables; Tt, Thermotypes; β, Slope. Values are in bold when *P* < 0.06.

*Marginally significant (0.05 ≤ *P* < 0.06).

No significant main effects or interactions were detected in FS and DVG regarding precipitation variables and bioclimatic groups (thermotypes), with the exception of a marginally significant difference in DOY means for FS in relation to spring P (Table 4).

The FBF phenophase showed no significant global effect of annual P on DOY. However, a significant effect of thermotypes was detected, as well as a marginally significant interaction between thermotypes and annual P. The post-hoc analysis indicated that the slopes were not significantly different from zero in the TM and MS. In contrast, in the OC, wetter years tended to advance FBF (−8.27 days/100 mm; *P* = 0.054), but the effect was marginally significant. Moreover, this divergence was not reflected in the pairwise comparisons, where the slope between TM, MS and OC showed no differences (TM-MS: *P* = 0.335; TM-OC: *P* = 0.083; MS-OC: *P* = 0.185). FBF showed a significant global effect on the DOY regarding spring P, delaying this phenophase occurrence with wetter springs (3.35 days/100 mm). Flowering (F) was the phenophase with significant main effects of both annual P (delay with wetter years: 0.7 days/100 mm) and spring P (delay with wetter spring: 3.4 days/100 mm).

Extended information regarding model selection, model validation, and results regarding the phenological trends with climatic variables by thermotype are in Supplementary Appendix S4.

#### Phenological relationships by taxon through climate variables

Phenophases exhibited more significant relationships with climatic models than with time-based models, with R^2^ values ranging from 0.12 to 0.96. Only two of the analysed taxa did not show significant responses with respect to any climatic variable (*Juniperus sabina* and *Leontodon boryi*). Considering long-term variables (annual and spring T and P), 22 taxa showed earlier phenophase dates with increased annual T or spring T, and decreased annual P or spring P in phenophase FBF. Three taxa showed later phenophase dates with increased annual T or spring T in FBF phenophase. In phenophase F, 49 taxa showed earlier phenophase dates with increased annual T or spring T, and decreased spring P. Six taxa delayed in phenophase F in relation to increased annual T or spring T and decreased annual P. In phenophase FS,12 taxa showed earlier phenophase dates with increased annual T or spring T, and five taxa showed later phenophase dates with increased spring T or decreased annual P. In phenophase DVG, 24 taxa showed earlier phenophase dates with increased annual T or spring T, and decreased annual P, and two taxa showed later phenophase dates with increased spring T and decreased spring P. The taxa with earlier or later trends in phenophase dates pertained to all the HCI groups. More information about the climatic models is available in Supplementary Appendix S5.

### Annual and spring temperatures

**Figure 6:**
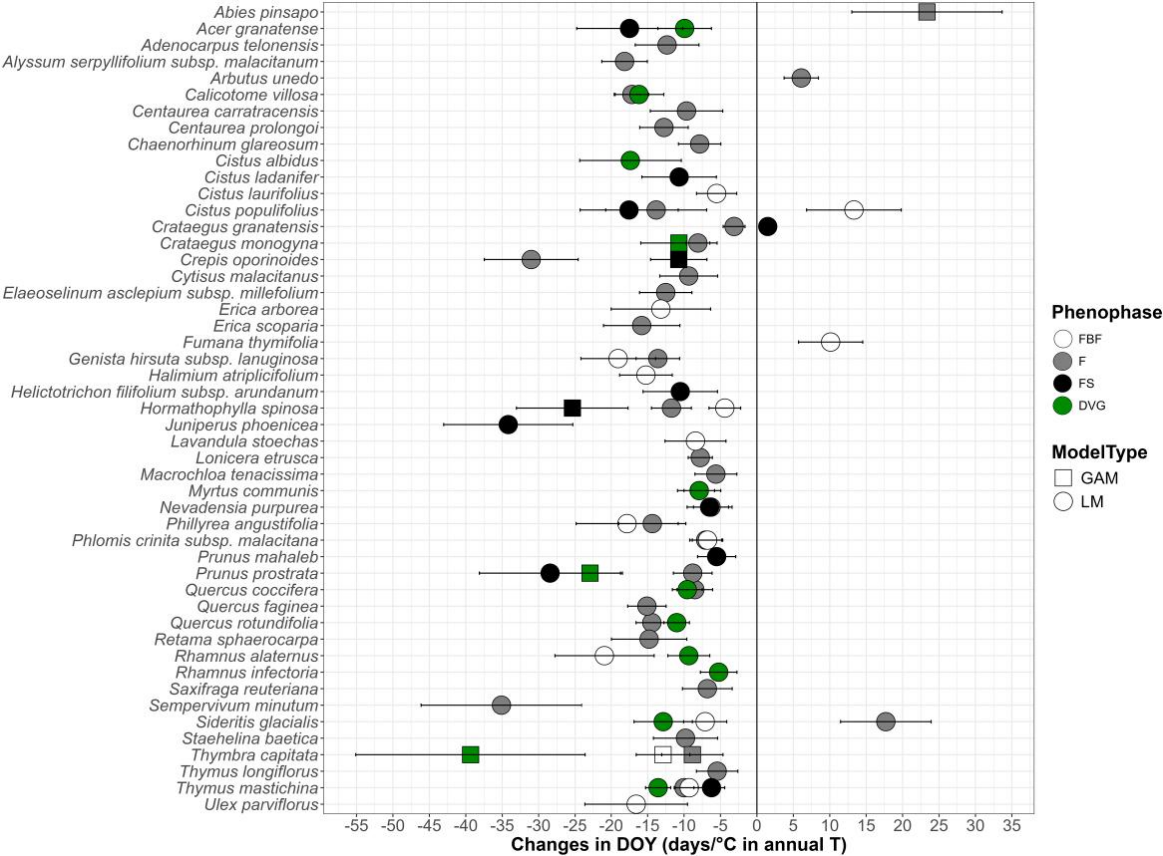
. Significant relationships of the day of year (DOY) in the climatic models for every taxon and phenophase over the annual average T. LM refers to the linear models and GAM to the generalized additive models. Regarding phenophases, FBF corresponds to preflowering, F to flowering, FS to fruiting, and DVG to growth. It should be noted that, despite not being clearly visible in the plot, *Nevadensia purpurea* showed advances in both F and FS (F = −6.29 ± 2.4 days/°C; FS = −6.49 ± 3.08 days/°C) and *Phlomis crinita* subsp. *malacitana* in FBF and F (FBF = −6.77 ± 2.09 days/°C; F = −7.02 ± 2.19 days/°C). The results show the slopes of the DOY with the increase of annual T, which can be expressed in days of advance or delay per increased °C. To facilitate understanding of the results, all slopes shown are from LM results. However, for species marked with squares, the interpretation is based on GAM models and readers should refer to the Supplementary Appendix S5 for the accurate model results.

Regarding annual T, 49 taxa displayed phenological shifts in one or more phenophases as annual temperatures increased (Fig. 6). The predominant response was an earlier phenophase occurrence with the increasing annual T in all the HCI groups, observed in 46 taxa, of which five were endemic and eight narrow endemic (Supplementary Table S1). In contrast, four taxa showed later phenophase occurrence with this variable, all of them pertaining to HCI4 and HCI5, with the exception of phenophase F in *Abies pinsapo*, which belong to the HCI9. Thirteen taxa showed earlier FBF occurrence dates and two showed later occurrence dates in phenophase FBF; 32 taxa showed earlier F occurrence dates and only three showed later F occurrence dates; 11 taxa exhibited earlier FS occurrence dates and one (*Crataegus granatensis*) exhibited a later FS occurrence; and 13 taxa demonstrated earlier DVG occurrence dates and no later occurrence dates with the increase of annual T.

**Figure 7.**
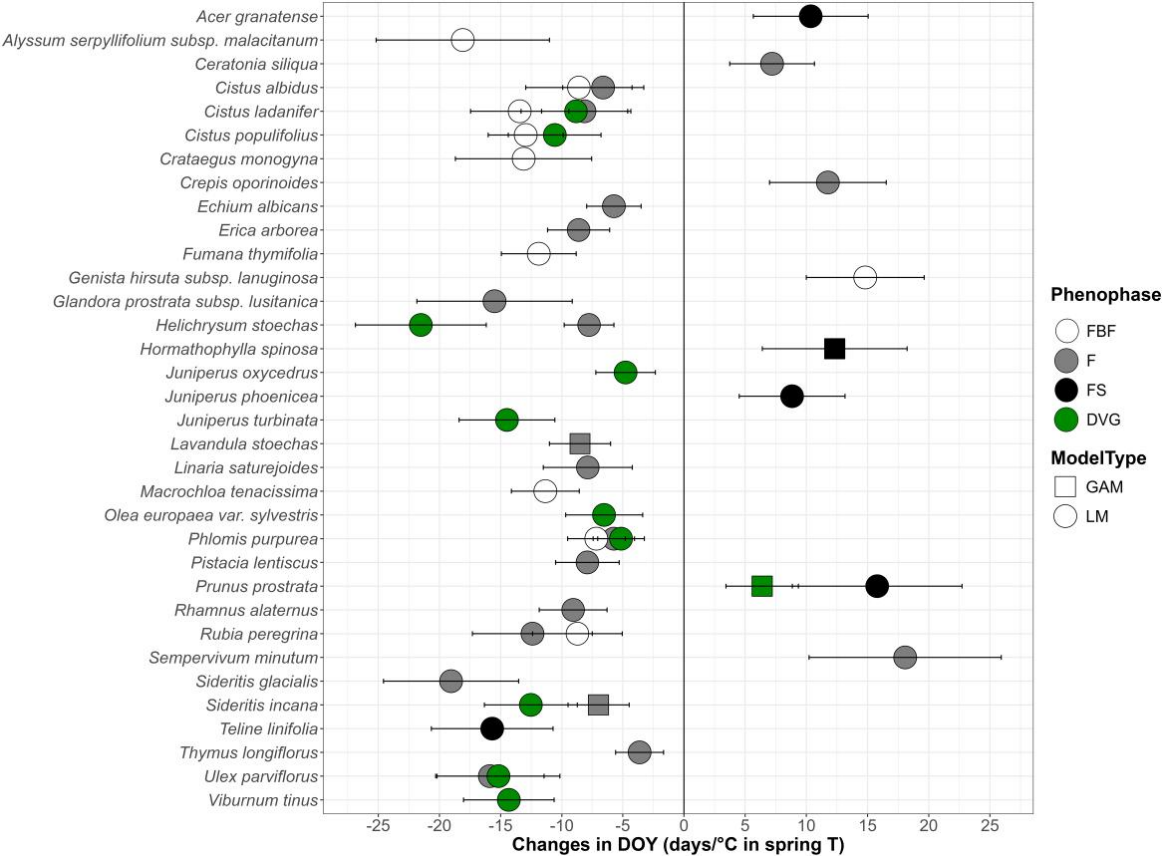
Significant relationships of the day of year (DOY) in the climatic models for every taxon and phenophase over the spring average T. LM refers to the linear models and GLM to the generalized linear models. Regarding phenophases, FBF corresponds to preflowering, F to flowering, FS to fruiting, and DVG to growth. The results show the slopes of the DOY with the increase of spring T, which can be expressed in days of advance or delay per increased °C. To facilitate understanding of the results all slopes shown are from LM results. However, for species marked with squares, the interpretation is based on GAM models and readers should refer to the Supplementary Appendix S5 for the accurate model results.

Regarding the spring temperature variable, 34 taxa displayed phenological shifts in one or more phenophases as spring T increased (Fig. 7). Of these, 26 taxa showed earlier phenophase occurrence dates, most belonging to HCI4 and HCI5, including four endemic taxa and one narrow endemic taxon (Supplementary Table S1), while eight taxa showed later occurrence dates. Nine taxa exhibited earlier FBF occurrence dates and one later occurrence dates; 16 taxa showed earlier F occurrence dates and three later occurrence dates; one taxon (*Teline linifolia*) displayed earlier FS occurrence dates and four later occurrence dates; and 10 taxa demonstrated earlier DVG occurrence dates and one (*Prunus prostrata*) later occurrence dates. It is noteworthy that, unlike the three taxa with divergent trends in annual T, the phenophase trends in relation to spring T followed a sequential pattern, maintaining consistency in the changes across all taxa.

### Annual and spring precipitation

Only seven taxa exhibited significant relationships with precipitation variables (Supplementary Appendix S5). *A. pinsapo* showed earlier F occurrence dates (−0.105 ± 0.026 days/mm) with increasing annual P, however, the same phenophase showed a trend of later occurrence dates (0.127 ± 0.05 days/mm) with the increase of spring P. *Genista hirsuta* subsp. *lanuginosa* showed later occurrence dates of FBF (0.046 ± 0.016 days/mm) with annual P, which was also detected for the spring P (0.169 ± 0.05 days/mm). *Cotoneaster granatensis* exhibited later DVG occurrences (0.047 ± 0.021 days/mm) and *Macrochloa tenacissima* later FBF occurrences (0.035 ± 0.013 days/mm) with the increase of annual P. In contrast, *Hormathophylla spinosa* showed earlier FS occurrences (−0.047 ± 0.019 days/mm)*. Halimium atriplicifolium* displayed later F occurrences with increasing spring P (0.101 ± 0.035 days/mm), whereas *Helichrysum stoechas* showed earlier DVG occurrences (−0.19 ± 0.071 days/mm) with the same variable. Among the affected taxa, three were endemic, and one was considered narrow endemic (Supplementary Table S1). Notably, precipitation variables were never included in the models independently; they were always accompanied by temperature-related variables.

## DISCUSSION

This study presents an analysis of phenological trends from 1822 to 2020 examining the responses of Mediterranean plant taxa in response to changing climatic conditions across different thermotypes and HCI groups, many of which have not been previously studied. A key observation is the internal consistency of phenological trends within species across different phenophases, i.e. sequence of phenophases. This coherence reinforces the reliability of the observed responses and underscores the value of assessing multiple phenophases to accurately characterize species phenological dynamics. Overall, these findings provide a more robust understanding of how climate change affects plant phenology.

### Phenological changes over time

#### Phenological trends in thermotypes over time

The results revealed significant trends of advance over time in flowering and growth. Flowering exhibited a global advancement of 0.87 days/decade, with no apparent effect of thermotype. While we had partially expected differences among thermotypes, since previous studies have reported different patterns of phenological advances along elevational and climatic gradients (Hopkins 1920, Recio et al. 2009, Legave et al. 2015), more recent studies suggest that these altitudinal differences are being masked by the effects of climate change, leading to more uniform phenological responses across elevations (Chen et al. 2018, Vitasse et al. 2018). For example, Vitasse et al. (2018) found a reduction of the ‘Hopkins’ bioclimatic law’ due to the effect of warming winters and late springs. This bioclimatic law, which marks a phenological difference as we ascend (Hopkins 1920), seems to be being altered by climate change, which could explain the lack of differences between thermotypes in the observed flowering trends. Alternatively, this could be translated as a homogeneous phenological trajectory of the advances found in flowering among the thermotypes. This contrasts with Rondinel-Mendoza et al. (2024), who reported significant differences between non-alpine and alpine trends, with stronger advances in the non-alpine group. Their study, conducted in the Sierra Nevada massif, which is included in the Baetic mountain ranges, used a similar methodological approach and a set of closely-related plant species. Using herbarium specimens, they found estimates of flowering advance in non-alpine plants of 3.1 days/decade earlier, and 0.9 days/decade earlier in alpine plants. Although we did not detect differences among elevational groups, our observed average flowering advance of 0.87 days per decade aligns closely with their alpine estimate.

In contrast, the growing phenophase exhibited a significant interaction effect between thermotypes and years. Advances were observed in the mid (MS, but marginally significant) and high (OC) mountain thermotypes, with 1.31 and 4.42 days/decade, respectively, whereas the thermo-Mediterranean (TM) group showed no significant trends. Post-hoc analysis confirmed that TM differed significantly from both MS and OC. These patterns suggest a stronger climate sensitivity in higher elevation or cooler thermotypes for the growing phenophase, potentially reflecting a greater climatic effect in those zones. In this sense, phenological advances in growth appear to reflect differential responses along altitudinal gradients, whereas flowering responds more homogeneously across the territory. This suggests that growth has been less affected than flowering by the attenuation of Hopkins’ bioclimatic law under climate change.

Finally, the absence of significant trends in fruiting (FS) and preflowering (FBF) across all thermotypes may indicate higher plasticity, or alternatively, could result from a possible sampling bias, as most herbarium specimens are concentrated in the flowering (F) phenophase (Daru et al. 2018, Ahlstrand et al. 2025).

#### Phenological trends over time by taxa

Our findings indicate that discernible temporal shifts occur in phenological events over time at the taxon level: taxa advanced by an average of 4.05 days/decade and delayed by an average of 7.69 days/decade among taxa with significant effects across the phenophases (i.e. average of significant slopes of every taxon, when advancing and when delaying separately). Overall, 31% of the studied taxa have shown phenological shifts in relation to time in the Baetic Ranges, where the average significant slopes of advancing taxa in reproductive phenology (FBF, F, and FS) was 3.6 days/decade, while in vegetative phenology (DVG) was 5.6 days/decade. These results, particularly the predominance of advances over delays, matched with other phenological studies of climate change that showed advances of 3.94 days/decade for flowering and leaf unfolding (Menzel et al. 2006b), 5.2 days/decade in flowering (Bock et al. 2014) and 5.9, 3.2 and 4.8 days/decade for flowering, fruiting and leaf unfolding respectively (Gordo and Sanz 2009).

### Relationship of phenology and climatic variables

The significant trends detected in temperature variables provide robust evidence of ongoing climate warming in the Baetic Ranges. Observed increases of 0.27°C/decade in annual T and 0.31°C/decade in spring T closely align with previous findings by Brunet et al. (2006) for the region. Similarly, the detected declines in annual (−33 mm/decade) and spring P (−14 mm/decade) are consistent with precipitation reduction trends reported across Spain by Luna et al. (2012a, 2012b). Given this dual pattern of increasing temperatures and decreasing precipitation in the Baetic mountains, and considering projections for further warming and aridification in the Mediterranean basin, exceeding global averages (Ali et al. 2022), it is reasonable to expect that the phenological responses observed in relation to climatic variables (advances or delays) will ultimately translate into significant long-term phenological shifts over time.

#### Phenological relationships with climate through thermotypes

The phenological responses to climatic drivers confirmed the strong influence of temperature, particularly on flowering. Significant earlier occurrences of F were associated with both annual and spring temperatures, with flowering occurring 5.14 days earlier per °C increased in annual T, and 2.86 days earlier per °C increased in spring T. These advances with warmer temperatures are occurring homogeneously throughout the territory showing no differences among thermotypes, and mirror the temporal trends, reinforcing the conclusion that flowering has become more uniform along the elevational gradient. For the other phenophases (FBF, FS, and DVG), no significant relationships were found. While some models indicated significant main effects of the climatic variables (e.g. FS with annual and spring T), the global slopes were not different from zero, meaning that no consistent shifts in phenology with temperature were detected for these phases. For DVG with spring T, thermotypes differed significantly in their DOY means, but not in their slopes, indicating altitudinal differences in baseline timing rather than in the climatic sensitivity of growth. In contrast, for fruiting (FS), only a marginal effect of thermotype was observed with spring T, which does not provide strong evidence for systematic differences among thermotypes.

Precipitation exerted weaker effects overall. Notably, flowering showed significant main effects with spring P, delaying with wetter springs by 3.4 days per 100 mm, and with annual P, delaying with wetter years by 0.7 days per increased 100 mm. However, given the long-term decline in precipitation in the Baetic Ranges (Luna et al. 2012a, 2012b, and the findings in the 3.2.1 section of this study) implies an indirect trend towards earlier flowering under increasing aridity. Preflowering also showed delays with wetter springs (3.3 days/100 mm), matching with the flowering results, but in the high-mountain bioclimatic group, wetter years were marginally associated with earlier FBF occurrence (−8.33 days/100 mm). These mixed results suggest that early phenophases may be more sensitive to water availability in alpine environments, where snowmelt and precipitation are crucial cues (Rose-Person et al. 2024). However, as these results were marginally significant, interpretations should be made cautiously.

Taken together, the climate-based models reinforce the temporal trends observed in this study: flowering responds strongly and uniformly to temperature, growth retains altitudinal structuring, and preflowering and fruiting show little systematic sensitivity. The reduction of altitudinal differences supports the idea that phenological timing is becoming more homogeneous under climate change (Chen et al. 2018, Vitasse et al. 2018).

#### Phenological relationships with climate by taxa

Most taxa (∼97%) showed significant relationships between their DOY and at least one climatic variable across their phenophases. Temperature again emerged as a primary driver, consistent with previous research emphasizing its influence on developmental processes in plants (Peñuelas et al. 2002, Gordo and Sanz 2009, Vogel 2022). Both annual and spring T showed strong correlations with phenological shifts, especially for middle and high mountain species, with the results for the former group presenting a novel contribution to the field of study. On average, an increase of 1°C in annual T was associated with an earlier occurrence of 4.3 days/°C on average in the vegetative phenophase, while an earlier occurrence of 12.7 days on average was found in the reproductive phenophases, similar to Bock et al. (2014) (9.7 days/°C earlier in the first flowering day). Although phenophase sequencing was generally consistent across taxa, some anomalies were detected in response to annual T. In *C. populifolius*, FBF occurred later, while F and FS occurred earlier. This pattern may reflect an alteration of the chilling and forcing requirements due to recent warming, potentially leading to delays instead of advances in some phenophases (Benmoussa et al. 2017, Wenden et al. 2020). *Crataegus granatensis* displayed a later FS but earlier F occurrence, increasing its reproductive period. This may indicate a positive response to longer periods of optimal temperatures at middle and high elevations, as warmer conditions in colder zones can extend the thermal window for reproduction by lengthening the forcing period (Wenden et al. 2020). *S. glacialis* presented an earlier occurrence of FBF and DVG, but F presented a later occurrence with increasing annual T. This is a high-mountain species that flowers and grows between July and August, with FBF starting 1 month earlier (Supplementary Figures S4–S7; Rivera et al. 1999). One possible explanation for these inconsistencies is that earlier phenophases advance to benefit from earlier summers and snowmelt (Vitasse et al. 2017).

Precipitation-related variables had weaker effects on phenological timing, as previously reported (Doi and Katano 2008). When significant, increased P was associated with later phenophase occurrences, as also observed in Matthews and Mazer (2016) . The relationships found with the climatic variables are something remarkable, since projected climatic changes in the Mediterranean region are expected to exceed those observed over the past century (Giorgi 2006, Alessandri et al. 2014).

### Phenological shifts and implications for the HCI

The findings of our study hold important implications for conservation efforts concerning the Habitats of Community Interest (HCI). The affected taxa of the HCI with orophilous ecology requirements ranged from habitats of different groups (Supplementary Table S3): HCI4 (*Cistus laurifolius* rockrose patches, mountain junipers and orophilous pulvinular thickets), HCI5 (*Rhamno-Prunetalia* communities), HCI6 (Mediterranean siliceous and basophilous high mountain grasslands), and HCI9 (Mediterranean maple forests and Spanish fir forests). These HCI grouped 19 taxa, of which 18 showed earlier phenology occurrence with time or climatic variables, highlighting summer deciduous trees and shrubs such as *Acer granatense*, *Crataegus granatensis* and *Cotoneaster granatensis*. In addition to these, we also considered four orophilous taxa that are associated with HCI encompassing broader ecological ranges (not exclusively orophilous) although the taxa themselves are ecologically restricted to high mountain environments. These were *Crepis oporinoides* and *S. minutum* typical of silicic crags, *C. glareosum* from western Mediterranean and thermophilous landslides, and *Quercus faginea* subp. *alpestris* from gall-oak forest habitats. These taxa showed advances in reproductive phenology (F, FBF, and FS), with the exception of the last one that surprisingly did not show significant trends. The advancing of orophilous taxa seems to be related to earlier snowmelt and the increase of late winter and early spring temperatures. An advance in high mountain habitat is linked to a higher risk to atypical frosts, which potentially increase the damage and mortality of its taxa (Chmielewski et al. 2004, Augspurger 2013), However, Anderson et al. (2012) found that individuals flowering earlier in high mountain conditions could benefit of the entire growing season maximizing fitness compared to individuals with late flowering.

The recent study by Pareja-Bonilla et al. (2025) analysed phenological changes in some thermophilous species; however, their methodological approach differed from ours as they did not use herbarium specimens. In our case, we examined a broader set of thermophilous species, highlighting their relevance to HCI across a nearby, though distinct, study area. The affected taxa with HCI of more thermophilous ecological requirements ranged across several habitat groups (Supplementary Table S4): HCI4 (heathlands and/or thermophilous and xeric rockrose patches), HCI5 (arid and semi-arid shrublands), HCI6 (*M. tenacissima* grasslands), and HCI9 (*Olea*/*Ceratonia* forests, cork oak forests and holm oak forests). These HCI grouped 49 species with thermophilous ecology, of which 39 showed phenological trends (∼80%). Affected taxa ranged from widely distributed tree and shrub species, such as *Quercus rotundifolia* and *Phillyrea angustifolia*, to more restricted range shrub species, such as the narrow endemic *A. serpyllifolium* subsp. *malacitanum*. Within this group of taxa, it is striking that species with similar phylogenetic or ecological characteristics exhibited different phenological trends. For instance, unlike *Q. rotundifolia*, *Quercus suber* did not show significant trends.

The high number of taxa with changes in the scrub-matorral (HCI5) and temperate heat and scrub (HCI4) groups is of concern, as a large part of the vegetation landscape in the Baetic Ranges is composed of these types of communities (Loidi 2017). Scrublands and shrublands encompass one of the highest floristic biodiversity in the study area (REDIAM 2020) and provide shelter and food resources to other species, and structure to other habitats as well (Van Andel et al. 1993). The phenological trends that these habitats are experiencing could impact not only the habitat composition and its diversity but also other types of habitats such as forests, since scrubs and shrublands are often its predecessors (Drury and Nisbet 1973, West et al. 1981, Swanson et al. 2011).

Given the importance of the ecosystem services provided by forest habitats (Morán-Ordóñez et al. 2021), we highlight that six characteristic taxa of this habitat group (HCI9) exhibited phenological advances and delays. A recent vulnerability assessment ranked from low to high the forests in the south of Spain, based on their sensitivity and adaptive capacity to climate change (Hidalgo-Triana et al. 2023b). In the assessment, the HCI of mixed mountain forest was highly vulnerable to climate change. Here, *A. granatense* showed significant advances in the DVG phenophase. Further, the results found could be indicating another potential vulnerability for this habitat. In the same vulnerability assessment, *Q. rotundifolia* and *Q. suber* forests were ranked mid-level in vulnerability; while here *Q. suber* did not show phenological trends, but *Q. rotundifolia* did in patterns of advance in F and DVG, as Alcázar et al. (2024) found in the same area. One of the highest vulnerable HCI assessed in the same study was the pinsapo fir forest HCI; here, the flowering phase of *A. pinsapo* showed a significant delay with annual T increase. However, the decrease of P seems to cause the contrary effect in this species, provoking an earlier flowering. Although, it should be noted that recent studies have discovered unexpected capacities of this species to cope with some climate change challenges (Cortés-Molino et al. 2022); therefore, this habitat could have more countering capacities than we expected at first. The study also reported *Olea* and *Ceratonia* forests as the second least vulnerable HCI with climate change, but here we found phenological trends in both characteristic taxa (*Olea europaea* var. *sylvestris* and *Ceratonia siliqua*), matching with the results of Gordo and Sanz (2005). This is something that would require greater focus of attention in the next few years since these phenological trends lead to greater uncertainty about the future of these habitats.

Results regarding the endemic taxa turn out to be important as they present a very reduced area of distribution across the Mediterranean Basin. Among the 22 endemisms with phenological trends, 11 were narrow endemic. Given that these types of endemisms usually present reduced populations with low genetic flow and a very narrow optimal range of climatic conditions and distribution (Médail and Baumel 2018), these will be highly affected in the face of future climate change scenarios. A big concern highlighted in phenology conservation is the potential mismatch between plant phenology and other trophic levels: primary consumers (granivorous birds or herbivorous insects) and secondary consumers (predatory birds, mammals, insects, etc.) (Thackeray et al. 2016), since many plants exhibit seasonal dependencies and interactions with these trophic groups (Visser and Both 2005, Piao et al. 2019). Alternatively, some interactions with other trophic levels could be beneficial due to a phenological advance. For instance, insect herbivores may have more food resources if young foliage is available longer, increasing food accessibility for insectivorous birds as well (Wood et al. 2012, Wood and Pidgeon 2015), while mammalian herbivores could benefit from an extension of high-quality forage period (Searle et al. 2015). Further, an earlier phenophase occurrence might help to gain a greater amount of carbon in seedlings or young trees (Harrington et al. 1989). Understanding how plant phenology responds to changing climatic conditions in the different thermotypes of the Baetic Ranges is crucial for devising effective conservation strategies and protecting its high biodiversity.

### Limitations of the study

One limitation of using herbarium specimens was the lack of samples in a specific phenophase in some taxa. More than half of the non-analysed taxa were Baetic endemisms, which tend to have fewer available preserved specimens compared to taxa with a broader distribution. Nevertheless, we believe that we have covered most of the available data of these endemisms, given that we collected all their available specimens from the herbaria of Granada, Sevilla, Málaga and Madrid, which are the main herbaria within the study area (except Madrid). Another limitation we found using herbarium specimens was the potential biases of samplers and plant collectors (Daru et al. 2018, Ahlstrand et al. 2025). Collectors often visit sites at the same time of the year, or have scheduled samplings which could alter the randomness and heterogeneity of the data. Besides, collectors usually pick up the specimens when they are flowering rather than fruiting, having less data for this phenophase. The lower number of phenological shifts found in FS compared to other earlier phenophases may also be attributed to a pattern whereby later phenophases seems to be less responsive to warming than earlier ones (Stuble et al. 2021, Rondinel-Mendoza et al. 2024). However, we acknowledge that differences in sample size among taxa could have influenced the results. Another challenge was to correctly identify specimens that were damaged, degraded, or had suffered from herbivory.

While our results provide valuable insights, further research is needed to clarify the specific mechanisms driving phenological shifts. Incorporating additional environmental drivers, such as chilling and forcing requirements or photoperiod, could enhance our understanding of phenological responses. Moreover, including other phenophases, such as leaf fall and senescence, would offer a more comprehensive view of the ongoing phenological shifts in the Baetic Ranges. Furthermore, it is essential to encourage and support herbarium collecting so that future researchers can continue the long-term tracking of phenological trends, particularly for endemic and rare taxa, as well as for those that could not be analysed due to a lack of preserved specimens.

## Supplementary Material

plaf064_Supplementary_Data

## Data Availability

The data underlying this article are available in the article and in its online Supplementary material.

## References

[plaf064-B1] Ahlstrand NI, Primack RB, Austin MW et al The promise of digital herbarium specimens in large-scale phenology research. New Phytol 2025;95:631–6. 10.1111/nph.7017840384489

[plaf064-B2] Aho K, Derryberry D, Peterson T. Model selection for ecologists: the worldviews of AIC and BIC. Ecology 2014;95:631–6. 10.1890/13-1452.1 24804445

[plaf064-B3] Alcázar P, Torres C, De Linares C et al Impacts of climate change on airborne Quercus pollen trends in Andalusia region (southern Spain). Reg Environ Change 2024;24:50. 10.1007/s10113-023-02181-5

[plaf064-B4] Alessandri A, De Felice M, Zeng N et al Robust assessment of the expansion and retreat of Mediterranean climate in the 21st century. Sci Rep 2014;4:7211. 10.1038/srep07211 25448867 PMC4250915

[plaf064-B5] Ali E, Cramer W, Carnicer J et al Cross-chapter paper 4: mediterranean region. In: Pörtner HO, Roberts DC, Tignor M, Poloczanska ES, Mintenbeck K, Alegría A, Craig M, Langsdorf S, Löschke S, Möller V (eds.) Climate Change 2022: Impacts, Adaptation and Vulnerability. Contribution of Working Group II to the Sixth Assessment Report of the Intergovernmental Panel on Climate Change. NY, USA and Cambridge, UK: Cambridge University Press, 2022, 2233–72.

[plaf064-B6] Anderson JT, Inouye DW, McKinney AM et al Phenotypic plasticity and adaptive evolution contribute to advancing flowering phenology in response to climate change. Proc R Soc Lond B Biol Sci 2012;279:3843–52. 10.1098/rspb.2012.1051 PMC3415914 22787021

[plaf064-B7] Armstrong-Herniman W, Greenwood S. The role of winter precipitation as a climatic driver of the spring phenology of five California Quercus species (Fagaceae). Madroño 2021;68:450–460. 10.3120/0024-9637-68.4.450

[plaf064-B8] Augspurger CK . Reconstructing patterns of temperature, phenology, and frost damage over 124 years: spring damage risk is increasing. Ecology 2013;94:41–50. 10.1890/12-0200.1 23600239

[plaf064-B9] Bates D, Mächler M, Bolker B et al Fitting linear mixed-effects models using **lme4**. J Stat Softw 2015;67. 10.18637/jss.v067.i01

[plaf064-B10] Beaubien EG, Johnson DL. Flowering plant phenology and weather in Alberta, Canada. Int J Biometeorol 1994;38:23–7. 10.1007/BF01241800

[plaf064-B11] Benmoussa H, Ghrab M, Ben Mimoun M et al Chilling and heat requirements for local and foreign almond (*Prunus dulcis* mill.) cultivars in a warm Mediterranean location based on 30 years of phenology records. Agric For Meteorol 2017;239:34–46. 10.1016/j.agrformet.2017.02.030

[plaf064-B12] Blanca G, Cabezudo B, Cueto M et al Flora Vascular de Andalucía Oriental (2.ª Edición Corregida y Aumentada). Granada: Universidad de Granada, Almería, Jaén y Málaga, 2011.

[plaf064-B13] Bock A, Sparks TH, Estrella N et al Changes in first flowering dates and flowering duration of 232 plant species on the island of Guernsey. Glob Chang Biol 2014;20:3508–19. 10.1111/gcb.12579 24639048

[plaf064-B14] Braun-Blanquet J . Fitosociologia: Bases Para el Estudio de las Comunidades Vegetales. Madrid: H. Blume Ediciones, 1979.

[plaf064-B15] Brunet M, SaladiÉ O, Jones P et al The development of a new dataset of Spanish daily adjusted temperature series (SDATS) (1850–2003). Int J Climatol 2006;26:1777–802. 10.1002/joc.1338

[plaf064-B16] Calinger KM, Queenborough S, Curtis PS. Herbarium specimens reveal the footprint of climate change on flowering trends across north-central North America. Ecol Lett 2013;16:1037–44. 10.1111/ele.12135 23786499 PMC3806244

[plaf064-B17] Cañadas EM, Fenu G, Peñas J et al Hotspots within hotspots: endemic plant richness, environmental drivers, and implications for conservation. Biol Conserv 2014;170:282–91. 10.1016/j.biocon.2013.12.007

[plaf064-B18] Casimiro-Soriguer F, Pérez Latorre AV, Hidalgo-Triana N et al Catálogo Actualizado de la flora Vascular Endémica de la Cordillera Bética Occidental (Serranía de Ronda, Andalucia, España): Taxones, Biogeografía y Conservación in Situ. Toledo (España): Primer Congr. Esp. Botánica, 2021.

[plaf064-B19] Chen L, Huang JG, Ma Q et al Spring phenology at different altitudes is becoming more uniform under global warming in Europe. Glob Chang Biol 2018;24:3969–75. 10.1111/gcb.14288 29697173

[plaf064-B20] Chmielewski FM, Müller A, Bruns E. Climate changes and trends in phenology of fruit trees and field crops in Germany, 1961–2000. Agric For Meteorol 2004;121:69–78. 10.1016/S0168-1923(03)00161-8

[plaf064-B21] Chytrý M, Tichý L. Diagnostic, constant and dominant species of vegetation classes and alliances of the Czech republic: a statistical revision. Folia Facultatis Scientiarum Naturalium Universitatis Masarykianae Brunensis Biologia 2003;108.

[plaf064-B22] Chytrý M, Tichý L, Hennekens SM et al EUNIS habitat classification: expert system, characteristic species combinations and distribution maps of European habitats. Appl Veg Sci 2020;23:648–75. 10.1111/avsc.12519

[plaf064-B23] Cleland E, Chuine I, Menzel A et al Shifting plant phenology in response to global change. Trends Ecol Evol 2007;22:357–65. 10.1016/j.tree.2007.04.003 17478009

[plaf064-B24] Cortés-Molino Á, Linares JC, Viñegla B et al Unexpected resilience in relict Abies pinsapo Boiss forests to dieback and mortality induced by climate change. Front Plant Sci 2022;13:991720. 10.3389/fpls.2022.991720 36618643 PMC9822712

[plaf064-B25] Council Directive 92/43/EEC . Council Directive 92/43/EEC of 21 May 1992 on the conservation of natural habitats 693 and of wild fauna and flora. Official Journal of the European Union 1992;206:694.

[plaf064-B26] Daru BH, Park DS, Primack RB et al Widespread sampling biases in herbaria revealed from large-scale digitization. New Phytol 2018;217:939–55. 10.1111/nph.14855 29083043

[plaf064-B27] Davis CC, WilliS CG, Connolly B et al Herbarium records are reliable sources of phenological change driven by climate and provide novel insights into species’ phenological cueing mechanisms. Am J Bot 2015;102:1599–609. 10.3732/ajb.1500237 26451038

[plaf064-B28] Del Río S, Álvarez-Esteban R, Cano E et al Potential impacts of climate change on habitat suitability of Fagus sylvatica L. forests in Spain. Plant Biosystems—An International Journal Dealing with all Aspects of Plant Biology 2018;152:1205–13. 10.1080/11263504.2018.1435572

[plaf064-B29] De Oliveira CC, Zandavalli RB, De Lima ALA et al Functional groups of woody species in semi-arid regions at low latitudes. Austral Ecol 2015;40:40–9. 10.1111/aec.12165

[plaf064-B30] Doi H, Katano I. Phenological timings of leaf budburst with climate change in Japan. Agric For Meteorol 2008;148:512–6. 10.1016/j.agrformet.2007.10.002

[plaf064-B31] Drury WH, Nisbet CT. Succession. J Arnold Arbor 1973;54:331–68. http://www.jstor.org/stable/43781773

[plaf064-B32] Dunnell KL, Travers SE. Shifts in the flowering phenology of the northern great plains: patterns over 100 years. Am J Bot 2011;98:935–45. 10.3732/ajb.1000363 21613073

[plaf064-B33] Estiarte M, Peñuelas J. Alteration of the phenology of leaf senescence and fall in winter deciduous species by climate change: effects on nutrient proficiency. Glob Chang Biol 2015;21:1005–17. 10.1111/gcb.12804 25384459

[plaf064-B34] European Commission, DG-ENV . Interpretation Manual of European Union Habitats, version EUR 28. 2013.

[plaf064-B35] Gallagher RV, Hughes L, Leishman MR. Phenological trends among Australian alpine species: using herbarium records to identify climate-change indicators. Australian Journal of Botany 2009;57:1–9. 10.1071/BT08051

[plaf064-B36] Giménez E, Melendo M, Valle F et al Endemic flora biodiversity in the south of the Iberian Peninsula: altitudinal distribution, life forms and dispersal modes. Biodivers Conserv 2004;13:2641–60. 10.1007/s10531-004-2140-7

[plaf064-B37] Giorgi F . Climate change hot-spots. Geophys Res Lett 2006;33:1–4. 10.1029/2006GL025734

[plaf064-B38] Gordo O, Sanz JJ. Phenology and climate change: a long-term study in a Mediterranean locality. Oecologia 2005;146:484–95. 10.1007/s00442-005-0240-z 16170564

[plaf064-B39] Gordo O, Sanz JJ. Long-term temporal changes of plant phenology in the western Mediterranean. Glob. Change Biol 2009;15:1930–48. 10.1111/j.1365-2486.2009.01851.x

[plaf064-B40] Harrington RA, Brown BJ, Reich PB et al Ecophysiology of exotic and native shrubs in southern Wisconsin: II. Annual growth and carbon gain. Oecologia 1989;80:368–73. 10.1007/BF00379038 28312064

[plaf064-B41] Hidalgo-Triana N, Casimiro-Soriguer Solanas F, Solakis Tena A et al Assessment protocol to evaluate the degree of conservation of habitats of community interest: a case study for the 5220* HCI in the westernmost localities of Europe. Land (Basel) 2023a;12:190. 10.3390/land12010190

[plaf064-B42] Hidalgo-Triana N, Solakis A, Casimiro-Soriguer F et al The high climate vulnerability of western Mediterranean forests. Sci Total Environ 2023b;895:164983. 10.1016/j.scitotenv.2023.164983 37353024

[plaf064-B43] Hopkins AD . The bioclimatic law. J. Wash. Acad. 1920;10:34–40.

[plaf064-B44] Inouye DW . Effects of climate change on phenology, frost damage, and floral abundance of montane wildflowers. Ecology 2008;89:353–62. 10.1890/06-2128.1 18409425

[plaf064-B45] Intergovernmental Panel on Climate Change (IPCC) . Climate Change 2021 – The Physical Science Basis: Working Group I Contribution to the Sixth Assessment Report of the Intergovernmental Panel on Climate Change. Cambridge University Press, 2023.

[plaf064-B46] Jin H, Jönsson AM, Olsson C et al New satellite-based estimates show significant trends in spring phenology and complex sensitivities to temperature and precipitation at northern European latitudes. Int J Biometeorol 2019;63:763–75. 10.1007/s00484-019-01690-5 30805728

[plaf064-B47] Jones CA, Daehler CC. Herbarium specimens can reveal impacts of climate change on plant phenology; a review of methods and applications. PeerJ 2018;6:e4576. 10.7717/peerj.4576 29632745 PMC5888139

[plaf064-B48] Keeling CD, Chin JFS, Whorf TP. Increased activity of northern vegetation inferred from atmospheric CO_2_ measurements. Nature 1996;382:146–9. 10.1038/382146a0

[plaf064-B49] Kottek M, Grieser J, Beck C et al World Map of the Köppen-Geiger climate classification updated. Meteorologische Zeitschrift 2006;15:259–63. 10.1127/0941-2948/2006/0130

[plaf064-B50] Kuznetsova A, Brockhoff PB, Christensen RHB. lmerTest package: tests in linear mixed effects models. J Stat Softw 2017;82:1–26. 10.18637/jss.v082.i13

[plaf064-B51] Legave JM, Guédon Y, Malagi G et al Differentiated responses of apple tree floral phenology to global warming in contrasting climatic regions. Front Plant Sci 2015;6:1054. 10.3389/fpls.2015.01054 26697028 PMC4678210

[plaf064-B52] Leys C, Ley C, Klein O et al Detecting outliers: do not use standard deviation around the mean, use absolute deviation around the median. J Exp Soc Psychol 2013;49:764–6. 10.1016/j.jesp.2013.03.013

[plaf064-B53] Lieth H . Purposes of a phenology book. In: Lieth H (ed.) Phenology and Seasonality Modeling, Ecological Studies. Berlin, Heidelberg: Springer, 1974, 3–19. 10.1007/978-3-642-51863-8_1

[plaf064-B54] Loidi J . The Vegetation of the Iberian Peninsula, Plant and Vegetation. Cham.: Springer, 2017.

[plaf064-B55] Love NLR, Park IW, Mazer SJ. A new phenological metric for use in pheno-climatic models: a case study using herbarium specimens of *Streptanthus tortuosus*. Appl Plant Sci 2019;7:e11276. 10.1002/aps3.11276 31346508 PMC6636619

[plaf064-B56] Lüdecke D, Ben-Shachar M, Patil I et al performance: An R package for assessment, comparison and testing of statistical models. J Open Source Softw 2021;6:3139. 10.21105/joss.03139

[plaf064-B57] Luedeling E, Schiffers K, Fohrmann T et al PhenoFlex—an integrated model to predict spring phenology in temperate fruit trees. Agric For Meteorol 2021;307:108491. 10.1016/j.agrformet.2021.108491

[plaf064-B58] Luna MY, Guijarro JA, López JA. A monthly precipitation database for Spain (1851–2008): reconstruction, homogeneity and trends. Adv Sci Res 2012a;8:1–4. 10.5194/asr-8-1-2012

[plaf064-B59] Luna MY, López JA, Guijarro JA. Tendencias observadas en España en precipitación y temperatura. Rev. Esp. Física 2012b;26:12–7.

[plaf064-B60] Maldonado A, Somoza L, Pallarés L. The Betic orogen and the Iberian–African boundary in the Gulf of Cadiz: geological evolution (central North Atlantic). Mar. Geol 1999;155:9–43. 10.1016/S0025-3227(98)00139-X

[plaf064-B61] Marra G, Wood SN. Practical variable selection for generalized additive models. Comput Stat Data Anal 2011;55:2372–87. 10.1016/j.csda.2011.02.004

[plaf064-B62] Matthews ER, Mazer SJ. Historical changes in flowering phenology are governed by temperature × precipitation interactions in a widespread perennial herb in western North America. New Phytol 2016;210:157–67. 10.1111/nph.13751 26595165

[plaf064-B63] Mazer SJ, Gerst KL, Matthews ER et al Species-specific phenological responses to winter temperature and precipitation in a water-limited ecosystem. Ecosphere 2015;6:art98. 10.1890/ES14-00433.1

[plaf064-B64] Médail F, Baumel A. Using phylogeography to define conservation priorities: the case of narrow endemic plants in the Mediterranean basin hotspot. Biol Conserv 2018;224:258–66. 10.1016/j.biocon.2018.05.028

[plaf064-B65] Medail F, Quezel P. Hot-spots analysis for conservation of plant biodiversity in the Mediterranean basin. Ann Mo Bot Gard 1997;84:112. 10.2307/2399957

[plaf064-B66] Medail F, Quezel P. Biodiversity hotspots in the Mediterranean basin: setting global conservation priorities. Conserv Biol 1999;13:1510–3. 10.1046/j.1523-1739.1999.98467.x

[plaf064-B67] Mendes SB, Olesen JM, Timóteo S et al Fruiting phenology matters. Plants People Planet 2023;5:324–8. 10.1002/ppp3.10359

[plaf064-B68] Menzel A, Sparks TH, Estrella N et al Altered geographic and temporal variability in phenology in response to climate change: phenological variability. Glob Ecol Biogeogr 2006a;15:498–504. 10.1111/j.1466-822X.2006.00247.x

[plaf064-B69] Menzel A, Sparks TH, Estrella N et al European phenological response to climate change matches the warming pattern. Glob Chang Biol 2006b;12:1969–76. 10.1111/j.1365-2486.2006.01193.x

[plaf064-B70] Menzel A, Yuan Y, Matiu M et al Climate change fingerprints in recent European plant phenology. Glob Chang Biol 2020;26:2599–612. 10.1111/gcb.15000 31950538

[plaf064-B71] Mestre I, Casado MJ, Rodríguez E. Tendencias observadas y proyecciones de cambio climático sobre España. In: Los Bosques y La Biodiversidad Frente al Cambio Climático: Impactos, Vulnerabilidad y Adaptación En España. Madrid (España): Ministerio de Agricultura, Alimentación y Medio Ambiente, Secretaría General Técnica, Centro de Publicaciones, 2015, 87–98.

[plaf064-B72] Milla R, Castro-Díez P, Montserrat-Martí G. Phenology of Mediterranean woody plants from NE Spain: synchrony, seasonality, and relationships among phenophases. Flora—Morphology, Distribution, Functional Ecology of Plants 2010;205:190–9. 10.1016/j.flora.2009.01.006

[plaf064-B73] Miller-Rushing AJ, Primack RB, Primack D et al Photographs and herbarium specimens as tools to document phenological changes in response to global warming. Am J Bot 2006;93:1667–74. 10.3732/ajb.93.11.1667 21642112

[plaf064-B74] Morán-Ordóñez A, Ramsauer J, Coll L et al Ecosystem services provision by Mediterranean forests will be compromised above 2°C warming. Glob Chang Biol 2021;27:4210–22. 10.1111/gcb.15745 34231282 PMC13420784

[plaf064-B75] Morellato LPC, Alberton B, Alvarado ST et al Linking plant phenology to conservation biology. Biol Conserv 2016;195:60–72. 10.1016/j.biocon.2015.12.033

[plaf064-B76] Myneni RB, Keeling CD, Tucker CJ et al Increased plant growth in the northern high latitudes from 1981 to 1991. Nature 1997;386:698–702. 10.1038/386698a0

[plaf064-B77] Navarro T, Cabezudo B. Estrategias fenomorfologicas de especies de un matorral mediterráneo (Andalucía, España). Acta Botanica Malacitana 1998;23:133–48. 10.24310/abm.v23i0.8556

[plaf064-B78] Navarro T, Hidalgo-Triana N. Variations in leaf traits modulate plant vegetative and reproductive phenological sequencing across arid Mediterranean shrublands. Front Plant Sci 2021;12:708367. 10.3389/fpls.2021.708367 34497623 PMC8420881

[plaf064-B79] Navarro T, Nieto JM, Pérez Latorre AV et al Estudios fenomorfológicos en la vegetación del sur de España. III: comportamiento estacional de una comunidad de badlands (Tabernas, Almería, España). Acta Botanica Malacitana 1993;18:189–98. 10.24310/abm.v18i.8993

[plaf064-B80] Nelder JA, Wedderburn RWM. Generalized linear models. J R Stat Soc Ser A 1972;135:370. 10.2307/2344614

[plaf064-B81] Orshan G . Plant Pheno-Morphological Studies in Mediterranean Type Ecosystems, Geobotany. Boston: Kluwer Academic, 1989.

[plaf064-B82] Panchen ZA, Primack RB, Aniśko T et al Herbarium specimens, photographs, and field observations show Philadelphia area plants are responding to climate change. Am J Bot 2012;99:751–6. 10.3732/ajb.1100198 22447982

[plaf064-B83] Pareja-Bonilla D, Arista M, Morellato LPC et al Better soon than never: climate change induces strong phenological reassembly in the flowering of a Mediterranean shrub community. Ann Bot 2025;135:239–54. 10.1093/aob/mcad193 38099507 PMC11805945

[plaf064-B84] Parmesan C, Yohe G. A globally coherent fingerprint of climate change impacts across natural systems. Nature 2003;421:37–42. 10.1038/nature01286 12511946

[plaf064-B85] Pau S, Wolkovich EM, Cook BI et al Predicting phenology by integrating ecology, evolution and climate science. Glob Chang Biol 2011;17:3633–43. 10.1111/j.1365-2486.2011.02515.x

[plaf064-B86] Peel MC, Finlayson BL, McMahon TA. Updated world map of the Köppen-Geiger climate classification. Hydrol Earth Syst Sci 2007;11:1633–44.

[plaf064-B87] Peñuelas J, Filella I, Comas PE. Changed plant and animal life cycles from 1952 to 2000 in the Mediterranean region. Glob Chang Biol 2002;8:531–44. 10.1046/j.1365-2486.2002.00489.x

[plaf064-B88] Piao S, Liu Q, Chen A et al Plant phenology and global climate change: current progresses and challenges. Glob Chang Biol 2019;25:1922–40. 10.1111/gcb.14619 30884039

[plaf064-B89] Picornell Antonio, Smith Matt, Rojo Jesús. Climate change related phenological decoupling in species belonging to the Betulaceae family. Int J Biometeorol 2023;67:195–209. 10.1007/s00484-022-02398-9 36308550

[plaf064-B90] Primack D, Imbres C, Primack RB et al Herbarium specimens demonstrate earlier flowering times in response to warming in Boston. Am J Bot 2004;91:1260–4. 10.3732/ajb.91.8.1260 21653483

[plaf064-B91] Primack RB, Miller-Rushing AJ. The role of botanical gardens in climate change research. New Phytol 2009;182:303–13. 10.1111/j.1469-8137.2009.02800.x 19338634

[plaf064-B92] Prisco I, Carboni M, Acosta ATR. The fate of threatened coastal dune habitats in Italy under climate change scenarios. PLoS One 2013;8:e68850. 10.1371/journal.pone.0068850 23874787 PMC3706318

[plaf064-B93] Rathcke B, Lacey EP. Phenological patterns of terrestrial plants. Annu Rev Ecol Syst 1985;16:179–214. 10.1146/annurev.es.16.110185.001143

[plaf064-B94] R Core Team . R: A language and environment for statistical computing. R Foundation for Statistical Computing. Austria. URL, Vienna. 2021. https://www.R-project.org/

[plaf064-B95] Recio M, Rodríguez-Rajo FJ, Jato MV et al The effect of recent climatic trends on Urticaceae pollination in two bioclimatically different areas in the Iberian Peninsula: Malaga and Vigo. Clim Change 2009;97:215–28. 10.1007/s10584-009-9620-4

[plaf064-B96] REDIAM . Red de Información Ambiental de Andalucía, 2023. https://www.juntadeandalucia.es/medioambiente/portal/acceso-rediam.

[plaf064-B97] REDIAM . Guía de Identificación de Hábitats de Interés Comunitario en Andalucía. Andalucía (España): Consejería de Agricultura, Ganadería, Pesca y Desarrollo Sostenible, Junta de Andalucía, 2020.

[plaf064-B98] REDIAM . Informe de Medio Ambiente en Andalucía Edición 2024. Consejería de Sostenibilidad y Medio Ambiente. Andalucía (España): Junta de Andalucía, 2024.

[plaf064-B99] Rivas-Martínez S, Asensi A, Díez-Garretas B et al Biogeographical synthesis of Andalusia (southern Spain). Journal Biogeogr 1997;24:915–28. 10.1046/j.1365-2699.1997.00149.x

[plaf064-B100] Rivas-Martínez S, Asensi A, Díez-Garretas B et al Mapa de series, geoseries y geopermaseries de vegetación de España, Memoria del mapa de vegetación potencial de España. Itinera Geobot 2007;17:5–436.

[plaf064-B101] Rivas-Martínez S, Sáenz SR, Penas A. Worldwide bioclimatic classification system. Glob. Geobot 2011;1:1–634. 10.5616/gg110001

[plaf064-B102] Rivera D, Obon C, Alcaraz F et al Systematics of the high mountain taxa of the genus Sideritis L. section Sideritis, subsection fruticulosae Obón & D. Rivera (Lamiaceae). Bot J Linn Soc 1999;129:249–65. 10.1111/j.1095-8339.1999.tb00504.x

[plaf064-B103] Rodrigo FS, Esteban-Parra MJ, Pozo-Vázquez D et al A 500-year precipitation record in southern Spain. Int J Climatol 1999;19:1233–53. 10.1002/(SICI)1097-0088(199909)19:11

[plaf064-B104] Rondinel-Mendoza KV, Lorite J, Marín-Rodulfo M et al Tracking phenological changes over 183 years in endemic species of a Mediterranean mountain (Sierra Nevada, SE Spain) using herbarium specimens. Plants 2024;13:522. 10.3390/plants13040522 38498521 PMC10892450

[plaf064-B105] Rose-Person A, Spasojevic MJ, Forrester C et al Early snowmelt advances flowering phenology and disrupts the drivers of pollinator visitation in an alpine ecosystem. Alp Bot 2024;134:141–50. 10.1007/s00035-024-00315-x

[plaf064-B106] Schwartz MD . Detecting the onset of spring: a possible application of phenological models. Clim Res 1990;1:23–9. 10.3354/cr001023

[plaf064-B107] Schwartz MD . Monitoring global change with phenology: the case of the spring green wave. Int J Biometeorol 1994;38:18–22. 10.1007/BF01241799

[plaf064-B108] Schwartz MD . Phenology: an Integrative Environmental Science, Tasks for Vegetation Science. Dordrecht, Netherlands: Springer, 2003. 10.1007/978-94-007-0632-3

[plaf064-B109] Searle KR, Rice MB, Anderson CR et al Asynchronous vegetation phenology enhances winter body condition of a large mobile herbivore. Oecologia 2015;179:377–91. 10.1007/s00442-015-3348-9 26009244

[plaf064-B110] Solakis-Tena A, Hidalgo-Triana N, Boynton R et al Phenological shifts since 1830 in 29 native plant species of California and their responses to historical climate change. Plants 2025;14:843. 10.3390/plants14060843 40265755 PMC11945038

[plaf064-B111] Sparks TH, Carey PD. The responses of species to climate over two centuries: an analysis of the Marsham phenological record, 1736–1947. J Ecol 1995;83:321. 10.2307/2261570

[plaf064-B112] Stuble KL, Bennion LD, Kuebbing SE. Plant phenological responses to experimental warming—a synthesis. Glob Chang Biol 2021;27:4110–24. 10.1111/gcb.15685 33993588

[plaf064-B113] Swanson ME, Franklin JF, Beschta RL et al The forgotten stage of forest succession: early-successional ecosystems on forest sites. Front Ecol Environ 2011;9:117–25. 10.1890/090157

[plaf064-B114] Tang J, Körner C, Muraoka H et al Emerging opportunities and challenges in phenology: a review. Ecosphere 2016;7:e01436. 10.1002/ecs2.1436

[plaf064-B115] Thackeray S, Henrys P, Hemming D et al Phenological sensitivity to climate across taxa and trophic levels. Nature 2016;535:241–5. 10.1038/nature18608 27362222

[plaf064-B116] Van Andel J, Bakker JP, Grootjans AP. Mechanisms of vegetation succession: a review of concepts and perspectives. Acta Botanica Neerlandica 1993;42:413–33. 10.1111/j.1438-8677.1993.tb00718.x

[plaf064-B117] Visser ME, Both C. Shifts in phenology due to global climate change: the need for a yardstick. Proc R Soc Lond B Biol Sci 2005;272:2561–9. 10.1098/rspb.2005.3356 PMC1559974 16321776

[plaf064-B118] Vitasse Y, Rebetez M, Filippa G et al Hearing’ alpine plants growing after snowmelt: ultrasonic snow sensors provide long-term series of alpine plant phenology. Int J Biometeorol 2017;61:349–61. 10.1007/s00484-016-1216-x 27539023

[plaf064-B119] Vitasse Y, Signarbieux C, Fu YH. Global warming leads to more uniform spring phenology across elevations. Proceedings of the National Academy of Sciences 2018;115:1004–8. 10.1073/pnas.1717342115 PMC5798366 29279381

[plaf064-B120] Vogel J . Drivers of phenological changes in Southern Europe. Int J Biometeorol 2022;66:1903–14. 10.1007/s00484-022-02331-0 35882643 PMC9418088

[plaf064-B121] VV.AA . Bases Ecológicas Preliminares Para la Conservación de los Tipos de Hábitat de Interés Comunitario en España. Madrid: Ministerio de Medio Ambiente, Rural y Marino, 2009.

[plaf064-B122] Wang J, Liu D, Ciais P et al Decreasing rainfall frequency contributes to earlier leaf onset in northern ecosystems. Nat Clim Chang 2022;12:386–92. 10.1038/s41558-022-01285-w

[plaf064-B123] Wenden B, Mariadassou M, Chmielewski F et al Shifts in the temperature-sensitive periods for spring phenology in European beech and pedunculate oak clones across latitudes and over recent decades. Glob Chang Biol 2020;26:1808–19. 10.1111/gcb.14918 31724292

[plaf064-B124] West DC, Shugart HH, Botkin DB. Forest Succession: Concepts and Application. New York: Springer, 1981.

[plaf064-B125] Westman WE . Seasonal dimorphism of foliage in Californian coastal sage scrub. Oecologia 1981;51:385–8. 10.1007/BF00540910 28310024

[plaf064-B126] Willis CG, Ellwood ER, Primack RB et al Old plants, new tricks: phenological research using herbarium specimens. Trends Ecol Evol 2017;32:531–46. 10.1016/j.tree.2017.03.015 28465044

[plaf064-B127] Wood SN . Generalized additive models: an introduction with R. In: Chapman and Hall/CRC Texts in Statistical Science, 2nd edn. Boca Raton: CRC Press/Taylor & Francis Group, 2017, 1–496.

[plaf064-B128] Wood EM, Pidgeon AM. Extreme variations in spring temperature affect ecosystem regulating services provided by birds during migration. Ecosphere 2015;6:1–16. 10.1890/ES15-00397.1

[plaf064-B129] Wood EM, Pidgeon AM, Liu F et al Birds see the trees inside the forest: the potential impacts of changes in forest composition on songbirds during spring migration. For Ecol Manage 2012;280:176–86. 10.1016/j.foreco.2012.05.041

