# Supplementary material for: Retrospective phenology in western Mediterranean plants: revealing climate change patterns through herbarium specimens
Source: AoB Plants. 2025 Nov 3;17(6):plaf064. doi: 10.1093/aobpla/plaf064 (PMC12611260; doi:10.1093/aobpla/plaf064)

Supporting Information for

Retrospective phenology in western Mediterranean plants: revealing climate change patterns through herbarium specimens

APPENDIX S1 – DOY with Time – Thermotypes

Contents

1. LMM results – Type II ANOVA

2. LMM estimated slopes

3. Model diagnostics

1. LMM results – Type II ANOVA

Table S1. Type II ANOVA results for fixed effects: time (Year_decim), thermotypes (BioclimGroup) and their interaction by phenophase. FBF: preflowering, F: flowering, FS: fruiting, DVG: growth.

Phenophase	Effect	Sum Sq	Mean Sq	df1	df2	F	p_value
FBF	Year_decim	212.264	212.264	1.000	1305.190	0.215	0.643
FBF	BioclimGroup	3015.598	1507.799	2.000	1295.727	1.526	0.218
FBF	Year_decim:BioclimGroup	2638.737	1319.369	2.000	1293.877	1.336	0.263
F	Year_decim	11424.364	11424.364	1.000	2730.423	15.026	0.000
F	BioclimGroup	3357.466	1678.733	2.000	2724.701	2.208	0.110
F	Year_decim:BioclimGroup	2453.193	1226.597	2.000	2722.410	1.613	0.199
FS	Year_decim	2108.099	2108.099	1.000	684.430	1.818	0.178
FS	BioclimGroup	889.877	444.938	2.000	662.157	0.384	0.682
FS	Year_decim:BioclimGroup	717.564	358.782	2.000	661.880	0.309	0.734
DVG	Year_decim	925.310	925.310	1.000	1644.242	0.832	0.362
DVG	BioclimGroup	15329.079	7664.540	2.000	1645.036	6.891	0.001
DVG	Year_decim:BioclimGroup	13937.844	6968.922	2.000	1644.808	6.265	0.002

2. LMM estimated slopes

Table S2 Global slopes of DOY trends over time (Year_decim) for each phenophase (LMM estimates). FBF: preflowering, F: flowering, FS: fruiting, DVG: growth.

Phenophase	Global_Slope (days/year)	SE	df	lower.CL	upper.CL	t.ratio	p_value	direction
FBF	-0.004	0.053	1302.456	-0.108	0.099	-0.085	0.932	Advance
F	-0.087	0.027	2737.292	-0.140	-0.034	-3.207	0.001	Advance
FS	-0.061	0.052	685.562	-0.163	0.041	-1.175	0.240	Advance
DVG	-0.004	0.048	1640.124	-0.098	0.091	-0.075	0.940	Advance

Table S3. Estimated slopes of DOY trends by thermotype (BioclimGroup)and phenophase from emtrends results. TM: thermo-Mediterranean, MS: meso-supra-Mediterranean, OC: oro-cryoro-Mediterranean. FBF: preflowering, F: flowering, FS: fruiting, DVG: growth.

Phenophase	BioclimGroup	Slope (days/year)	SE	df	lower.CL	upper.CL	t.ratio	p_value	direction
FBF	TM	0.039	0.073	1280.408	-0.105	0.182	0.526	0.599	Delay
FBF	MS	-0.042	0.077	1320.684	-0.193	0.109	-0.547	0.584	Advance
FBF	OC	-0.257	0.170	1297.437	-0.591	0.077	-1.511	0.131	Advance
F	TM	-0.049	0.040	2729.236	-0.128	0.031	-1.202	0.230	Advance
F	MS	-0.116	0.040	2735.034	-0.195	-0.038	-2.906	0.004	Advance
F	OC	-0.168	0.057	2735.722	-0.280	-0.056	-2.943	0.003	Advance
FS	TM	-0.072	0.097	668.628	-0.263	0.119	-0.739	0.460	Advance
FS	MS	-0.033	0.071	693.010	-0.172	0.106	-0.468	0.640	Advance

FS	OC	-0.121	0.088	646.253	-0.293	0.051	-1.383	0.167	Advance
DVG	TM	0.124	0.071	1629.740	-0.015	0.263	1.743	0.081	Delay
DVG	MS	-0.131	0.067	1651.825	-0.263	0.000	-1.956	0.051	Advance
DVG	OC	-0.442	0.172	1653.998	-0.780	-0.104	-2.563	0.010	Advance

Table S4. Pairwise comparisons of slopes between thermotypes (post-hoc contrasts) in the phenophases with significant interaction effect. TM: thermo-Mediterranean, MS: meso-supra-Mediterranean, OC: oro-cryoro-Mediterranean. DVG: growth.

Phenophase	contrast	estimate	SE	df	t.ratio	p_value	Interpretation
DVG	TM - MS	0.255	0.098	1641.643	2.609	0.025	TM vs MS: different slopes
DVG	TM - OC	0.566	0.186	1651.472	3.034	0.007	TM vs OC: different slopes
DVG	MS - OC	0.311	0.184	1651.036	1.687	0.210	MS vs OC: ns

3. Model diagnostics

3.1. Residuals for FBF phenophase over time

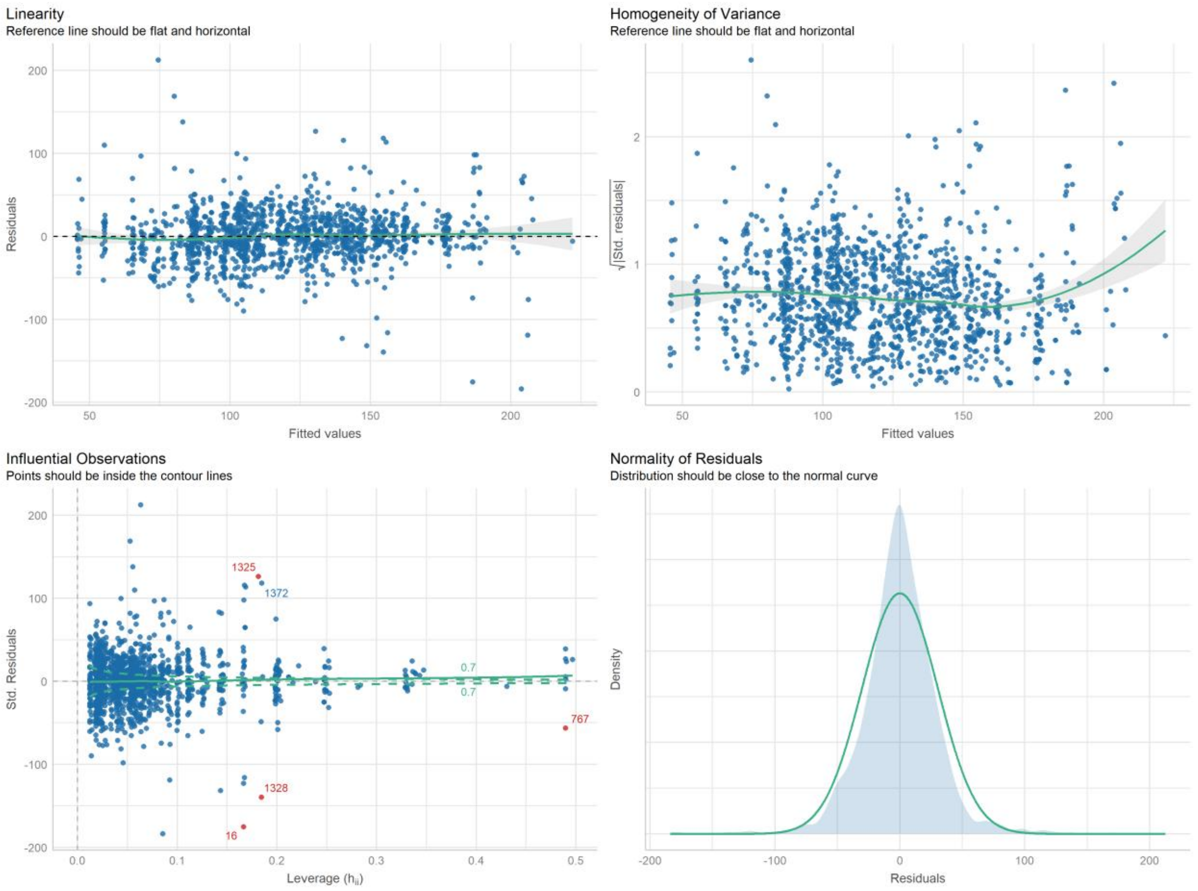

3.2. Residuals for F phenophase over time

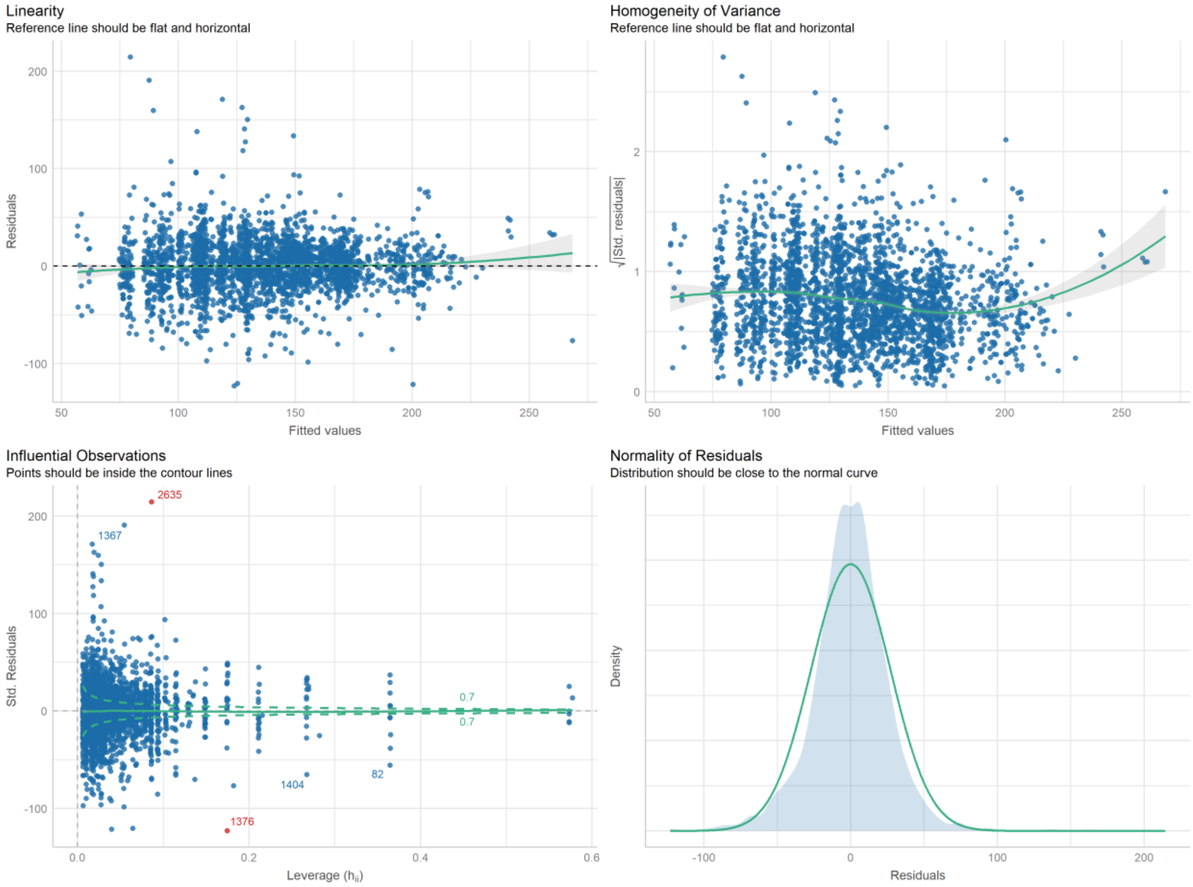

3.3. Residuals for FS phenophase over time

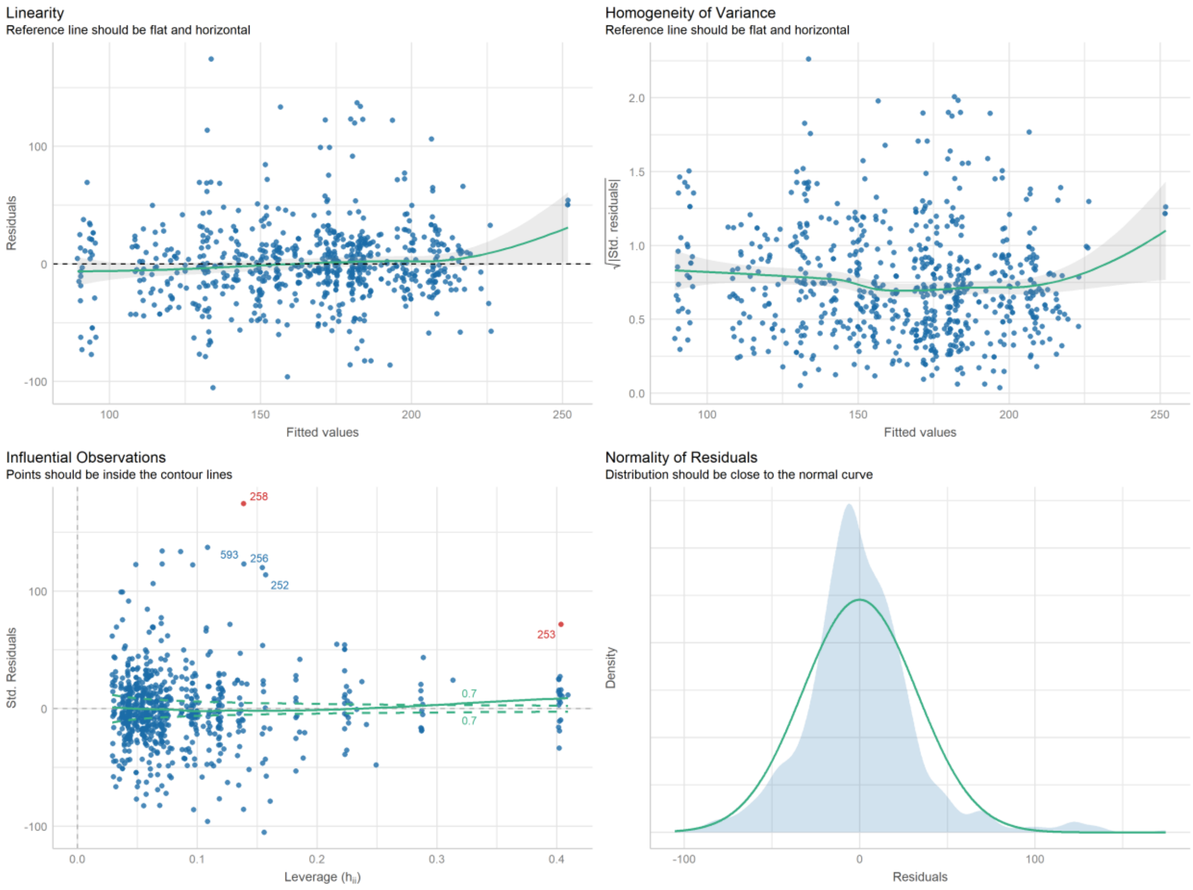

3.4. Residuals for DVG phenophase over time

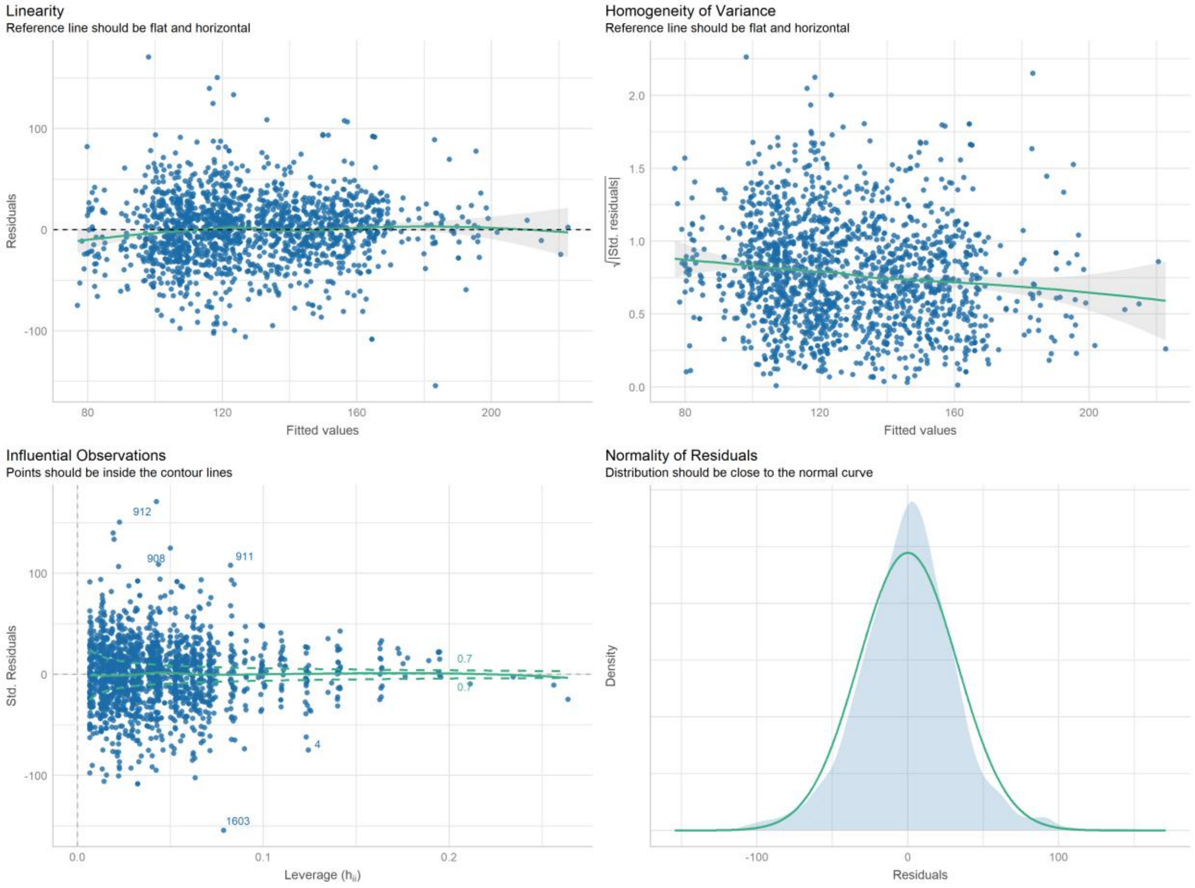

Supplement: plaf064_Supplementary_Data [file plaf064_supplementary_data.zip › Appendix_S1.pdf]
