# Supplementary material for: Retrospective phenology in western Mediterranean plants: revealing climate change patterns through herbarium specimens
Source: AoB Plants. 2025 Nov 3;17(6):plaf064. doi: 10.1093/aobpla/plaf064 (PMC12611260; doi:10.1093/aobpla/plaf064)
Supplement: plaf064_Supplementary_Data [file plaf064_supplementary_data.zip › Appendix_S2.pdf]

Supporting Information for

Retrospective phenology in western Mediterranean plants: revealing climate change patterns through herbarium specimens

APPENDIX S2 – DOY with Time – Taxa

Contents

1. LM Model Results by taxa with diagnostics

2. GLM Model Results by taxa with diagnostics

3. AIC comparison between GLMs and GAMs

1. LM Model Results

Table S1. Linear Model results by taxon and phenophase of DOY over time. Preflowering: FBF, flowering: F, fruiting: FS, growth: DVG. BP test: studentized Breusch-Pagan Test. KS: Kolmogorov-Smirnov test. SW: Saphiro-Wilk test.

Phenophase	Taxon	Intercept	Slope	SE	R^2	Adj.R^2	p value	Normality_p value	BPTest	TestUsed
F	*Phlomis purpurea*	-30.01061	0.072902192	0.08011756	0.005180515	-0.001076211	0.364231328	0.90069586	0.22967371	KS
F	*Phillyrea angustifolia*	119.96394	-0.011945902	0.26535445	0.000061400	-0.030239759	0.964363756	0.35451801	0.03985798	SW

**Phenophase**	**Taxon**	**Intercept**	**Slope**	**SE**	**R^2**	**Adj.R^2**	**p value**	**Normality_p value**	**BPTest**	**TestUsed**
F	*Salvia rosmarinus*	3,118.35304	-1.475887437	1.67488999	0.032657747	-0.009400612	0.387333781	0.01049385	0.07550466	SW
F	*Glandora prostrata subsp. lusitanica*	-134.22772	0.116374031	0.29722042	0.005084169	-0.028079692	0.698164650	0.71900734	0.53802178	SW
F	*Cistus albidus*	-1,037.97039	0.568603482	0.36056577	0.041113936	0.024581418	0.120240284	0.49624160	0.06284971	KS
F	*Rhamnus alaternus*	-61.60322	0.069836702	0.24062210	0.002001599	-0.021760268	0.773065482	0.11292456	0.97103418	SW
F	*Ulex parviflorus*	2,149.55058	-1.026253066	0.59228552	0.069816093	0.046561495	0.090853852	0.03615786	0.51520084	SW
F	*Juniperus oxycedrus*	-520.99612	0.422457924	0.27659640	0.104455443	0.059678215	0.142336390	0.16477880	0.35427550	SW
F	*Helichrysum stoechas*	-319.38986	0.226363773	0.17024820	0.017719771	0.007696503	0.186733349	0.71457998	0.41923071	KS
F	*Ceratonia siliqua*	395.83617	-0.046198435	0.23009177	0.002680374	-0.063807601	0.843565622	0.18812297	0.17451157	SW

**Phenophase**	**Taxon**	**Intercept**	**Slope**	**SE**	**R^2**	**Adj.R^2**	**p value**	**Normality_p value**	**BPTest**	**TestUsed**
F	*Lavandula stoechas*	-363.88219	0.235059194	0.22901491	0.010529185	0.000534531	0.307207915	0.91911993	0.04973365	KS
F	*Erica arborea*	230.20935	-0.070224739	0.10469113	0.008418083	-0.010291010	0.505273341	0.67108195	0.55467028	KS
F	*Calicotome villosa*	461.06852	-0.191985870	0.21974019	0.028521948	-0.008842592	0.390283053	0.64758079	0.89580530	SW
F	*Calluna vulgaris*	425.76251	-0.055270339	0.34666217	0.001493047	-0.057242656	0.875204506	0.01879426	0.60583089	SW
F	*Viburnum tinus*	-304.14307	0.199817157	0.30231260	0.014353343	-0.018501546	0.513680907	0.12682324	0.21903527	SW
F	*Quercus coccifera*	-209.86986	0.166660199	0.15193302	0.023499673	0.003969666	0.277926109	0.80783401	0.81949917	KS
F	*Cytisus malacitanus*	1,675.29822	-0.797112660	0.47235651	0.110173489	0.071485379	0.105019555	0.98843677	0.30471461	SW
F	*Pistacia lentiscus*	349.79068	-0.123701692	0.17450802	0.010150642	-0.010050366	0.481770707	0.16074688	0.64026031	KS

Phenophase	Taxon	Intercept	Slope	SE	R^2	$Adj.R^2$	p value	Normality_p value	BPTest	TestUsed
F	*Cistus ladanifer*	-449.66674	0.280866196	0.36632405	0.011178477	-0.007837321	0.446717912	0.93303295	0.07929930	KS
F	*Alyssum serpyllifolium subsp. malacitanum*	1,014.90454	-0.443552813	0.29425225	0.064419635	0.036068715	0.141225213	0.02545050	0.67395712	SW
F	*Linaria saturejoides*	-576.98397	0.346487318	0.33524287	0.023702017	0.001513426	0.306998196	0.98126987	0.07508061	SW
F	*Helianthemum syriacum*	1,382.38958	-0.616401991	0.44954022	0.078732498	0.036856703	0.184143772	0.81944088	0.78665531	SW
F	*Thymbra capitata*	1,142.27399	-0.484908983	0.30897796	0.075871159	0.045066864	0.127044238	0.72475860	0.82054082	SW
F	*Klasea baetica*	-1,212.60072	0.681768110	0.22457764	0.380573662	0.339278573	0.008341903	0.92429684	0.38917836	SW
F	*Myrtus communis*	438.58346	-0.141473186	0.40329056	0.007186721	-0.051214060	0.730049974	0.06670961	0.99209355	SW
F	*Cistus populifolius*	1,094.05276	-0.479190144	0.23972687	0.210343577	0.157699816	0.064075329	0.18201353	0.63164078	SW

Phenophase	Taxon	Intercept	Slope	SE	R^2	Adj.R^2	p value	Normality_p value	BPTest	TestUsed
F	*Quercus rotundifolia*	812.39018	-0.340984623	0.19228221	0.047544003	0.032425654	0.081003231	0.92188592	0.02863486	KS
F	*Genista hirsuta subsp. lanuginosa*	1,315.12770	-0.587021654	0.26477090	0.126312146	0.100615445	0.033406227	0.72552799	0.87247075	SW
F	*Quercus faginea*	614.85913	-0.244206033	0.20468858	0.023557025	0.007007144	0.237621079	0.97780354	0.17218421	KS
F	*Quercus suber*	-227.00814	0.179873266	0.48797414	0.005629588	-0.035802513	0.715648417	0.20258537	0.65081816	SW
F	*Crataegus monogyna*	183.60505	-0.026871445	0.13050022	0.000770304	-0.017397509	0.837620644	0.83244356	0.70927256	KS
F	*Macrochloa tenacissima*	-1,163.03681	0.640861207	0.37332782	0.092240195	0.060938132	0.096713817	0.86821265	0.90166977	SW
F	*Erica scoparia*	1,017.40241	-0.434747535	0.38907323	0.053705081	0.010691676	0.275881130	0.41936037	0.39078894	SW
F	*Adenocarpus telonensis*	-457.80682	0.291498205	0.30389765	0.026347662	-0.002289171	0.344230509	0.61066241	0.28994075	SW

Phenophase	Taxon	Intercept	Slope	SE	R^2	$Adj.R^2$	p value	Normality_p value	BPTest	TestUsed
F	*Retama sphaerocarpa*	1,588.78734	-0.729630892	0.40887667	0.175115190	0.120122869	0.094581677	0.71718301	0.07551615	SW
F	*Thymus mastichina*	797.62979	-0.321535779	0.07267987	0.220970835	0.209680557	0.000035300	0.97774206	0.26646695	KS
F	*Echium albicans*	-286.04700	0.212136694	0.18966271	0.022642654	0.004543444	0.268306937	0.87541040	0.21175411	KS
F	*Chaenorhinum glareosum*	616.25596	-0.207118780	0.10542694	0.138535727	0.102641382	0.061144059	0.27113635	0.37874776	SW
F	*Halimium atriplicifolium*	1,310.88693	-0.584972088	0.48957920	0.073485883	0.022012876	0.247653659	0.27319801	0.26909967	SW
F	*Cistus laurifolius*	35.63681	0.065156595	0.20693067	0.007568749	-0.068772116	0.757855996	0.51250904	0.10988853	SW
F	*Staehelina baetica*	-72.57057	0.117987310	0.21740229	0.014513200	-0.034761140	0.593323639	0.51070649	0.16007347	SW
F	*Centaurea prolongoi*	-176.72474	0.167285876	0.33888967	0.006209152	-0.019272665	0.624336571	0.24857875	0.87741927	SW

Phenophase	Taxon	Intercept	Slope	SE	R^2	Adj.R^2	p value	Normality_p value	BPTest	TestUsed
F	*Crataegus granatensis*	468.67245	-0.153541774	0.07090388	0.169355862	0.133240900	0.040965954	0.08087648	0.07051831	SW
F	*Sideritis incana*	-655.83004	0.401495246	0.24234018	0.074698887	0.047484149	0.106772811	0.17673193	0.21991119	SW
F	*Prunus prostrata*	580.53474	-0.215284071	0.19083821	0.038247791	0.008193034	0.267666068	0.19094520	0.19774348	SW
F	*Centaurea carratracensis*	-505.62860	0.325919612	0.42724271	0.028273888	-0.020312417	0.454460554	0.21015580	0.20467208	SW
F	*Salvia candelabrum*	-78.01067	0.117795187	0.36203349	0.008077777	-0.068223933	0.750077672	0.94406351	0.87549613	SW
F	*Lonicera etrusca*	763.83427	-0.296304758	0.10343889	0.166761243	0.146438347	0.006556677	0.47801816	0.10541175	SW
F	*Saxifraga reuteriana*	294.24802	-0.072040300	0.26795989	0.003430046	-0.044025666	0.790670766	0.06431568	0.40252363	SW
F	*Thymus longiflorus*	352.89994	-0.094093127	0.07234252	0.017496018	0.007153870	0.196520639	0.79476319	0.01670949	KS

Phenophase	Taxon	Intercept	Slope	SE	R^2	Adj.R^2	p value	Normality_p value	BPTest	TestUsed
F	*Hormathophylla spinosa*	1,212.06295	-0.523744917	0.12976018	0.425457532	0.399341966	0.000552120	0.10785369	0.16142346	SW
F	*Elaeoselinum asclepium subsp. millefolium*	984.02561	-0.407674110	0.29789677	0.094240006	0.043920006	0.187992654	0.85706945	0.50459494	SW
F	*Phlomis crinita subsp. malacitana*	652.73869	-0.239516508	0.11321495	0.096302408	0.074785798	0.040351520	0.54098552	0.29313202	SW
F	*Crepis oporinoides*	1,199.85231	-0.507592050	0.15071303	0.465965635	0.424886068	0.005043734	0.26682543	0.12562244	SW
F	*Leontodon boryi*	56.13809	0.072362561	0.11085002	0.025943030	-0.034935530	0.523156909	0.39963778	0.55131286	SW
F	*Sempervivum minutum*	955.35925	-0.383001349	0.18951074	0.184946651	0.139665910	0.058409625	0.44554947	0.04084672	SW
F	*Nevadensia purpurea*	287.74546	-0.045539943	0.04926025	0.033056207	-0.005621545	0.364076222	0.11392684	0.43696662	SW

**Phenophase**	**Taxon**	**Intercept**	**Slope**	**SE**	**R^2**	**Adj.R^2**	**p value**	**Normality_p value**	**BPTest**	**TestUsed**
FBF	*Phlomis purpurea*	-77.51611	0.092908347	0.18008331	0.003400857	-0.009376055	0.607370611	0.99873827	0.18631084	KS
FBF	*Phillyrea angustifolia*	-804.78867	0.442108191	0.58849325	0.027444699	-0.021183066	0.461248631	0.48790942	0.59373540	SW
FBF	*Salvia rosmarinus*	-1,251.61207	0.666040143	0.85934024	0.038505781	-0.025593834	0.450350627	0.01044487	0.71919517	SW
FBF	*Cistus albidus*	-1,577.41976	0.835248684	0.37599116	0.114938655	0.091647567	0.032352603	0.81557483	0.30790961	SW
FBF	*Ulex parviflorus*	-1,381.37238	0.718047316	0.65796844	0.078399064	0.012570425	0.293562558	0.48406171	0.64830505	SW
FBF	*Juniperus oxycedrus*	2,450.72975	-1.112562020	0.90270468	0.097879876	0.033442724	0.238068618	0.15909080	0.60966622	SW
FBF	*Helichrysum stoechas*	-1,794.66772	0.948436735	0.59703908	0.136234496	0.082249152	0.131720793	0.75263369	0.80786892	SW
FBF	*Lavandula stoechas*	-685.55390	0.393912806	0.36984422	0.021349456	0.002529253	0.291761462	0.79410993	0.79811418	KS

**Phenophase**	**Taxon**	**Intercept**	**Slope**	**SE**	**R^2**	**Adj.R^2**	**p value**	**Normality_p value**	**BPTest**	**TestUsed**
FBF	*Erica arborea*	75.35134	-0.004628650	0.64102170	0.000002480	-0.047616447	0.994306891	0.60426789	0.21805979	SW
FBF	*Fumana thymifolia*	-1,324.57612	0.713106915	0.22682074	0.183434874	0.164876575	0.002983541	0.26415500	0.90175063	SW
FBF	*Cistus ladanifer*	-1,125.93783	0.607903388	0.29365056	0.085224446	0.065338021	0.044078357	0.28638547	0.68796386	SW
FBF	*Alyssum serpyllifolium subsp. malacitanum*	-2,122.20816	1.115932887	0.51499973	0.226877117	0.178556937	0.045683199	0.84018280	0.65689555	SW
FBF	*Helianthemum syriacum*	-773.84840	0.455929918	0.39266575	0.038140203	0.009850209	0.253682645	0.71946644	0.42792702	SW
FBF	*Rubia peregrina*	409.31312	-0.144237513	0.46912866	0.004093204	-0.039207092	0.761261317	0.01653613	0.22029787	SW
FBF	*Olea europaea var. sylvestris*	-548.34063	0.334680536	0.29243698	0.085551487	0.020233736	0.271625743	0.13686411	0.85557400	SW
FBF	*Thymbra capitata*	136.61909	0.004877394	0.72935306	0.000002350	-0.052629101	0.994734072	0.45253793	0.14511174	SW

**Phenophase**	**Taxon**	**Intercept**	**Slope**	**SE**	**R^2**	**Adj.R^2**	**p value**	**Normality _p value**	**BPTest**	**TestUsed**
FBF	*Cistus populifolius*	1,257.81217	-0.574091991	0.42167520	0.062088437	0.028591595	0.184225669	0.09355085	0.16757607	SW
FBF	*Genista hirsuta subsp. lanuginosa*	392.30229	-0.147225745	0.33259578	0.009244454	-0.037934381	0.662539975	0.06631355	0.74725484	SW
FBF	*Crataegus monogyna*	-28.93134	0.064682421	0.25079386	0.003897569	-0.054696692	0.799572072	0.57160376	0.58373362	SW
FBF	*Macrochloa tenacissima*	583.94254	-0.251857385	0.41676791	0.027324086	-0.047497138	0.556037235	0.07002519	0.50352576	SW
FBF	*Erica scoparia*	372.36555	-0.134979043	0.31220277	0.009742129	-0.042376706	0.670358496	0.88085484	0.19986931	SW
FBF	*Thymus mastichina*	-115.51080	0.127865478	0.13655099	0.019983963	-0.002807108	0.354300131	0.61892502	0.46580822	SW
FBF	*Halimium atriplicifolium*	536.48190	-0.200407210	0.30539072	0.007631326	-0.010089543	0.514362263	0.79139946	0.58403473	KS
FBF	*Cistus laurifolius*	633.27642	-0.237222292	0.22709398	0.060316015	0.005040487	0.310835240	0.81963996	0.18265703	SW

**Phenophase**	**Taxon**	**Intercept**	**Slope**	**SE**	**R^2**	**Adj.R^2**	**p value**	**Normality_p value**	**BPTest**	**TestUsed**
FBF	*Salvia lavandulifolia*	-128.85505	0.148455387	0.48194912	0.007245826	-0.069119880	0.762938481	0.99732554	0.13541549	SW
FBF	*Centaurea prolongoi*	-527.64349	0.330087632	0.33161958	0.061959581	-0.000576447	0.335338255	0.12384510	0.92217494	SW
FBF	*Salvia candelabrum*	5.42517	0.069942575	0.19597661	0.008419984	-0.057685350	0.726143285	0.99999421	0.05075117	SW
FBF	*Lonicera etrusca*	1,198.41908	-0.523839579	0.23845837	0.270720941	0.214622552	0.046773627	0.56986387	0.63500367	SW
FBF	*Hormathophylla spinosa*	61.68286	0.049605485	0.29665248	0.001642104	-0.057084831	0.869171897	0.75506087	0.54676915	SW
FBF	*Lavandula lanata*	1,022.42359	-0.421429949	0.15247113	0.234061285	0.203423737	0.010563451	0.38458119	0.06644941	SW
FBF	*Phlomis crinita subsp. malacitana*	620.87236	-0.228277620	0.12568862	0.135754061	0.094599493	0.083641592	0.02924504	0.21807002	SW
FBF	*Sideritis glacialis*	620.10520	-0.216267398	0.07081529	0.341303565	0.304709318	0.006833092	0.49931617	0.80006998	SW

**Phenophase**	**Taxon**	**Intercept**	**Slope**	**SE**	**R^2**	**Adj.R^2**	**p value**	**Normality_p value**	**BPTest**	**TestUsed**
FS	*Cytisus malacitanus*	2,707.19767	-1.308796205	0.77914907	0.167739129	0.108291924	0.115168852	0.08185170	0.70973275	SW
FS	*Cistus ladanifer*	1,998.62330	-0.918246029	0.71045302	0.073686211	0.029576031	0.210232320	0.31685298	0.10908102	SW
FS	*Cistus populifolius*	-543.41731	0.352652742	0.33779644	0.067737818	0.005587006	0.313028382	0.13665504	0.48275336	SW
FS	*Crataegus monogyna*	-257.94595	0.211772141	0.29980513	0.019567583	-0.019649714	0.486492146	0.09008637	0.85447112	SW
FS	*Thymus mastichina*	417.94206	-0.123574049	0.15476663	0.028162481	-0.016011952	0.433142358	0.07500482	0.14769133	SW
FS	*Acer granatense*	179.65150	-0.000353684	0.15725180	0.000000266	-0.052631299	0.998228881	0.05412343	0.33280003	SW
FS	*Helictotrichon filifolium subsp. arundanum*	1,022.50655	-0.432509027	0.25949370	0.156261907	0.100012701	0.116303966	0.99369172	0.94779335	SW
FS	*Hormathophylla spinosa*	319.75540	-0.065685311	0.11652129	0.012074710	-0.025922417	0.577775450	0.08561824	0.89115338	SW

Phenophase	Taxon	Intercept	Slope	SE	R^2	Adj.R^2	p value	Normality_p value	BPTest	TestUsed
FS	*Crepis oporinoides*	288.05976	-0.041134164	0.14981817	0.005000437	-0.061332867	0.787399213	0.21962887	0.21244793	SW
FS	*Nevadensia purpurea*	295.44075	-0.046276821	0.06590348	0.024060421	-0.024736558	0.490652383	0.06378253	0.17058618	SW
DVG	*Phlomis purpurea*	-490.80586	0.302104229	0.13700241	0.032446235	0.025773450	0.029022085	0.53715434	0.52936941	KS
DVG	*Phillyrea angustifolia*	-1,716.54083	0.919396681	0.77597088	0.091135222	0.026216309	0.255807155	0.23060310	0.66877527	SW
DVG	*Cistus albidus*	-2,132.72044	1.118637648	0.55726179	0.108820692	0.081815258	0.052955953	0.04416054	0.64150993	SW
DVG	*Rhamnus alaternus*	-1,447.64599	0.778357399	0.32382878	0.071521685	0.059141974	0.018705195	0.86188929	0.63451123	KS
DVG	*Ulex parviflorus*	1,147.12329	-0.510366926	0.46856852	0.065233833	0.010247588	0.291263767	0.58199073	0.19839083	SW
DVG	*Juniperus oxycedrus*	224.57739	-0.046784009	0.21685785	0.000581436	-0.011911296	0.829742765	0.95535211	0.07740021	KS

**Phenophase**	**Taxon**	**Intercept**	**Slope**	**SE**	**R^2**	**Adj.R^2**	**p value**	**Normality_p value**	**BPTest**	**TestUsed**
DVG	*Helichrysum stoechas*	-578.02471	0.345903267	0.55736394	0.018010230	-0.028751188	0.541536542	0.39162624	0.31163854	SW
DVG	*Calicotome villosa*	331.04807	-0.126497531	0.36320607	0.004313428	-0.031246807	0.730234706	0.49818427	0.86648052	SW
DVG	*Viburnum tinus*	125.69137	-0.012487666	0.40895813	0.000071700	-0.076845842	0.976103947	0.47349661	0.62124148	SW
DVG	*Quercus coccifera*	178.76463	-0.027991812	0.15999496	0.000679738	-0.021527379	0.861900343	0.81296847	0.35770382	SW
DVG	*Juniperus turbinata*	1,816.50938	-0.848289290	0.70092509	0.037113858	0.011774749	0.233660410	0.01323536	0.28808130	SW
DVG	*Fumana thymifolia*	-2,889.22434	1.512300710	0.92804628	0.159433965	0.099393533	0.125481475	0.28370546	0.90460334	SW
DVG	*Cistus ladanifer*	-1,319.13028	0.712958020	0.56936227	0.056878094	0.020604175	0.221642852	0.97637636	0.86127403	SW
DVG	*Rubia peregrina*	662.69020	-0.282368760	0.53892934	0.009708998	-0.025658537	0.604440541	0.19538339	0.32448384	SW

Phenophase	Taxon	Intercept	Slope	SE	R^2	Adj.R^2	p value	Normality_p value	BPTest	TestUsed
DVG	*Olea europaea var. sylvestris*	-101.92028	0.113635520	0.35680001	0.002108728	-0.018680674	0.751498607	0.92448712	0.24675695	SW
DVG	*Thymbra capitata*	3,254.64026	-1.556223046	1.21799354	0.083153028	0.032217085	0.217590790	0.22310276	0.45454654	SW
DVG	*Myrtus communis*	298.23898	-0.080844912	0.27841488	0.002899089	-0.031483701	0.773595239	0.61763943	0.93447318	SW
DVG	*Cistus populifolius*	-638.70722	0.368774847	0.50838231	0.030023010	-0.027034460	0.478081062	0.30732216	0.52837829	SW
DVG	*Quercus rotundifolia*	129.72562	-0.003856890	0.18620331	0.000007150	-0.016659397	0.983543067	0.68513942	0.79334299	KS
DVG	*Quercus faginea*	1,056.43798	-0.470535981	0.16402869	0.170623147	0.149888726	0.006552428	0.91279413	0.73299513	SW
DVG	*Quercus suber*	-29.31277	0.080029800	0.29514443	0.003854813	-0.048573881	0.789196093	0.20760932	0.76851781	SW
DVG	*Crataegus monogyna*	-214.33238	0.176961354	0.24055244	0.021188317	-0.017964150	0.468792587	0.72422618	0.76486523	SW

Phenophase	Taxon	Intercept	Slope	SE	R^2	Adj.R^2	p value	Normality_p value	BPTest	TestUsed
DVG	*Adenocarpus telonensis*	84.38504	0.023102707	0.31697586	0.000354020	-0.066289045	0.942860834	0.46673104	0.41249420	SW
DVG	*Thymus mastichina*	662.56002	-0.259916241	0.17021276	0.055082842	0.031459913	0.134628515	0.80975940	0.01331906	SW
DVG	*Erinacea anthyllis*	-76.15934	0.108707038	0.57302914	0.002760692	-0.073950024	0.852469565	0.61273454	0.83304295	SW
DVG	*Sideritis incana*	-12.85300	0.080891379	0.35446342	0.002597181	-0.047272960	0.821801586	0.44156777	0.11367243	SW
DVG	*Prunus prostrata*	1,374.09471	-0.604645548	0.18465929	0.234497388	0.212625885	0.002389779	0.11166793	0.30103422	SW
DVG	*Acer granatense*	970.41331	-0.409353695	0.22898711	0.143980990	0.098927358	0.089791154	0.70321796	0.28467815	SW
DVG	*Rhamnus infectoria*	450.66373	-0.146161846	0.20971789	0.027778784	-0.029410699	0.495258167	0.70619577	0.82379715	SW
DVG	*Lavandula lanata*	467.24318	-0.148401657	0.28691366	0.014645196	-0.040096738	0.611290207	0.42181629	0.27665990	SW

Phenophase	Taxon	Intercept	Slope	SE	R^2	Adj.R^2	p value	Normality_p value	BPTest	TestUsed
DVG	*Sideritis glacialis*	1,245.00195	-0.527823491	0.11826610	0.605085404	0.574707358	0.000639169	0.78595180	0.77756928	SW

***In the following section, each taxon with >15 records in a single phenophase, has a figure which represents the linear model trends with time (years), and its corresponding model diagnostic analysis (Diagnostics). FBF: preflowering, F: flowering, FS: fruiting, DVG: growth.**

1.1. LM - FS - Acer granatense

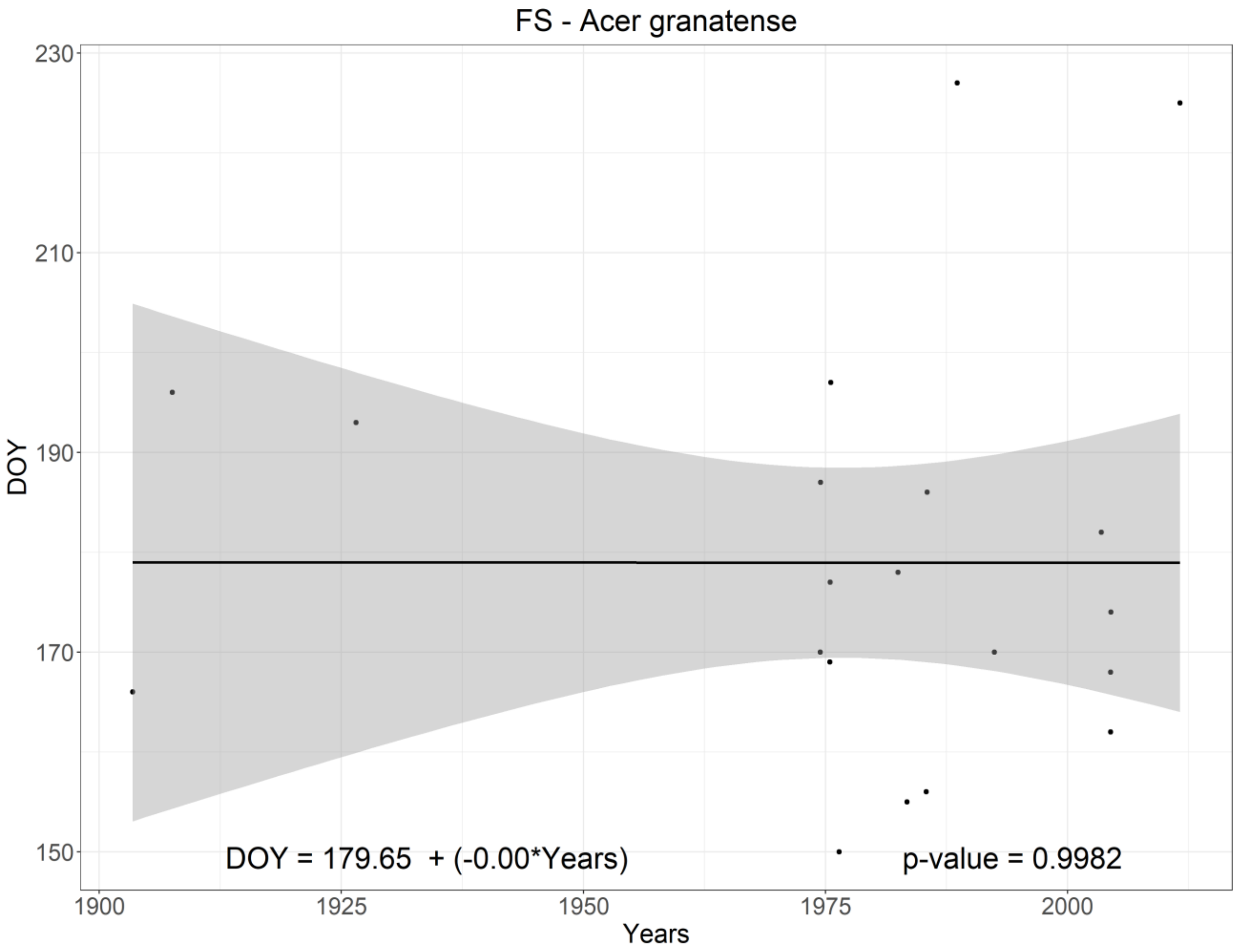

1.1.1. Diagnostics - LM - FS - Acer granatense

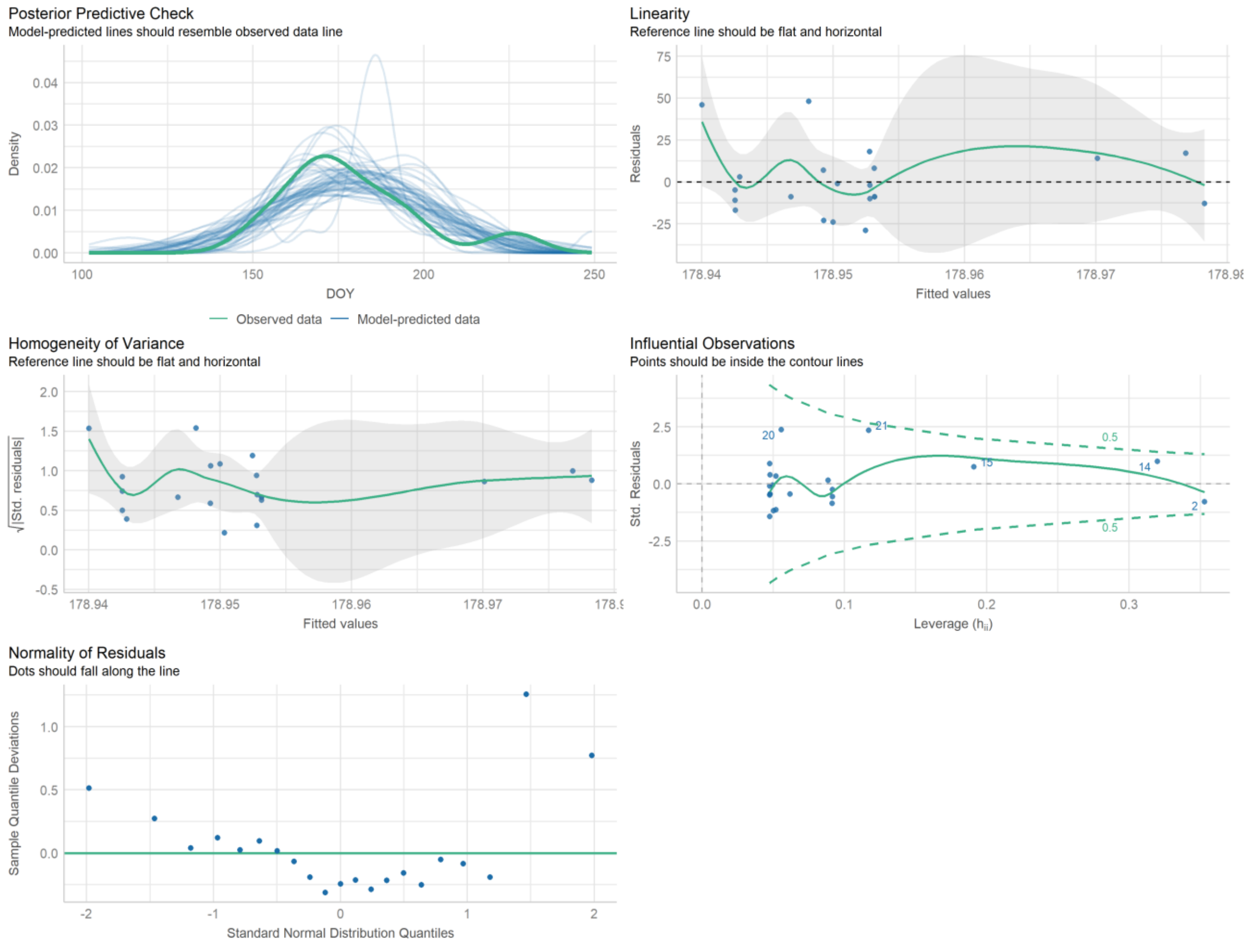

1.2. LM - DVG - Acer granatense

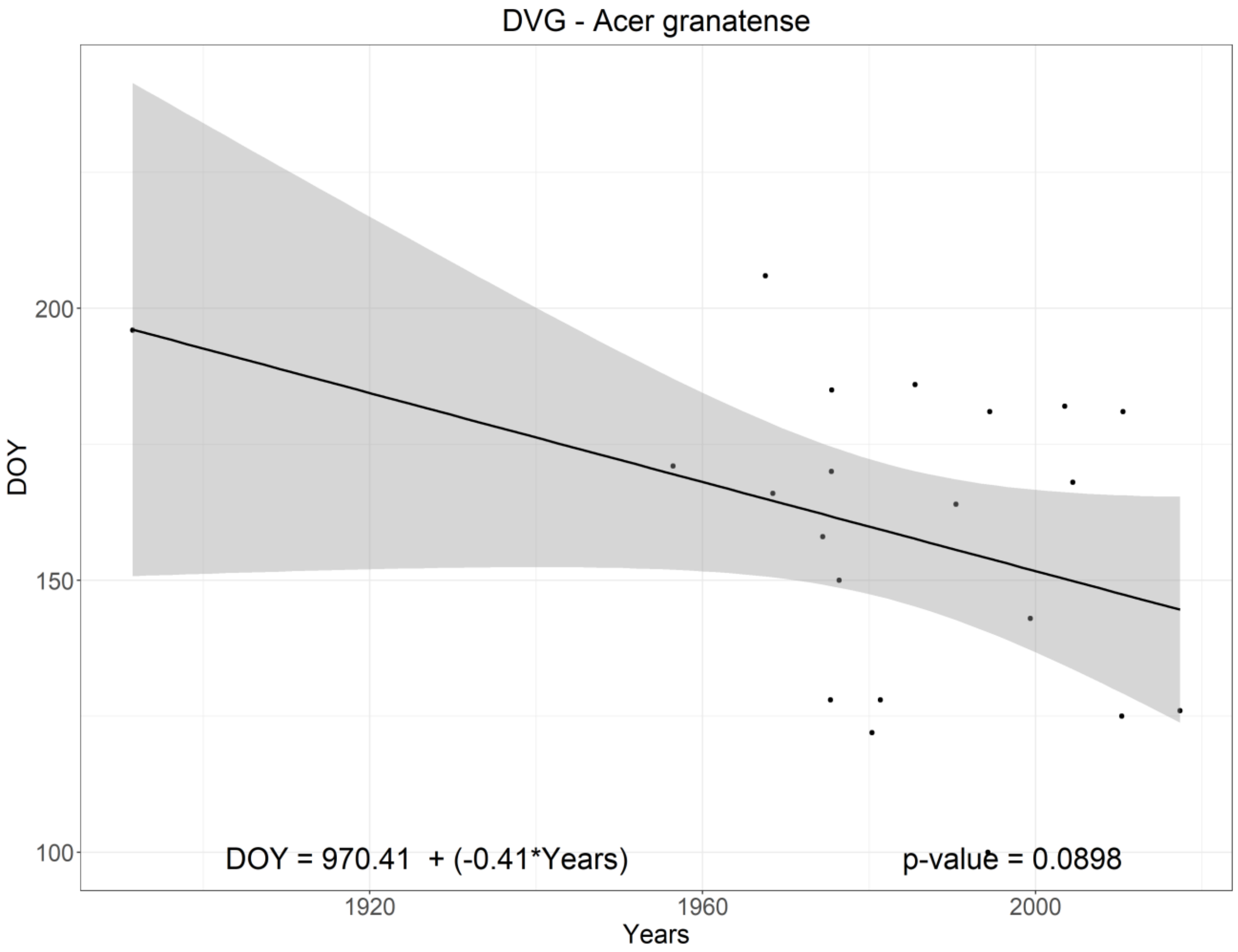

1.2.1. Diagnostics - LM - DVG - Acer granatense

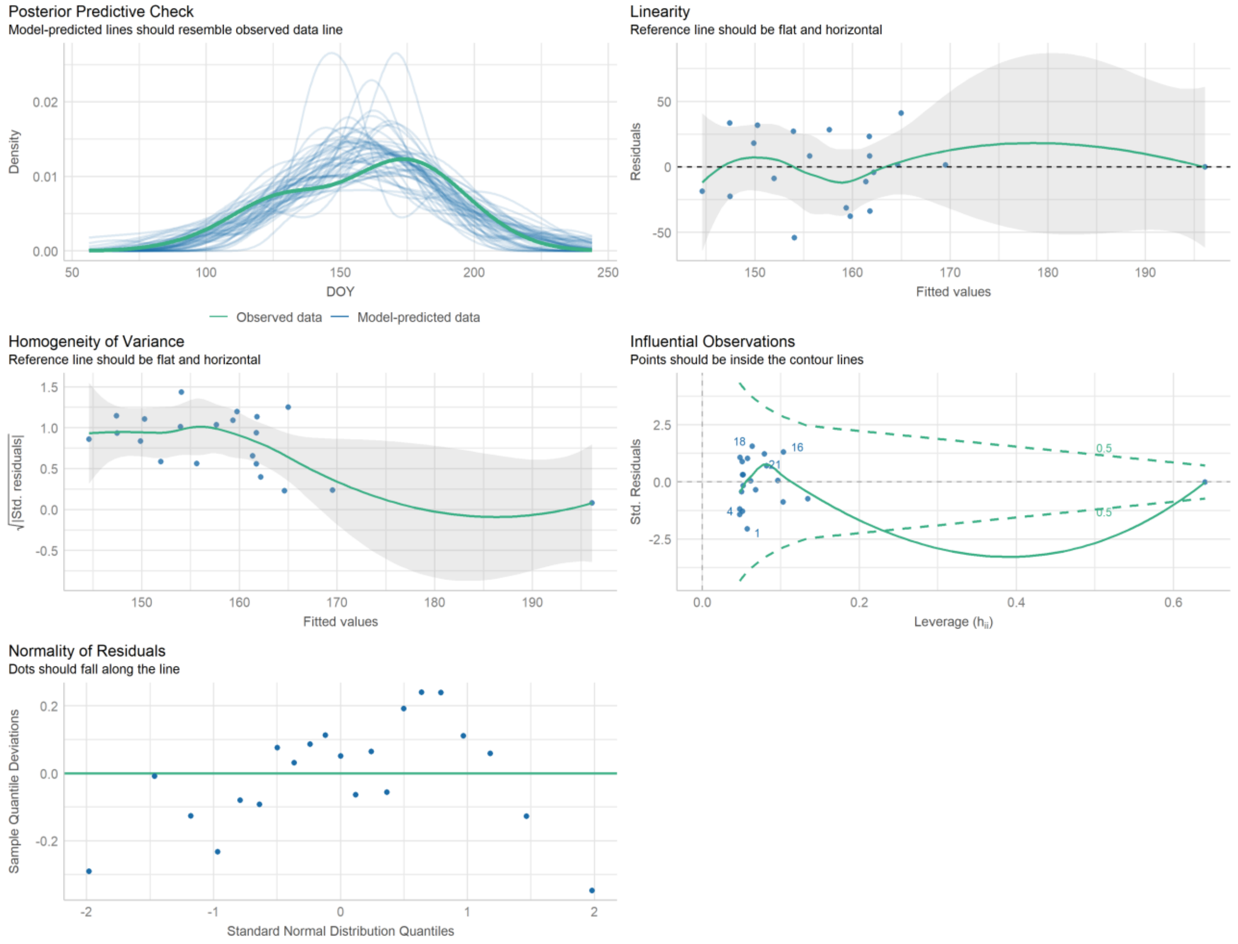

1.3. LM - F - Adenocarpus telonensis

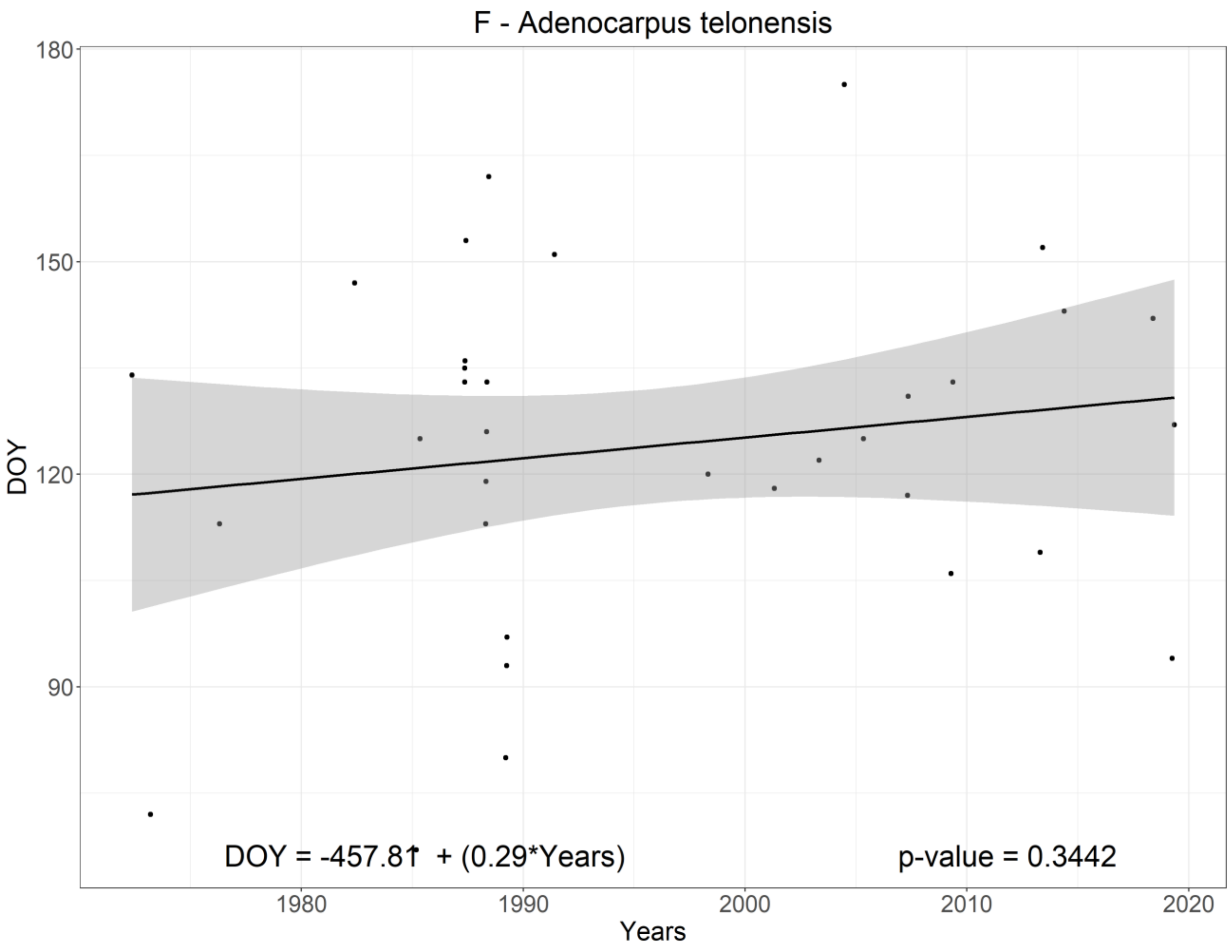

1.3.1. Diagnostics - LM - F - Adenocarpus telonensis

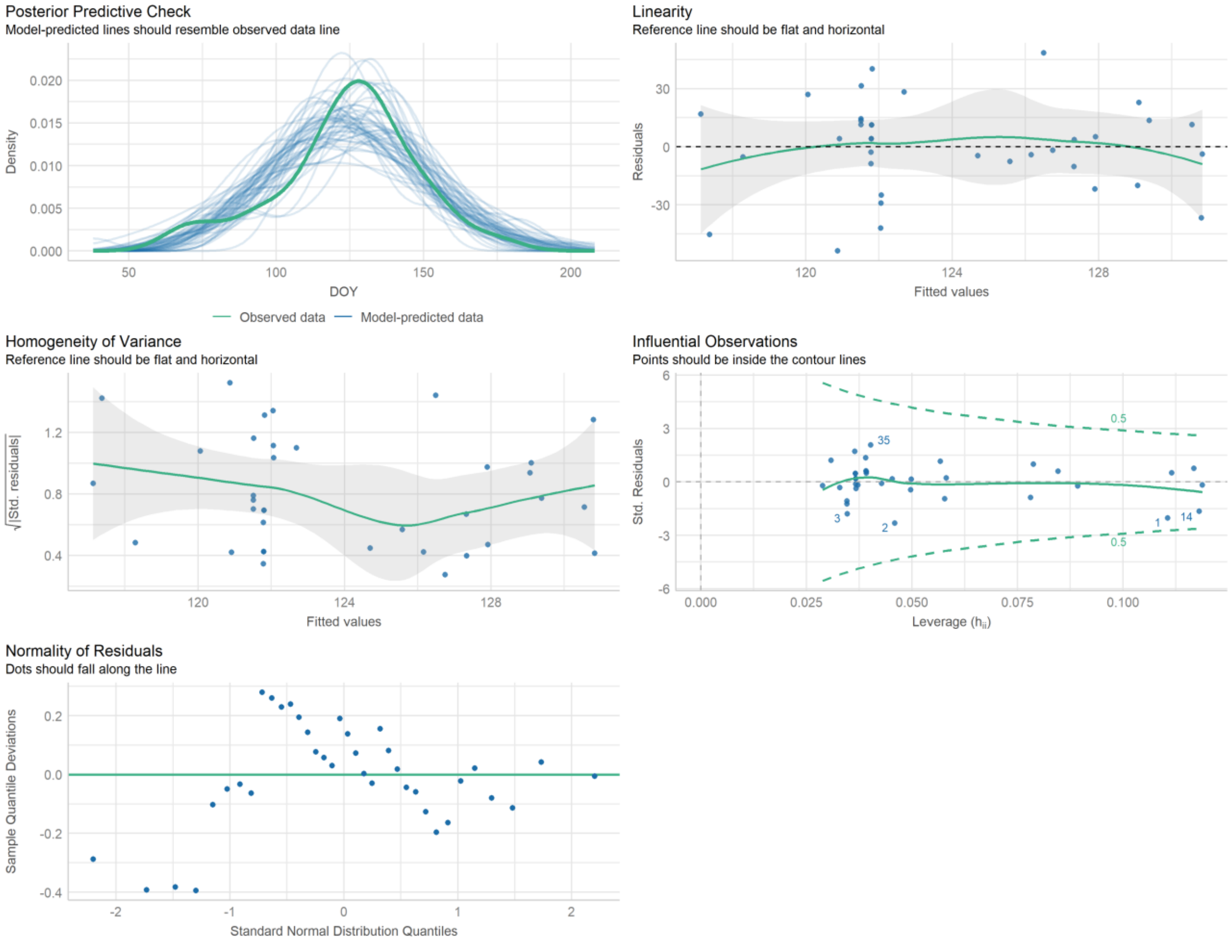

1.4. LM - DVG - Adenocarpus telonensis

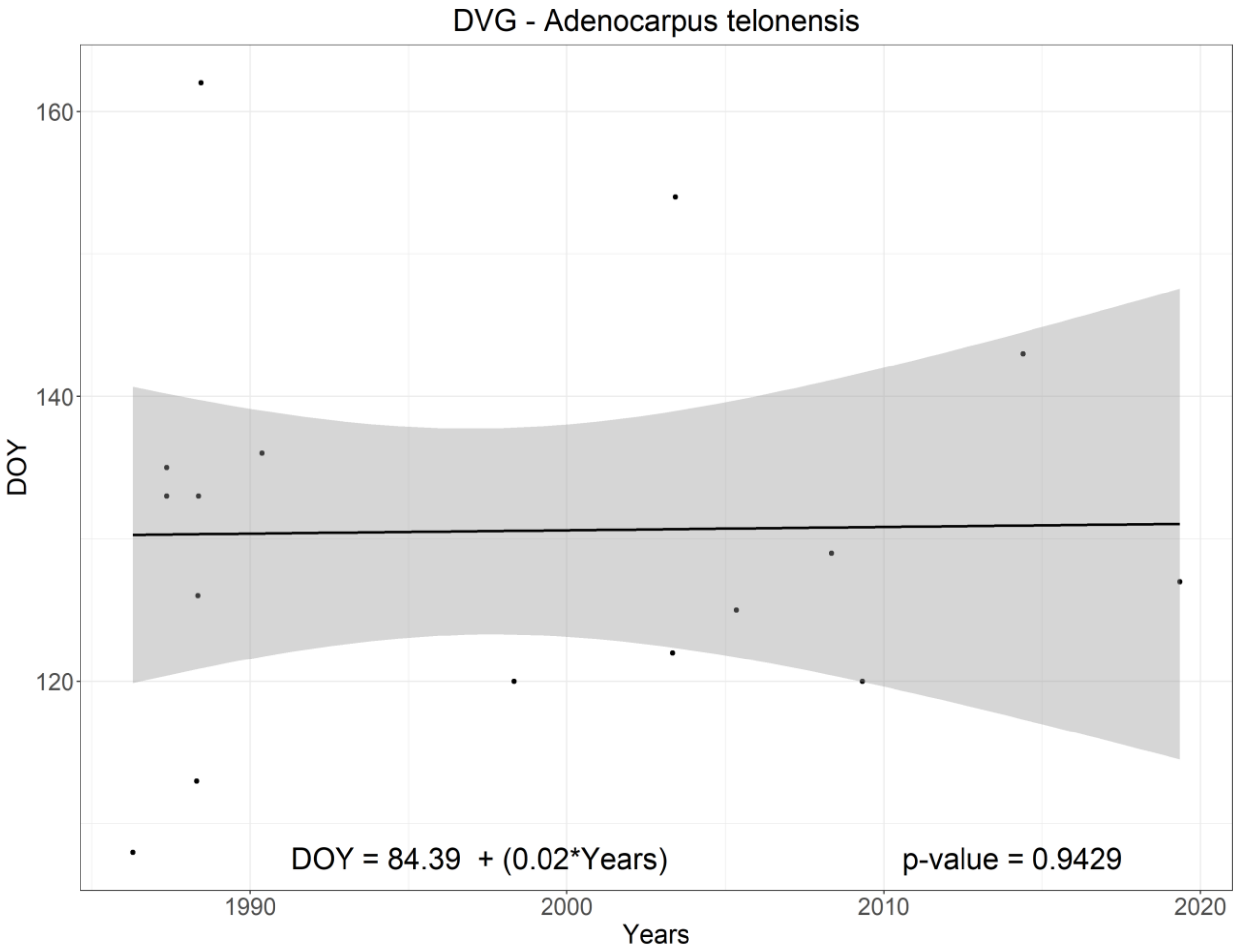

1.4.1. Diagnostics - LM - DVG - Adenocarpus telonensis

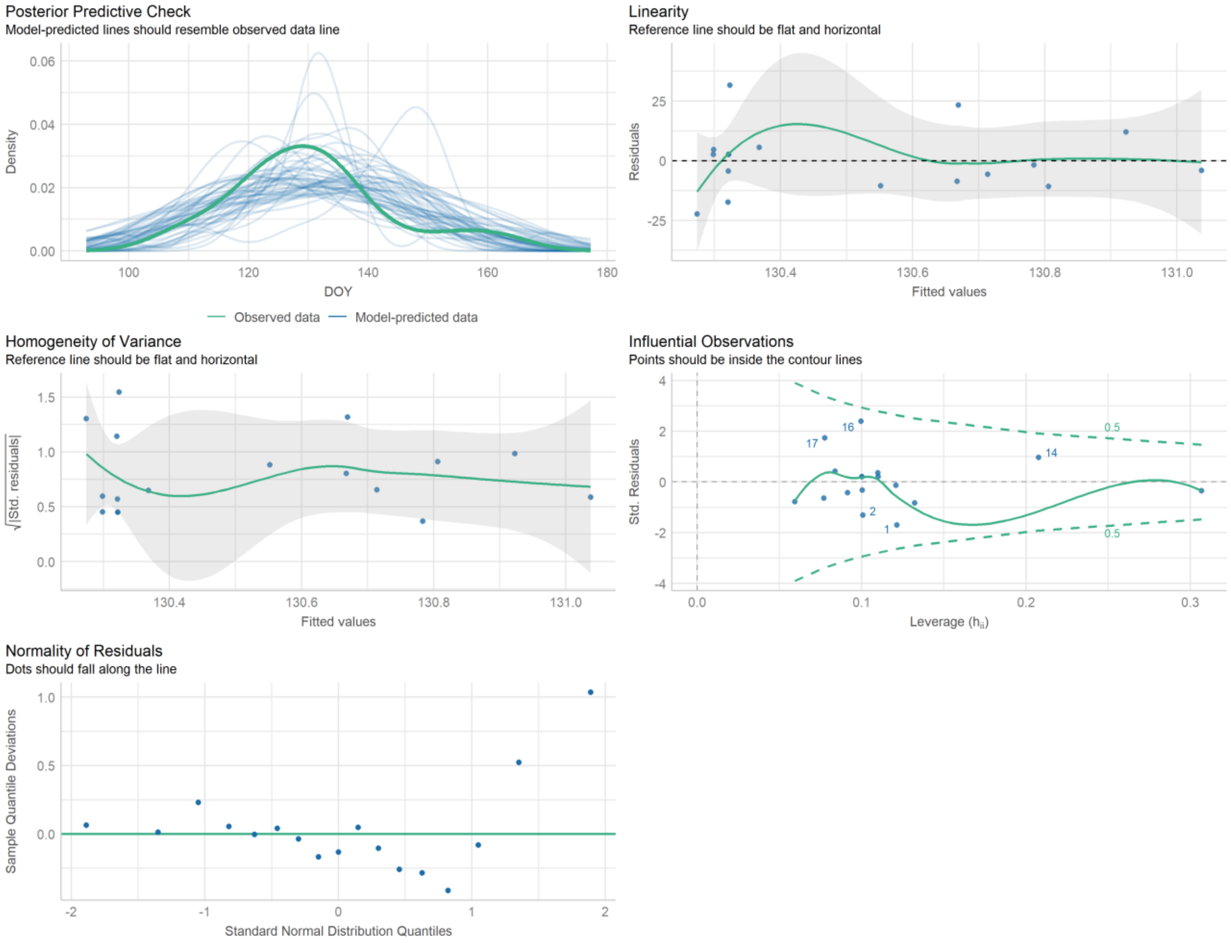

1.5. LM - FBF - Alyssum serpyllifolium subsp. malacitanum

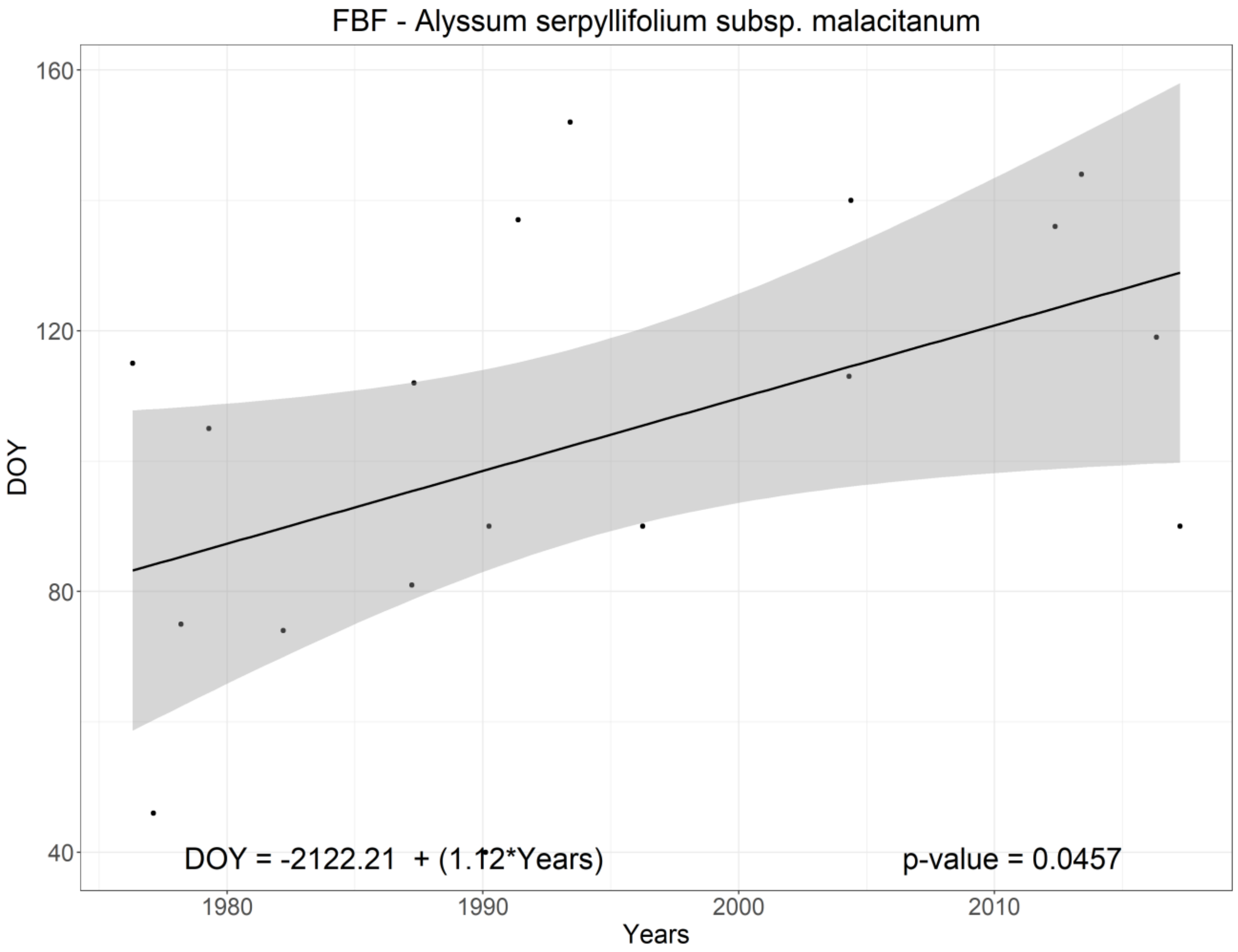

1.5.1. Diagnostics - LM - FBF - Alyssum serpyllifolium subsp. malacitanum

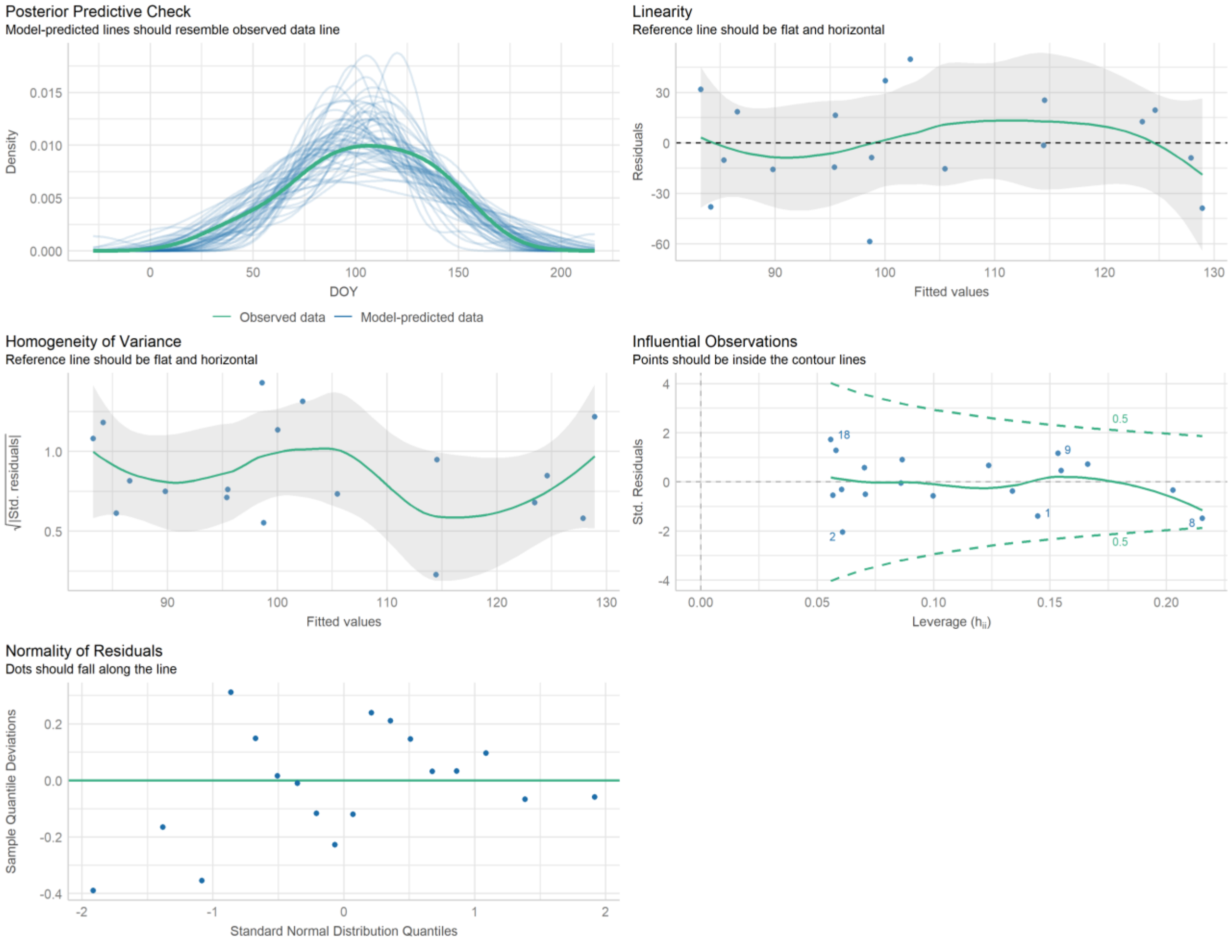

1.6. LM - F - Alyssum serpyllifolium subsp. malacitanum

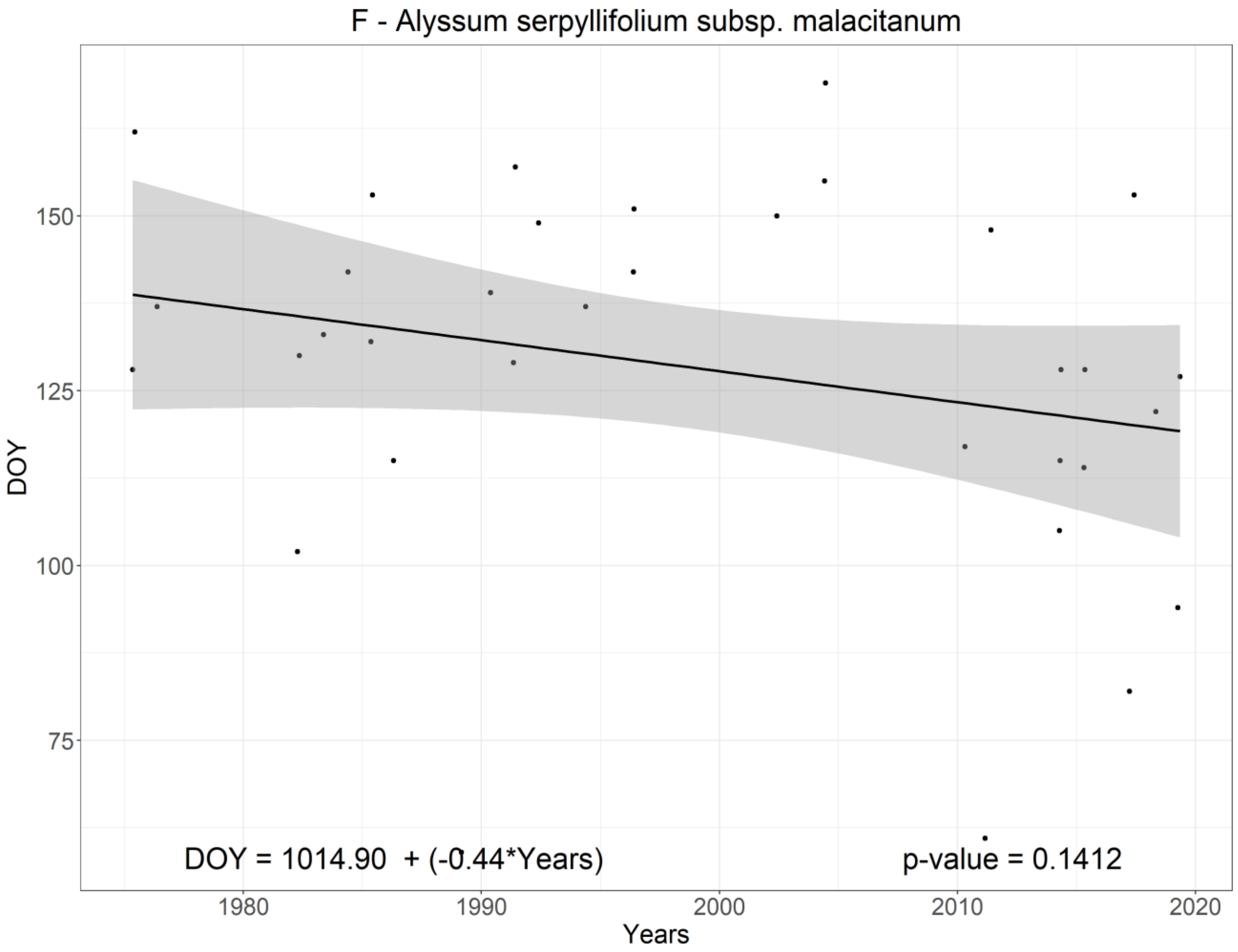

1.6.1. Diagnostics - LM - F - Alyssum serpyllifolium subsp. malacitanum

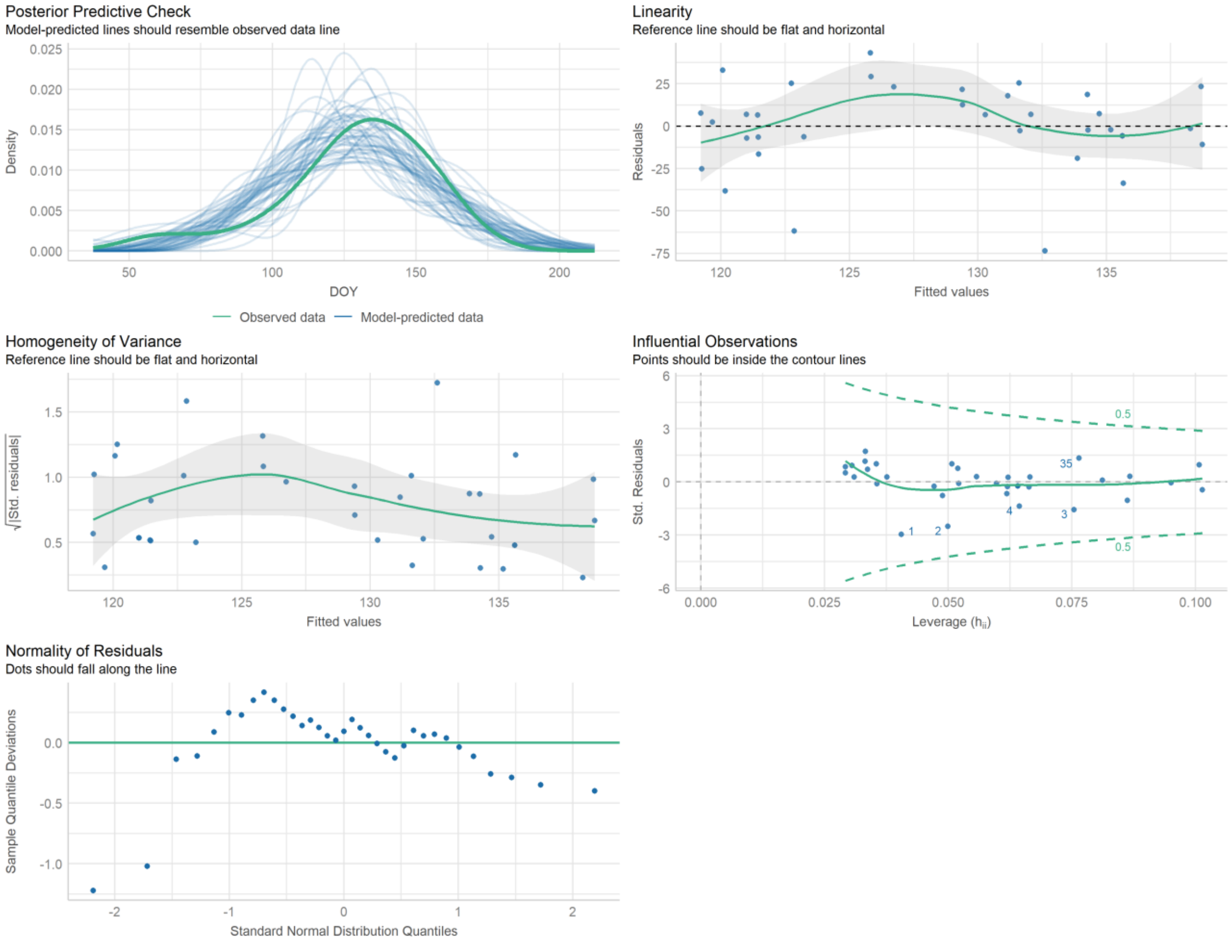

1.7. LM - F - Calicotome villosa

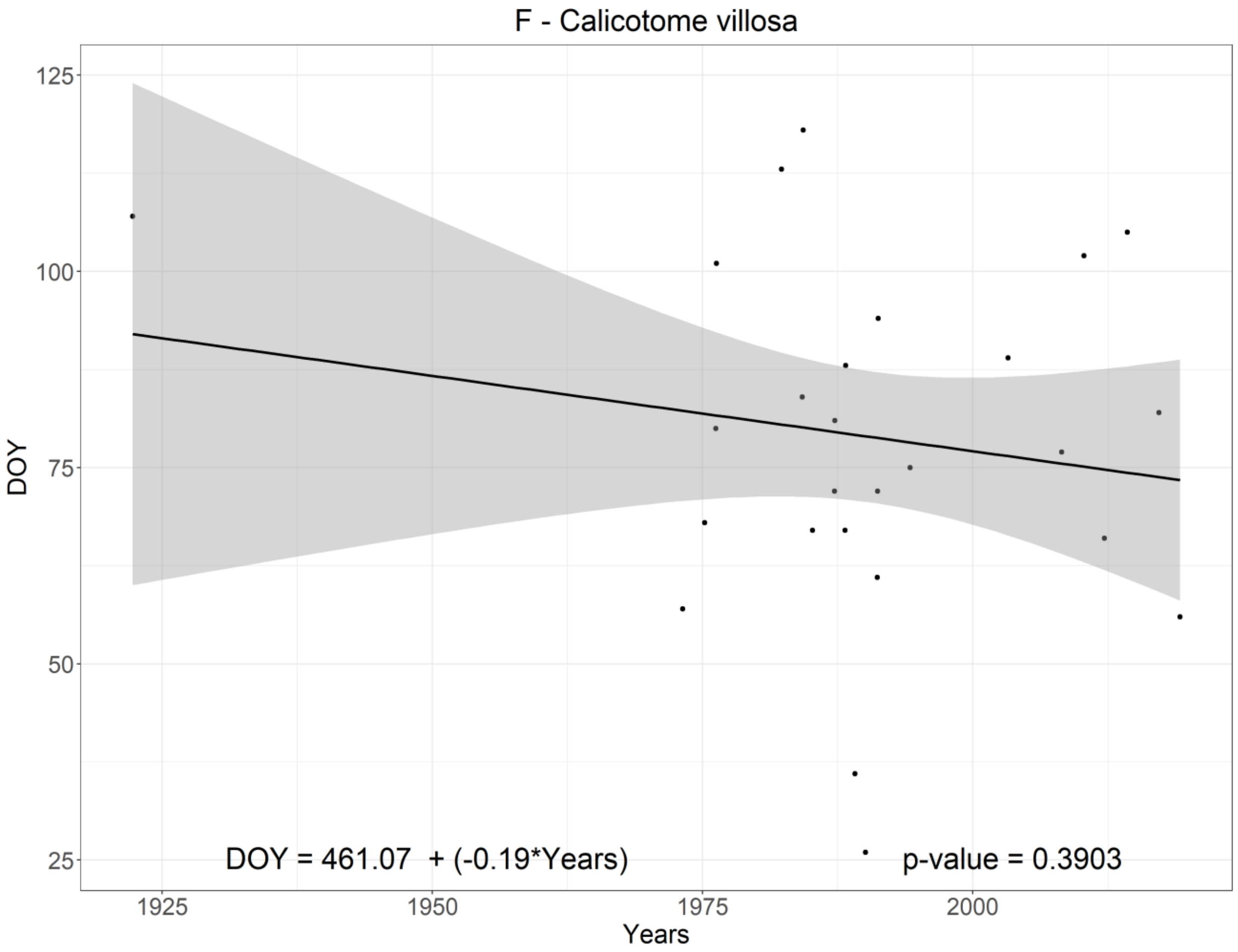

1.7.1. Diagnostics - LM - F - Calicotome villosa

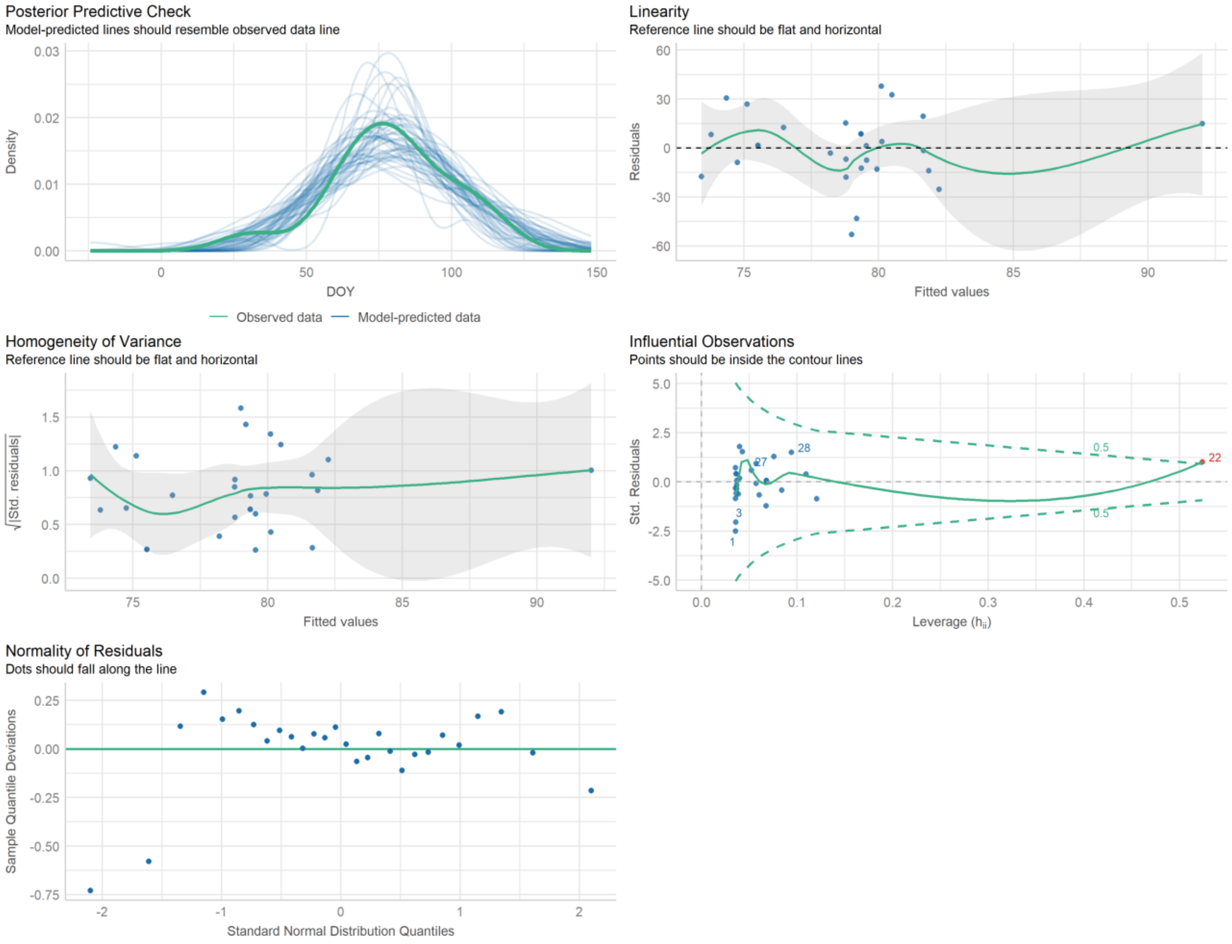

1.8. LM - DVG - Calicotome villosa

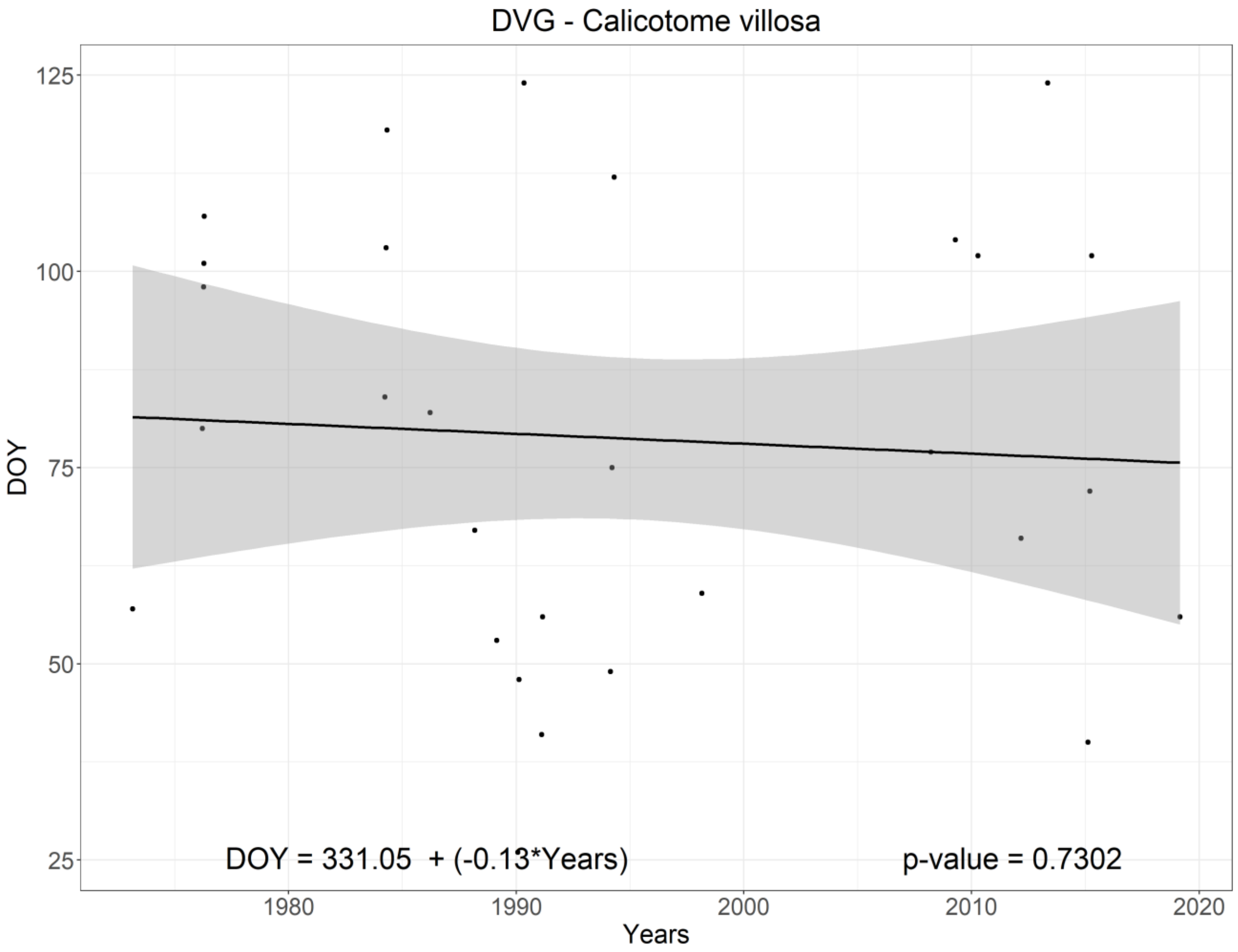

1.8.1. Diagnostics - LM - DVG - Calicotome villosa

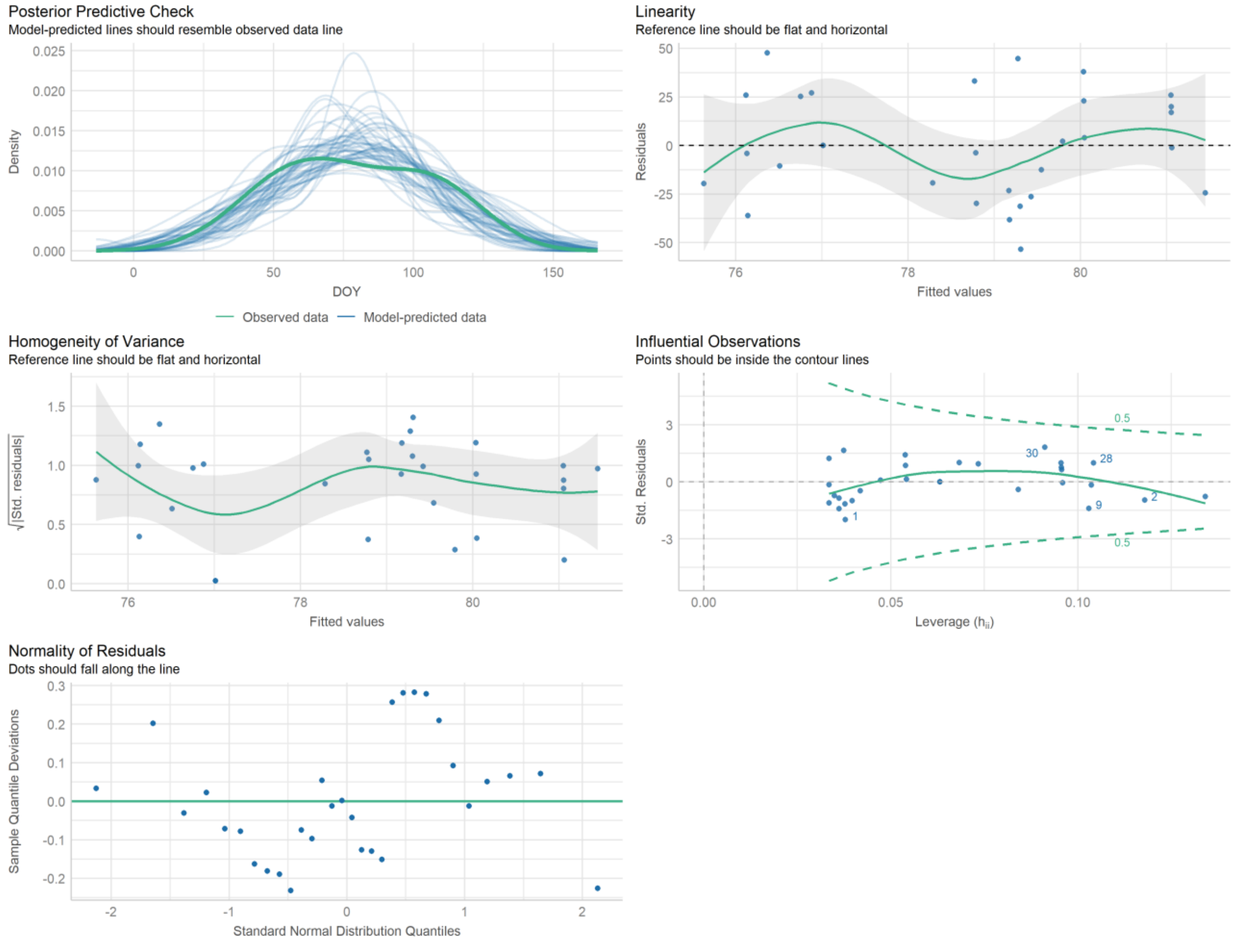

1.9. LM - F - Calluna vulgaris

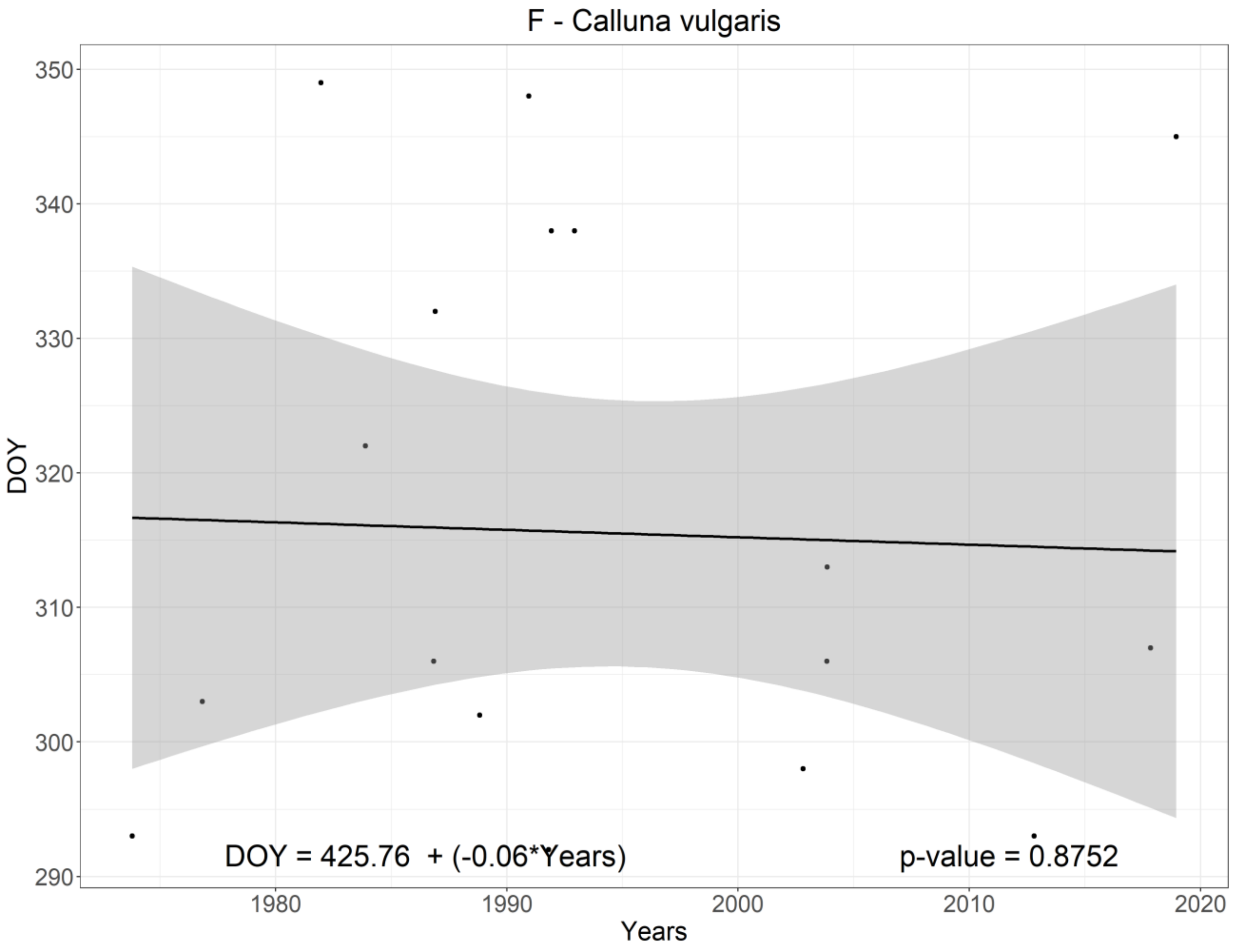

1.9.1. Diagnostics - LM - F - Calluna vulgaris

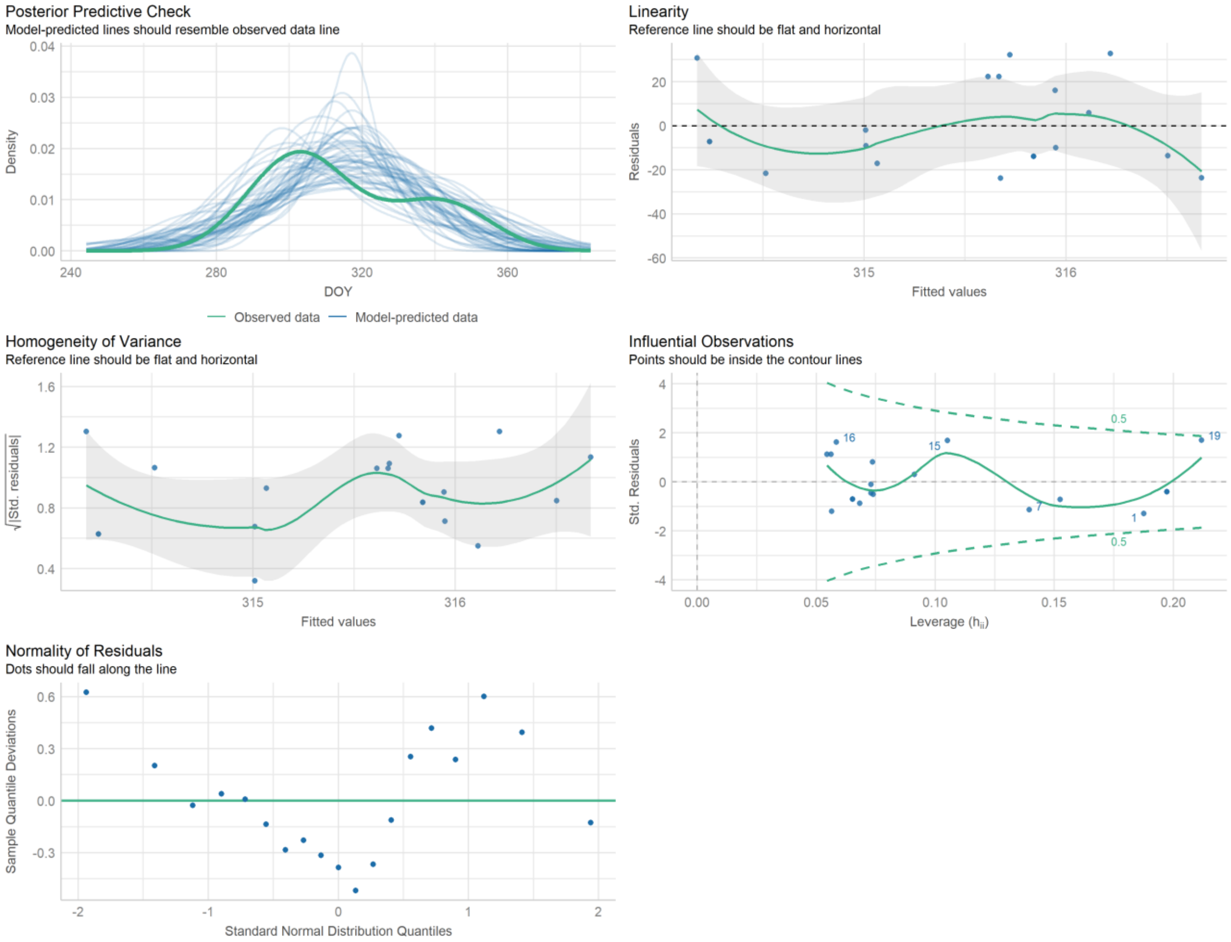

1.10. LM - F - Centaurea carratracensis

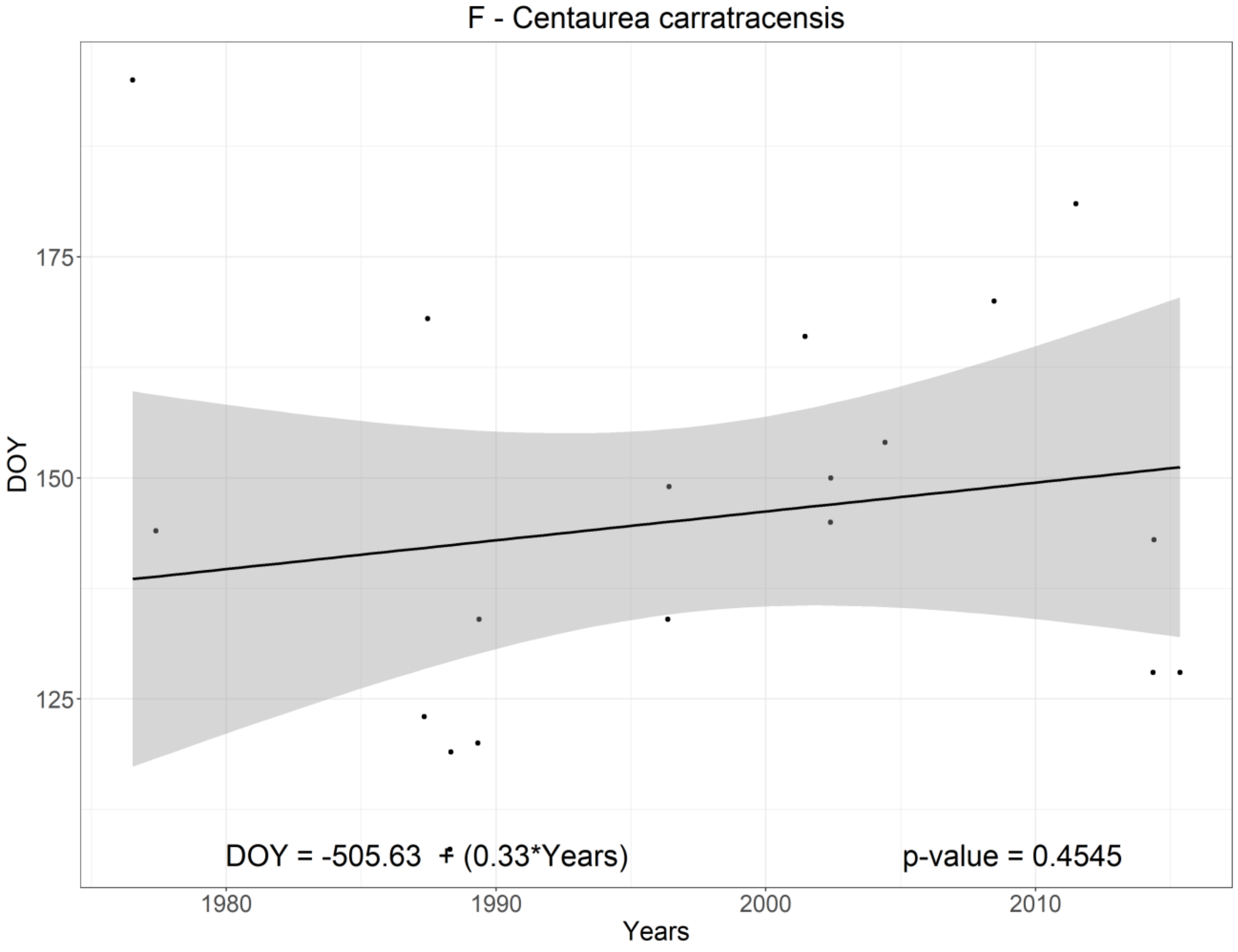

1.10.1. Diagnostics - LM - F - Centaurea carratracensis

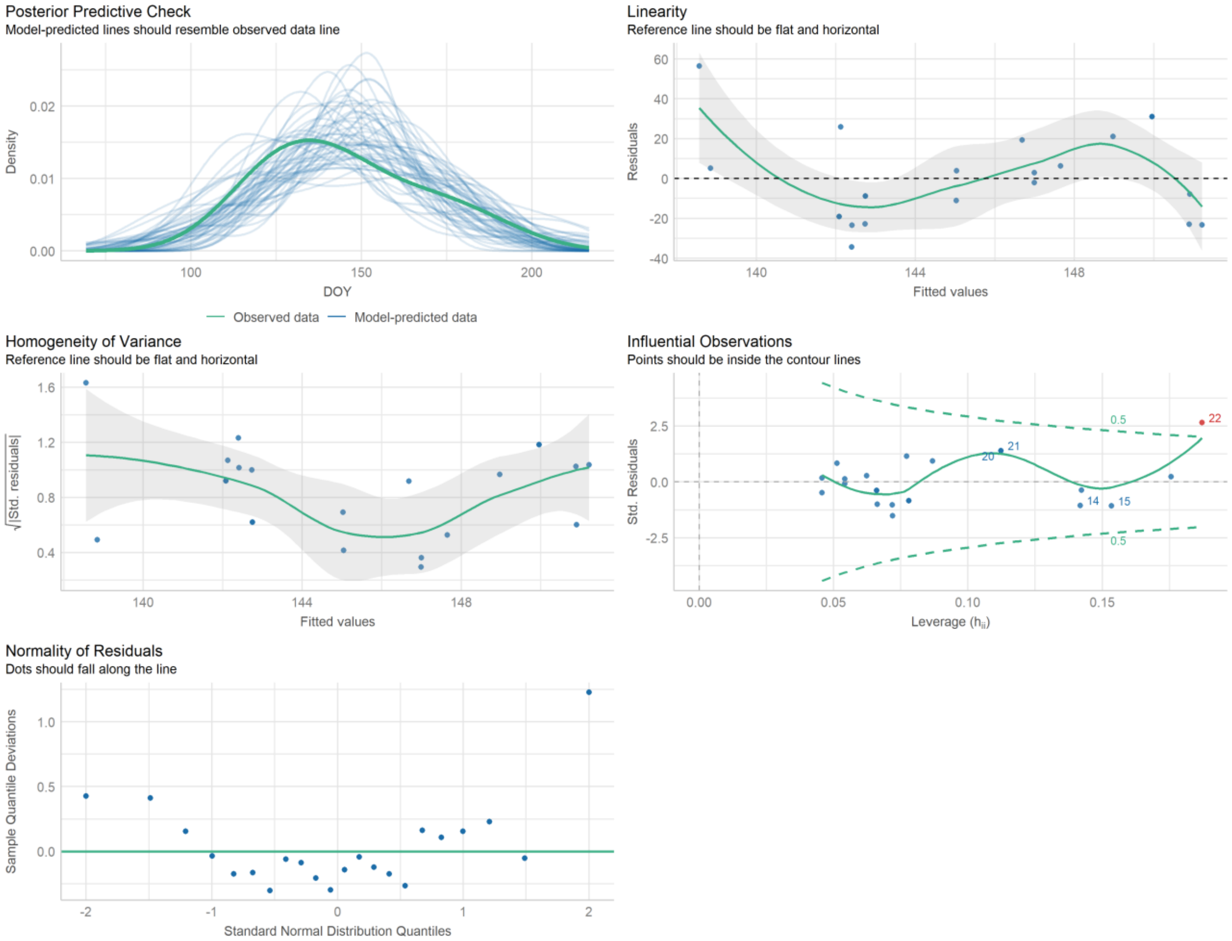

1.11. LM - FBF - Centaurea prolongoi

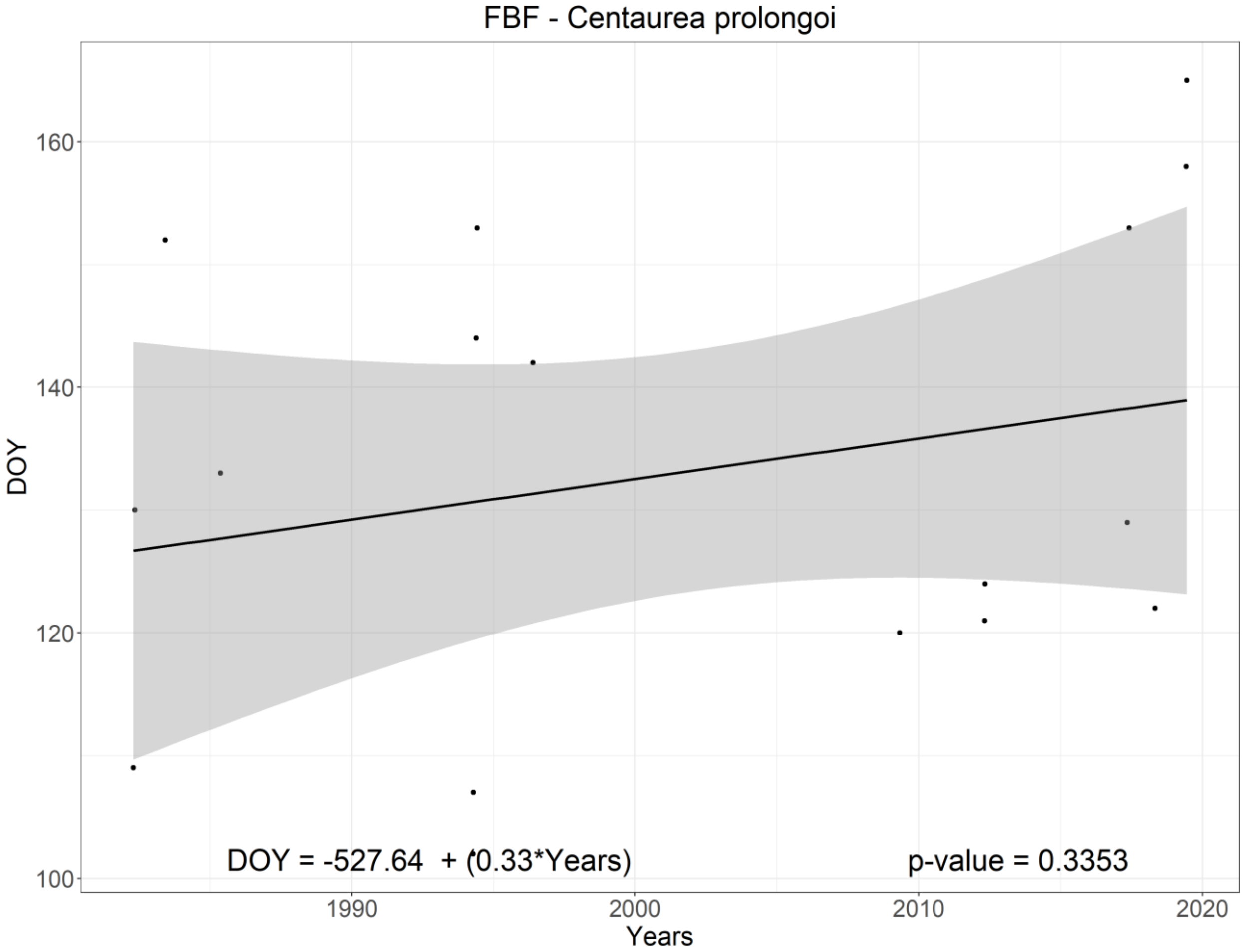

1.11.1. Diagnostics - LM - FBF - Centaurea prolongoi

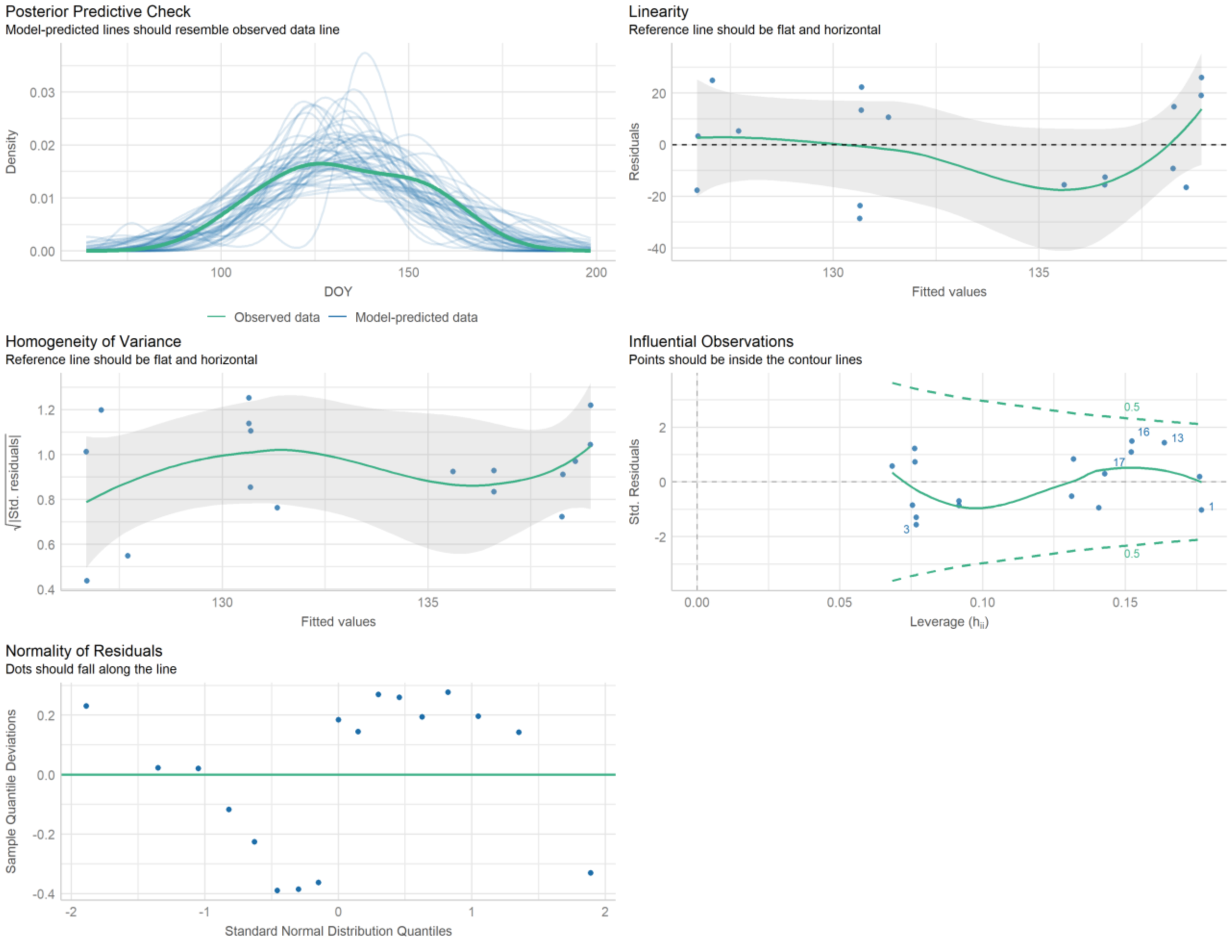

1.12. LM - F - Centaurea prolongoi

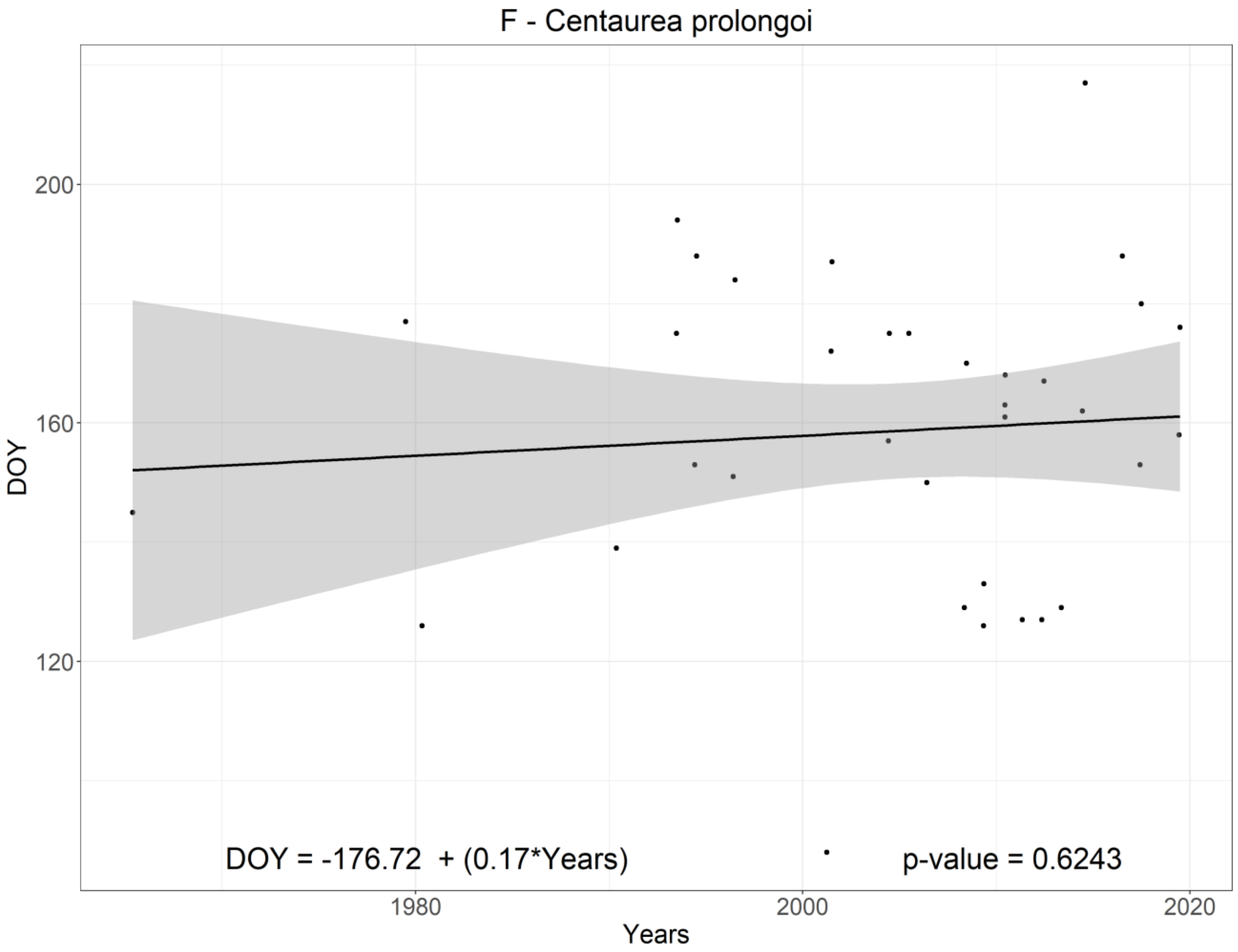

1.12.1. Diagnostics - LM - F - Centaurea prolongoi

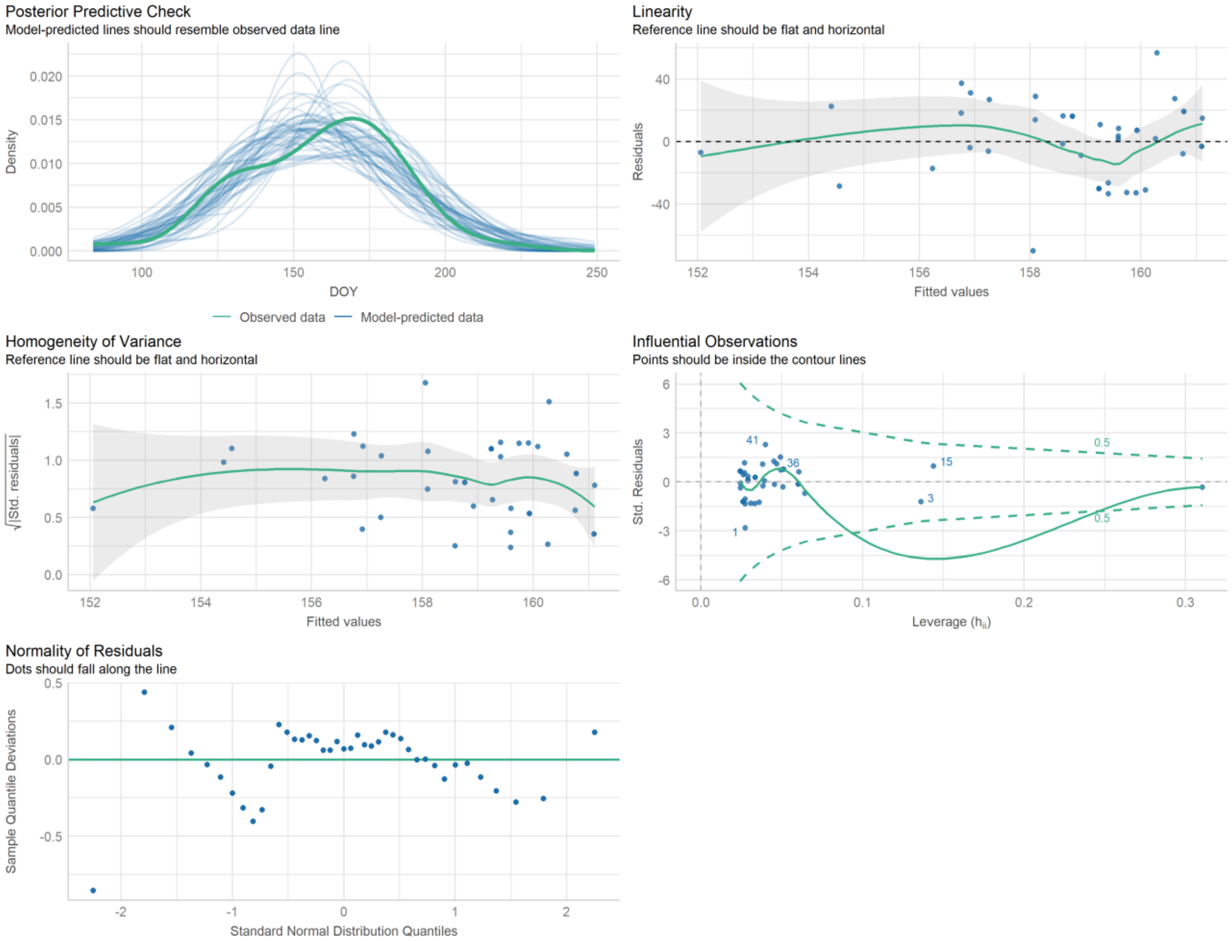

1.13. LM - F - Ceratonia siliqua

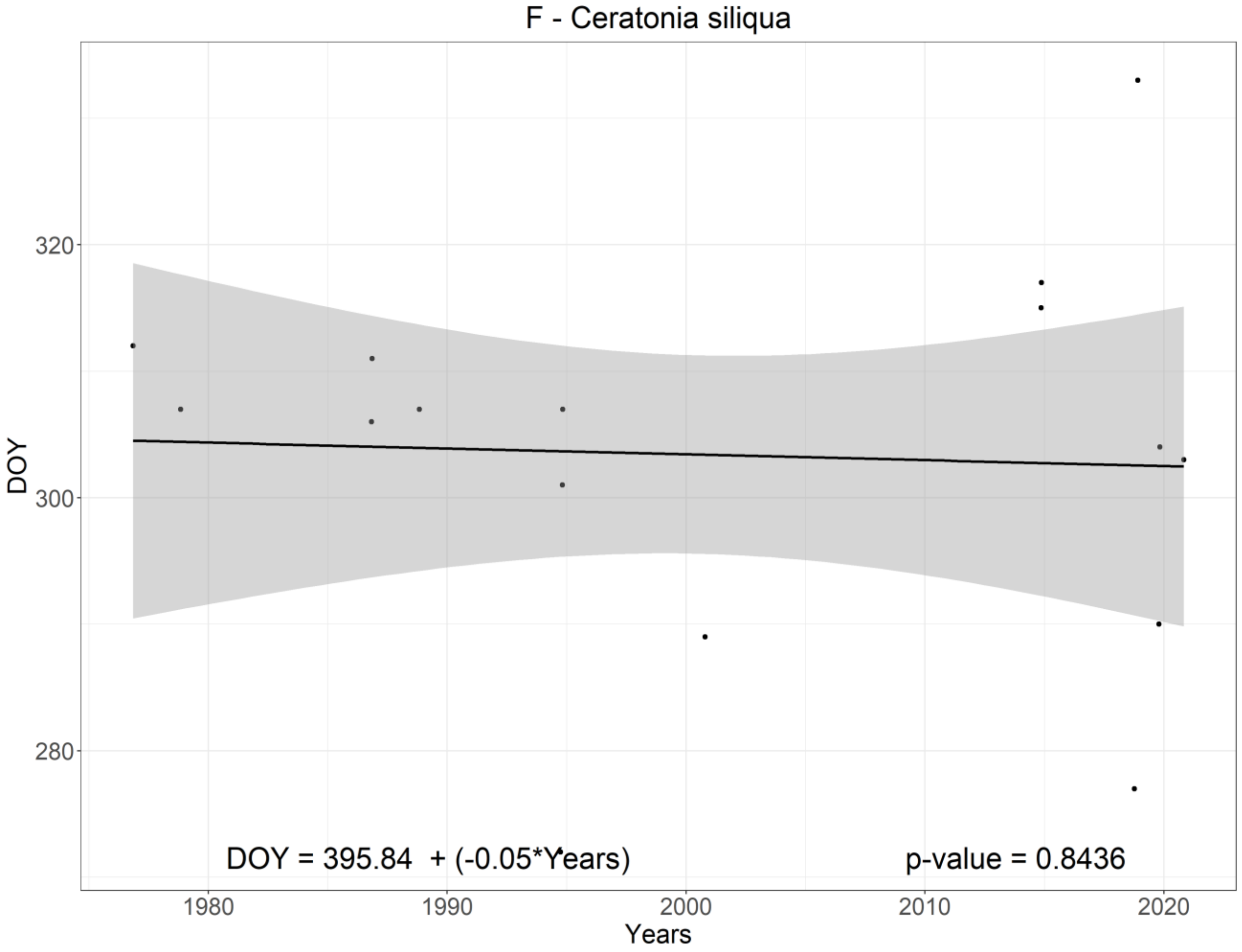

1.13.1. Diagnostics - LM - F - Ceratonia siliqua

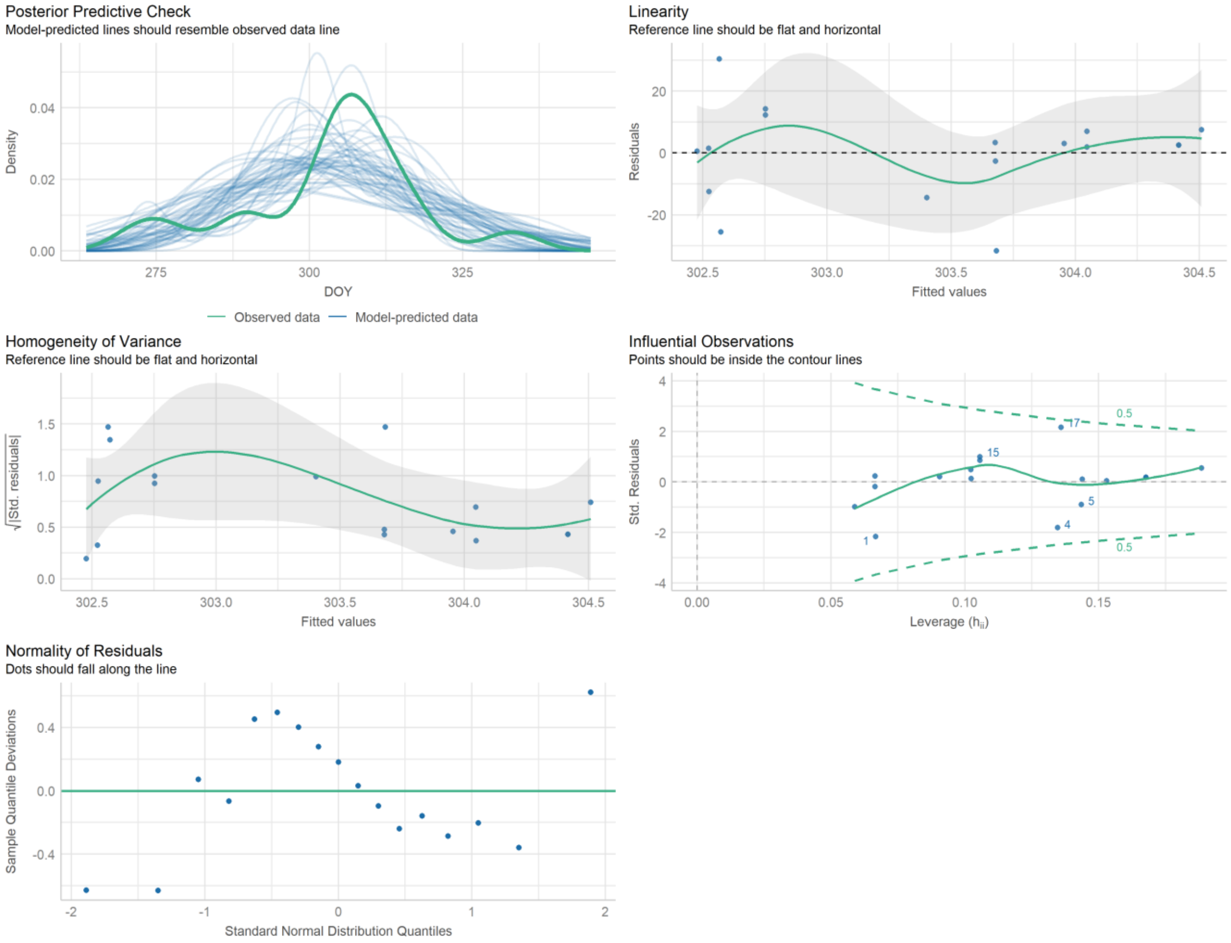

1.14. LM - F - Chaenorhinum glareosum

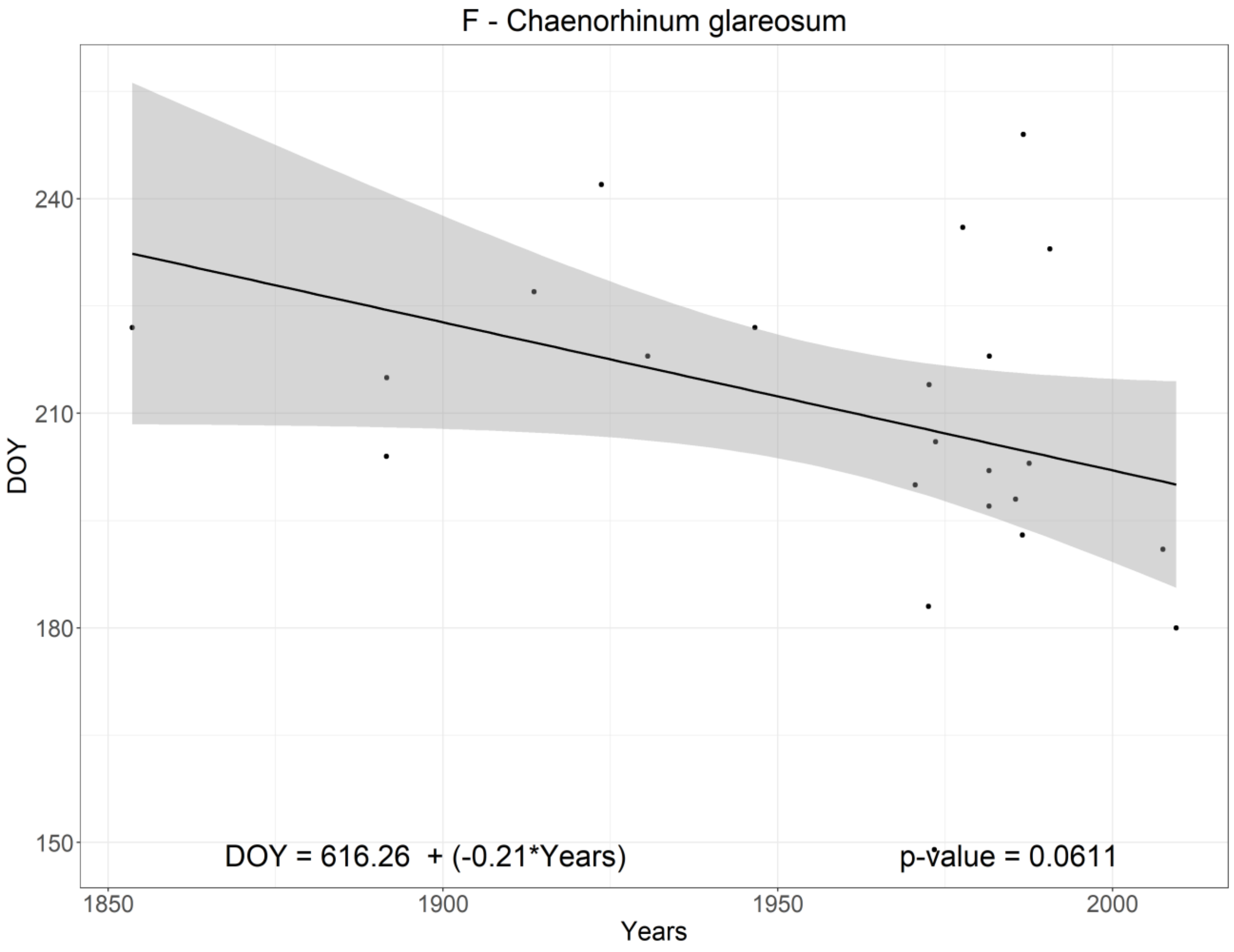

1.14.1. Diagnostics - LM - F - Chaenorhinum glareosum

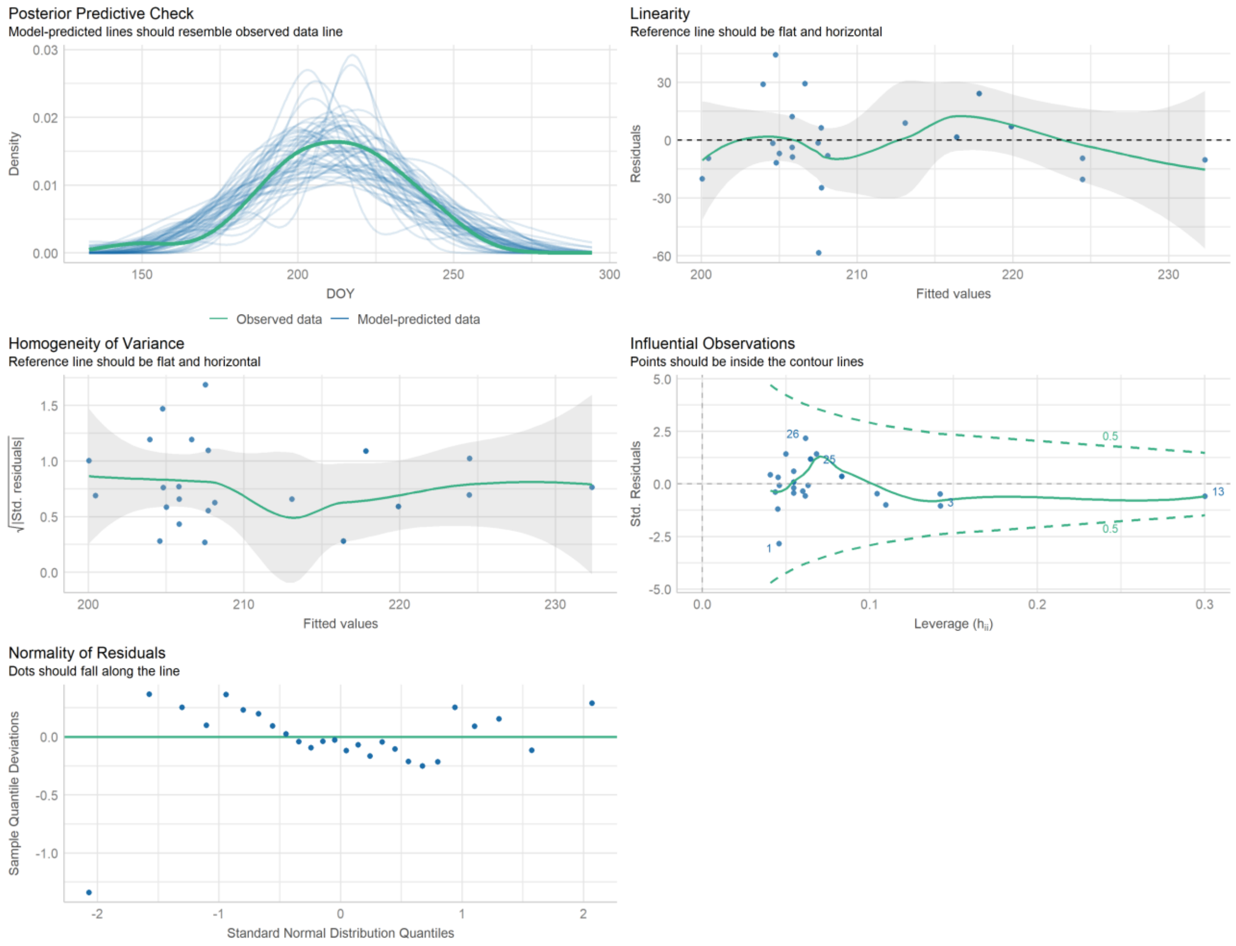

1.15. LM - FBF - Cistus albidus

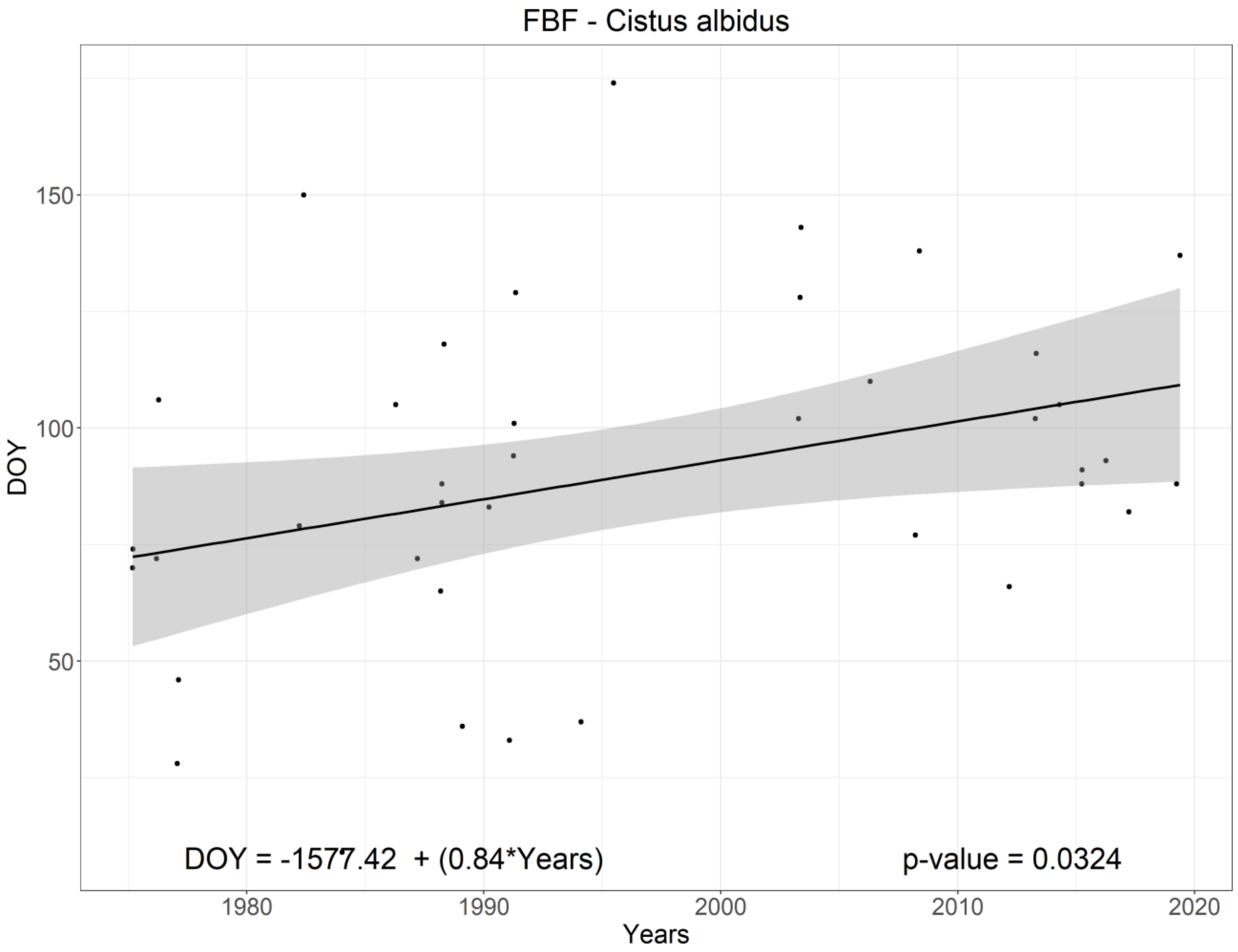

1.15.1. Diagnostics - LM - FBF - Cistus albidus

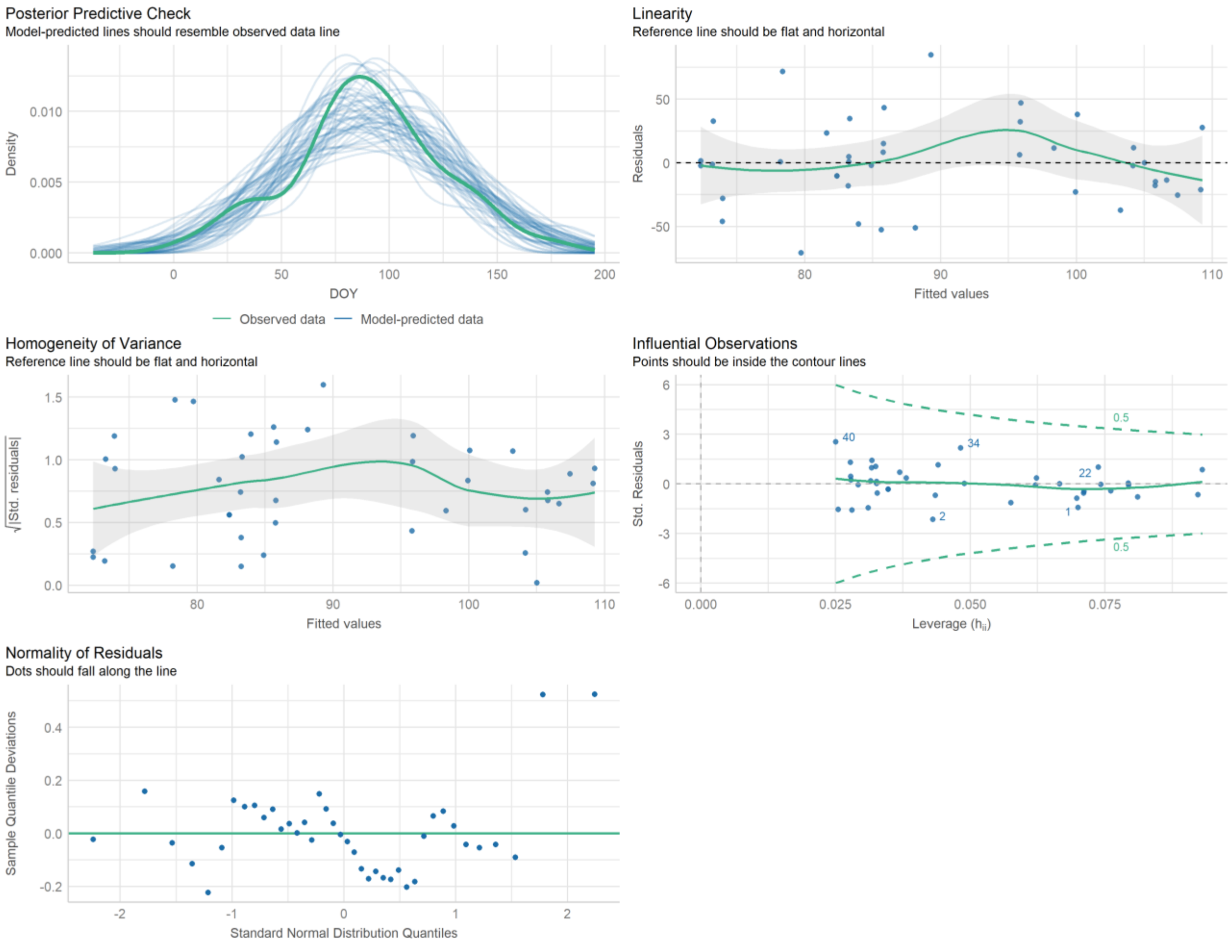

1.16. LM - F - Cistus albidus

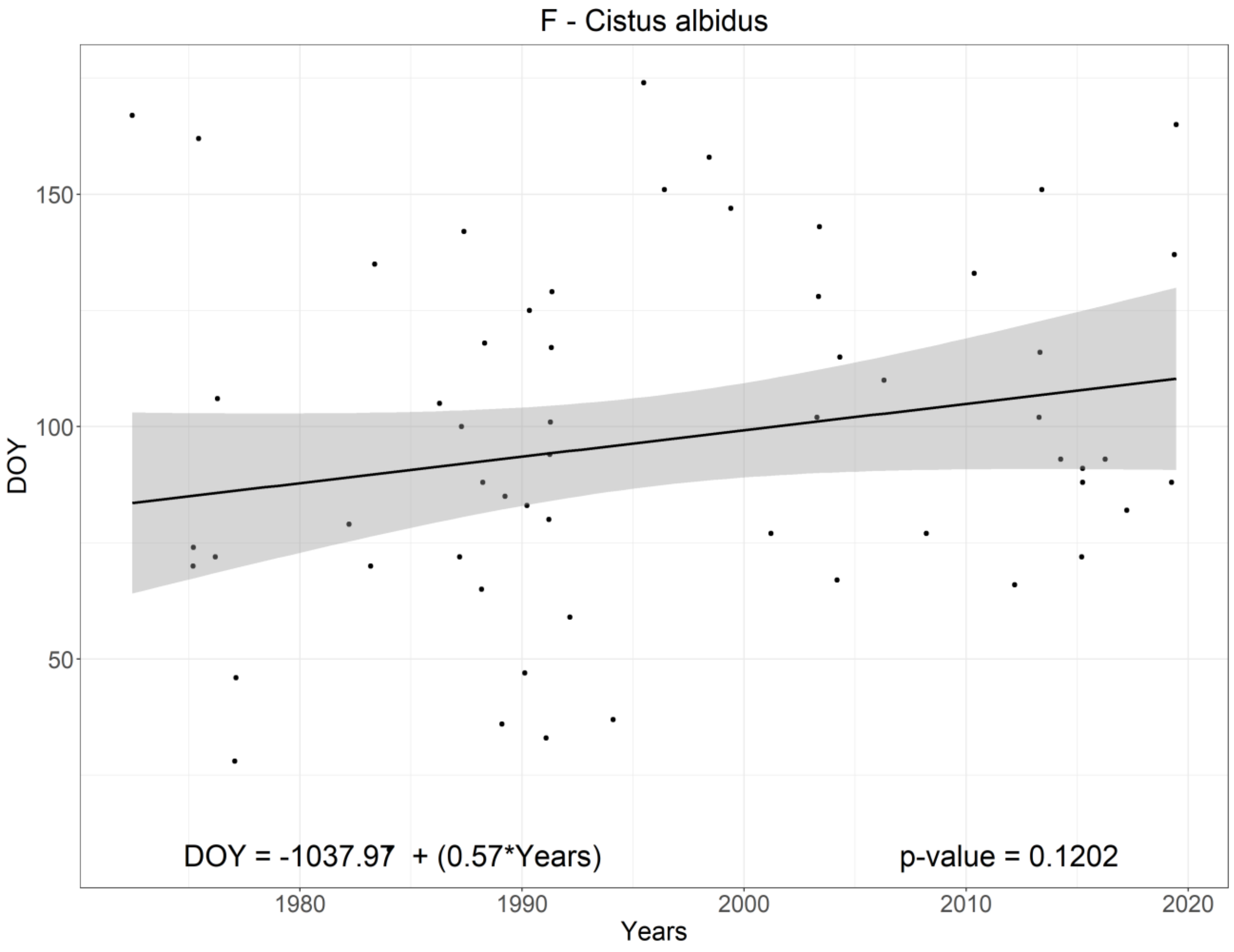

1.16.1. Diagnostics - LM - F - Cistus albidus

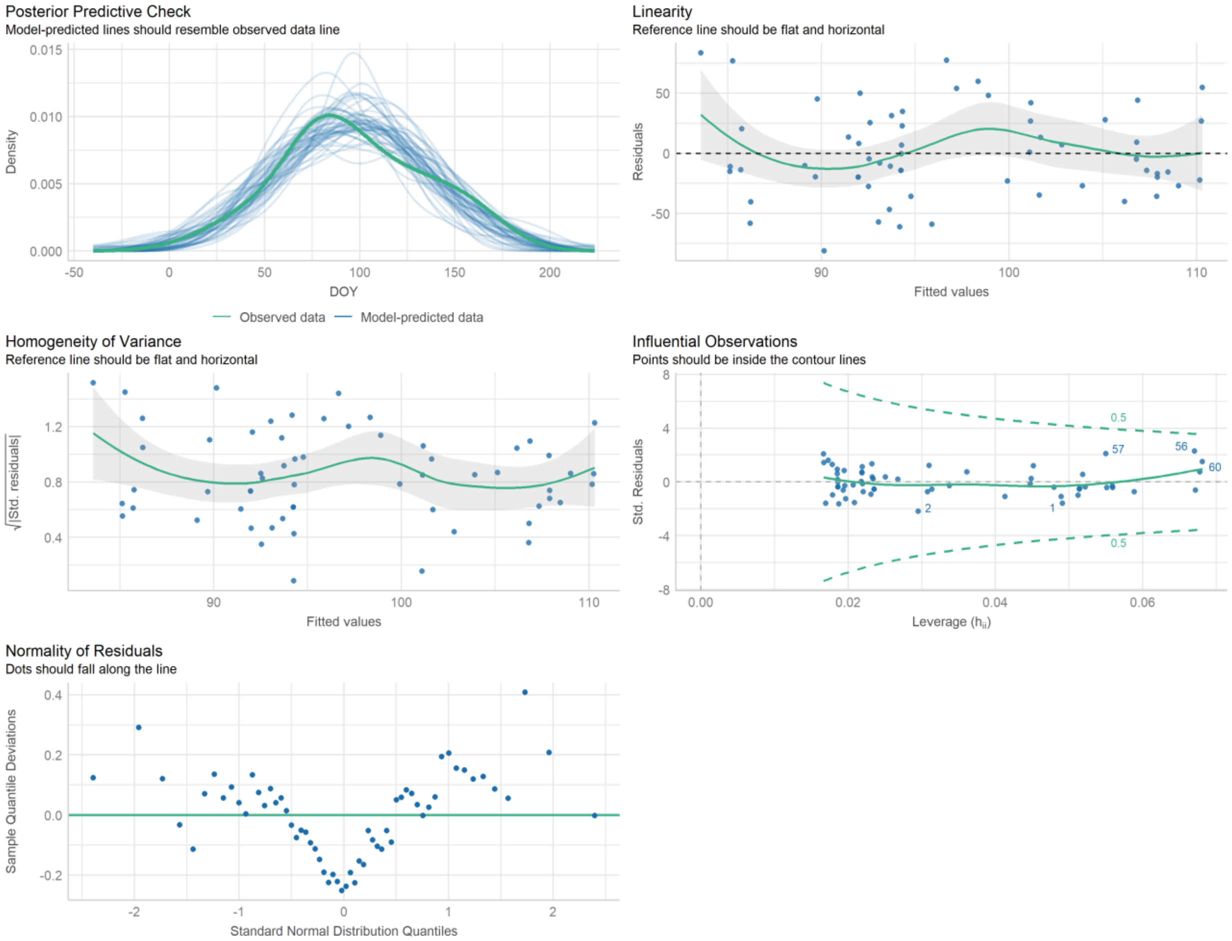

1.17. LM - DVG - Cistus albidus

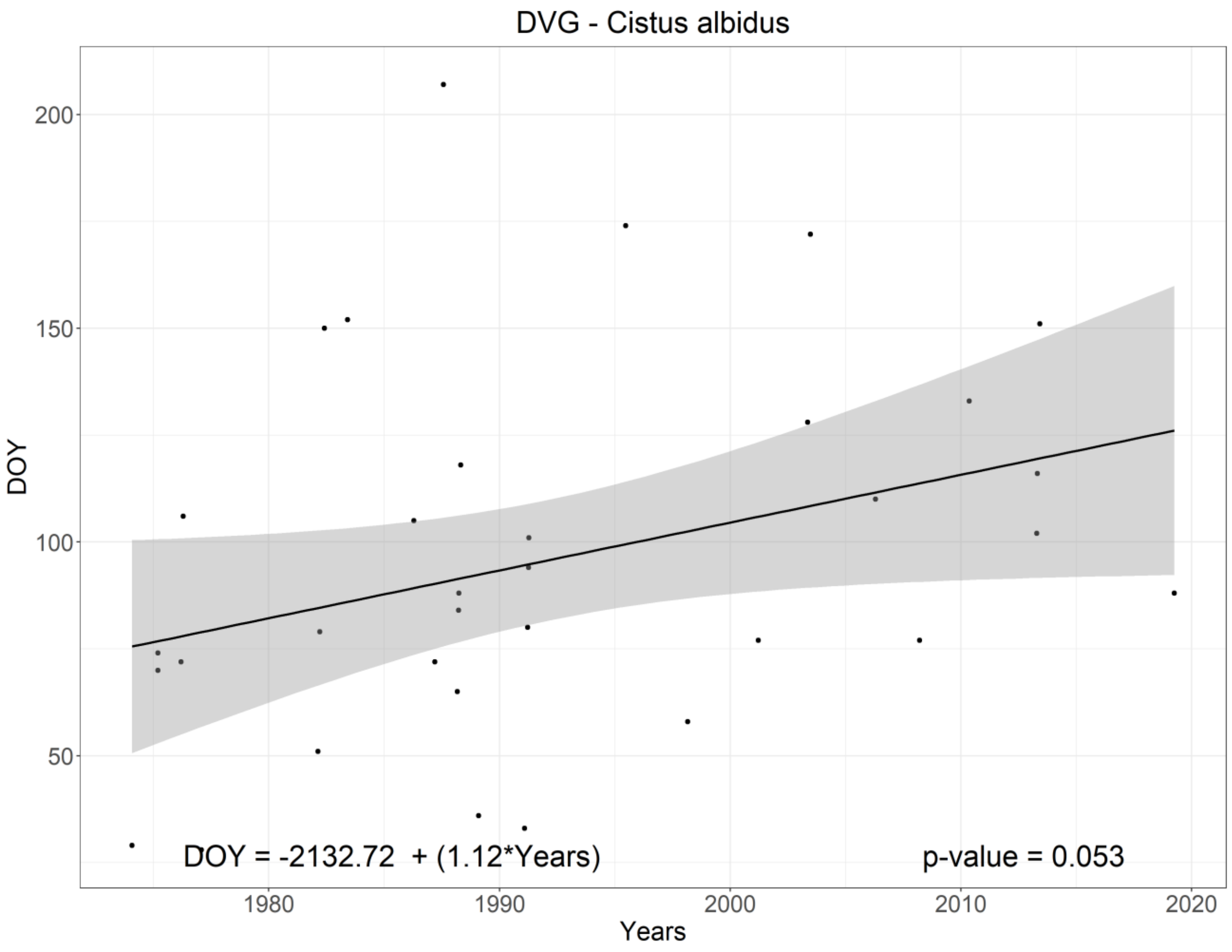

1.17.1. Diagnostics - LM - DVG - Cistus albidus

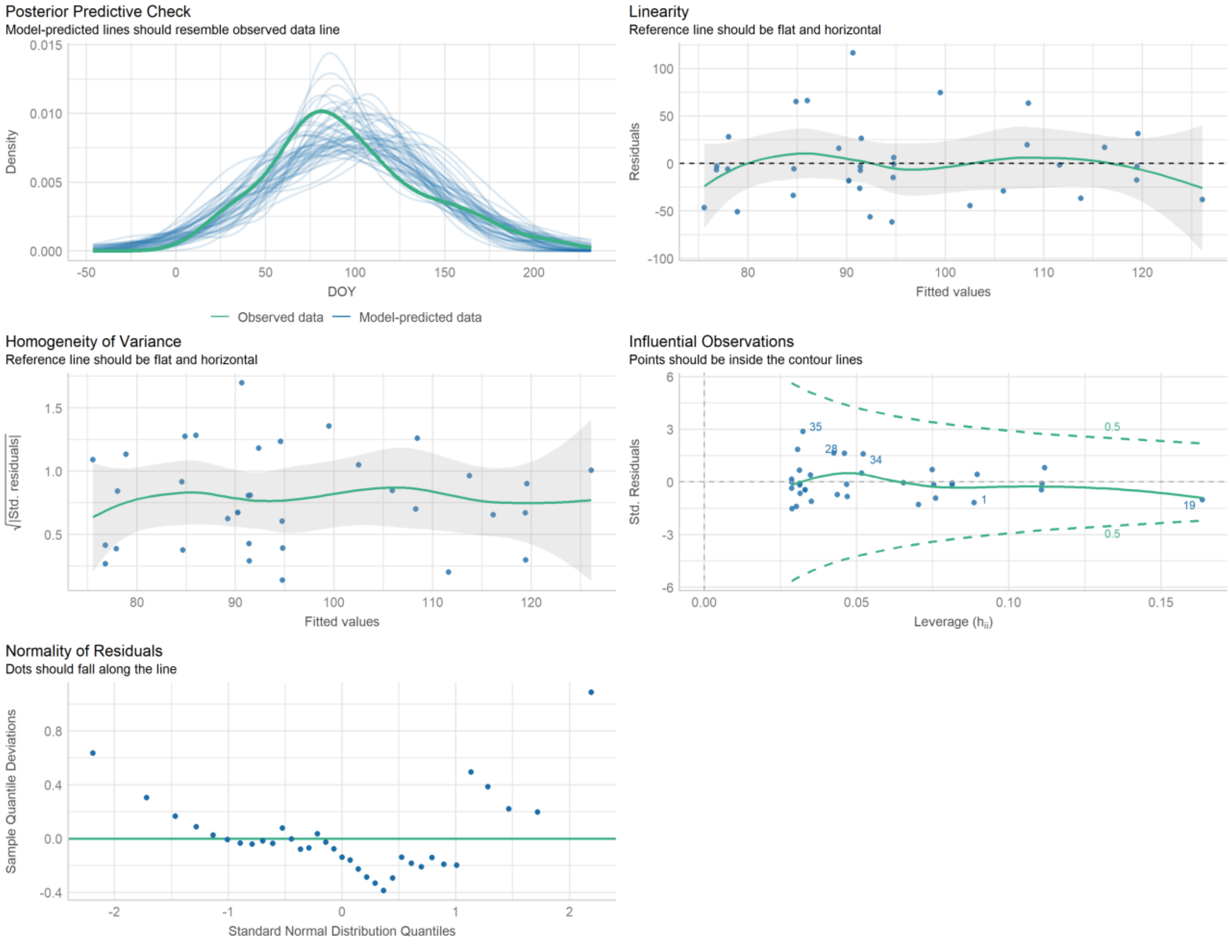

1.18. LM - FBF - Cistus ladanifer

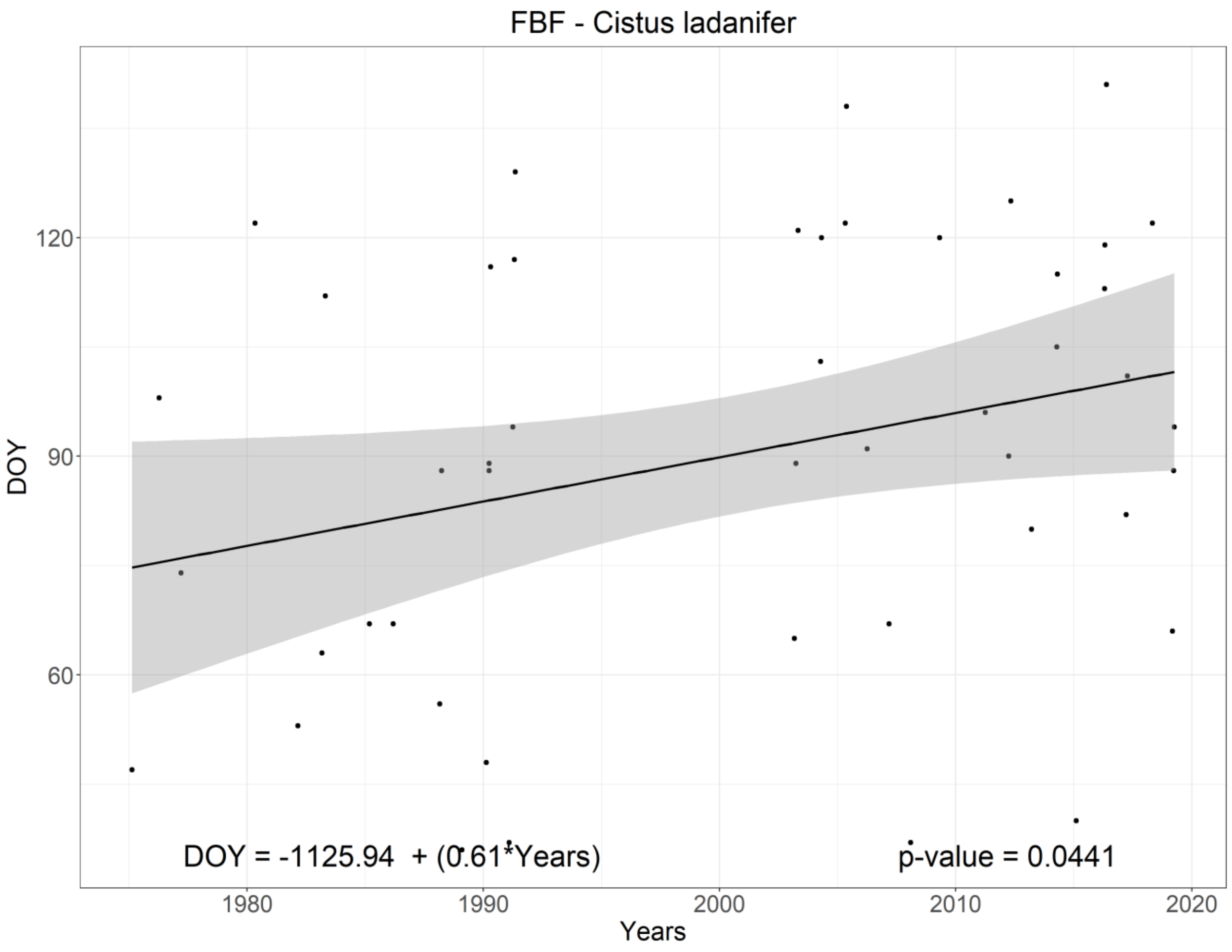

1.18.1. Diagnostics - LM - FBF - Cistus ladanifer

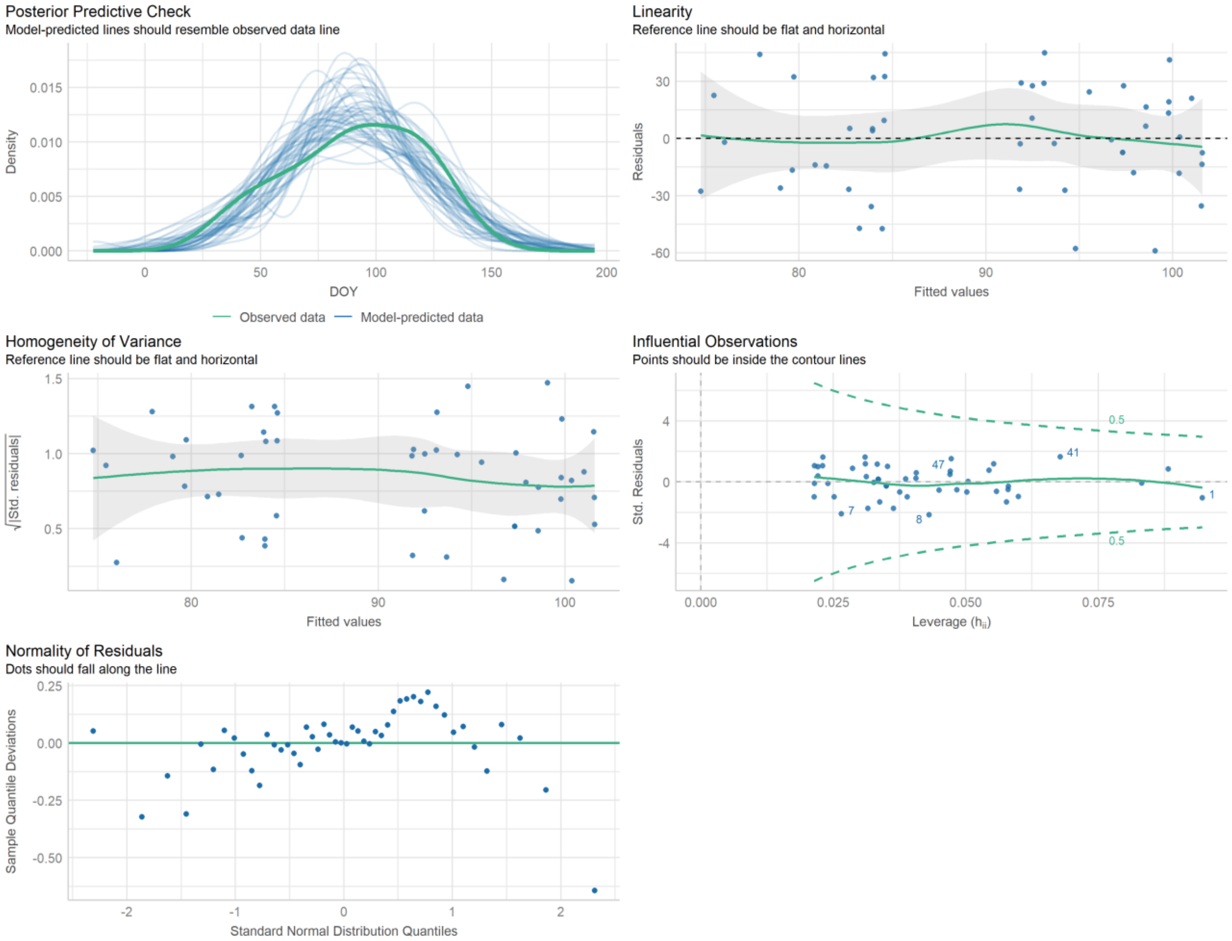

1.19. LM - F - Cistus ladanifer

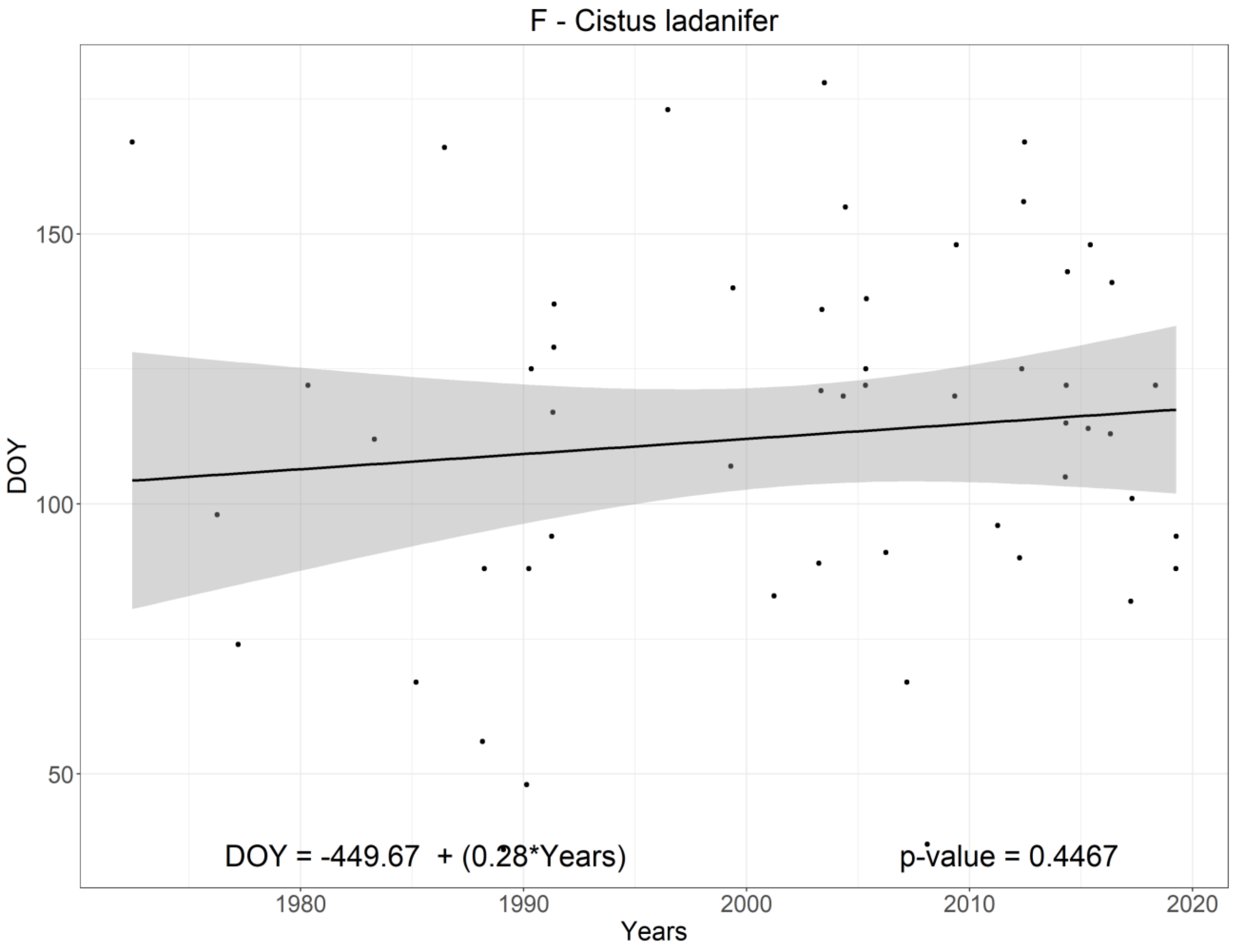

1.19.1. Diagnostics - LM - F - Cistus ladanifer

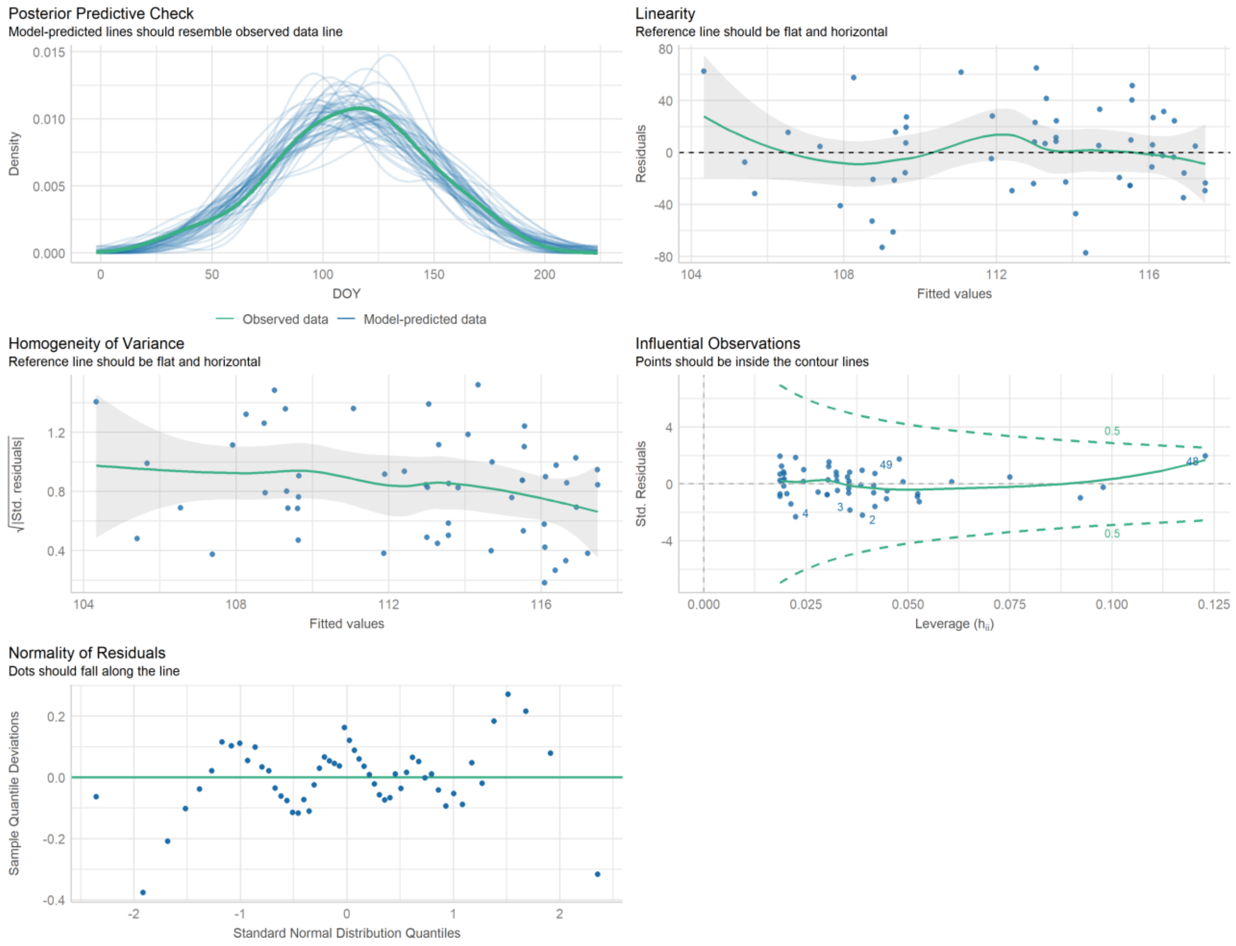

1.20. LM - FS - Cistus ladanifer

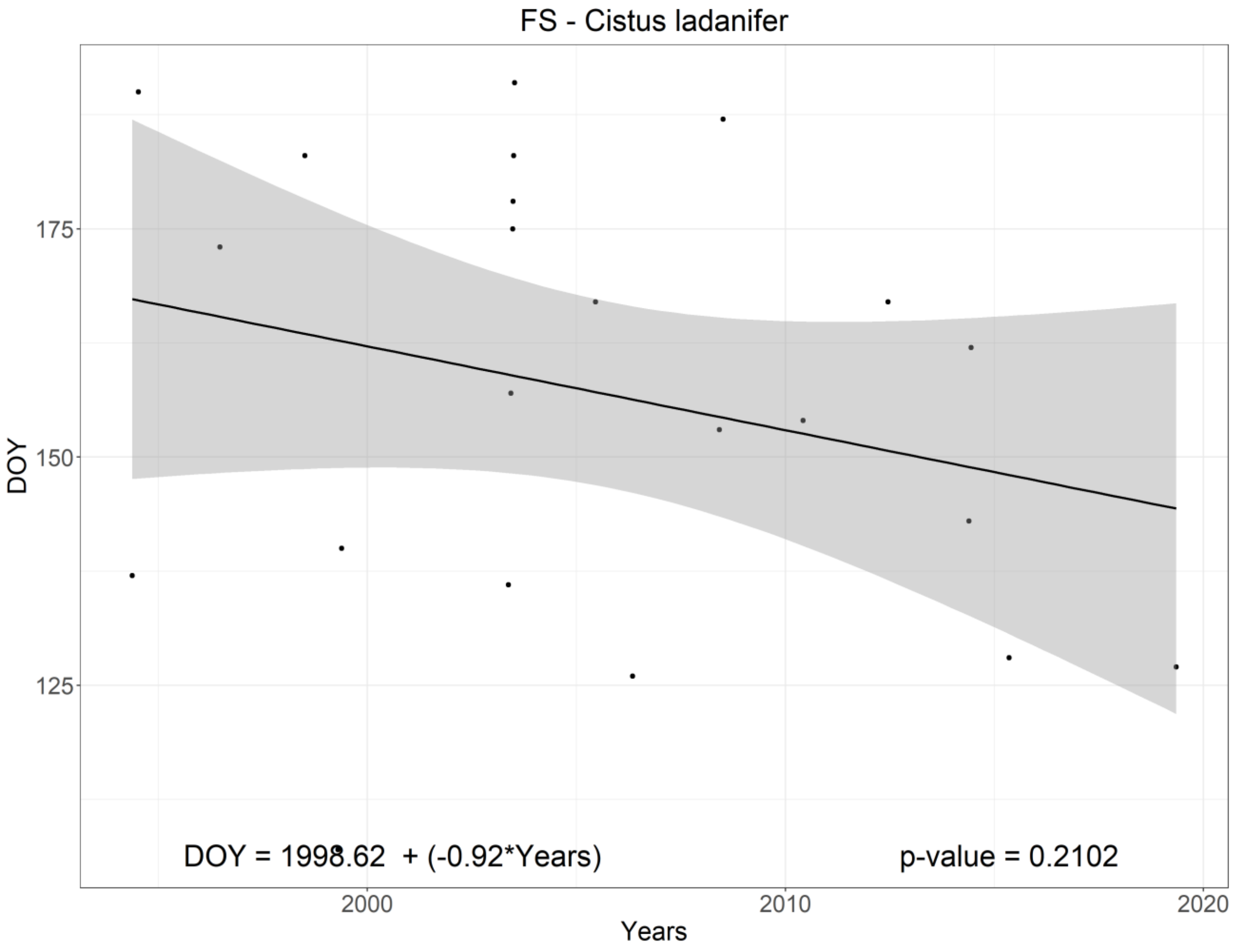

1.20.1. Diagnostics - LM - FS - Cistus ladanifer

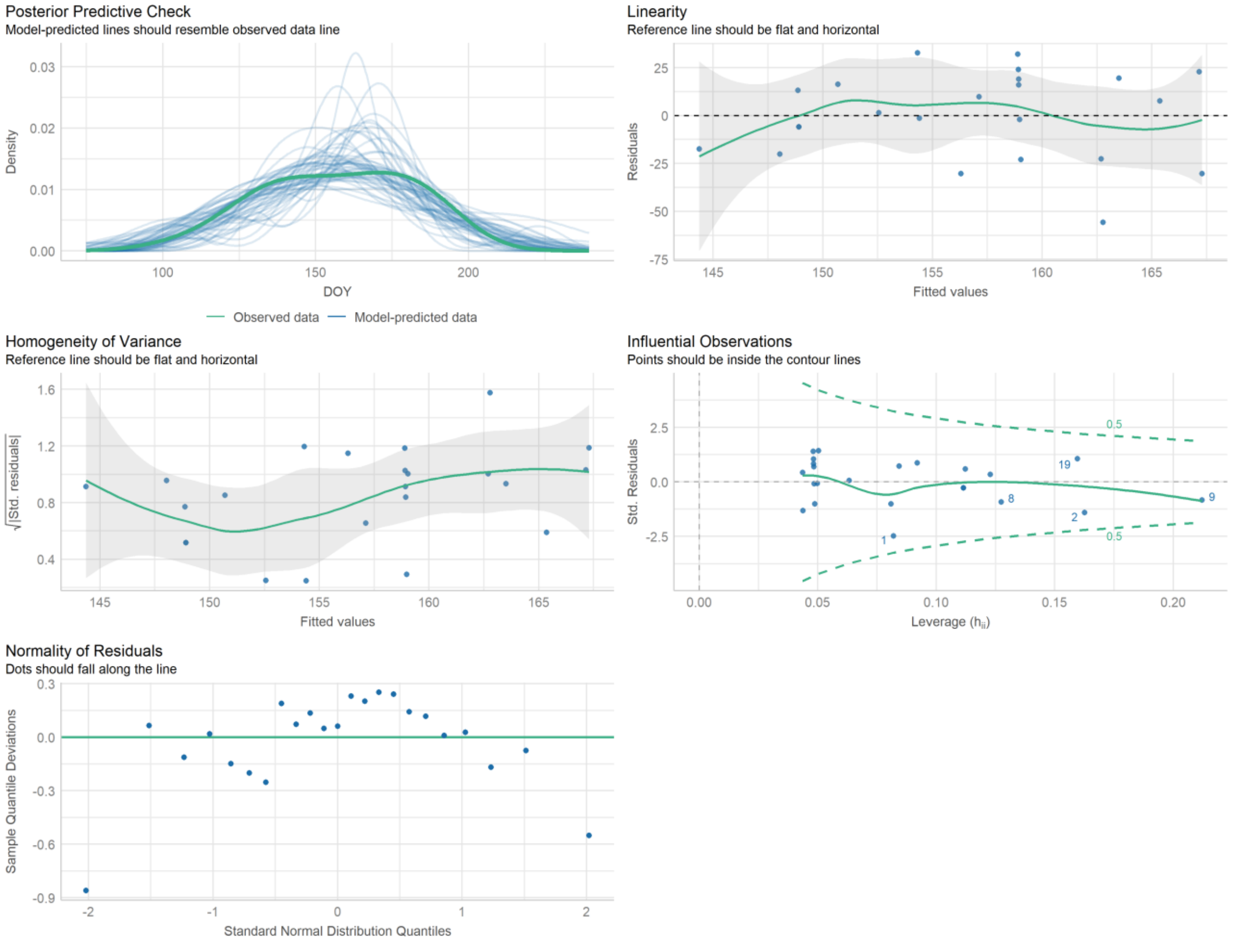

1.21. LM - DVG - Cistus ladanifer

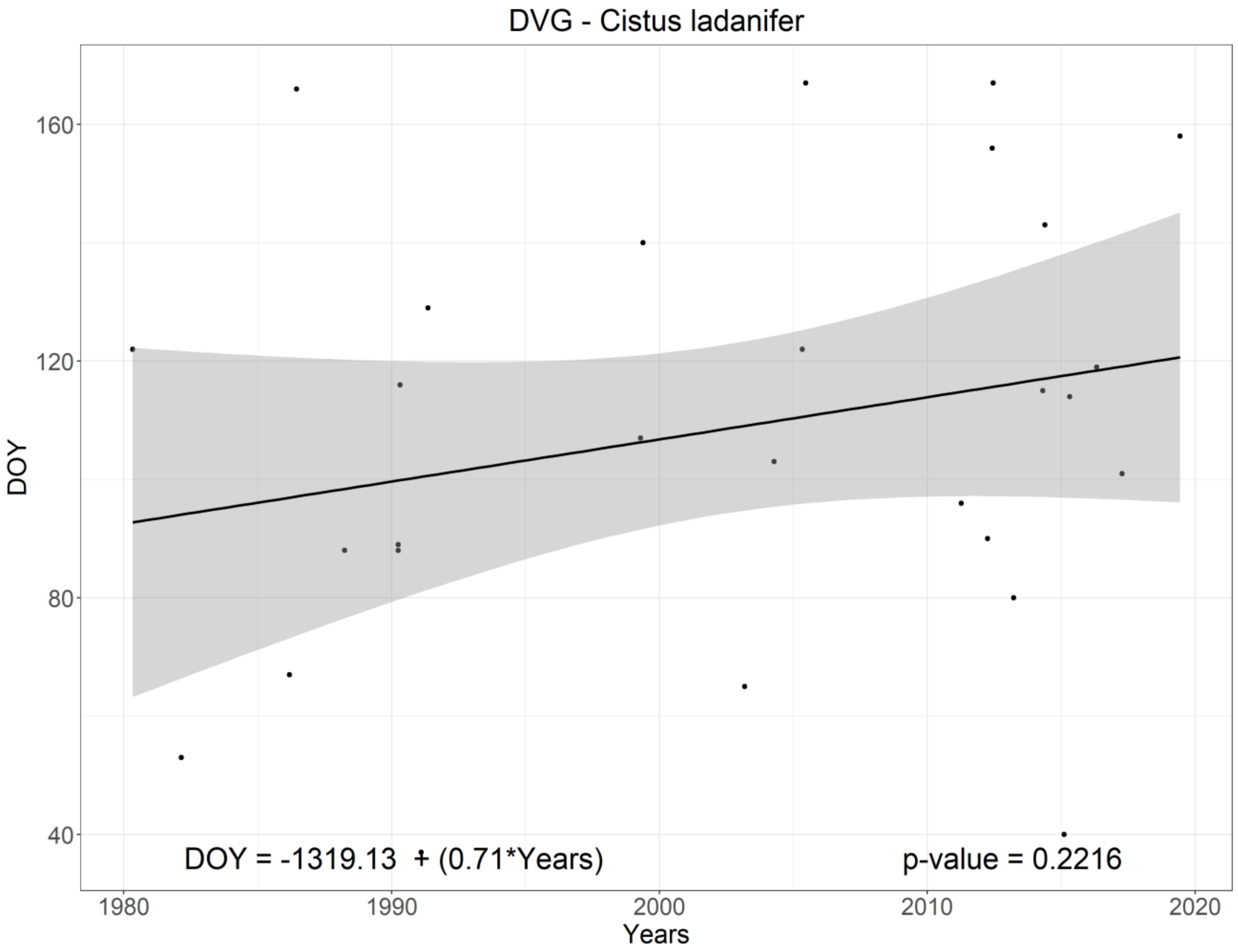

1.21.1. Diagnostics - LM - DVG - Cistus ladanifer

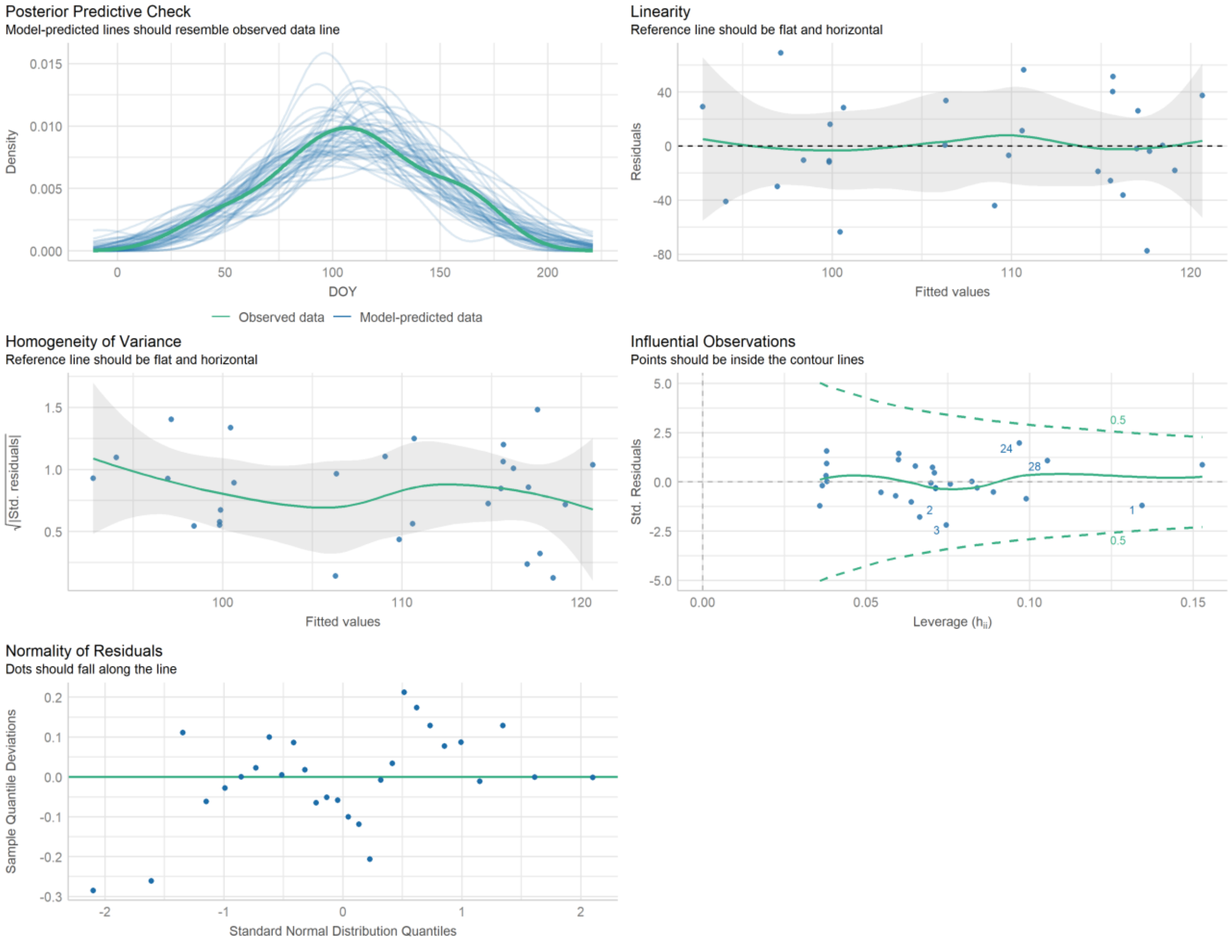

1.22. LM - FBF - Cistus laurifolius

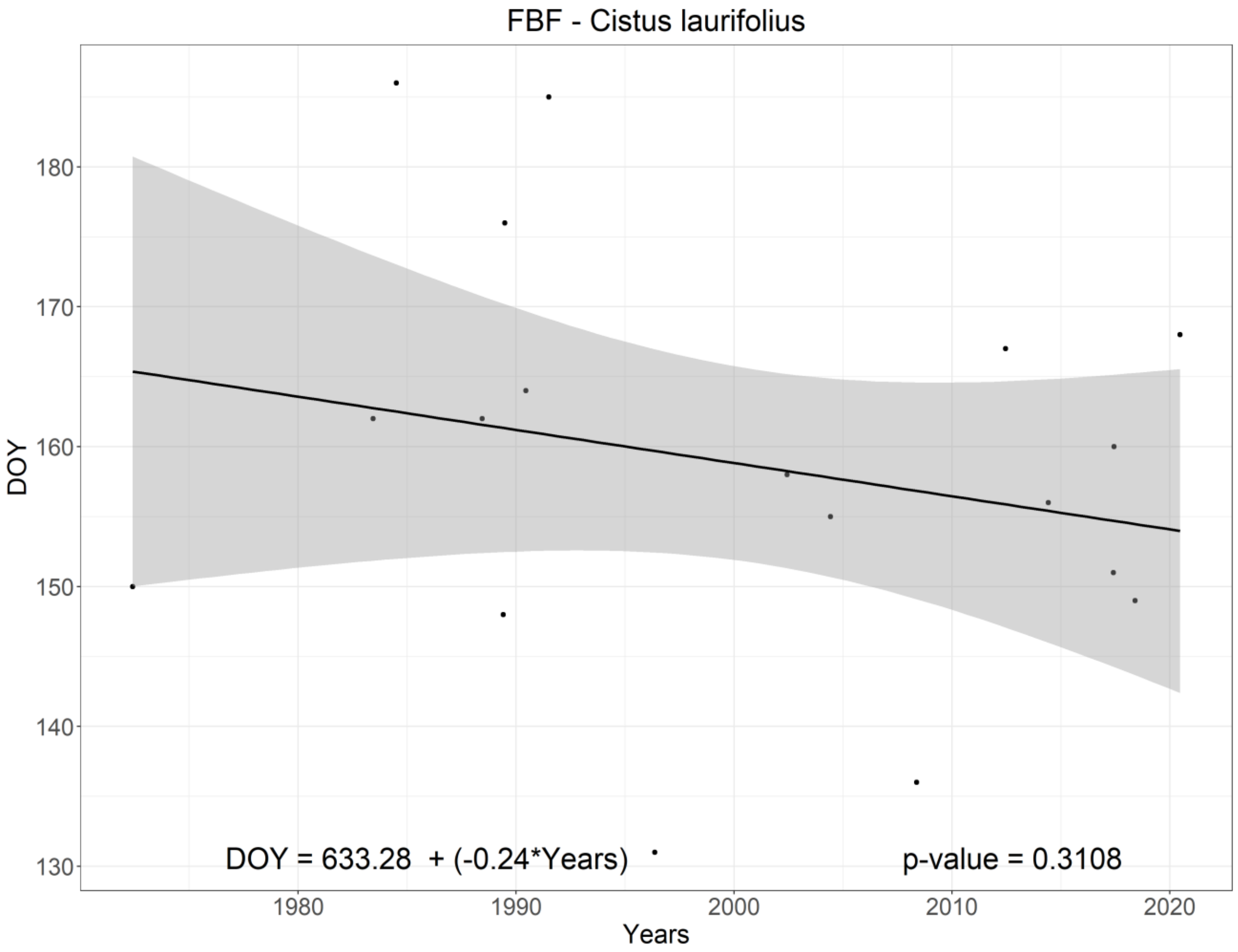

1.22.1. Diagnostics - LM - FBF - Cistus laurifolius

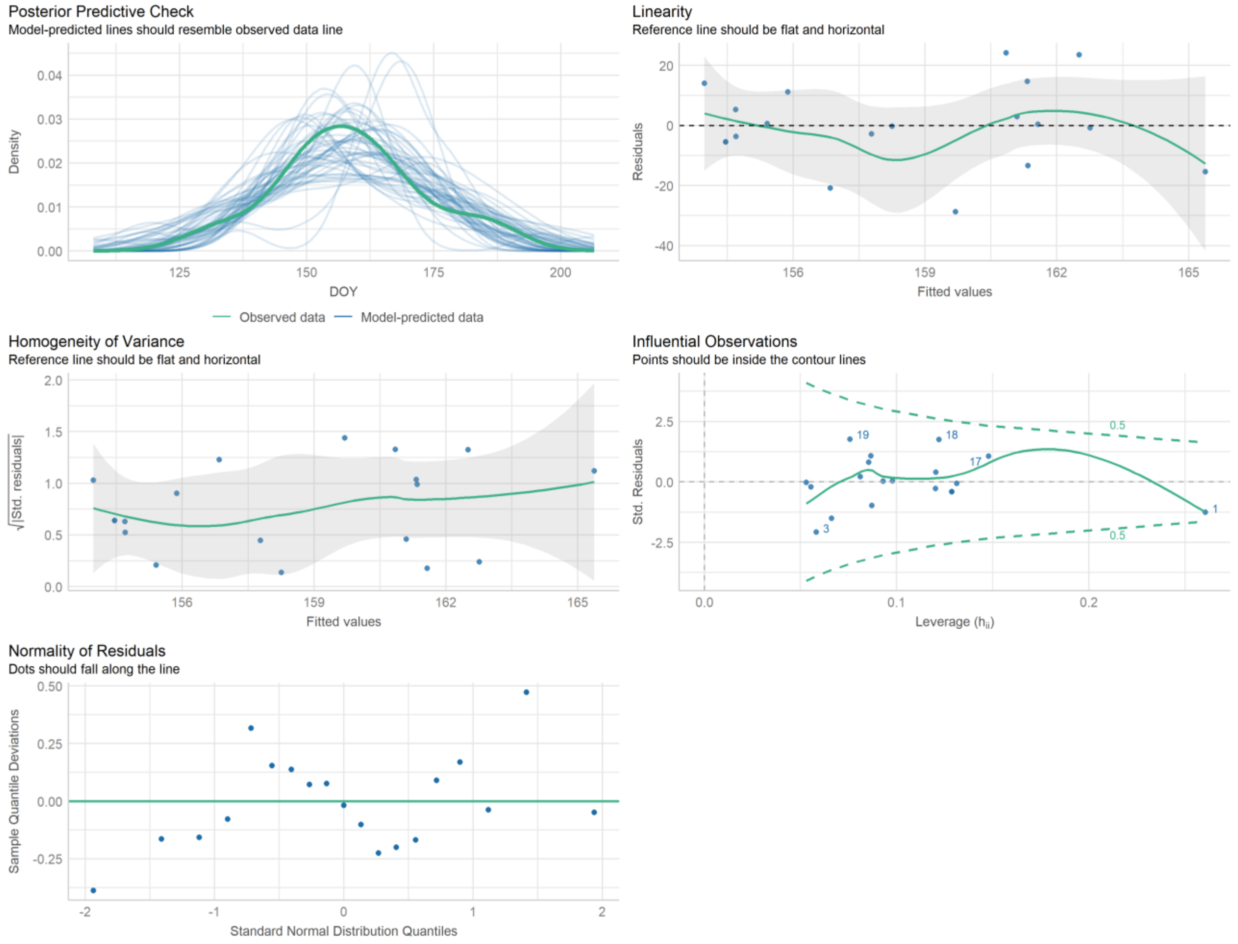

1.23. LM - F - Cistus laurifolius

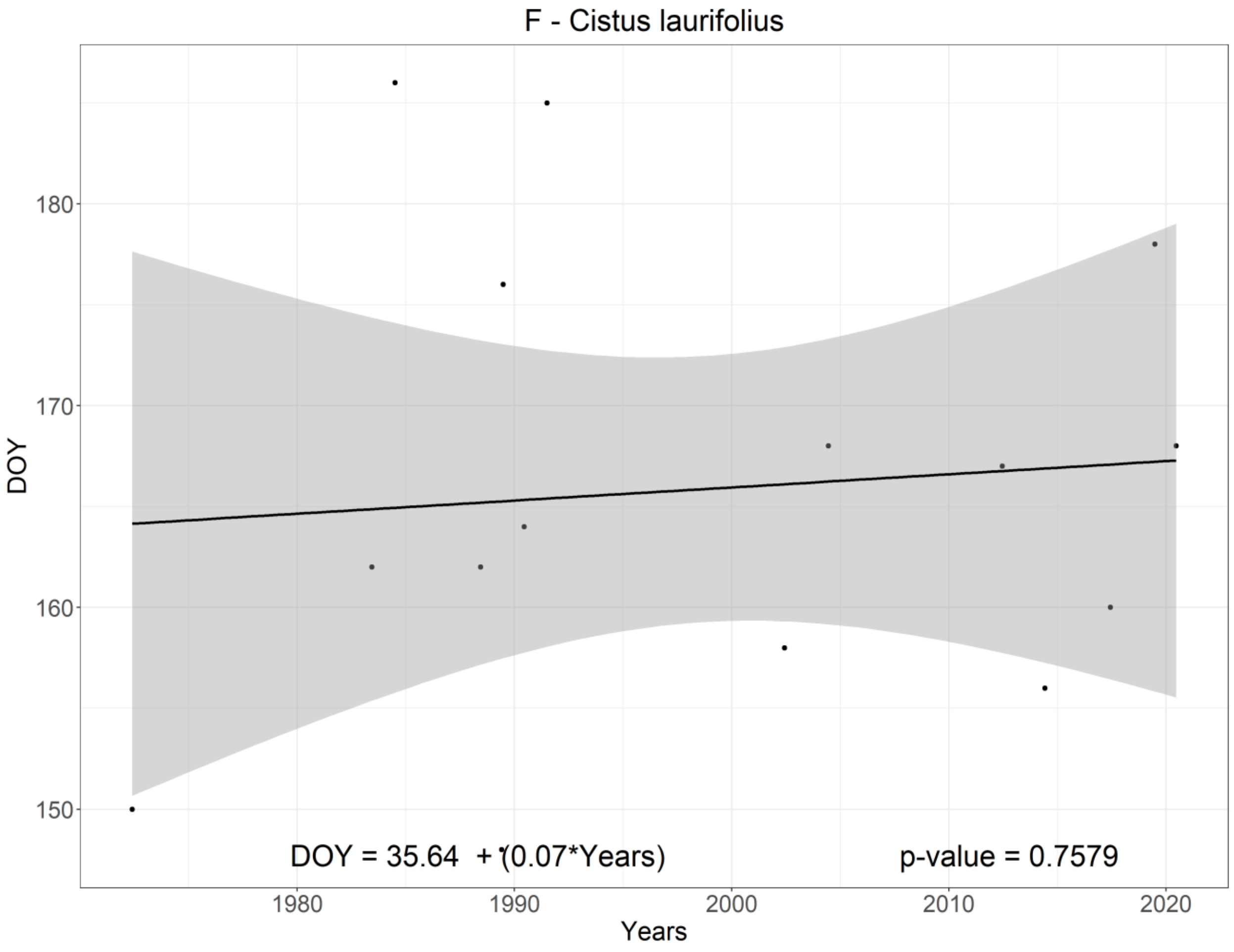

1.23.1. Diagnostics - LM - F - Cistus laurifolius

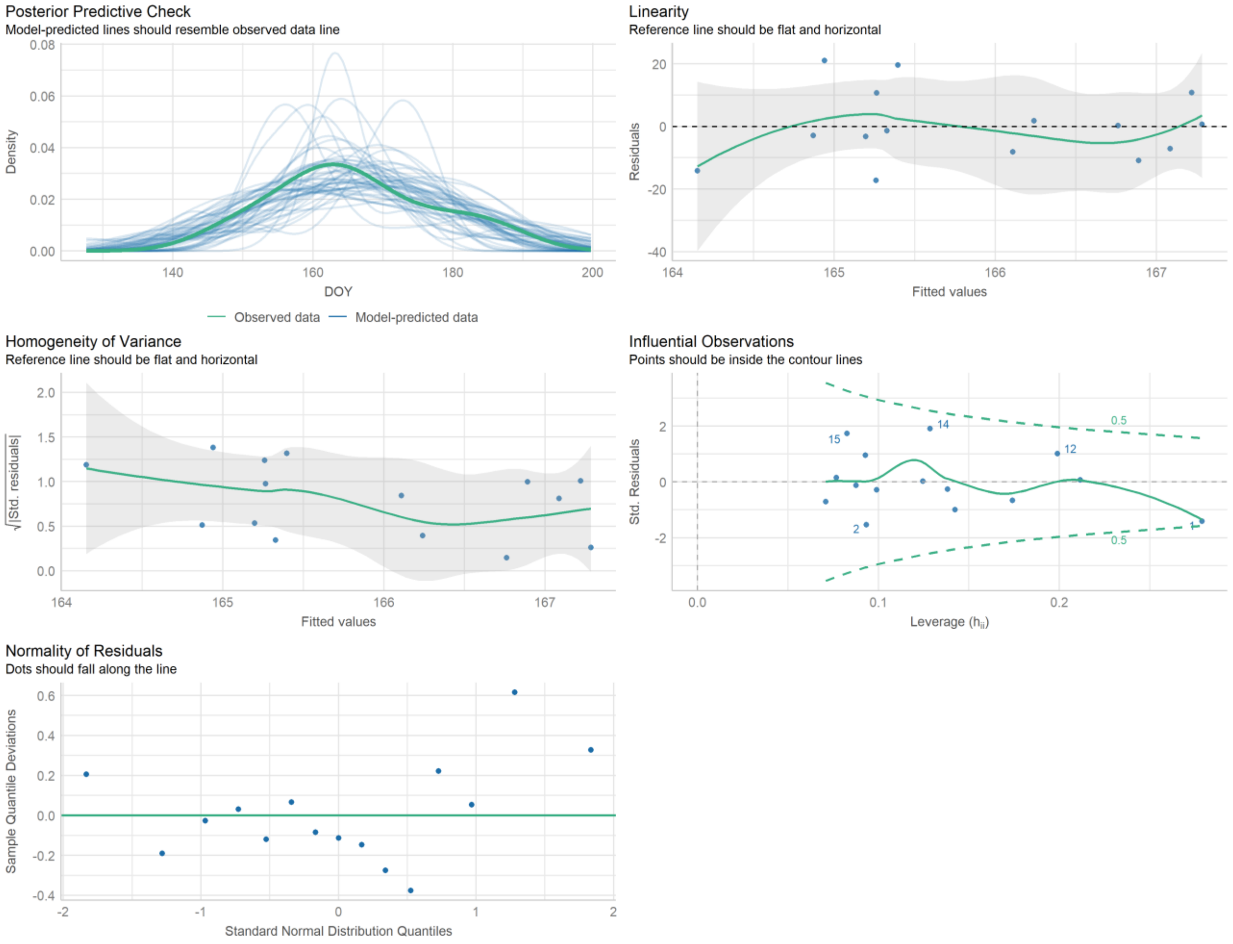

1.24. LM - FBF - Cistus populifolius

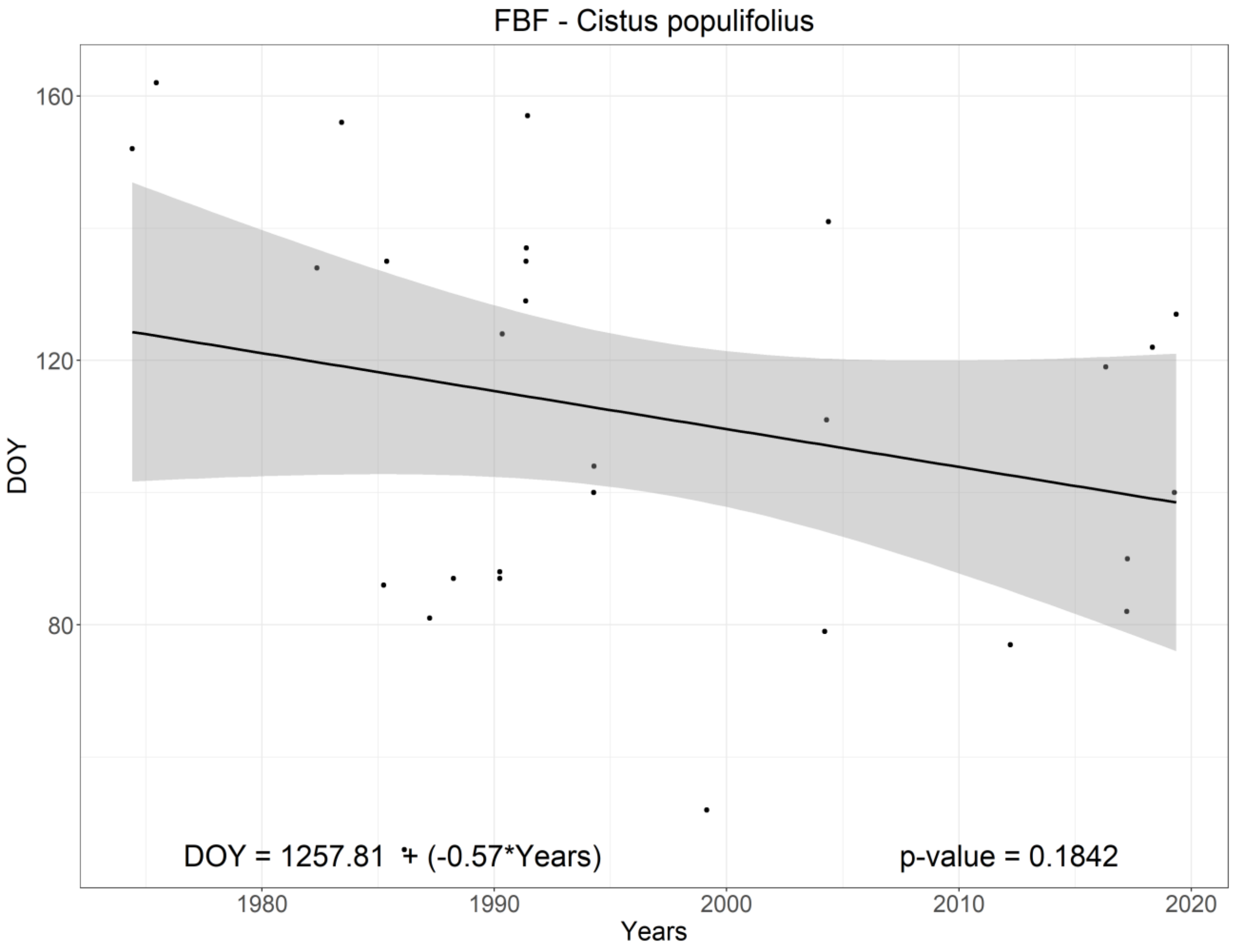

1.24.1. Diagnostics - LM - FBF - Cistus populifolius

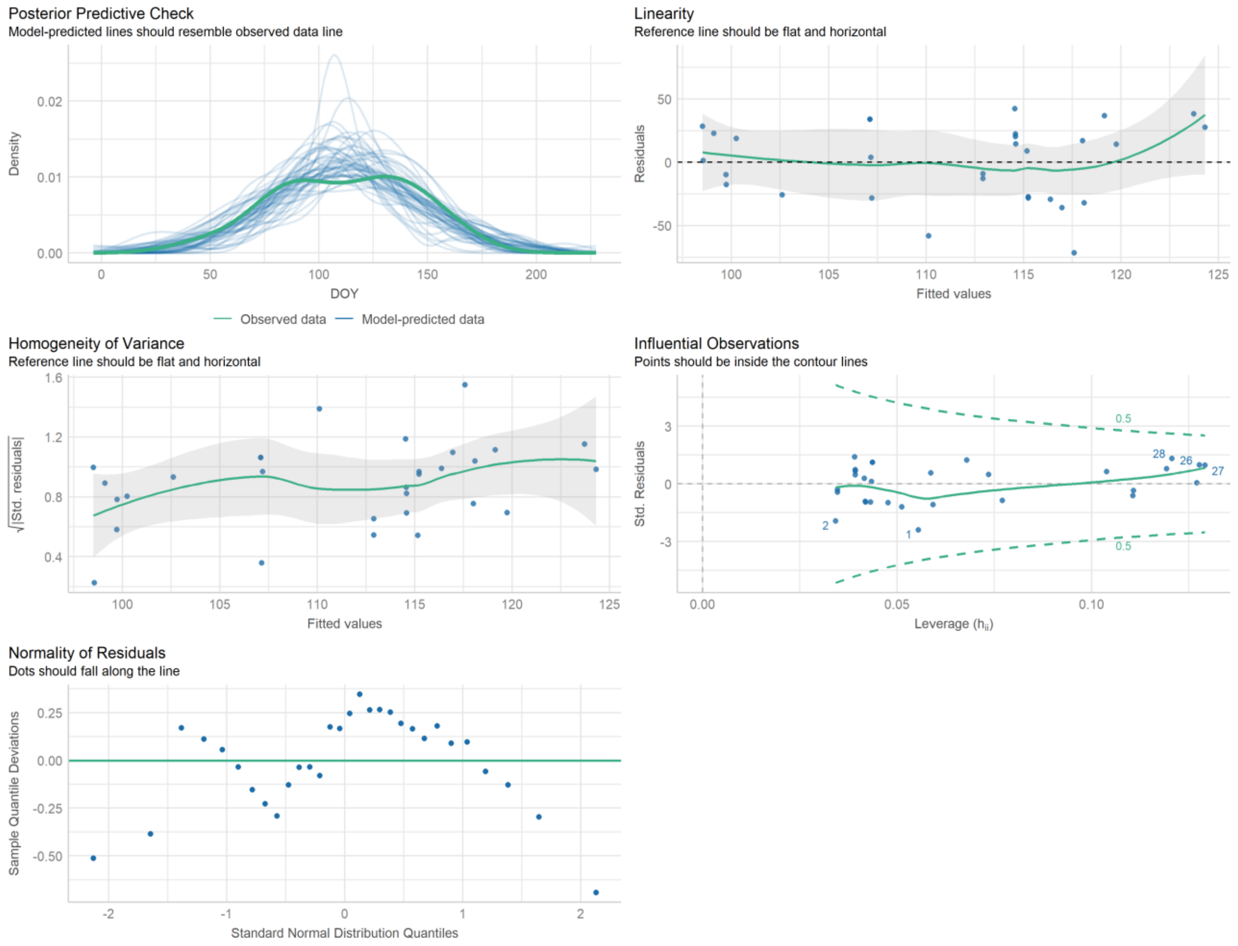

1.25. LM - F - Cistus populifolius

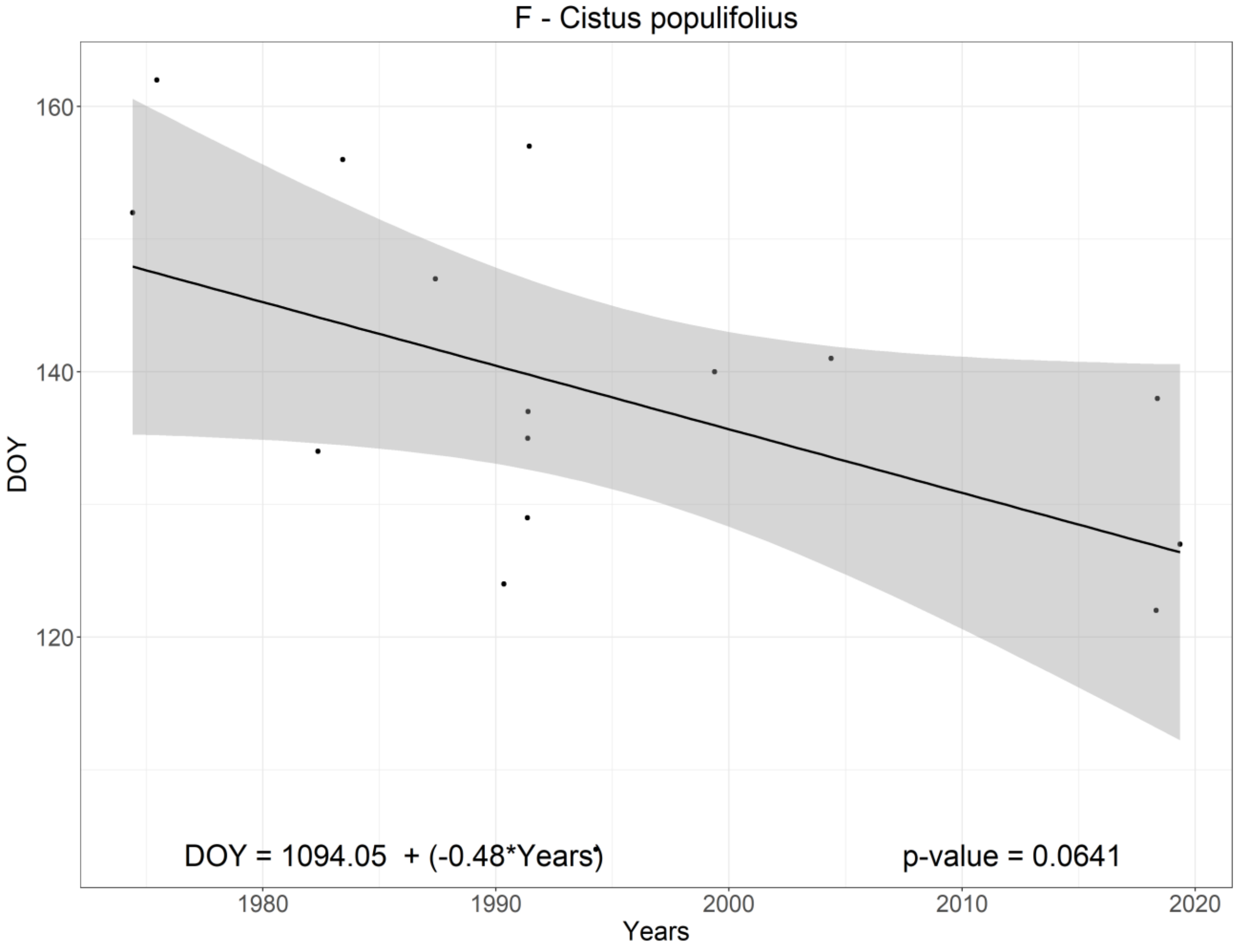

1.25.1. Diagnostics - LM - F - Cistus populifolius

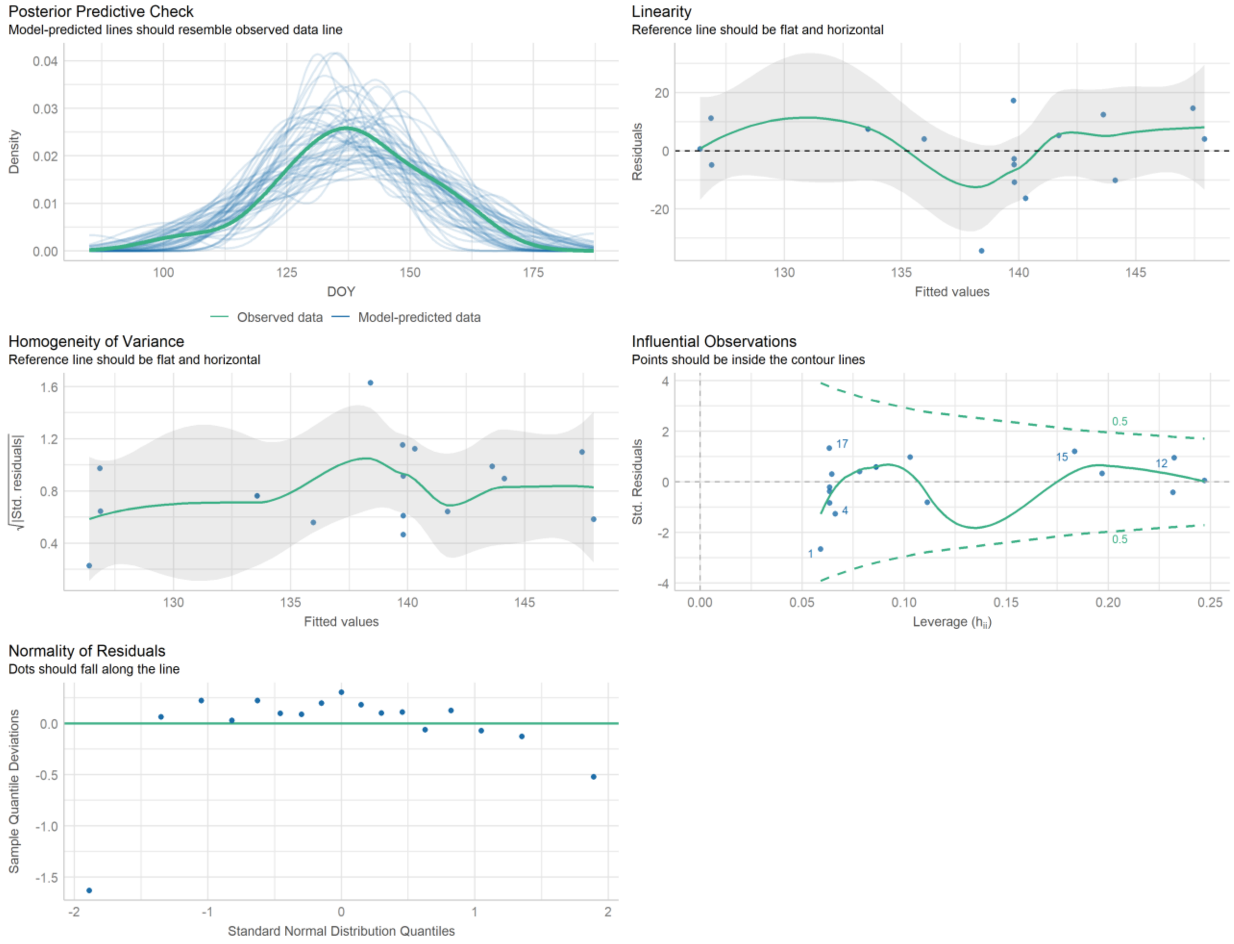

1.26. LM - FS - Cistus populifolius

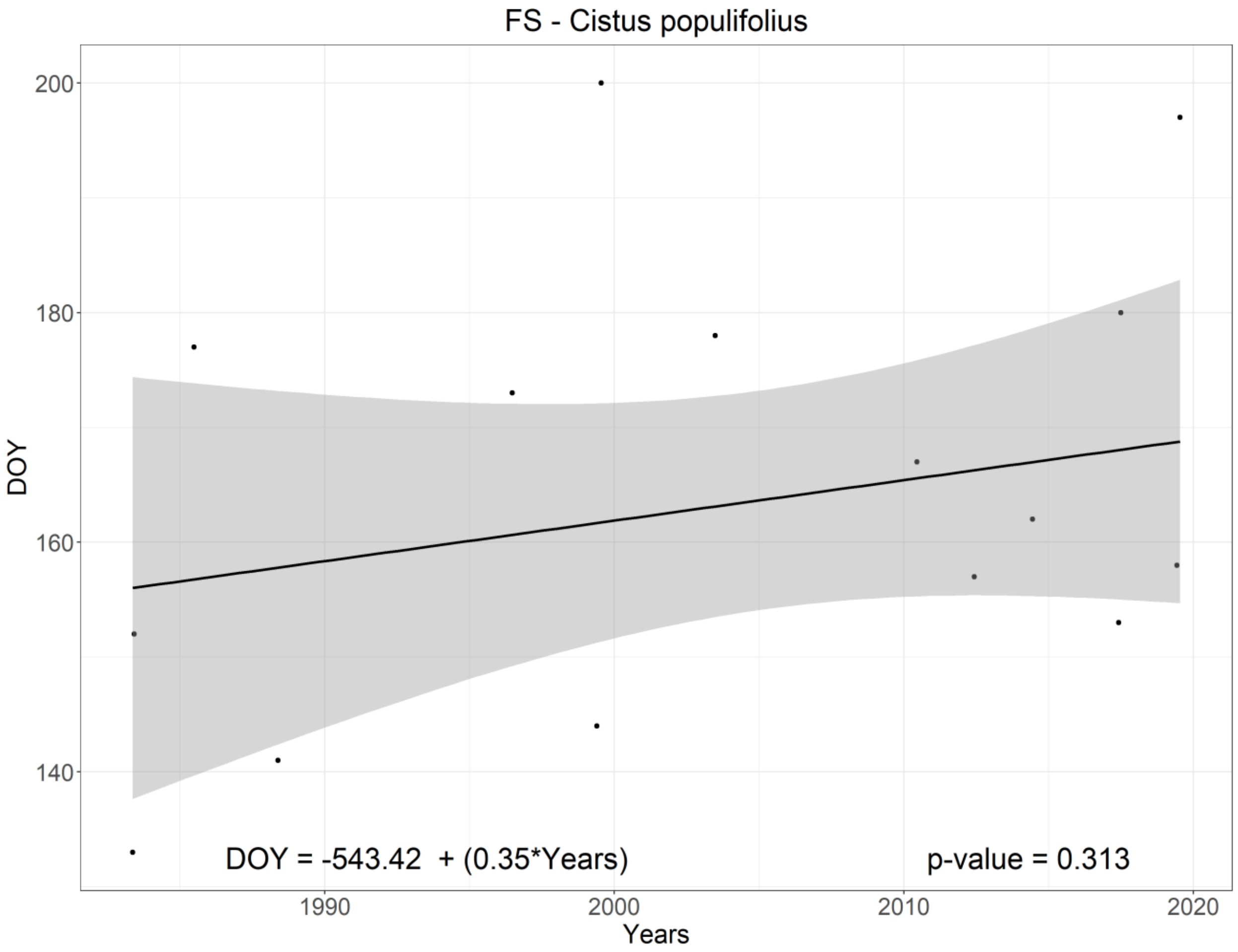

1.26.1. Diagnostics - LM - FS - Cistus populifolius

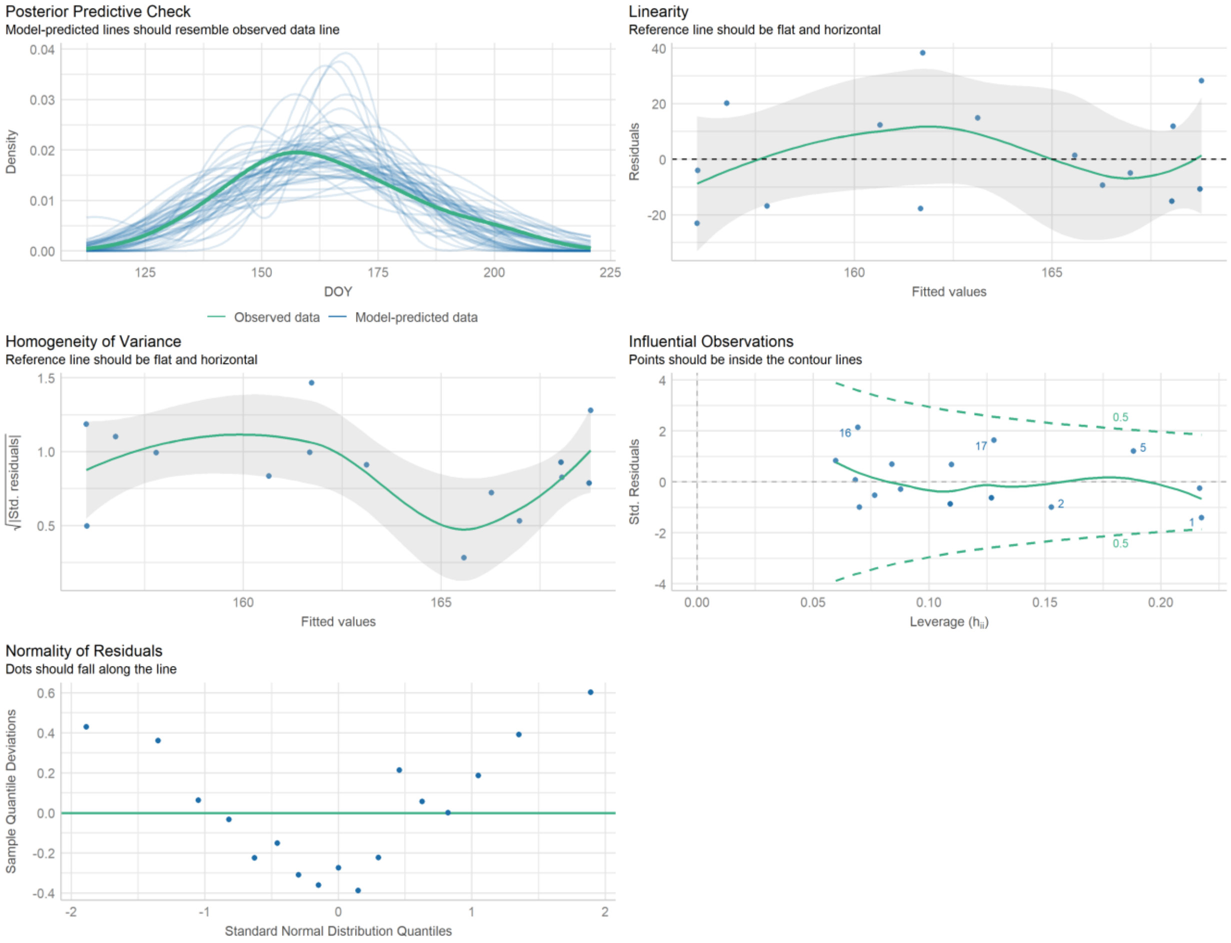

1.27. LM - DVG - Cistus populifolius

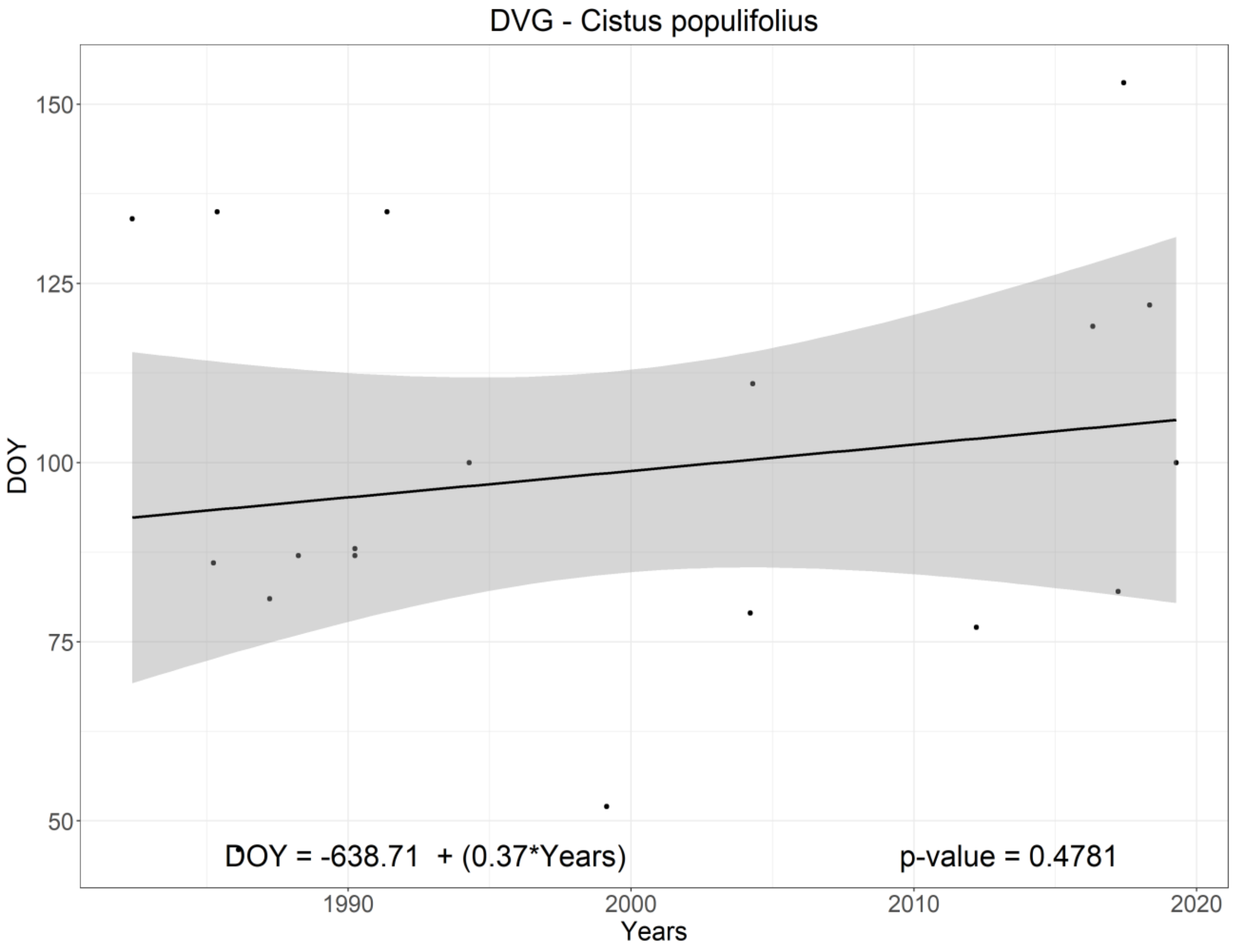

1.27.1. Diagnostics - LM - DVG - Cistus populifolius

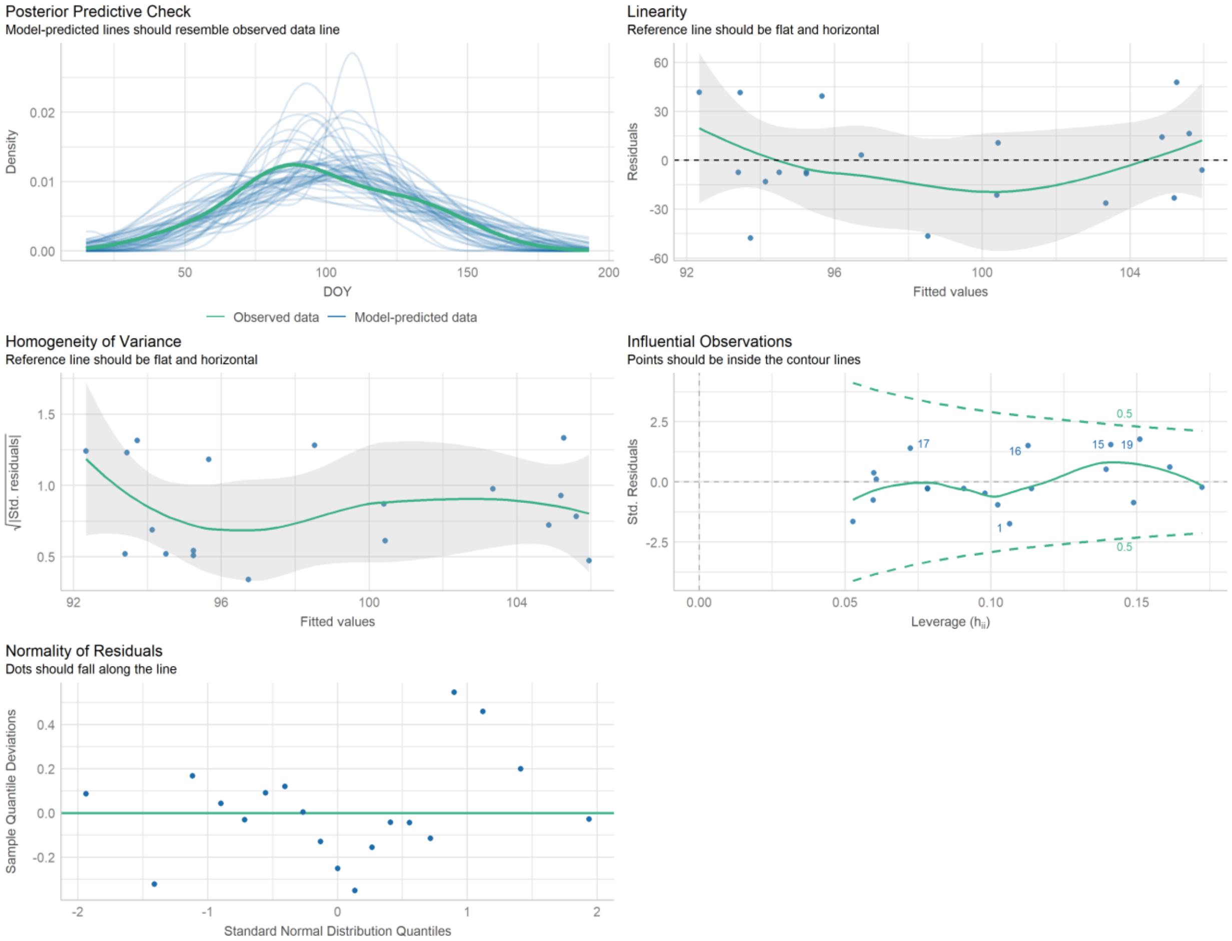

1.28. LM - F - Crataegus granatensis

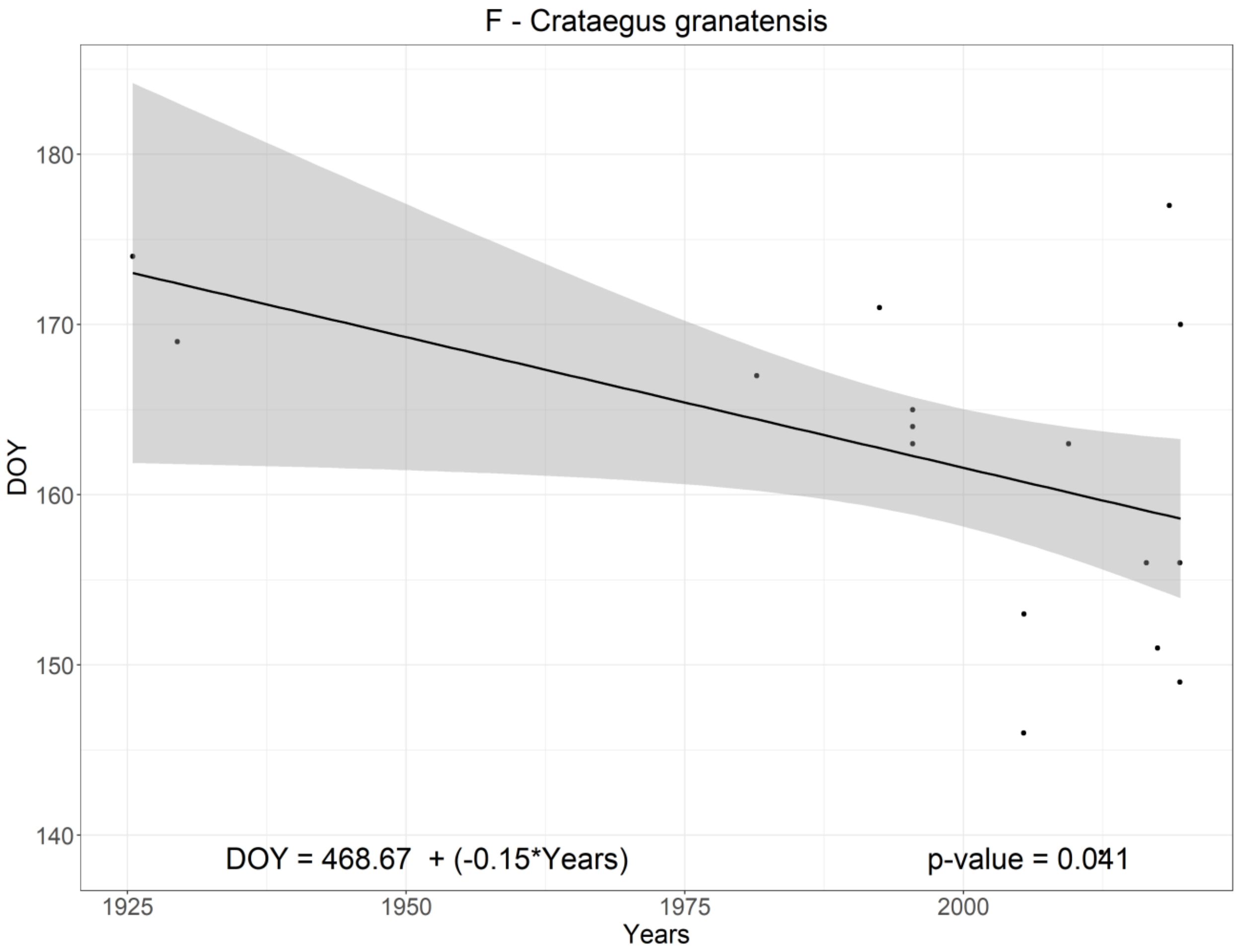

1.28.1. Diagnostics - LM - F - Crataegus granatensis

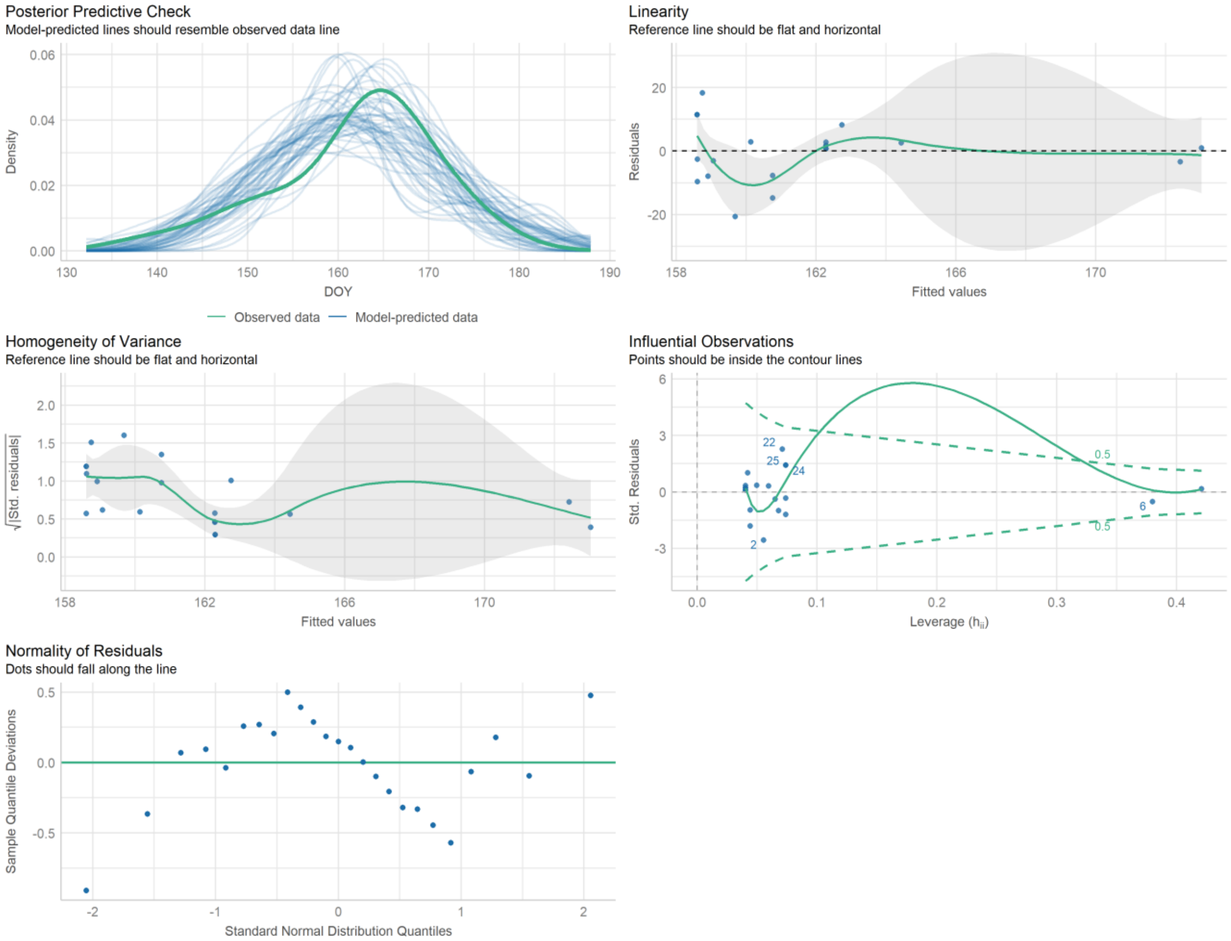

1.29. LM - FBF - Crataegus monogyna

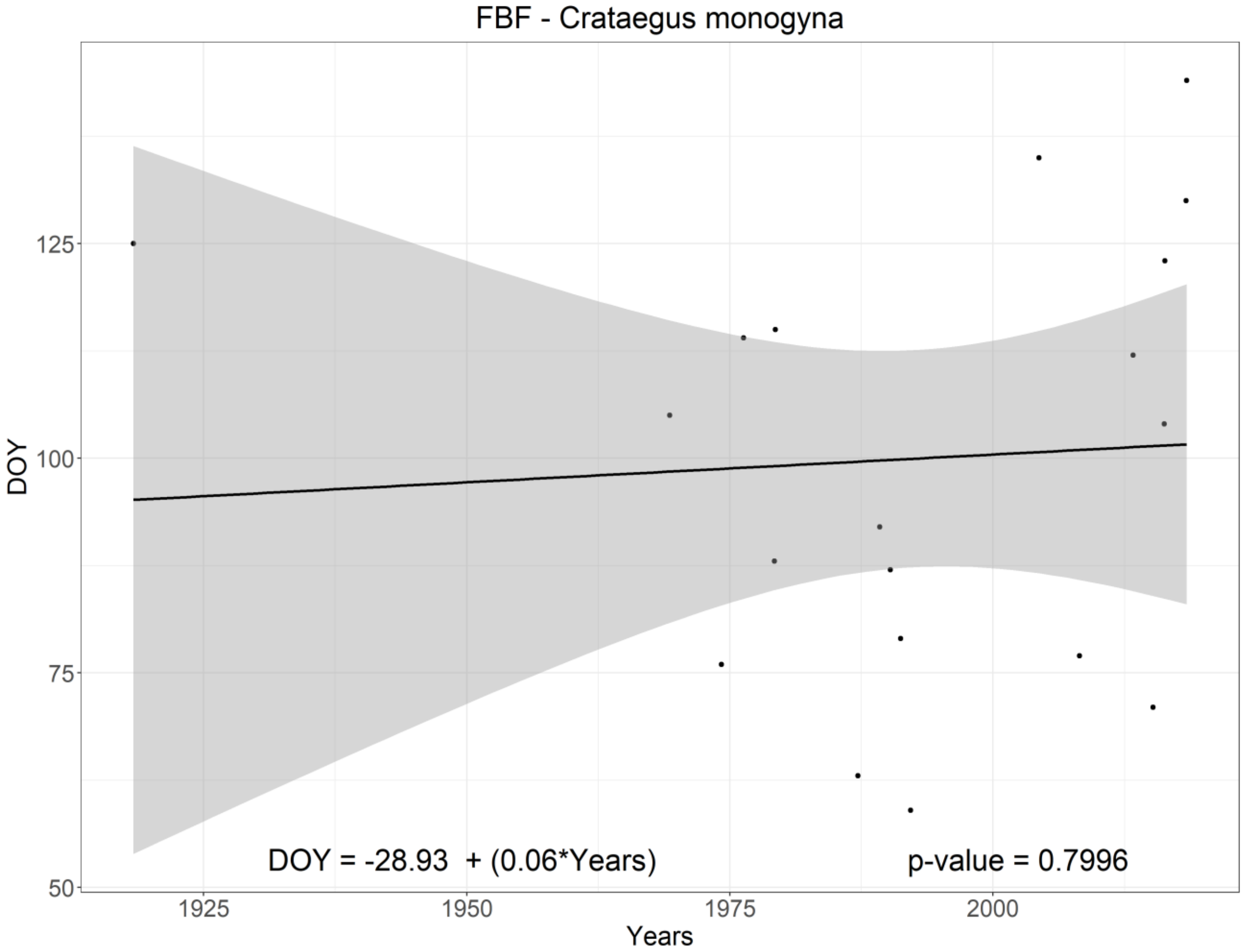

1.29.1. Diagnostics - LM - FBF - Crataegus monogyna

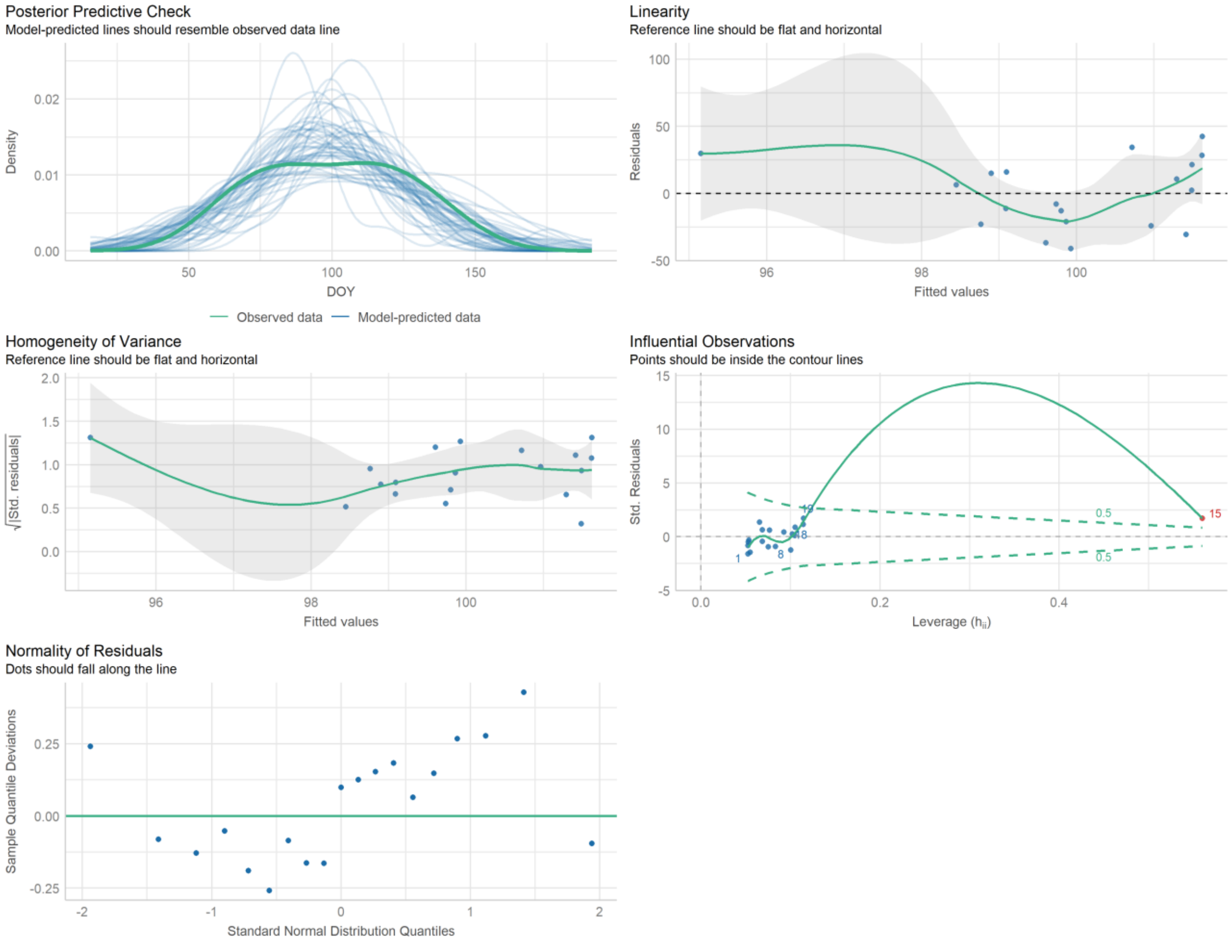

1.30. LM - F - Crataegus monogyna

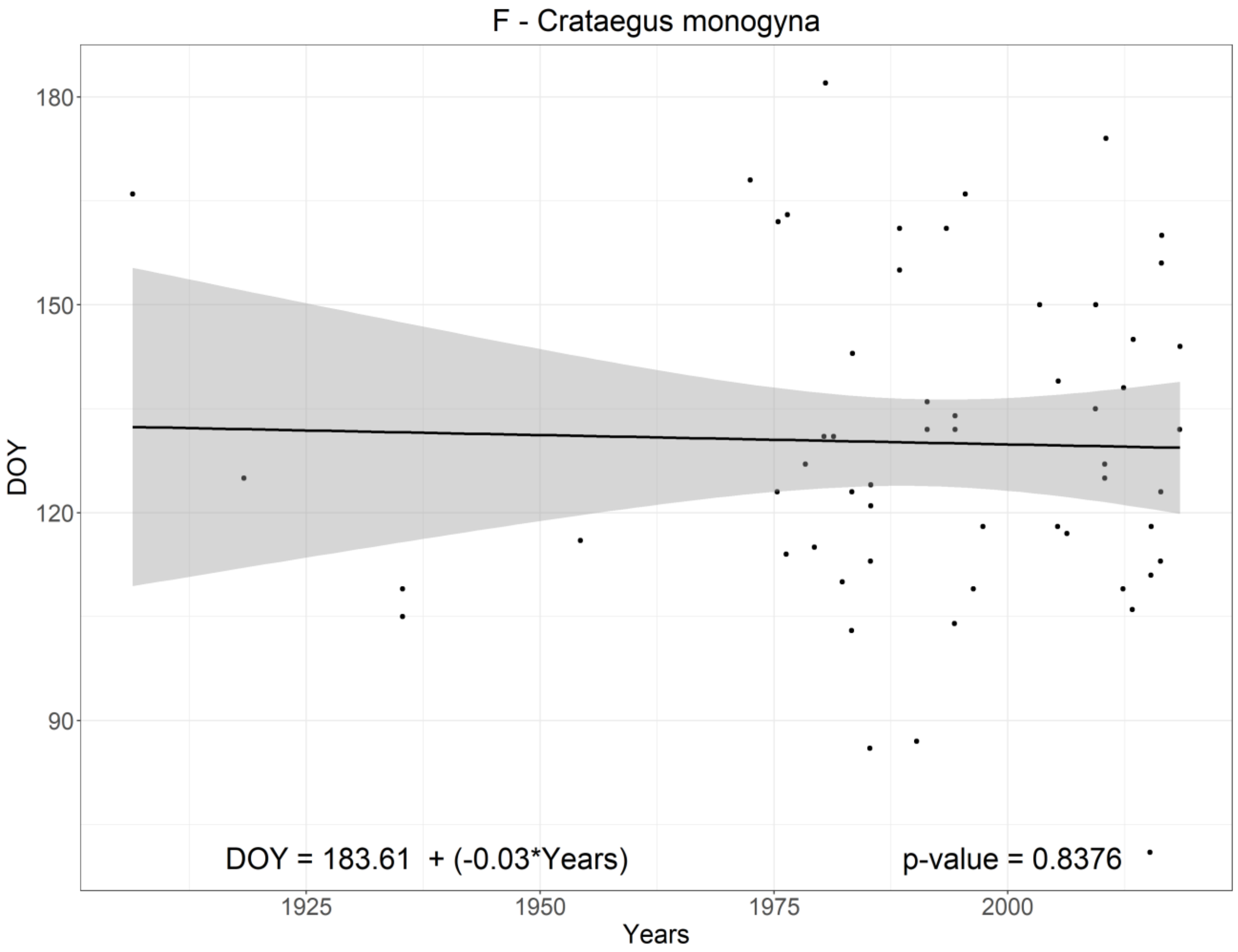

1.30.1. Diagnostics - LM - F - Crataegus monogyna

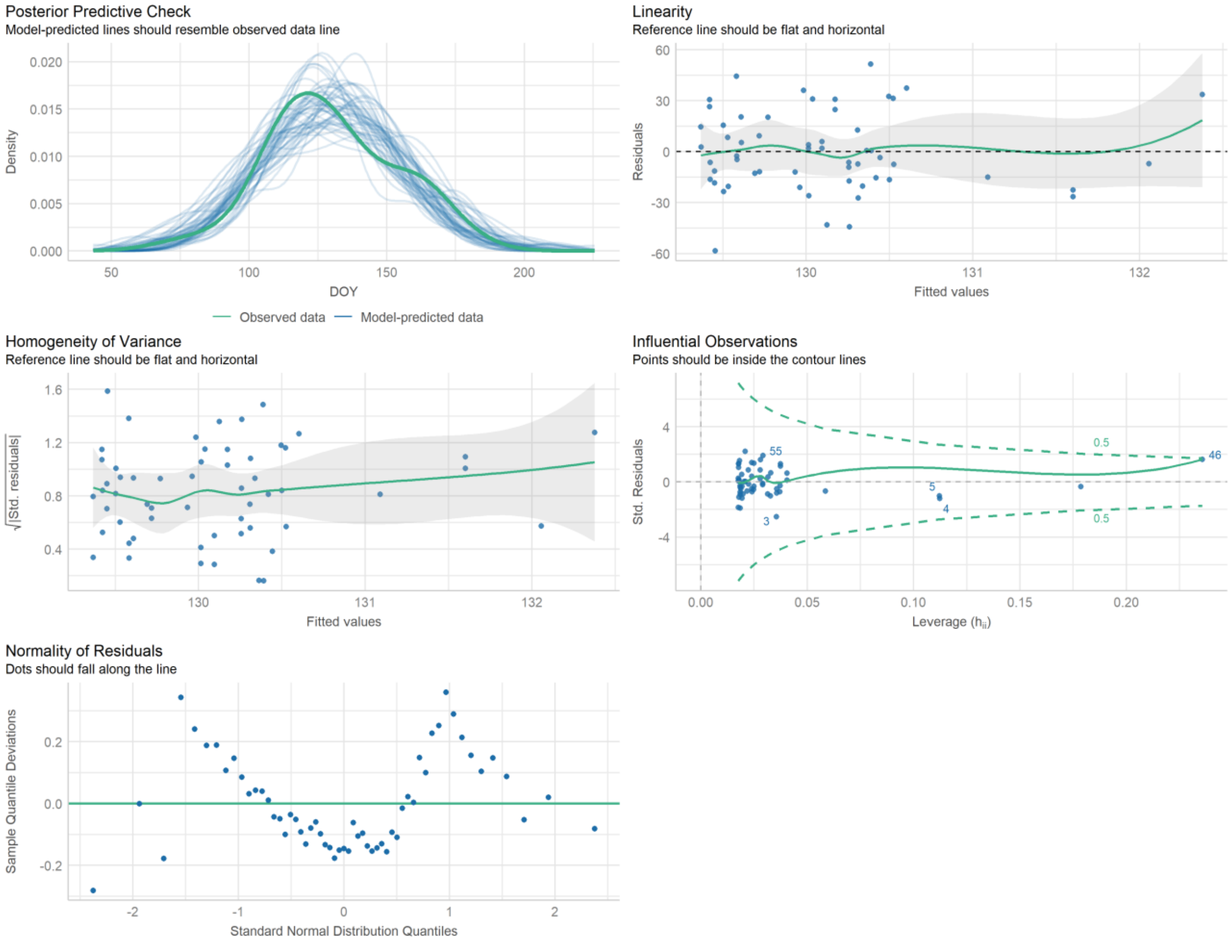

1.31. LM - FS - Crataegus monogyna

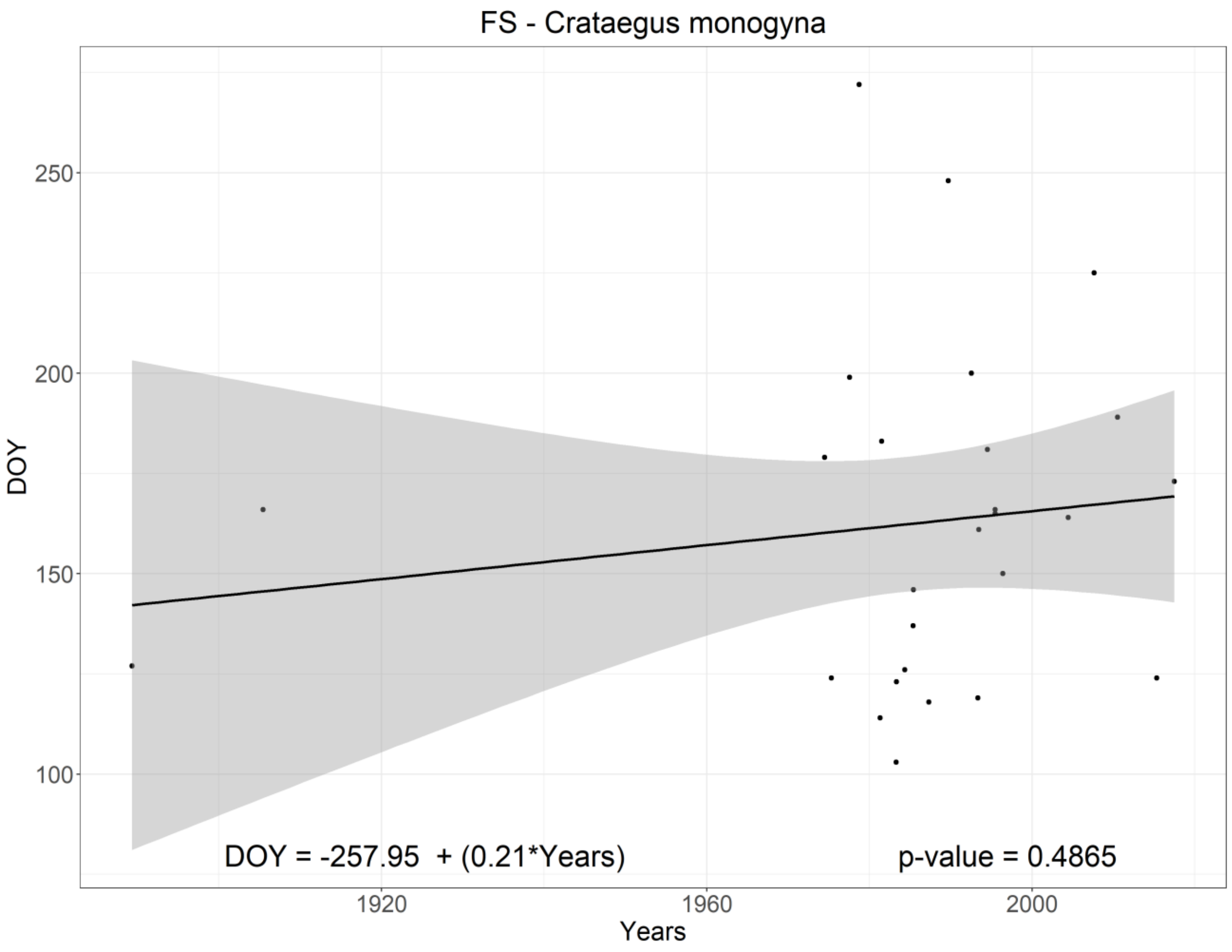

1.31.1. Diagnostics - LM - FS - Crataegus monogyna

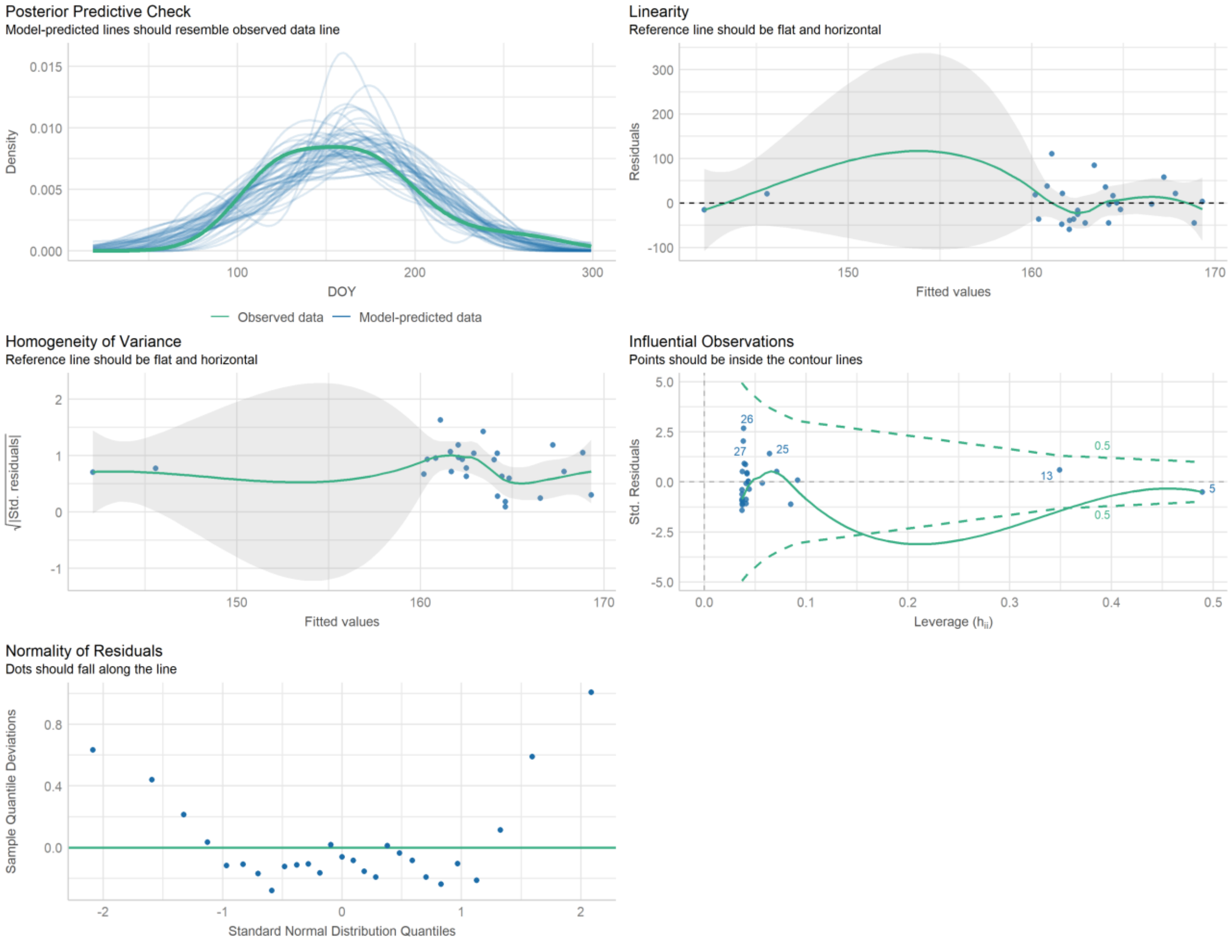

1.32. LM - DVG - Crataegus monogyna

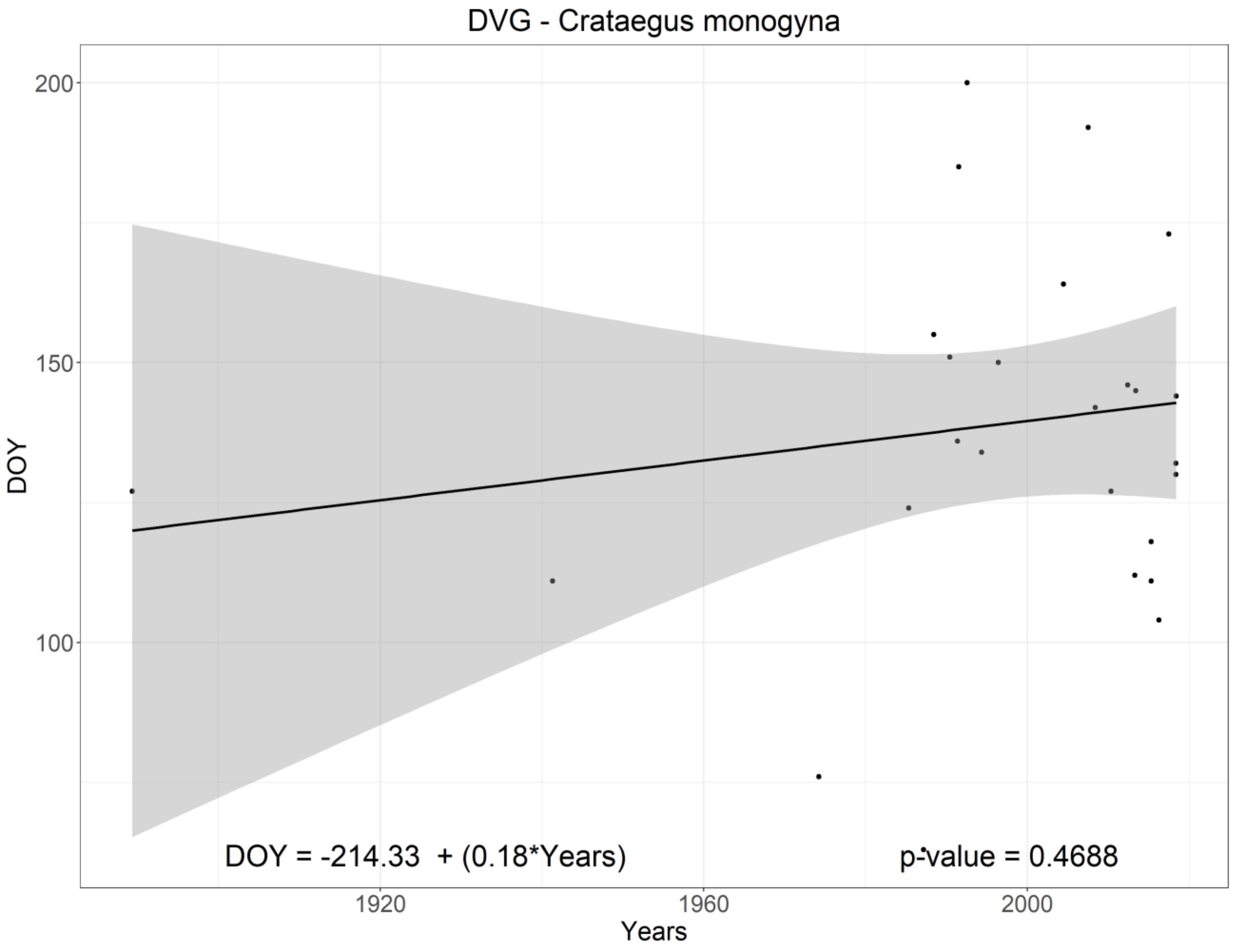

1.32.1. Diagnostics - LM - DVG - Crataegus monogyna

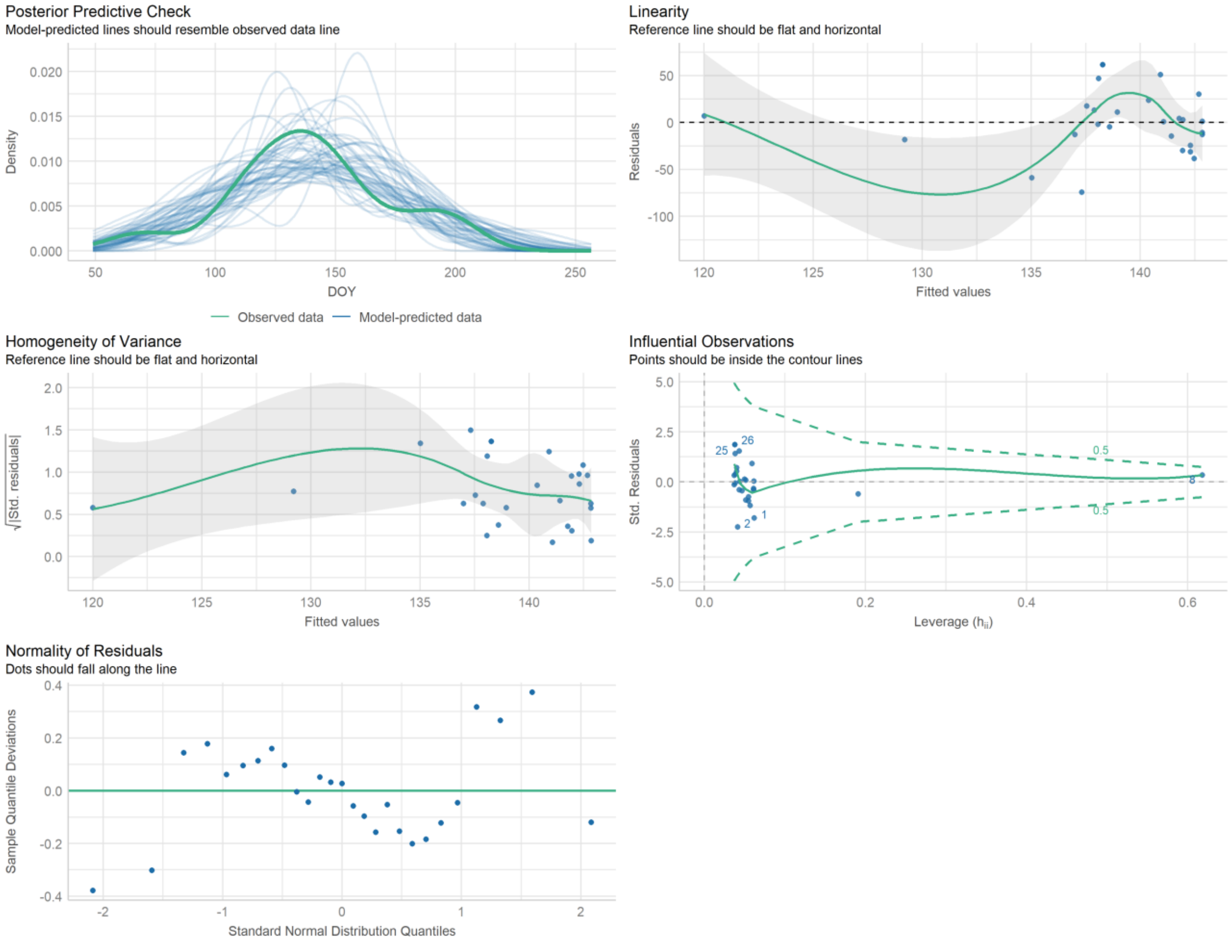

1.33. LM - F - Crepis oporinoides

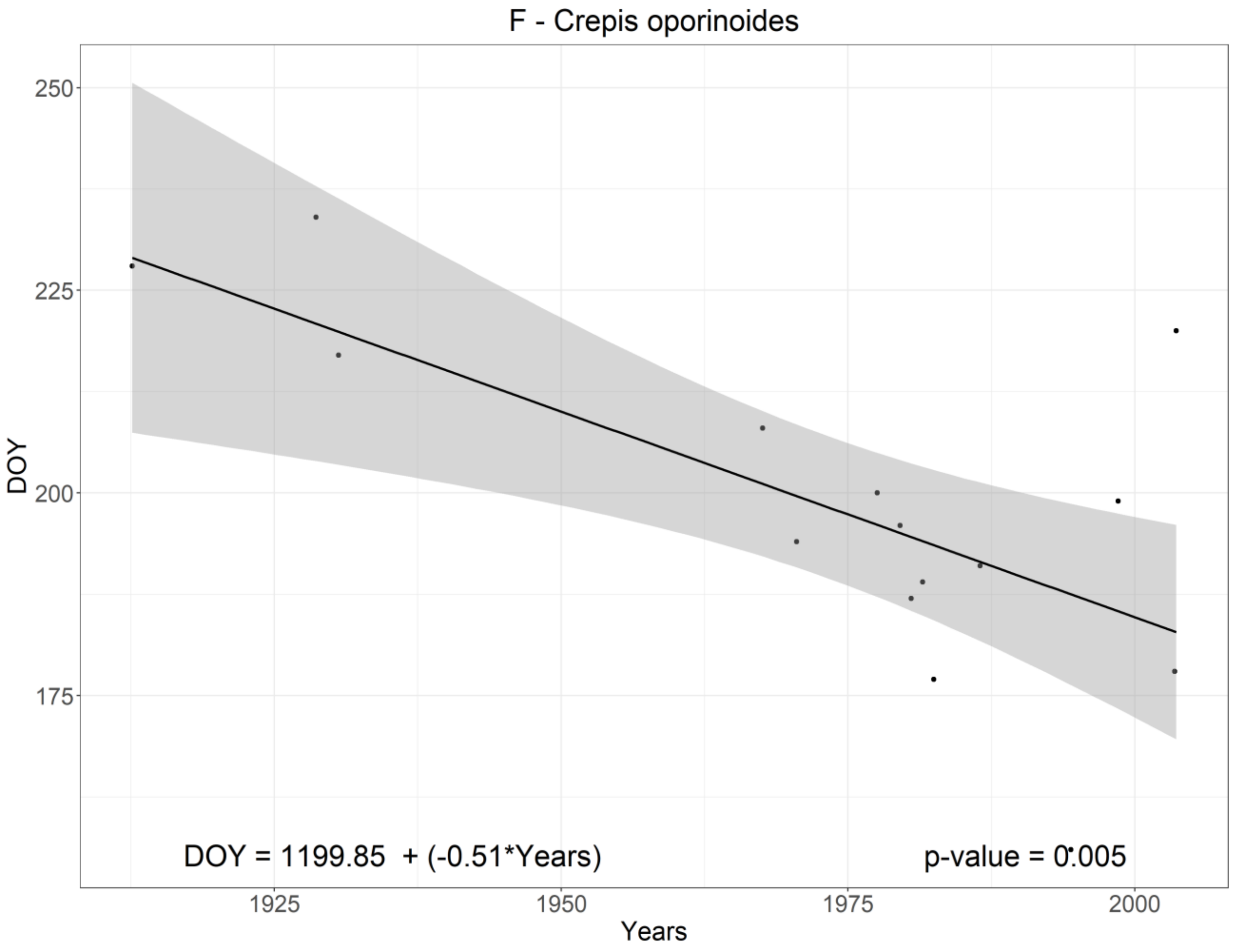

1.33.1. Diagnostics - LM - F - Crepis oporinoides

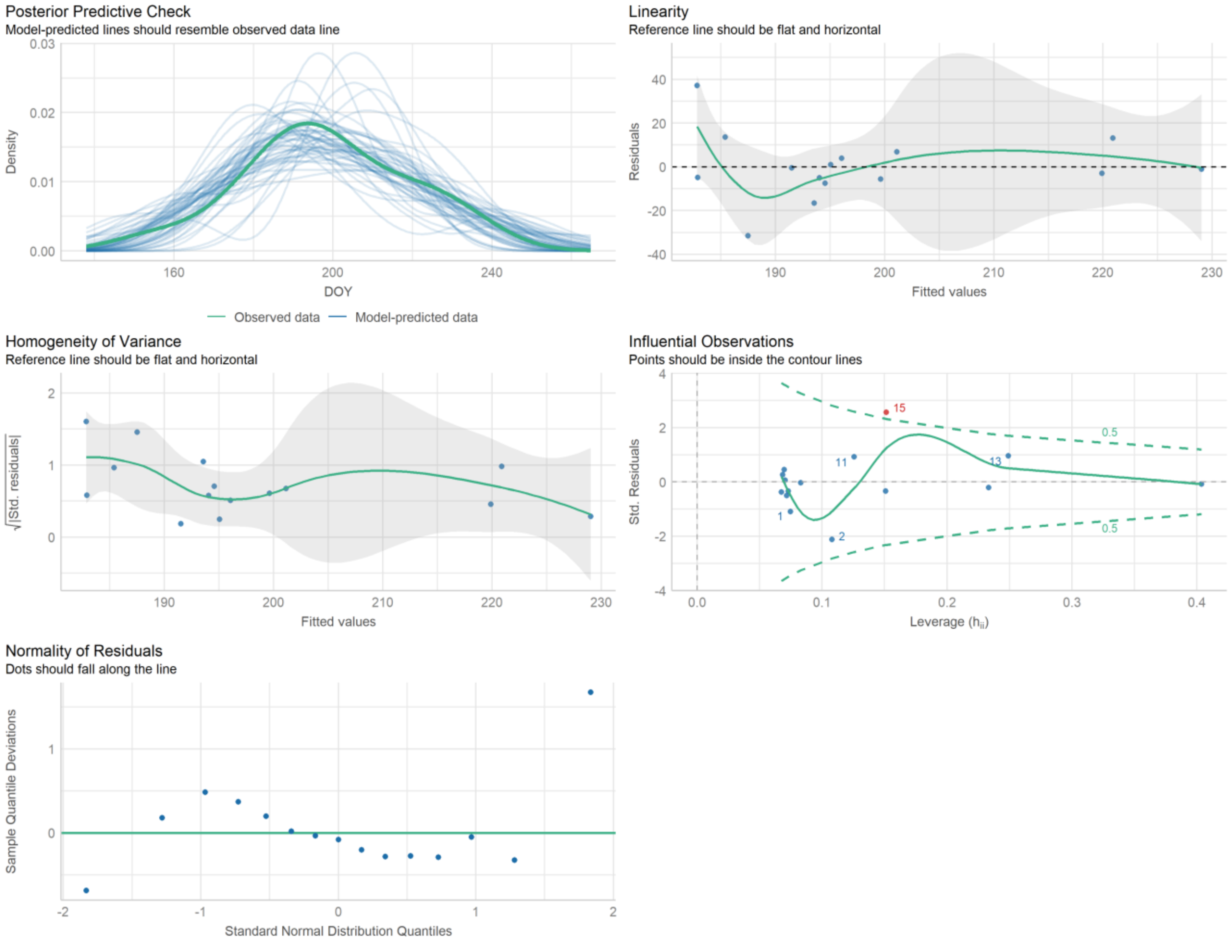

1.34. LM - FS - Crepis oporinoides

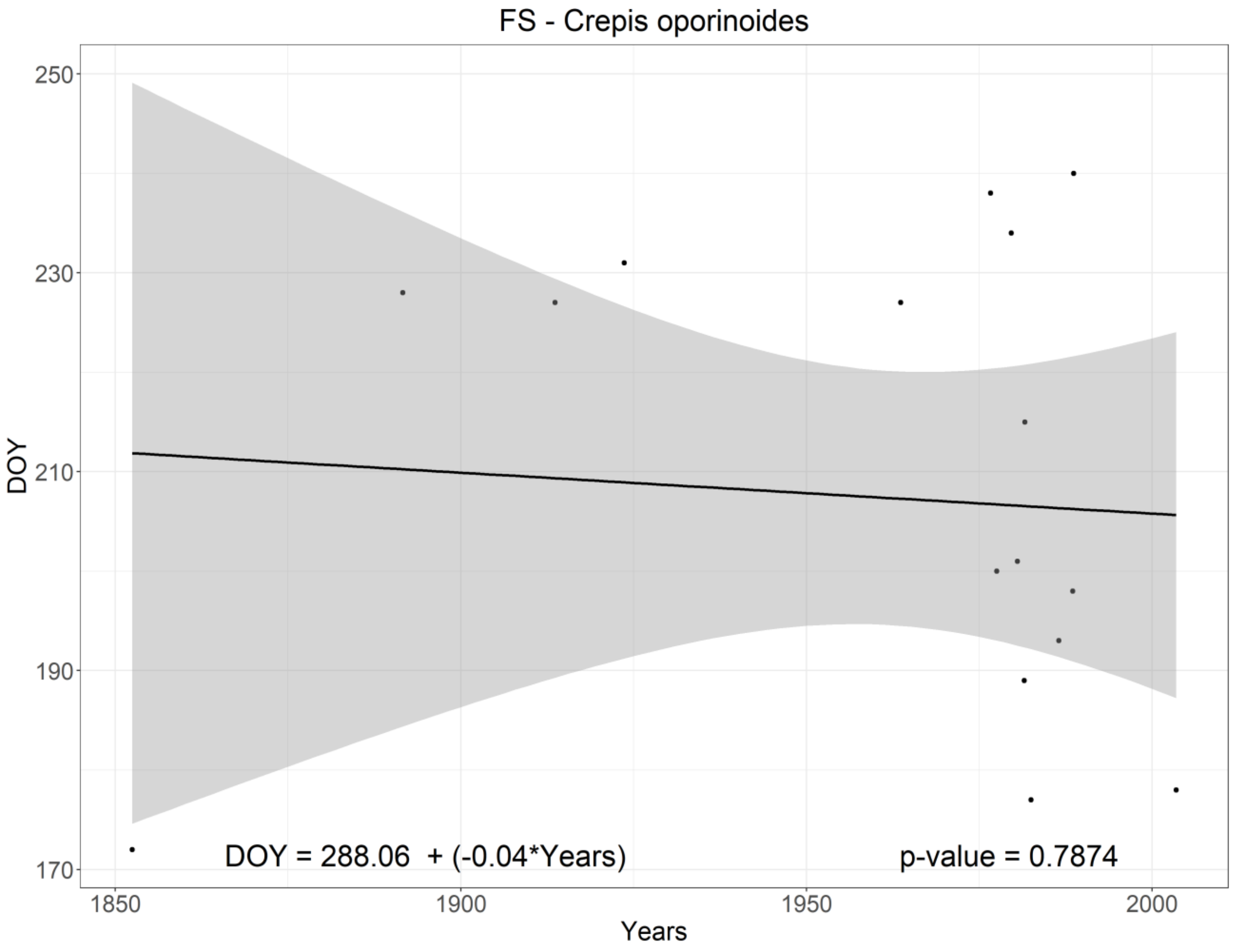

1.34.1. Diagnostics - LM - FS - Crepis oporinoides

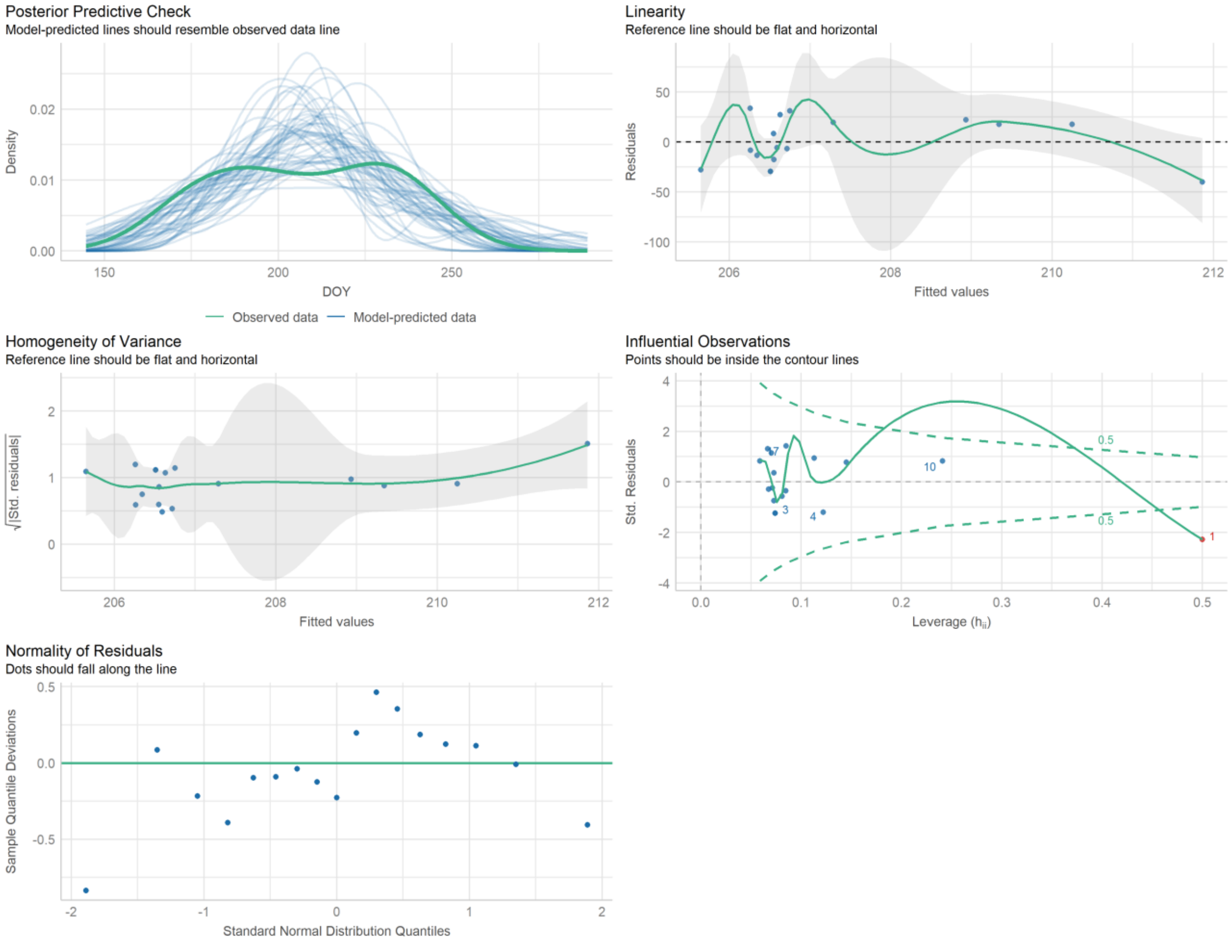

1.35. LM - F - Cytisus malacitanus

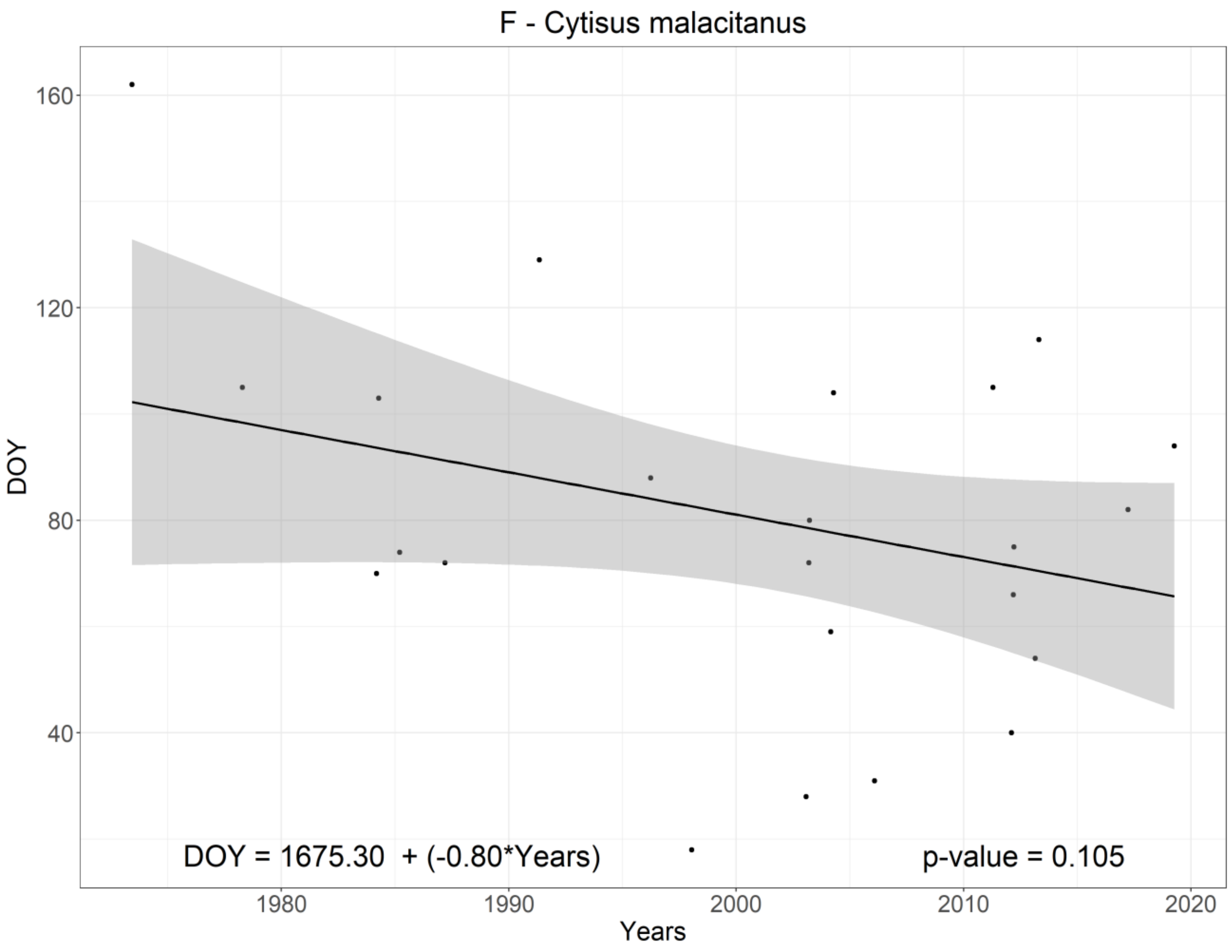

1.35.1. Diagnostics - LM - F - Cytisus malacitanus

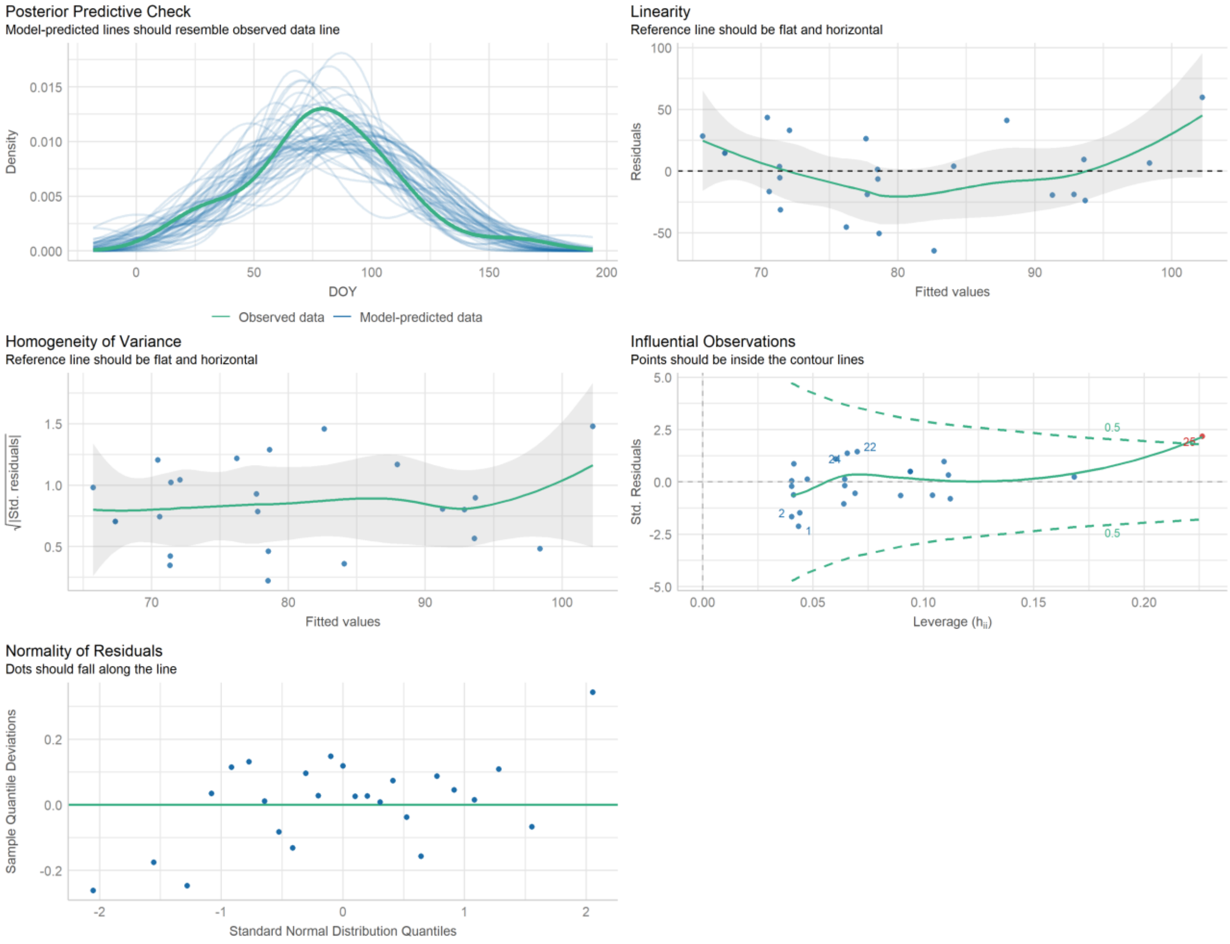

1.36. LM - FS - Cytisus malacitanus

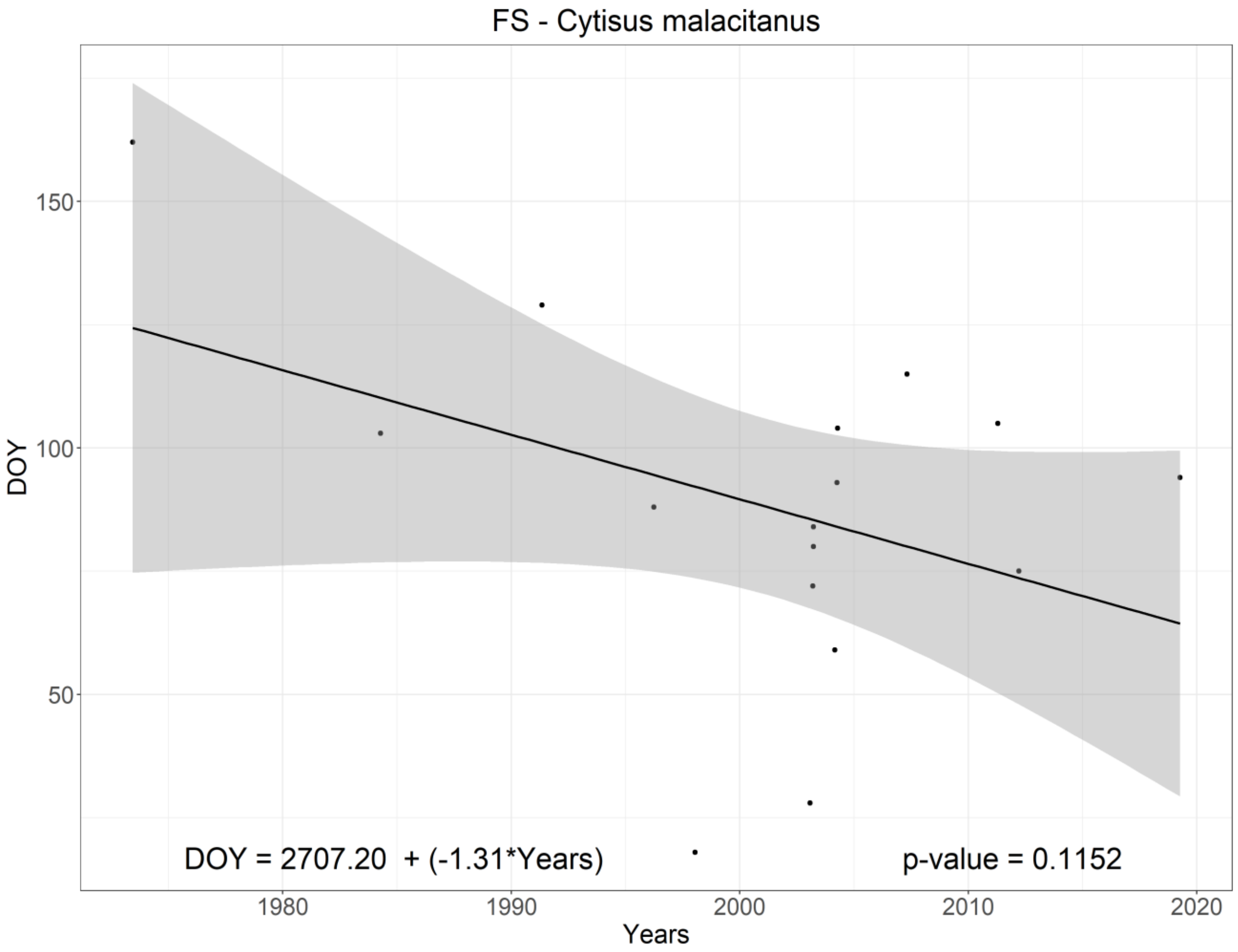

1.36.1. Diagnostics - LM - FS - Cytisus malacitanus

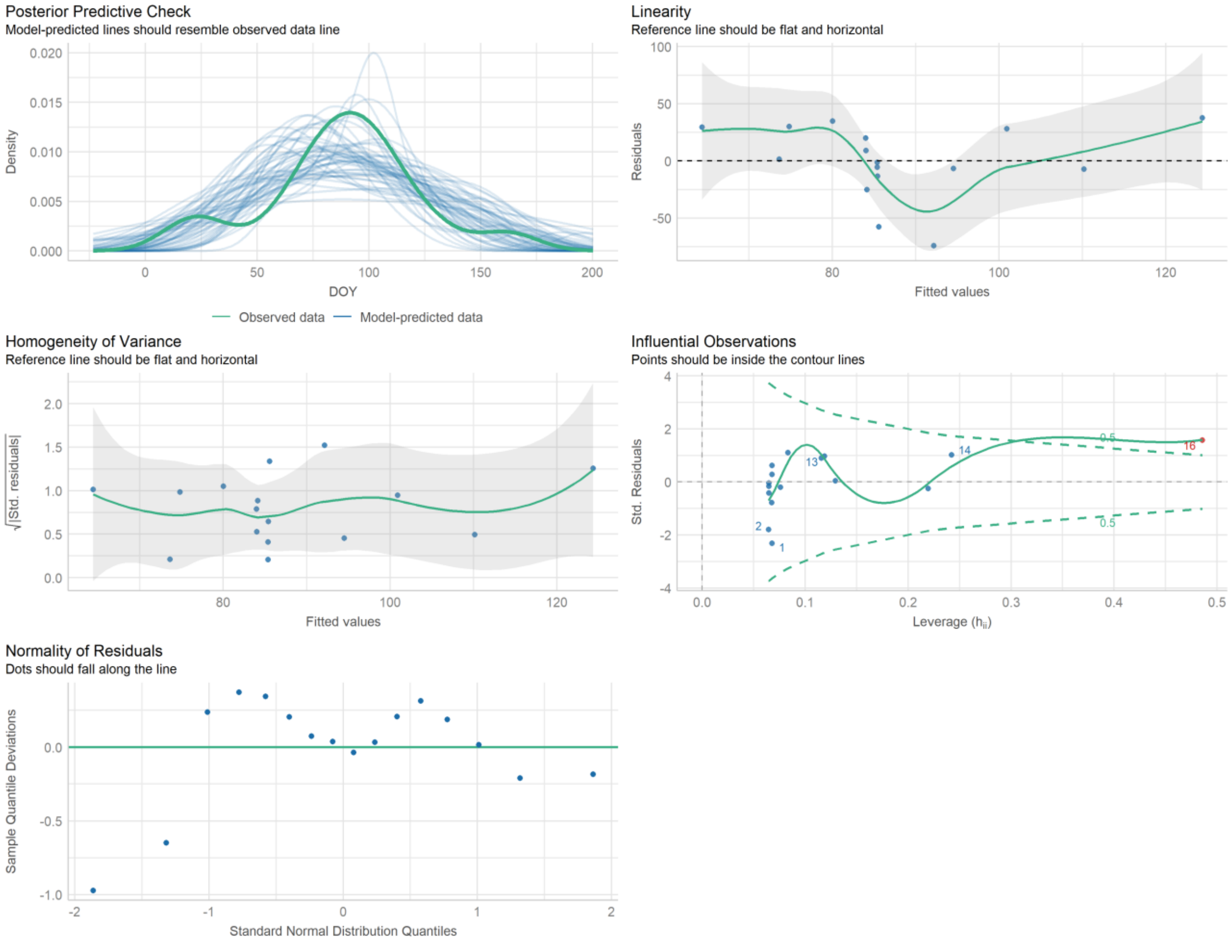

1.37. LM - F - Echium albicans

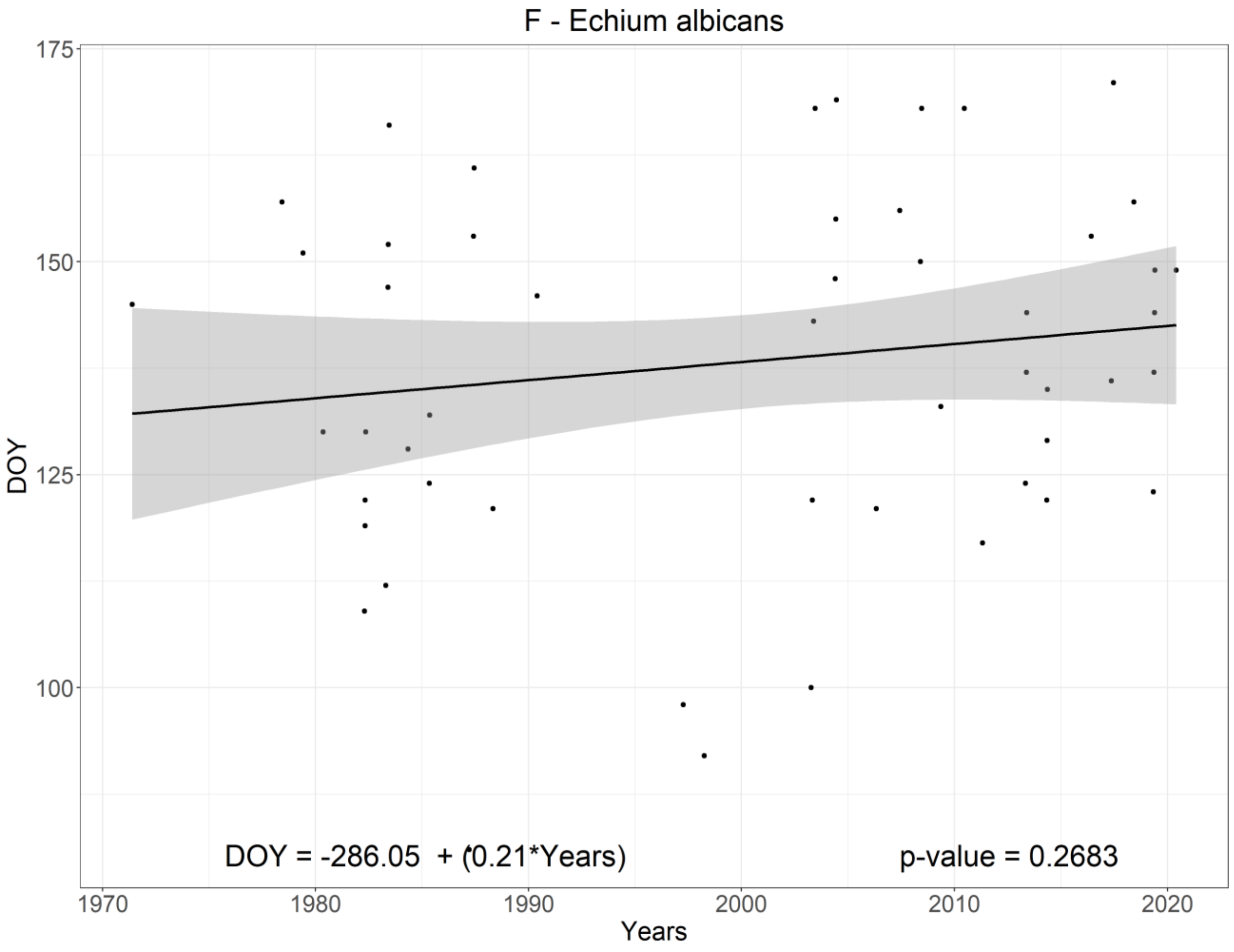

1.37.1. Diagnostics - LM - F - Echium albicans

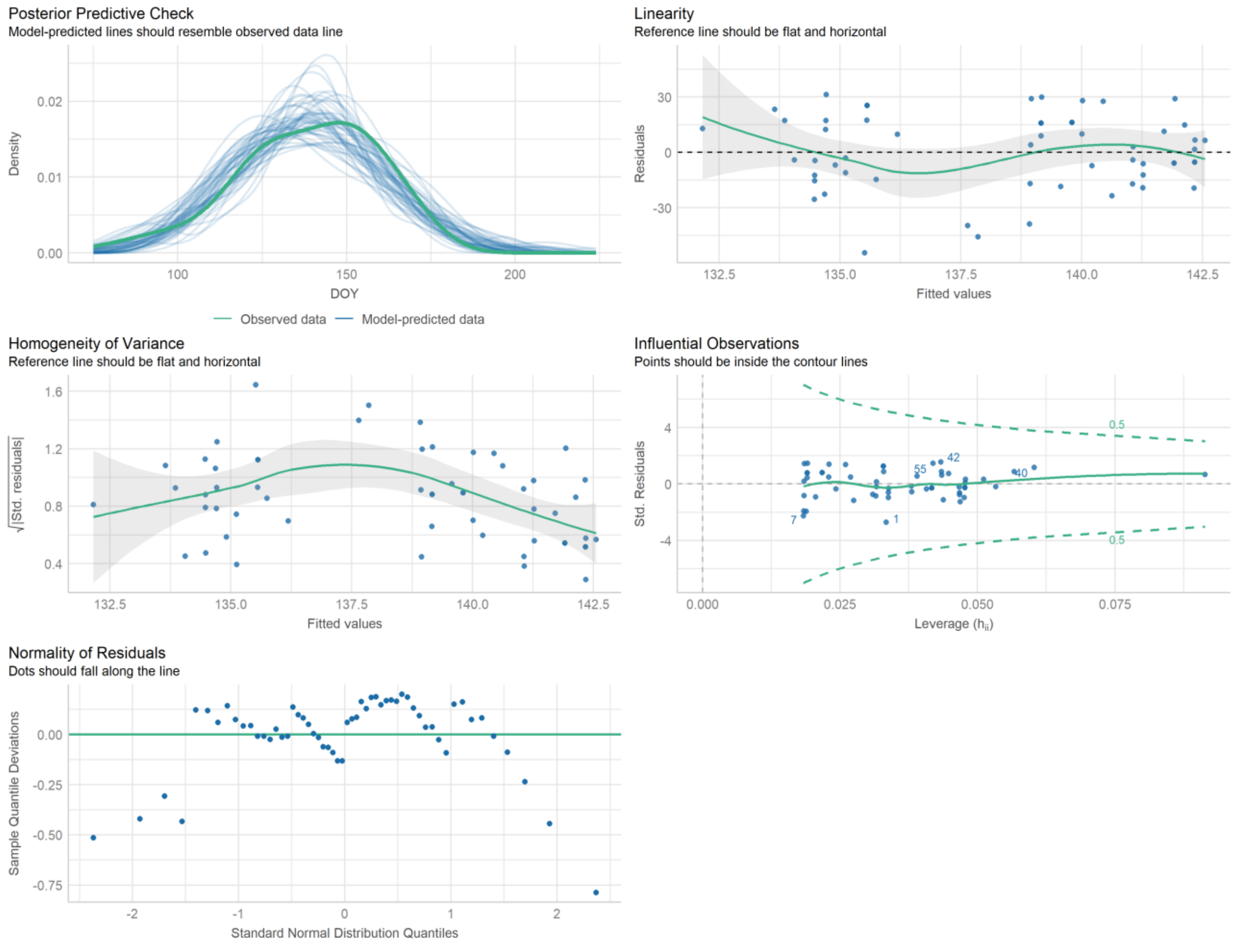

1.38. LM - F - Elaeoselinum asclepium subsp. millefolium

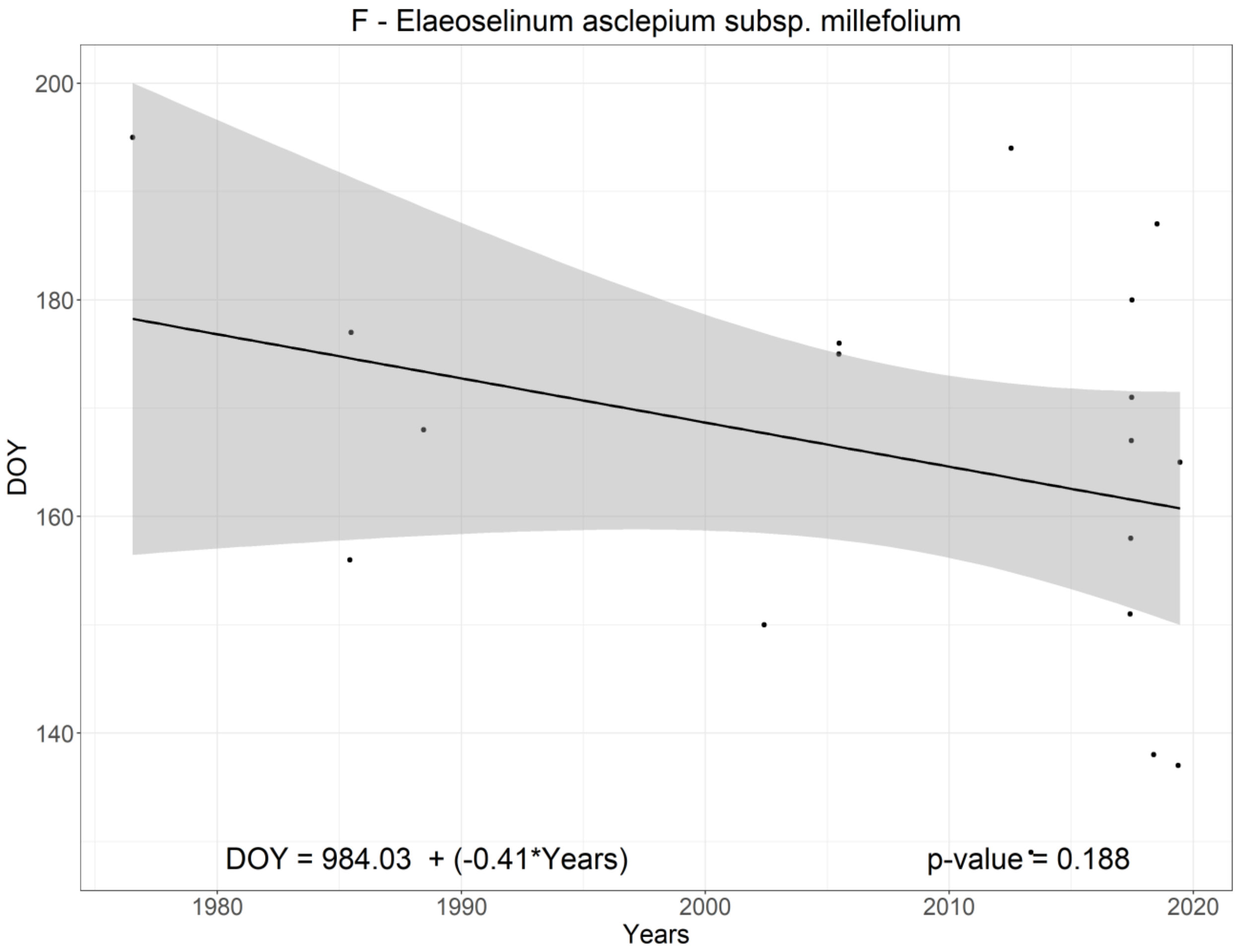

1.38.1. Diagnostics - LM - F - Elaeoselinum asclepium subsp. millefolium

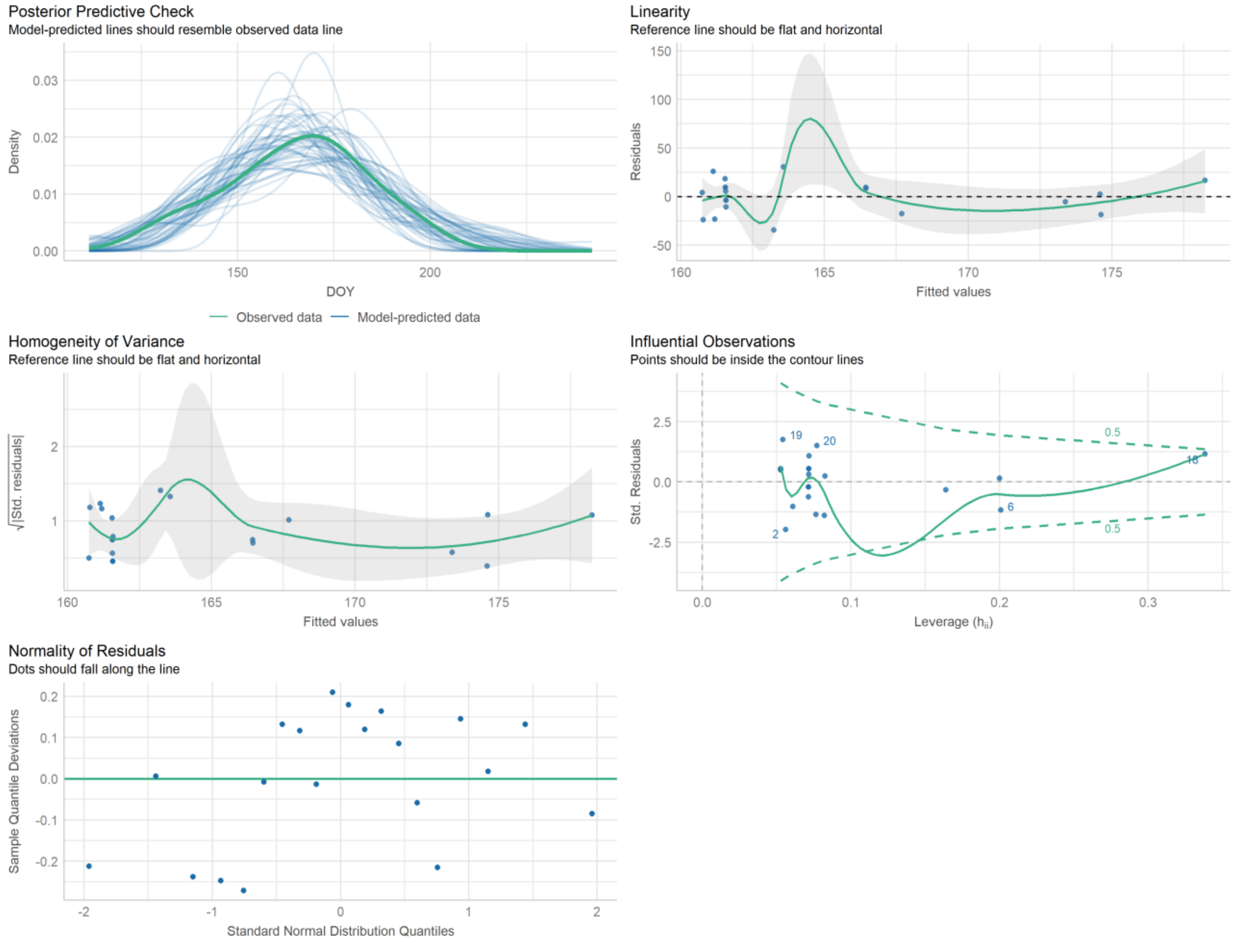

1.39. LM - FBF - Erica arborea

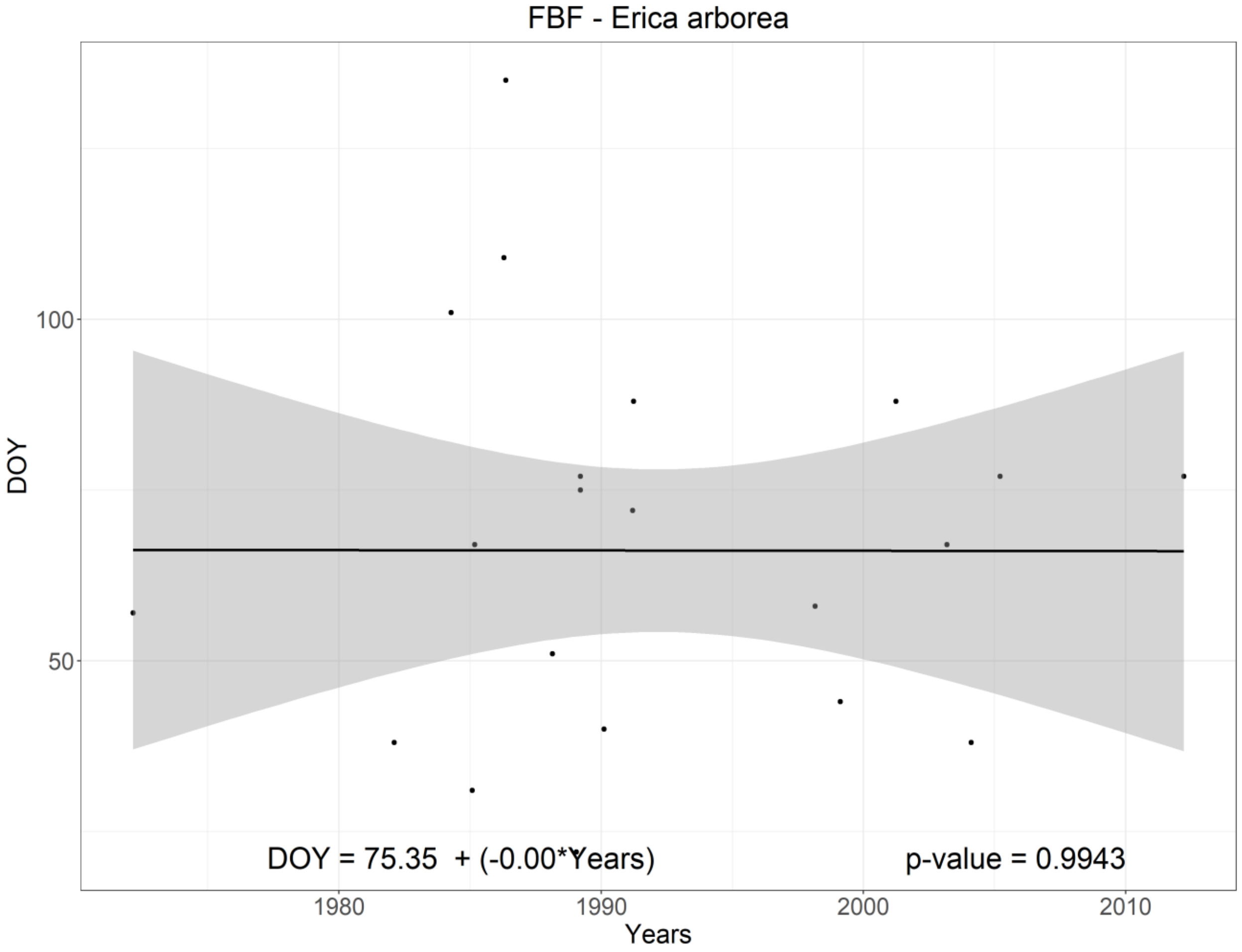

1.39.1. Diagnostics - LM - FBF - Erica arborea

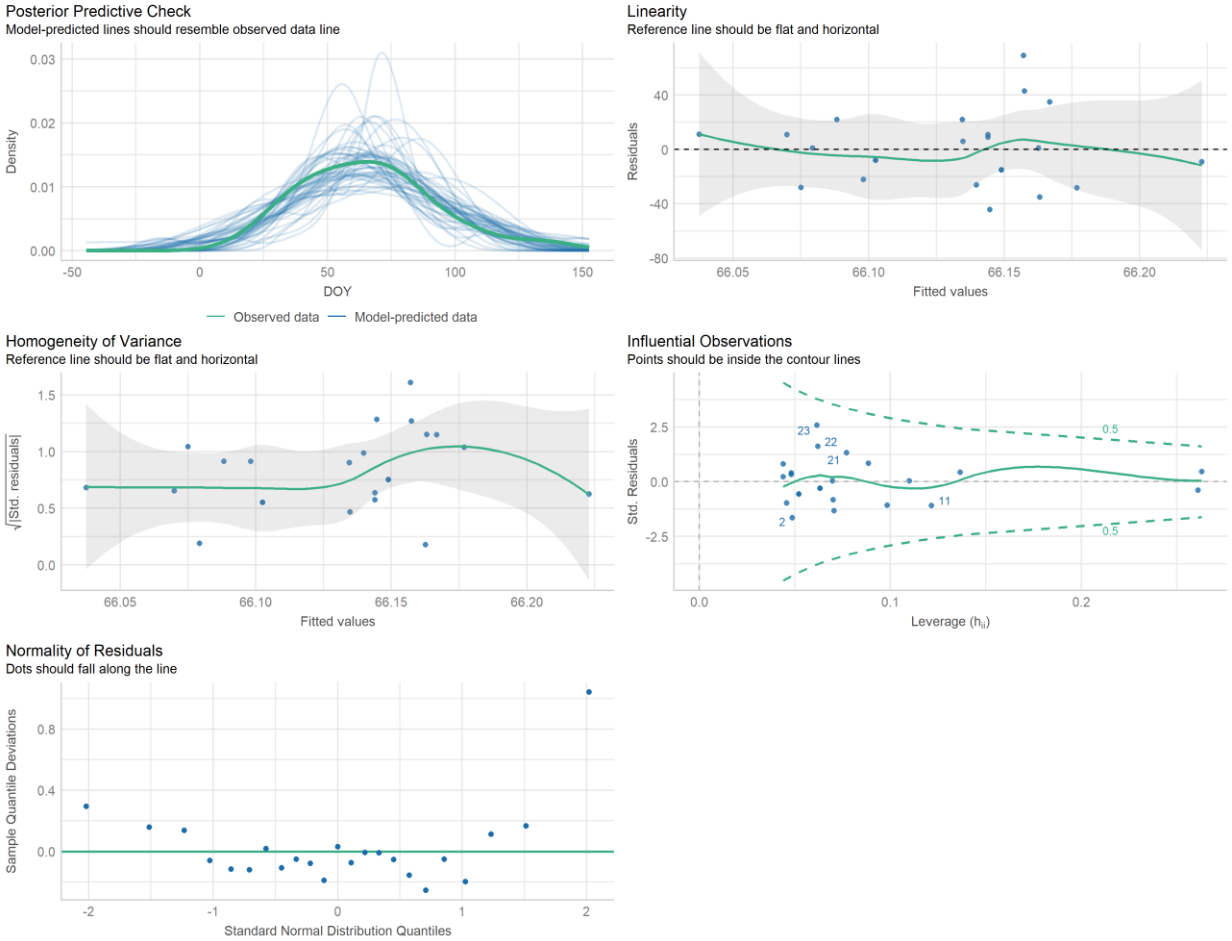

1.40. LM - F - Erica arborea

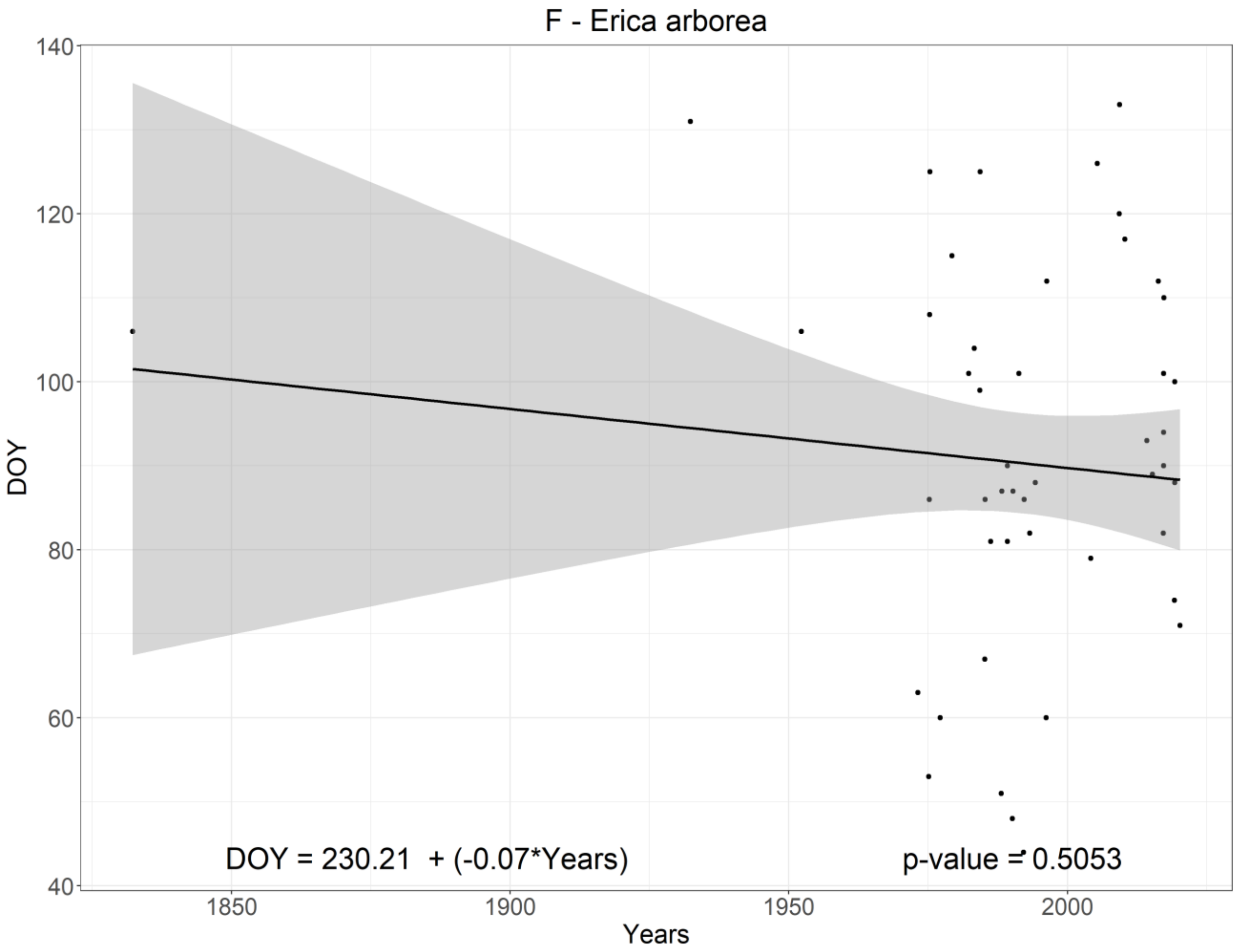

1.40.1. Diagnostics - LM - F - Erica arborea

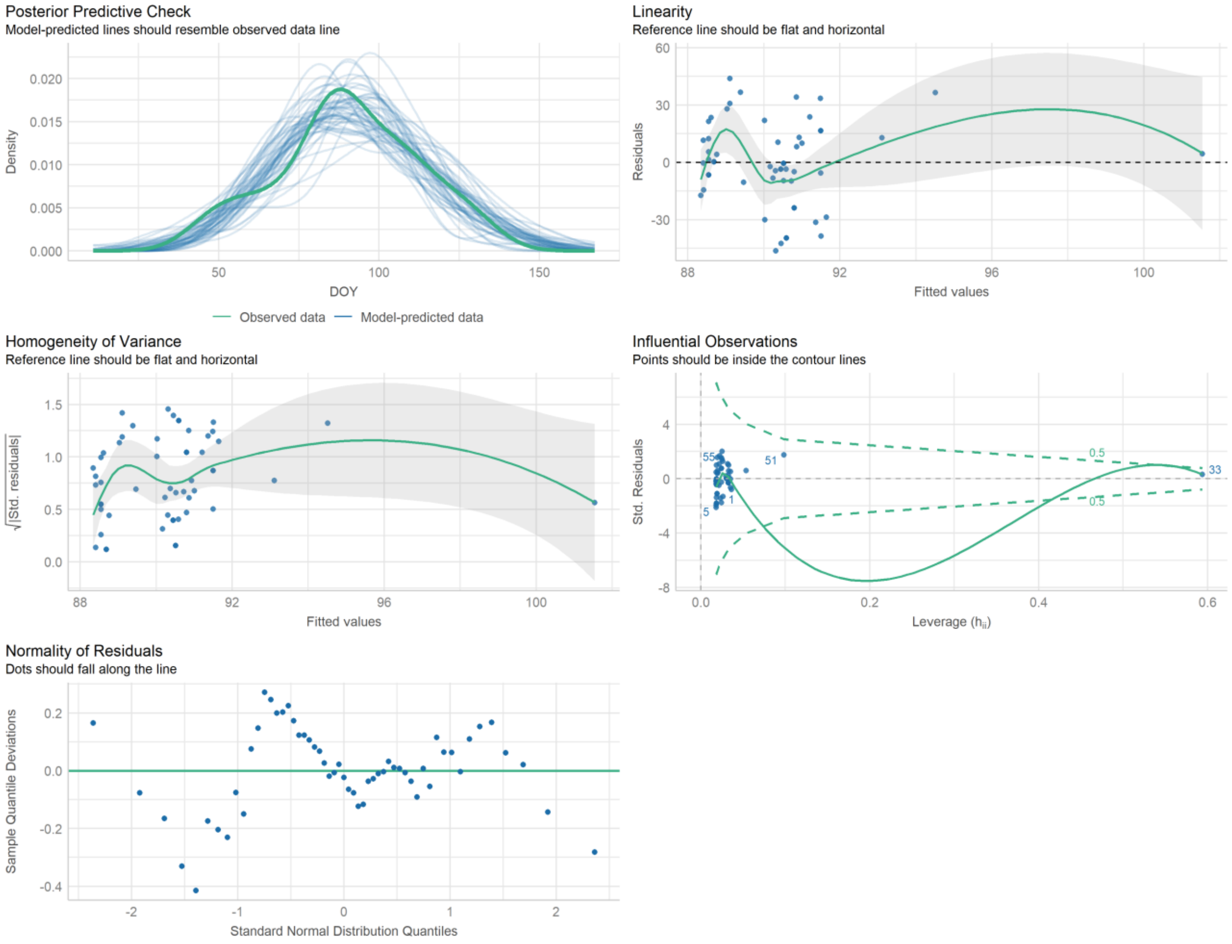

1.41. LM - FBF - Erica scoparia

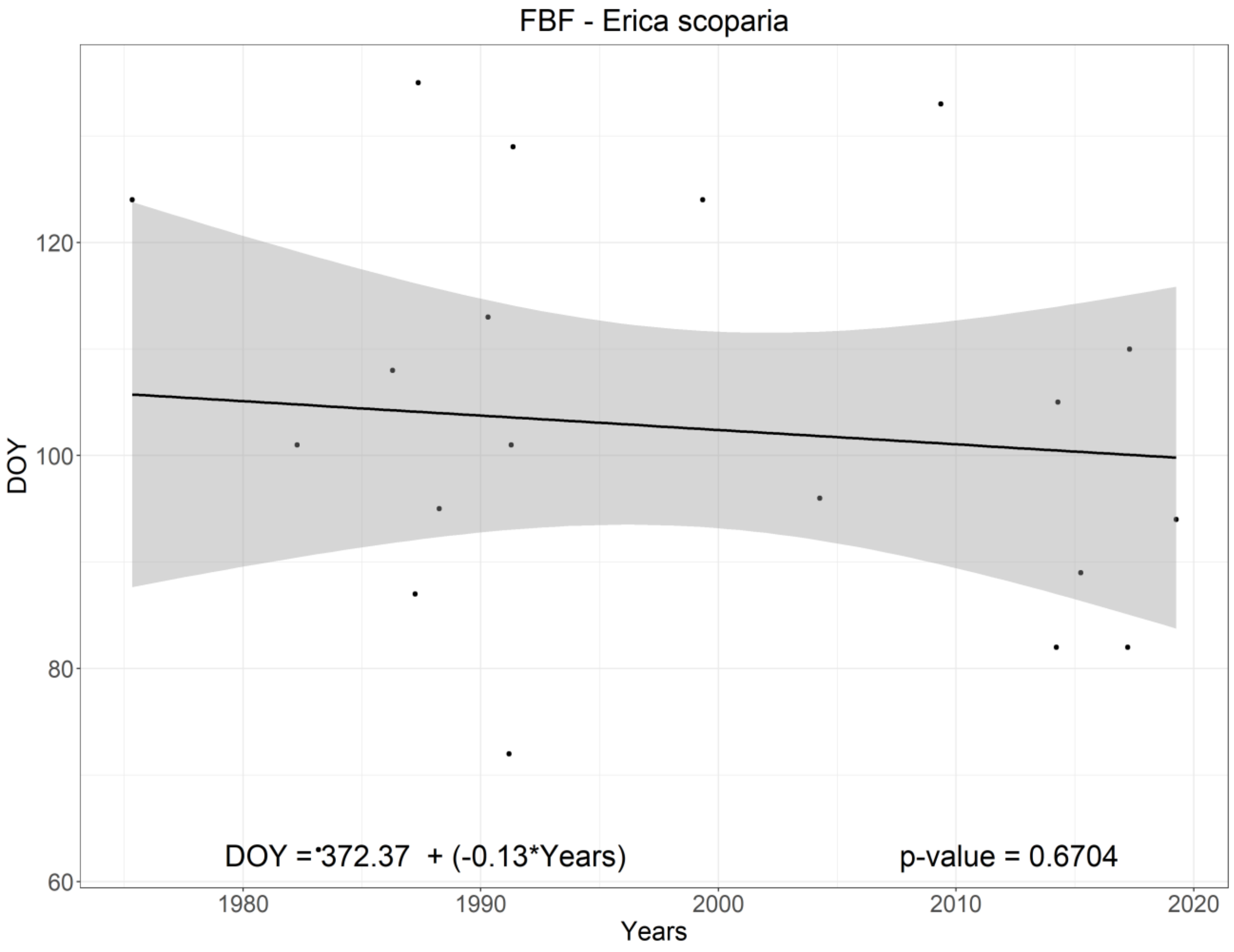

1.41.1. Diagnostics - LM - FBF - Erica scoparia

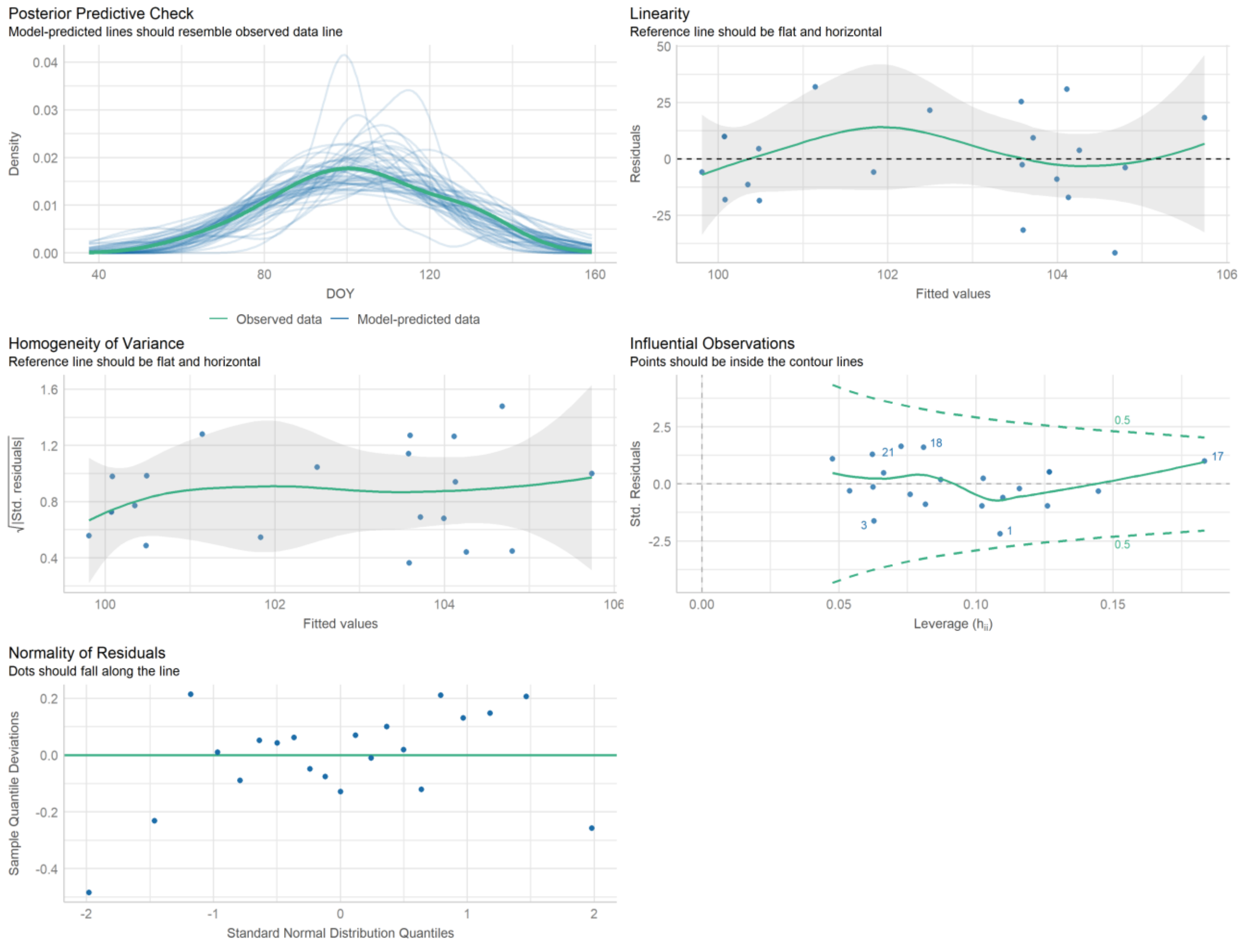

1.42. LM - F - Erica scoparia

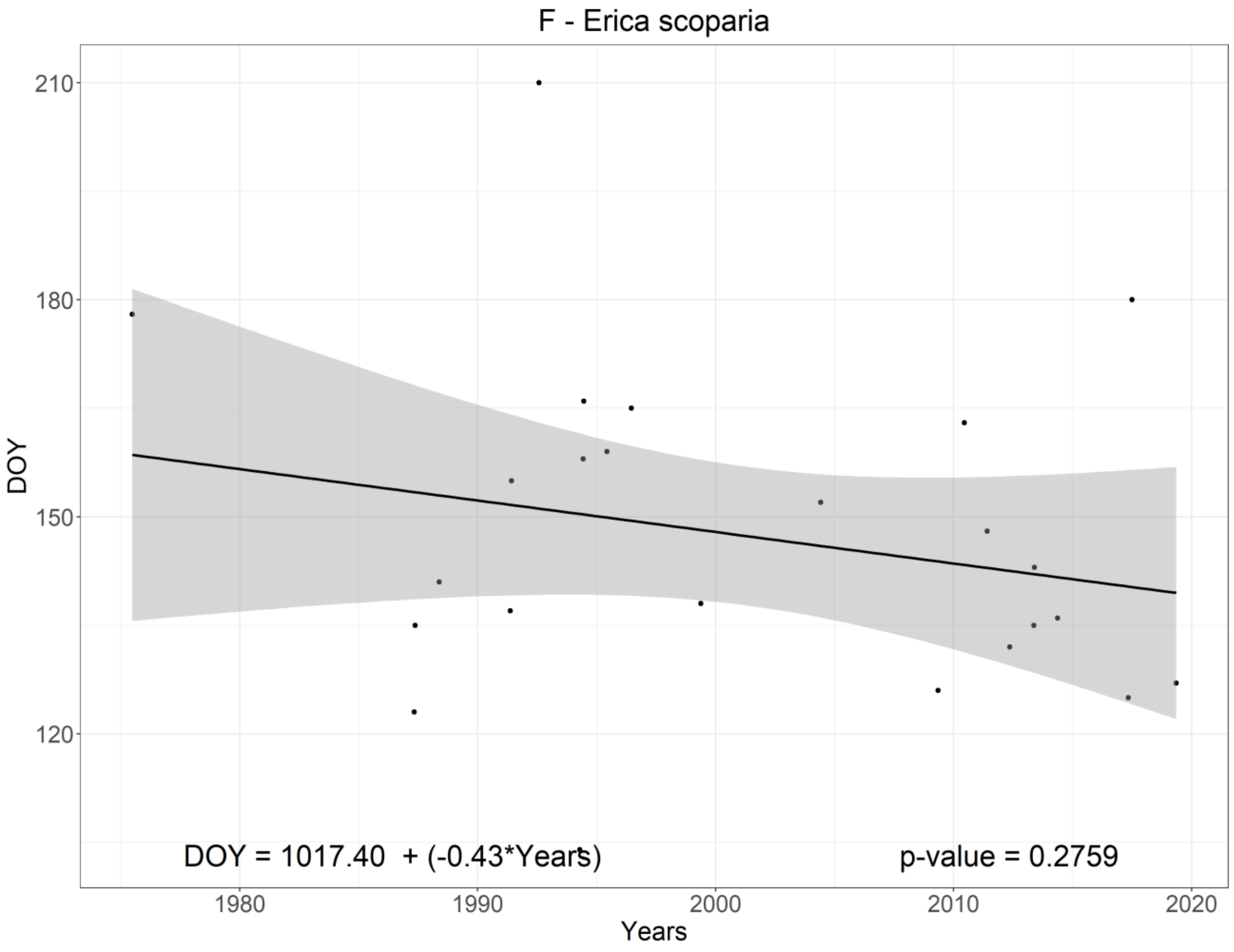

1.42.1. Diagnostics - LM - F - Erica scoparia

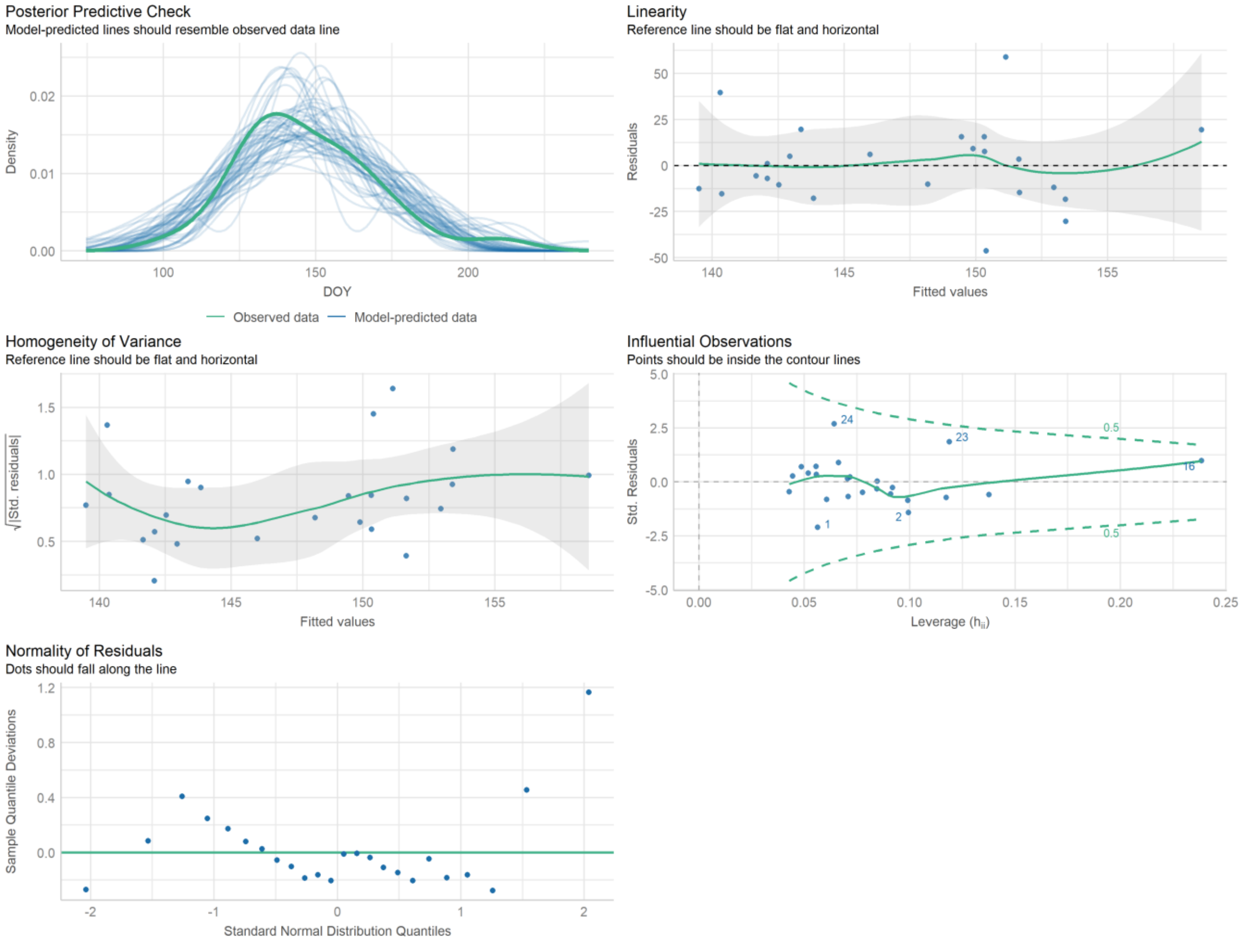

1.43. LM - DVG - Erinacea anthyllis

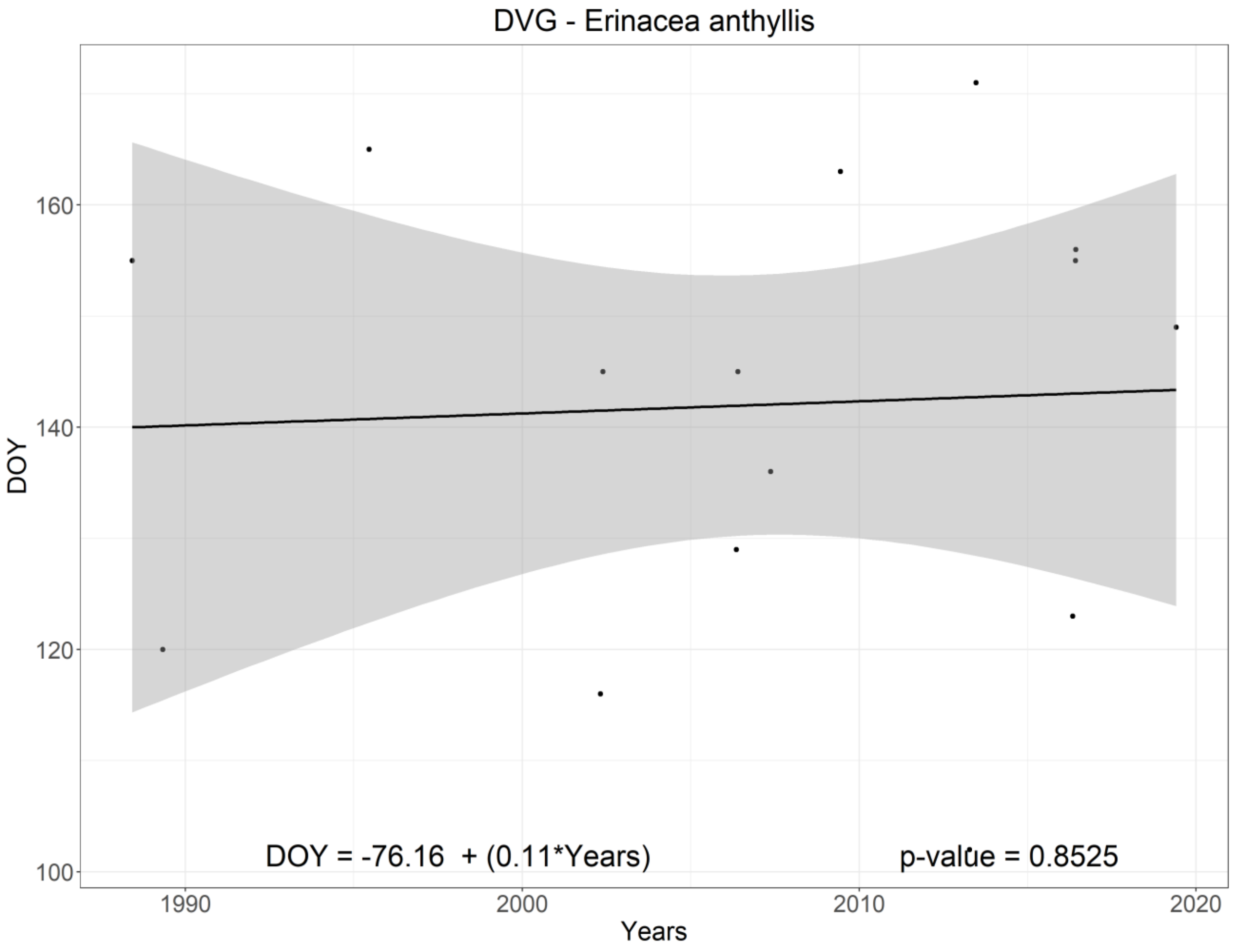

1.43.1. Diagnostics - LM - DVG - Erinacea anthyllis

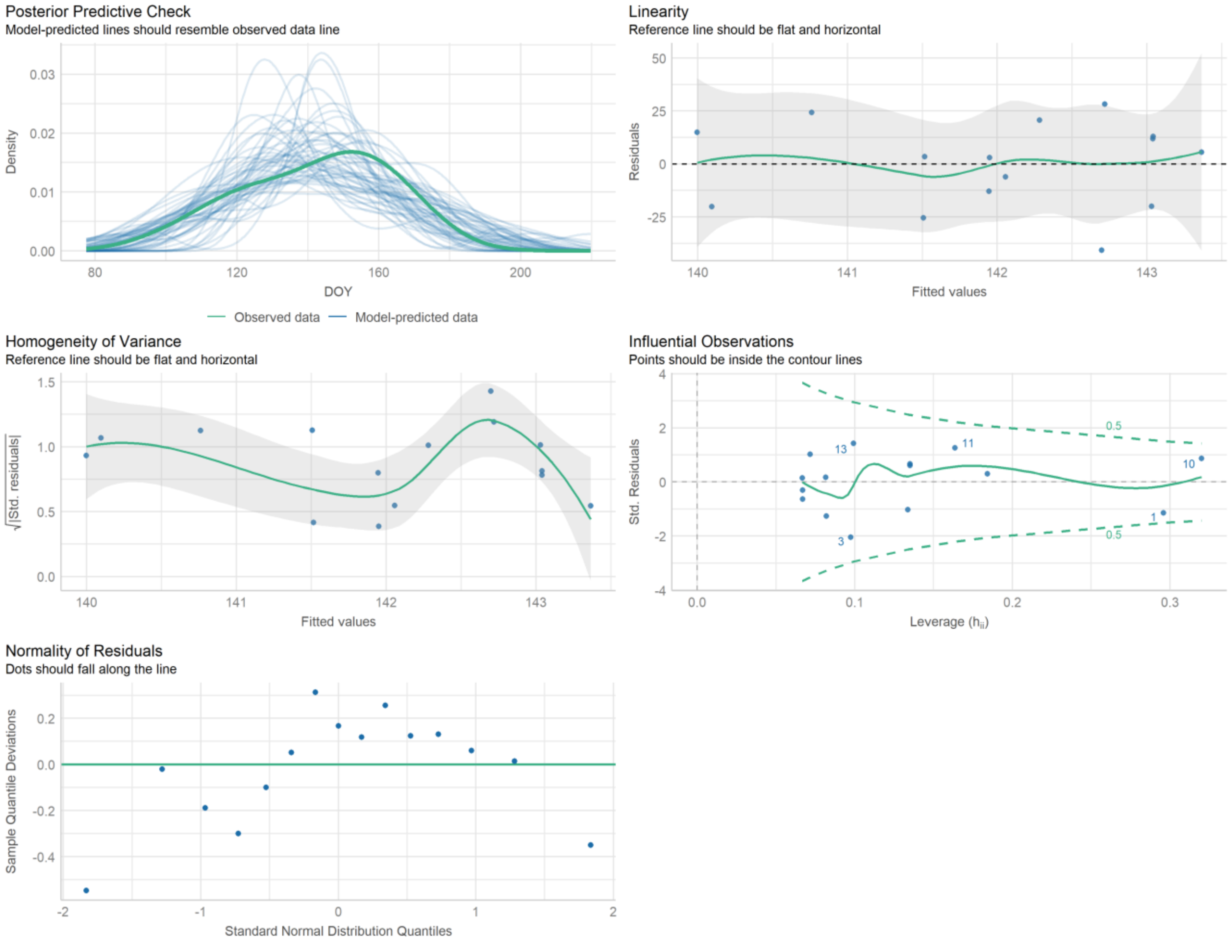

1.44. LM - FBF - Fumana thymifolia

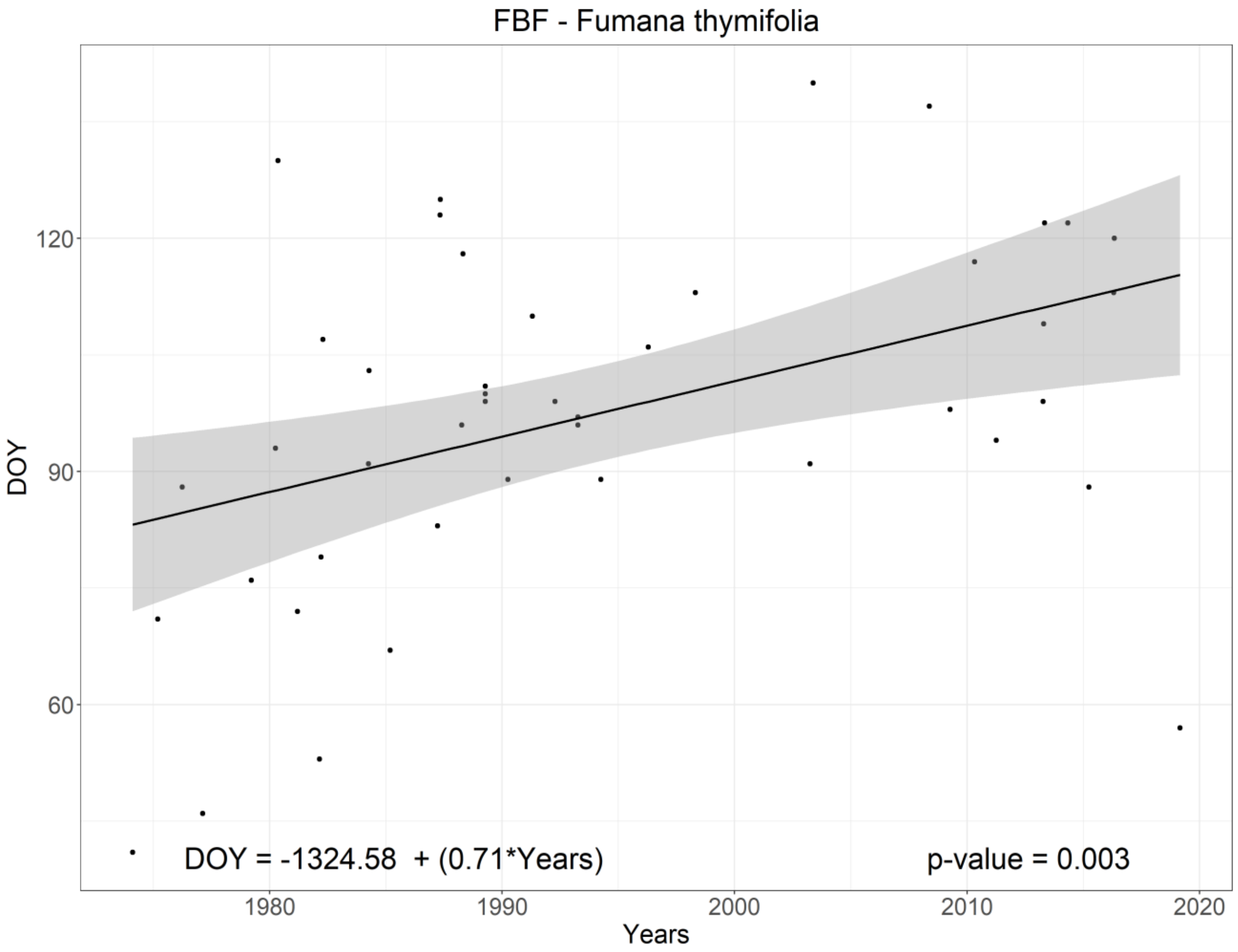

1.44.1. Diagnostics - LM - FBF - Fumana thymifolia

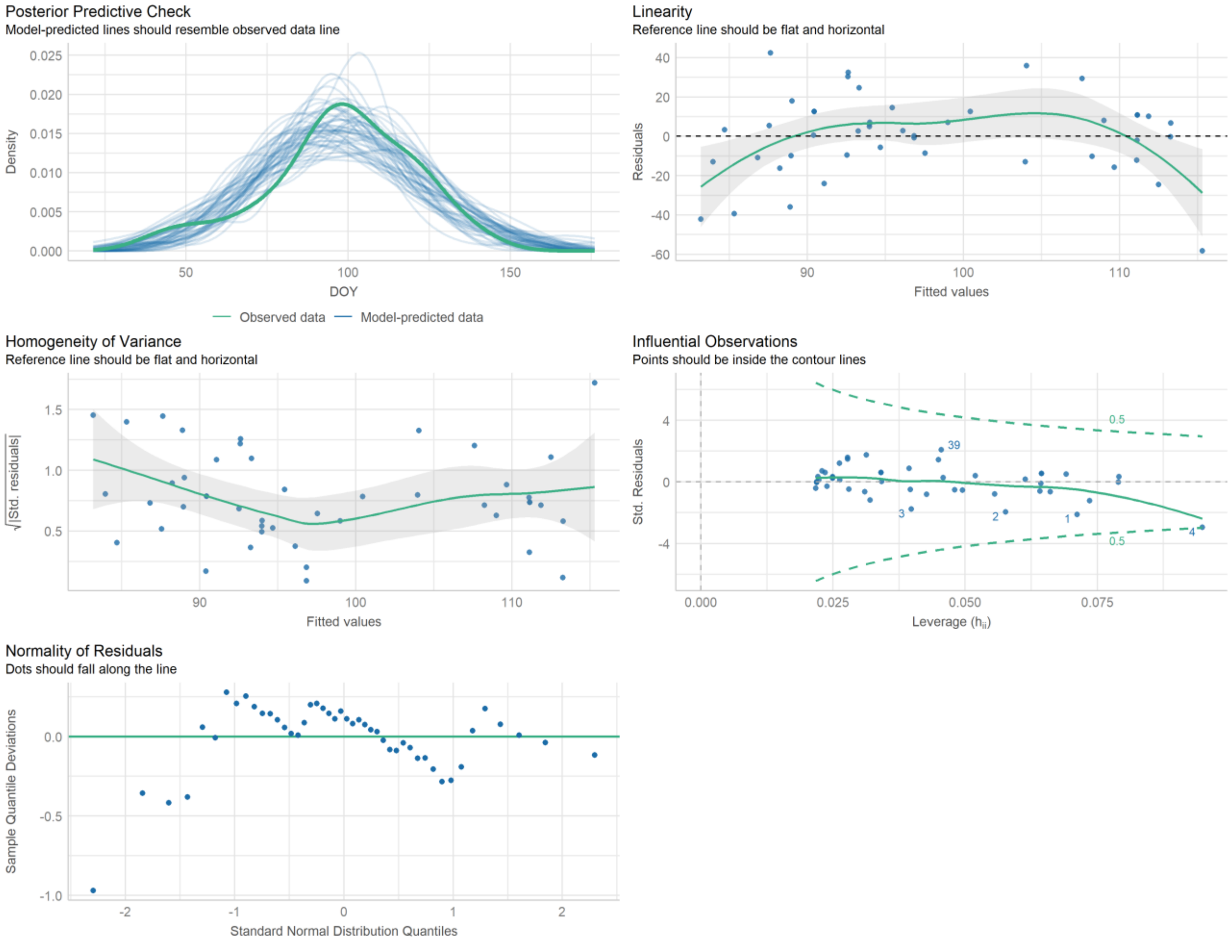

1.45. LM - DVG - Fumana thymifolia

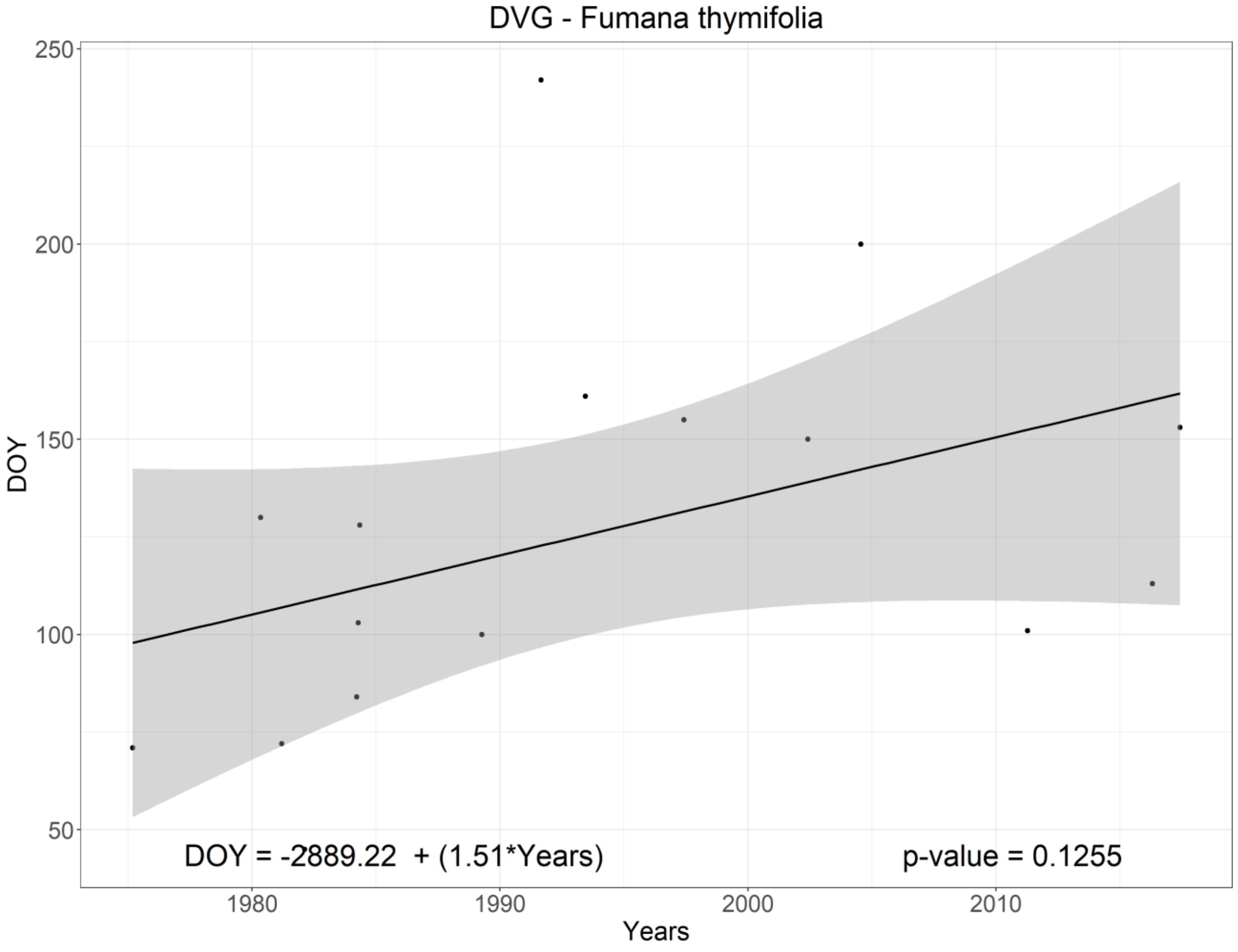

1.45.1. Diagnostics - LM - DVG - Fumana thymifolia

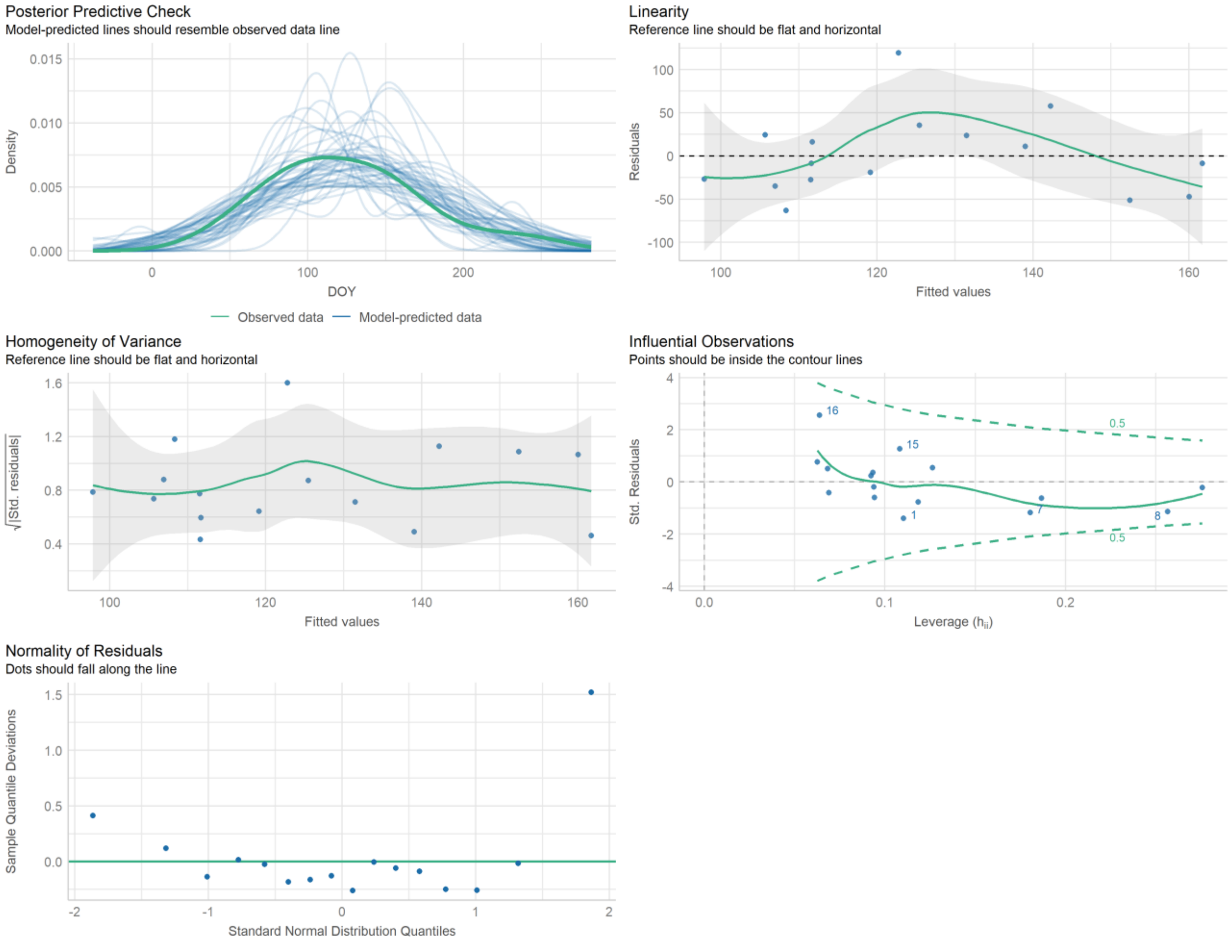

1.46. LM - FBF - Genista hirsuta subsp. lanuginosa

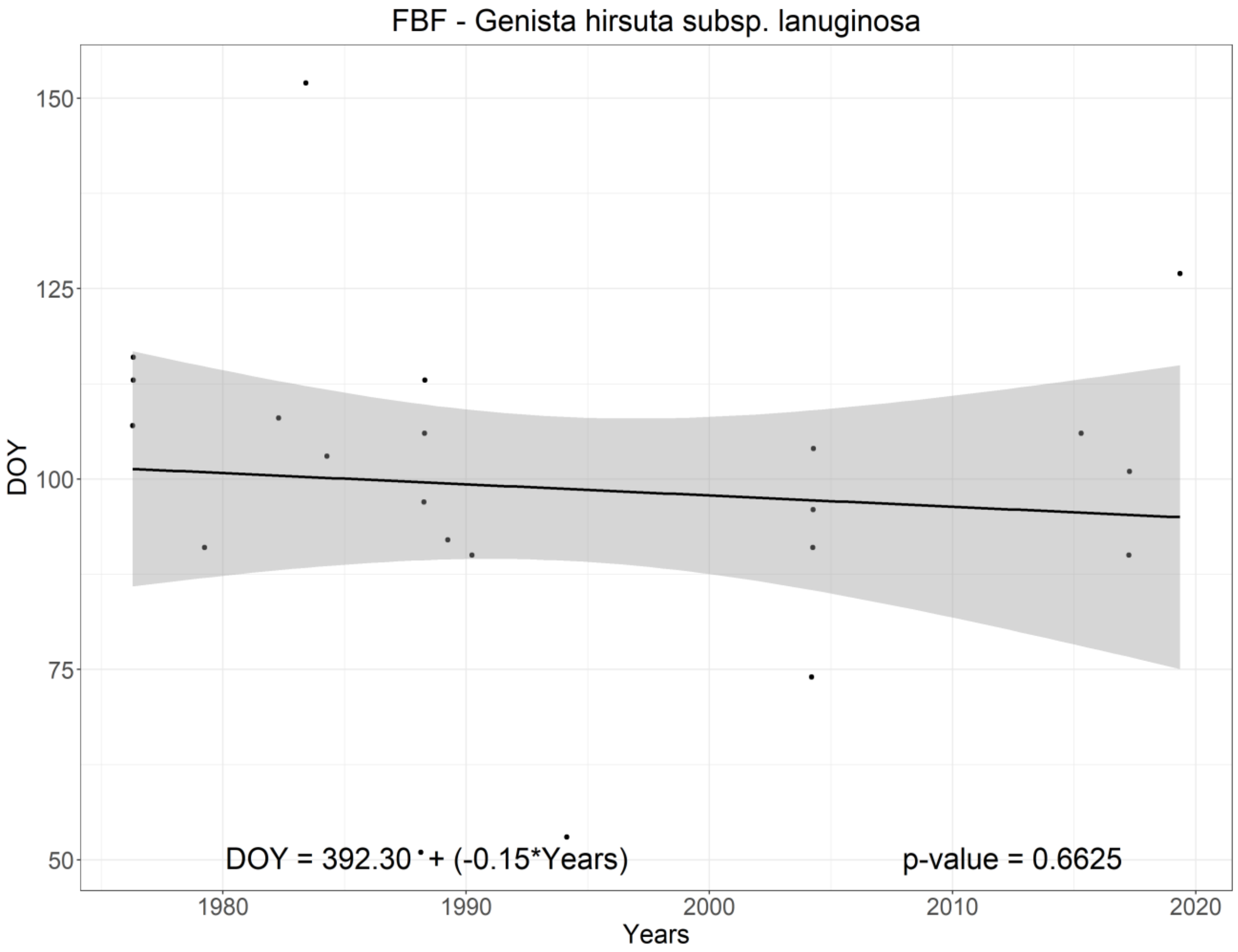

1.46.1. Diagnostics - LM - FBF - Genista hirsuta subsp. lanuginosa

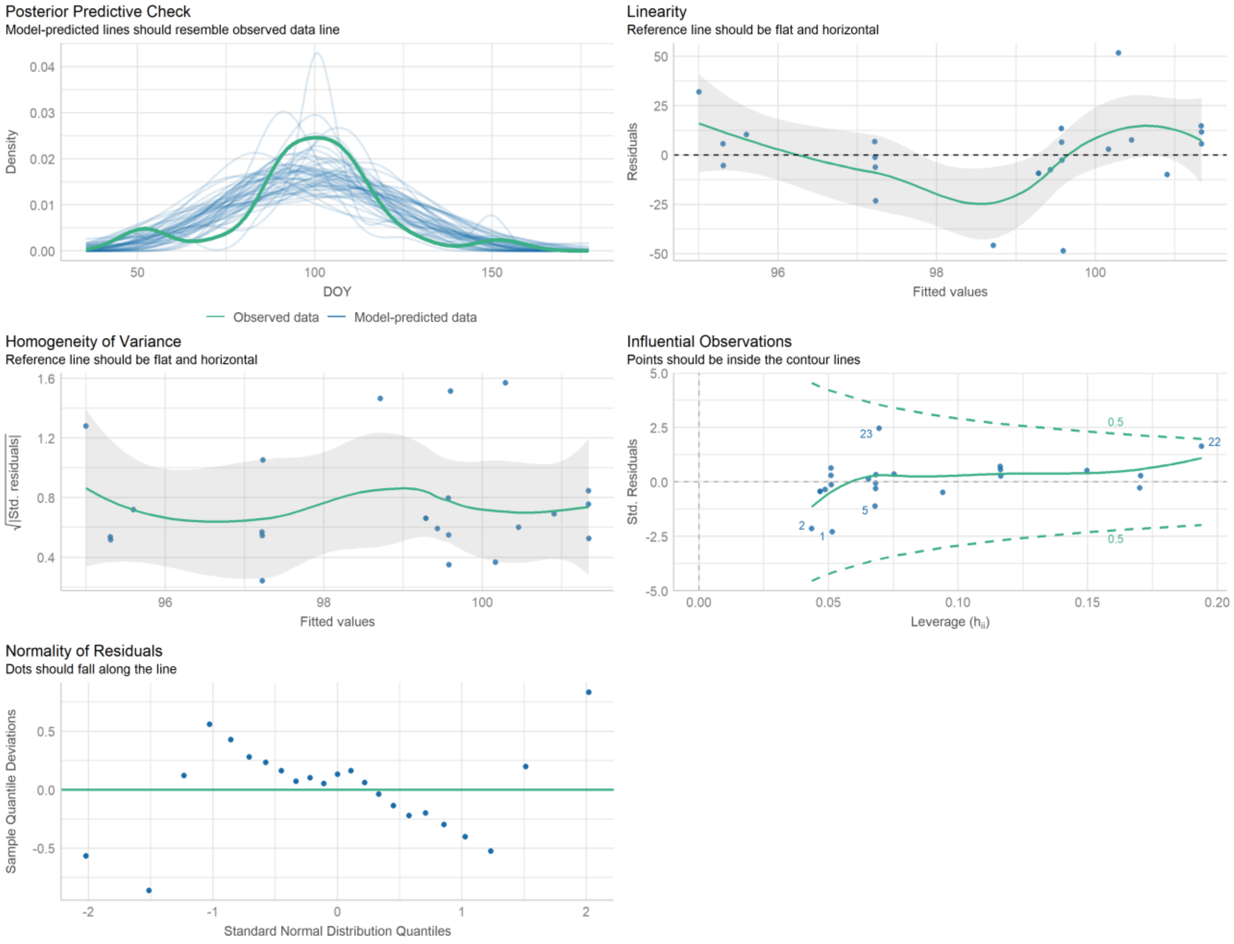

1.47. LM - F - Genista hirsuta subsp. lanuginosa

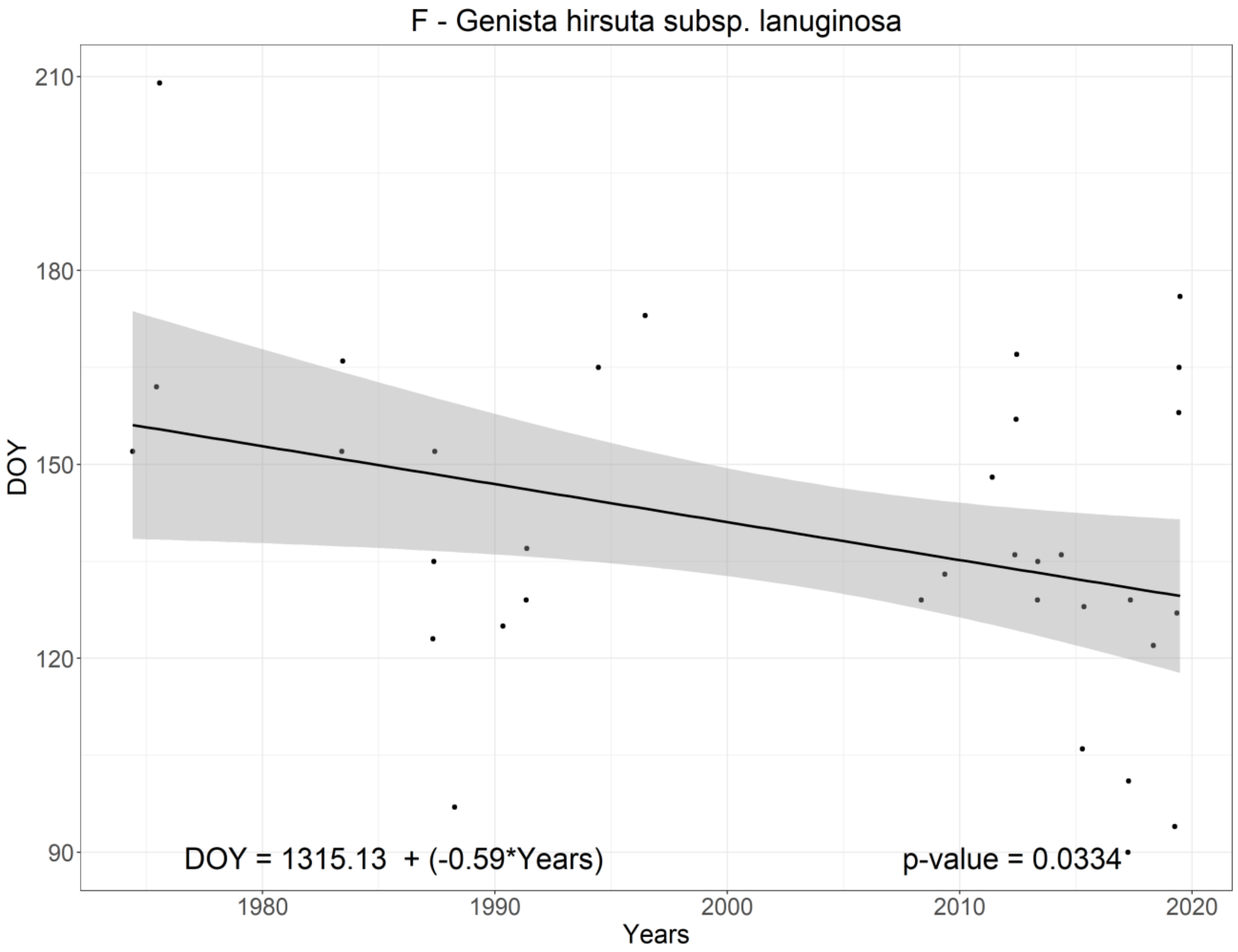

1.47.1. Diagnostics - LM - F - Genista hirsuta subsp. lanuginosa

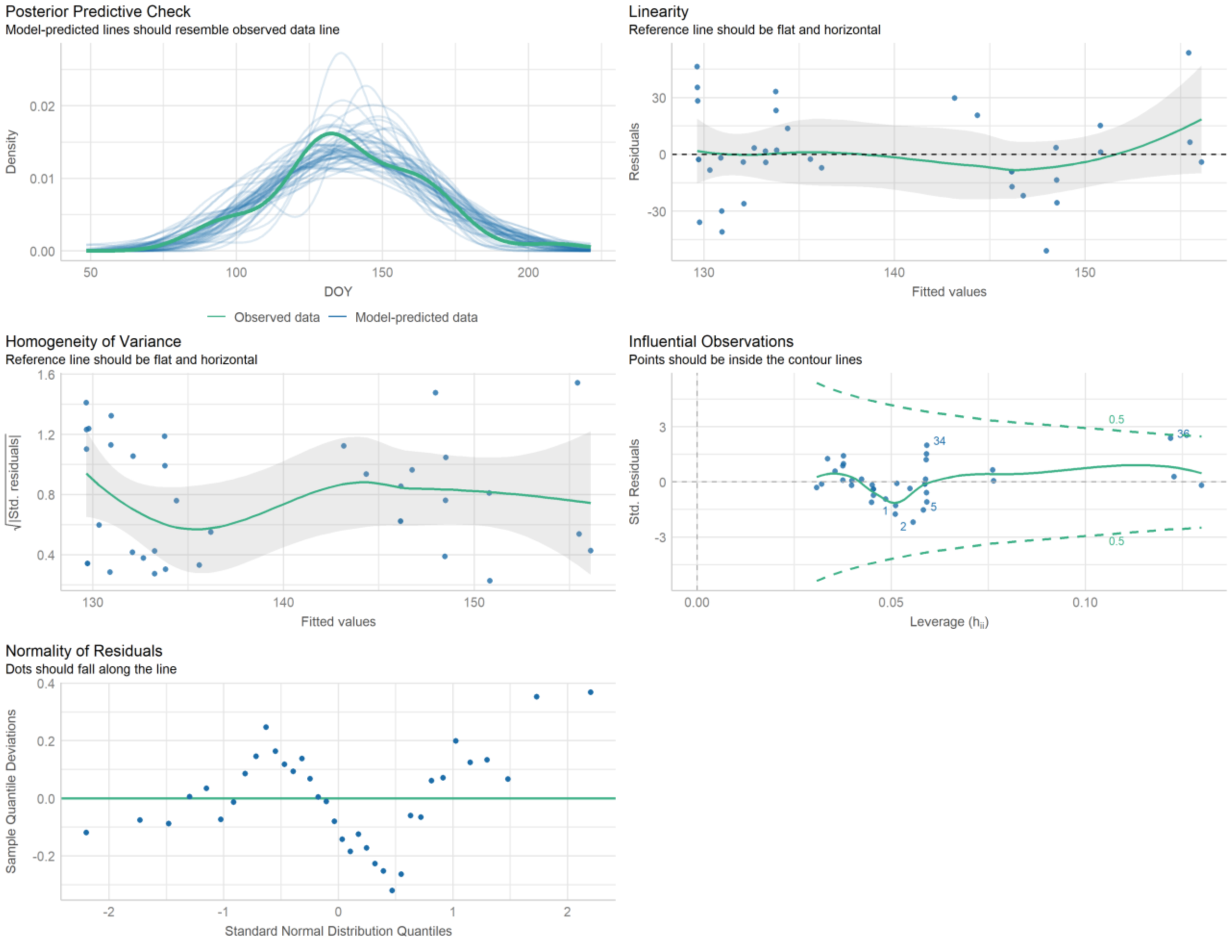

1.48. LM - F - Glandora prostrata subsp. lusitanica

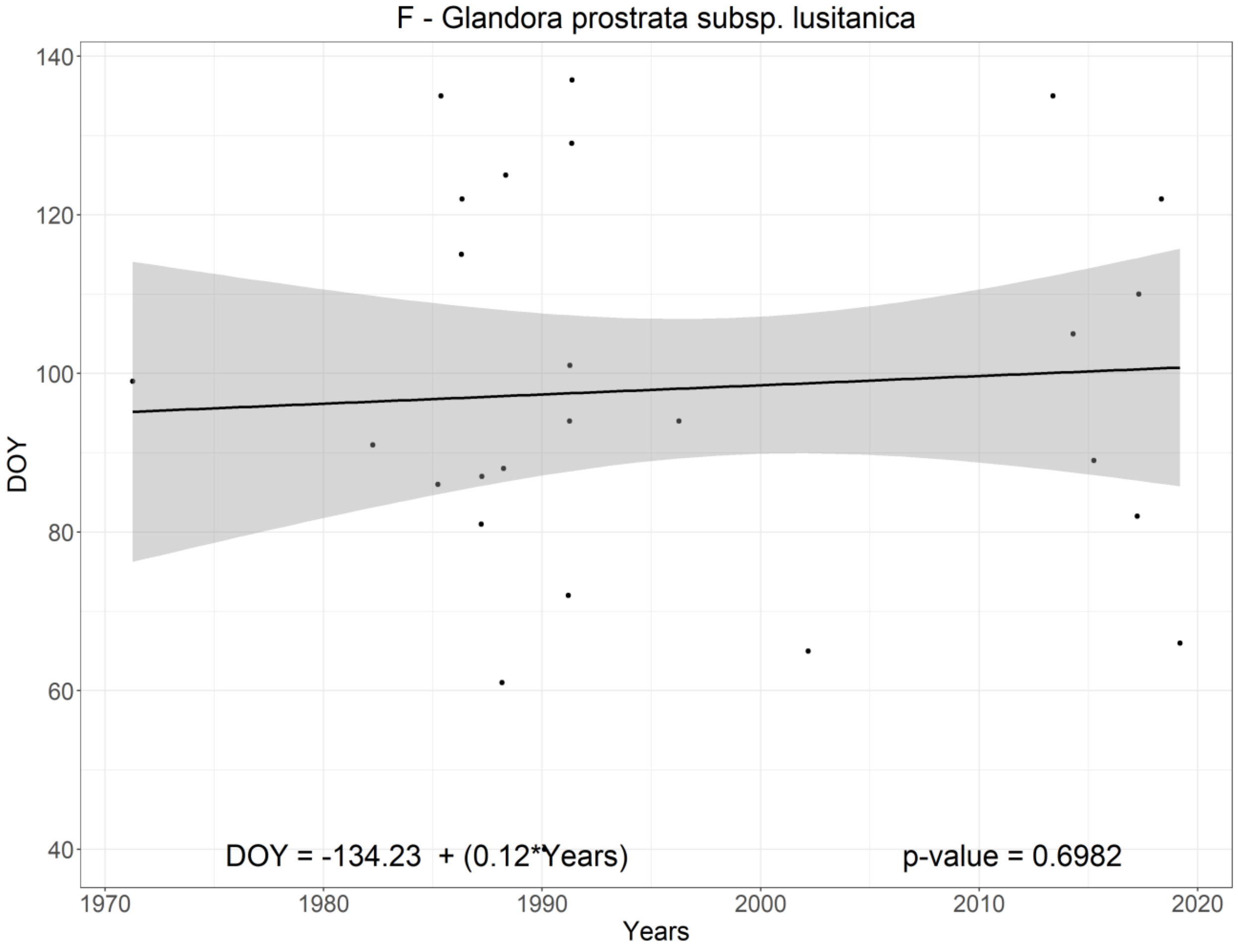

1.48.1. Diagnostics - LM - F - Glandora prostrata subsp. lusitanica

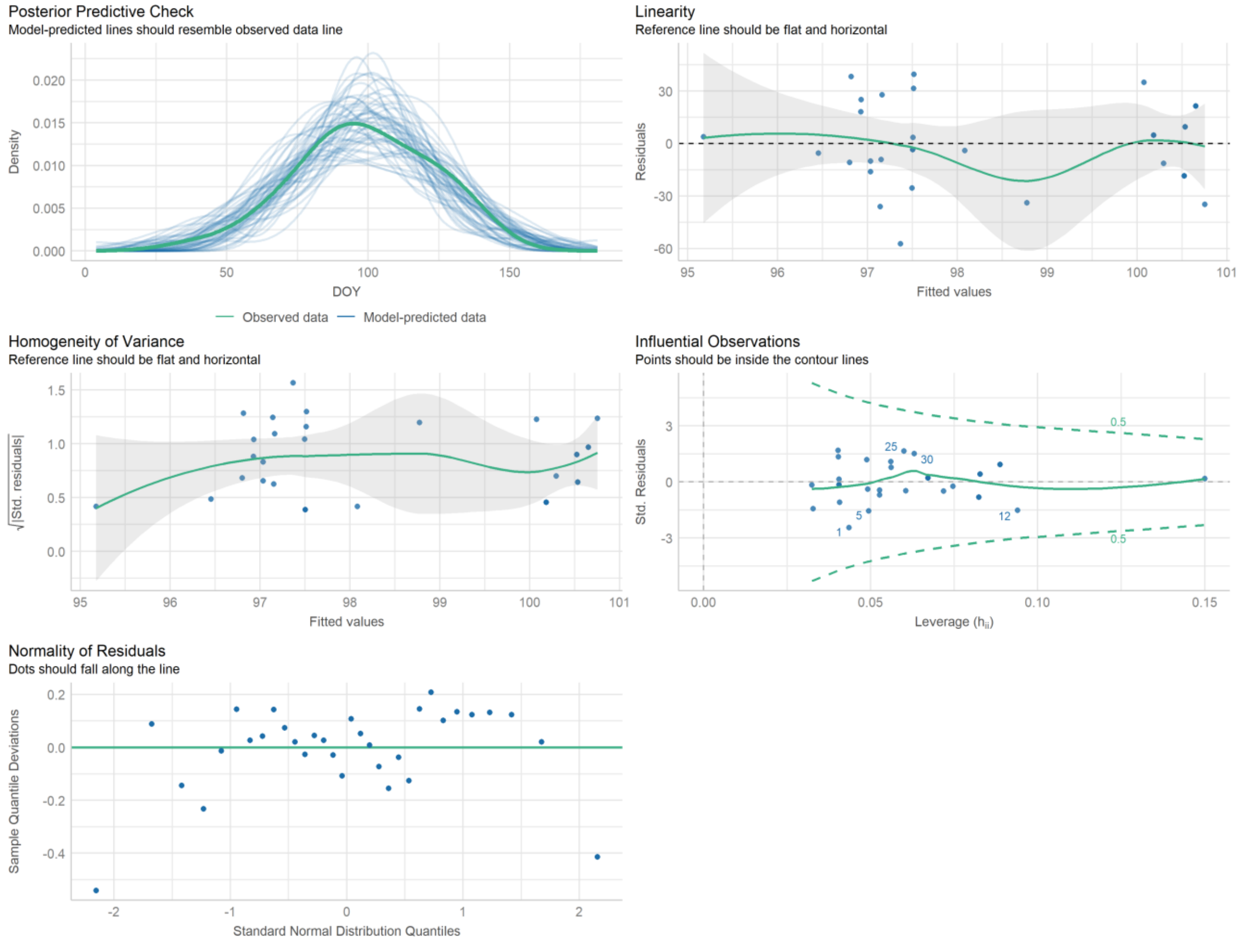

1.49. LM - FBF - Halimium atriplicifolium

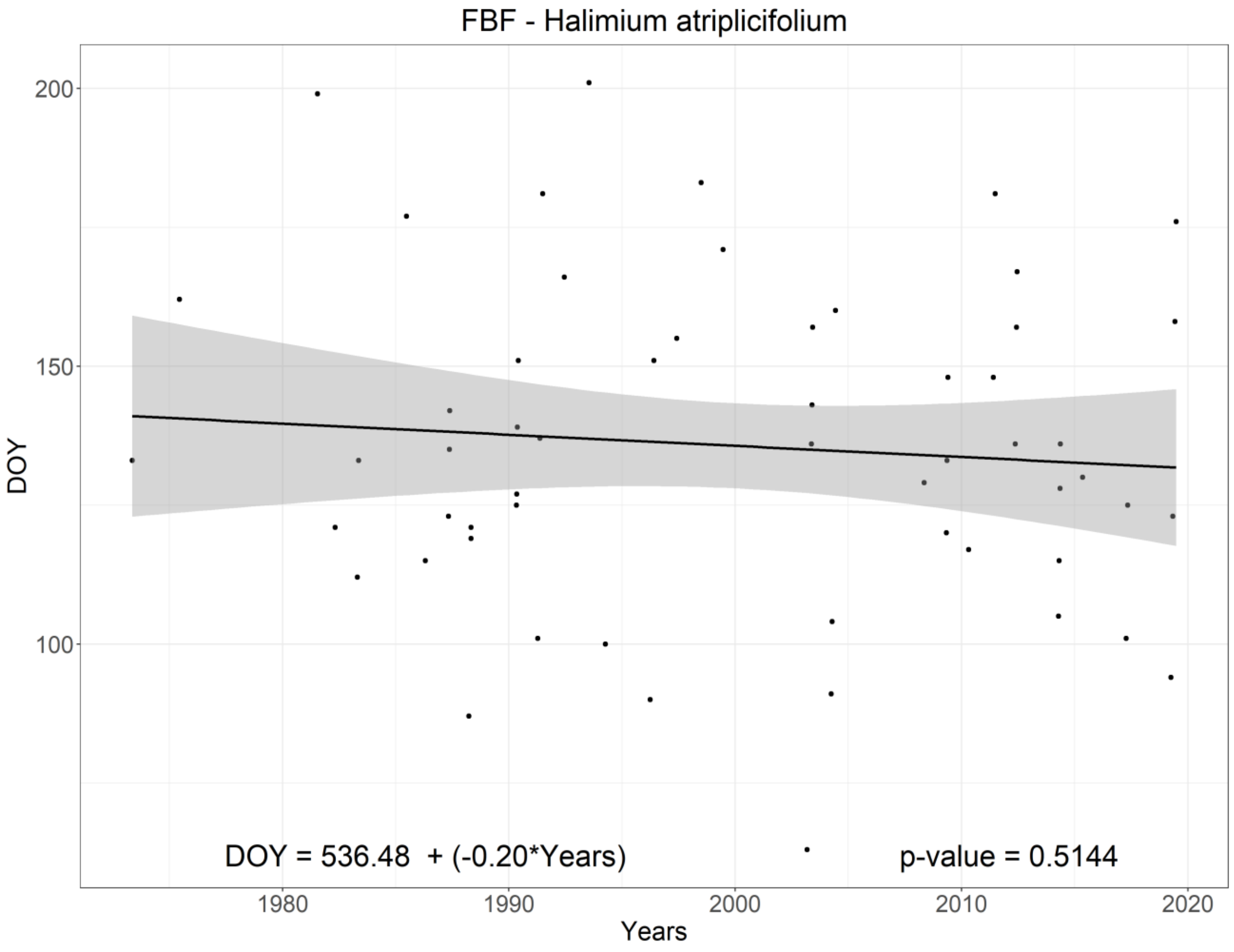

1.49.1. Diagnostics - LM - FBF - Halimium atriplicifolium

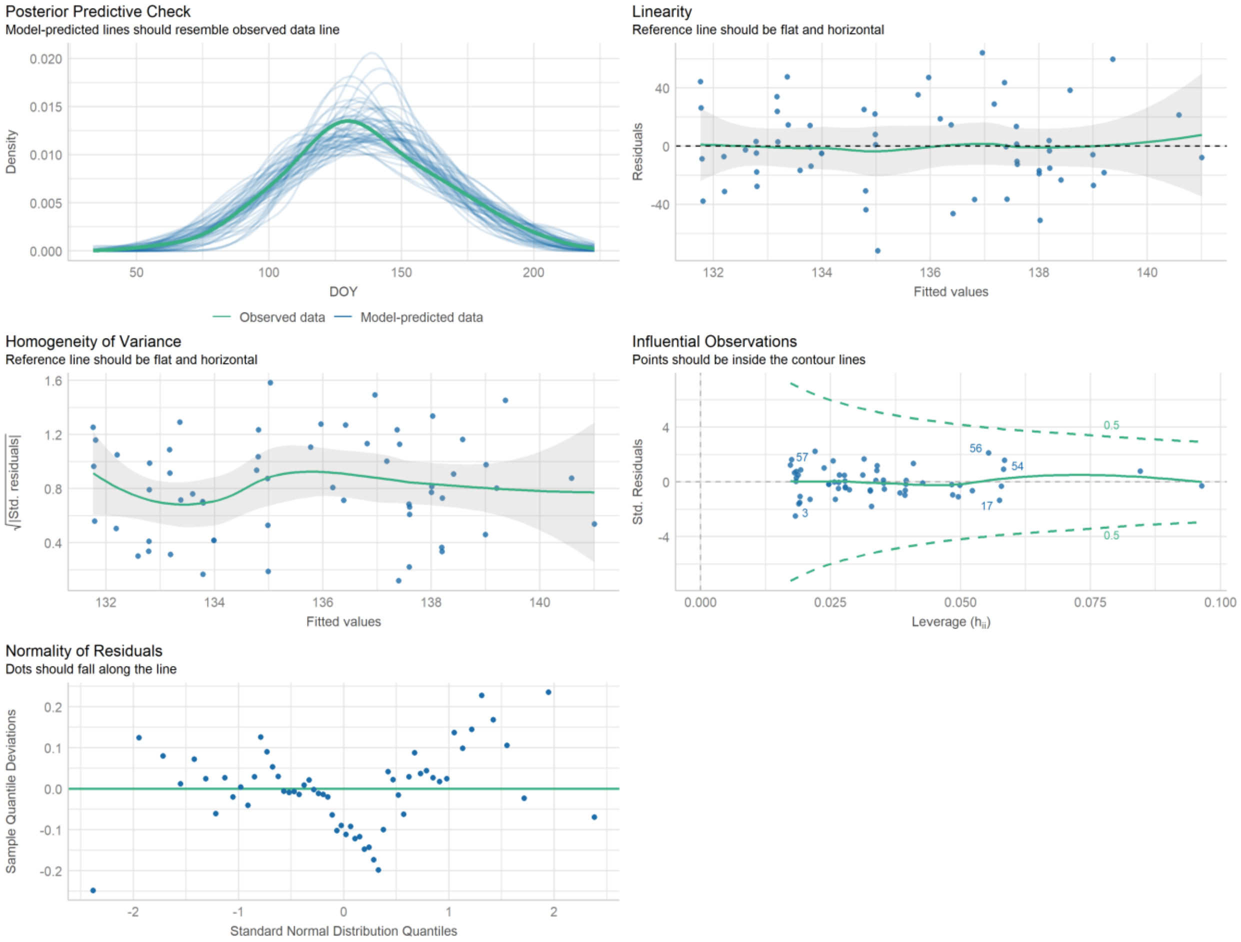

1.50. LM - F - Halimium atriplicifolium

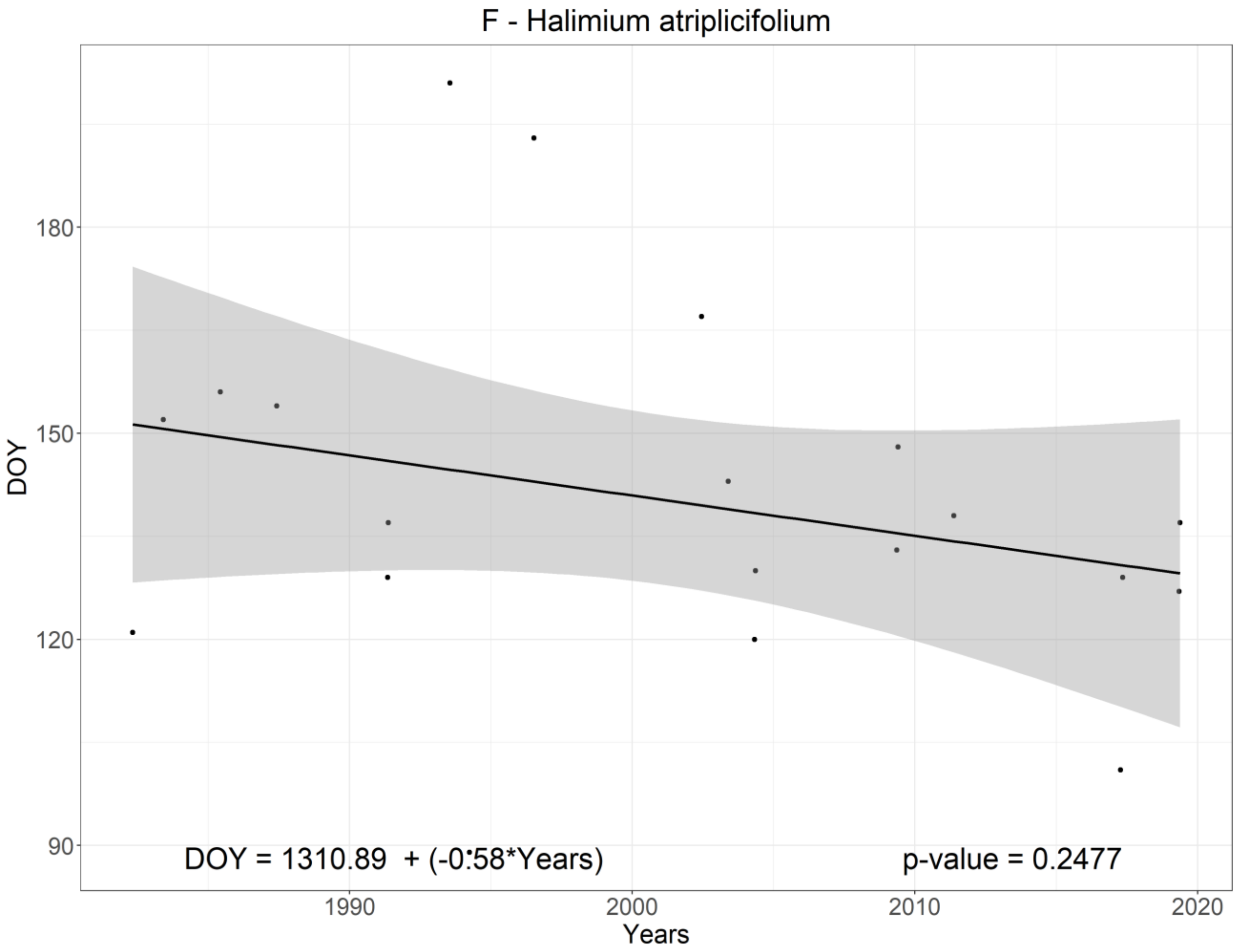

1.50.1. Diagnostics - LM - F - Halimium atriplicifolium

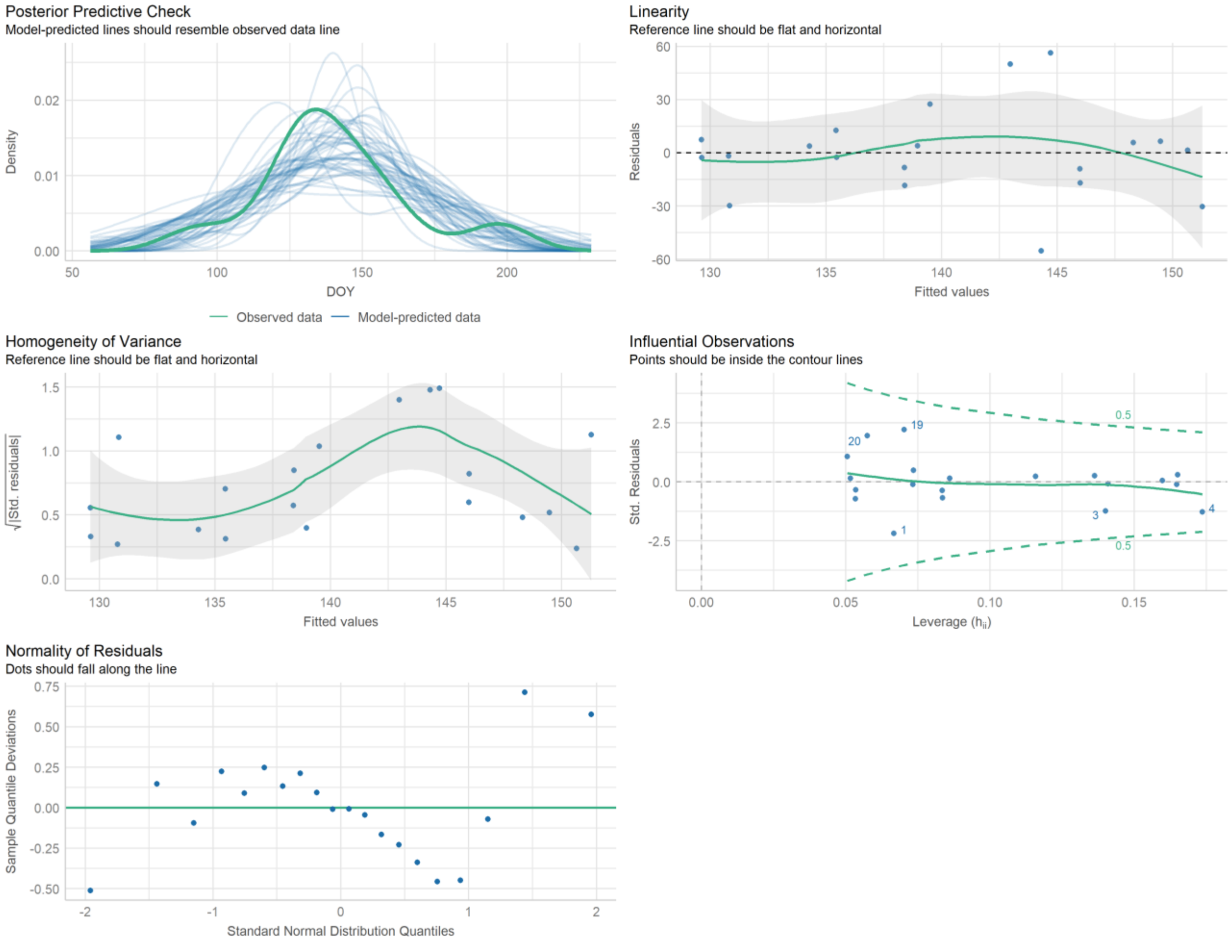

1.51. LM - FBF - Helianthemum syriacum

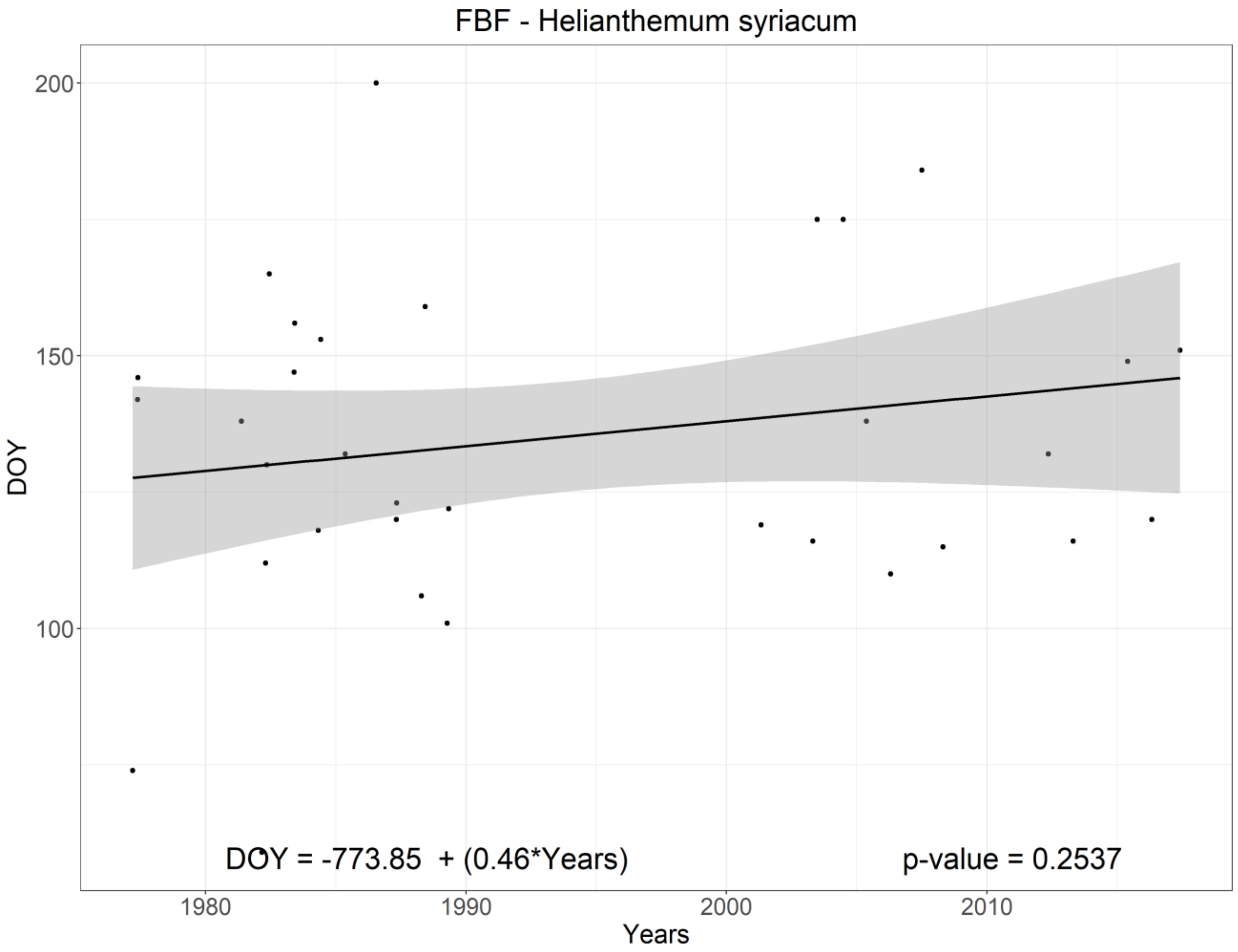

1.51.1. Diagnostics - LM - FBF - Helianthemum syriacum

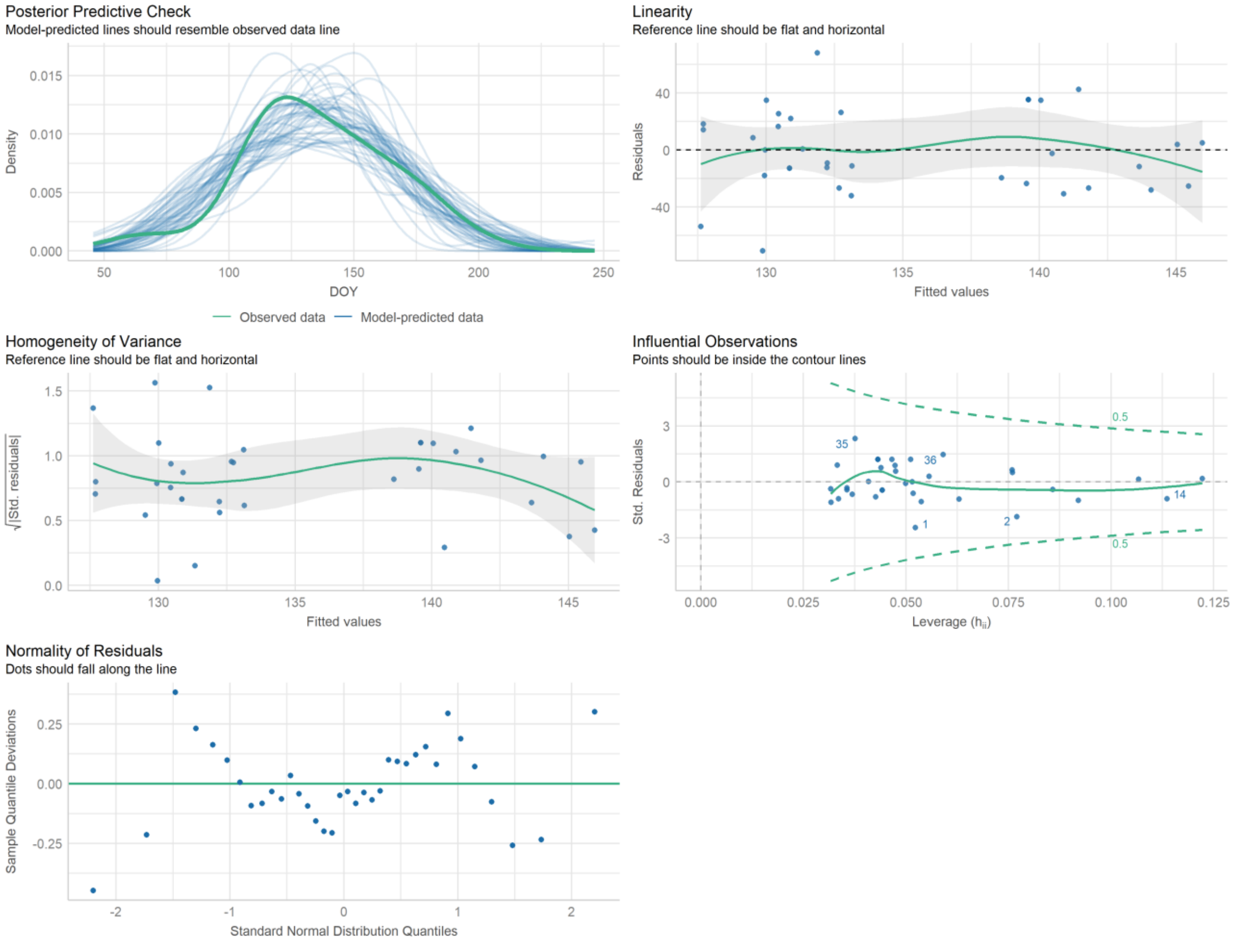

1.52. LM - F - Helianthemum syriacum

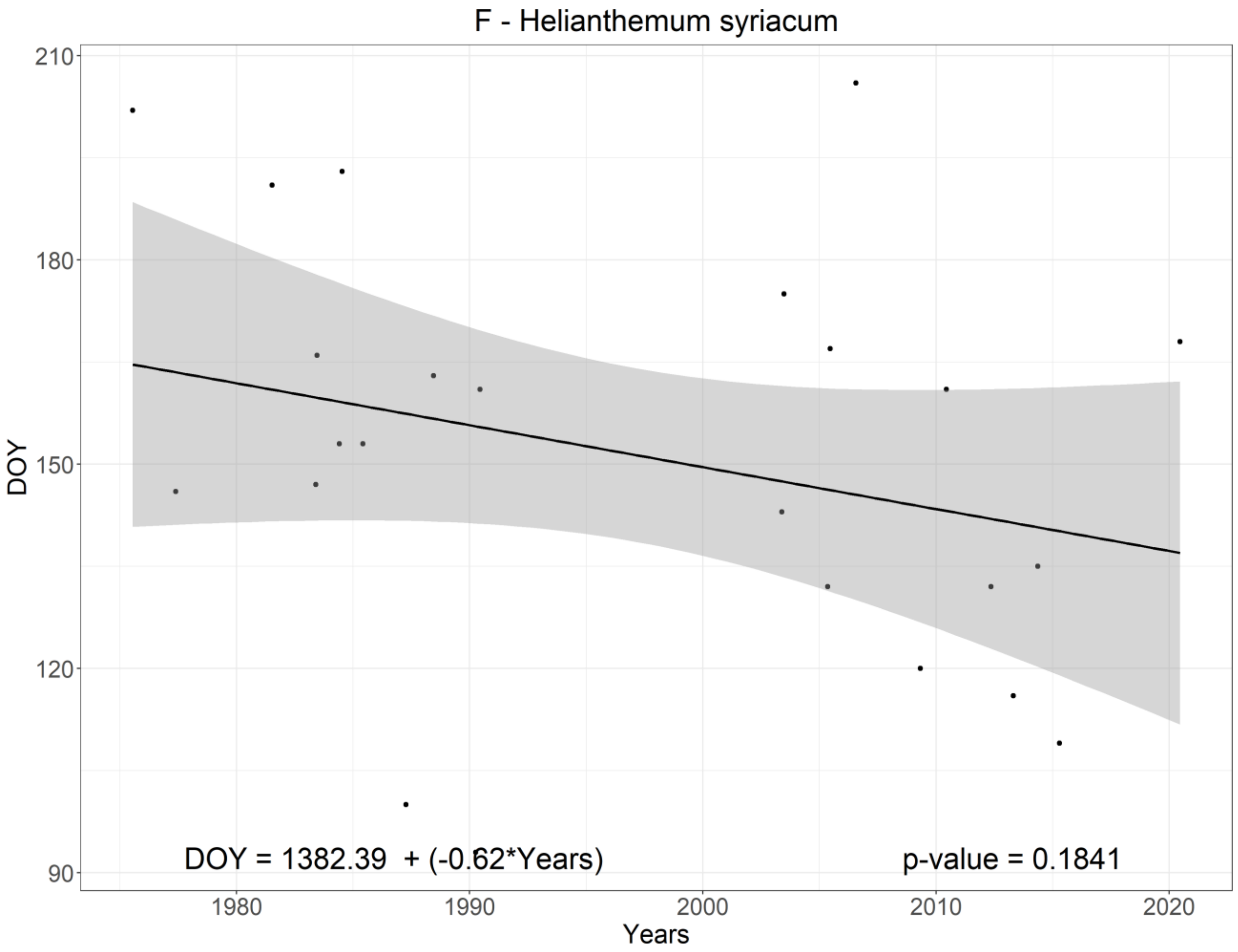

1.52.1. Diagnostics - LM - F - Helianthemum syriacum

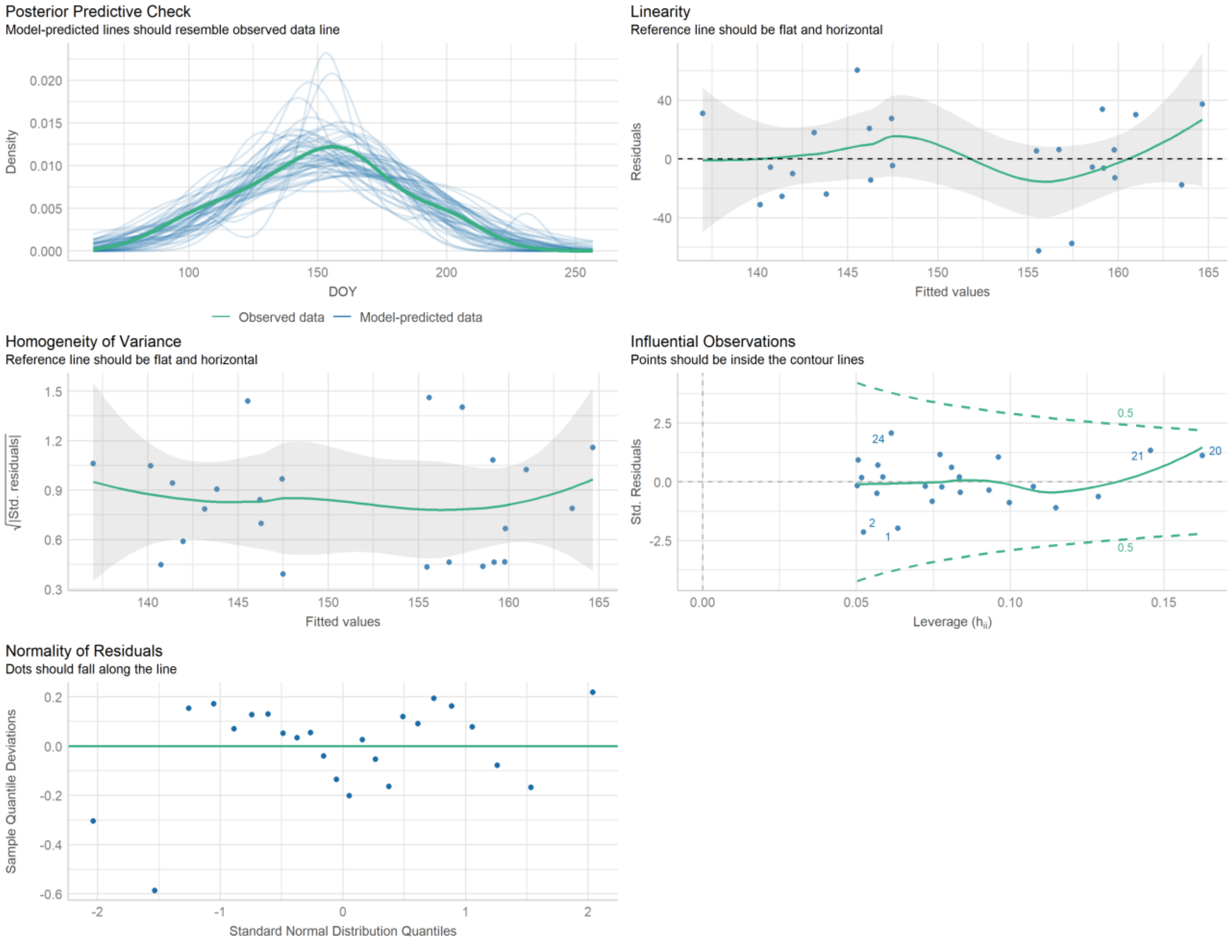

1.53. LM - FBF - Helichrysum stoechas

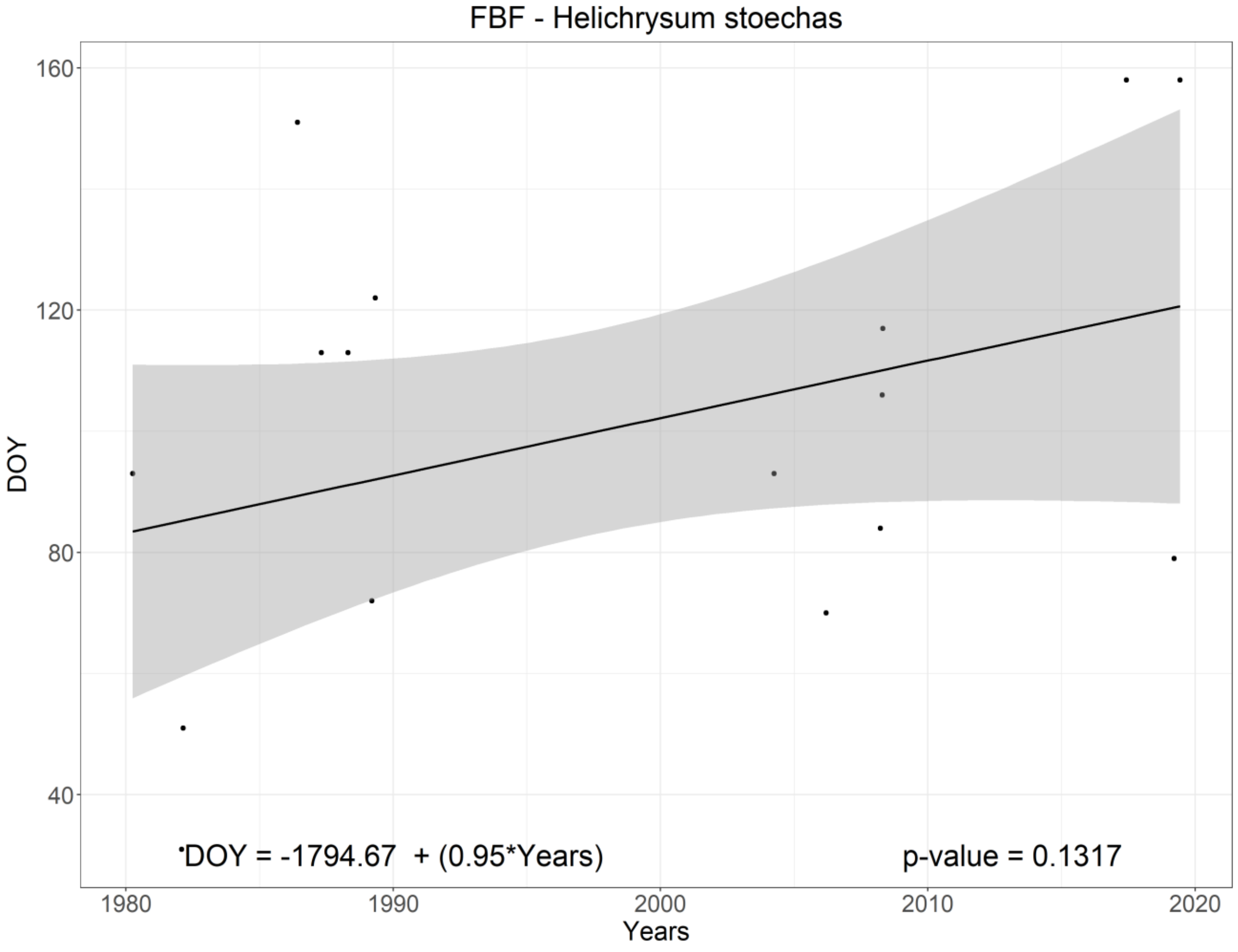

1.53.1. Diagnostics - LM - FBF - Helichrysum stoechas

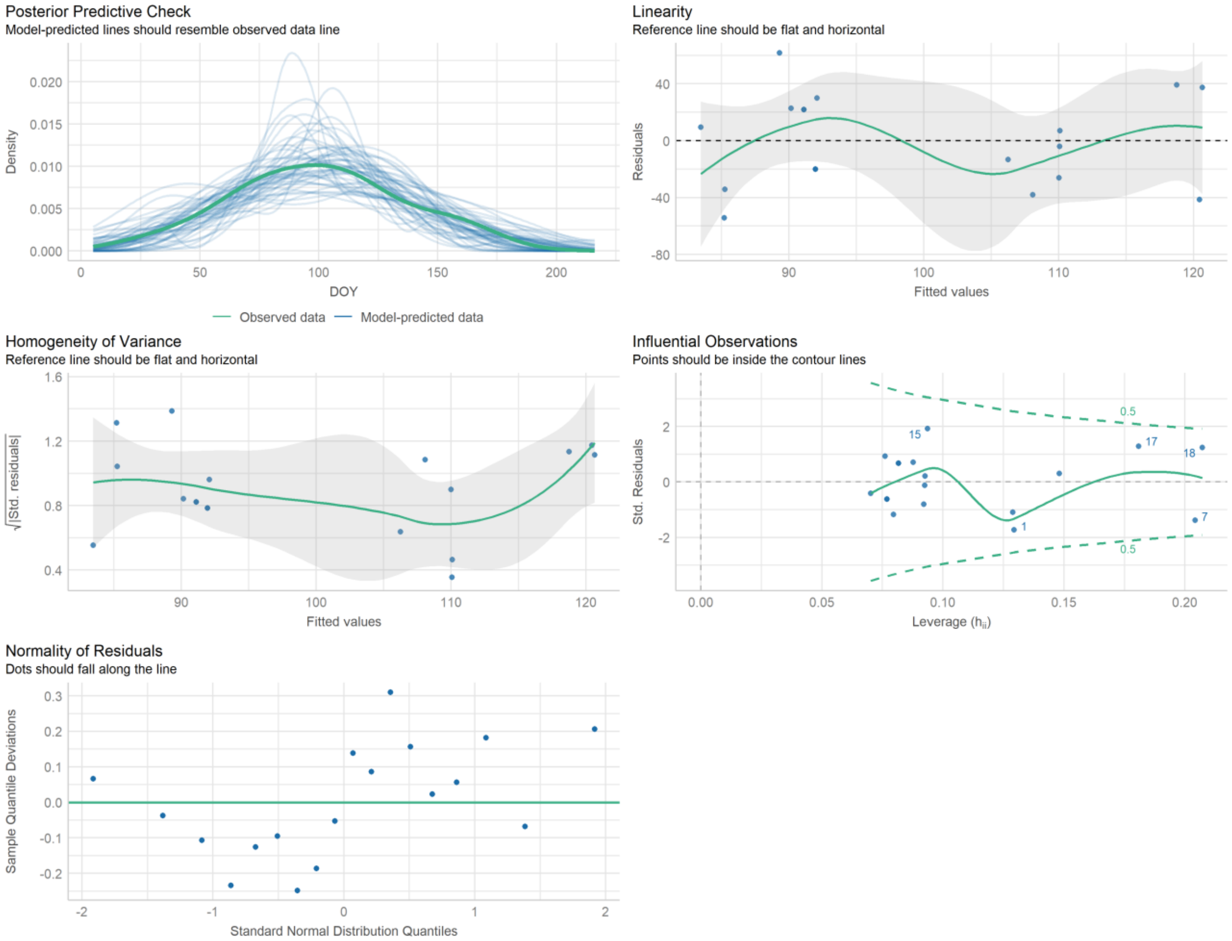

1.54. LM - F - Helichrysum stoechas

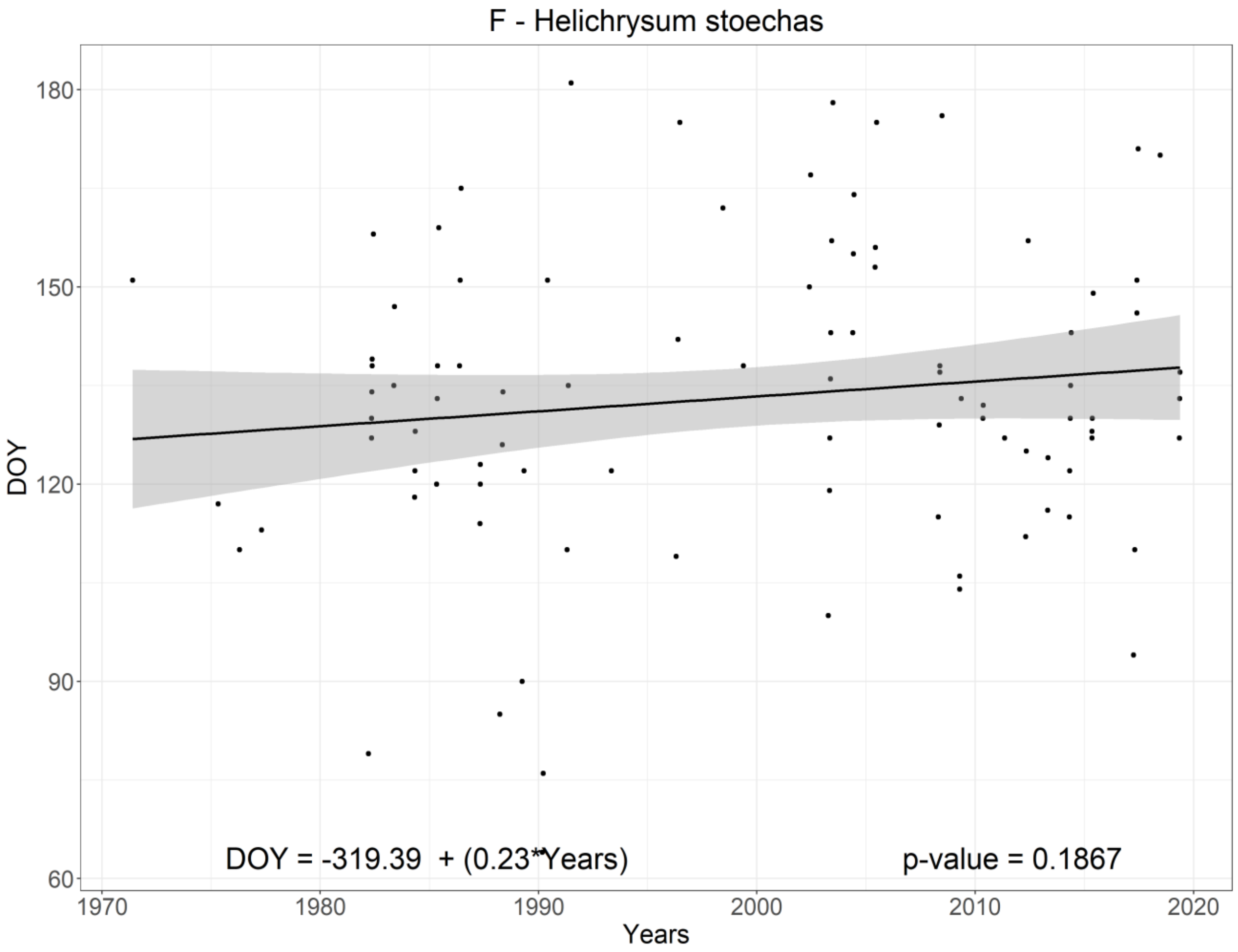

1.54.1. Diagnostics - LM - F - Helichrysum stoechas

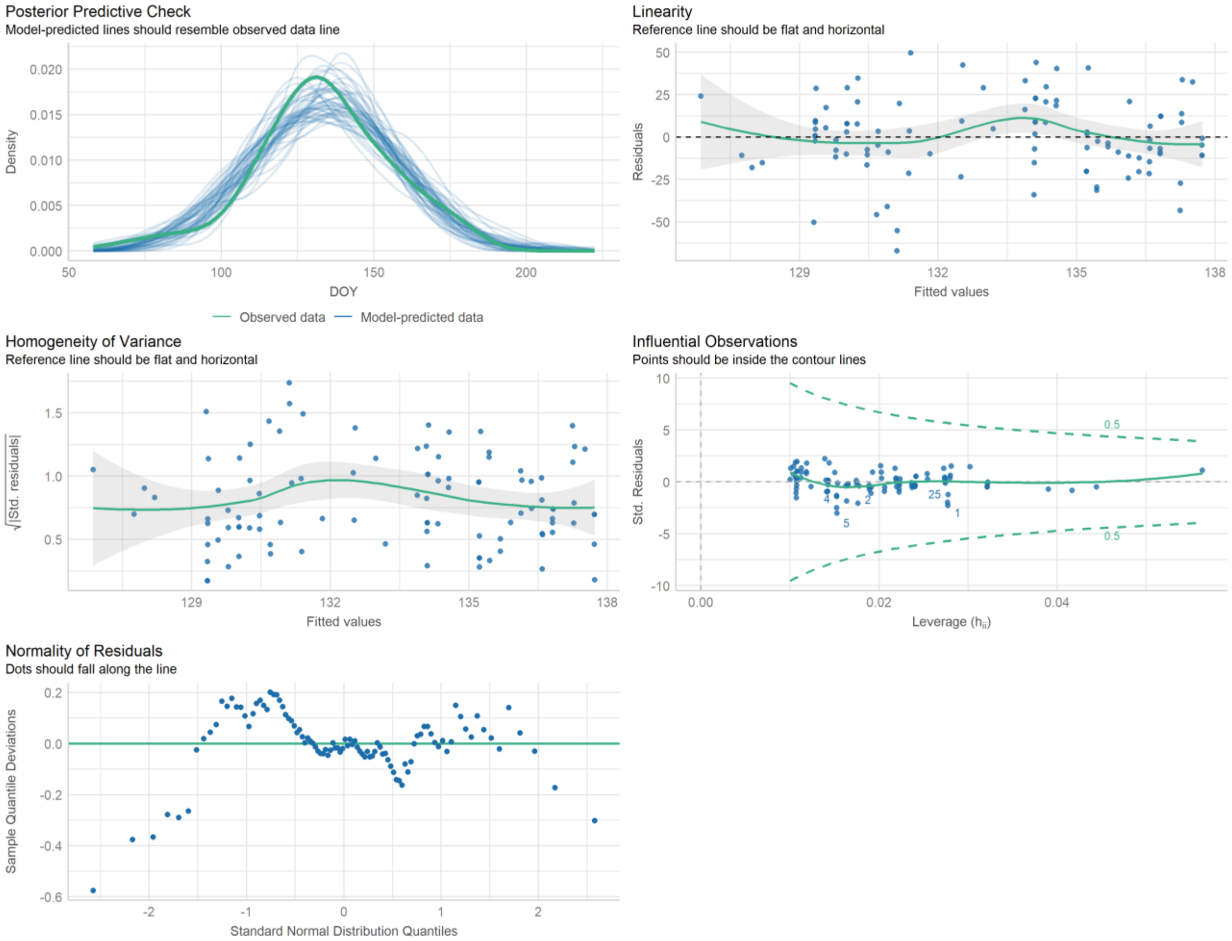

1.55. LM - DVG - Helichrysum stoechas

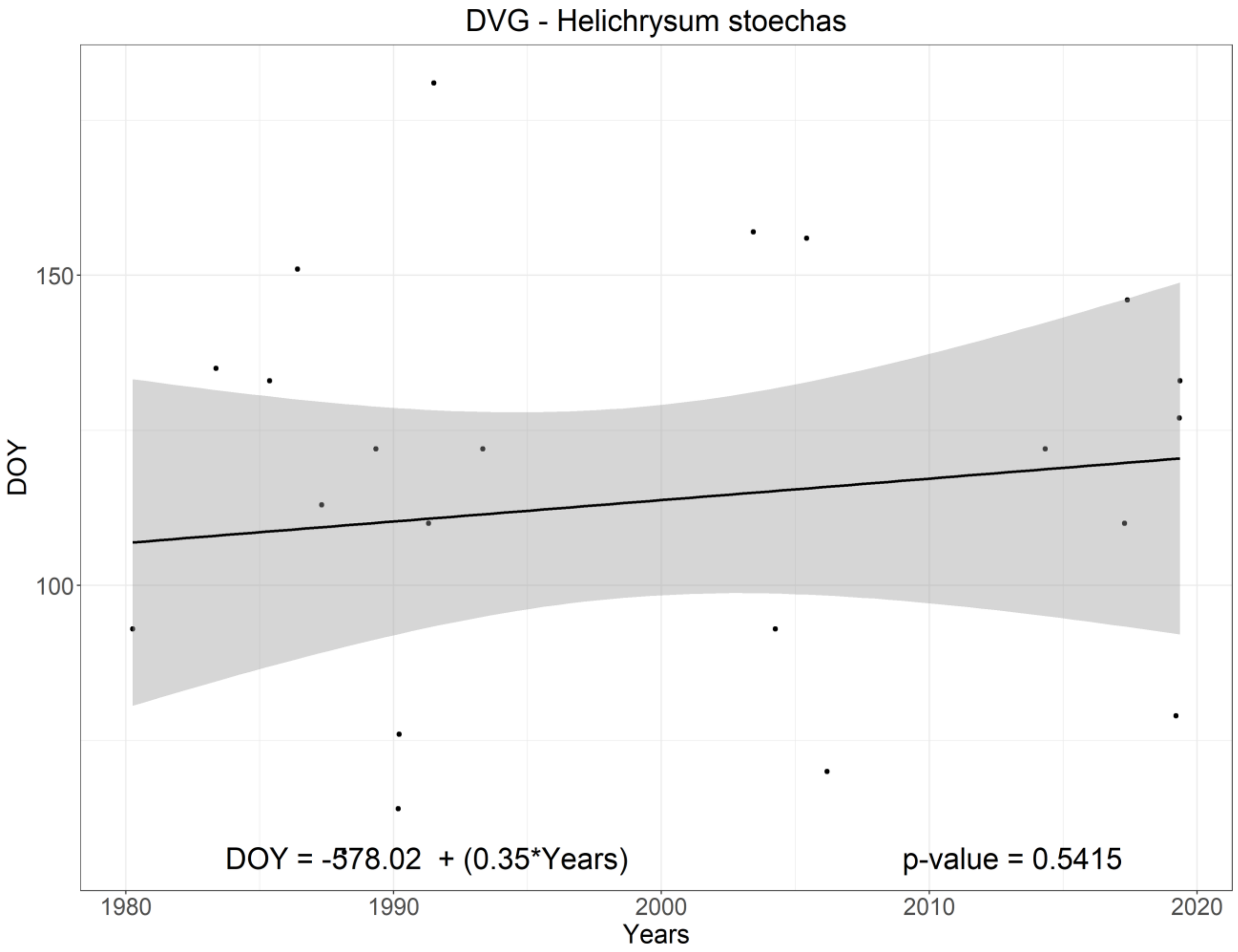

1.55.1. Diagnostics - LM - DVG - Helichrysum stoechas

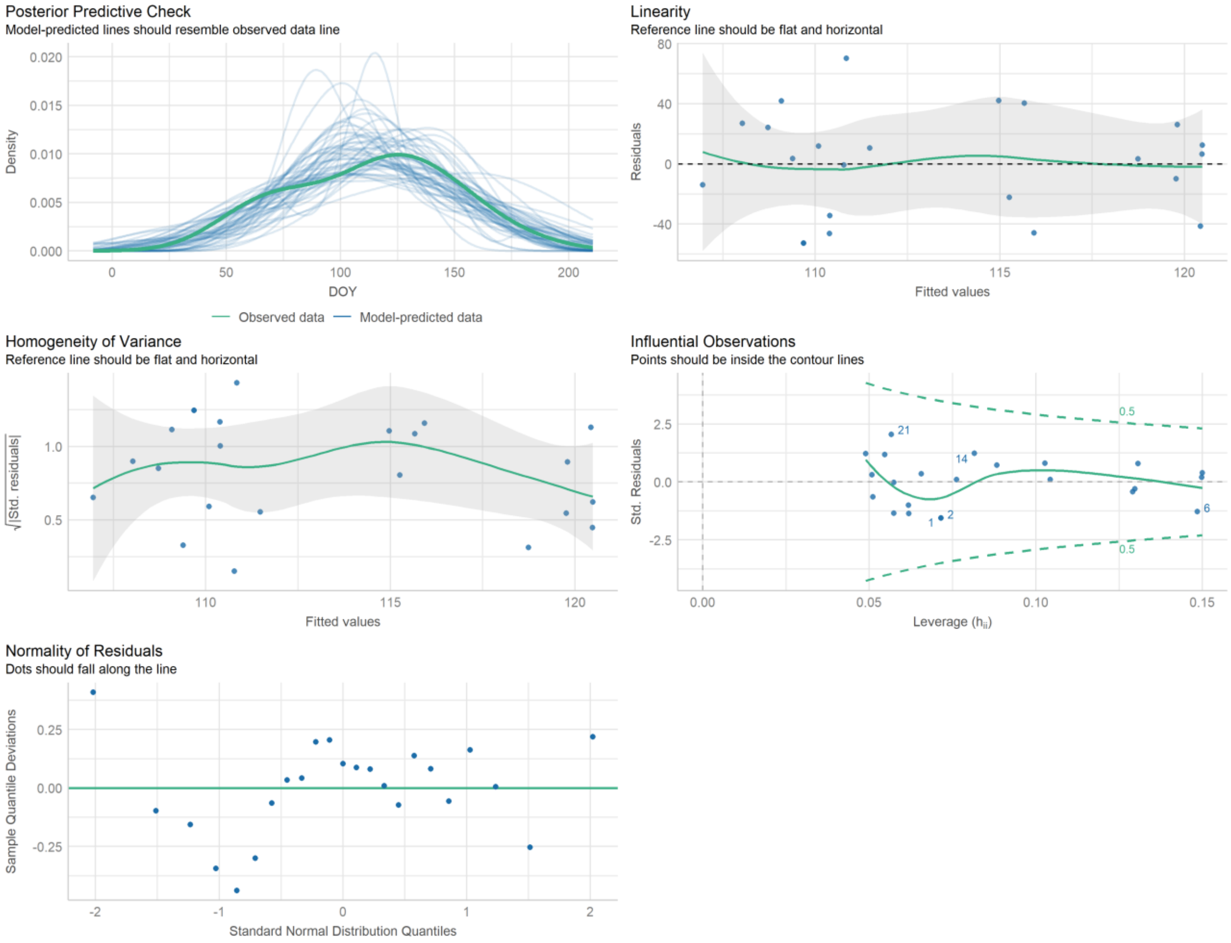

1.56. LM - FS - Helictotrichon filifolium subsp. arundanum

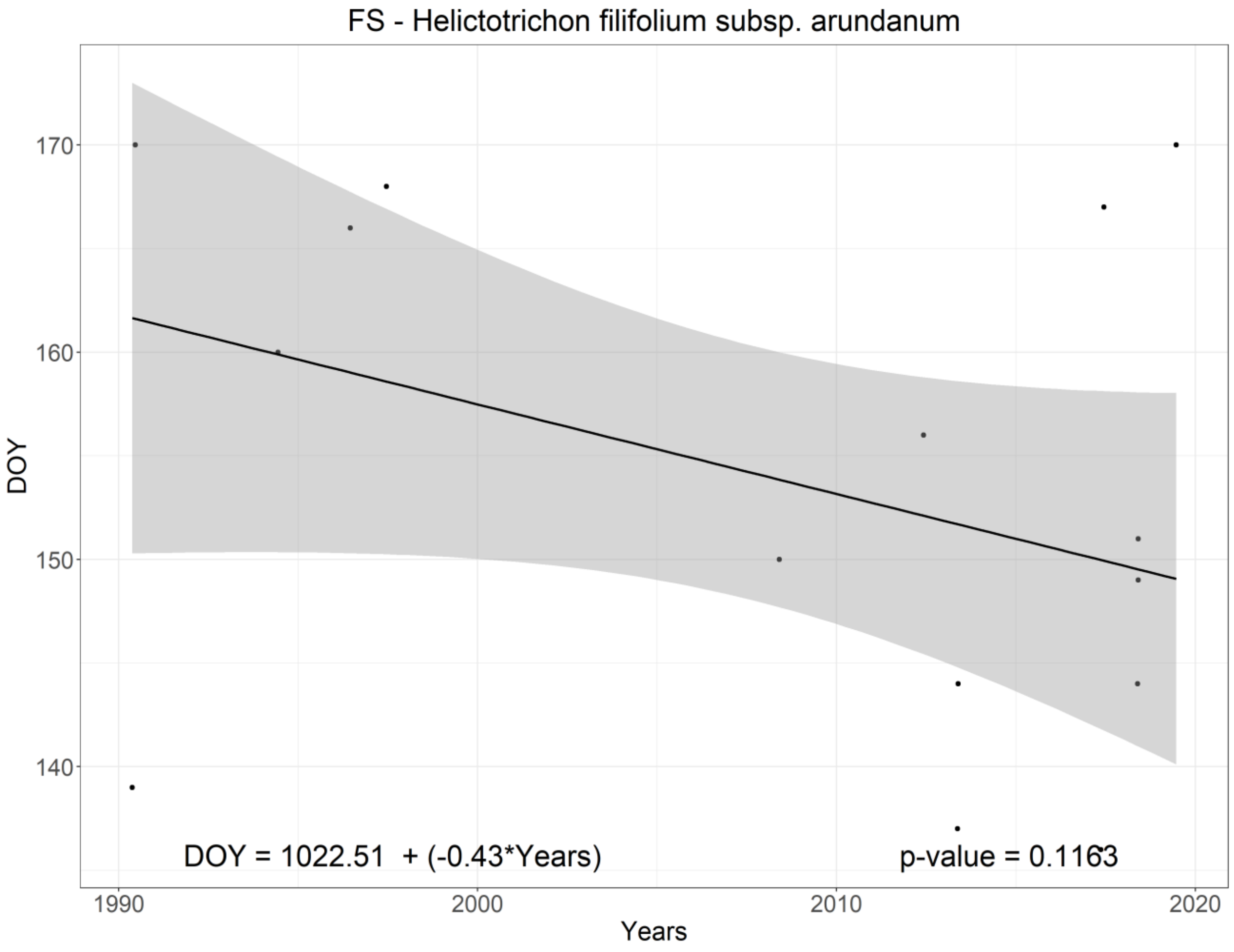

1.56.1. Diagnostics - LM - FS - Helictotrichon filifolium subsp. arundanum

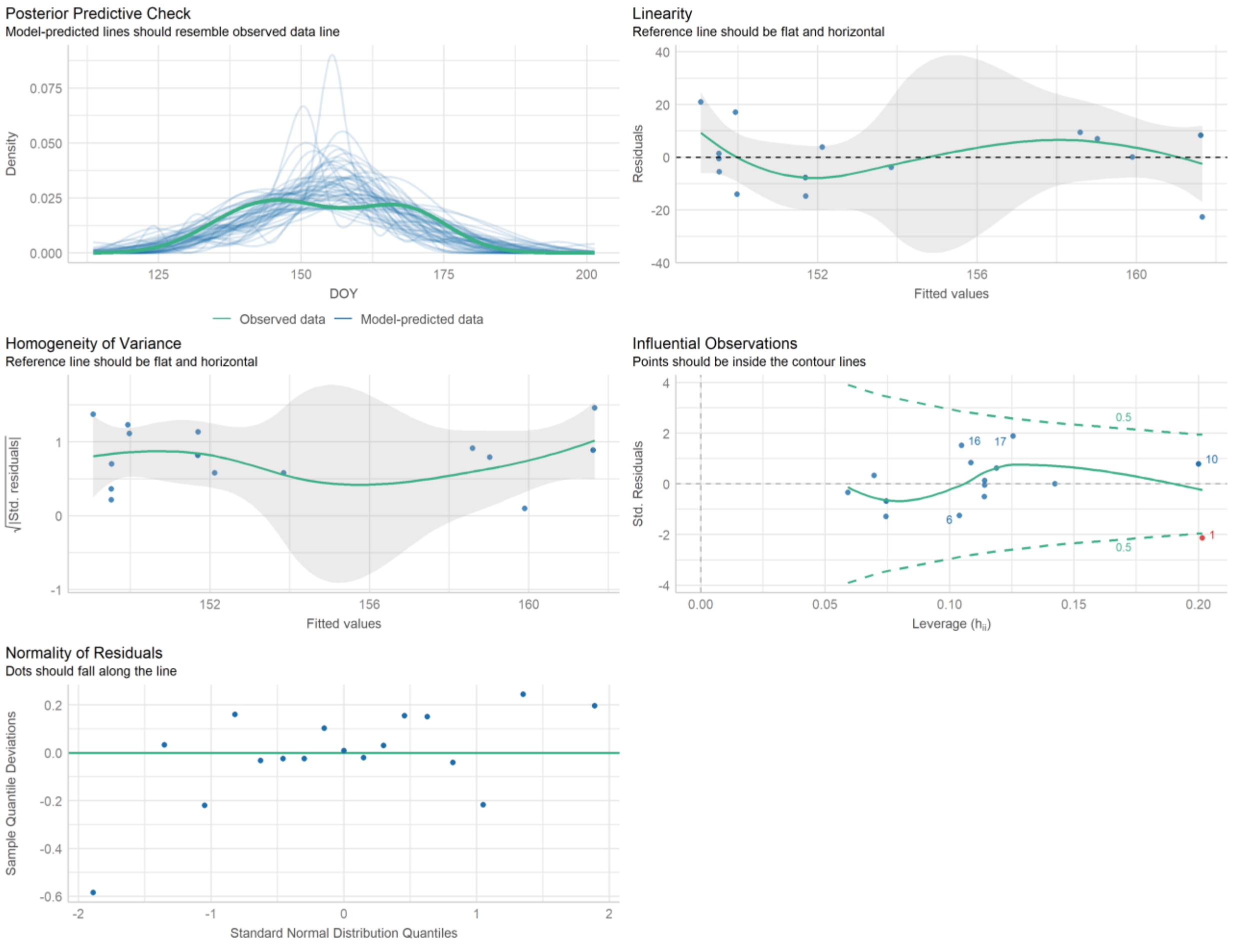

1.57. LM - FBF - Hormathophylla spinosa

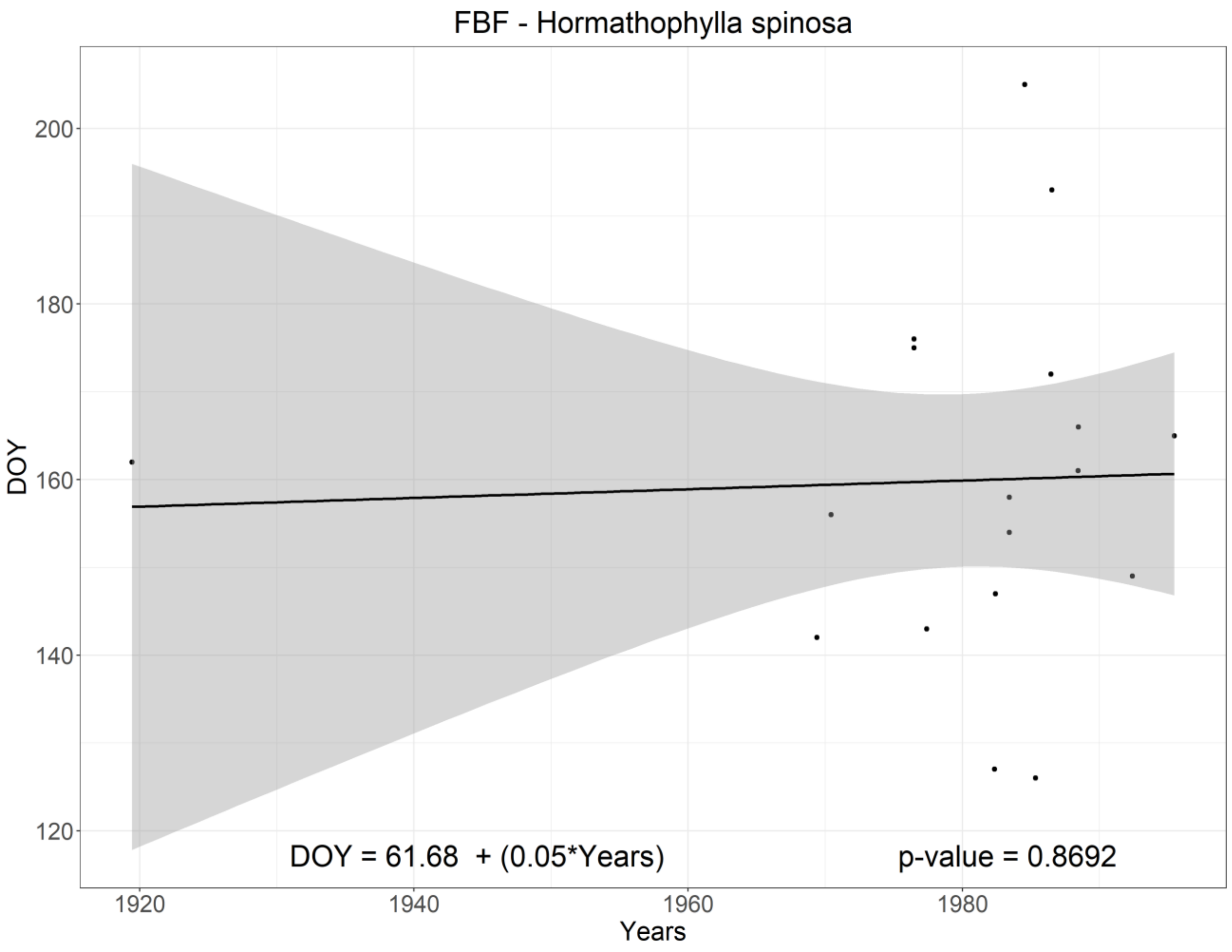

1.57.1. Diagnostics - LM - FBF - Hormathophylla spinosa

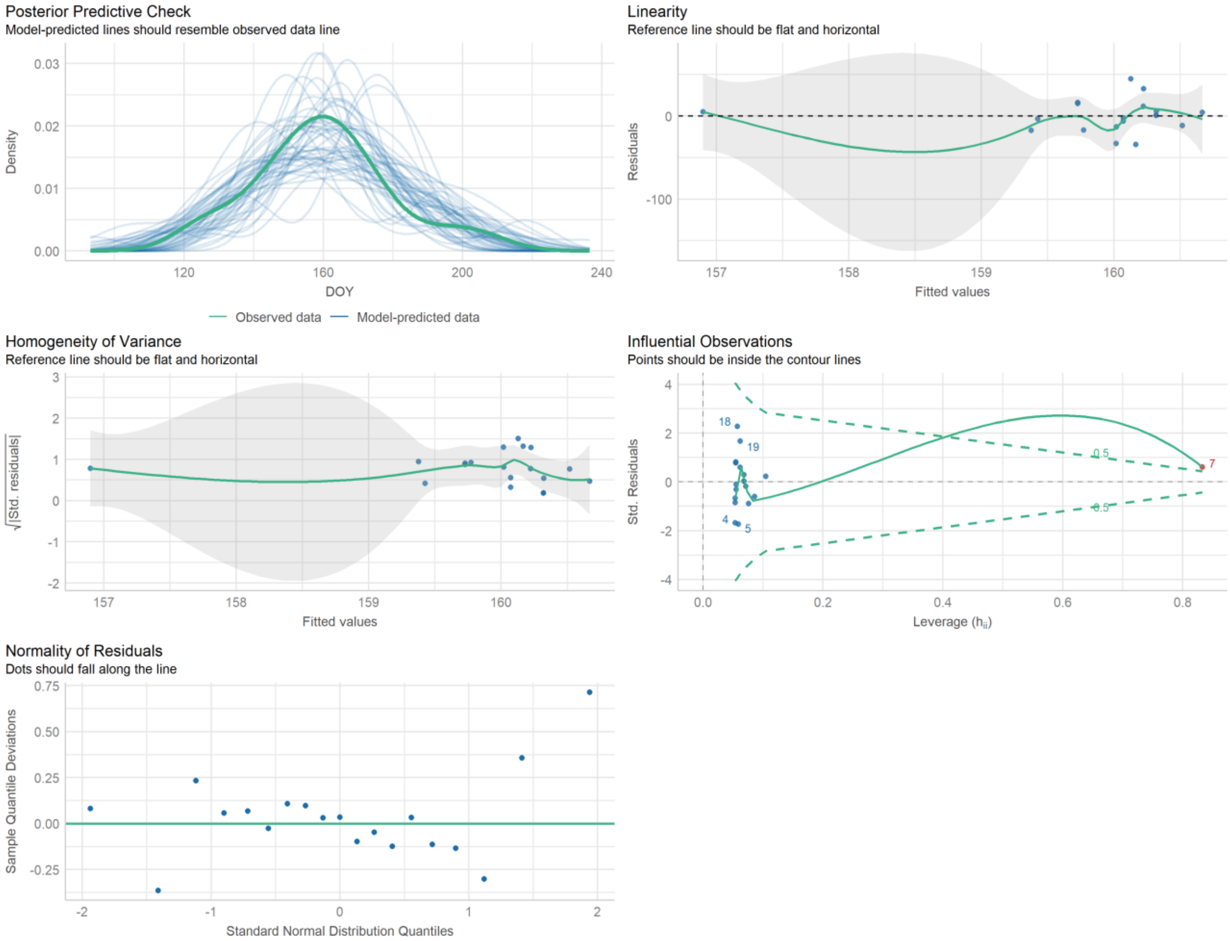

1.58. LM - F - Hormathophylla spinosa

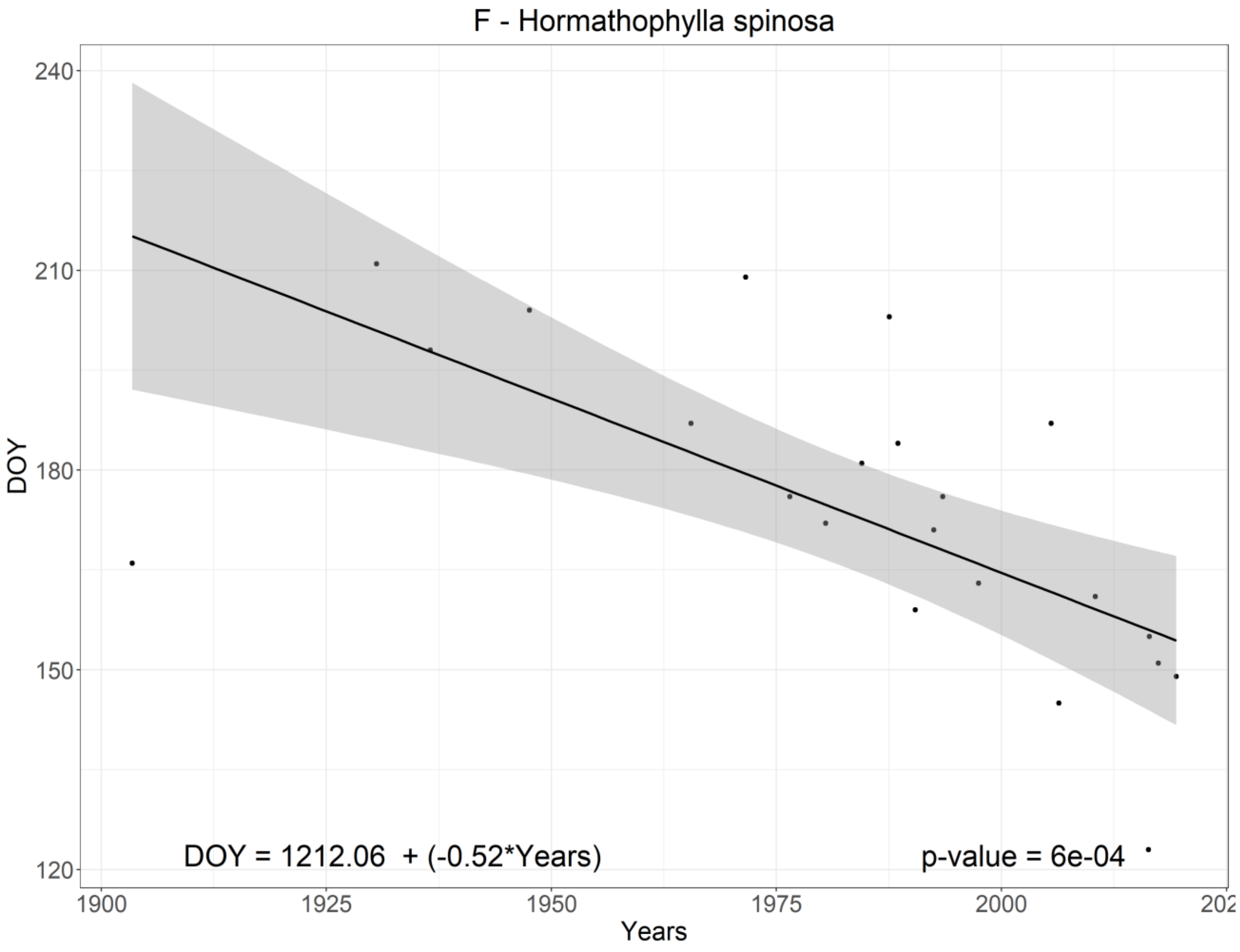

1.58.1. Diagnostics - LM - F - Hormathophylla spinosa

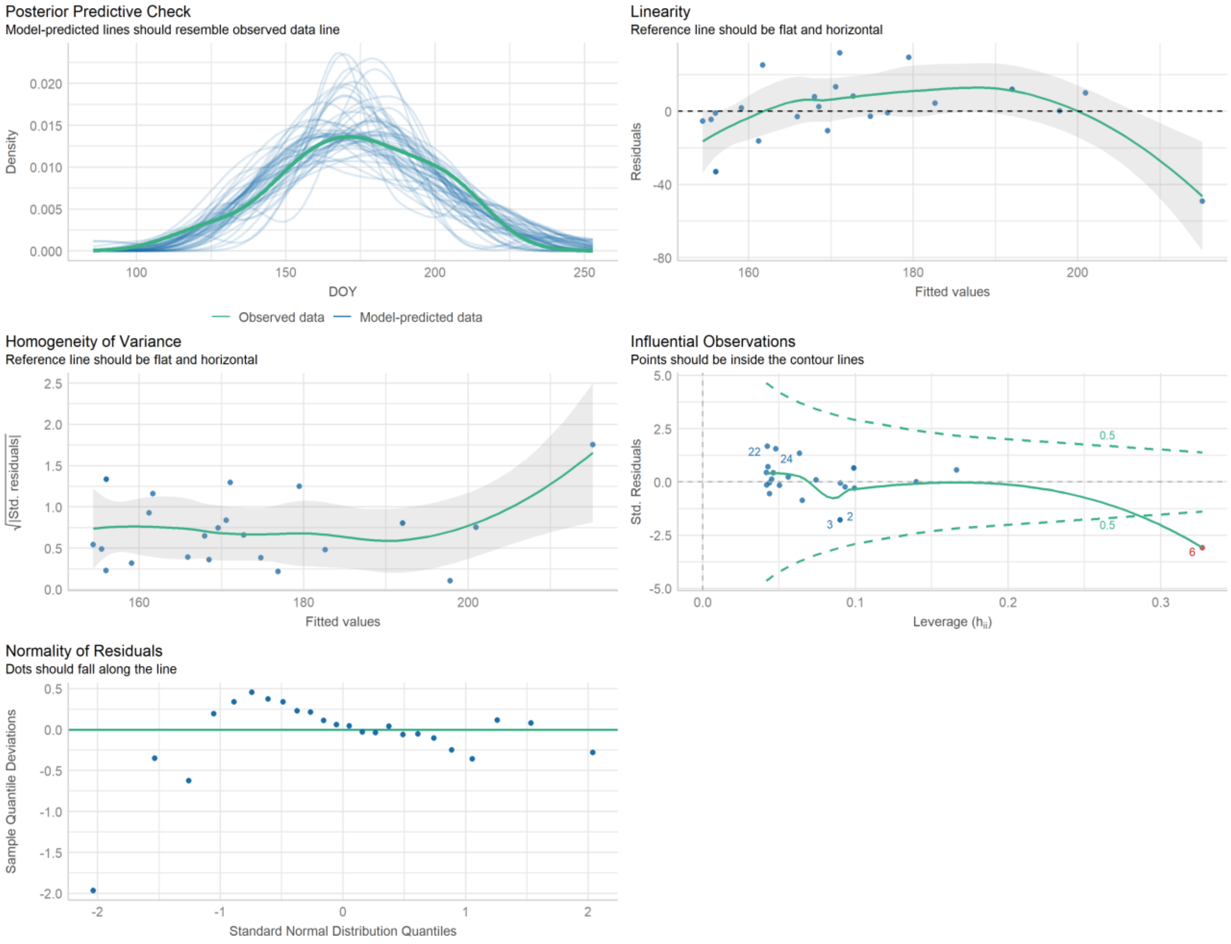

1.59. LM - FS - Hormathophylla spinosa

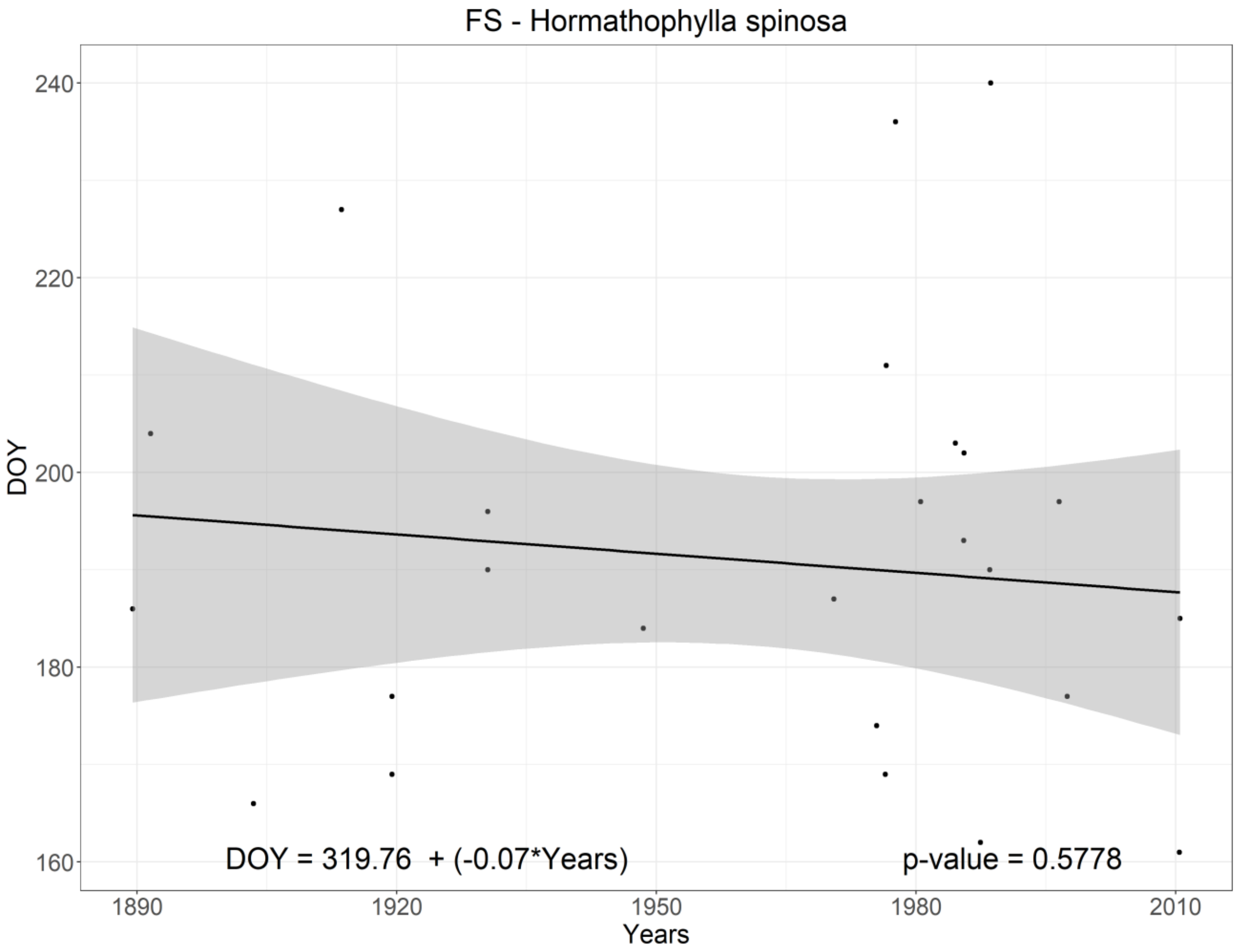

1.59.1. Diagnostics - LM - FS - Hormathophylla spinosa

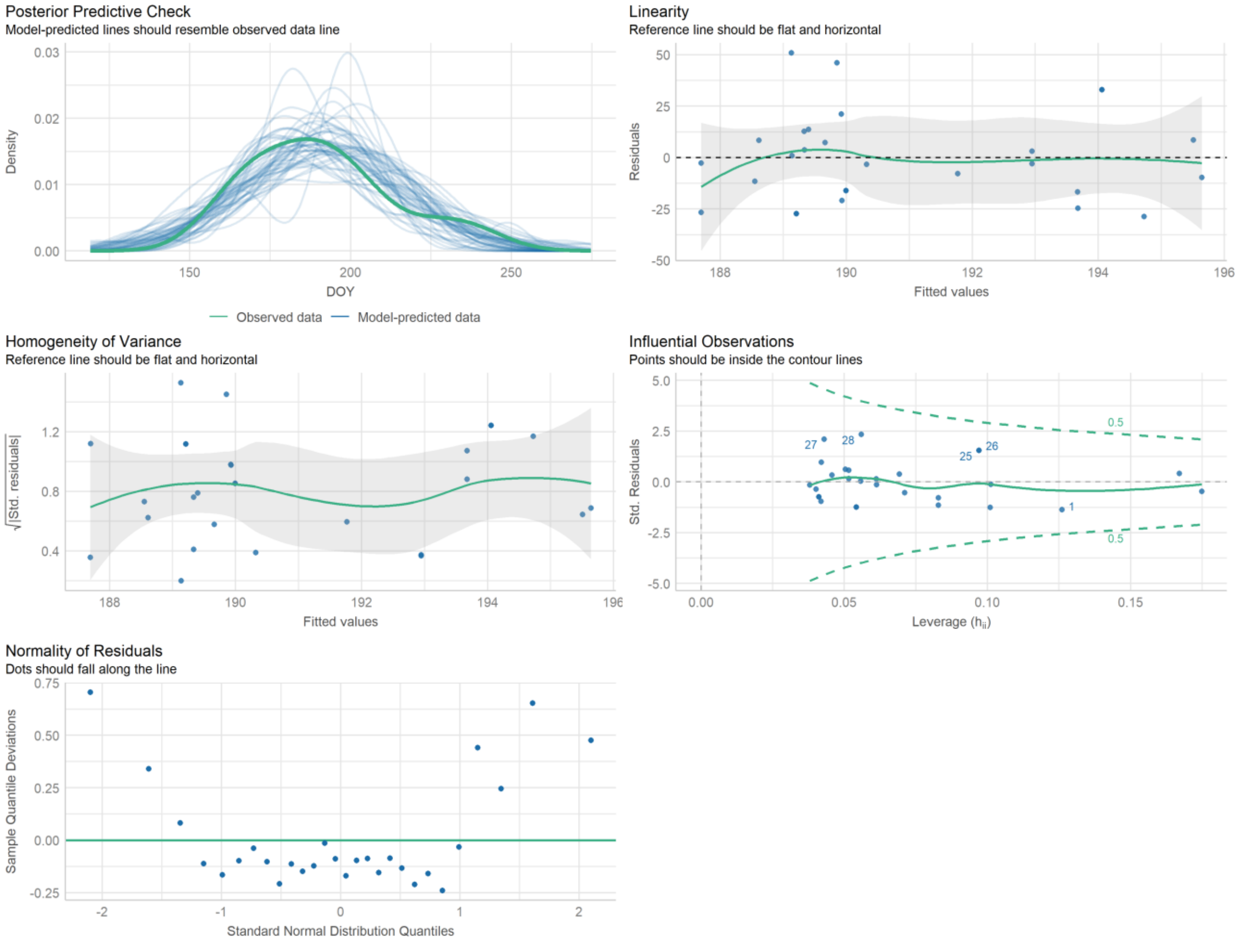

1.60. LM - FBF - Juniperus oxycedrus

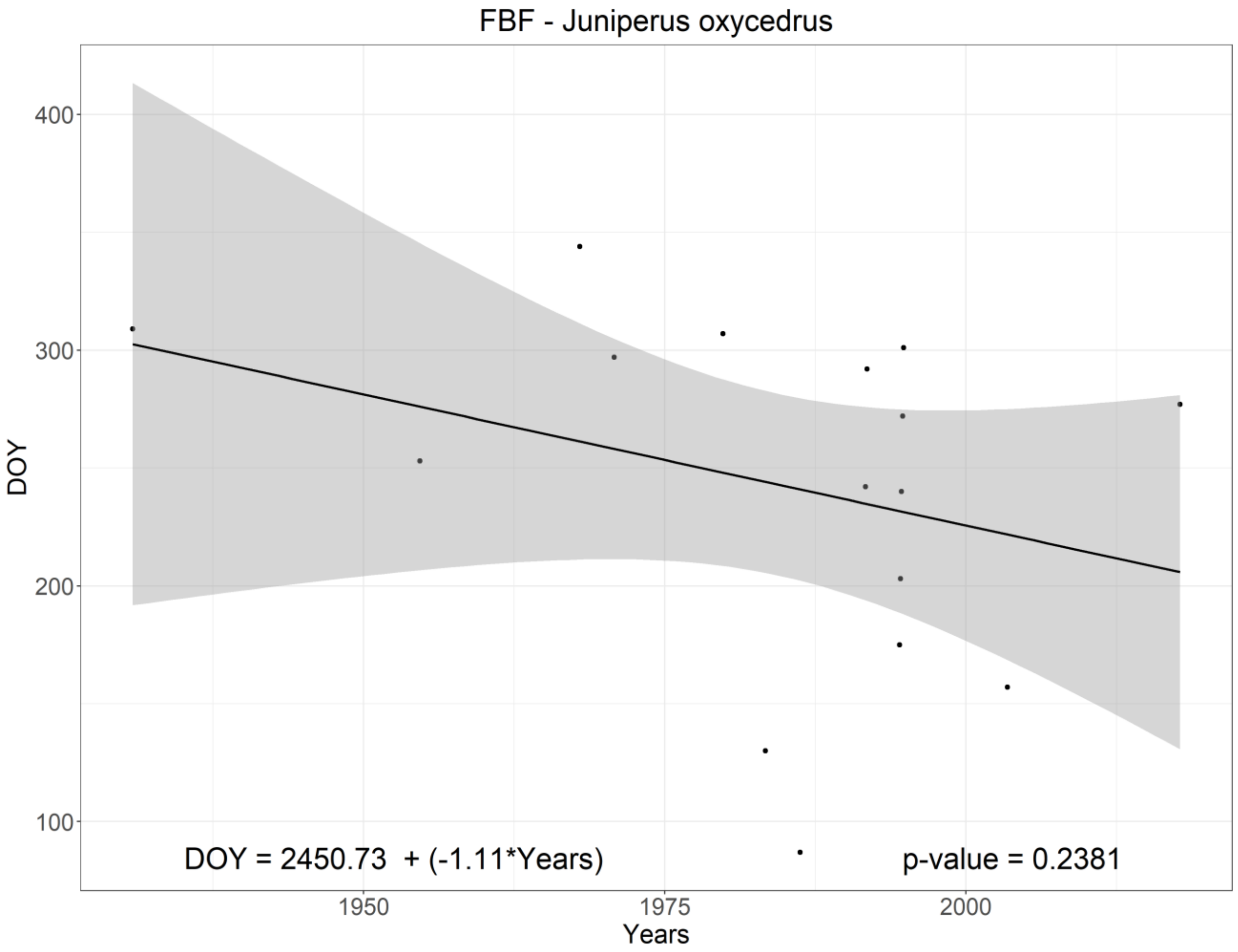

1.60.1. Diagnostics - LM - FBF - Juniperus oxycedrus

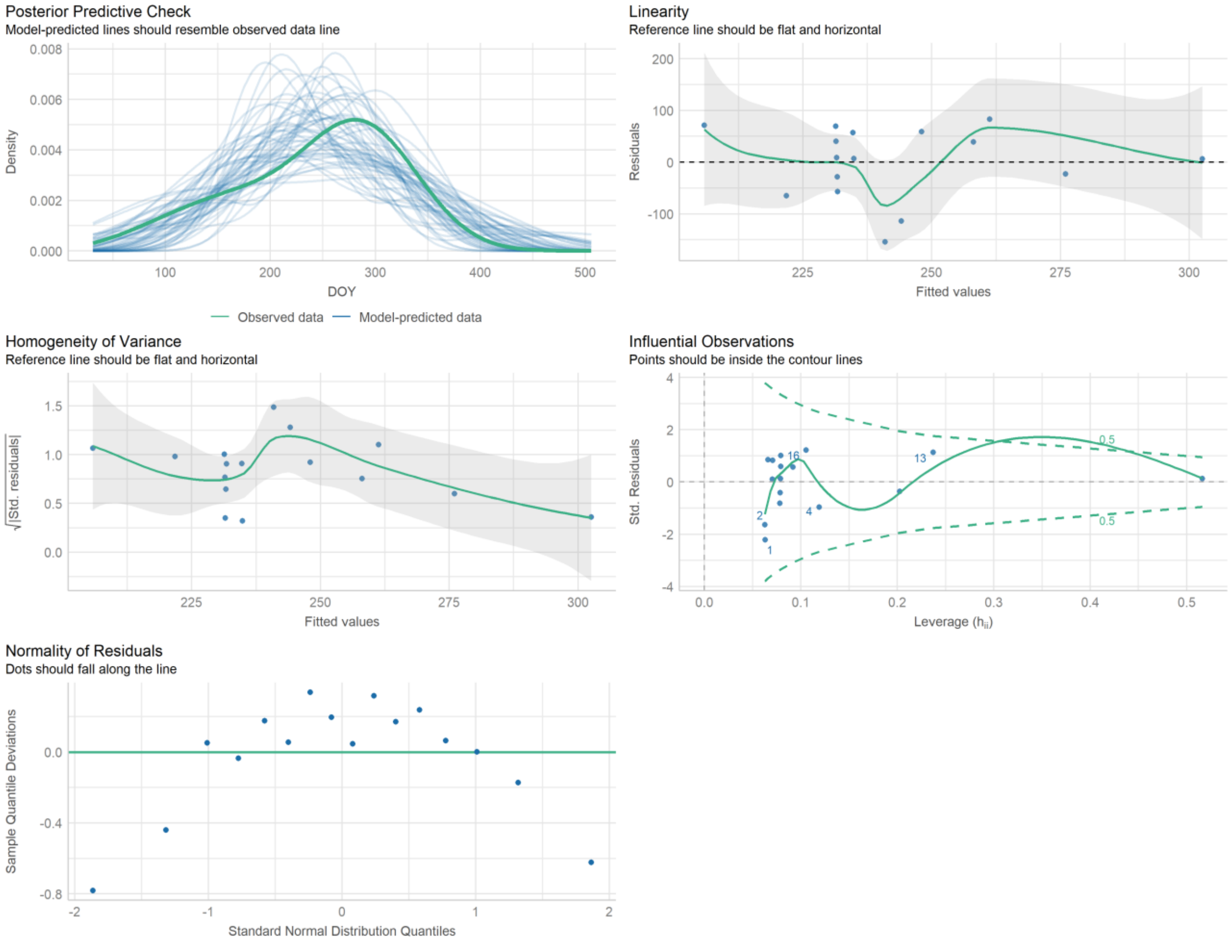

1.61. LM - F - Juniperus oxycedrus

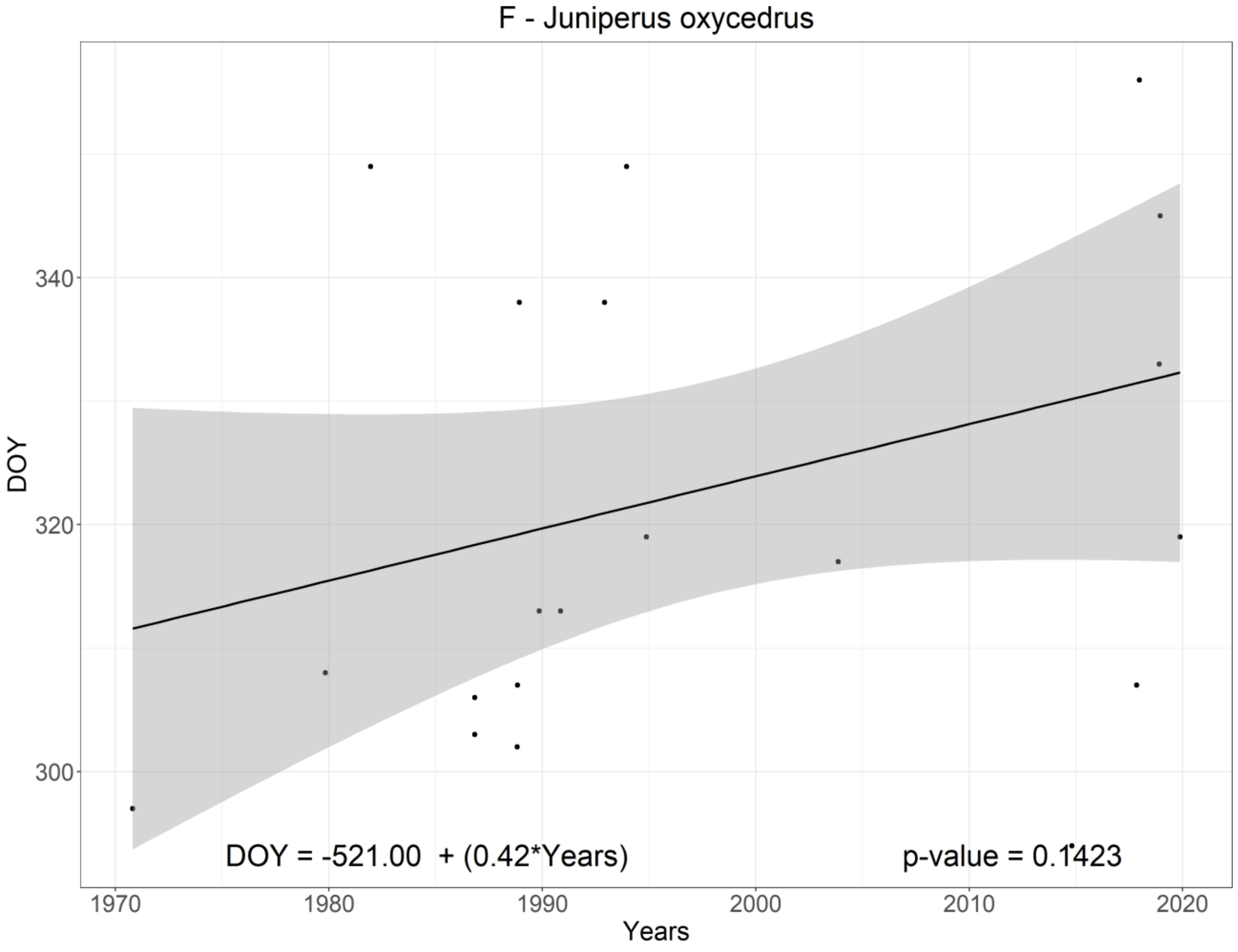

1.61.1. Diagnostics - LM - F - Juniperus oxycedrus

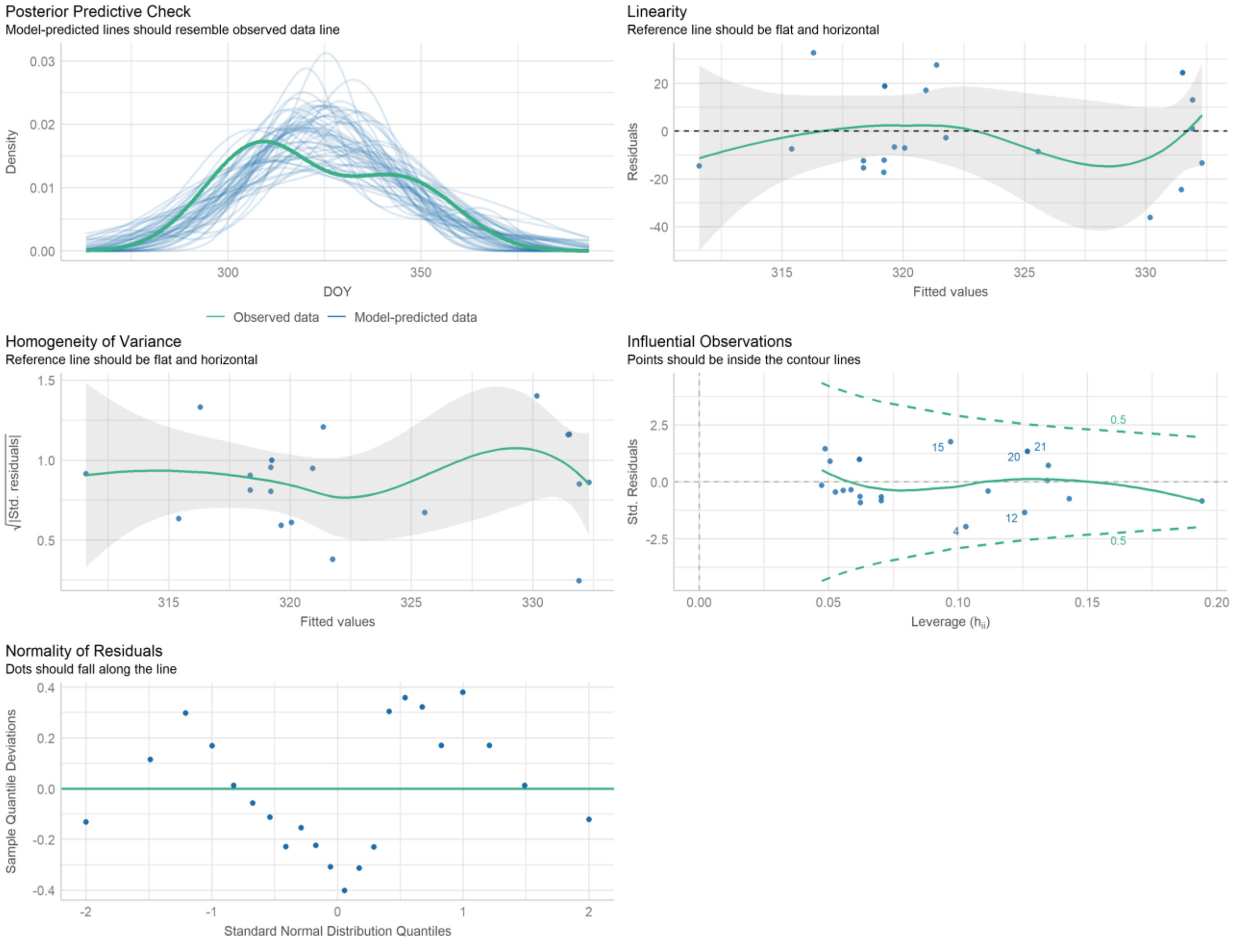

1.62. LM - DVG - Juniperus oxycedrus

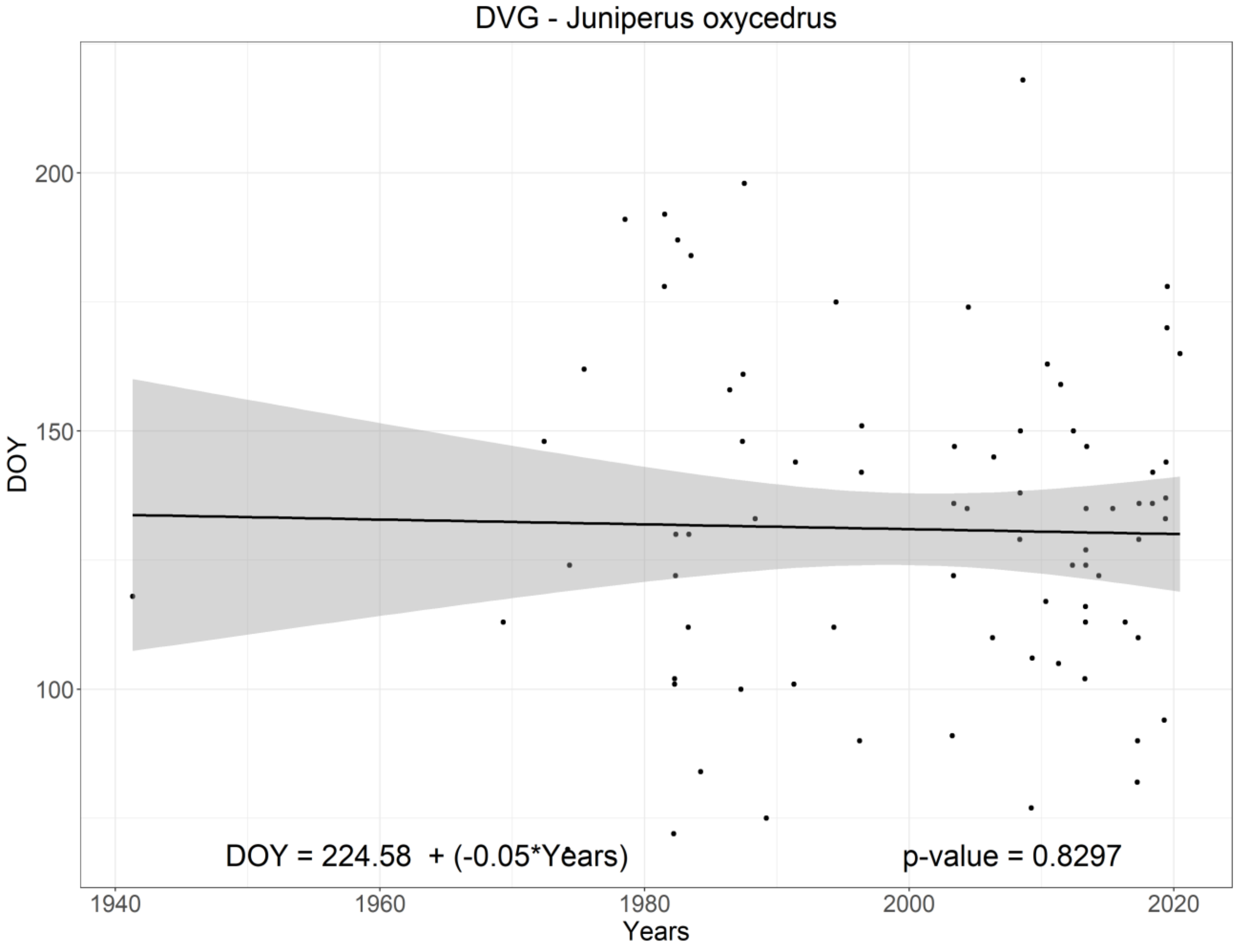

1.62.1. Diagnostics - LM - DVG - Juniperus oxycedrus

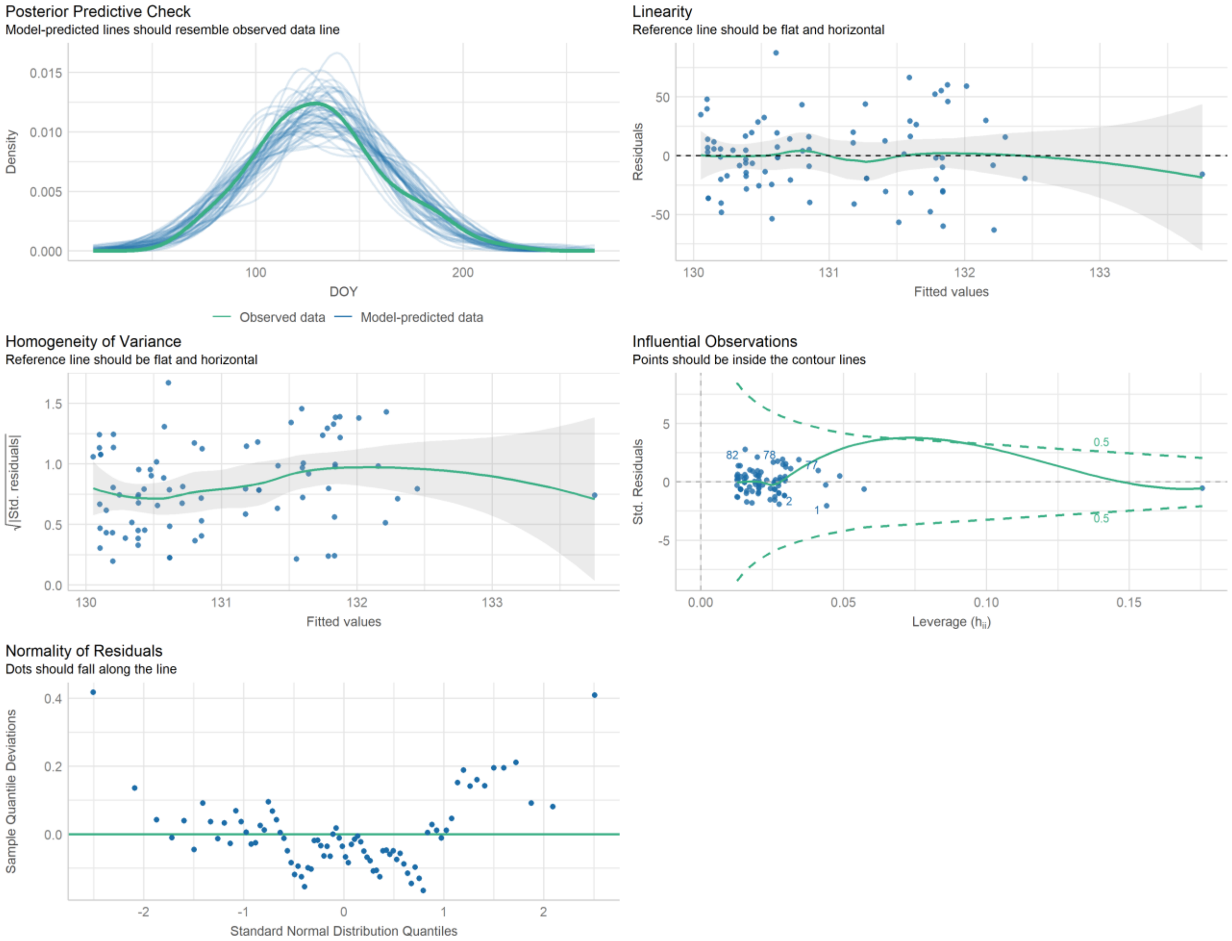

1.63. LM - DVG - Juniperus turbinata

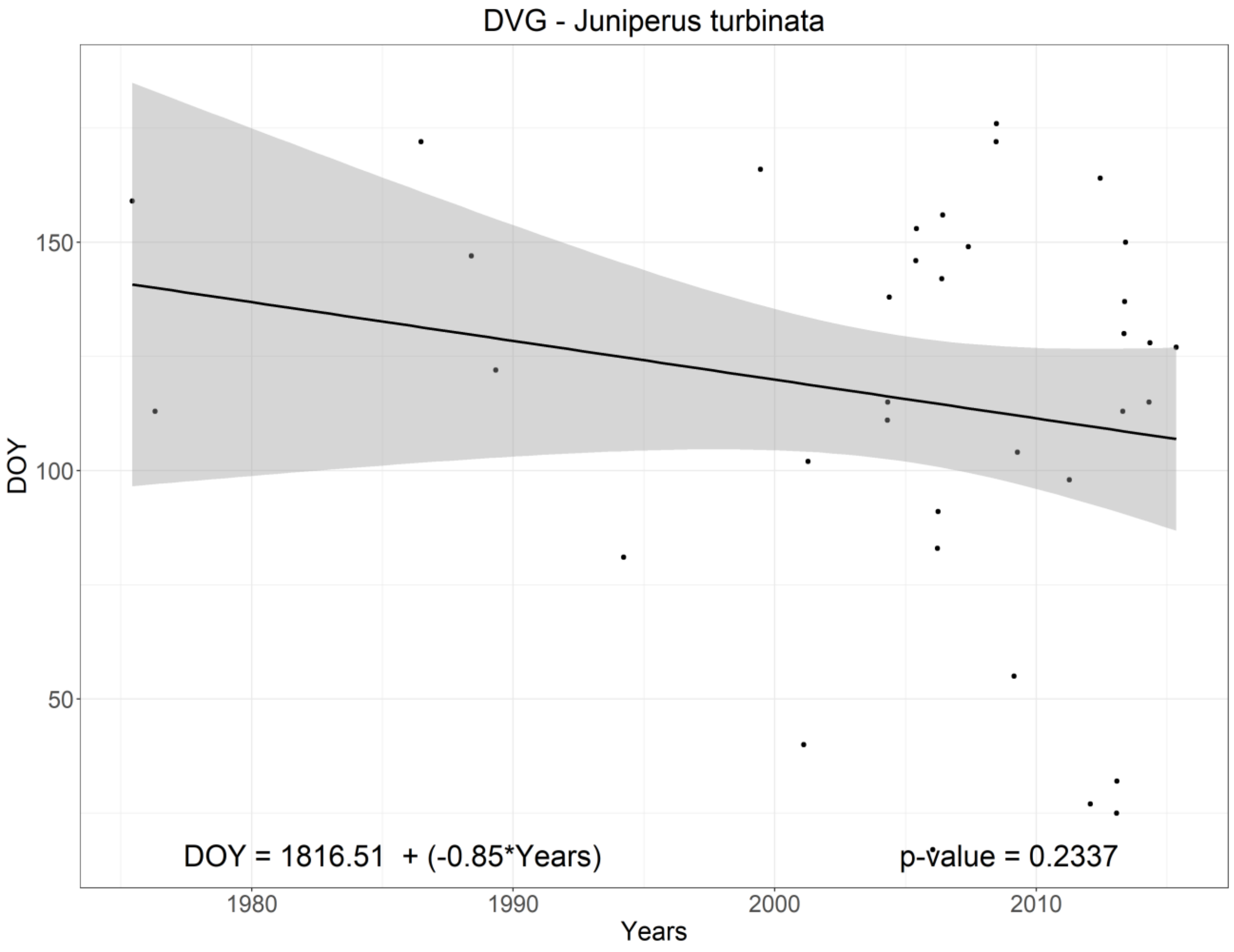

1.63.1. Diagnostics - LM - DVG - Juniperus turbinata

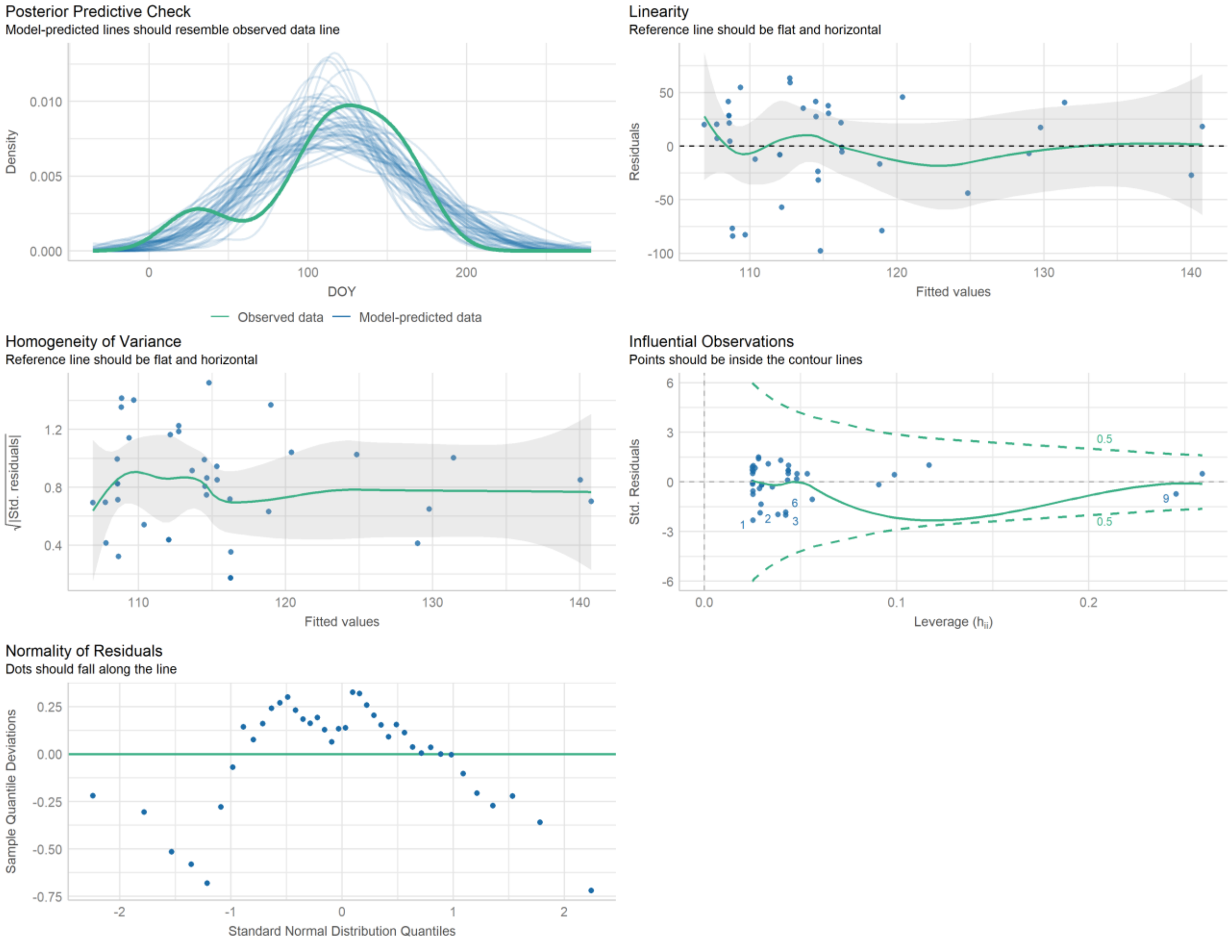

1.64. LM - F - Klasea baetica

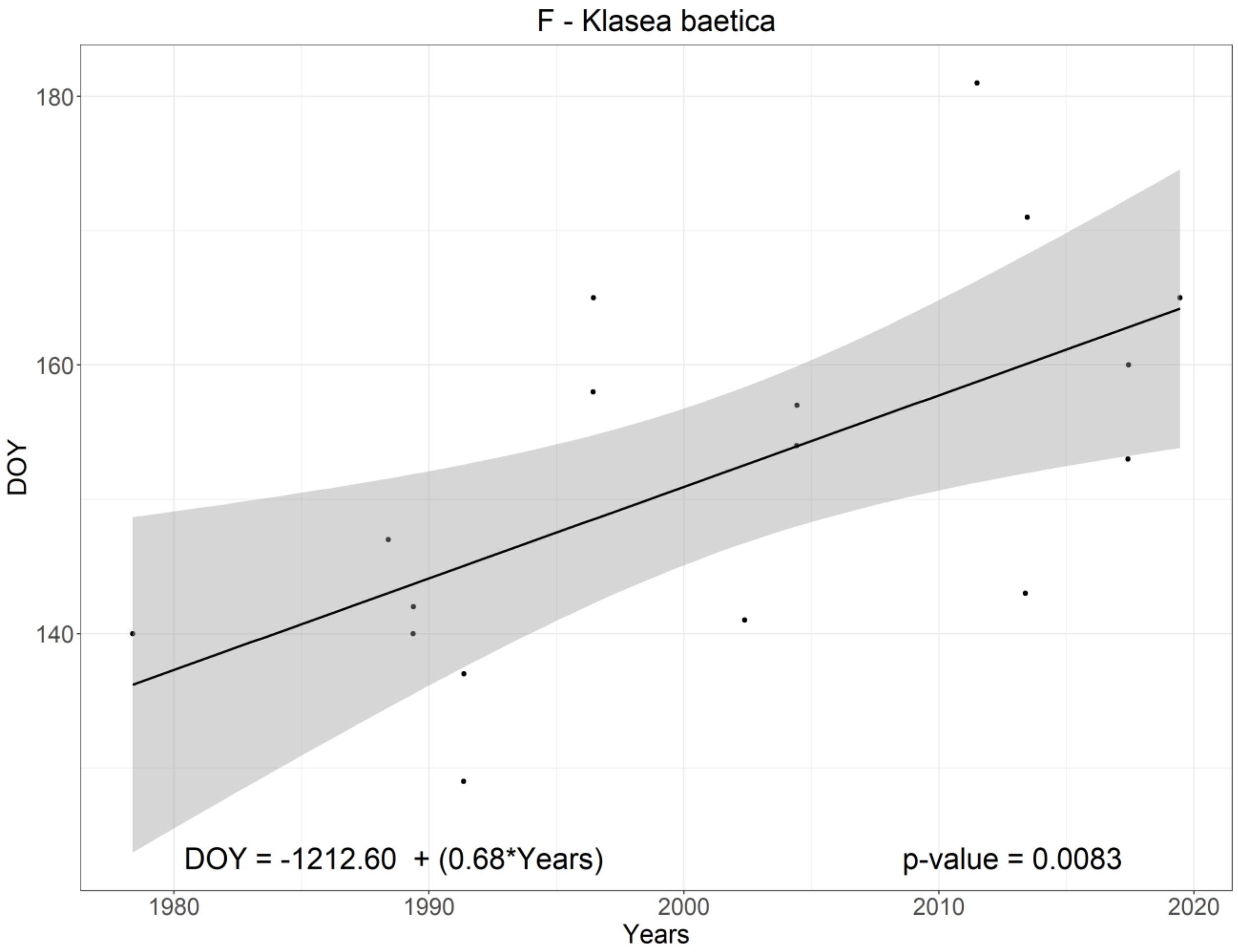

1.64.1. Diagnostics - LM - F - Klasea baetica

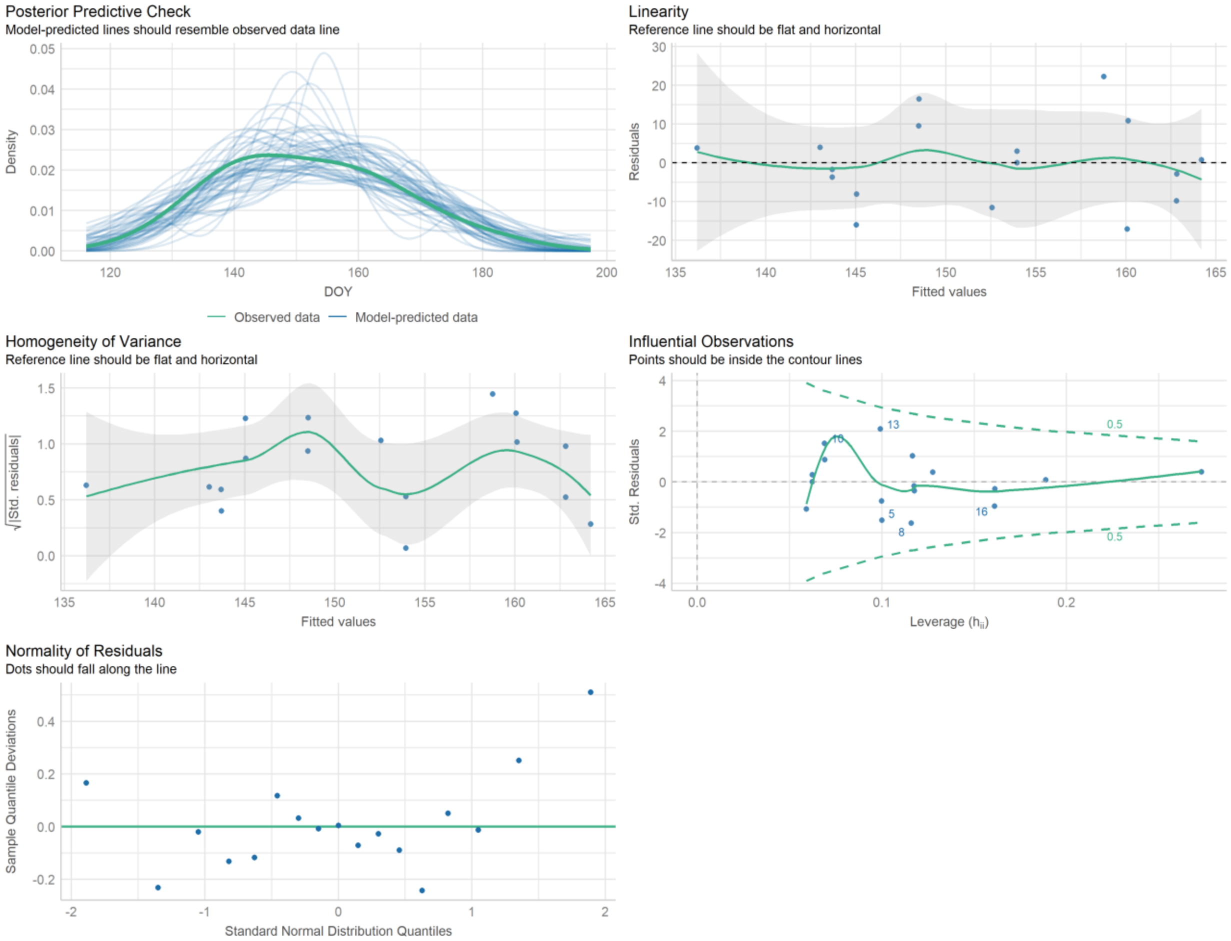

1.65. LM - FBF - Lavandula lanata

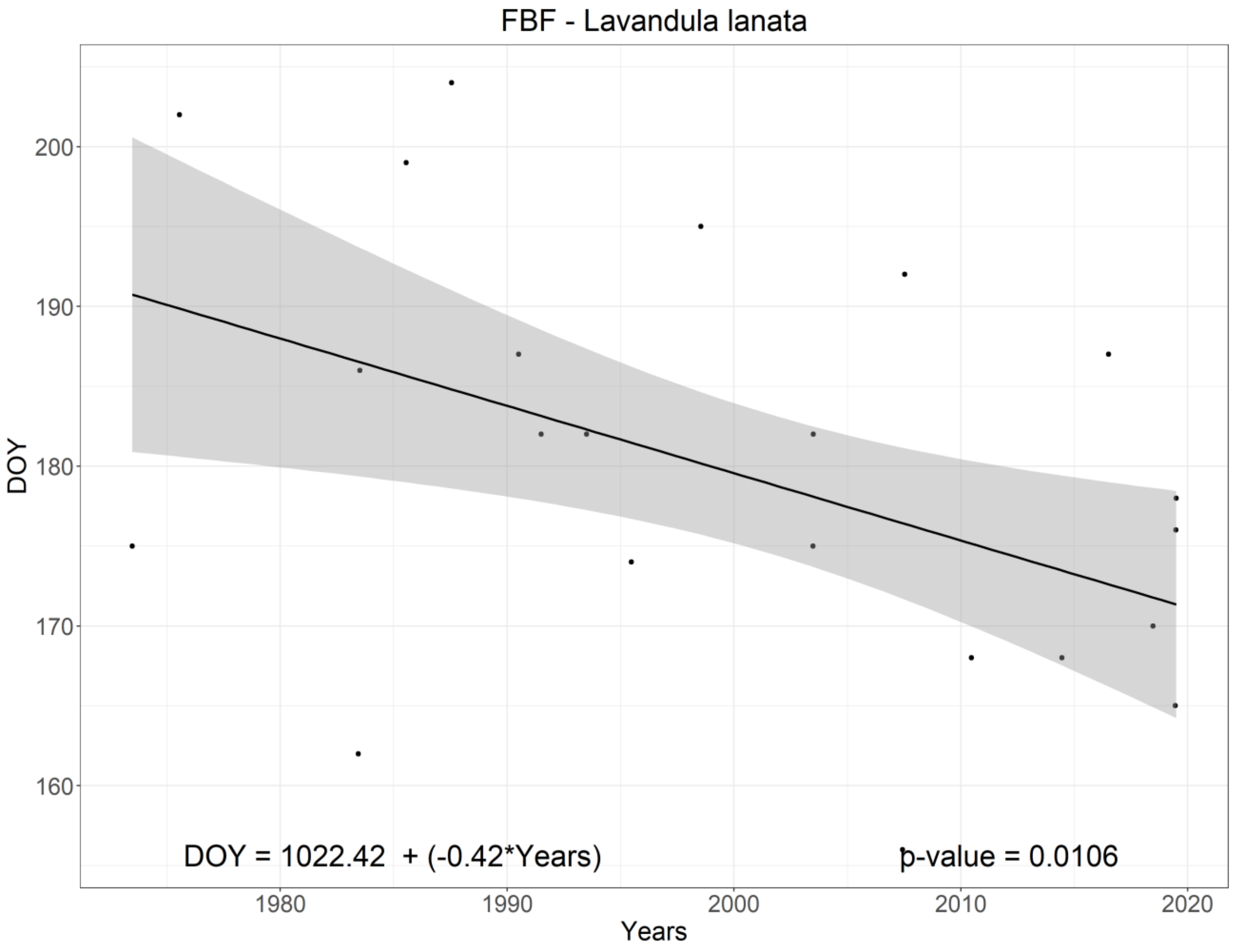

1.65.1. Diagnostics - LM - FBF - Lavandula lanata

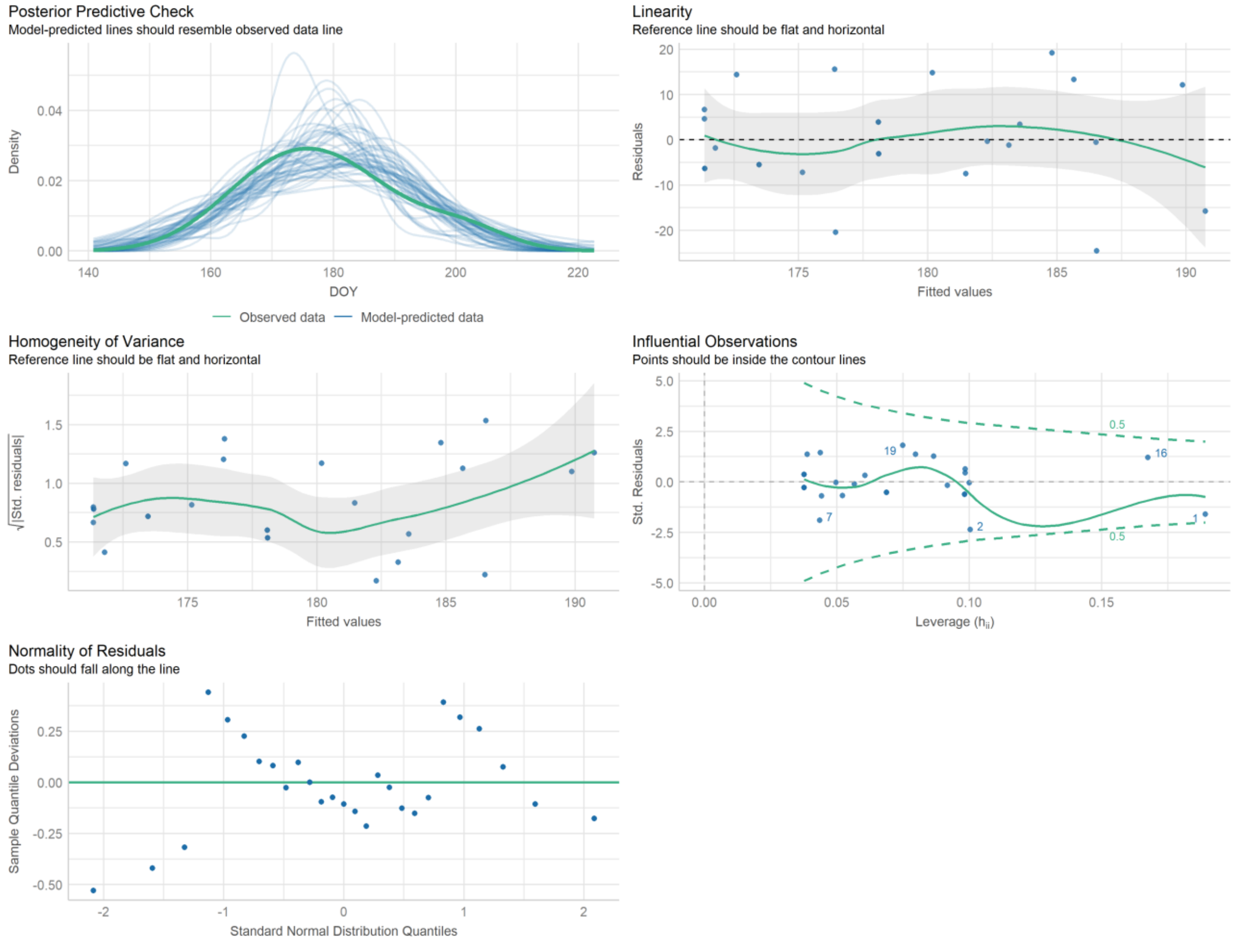

1.66. LM - DVG - Lavandula lanata

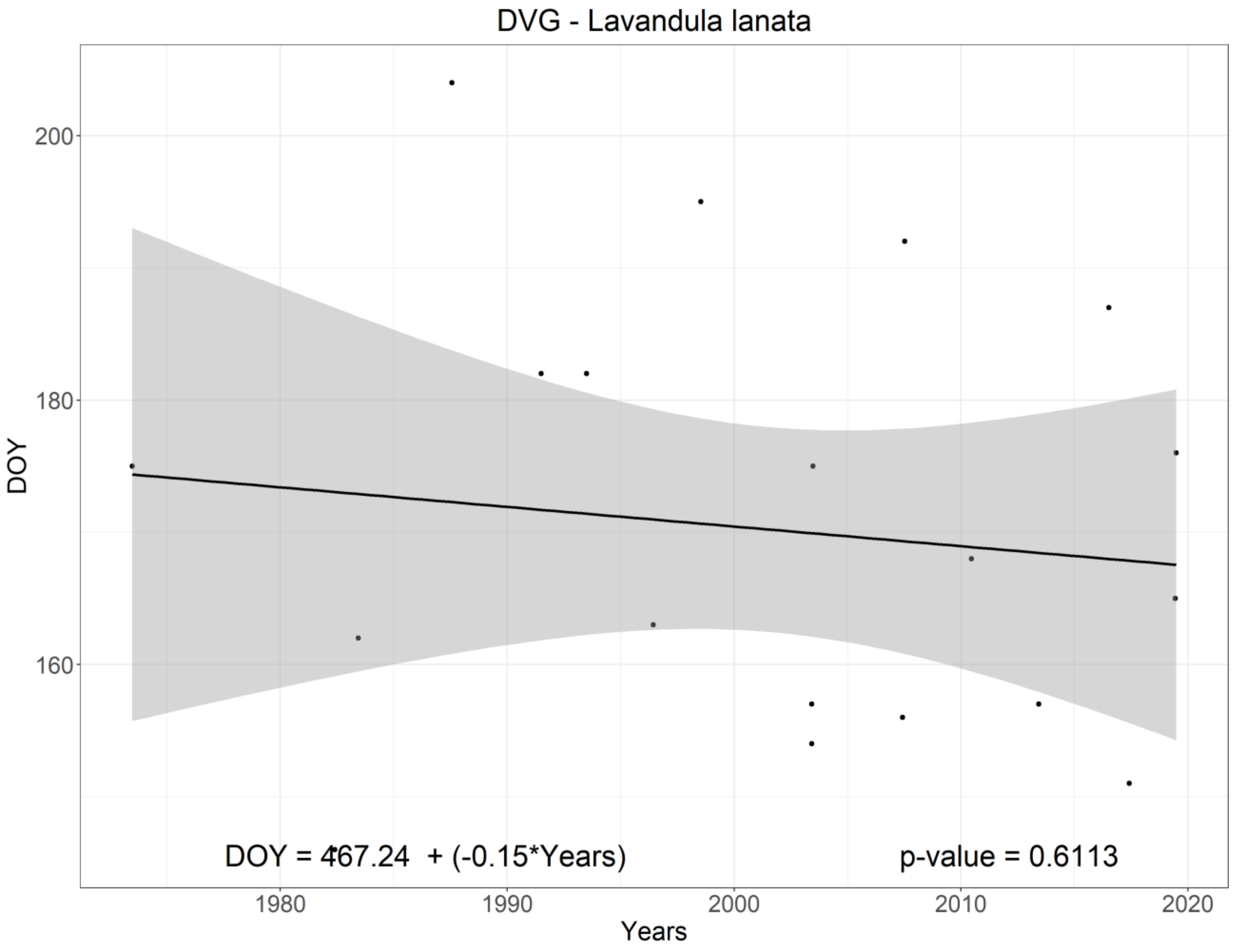

1.66.1. Diagnostics - LM - DVG - Lavandula lanata

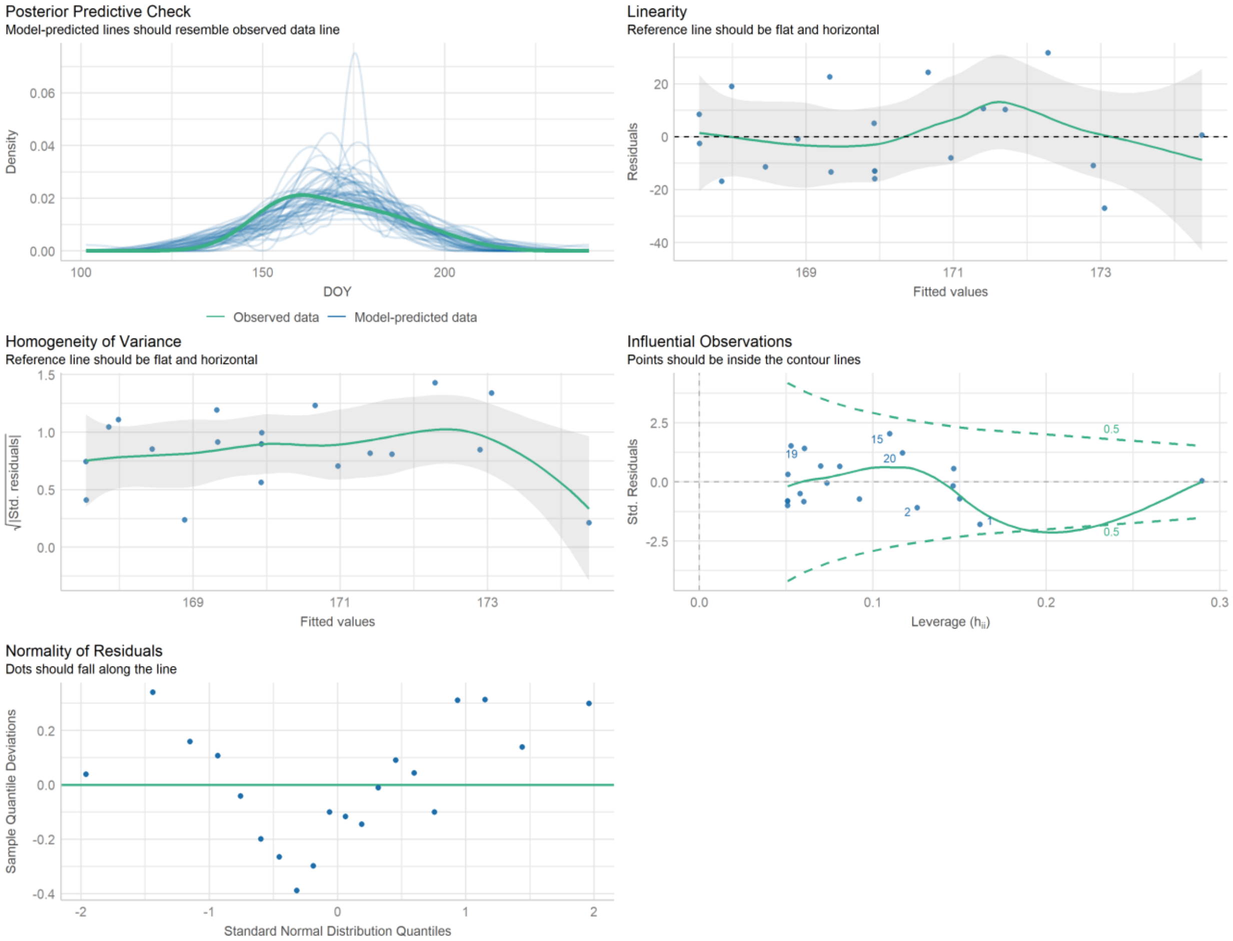

1.67. LM - FBF - Lavandula stoechas

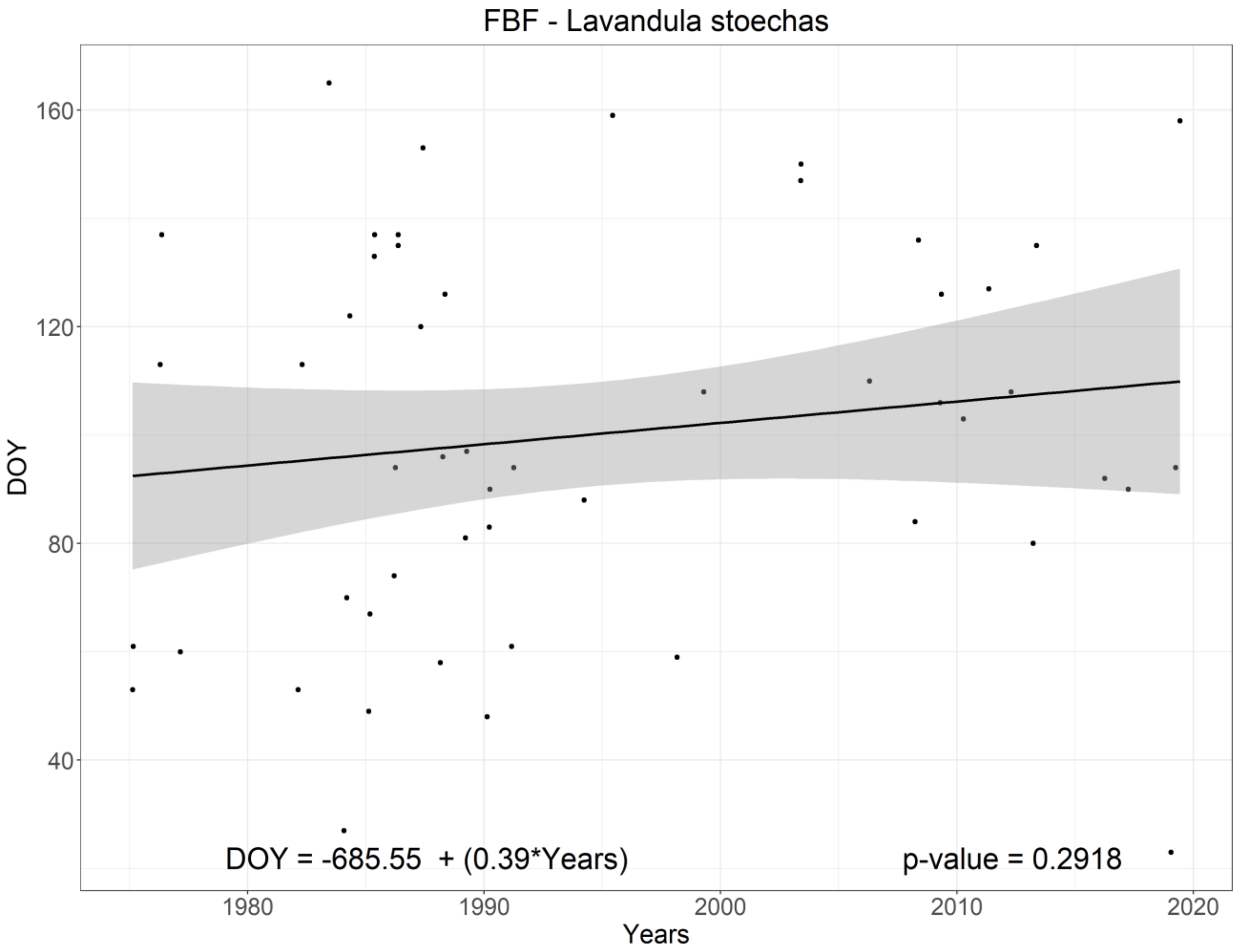

1.67.1. Diagnostics - LM - FBF - Lavandula stoechas

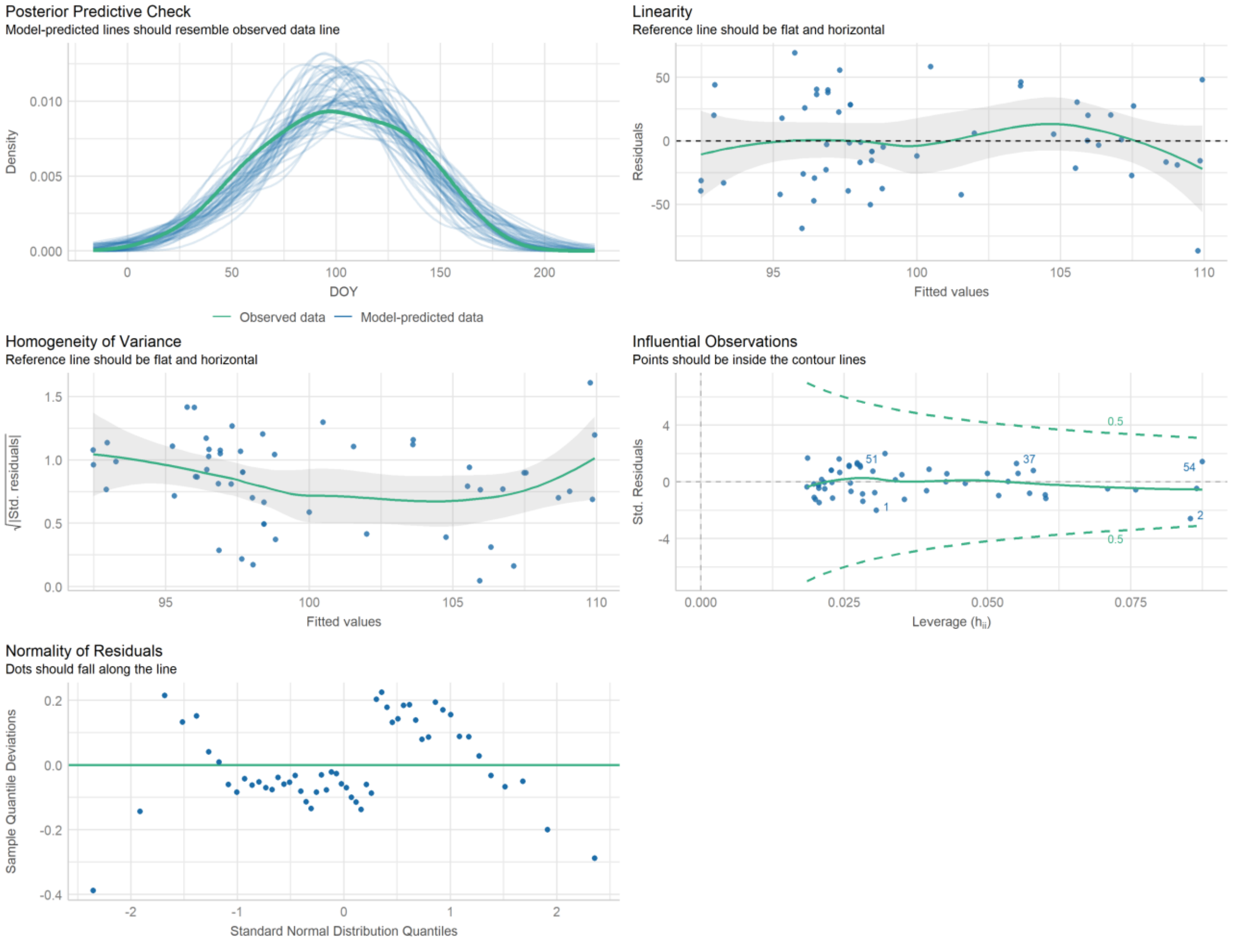

1.68. LM - F - Lavandula stoechas

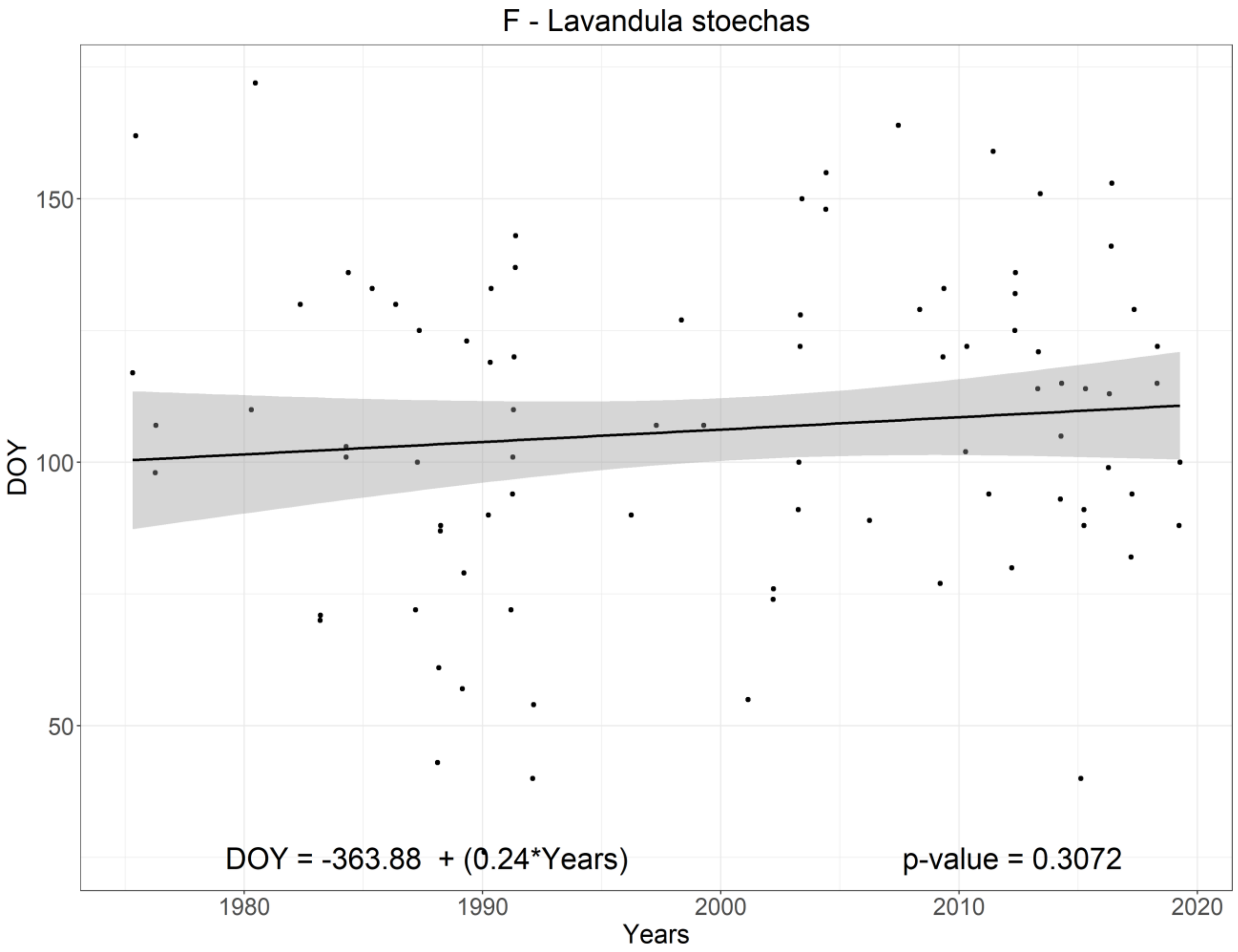

1.68.1. Diagnostics - LM - F - Lavandula stoechas

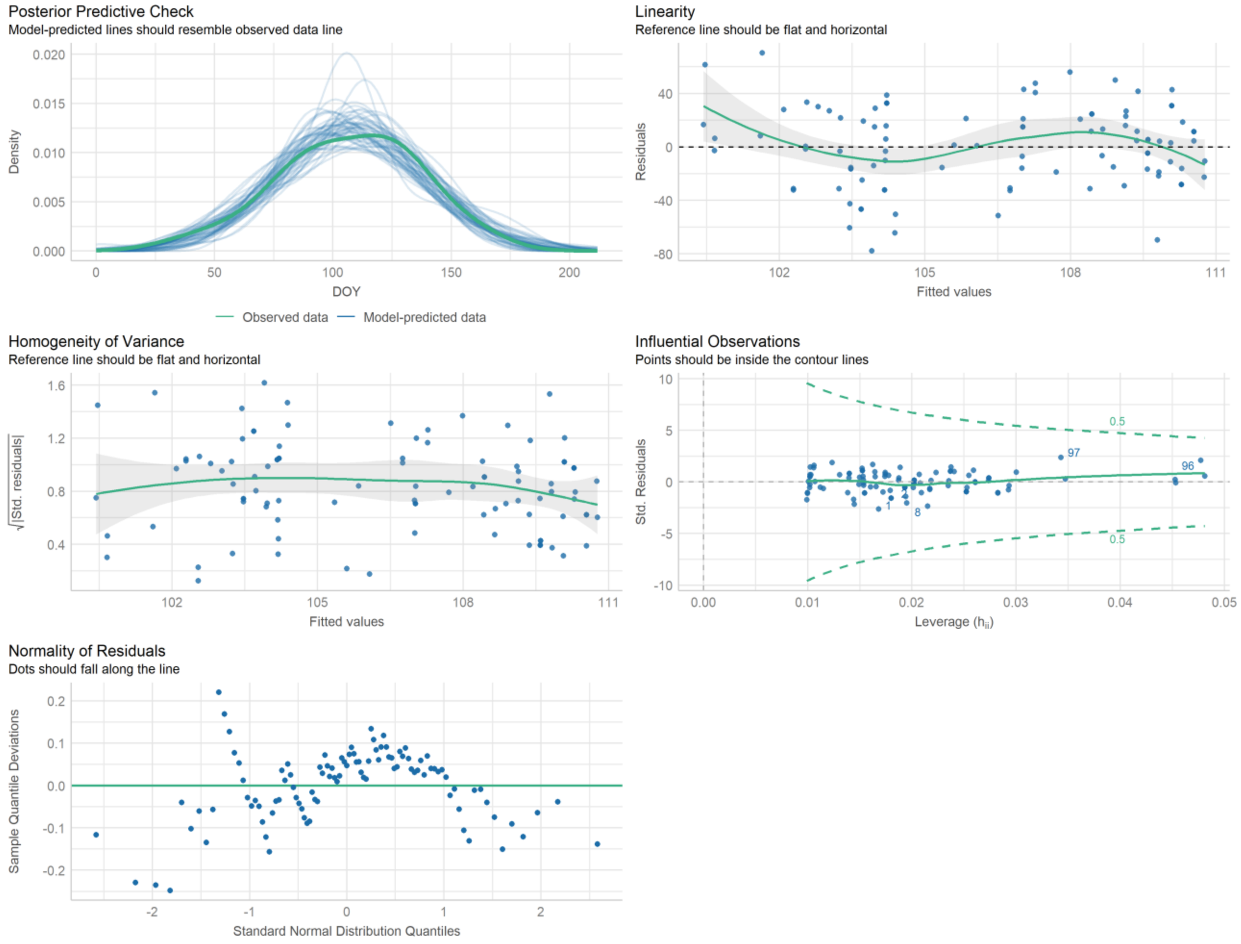

1.69. LM - F - Leontodon boryi

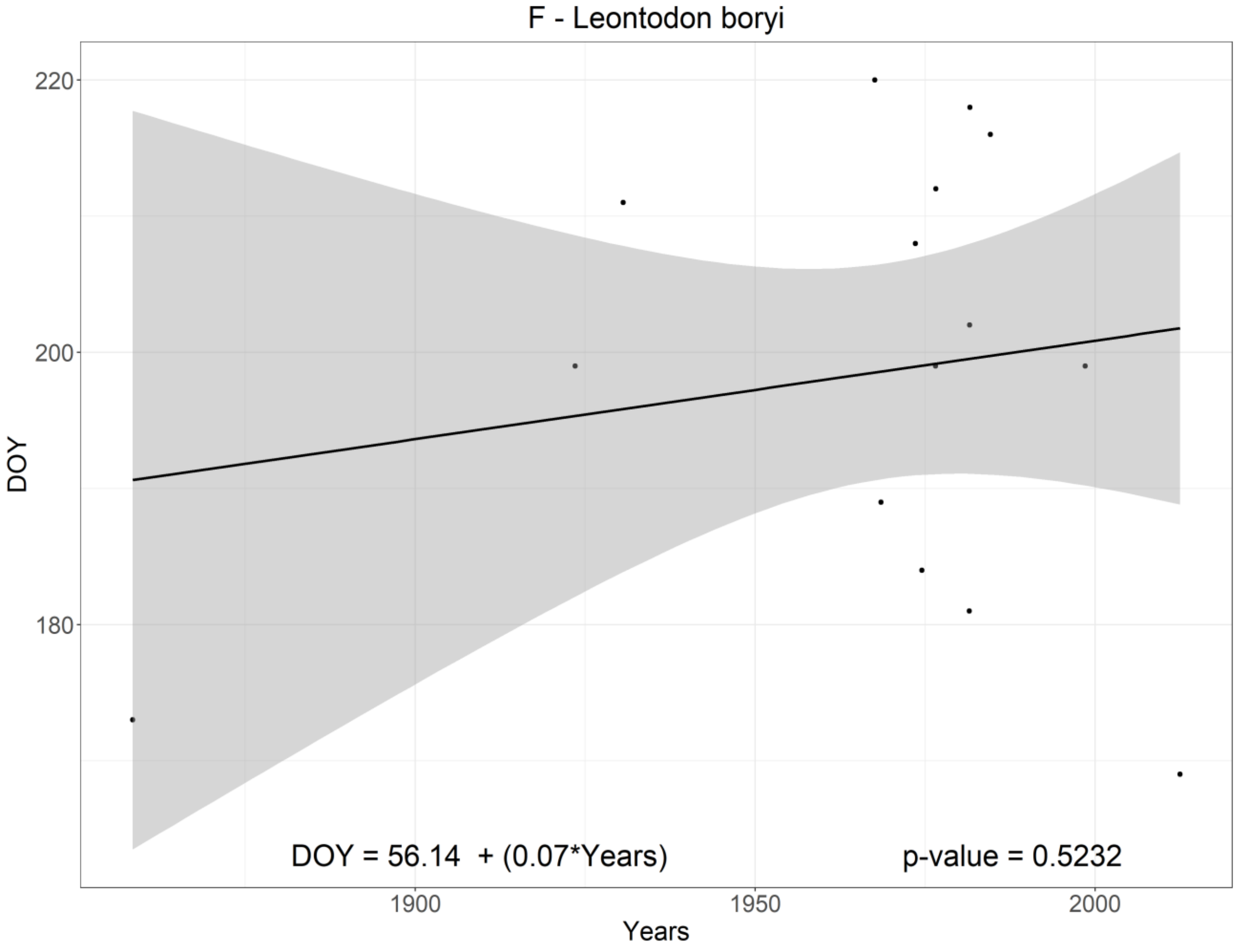

1.69.1. Diagnostics - LM - F - Leontodon boryi

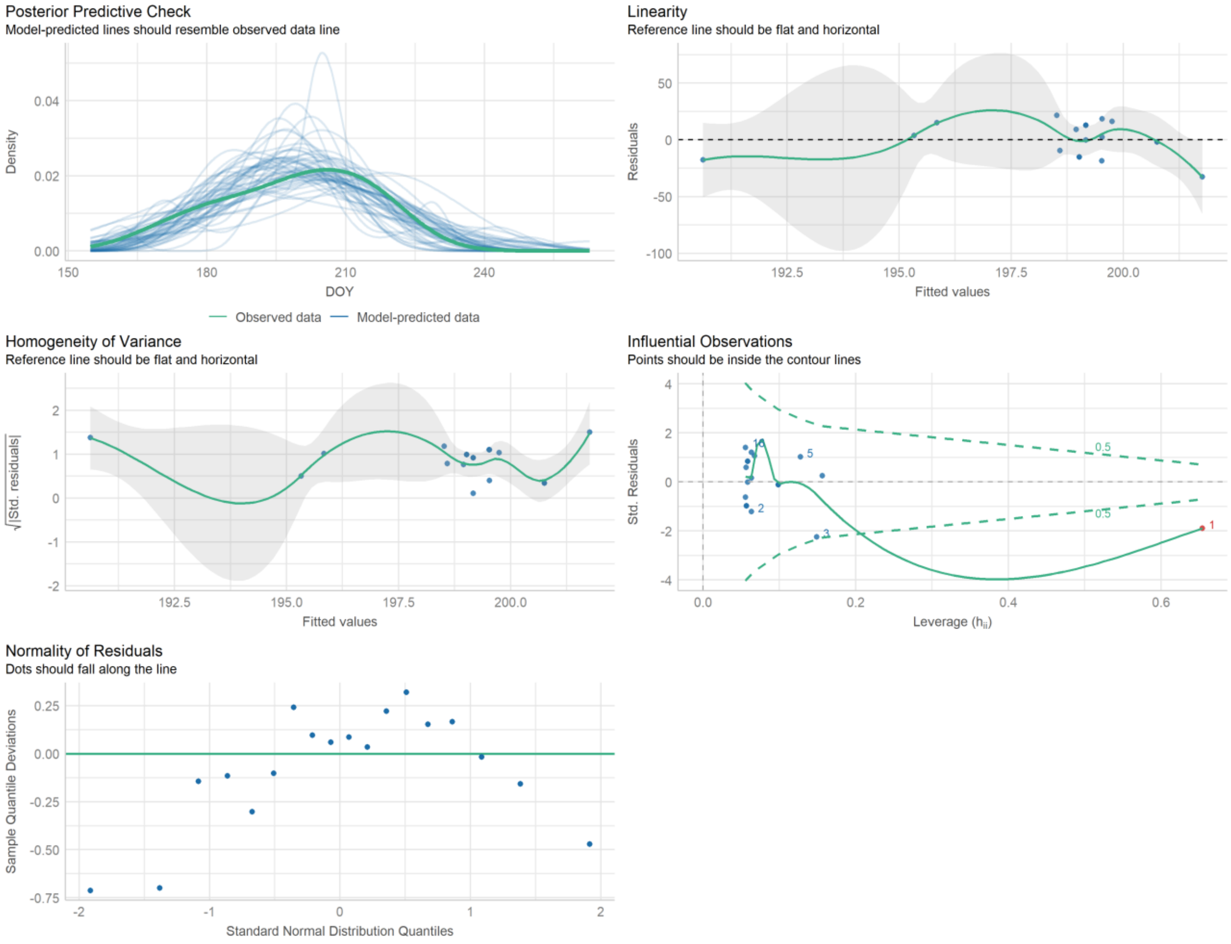

1.70. LM - F - Linaria saturejoides

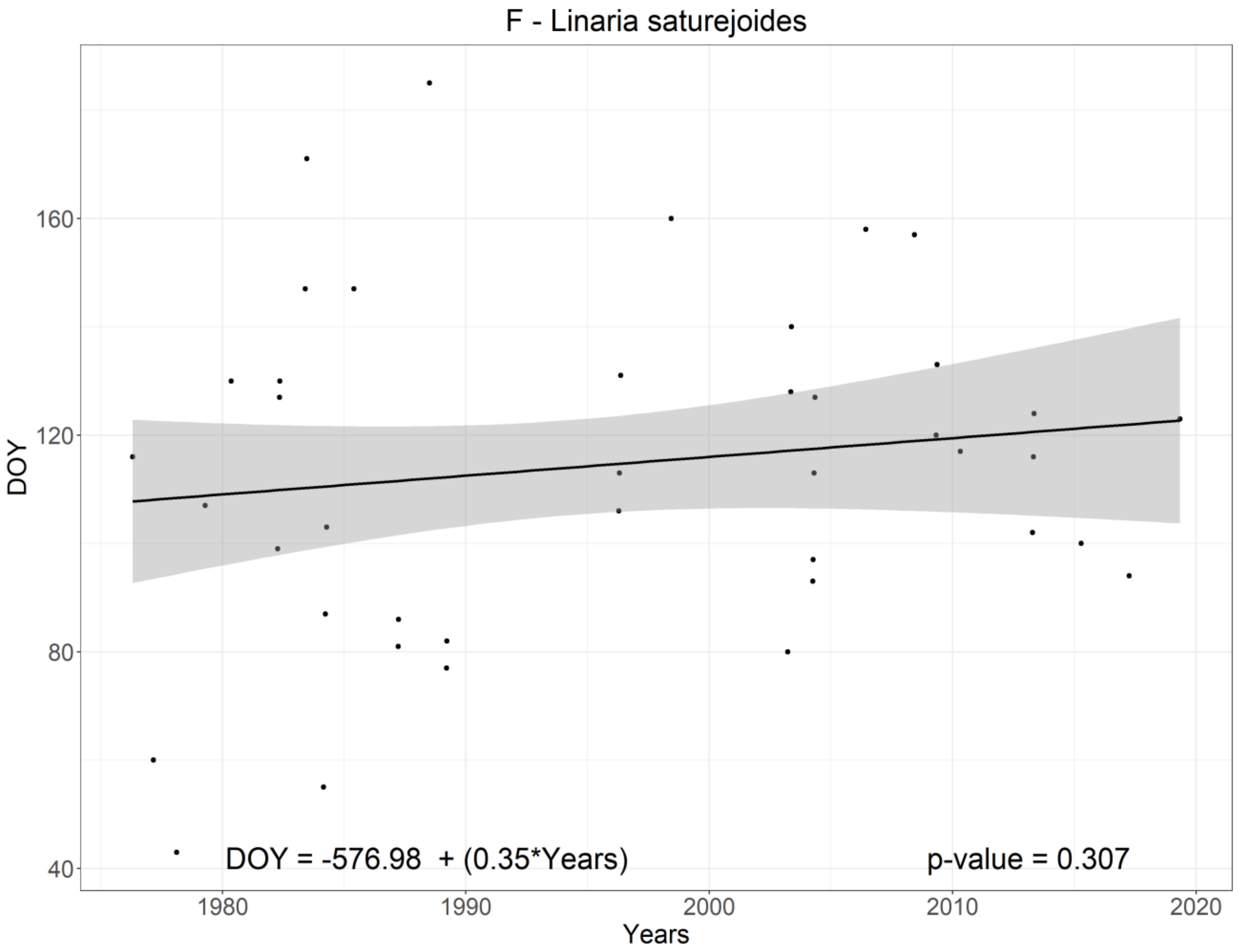

1.70.1. Diagnostics - LM - F - Linaria saturejoides

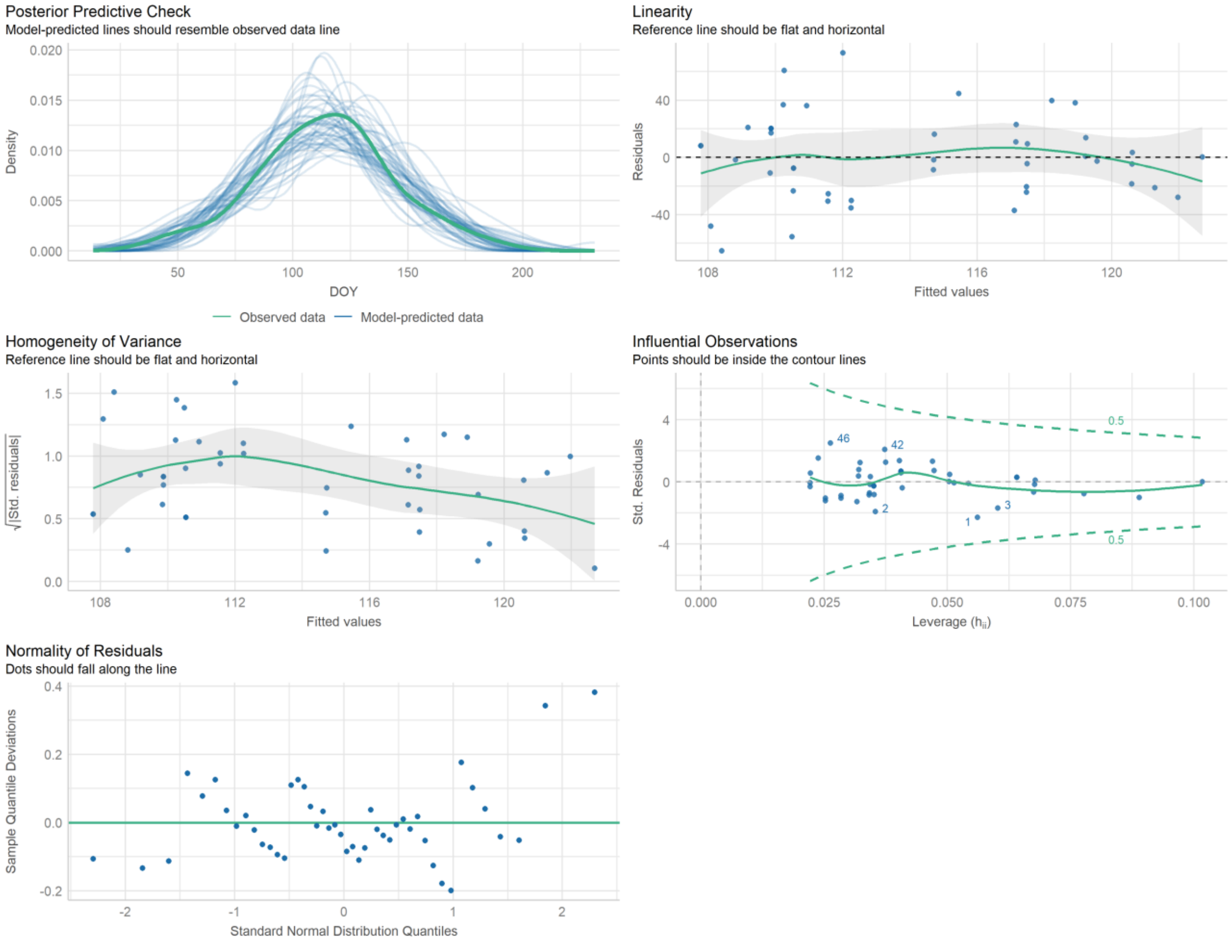

1.71. LM - FBF - Lonicera etrusca

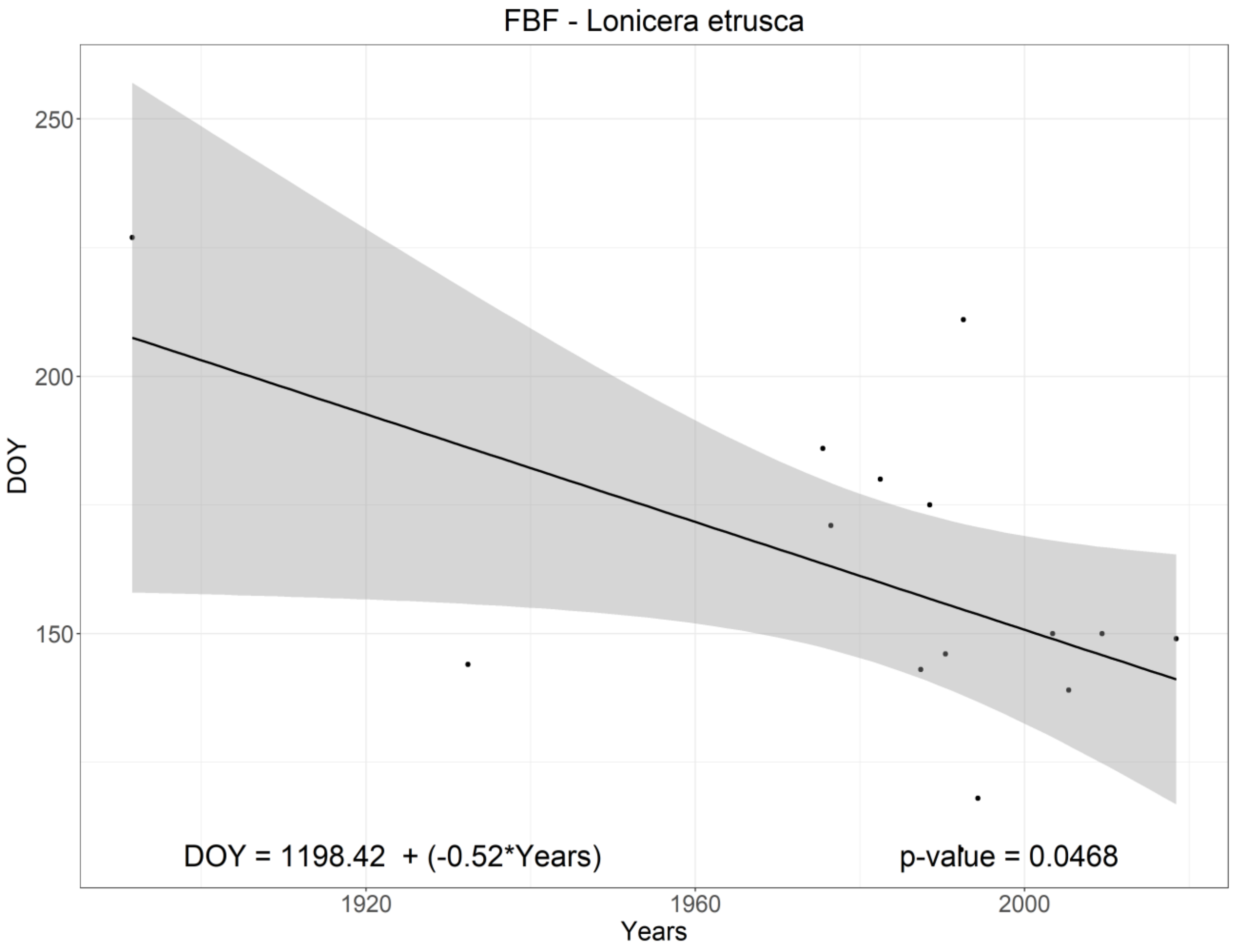

1.71.1. Diagnostics - LM - FBF - Lonicera etrusca

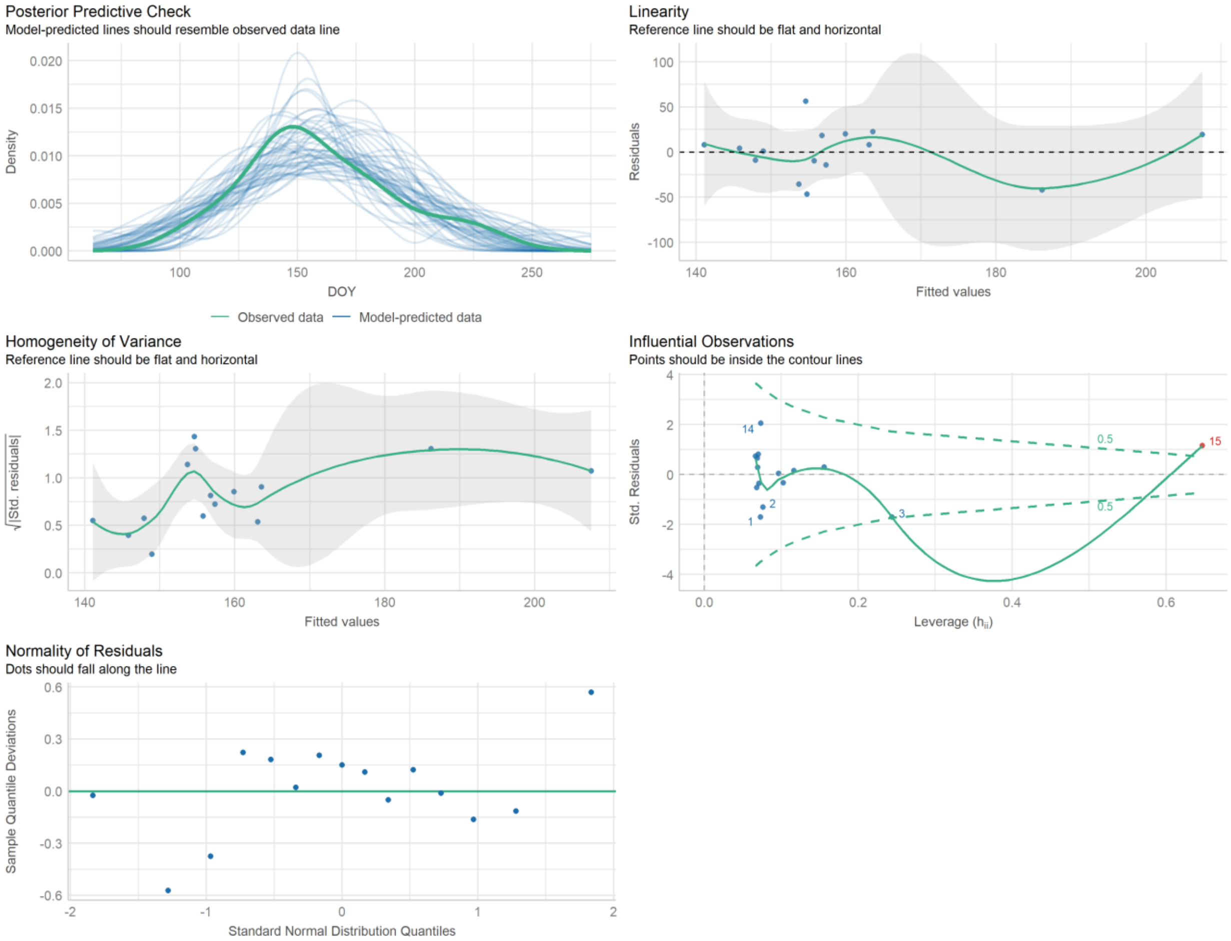

1.72. LM - F - Lonicera etrusca

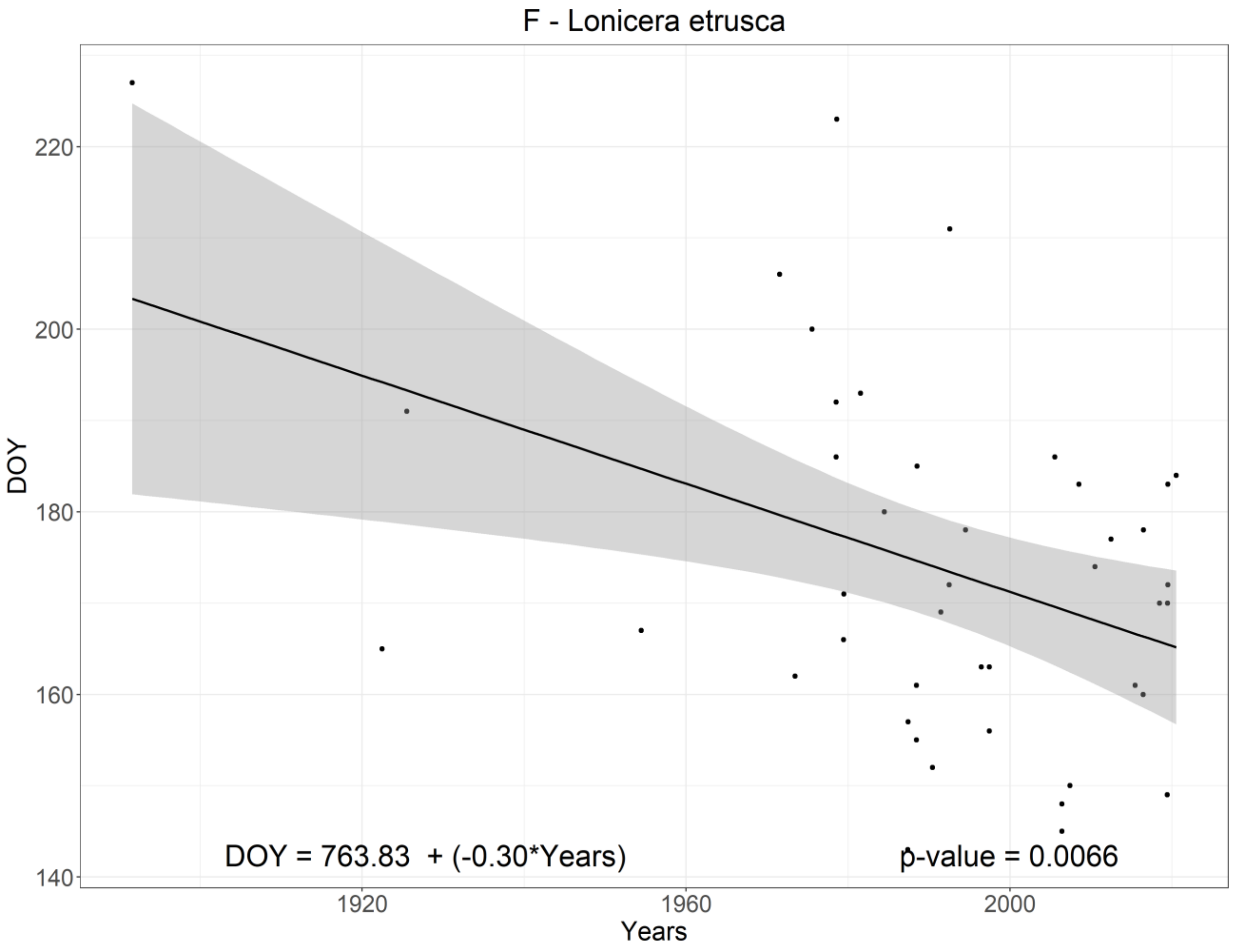

1.72.1. Diagnostics - LM - F - Lonicera etrusca

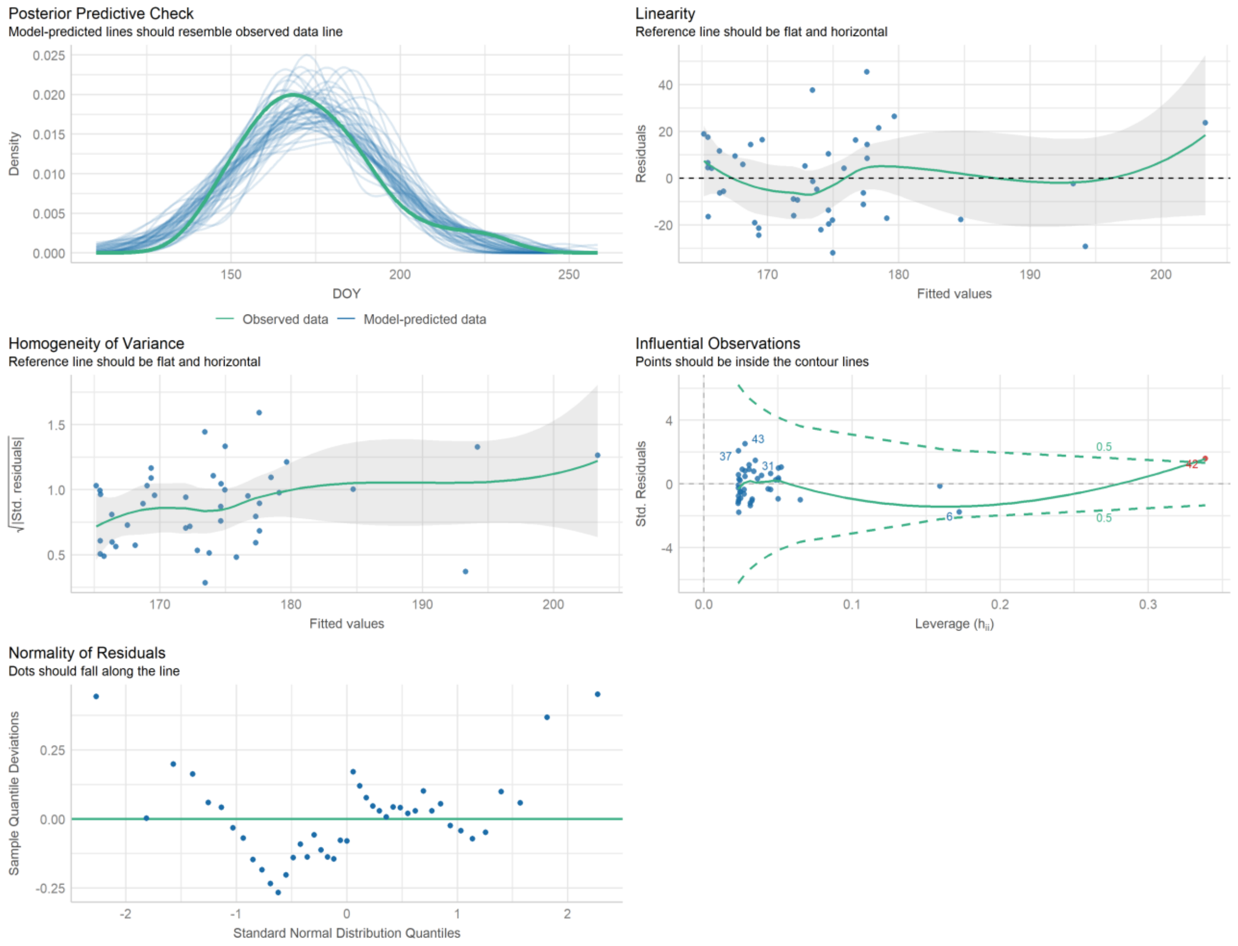

1.73. LM - FBF - Macrochloa tenacissima

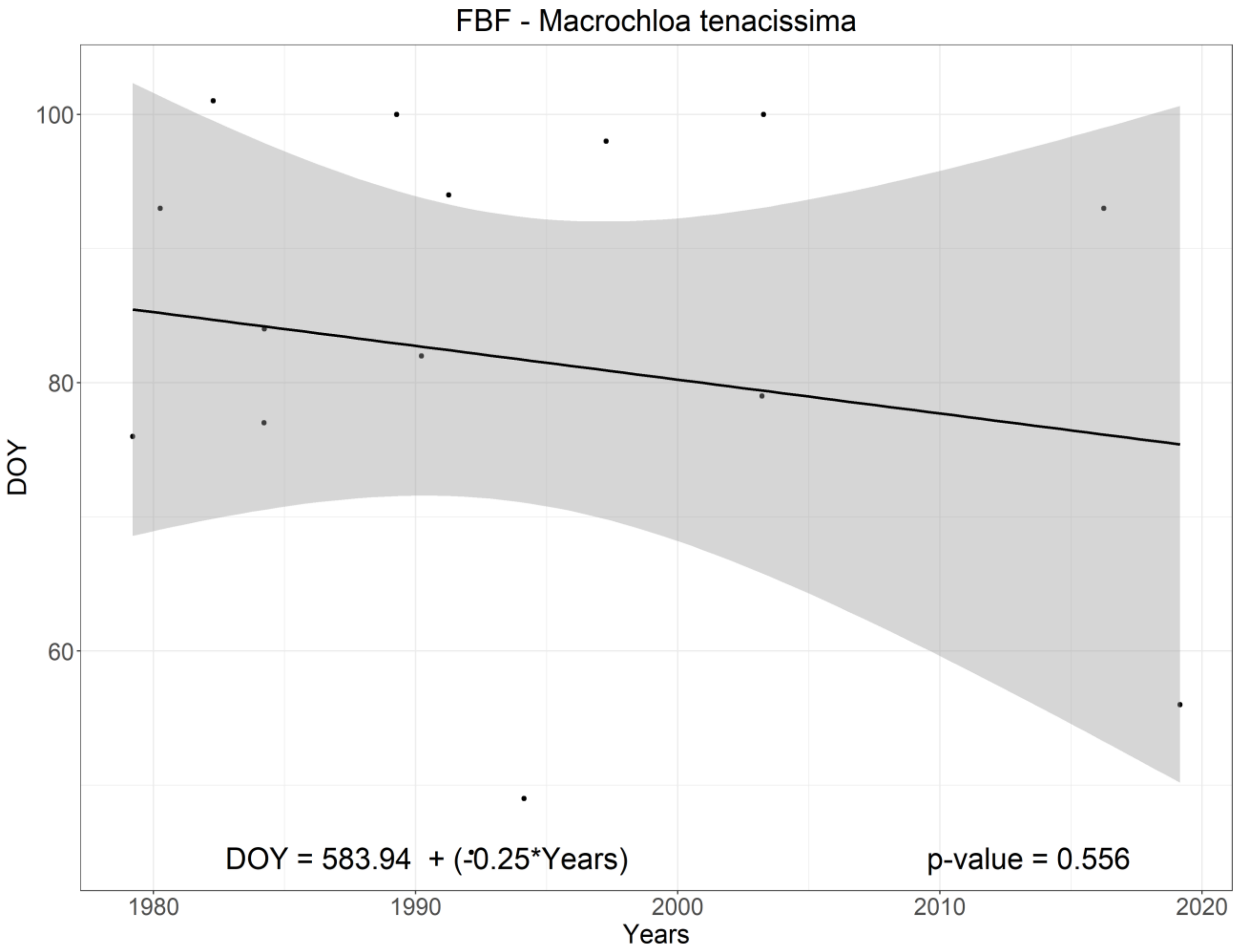

1.73.1. Diagnostics - LM - FBF - Macrochloa tenacissima

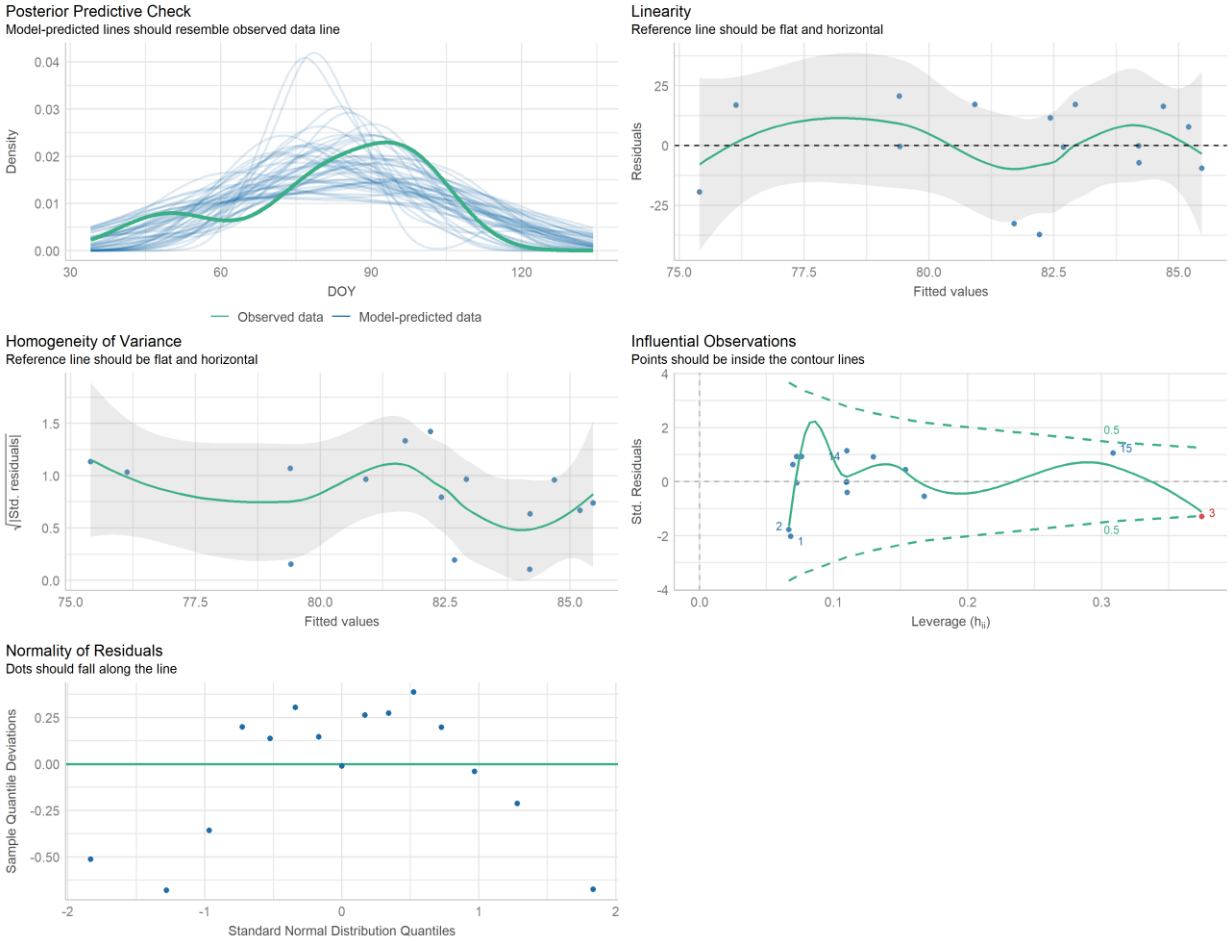

1.74. LM - F - Macrochloa tenacissima

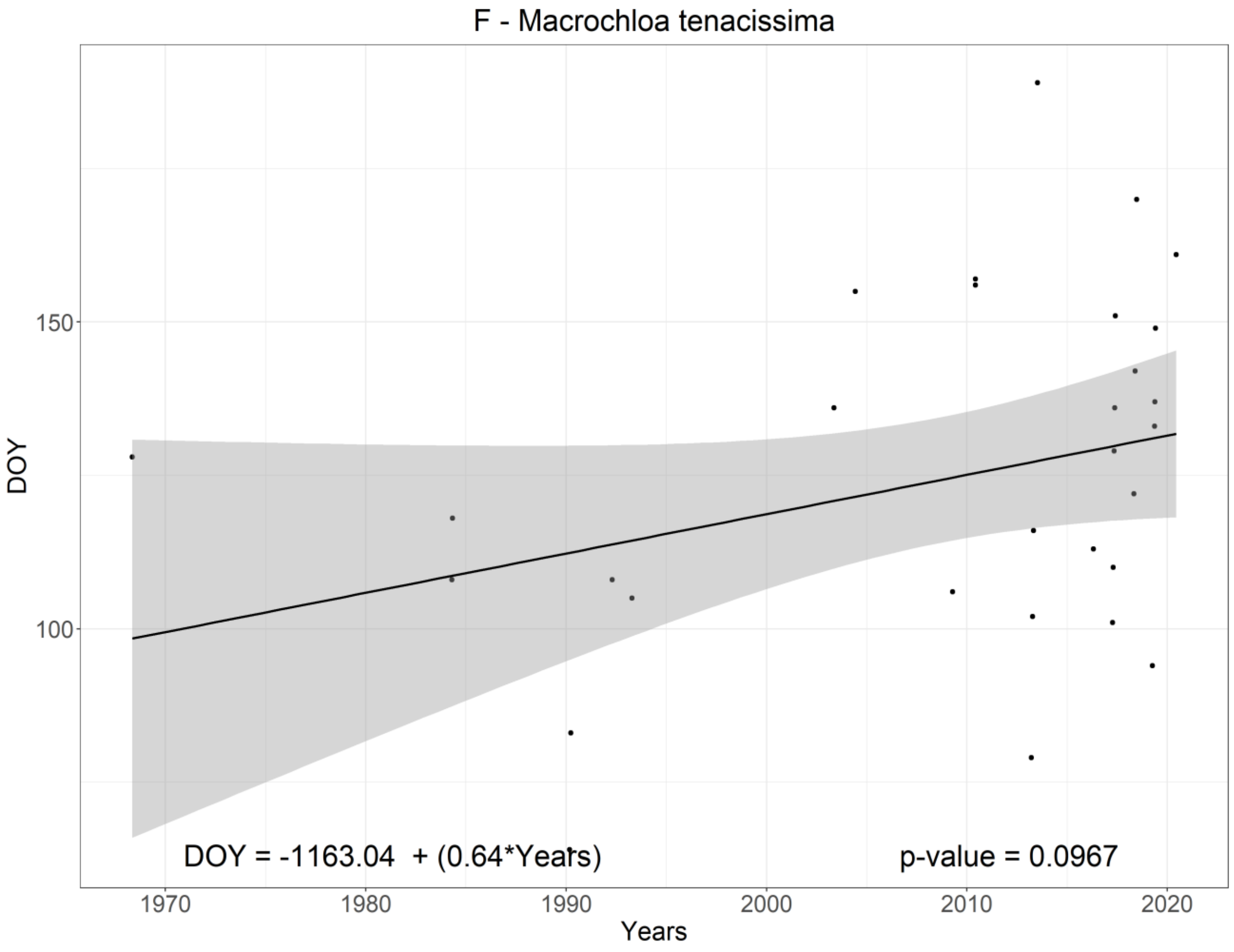

1.74.1. Diagnostics - LM - F - Macrochloa tenacissima

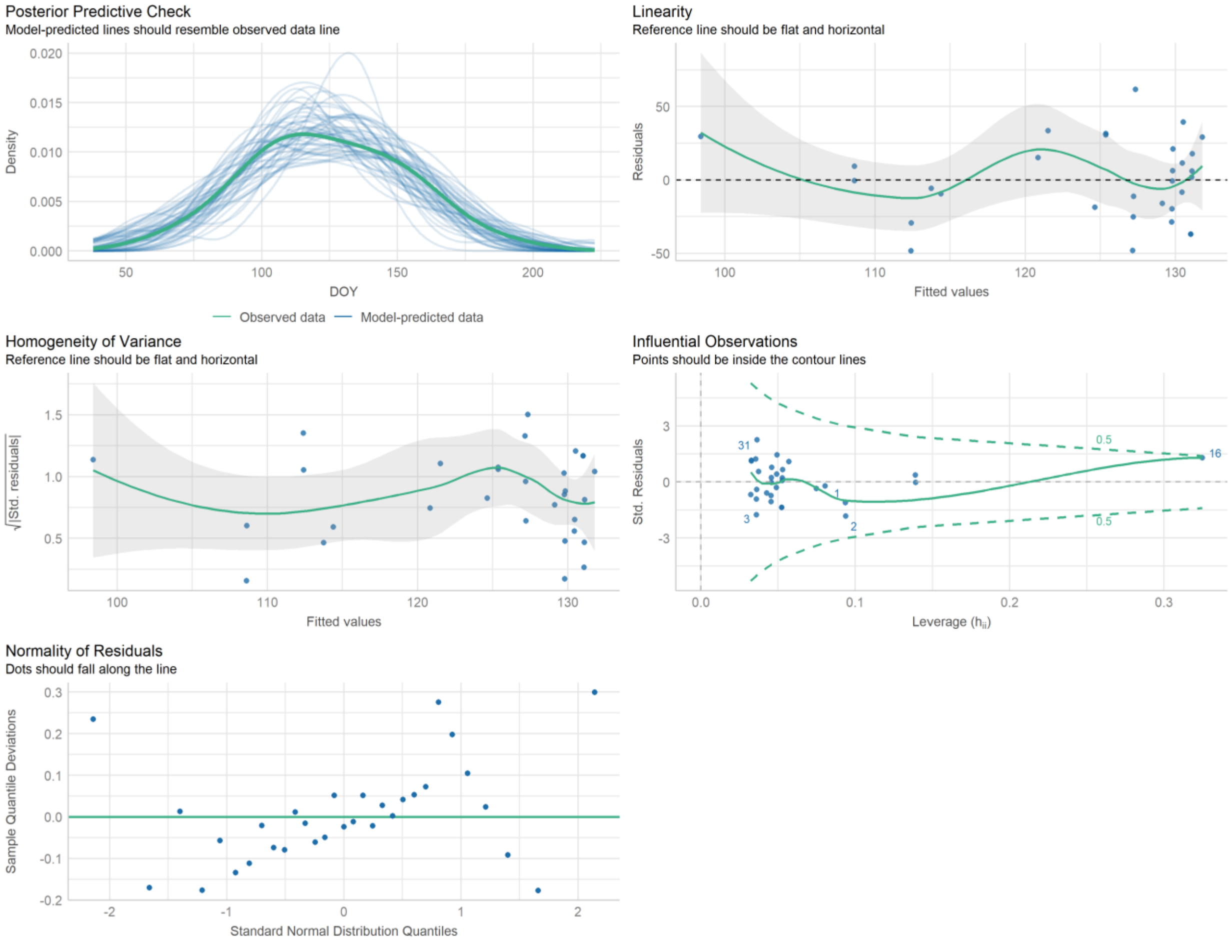

1.75. LM - F - Myrtus communis

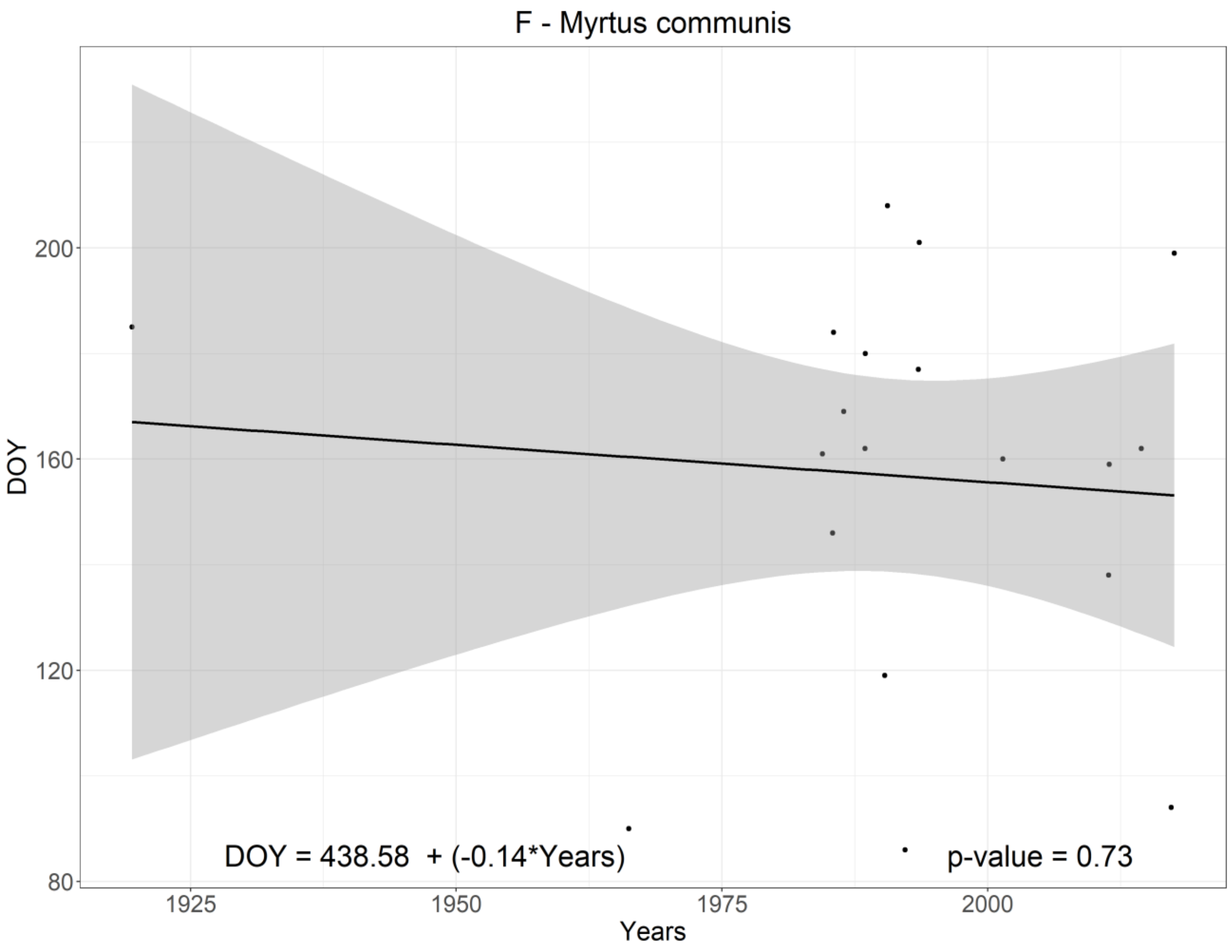

1.75.1. Diagnostics - LM - F - Myrtus communis

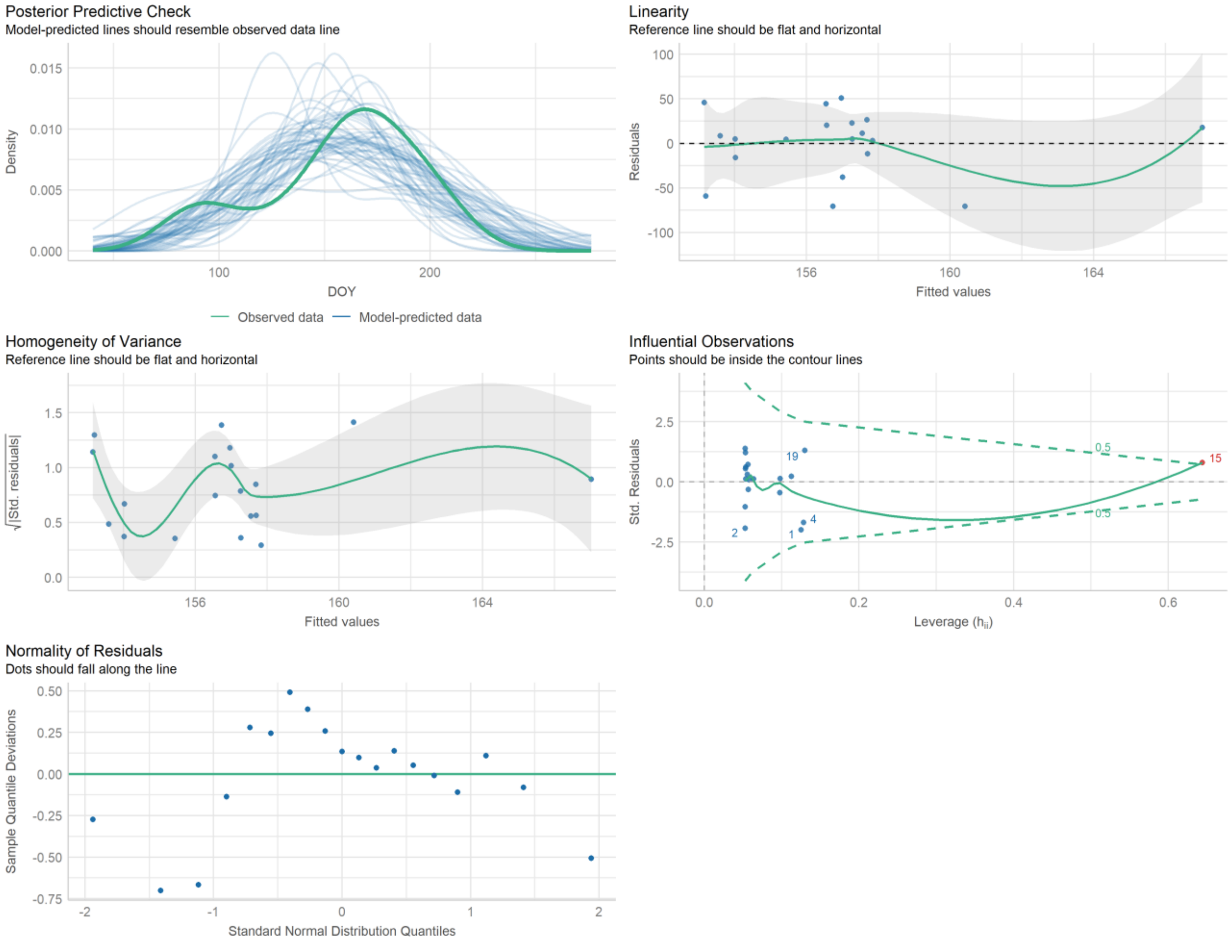

1.76. LM - DVG - Myrtus communis

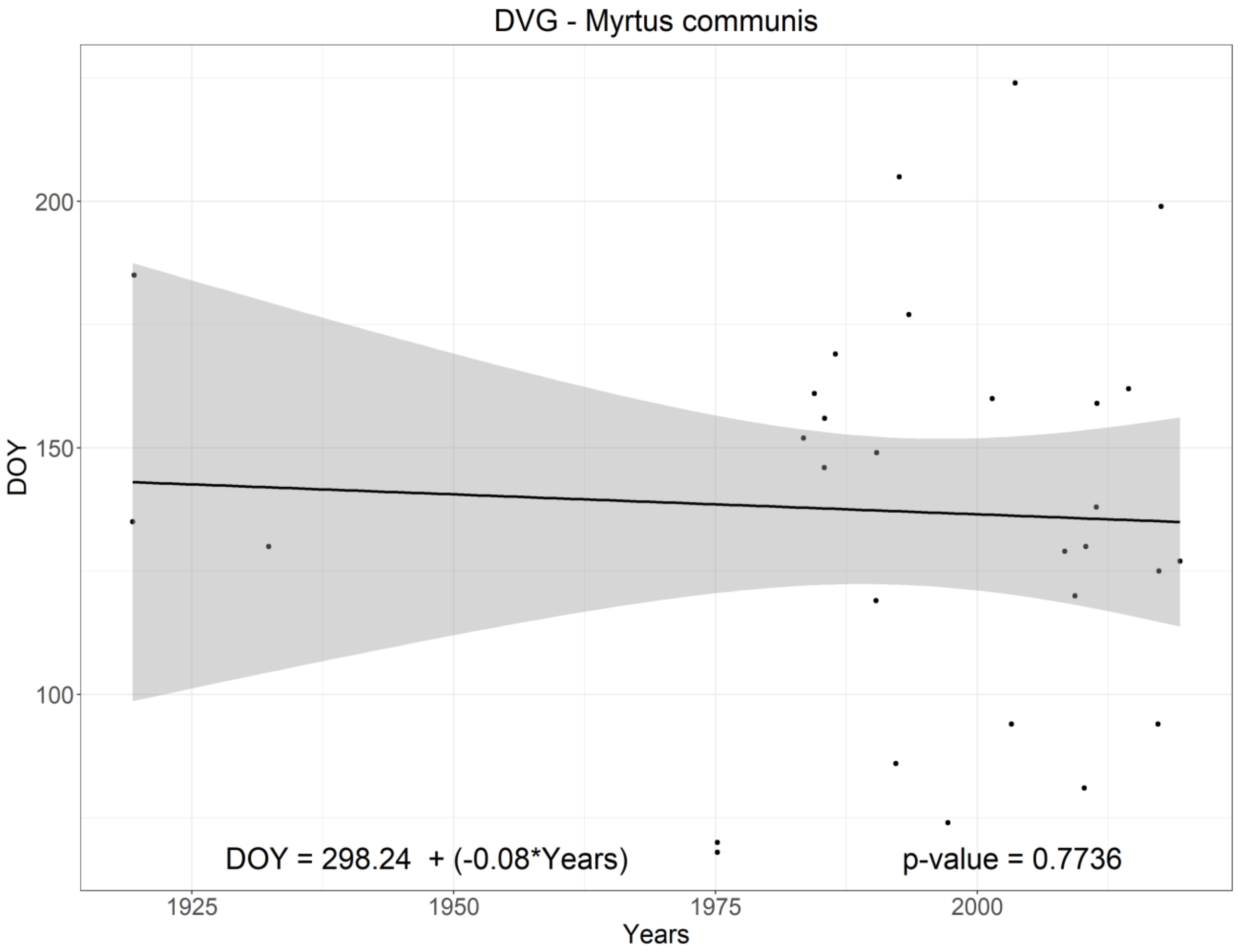

1.76.1. Diagnostics - LM - DVG - Myrtus communis

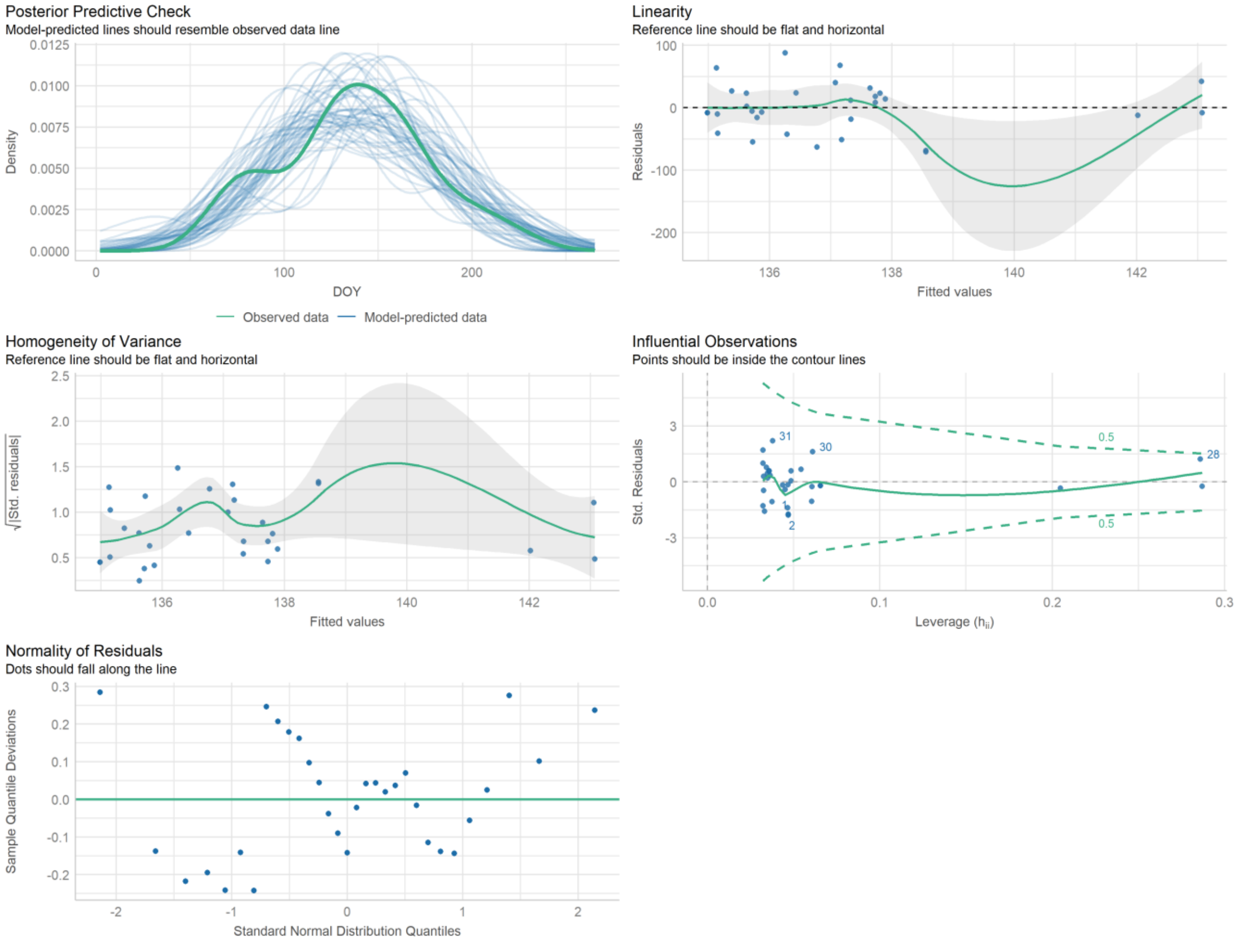

1.77. LM - F - Nevadensia purpurea

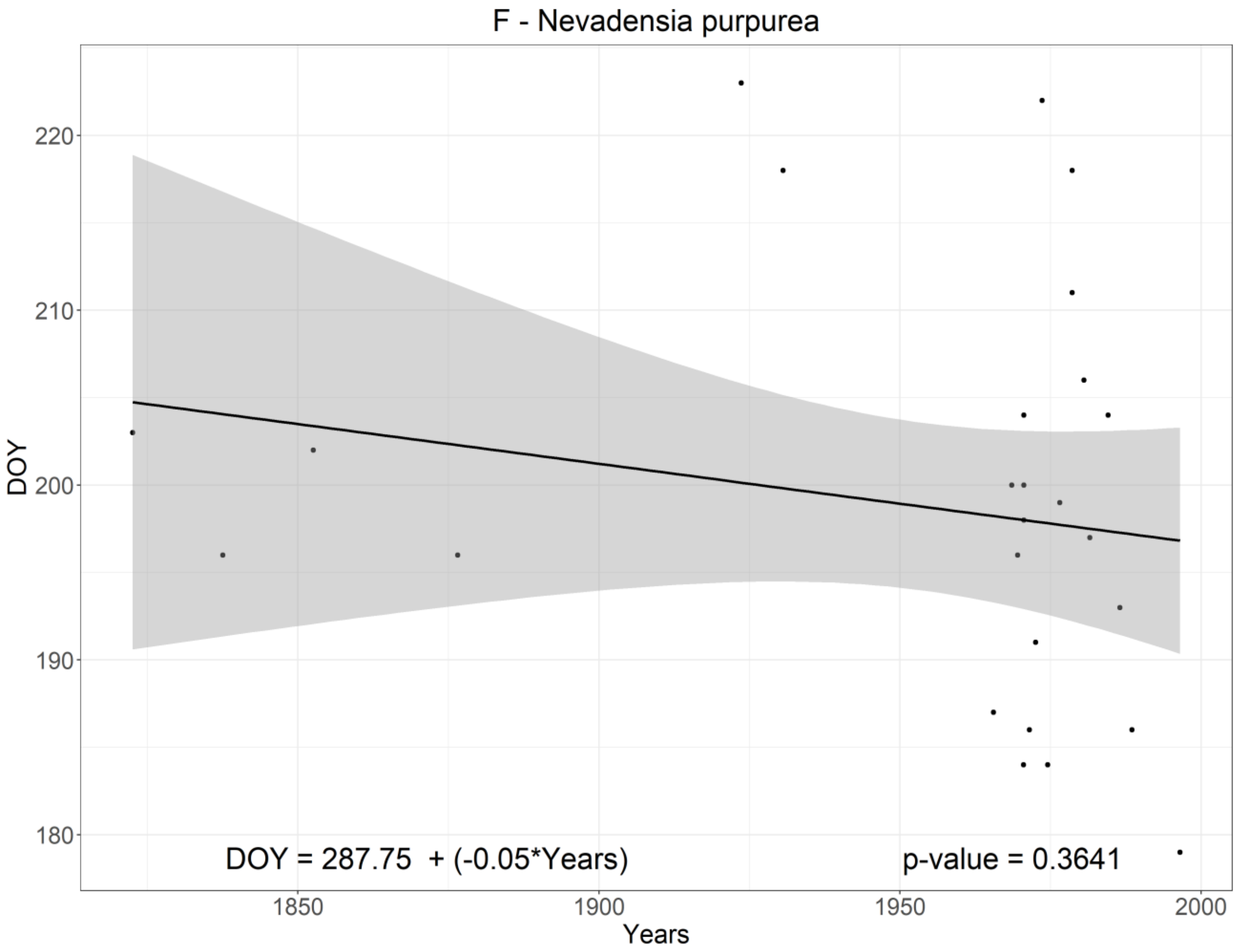

1.77.1. Diagnostics - LM - F - Nevadensia purpurea

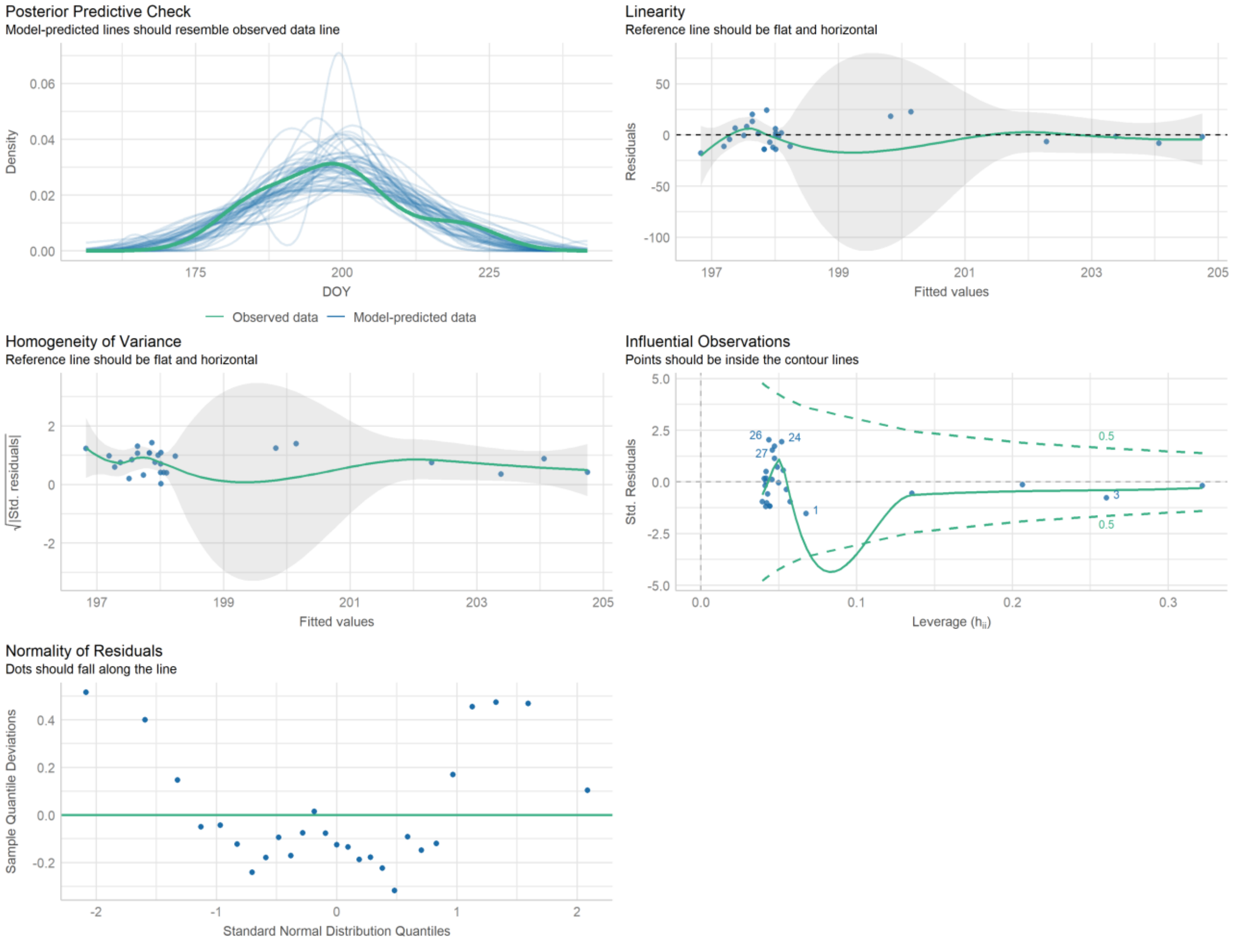

1.78. LM - FS - Nevadensia purpurea

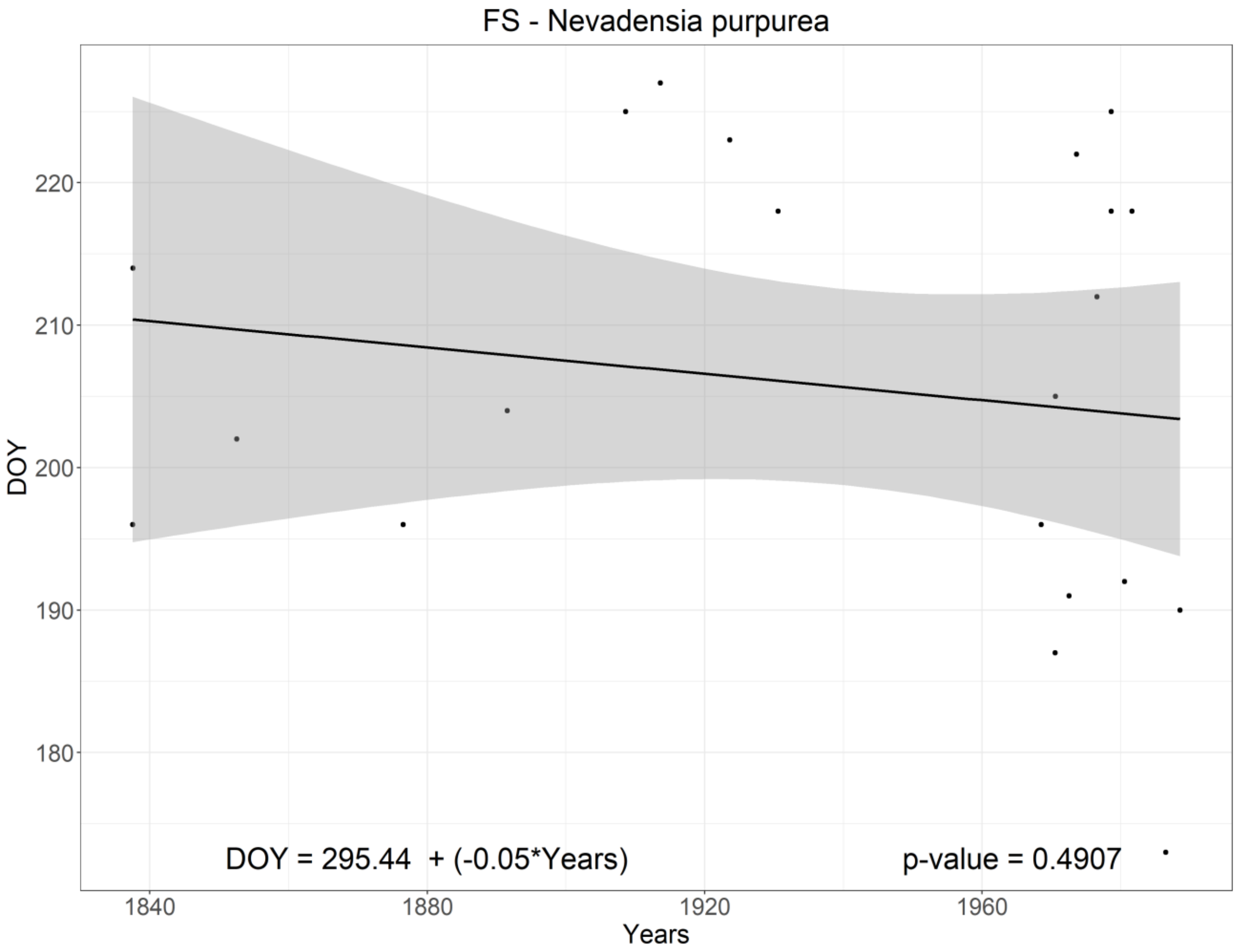

1.78.1. Diagnostics - LM - FS - Nevadensia purpurea

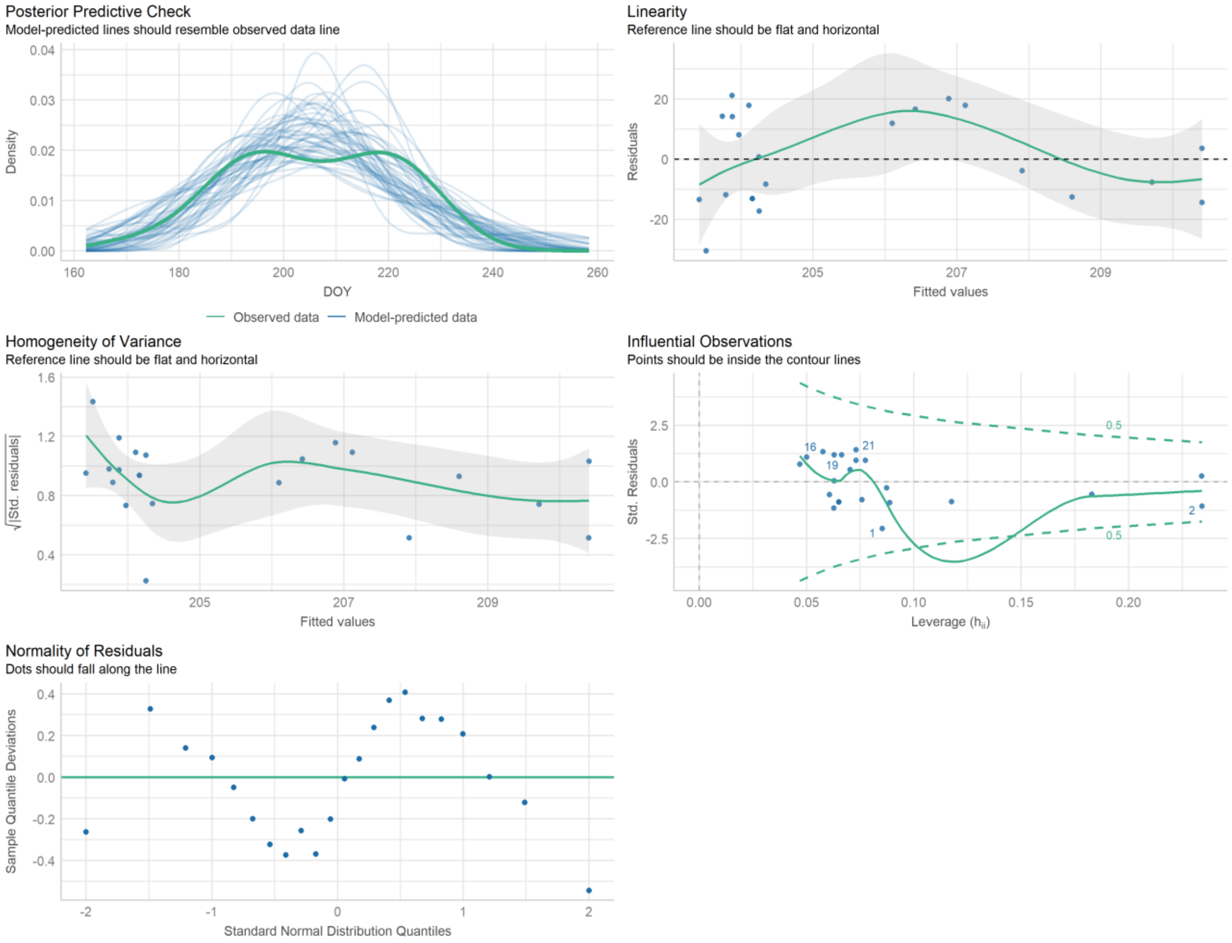

1.79. LM - FBF - Olea europaea var. sylvestris

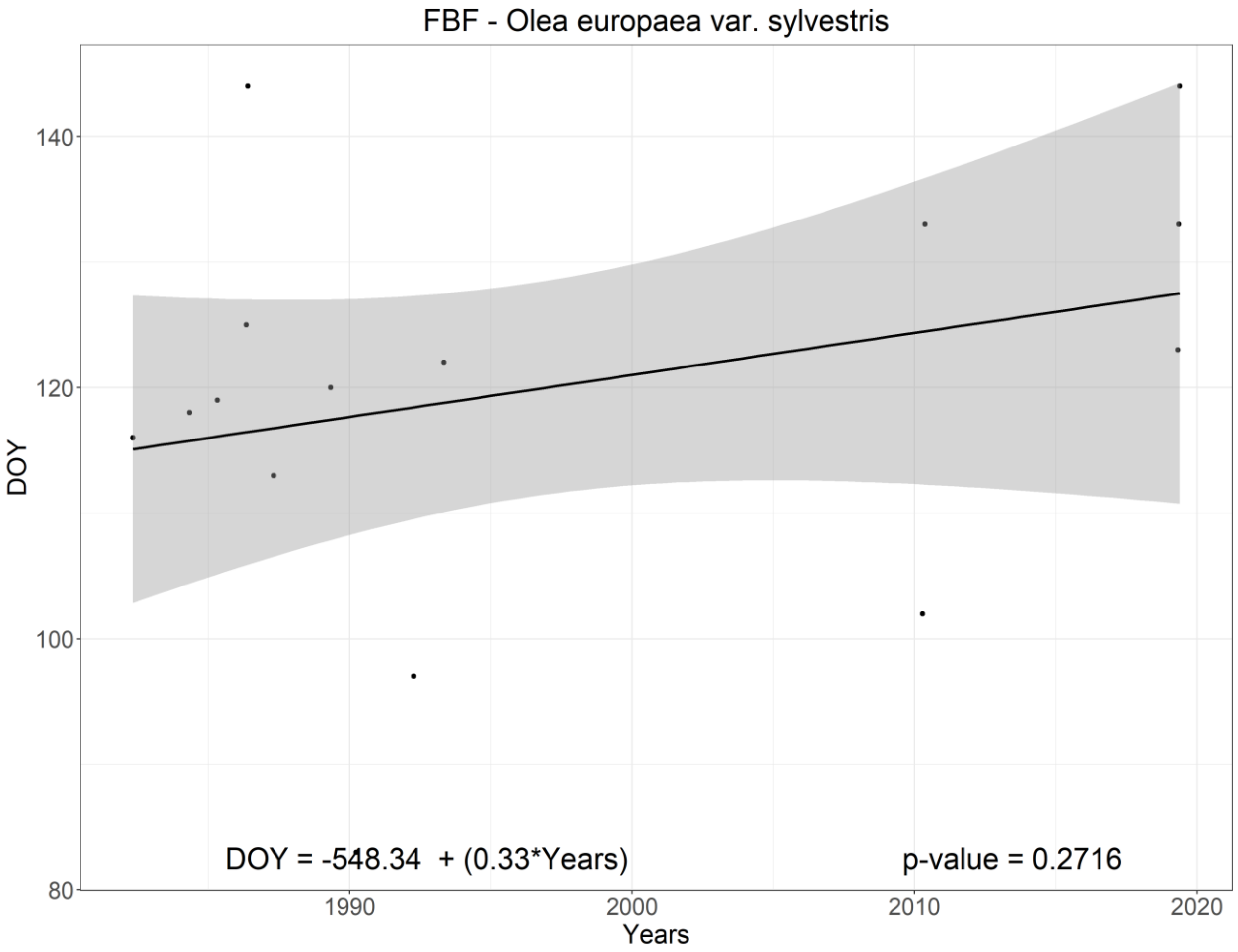

1.79.1. Diagnostics - LM - FBF - Olea europaea var. sylvestris

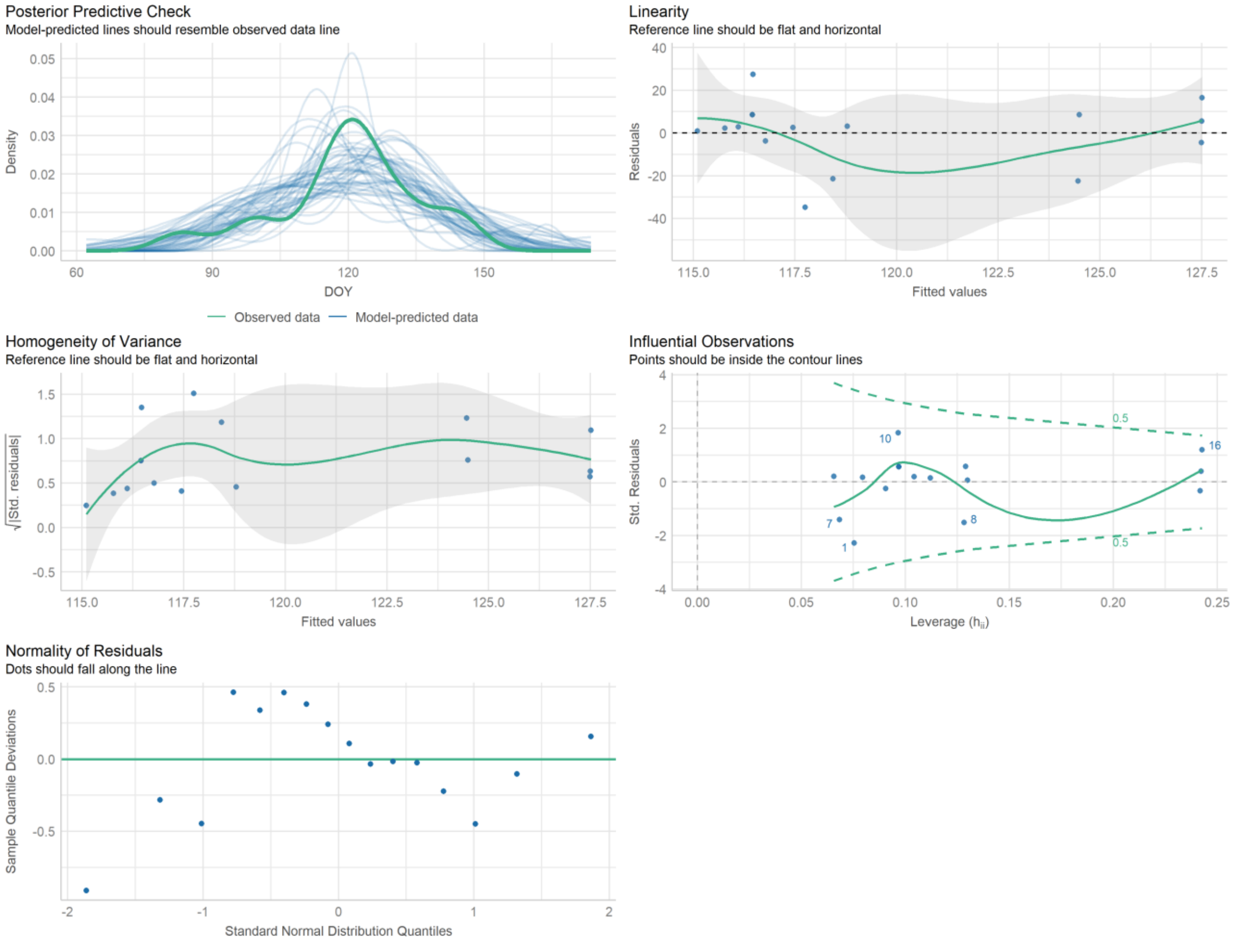

1.80. LM - DVG - Olea europaea var. sylvestris

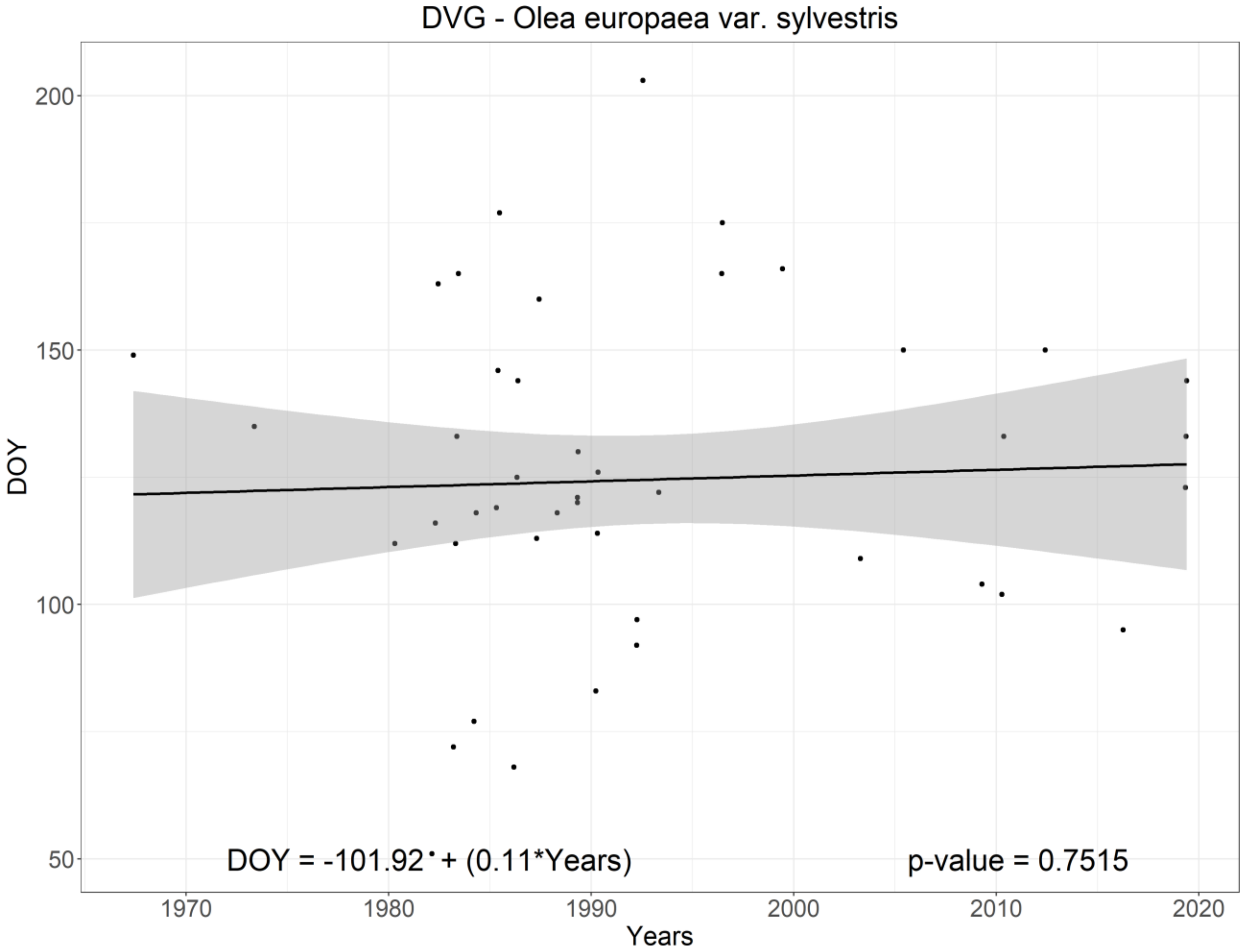

1.80.1. Diagnostics - LM - DVG - Olea europaea var. sylvestris

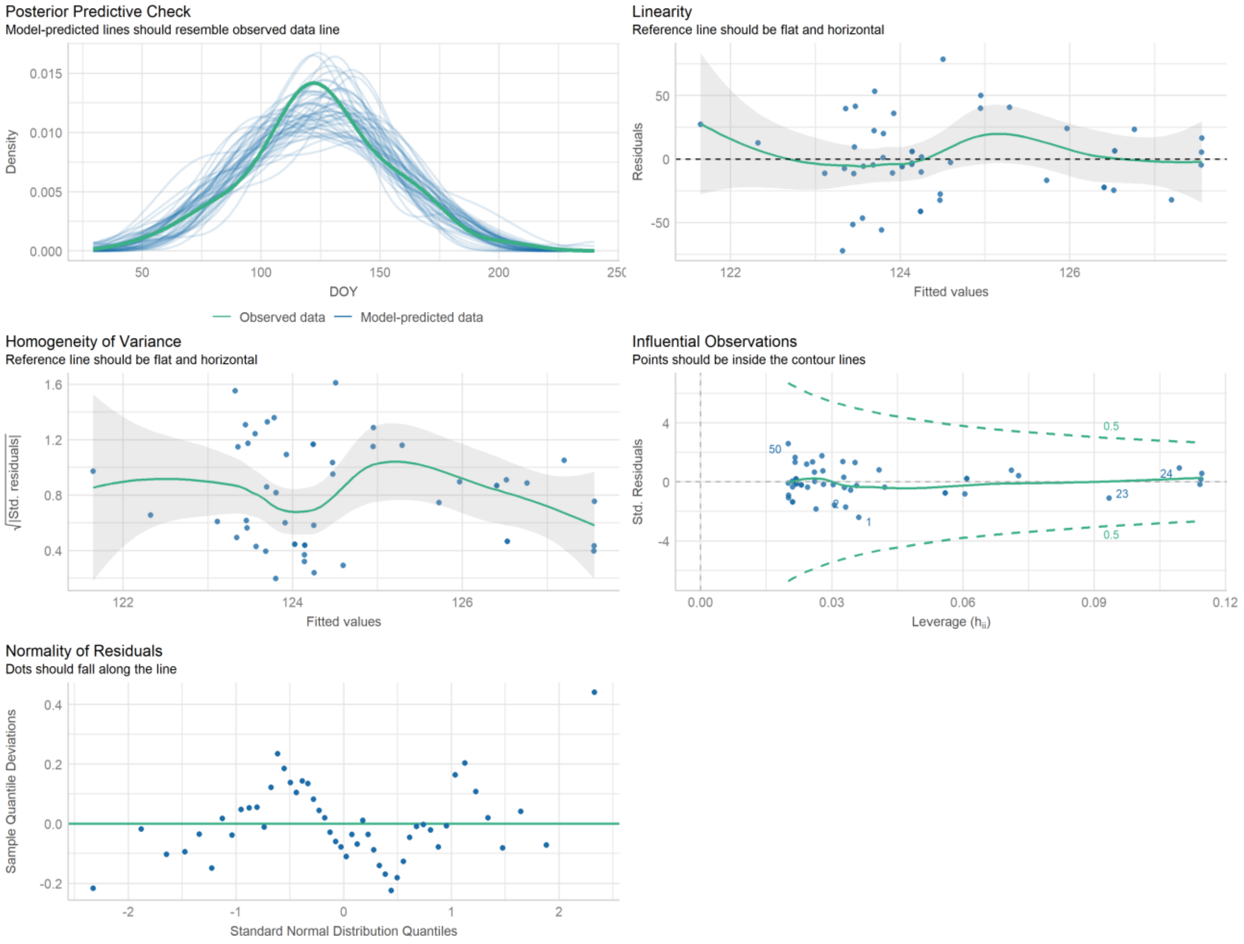

1.81. LM - FBF - Phillyrea angustifolia

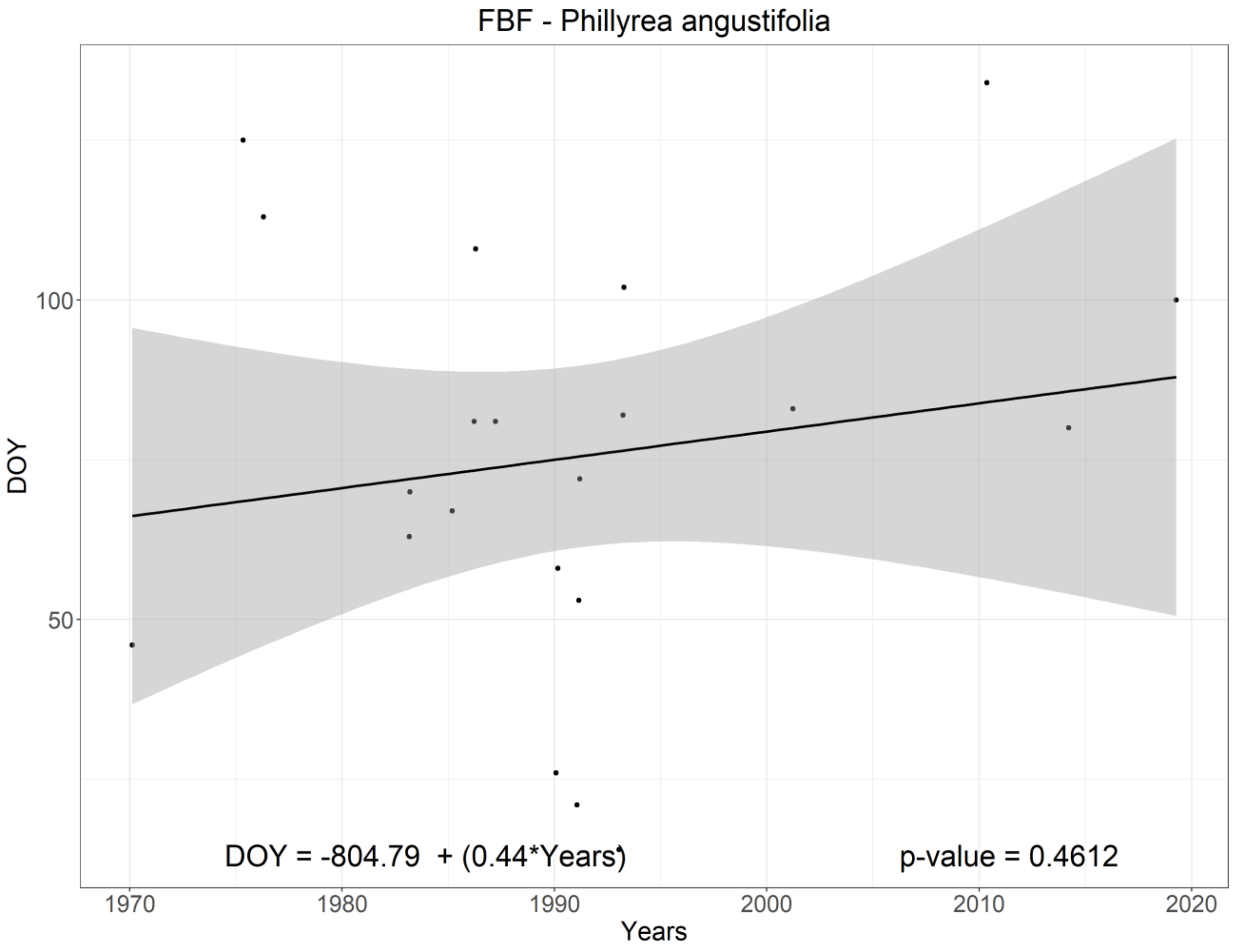

1.81.1. Diagnostics - LM - FBF - Phillyrea angustifolia

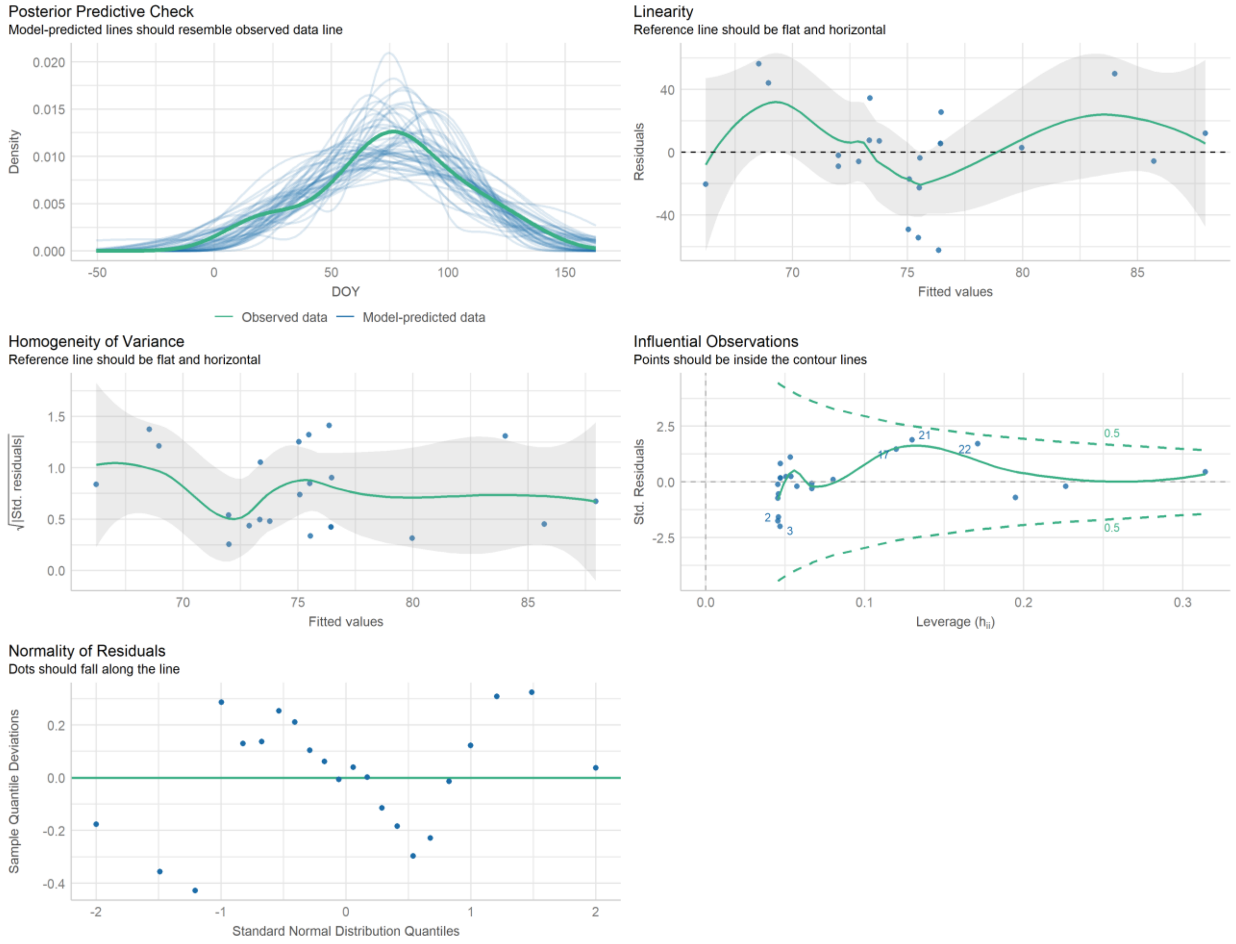

1.82. LM - F - Phillyrea angustifolia

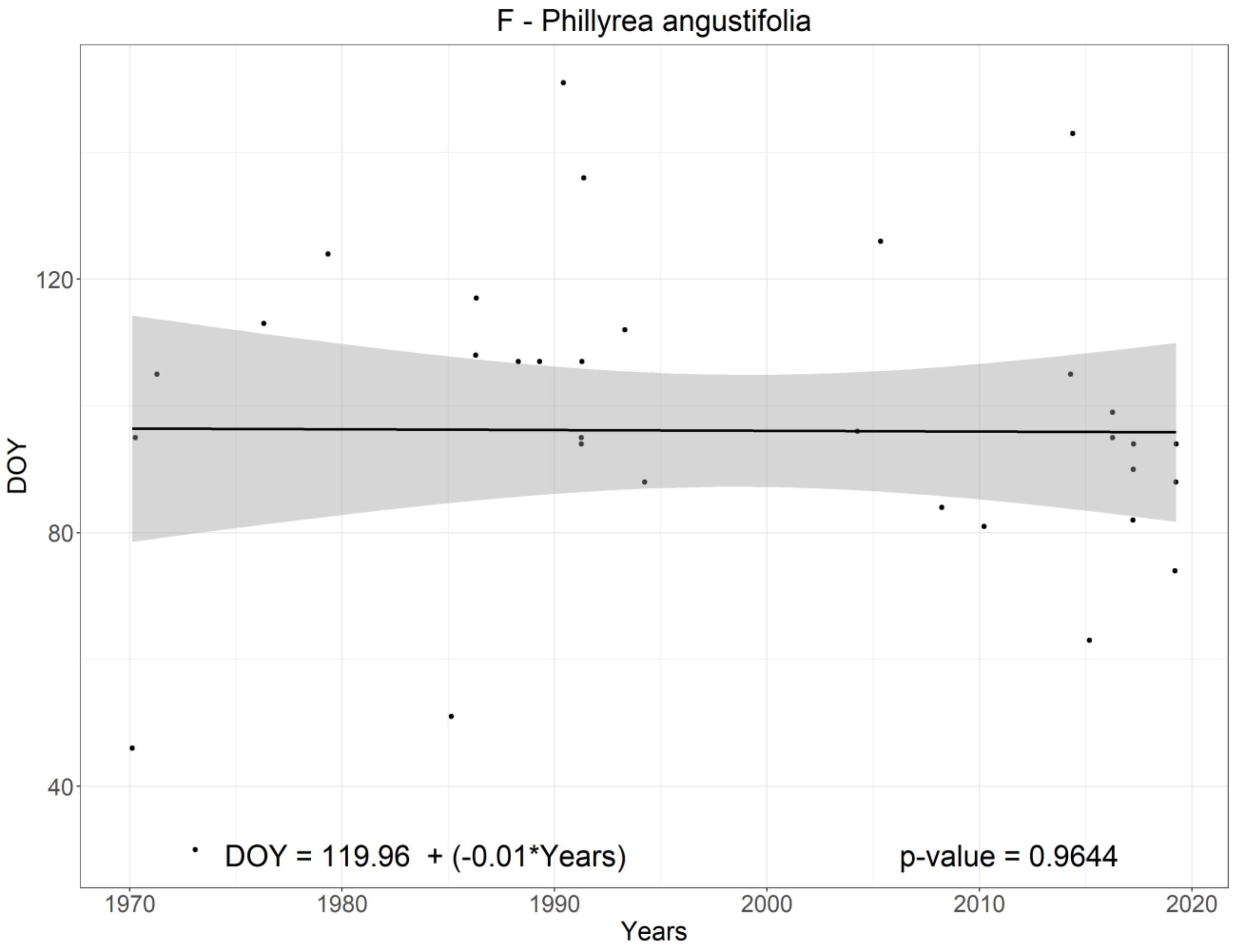

1.82.1. Diagnostics - LM - F - Phillyrea angustifolia

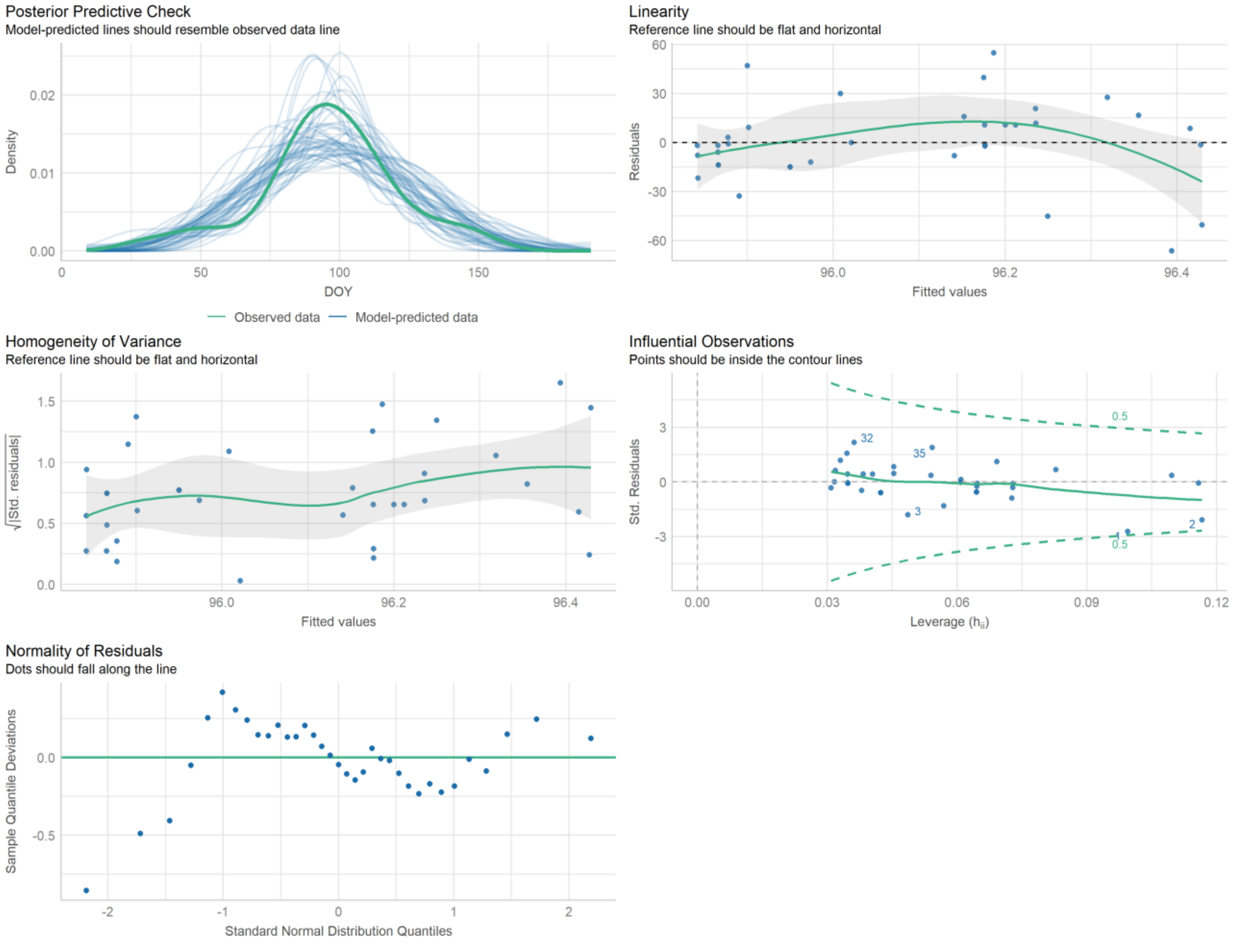

1.83. LM - DVG - Phillyrea angustifolia

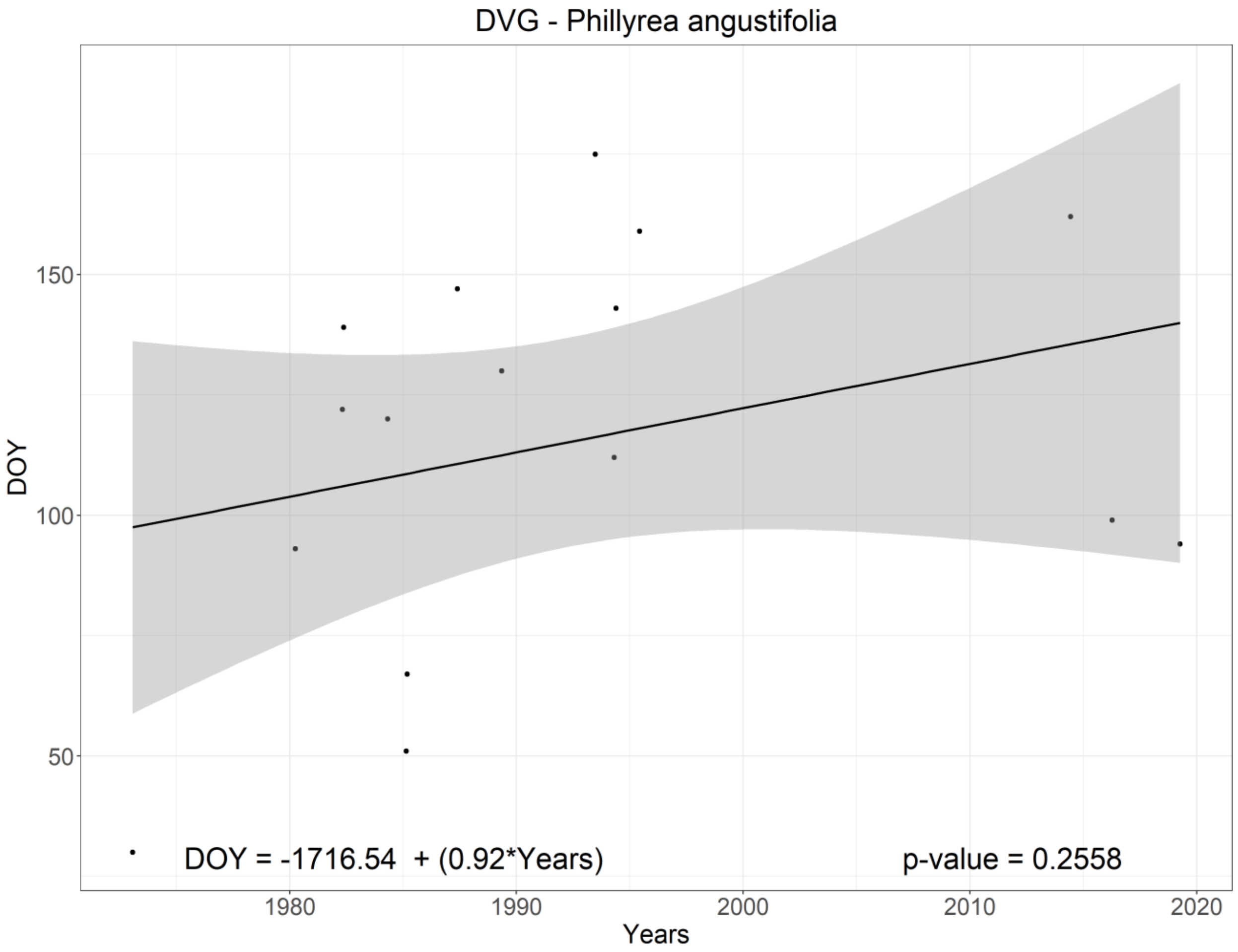

1.83.1. Diagnostics - LM - DVG - Phillyrea angustifolia

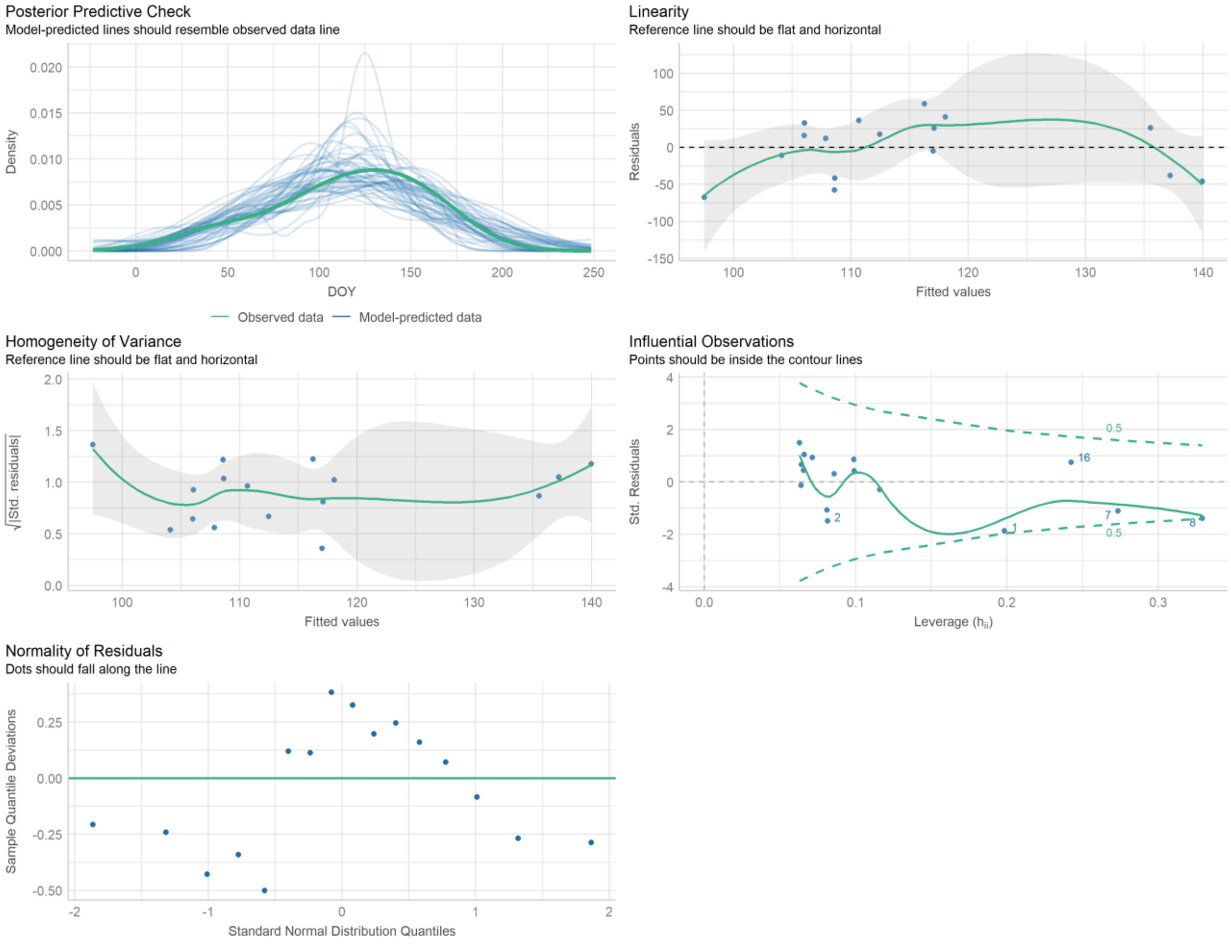

1.84. LM - FBF - Phlomis crinita subsp. malacitana

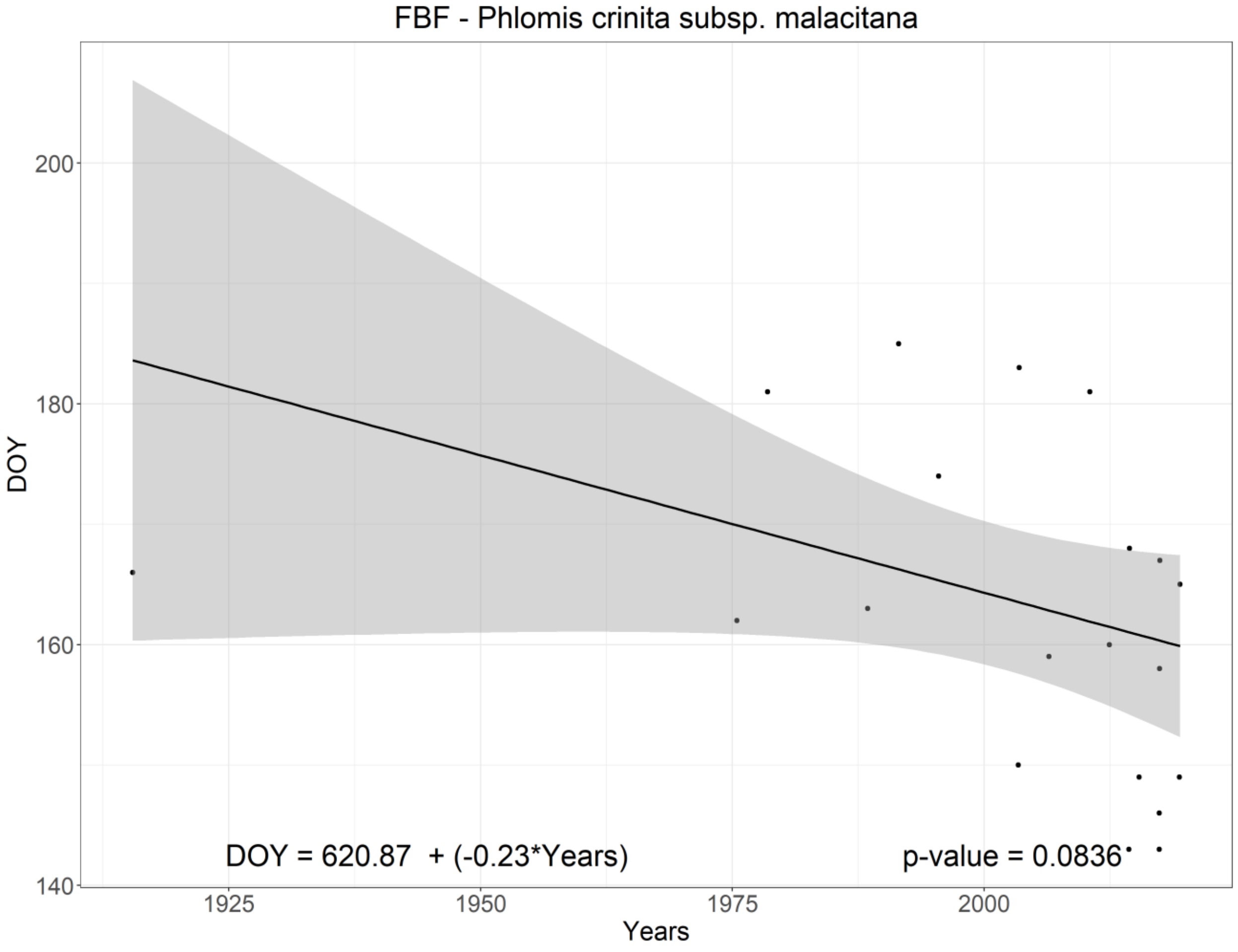

1.84.1. Diagnostics - LM - FBF - Phlomis crinita subsp. malacitana

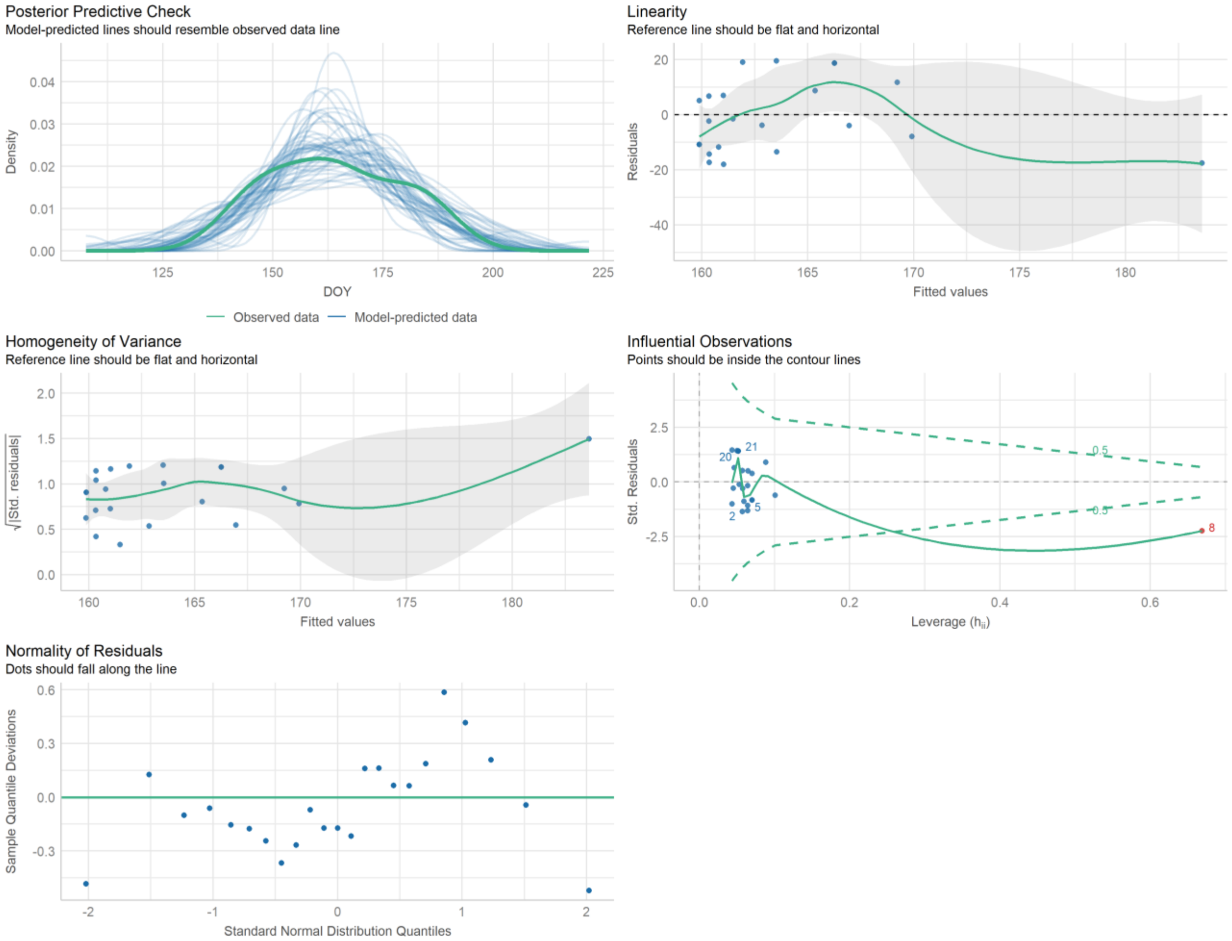

1.85. LM - F - Phlomis crinita subsp. malacitana

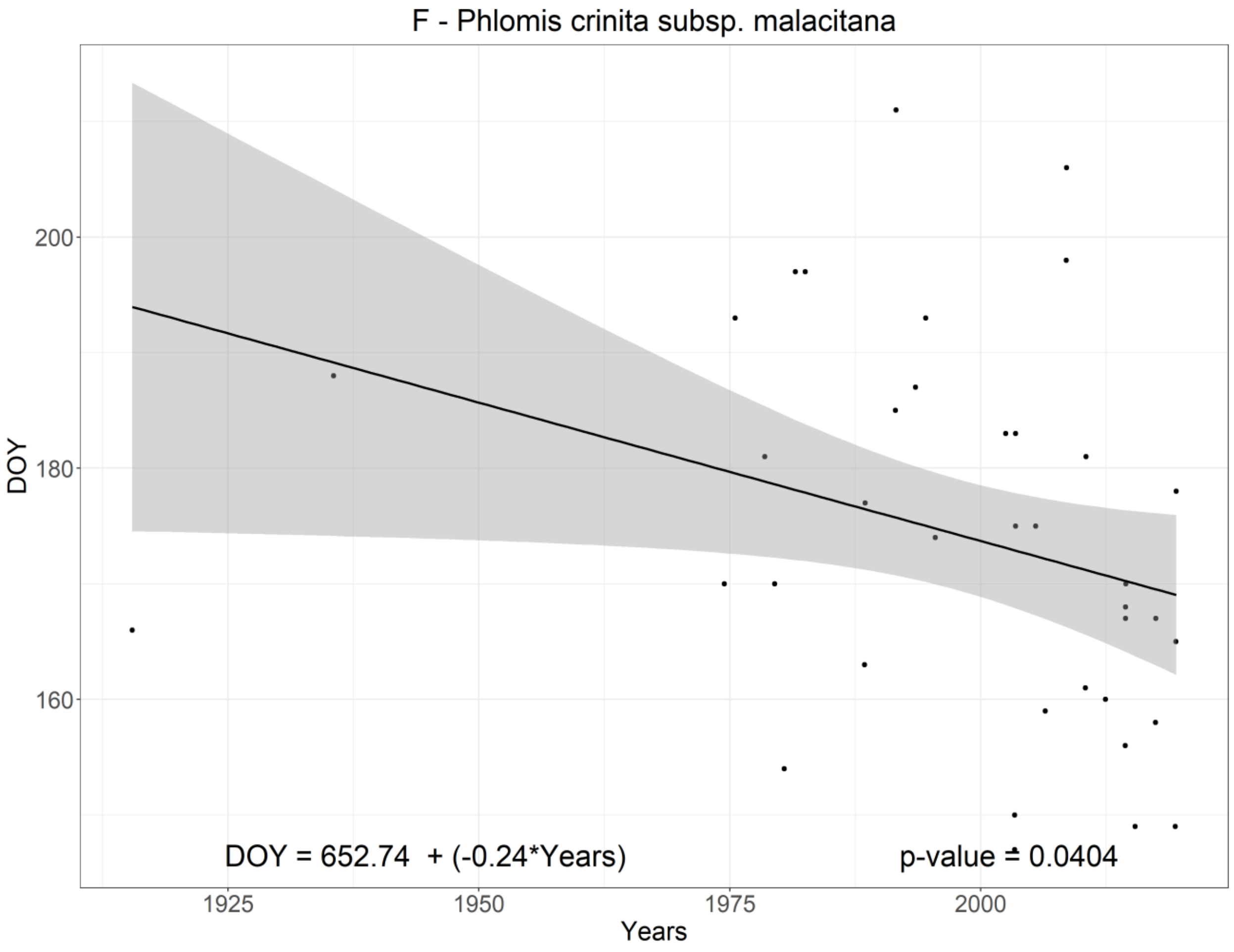

1.85.1. Diagnostics - LM - F - Phlomis crinita subsp. malacitana

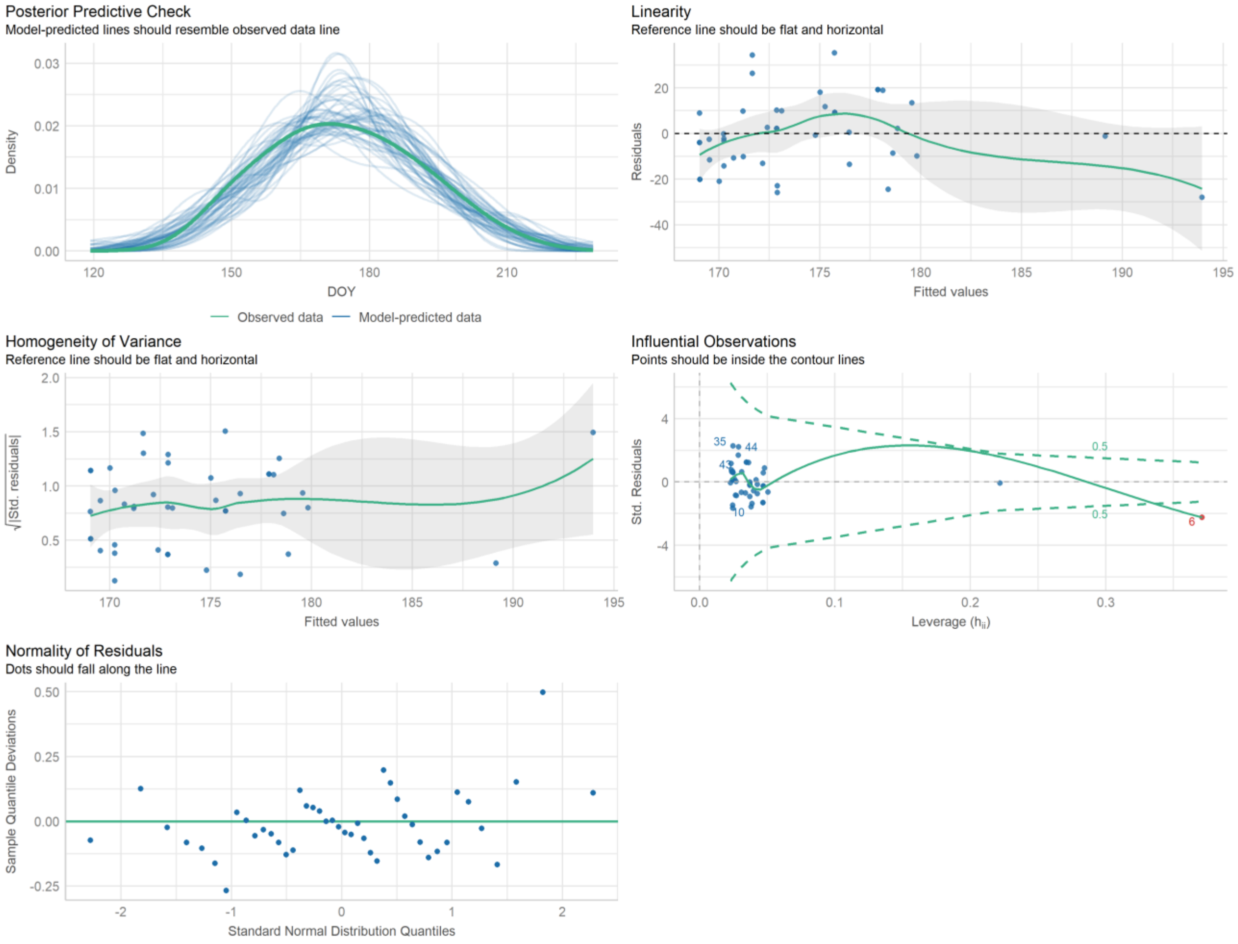

1.86. LM - FBF - Phlomis purpurea

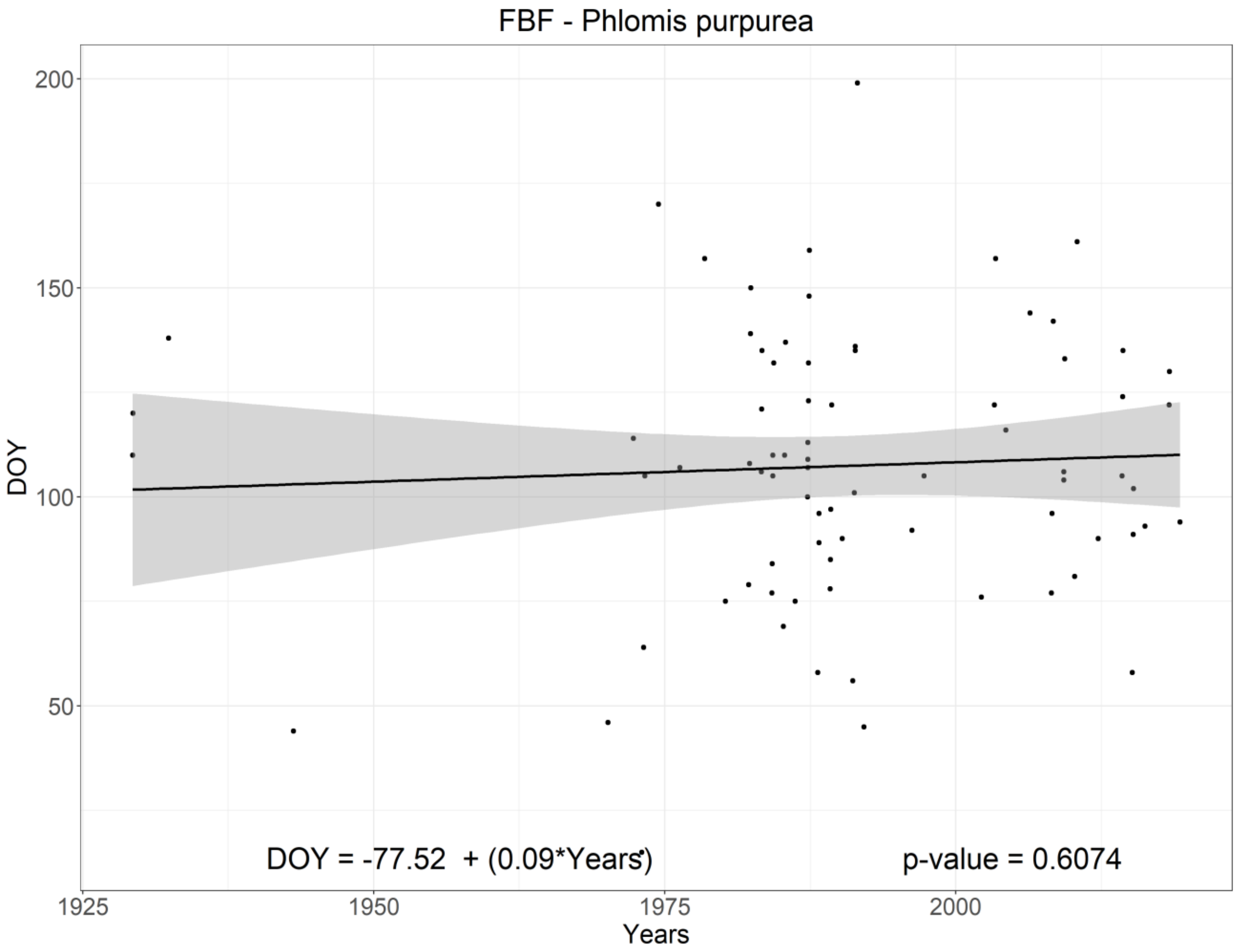

1.86.1. Diagnostics - LM - FBF - Phlomis purpurea

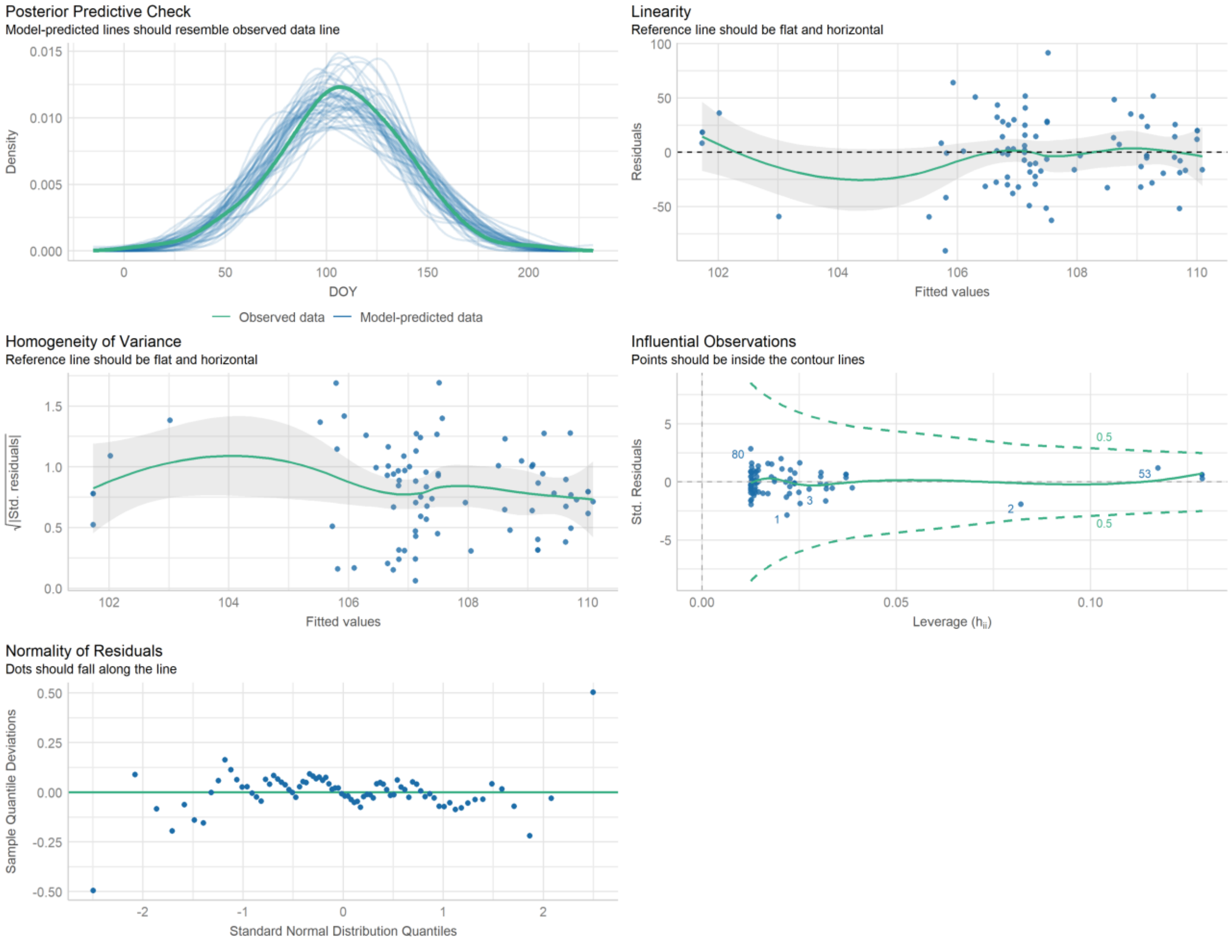

1.87. LM - F - Phlomis purpurea

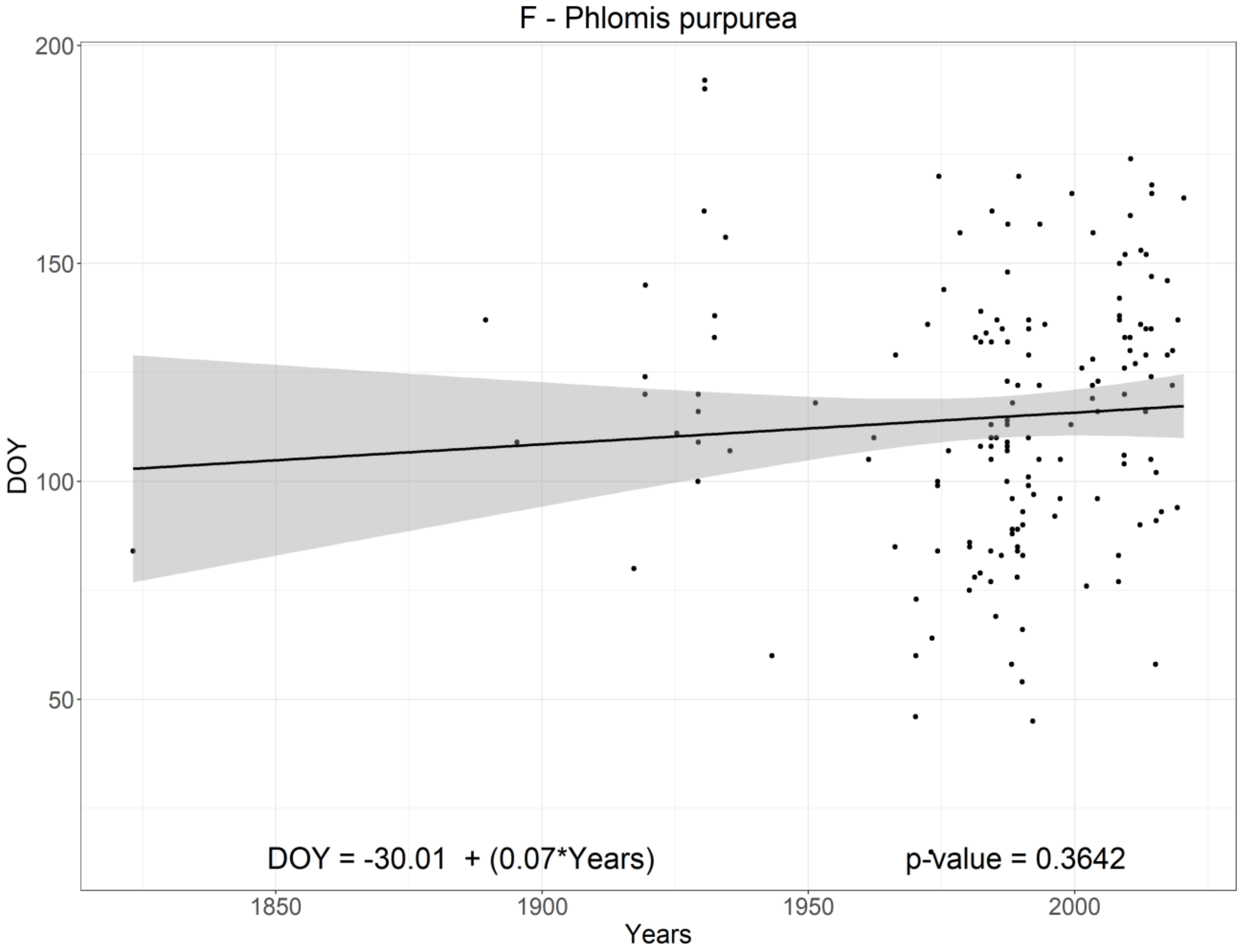

1.87.1. Diagnostics - LM - F - Phlomis purpurea

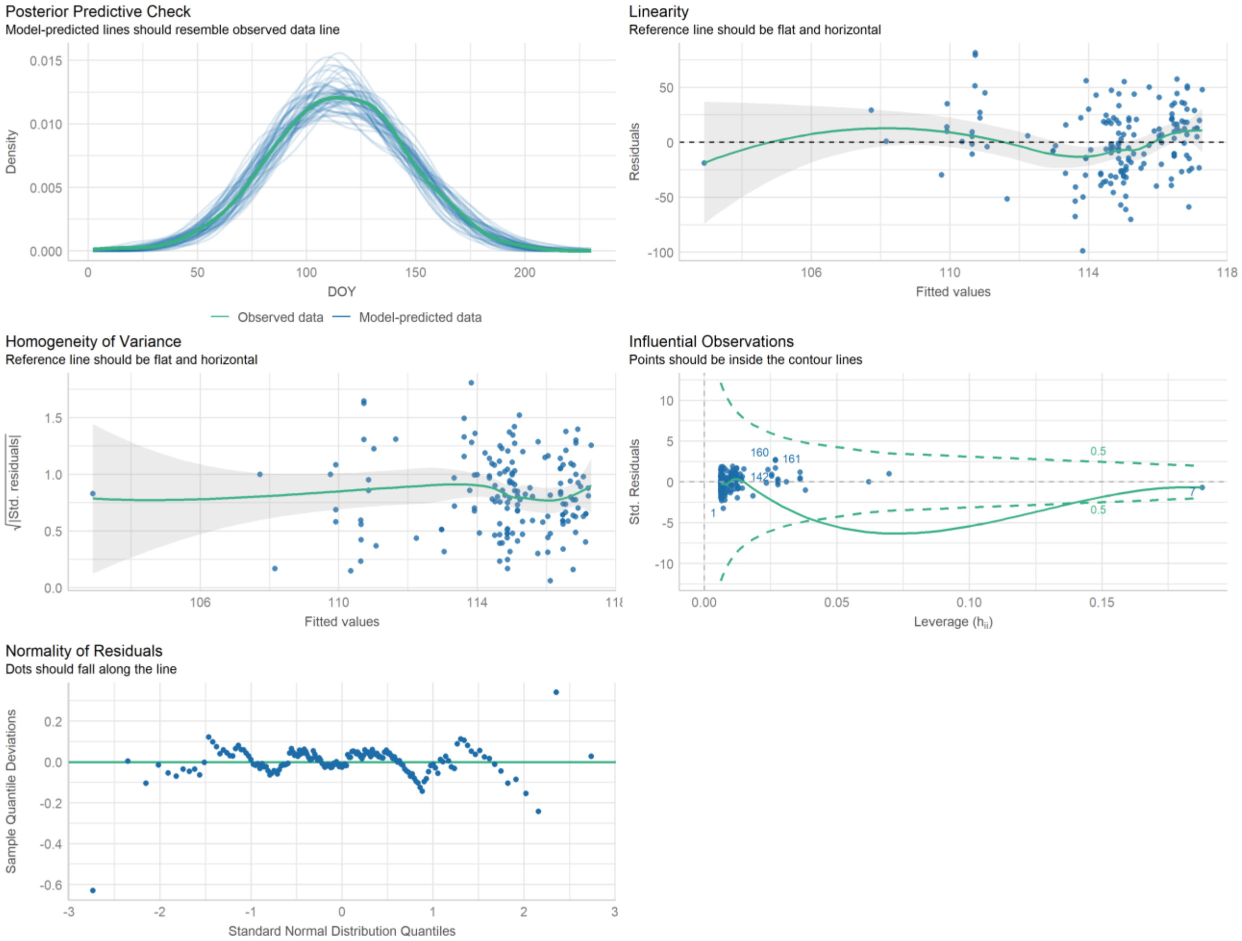

1.88. LM - DVG - Phlomis purpurea

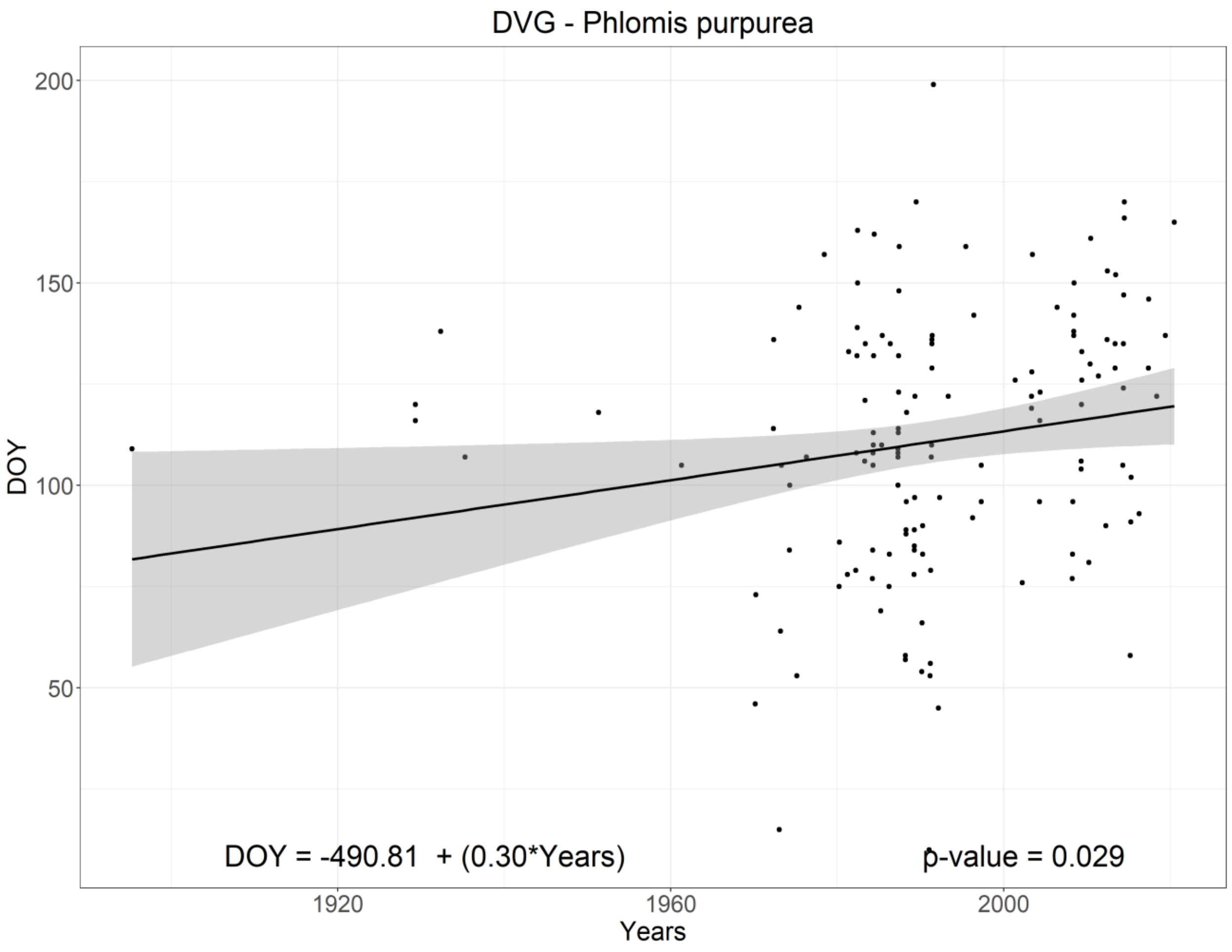

1.88.1. Diagnostics - LM - DVG - Phlomis purpurea

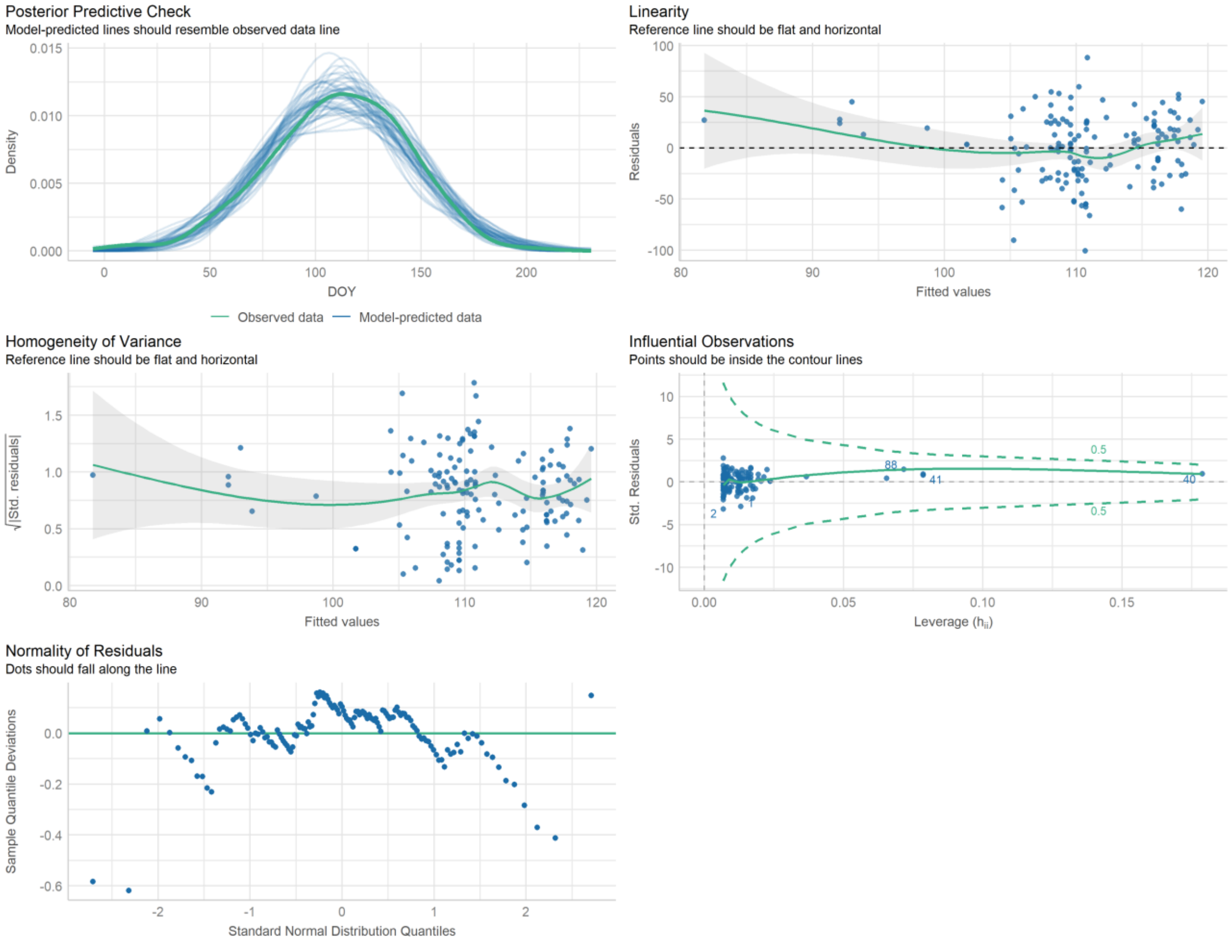

1.89. LM - F - Pistacia lentiscus

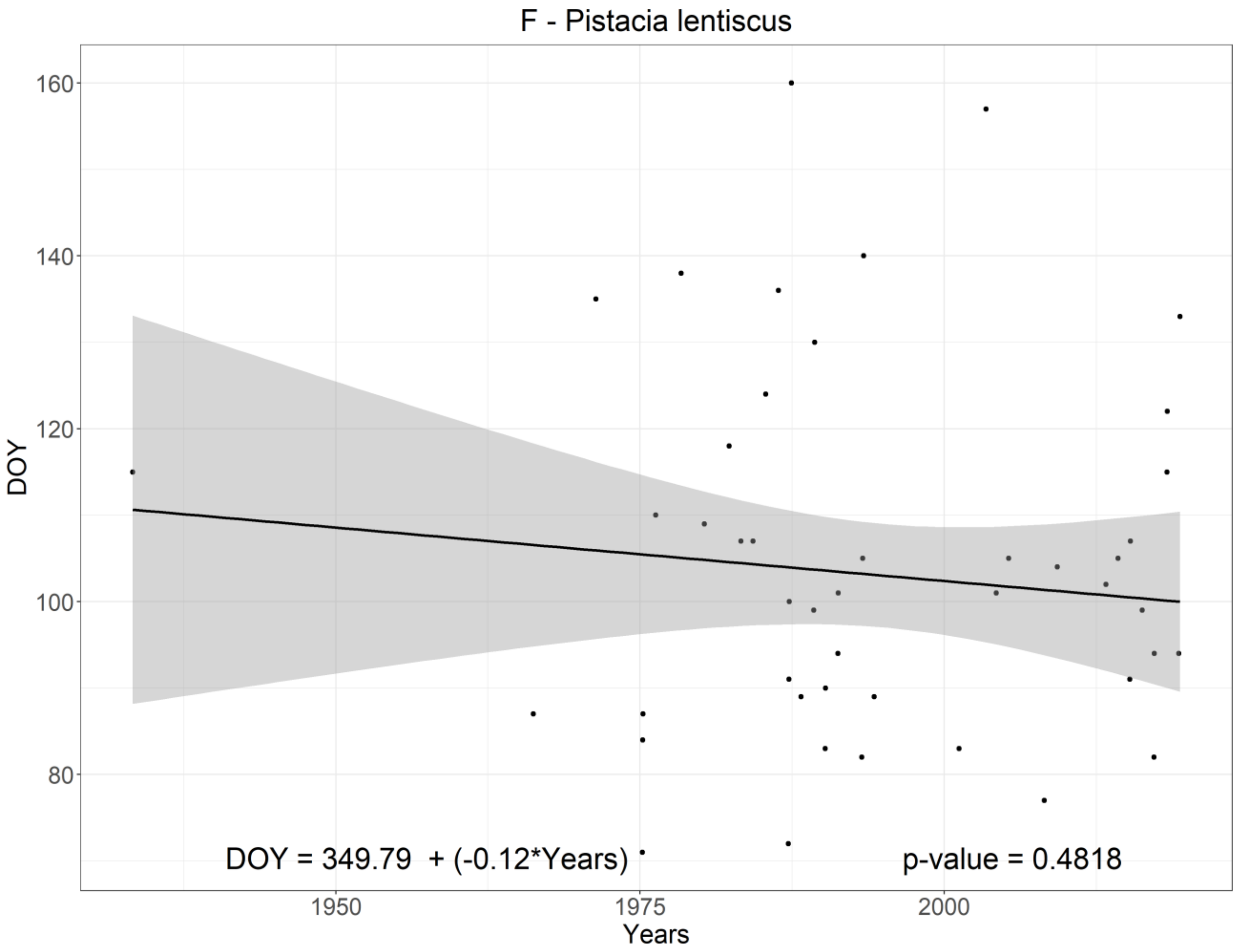

1.89.1. Diagnostics - LM - F - Pistacia lentiscus

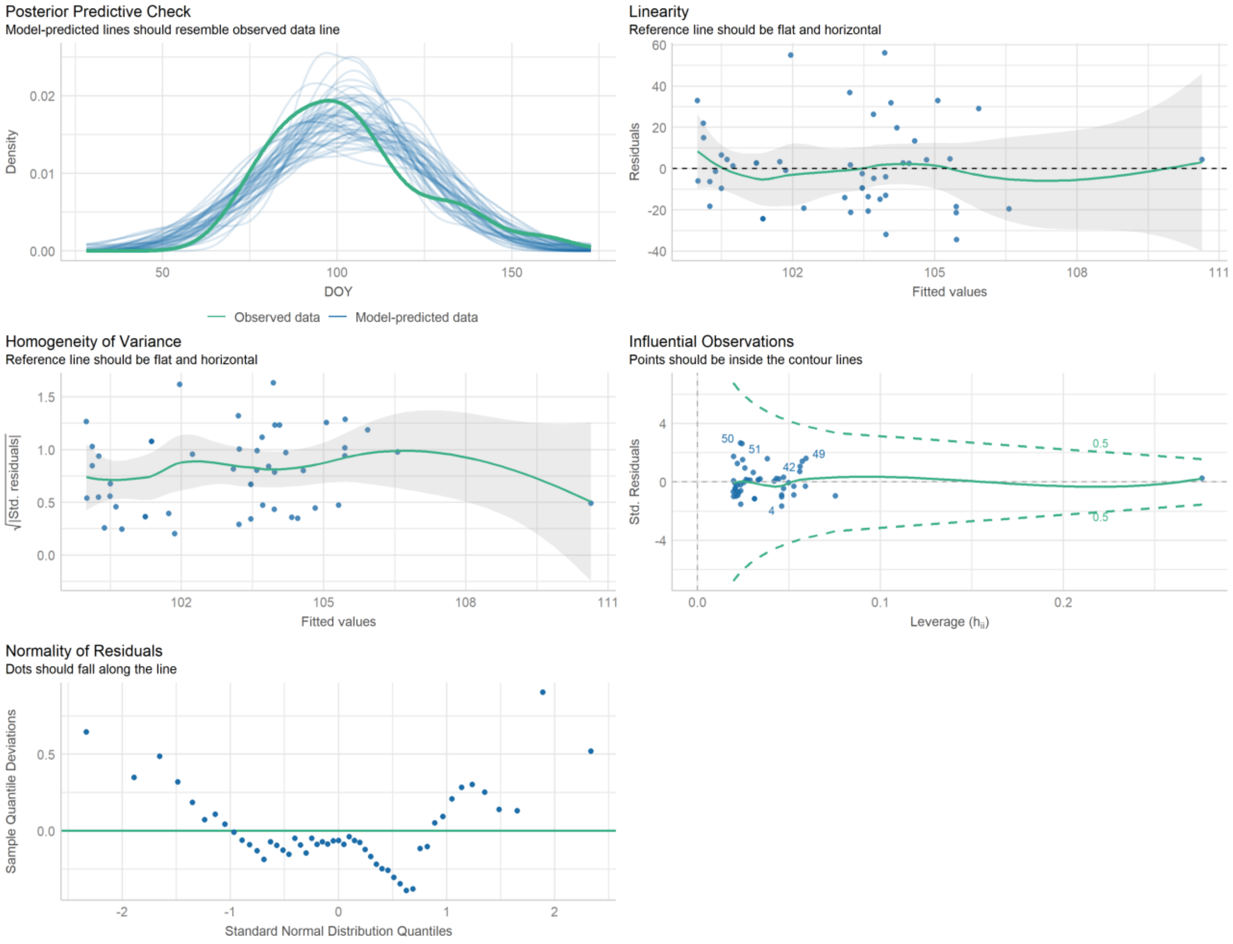

1.90. LM - F - Prunus prostrata

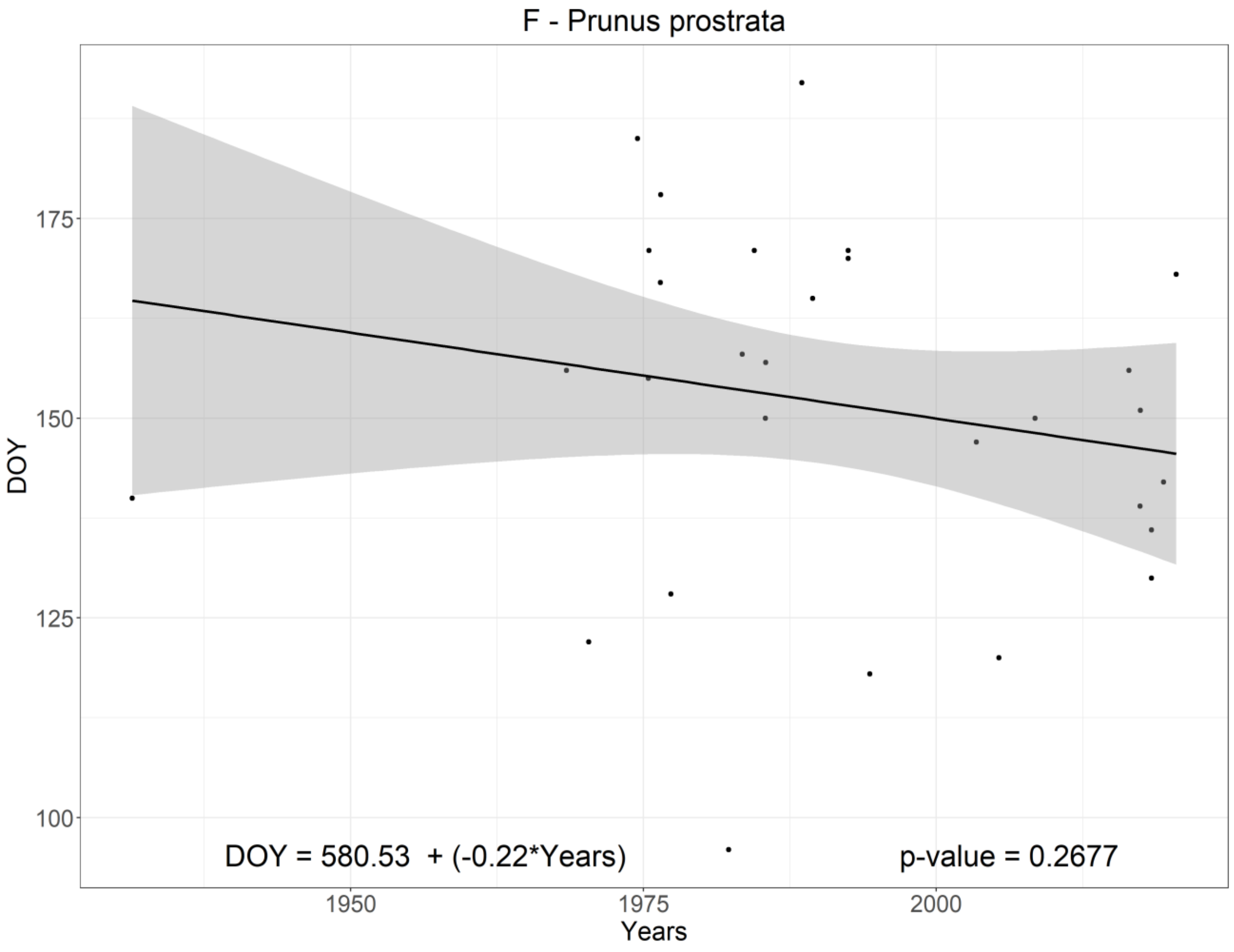

1.90.1. Diagnostics - LM - F - Prunus prostrata

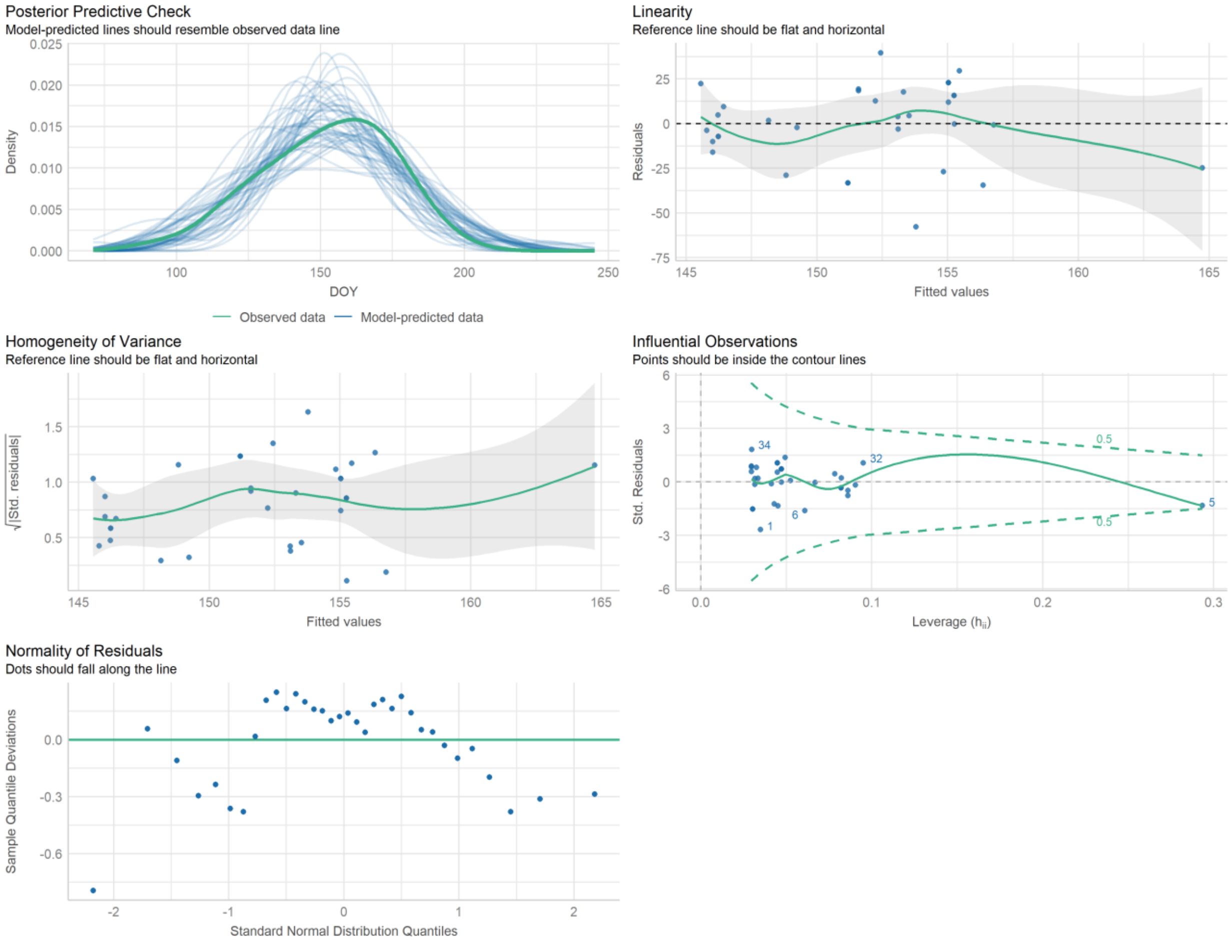

1.91. LM - DVG - Prunus prostrata

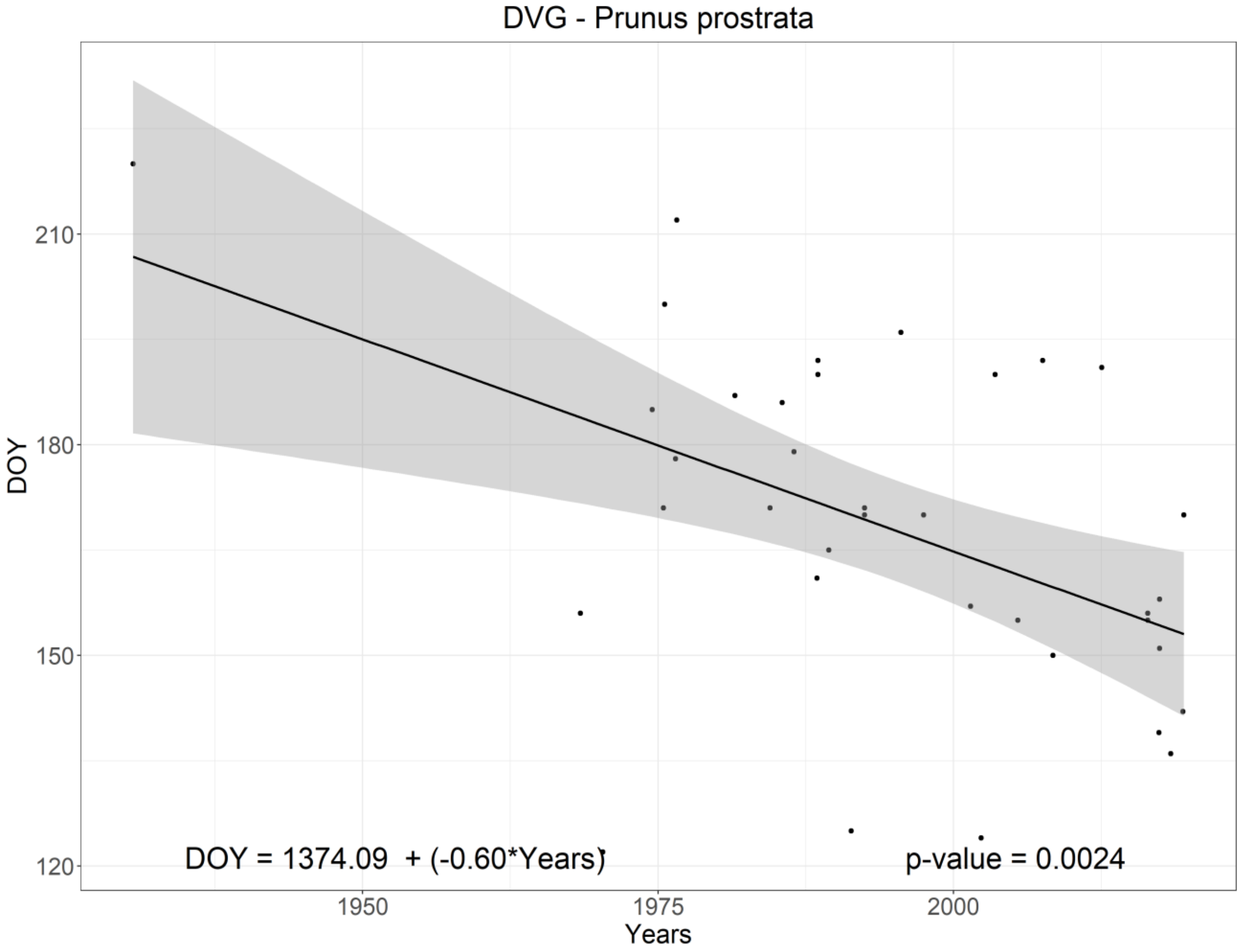

1.91.1. Diagnostics - LM - DVG - Prunus prostrata

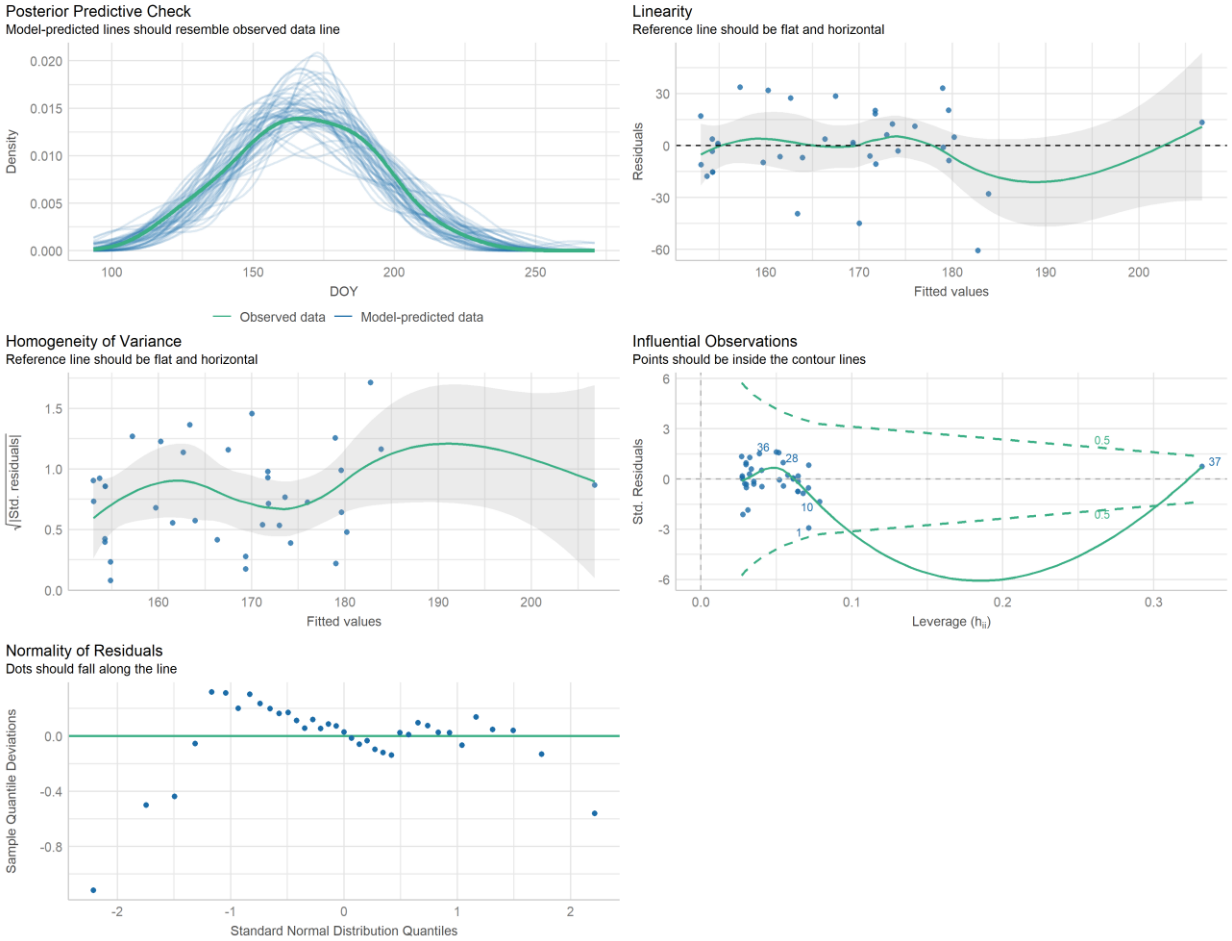

1.92. LM - F - Quercus coccifera

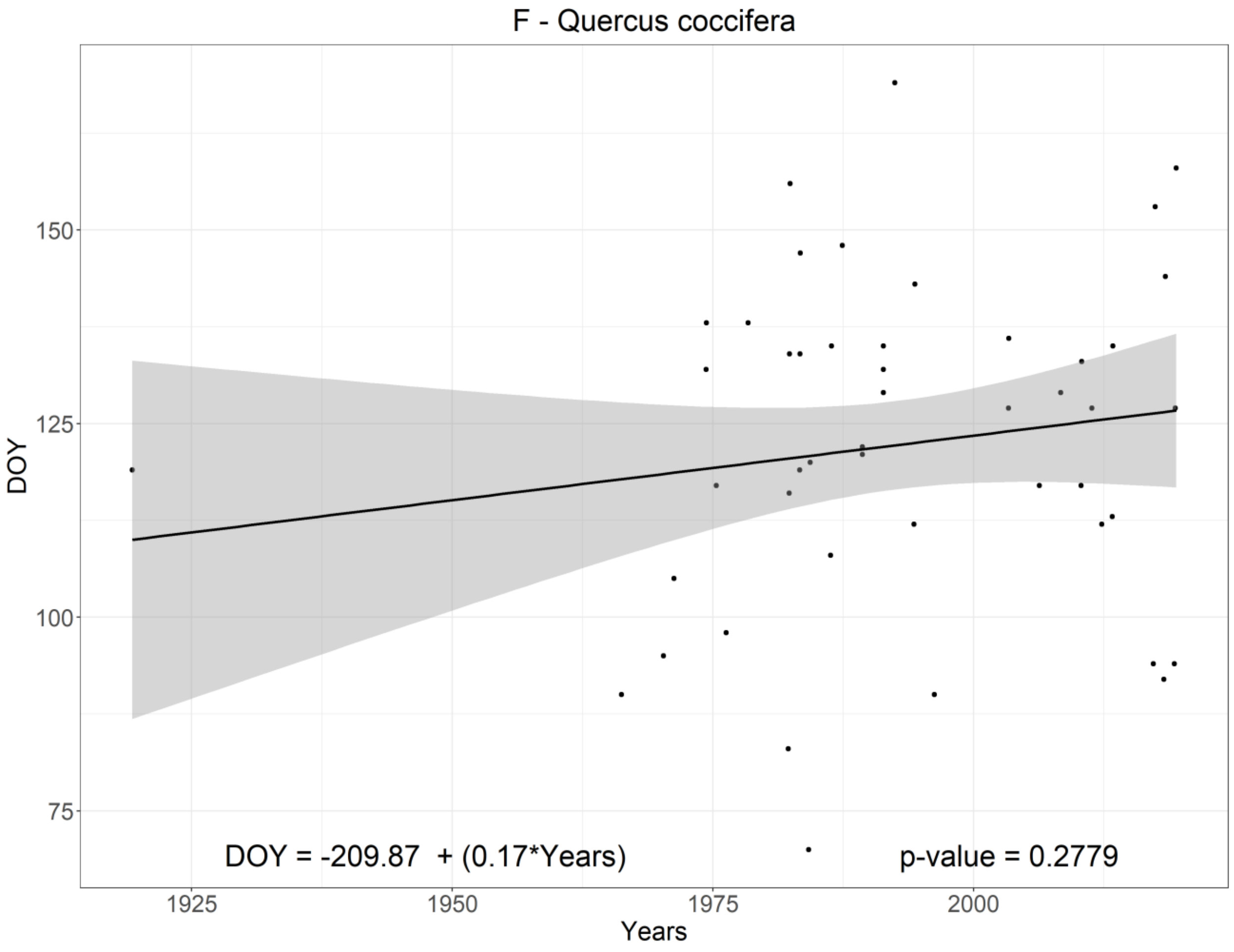

1.92.1. Diagnostics - LM - F - Quercus coccifera

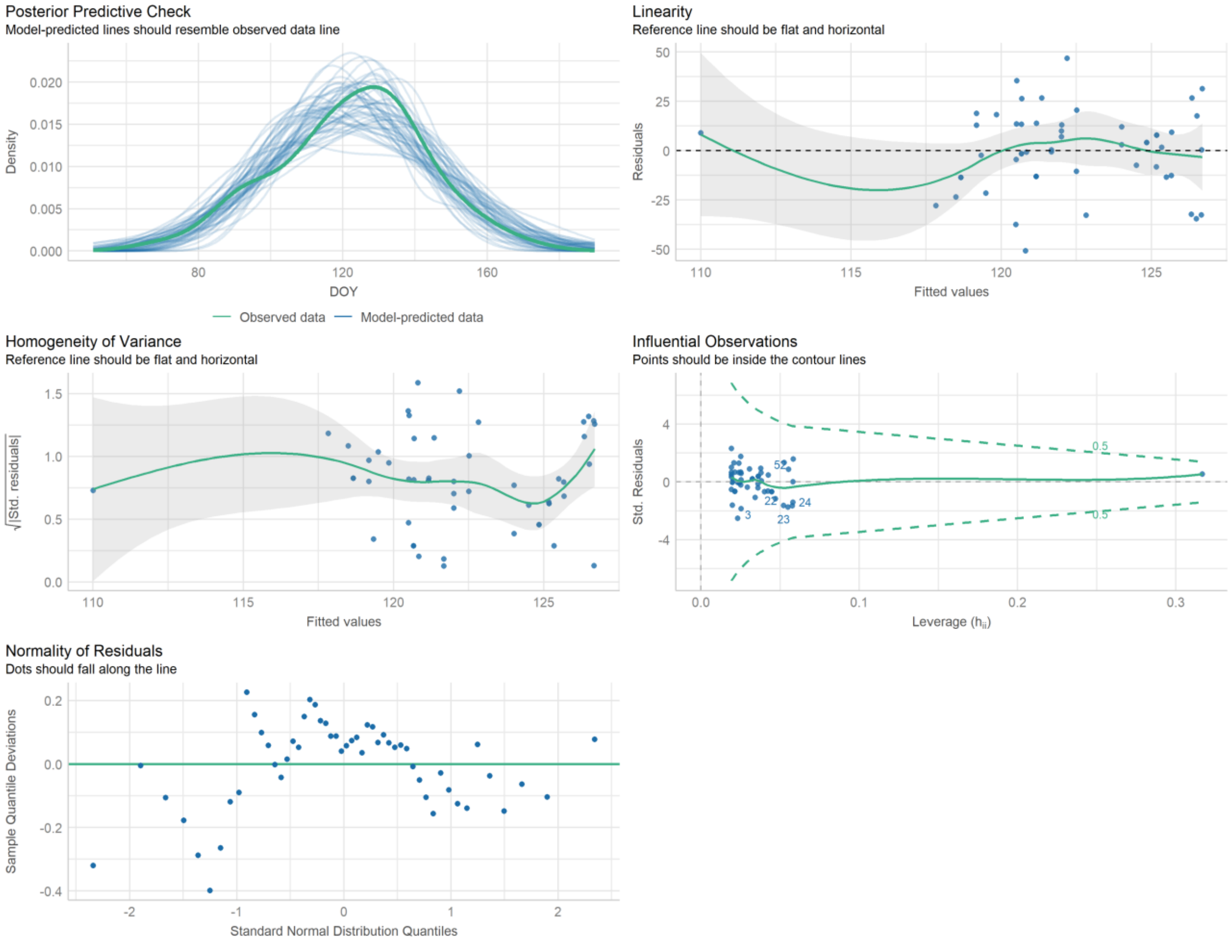

1.93. LM - DVG - Quercus coccifera

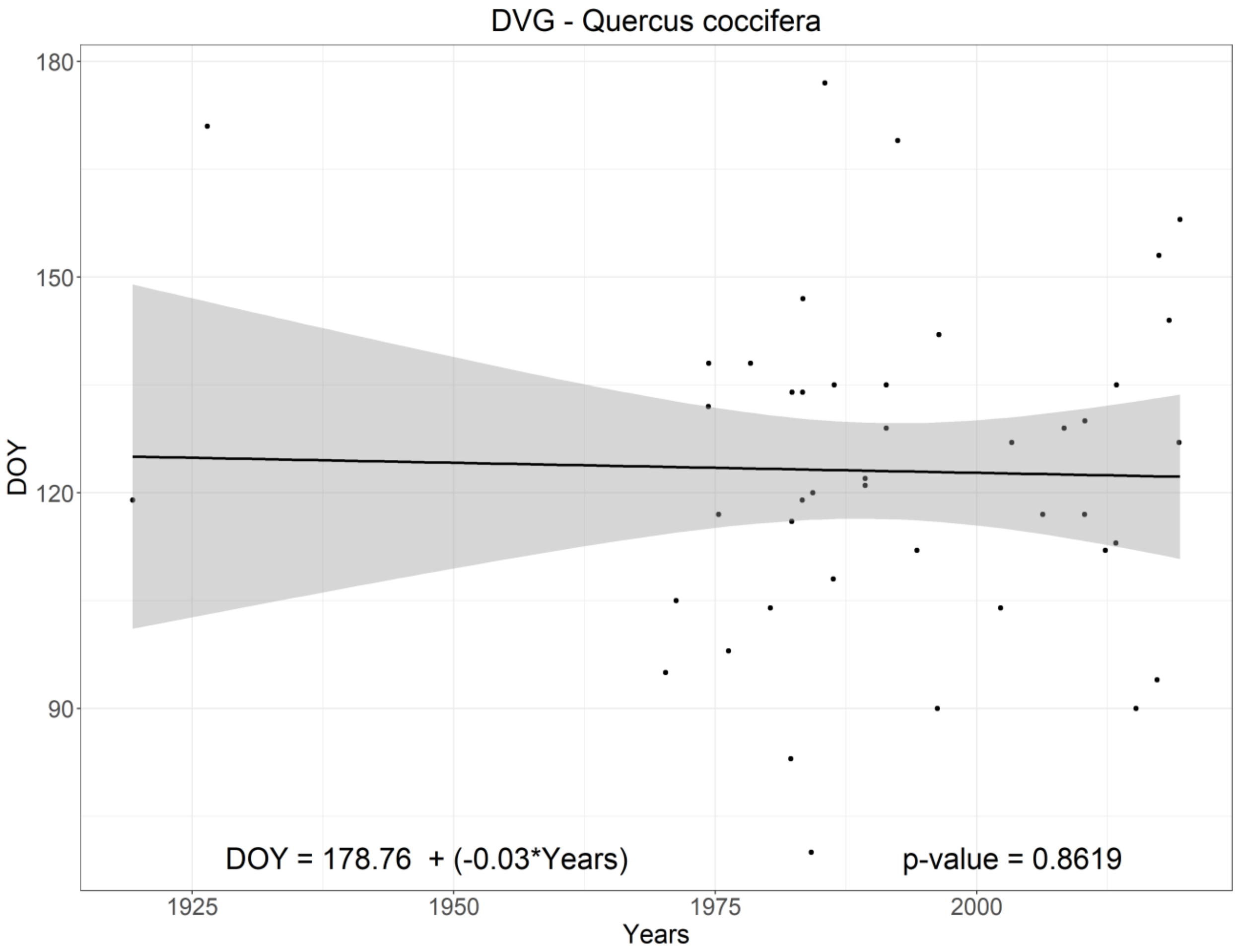

1.93.1. Diagnostics - LM - DVG - Quercus coccifera

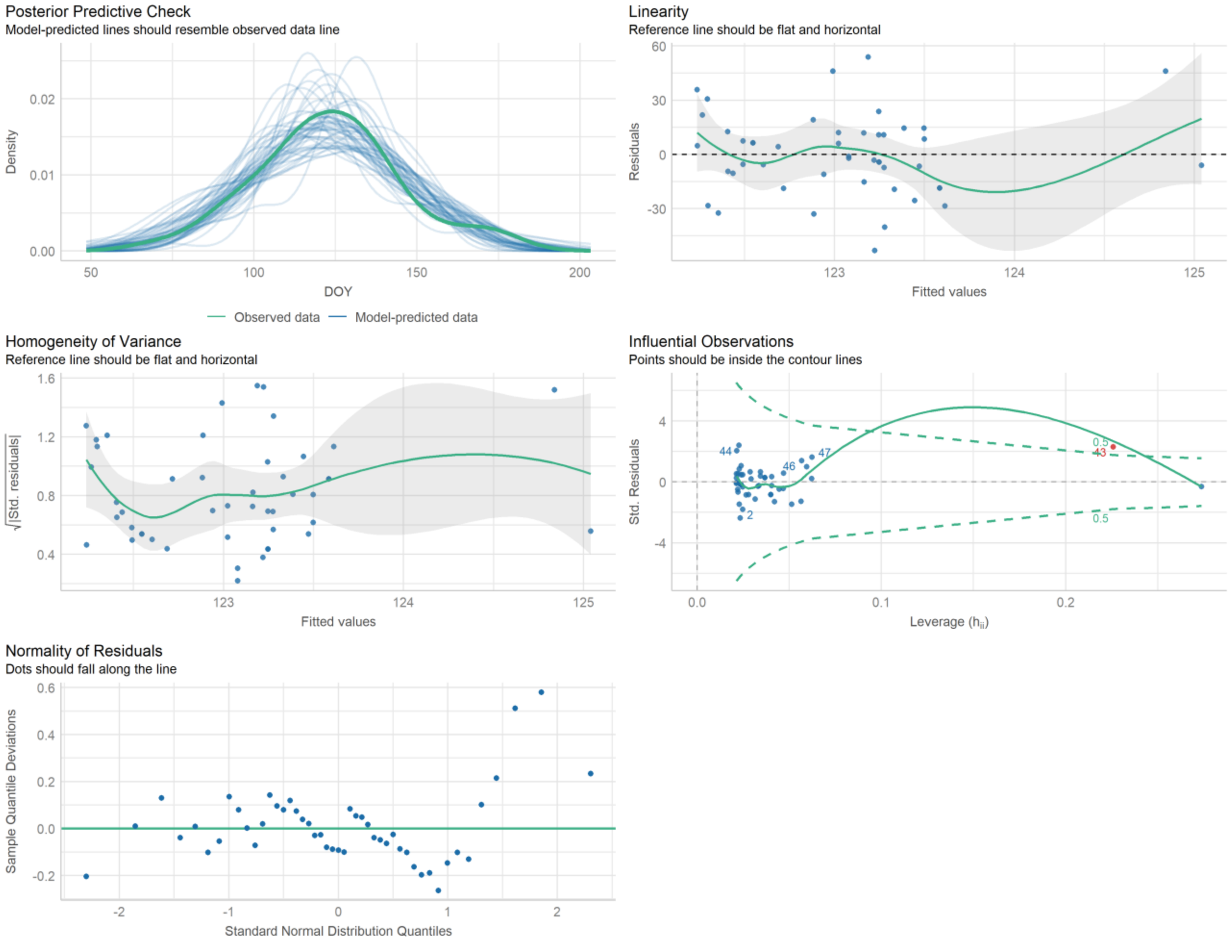

1.94. LM - F - Quercus faginea

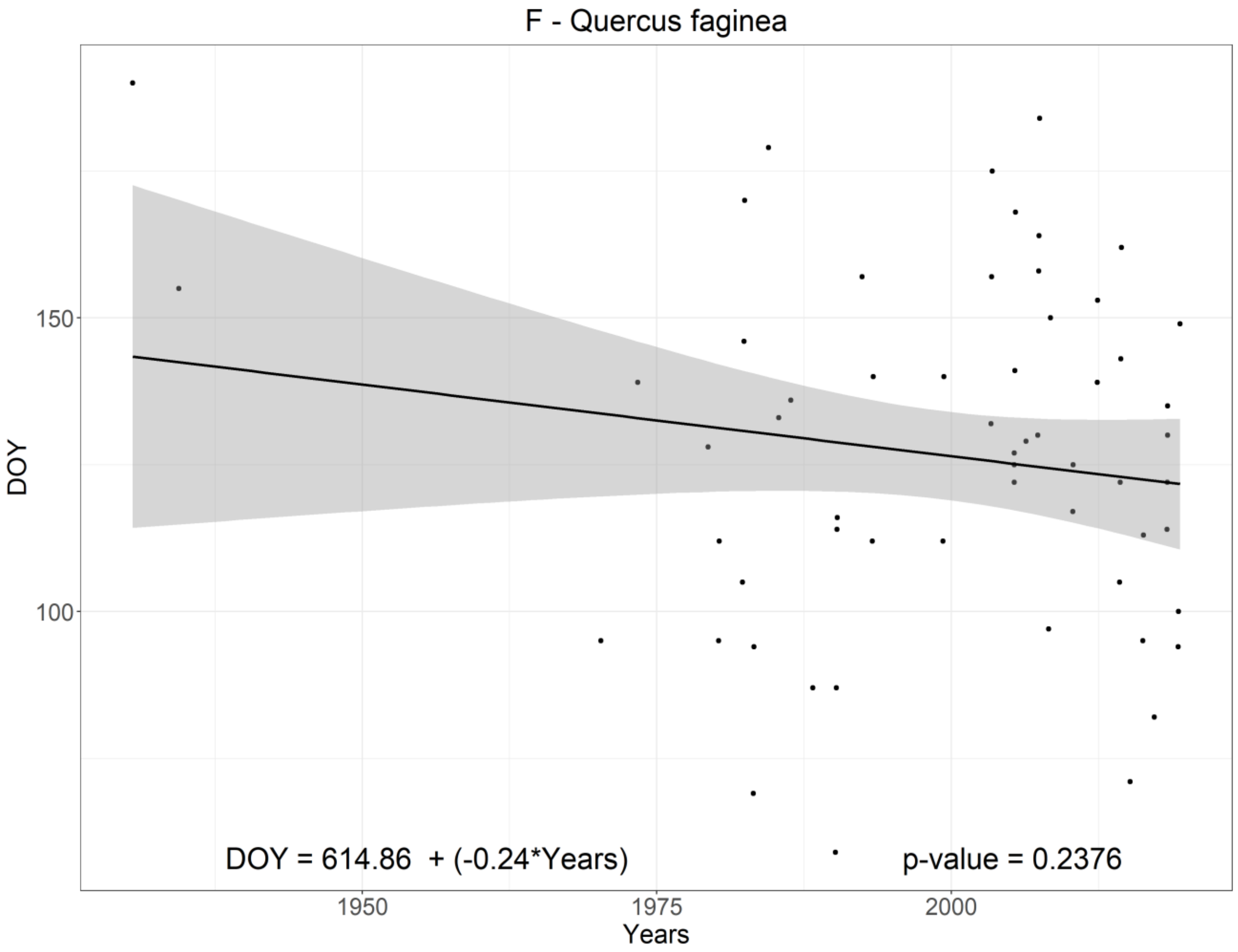

1.94.1. Diagnostics - LM - F - Quercus faginea

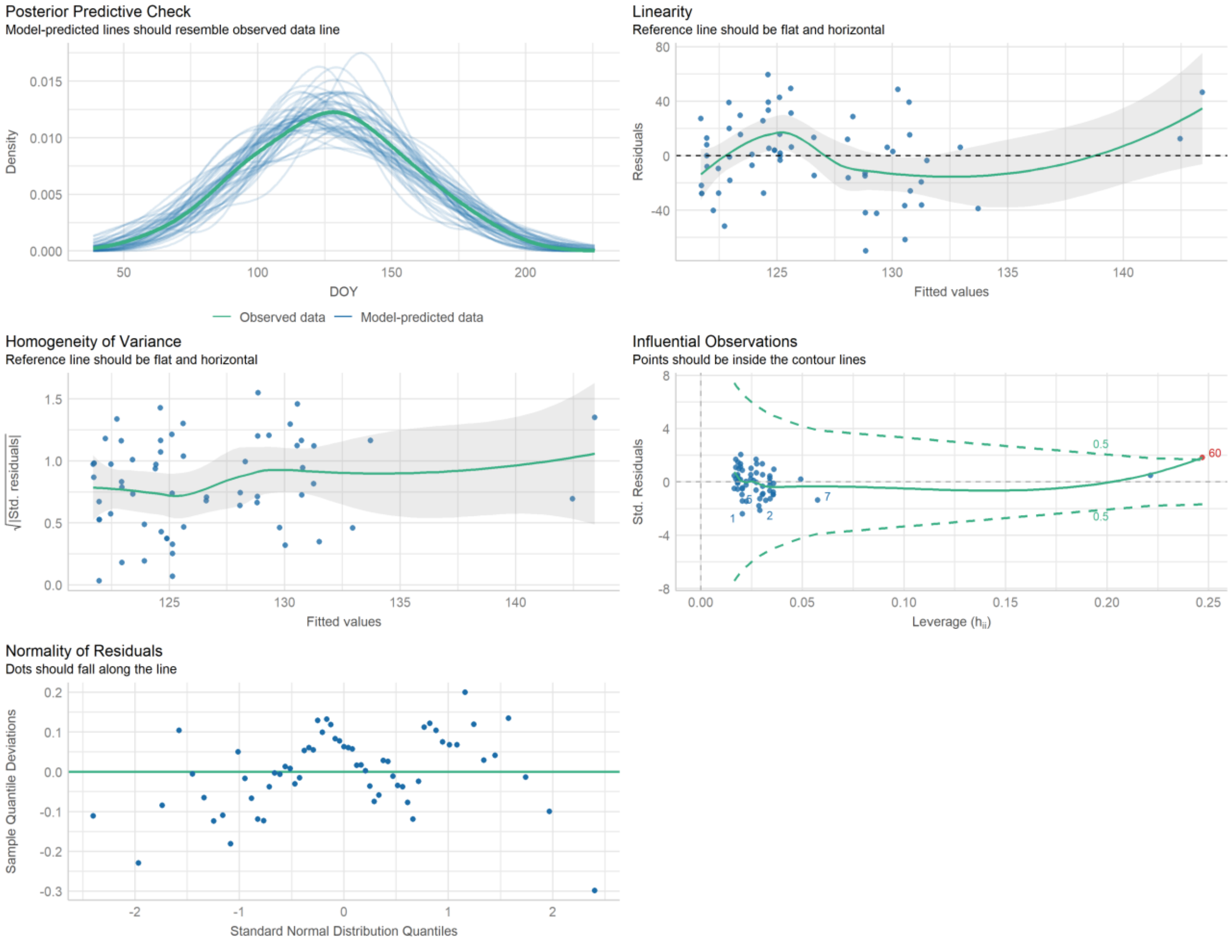

1.95. LM - DVG - Quercus faginea

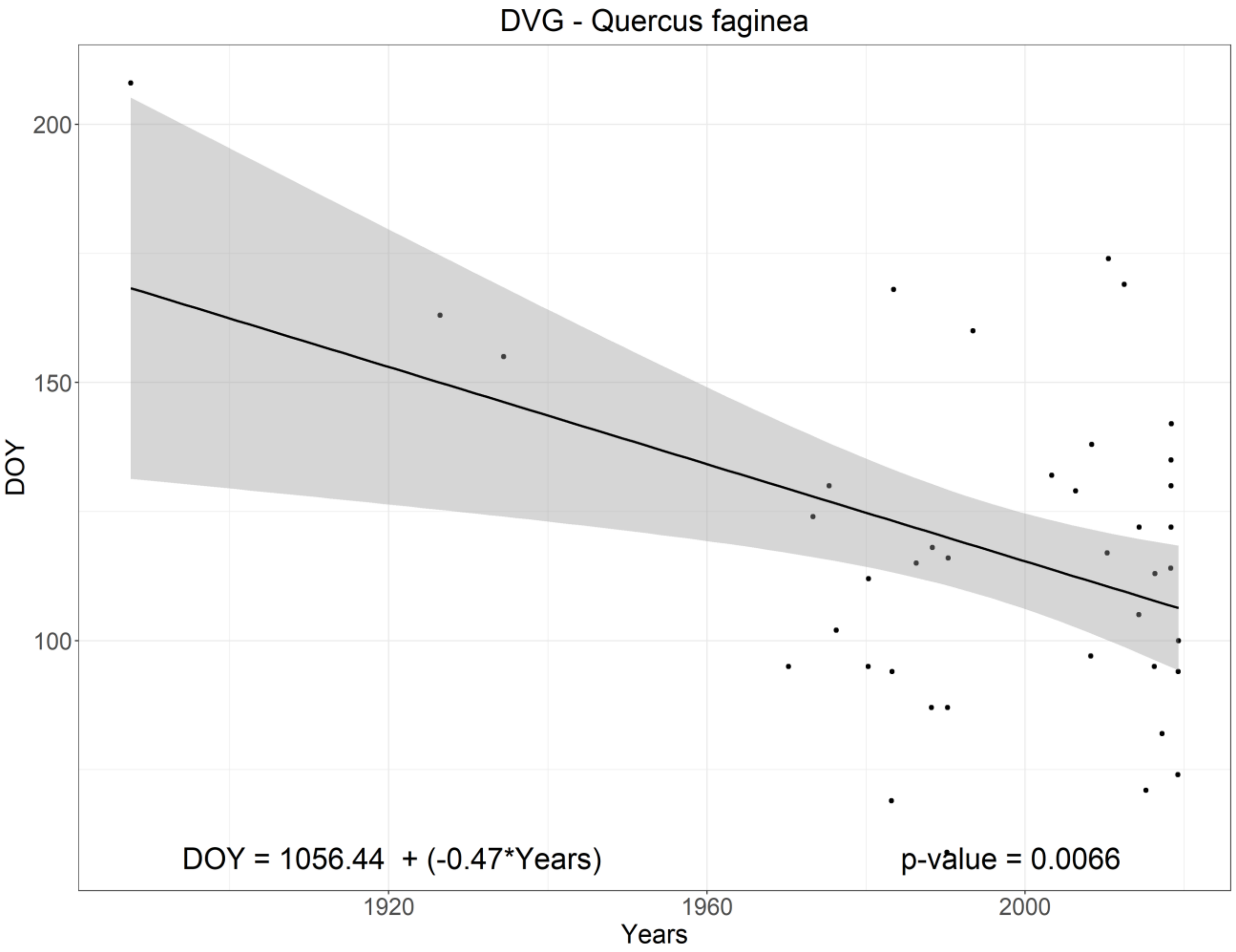

1.95.1. Diagnostics - LM - DVG - Quercus faginea

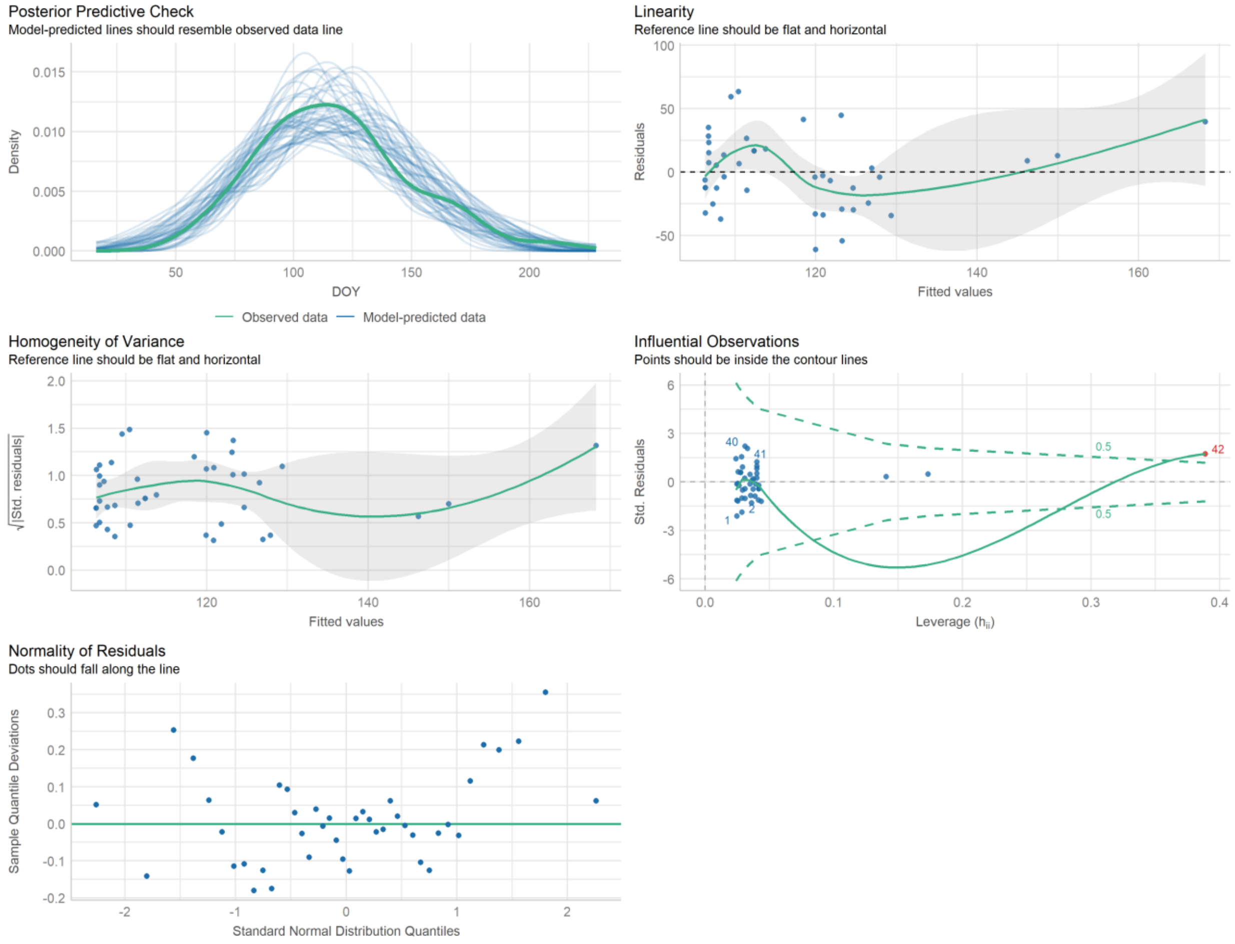

1.96. LM - F - Quercus rotundifolia

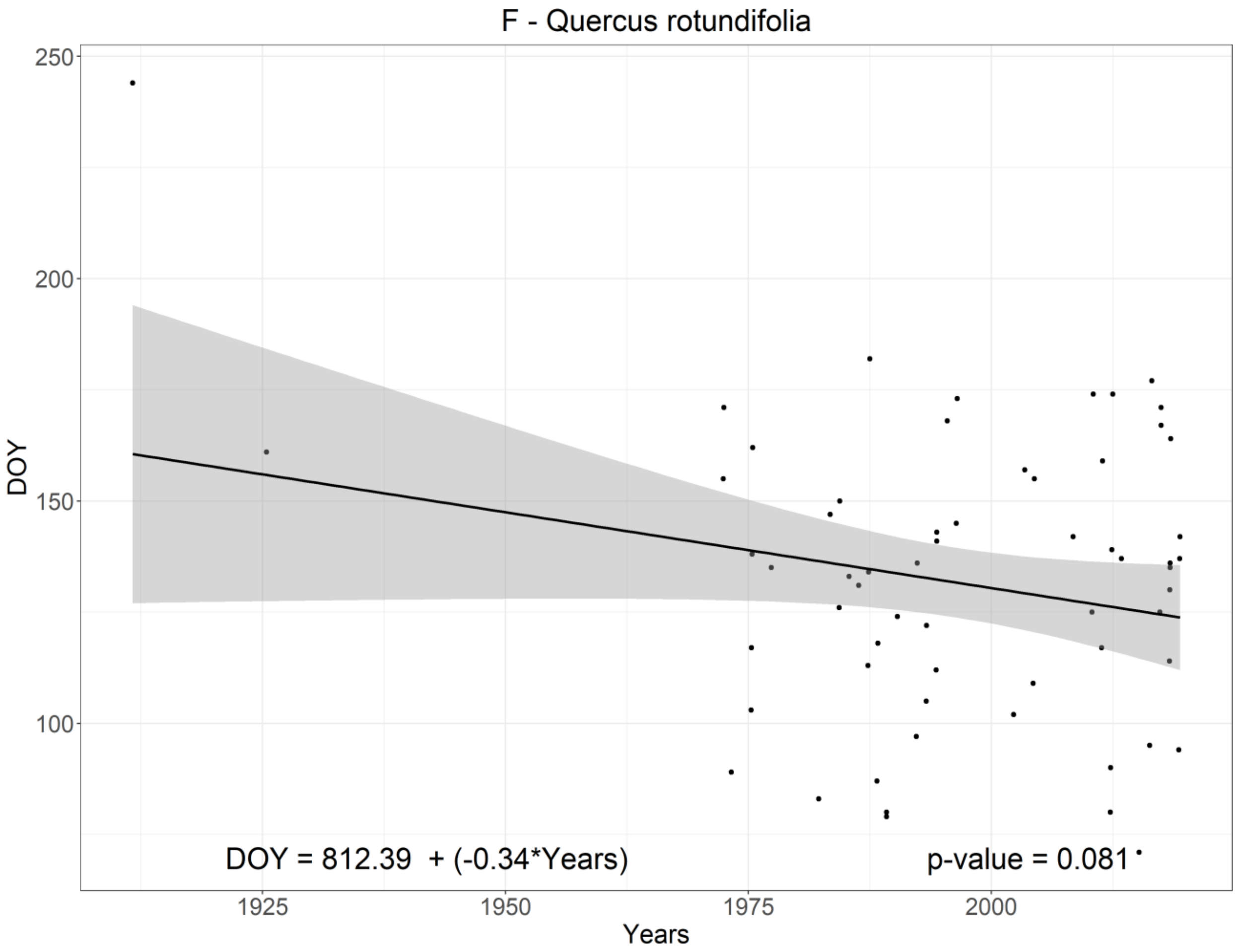

1.96.1. Diagnostics - LM - F - Quercus rotundifolia

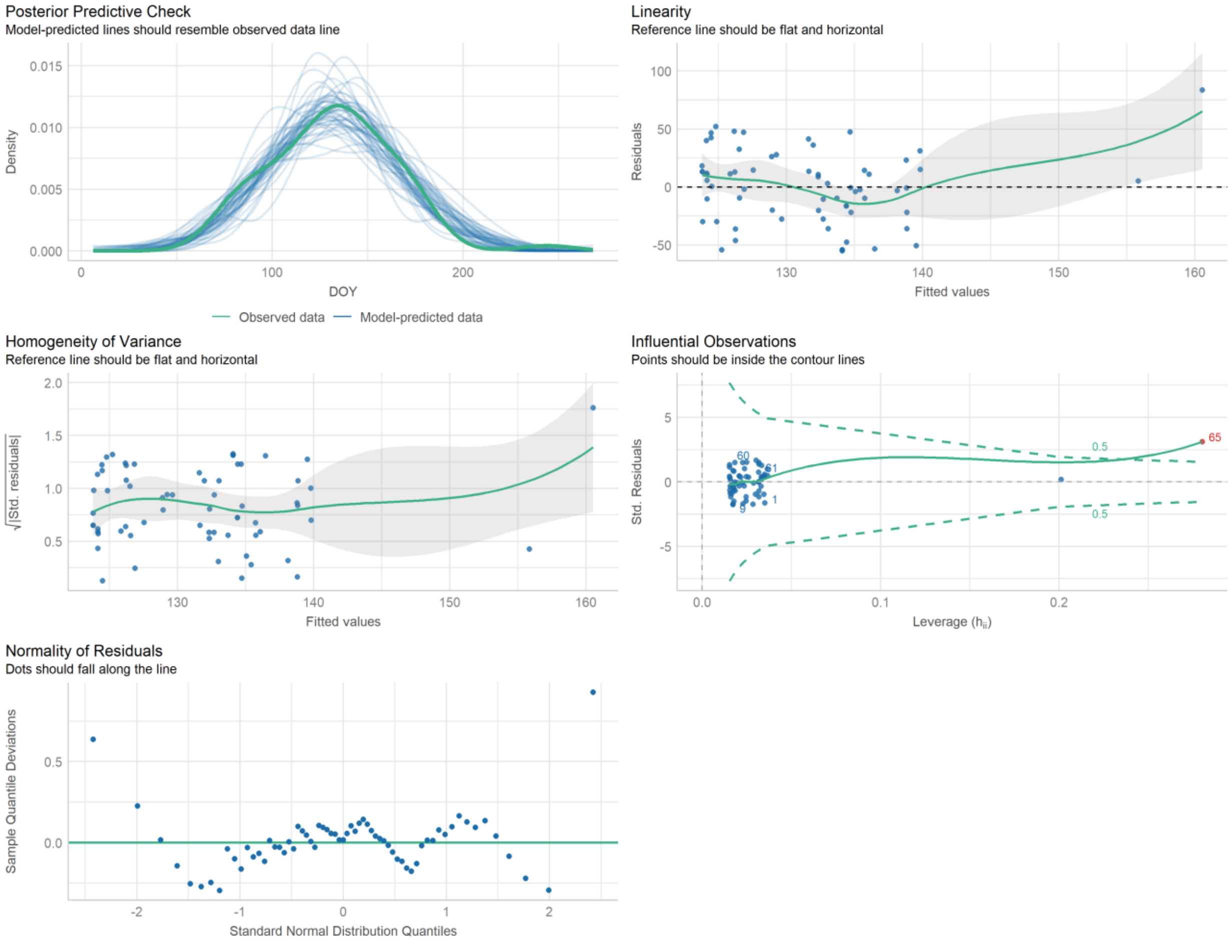

1.97. LM - DVG - Quercus rotundifolia

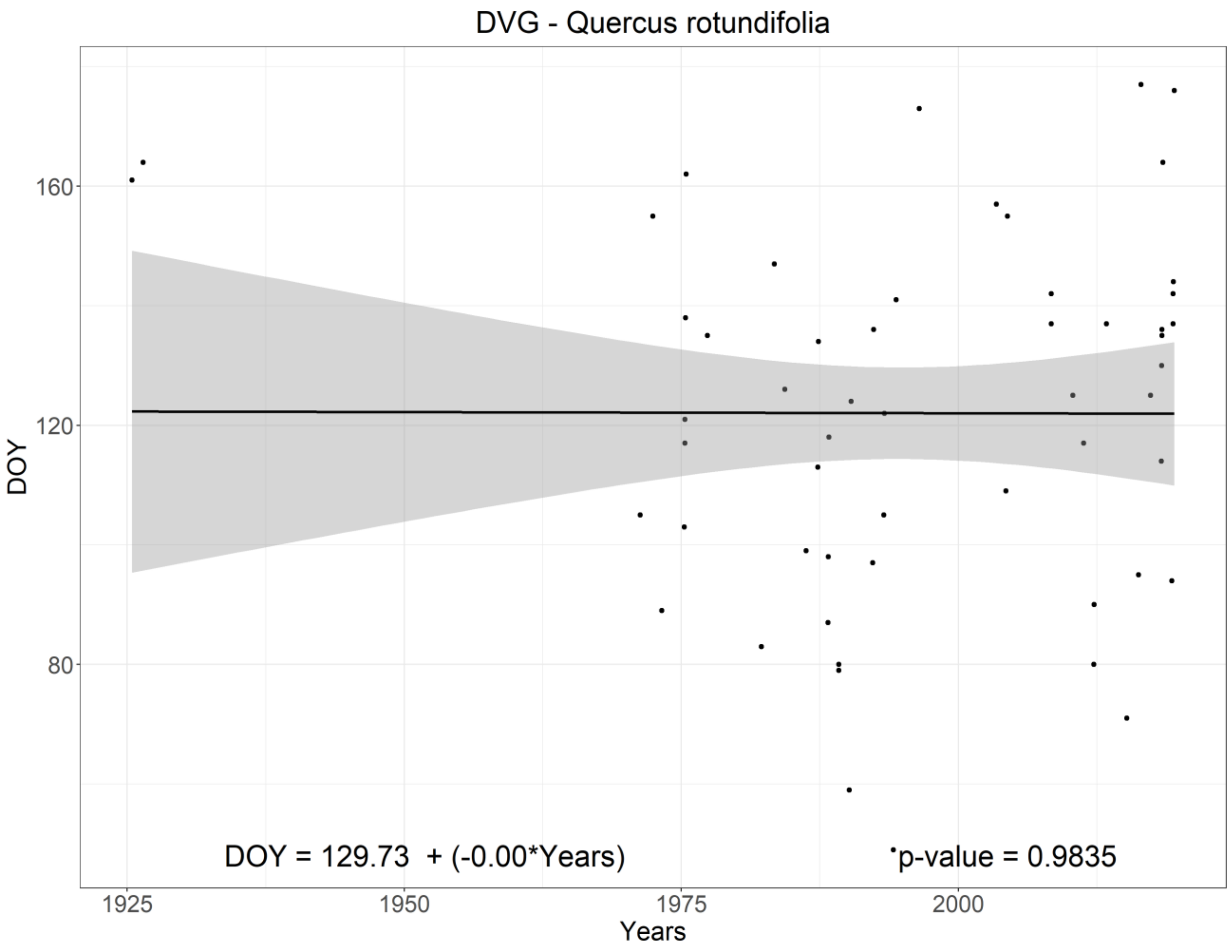

1.97.1. Diagnostics - LM - DVG - Quercus rotundifolia

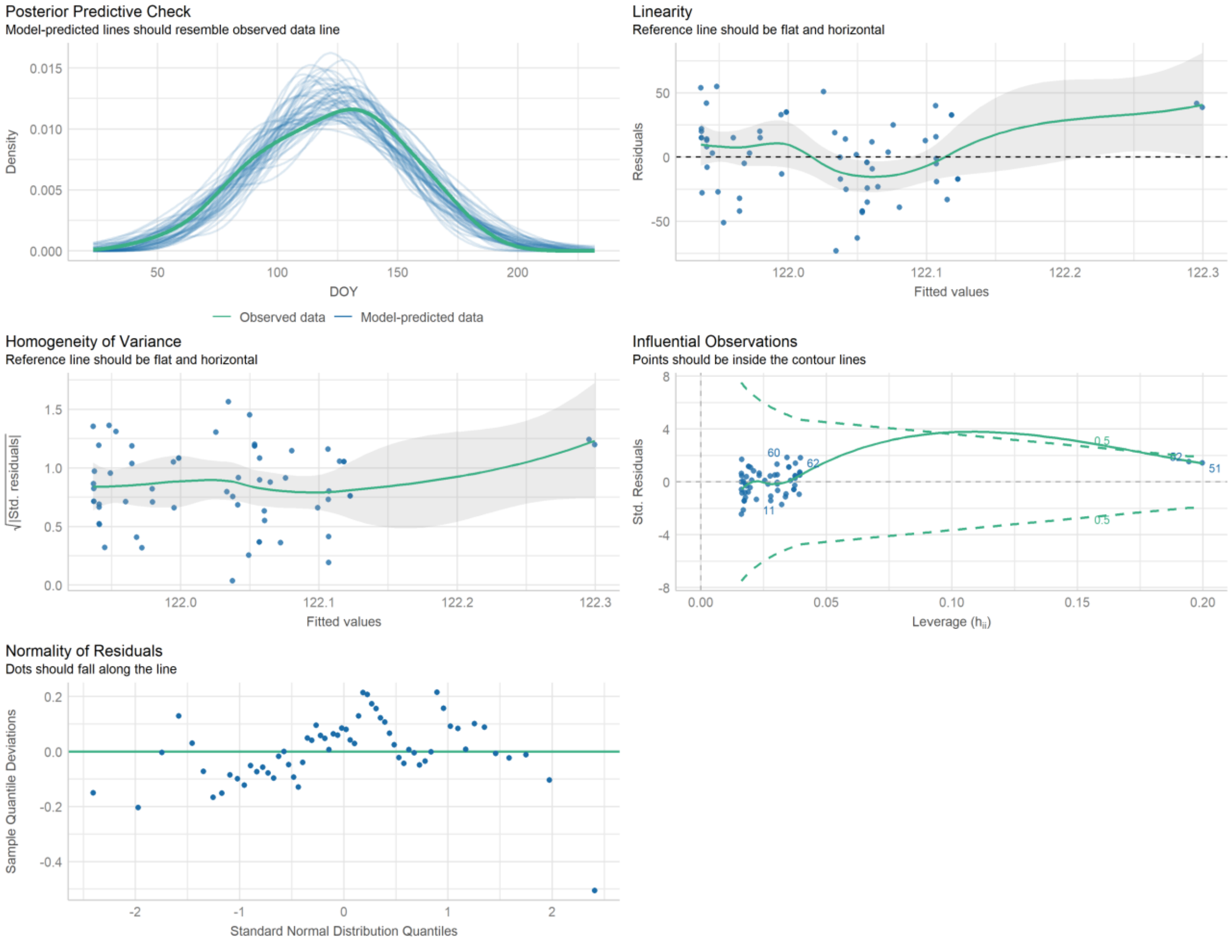

1.98. LM - F - Quercus suber

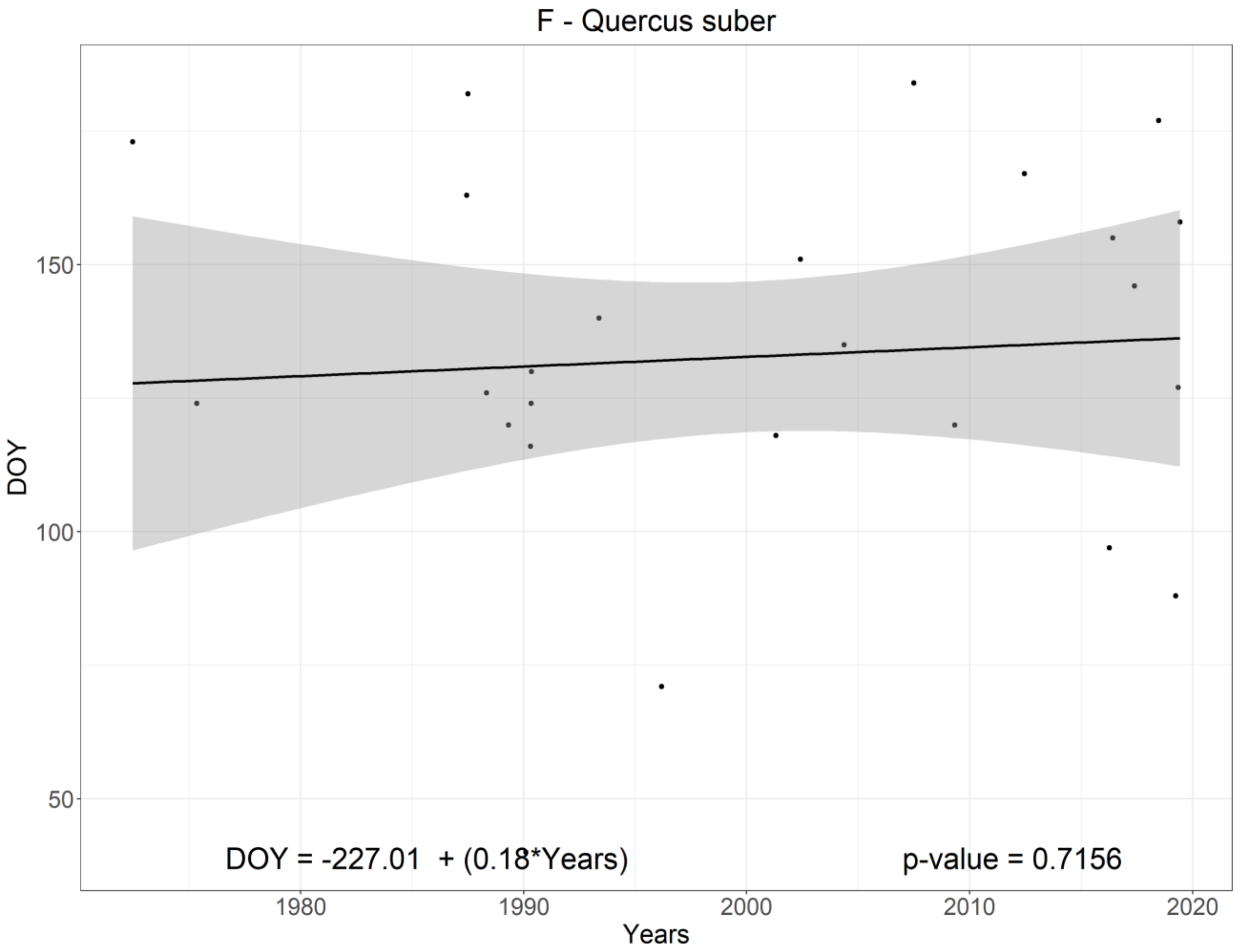

1.98.1. Diagnostics - LM - F - Quercus suber

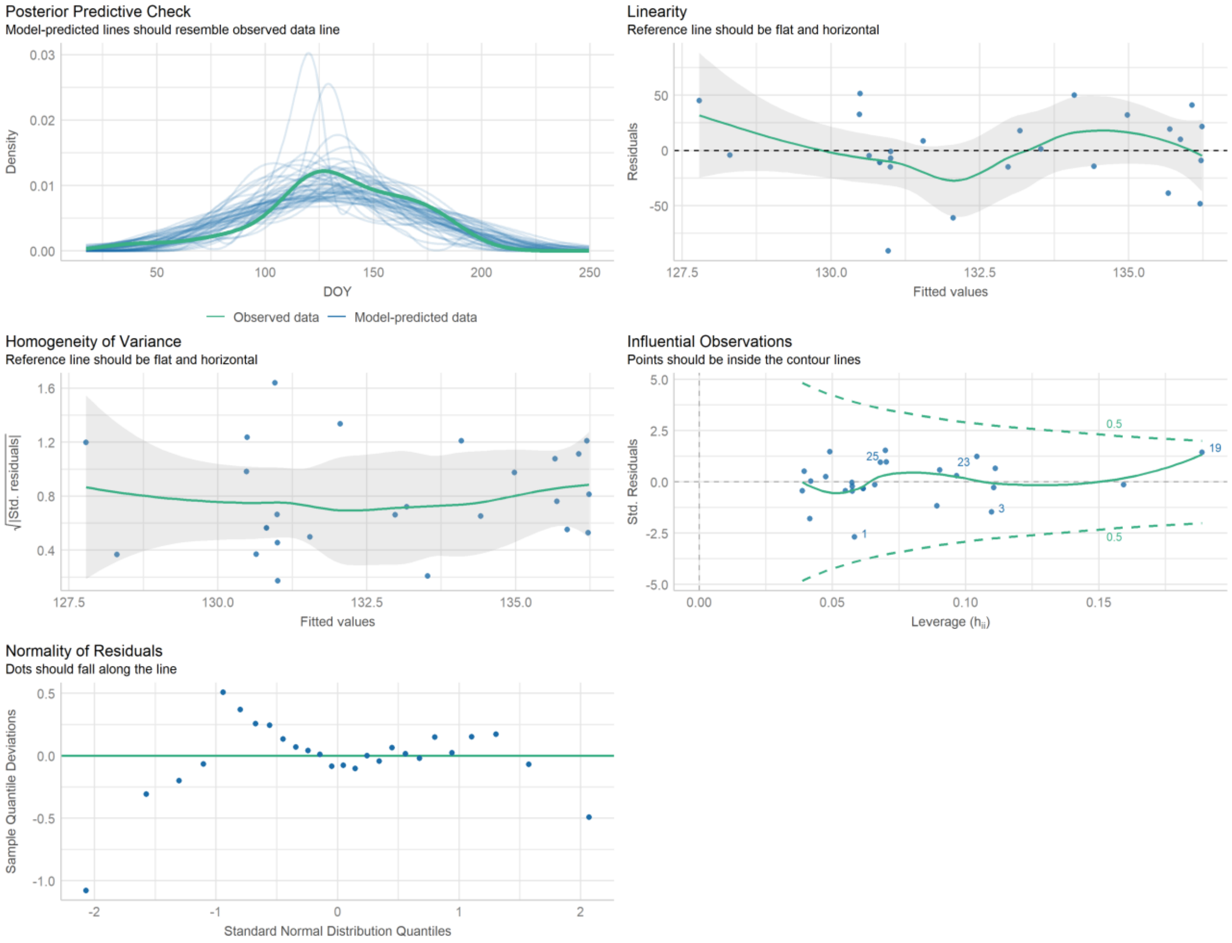

1.99. LM - DVG - Quercus suber

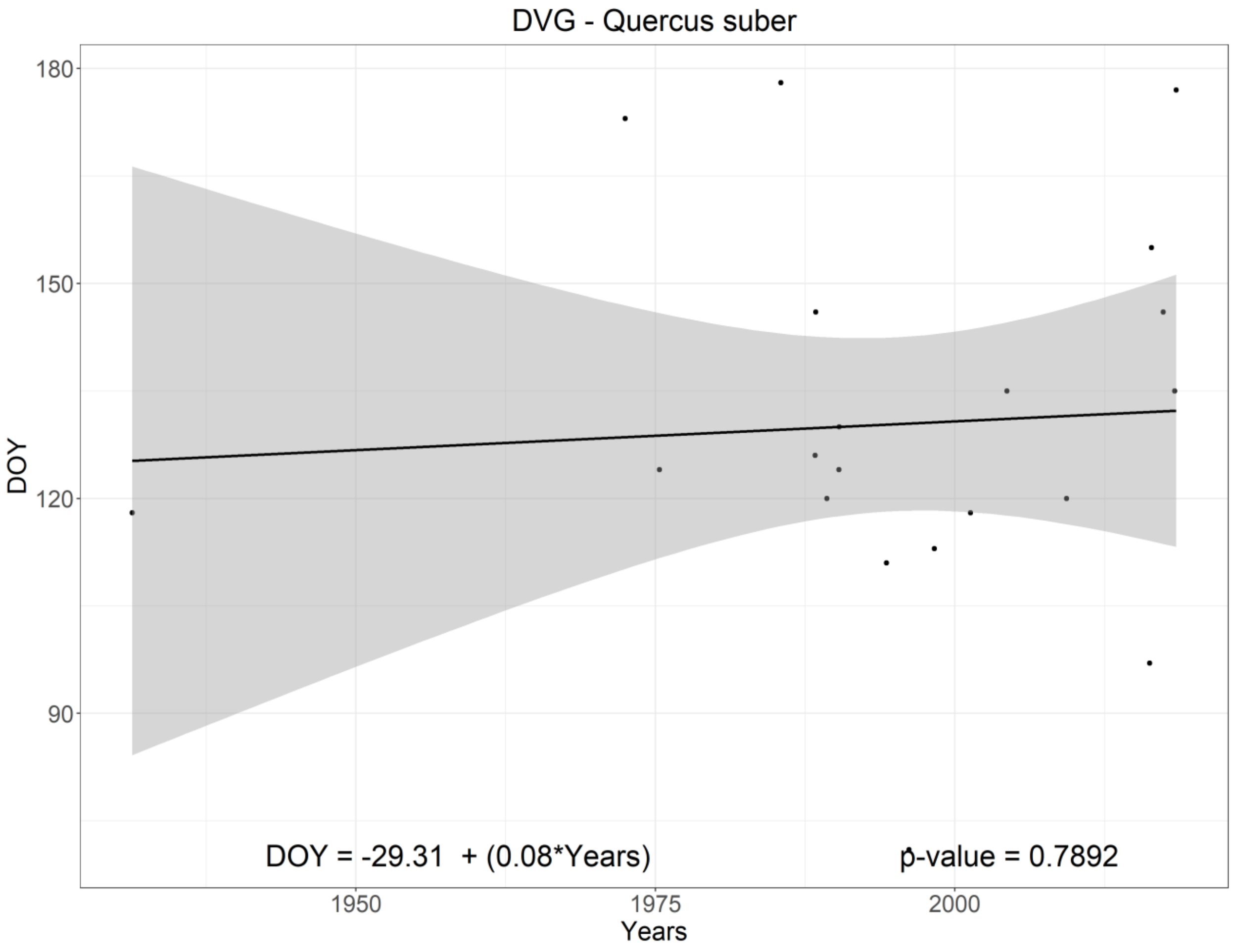

1.99.1. Diagnostics - LM - DVG - Quercus suber

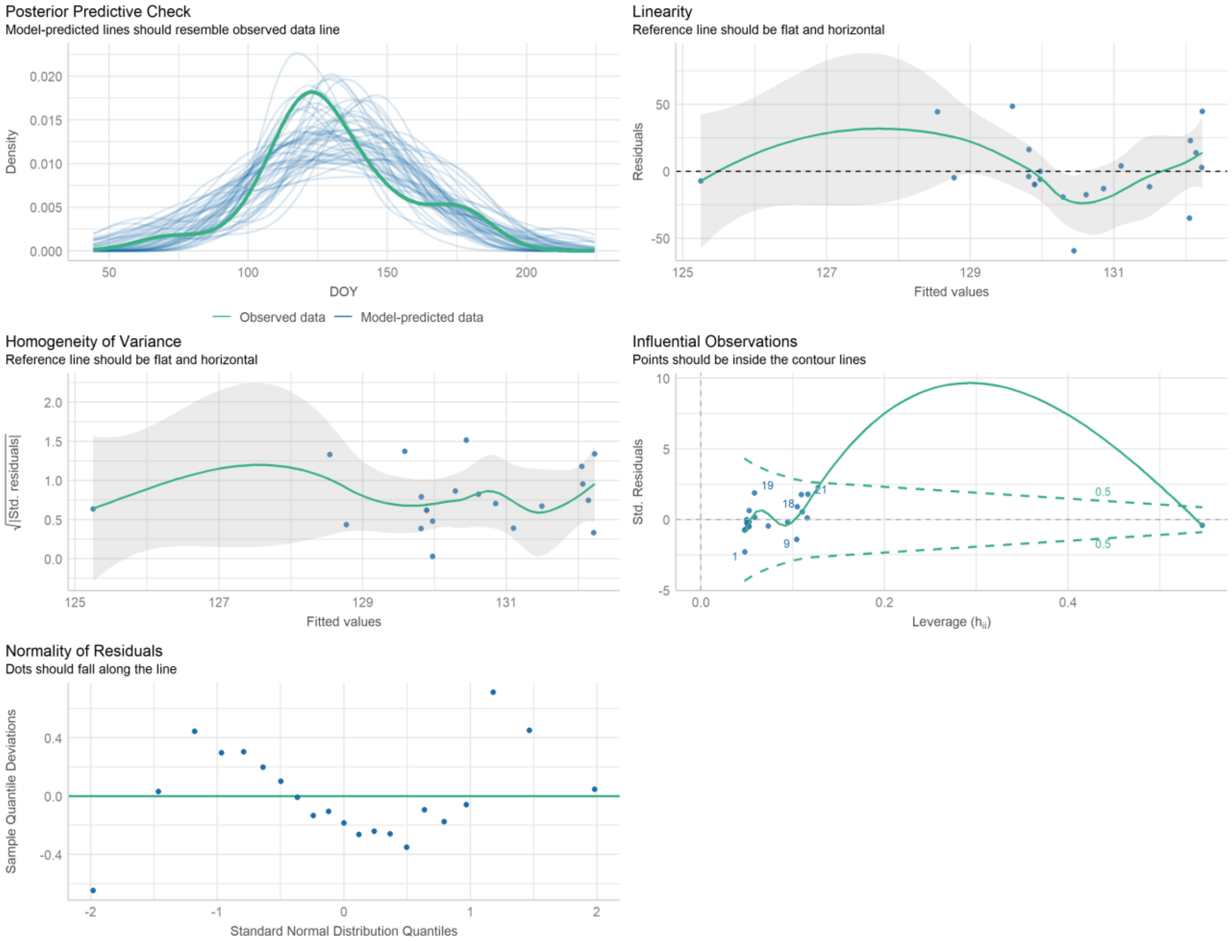

1.100. LM - F - Retama sphaerocarpa

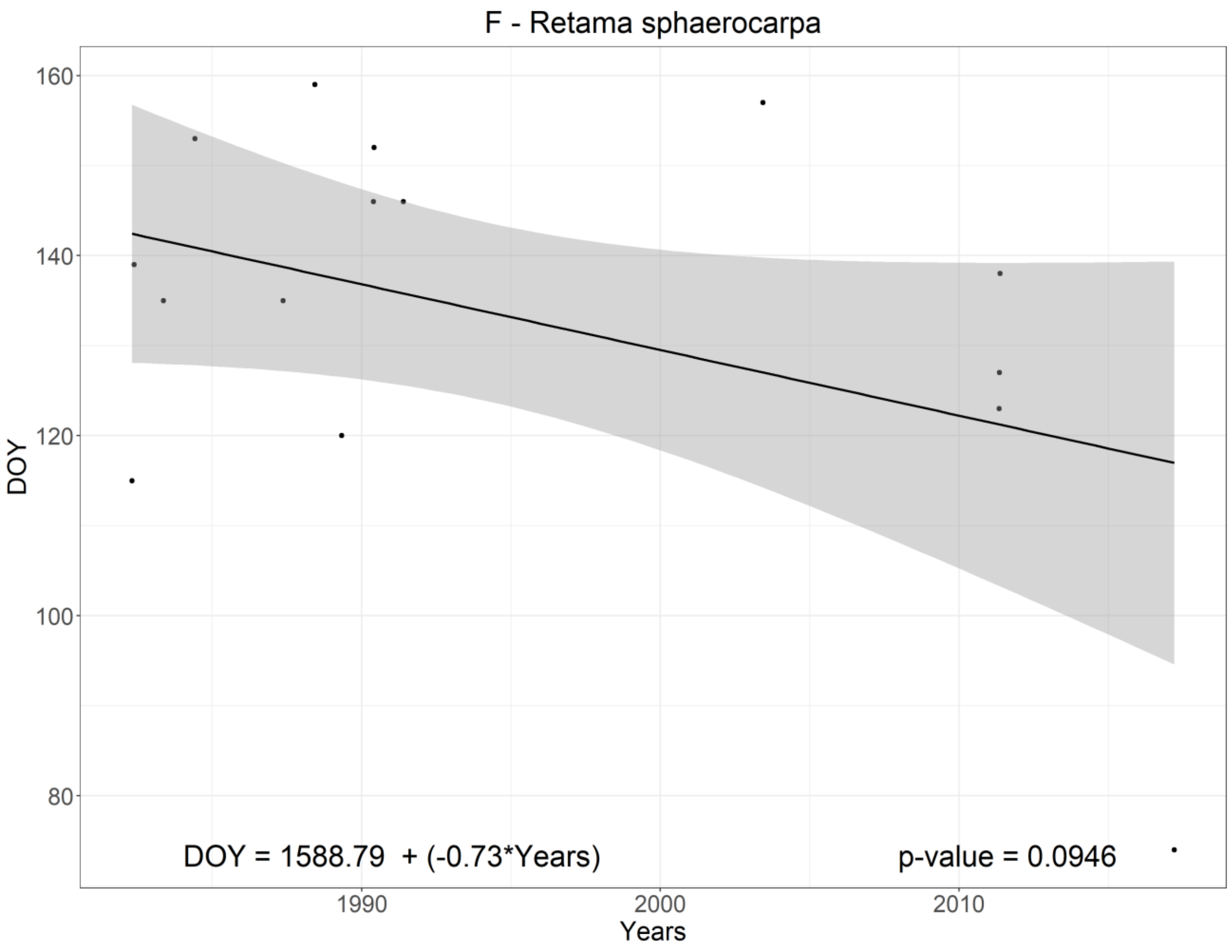

1.100.1. Diagnostics - LM - F - Retama sphaerocarpa

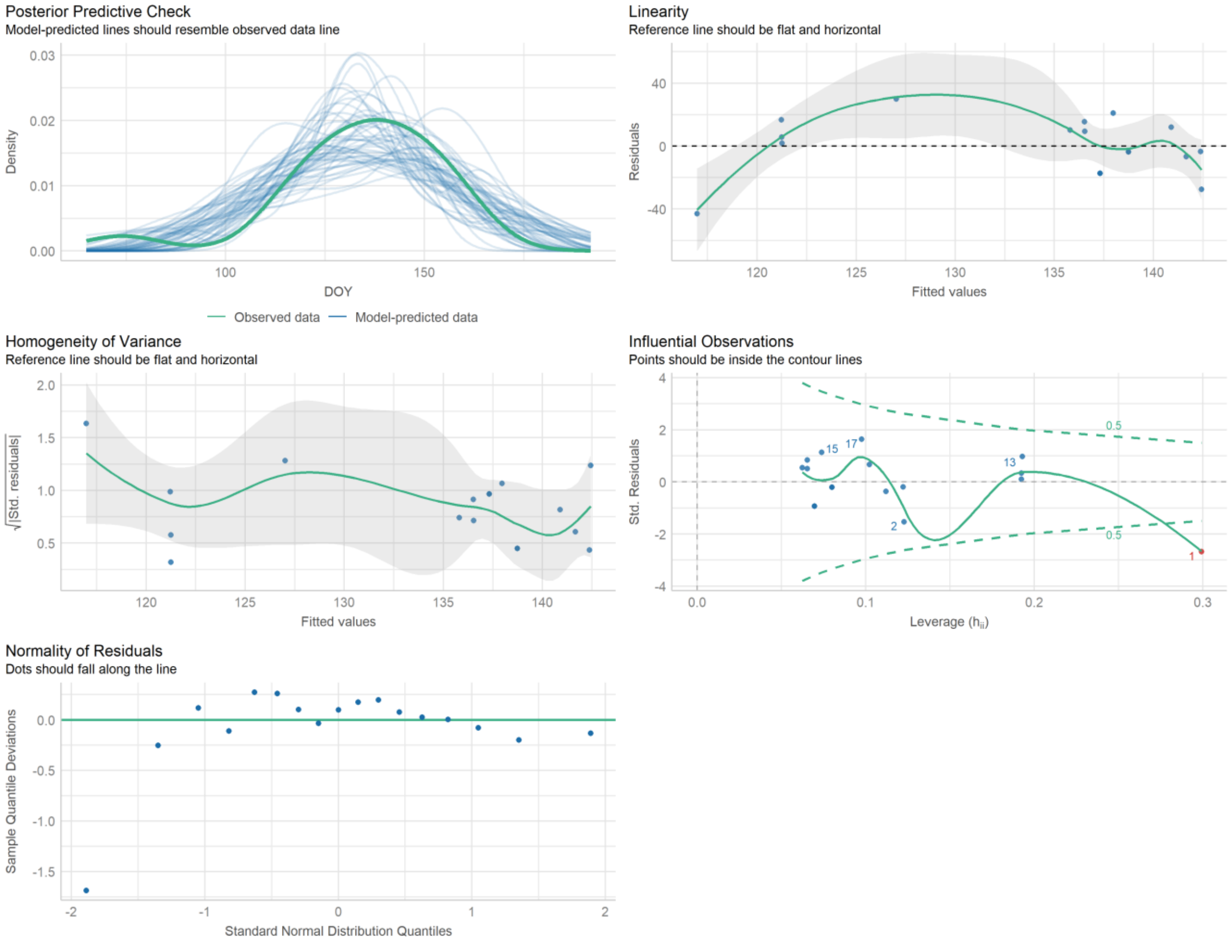

1.101. LM - F - Rhamnus alaternus

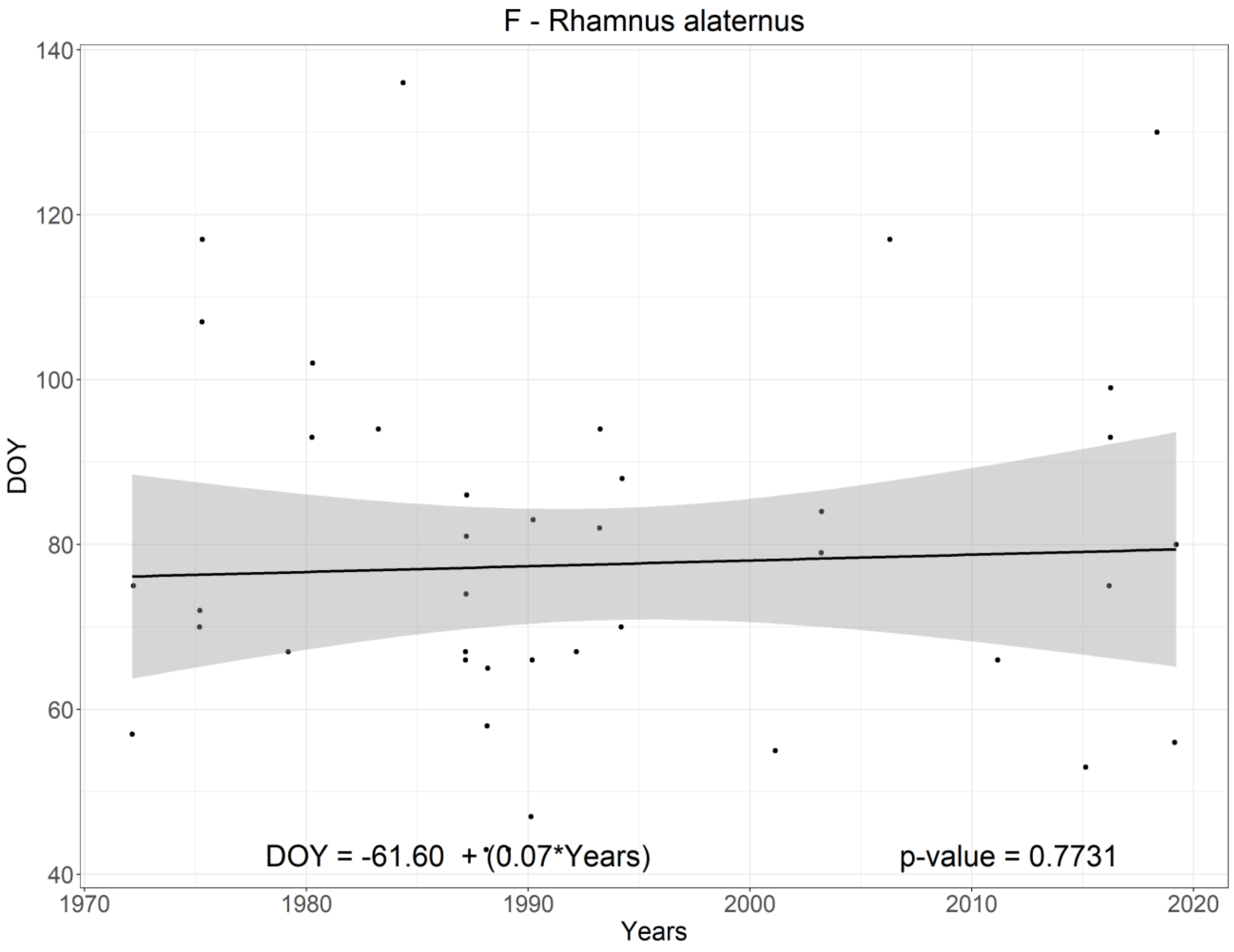

1.101.1. Diagnostics - LM - F - Rhamnus alaternus

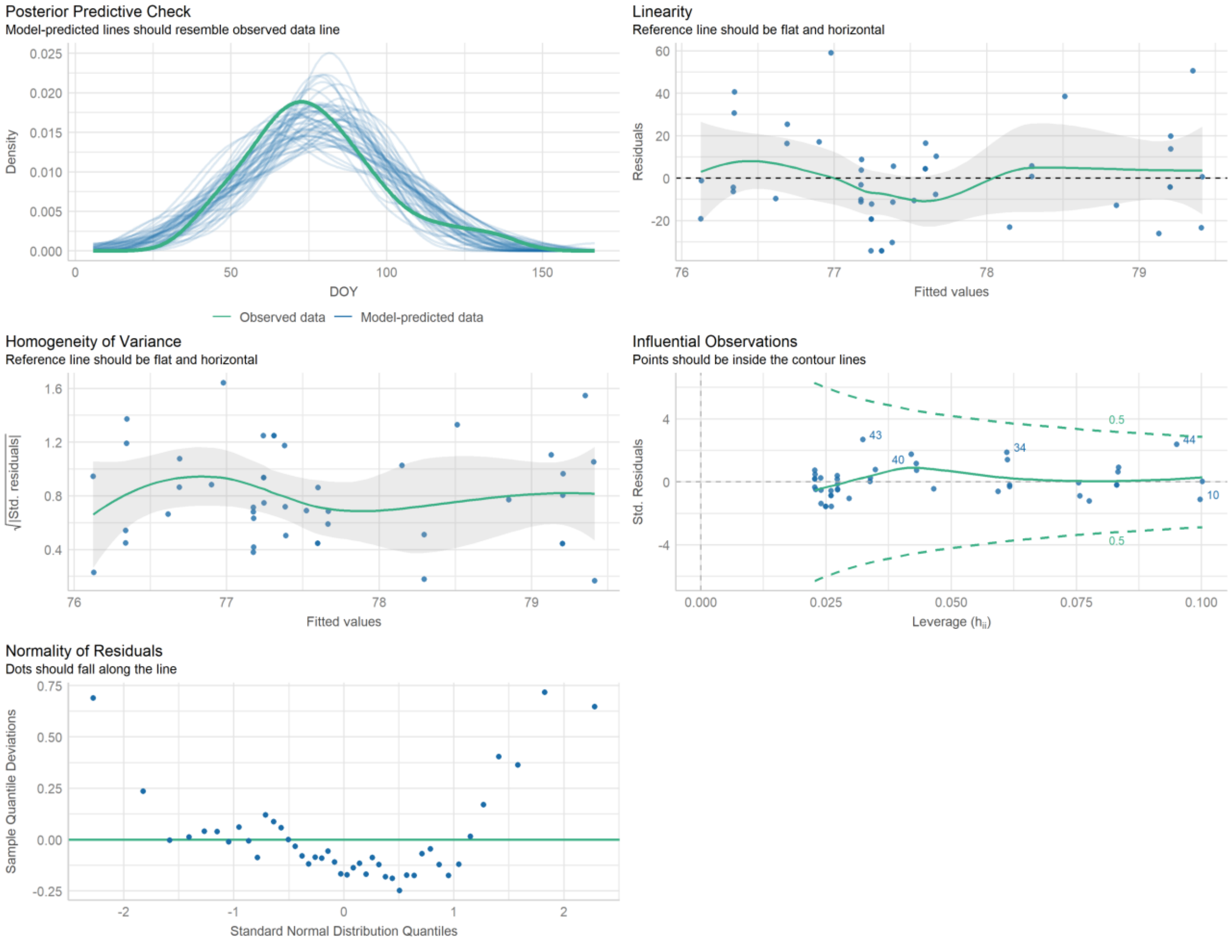

1.102. LM - DVG - Rhamnus alaternus

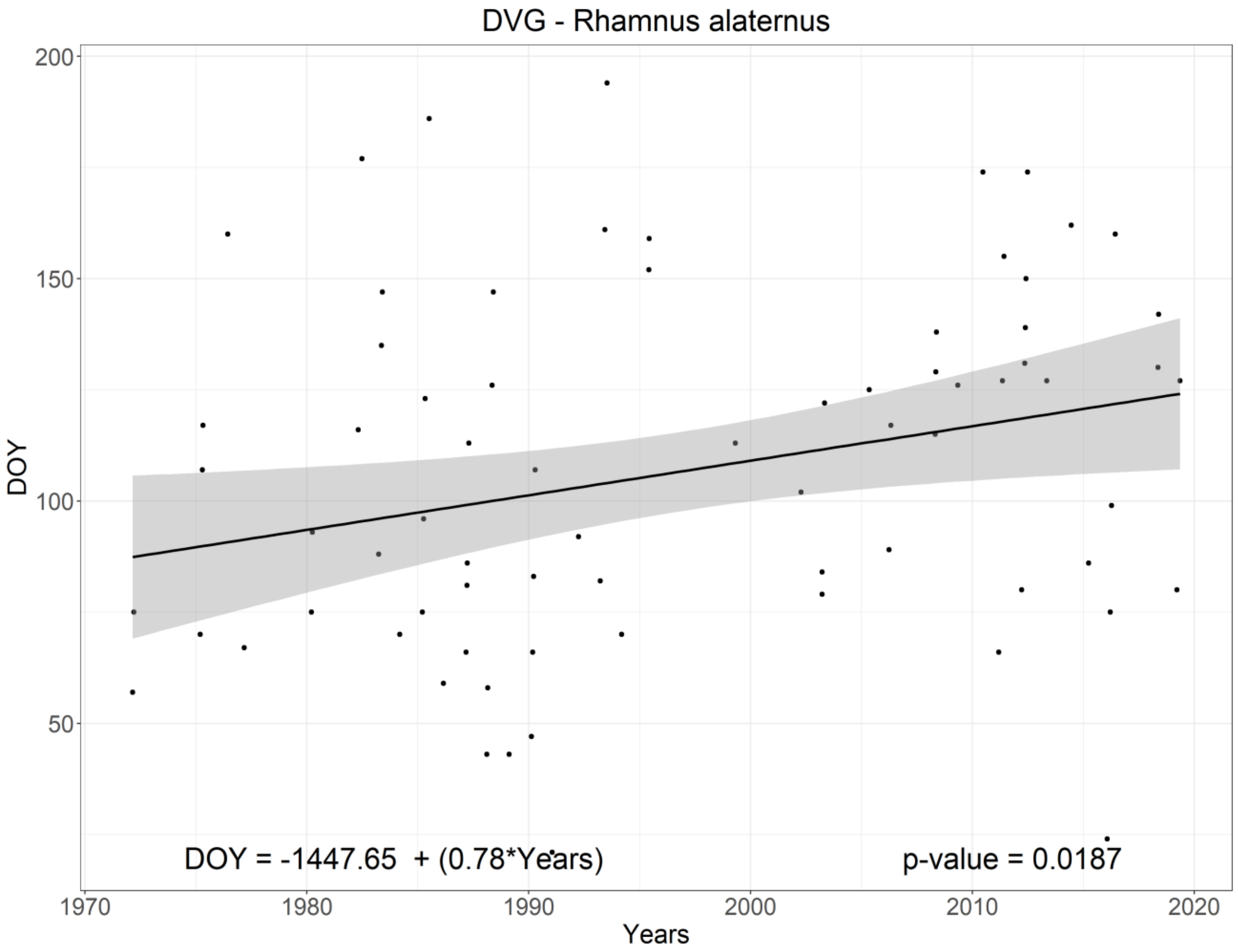

1.102.1. Diagnostics - LM - DVG - Rhamnus alaternus

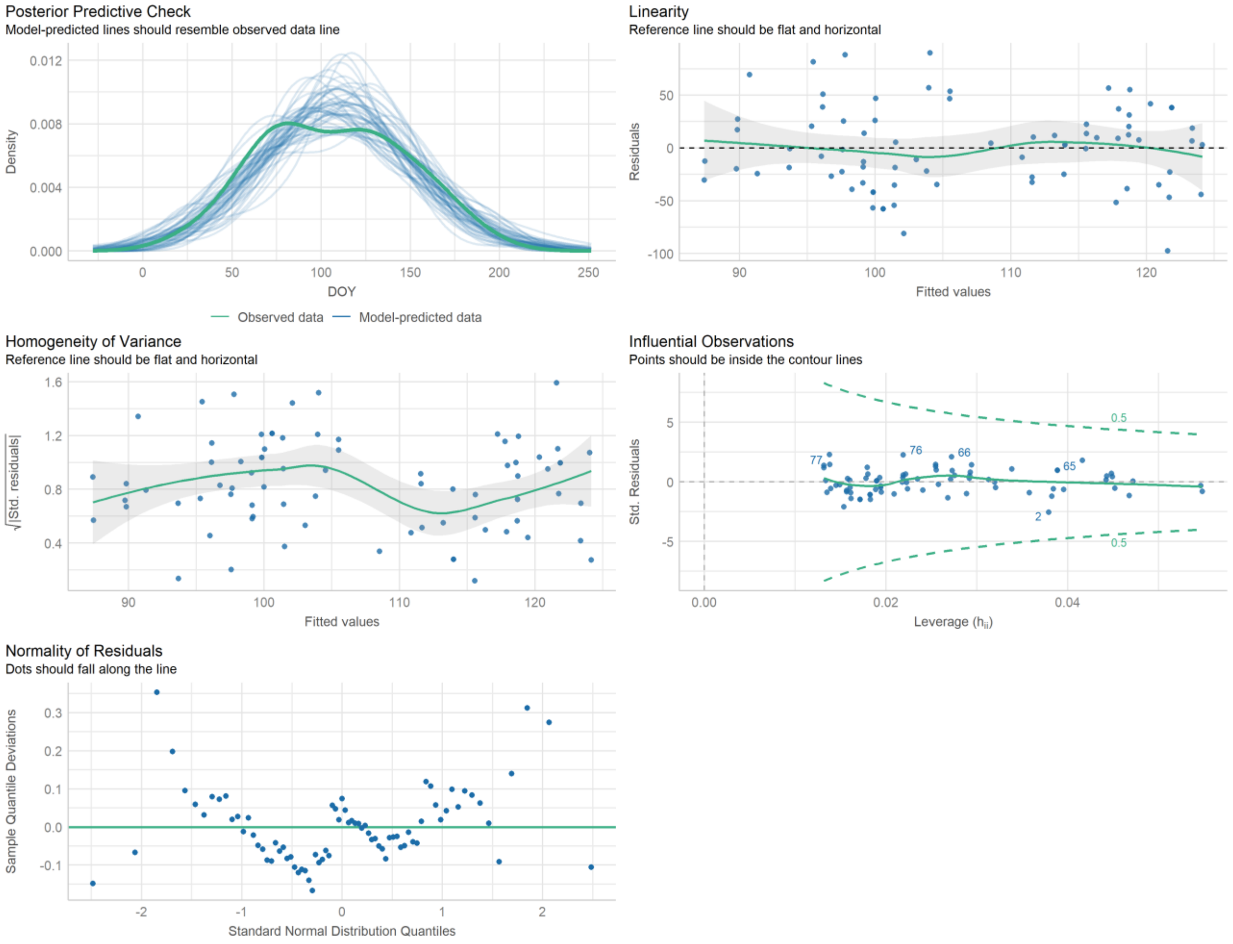

1.103. LM - DVG - Rhamnus infectoria

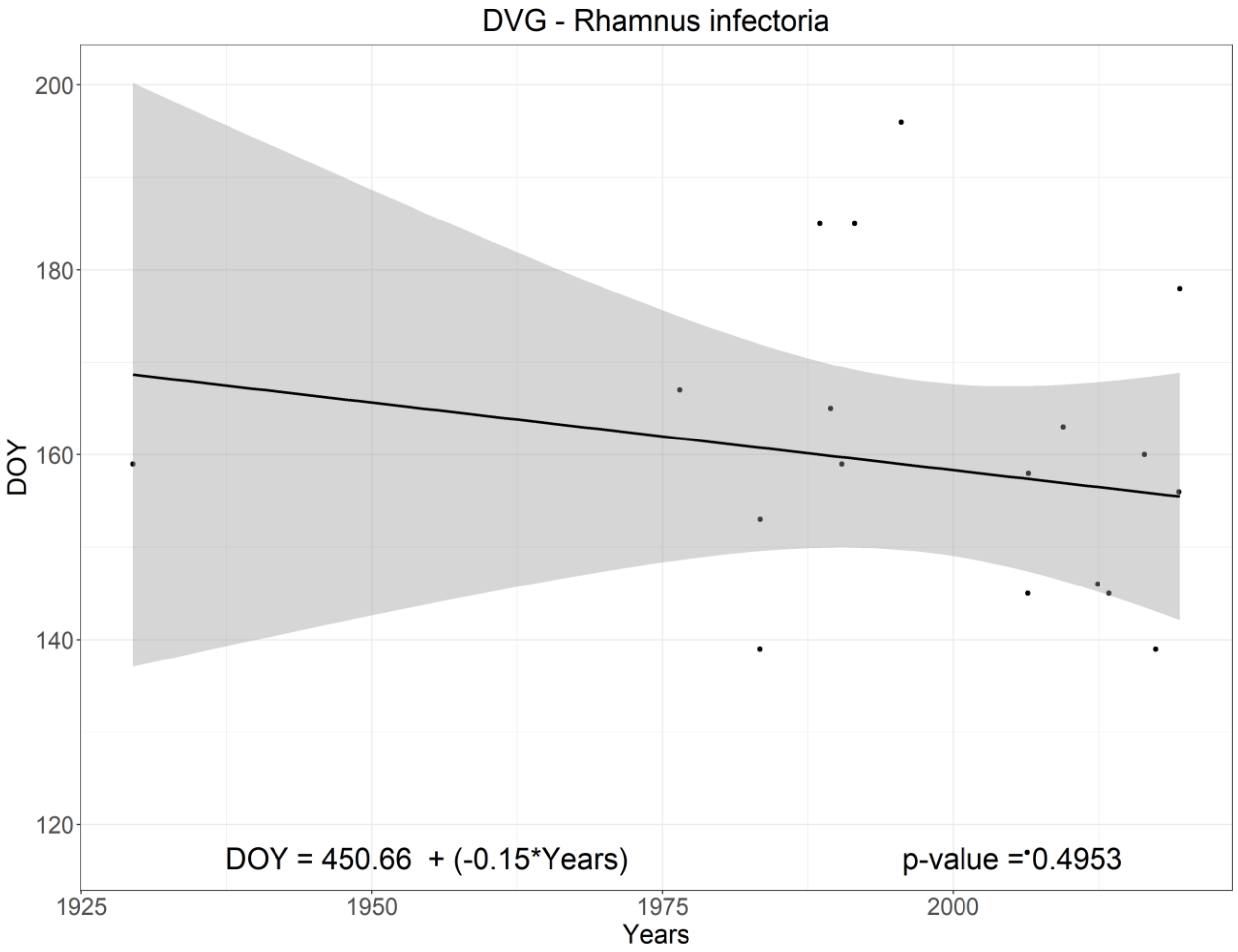

1.103.1. Diagnostics - LM - DVG - Rhamnus infectoria

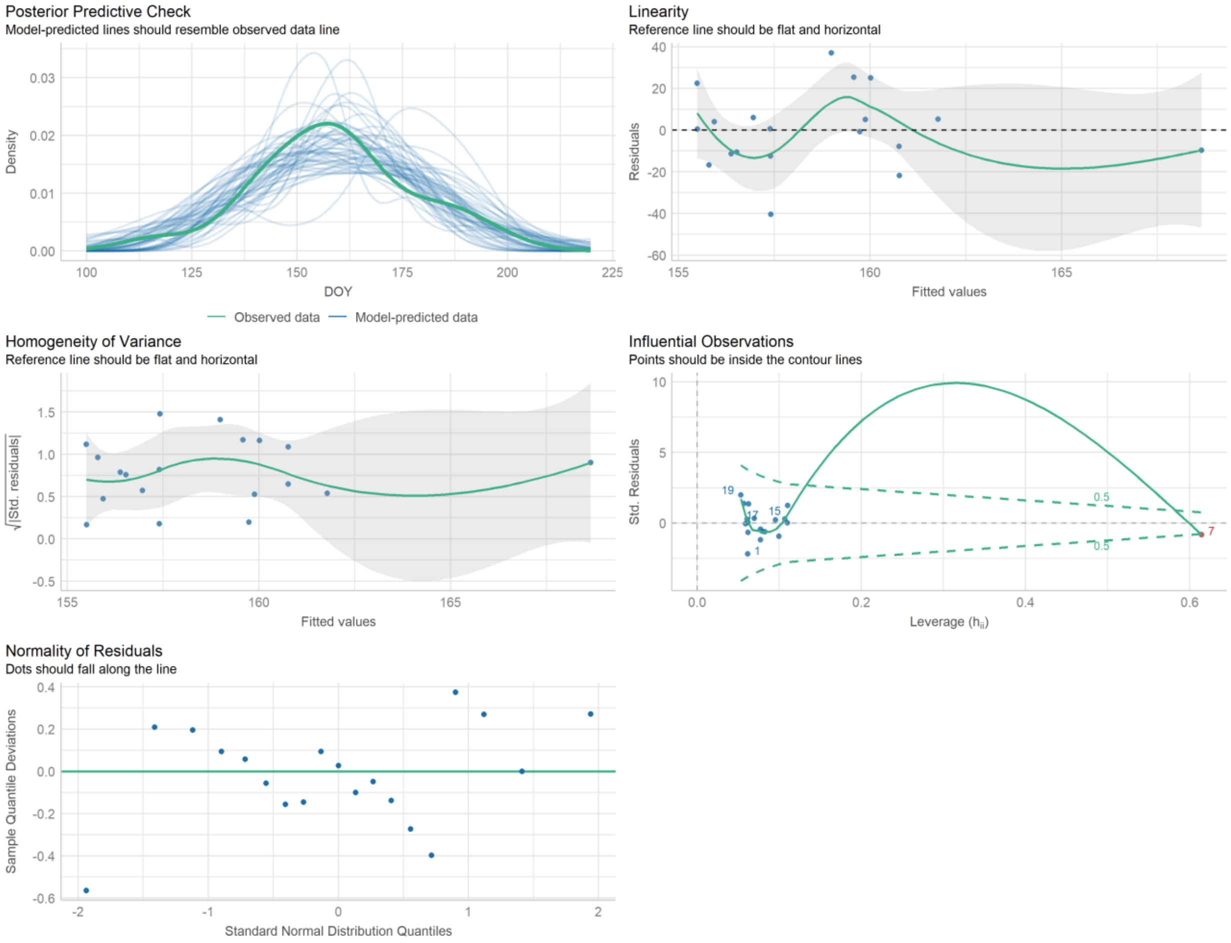

1.104. LM - FBF - Rubia peregrina

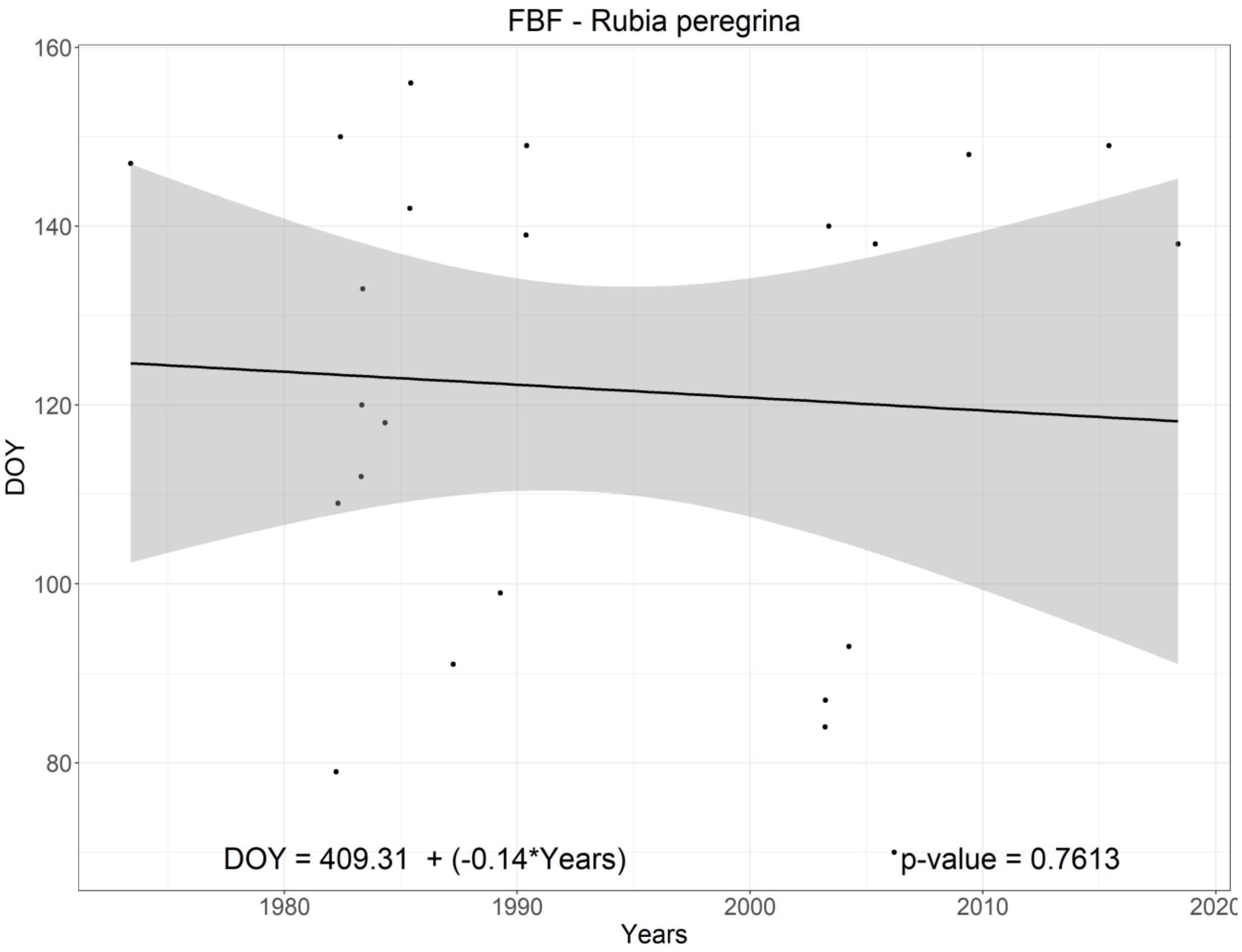

1.104.1. Diagnostics - LM - FBF - Rubia peregrina

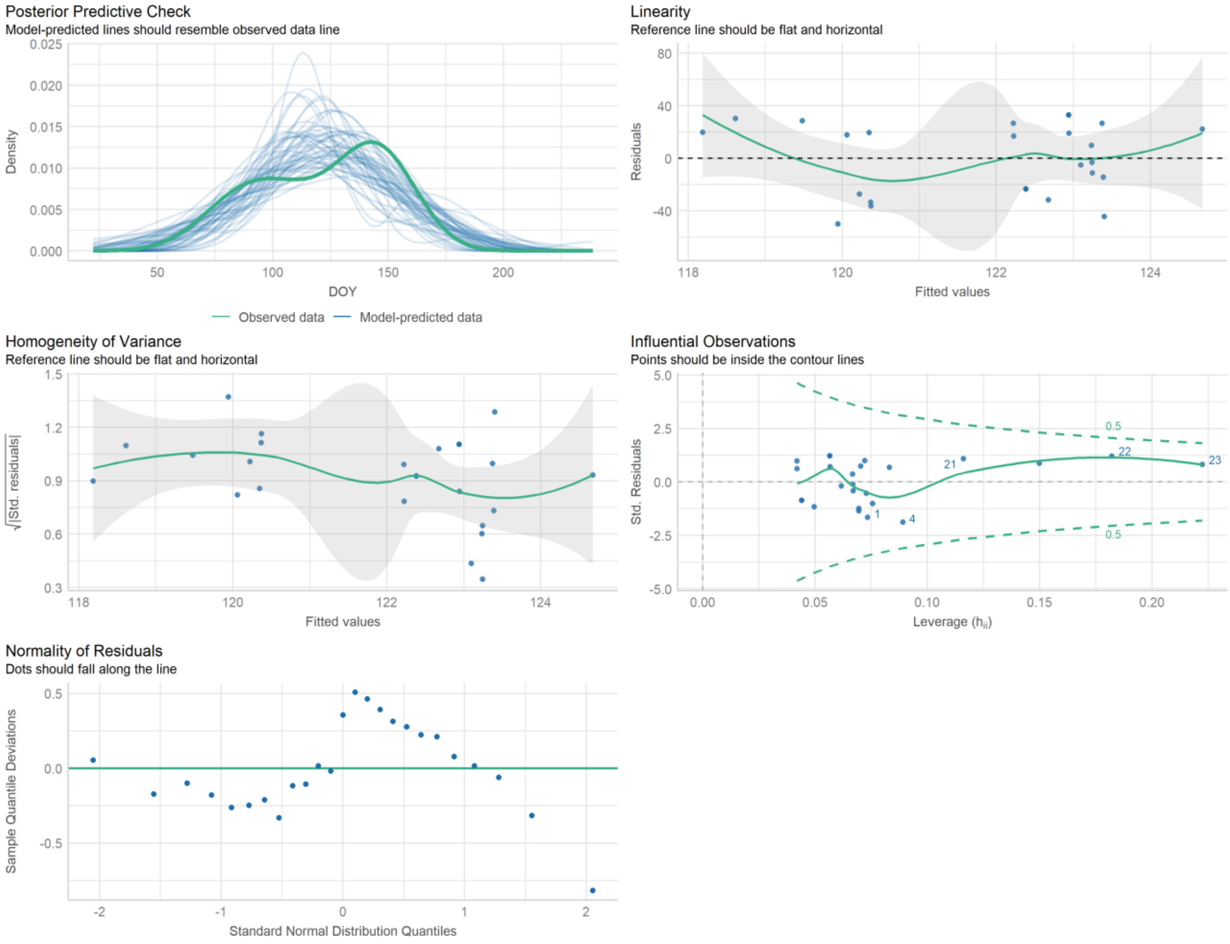

1.105. LM - DVG - Rubia peregrina

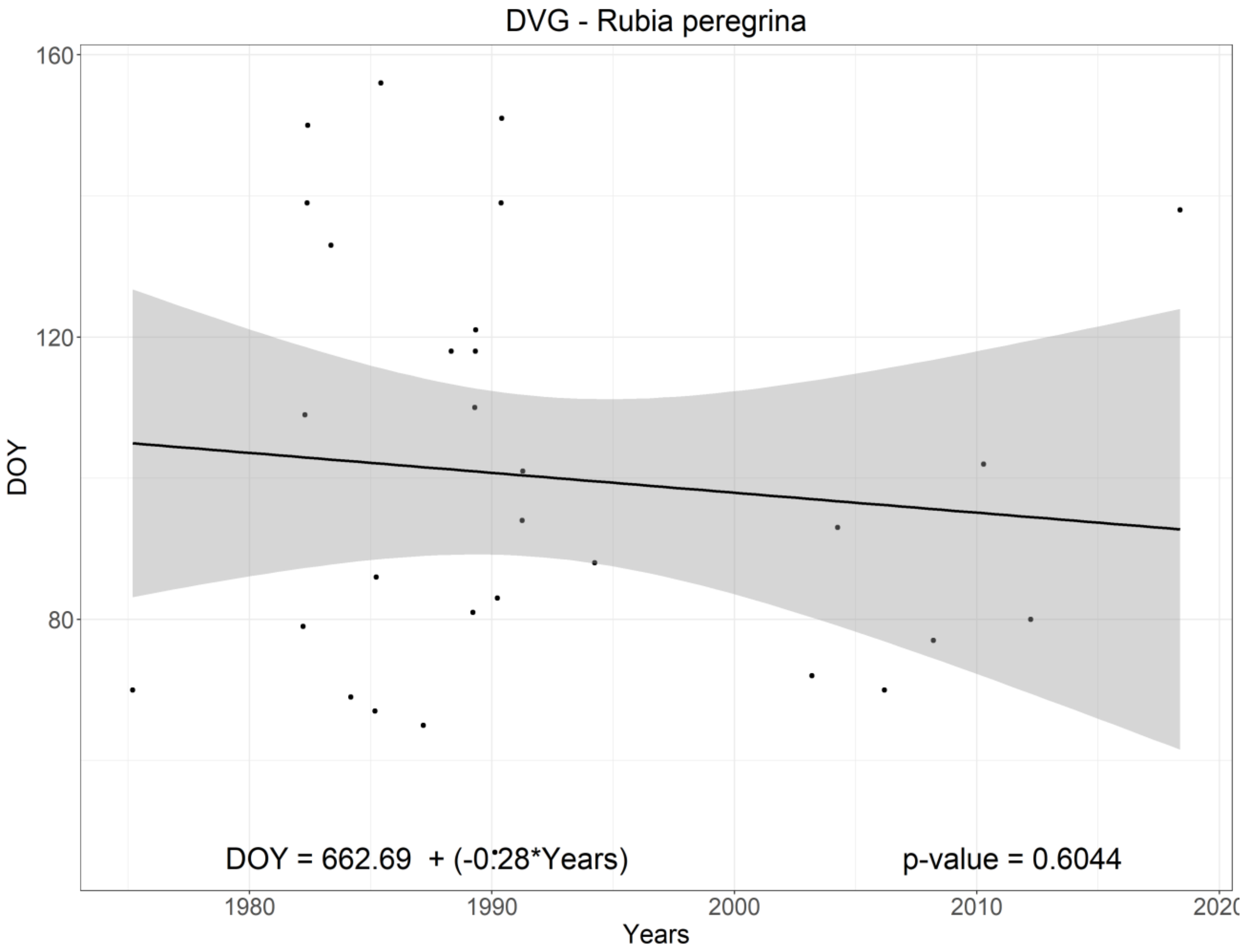

1.105.1. Diagnostics - LM - DVG - Rubia peregrina

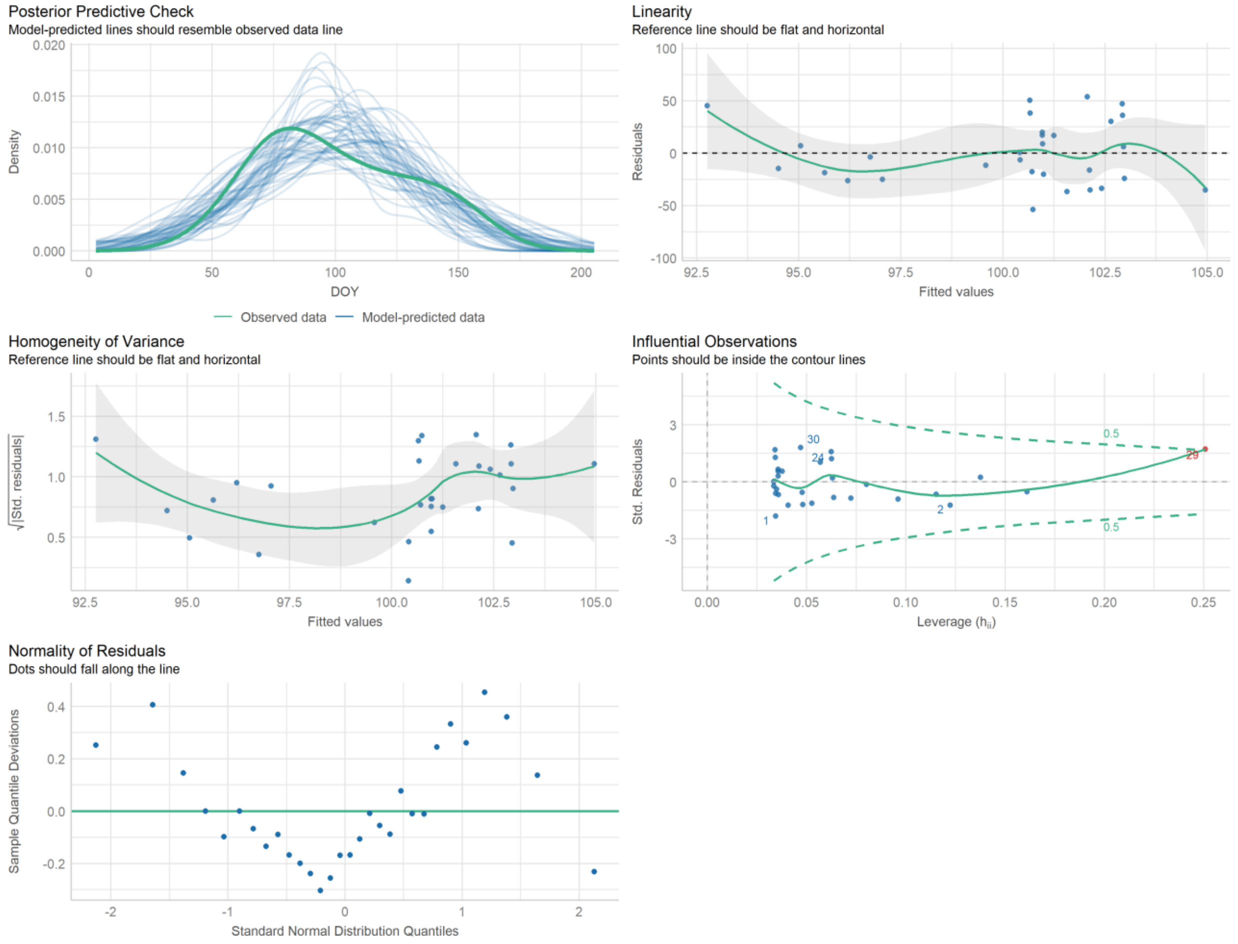

1.106. LM - FBF - Salvia candelabrum

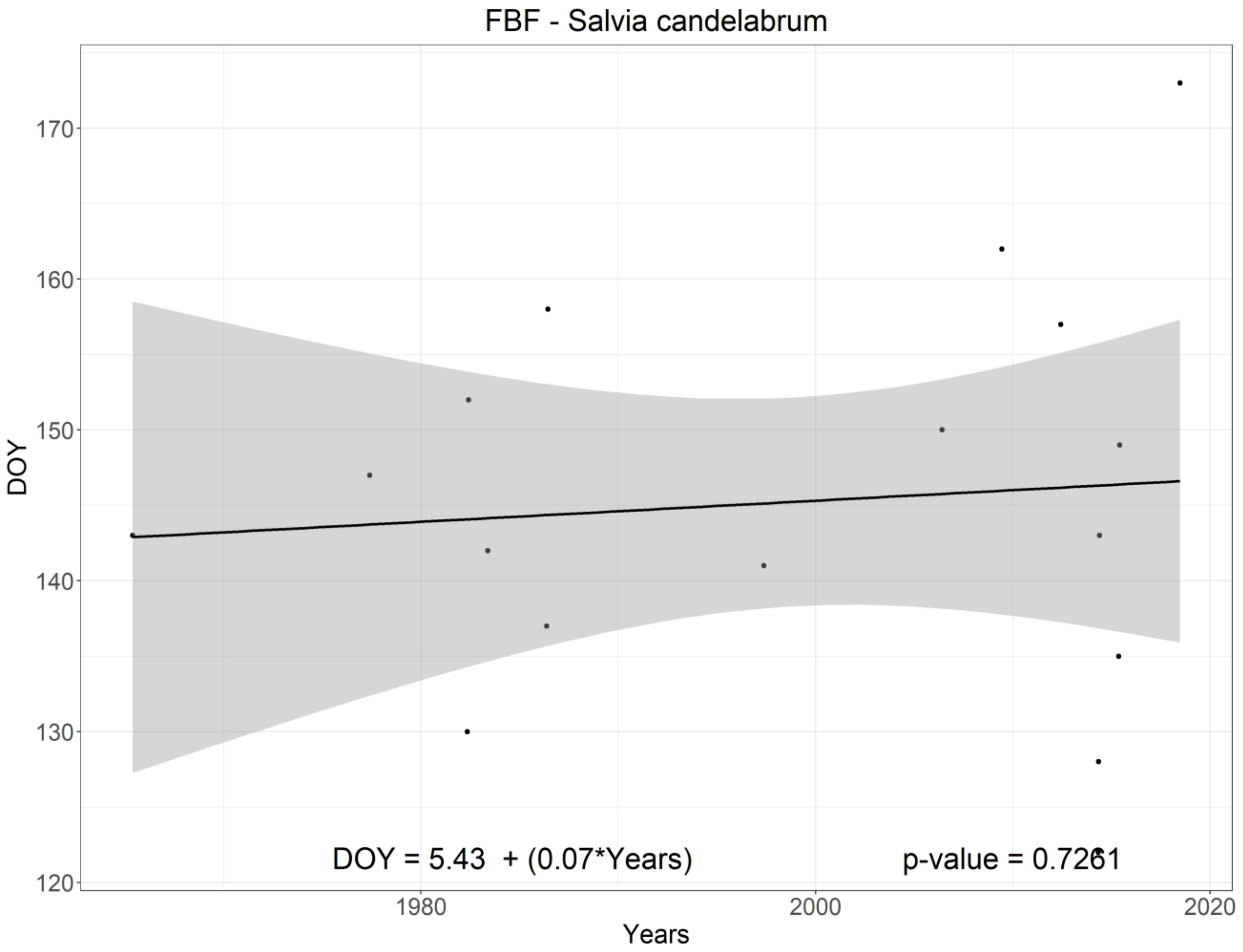

1.106.1. Diagnostics - LM - FBF - Salvia candelabrum

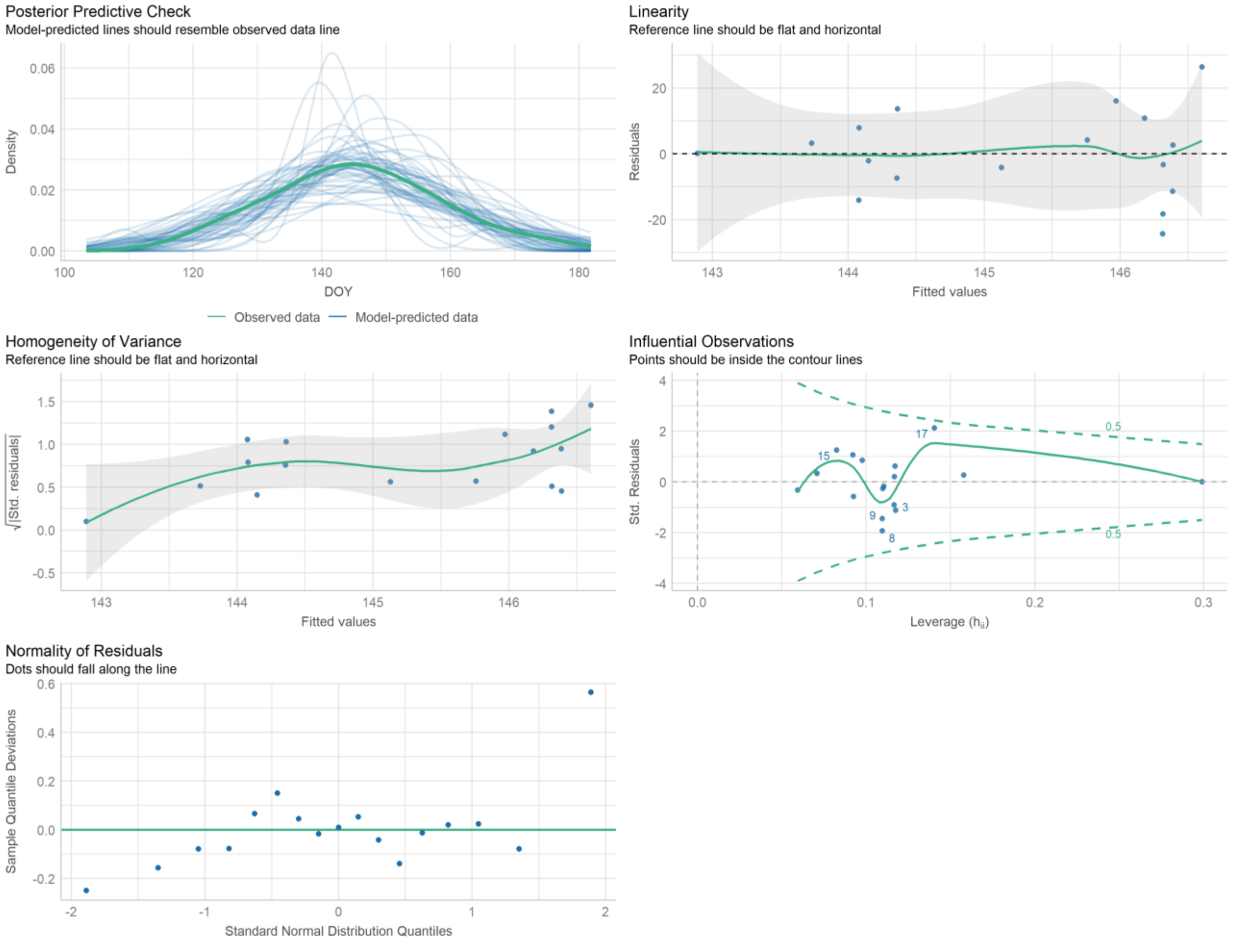

1.107. LM - F - Salvia candelabrum

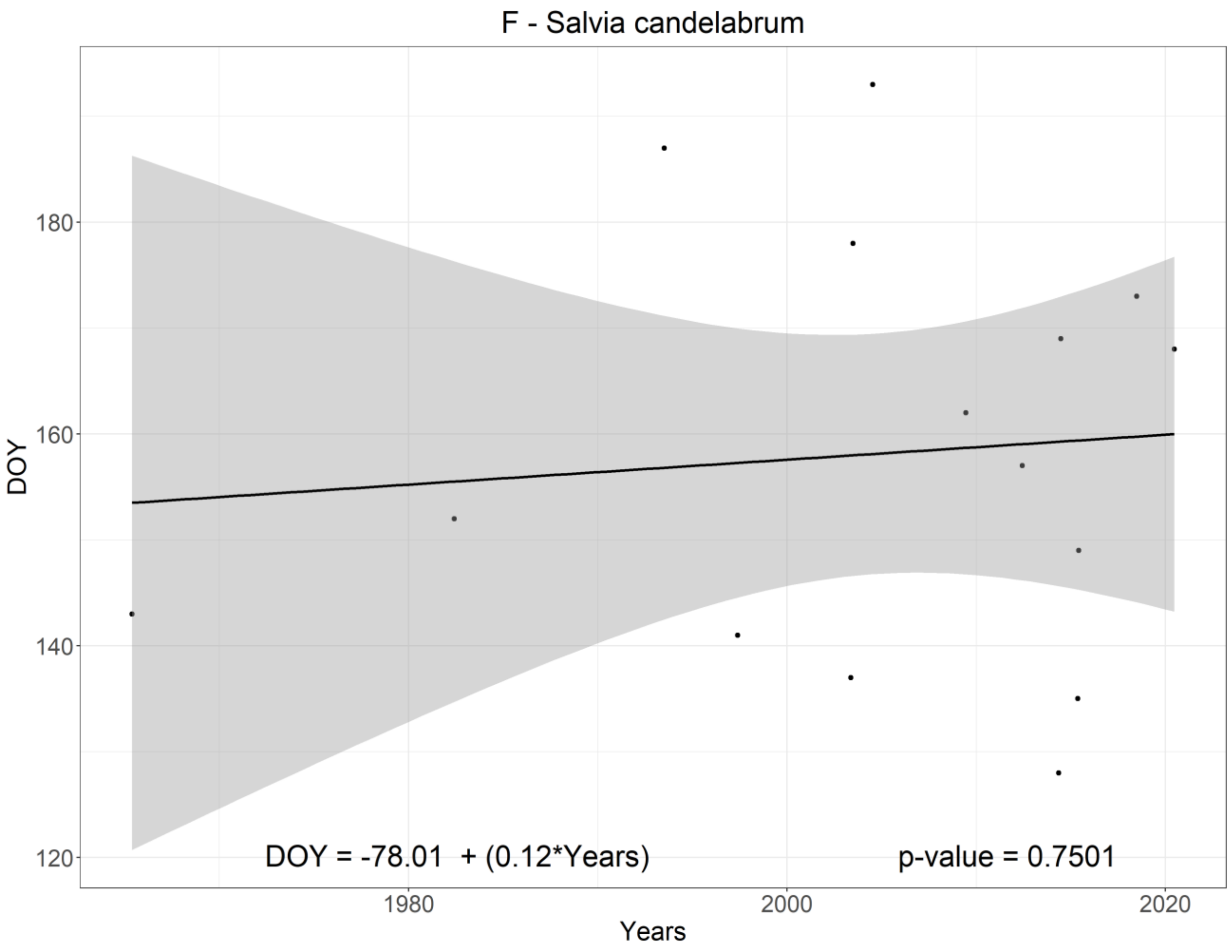

1.107.1. Diagnostics - LM - F - Salvia candelabrum

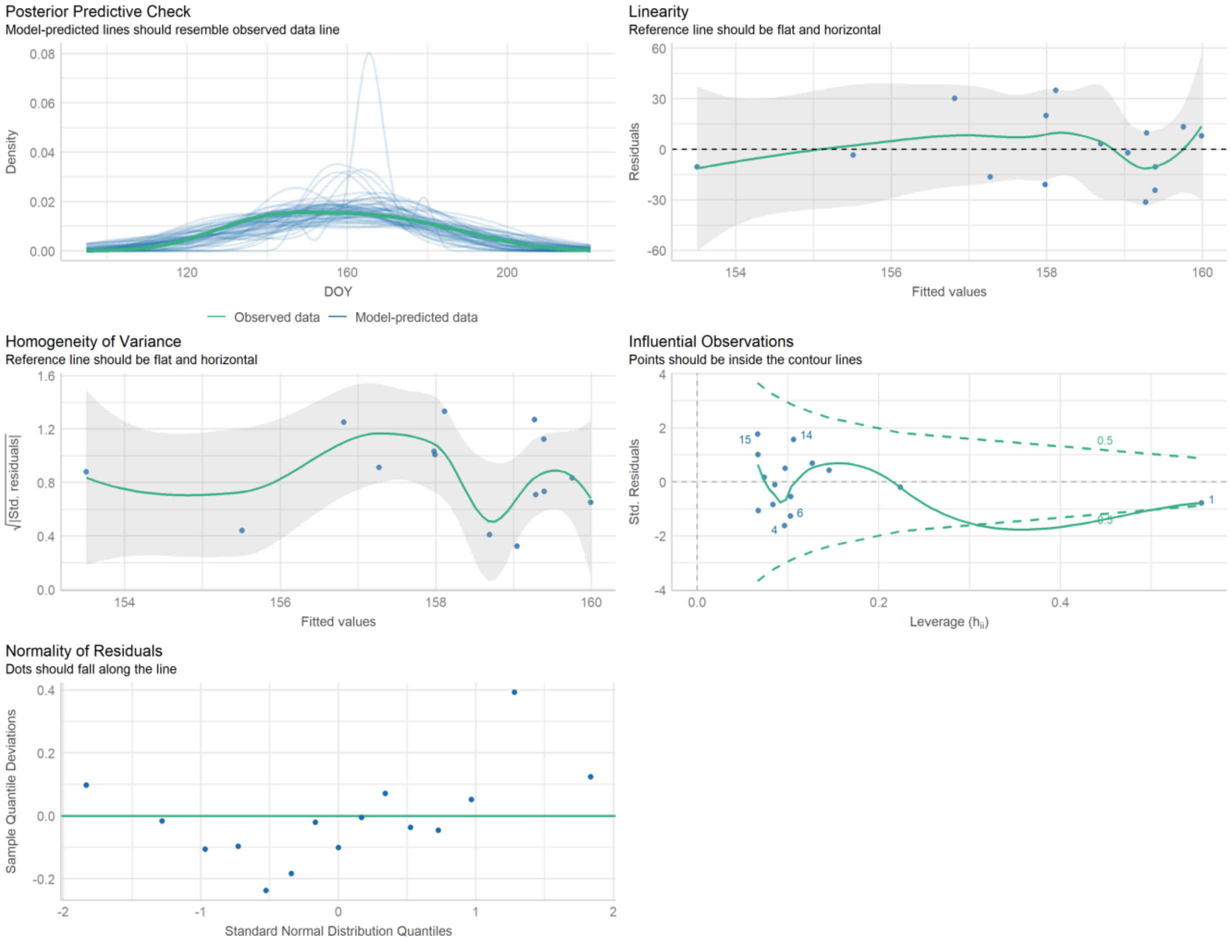

1.108. LM - FBF - Salvia lavandulifolia

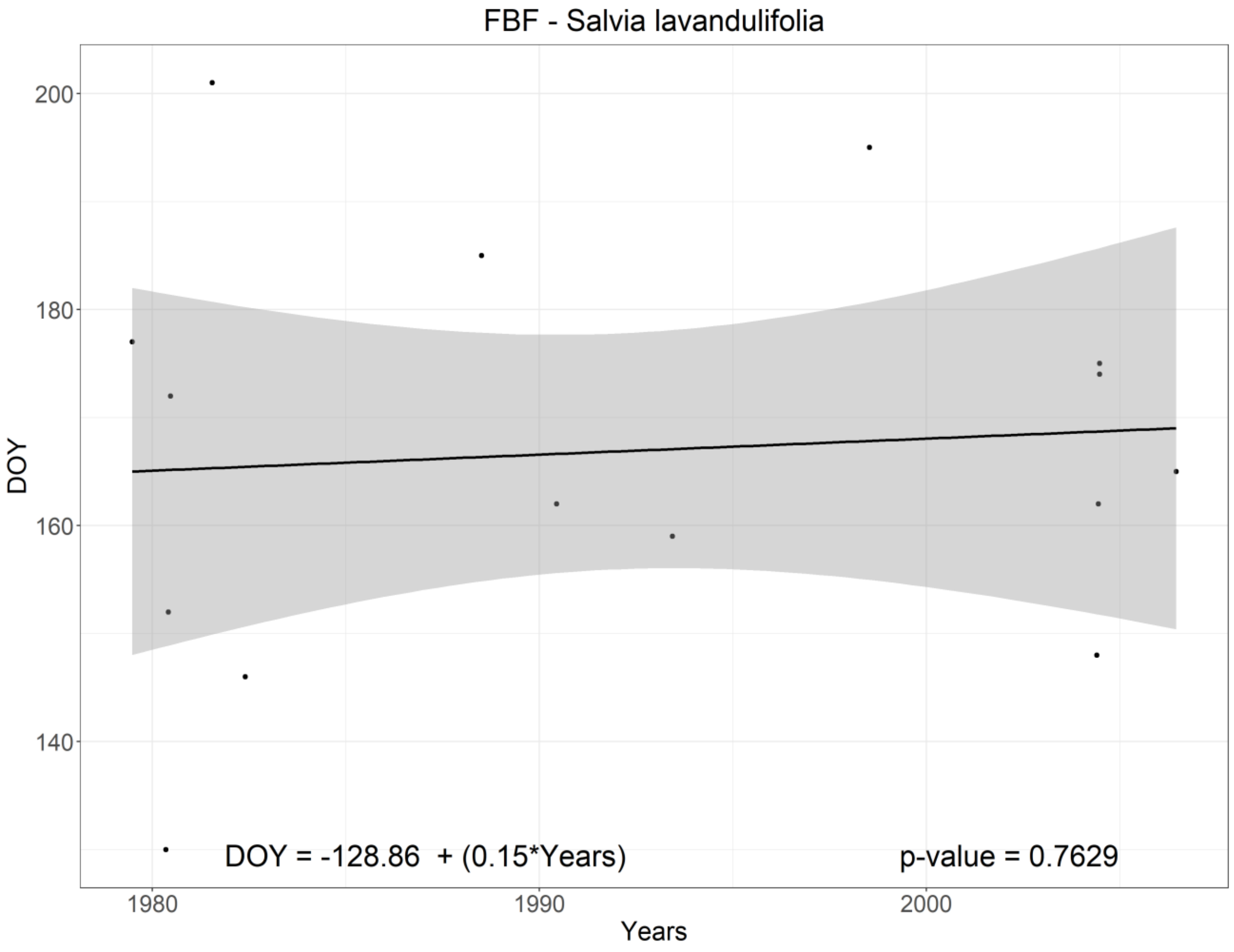

1.108.1. Diagnostics - LM - FBF - Salvia lavandulifolia

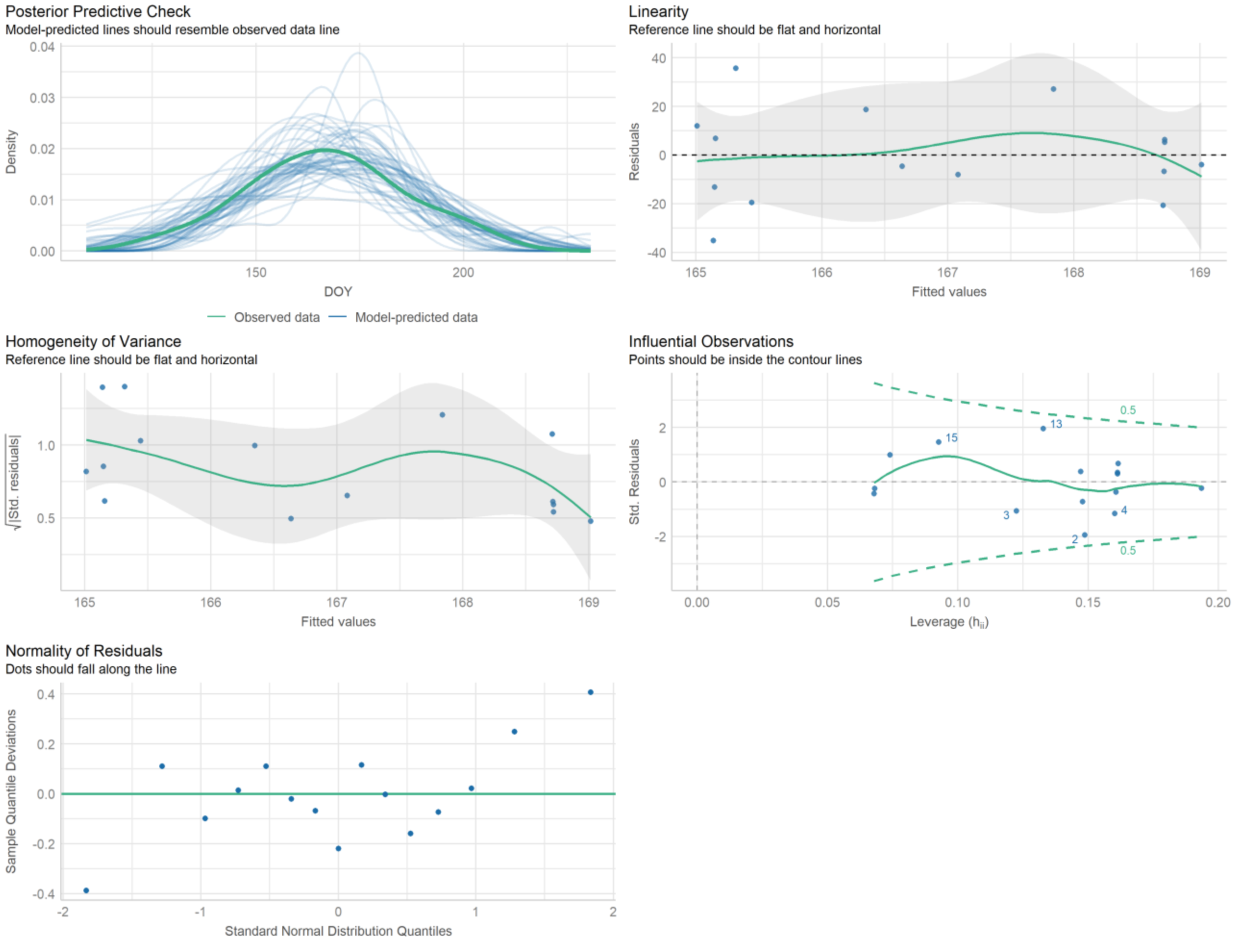

1.109. LM - FBF - Salvia rosmarinus

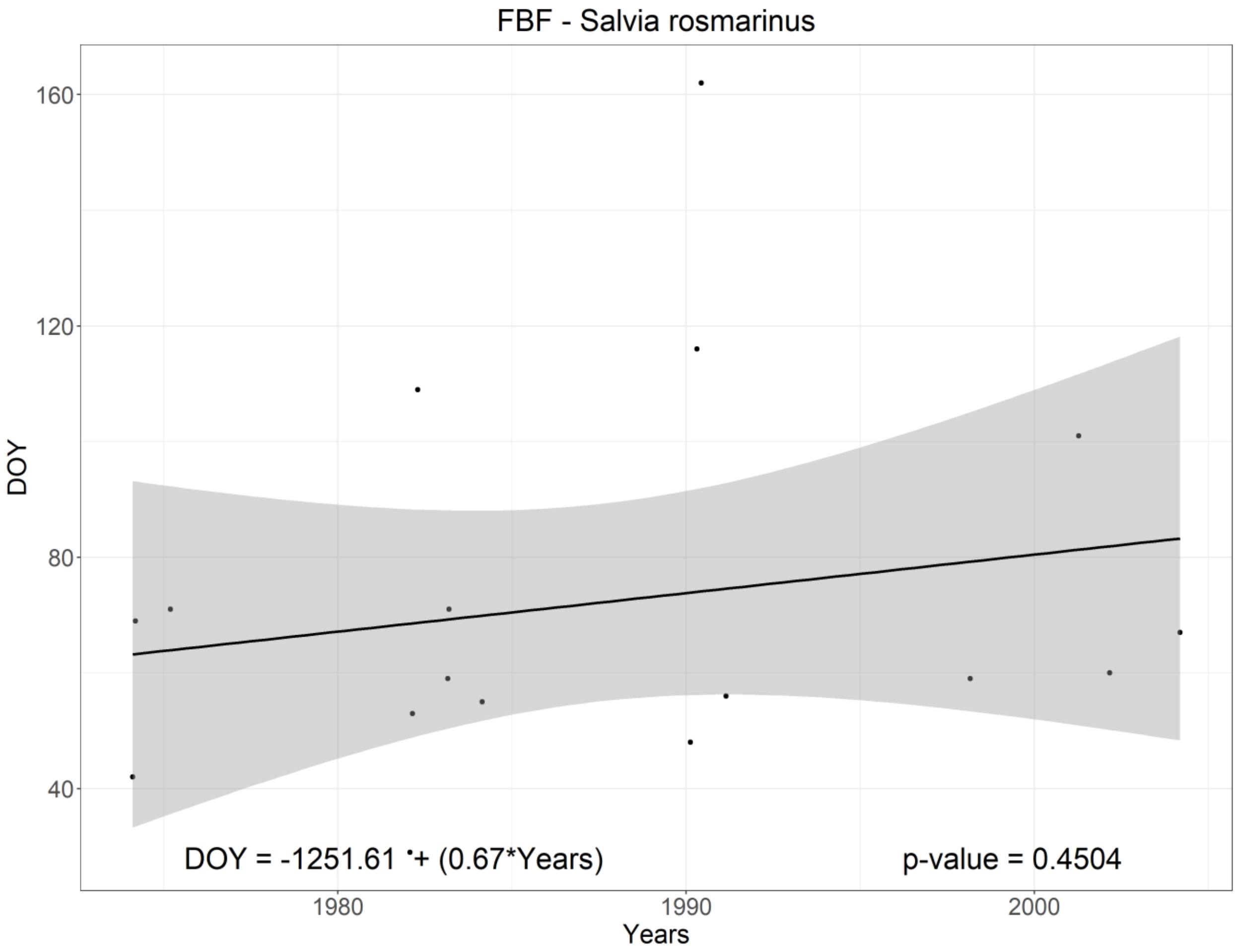

1.109.1. Diagnostics - LM - FBF - Salvia rosmarinus

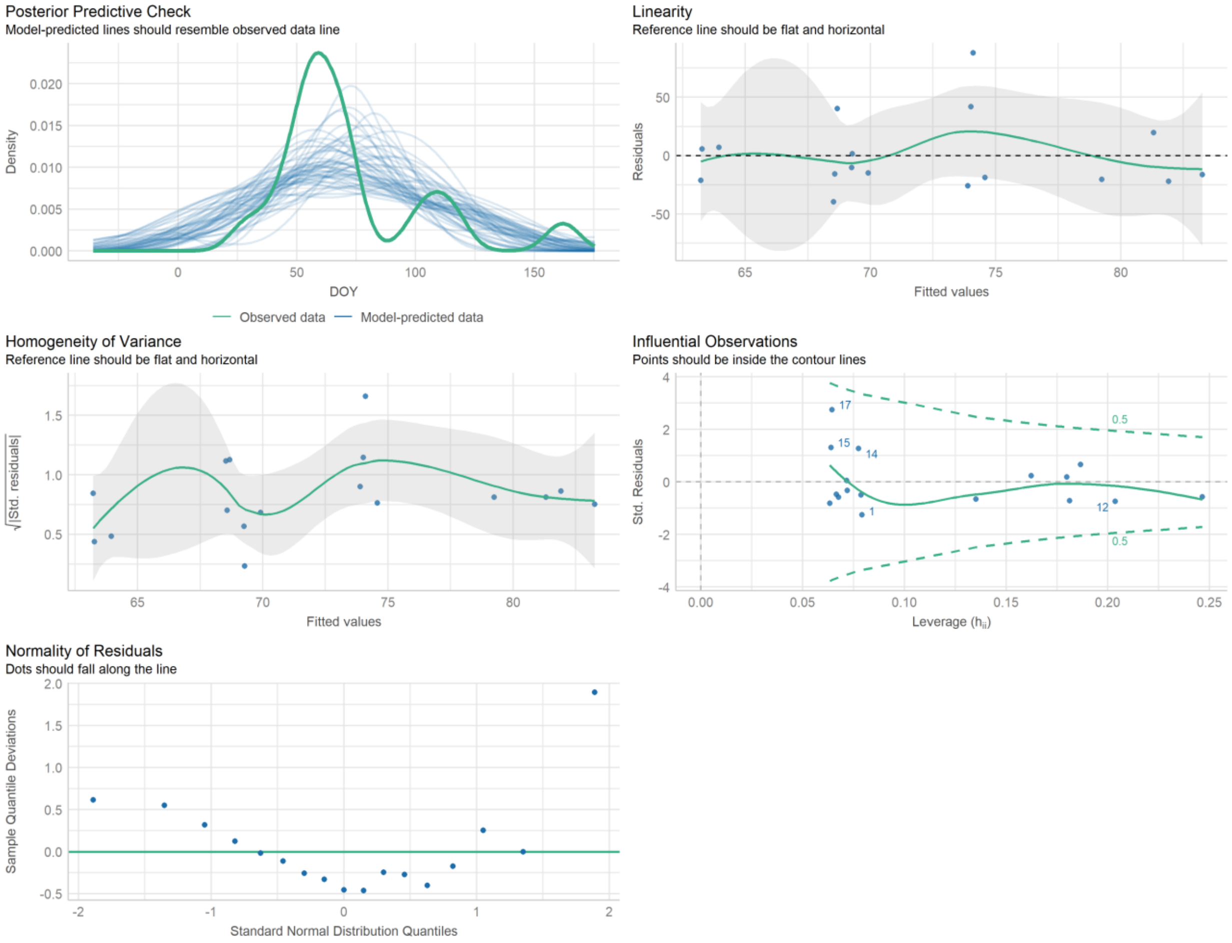

1.110. LM - F - Salvia rosmarinus

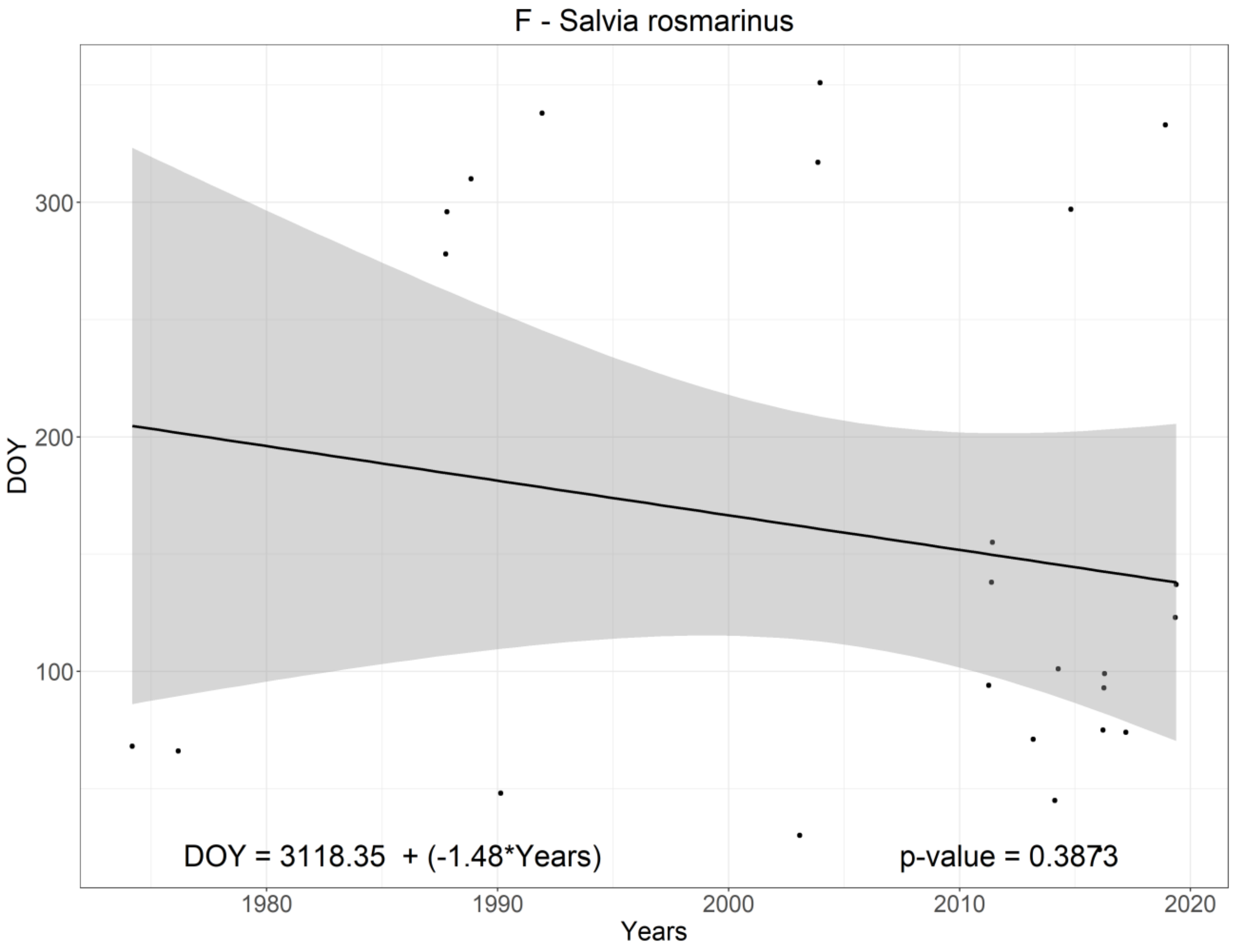

1.110.1. Diagnostics - LM - F - Salvia rosmarinus

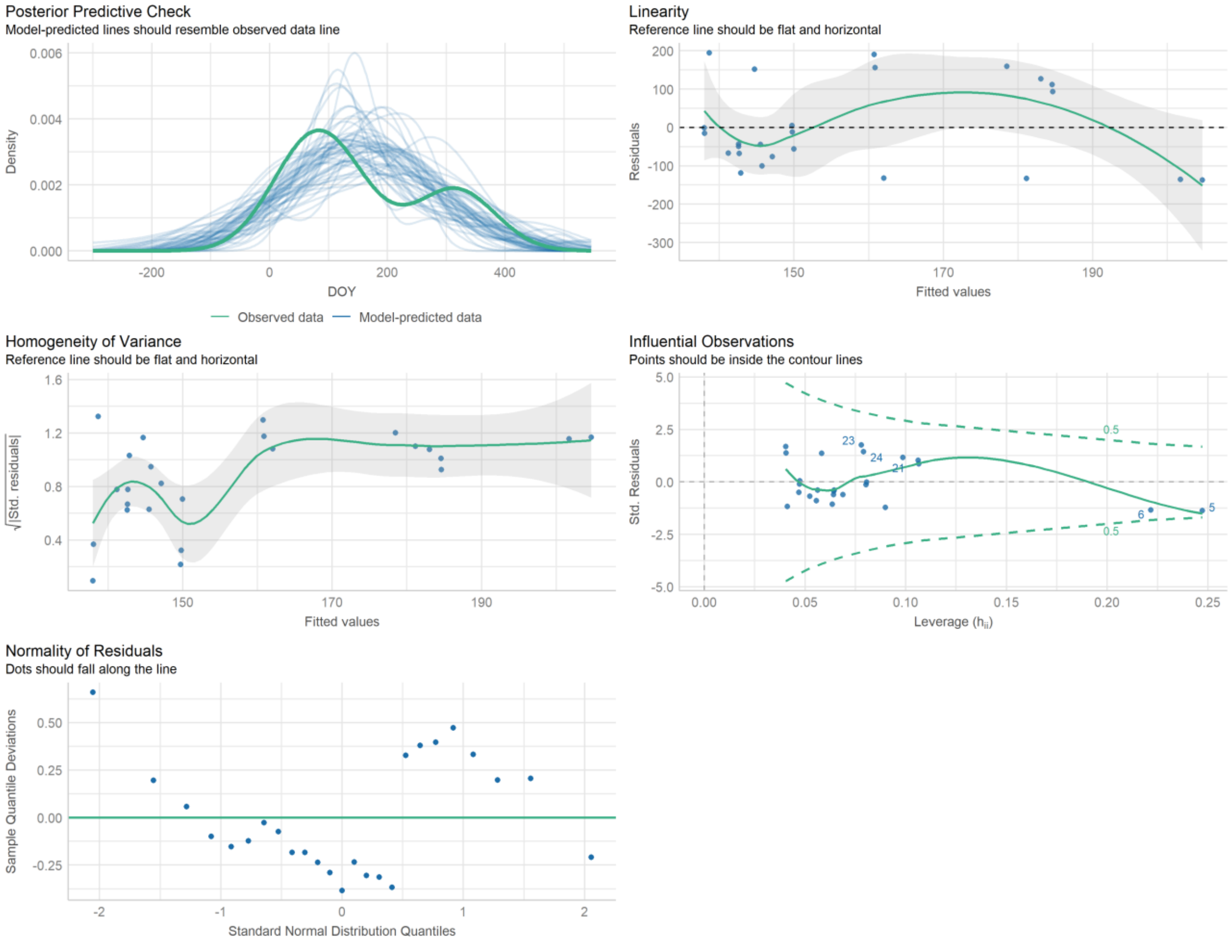

1.111. LM - F - Saxifraga reuteriana

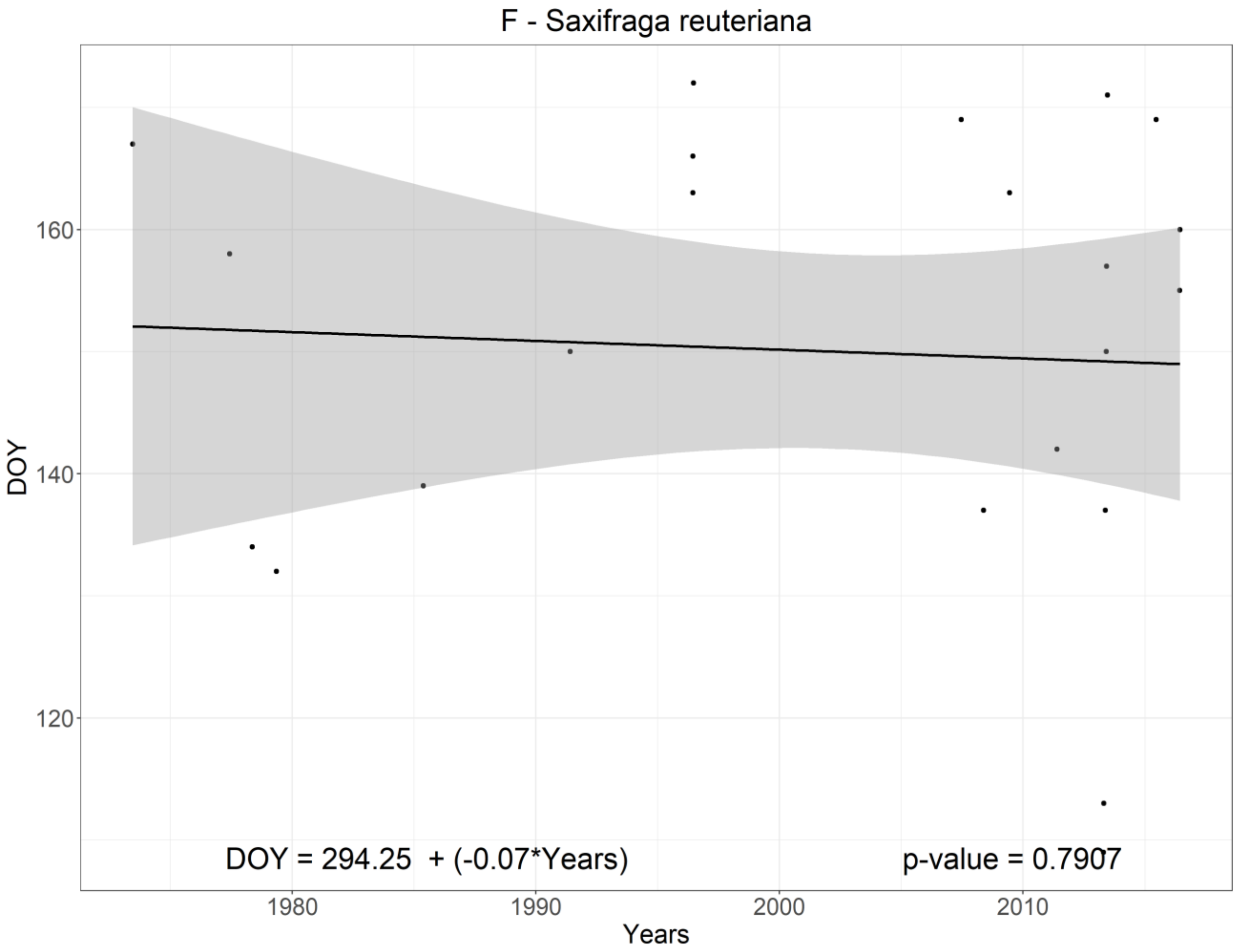

1.111.1. Diagnostics - LM - F - Saxifraga reuteriana

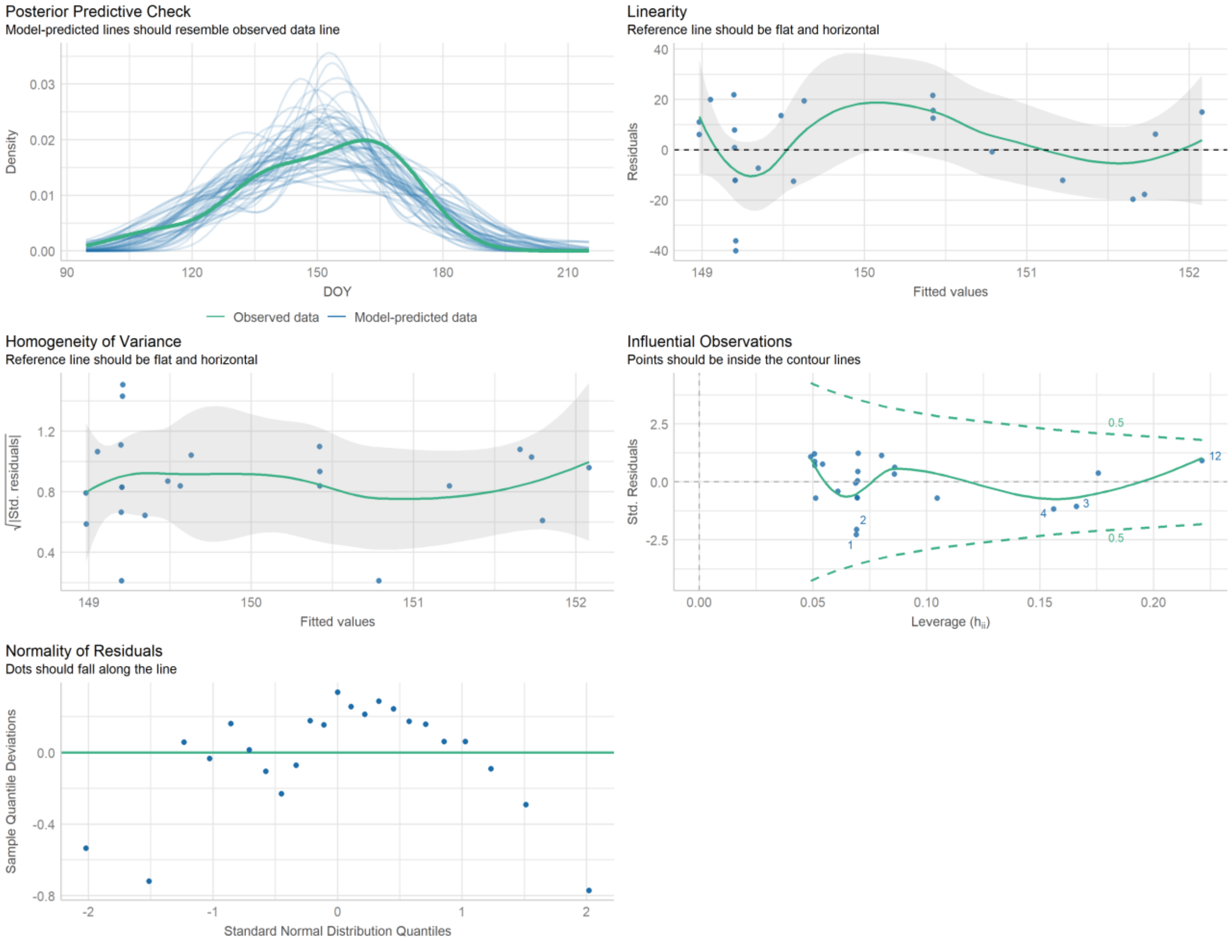

1.112. LM - F - Sempervivum minutum

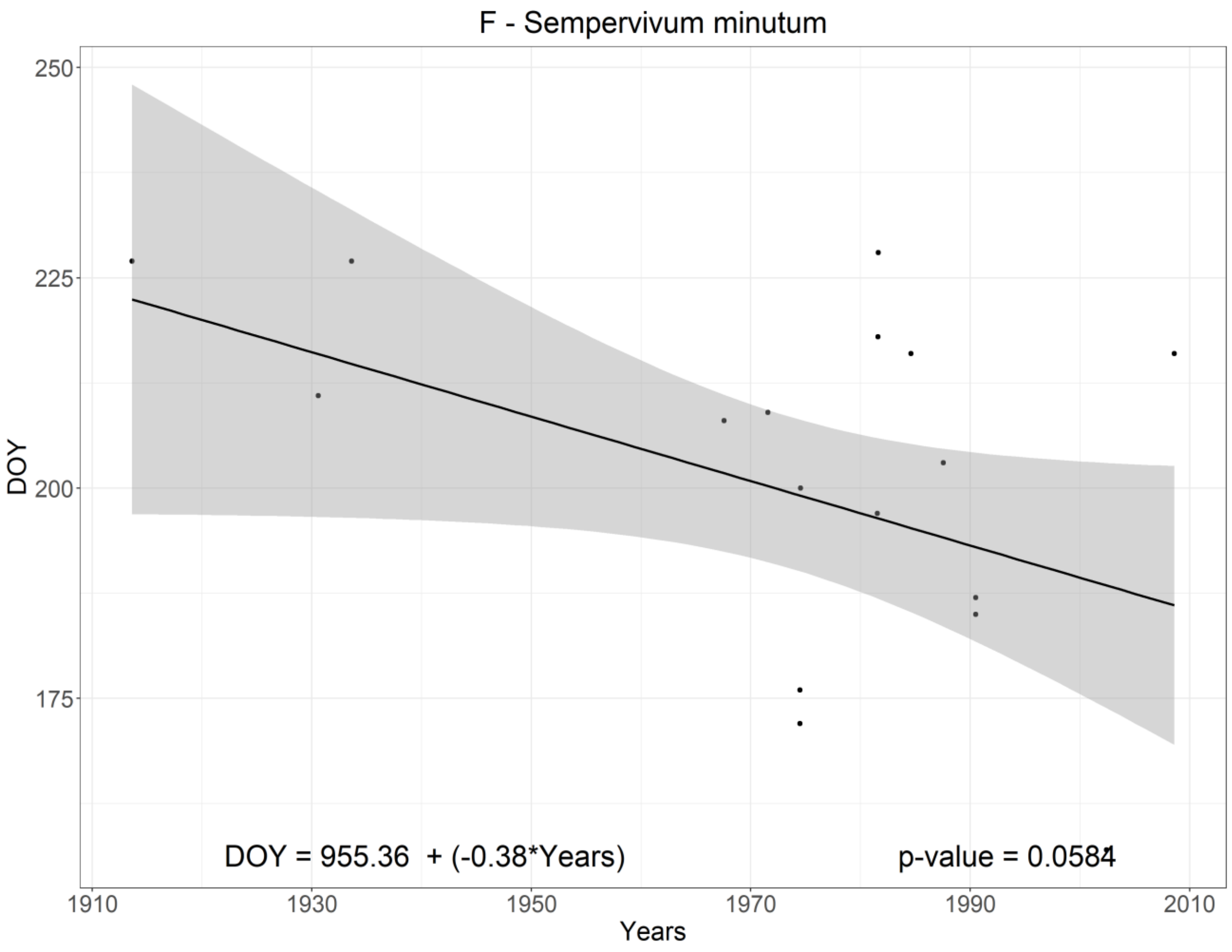

1.112.1. Diagnostics - LM - F - Sempervivum minutum

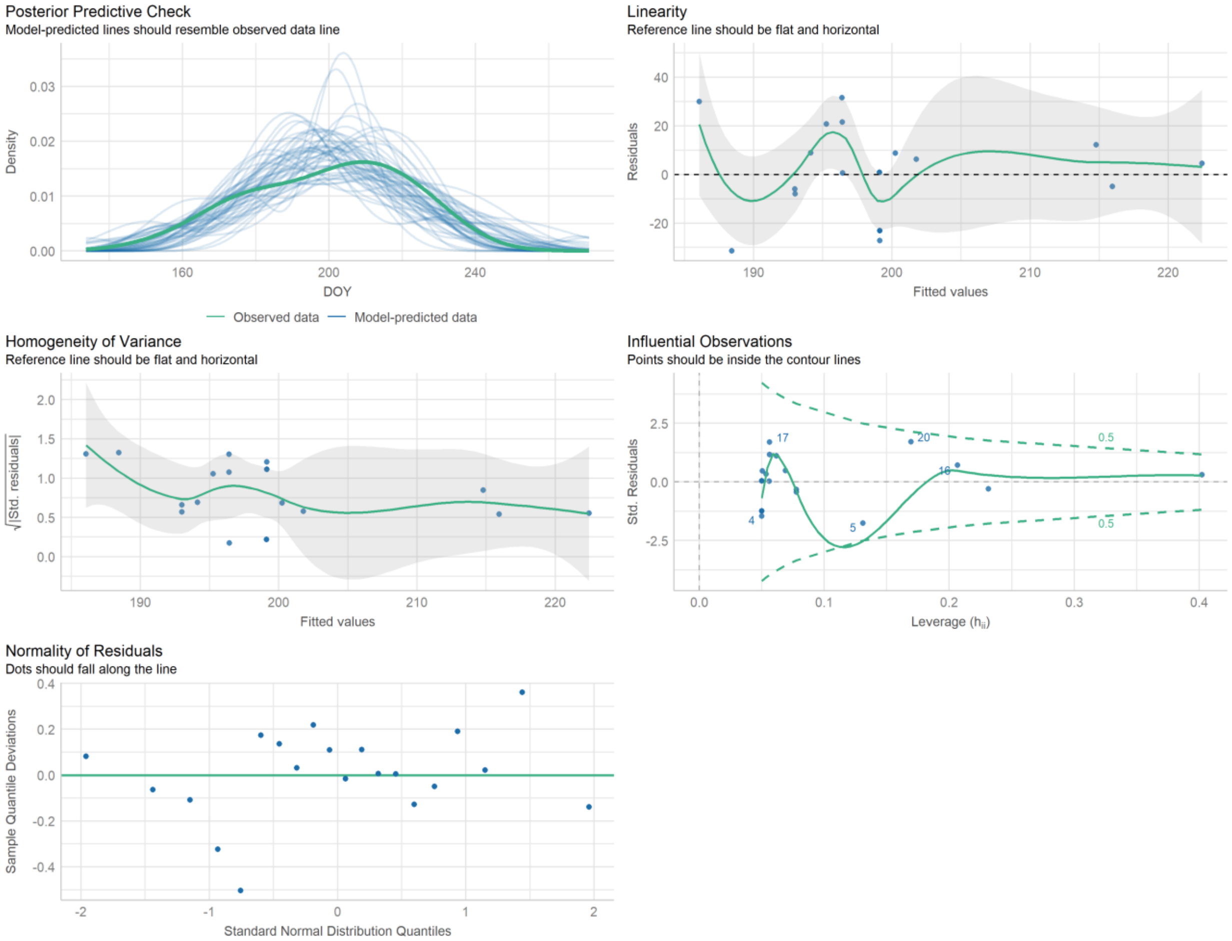

1.113. LM - FBF - Sideritis glacialis

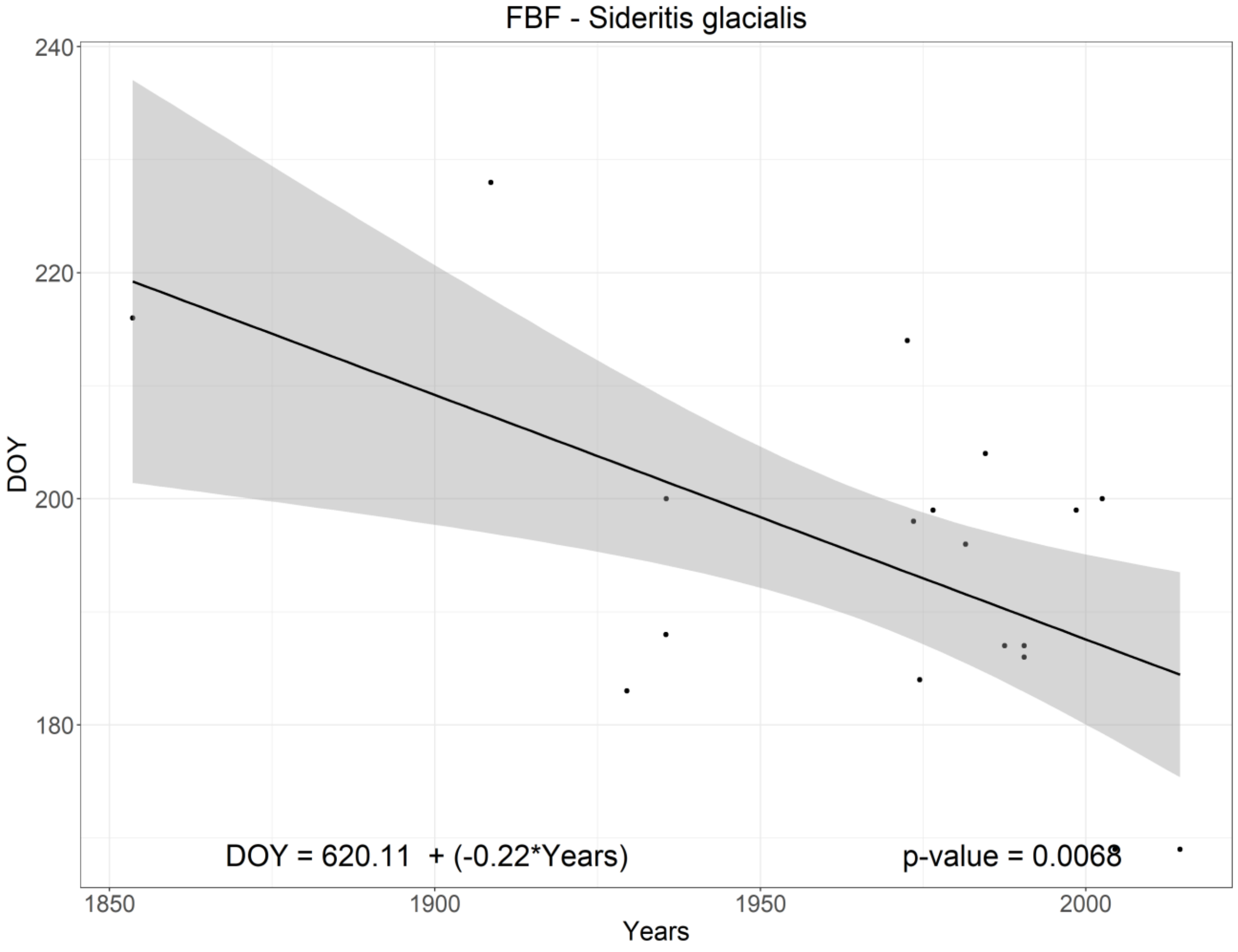

1.113.1. Diagnostics - LM - FBF - Sideritis glacialis

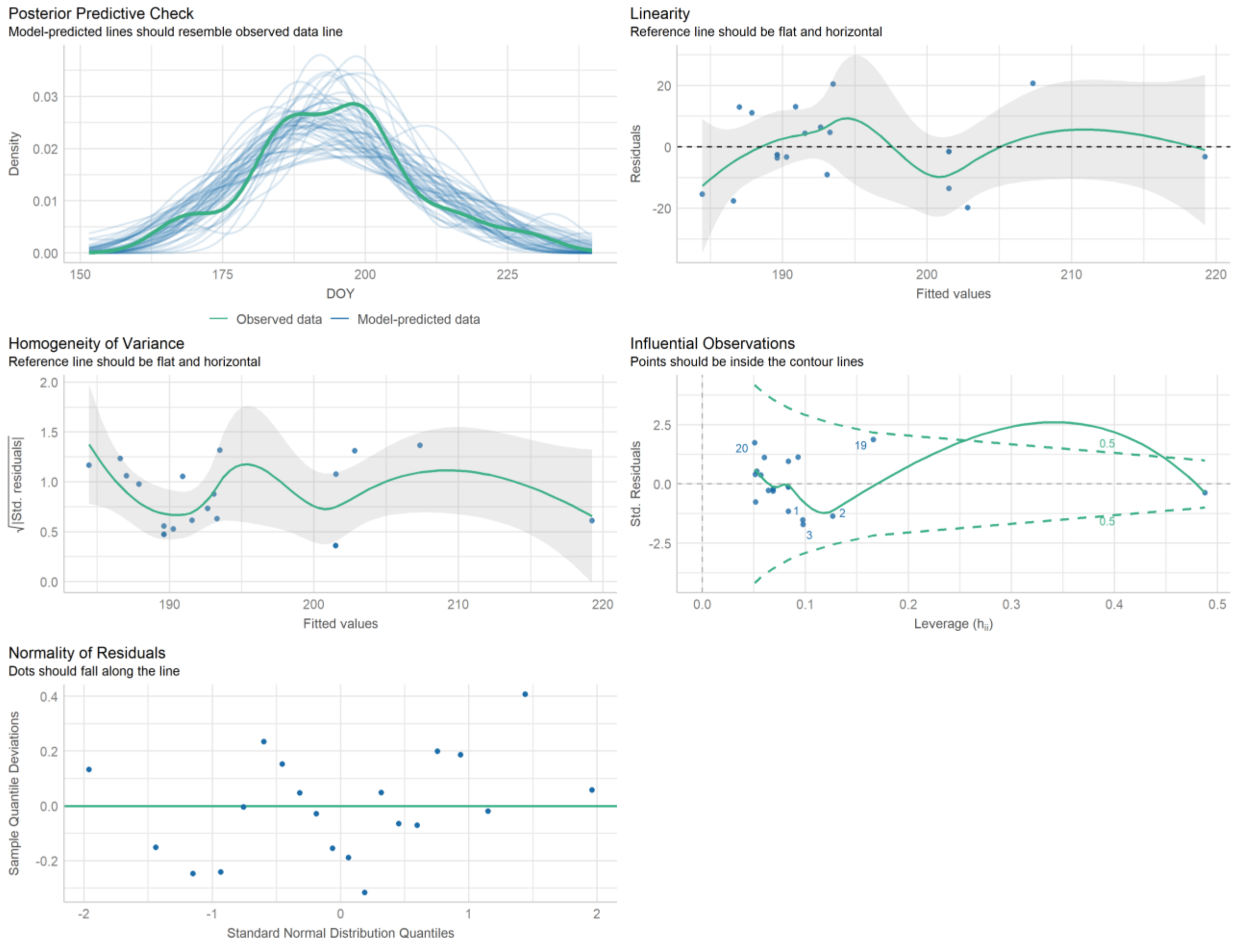

1.114. LM - DVG - Sideritis glacialis

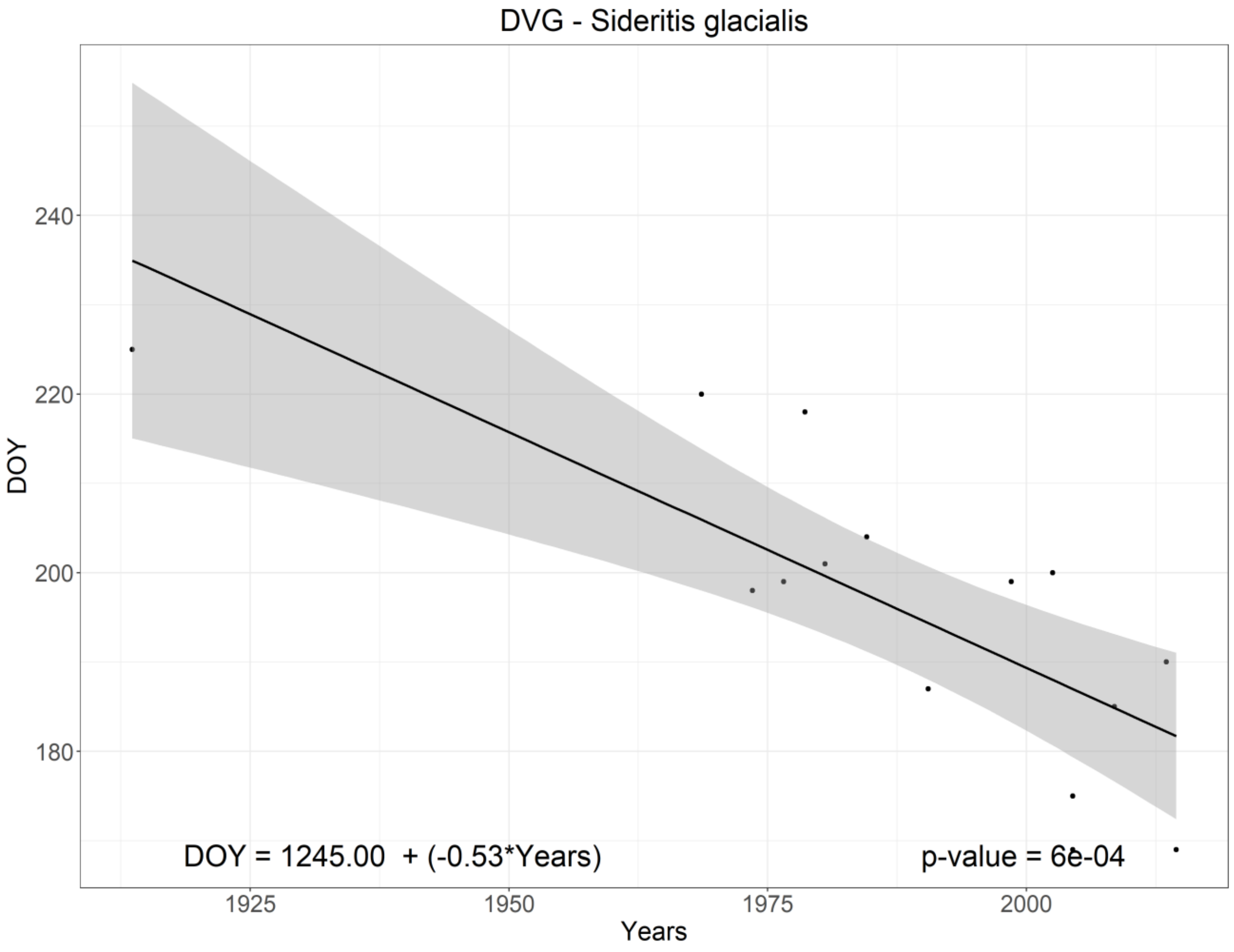

1.114.1. Diagnostics - LM - DVG - Sideritis glacialis

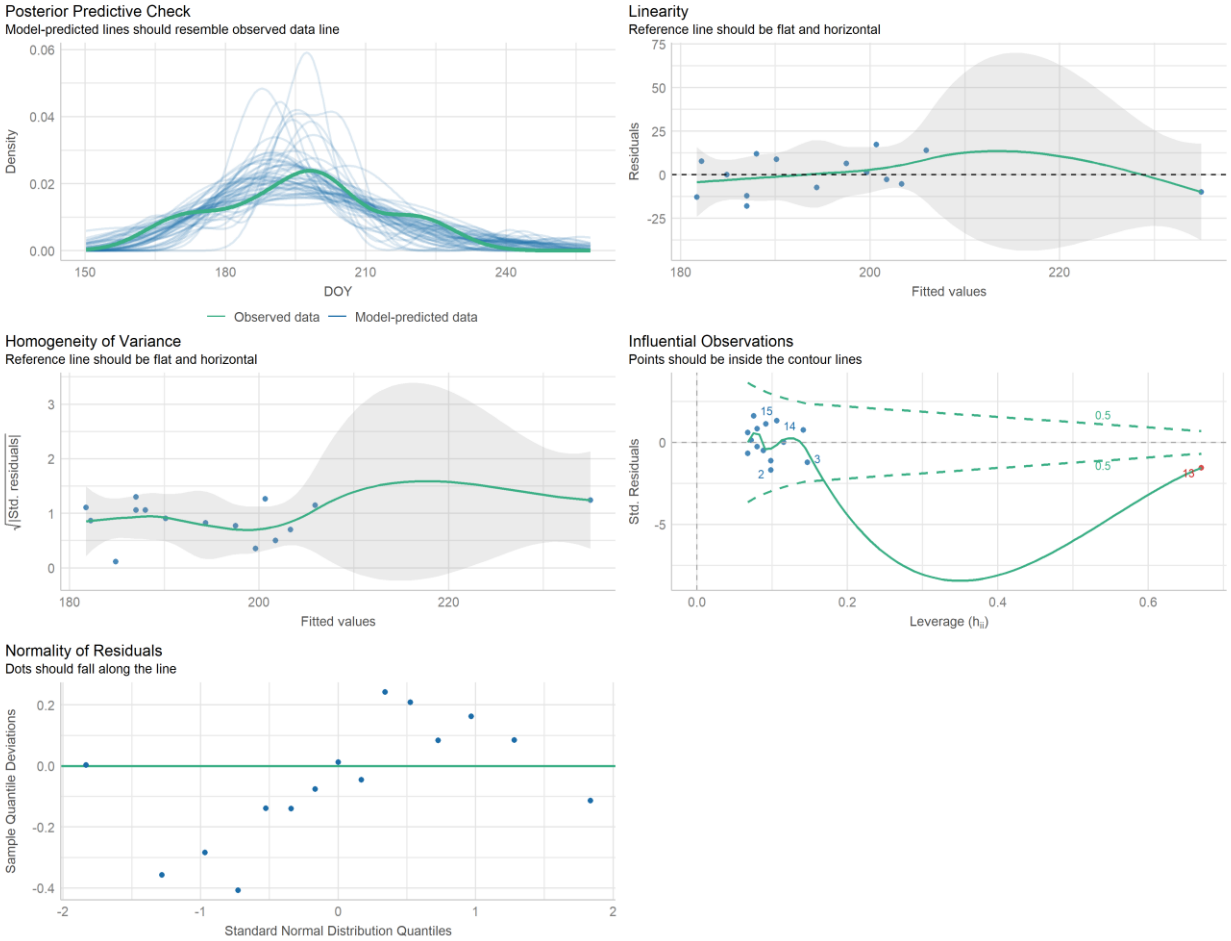

1.115. LM - F - Sideritis incana

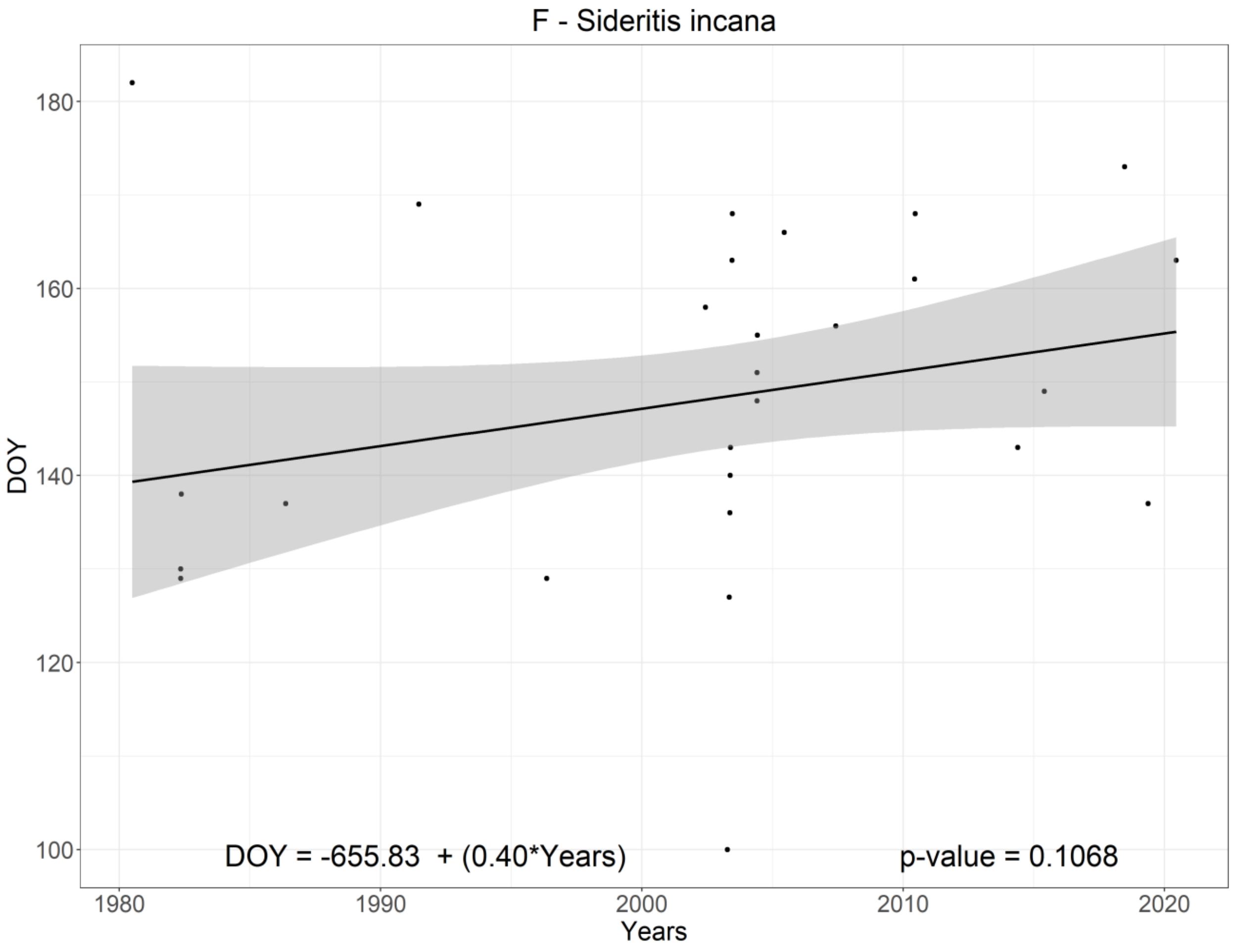

1.115.1. Diagnostics - LM - F - Sideritis incana

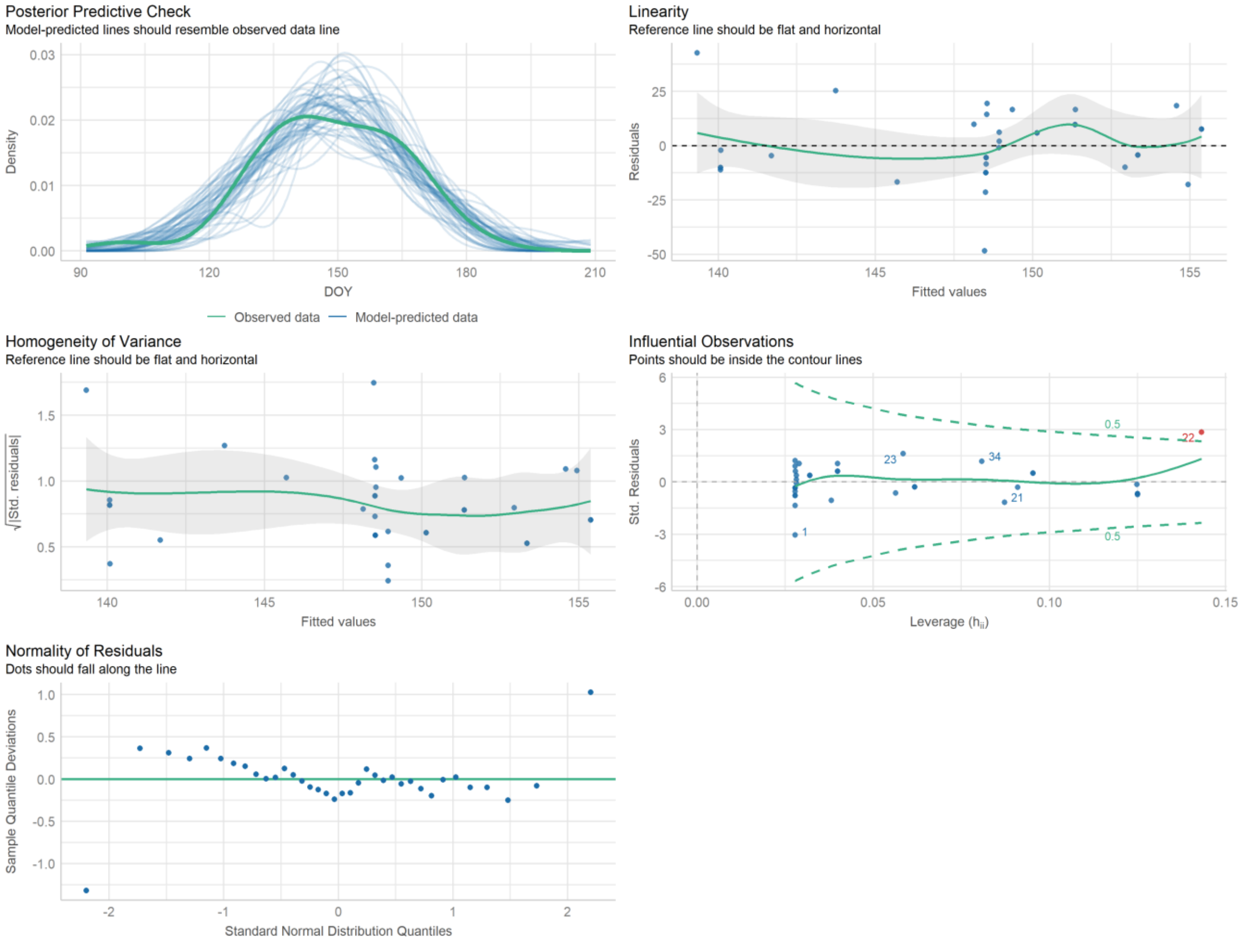

1.116. LM - DVG - Sideritis incana

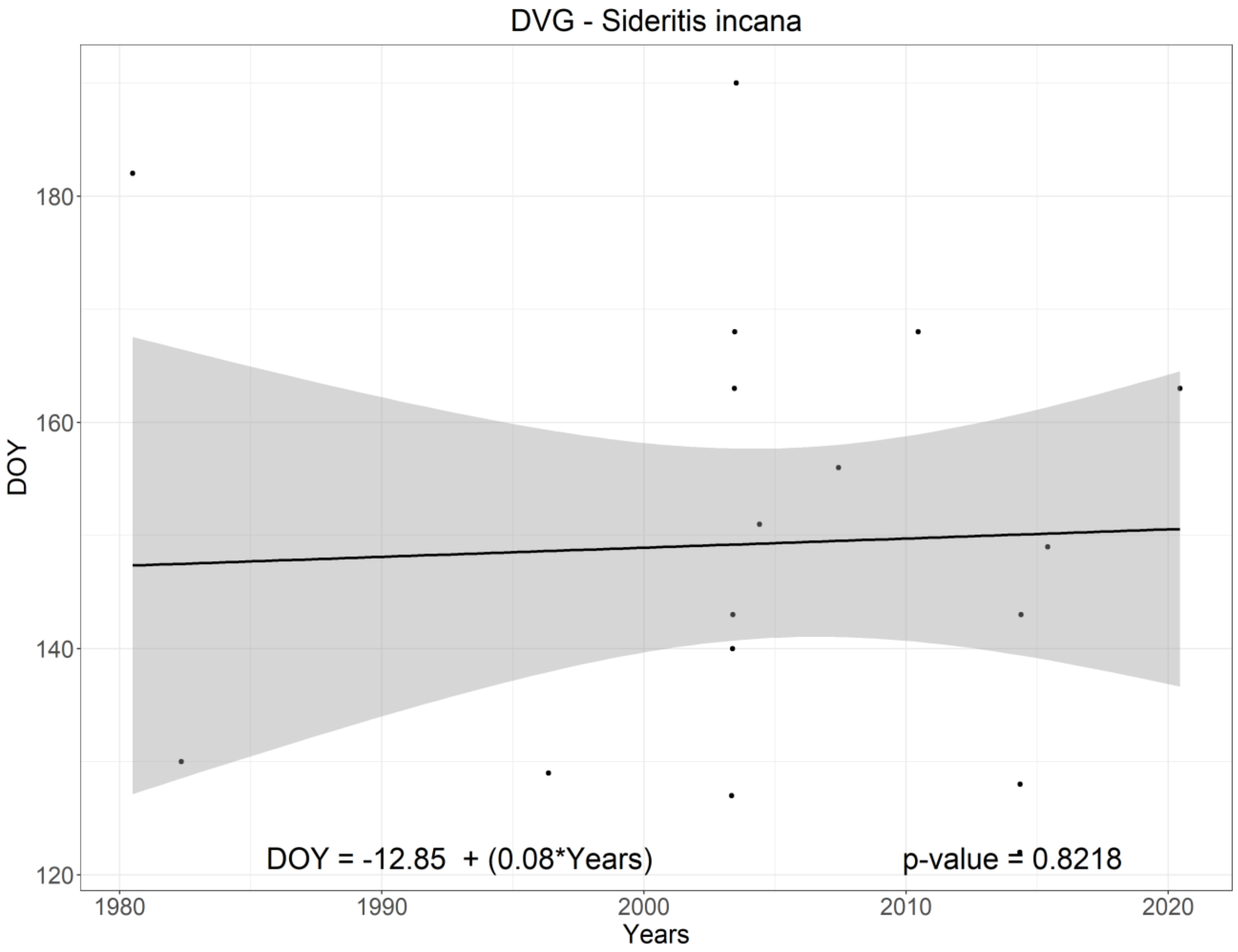

1.116.1. Diagnostics - LM - DVG - Sideritis incana

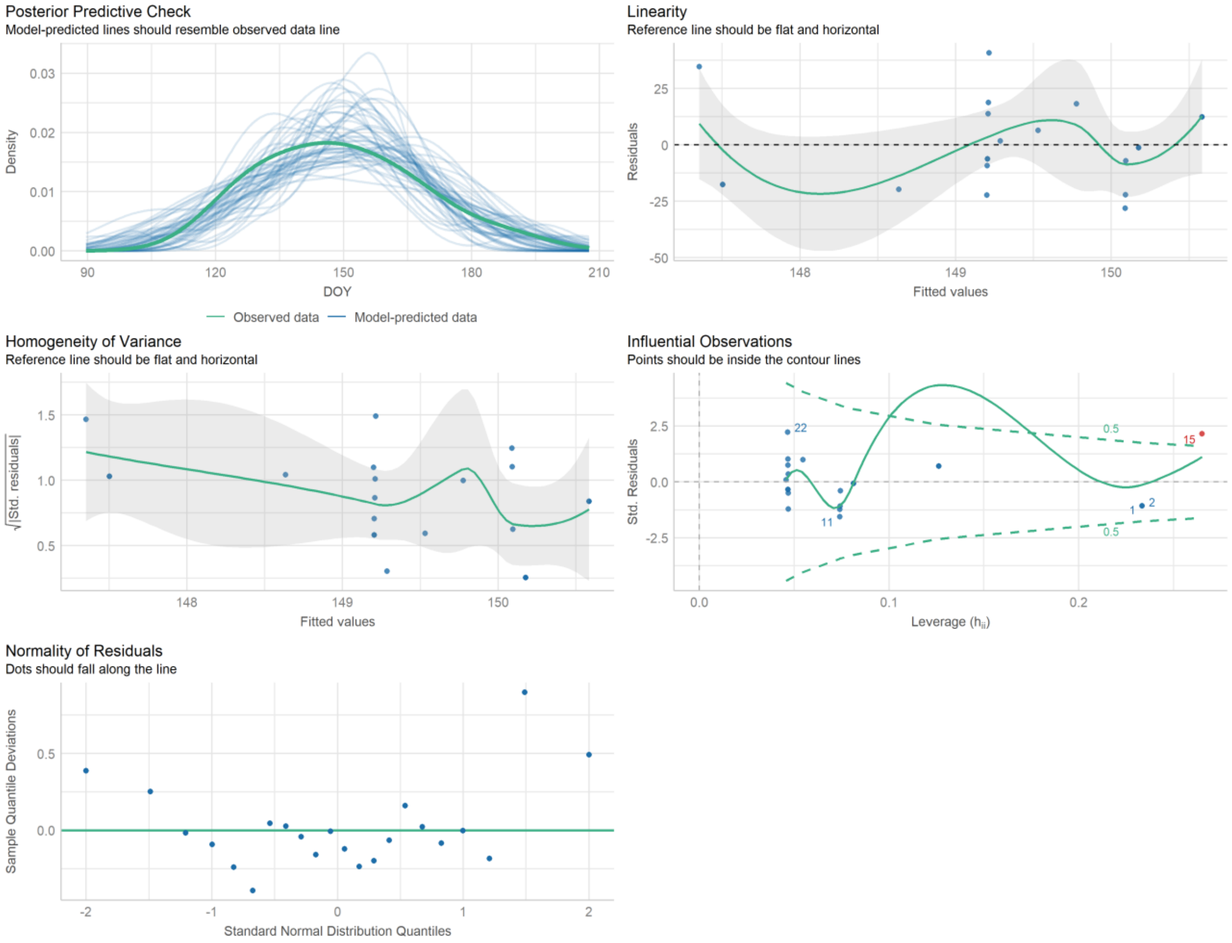

1.117. LM - F - Staehelina baetica

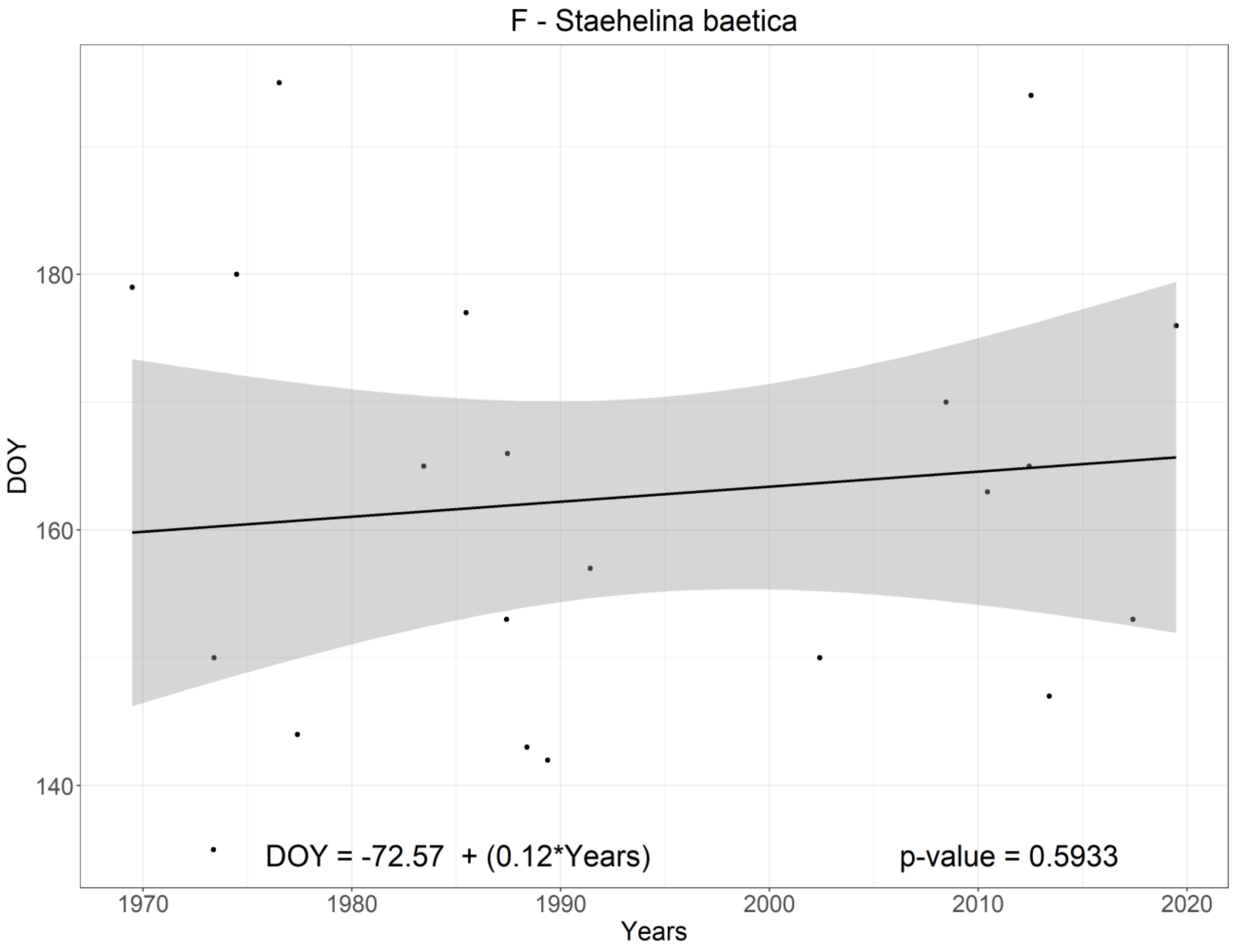

1.117.1. Diagnostics - LM - F - Staehelina baetica

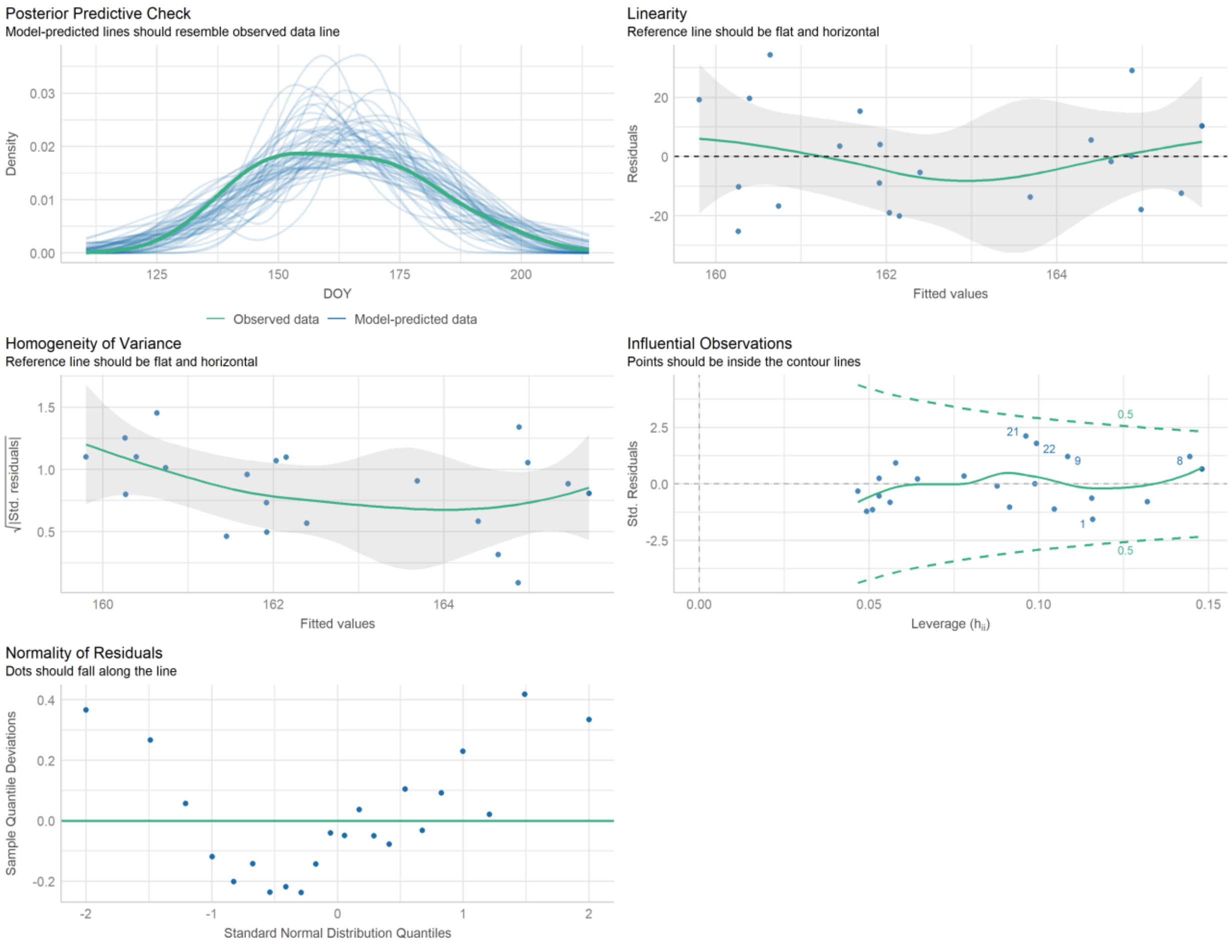

1.118. LM - FBF - Thymbra capitata

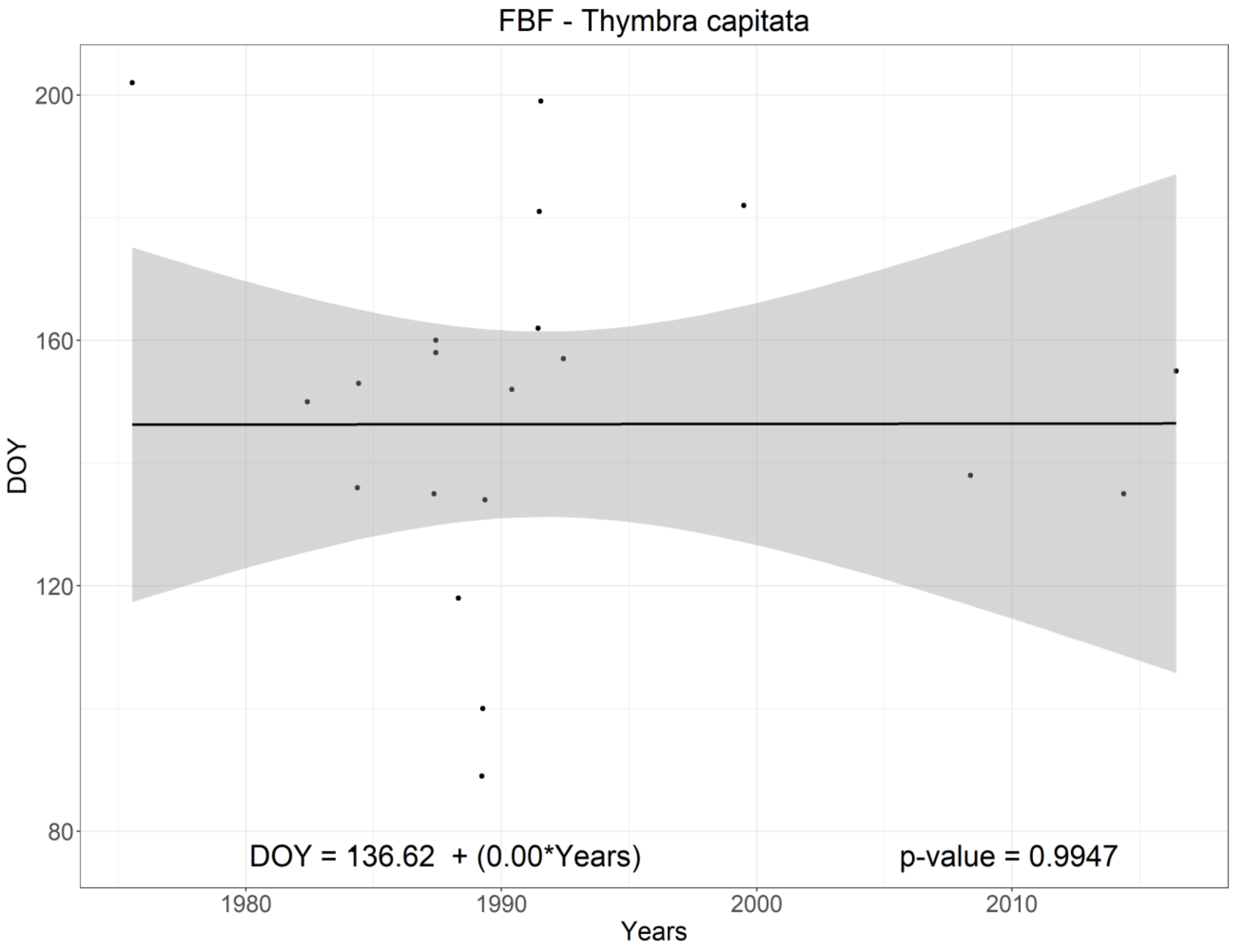

1.118.1. Diagnostics - LM - FBF - Thymbra capitata

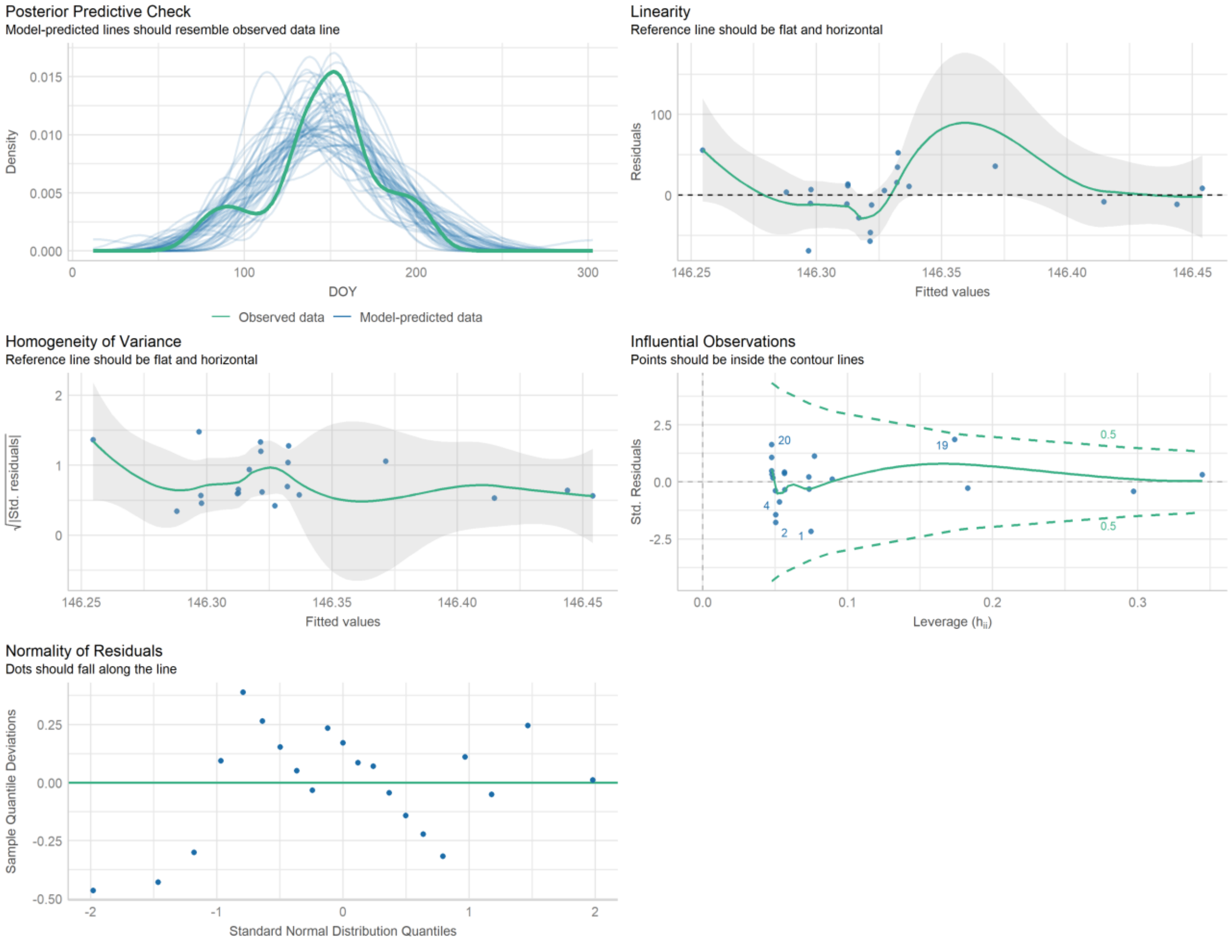

1.119. LM - F - Thymbra capitata

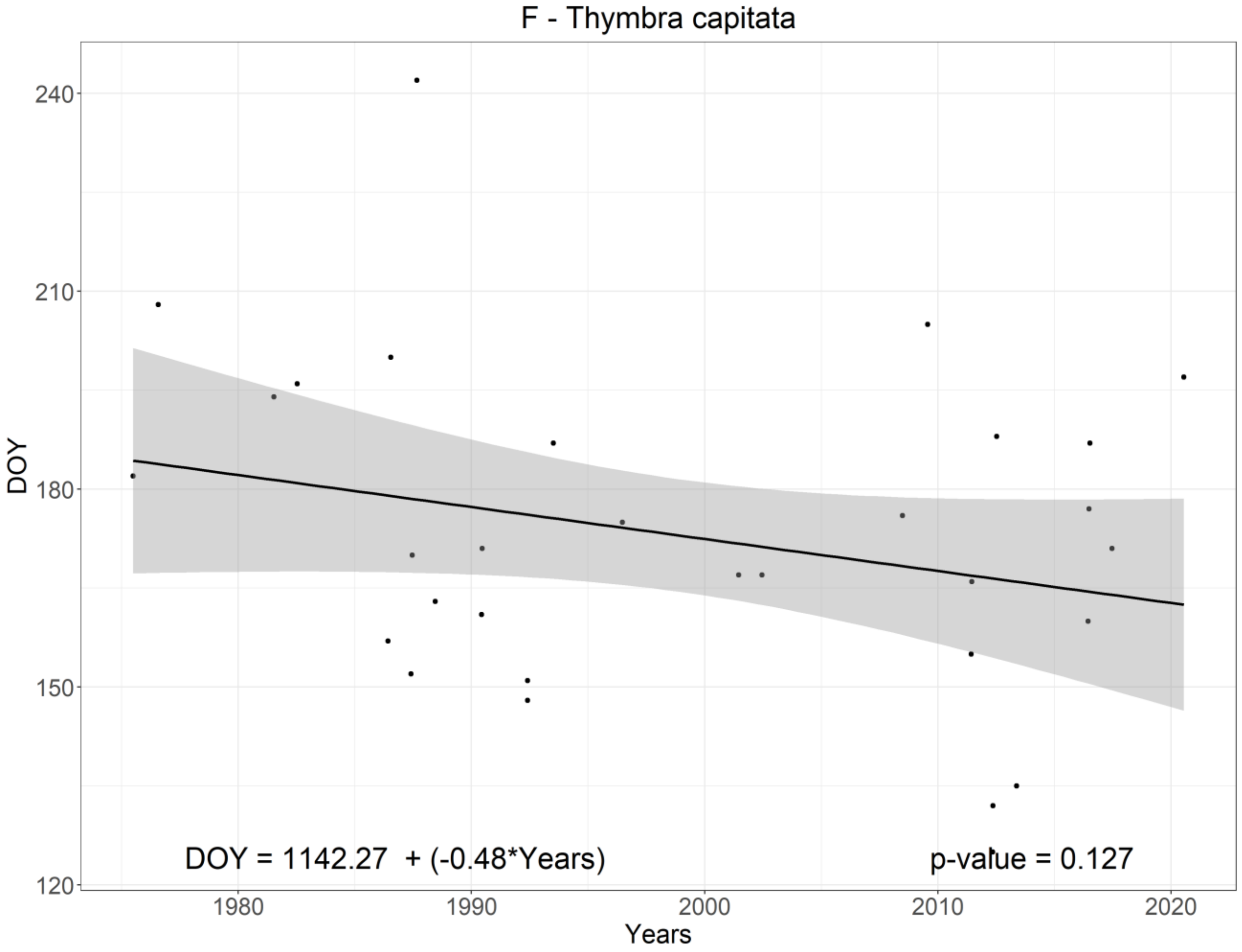

1.119.1. Diagnostics - LM - F - Thymbra capitata

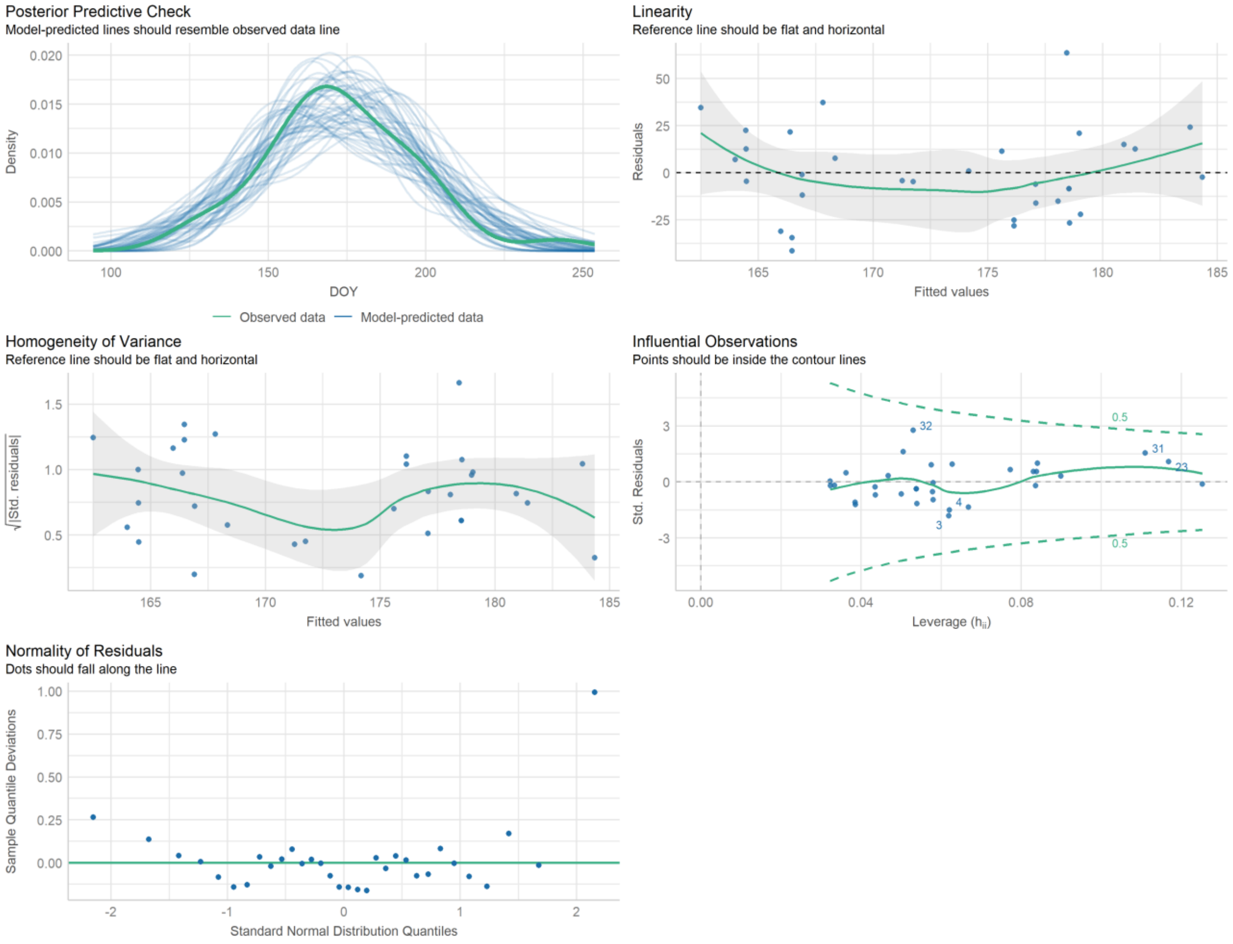

1.120. LM - DVG - Thymbra capitata

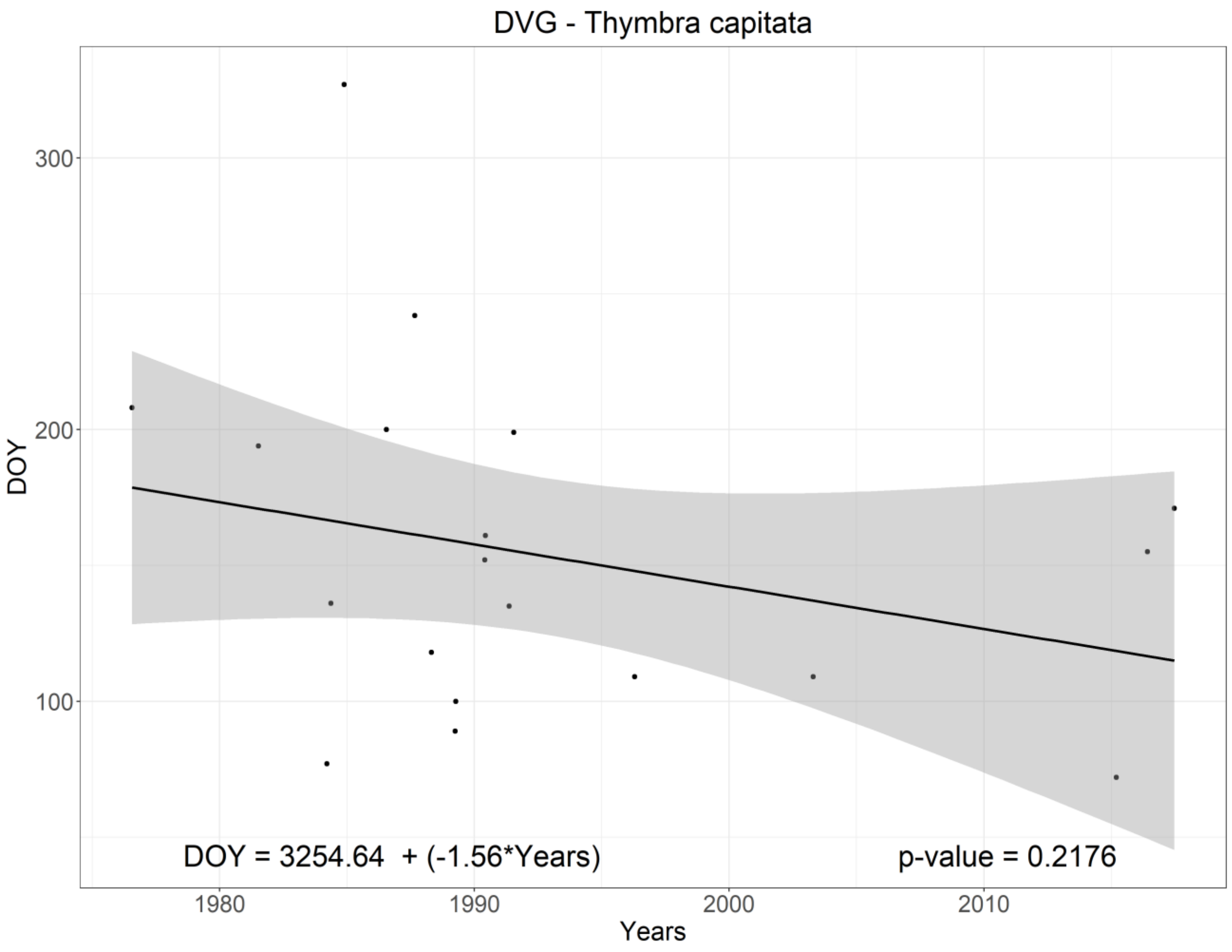

1.120.1. Diagnostics - LM - DVG - Thymbra capitata

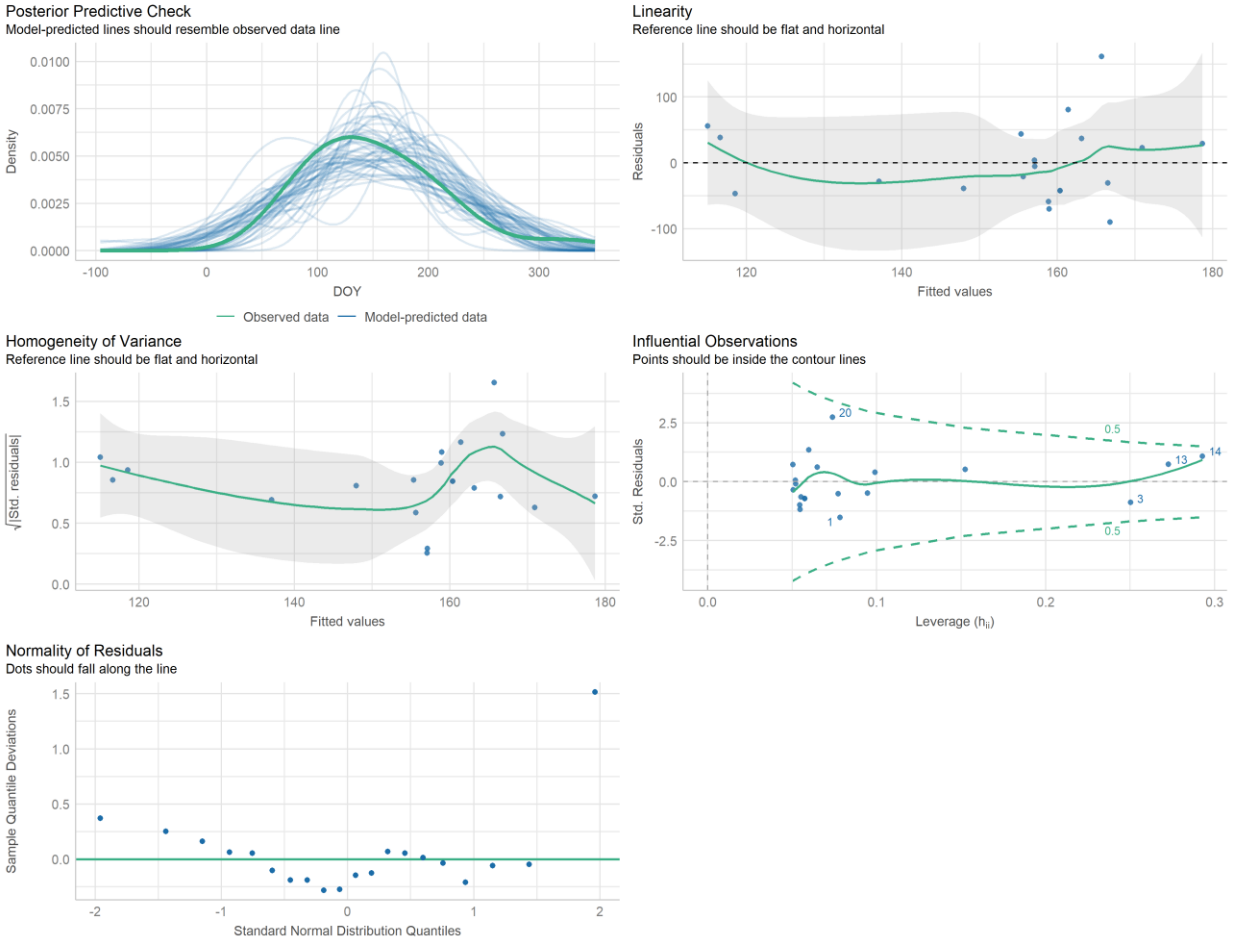

1.121. LM - F - Thymus longiflorus

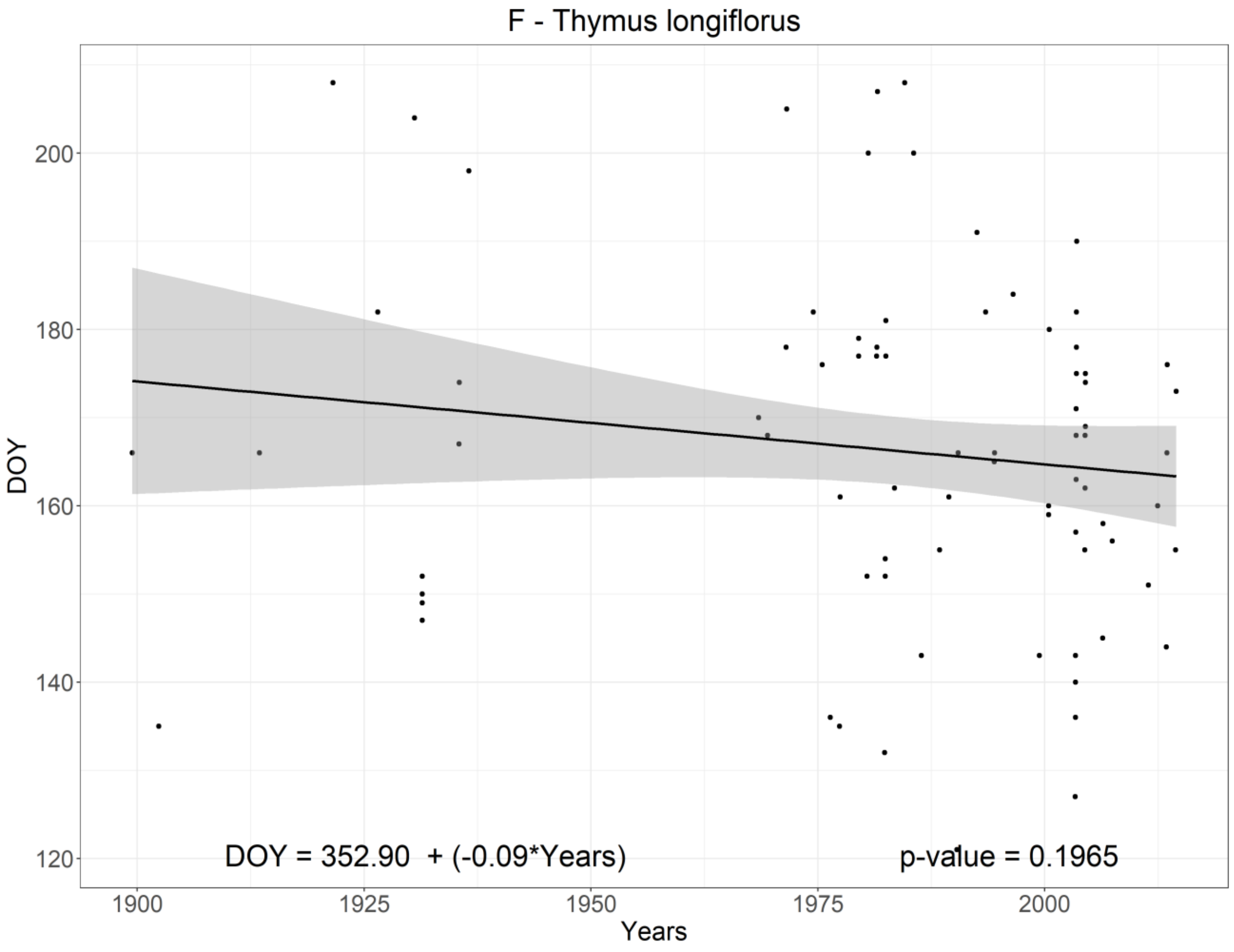

1.121.1. Diagnostics - LM - F - Thymus longiflorus

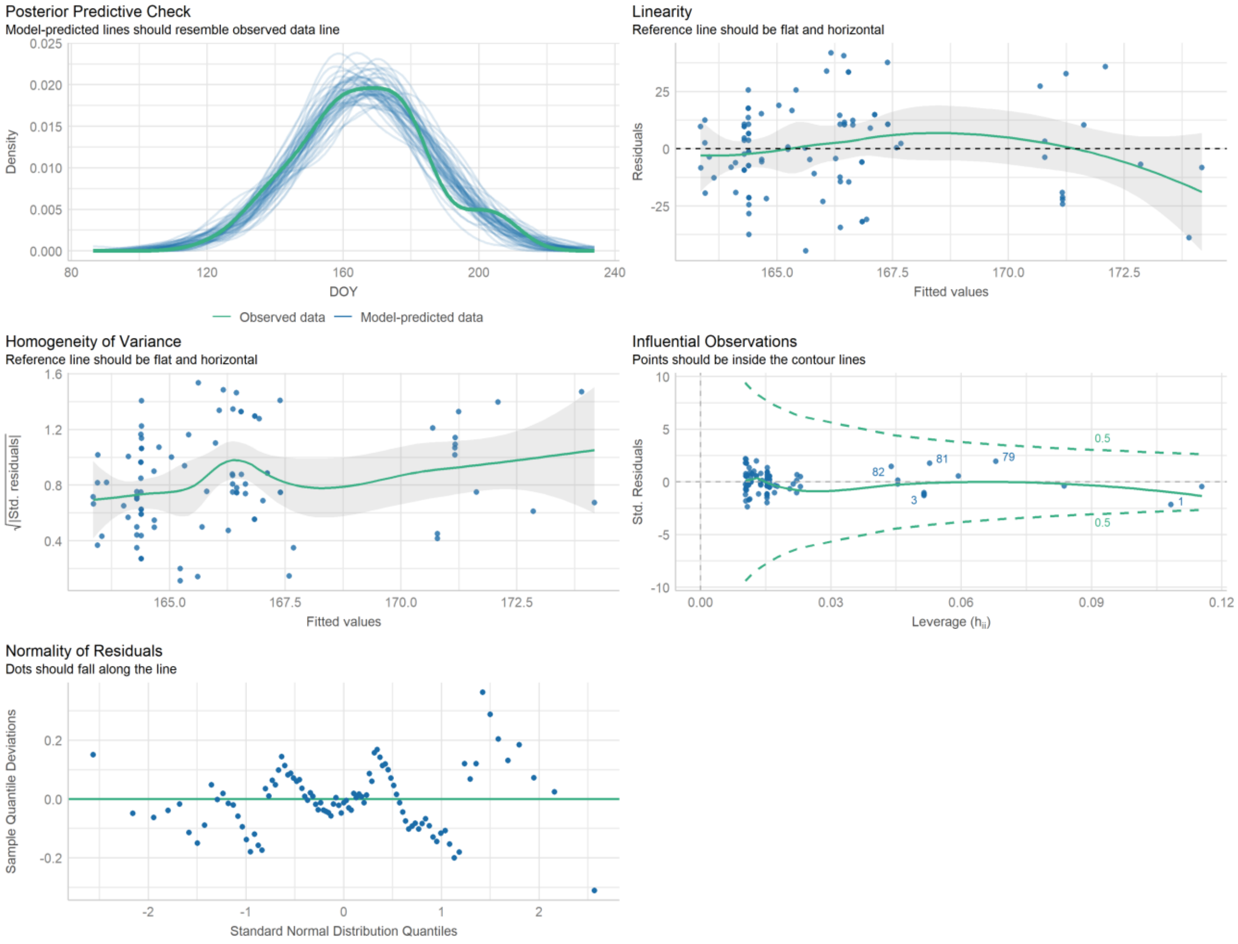

1.122. LM - FBF - Thymus mastichina

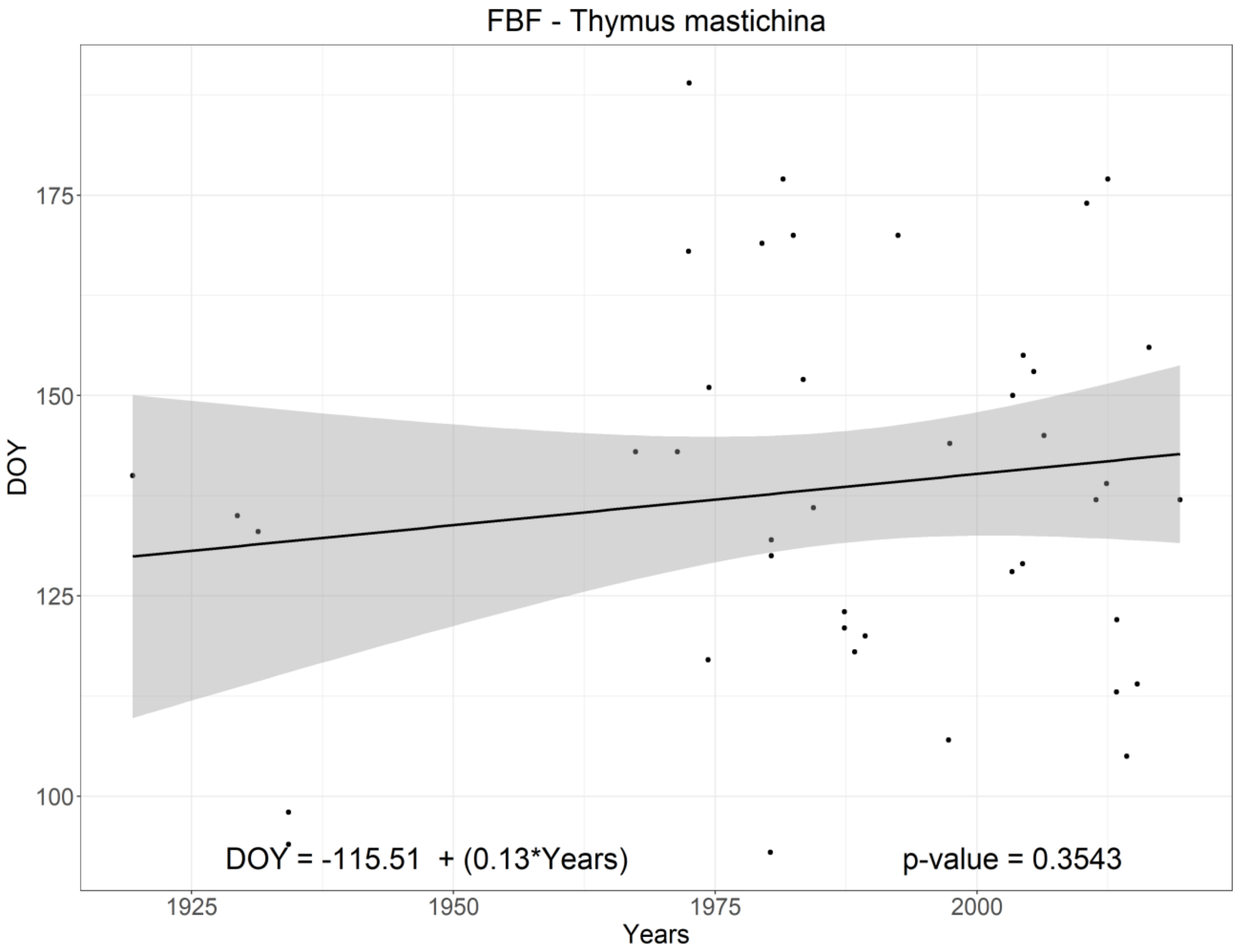

1.122.1. Diagnostics - LM - FBF - Thymus mastichina

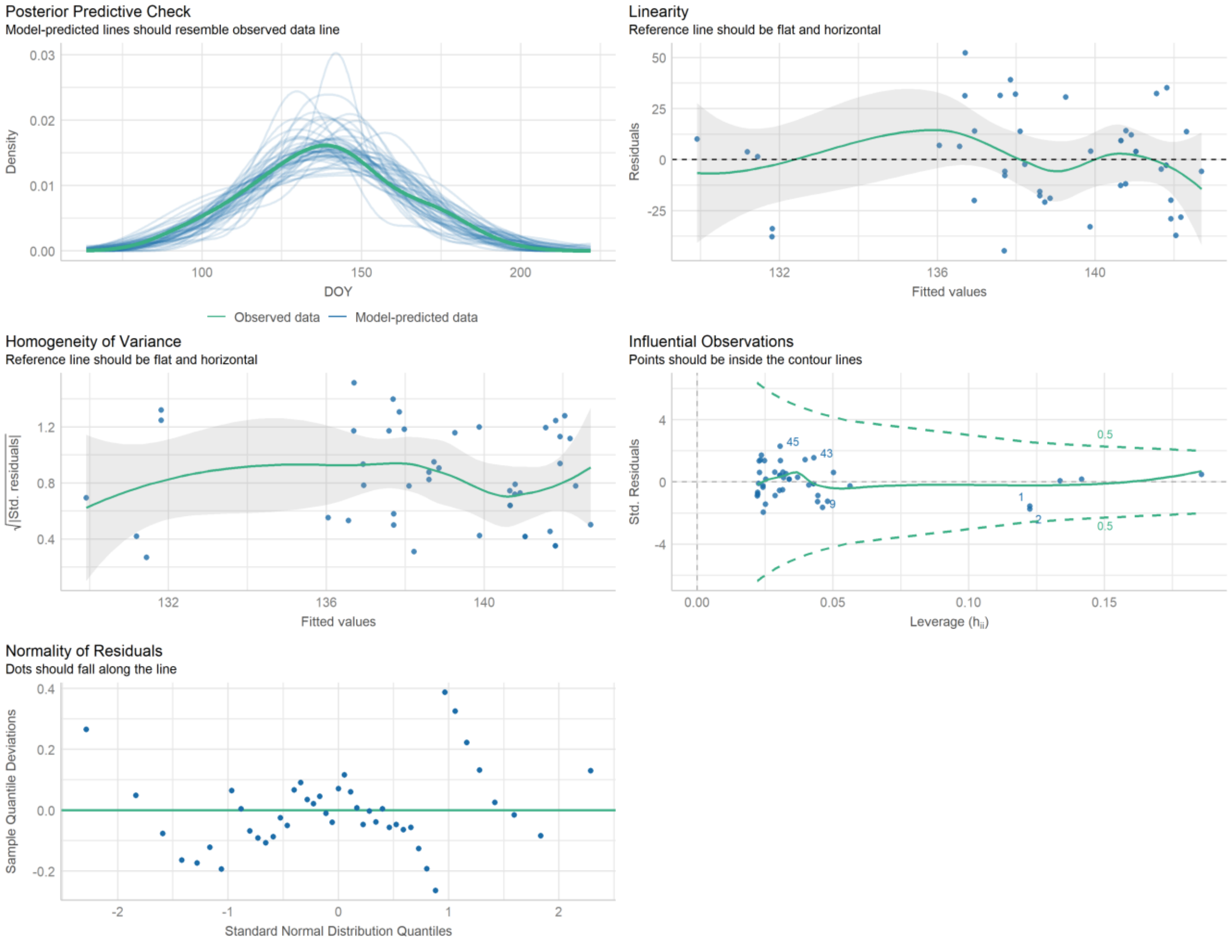

1.123. LM - F - Thymus mastichina

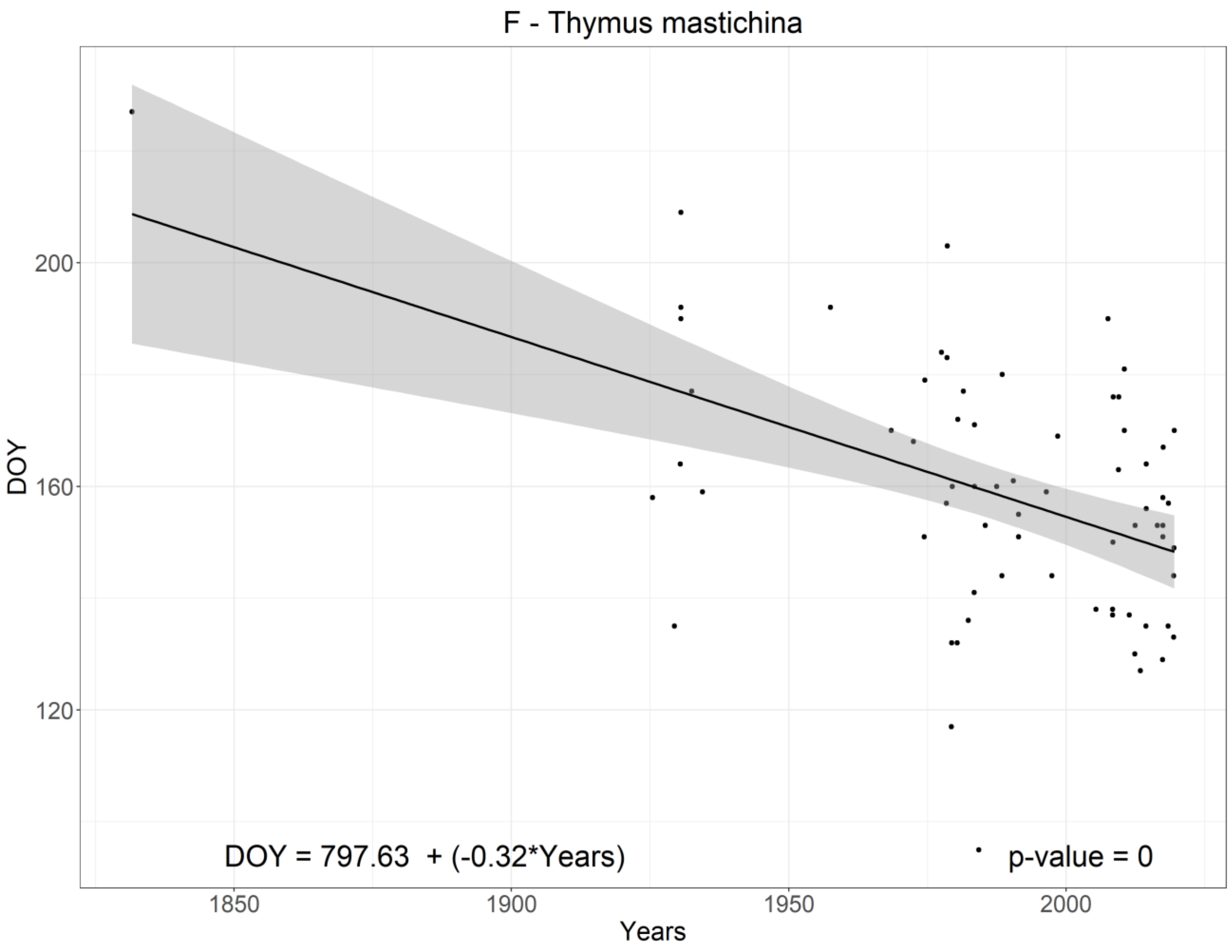

1.123.1. Diagnostics - LM - F - Thymus mastichina

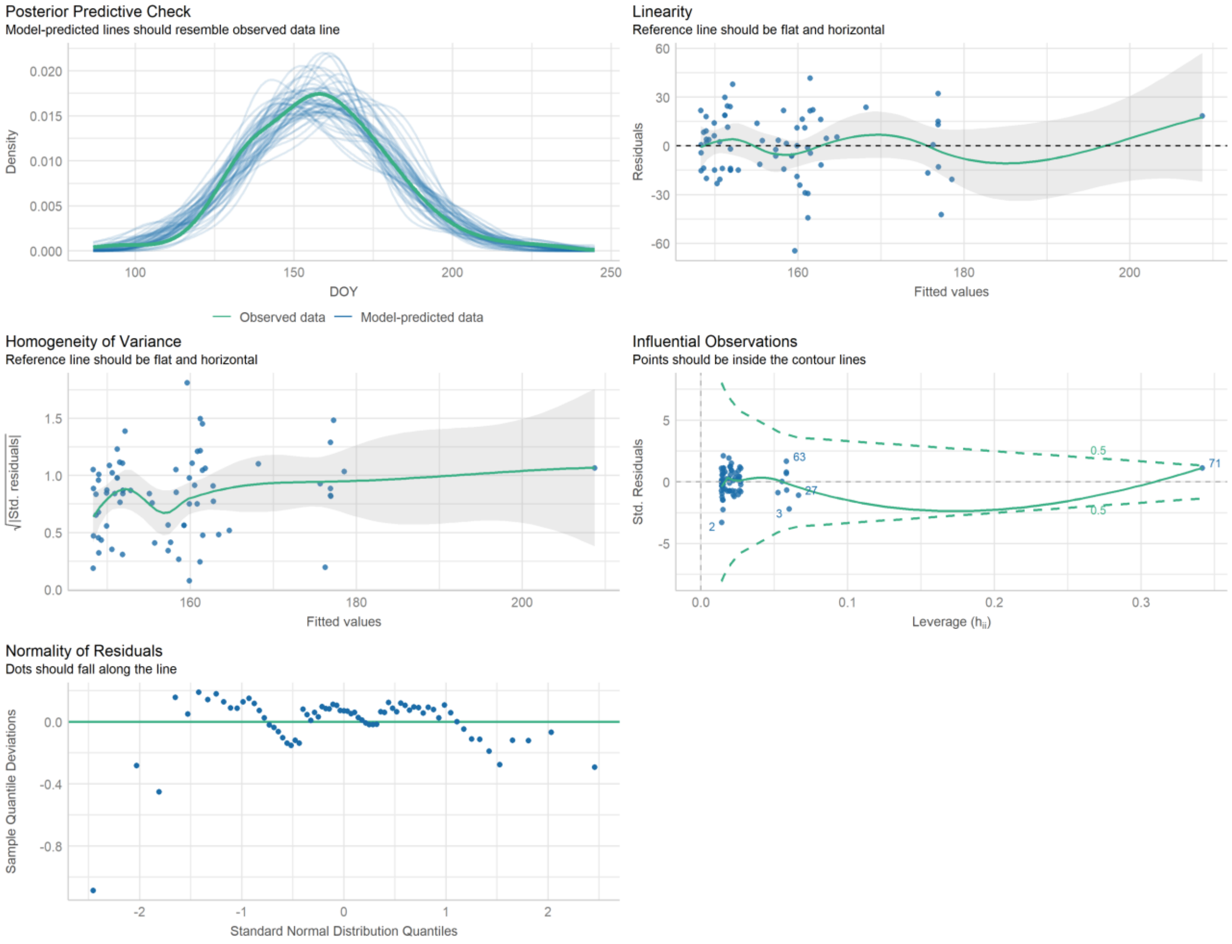

1.124. LM - FS - Thymus mastichina

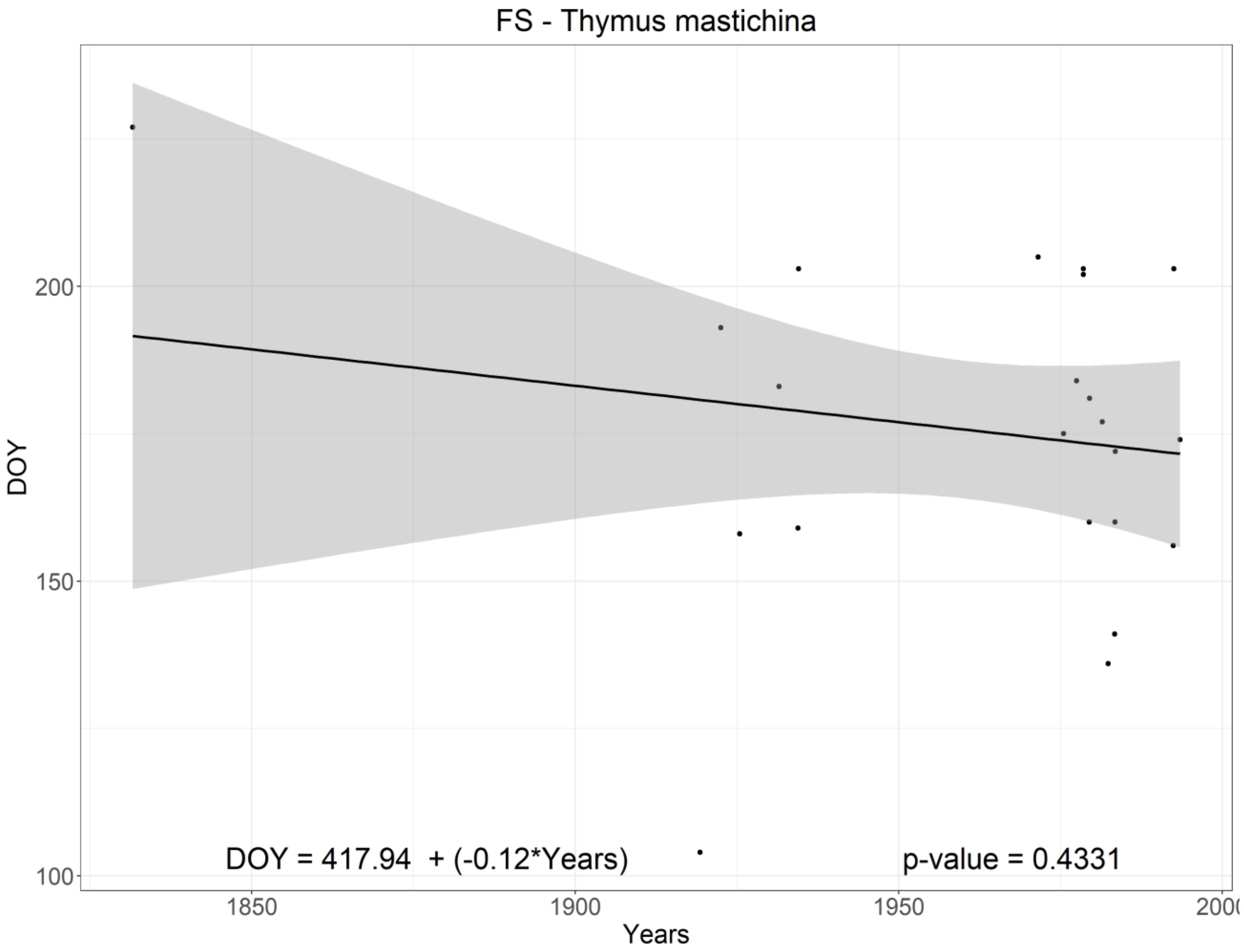

1.124.1. Diagnostics - LM - FS - Thymus mastichina

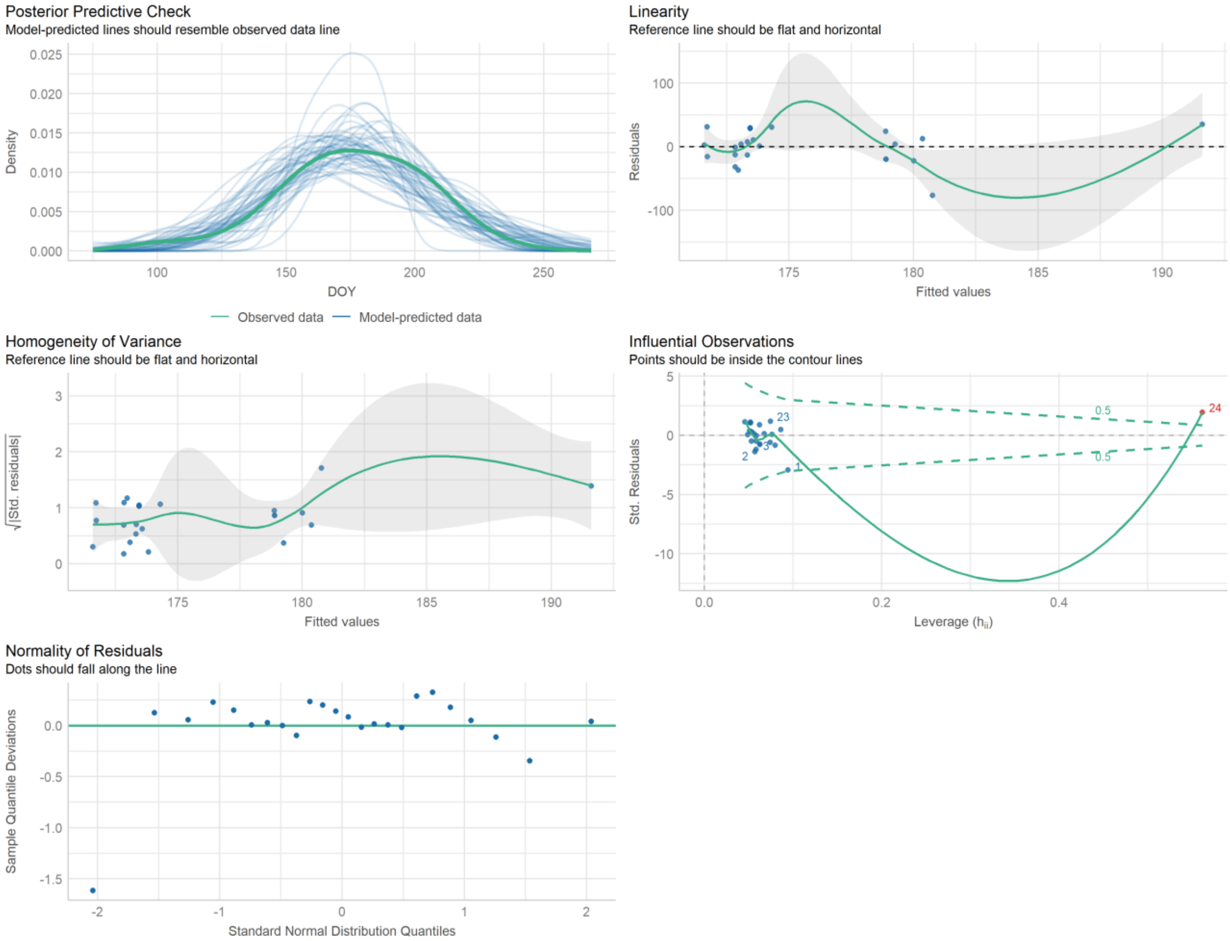

1.125. LM - DVG - Thymus mastichina

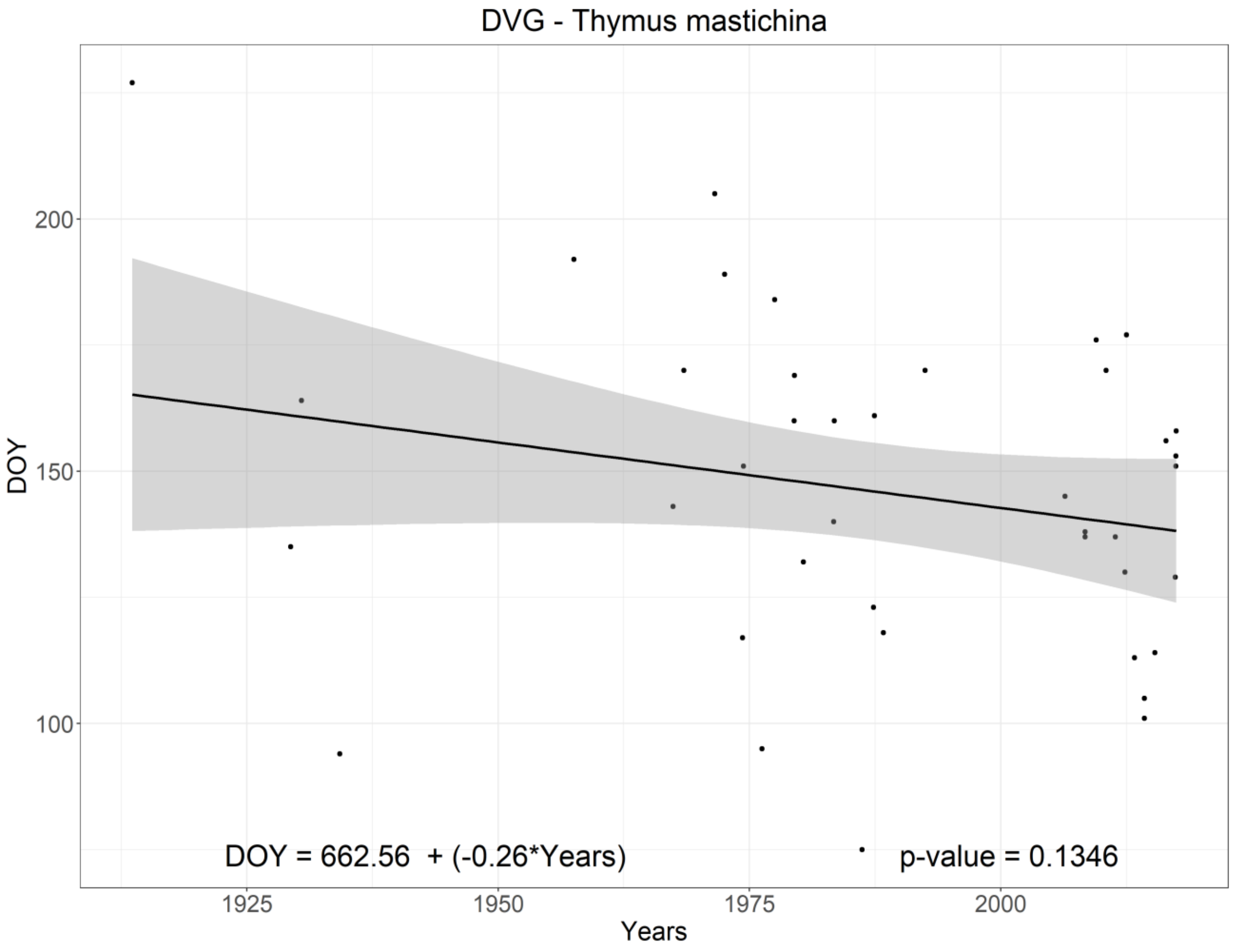

1.125.1. Diagnostics - LM - DVG - Thymus mastichina

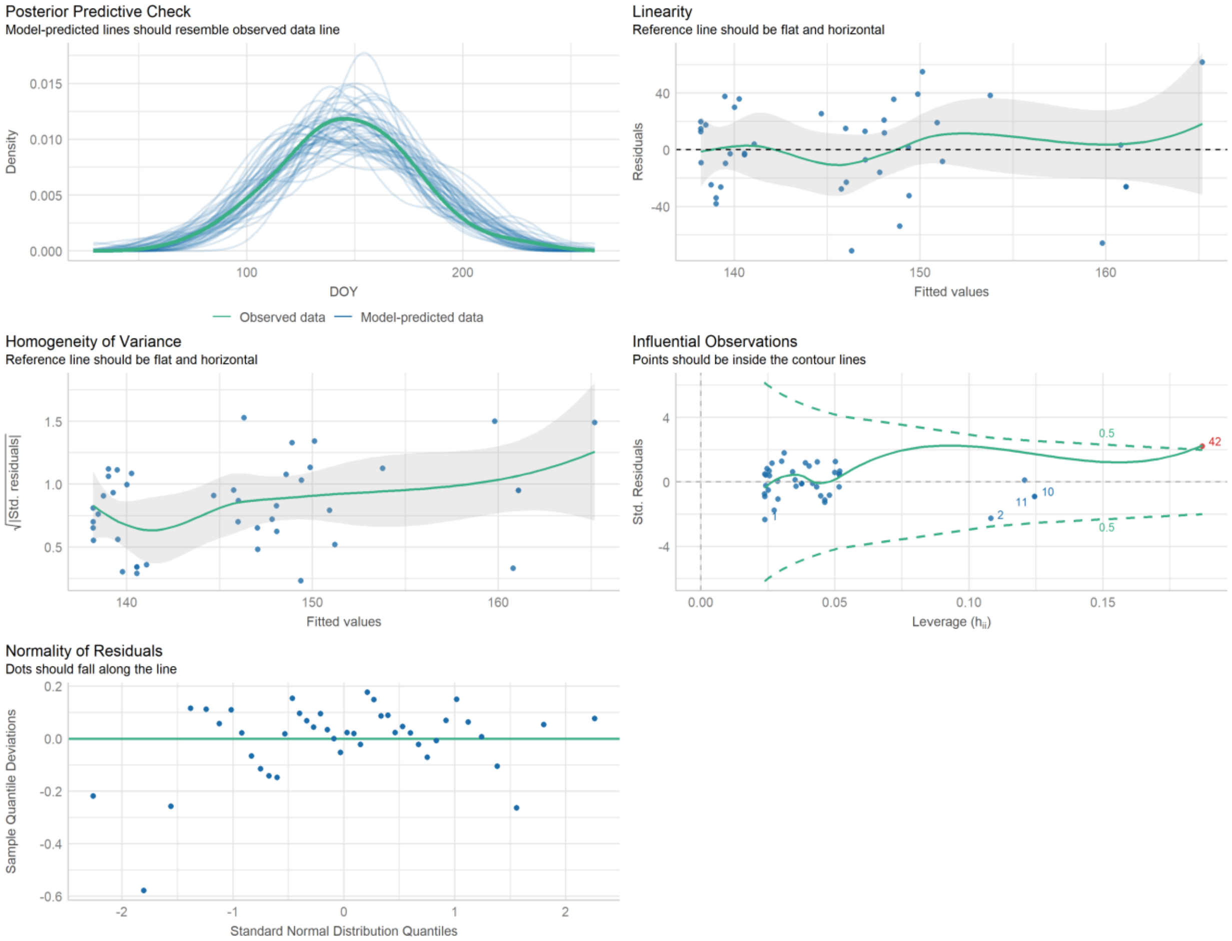

1.126. LM - FBF - Ulex parviflorus

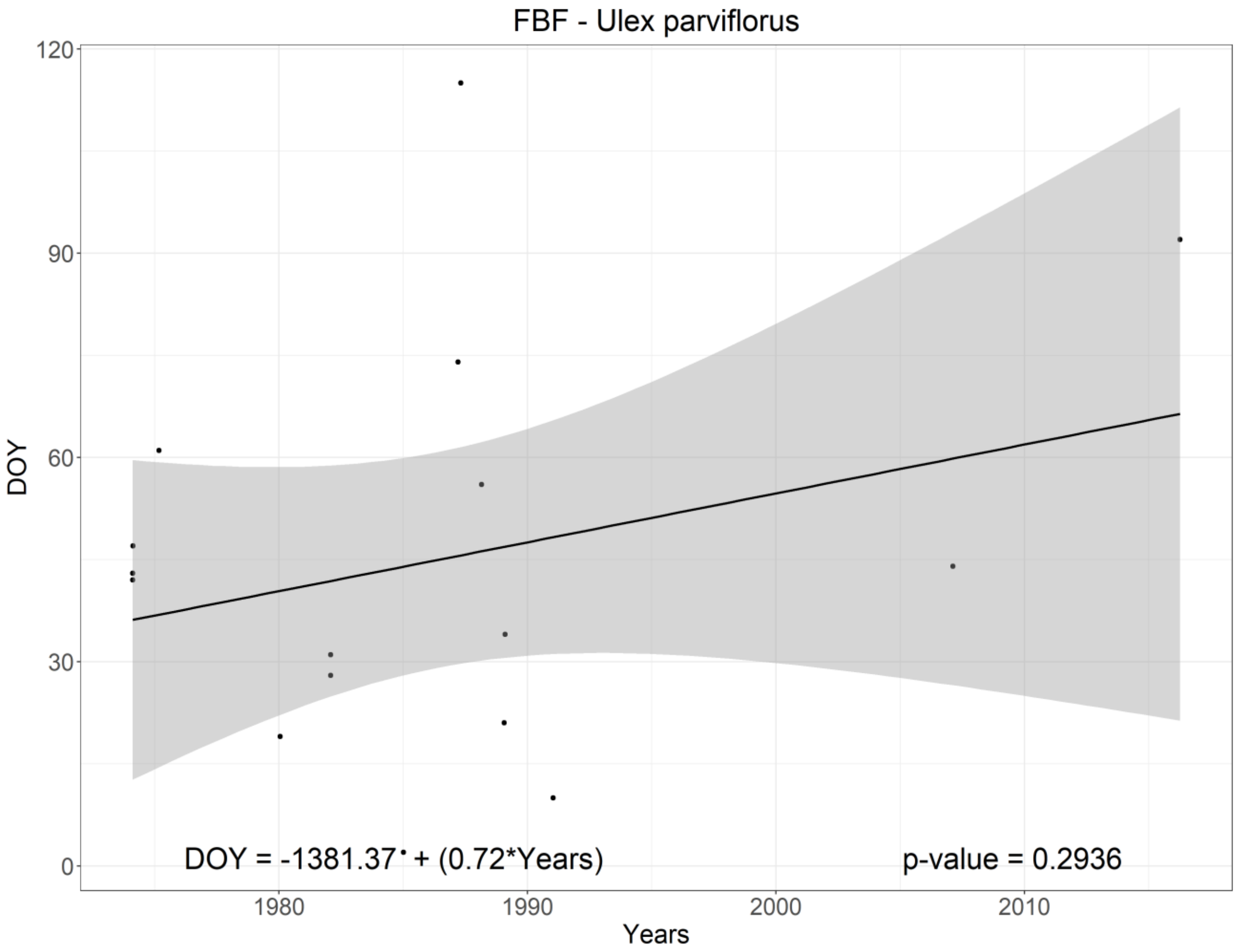

1.126.1. Diagnostics - LM - FBF - Ulex parviflorus

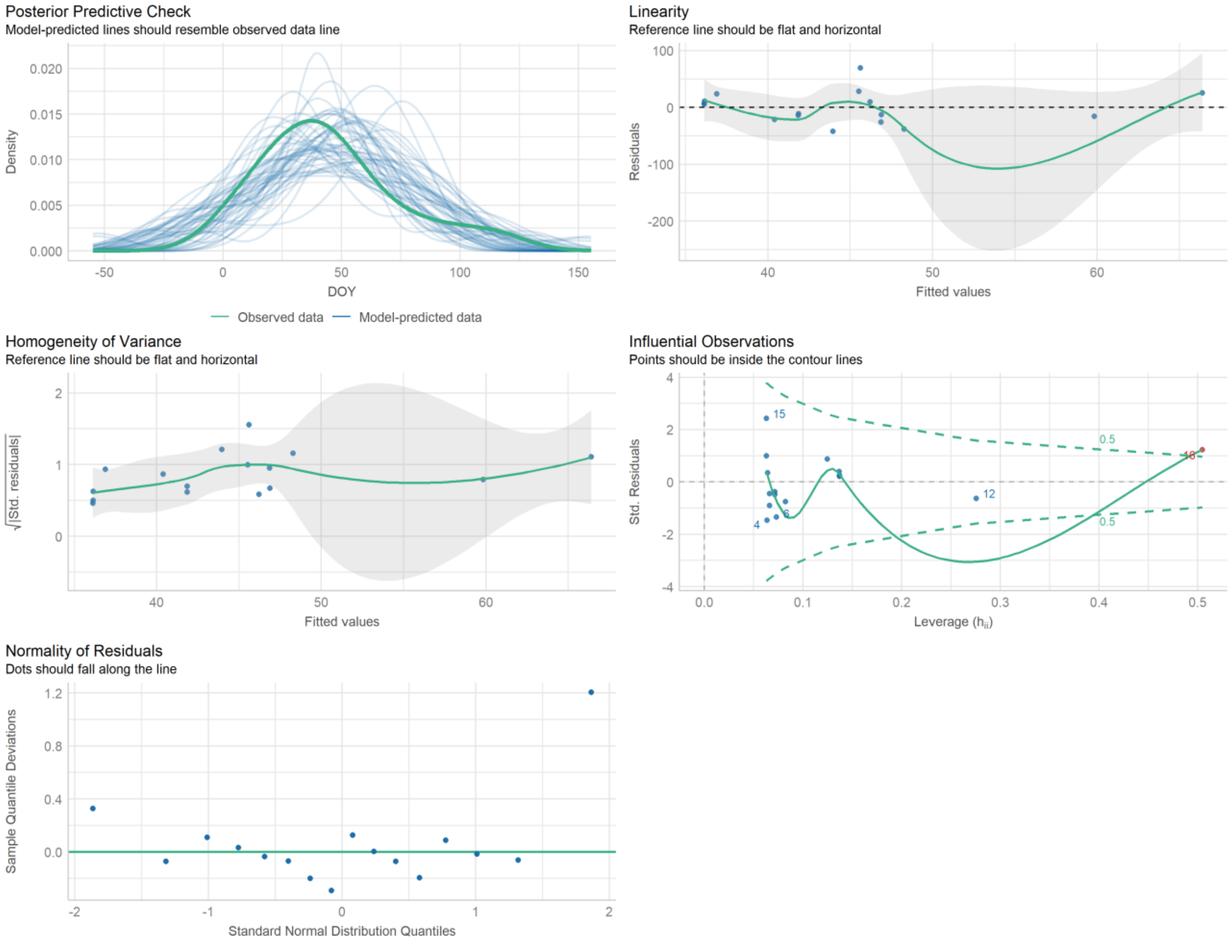

1.127. LM - F - Ulex parviflorus

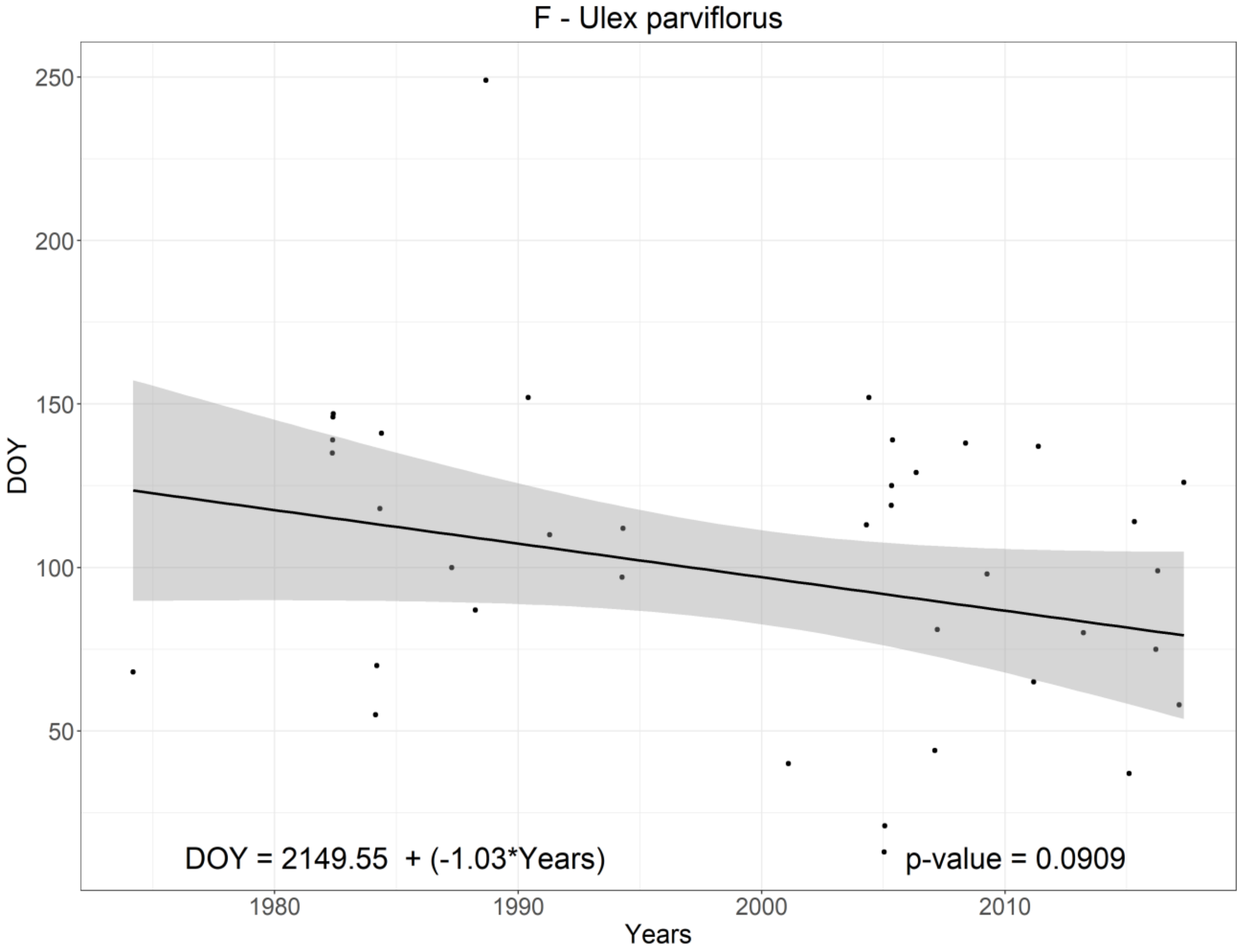

1.127.1. Diagnostics - LM - F - Ulex parviflorus

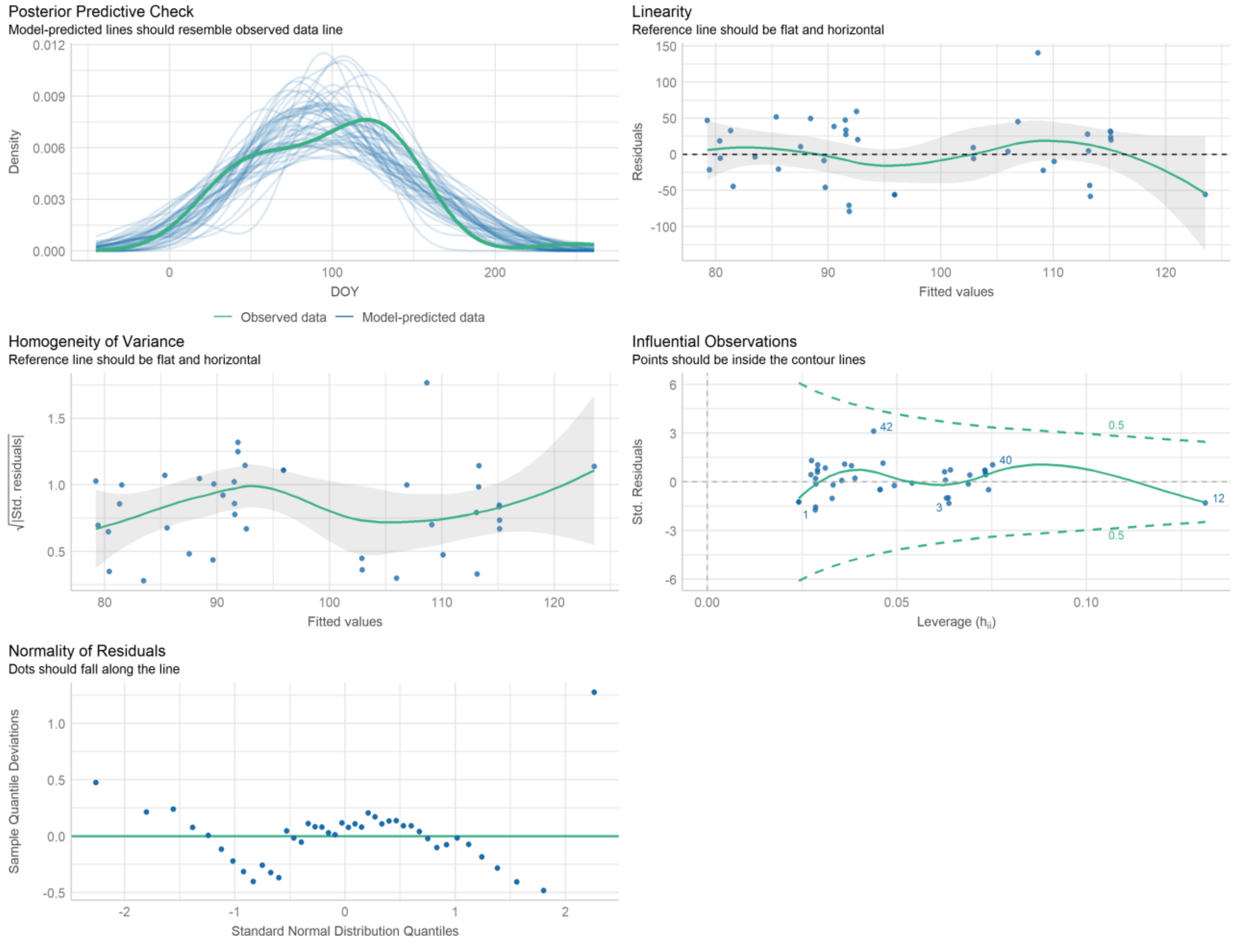

1.128. LM - DVG - Ulex parviflorus

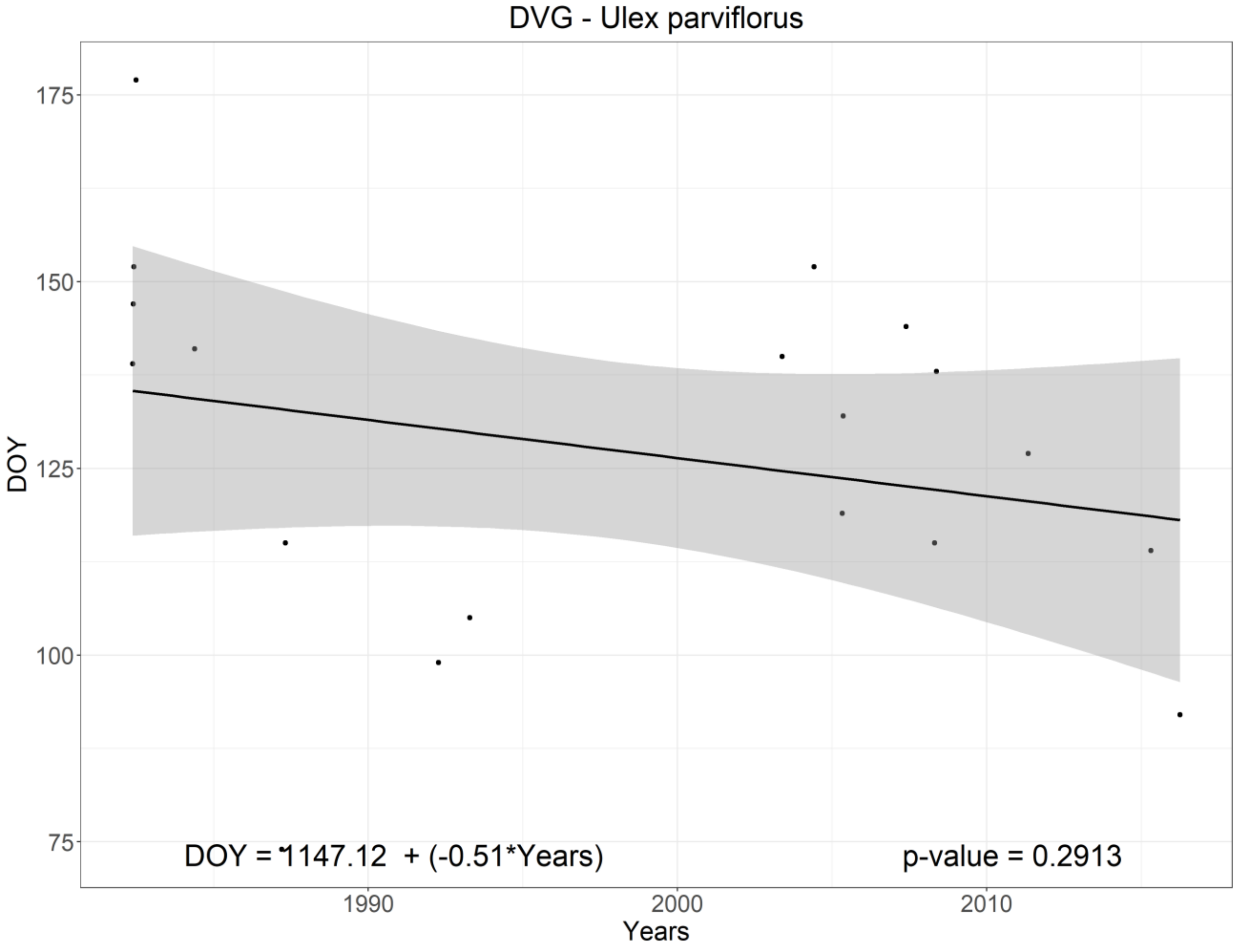

1.128.1. Diagnostics - LM - DVG - Ulex parviflorus

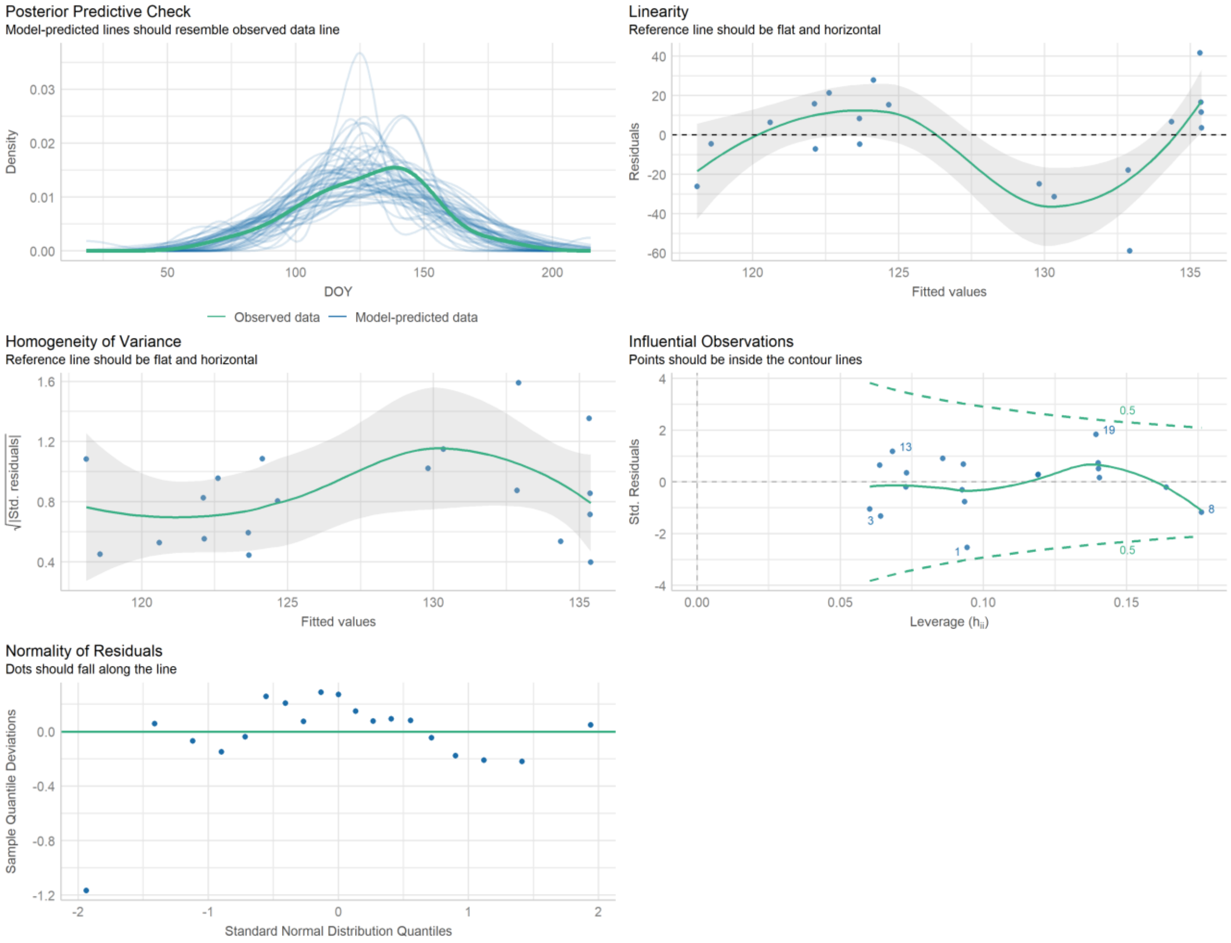

1.129. LM - F - Viburnum tinus

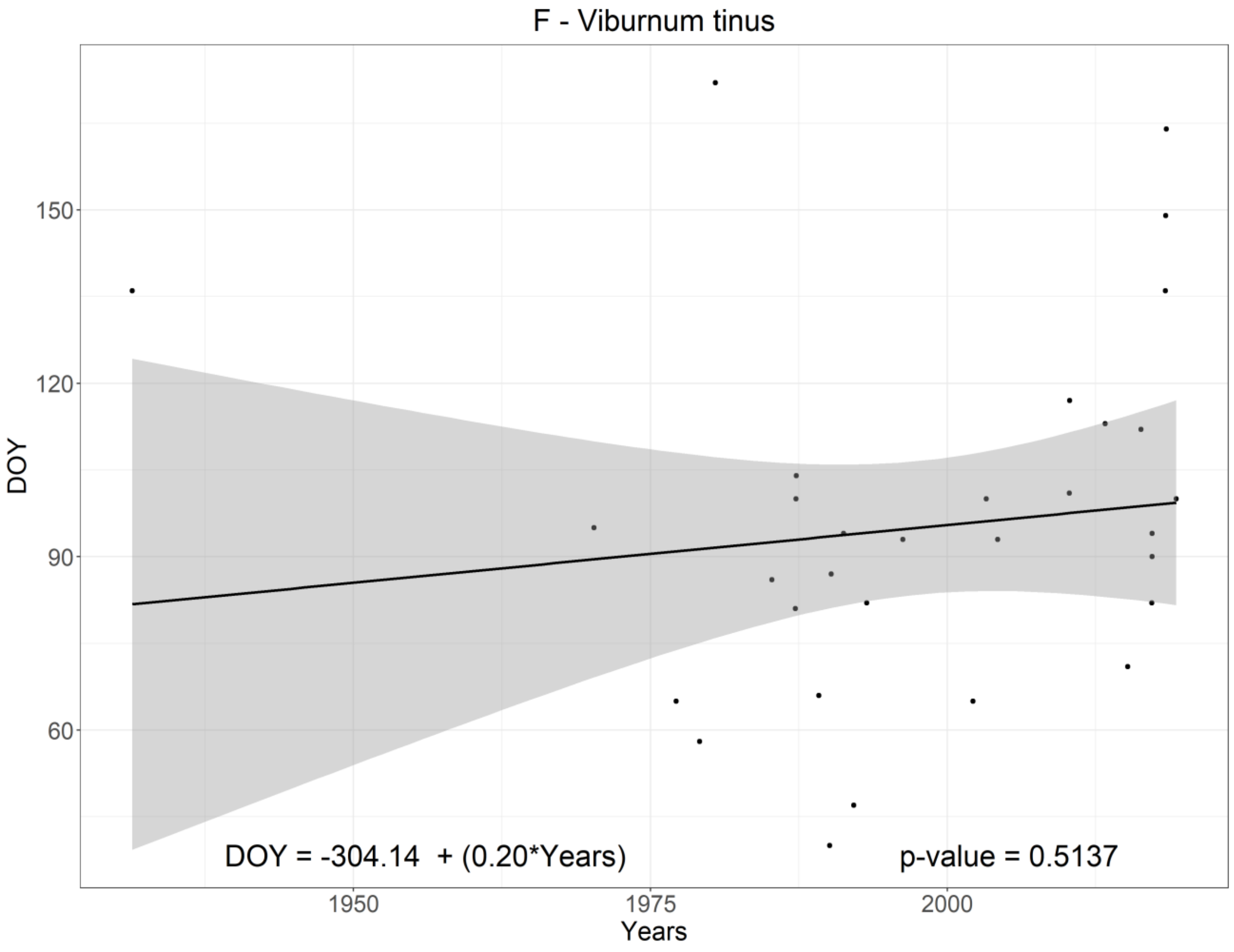

1.129.1. Diagnostics - LM - F - Viburnum tinus

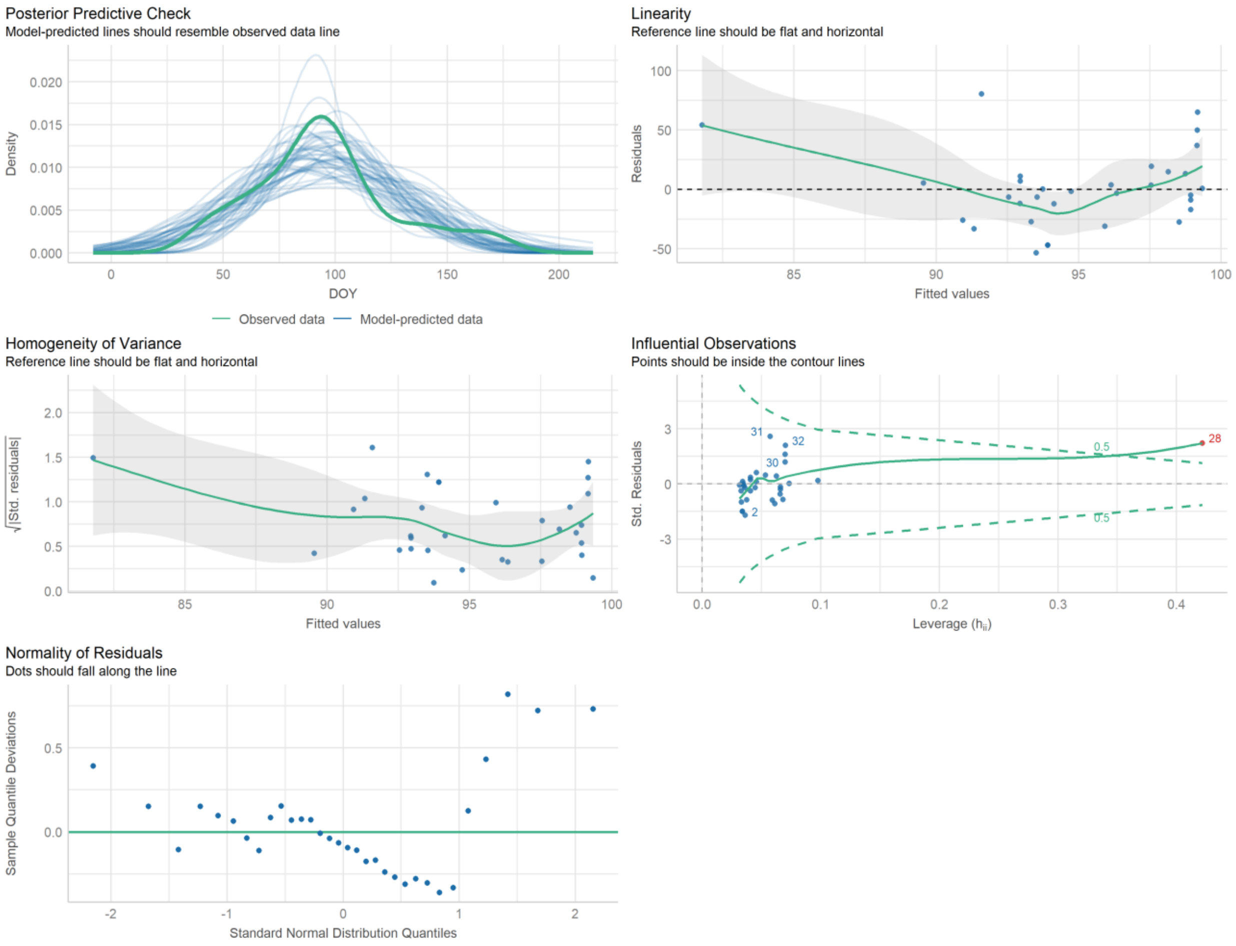

1.130. LM - DVG - Viburnum tinus

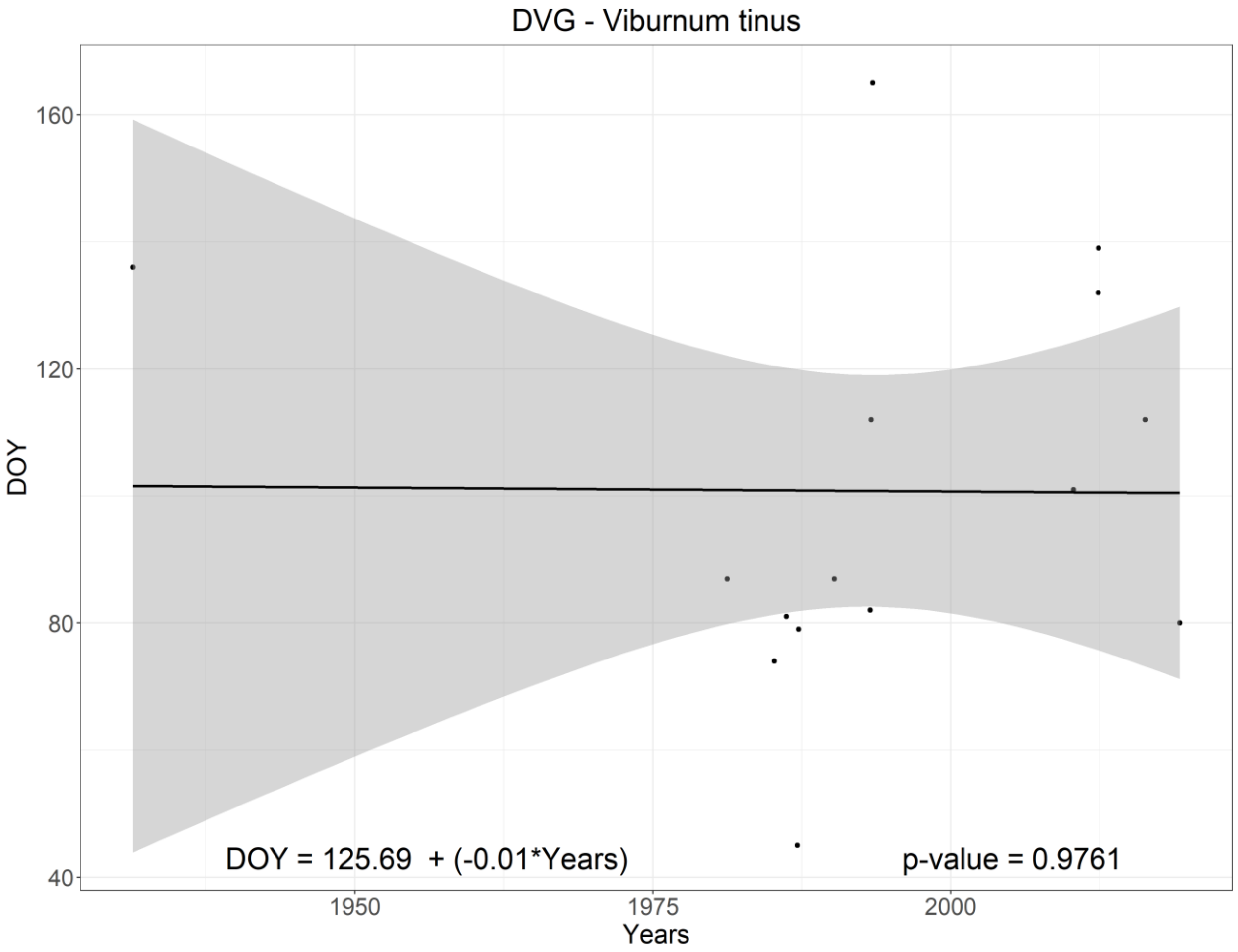

1.130.1. Diagnostics - LM - DVG - Viburnum tinus

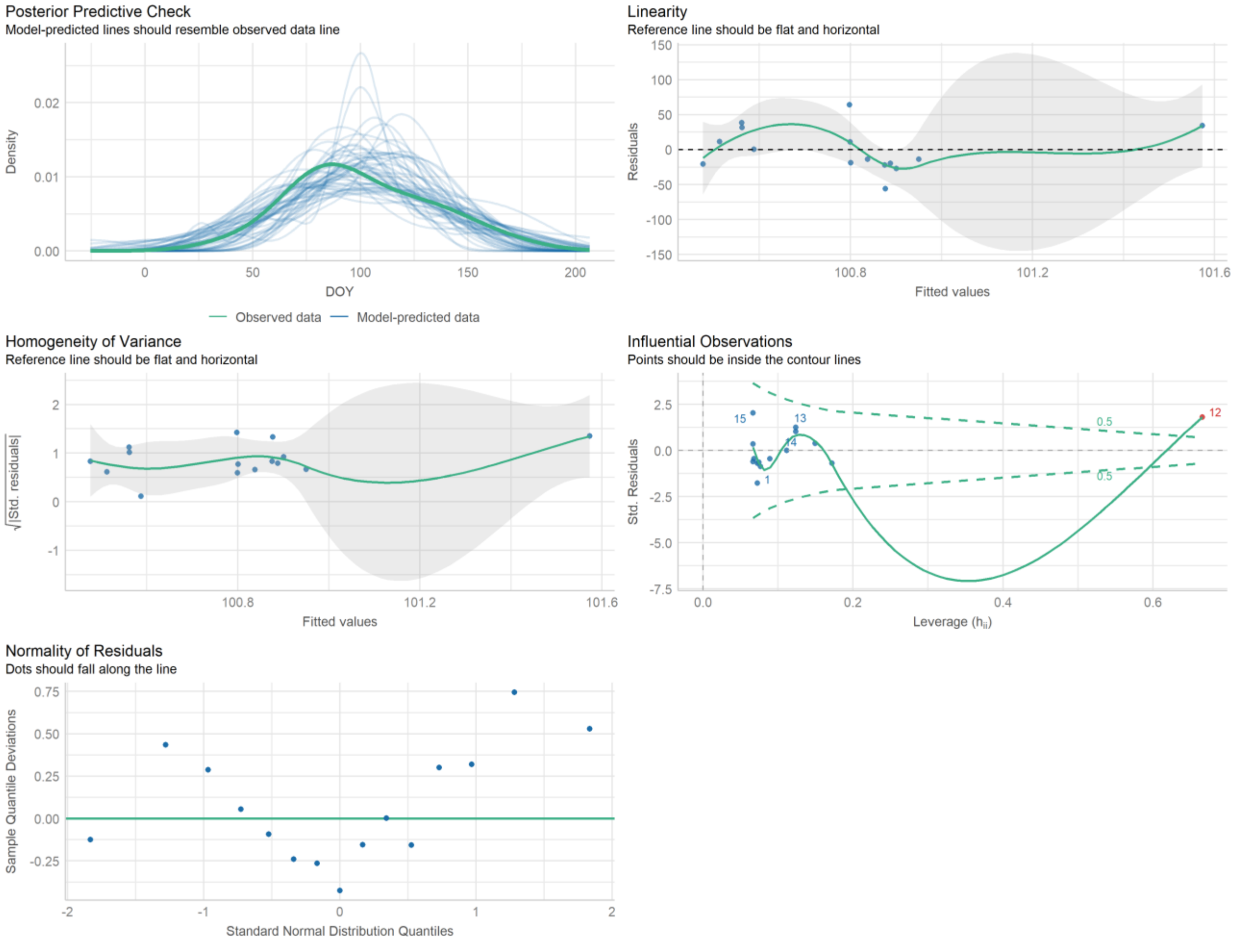

2. GLM Model Results

Table S2. Generalized Linear Model results by taxon and phenophase of DOY over time. Preflowering: FBF, flowering: F, fruiting: FS, growth: DVG. BP test: studentized Breusch-Pagan Test. SW: Saphiro-Wilk test.

Phenophase	Taxon	Intercept	Slope	SE	Ad_R^2	BPTest	p value	Normality_p	TestUsed
F	*Rubia peregrina*	-4.6600402	0.0047906777	0.0037828494	0.0043724141	0.4183786018	0.208953951	0.00415708096	SW
F	*Salvia lavandulifolia*	10.7822658	-0.0028138950	0.0020113893	0.0104176715	0.0007175336	0.151610235	0.74012677477	SW
F	*Sideritis glacialis*	7.1815636	-0.0009356325	0.0003680373	0.0162177379	0.2371861921	0.013186073	0.00000118729	SW
FBF	*Rhamnus alaternus*	65.5480840	-0.0309520243	0.0250650243	0.0155627866	0.6473890370	0.106207185	0.00283556405	SW
FS	*Rhamnus alaternus*	0.7271202	0.0020993663	0.0031107880	0.0014176154	0.0075018153	0.491251474	0.72008859740	SW
DVG	*Juniperus sabina*	3.6694928	0.0007783933	0.0022544561	0.0005944658	0.6205659957	0.708426109	0.00663669264	SW
DVG	*Cotoneaster granatensis*	12.2456362	-0.0035504230	0.0011937816	0.0554562267	0.4359806911	0.003947896	0.01757633574	SW

***In the following section, each taxon with >15 records in a single phenophase, has a figure which represents the generalized linear model trends with time (years), and its corresponding model diagnostic analysis (Diagnostics). FBF: preflowering, F: flowering, FS: fruiting, DVG: growth.**

2.1. GLM - DVG - Cotoneaster granatensis

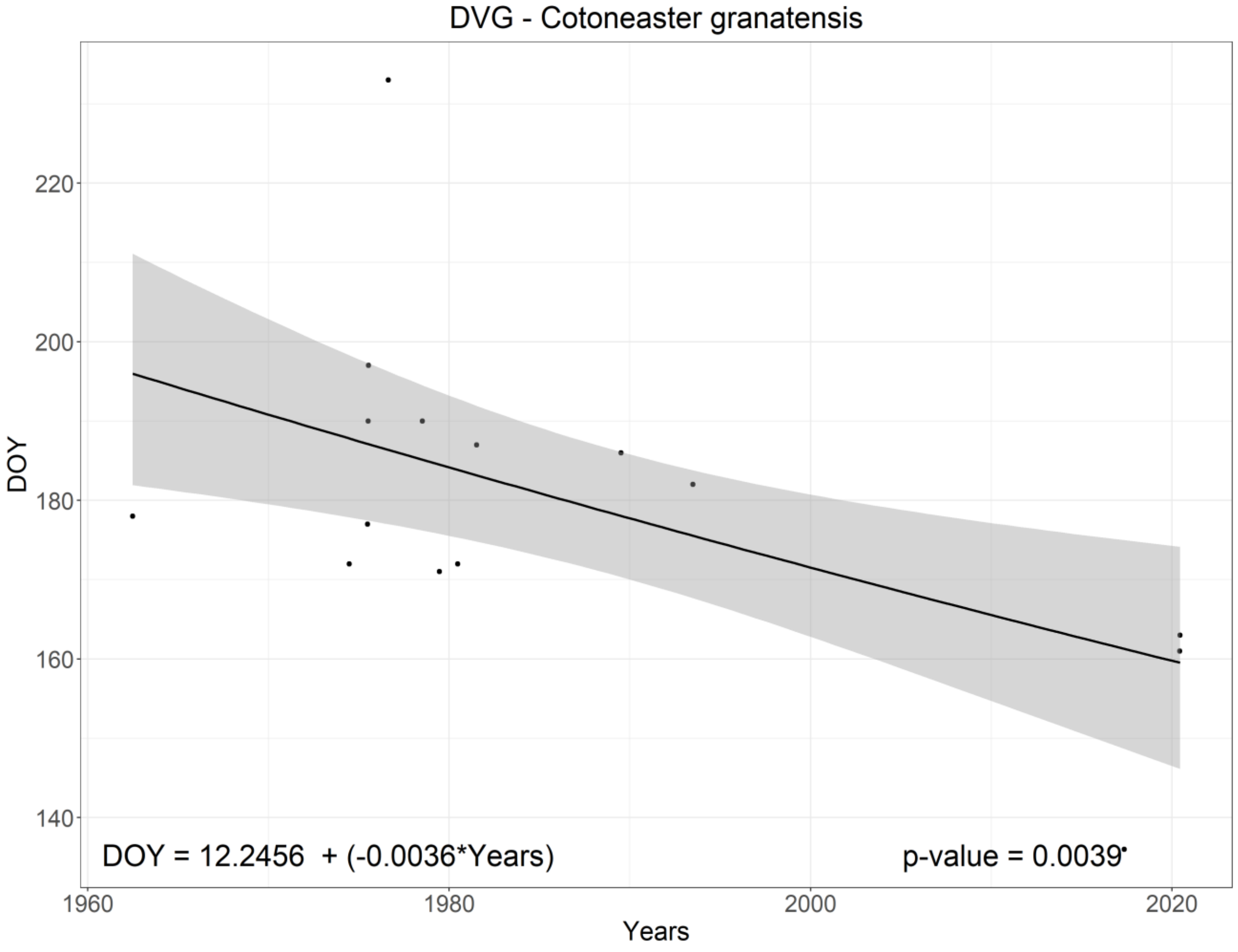

2.1.1. Diagnostics - GLM - DVG - Cotoneaster granatensis

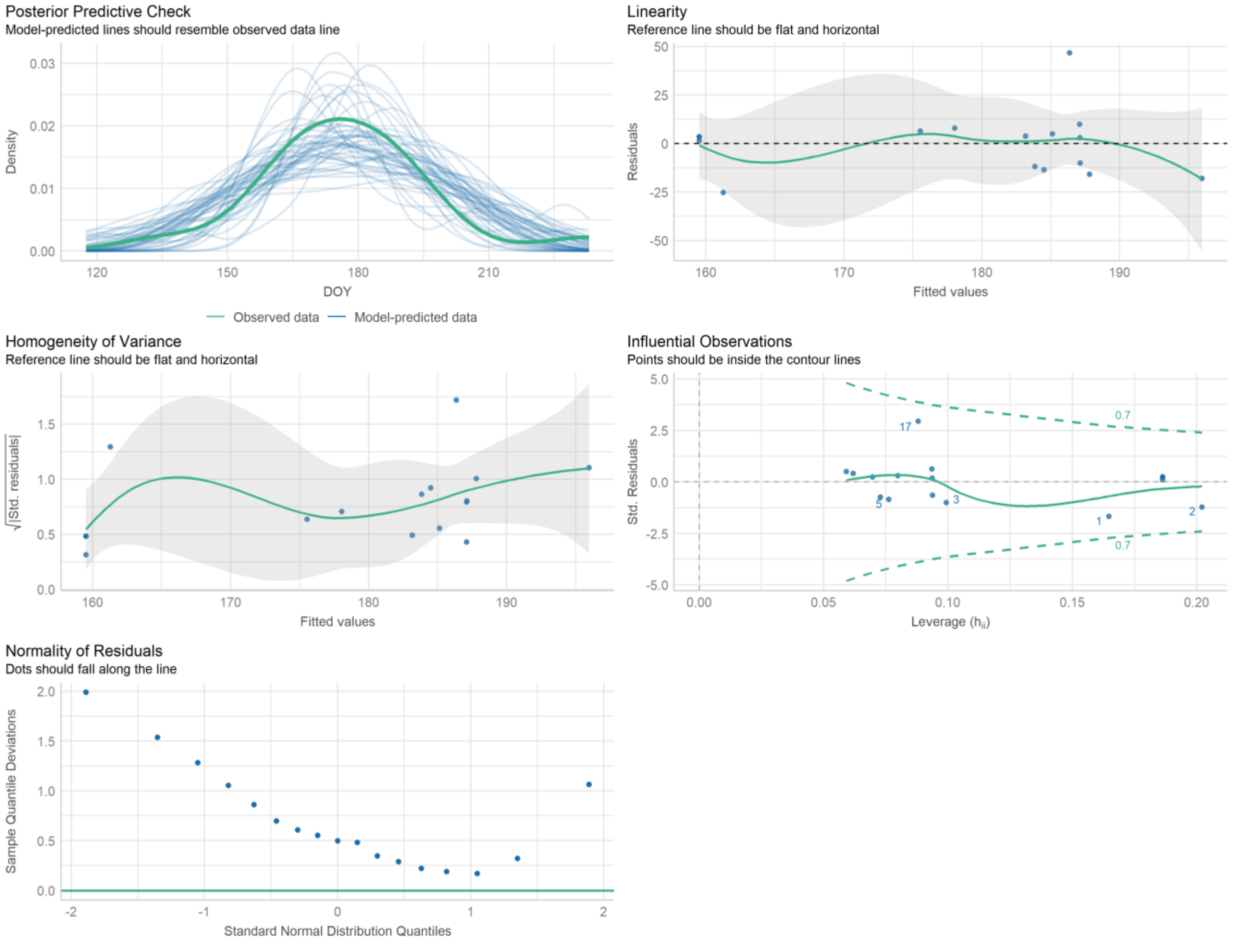

2.2. GLM - DVG - Juniperus sabina

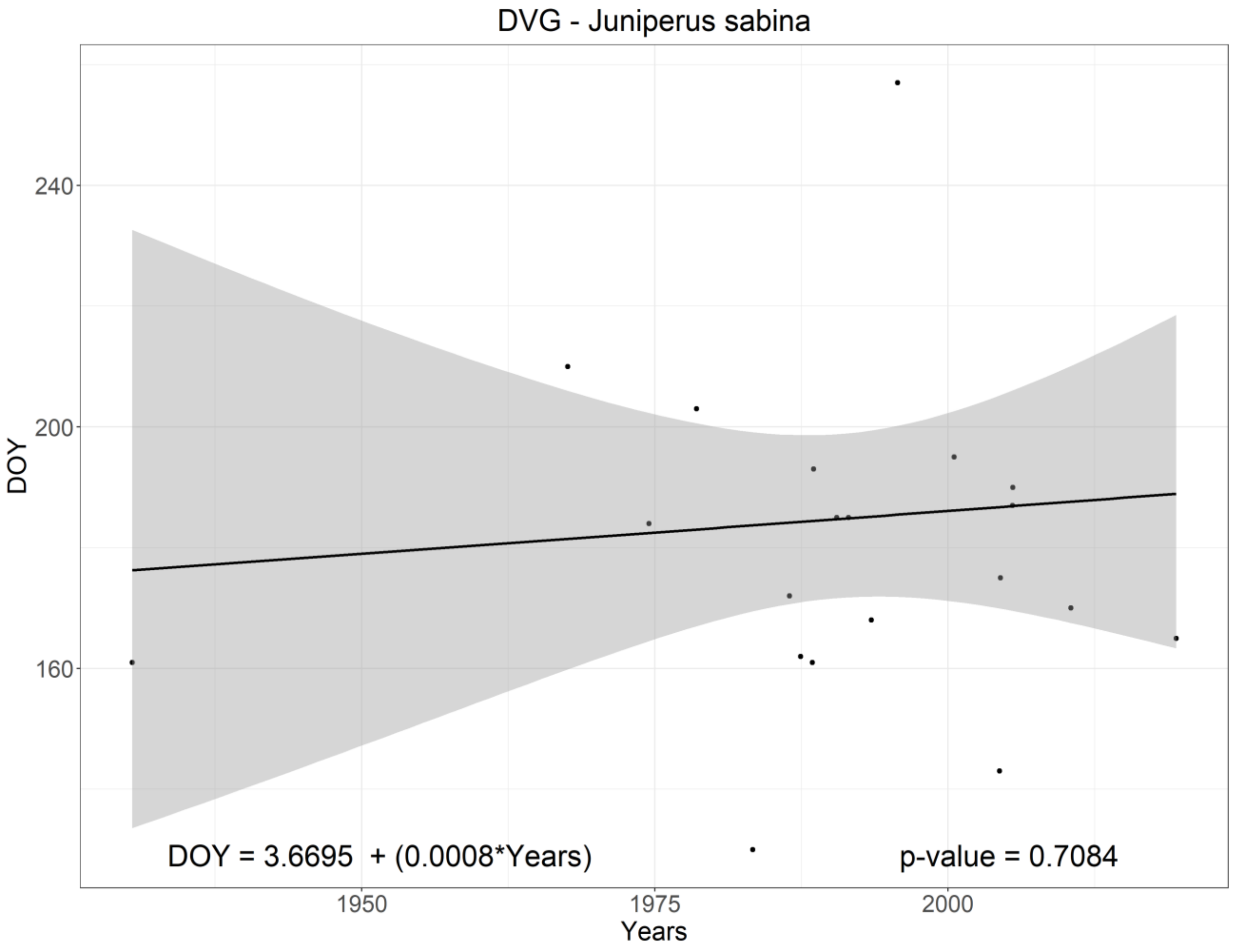

2.2.1. Diagnostics - GLM - DVG - Juniperus sabina

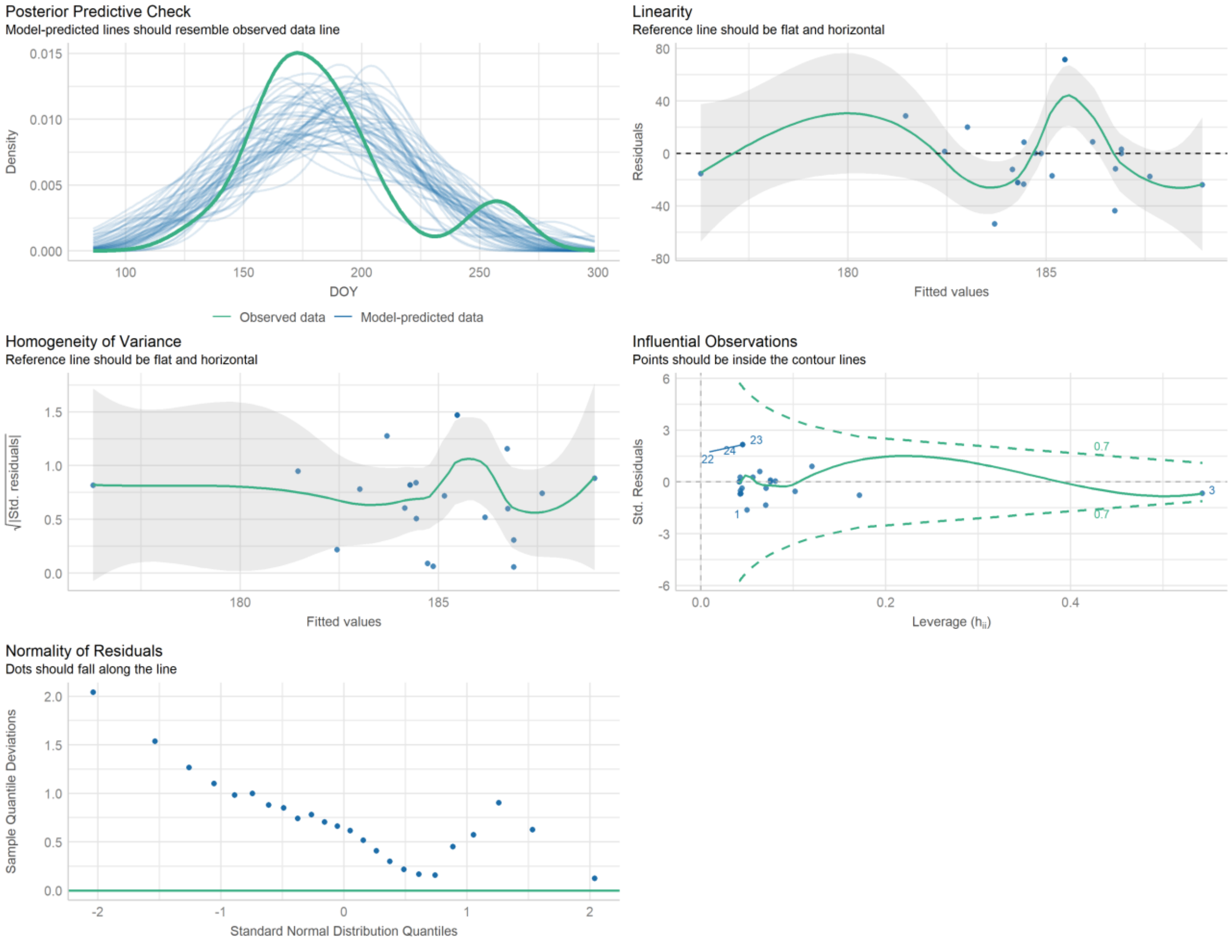

2.3. GLM - FBF - Rhamnus alaternus

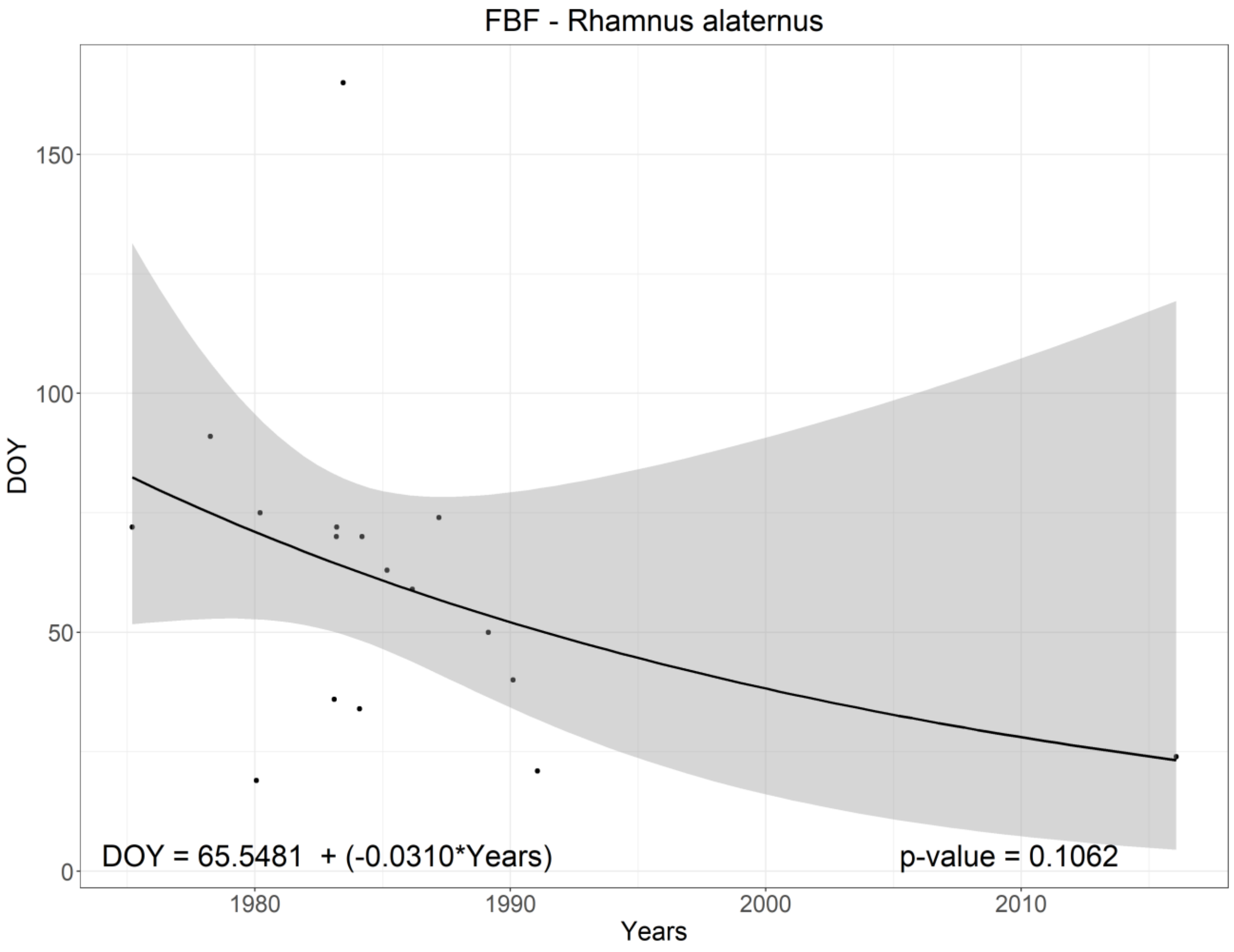

2.3.1. Diagnostics - GLM - FBF - Rhamnus alaternus

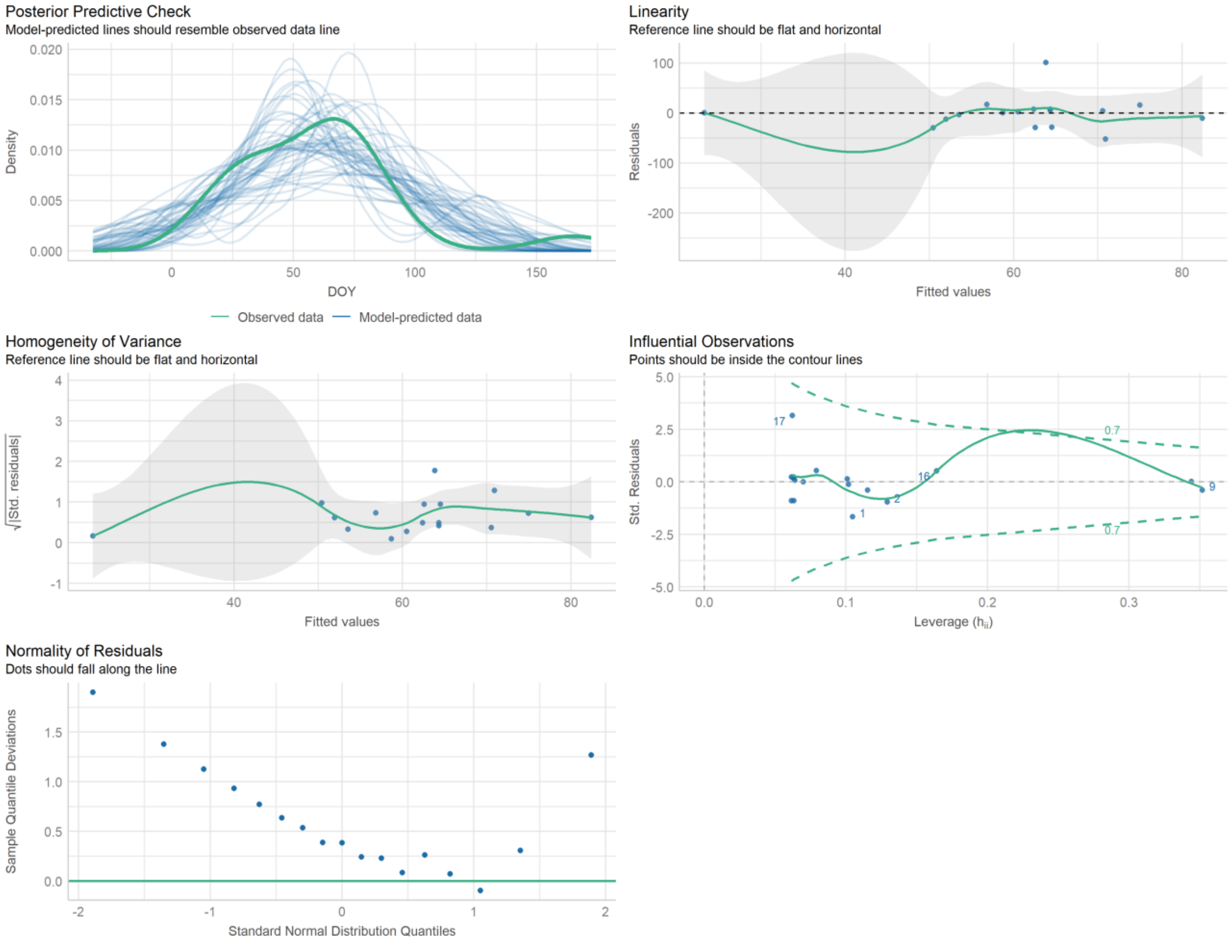

2.4. GLM - FS - Rhamnus alaternus

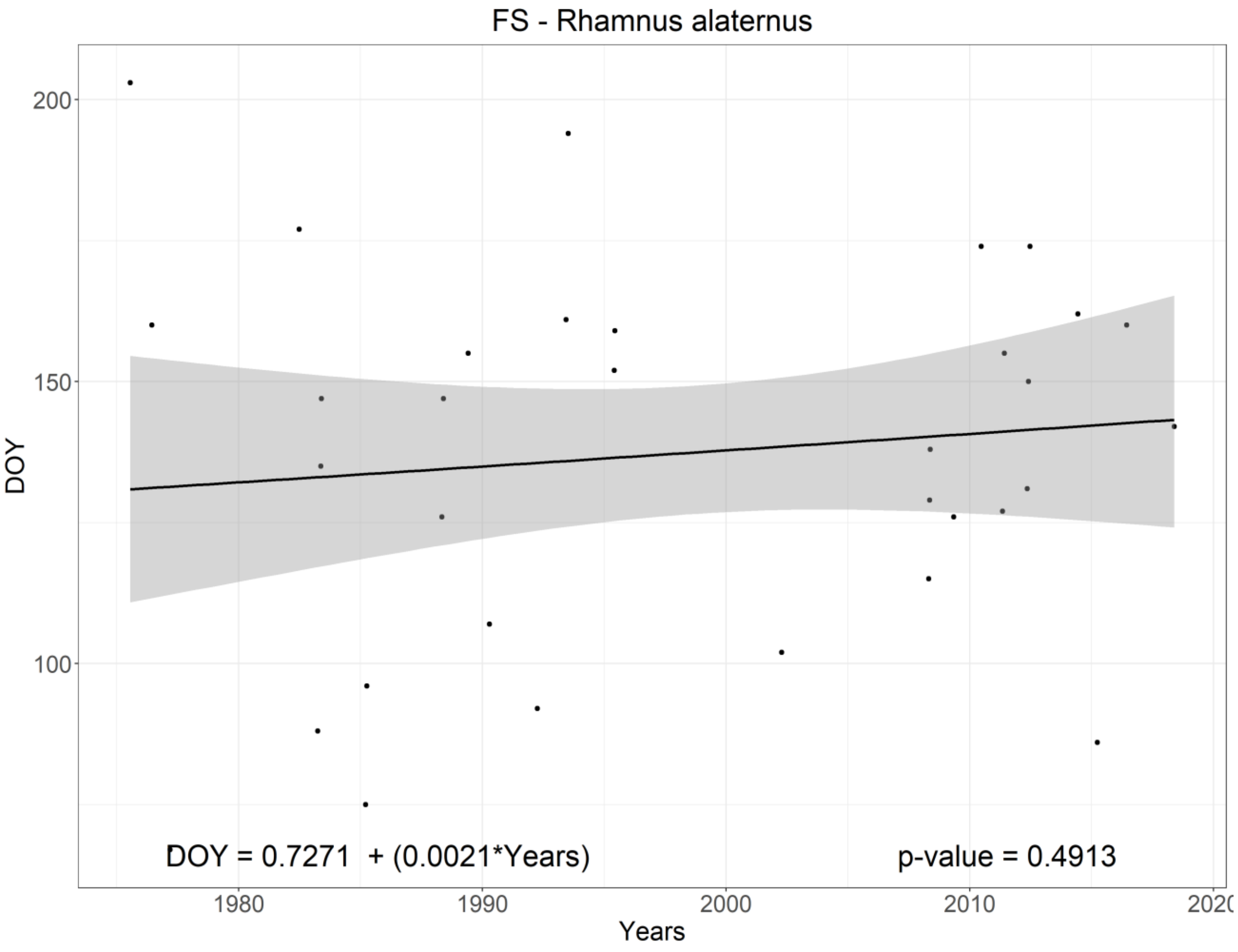

2.4.1. Diagnostics - GLM - FS - Rhamnus alaternus

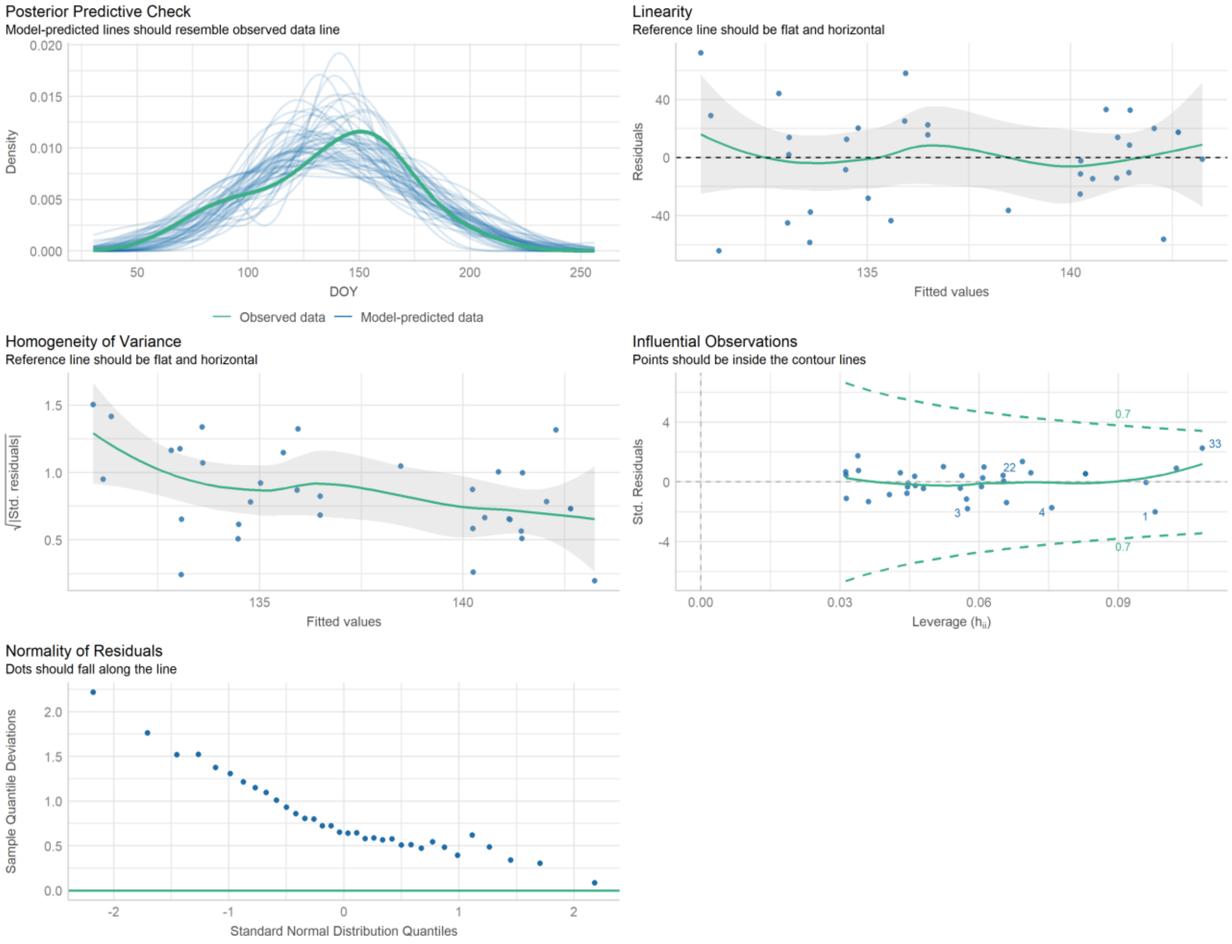

2.5. GLM - F - Rubia peregrina

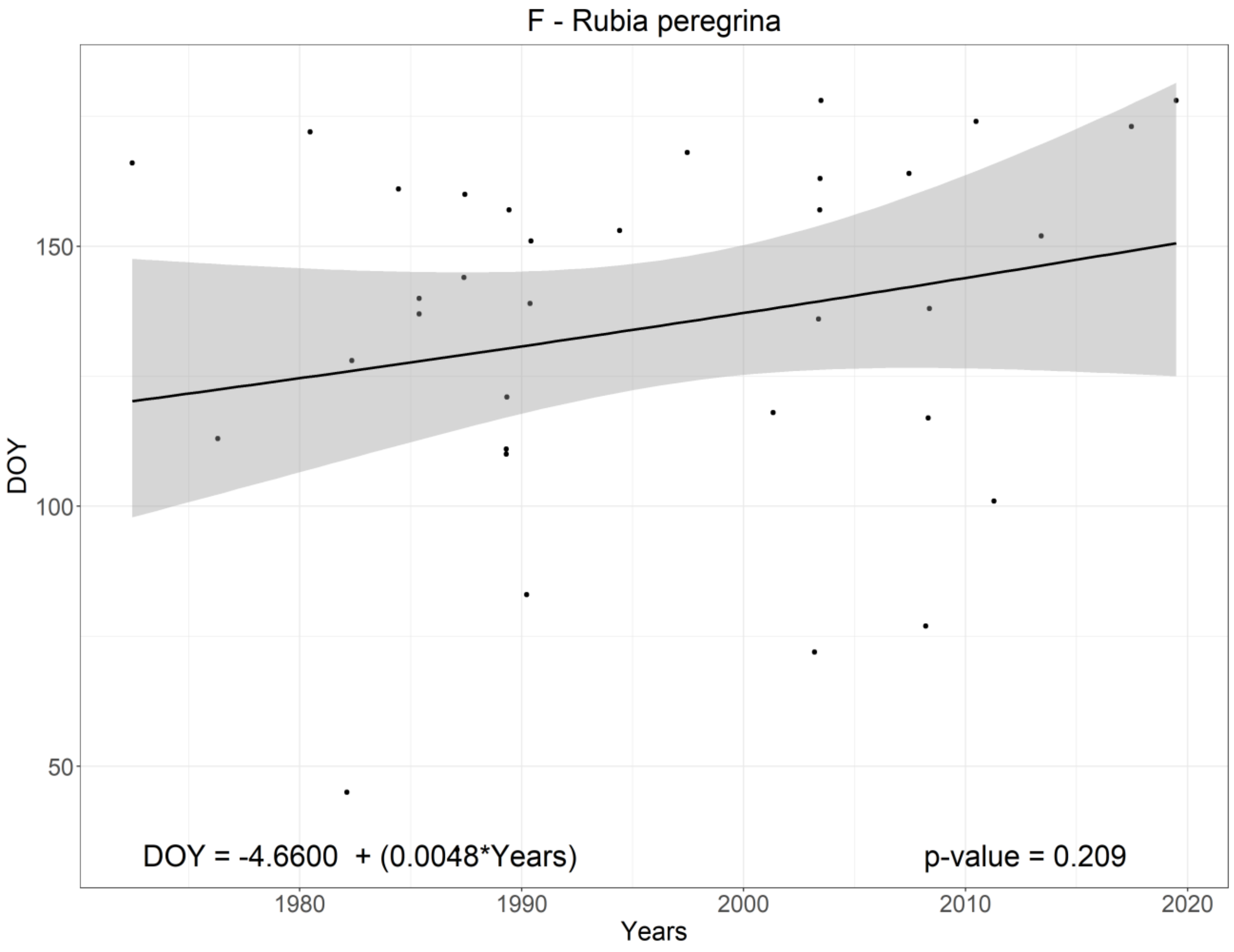

2.5.1. Diagnostics - GLM - F - Rubia peregrina

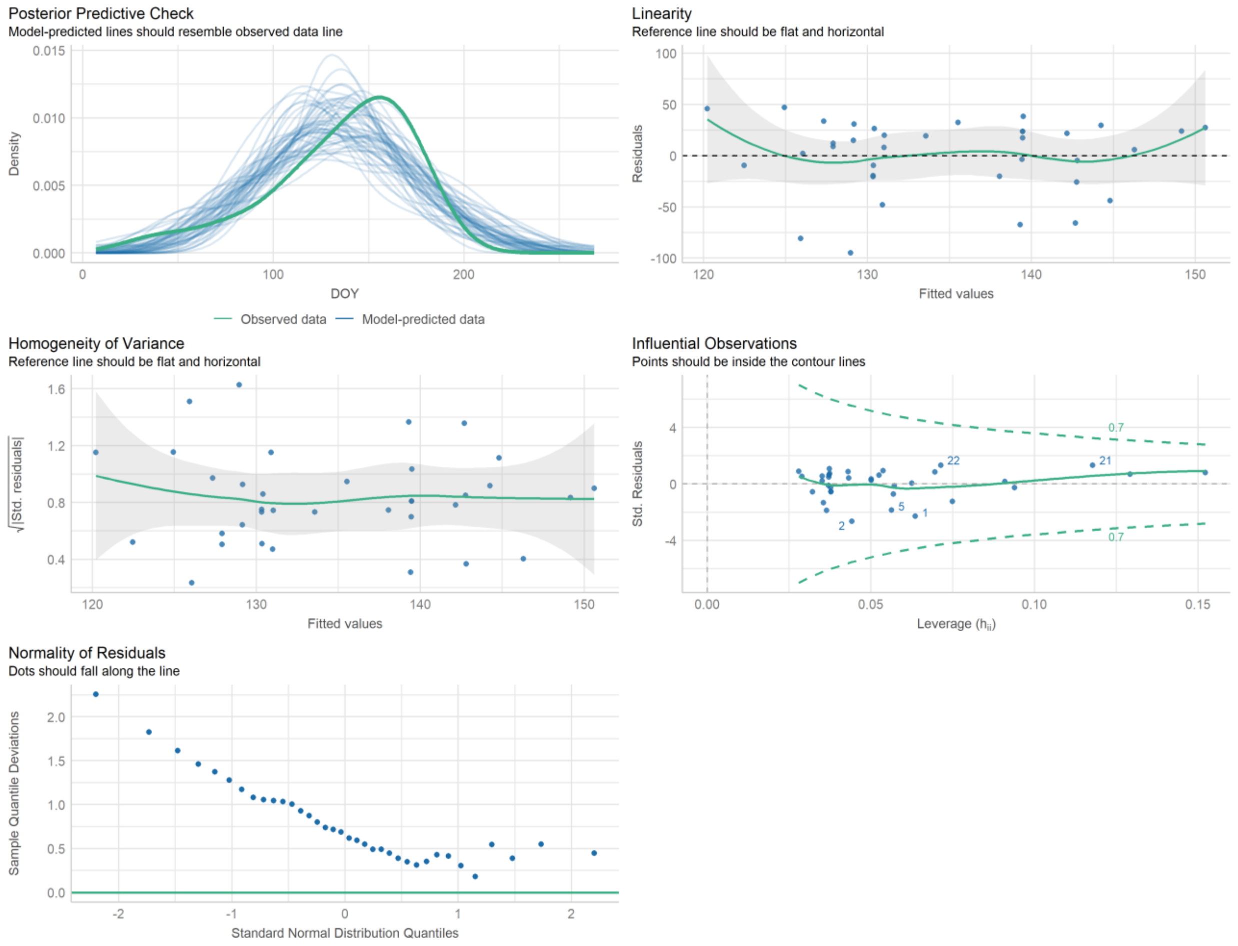

2.6. GLM - F - Salvia lavandulifolia

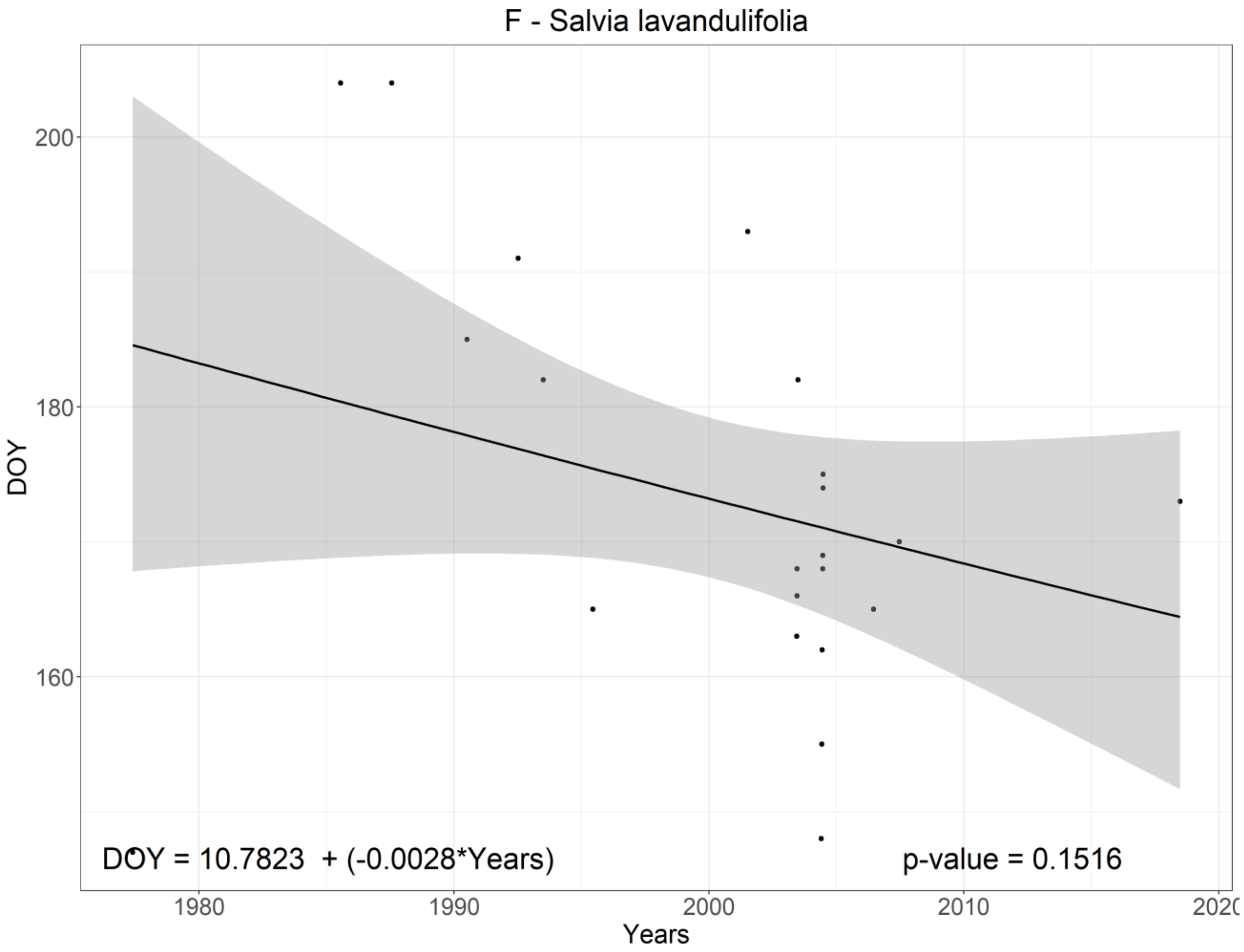

2.6.1. Diagnostics - GLM - F - Salvia lavandulifolia

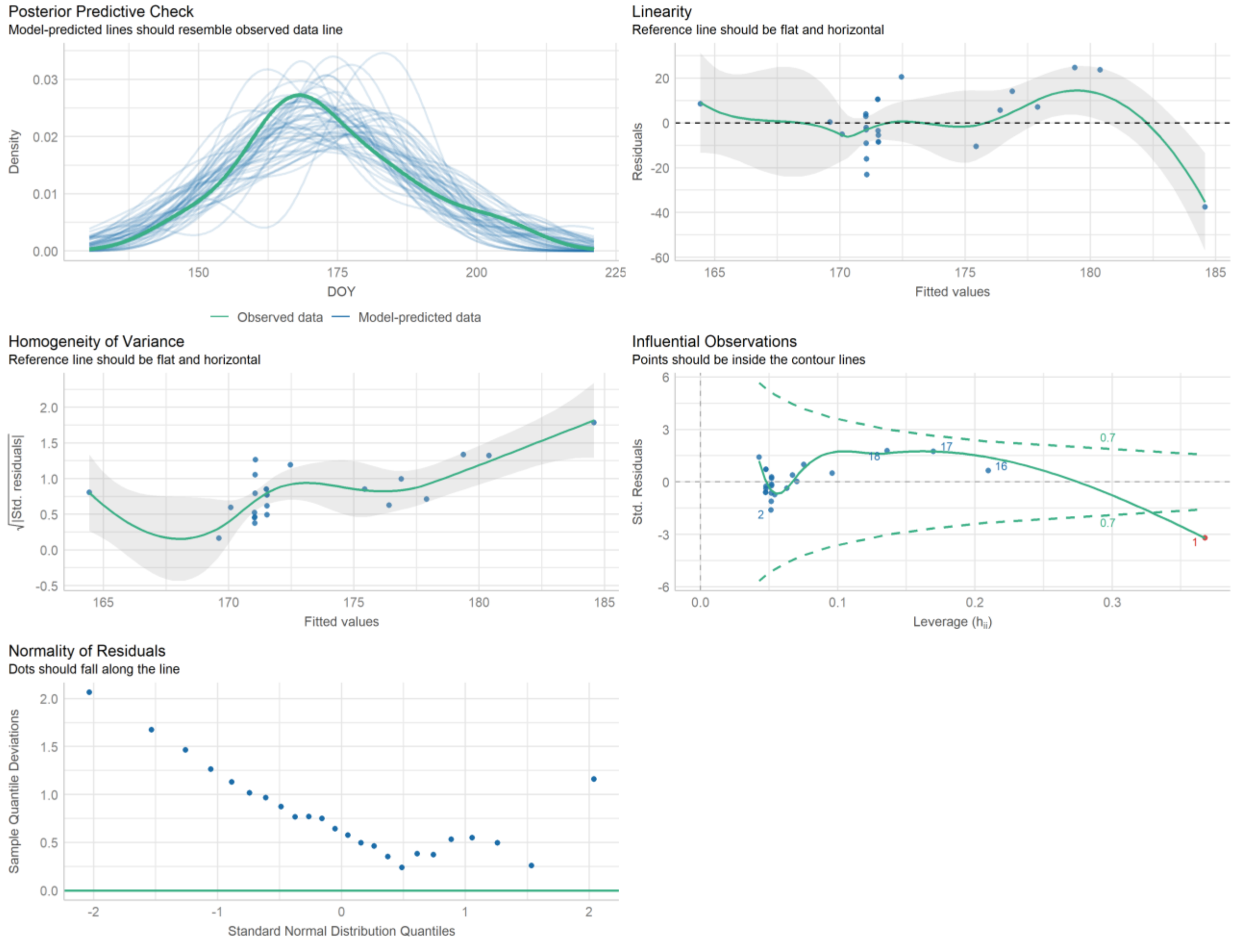

2.7. GLM - F - Sideritis glacialis

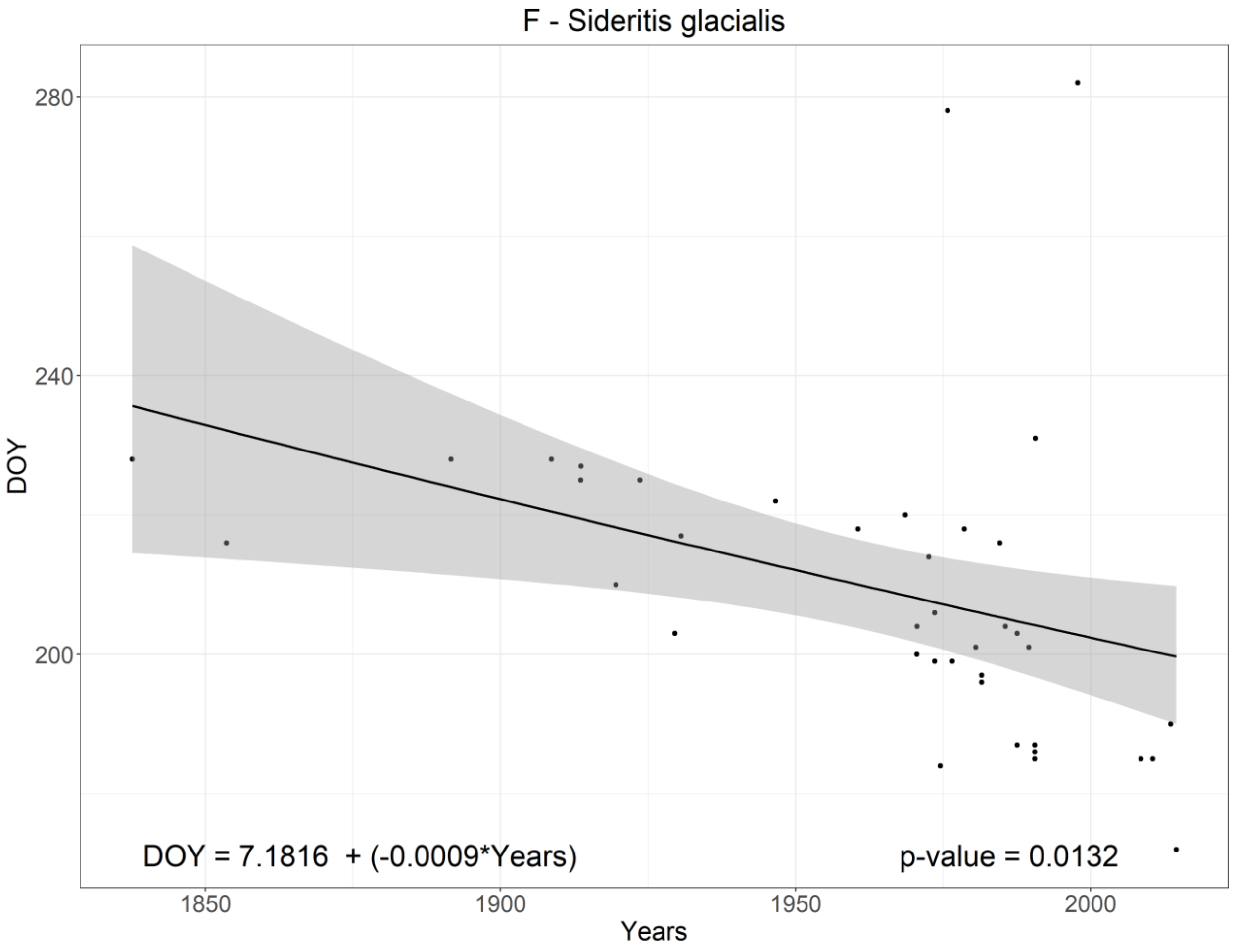

2.7.1. Diagnostics - GLM - F - Sideritis glacialis

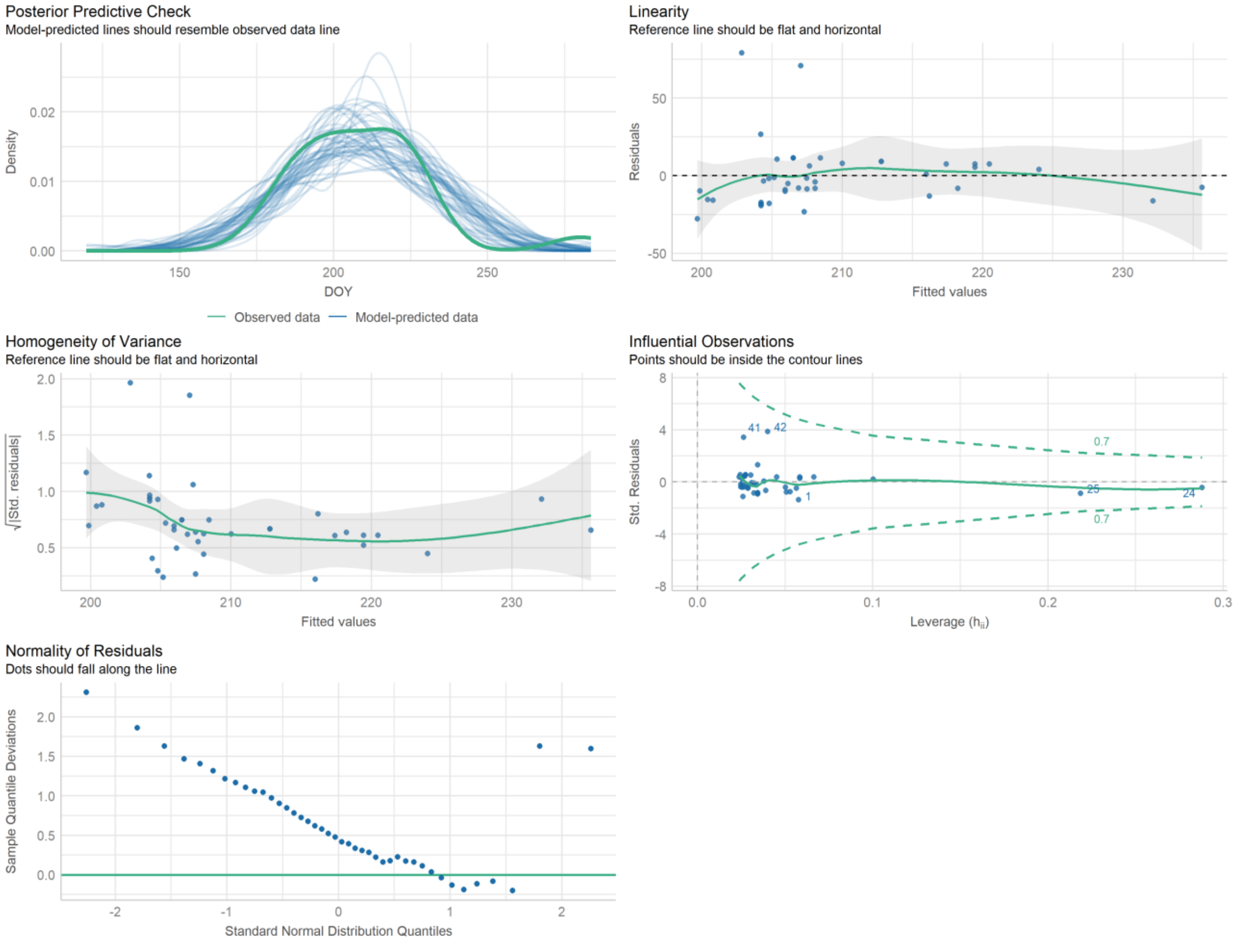

3. AIC Comparison Between GLMs and GAMs

Table S3. Akaike Information Criterion analysis on the generalized additive models (GAM) and generalized linear models (GLM) by taxon and phenophase.

**Phenophase**	**Taxon**	**AIC_GLM**	**AIC_GAM**
F	*Rubia peregrina*	365.4711	365.5485
F	*Salvia lavandulifolia*	201.2985	189.4860
F	*Sideritis glacialis*	378.6988	378.8946
FBF	*Rhamnus alaternus*	171.0832	171.1469
FS	*Rhamnus alaternus*	339.7382	339.7470
DVG	*Juniperus sabina*	241.1203	241.1130
DVG	*Cotoneaster granatensis*	147.4975	147.3390
