# Supplementary material for: Retrospective phenology in western Mediterranean plants: revealing climate change patterns through herbarium specimens
Source: AoB Plants. 2025 Nov 3;17(6):plaf064. doi: 10.1093/aobpla/plaf064 (PMC12611260; doi:10.1093/aobpla/plaf064)

Supporting Information for

Retrospective phenology in western Mediterranean plants: revealing climate change patterns through herbarium specimens

APPENDIX S3 – Climate trends in the Baetic Ranges

Contents

1. LM Summaries

2. Model diagnostics

1. LM Summaries

Table S1. Trends in the linear model results of each climatic variable through time (1911-2020).

Variable	Intercept	Slope (per day)	Std.Error	R.squared	p_value	Slope (per year)	Slope (per decade)	Std.Error (per year)	Std.Error (per decade)
spring P (mm)	187.185311	-0.003760973	0.00018279	0.24311607	8.4776E-82	-1.373695514	-13.73695514	0.06676373	0.667637292
annual P (mm)	638.169385	-0.009032758	0.00039758	0.28142289	1.0773E-96	-3.299214964	-32.99214964	0.14521447	1.452144687
spring T (ºC)	14.2355035	8.38507E-05	3.5832E-06	0.29352332	1.455E-101	0.030626474	0.306264736	0.00130878	0.013087808
annual T (ºC)	16.0645827	7.42235E-05	2.797E-06	0.34824211	1.13E-124	0.027110116	0.271101163	0.00102159	0.010215889

2. Model Diagnostics

2.1. Diagnostics for mean annual T

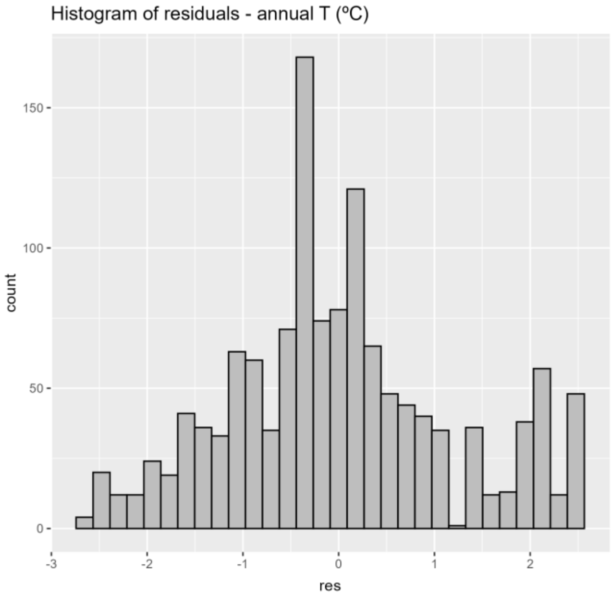

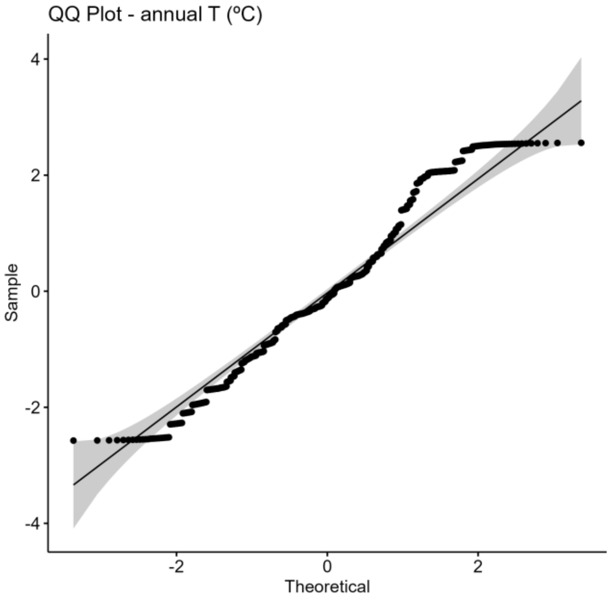

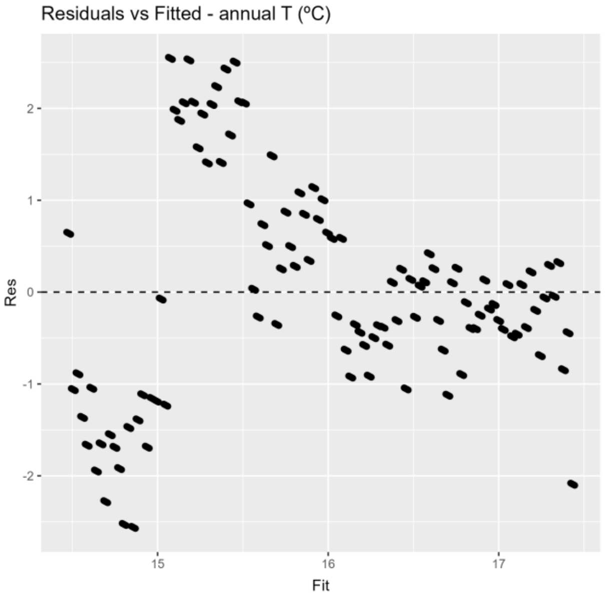

2.2. Diagnostics for spring T

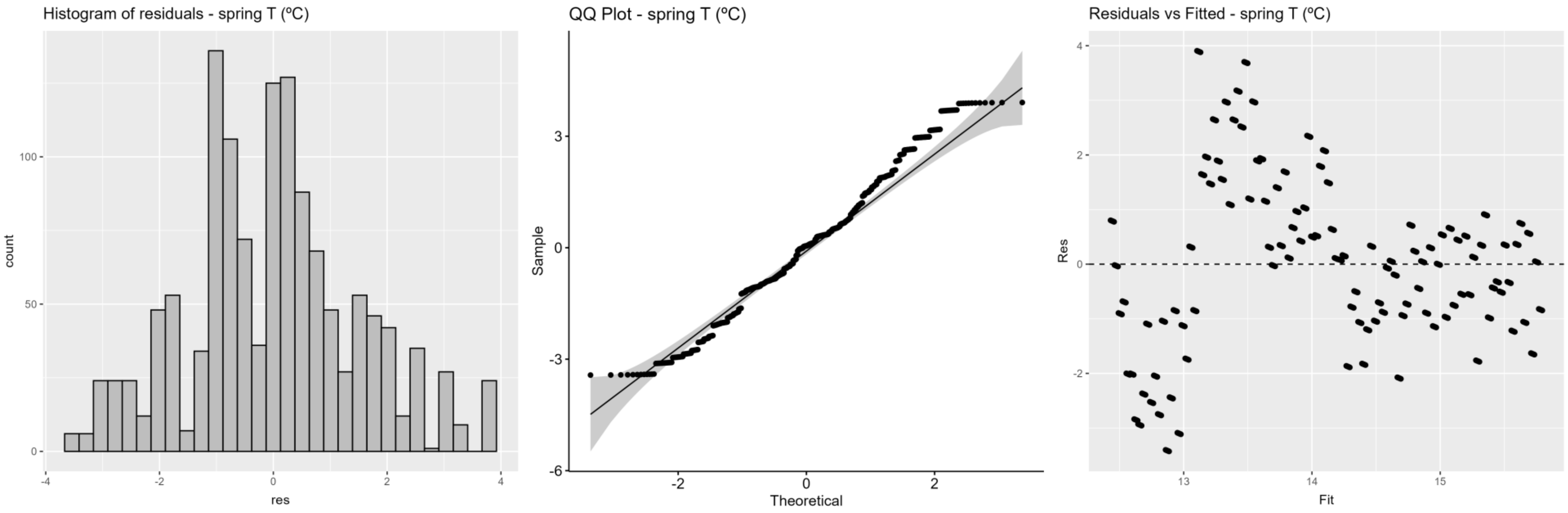

2.3. Diagnostics for annual P

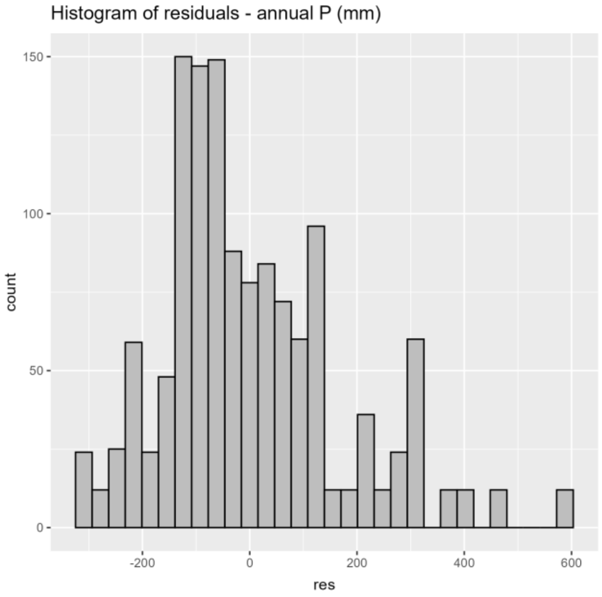

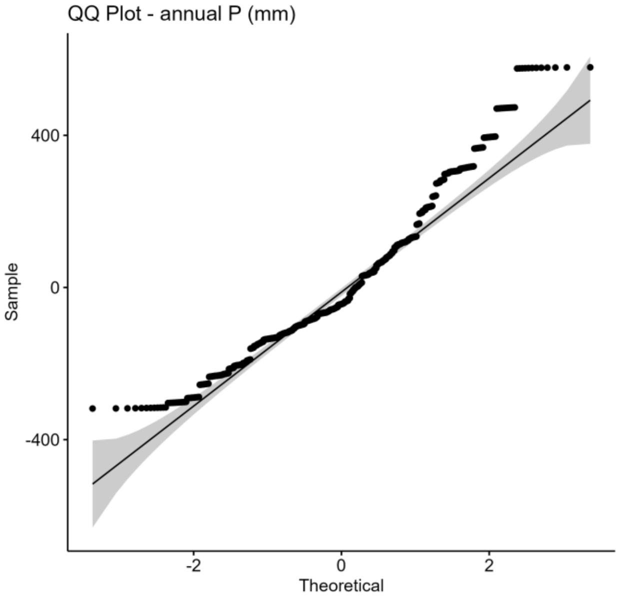

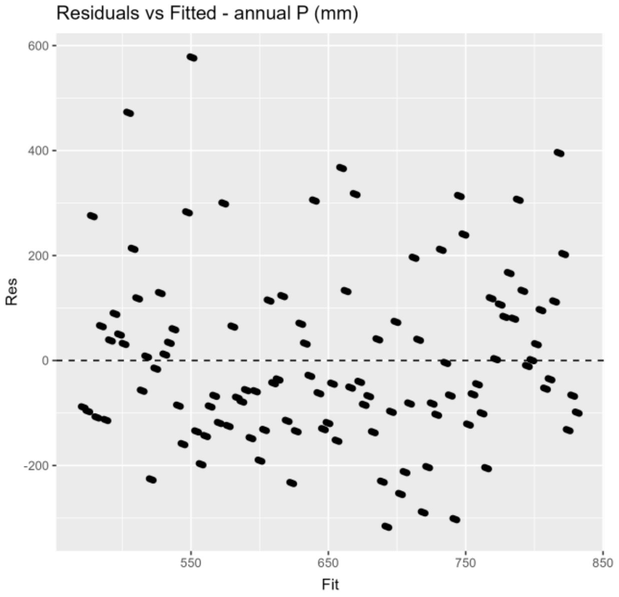

2.4. Diagnostics for spring P

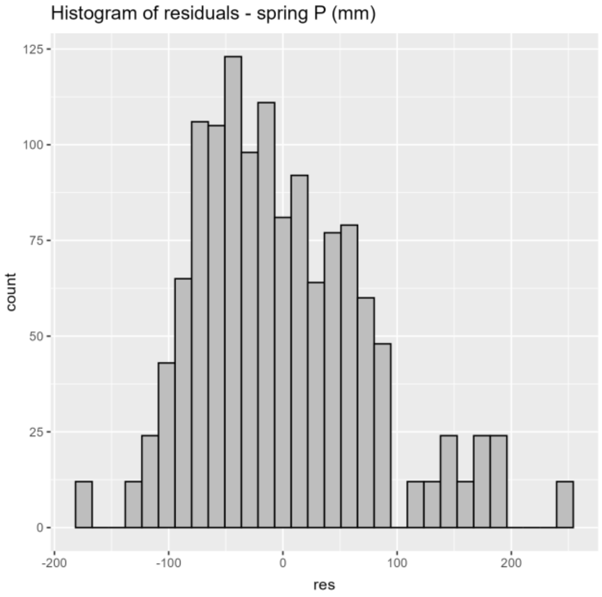

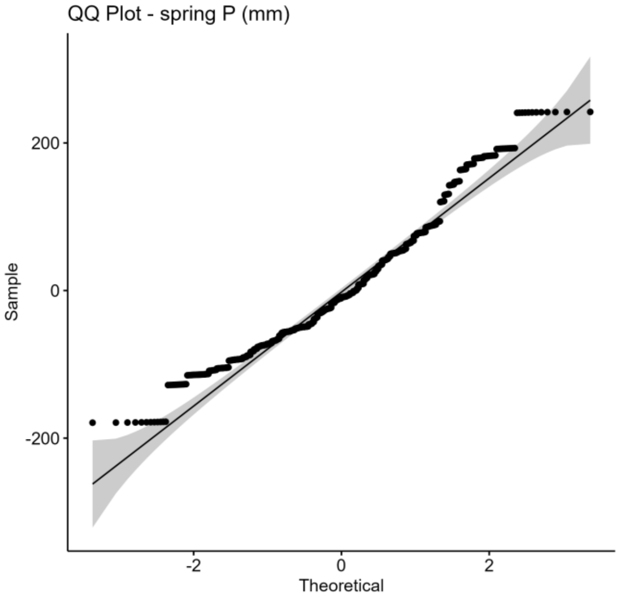

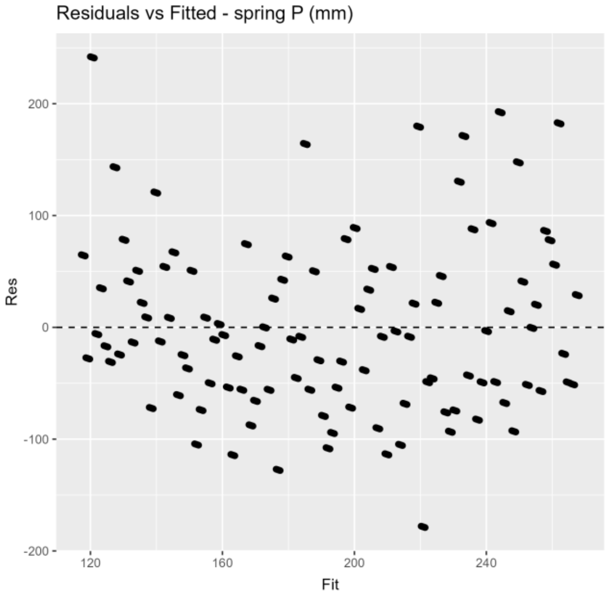

Supplement: plaf064_Supplementary_Data [file plaf064_supplementary_data.zip › Appendix_S3.pdf]
