# Supplementary material for: Retrospective phenology in western Mediterranean plants: revealing climate change patterns through herbarium specimens
Source: AoB Plants. 2025 Nov 3;17(6):plaf064. doi: 10.1093/aobpla/plaf064 (PMC12611260; doi:10.1093/aobpla/plaf064)

Supporting Information for

Retrospective phenology in western Mediterranean plants: revealing climate change patterns through herbarium specimens

APPENDIX S4 – DOY with climate variables – Thermotypes

Contents

1. LMM results – Type II ANOVA

2. LMM estimated slopes

3. Model diagnostics

1. LMM results – Type II ANOVA

Table S1. Type II ANOVA results for fixed effects: climatic variable, thermotypes (BioclimGroup) and their interaction by phenophase. FBF: preflowering, F: flowering, FS: fruiting, DVG: growth. Annual mean temperature: Tmed.Y, total annual precipitation: Pannual_Y, spring temperature: T.MAM, spring precipitation: P.MAM.

Phenophase	Variable	Effect	Sum Sq	Mean Sq	df1	df2	F	p_value
FBF	annual T (ºC)	Tmed.Y	119.712	119.712	1.000	1292.398	0.121	0.728
FBF	annual T (ºC)	BioclimGroup	246.321	123.160	2.000	1307.023	0.124	0.883
FBF	annual T (ºC)	Tmed.Y:BioclimGroup	109.880	54.940	2.000	1295.689	0.055	0.946
FBF	annual P (mm)	Pannual.Y	23.122	23.122	1.000	1296.770	0.023	0.878
FBF	annual P (mm)	BioclimGroup	23859.444	11929.722	2.000	1332.351	12.094	0.000
FBF	annual P (mm)	Pannual.Y:BioclimGroup	5777.554	2888.777	2.000	1319.968	2.929	0.054
FBF	spring T (ºC)	T.MAM	3719.267	3719.267	1.000	1286.442	3.767	0.052
FBF	spring T (ºC)	BioclimGroup	1723.381	861.691	2.000	1312.801	0.873	0.418
FBF	spring T (ºC)	T.MAM:BioclimGroup	242.598	121.299	2.000	1286.195	0.123	0.884
FBF	spring P (mm)	P.MAM	5496.544	5496.544	1.000	1286.174	5.572	0.018
FBF	spring P (mm)	BioclimGroup	21102.775	10551.387	2.000	1329.679	10.696	0.000
FBF	spring P (mm)	P.MAM:BioclimGroup	397.716	198.858	2.000	1303.457	0.202	0.817
F	annual T (ºC)	Tmed.Y	31786.984	31786.984	1.000	2648.364	44.106	0.000

F	annual T (ºC)	BioclimGroup	398.391	199.196	2.000	2667.572	0.276	0.759
F	annual T (ºC)	Tmed.Y:BioclimGroup	260.748	130.374	2.000	2653.386	0.181	0.835
F	annual P (mm)	Pannual.Y	3867.280	3867.280	1.000	2669.342	5.175	0.023
F	annual P (mm)	BioclimGroup	24324.467	12162.233	2.000	2713.036	16.274	0.000
F	annual P (mm)	Pannual.Y:BioclimGroup	7.035	3.517	2.000	2668.056	0.005	0.995
F	spring T (ºC)	T.MAM	28171.445	28171.445	1.000	2645.332	39.028	0.000
F	spring T (ºC)	BioclimGroup	1776.263	888.132	2.000	2685.857	1.230	0.292
F	spring T (ºC)	T.MAM:BioclimGroup	664.974	332.487	2.000	2646.840	0.461	0.631
F	spring P (mm)	P.MAM	18081.030	18081.030	1.000	2660.950	24.386	0.000
F	spring P (mm)	BioclimGroup	36132.224	18066.112	2.000	2724.879	24.366	0.000
F	spring P (mm)	P.MAM:BioclimGroup	1237.898	618.949	2.000	2668.944	0.835	0.434
FS	annual T (ºC)	Tmed.Y	5075.685	5075.685	1.000	624.256	4.474	0.035
FS	annual T (ºC)	BioclimGroup	2743.218	1371.609	2.000	613.204	1.209	0.299
FS	annual T (ºC)	Tmed.Y:BioclimGroup	2848.592	1424.296	2.000	607.393	1.255	0.286
FS	annual P (mm)	Pannual.Y	226.433	226.433	1.000	613.101	0.189	0.664
FS	annual P (mm)	BioclimGroup	2356.368	1178.184	2.000	631.126	0.982	0.375
FS	annual P (mm)	Pannual.Y:BioclimGroup	6272.180	3136.090	2.000	603.937	2.615	0.074
FS	spring T (ºC)	T.MAM	4823.614	4823.614	1.000	623.309	4.262	0.039
FS	spring T (ºC)	BioclimGroup	6551.669	3275.835	2.000	611.471	2.894	0.056
FS	spring T (ºC)	T.MAM:BioclimGroup	4859.825	2429.912	2.000	599.834	2.147	0.118
FS	spring P (mm)	P.MAM	1578.104	1578.104	1.000	626.584	1.316	0.252
FS	spring P (mm)	BioclimGroup	6819.076	3409.538	2.000	643.687	2.843	0.059
FS	spring P (mm)	P.MAM:BioclimGroup	4450.368	2225.184	2.000	613.175	1.855	0.157
DVG	annual T (ºC)	Tmed.Y	4203.213	4203.213	1.000	1625.469	3.762	0.053
DVG	annual T (ºC)	BioclimGroup	4267.001	2133.500	2.000	1622.073	1.909	0.149
DVG	annual T (ºC)	Tmed.Y:BioclimGroup	2554.177	1277.089	2.000	1623.891	1.143	0.319
DVG	annual P (mm)	Pannual.Y	2166.042	2166.042	1.000	1622.005	1.937	0.164

DVG	annual P (mm)	BioclimGroup	25563.667	12781.833	2.000	1648.403	11.428	0.000
DVG	annual P (mm)	Pannual.Y:BioclimGroup	40.823	20.411	2.000	1630.066	0.018	0.982
DVG	spring T (ºC)	T.MAM	226.459	226.459	1.000	1625.866	0.202	0.653
DVG	spring T (ºC)	BioclimGroup	16153.192	8076.596	2.000	1610.492	7.219	0.001
DVG	spring T (ºC)	T.MAM:BioclimGroup	5039.936	2519.968	2.000	1625.195	2.252	0.105
DVG	spring P (mm)	P.MAM	91.946	91.946	1.000	1613.845	0.082	0.775
DVG	spring P (mm)	BioclimGroup	49750.700	24875.350	2.000	1650.036	22.211	0.000
DVG	spring P (mm)	P.MAM:BioclimGroup	178.048	89.024	2.000	1622.678	0.079	0.924

2. LMM estimated slopes

Table S2 Global slopes of DOY trends over climatic variables for each phenophase (LMM estimates). FBF: preflowering, F: flowering, FS: fruiting, DVG: growth. Annual mean temperature: Tmed.Y, total annual precipitation: Pannual_Y, spring temperature: T.MAM, spring precipitation: P.MAM.

Phenophase	Variable	Global_Slope	SE	df	lower.CL	upper.CL	t.ratio	p_value	direction
FBF	annual T (ºC)	-0.541	1.450	1288.312	-3.386	2.304	-0.373	0.709	Advance
FBF	annual P (mm)	0.001	0.006	1298.531	-0.011	0.013	0.172	0.864	Delay
FBF	spring T (ºC)	-1.528	0.893	1280.871	-3.281	0.224	-1.711	0.087	Advance
FBF	spring P (mm)	0.033	0.014	1281.067	0.006	0.060	2.426	0.015	Delay
F	annual T (ºC)	-5.144	0.794	2645.636	-6.702	-3.587	-6.479	0.000	Advance
F	annual P (mm)	0.007	0.003	2677.162	0.001	0.014	2.252	0.024	Delay
F	spring T (ºC)	-2.862	0.500	2644.651	-3.841	-1.882	-5.728	0.000	Advance
F	spring P (mm)	0.034	0.008	2667.339	0.019	0.049	4.379	0.000	Delay
FS	annual T (ºC)	-2.379	2.047	625.156	-6.399	1.642	-1.162	0.246	Advance

FS	annual P (mm)	-0.001	0.008	618.669	-0.017	0.015	-0.091	0.928	Advance
FS	spring T (ºC)	-1.358	1.308	617.421	-3.927	1.210	-1.039	0.299	Advance
FS	spring P (mm)	0.010	0.020	623.025	-0.030	0.050	0.487	0.626	Delay
DVG	annual T (ºC)	-2.018	1.350	1617.396	-4.665	0.630	-1.495	0.135	Advance
DVG	annual P (mm)	0.007	0.005	1627.511	-0.003	0.018	1.400	0.162	Delay
DVG	spring T (ºC)	0.156	0.841	1614.751	-1.494	1.807	0.186	0.853	Delay
DVG	spring P (mm)	0.004	0.013	1611.569	-0.022	0.029	0.277	0.782	Delay

Table S3. Estimated slopes of DOY trends by thermotype (BioclimGroup)and phenophase from emtrends results. TM: thermo-Mediterranean, MS: meso-supra-Mediterranean, OC: oro-cryoro-Mediterranean. FBF: preflowering, F: flowering, FS: fruiting, DVG: growth. Annual mean temperature: Tmed.Y, total annual precipitation: Pannual_Y, spring temperature: T.MAM, spring precipitation: P.MAM.

Phenophase	Variable	Bioclim Group	Slope	SE	df	lower .CL	upper .CL	t.ratio	p_value	direction
FBF	annual T (ºC)	TM	-0.484	2.118	1277.512	-4.638	3.671	-0.228	0.819	Advance
FBF	annual T (ºC)	MS	-0.720	1.955	1299.550	-4.554	3.115	-0.368	0.713	Advance
FBF	annual T (ºC)	OC	0.997	4.756	1313.391	-8.333	10.326	0.210	0.834	Delay
FBF	annual P (mm)	TM	0.011	0.008	1278.799	-0.005	0.026	1.298	0.194	Delay
FBF	annual P (mm)	MS	-0.006	0.009	1310.441	-0.023	0.011	-0.704	0.482	Advance
FBF	annual P (mm)	OC	-0.083	0.043	1351.816	-0.167	0.001	-1.928	0.054	Advance
FBF	spring T (ºC)	TM	-1.196	1.304	1267.291	-3.754	1.363	-0.917	0.359	Advance
FBF	spring T (ºC)	MS	-1.916	1.209	1298.577	-4.288	0.457	-1.584	0.113	Advance
FBF	spring T (ºC)	OC	-2.478	3.068	1298.547	-8.497	3.541	-0.808	0.419	Advance

FBF	spring P (mm)	TM	0.039	0.020	1267.982	0.000	0.078	1.951	0.051	Delay
FBF	spring P (mm)	MS	0.028	0.019	1298.408	-0.008	0.064	1.510	0.131	Delay
FBF	spring P (mm)	OC	-0.003	0.070	1332.422	-0.141	0.136	-0.036	0.972	Advance
F	annual T (ºC)	TM	-5.374	1.273	2634.989	-7.870	-2.878	-4.222	0.000	Advance
F	annual T (ºC)	MS	-5.045	1.019	2656.964	-7.042	-3.048	-4.953	0.000	Advance
F	annual T (ºC)	OC	-3.772	2.342	2676.792	-8.364	0.820	-1.611	0.107	Advance
F	annual P (mm)	TM	0.007	0.005	2674.939	-0.002	0.017	1.473	0.141	Delay
F	annual P (mm)	MS	0.008	0.005	2674.124	-0.001	0.017	1.666	0.096	Delay
F	annual P (mm)	OC	0.009	0.016	2679.899	-0.023	0.041	0.533	0.594	Delay
F	spring T (ºC)	TM	-2.401	0.801	2635.447	-3.973	-0.830	-2.996	0.003	Advance
F	spring T (ºC)	MS	-3.374	0.640	2653.632	-4.630	-2.119	-5.270	0.000	Advance
F	spring T (ºC)	OC	-2.783	1.537	2665.562	-5.797	0.232	-1.810	0.070	Advance
F	spring P (mm)	TM	0.024	0.012	2660.935	0.000	0.047	1.925	0.054	Delay
F	spring P (mm)	MS	0.042	0.009	2669.043	0.024	0.061	4.506	0.000	Delay
F	spring P (mm)	OC	0.051	0.036	2691.830	-0.019	0.121	1.418	0.156	Delay
FS	annual T (ºC)	TM	0.601	4.117	620.042	-7.484	8.687	0.146	0.884	Delay
FS	annual T (ºC)	MS	-3.508	2.213	621.593	-7.855	0.838	-1.585	0.113	Advance
FS	annual T (ºC)	OC	-8.050	3.665	609.752	-15.247	-0.853	-2.197	0.028	Advance
FS	annual P (mm)	TM	-0.025	0.015	621.404	-0.055	0.004	-1.691	0.091	Advance
FS	annual P (mm)	MS	0.014	0.011	617.798	-0.007	0.035	1.335	0.182	Delay
FS	annual P (mm)	OC	0.018	0.020	597.049	-0.021	0.058	0.912	0.362	Delay
FS	spring T (ºC)	TM	2.063	2.494	603.163	-2.836	6.962	0.827	0.409	Delay

FS	spring T (ºC)	MS	-3.444	1.516	625.086	-6.421	-0.468	-2.272	0.023	Advance
FS	spring T (ºC)	OC	-4.477	2.792	606.699	-9.961	1.007	-1.603	0.109	Advance
FS	spring P (mm)	TM	-0.041	0.039	618.458	-0.117	0.035	-1.060	0.289	Advance
FS	spring P (mm)	MS	0.035	0.023	634.027	-0.011	0.081	1.511	0.131	Delay
FS	spring P (mm)	OC	0.071	0.057	610.511	-0.041	0.183	1.253	0.211	Delay
DVG	annual T (ºC)	TM	-1.484	1.989	1601.861	-5.384	2.417	-0.746	0.456	Advance
DVG	annual T (ºC)	MS	-2.230	1.831	1629.337	-5.823	1.362	-1.218	0.223	Advance
DVG	annual T (ºC)	OC	-8.610	4.325	1639.556	-17.093	-0.128	-1.991	0.047	Advance
DVG	annual P (mm)	TM	0.008	0.007	1620.452	-0.006	0.022	1.119	0.263	Delay
DVG	annual P (mm)	MS	0.006	0.008	1618.672	-0.009	0.021	0.801	0.423	Delay
DVG	annual P (mm)	OC	0.010	0.034	1651.668	-0.056	0.076	0.299	0.765	Delay
DVG	spring T (ºC)	TM	1.386	1.242	1596.410	-1.049	3.822	1.116	0.264	Delay
DVG	spring T (ºC)	MS	-1.119	1.132	1631.853	-3.338	1.101	-0.989	0.323	Advance
DVG	spring T (ºC)	OC	-4.747	3.019	1639.655	-10.669	1.174	-1.572	0.116	Advance
DVG	spring P (mm)	TM	0.003	0.020	1598.070	-0.035	0.042	0.156	0.876	Delay
DVG	spring P (mm)	MS	0.002	0.016	1616.986	-0.030	0.034	0.131	0.896	Delay
DVG	spring P (mm)	OC	0.028548	0.06463	1650.6649	-0.09822	0.15531	0.44172	0.65875011	Delay

Table S4. Pairwise comparisons of slopes between thermotypes (post-hoc contrasts) in the phenophases with significant interaction effect. TM: thermo-Mediterranean, MS: meso-supra-Mediterranean, OC: oro-cryoro-Mediterranean. DVG: growth.

Phenophase	contrast	estimate	SE	df	t.ratio	p_value	Interpretation
FBF	TM - MS	0.017	0.012	1293.777	1.410	0.336	TM vs MS: ns
FBF	TM - OC	0.093	0.044	1350.690	2.135	0.083	TM vs OC: ns
FBF	MS - OC	0.077	0.044	1350.705	1.756	0.185	MS vs OC: ns

3. Model diagnostics

3.1. Residuals for FBF phenophase over annual T

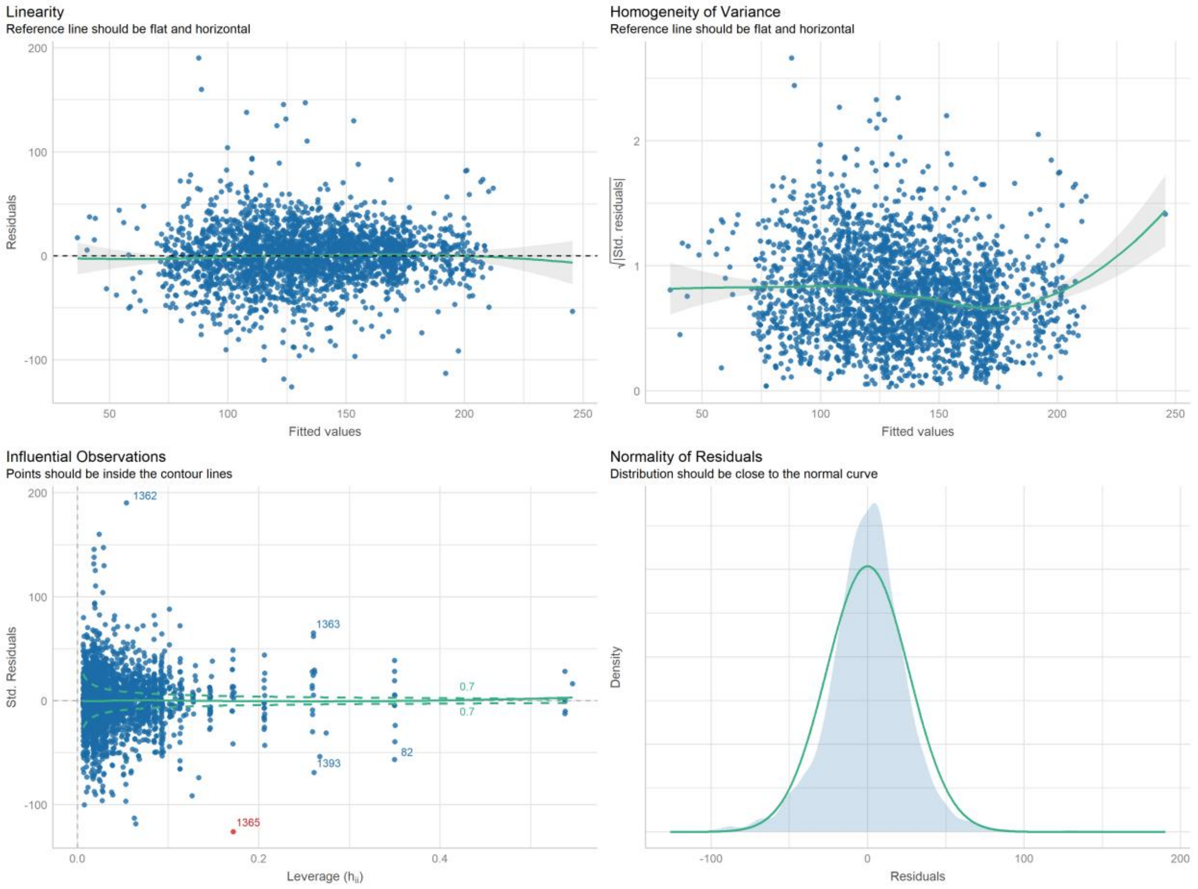

3.2. Residuals for F phenophase over annual T

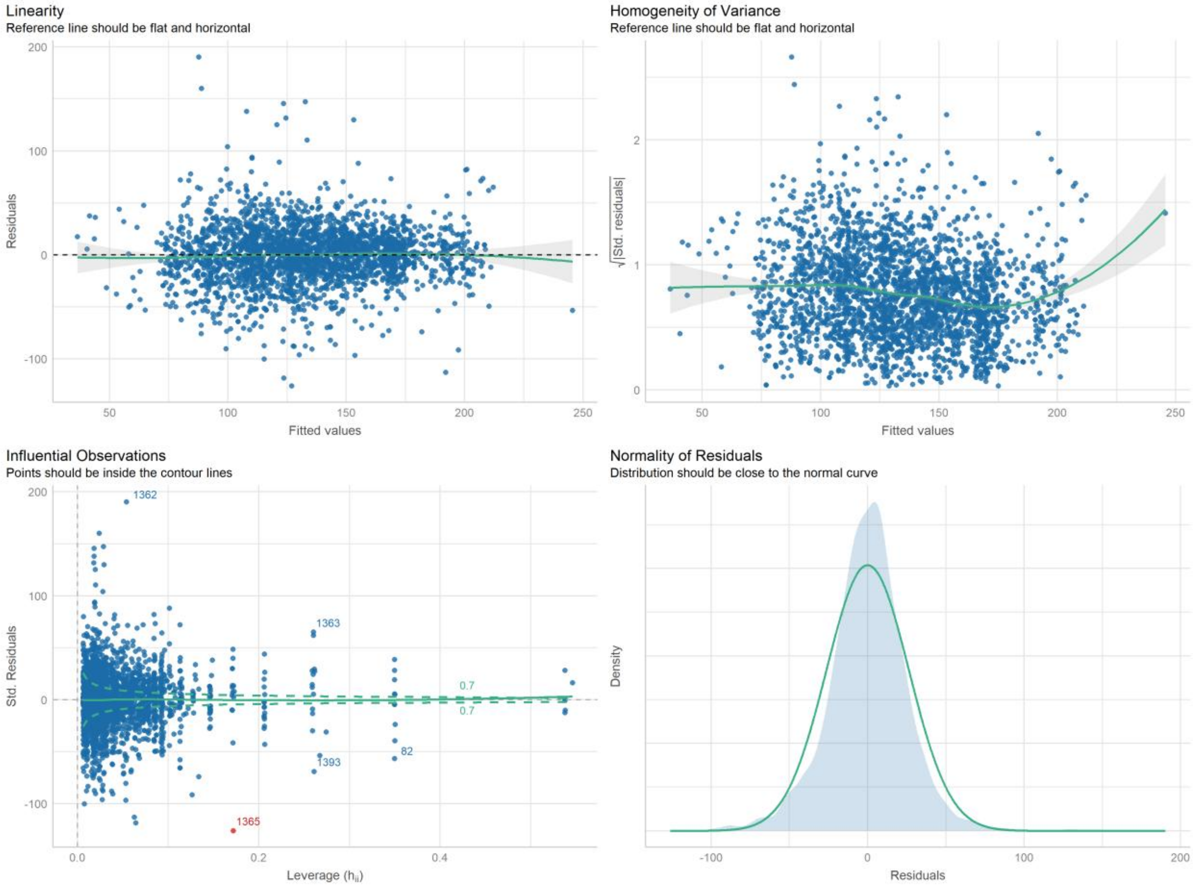

3.3 Residuals for FS phenophase over annual T

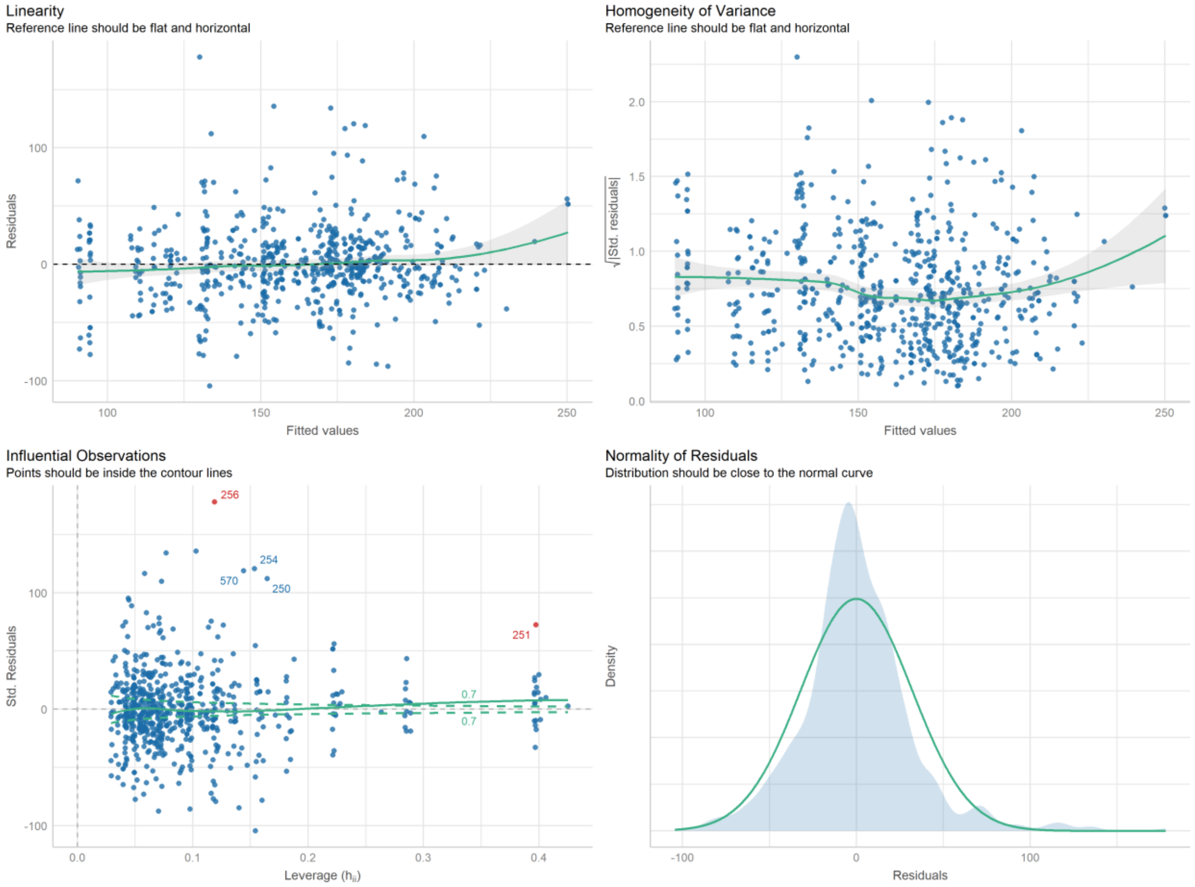

3.4 Residuals for DVG phenophase over annual T

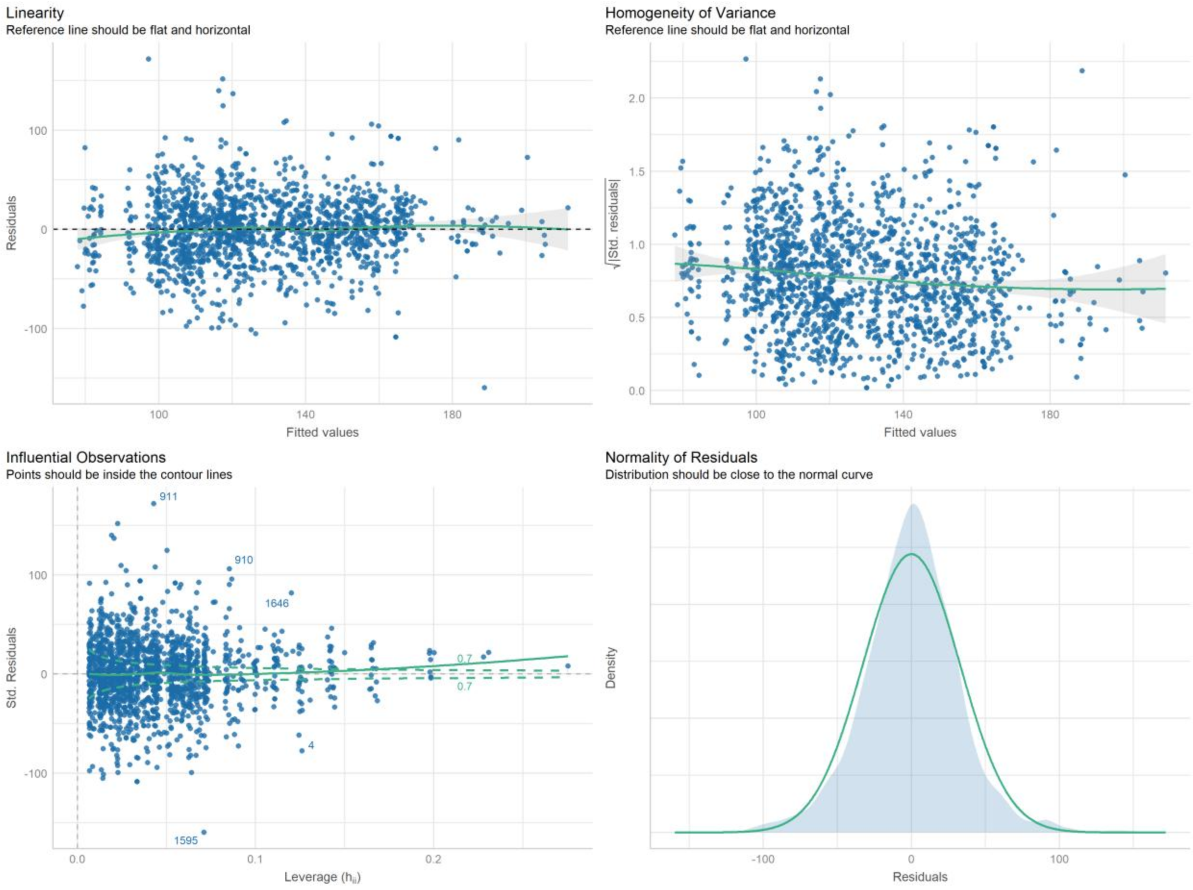

3.5 Residuals for FBF phenophase over spring T

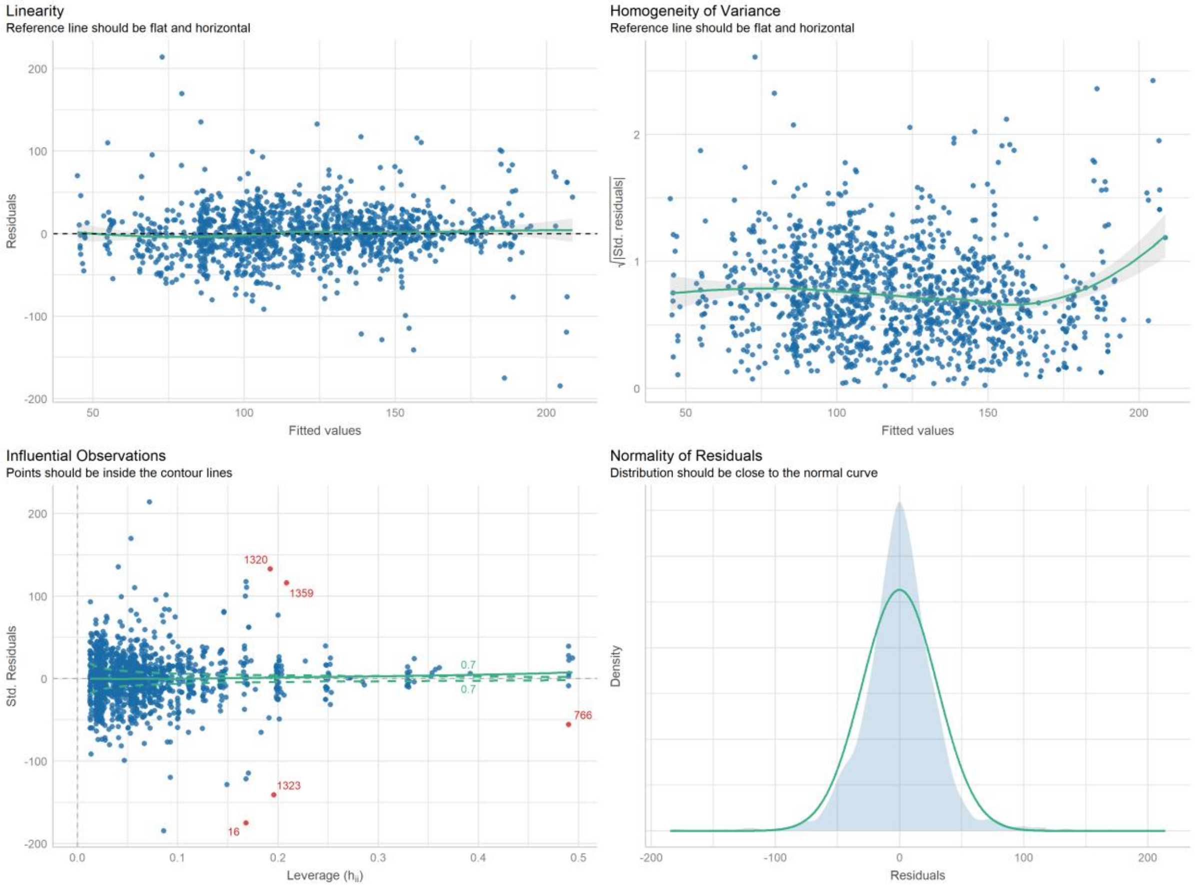

3.6 Residuals for F phenophase over spring T

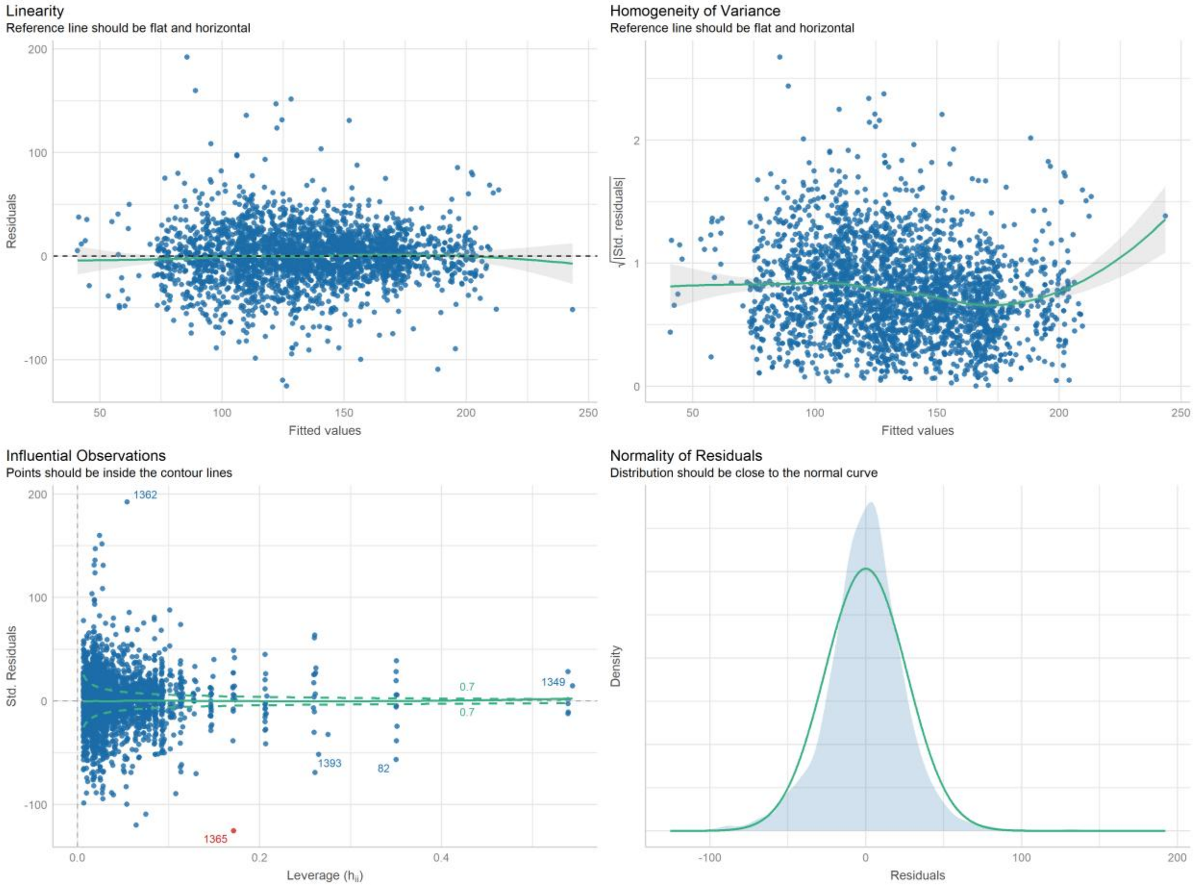

3.7 Residuals for FS phenophase over spring T

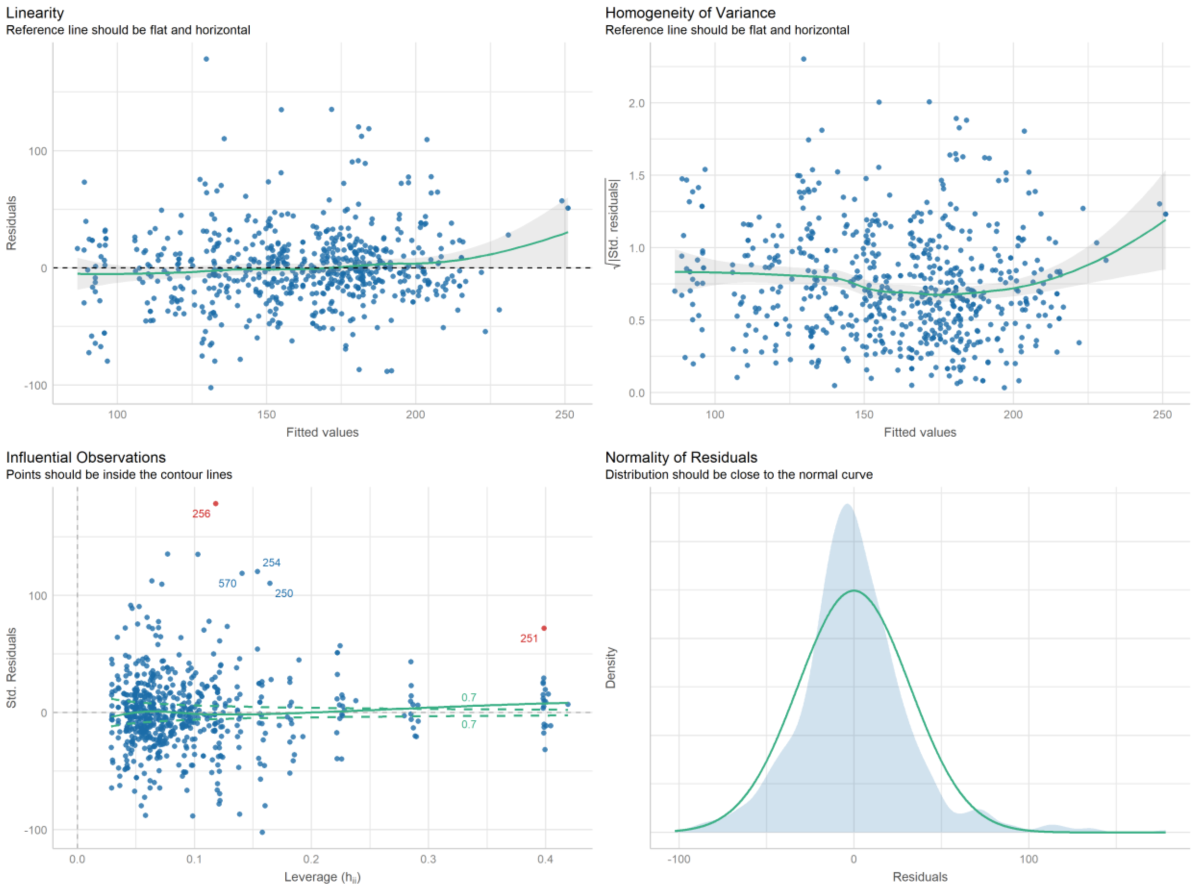

3.8 Residuals for DVG phenophase over spring T

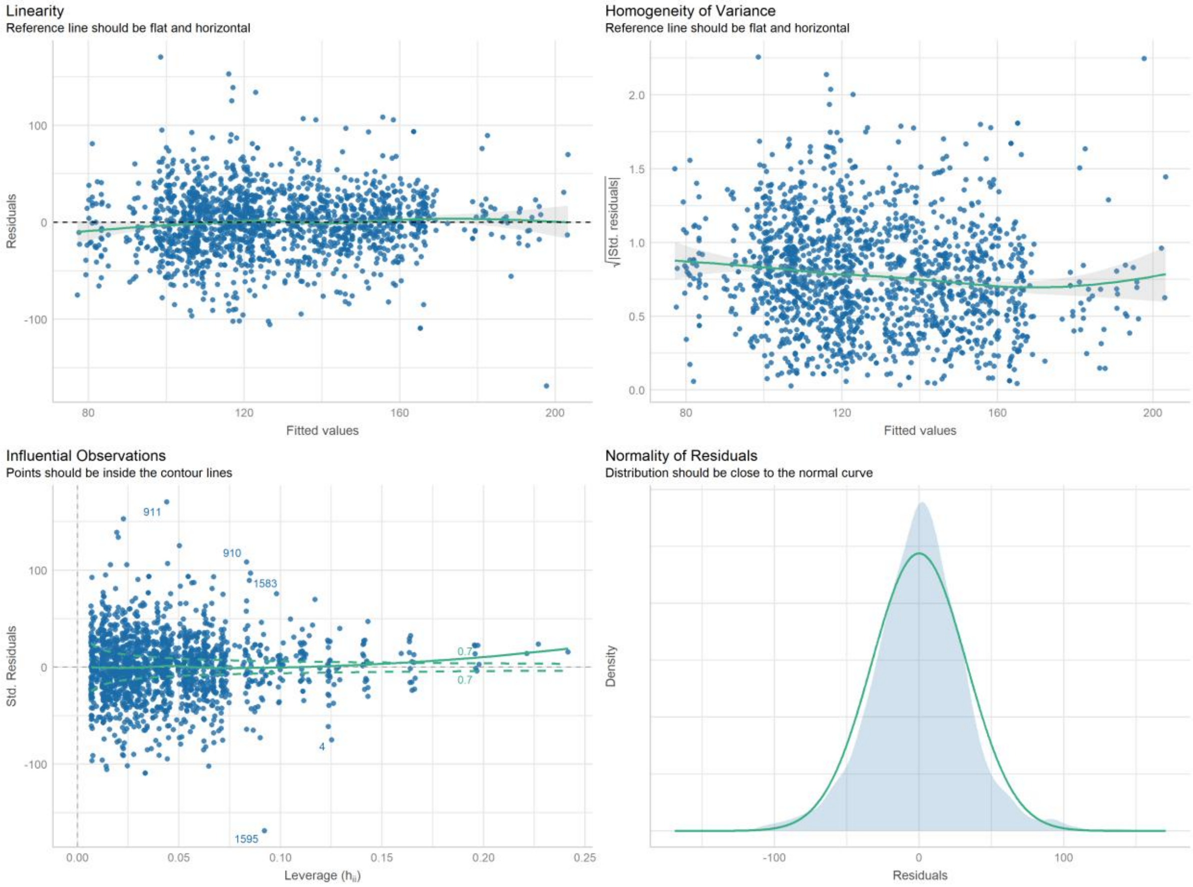

3.9 Residuals for FBF phenophase over annual P

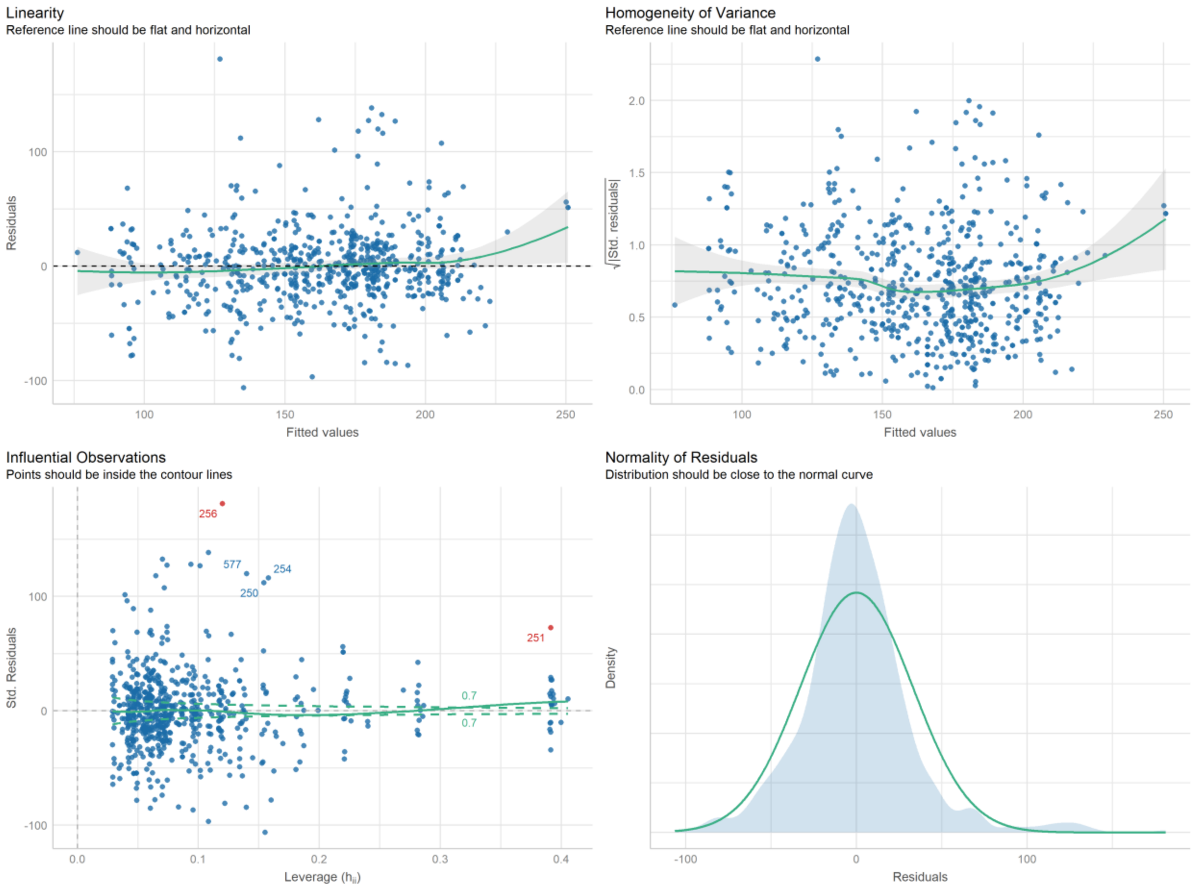

3.10 Residuals for F phenophase over annual P

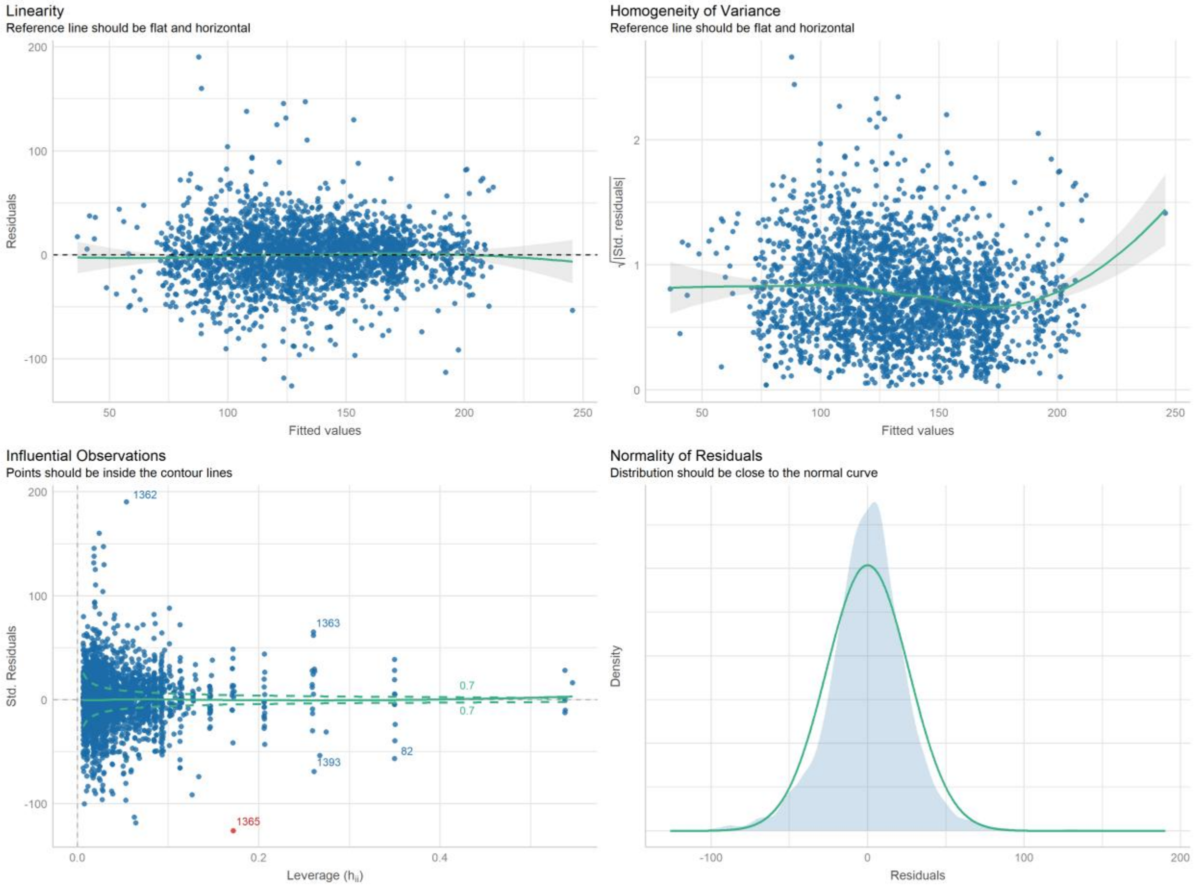

3.11 Residuals for FS phenophase over annual P

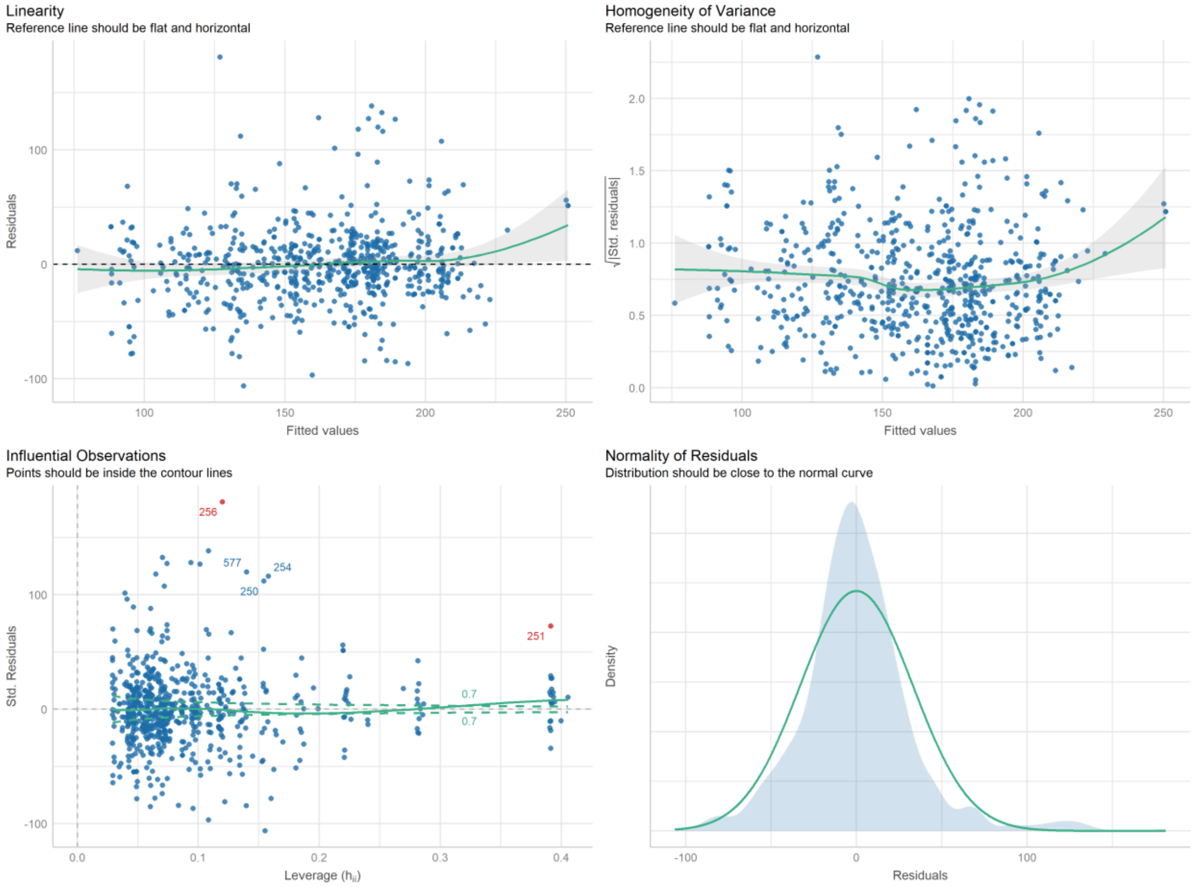

3.12 Residuals for DVG phenophase over annual P

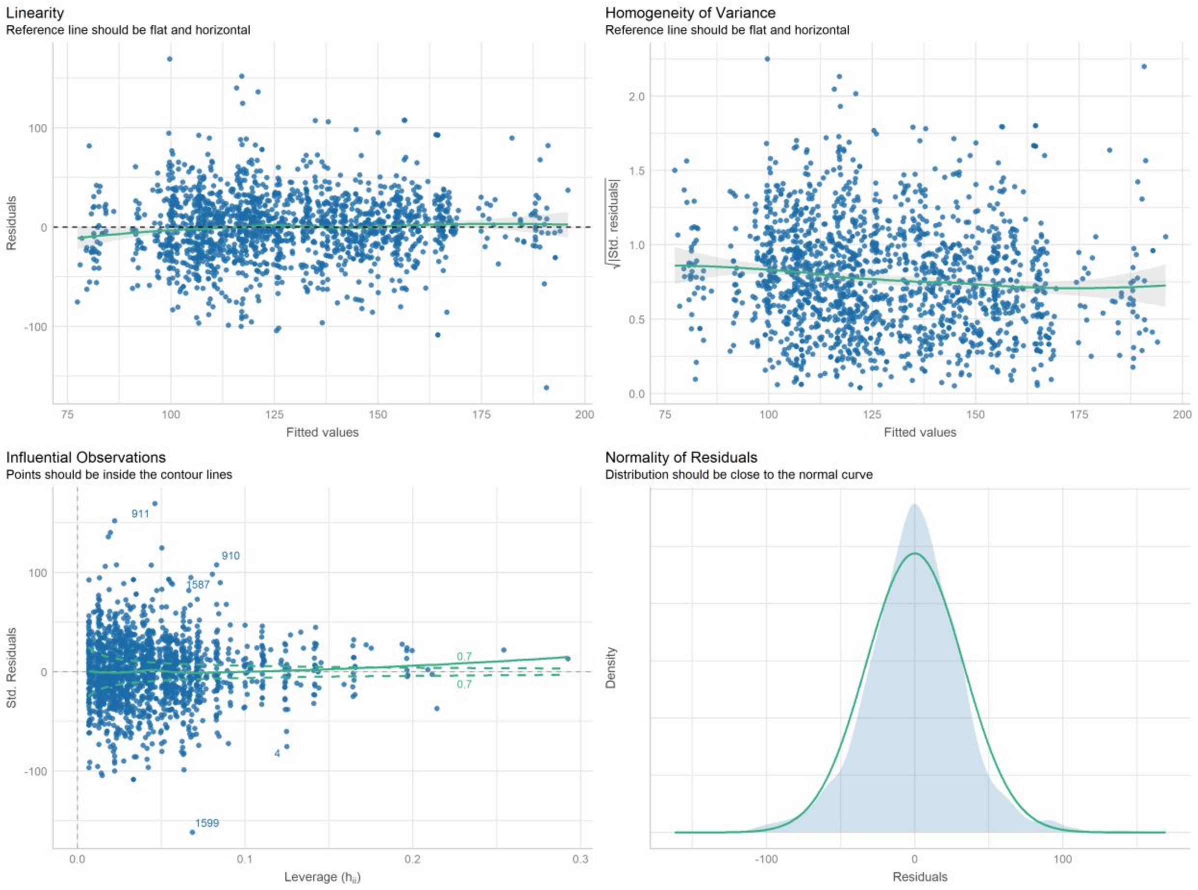

3.13 Residuals for FBF phenophase over spring P

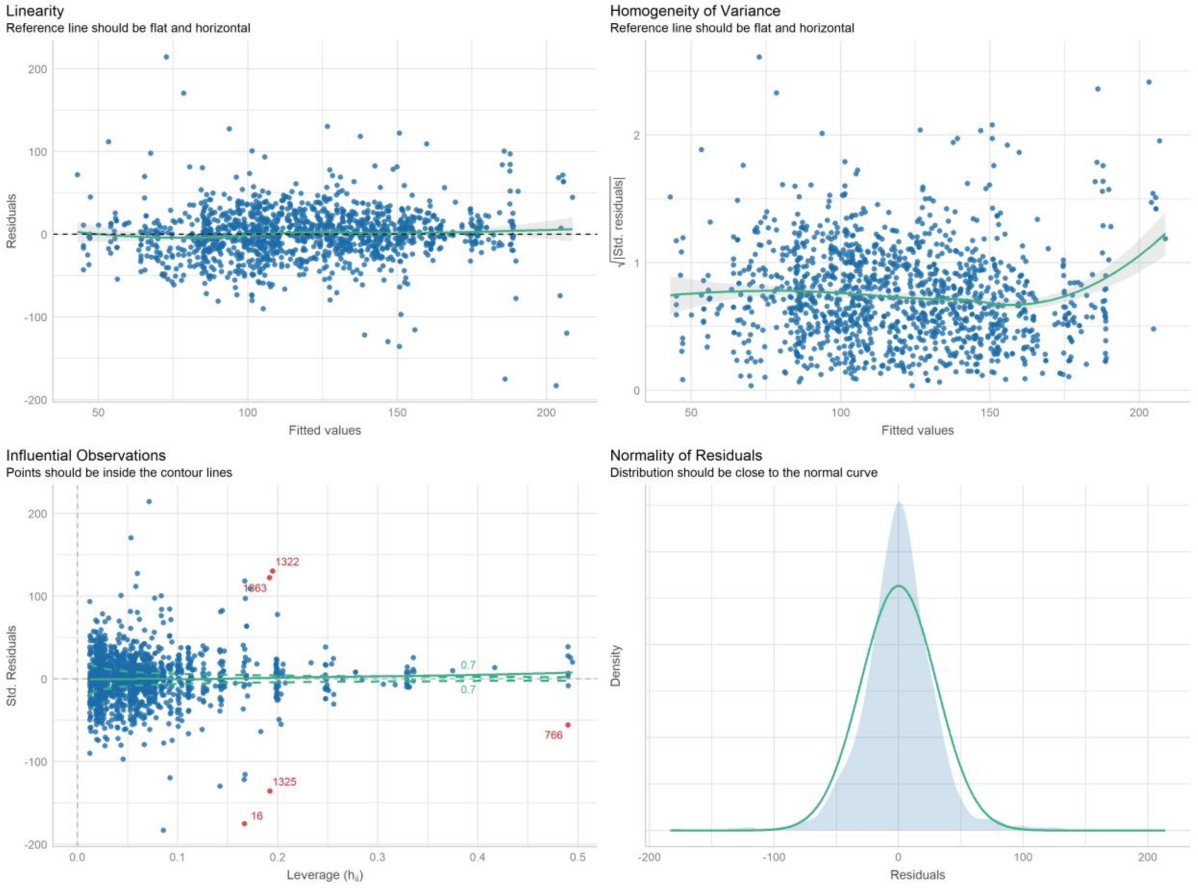

3.14Residuals for F phenophase over spring P

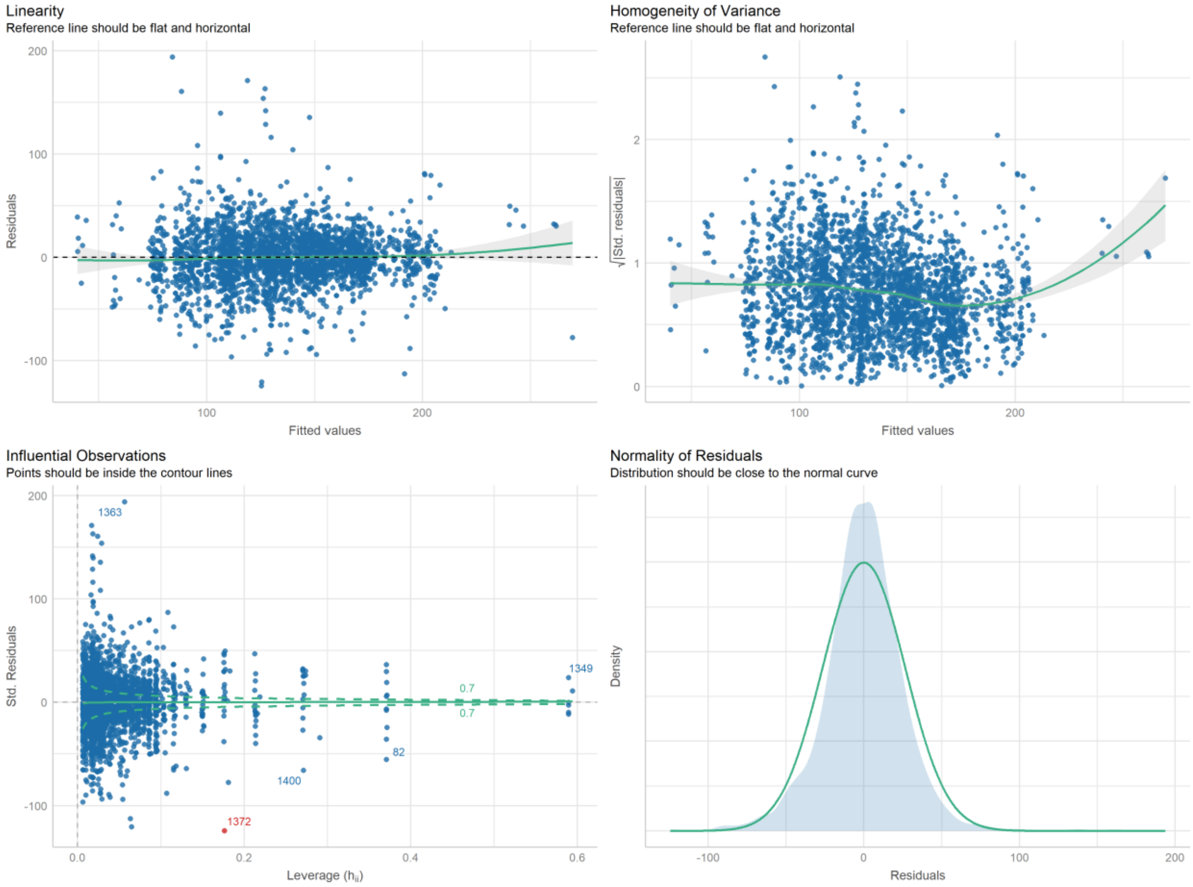

3.15 Residuals for FS phenophase over spring P

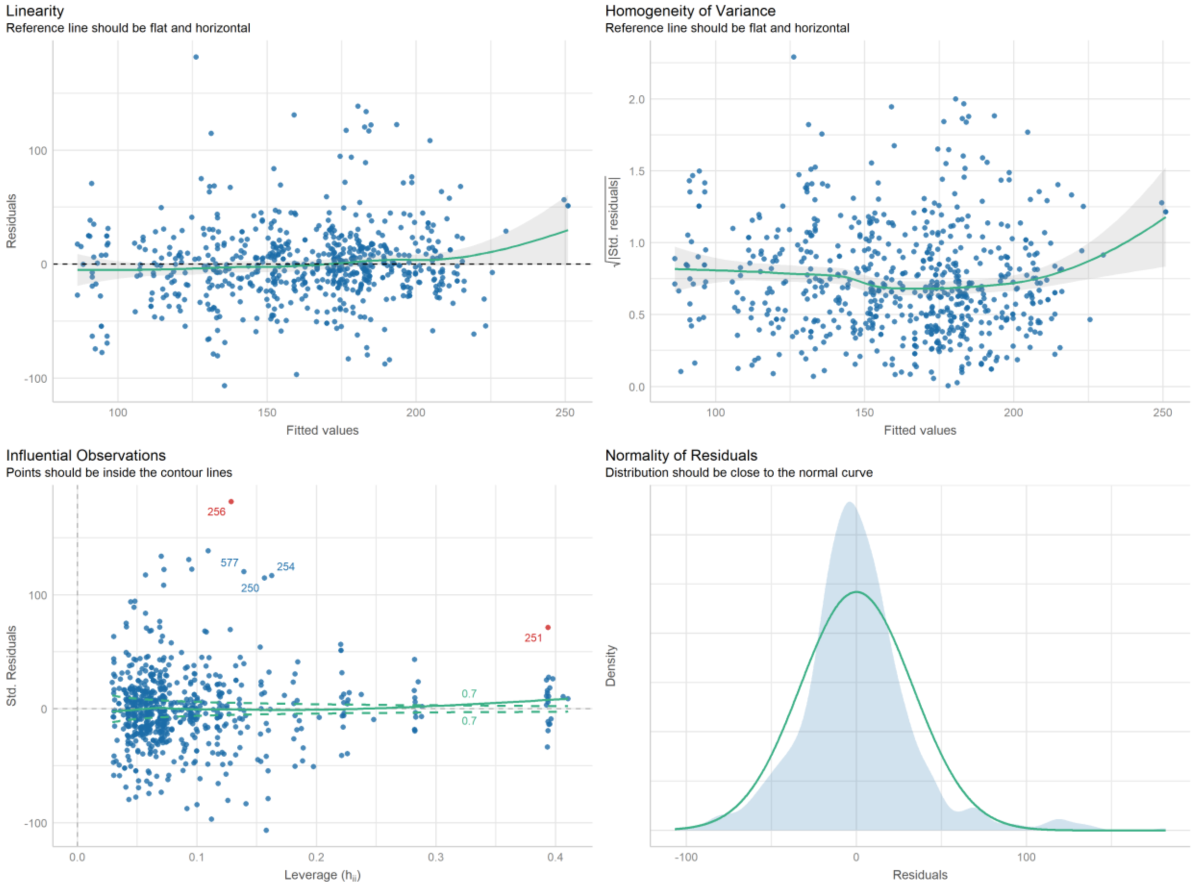

3.16 Residuals for DVG phenophase over spring P

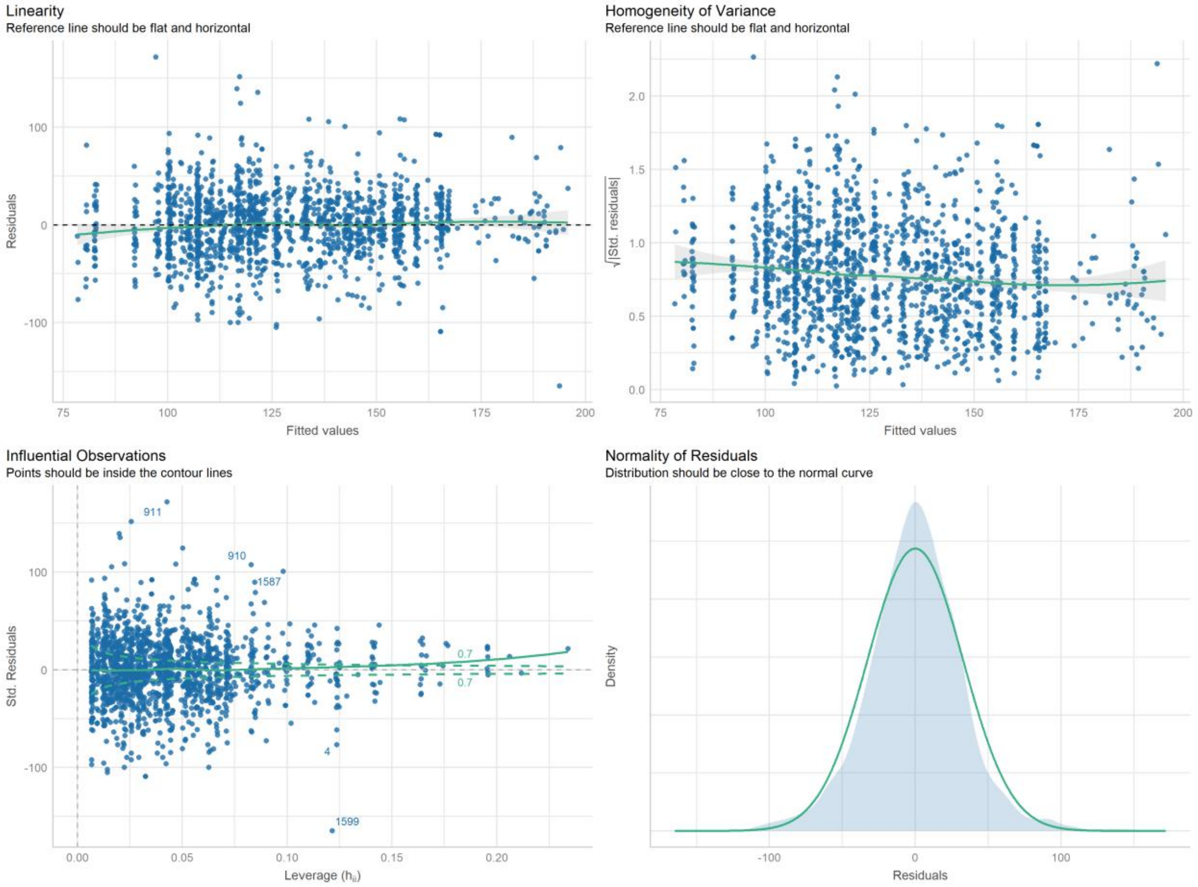

Supplement: plaf064_Supplementary_Data [file plaf064_supplementary_data.zip › Appendix_S4.pdf]
