# Supplementary material for: Retrospective phenology in western Mediterranean plants: revealing climate change patterns through herbarium specimens
Source: AoB Plants. 2025 Nov 3;17(6):plaf064. doi: 10.1093/aobpla/plaf064 (PMC12611260; doi:10.1093/aobpla/plaf064)
Supplement: plaf064_Supplementary_Data [file plaf064_supplementary_data.zip › Appendix_S5.pdf]

Supporting Information for

Retrospective phenology in western Mediterranean plants: revealing climate change patterns through herbarium specimens

APPENDIX S5 – DOY relationships with Climatic Variables – Taxa

Contents

1. MLM Model Results by taxa with diagnostics

2. GAM Model Results by taxa with diagnostics

3. AIC comparison between GLMs and GAMs

1. MLM Model Results by taxa with diagnostics

Table S1. Multiple Linear Model results by taxon and phenophase. Preflowering: FBF, flowering: F, fruiting: FS, growth: DVG. Annual mean temperature: Tmed.Y, monthly temperature: Tmon, spring temperature: T.MAM, total annual precipitation: Pannual_Y, spring precipitation: P.MAM. BP test: studentized Breusch-Pagan Test. KS: Kolmogorov-Smirnov test. SW: Saphiro-Wilk test.

Phenophase	Taxon	Intercept	Model	R^2	Ad. R^2	*p* value	Normality_p	BP Test	Test Used	p_value_Tmed.Y	slope_Tmed.Y	p_value_Tmon	slope_Tmon	p_value_T.MAM	slope_T.MAM	se_Tmed.Y	se_Tmon	se_T.MAM	p_value_Pannual.Y	slope_Pannual.Y	p_value_P.MAM	slope_P.MAM	se_Pannual.Y	se_P.MAM
**F**	*Phlomis purpurea*	127.6916019	lm3	0.84	0.84	6.67E-62	0.88	0.26	KS	0.08	-3.73	1.80E-63	8.39	8.84E-04	-5.73	2.12	0.29	1.69						
**F**	*Phillyrea angustifolia*	219.5080933	lm2	0.62	0.60	1.89E-07	0.79	0.06	SW	0.00	-14.35	4.65E-08	8.42			4.60	1.19							
**F**	*Glandora prostrata*	-56.0	lm3	0.	0.56	8.27E-06	0.01	0.43	SW	0.18	15.98	2.22E-06	8.05	2.12E-02	-15.50	11.75	1.36	6.35						

Phenophase	Taxon	Intercept	Model	R^2	Ad. R^2	*p* value	Normality_p	BP Test	Test Used	p_value_Tmed.Y	slope_Tmed.Y	p_value_Tmon	slope_Tmon	p_value_T.MAM	slope_T.MAM	se_Tmed.Y	se_Tmon	se_T.MAM	p_value_Pannual.Y	slope_Pannual.Y	p_value_P.MAM	slope_P.MAM	se_Pannual.Y	se_P.MAM
	subsp. lusitanica	4916937		60																				
**F**	*Cistus albidus*	87.32407942	lm3	0.85	0.84	9.27E-23	0.83	0.17	KS	0.61	-2.92	2.35E-24	10.84	5.18E-02	-6.61	5.62	0.62	3.33						
**F**	*Rhamnus alaternus*	135.1981547	lm3	0.74	0.72	7.41E-12	0.12	0.66	SW	0.54	-2.70	5.49E-12	9.62	2.30E-03	-9.07	4.34	1.00	2.78						
**F**	*Ulex parviflorus*	75.08582871	lm3	0.88	0.87	1.61E-17	0.11	0.01	SW	0.45	5.82	8.18E-19	11.49	9.39E-04	-15.88	7.69	0.70	4.43						
**F**	*Juniperus oxycedrus*	353.8877744	lm2	0.70	0.67	1.06E-05	0.08	0.62	SW	0.60	2.45	2.26E-06	-5.61			4.67	0.84							
**F**	*Helichrysum stoechas*	147.309198	lm3	0.76	0.76	5.71E-30	0.85	0.10	KS	0.85	-0.66	8.15E-32	6.24	2.56E-04	-7.76	3.46	0.35	2.04						
**F**	*Arbutus unedo*	292.8797806	lm2	0.75	0.71	2.42E-04	0.96	0.24	SW	0.02	6.09	3.60E-04	-5.60			2.36	1.14							
**F**	*Ceratonia siliqua*	260.0280904	lm3	0.78	0.73	1.59E-04	0.01	0.73	SW	0.79	1.43	1.54E-05	-5.81	5.79E-02	7.20	5.17	0.87	3.46						
**F**	*Erica arborea*	57.77901505	lm3	0.68	0.67	1.40E-12	0.59	0.95	KS	0.47	2.66	7.09E-14	8.66	1.40E-03	-8.62	3.67	0.85	2.55						
**F**	*Calicotome villosa*	239.09942	lm2	0.80	0.78	2.01E-09	0.38	0.43	SW	0.00	-17.15	2.31E-09	9.71			2.31	1.07							
**F**	*Pinus pinaster*	21.5405212	lm2	0.90	0.88	1.06E-06	0.38	0.52	SW	0.34	-5.27	4.09E-07	11.62			5.26	1.18							

Phenophase	Taxon	Intercept	Model	R^2	Ad. R^2	*p* value	Normality_p	BP Test	Test Used	p_value_Tmed.Y	slope_Tmed.Y	p_value_Tmon	slope_Tmon	p_value_T.MAM	slope_T.MAM	se_Tmed.Y	se_Tmon	se_T.MAM	p_value_Pannual.Y	slope_Pannual.Y	p_value_P.MAM	slope_P.MAM	se_Pannual.Y	se_P.MAM
**F**	*Calluna vulgaris*	420.2007275	lm2	0.70	0.66	6.55E-05	0.11	0.89	SW	0.77	-1.45	1.55E-05	-5.85			4.84	0.96							
**F**	*Viburnum tinus*	47.53666705	lm3	0.83	0.82	4.60E-11	0.16	0.59	SW	0.92	0.57	1.64E-11	10.24	8.25E-02	-7.27	5.45	0.95	4.03						
**F**	*Quercus coccifera*	165.2533976	lm2	0.67	0.66	1.34E-12	1.00	0.70	KS	0.00	-8.52	3.06E-13	6.11			2.44	0.62							
**F**	*Cytisus malacitanus*	116.9157354	lm2	0.87	0.86	2.07E-10	0.08	0.42	SW	0.03	-9.36	9.36E-11	9.50			3.97	0.83							
**F**	*Pistacia lentiscus*	130.8492504	lm3	0.78	0.77	1.70E-15	0.85	0.08	KS	0.76	-1.37	6.33E-17	7.62	4.00E-03	-7.90	4.44	0.60	2.61						
**F**	*Cistus ladanifer*	97.61060332	lm3	0.82	0.80	2.24E-18	0.66	0.09	KS	0.96	0.31	7.91E-20	8.06	2.55E-02	-8.13	5.71	0.55	3.53						
**F**	*Alyssum serpyllifolium subsp. malacitanum*	325.3952395	lm2	0.80	0.79	6.19E-12	0.46	0.06	SW	0.00	-18.16	1.08E-11	6.09			3.13	0.59							
**F**	*Linaria saturejoides*	68.02990448	lm3	0.77	0.75	2.11E-13	0.12	0.05	SW	0.74	1.88	2.38E-14	8.19	3.68E-02	-7.87	5.59	0.72	3.65						
**F**	*Helianthemum syriacum*	138.8815307	lm2	0.87	0.86	5.85E-10	0.41	0.17	SW	0.09	-7.36	7.56E-10	6.69			4.19	0.63							
**F**	*Rubia peregrina*	134.7620931	lm3	0.84	0.82	1.02E-12	0.19	0.03	SW	0.75	2.39	3.80E-14	7.90	1.67E-02	-12.40	7.50	0.62	4.91						
**F**	*Klasea baetica*	120.3560429	lm2	0.55	0.49	3.56E-03	0.27	0.22	SW	0.71	-1.91	1.58E-03	3.15			4.94	0.81							

Phenophase	Taxon	Intercept	Model	R^2	Ad. R^2	*p* value	Normality_p	BP Test	Test Used	p_value_Tmed.Y	slope_Tmed.Y	p_value_Tmon	slope_Tmon	p_value_T.MAM	slope_T.MAM	se_Tmed.Y	se_Tmon	se_T.MAM	p_value_Pannual.Y	slope_Pannual.Y	p_value_P.MAM	slope_P.MAM	se_Pannual.Y	se_P.MAM
**F**	*Myrtus communis*	142.2584761	lm2	0.85	0.83	2.32E-07	0.97	0.29	SW	0.02	-7.93	8.50E-08	7.21			2.97	0.78							
**F**	*Cistus populifolius*	303.2032635	lm2	0.63	0.58	9.31E-04	0.30	0.04	SW	0.07	-13.82	3.70E-03	3.45			6.93	0.99							
**F**	*Quercus rotundifolia*	238.0960955	lm2	0.81	0.80	4.37E-23	0.79	0.01	KS	0.00	-14.42	1.99E-23	7.60			2.17	0.48							
**F**	*Genista hirsuta subsp. lanuginosa*	237.7148513	lm2	0.88	0.87	1.25E-15	0.23	0.56	SW	0.00	-13.59	2.42E-14	6.75			2.99	0.53							
**F**	*Quercus faginea*	255.6298645	lm2	0.83	0.83	3.28E-23	0.44	0.26	KS	0.00	-15.10	2.60E-22	6.93			2.63	0.45							
**F**	*Quercus suber*	166.5385221	lm2	0.72	0.70	3.92E-07	0.04	0.09	SW	0.18	-9.28	8.93E-08	6.89			6.67	0.90							
**F**	*Crataegus monogyna*	142.2676629	lm2	0.85	0.85	9.48E-23	0.99	0.78	KS	0.00	-8.09	3.30E-23	7.22			1.62	0.42							
**F**	*Macrochloa tenacissima*	83.6120953	lm2	0.88	0.87	1.12E-13	0.11	0.69	SW	0.06	-5.63	1.82E-14	7.80			2.88	0.54							
**F**	*Erica scoparia*	294.1017811	lm2	0.72	0.69	1.65E-06	0.49	0.83	SW	0.01	-15.81	1.34E-06	6.04			5.24	0.91							
**F**	*Adenocarpus telonensis*	215.5483018	lm2	0.79	0.78	5.50E-12	0.53	0.53	SW	0.01	-12.33	8.89E-13	6.92			4.37	0.62							
**F**	*Retama sphaerocarpa*	305.7216778	lm2	0.71	0.67	1.70E-04	0.30	0.08	SW	0.01	-14.79	2.69E-04	4.33			5.17	0.90							

Phenophase	Taxon	Intercept	Model	R^2	Ad. R^2	*p* value	Normality_p	BP Test	Test Used	p_value_Tmed.Y	slope_Tmed.Y	p_value_Tmon	slope_Tmon	p_value_T.MAM	slope_T.MAM	se_Tmed.Y	se_Tmon	se_T.MAM	p_value_Pannual.Y	slope_Pannual.Y	p_value_P.MAM	slope_P.MAM	se_Pannual.Y	se_P.MAM
**F**	*Thymus mastichina*	200.6078779	lm2	0.71	0.70	1.68E-18	0.98	0.54	KS	0.00	-10.00	1.76E-18	5.89			1.35	0.49							
**F**	*Echium albicans*	197.6640398	lm3	0.73	0.72	7.48E-15	0.84	0.48	KS	0.29	-4.71	1.52E-15	5.59	1.30E-02	-5.72	4.38	0.50	2.22						
**F**	*Chaenorhinum glareosum*	222.3971268	lm2	0.32	0.25	2.06E-02	0.24	0.05	SW	0.01	-7.86	7.57E-02	4.59			2.90	2.45							
**F**	*Halimium atriplicifolium*	136.8972131	lm5	0.95	0.93	1.26E-08	0.68	0.65	SW	0.26	-6.26	1.50E-08	6.81	4.15E-01	-2.84	5.32	0.59	3.38	0.27	0.01	0.01	0.10	0.01	0.04
**F**	*Cistus laurifolius*	191.4171477	lm2	0.59	0.52	4.98E-03	0.16	0.51	SW	0.08	-5.71	1.78E-03	3.12			3.01	0.78							
**F**	*Salvia lavandulifolia*	190.8670128	lm2	0.43	0.38	2.55E-03	0.19	0.66	SW	0.36	-6.44	6.33E-04	3.81			6.91	0.95							
**F**	*Staehelina baetica*	224.8599568	lm2	0.62	0.57	1.15E-04	0.23	0.35	SW	0.04	-9.80	2.75E-05	4.77			4.40	0.87							
**F**	*Centaurea prolongoi*	220.757224	lm2	0.83	0.82	3.15E-15	0.04	0.39	SW	0.00	-12.75	4.78E-16	7.05			3.33	0.52							
**F**	*Crataegus granatensis*	213.9453043	lm1	0.16	0.12	4.84E-02	0.04	0.56	SW	0.05	-3.13					1.50								
**F**	*Prunus prostrata*	188.8243792	lm2	0.75	0.74	3.54E-10	0.38	0.10	SW	0.00	-8.81	6.21E-10	5.55			2.66	0.63							
**F**	*Centaurea carratracensis*	198.2115716	lm2	0.72	0.69	5.32E-06	0.29	0.07	SW	0.07	-9.66	1.37E-06	5.73			4.97	0.83							

Phenophase	Taxon	Intercept	Model	R^2	Ad. R^2	*p* value	Normality_p	BP Test	Test Used	p_value_Tmed.Y	slope_Tmed.Y	p_value_Tmon	slope_Tmon	p_value_T.MAM	slope_T.MAM	se_Tmed.Y	se_Tmon	se_T.MAM	p_value_Pannual.Y	slope_Pannual.Y	p_value_P.MAM	slope_P.MAM	se_Pannual.Y	se_P.MAM
**F**	*Salvia candelabrum*	148.9167938	lm2	0.76	0.72	1.90E-04	0.72	0.90	SW	0.12	-7.37	2.03E-04	6.05			4.43	1.15							
**F**	*Lonicera etrusca*	179.596304	lm2	0.68	0.66	2.88E-10	0.14	0.16	SW	0.00	-7.77	4.28E-10	5.41			1.66	0.66							
**F**	*Saxifraga reuteriana*	160.3989613	lm2	0.79	0.77	1.40E-07	0.80	0.14	SW	0.06	-6.80	2.74E-08	5.28			3.41	0.60							
**F**	*Thymus longiflorus*	196.6863644	lm3	0.63	0.61	2.14E-19	0.93	0.05	KS	0.06	-5.46	1.57E-17	5.02	6.76E-02	-3.62	2.84	0.47	1.96						
**F**	*Hormathophylla spinosa*	202.3224524	lm2	0.78	0.76	2.48E-07	0.26	0.13	SW	0.00	-11.73	1.80E-07	7.53			2.74	0.97							
**F**	*Quercus faginea subsp. alpestris*	153.876067	lm2	0.68	0.62	1.12E-03	0.11	0.74	SW	0.08	-6.38	6.13E-04	5.17			3.33	1.12							
**F**	*Elaeoselinum asclepium subsp. millefolium*	260.5399536	lm2	0.77	0.74	4.35E-06	0.14	0.32	SW	0.00	-12.51	1.19E-06	5.24			3.59	0.71							
**F**	*Phlomis crinita subsp. malacitana*	186.4933885	lm2	0.57	0.55	2.41E-08	0.01	0.45	SW	0.00	-7.02	1.44E-08	4.55			2.19	0.65							
**F**	*Crepis oporinoides*	272.8991885	lm3	0.85	0.81	7.76E-05	0.68	0.24	SW	0.00	-31.02	2.90E-04	10.39	3.13E-02	11.76	6.44	2.00	4.77						
**F**	*Leontodon boryi*	245.6715972	lm1	0.05	-0.01	3.67E-01	0.80	0.14	SW	0.37	-2.90					3.11								
**F**	*Sempervivum minutum*	335.2187998	lm3	0.55	0.47	4.14E-03	0.29	0.54	SW	0.01	-35.11	4.22E-03	7.34	3.51E-02	18.07	11.03	2.20	7.85						

Phenophase	Taxon	Intercept	Model	R^2	Ad. R^2	*p* value	Normality_p	BP Test	Test Used	p_value_Tmed.Y	slope_Tmed.Y	p_value_Tmon	slope_Tmon	p_value_T.MAM	slope_T.MAM	se_Tmed.Y	se_Tmon	se_T.MAM	p_value_Pannual.Y	slope_Pannual.Y	p_value_P.MAM	slope_P.MAM	se_Pannual.Y	se_P.MAM
**F**	*Nevadensia purpurea*	298.1503959	lm1	0.25	0.21	1.61E-02	0.73	0.82	SW	0.02	-6.29					2.41								
**F**	*Sideritis glacialis*	300.4708394	lm3	0.46	0.41	1.39E-04	0.31	0.27	SW	0.01	17.70	7.75E-03	-4.60	1.55E-03	-19.05	6.22	1.62	5.52						
**FBF**	*Phlomis purpurea*	127.6775174	lm3	0.86	0.85	2.31E-32	0.99	0.76	KS	0.36	-2.91	4.85E-34	8.86	3.43E-03	-7.16	3.17	0.41	2.37						
**FBF**	*Phillyrea angustifolia*	224.5493138	lm2	0.71	0.68	8.14E-06	0.86	0.26	SW	0.02	-17.83	2.25E-06	11.75			7.03	1.76							
**FBF**	*Salvia rosmarinus*	118.6691732	lm2	0.73	0.69	1.06E-04	0.95	0.27	SW	0.41	-9.32	9.53E-05	8.92			11.07	1.65							
**FBF**	*Cistus albidus*	77.07552565	lm3	0.85	0.84	3.91E-15	0.20	0.28	SW	0.84	-1.47	7.11E-16	12.05	5.62E-02	-8.60	7.00	0.88	4.36						
**FBF**	*Rhamnus alaternus*	287.95748	lm2	0.84	0.82	2.59E-06	0.13	0.16	SW	0.01	-20.93	5.86E-07	9.96			6.83	1.16							
**FBF**	*Ulex parviflorus*	179.4407833	lm2	0.85	0.82	5.02E-06	0.57	0.53	SW	0.04	-16.56	1.61E-06	13.24			7.05	1.61							
**FBF**	*Juniperus oxycedrus*	539.6475693	lm1	0.04	-0.03	4.57E-01	0.27	0.75	SW	0.46	-18.02					23.56								
**FBF**	*Helichrysum stoechas*	209.580595	lm2	0.87	0.86	1.92E-07	0.24	0.94	SW	0.09	-15.32	1.15E-07	9.67			8.60	1.03							
**FBF**	*Lavandula stoechas*	100.3563632	lm2	0.81	0.80	4.79E-19	0.89	0.14	KS	0.05	-8.43	1.81E-19	9.59			4.18	0.67							

Phenophase	Taxon	Intercept	Model	R^2	Ad. R^2	*p* value	Normality_p	BP Test	Test Used	p_value_Tmed.Y	slope_Tmed.Y	p_value_Tmon	slope_Tmon	p_value_T.MAM	slope_T.MAM	se_Tmed.Y	se_Tmon	se_T.MAM	p_value_Pannual.Y	slope_Pannual.Y	p_value_P.MAM	slope_P.MAM	se_Pannual.Y	se_P.MAM
**FBF**	*Erica arborea*	180.549132	lm2	0.71	0.68	4.66E-06	0.06	0.93	SW	0.07	-13.18	1.68E-06	8.81			6.82	1.32							
**FBF**	*Fumana thymifolia*	-20.56862185	lm3	0.77	0.75	2.03E-13	0.82	0.81	SW	0.03	10.12	2.73E-14	8.96	3.53E-04	-11.89	4.41	0.79	3.06						
**FBF**	*Cistus ladanifer*	-41.13779063	lm3	0.71	0.69	5.56E-12	0.12	0.34	SW	0.09	12.03	2.82E-13	9.38	1.67E-03	-13.45	6.88	0.91	4.01						
**FBF**	*Alyssum serpyllifolium subsp. malacitanum*	53.53930187	lm3	0.74	0.68	2.52E-04	0.10	0.86	SW	0.26	11.36	1.01E-04	8.72	2.29E-02	-18.09	9.70	1.63	7.08						
**FBF**	*Helianthemum syriacum*	64.25965538	lm2	0.86	0.85	6.19E-15	0.85	0.10	SW	0.37	-3.19	1.22E-15	6.91			3.49	0.49							
**FBF**	*Rubia peregrina*	73.67914086	lm3	0.83	0.80	3.11E-08	0.15	0.52	SW	0.69	2.85	3.31E-09	7.97	2.74E-02	-8.72	7.00	0.82	3.68						
**FBF**	*Olea europaea var. sylvestris*	56.81399313	lm2	0.48	0.41	1.34E-02	0.78	0.03	SW	0.90	-0.81	4.00E-03	4.63			6.34	1.33							
**FBF**	*Cistus populifolius*	-38.07562042	lm3	0.90	0.88	6.73E-13	0.41	0.20	SW	0.05	13.32	1.78E-12	7.60	2.57E-04	-12.96	6.50	0.61	3.06						
**FBF**	*Genista hirsuta subsp. lanuginosa*	42.60905239	lm5	0.90	0.87	8.44E-08	0.47	0.52	SW	0.00	-19.05	1.56E-09	7.72	6.84E-03	14.81	5.15	0.66	4.82	0.01	0.05	4.14E-03	0.17	0.02	0.05
**FBF**	*Crataegus monogyna*	69.89168771	lm3	0.80	0.76	1.92E-05	0.97	0.19	SW	0.39	6.74	3.59E-04	7.81	3.28E-02	-13.12	7.65	1.70	5.58						
**FBF**	*Macrochloa tenacissima*	110.4338626	lm4	0.91	0.87	3.38E-05	0.28	0.46	SW	0.86	0.87	2.46E-05	8.27	2.16E-03	-11.35	4.80	1.13	2.77	0.02	0.03			0.01	

Phenophase	Taxon	Intercept	Model	R^2	Ad. R^2	*p* value	Normality_p	BP Test	Test Used	p_value_Tmed.Y	slope_Tmed.Y	p_value_Tmon	slope_Tmon	p_value_T.MAM	slope_T.MAM	se_Tmed.Y	se_Tmon	se_T.MAM	p_value_Pannual.Y	slope_Pannual.Y	p_value_P.MAM	slope_P.MAM	se_Pannual.Y	se_P.MAM
**FBF**	*Erica scoparia*	153.1697868	lm2	0.58	0.54	3.67E-04	0.57	0.31	SW	0.12	-8.74	1.15E-04	6.57			5.29	1.34							
**FBF**	*Thymus mastichina*	184.1974158	lm2	0.75	0.74	2.64E-13	0.96	0.59	SW	0.00	-9.28	6.07E-14	5.91			1.93	0.54							
**FBF**	*Halimium atriplicifolium*	265.7784461	lm2	0.81	0.81	7.72E-21	0.96	0.87	KS	0.00	-15.23	2.59E-21	6.86			3.61	0.45							
**FBF**	*Cistus laurifolius*	174.0434692	lm2	0.72	0.68	3.92E-05	0.83	0.97	SW	0.06	-5.53	9.83E-06	3.64			2.75	0.57							
**FBF**	*Salvia lavandulifolia*	140.067333	lm2	0.74	0.69	3.39E-04	0.95	0.98	SW	0.49	-4.72	1.64E-04	4.88			6.56	0.91							
**FBF**	*Staehelina baetica*	88.3180859	lm2	0.71	0.66	5.70E-04	0.25	0.67	SW	0.67	-2.19	2.64E-04	4.54			5.06	0.89							
**FBF**	*Centaurea prolongoi*	163.4481799	lm2	0.74	0.70	7.87E-05	0.07	0.99	SW	0.27	-6.60	2.27E-05	4.50			5.69	0.72							
**FBF**	*Salvia candelabrum*	196.189821	lm2	0.45	0.37	1.48E-02	0.48	0.92	SW	0.14	-7.48	5.00E-03	3.75			4.84	1.13							
**FBF**	*Hormathophylla spinosa*	101.7890939	lm2	0.80	0.78	2.44E-06	0.13	0.36	SW	0.06	-4.40	6.63E-07	6.38			2.17	0.81							
**FBF**	*Lavandula lanata*	182.6270939	lm2	0.52	0.48	1.44E-04	0.43	0.73	SW	0.17	-5.91	1.52E-04	3.99			4.14	0.89							
**FBF**	*Phlomis crinita subsp. malacitana*	200.1013965	lm2	0.59	0.55	1.37E-04	0.07	0.24	SW	0.00	-6.77	1.15E-04	3.56			2.09	0.74							

Phenophase	Taxon	Intercept	Model	R^2	Ad. R^2	*p* value	Normality_p	BP Test	Test Used	p_value_Tmed.Y	slope_Tmed.Y	p_value_Tmon	slope_Tmon	p_value_T.MAM	slope_T.MAM	se_Tmed.Y	se_Tmon	se_T.MAM	p_value_Pannual.Y	slope_Pannual.Y	p_value_P.MAM	slope_P.MAM	se_Pannual.Y	se_P.MAM
**FBF**	*Sideritis glacialis*	167.6206948	lm2	0.39	0.31	2.46E-02	0.44	0.19	SW	0.03	-7.10	1.27E-02	5.60			2.95	1.98							
**FS**	*Rhamnus alaternus*	61.26166598	lm2	0.87	0.86	3.17E-14	0.85	0.05	SW	0.32	-3.92	6.72E-15	7.58			3.86	0.54							
**FS**	*Cytisus malacitanus*	88.10633417	lm2	0.85	0.83	3.91E-06	0.61	0.40	SW	0.29	-8.35	1.91E-06	10.31			7.57	1.27							
**FS**	*Juniperus phoenicea*	342.9666366	lm3	0.94	0.93	4.50E-07	0.78	0.16	SW	0.00	-34.17	3.53E-07	12.16	6.56E-02	8.84	8.86	1.13	4.32						
**FS**	*Cistus ladanifer*	187.5089295	lm2	0.89	0.88	1.71E-10	0.71	0.05	SW	0.05	-10.67	6.95E-11	6.70			5.11	0.54							
**FS**	*Cistus populifolius*	330.0534557	lm2	0.66	0.61	5.54E-04	0.54	0.12	SW	0.02	-17.55	1.45E-04	5.81			6.75	1.13							
**FS**	*Teline linifolia*	-47.67595	lm3	0.97	0.96	7.85E-09	0.96	0.66	SW	0.10	15.11	1.79E-08	9.06	9.14E-03	-15.69	8.32	0.63	4.97						
**FS**	*Thymus mastichina*	115.2494545	lm2	0.87	0.85	1.86E-09	0.02	0.07	SW	0.00	-6.23	6.02E-10	7.27			1.81	0.66							
**FS**	*Prunus mahaleb*	127.851445	lm2	0.75	0.72	1.11E-04	0.29	0.05	SW	0.05	-5.52	3.78E-05	6.33			2.62	1.04							
**FS**	*Crataegus granatensis*	138.5858682	lm1	0.77	0.75	1.80E-05	0.07	0.41	SW	0.00	1.49					0.23								
**FS**	*Prunus prostrata*	206.8933423	lm3	0.68	0.59	4.91E-03	0.80	0.61	SW	0.01	-28.43	6.15E-04	8.89	4.37E-02	15.79	9.71	1.88	6.93						

Phenophase	Taxon	Intercept	Model	R^2	Ad. R^2	*p* value	Normality_p	BP Test	Test Used	p_value_Tmed.Y	slope_Tmed.Y	p_value_Tmon	slope_Tmon	p_value_T.MAM	slope_T.MAM	se_Tmed.Y	se_Tmon	se_T.MAM	p_value_Pannual.Y	slope_Pannual.Y	p_value_P.MAM	slope_P.MAM	se_Pannual.Y	se_P.MAM
**FS**	*Acer granatense*	180.5957356	lm3	0.62	0.55	1.71E-03	0.28	0.04	SW	0.03	-17.48	2.77E-04	6.03	4.32E-02	10.36	7.29	1.28	4.69						
**FS**	*Helictotrichon filifolium subsp. arundanum*	256.1582172	lm2	0.66	0.61	5.63E-04	0.47	0.17	SW	0.06	-10.52	1.42E-04	3.76			5.11	0.73							
**FS**	*Nevadensia purpurea*	306.2573149	lm1	0.24	0.19	5.40E-02	0.78	0.22	SW	0.05	-6.49					3.09								
**DVG**	*Phlomis purpurea*	118.3496439	lm3	0.84	0.83	1.00E-55	0.45	0.30	KS	0.13	-4.11	6.23E-57	8.84	7.81E-03	-5.14	2.72	0.33	1.90						
**DVG**	*Phillyrea angustifolia*	44.06987063	lm2	0.92	0.91	8.33E-08	1.00	0.46	SW	0.33	-5.50	2.03E-08	10.19			5.47	0.85							
**DVG**	*Cistus albidus*	231.3539575	lm2	0.86	0.85	2.60E-14	0.87	0.30	SW	0.02	-17.37	2.76E-14	10.58			6.99	0.82							
**DVG**	*Juniperus sabina*	-45.97897513	lm1	0.08	0.04	1.76E-01	0.09	0.07	SW	0.18	14.02					10.03								
**DVG**	*Rhamnus alaternus*	122.8469193	lm2	0.88	0.88	6.32E-35	1.00	0.45	KS	0.00	-9.35	7.20E-36	8.97			2.87	0.38							
**DVG**	*Ulex parviflorus*	173.8233943	lm3	0.75	0.70	8.94E-05	0.10	0.41	SW	0.65	3.58	1.70E-05	7.16	8.54E-03	-15.18	7.71	1.15	5.02						
**DVG**	*Juniperus oxycedrus*	140.9881539	lm3	0.87	0.87	9.44E-35	0.99	0.61	KS	0.22	-4.70	3.09E-36	8.03	5.34E-02	-4.77	3.81	0.35	2.43						
**DVG**	*Helichrysum stoechas*	72.77680512	lm5	0.92	0.90	7.00E-09	0.79	0.54	SW	0.08	15.69	2.19E-10	8.02	8.76E-04	-21.52	8.33	0.61	5.35	0.45	-0.02	0.02	-0.19	0.02	0.07

Phenophase	Taxon	Intercept	Model	R^2	Ad. R^2	*p* value	Normality_p	BP Test	Test Used	p_value_Tmed.Y	slope_Tmed.Y	p_value_Tmon	slope_Tmon	p_value_T.MAM	slope_T.MAM	se_Tmed.Y	se_Tmon	se_T.MAM	p_value_Pannual.Y	slope_Pannual.Y	p_value_P.MAM	slope_P.MAM	se_Pannual.Y	se_P.MAM
**DVG**	*Calicotome villosa*	236.615229	lm2	0.78	0.76	1.52E-09	0.83	0.52	SW	0.00	-16.18	1.05E-09	8.86			3.40	0.97							
**DVG**	*Calluna vulgaris*	551.8559967	lm2	0.58	0.51	5.54E-03	0.06	0.12	SW	0.62	-7.43	1.58E-03	-8.73			14.55	2.15							
**DVG**	*Viburnum tinus*	182.6189371	lm3	0.96	0.95	3.50E-08	0.43	0.29	SW	0.98	-0.13	5.52E-09	8.88	2.59E-03	-14.33	4.19	0.55	3.70						
**DVG**	*Quercus coccifera*	173.2356105	lm2	0.73	0.72	3.12E-13	0.64	0.57	SW	0.00	-9.56	4.45E-14	6.72			2.05	0.62							
**DVG**	*Juniperus turbinata*	145.6873105	lm3	0.88	0.87	8.78E-17	0.07	0.26	SW	0.82	2.07	1.49E-17	9.30	7.09E-04	-14.49	8.93	0.60	3.91						
**DVG**	*Fumana thymifolia*	206.5306175	lm2	0.86	0.84	2.51E-06	0.20	0.46	SW	0.08	-14.10	7.28E-07	8.92			7.43	1.01							
**DVG**	*Cistus ladanifer*	-6.938340619	lm3	0.90	0.89	4.42E-12	0.63	0.90	SW	0.36	7.12	2.64E-13	8.14	6.13E-02	-8.83	7.65	0.57	4.50						
**DVG**	*Rubia peregrina*	108.7592112	lm3	0.77	0.74	2.11E-08	0.69	0.43	SW	0.87	-1.45	1.04E-09	9.84	7.96E-02	-8.53	8.63	1.06	4.67						
**DVG**	*Olea europaea var. sylvestris*	11.22306938	lm3	0.84	0.83	4.39E-18	0.94	0.24	SW	0.34	4.44	1.95E-19	8.10	4.44E-02	-6.53	4.65	0.54	3.16						
**DVG**	*Myrtus communis*	118.1924286	lm2	0.87	0.86	3.44E-13	0.56	0.21	SW	0.00	-7.91	5.82E-14	8.15			2.10	0.59							
**DVG**	*Cistus populifolius*	121.9373498	lm3	0.87	0.85	5.72E-07	0.21	0.87	SW	0.87	1.35	7.19E-08	7.83	1.40E-02	-10.57	8.20	0.81	3.80						

**Phenophase**	**Taxon**	**Intercept**	**Model**	**R^2**	**Ad. R^2**	***p* value**	**Normality_p**	**BP Test**	**Test Used**	**p_value_Tmed.Y**	**slope_Tmed.Y**	**p_value_Tmon**	**slope_Tmon**	**p_value_T.MAM**	**slope_T.MAM**	**se_Tmed.Y**	**se_Tmon**	**se_T.MAM**	**p_value_Pannual.Y**	**slope_Pannual.Y**	**p_value_P.MAM**	**slope_P.MAM**	**se_Pannual.Y**	**se_P.MAM**
**DVG**	*Quercus rotundifolia*	180.7882256	lm2	0.84	0.83	3.72E-24	0.96	0.28	KS	0.00	-11.00	3.66E-24	7.45			1.75	0.44							
**DVG**	*Quercus faginea*	109.7039814	lm4	0.83	0.81	2.66E-13	0.52	0.17	SW	0.77	-1.93	1.39E-12	7.00	1.81E-01	-6.06	6.51	0.66	4.44	0.09	0.03			0.01	
**DVG**	*Quercus suber*	116.9690102	lm2	0.75	0.72	4.45E-06	0.23	0.49	SW	0.23	-5.70	9.41E-07	6.60			4.56	0.91							
**DVG**	*Adenocarpus telonensis*	54.08401023	lm2	0.57	0.51	2.64E-03	0.04	0.49	SW	0.91	1.15	1.37E-03	3.30			10.44	0.83							
**DVG**	*Thymus mastichina*	232.7699499	lm2	0.87	0.86	6.52E-18	0.46	0.42	SW	0.00	-13.55	4.96E-18	7.13			1.72	0.47							
**DVG**	*Erinacea anthyllis*	357.0828357	lm2	0.75	0.71	2.53E-04	0.77	0.14	SW	0.14	-19.08	7.89E-05	5.72			11.98	0.98							
**DVG**	*Prunus mahaleb*	144.7959144	lm2	0.82	0.79	3.72E-05	0.47	0.25	SW	0.19	-5.32	1.00E-05	4.81			3.87	0.66							
**DVG**	*Sideritis incana*	138.375058	lm3	0.78	0.75	3.11E-06	0.02	0.30	SW	0.34	4.42	3.16E-07	6.32	3.96E-03	-12.53	4.50	0.80	3.80						
**DVG**	*Acer granatense*	177.5398484	lm2	0.88	0.87	1.13E-08	0.61	0.31	SW	0.02	-9.91	2.33E-09	6.86			3.67	0.60							
**DVG**	*Rhamnus infectoria*	126.68686	lm2	0.80	0.77	2.94E-06	0.07	0.78	SW	0.05	-5.25	7.90E-07	5.61			2.51	0.72							
**DVG**	*Lavandula lanata*	159.1032232	lm2	0.42	0.35	9.36E-03	0.73	0.54	SW	0.39	-5.24	3.22E-03	4.25			6.00	1.24							

Phenophase	Taxon	Intercept	Model	R²	Ad. R²	*p* value	Normality_p	BP Test	Test Used	p_value_Tmed.Y	slope_Tmed.Y	p_value_Tmon	slope_Tmon	p_value_T.MAM	slope_T.MAM	se_Tmed.Y	se_Tmon	se_T.MAM	p_value_Pannual.Y	slope_Pannual.Y	p_value_P.MAM	slope_P.MAM	se_Pannual.Y	se_P.MAM
**DVG**	*Sideritis glacialis*	403.6673672	lm1	0.44	0.40	6.84E-03	0.37	0.27	SW	0.01	-12.86					4.01								

***In the following section, each taxon with >15 records in a single phenophase, has a figure which represents the multiple linear model trends with the final combination of climate variables for the model with best AIC (MLM), and its corresponding model diagnostic analysis (Diagnostics). FBF: preflowering, F: flowering, FS: fruiting, DVG: growth.**

1.1. MLM - FS - Acer granatense

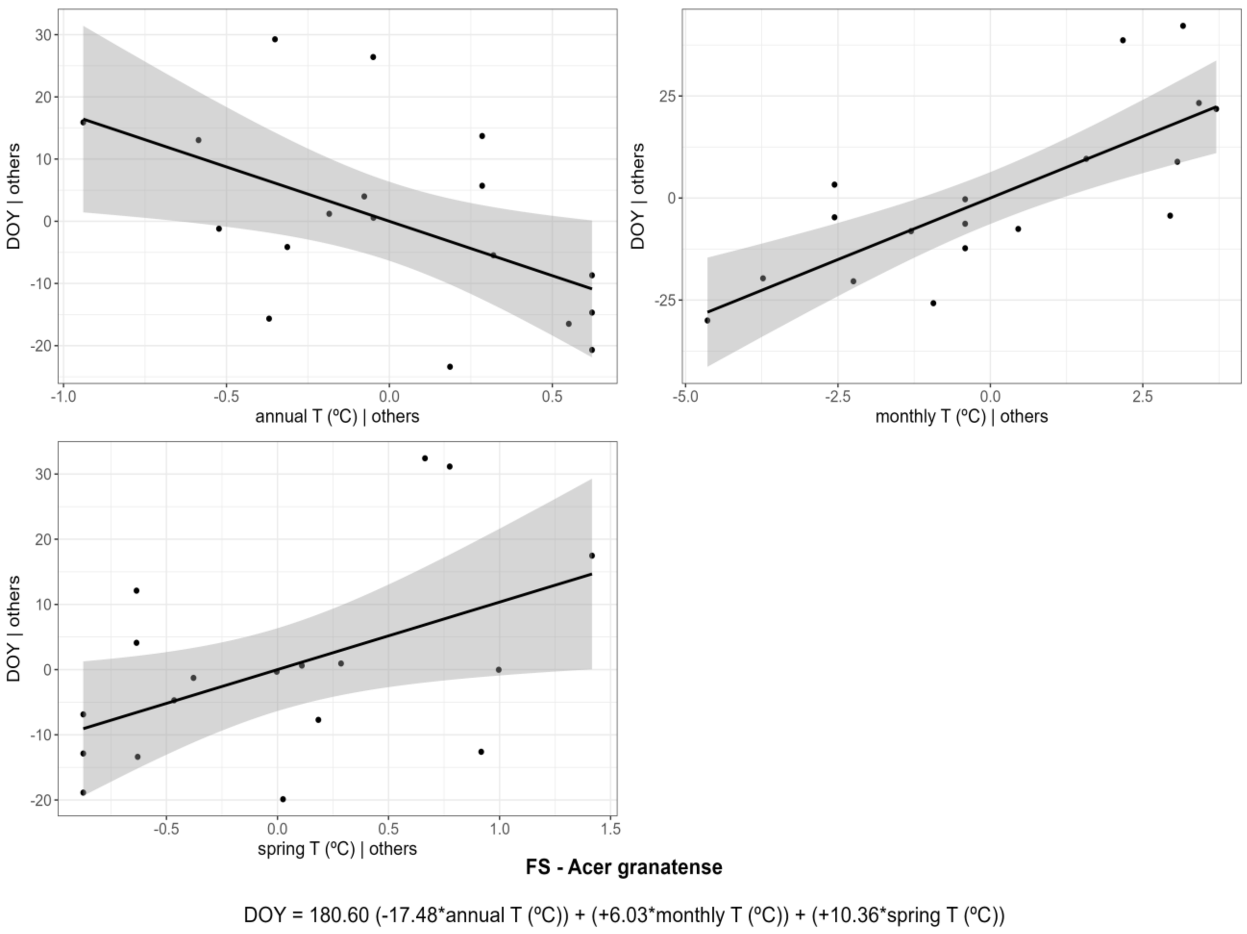

1.1.1. Diagnostics - MLM - FS - Acer granatense

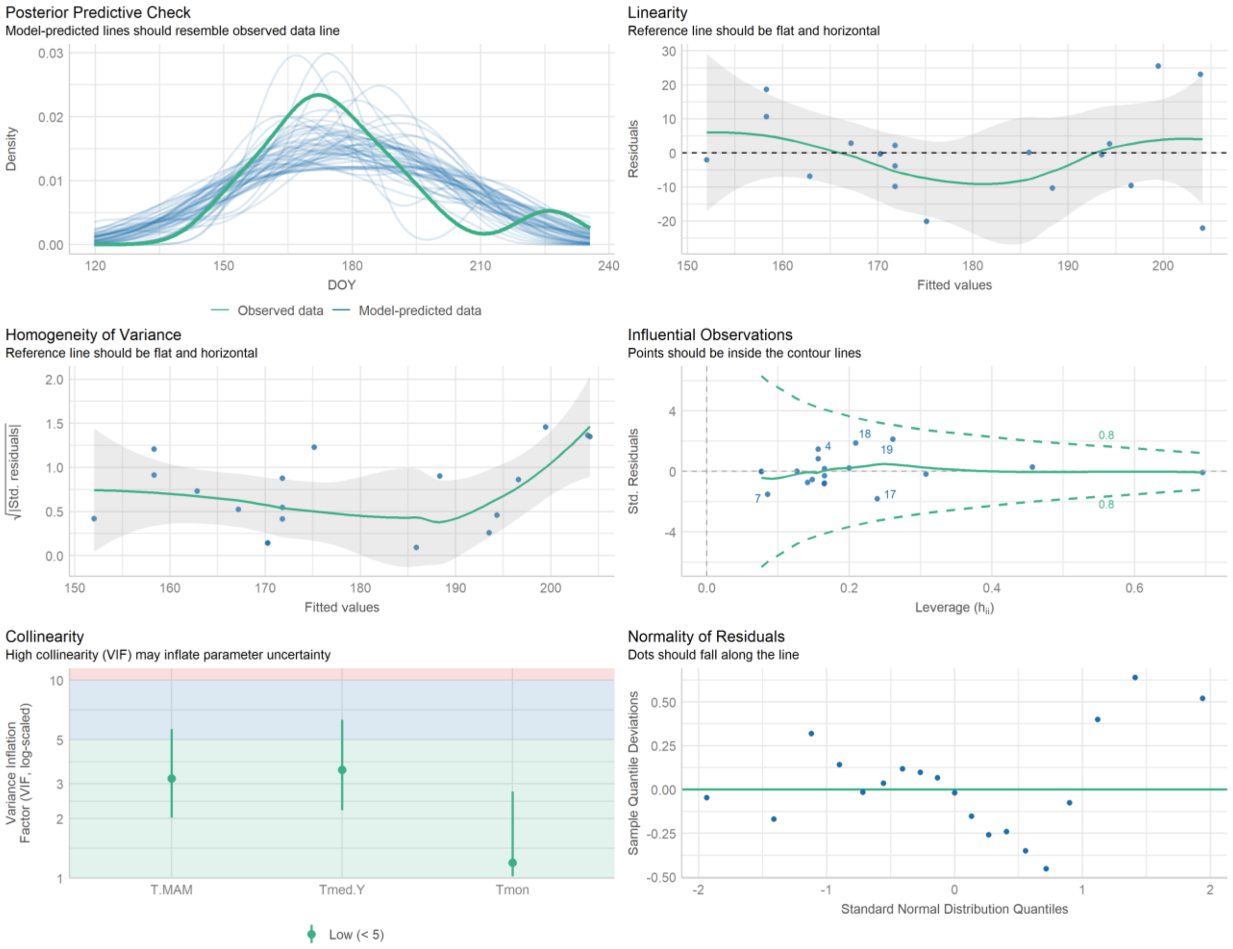

1.2. MLM - DVG - Acer granatense

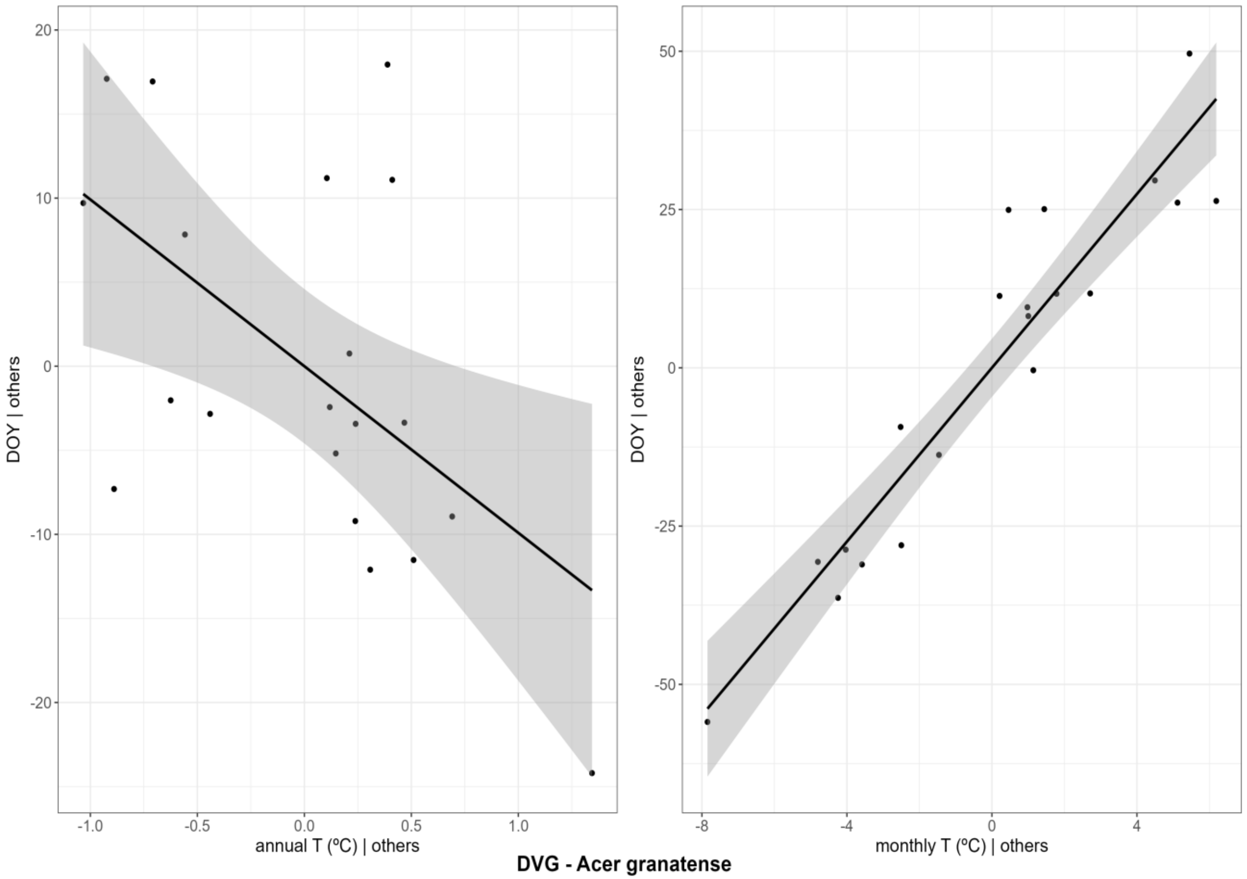

DOY = 177.54 (-9.91*annual T (ºC)) + (+6.86*monthly T (ºC))

1.2.1. Diagnostics - MLM - DVG - Acer granatense

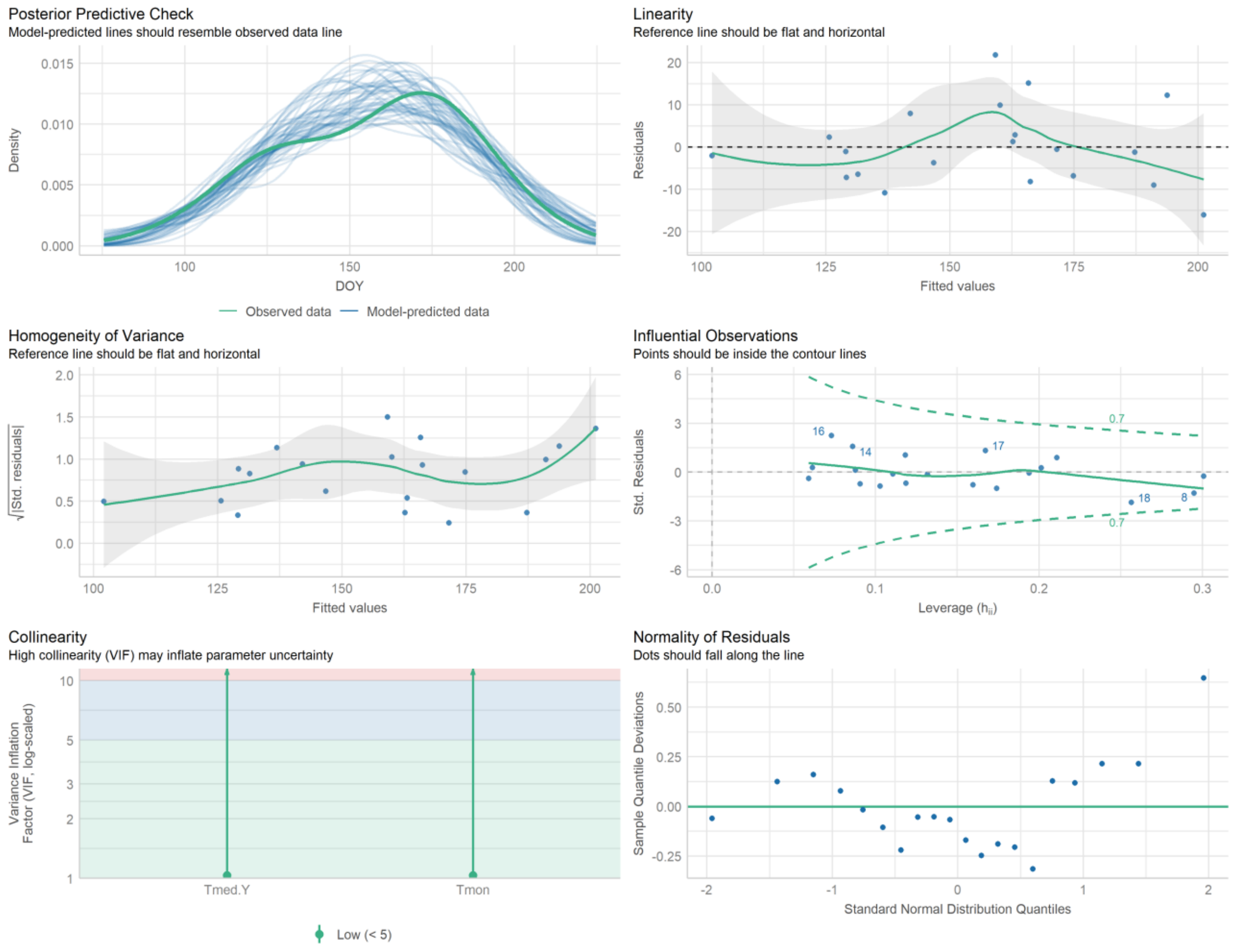

1.3. MLM - F - Adenocarpus telonensis

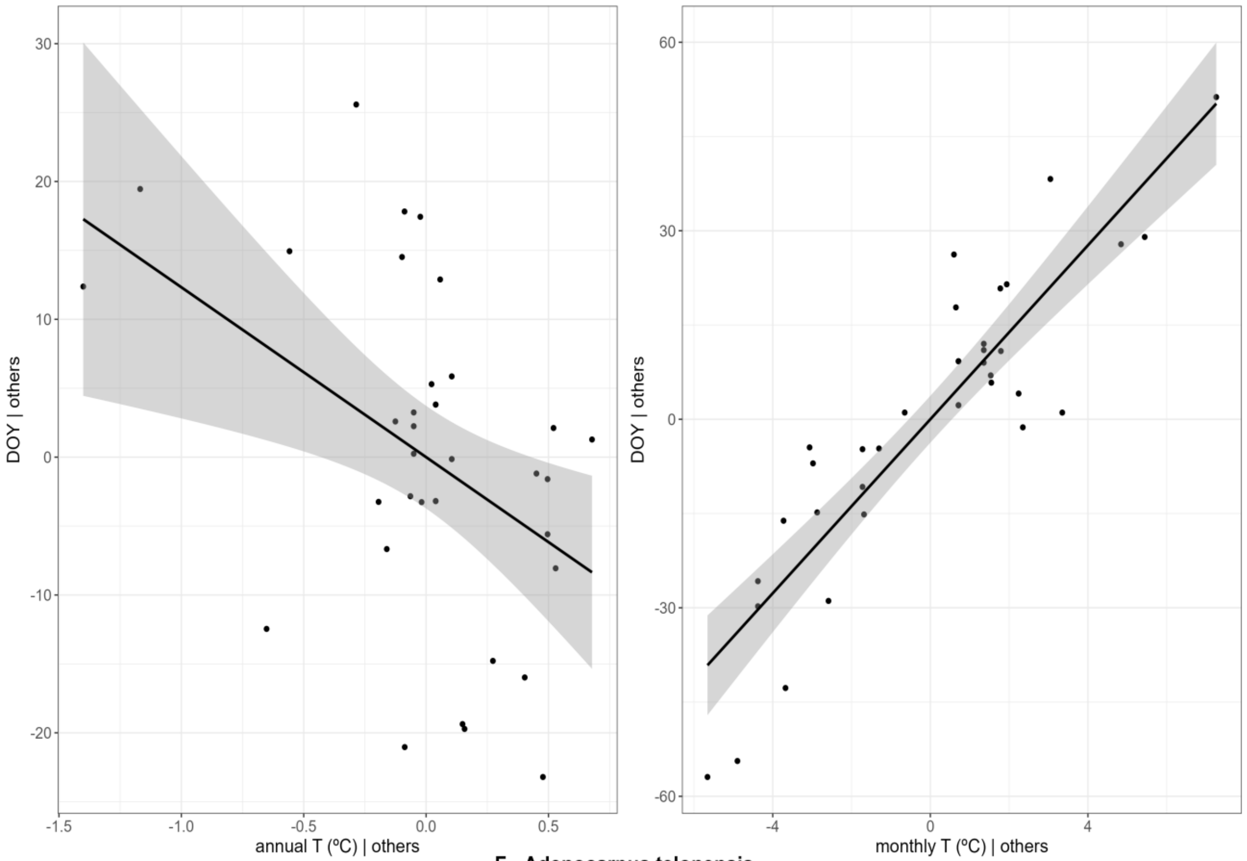

DOY = 215.55 (-12.33*annual T (ºC)) + (+6.92*monthly T (ºC))

1.3.1. Diagnostics - MLM - F - Adenocarpus telonensis

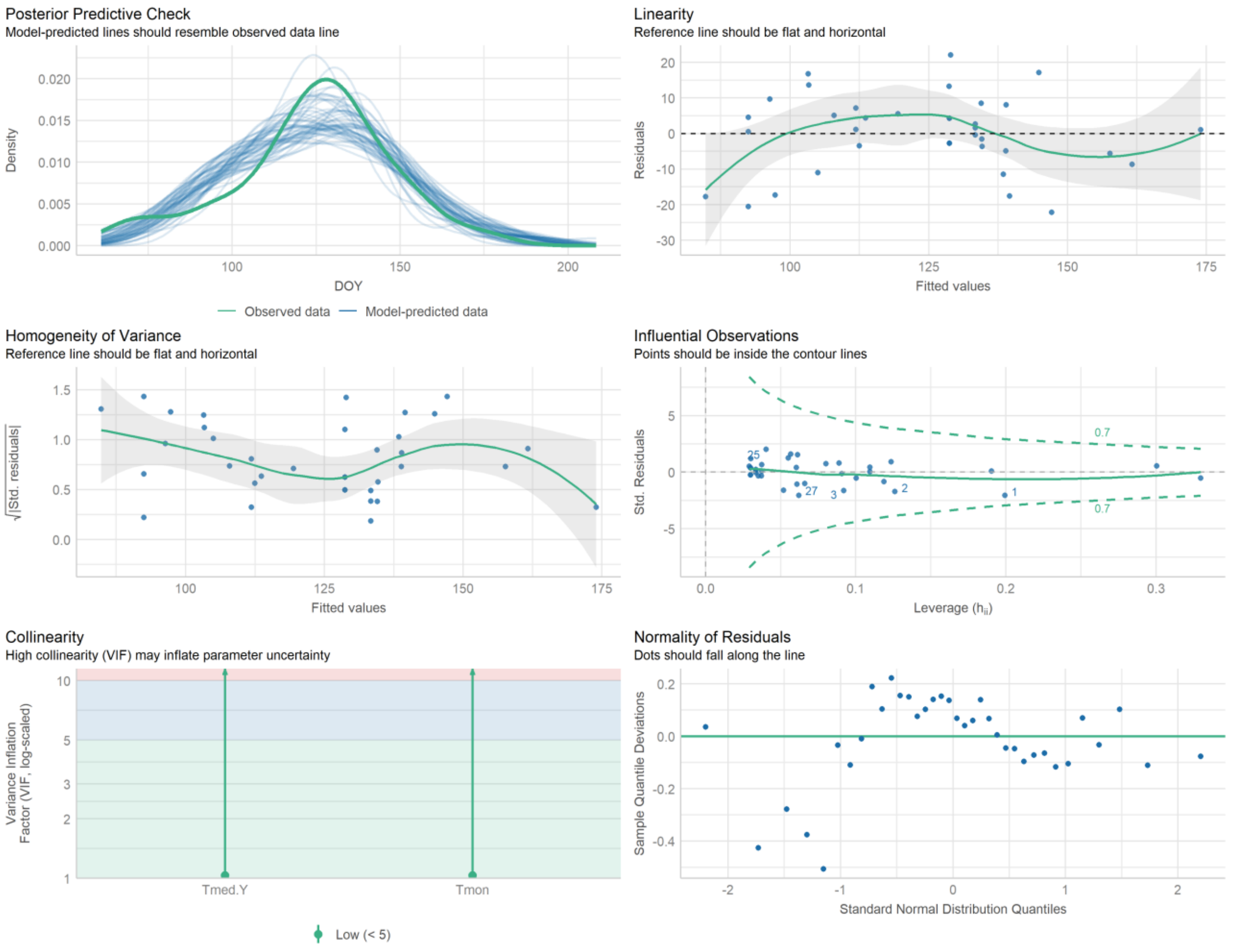

1.4. MLM - DVG - Adenocarpus telonensis

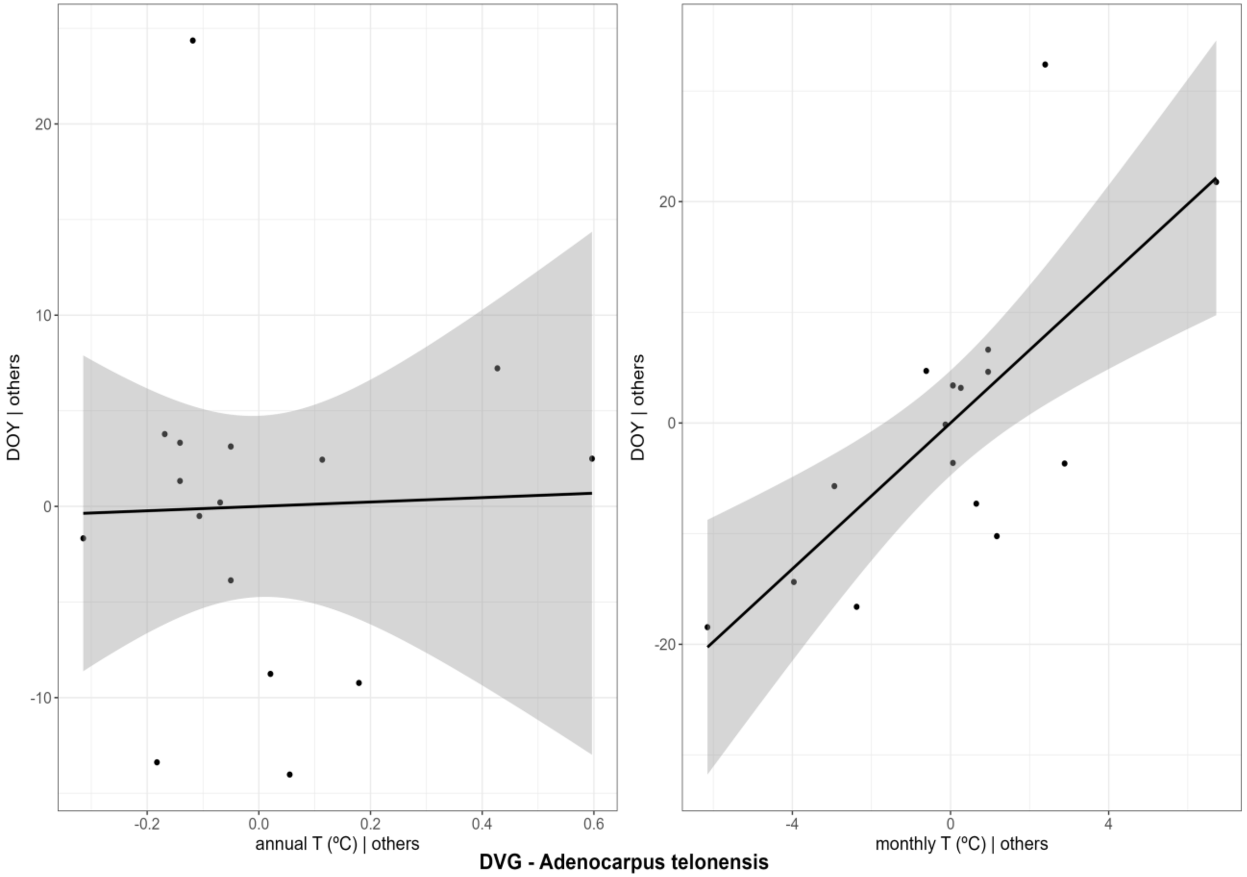

DOY = 54.08 (+1.15*annual T (ºC)) + (+3.30*monthly T (ºC))

1.4.1. Diagnostics - MLM - DVG - Adenocarpus telonensis

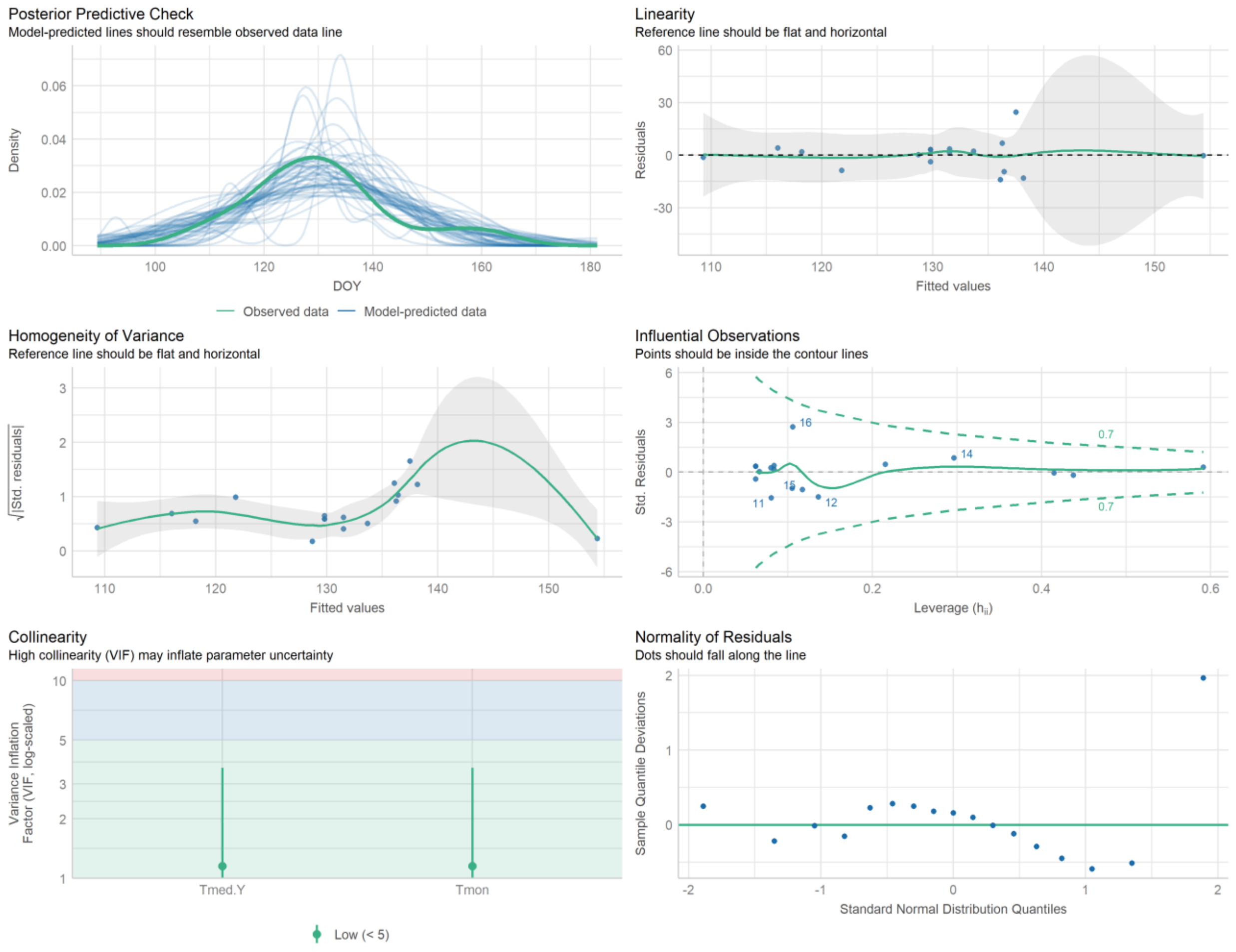

1.5. MLM - FBF - Alyssum serpyllifolium subsp. malacitanum

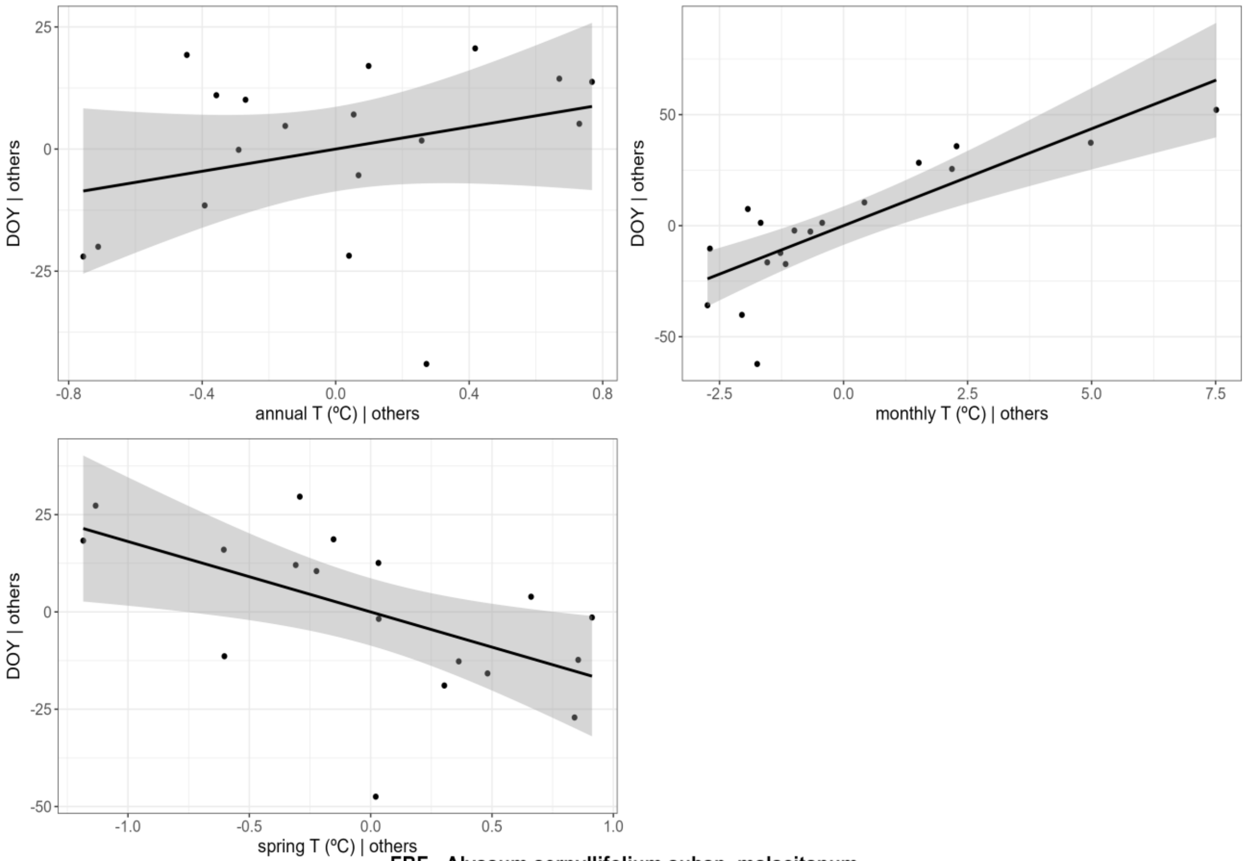

FBF - Alyssum serpyllifolium subsp. malacitanum

DOY = 53.54 (+11.36*annual T (ºC)) + (+8.72*monthly T (ºC)) + (-18.09*spring T (ºC))

1.5.1. Diagnostics - MLM - FBF - Alyssum serpyllifolium subsp. malacitanum

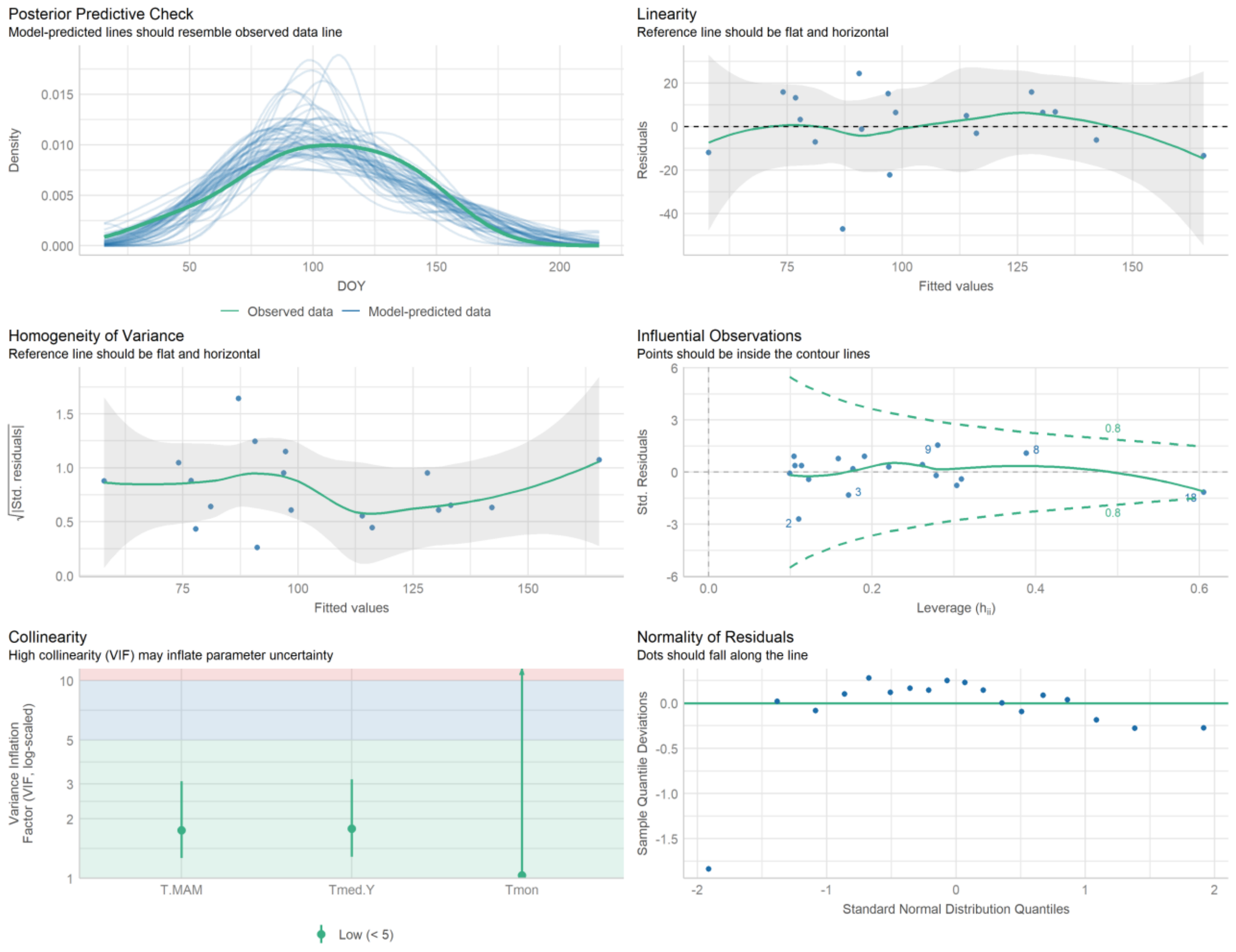

1.6. MLM - F - Alyssum serpyllifolium subsp. malacitanum

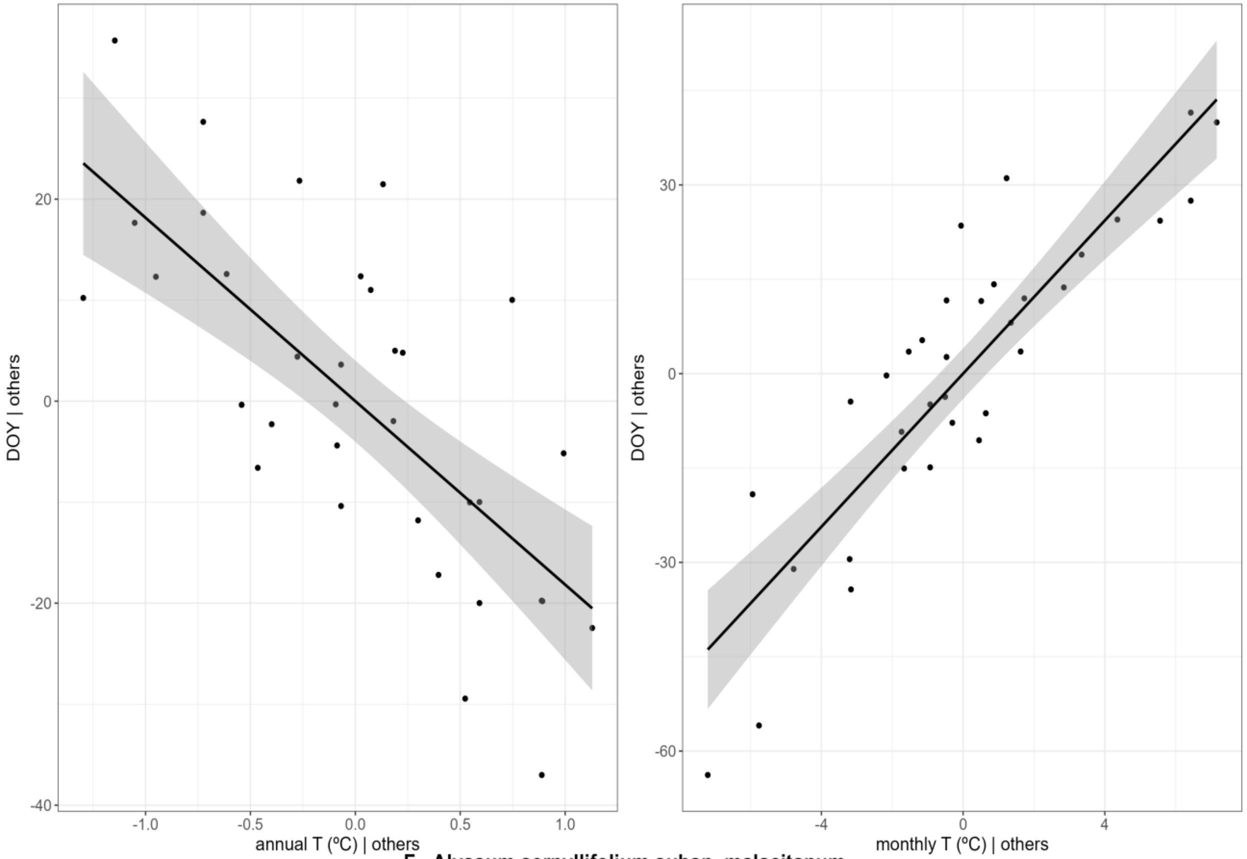

F - Alyssum serpyllifolium subsp. malacitanum

DOY = 325.40 (-18.16*annual T (ºC)) + (+6.09*monthly T (ºC))

1.6.1. Diagnostics - MLM - F - Alyssum serpyllifolium subsp. malacitanum

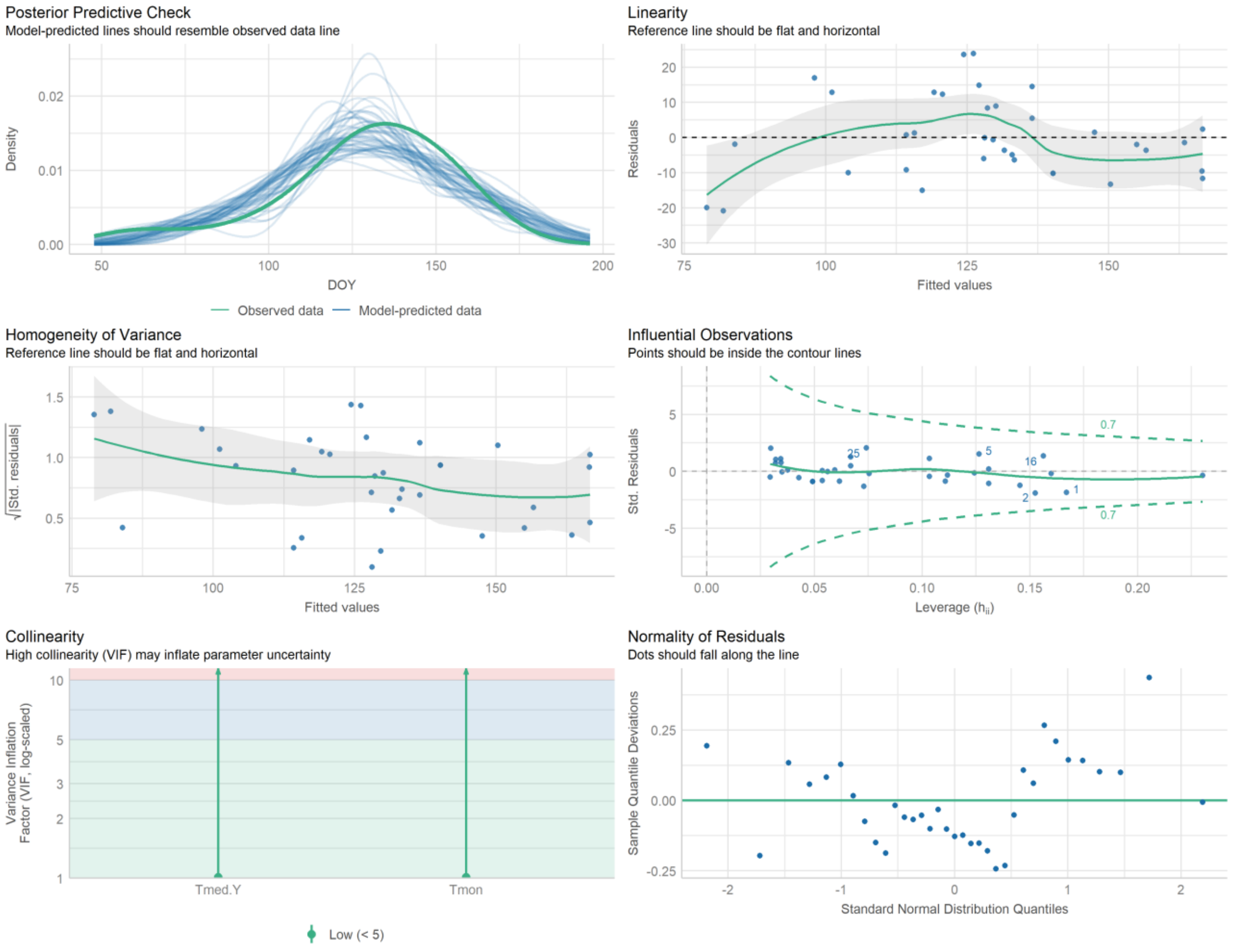

1.7. MLM - F - Arbutus unedo

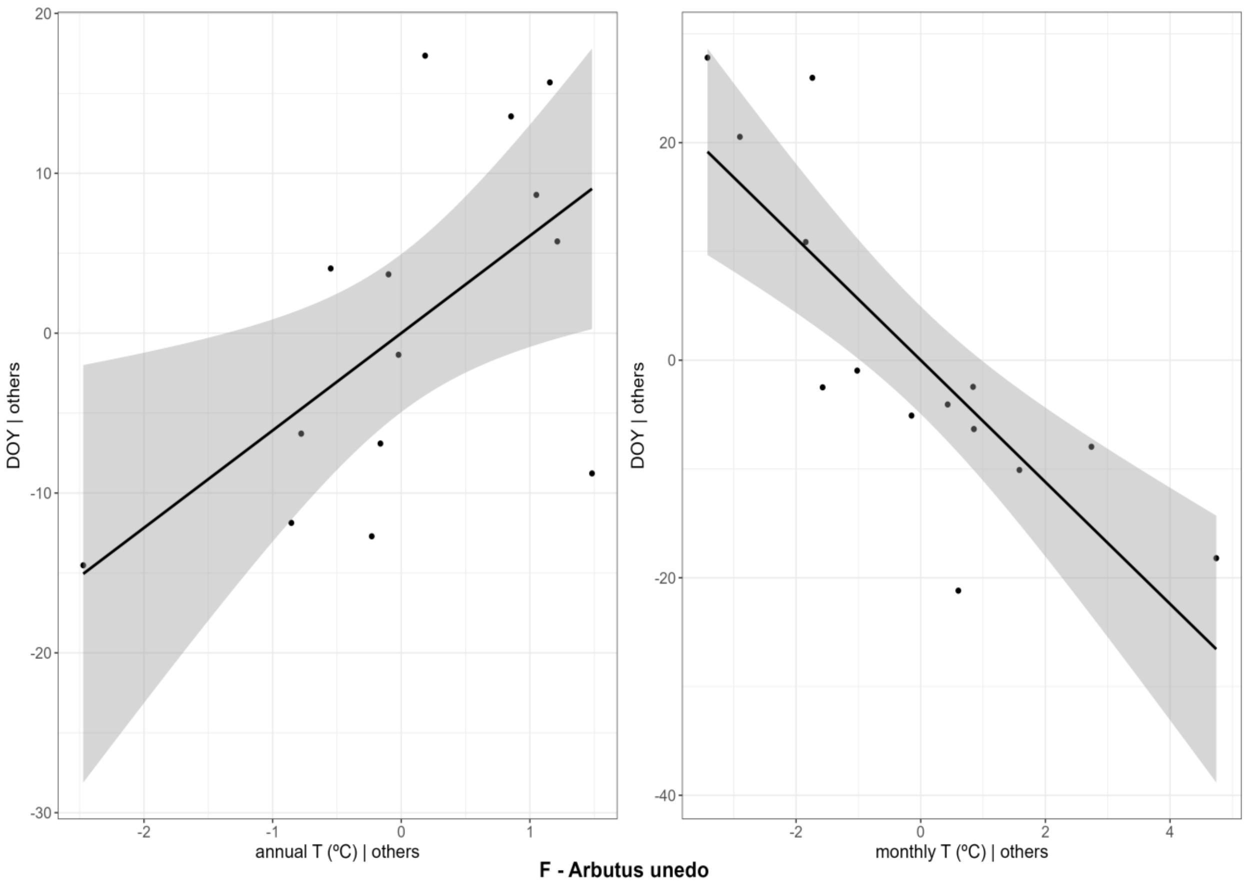

DOY = 292.88 (+6.09*annual T (ºC)) + (-5.60*monthly T (ºC))

1.7.1. Diagnostics - MLM - F - Arbutus unedo

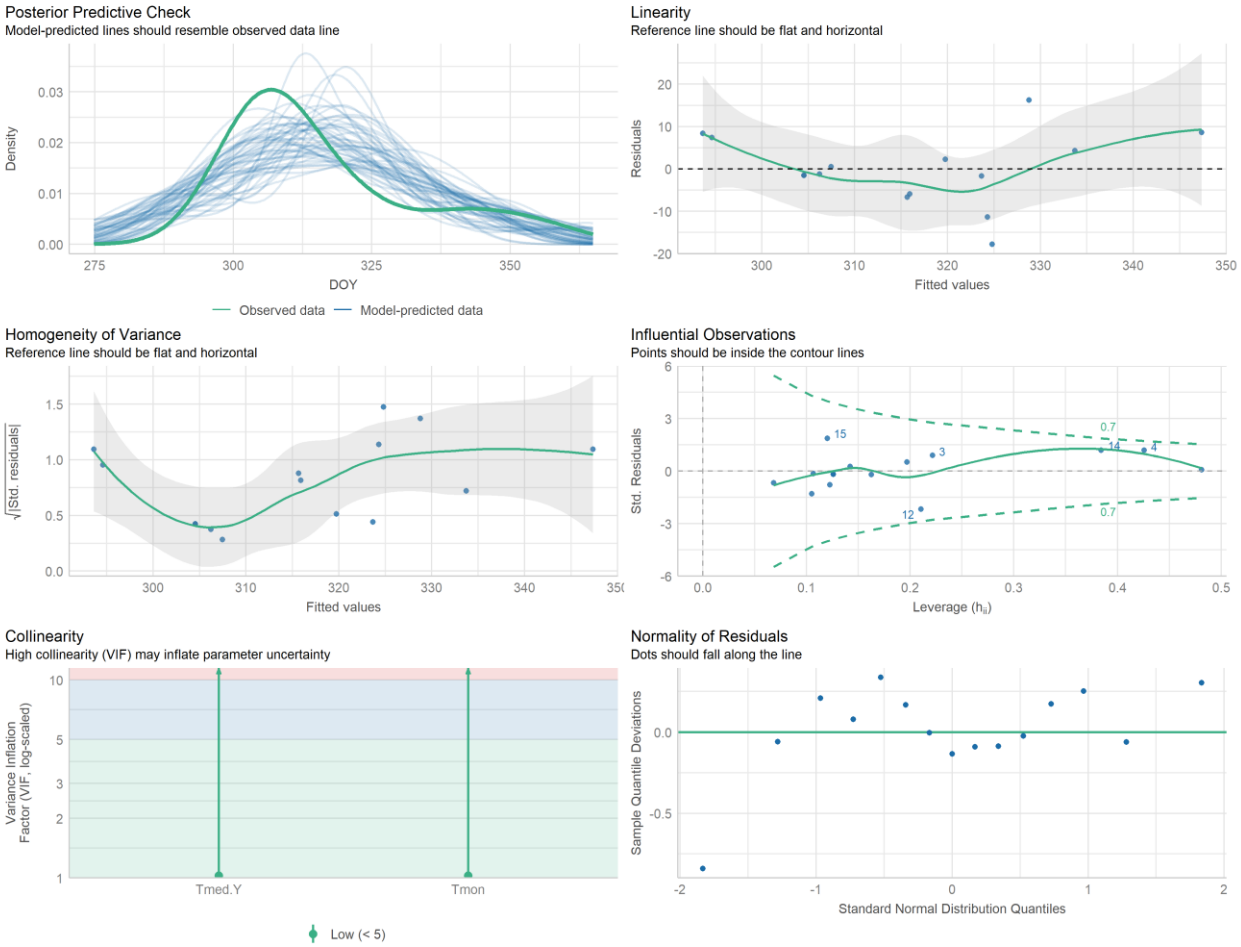

1.8. MLM - F - Calicotome villosa

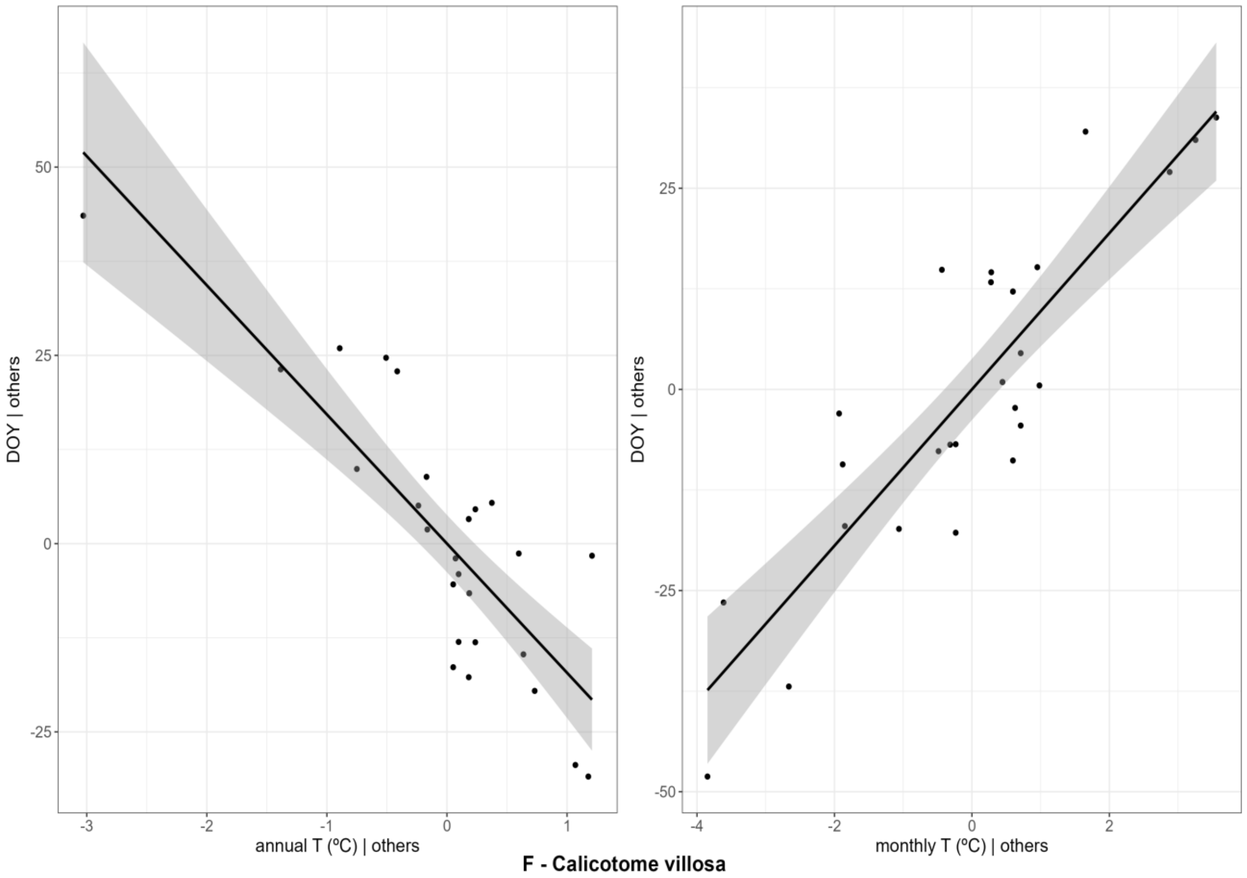

DOY = 239.10 (-17.15*annual T (ºC)) + (+9.71*monthly T (ºC))

1.8.1. Diagnostics - MLM - F - Calicotome villosa

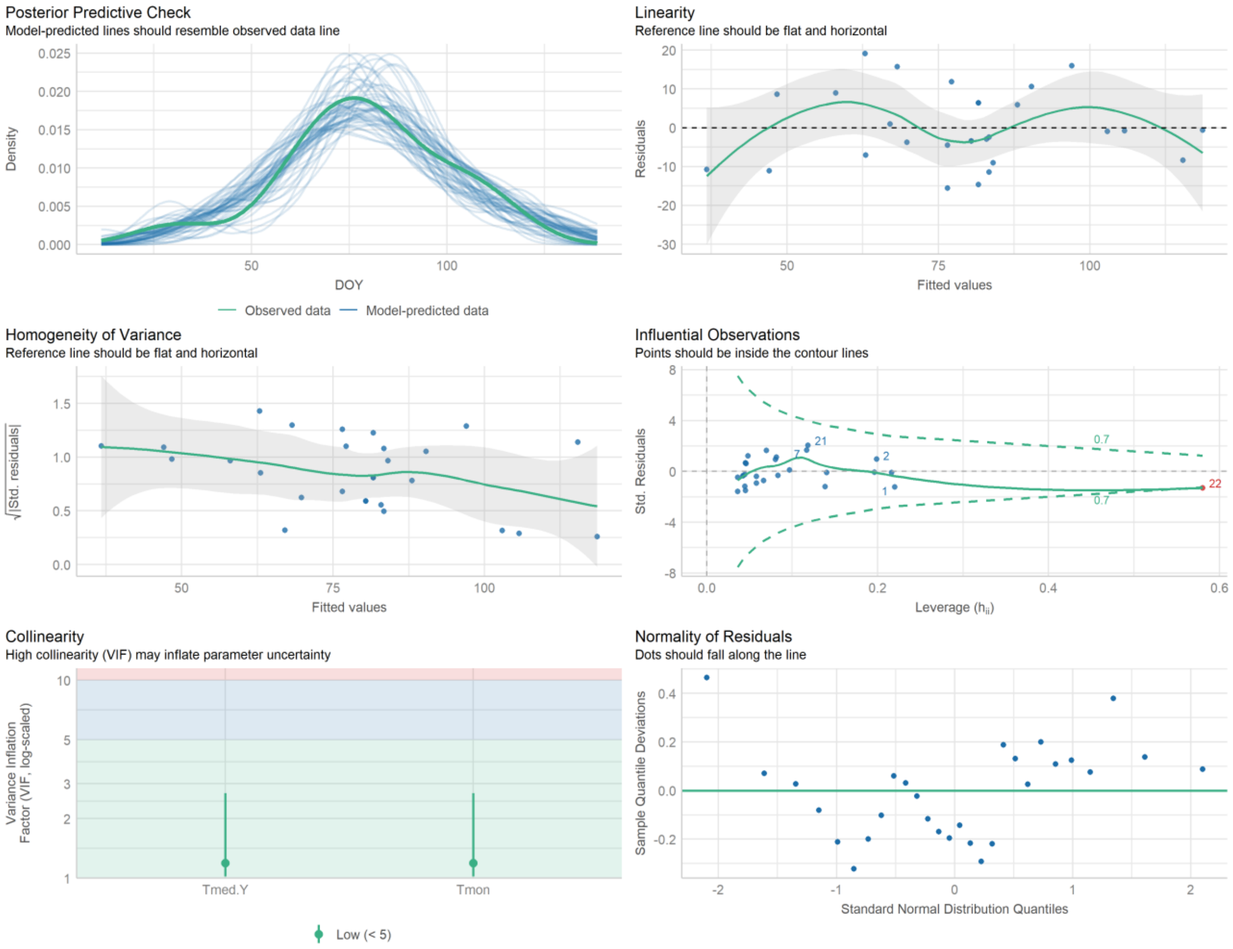

1.9. MLM - DVG - Calicotome villosa

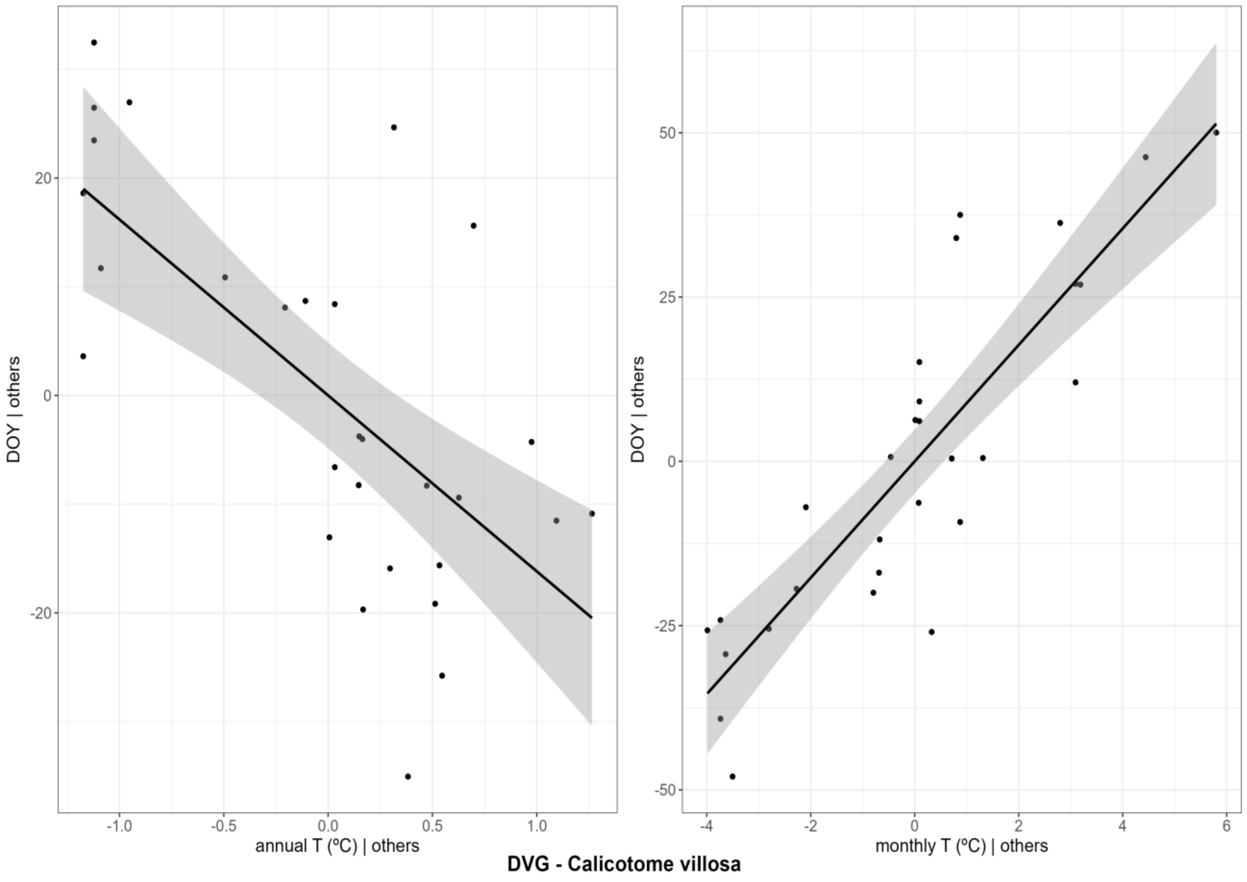

DOY = 236.62 (-16.18*annual T (ºC)) + (+8.86*monthly T (ºC))

1.9.1. Diagnostics - MLM - DVG - Calicotome villosa

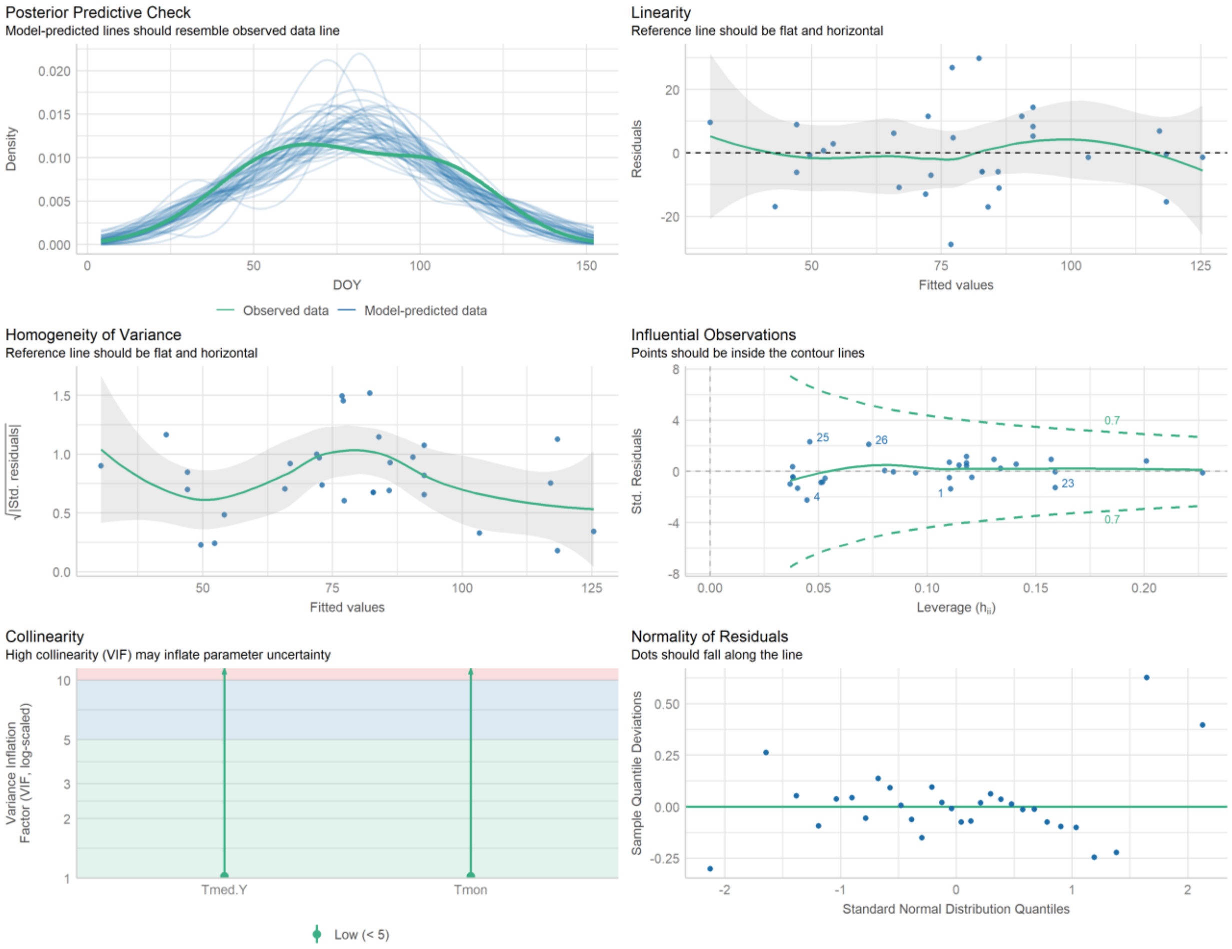

1.10. MLM - F - Calluna vulgaris

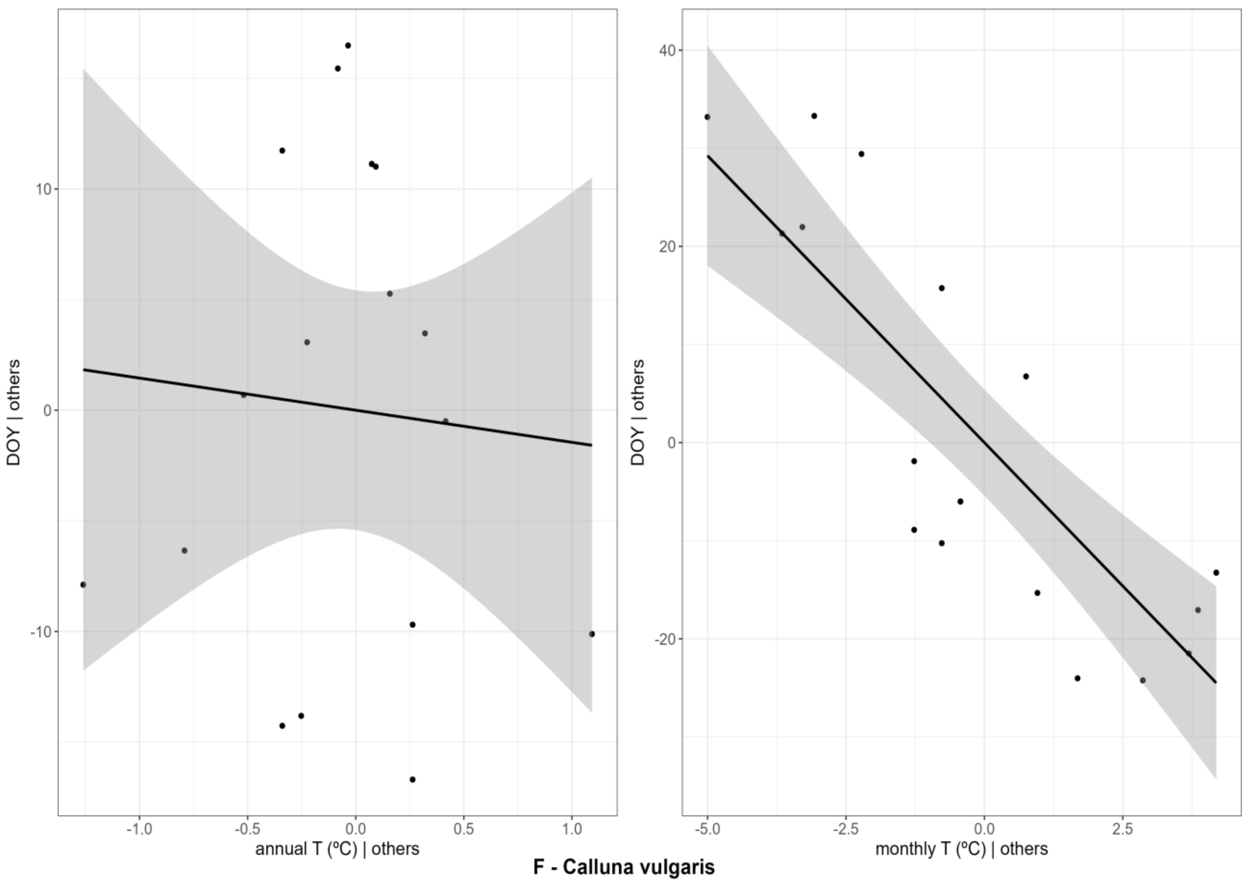

DOY = 420.20 (-1.45*annual T (ºC)) + (-5.85*monthly T (ºC))

1.10.1. Diagnostics - MLM - F - Calluna vulgaris

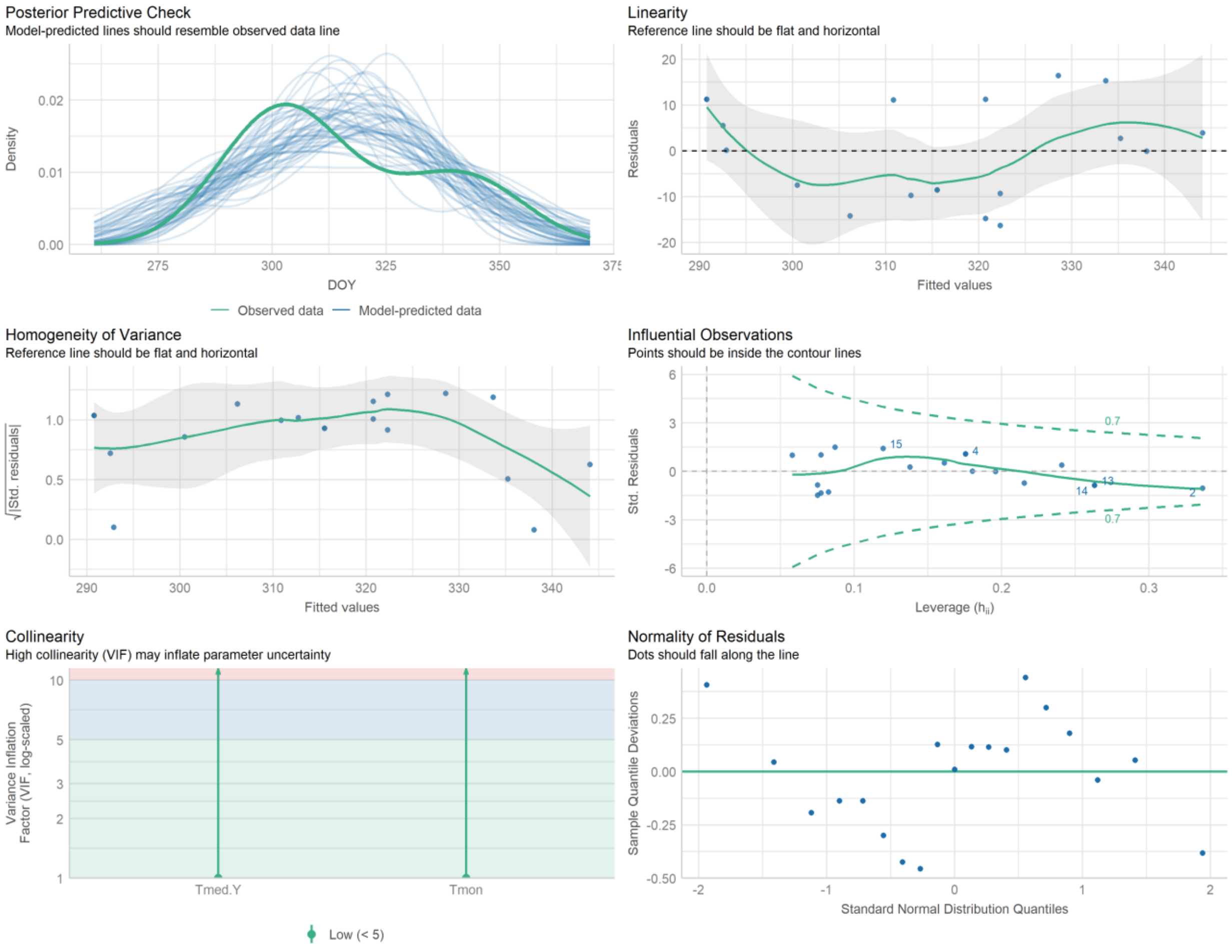

1.11. MLM - DVG - Calluna vulgaris

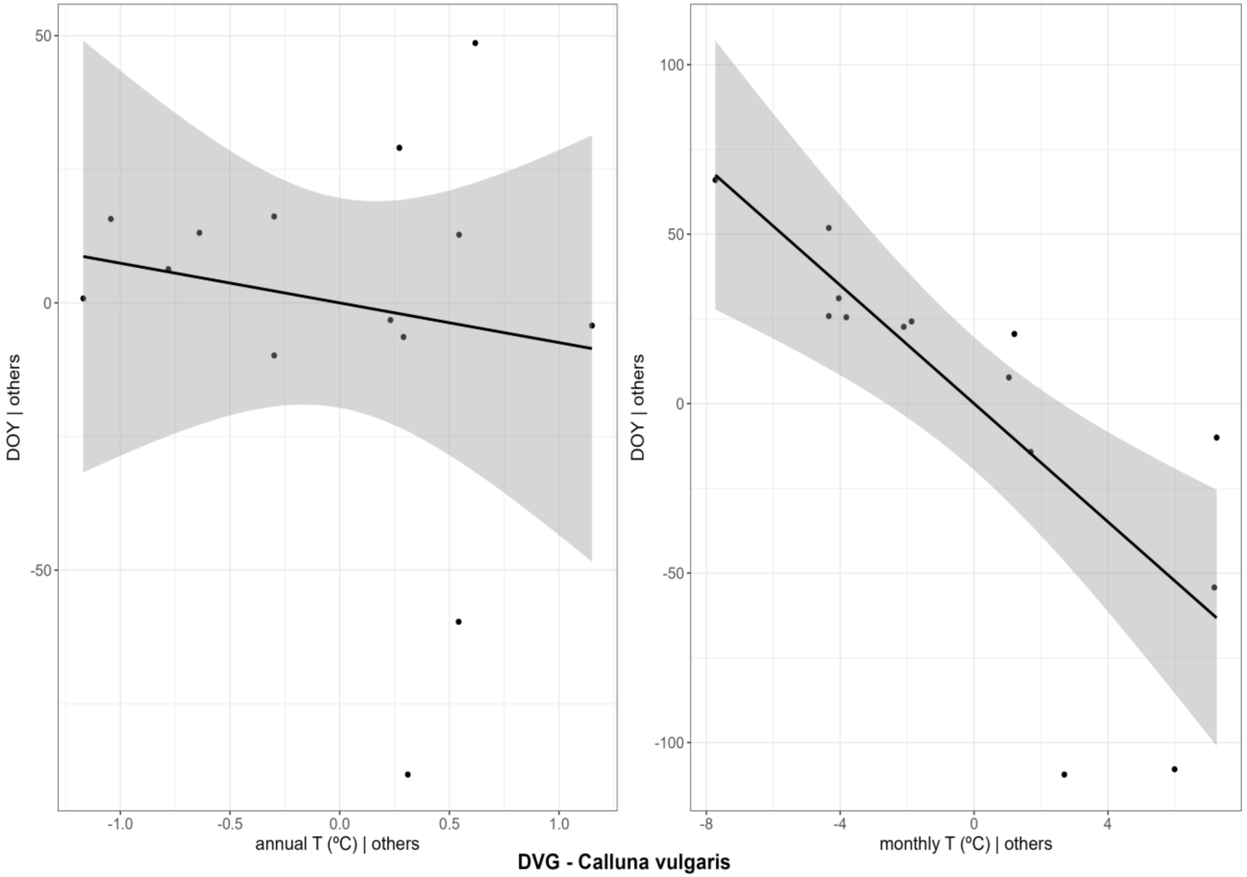

DOY = 551.86 (-7.43*annual T (ºC)) + (-8.73*monthly T (ºC))

1.11.1. Diagnostics - MLM - DVG - Calluna vulgaris

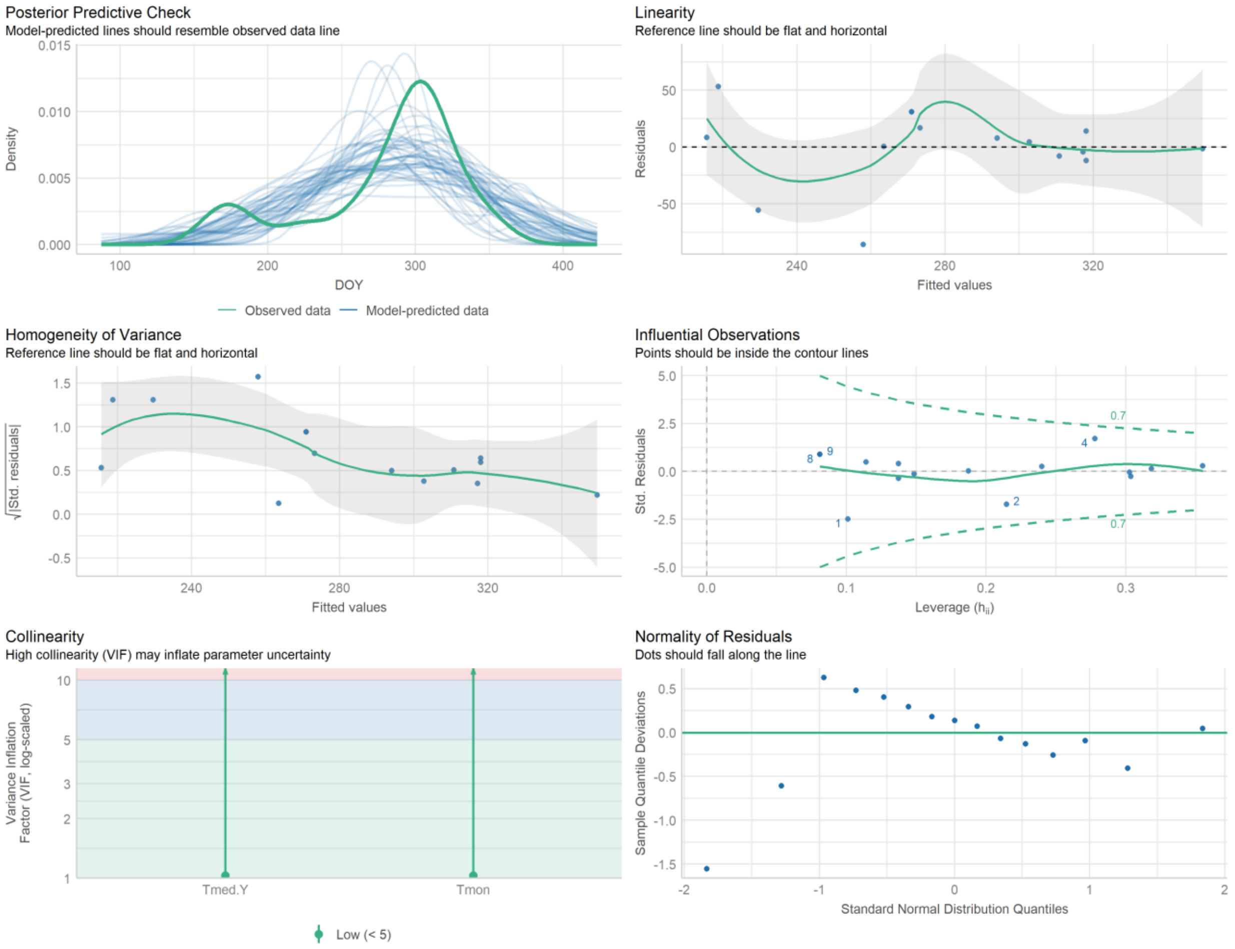

1.12. MLM - F - Centaurea carratracensis

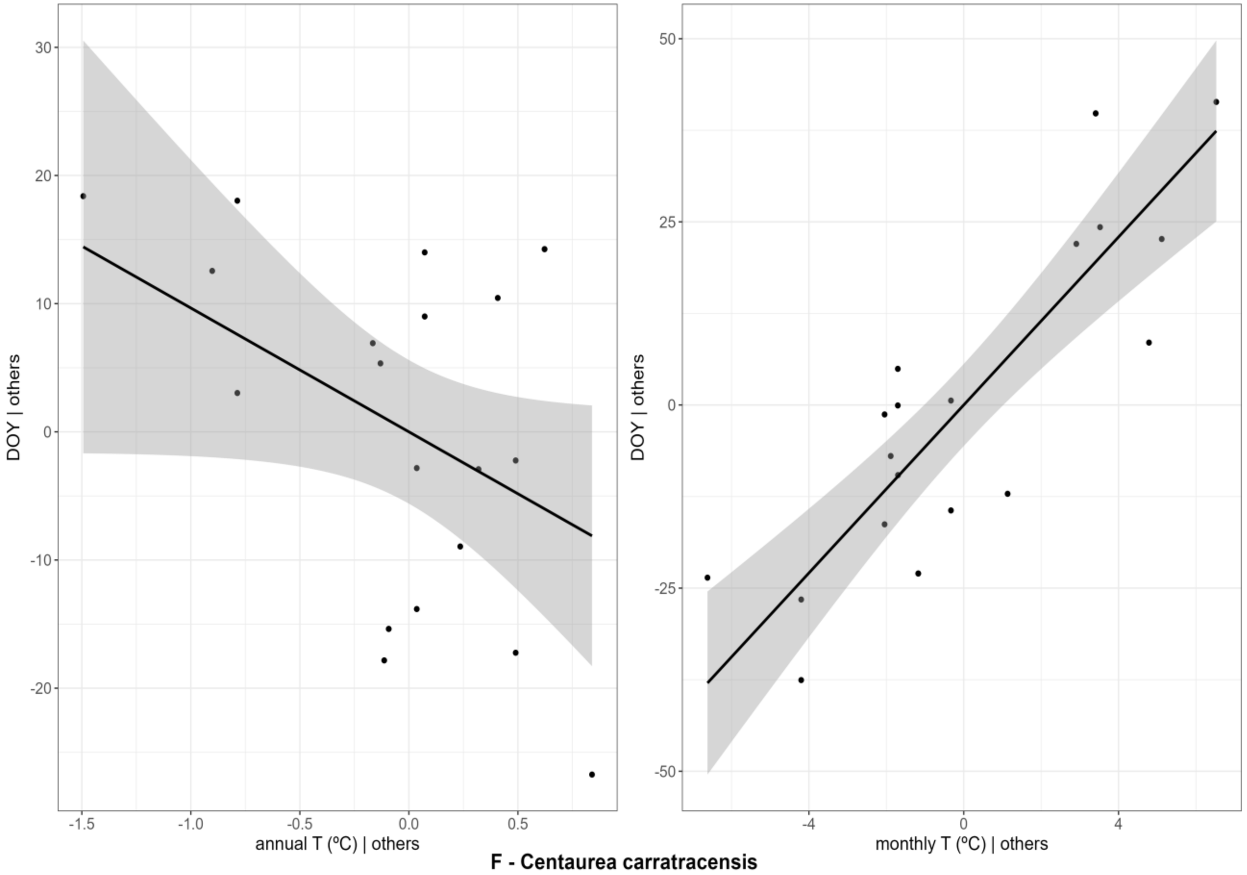

DOY = 198.21 (-9.66*annual T (ºC)) + (+5.73*monthly T (ºC))

1.12.1. Diagnostics - MLM - F - Centaurea carratracensis

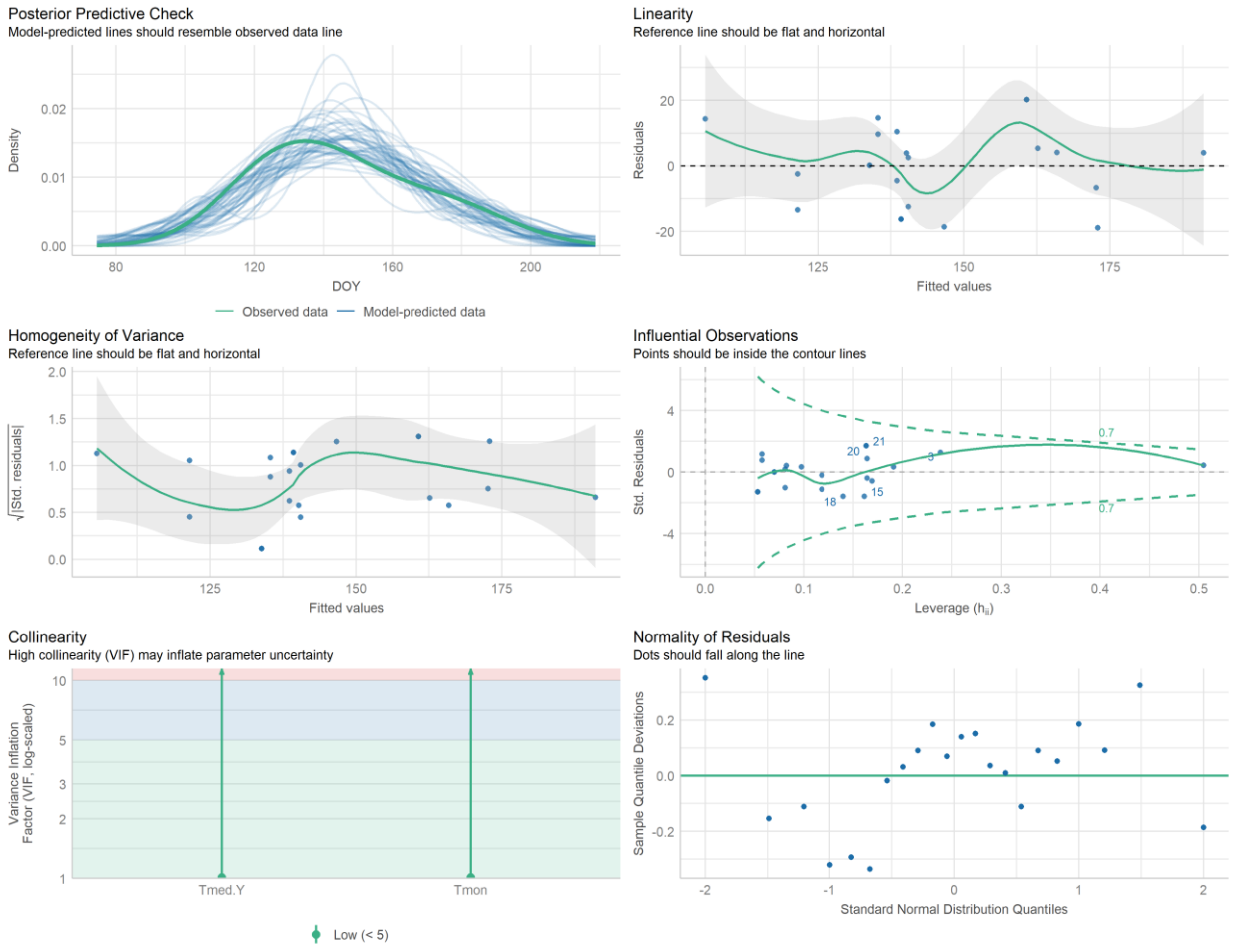

1.13. MLM - FBF - Centaurea prolongoi

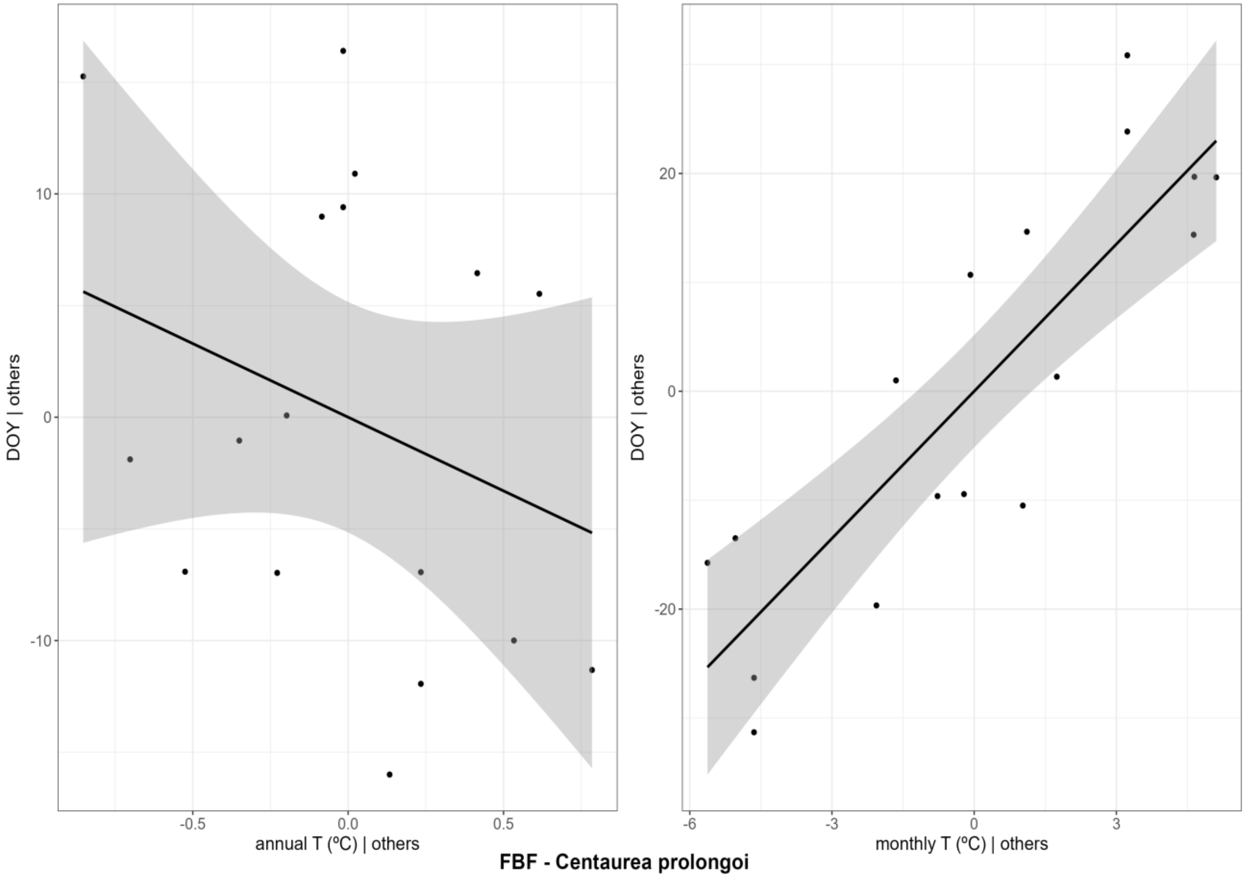

DOY = 163.45 (-6.60*annual T (ºC)) + (+4.50*monthly T (ºC))

1.13.1. Diagnostics - MLM - FBF - Centaurea prolongoi

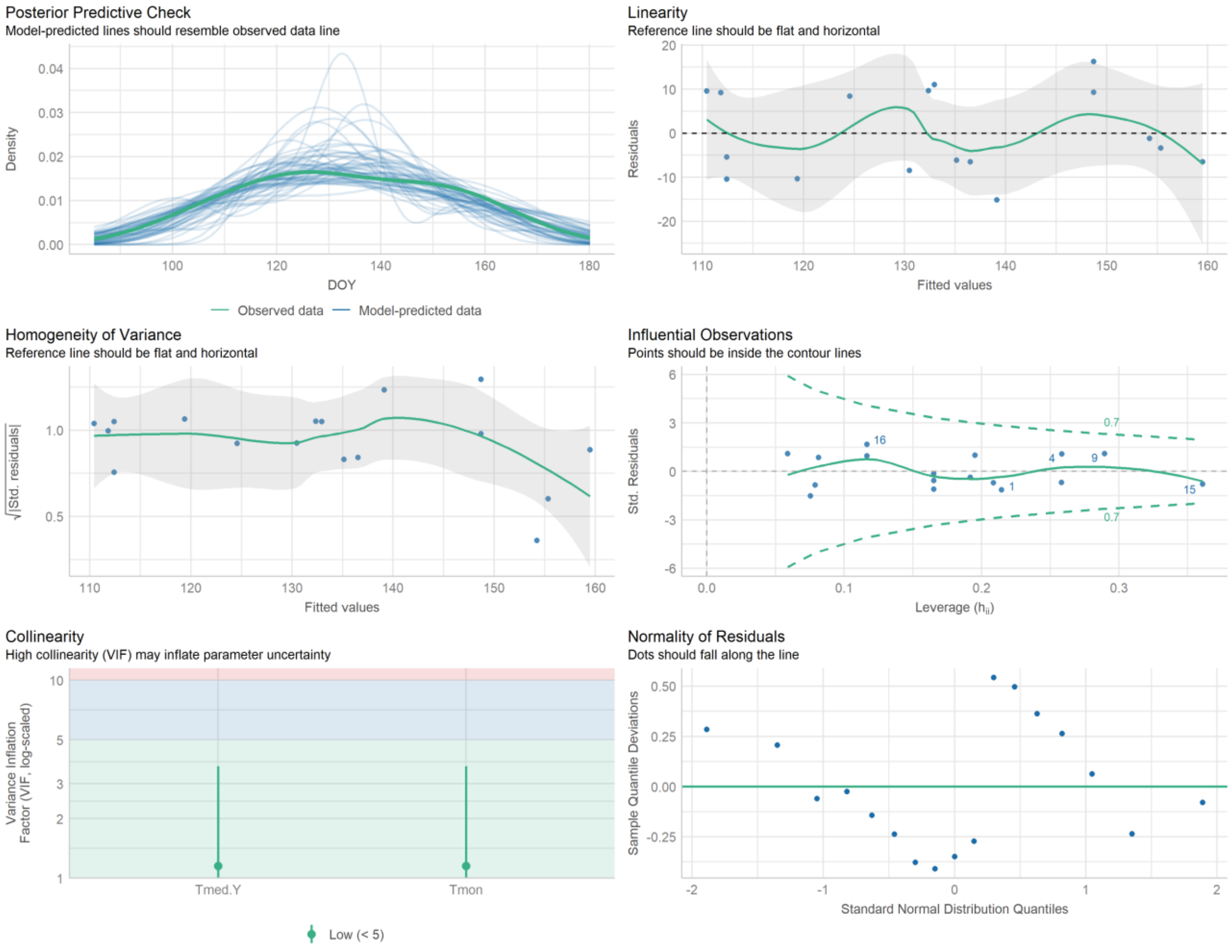

1.14. MLM - F - Centaurea prolongoi

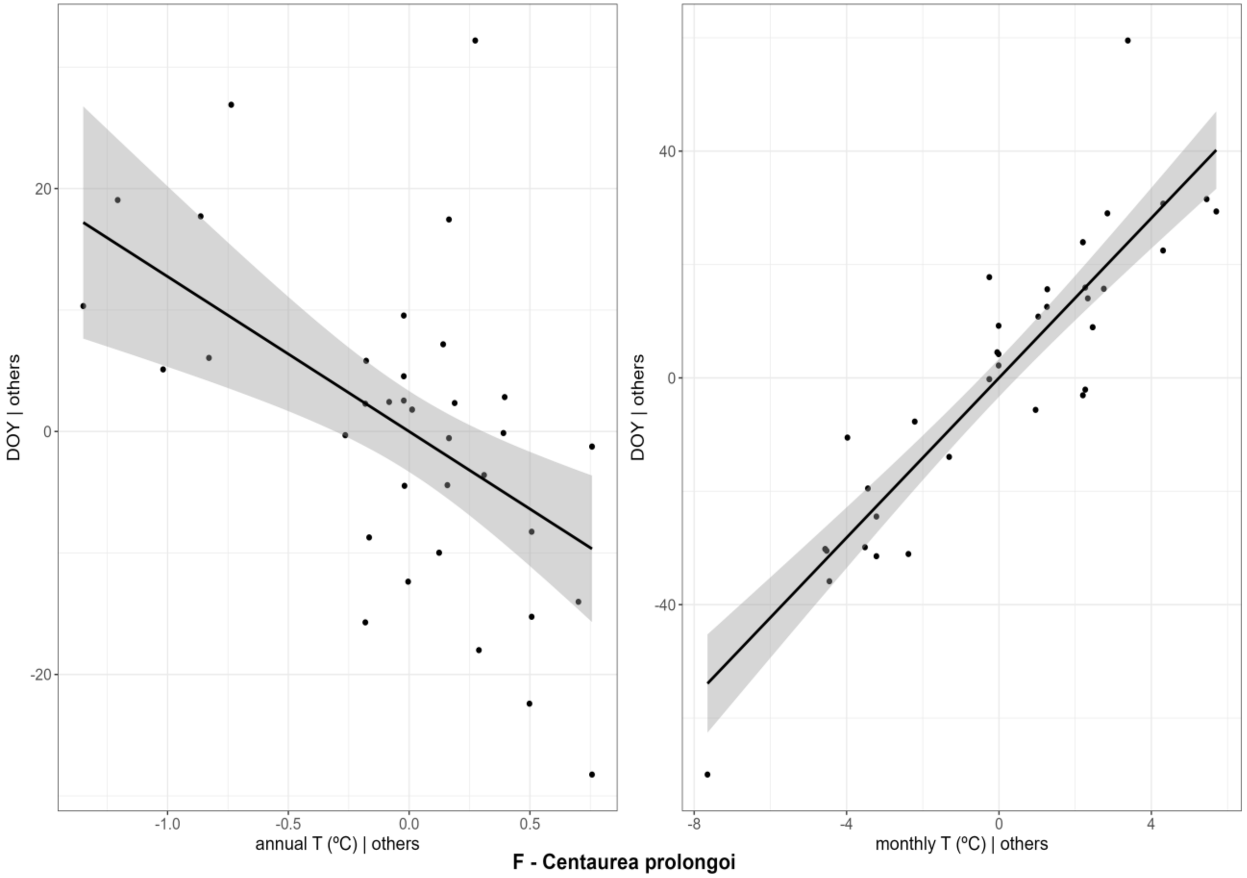

DOY = 220.76 (-12.75*annual T (ºC)) + (+7.05*monthly T (ºC))

1.14.1. Diagnostics - MLM - F - Centaurea prolongoi

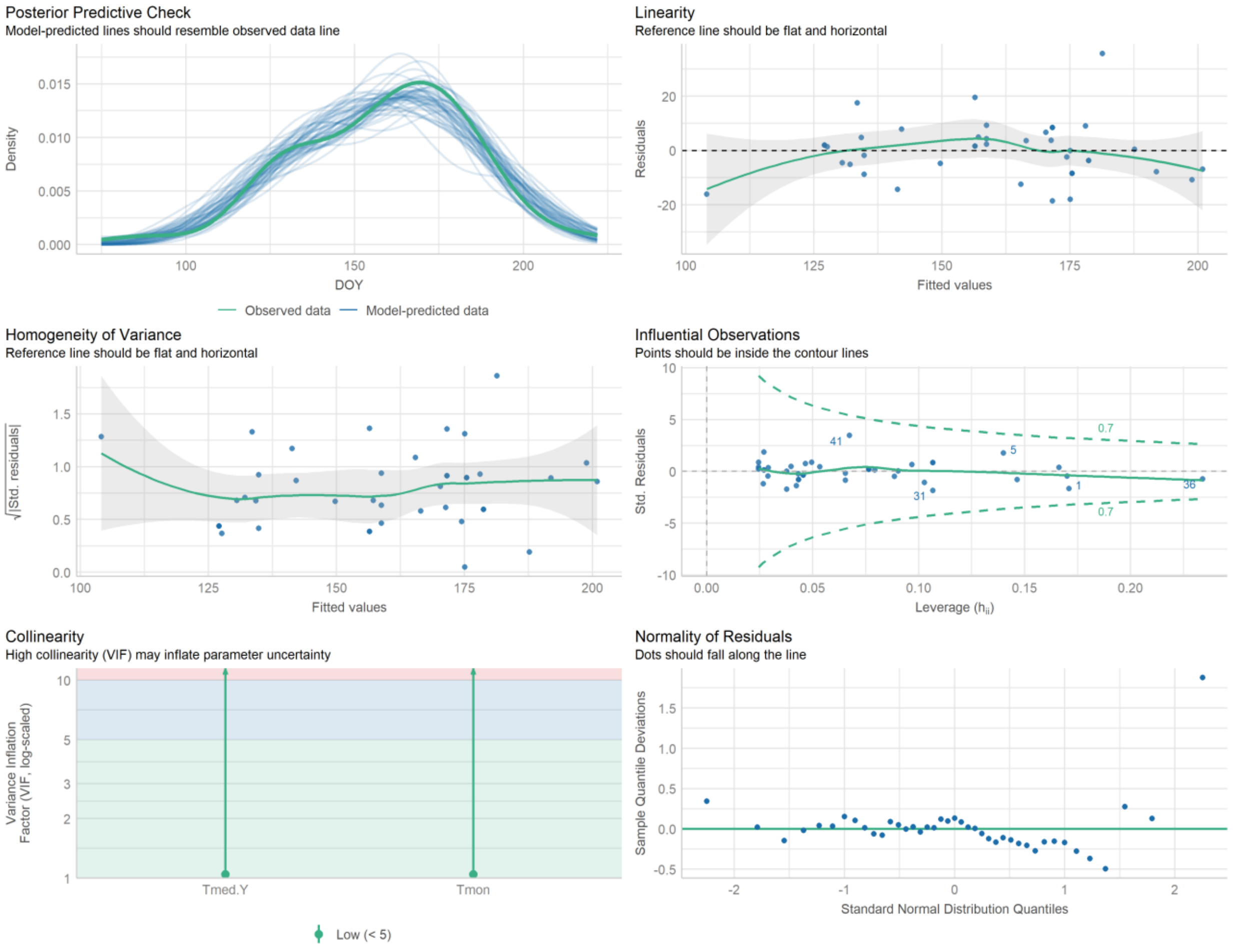

1.15. MLM - F - Ceratonia siliqua

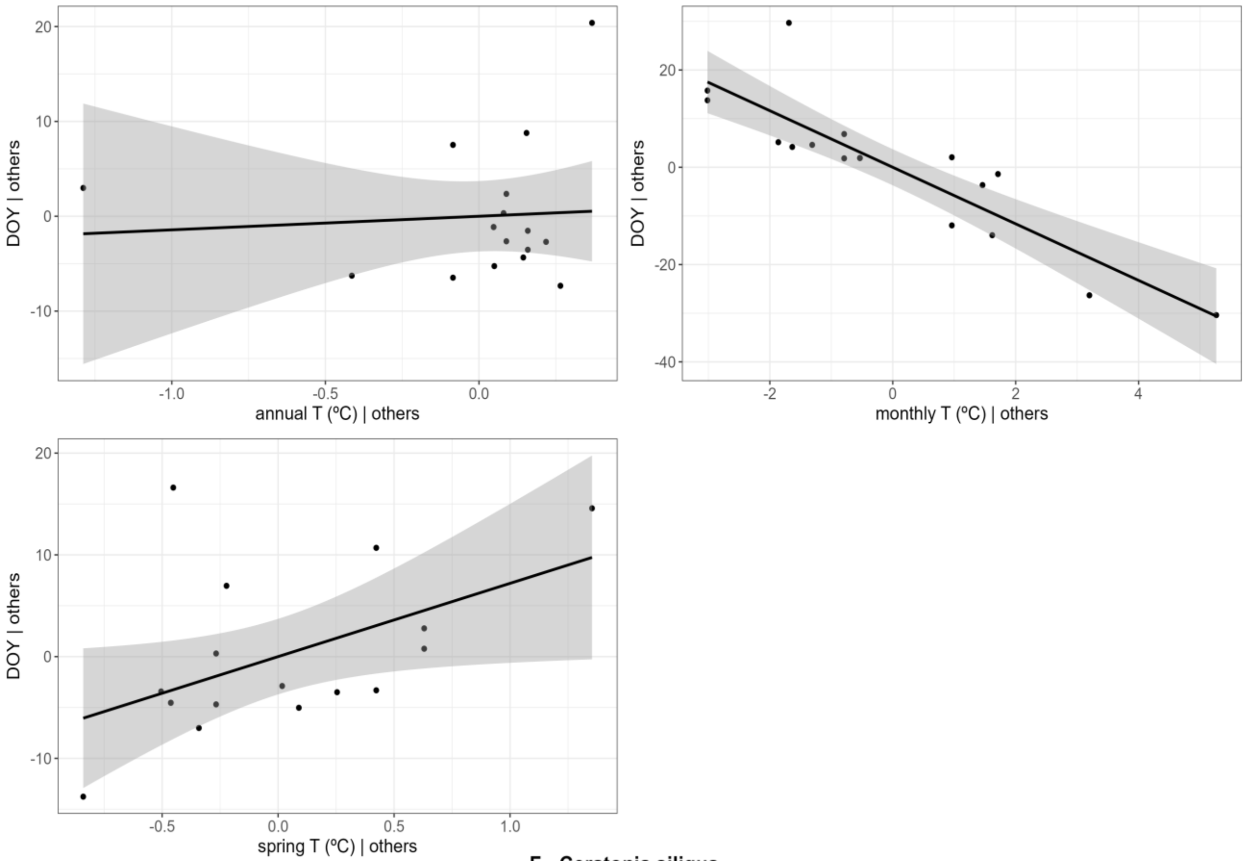

F - Ceratonia siliqua

DOY = 260.03 (+1.43*annual T (ºC)) + (-5.81*monthly T (ºC)) + (+7.20*spring T (ºC))

1.15.1. Diagnostics - MLM - F - Ceratonia siliqua

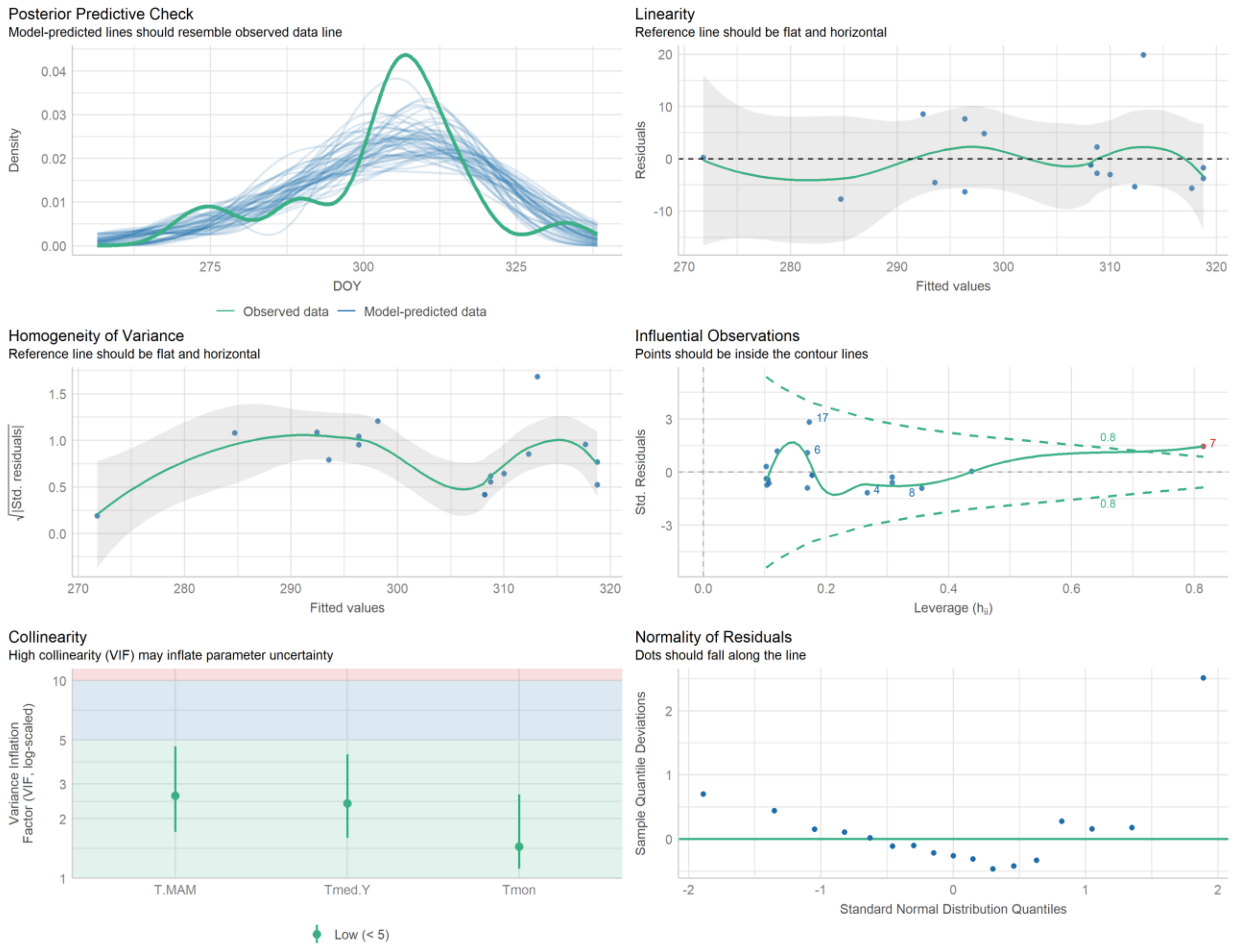

1.16. MLM - F - Chaenorhinum glareosum

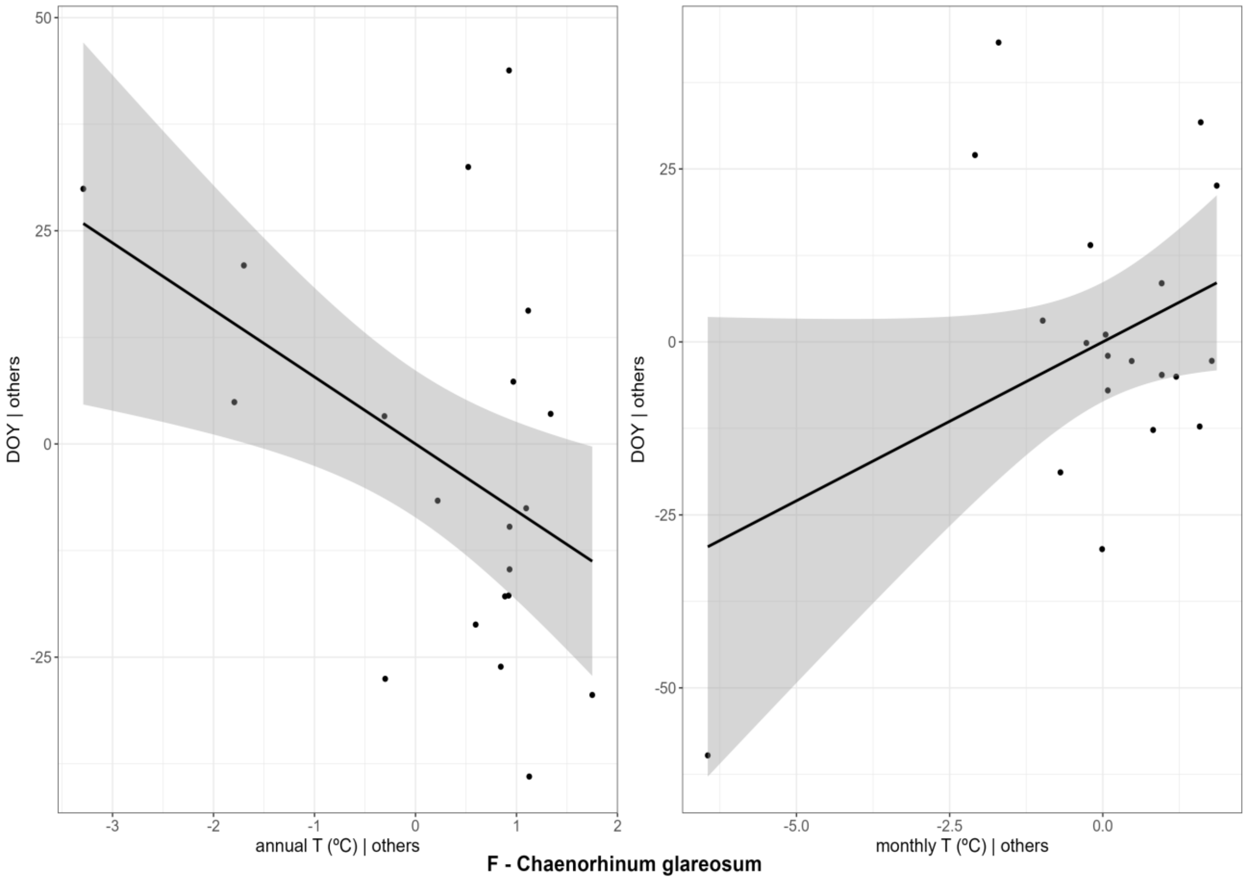

DOY = 222.40 (-7.86*annual T (ºC)) + (+4.59*monthly T (ºC))

1.16.1. Diagnostics - MLM - F - Chaenorhinum glareosum

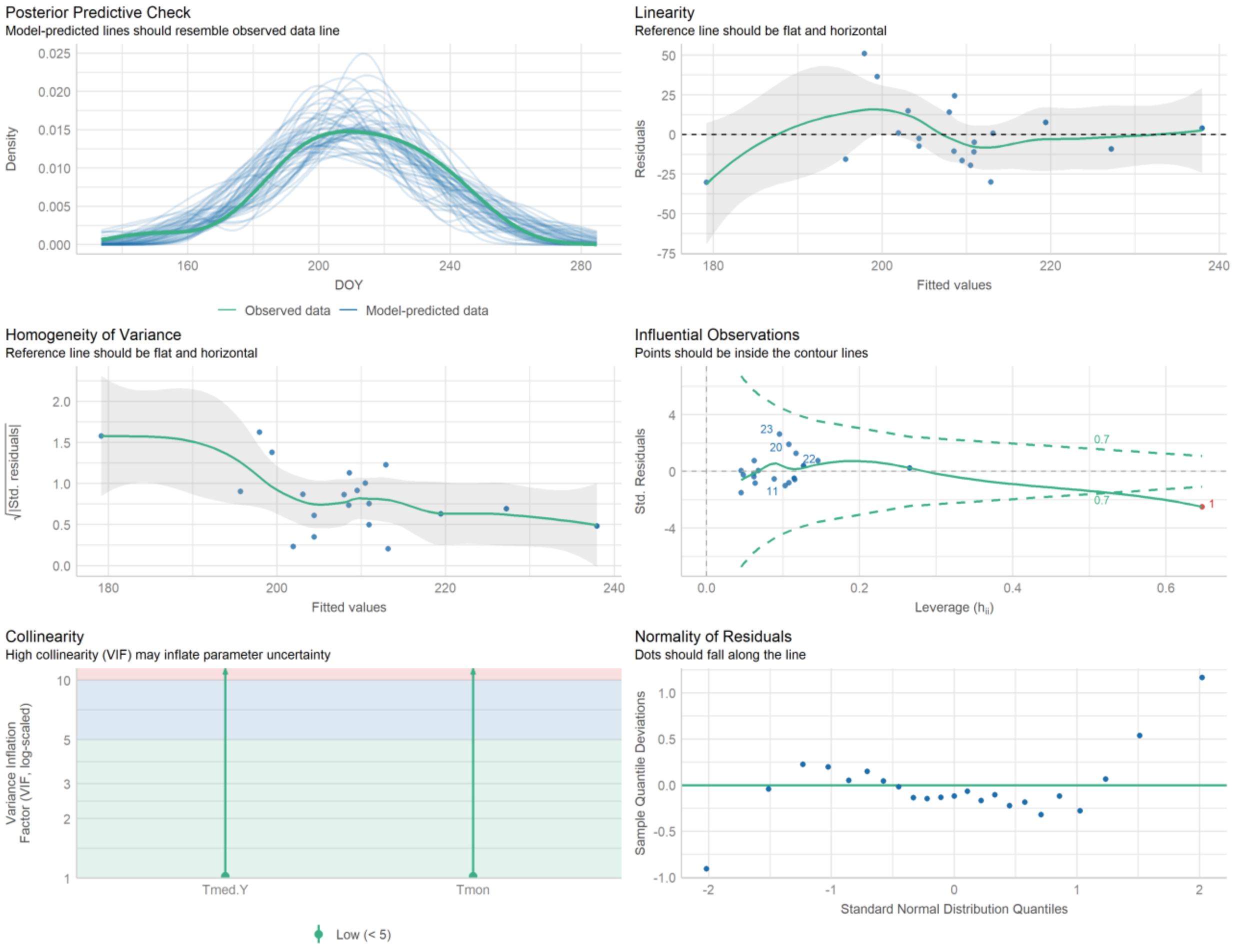

1.17. MLM - FBF - Cistus albidus

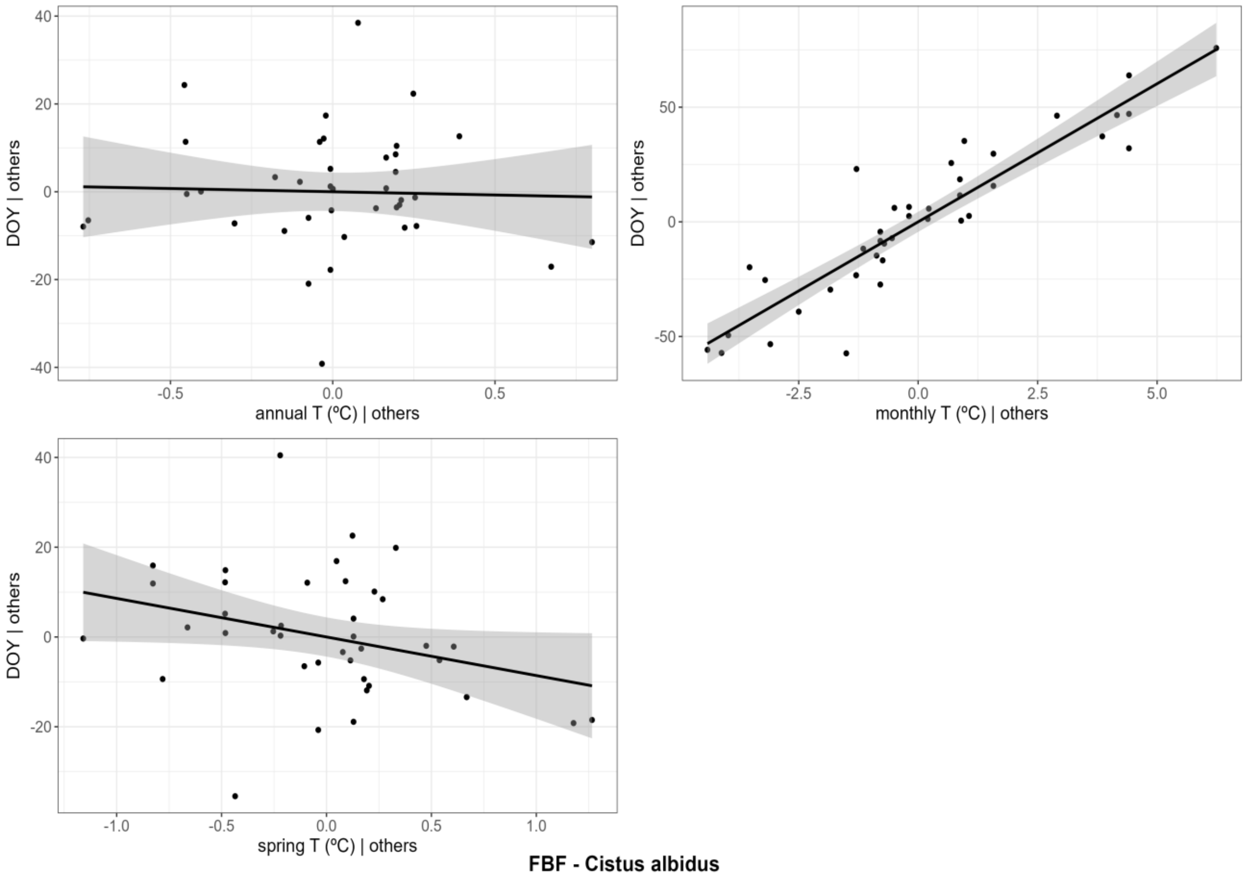

FBF - Cistus albidus

DOY = 77.08 (-1.47*annual T (ºC)) + (+12.05*monthly T (ºC)) + (-8.60*spring T (ºC))

1.17.1. Diagnostics - MLM - FBF - Cistus albidus

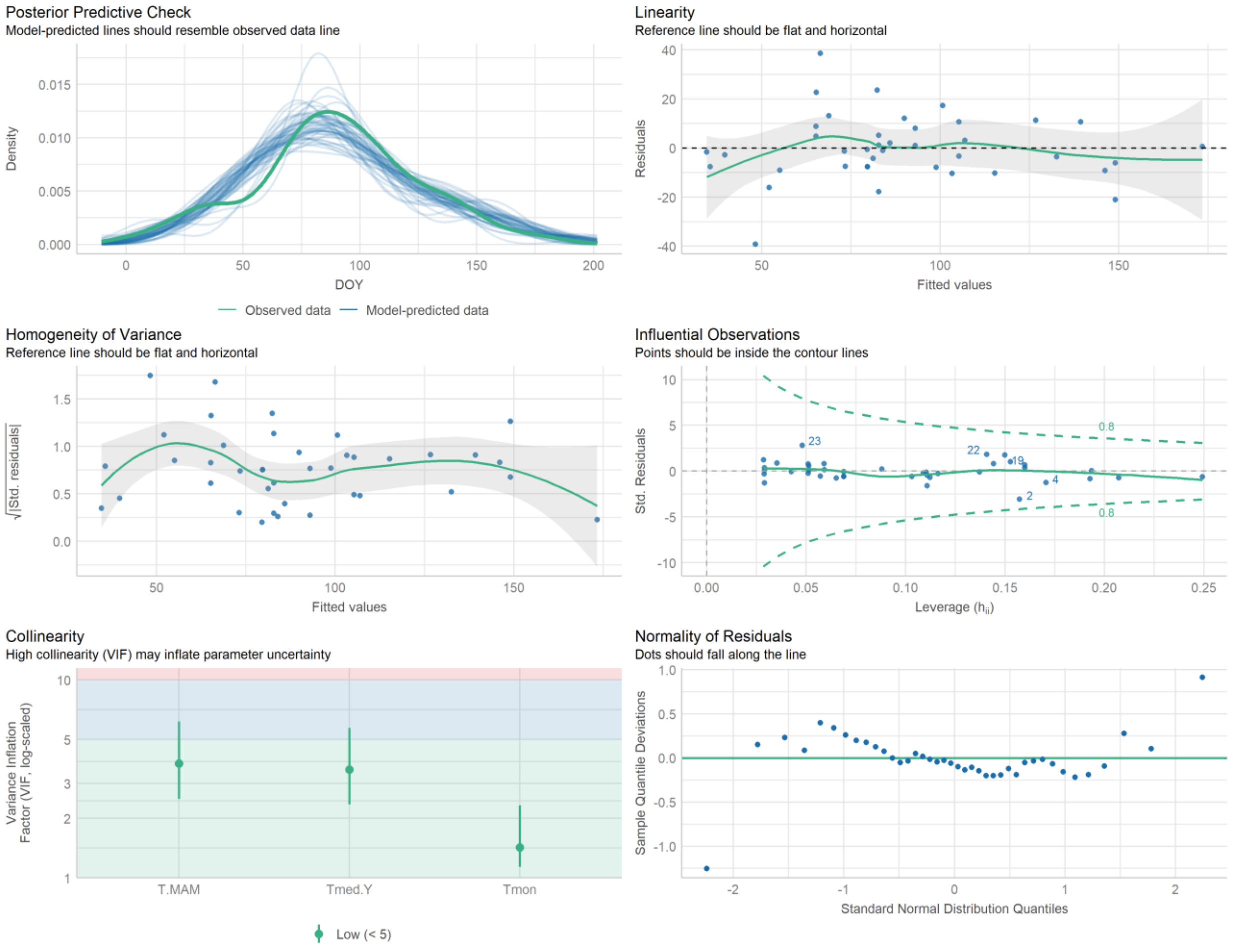

1.18. MLM - F - Cistus albidus

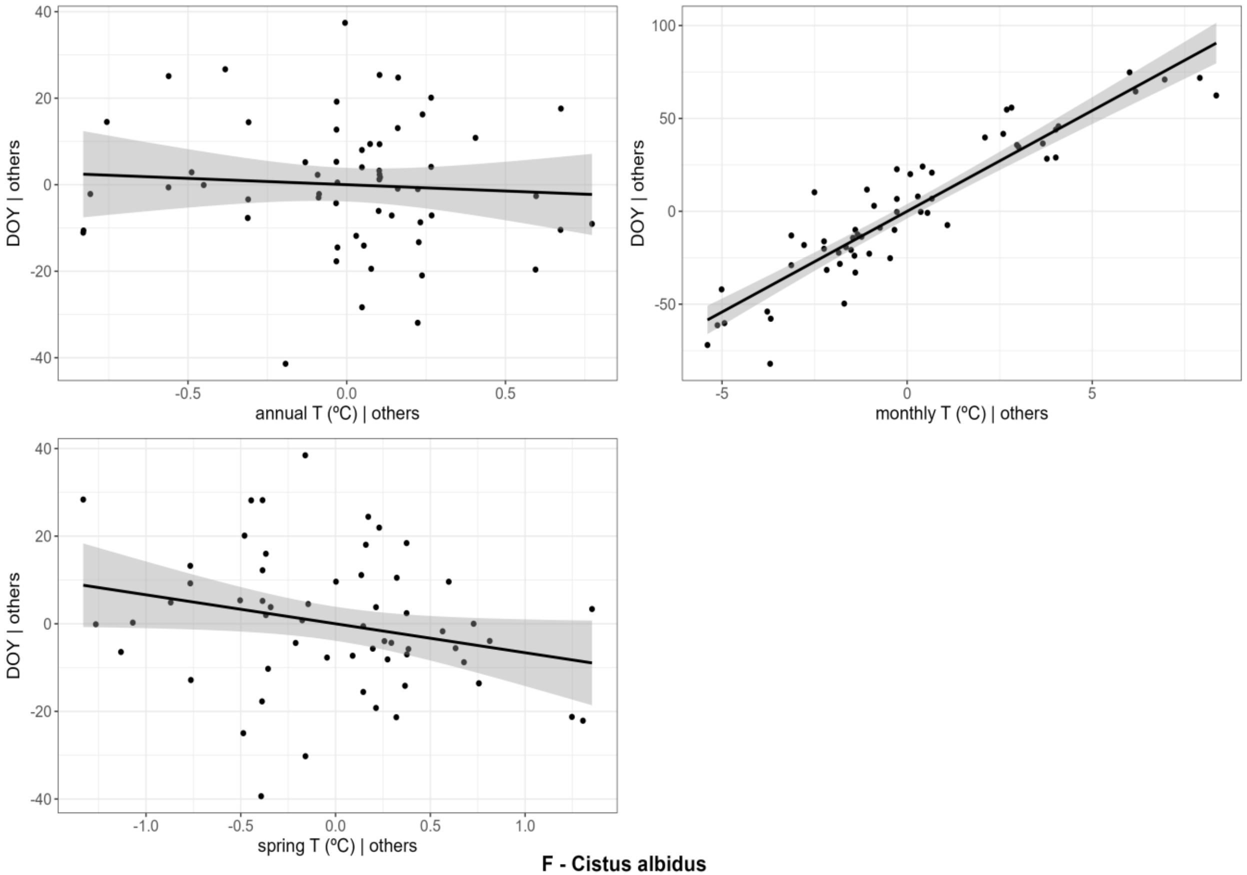

F - Cistus albidus

DOY = 87.32 (-2.92*annual T (ºC)) + (+10.84*monthly T (ºC)) + (-6.61*spring T (ºC))

1.18.1. Diagnostics - MLM - F - Cistus albidus

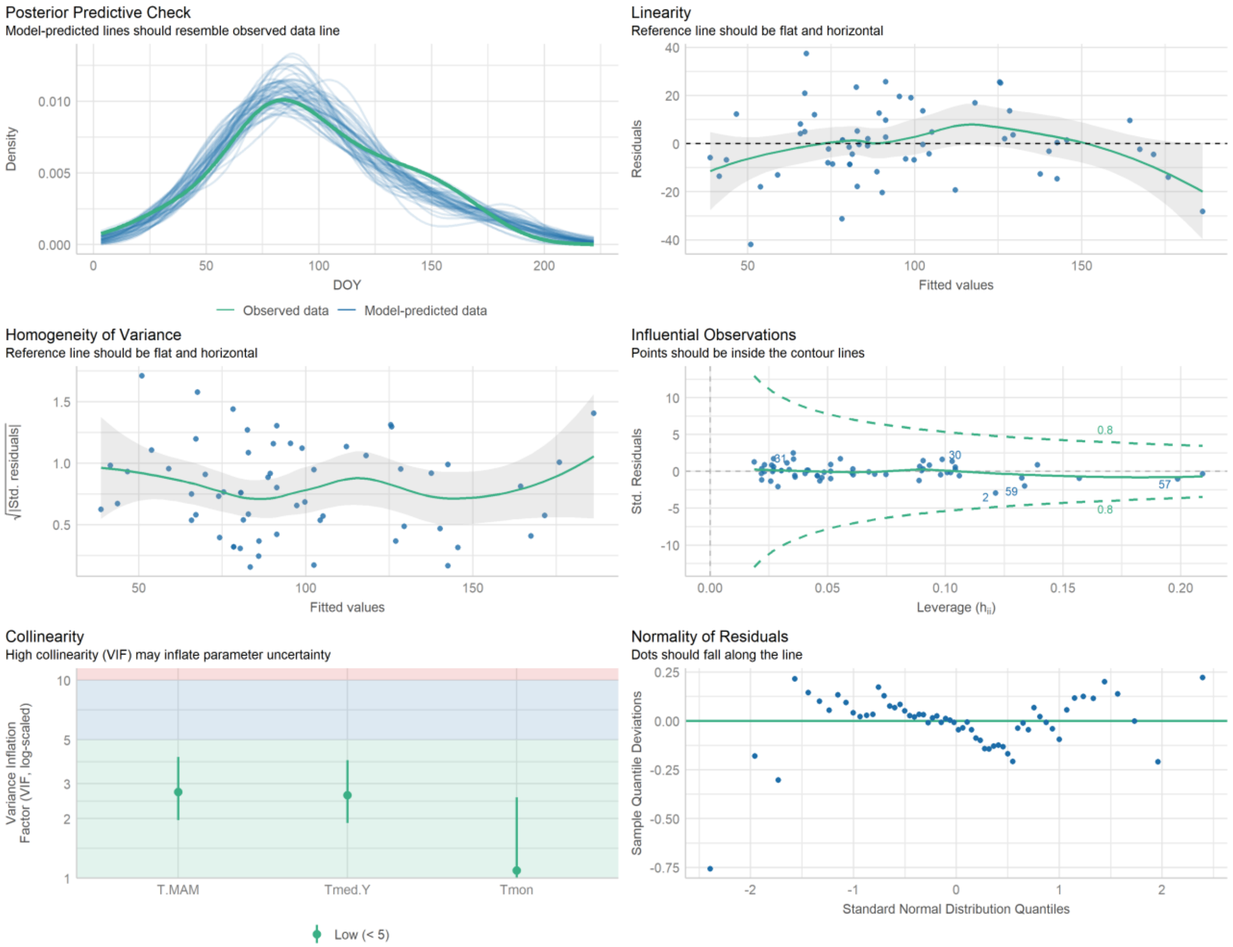

1.19. MLM - DVG - Cistus albidus

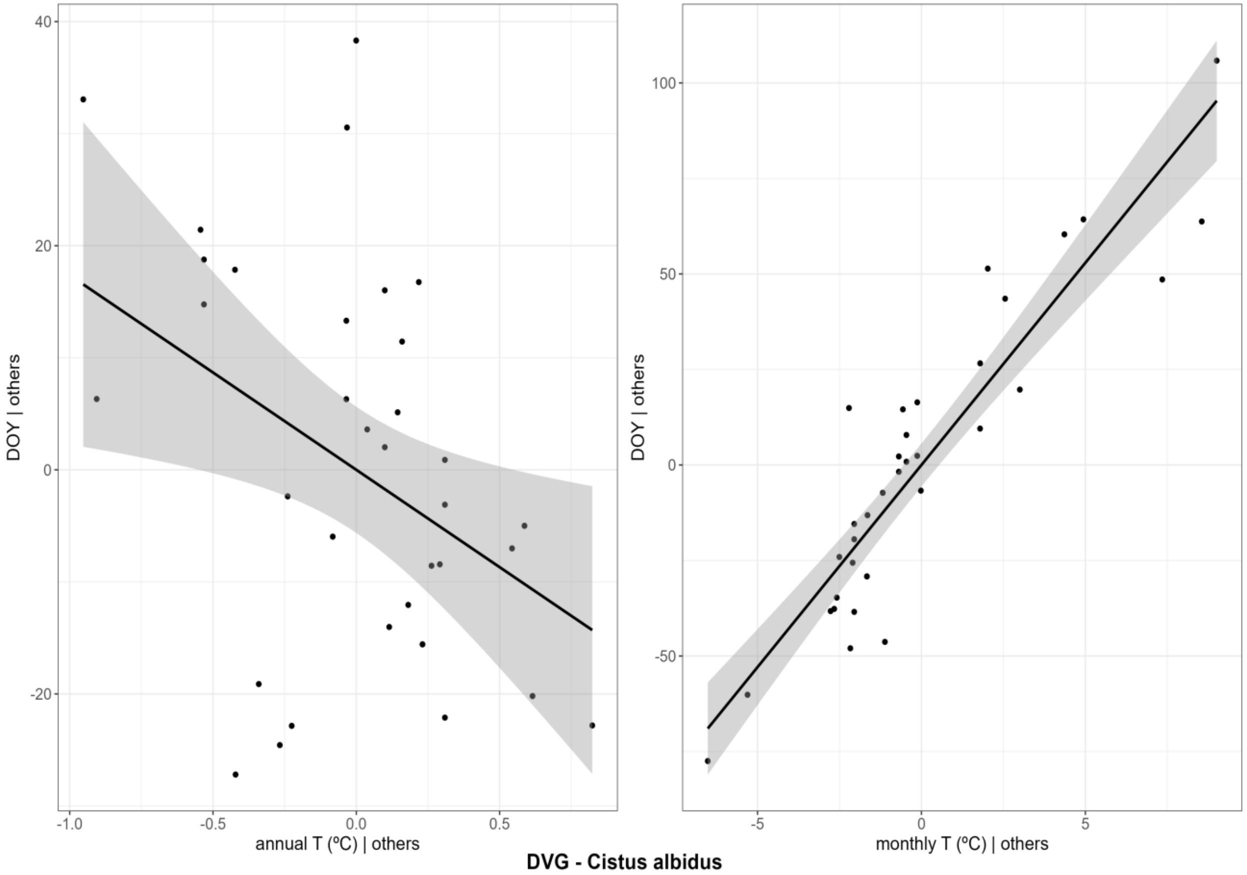

DOY = 231.35 (-17.37*annual T (ºC)) + (+10.58*monthly T (ºC))

1.19.1. Diagnostics - MLM - DVG - Cistus albidus

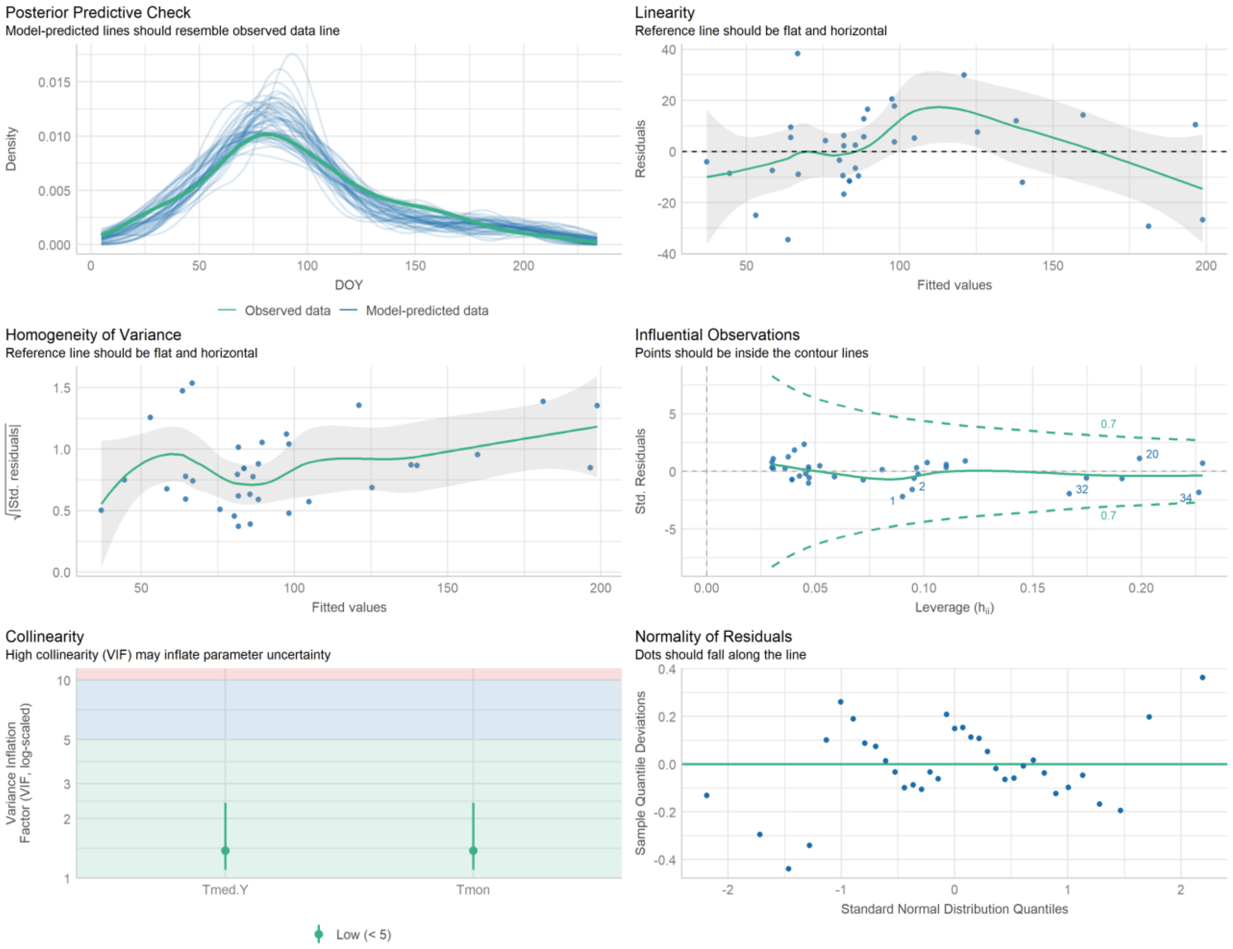

1.20. MLM - FBF - Cistus ladanifer

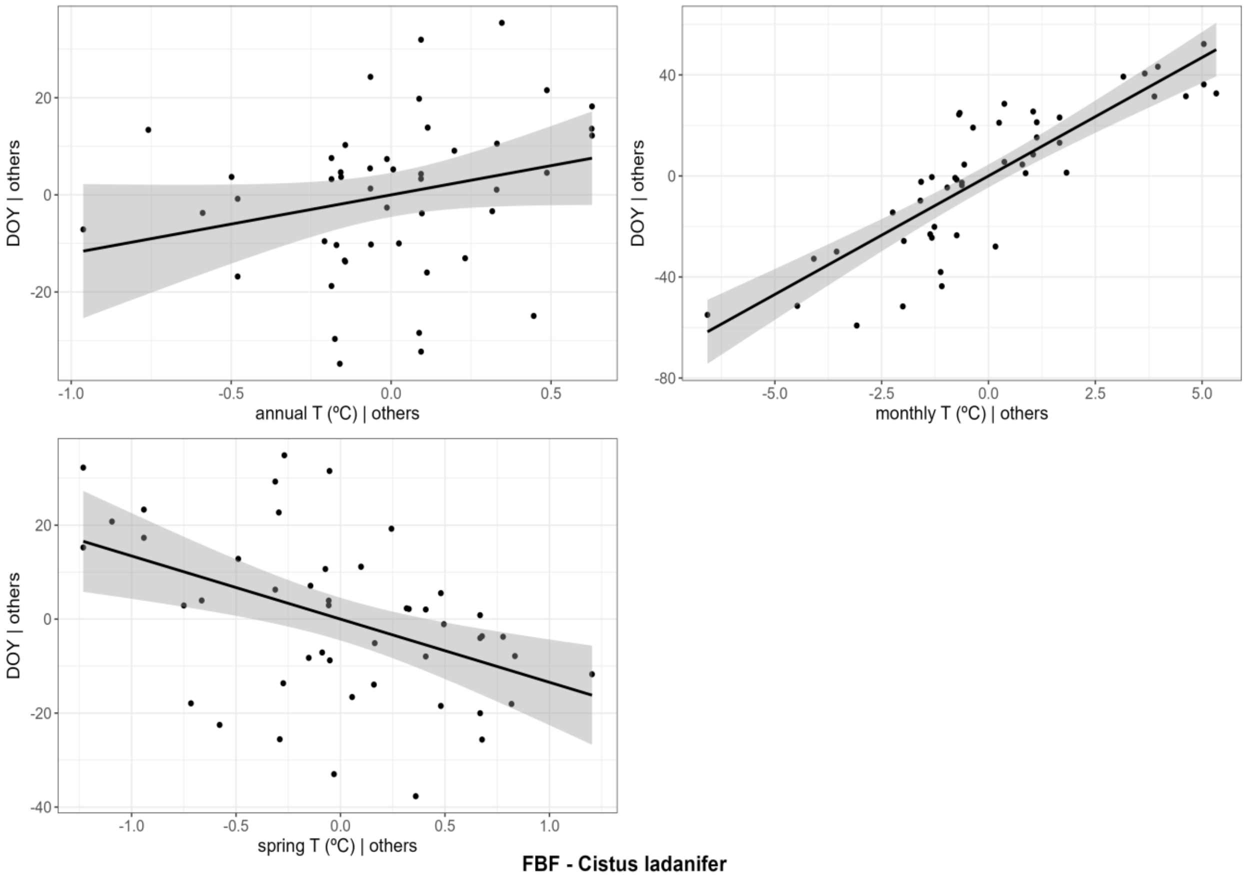

FBF - Cistus ladanifer

DOY = -41.14 (+12.03*annual T (ºC)) + (+9.38*monthly T (ºC)) + (-13.45*spring T (ºC))

1.20.1. Diagnostics - MLM - FBF - Cistus ladanifer

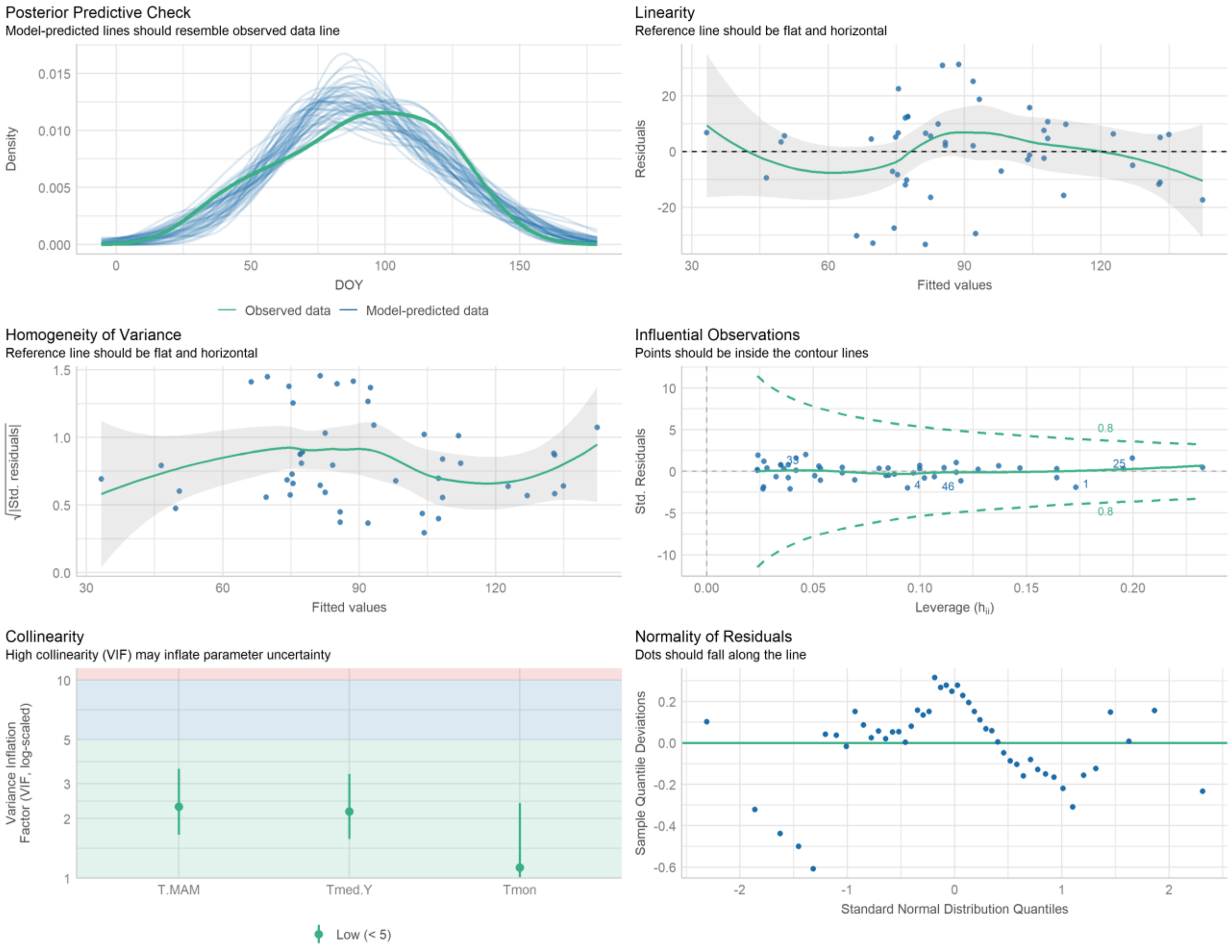

1.21. MLM - F - Cistus ladanifer

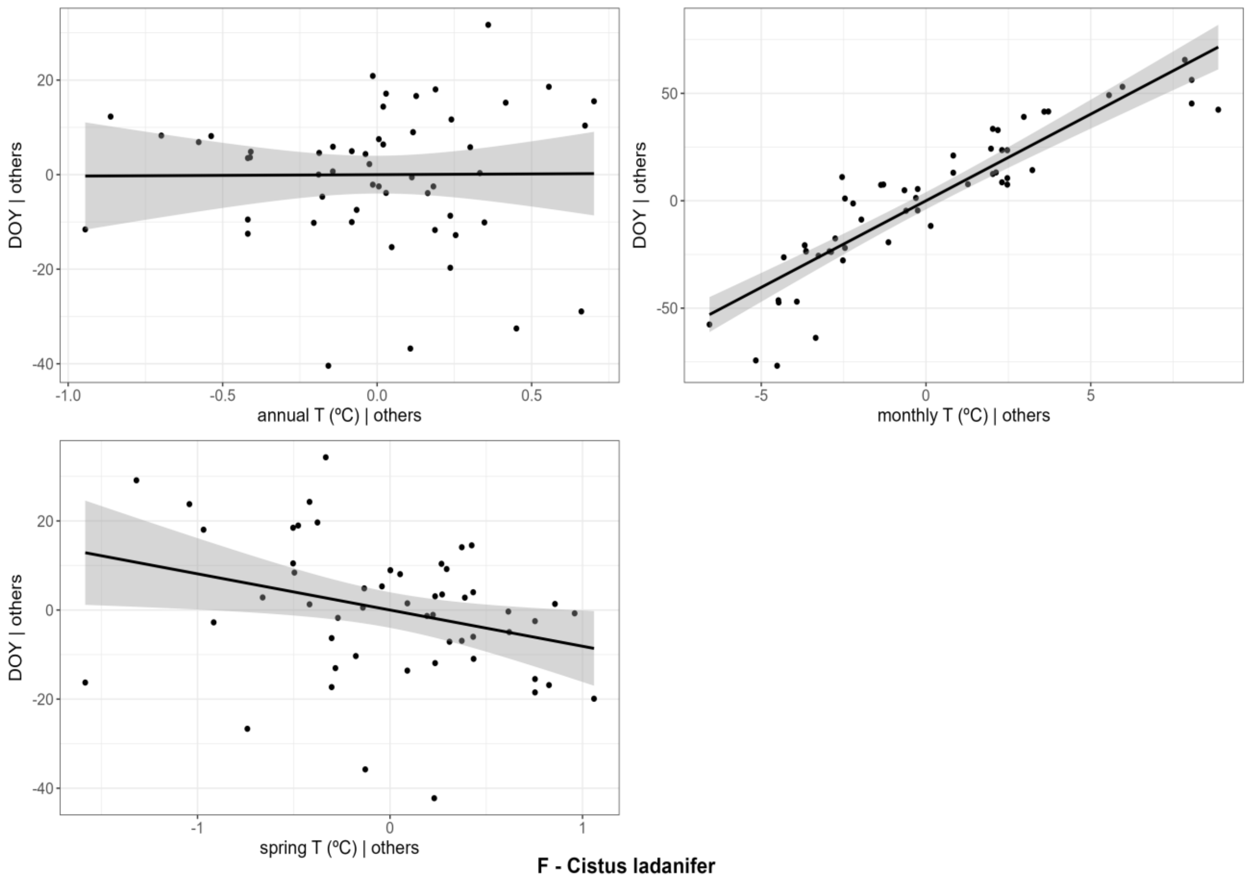

F - Cistus ladanifer

DOY = 97.61 (+0.31*annual T (ºC)) + (+8.06*monthly T (ºC)) + (-8.13*spring T (ºC))

1.21.1. Diagnostics - MLM - F - Cistus ladanifer

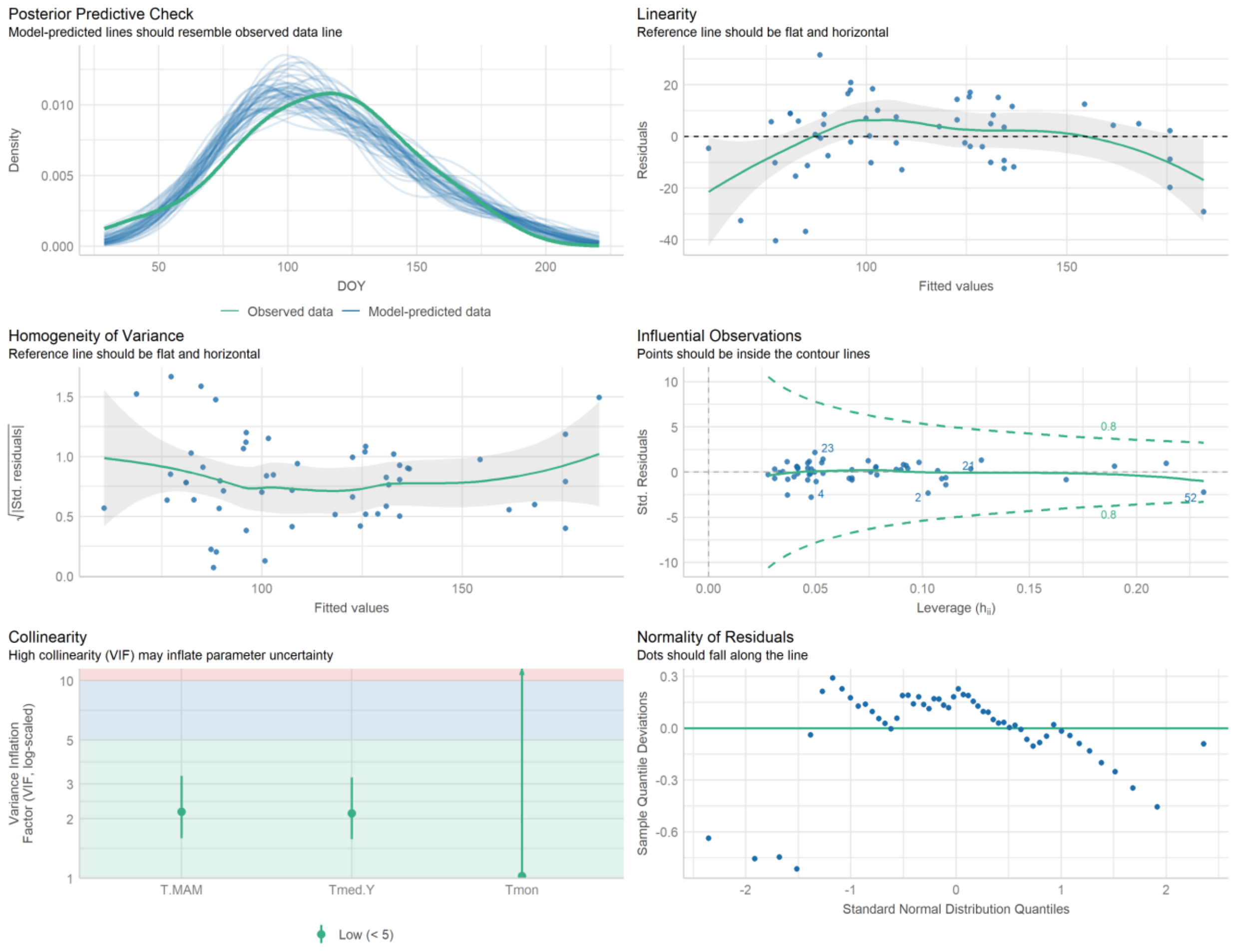

1.22. MLM - FS - Cistus ladanifer

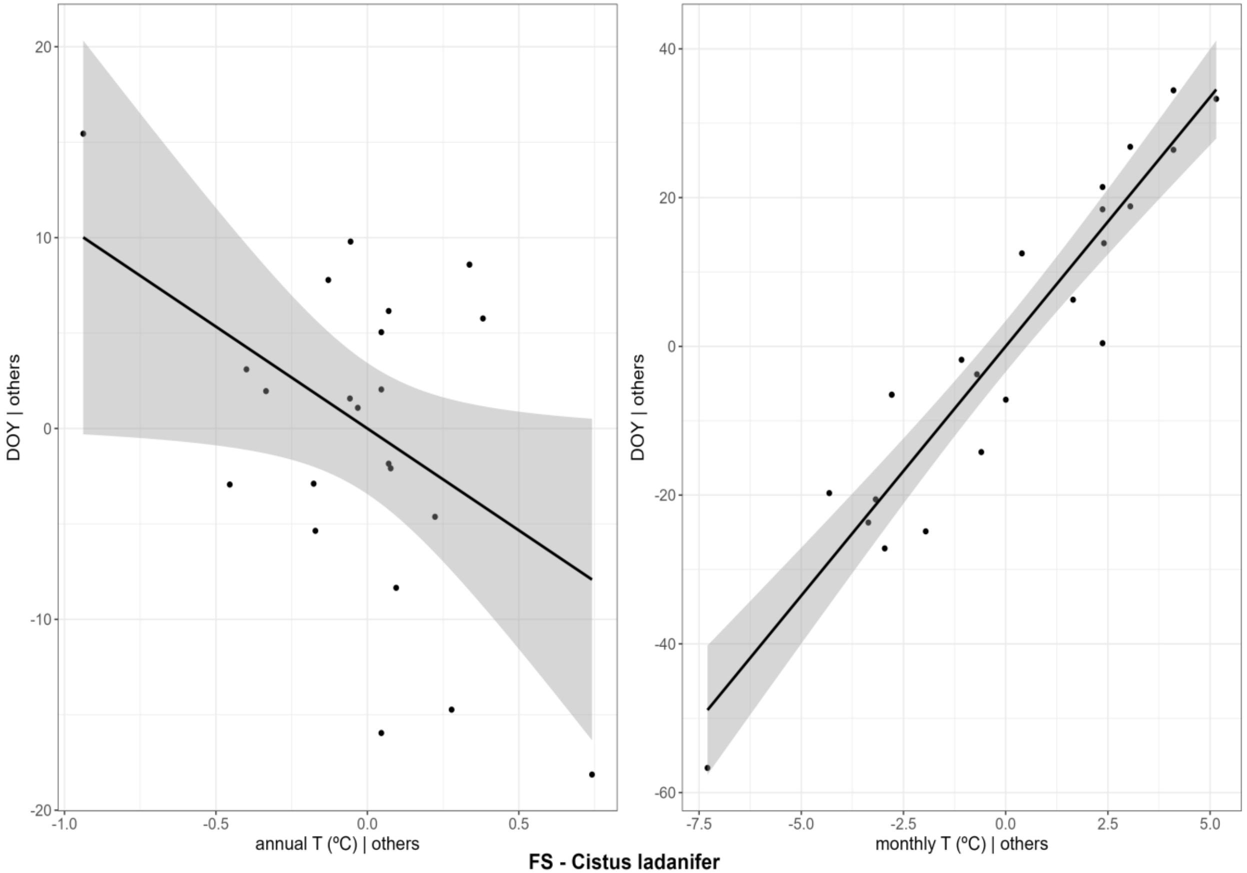

FS - Cistus ladanifer

DOY = 187.51 (-10.67*annual T (ºC)) + (+6.70*monthly T (ºC))

1.22.1. Diagnostics - MLM - FS - Cistus ladanifer

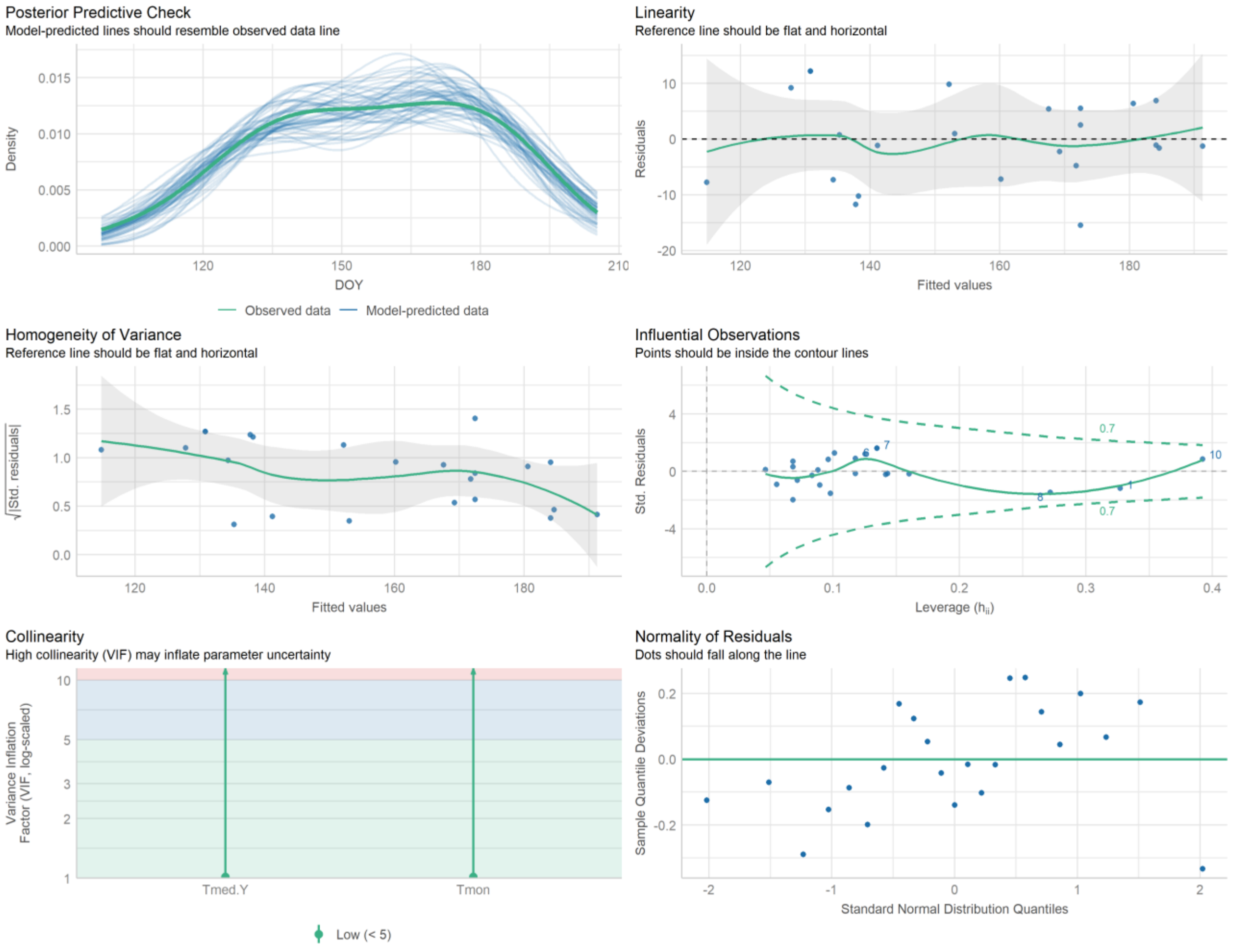

1.23. MLM - DVG - Cistus ladanifer

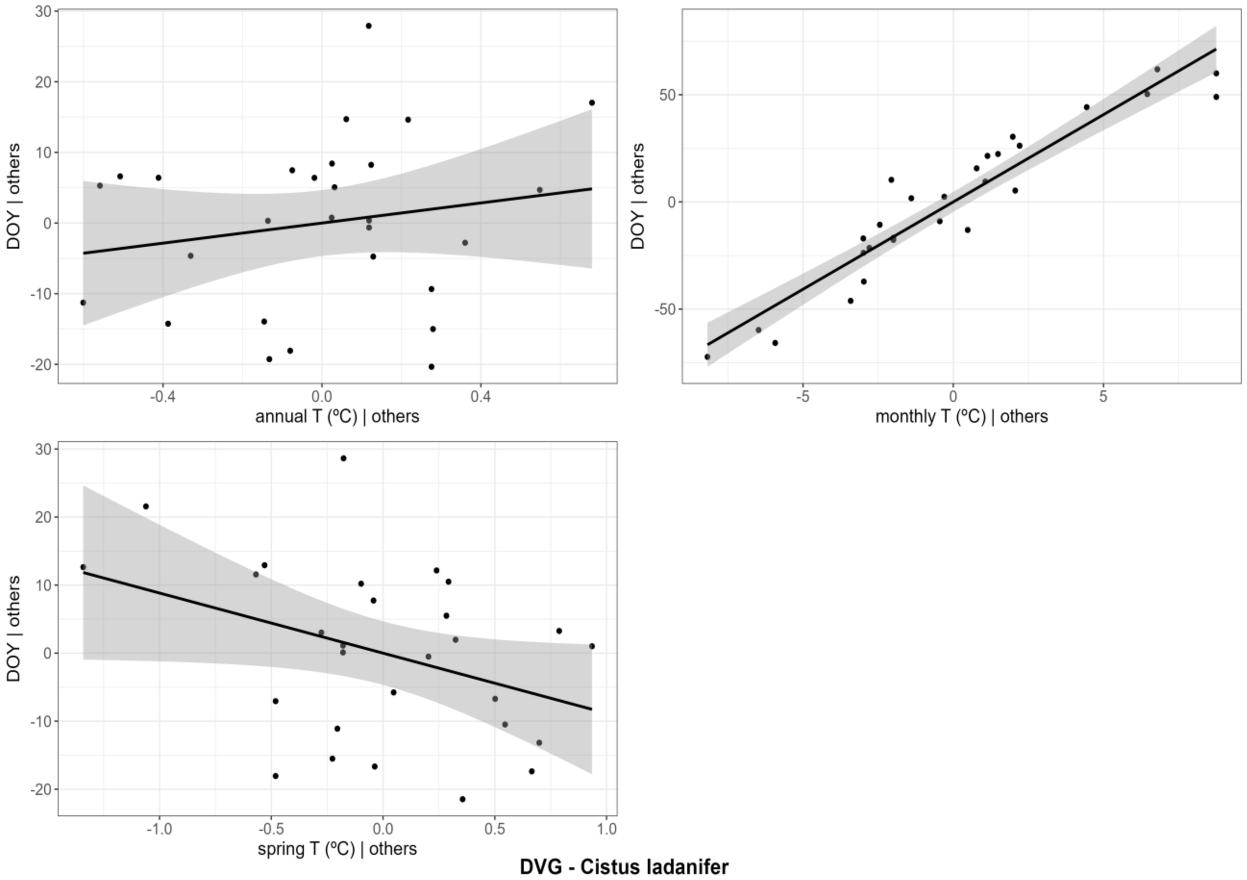

DOY = -6.94 (+7.12*annual T (ºC)) + (+8.14*monthly T (ºC)) + (-8.83*spring T (ºC))

1.23.1. Diagnostics - MLM - DVG - Cistus ladanifer

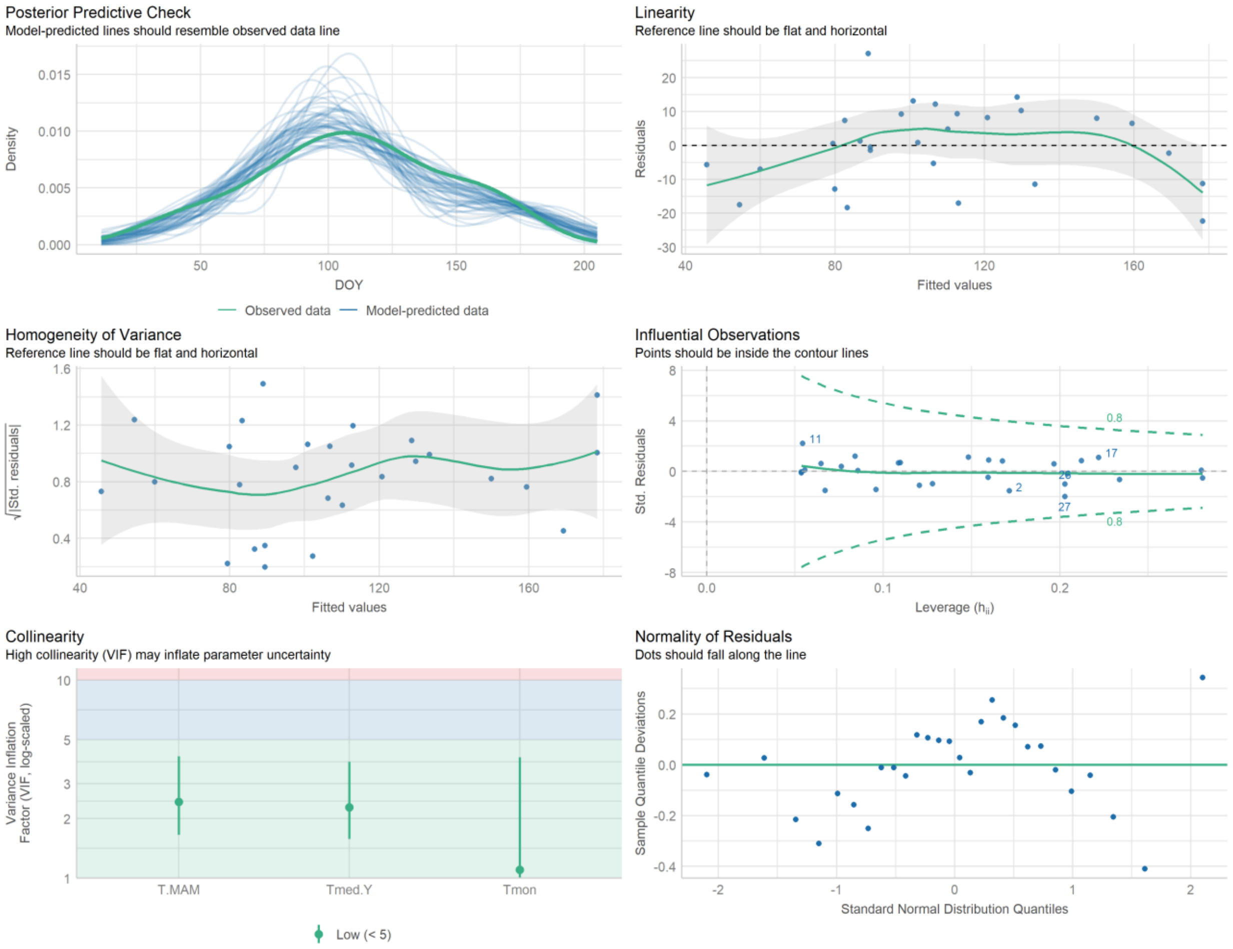

1.24. MLM - FBF - Cistus laurifolius

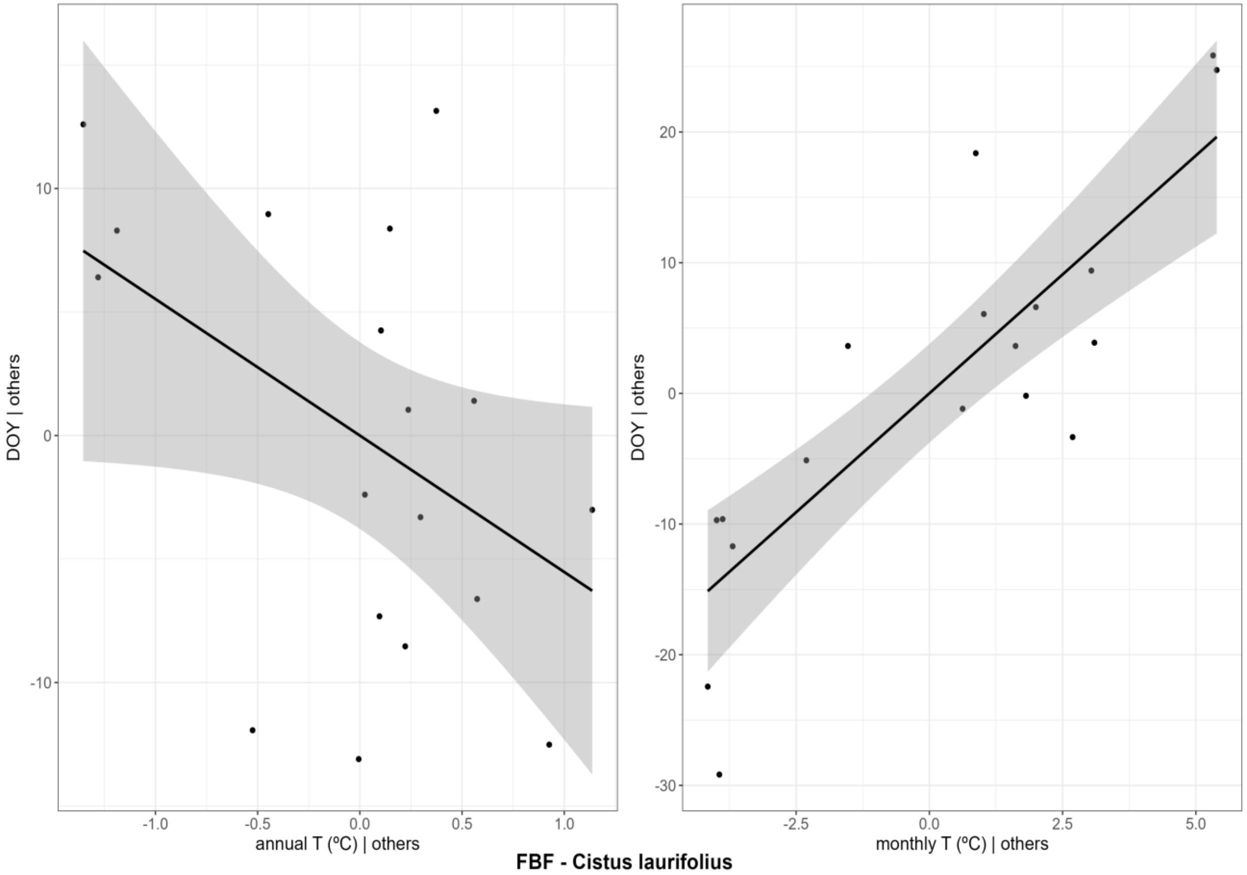

DOY = 174.04 (-5.53*annual T (ºC)) + (+3.64*monthly T (ºC))

1.24.1. Diagnostics - MLM - FBF - Cistus laurifolius

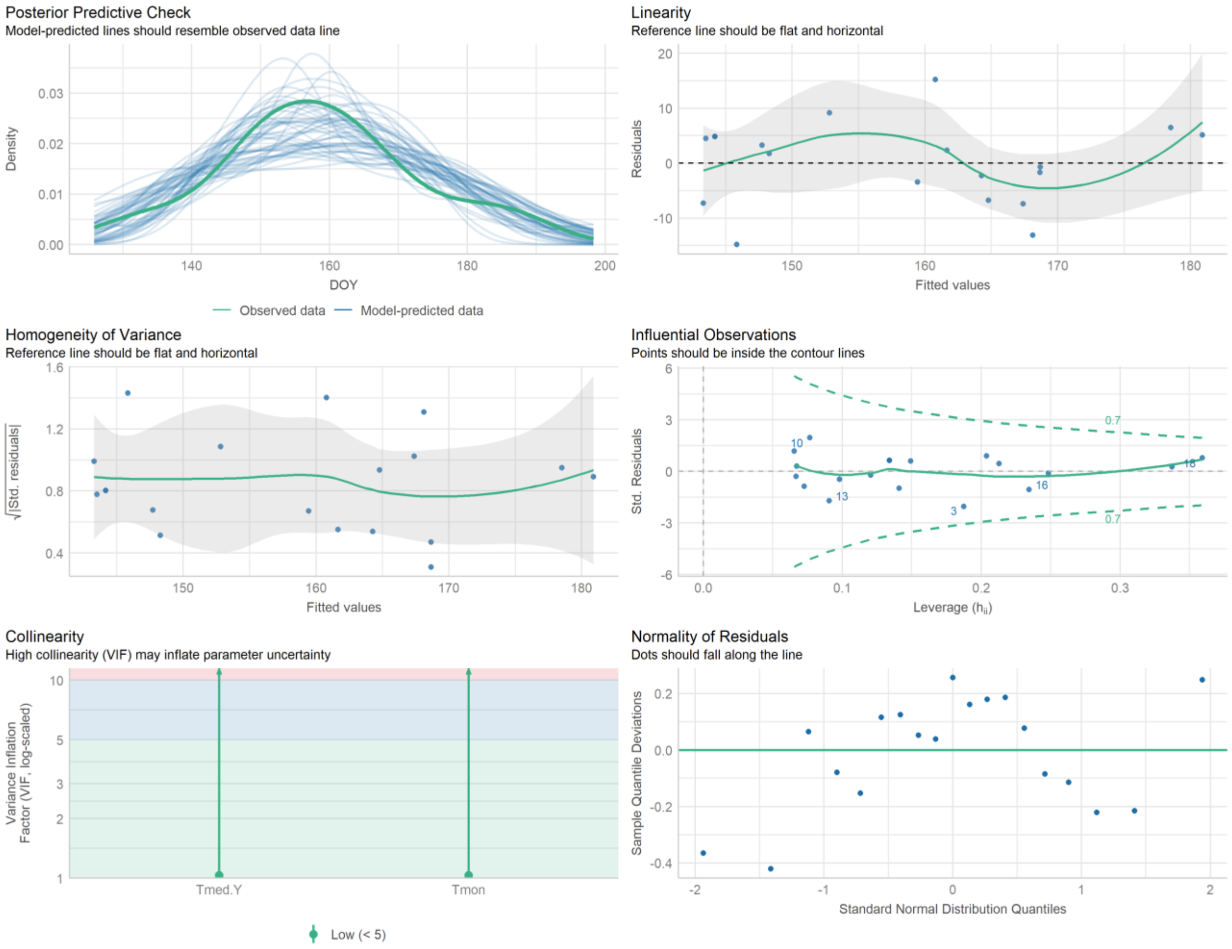

1.25. MLM - F - Cistus laurifolius

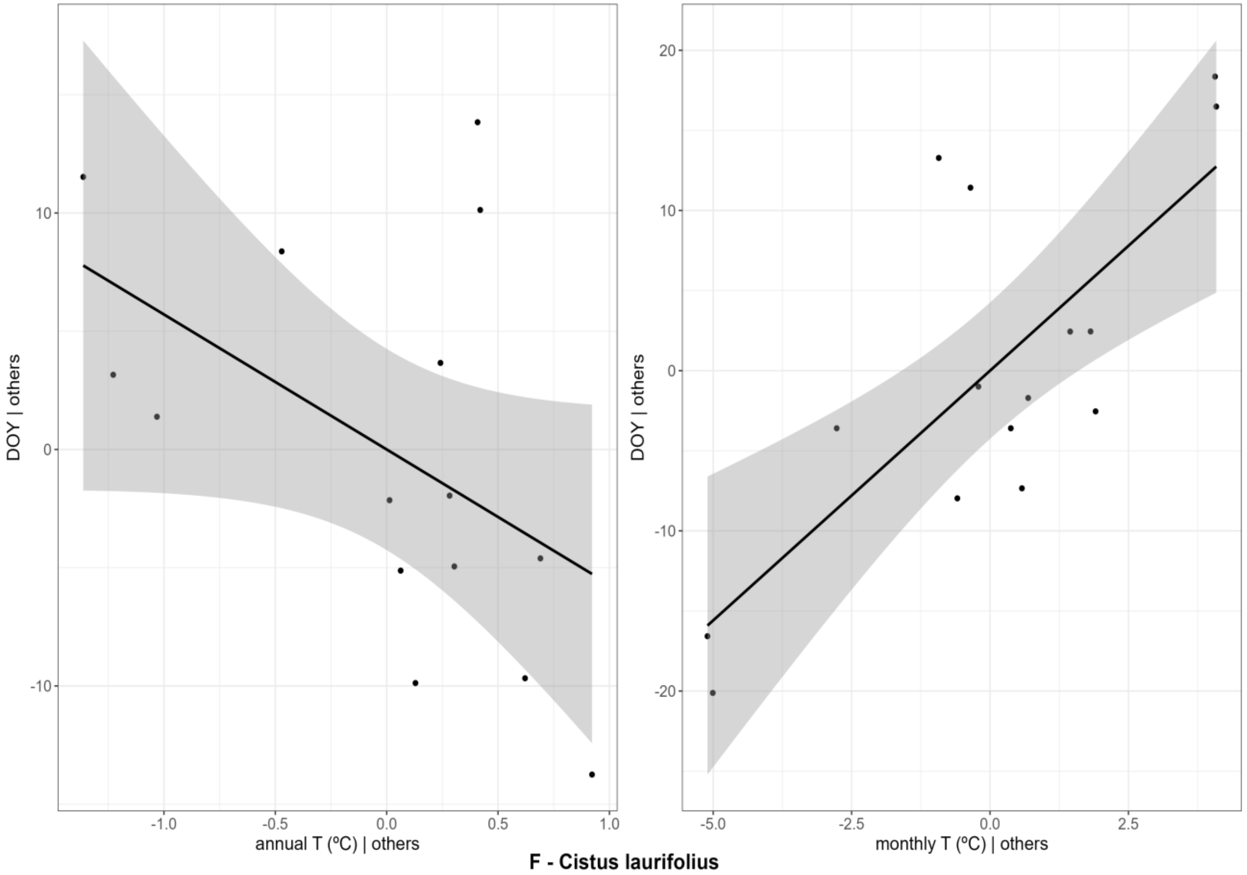

DOY = 191.42 (-5.71*annual T (ºC)) + (+3.12*monthly T (ºC))

1.25.1. Diagnostics - MLM - F - Cistus laurifolius

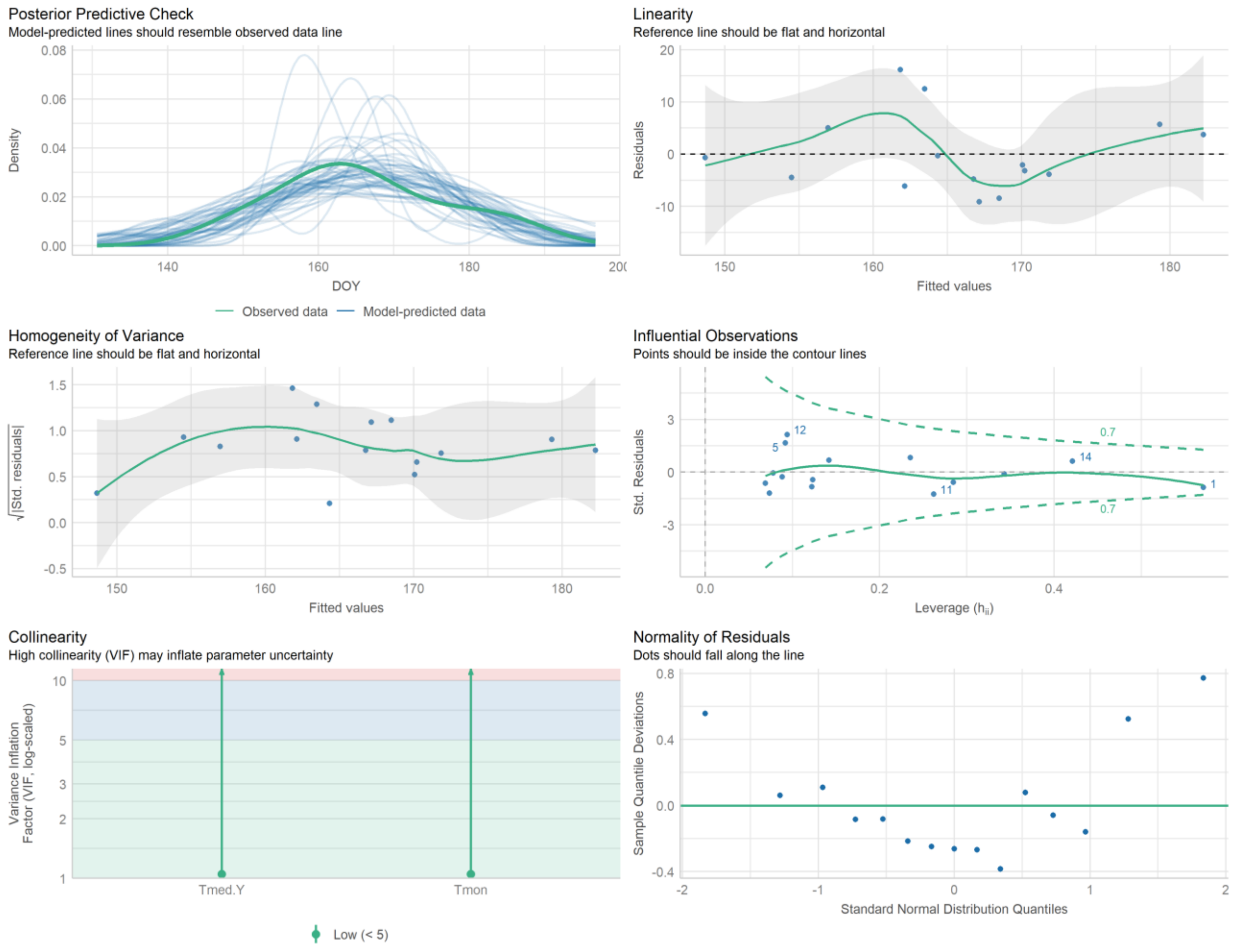

1.26. MLM - FBF - Cistus populifolius

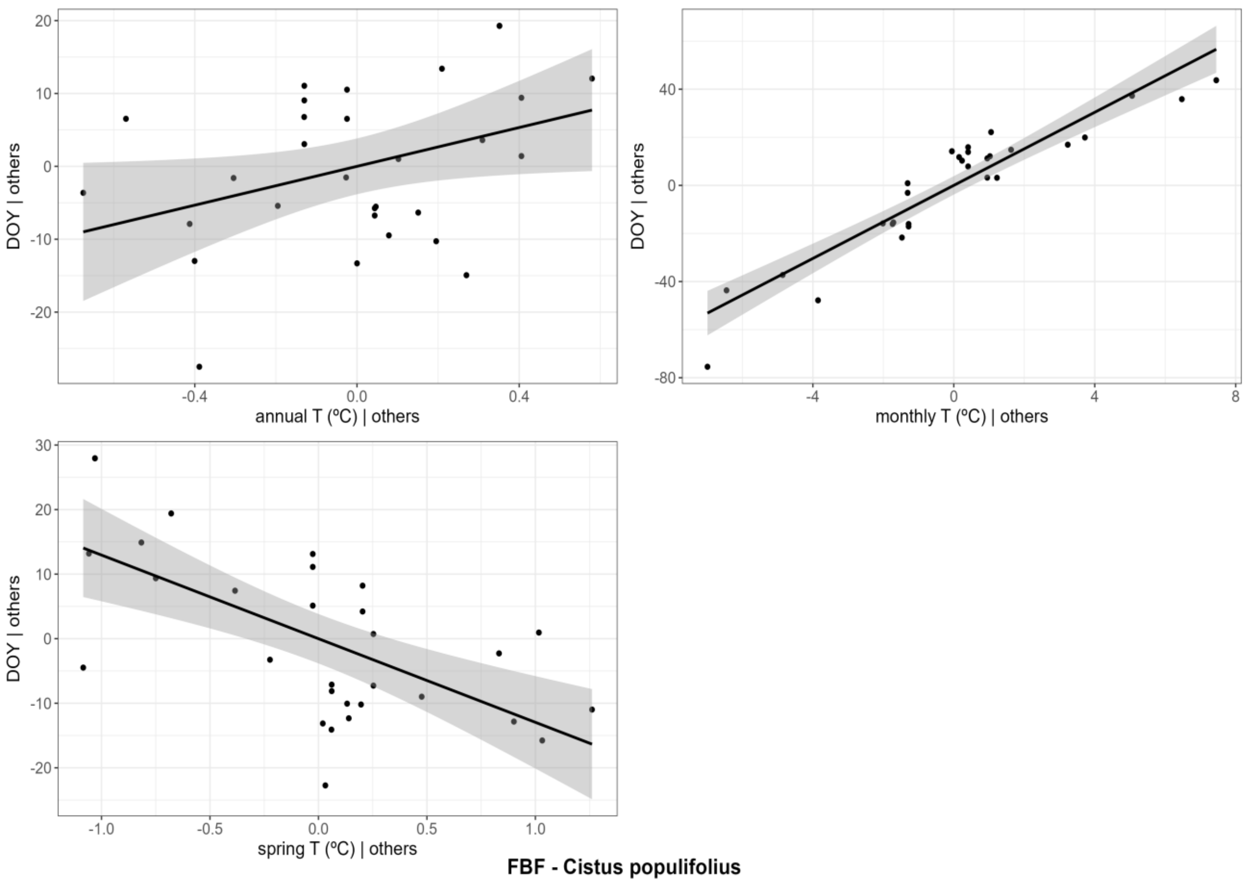

FBF - Cistus populifolius

DOY = -38.08 (+13.32*annual T (ºC)) + (+7.60*monthly T (ºC)) + (-12.96*spring T (ºC))

1.26.1. Diagnostics - MLM - FBF - Cistus populifolius

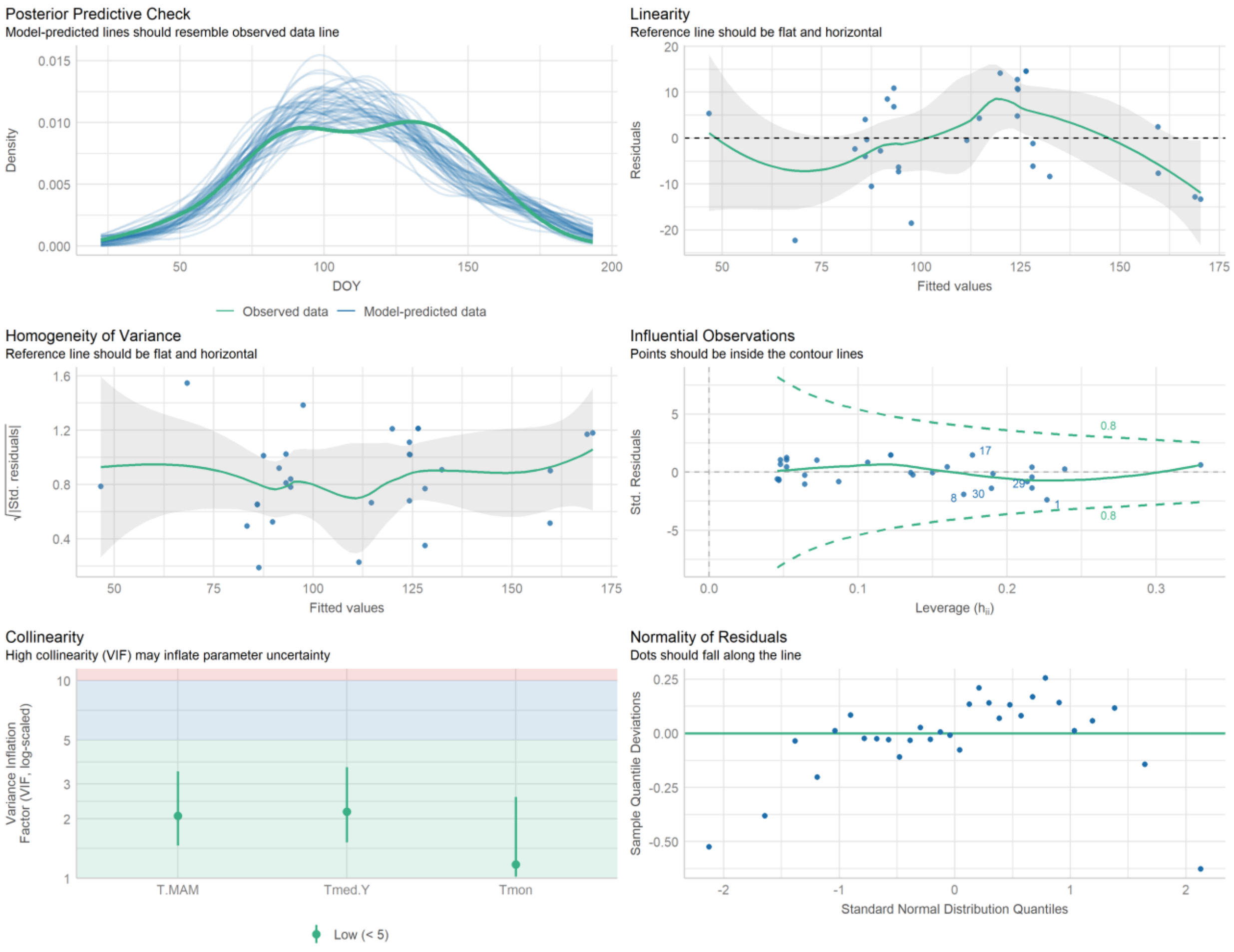

1.27. MLM - F - Cistus populifolius

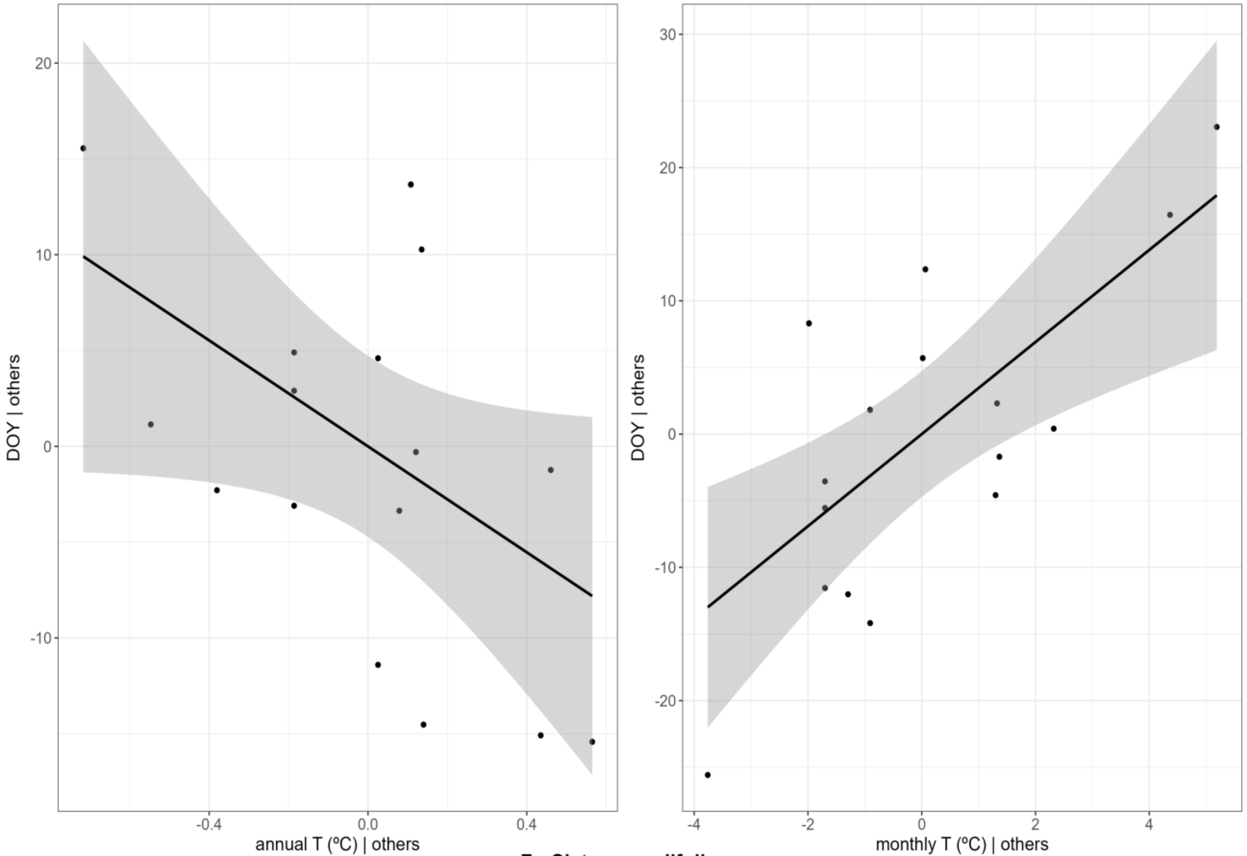

DOY = 303.20 (-13.82*annual T (ºC)) + (+3.45*monthly T (ºC))

1.27.1. Diagnostics - MLM - F - Cistus populifolius

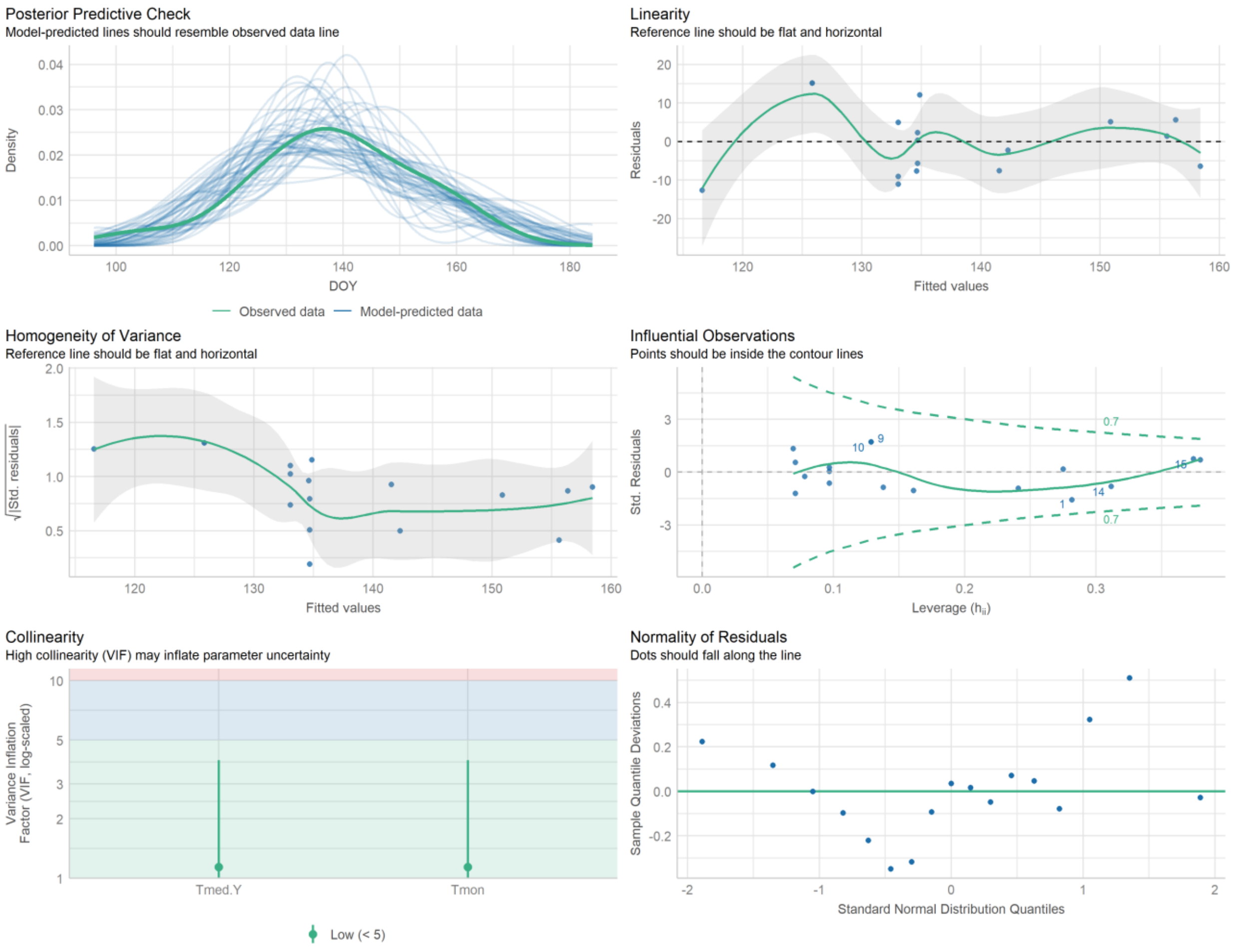

1.28. MLM - FS - Cistus populifolius

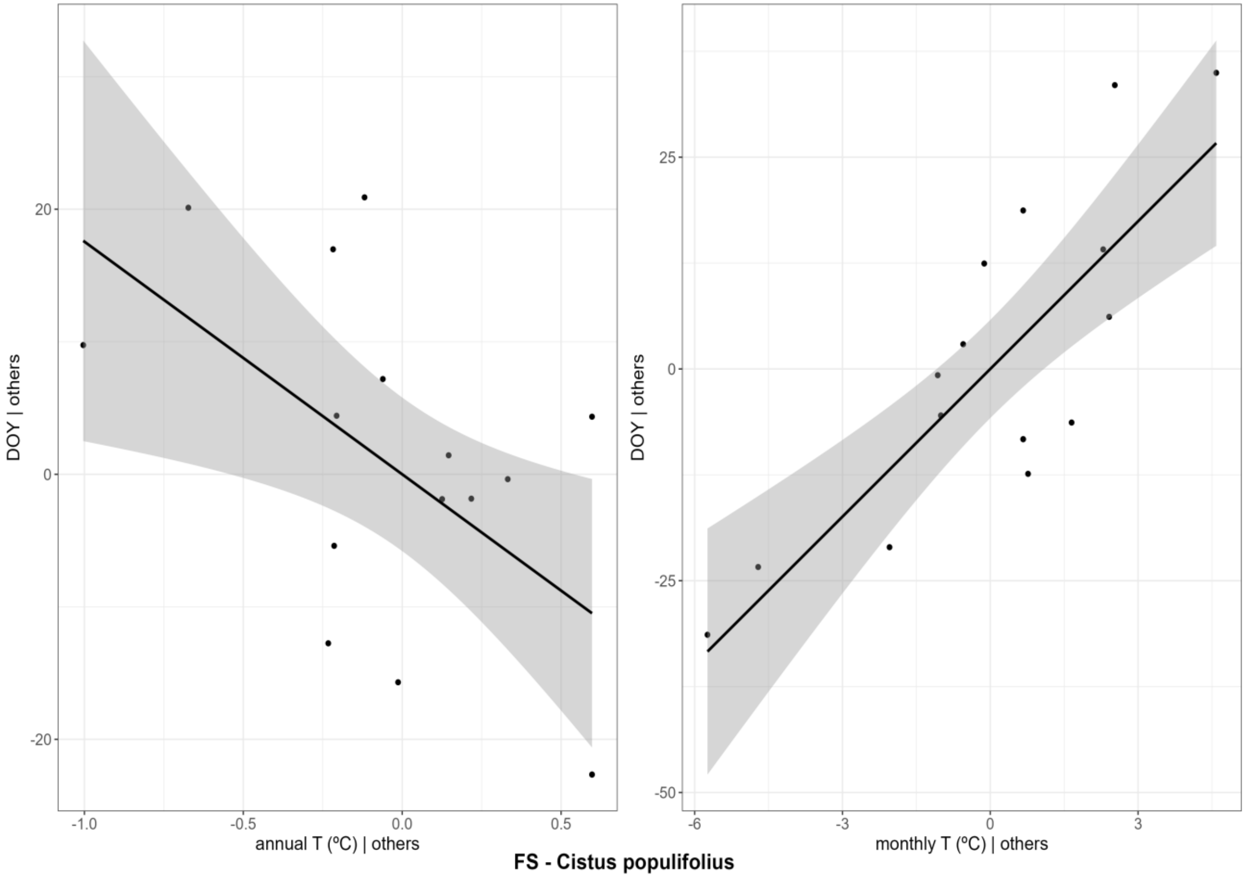

DOY = 330.05 (-17.55*annual T (ºC)) + (+5.81*monthly T (ºC))

1.28.1. Diagnostics - MLM - FS - Cistus populifolius

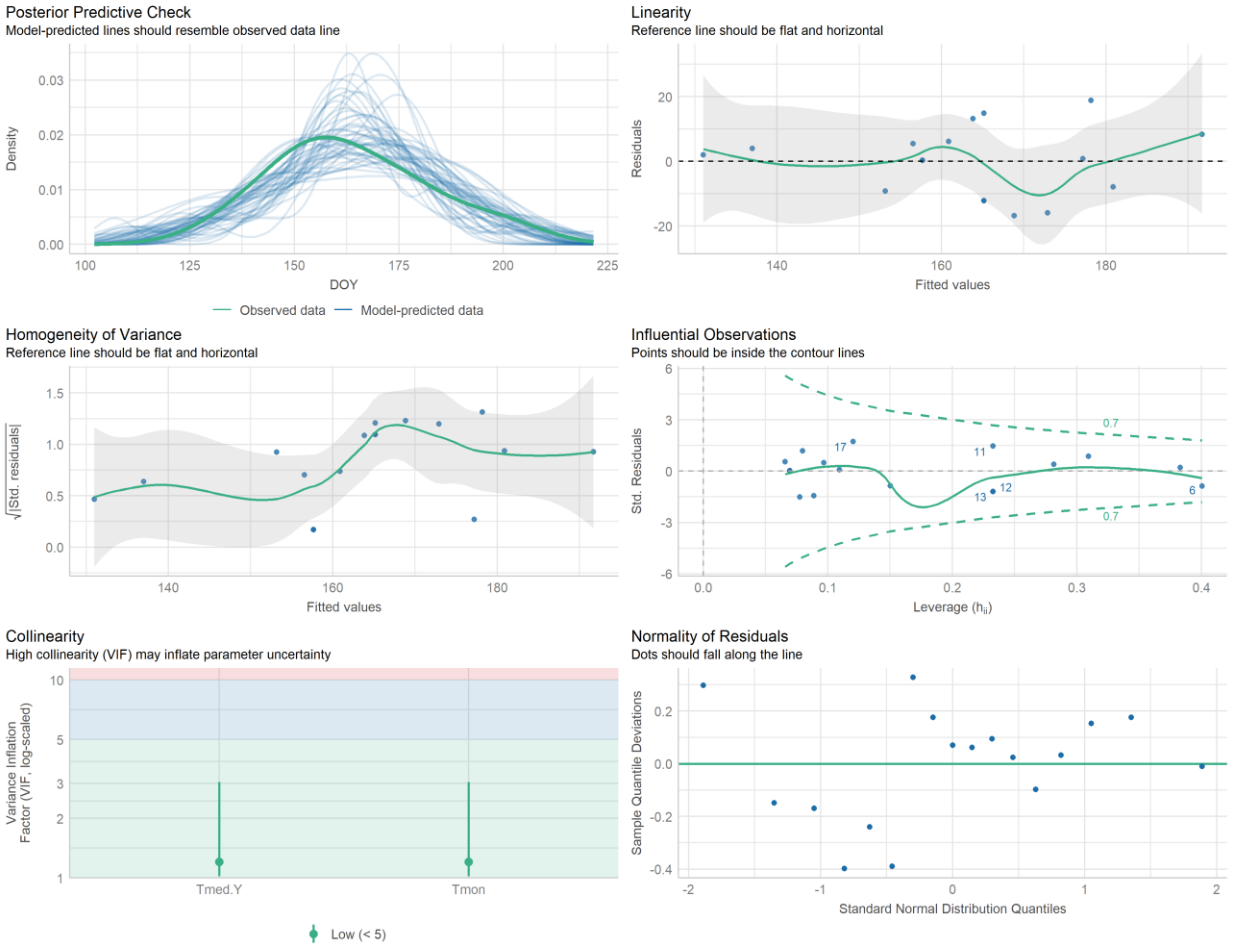

1.29. MLM - DVG - Cistus populifolius

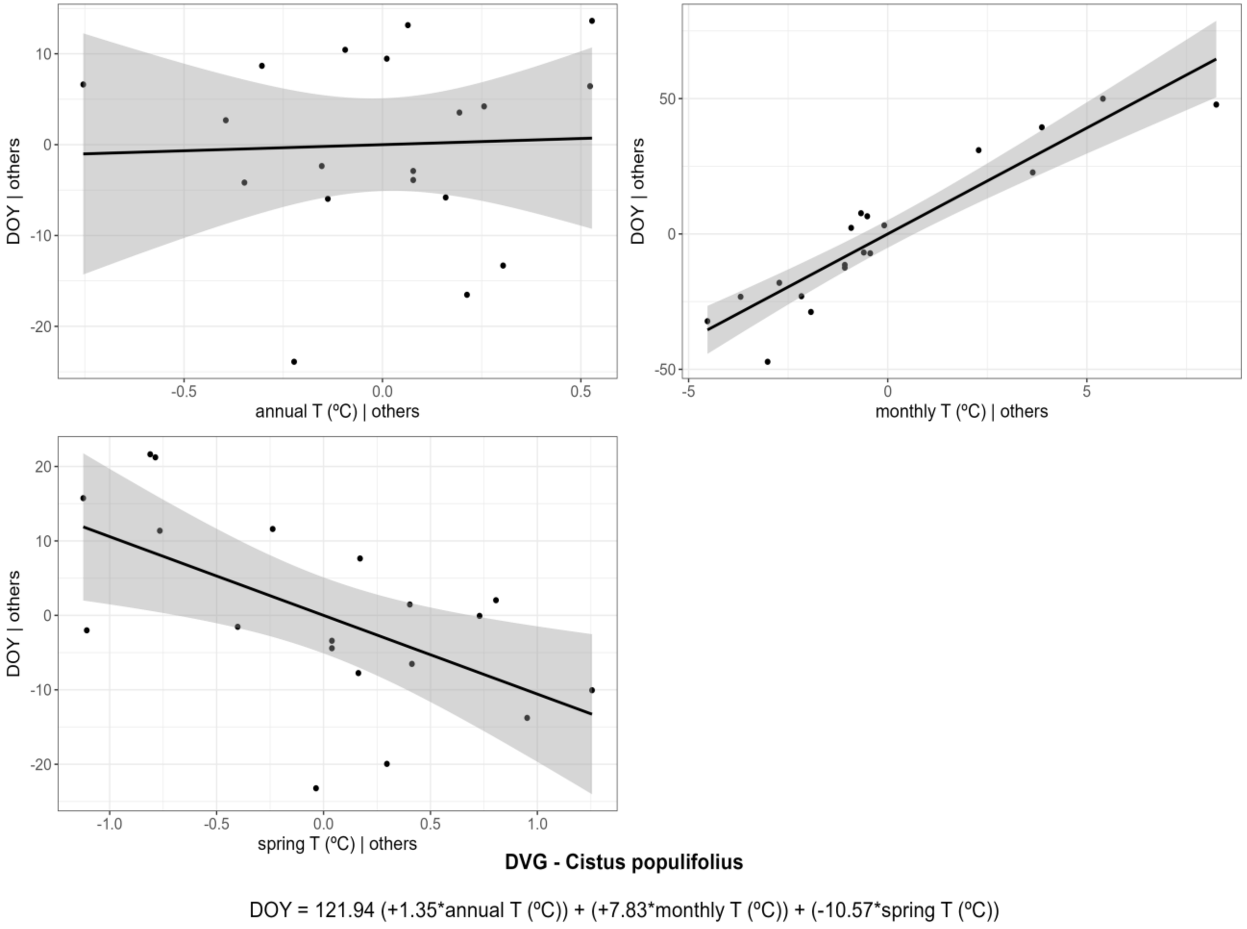

1.29.1. Diagnostics - MLM - DVG - Cistus populifolius

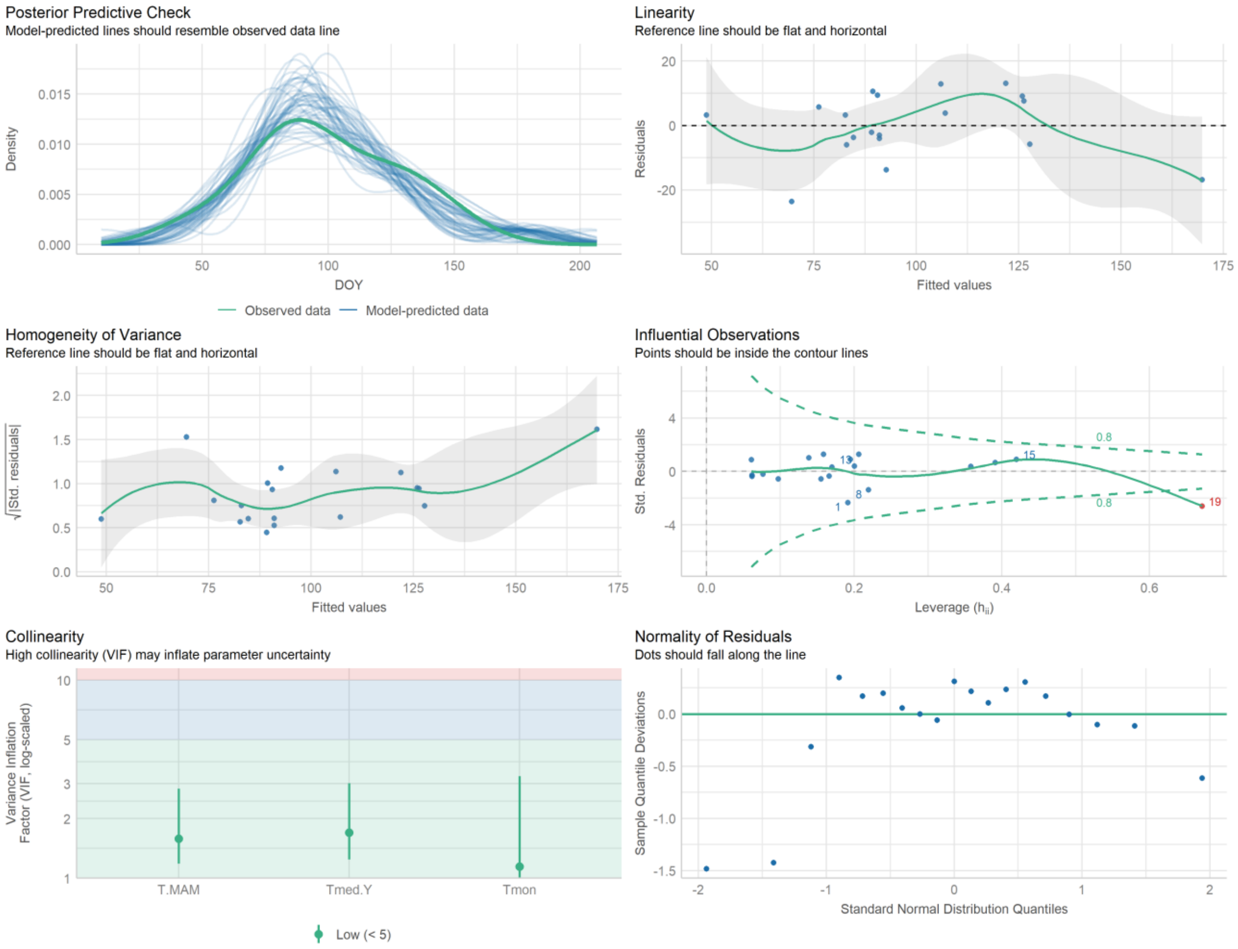

1.30. MLM - F - Crataegus granatensis

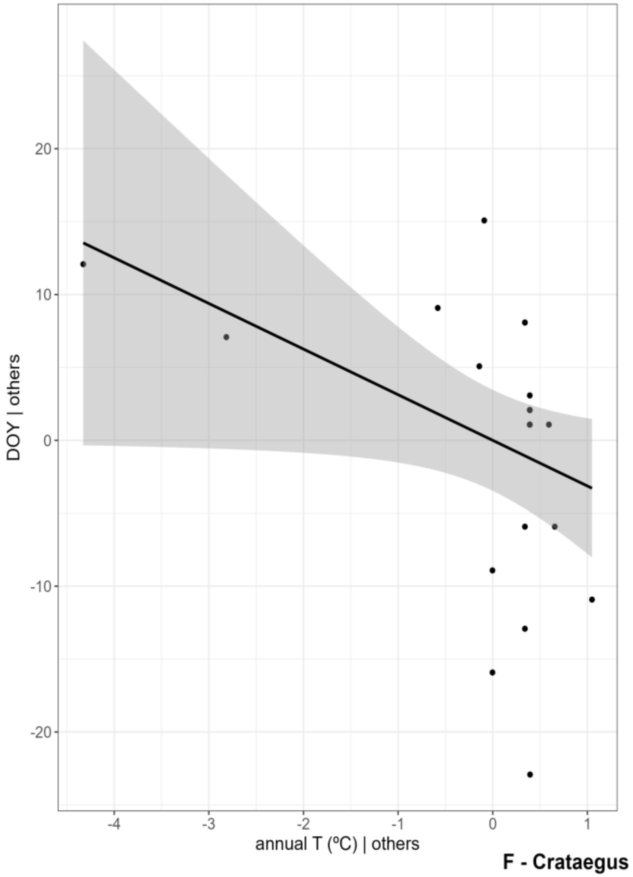

F - Crataegus granatensis

DOY = 213.95 (-3.13*annual T (ºC))

1.30.1. Diagnostics - MLM - F - Crataegus granatensis

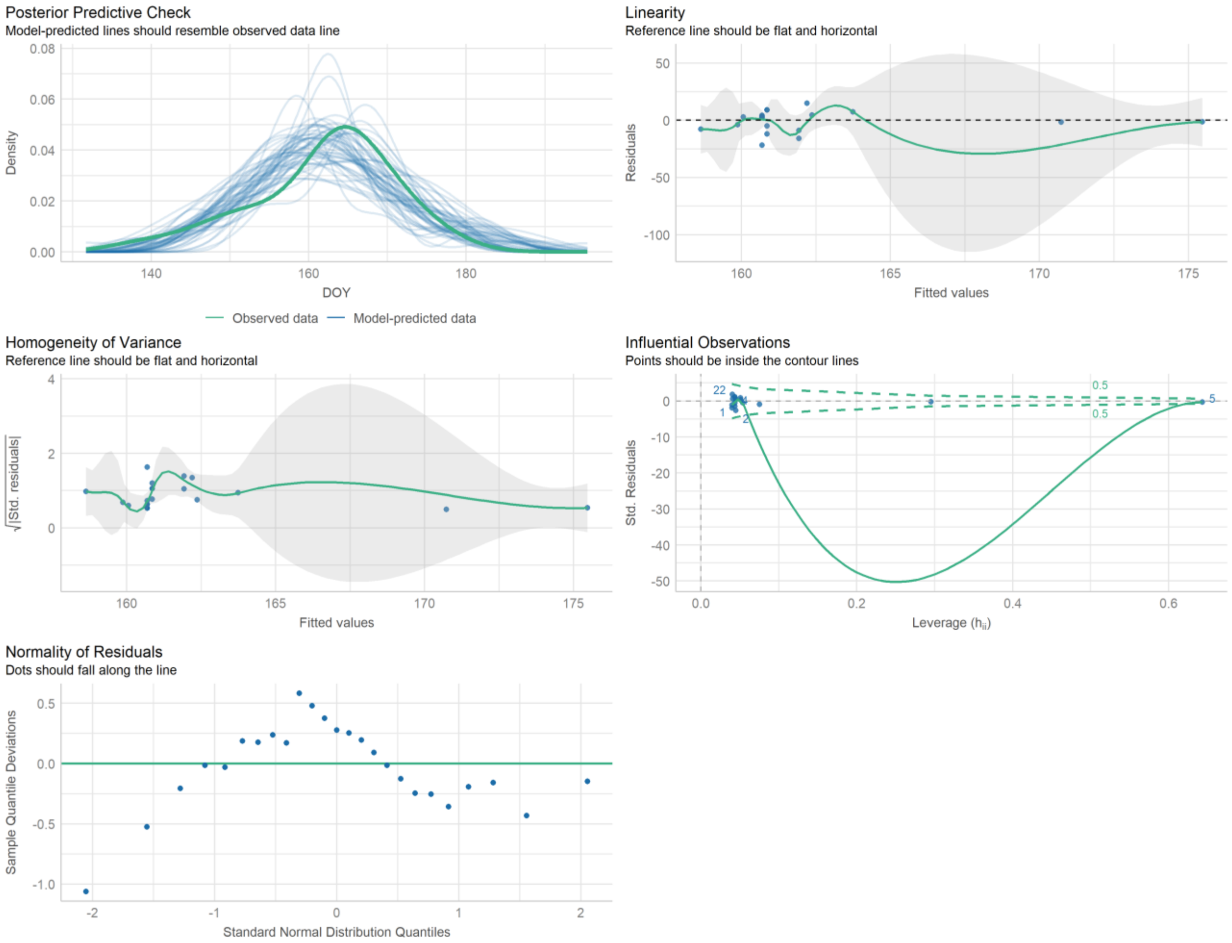

1.31. MLM - FS - Crataegus granatensis

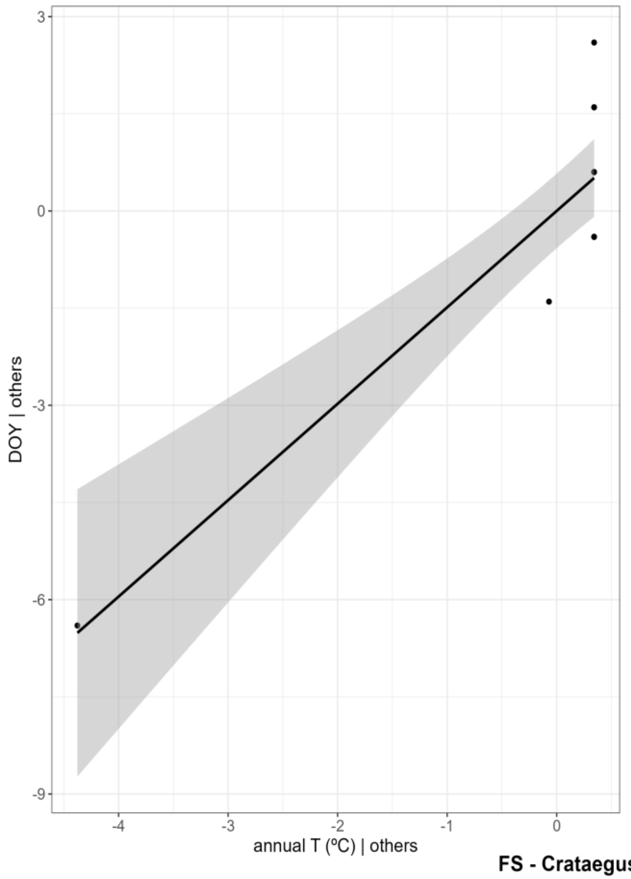

FS - Crataegus granatensis

DOY = 138.59 (+1.49*annual T (ºC))

1.31.1. Diagnostics - MLM - FS - Crataegus granatensis

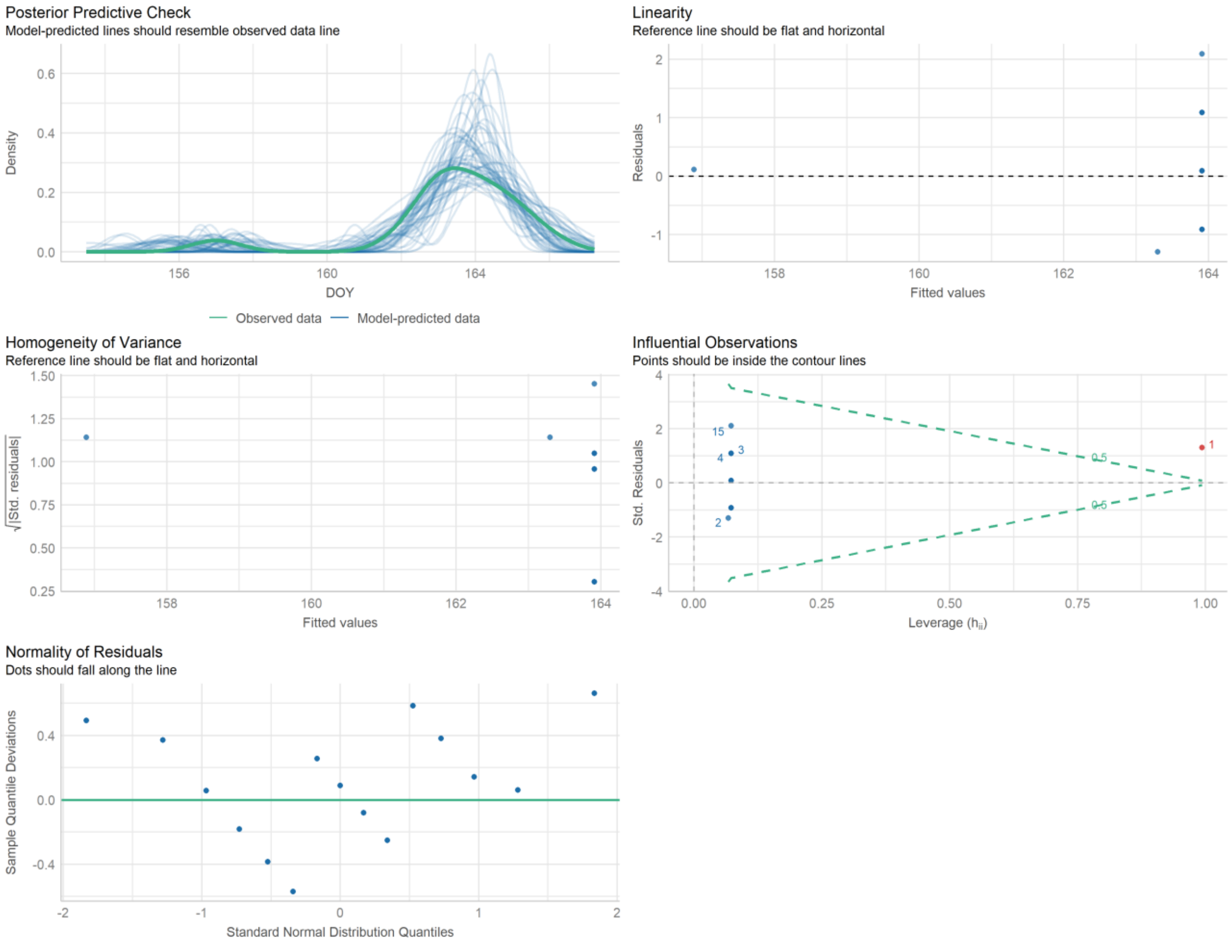

1.32. MLM - FBF - Crataegus monogyna

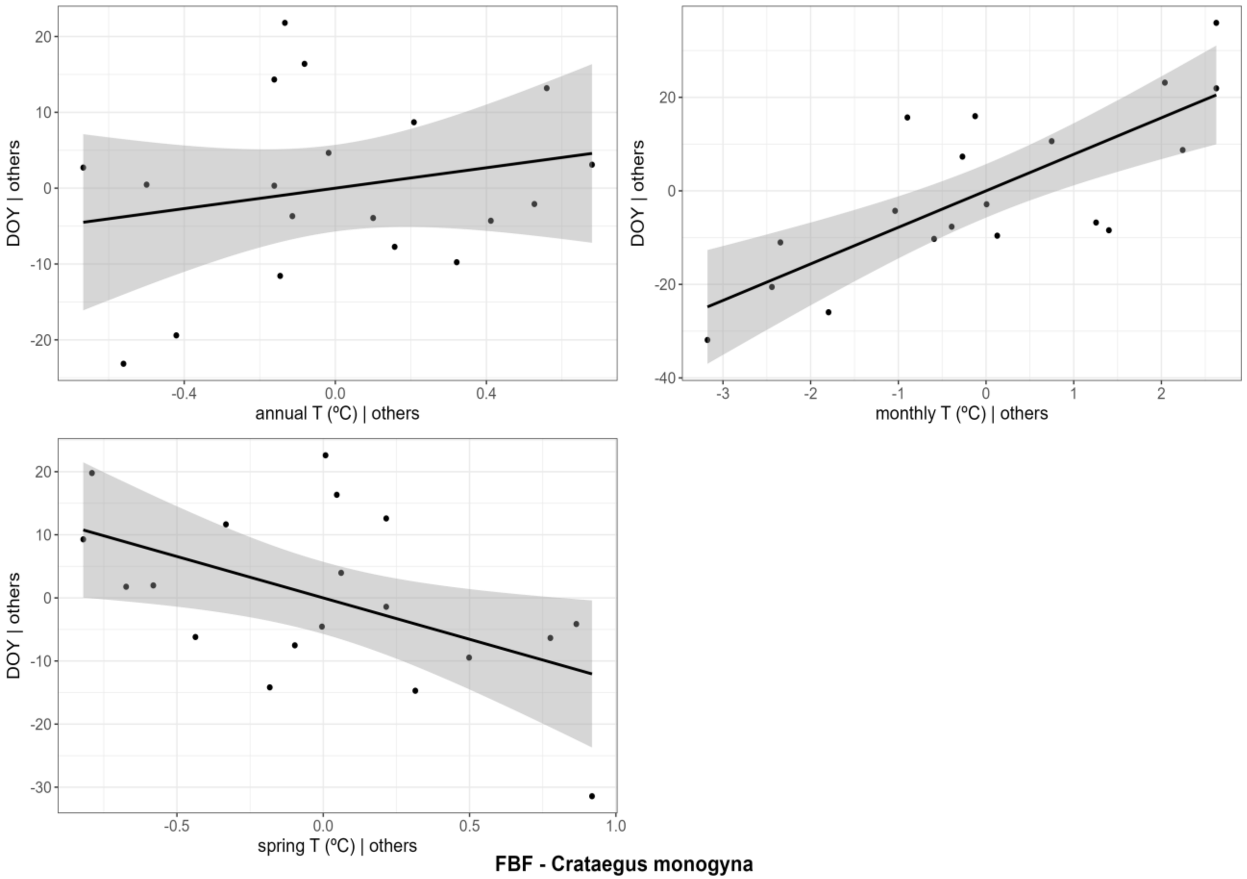

DOY = 69.89 (+6.74*annual T (ºC)) + (+7.81*monthly T (ºC)) + (-13.12*spring T (ºC))

1.32.1. Diagnostics - MLM - FBF - Crataegus monogyna

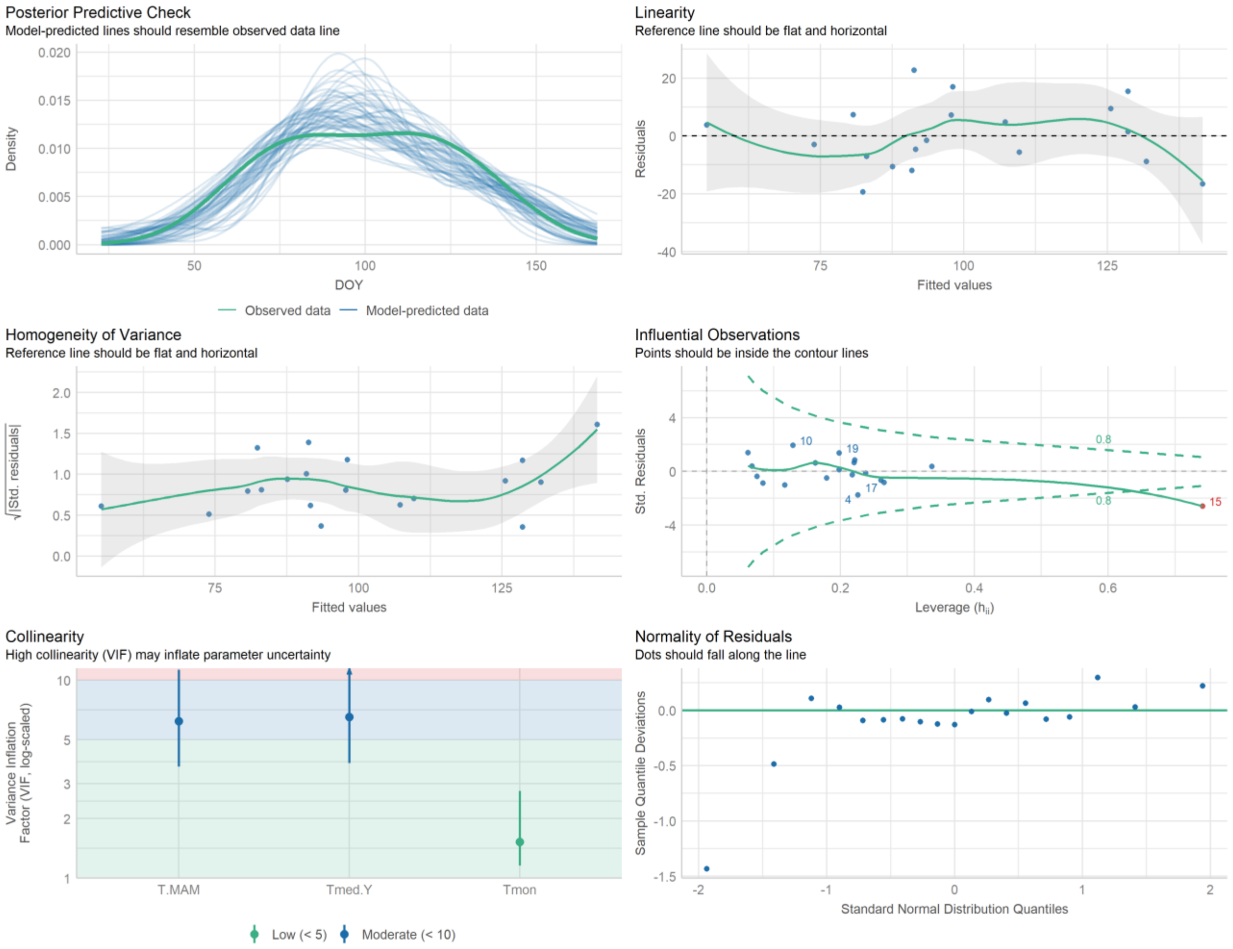

1.33. MLM - F - Crataegus monogyna

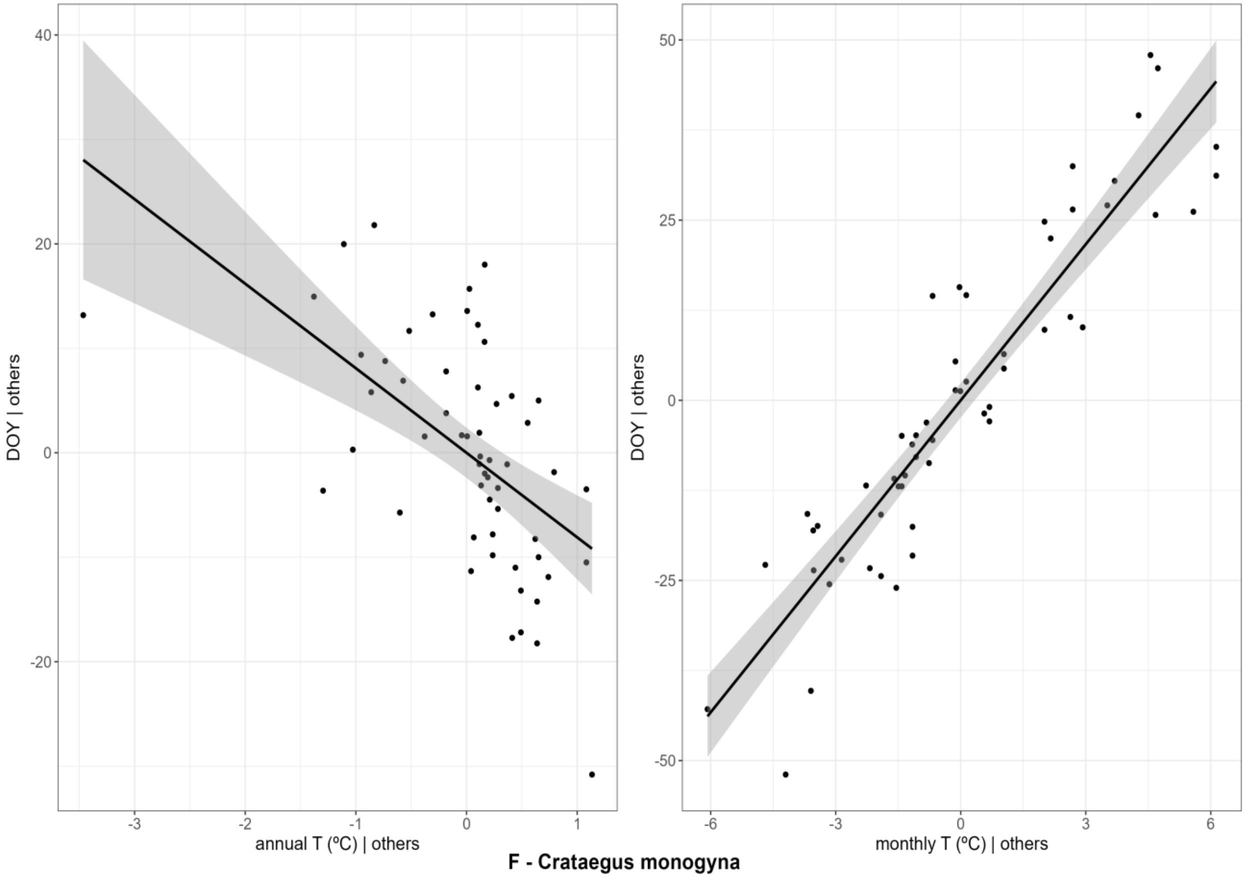

DOY = 142.27 (-8.09*annual T (ºC)) + (+7.22*monthly T (ºC))

1.33.1. Diagnostics - MLM - F - Crataegus monogyna

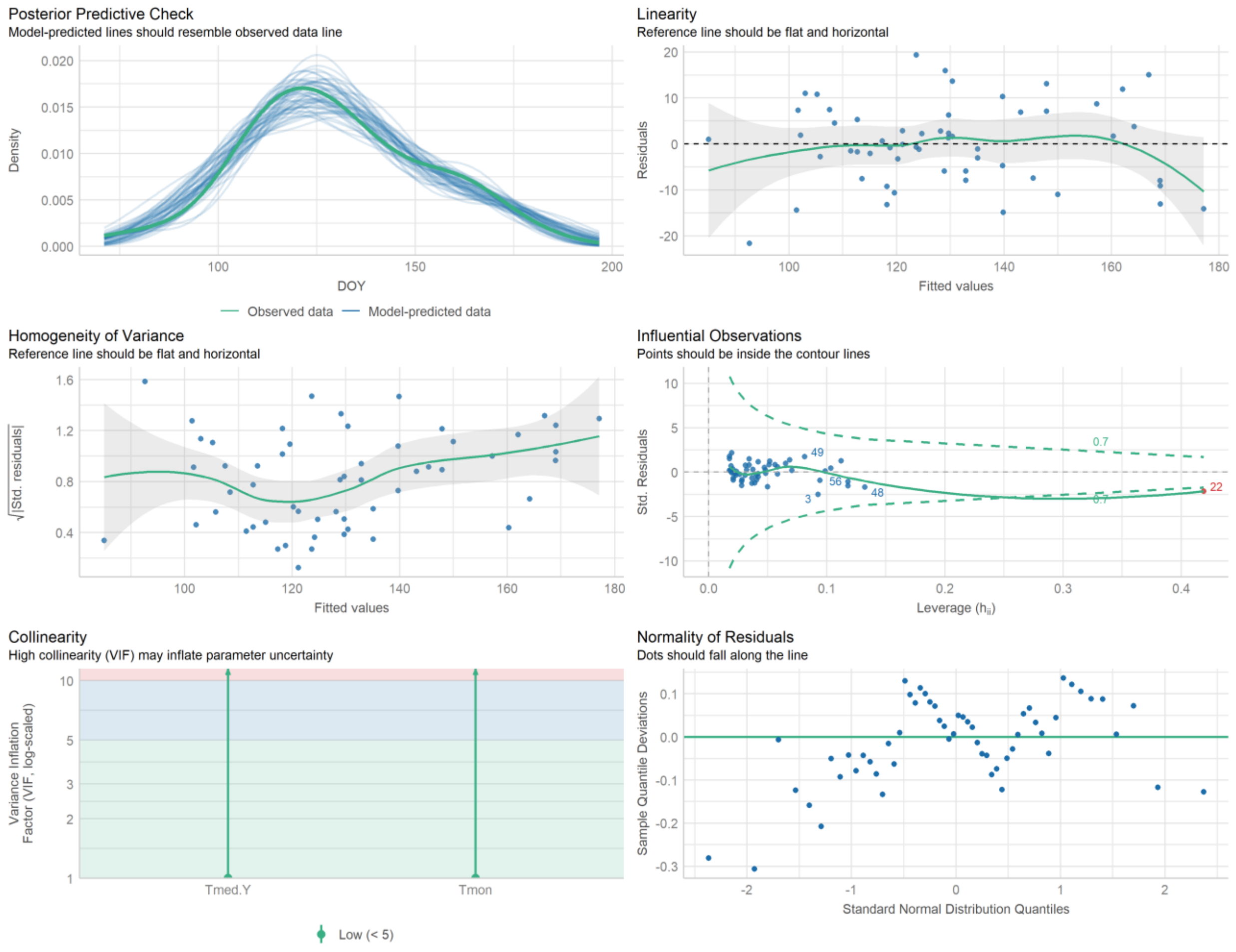

1.34. MLM - F - Crepis oporinoides

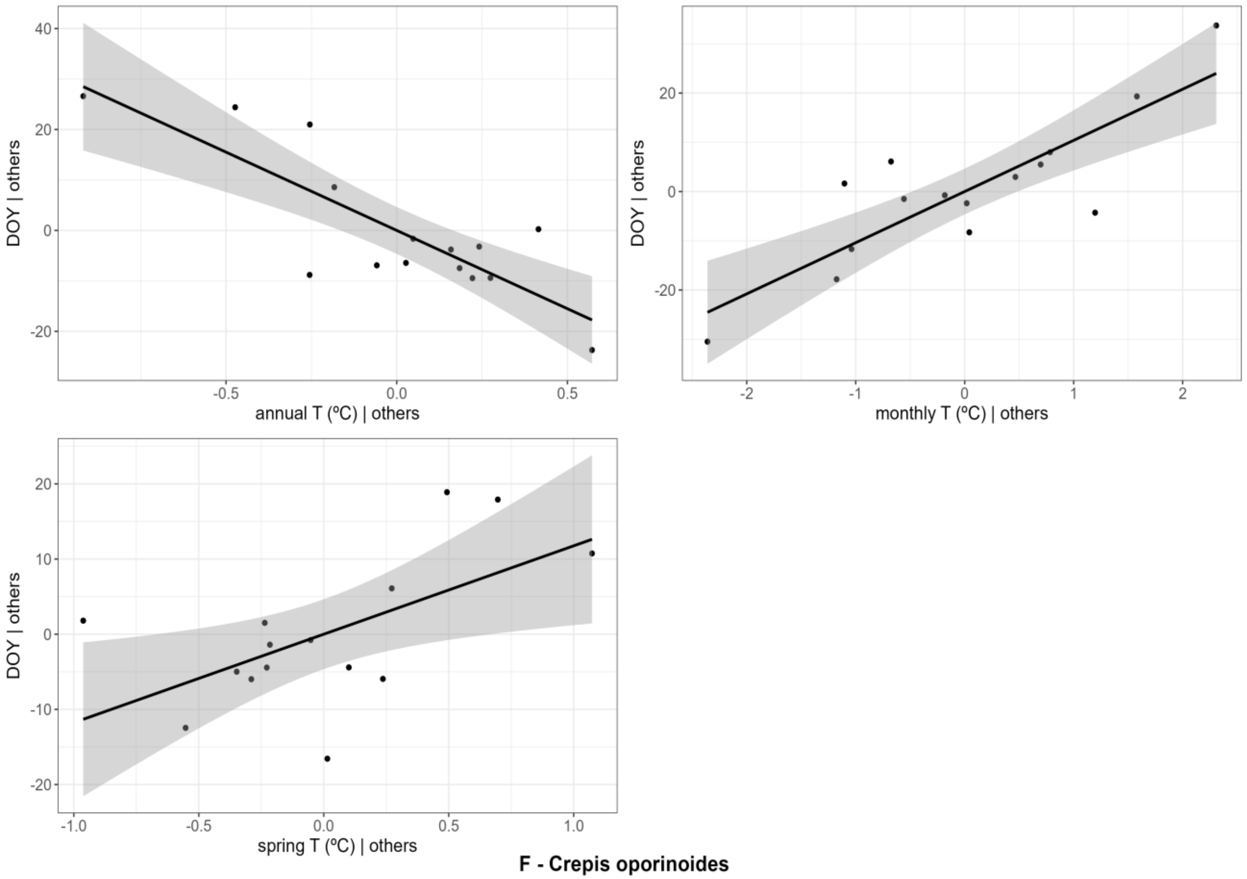

F - Crepis oporinoides

DOY = 272.90 (-31.02*annual T (ºC)) + (+10.39*monthly T (ºC)) + (+11.76*spring T (ºC))

1.34.1. Diagnostics - MLM - F - Crepis oporinoides

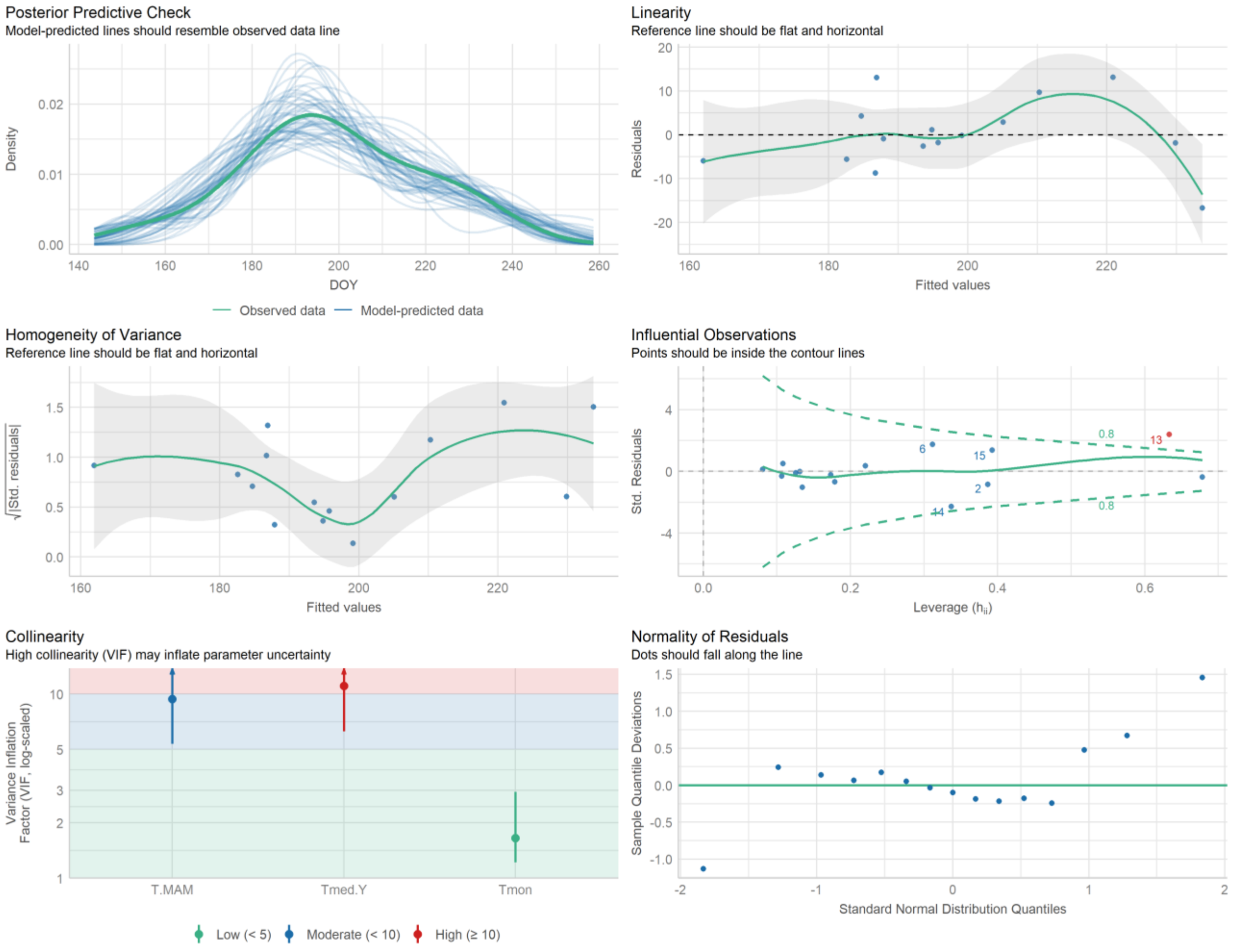

1.35. MLM - F - Cytisus malacitanus

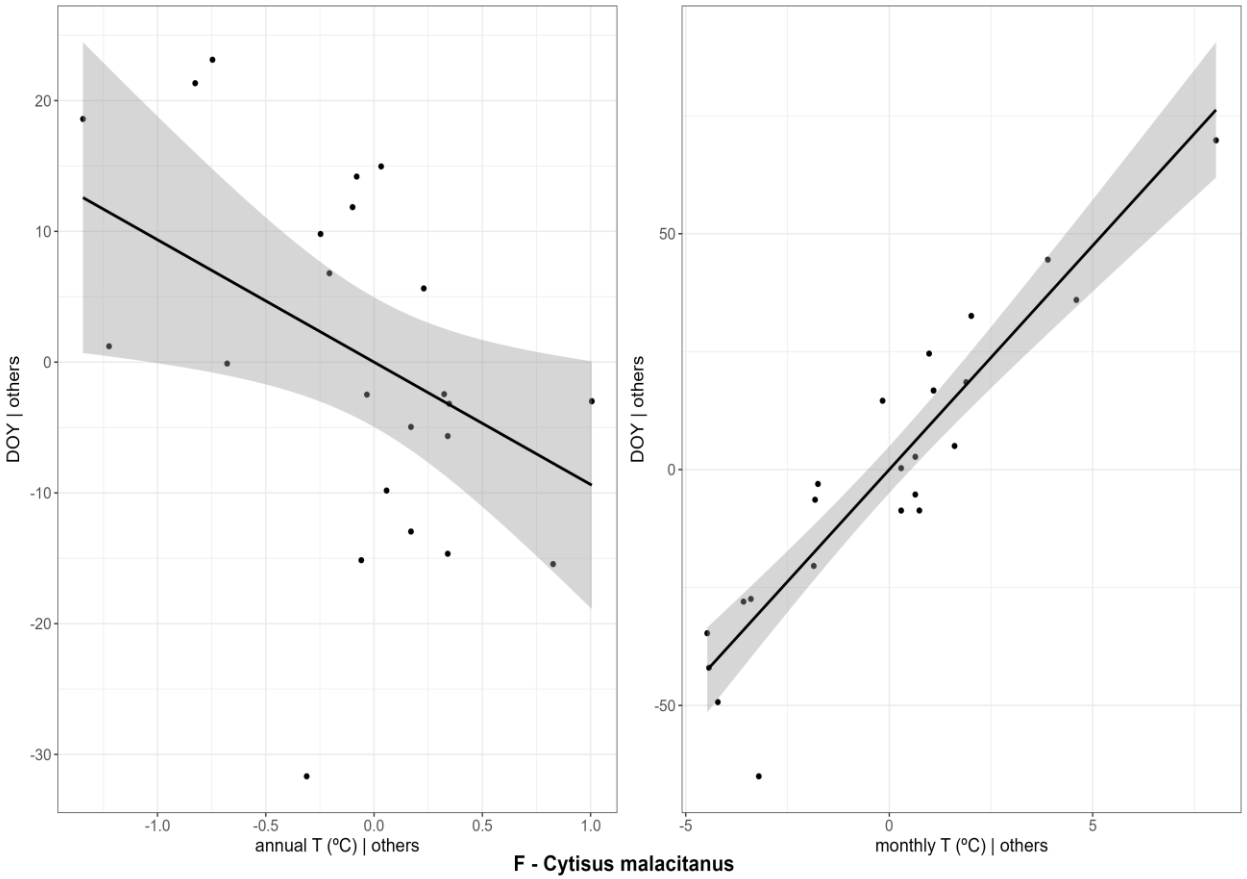

DOY = 116.92 (-9.36*annual T (ºC)) + (+9.50*monthly T (ºC))

1.35.1. Diagnostics - MLM - F - Cytisus malacitanus

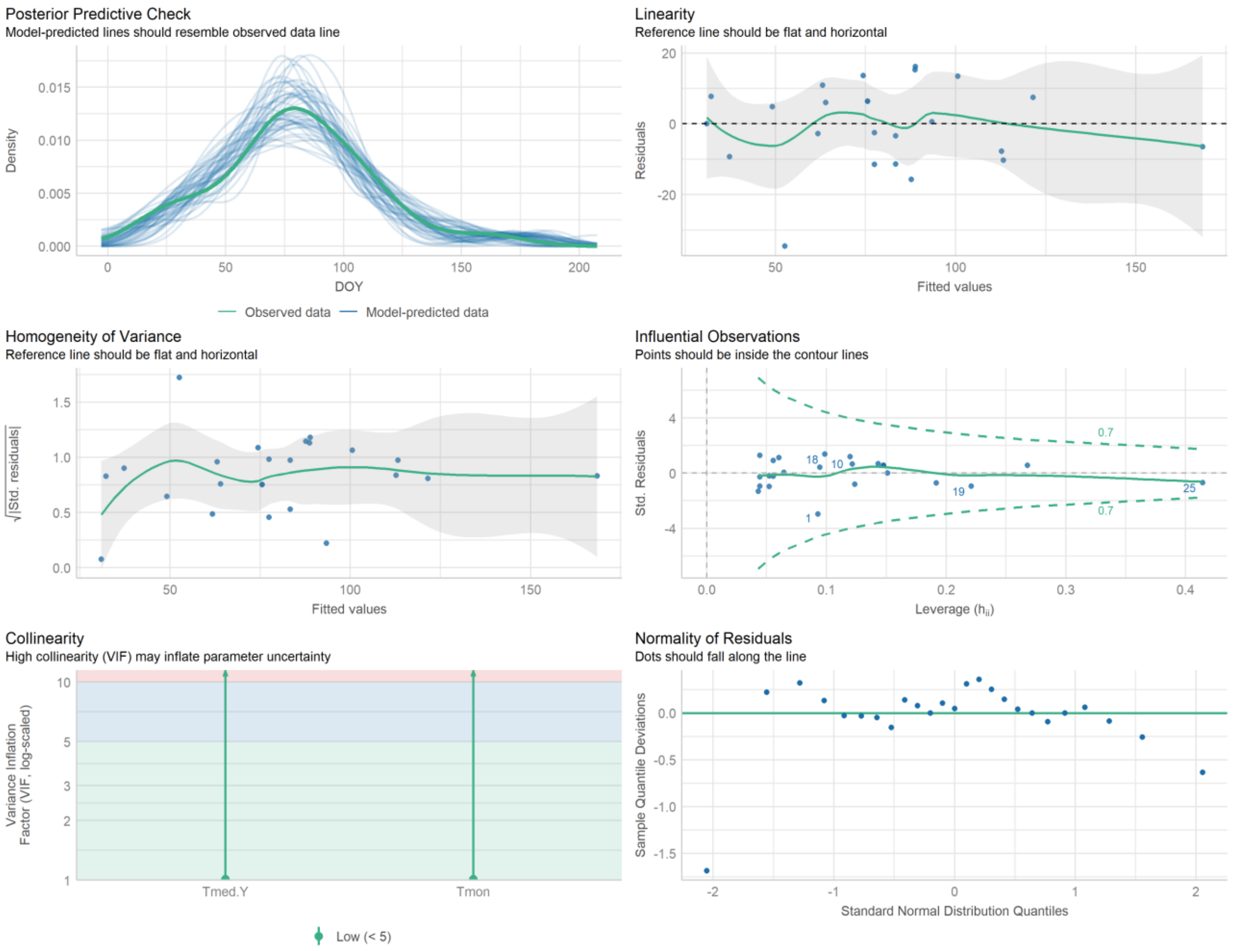

1.36. MLM - FS - Cytisus malacitanus

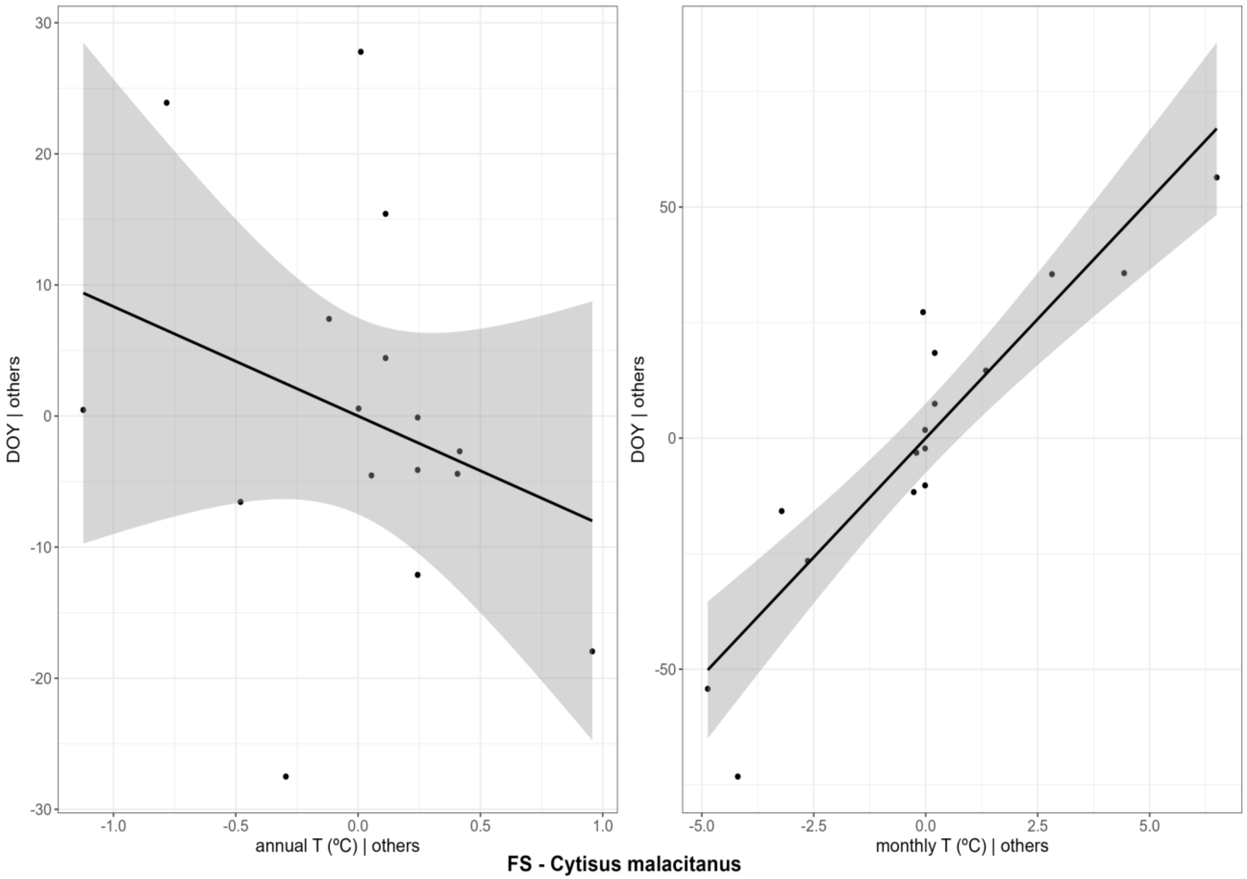

DOY = 88.11 (-8.35*annual T (ºC)) + (+10.31*monthly T (ºC))

1.36.1. Diagnostics - MLM - FS - Cytisus malacitanus

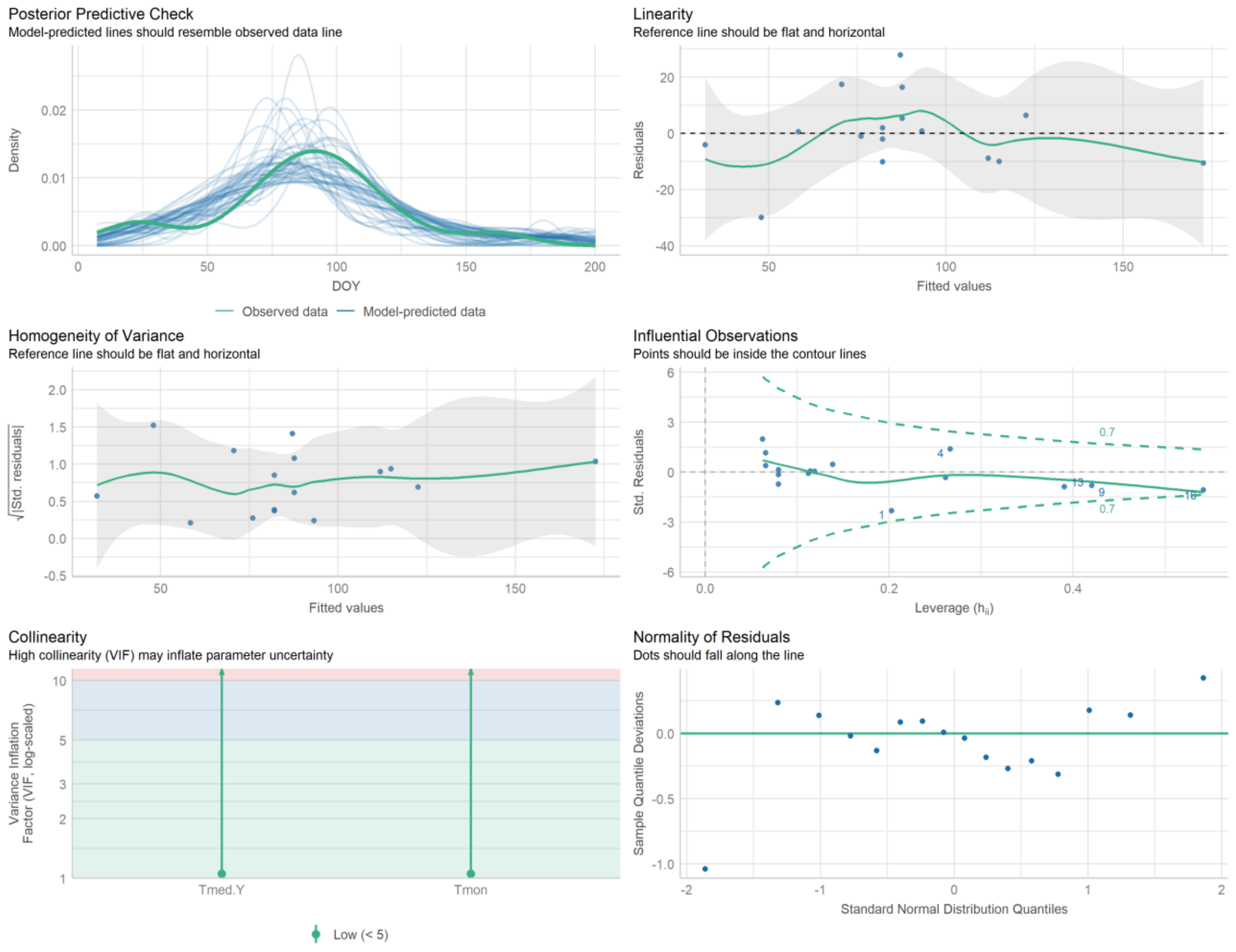

1.37. MLM - F - Echium albicans

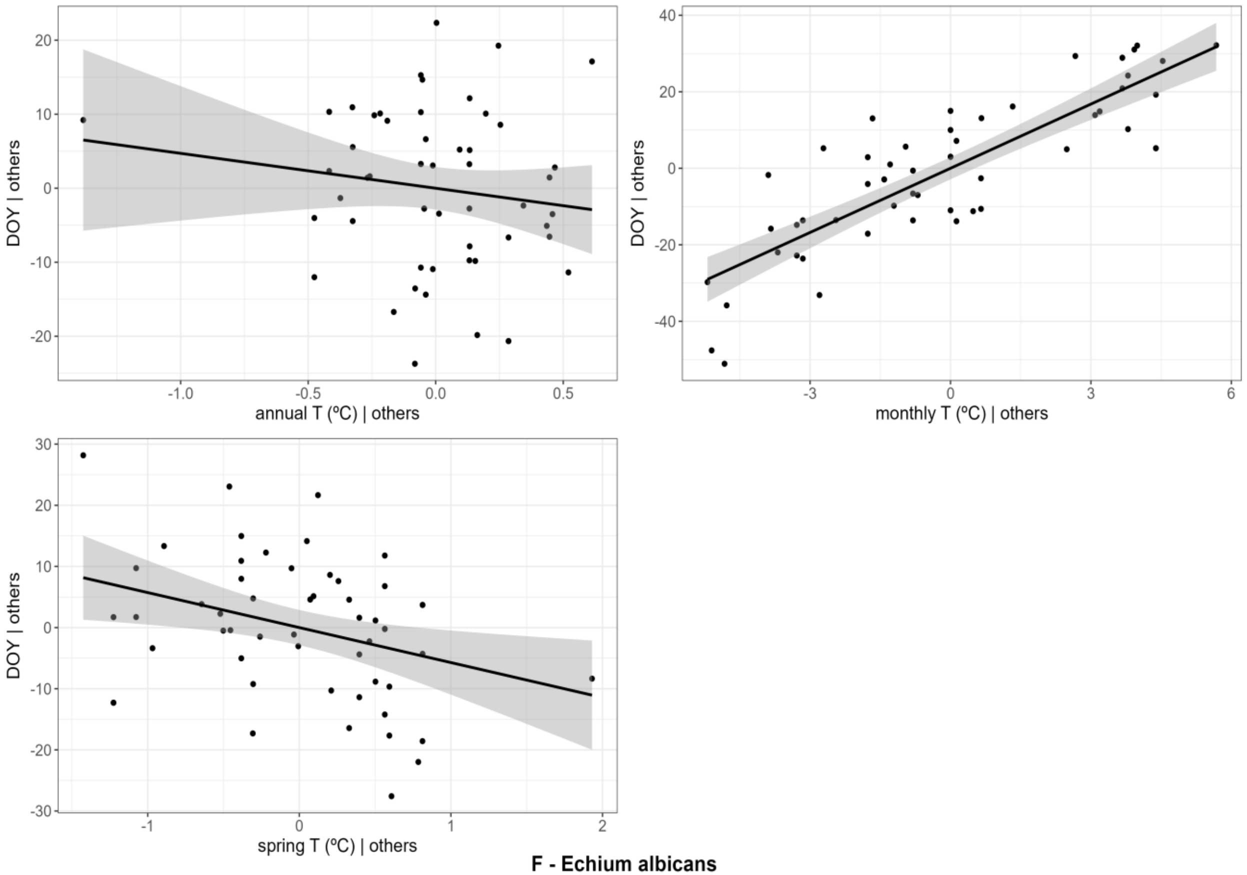

F - Echium albicans

DOY = 197.66 (-4.71*annual T (ºC)) + (+5.59*monthly T (ºC)) + (-5.72*spring T (ºC))

1.37.1. Diagnostics - MLM - F - Echium albicans

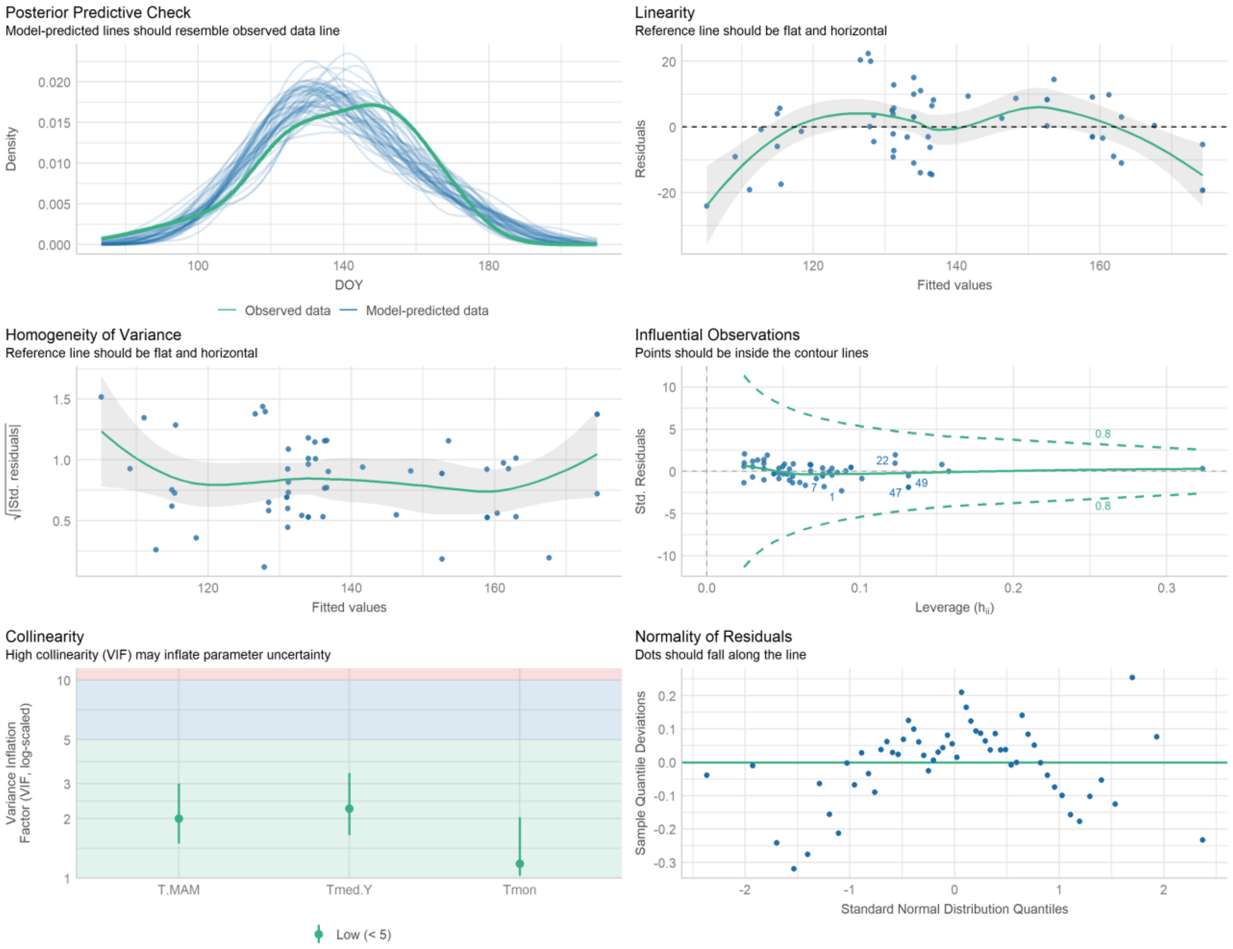

1.38. MLM - F - Elaeoselinum asclepium subsp. millefolium

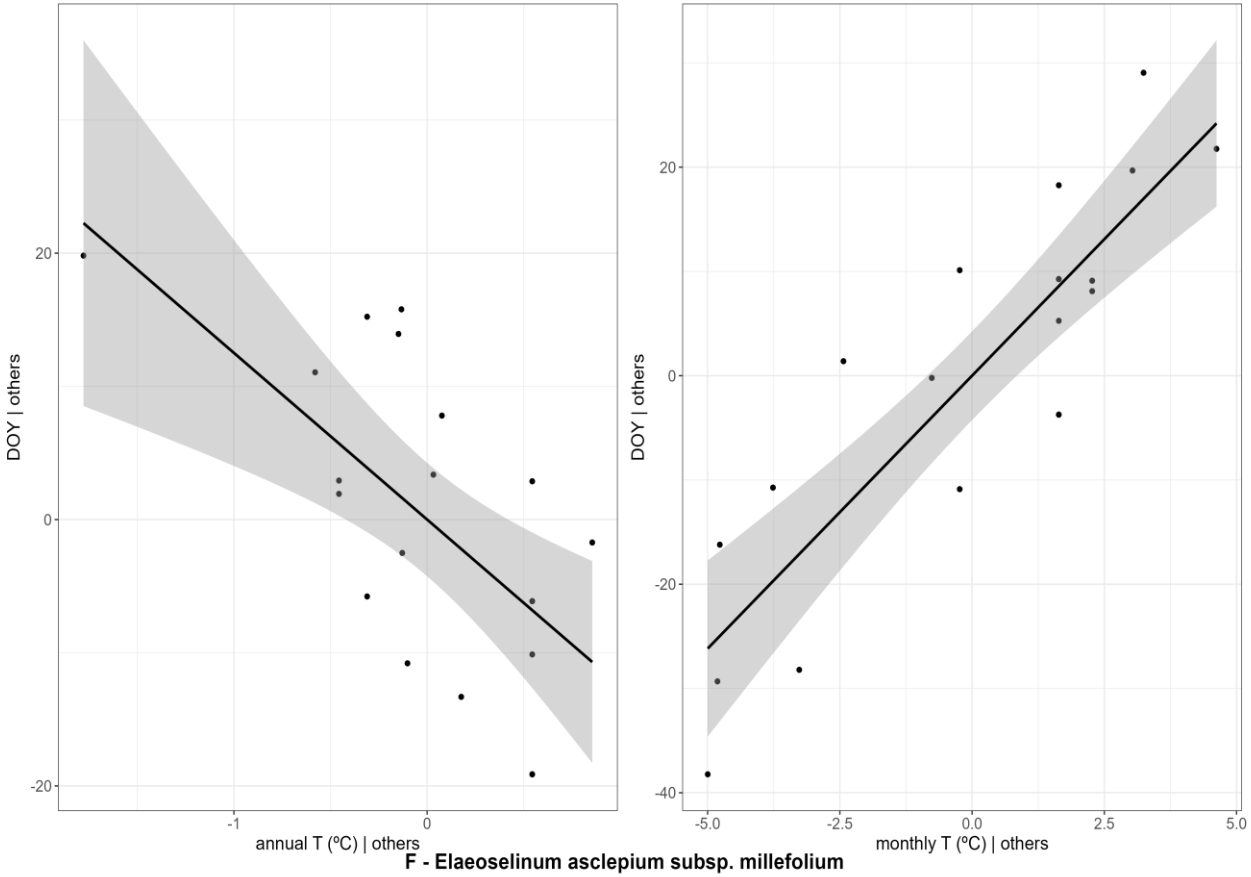

DOY = 260.54 (-12.51*annual T (ºC)) + (+5.24*monthly T (ºC))

1.38.1. Diagnostics - MLM - F - Elaeoselinum asclepium subsp. millefolium

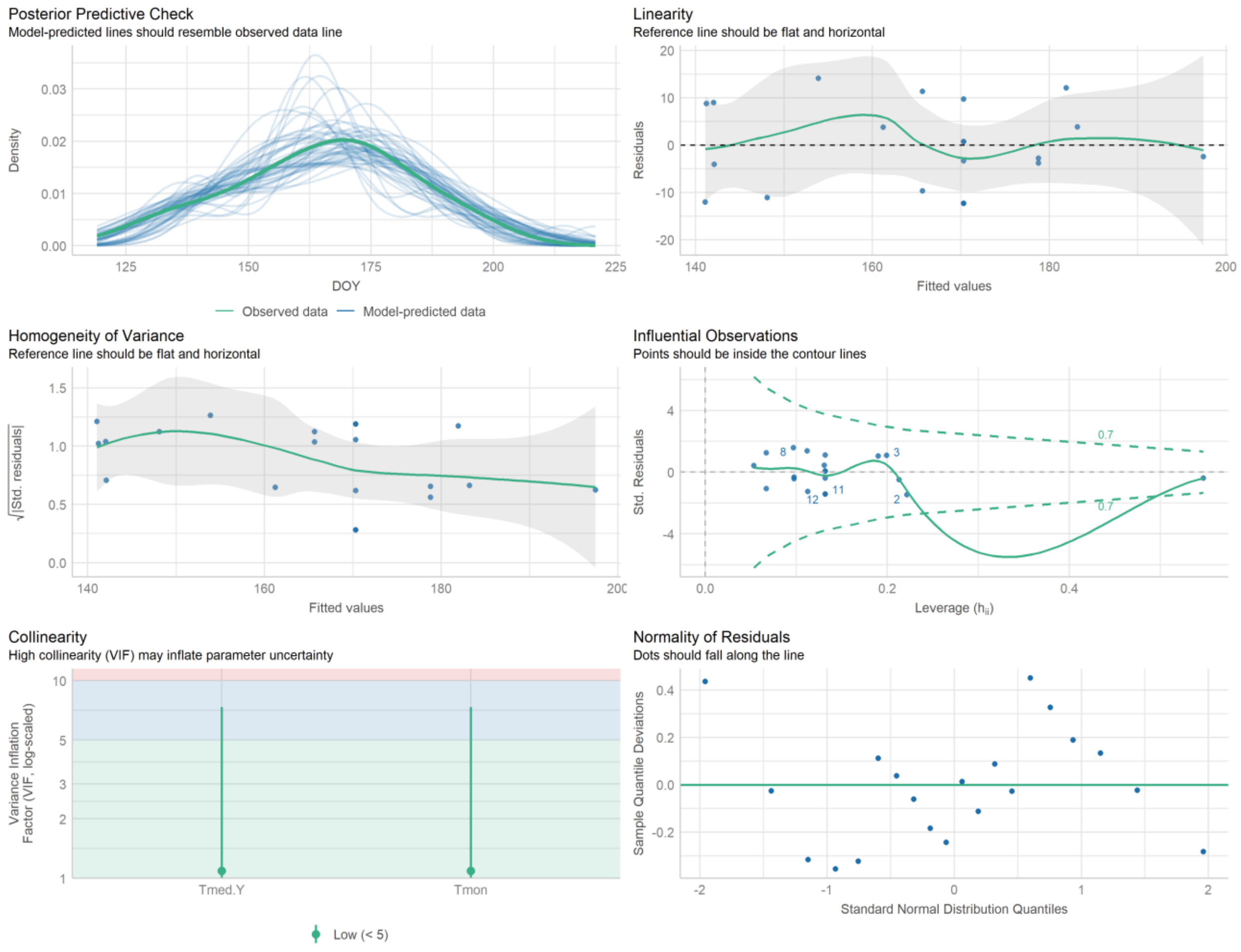

1.39. MLM - FBF - Erica arborea

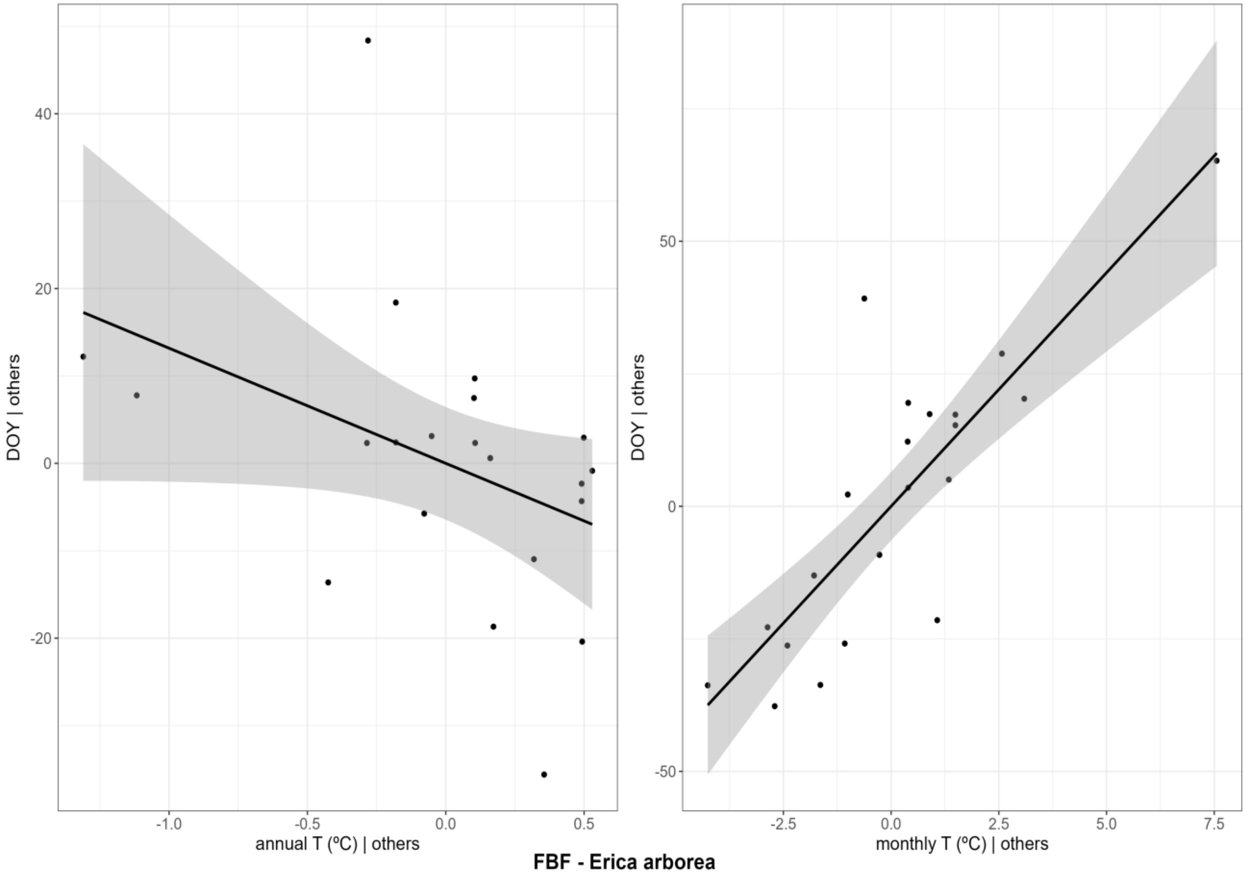

FBF - Erica arborea

DOY = 180.55 (-13.18*annual T (ºC)) + (+8.81*monthly T (ºC))

1.39.1. Diagnostics - MLM - FBF - Erica arborea

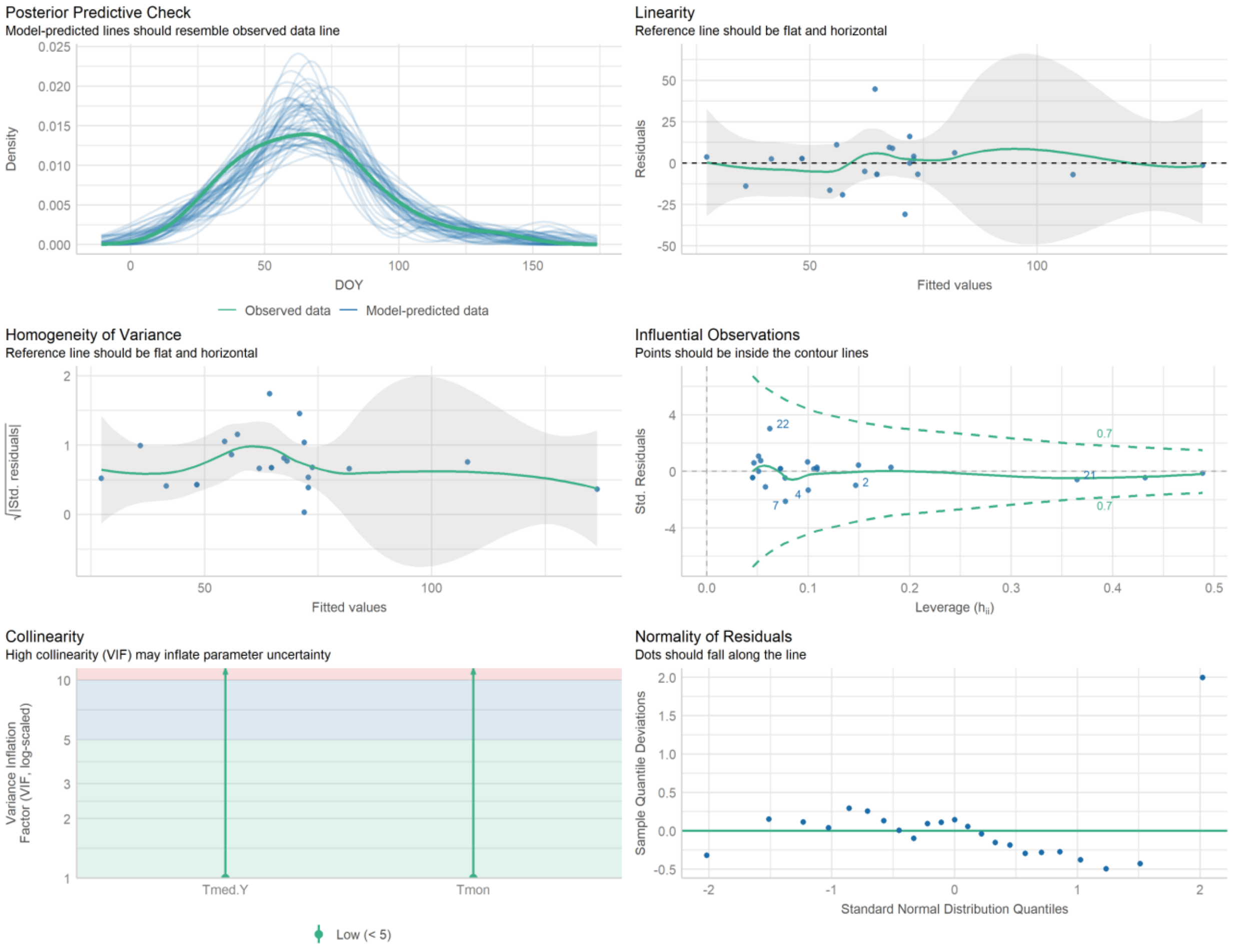

1.40. MLM - F - Erica arborea

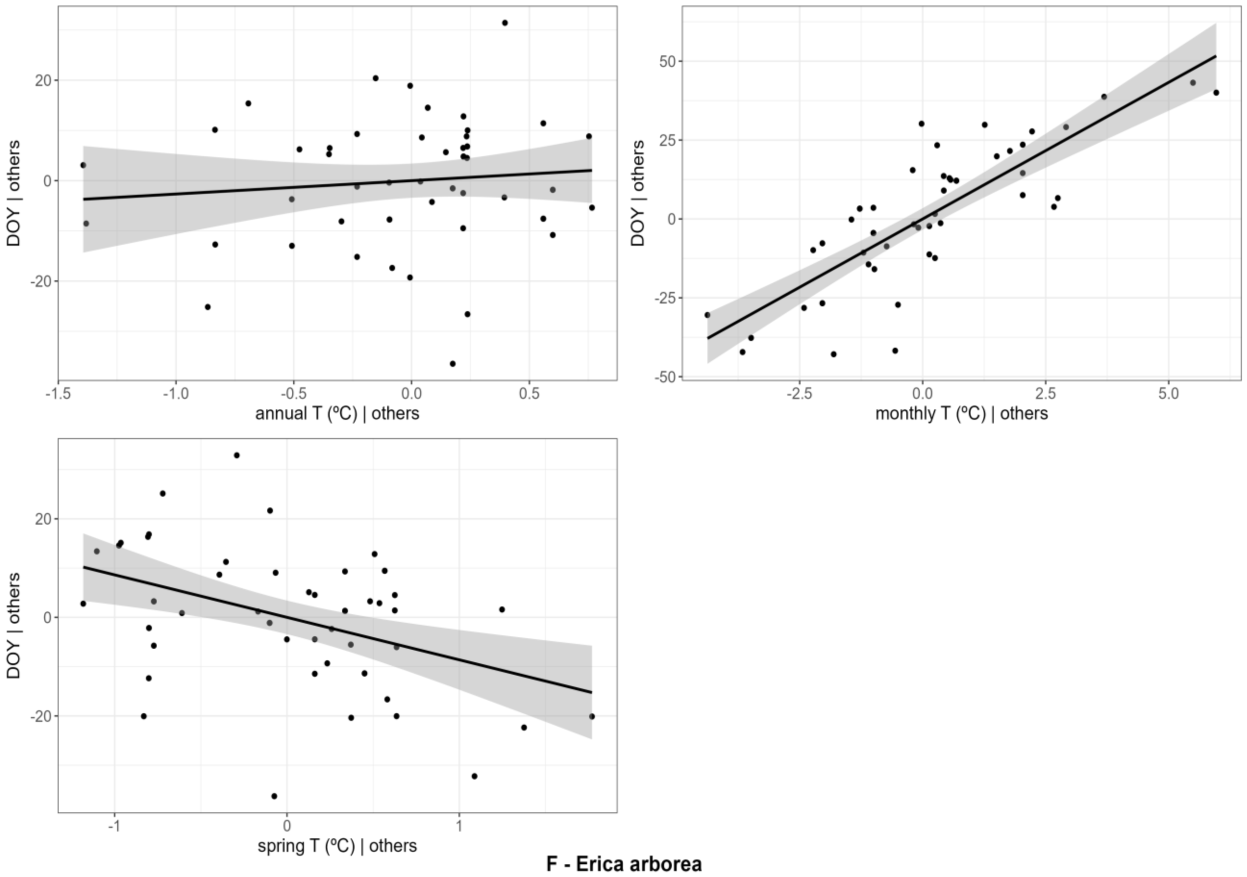

DOY = 57.78 (+2.66*annual T (ºC)) + (+8.66*monthly T (ºC)) + (-8.62*spring T (ºC))

1.40.1. Diagnostics - MLM - F - Erica arborea

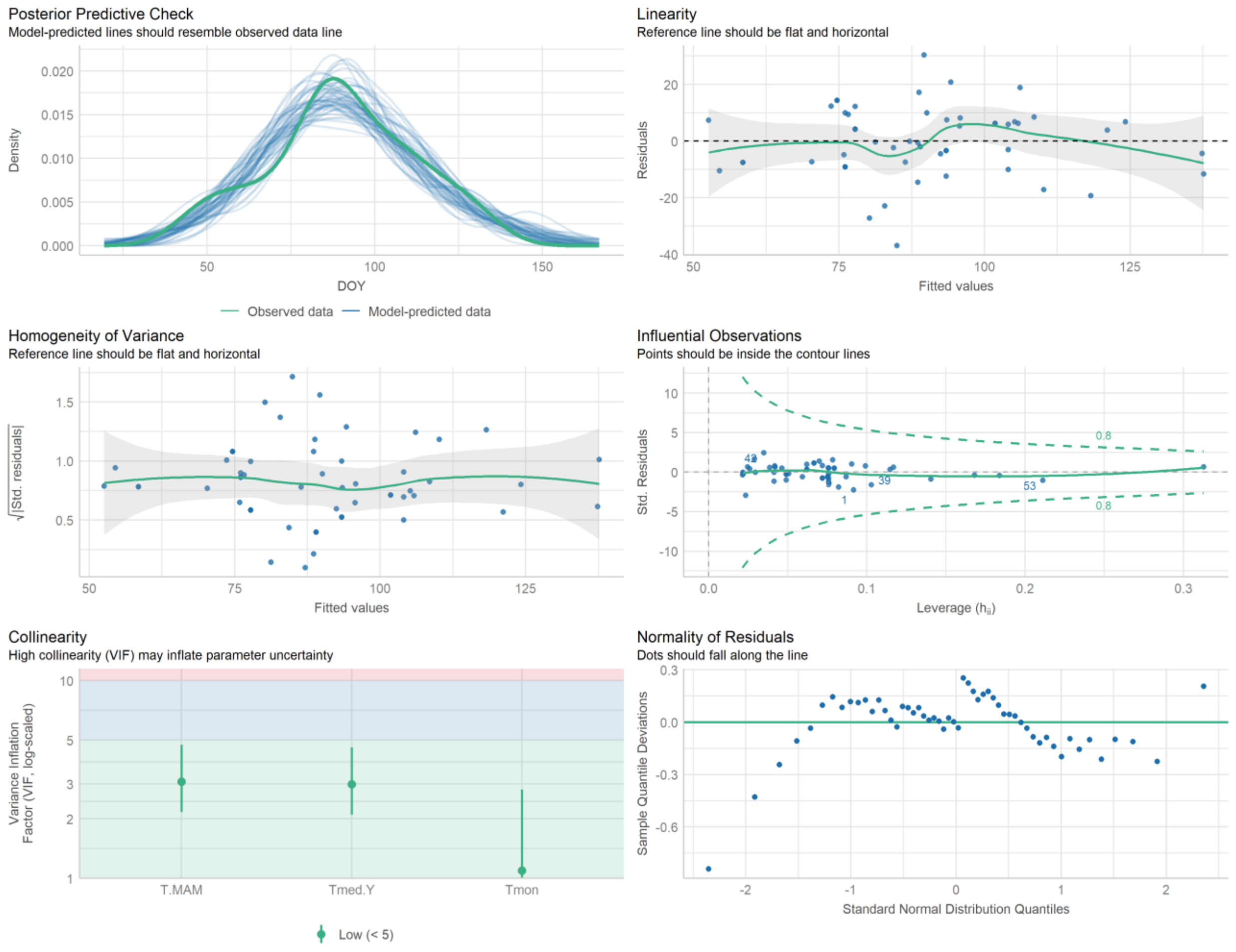

1.41. MLM - FBF - Erica scoparia

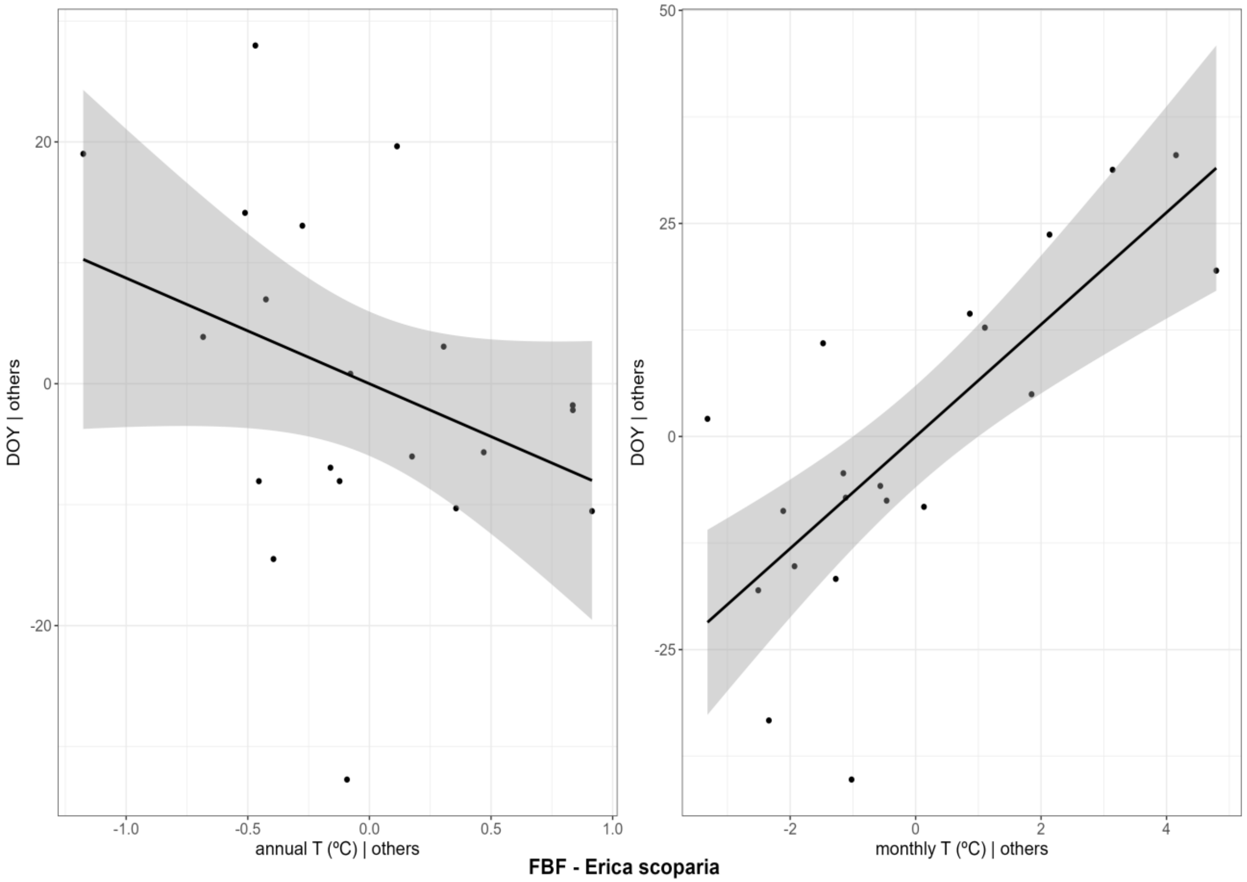

DOY = 153.17 (-8.74*annual T (ºC)) + (+6.57*monthly T (ºC))

1.41.1. Diagnostics - MLM - FBF - Erica scoparia

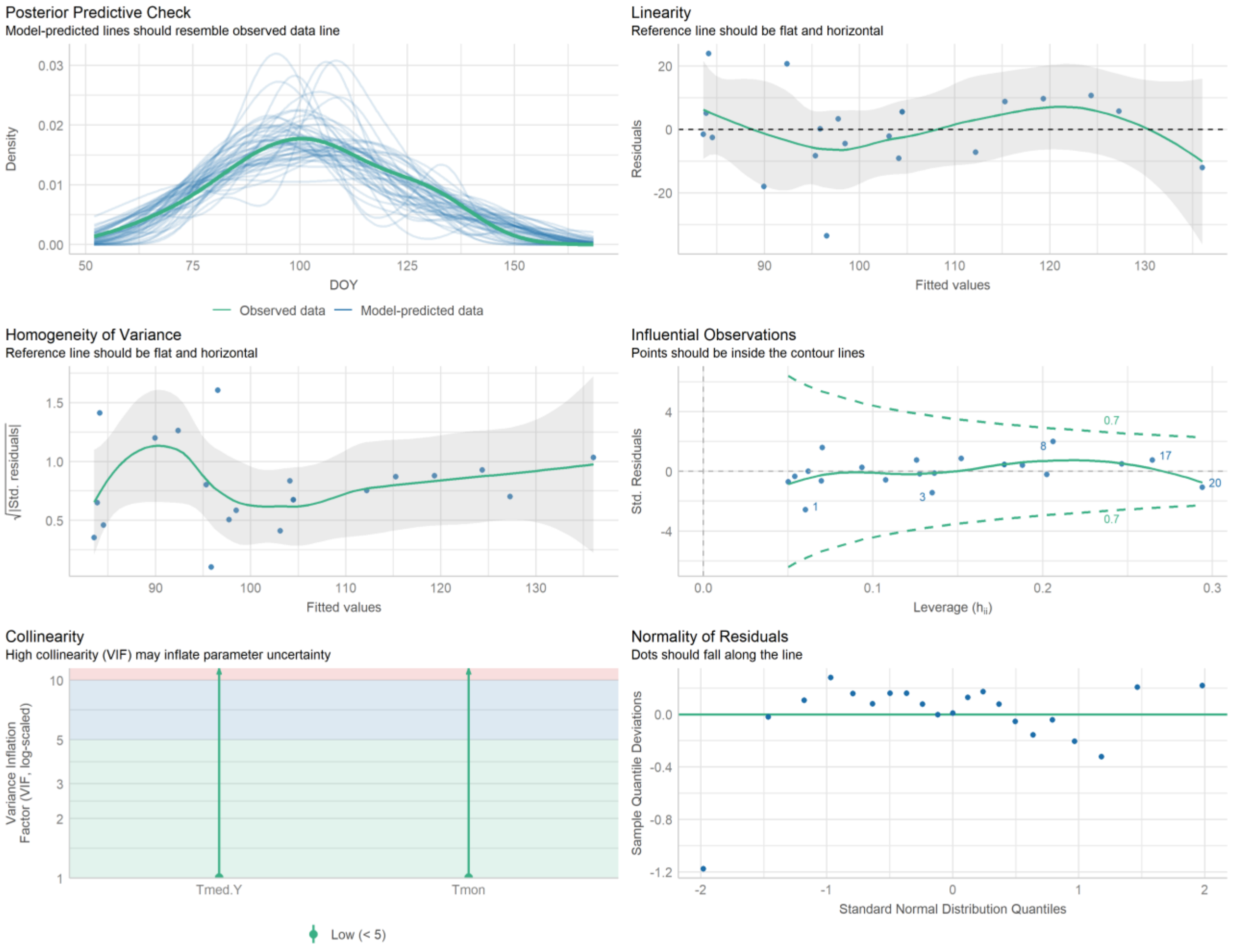

1.42. MLM - F - Erica scoparia

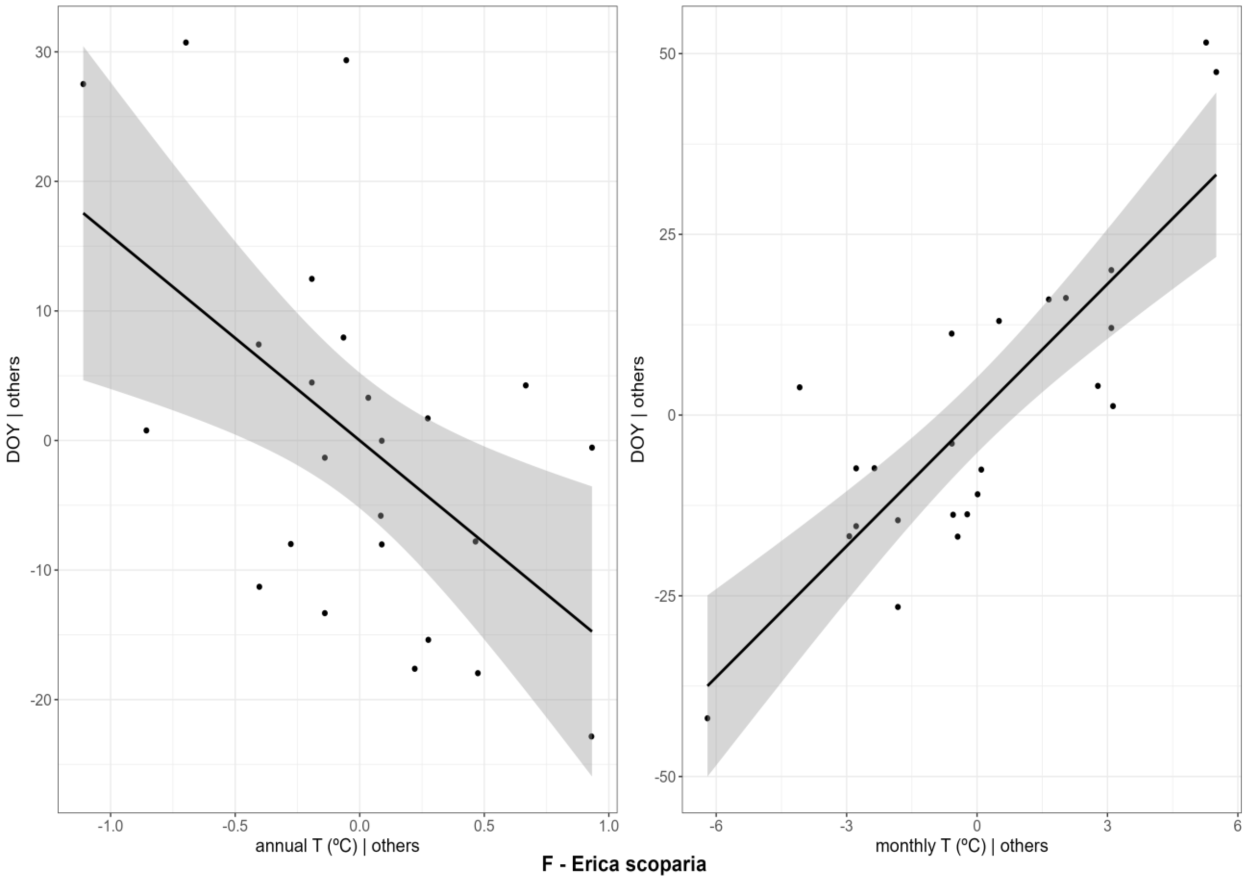

DOY = 294.10 (-15.81*annual T (ºC)) + (+6.04*monthly T (ºC))

1.42.1. Diagnostics - MLM - F - Erica scoparia

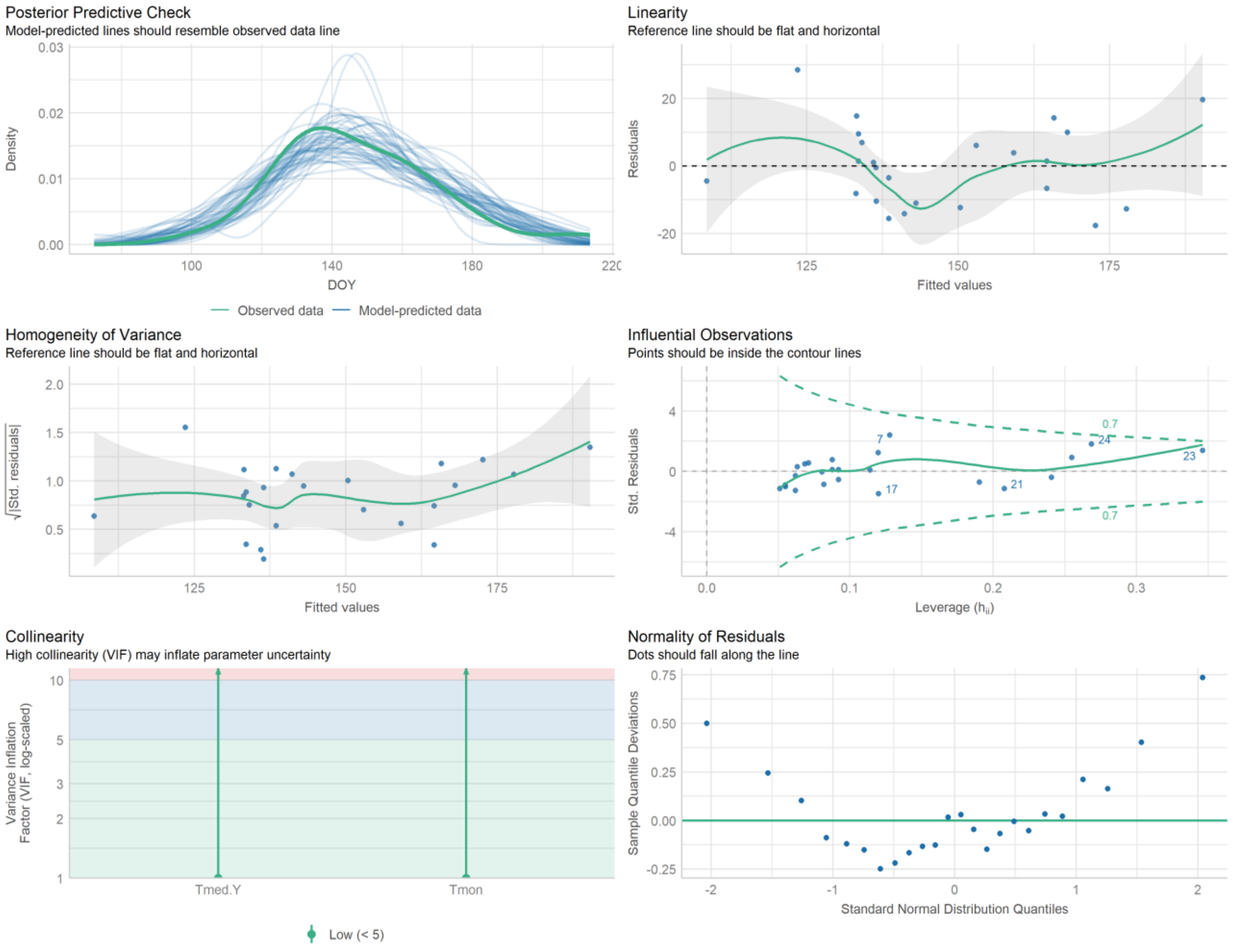

1.43. MLM - DVG - Erinacea anthyllis

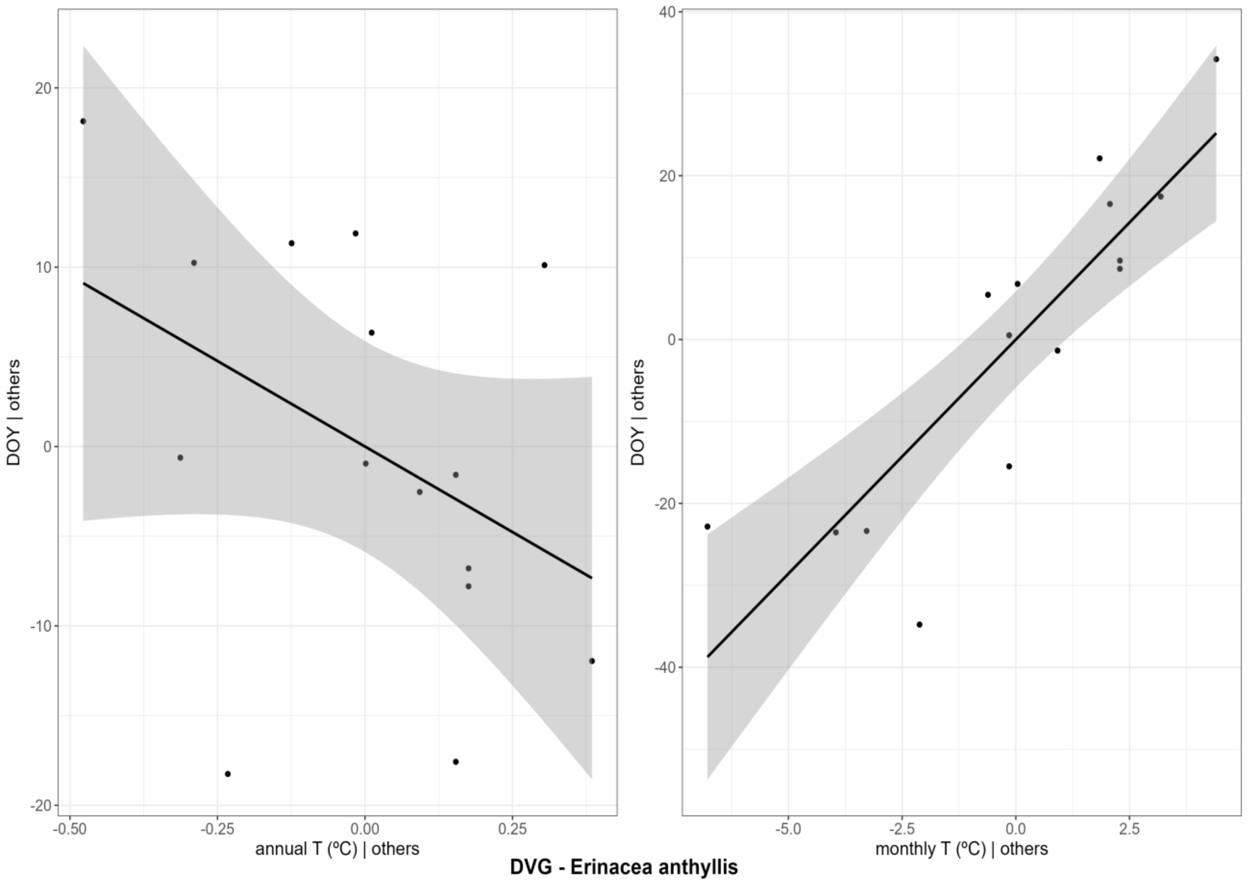

DOY = 357.08 (-19.08*annual T (ºC)) + (+5.72*monthly T (ºC))

1.43.1. Diagnostics - MLM - DVG - Erinacea anthyllis

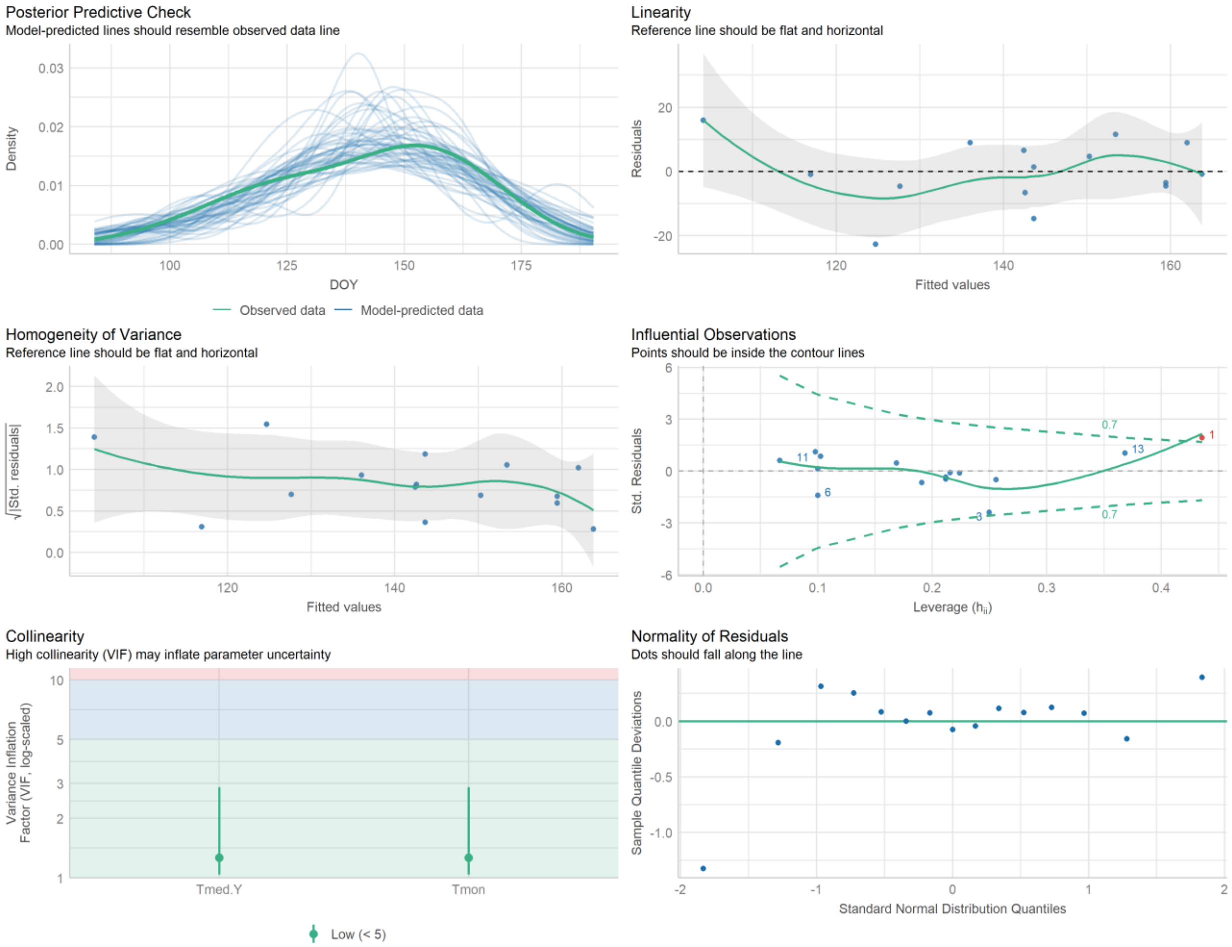

1.44. MLM - FBF - Fumana thymifolia

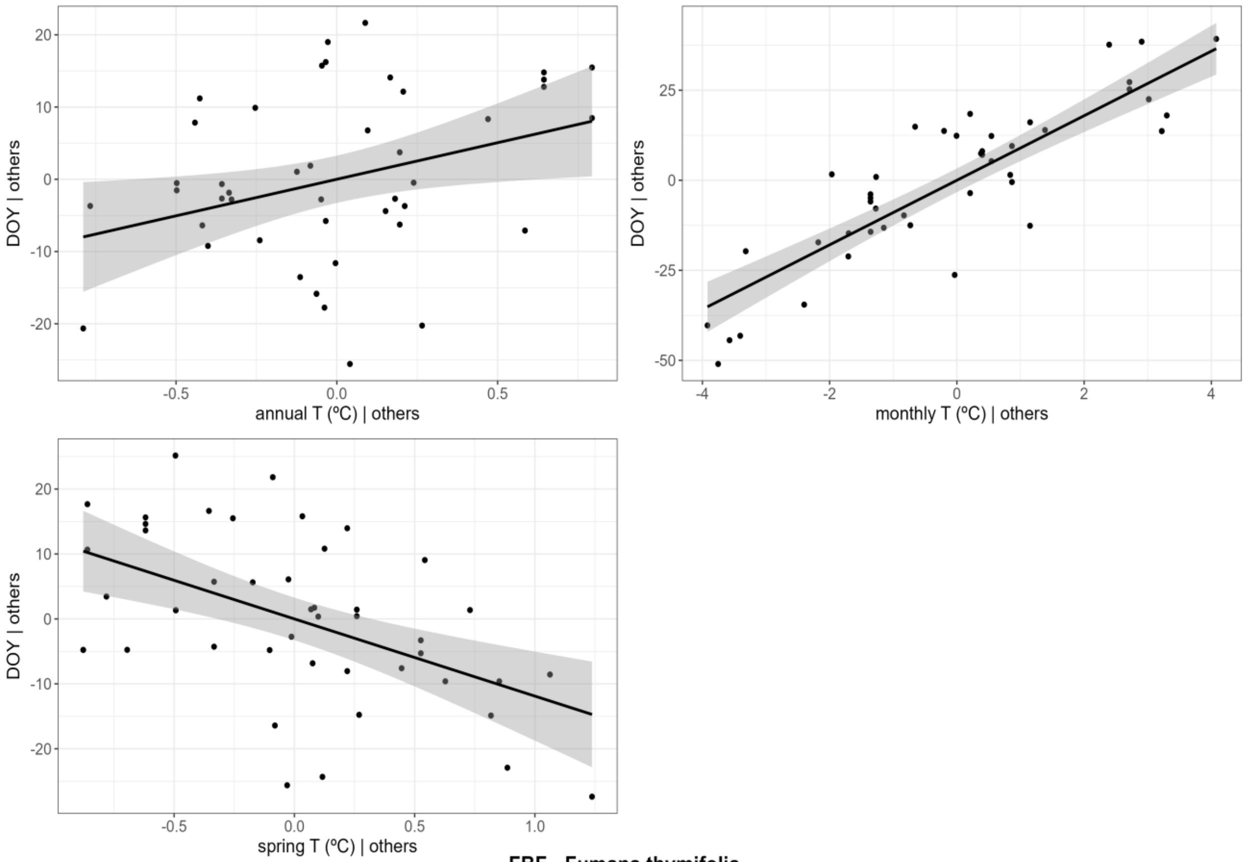

FBF - Fumana thymifolia

DOY = -20.57 (+10.12*annual T (ºC)) + (+8.96*monthly T (ºC)) + (-11.89*spring T (ºC))

1.44.1. Diagnostics - MLM - FBF - Fumana thymifolia

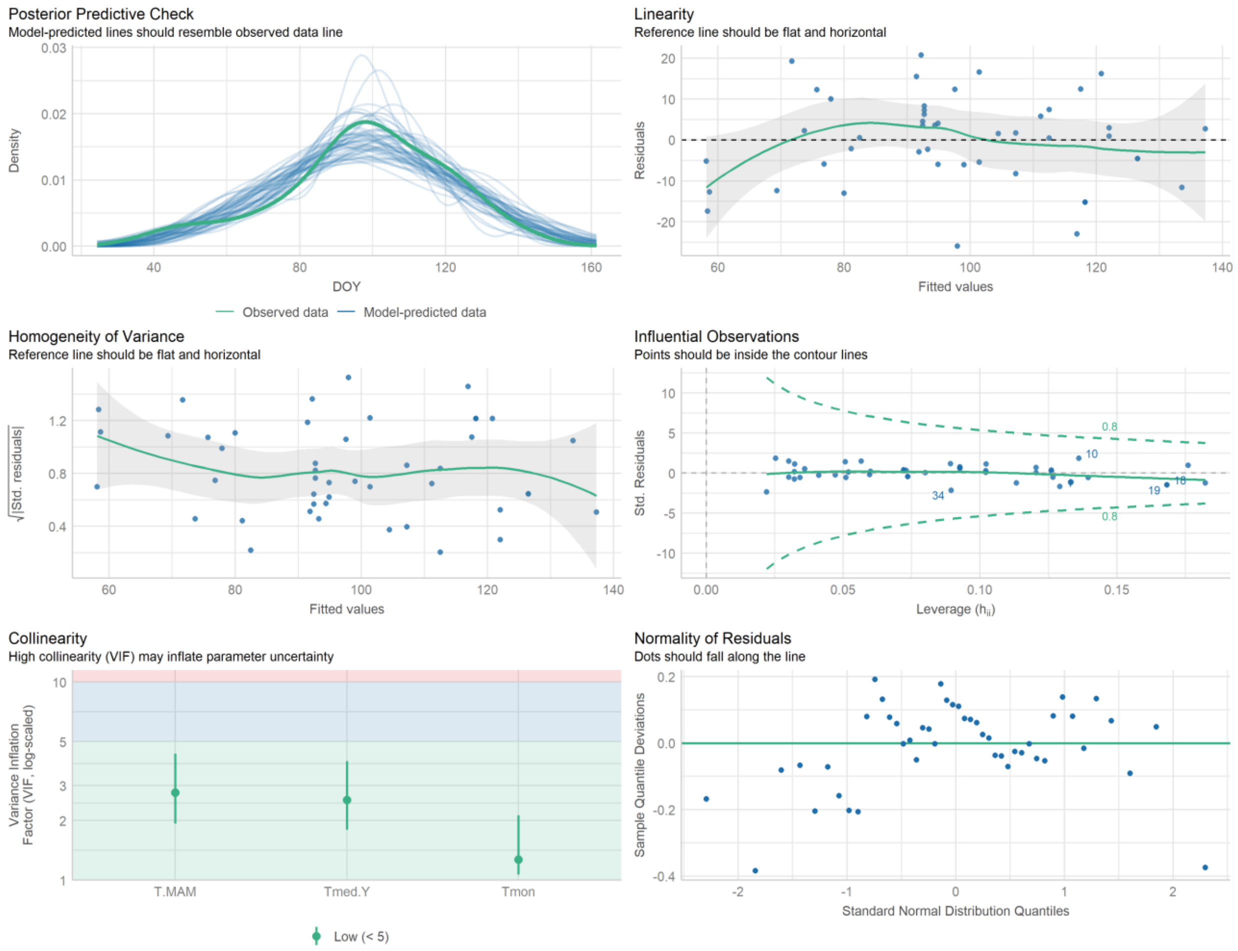

1.45. MLM - DVG - Fumana thymifolia

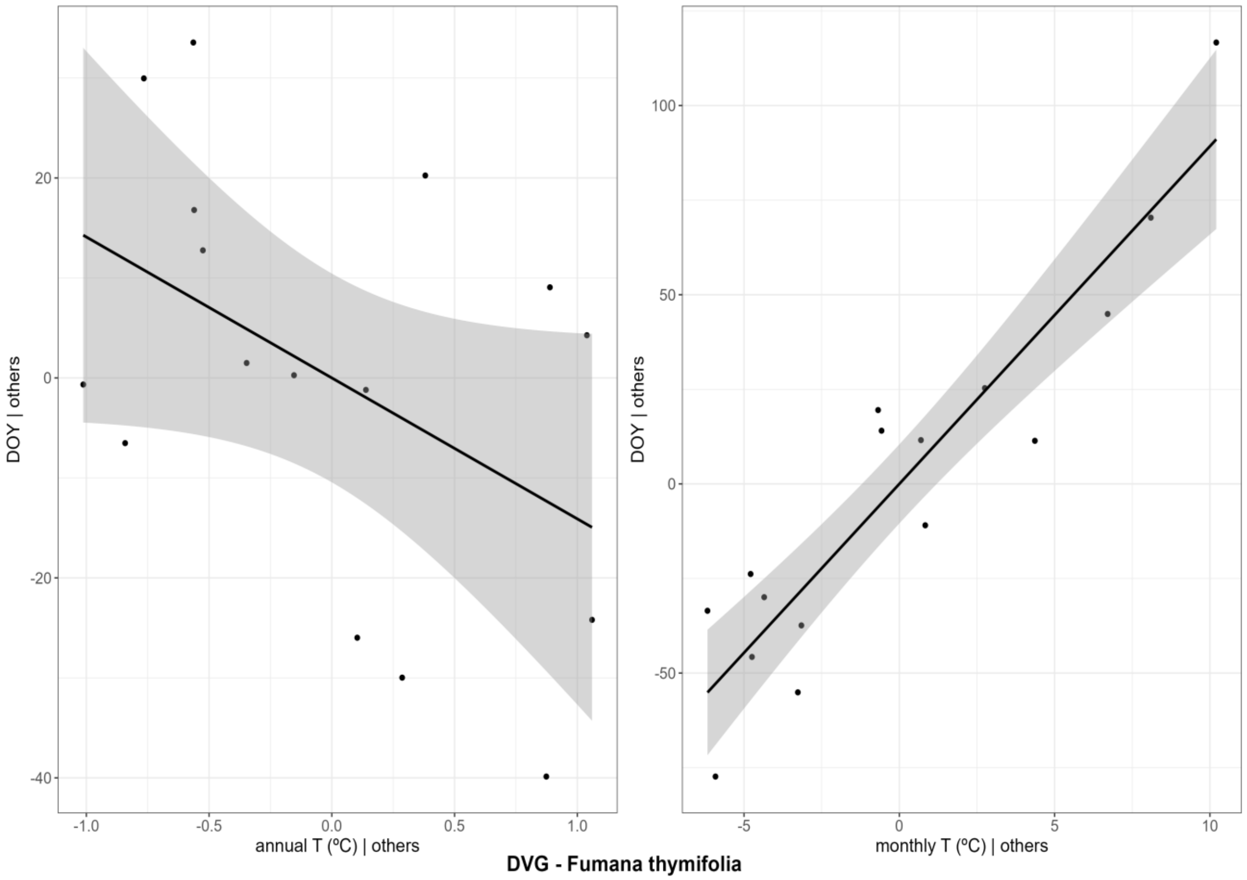

DOY = 206.53 (-14.10*annual T (ºC)) + (+8.92*monthly T (ºC))

1.45.1. Diagnostics - MLM - DVG - Fumana thymifolia

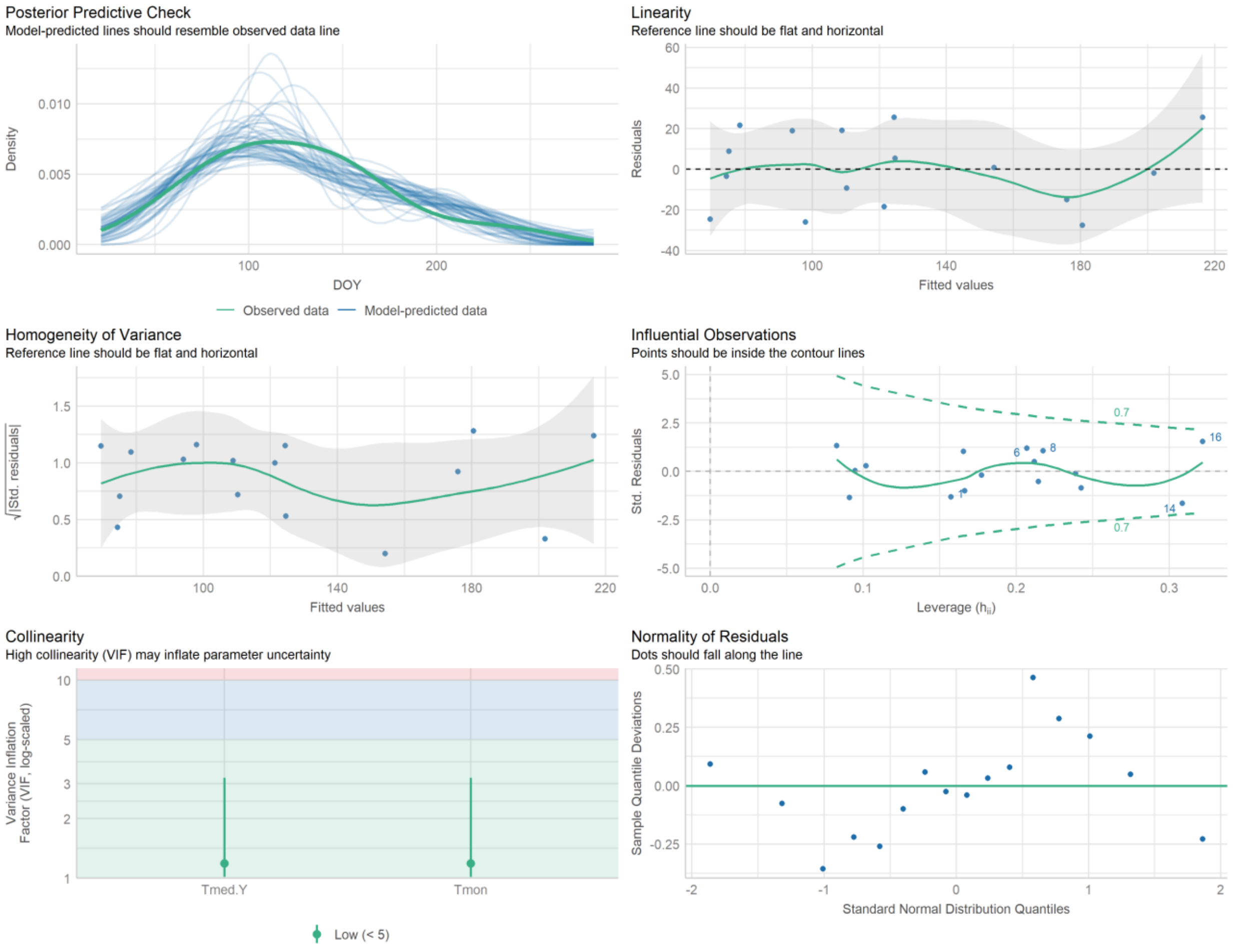

1.46. MLM - FBF - Genista hirsuta subsp. lanuginosa

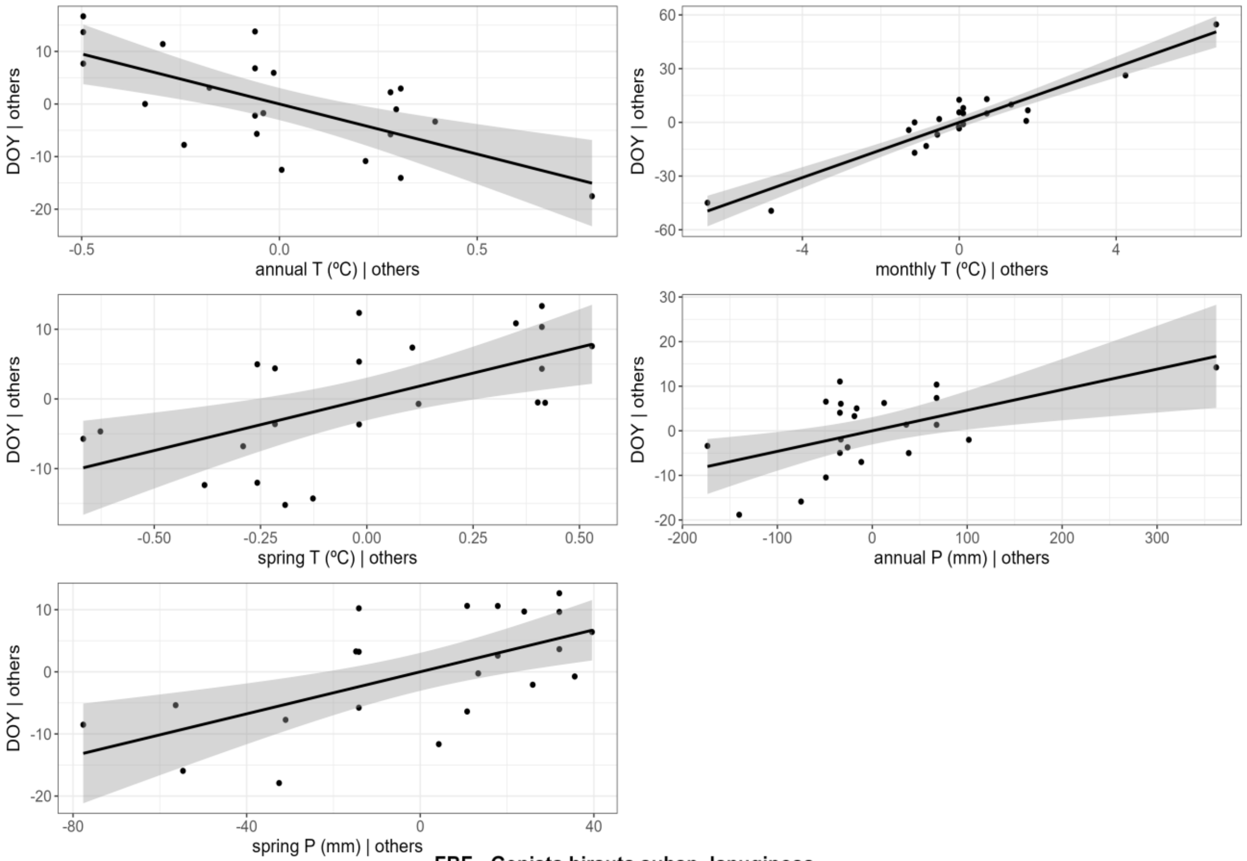

FBF - Genista hirsuta subsp. lanuginosa

DOY = 42.61 (-19.05*annual T (ºC)) + (+7.72*monthly T (ºC)) + (+14.81*spring T (ºC)) + (+0.05*annual P (mm)) + (+0.17*spring P (mm))

1.46.1. Diagnostics - MLM - FBF - Genista hirsuta subsp. lanuginosa

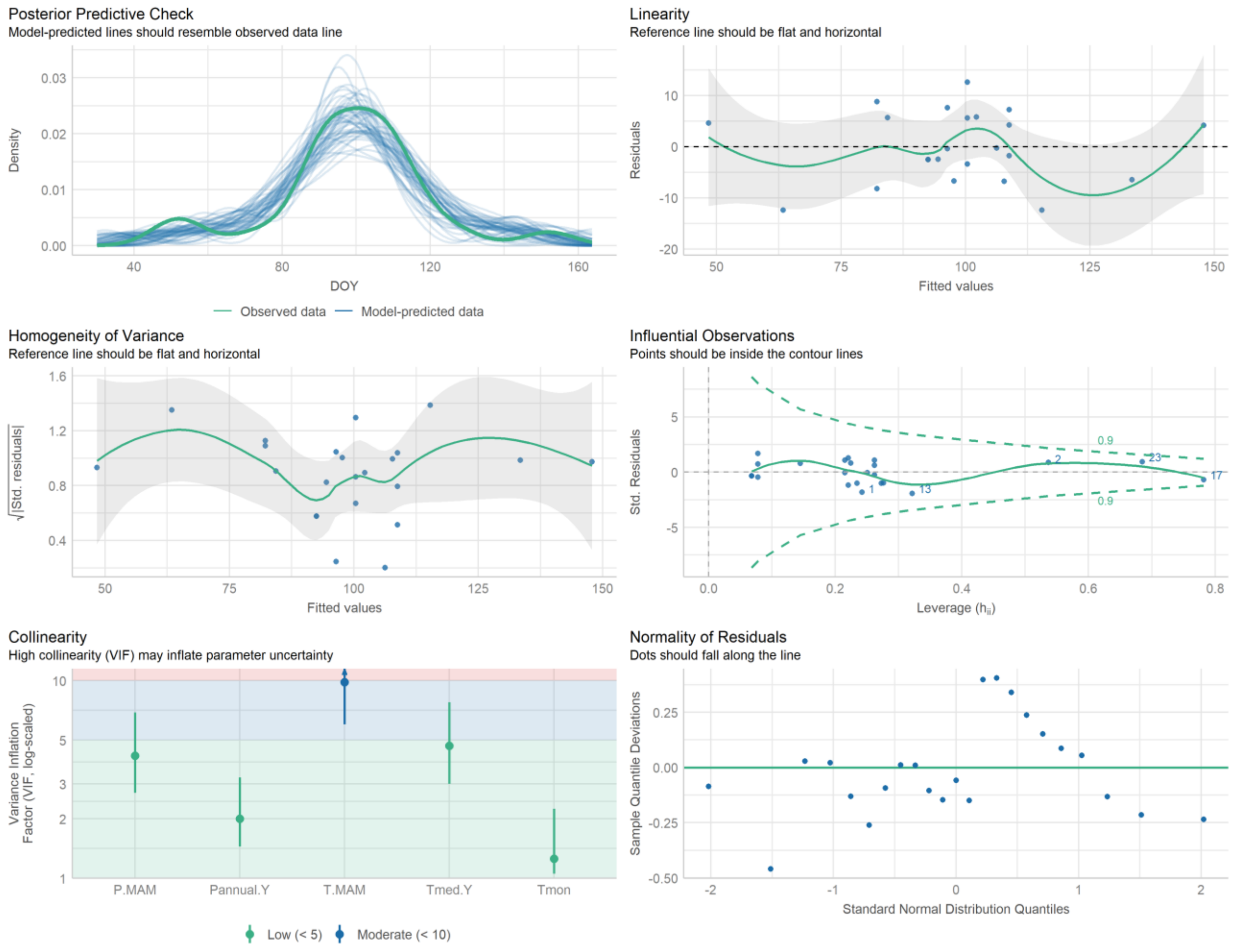

1.47. MLM - F - Genista hirsuta subsp. lanuginosa

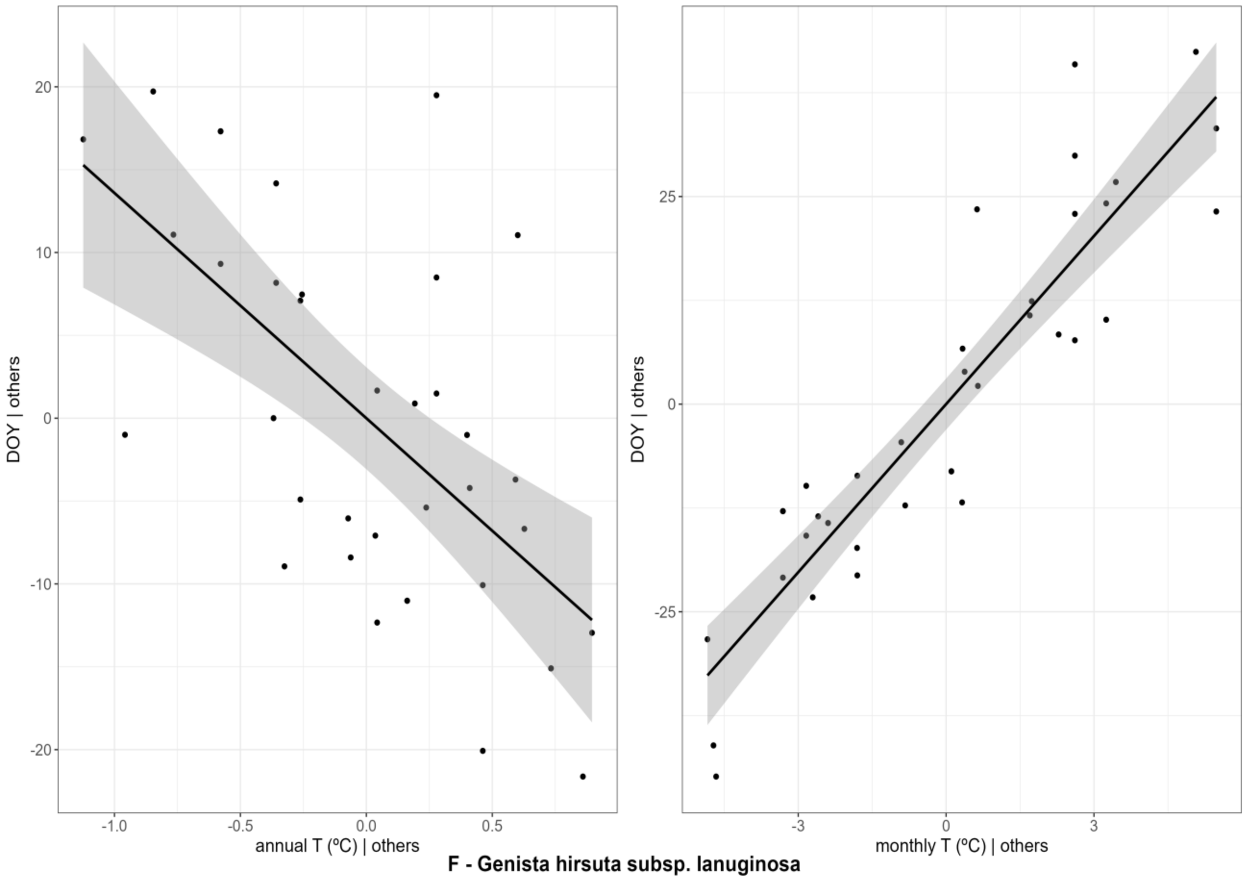

DOY = 237.71 (-13.59*annual T (ºC)) + (+6.75*monthly T (ºC))

1.47.1. Diagnostics - MLM - F - Genista hirsuta subsp. lanuginosa

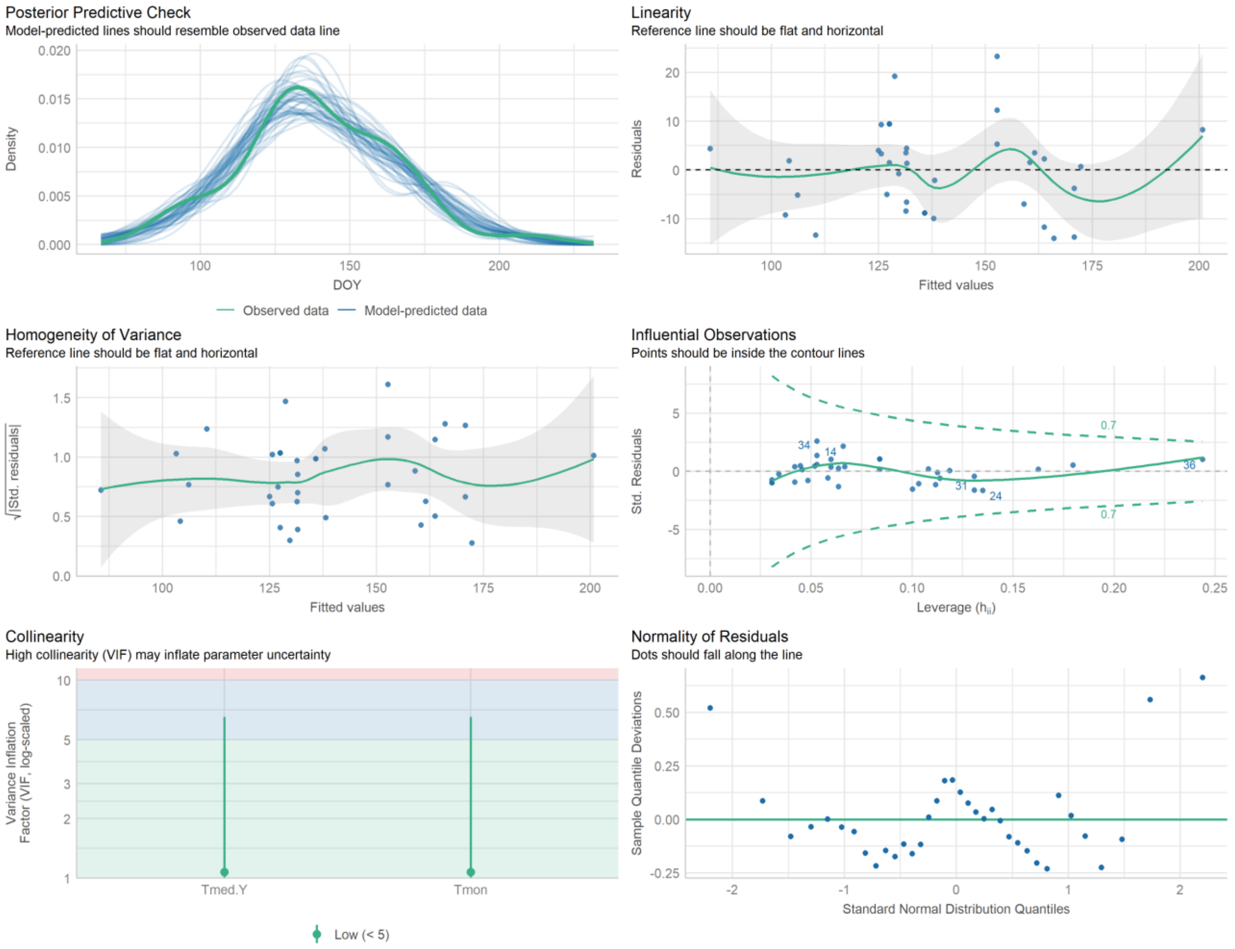

1.48. MLM - F - Glandora prostrata subsp. lusitanica

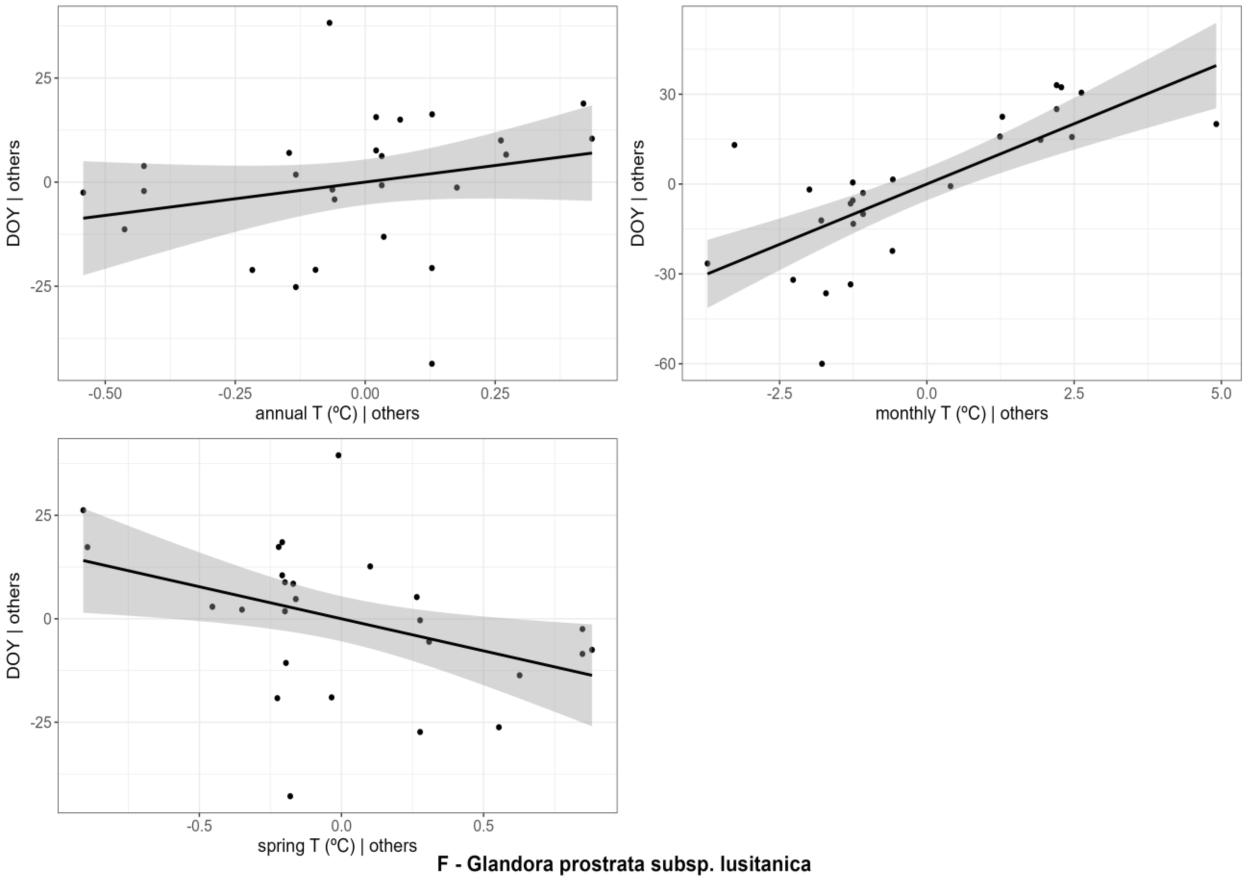

F - Glandora prostrata subsp. lusitanica

DOY = -56.05 (+15.98*annual T (ºC)) + (+8.05*monthly T (ºC)) + (-15.50*spring T (ºC))

1.48.1. Diagnostics - MLM - F - Glandora prostrata subsp. lusitanica

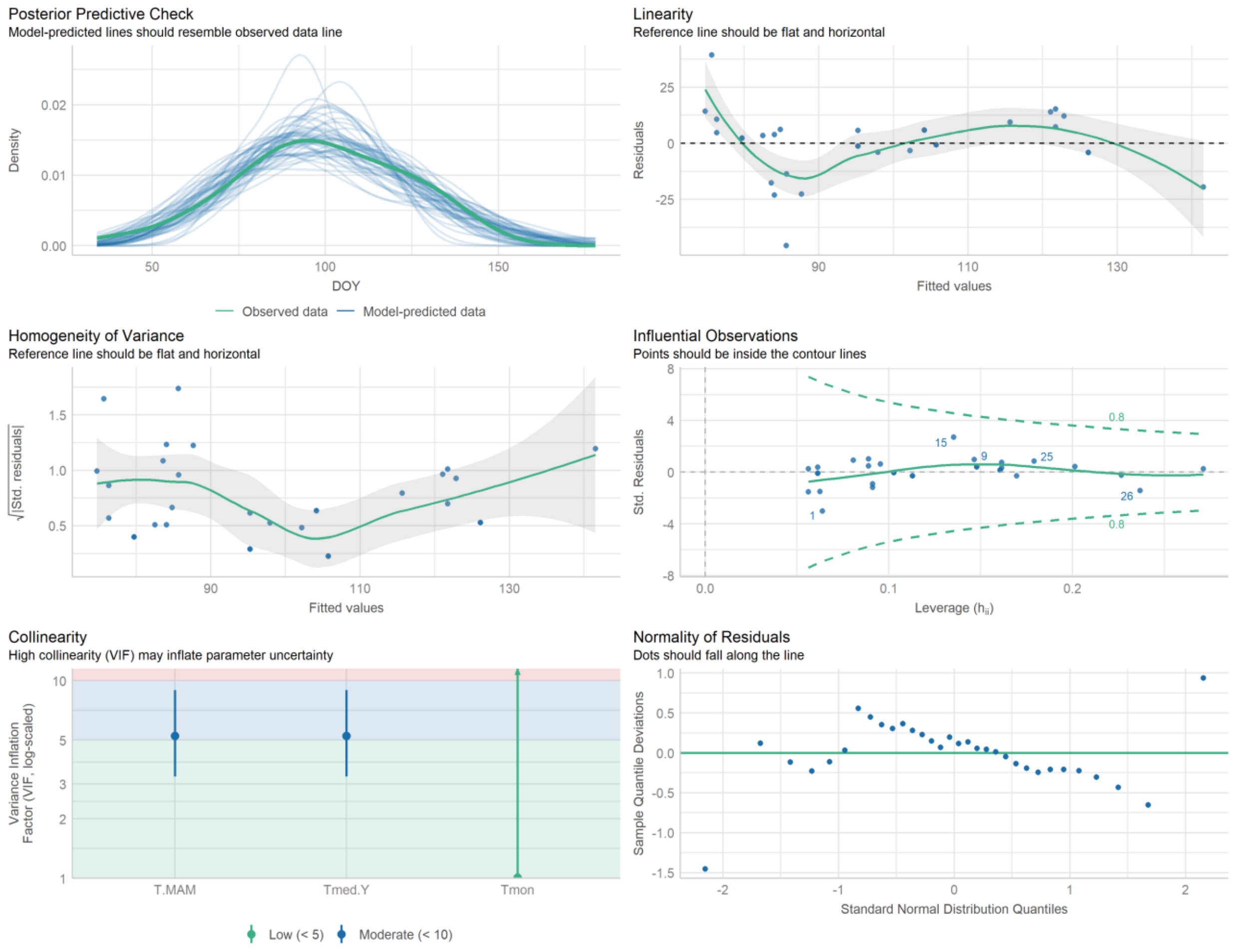

1.49. MLM - FBF - Halimium atriplicifolium

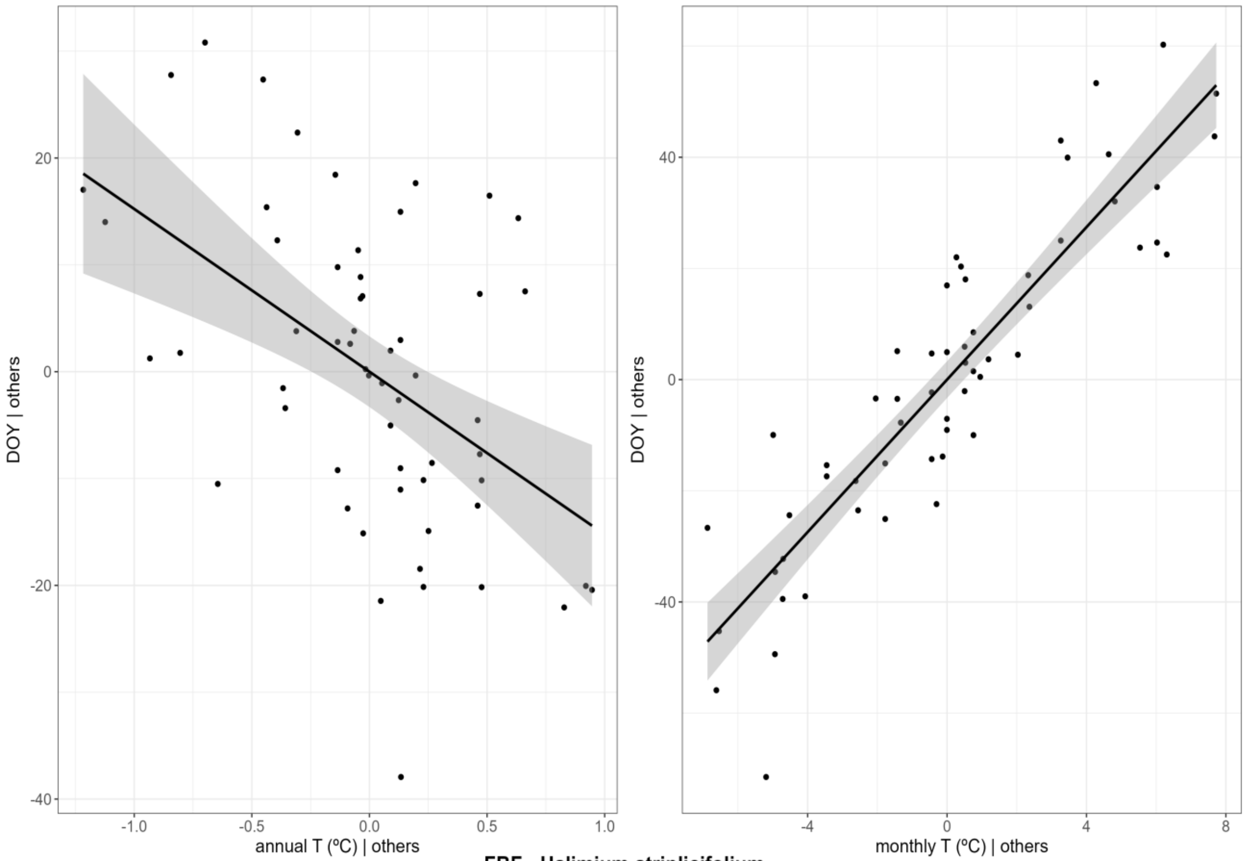

DOY = 265.78 (-15.23*annual T (ºC)) + (+6.86*monthly T (ºC))

1.49.1. Diagnostics - MLM - FBF - Halimium atriplicifolium

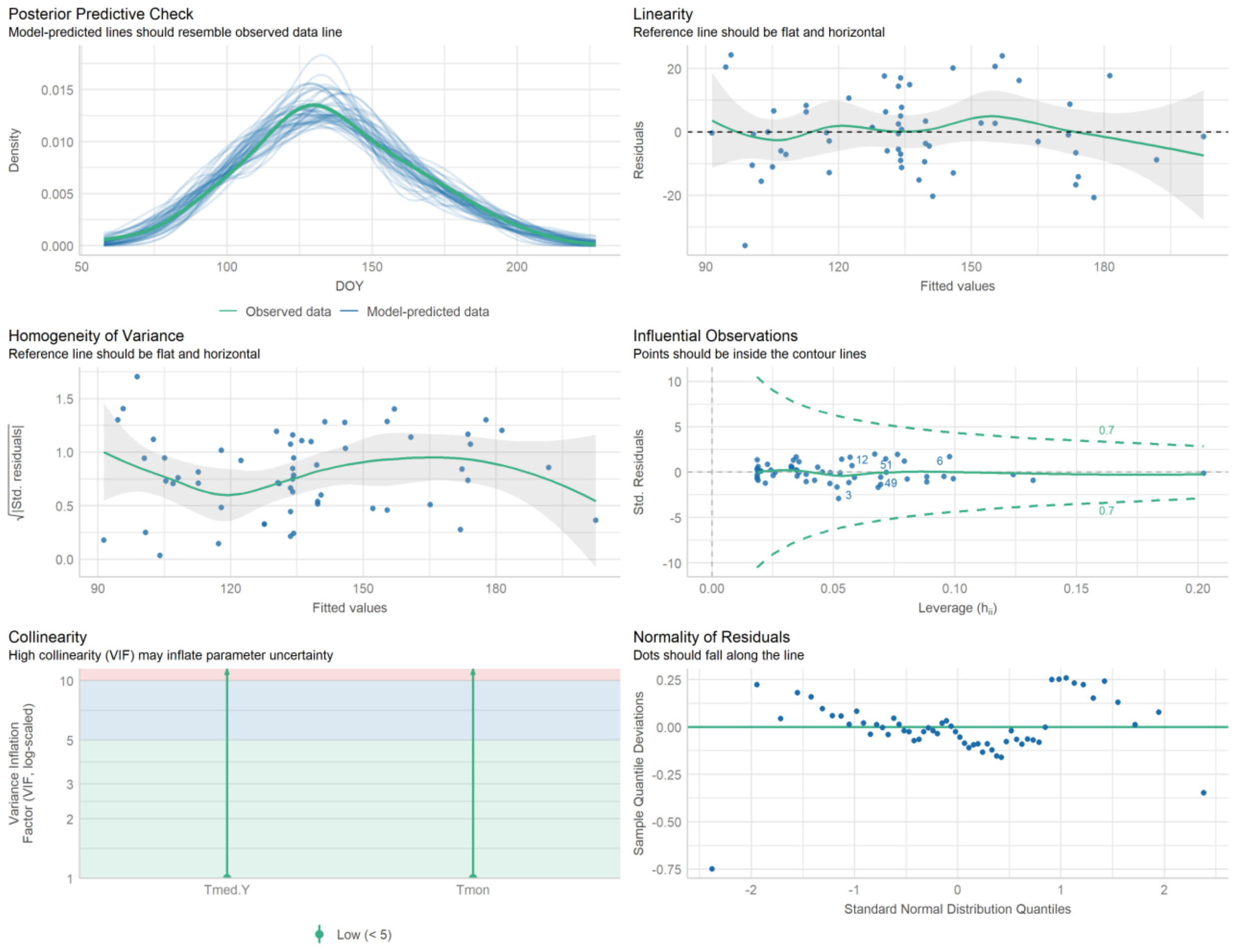

1.50. MLM - F - Halimium atriplicifolium

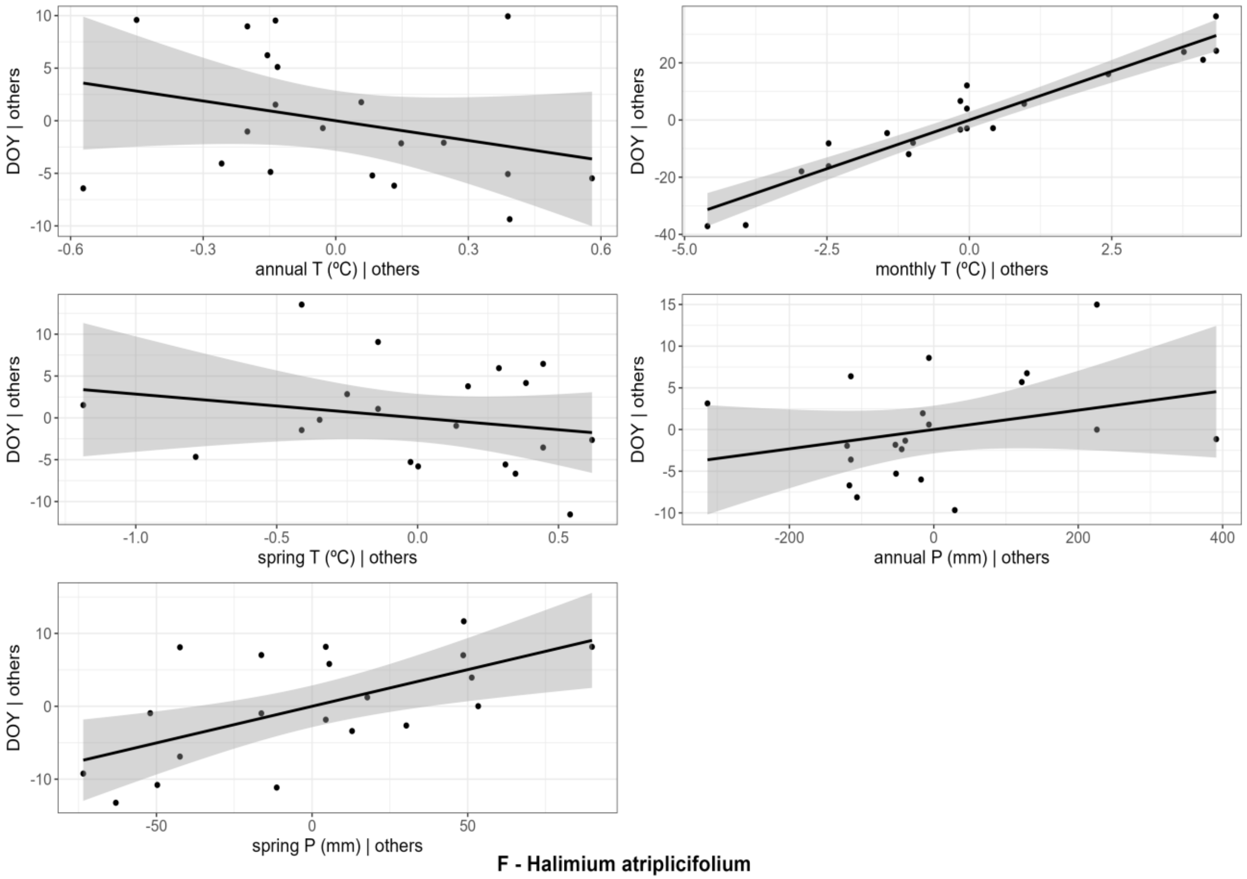

F - Halimium atriplicifolium

DOY = 136.90 (-6.26*annual T (ºC)) + (+6.81*monthly T (ºC)) + (-2.84*spring T (ºC)) + (+0.01*annual P (mm)) + (+0.10*spring P (mm))

1.50.1. Diagnostics - MLM - F - Halimium atriplicifolium

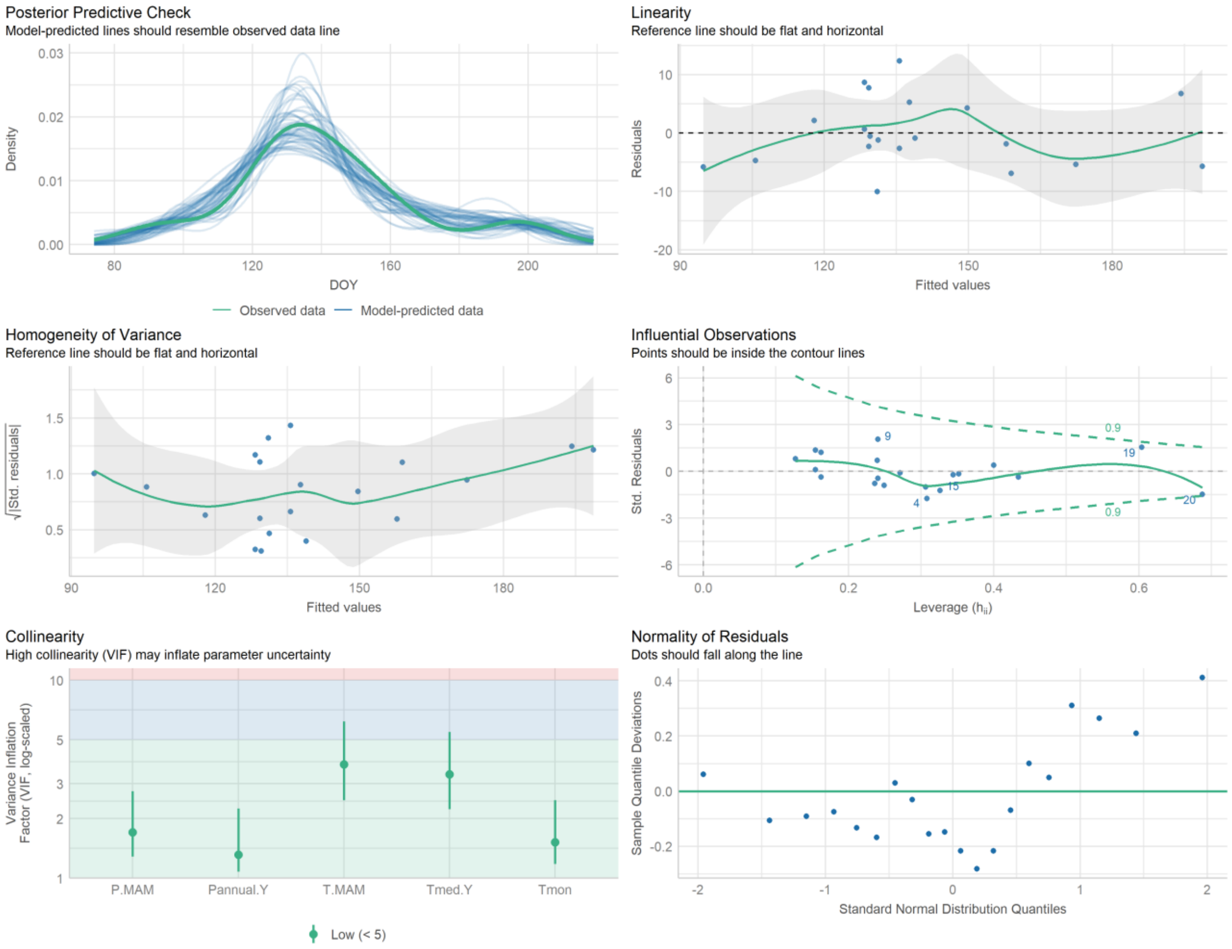

1.51. MLM - FBF - Helianthemum syriacum

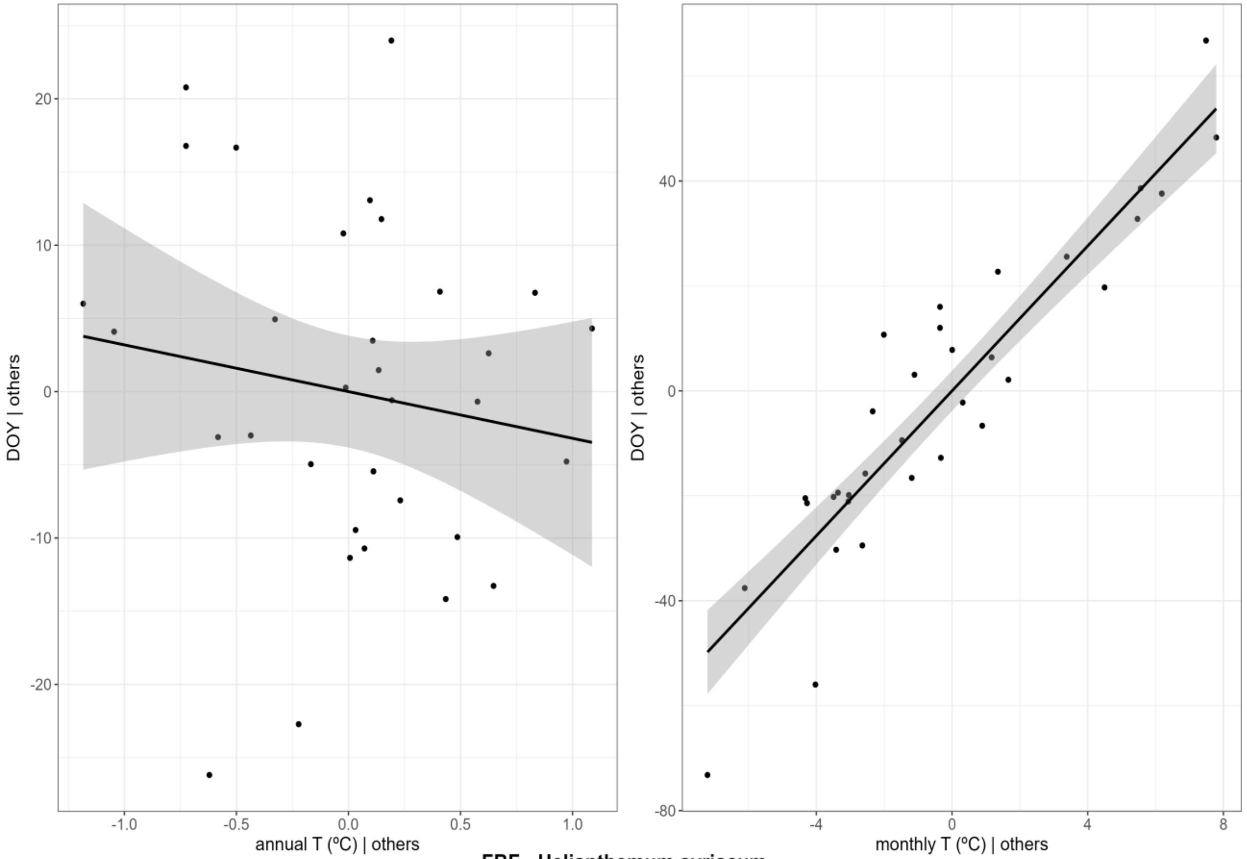

FBF - Helianthemum syriacum

DOY = 64.26 (-3.19*annual T (ºC)) + (+6.91*monthly T (ºC))

1.51.1. Diagnostics - MLM - FBF - Helianthemum syriacum

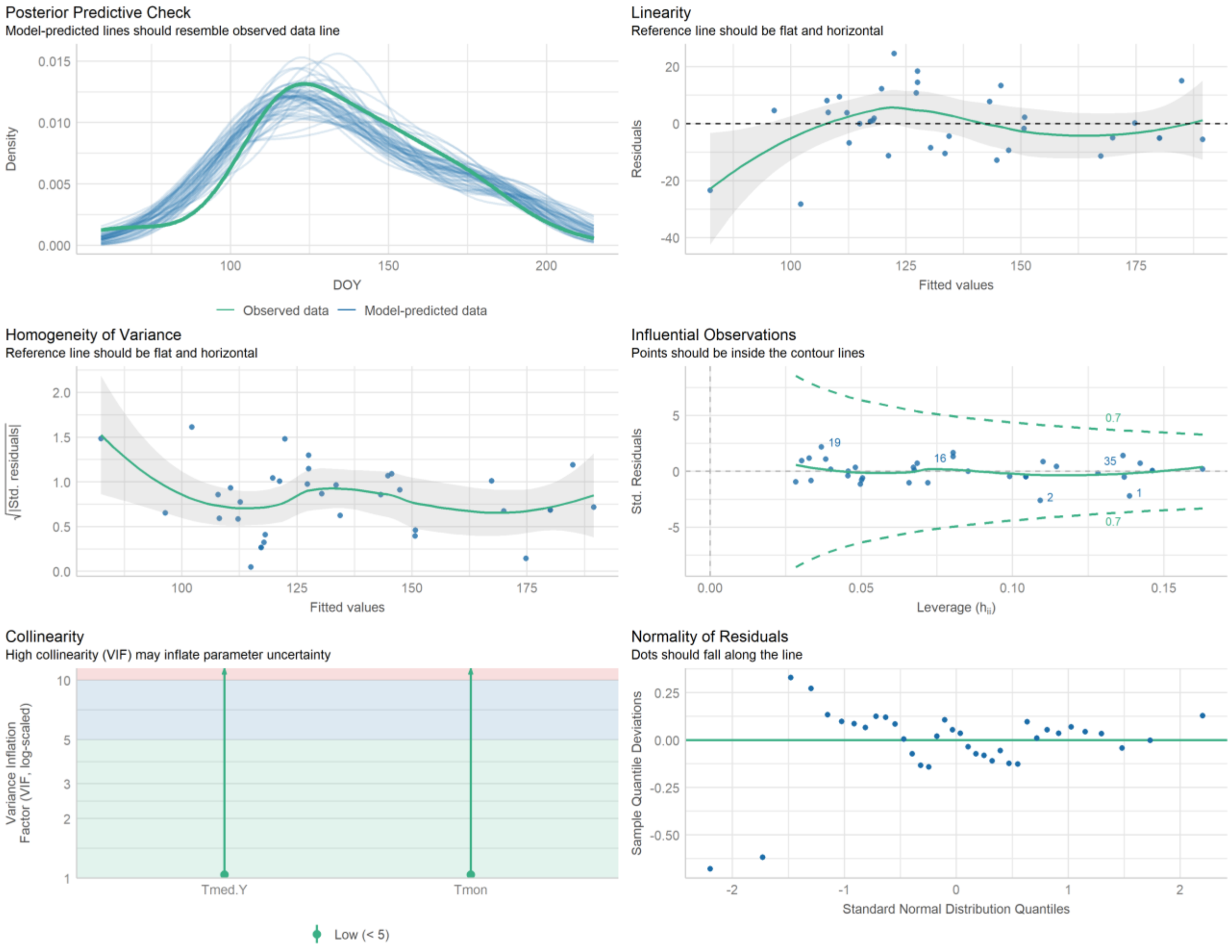

1.52. MLM - F - Helianthemum syriacum

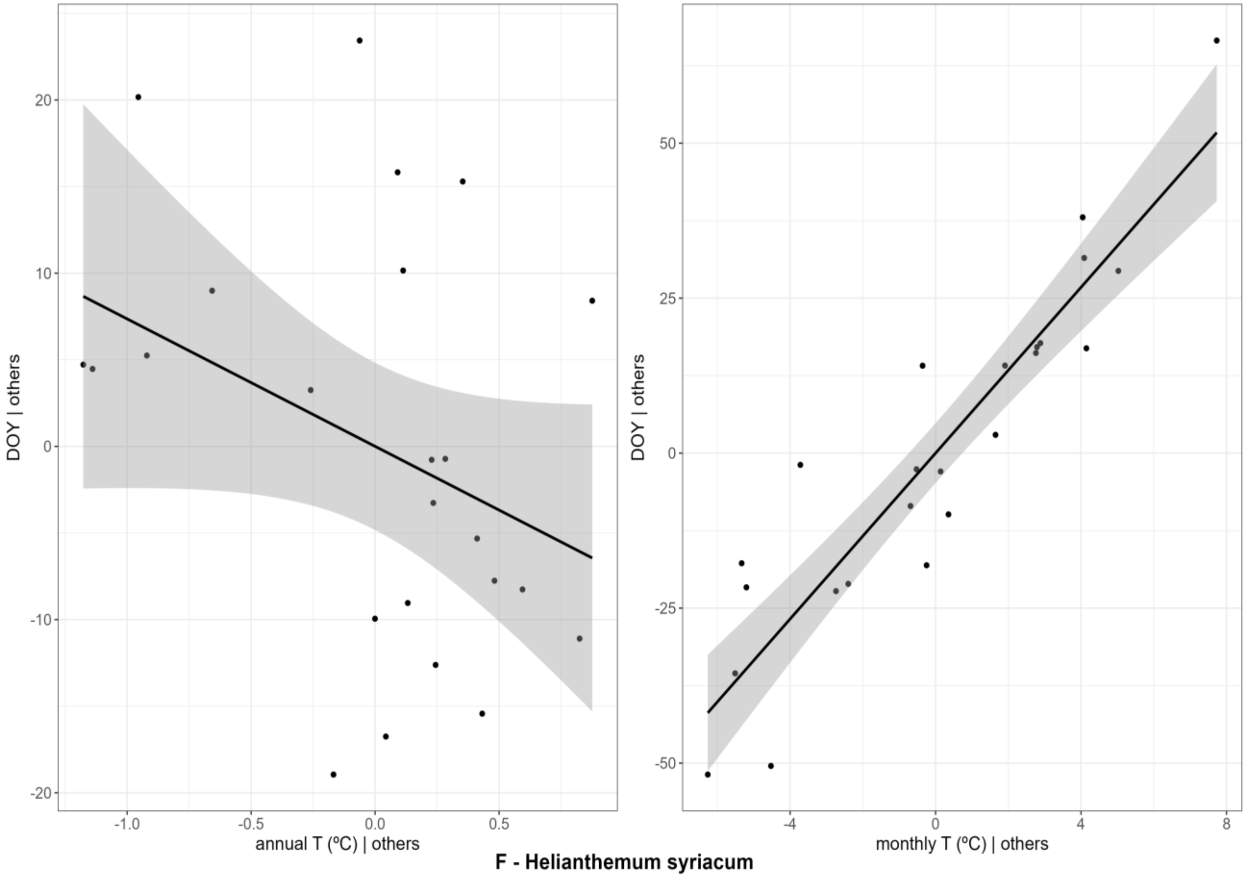

DOY = 138.88 (-7.36*annual T (ºC)) + (+6.69*monthly T (ºC))

1.52.1. Diagnostics - MLM - F - Helianthemum syriacum

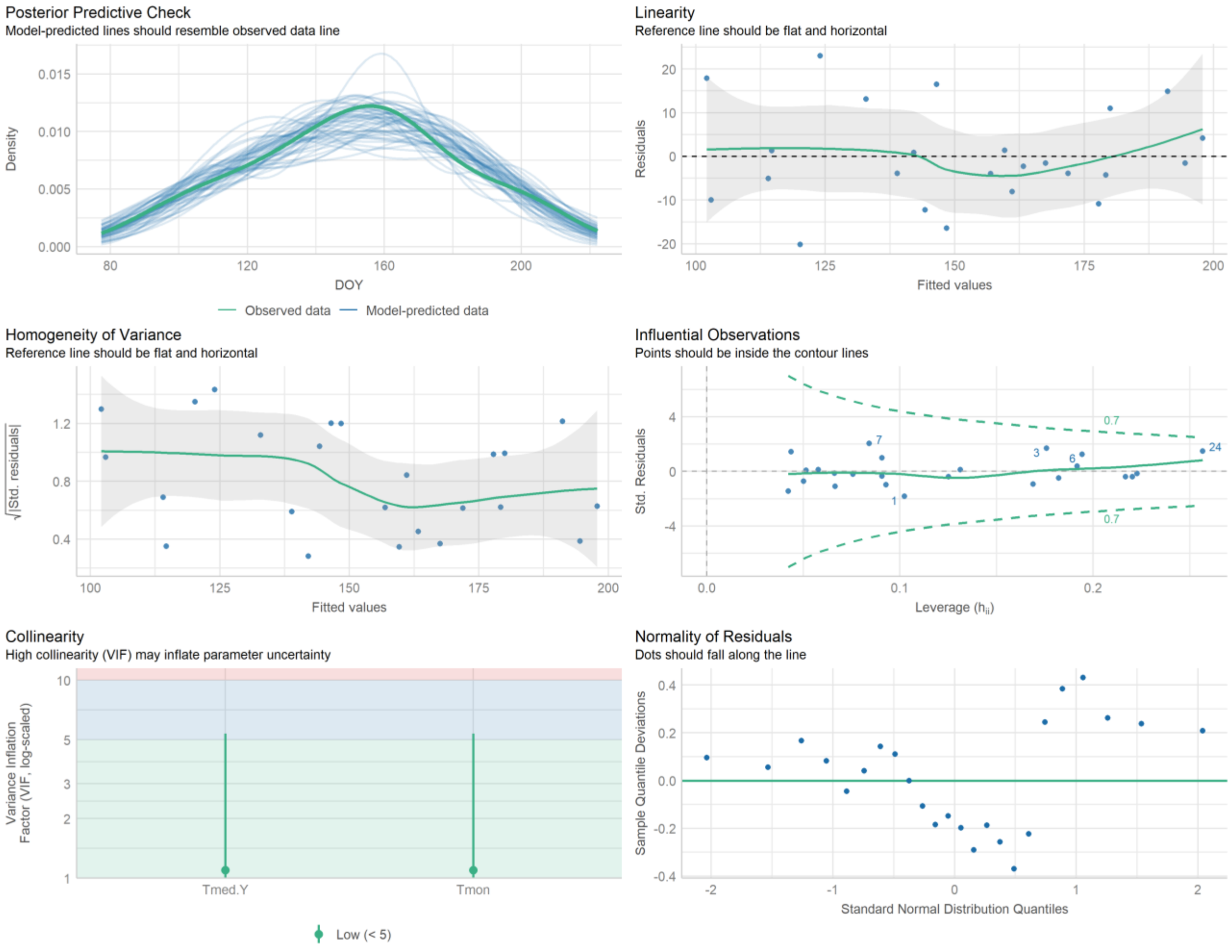

1.53. MLM - FBF - Helichrysum stoechas

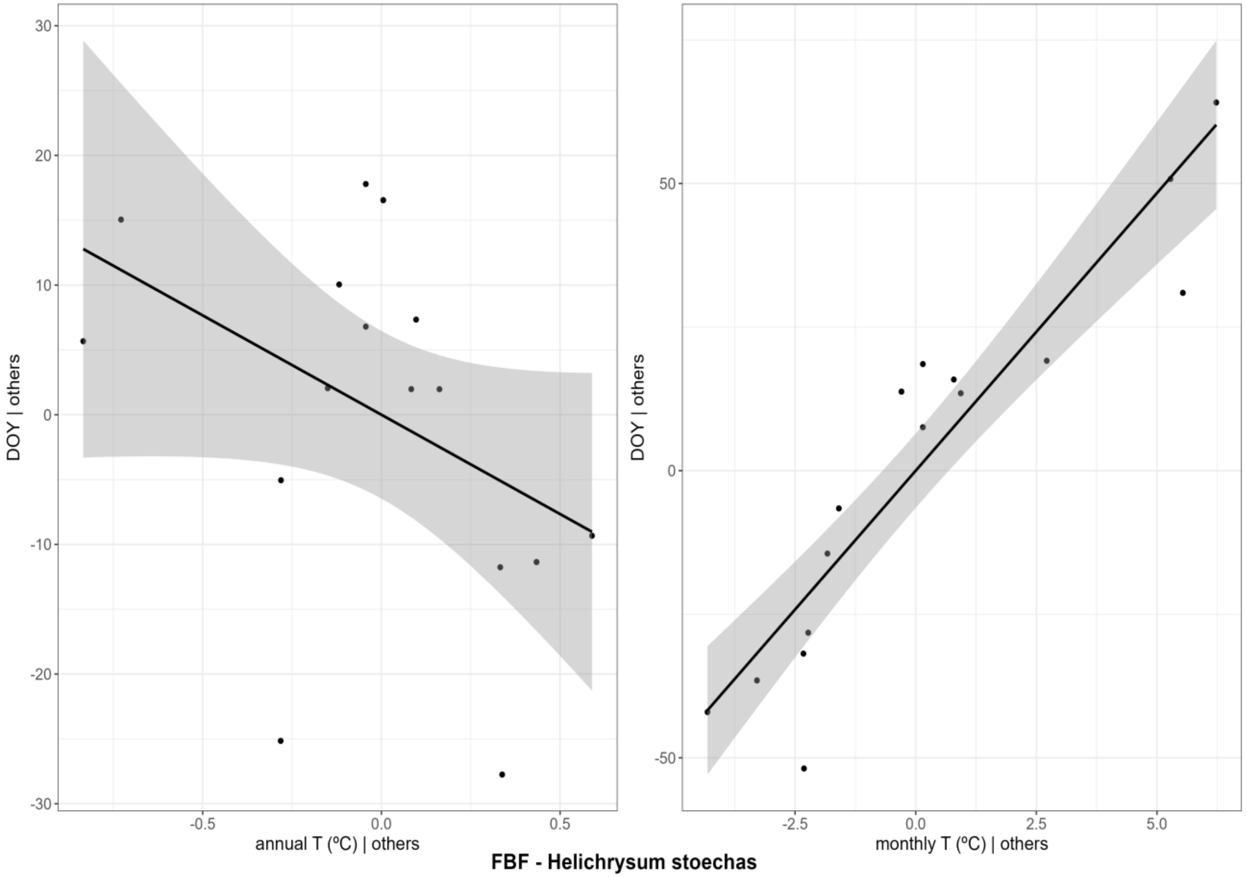

FBF - Helichrysum stoechas

DOY = 209.58 (-15.32*annual T (ºC)) + (+9.67*monthly T (ºC))

1.53.1. Diagnostics - MLM - FBF - Helichrysum stoechas

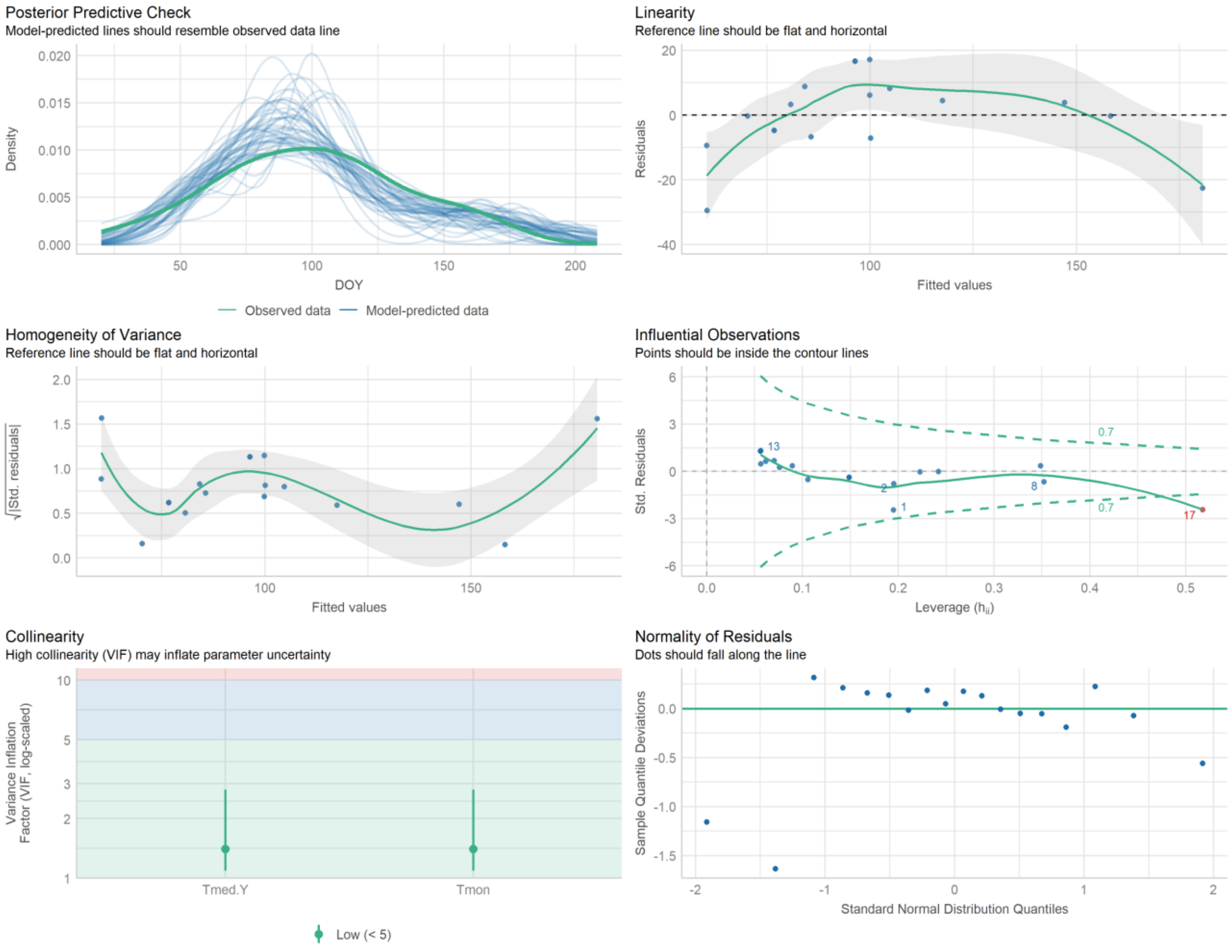

1.54. MLM - F - Helichrysum stoechas

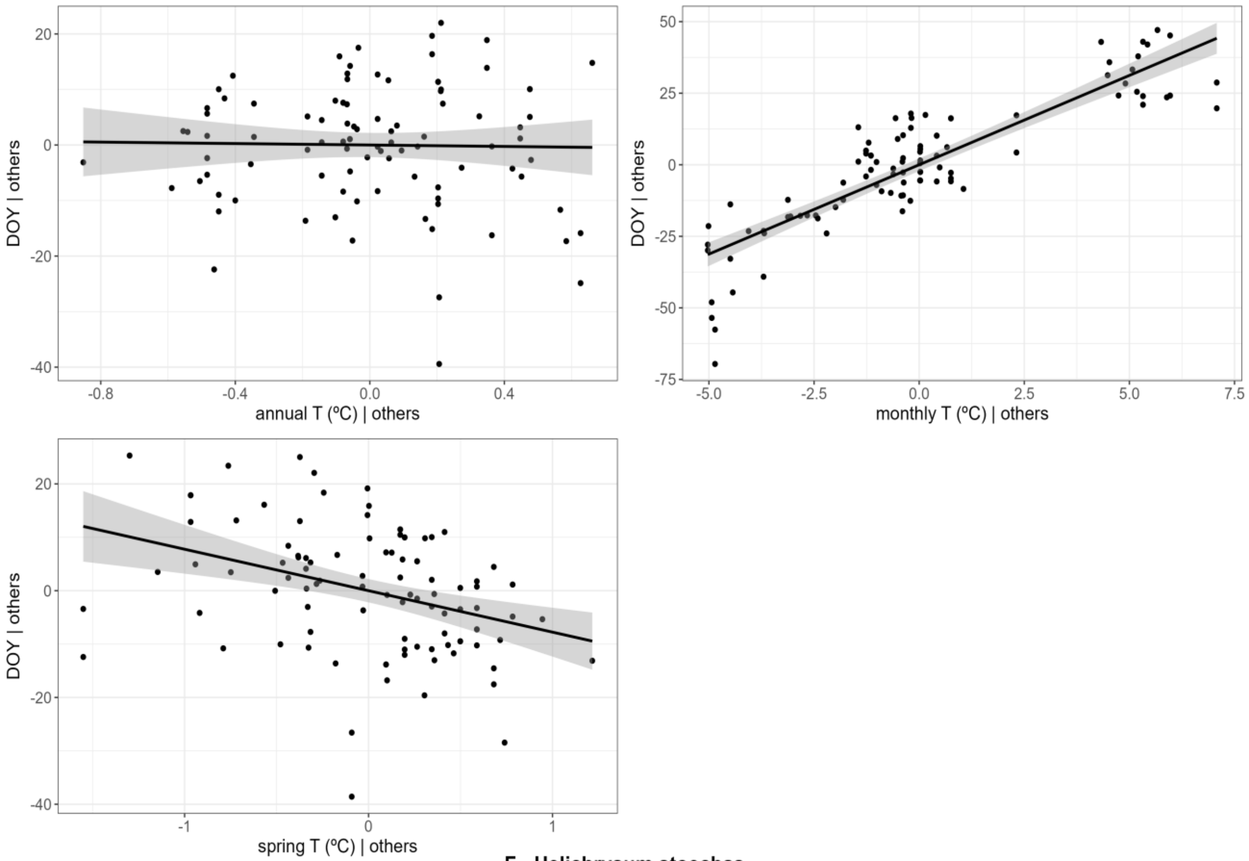

DOY = 147.31 (-0.66*annual T (ºC)) + (+6.24*monthly T (ºC)) + (-7.76*spring T (ºC))

1.54.1. Diagnostics - MLM - F - Helichrysum stoechas

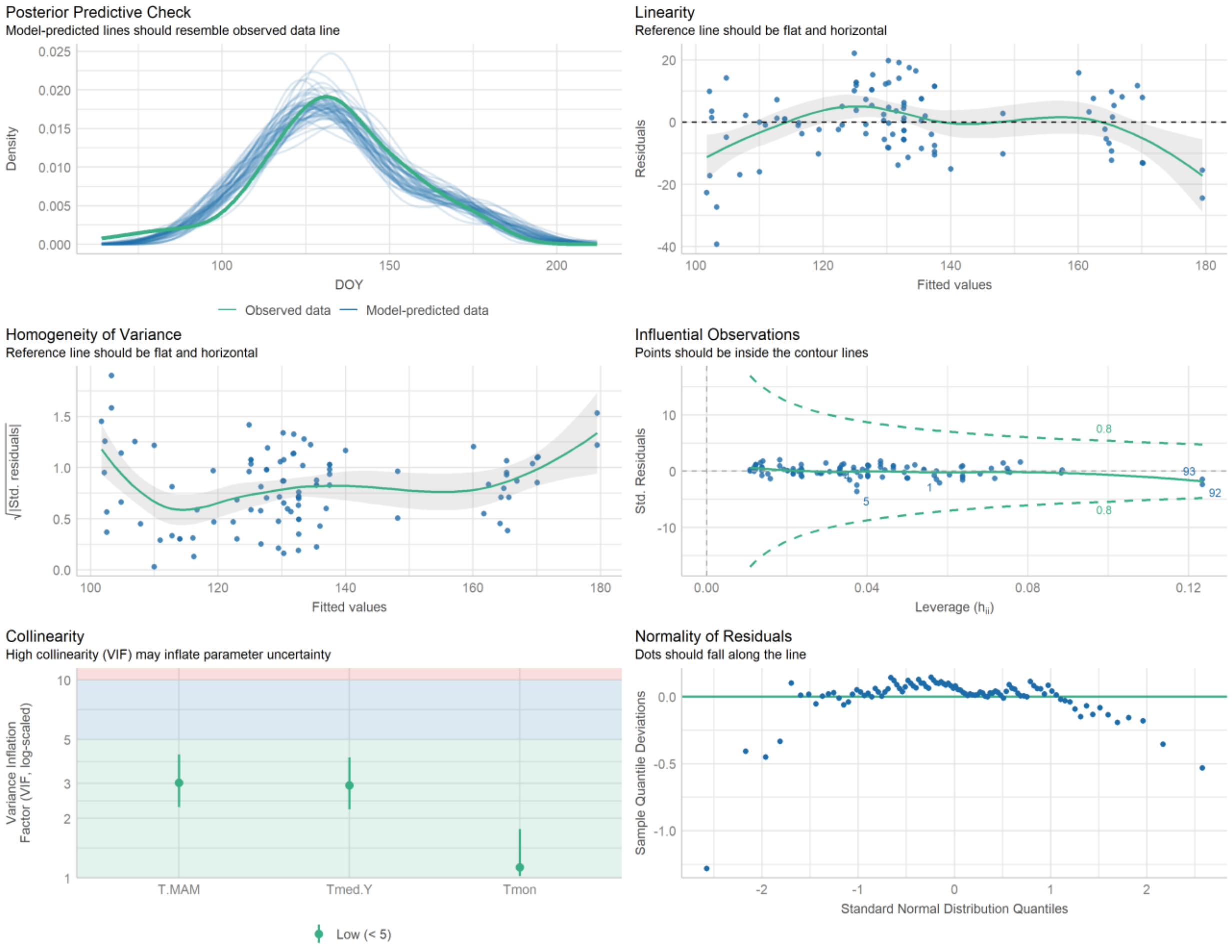

1.55. MLM - DVG - Helichrysum stoechas

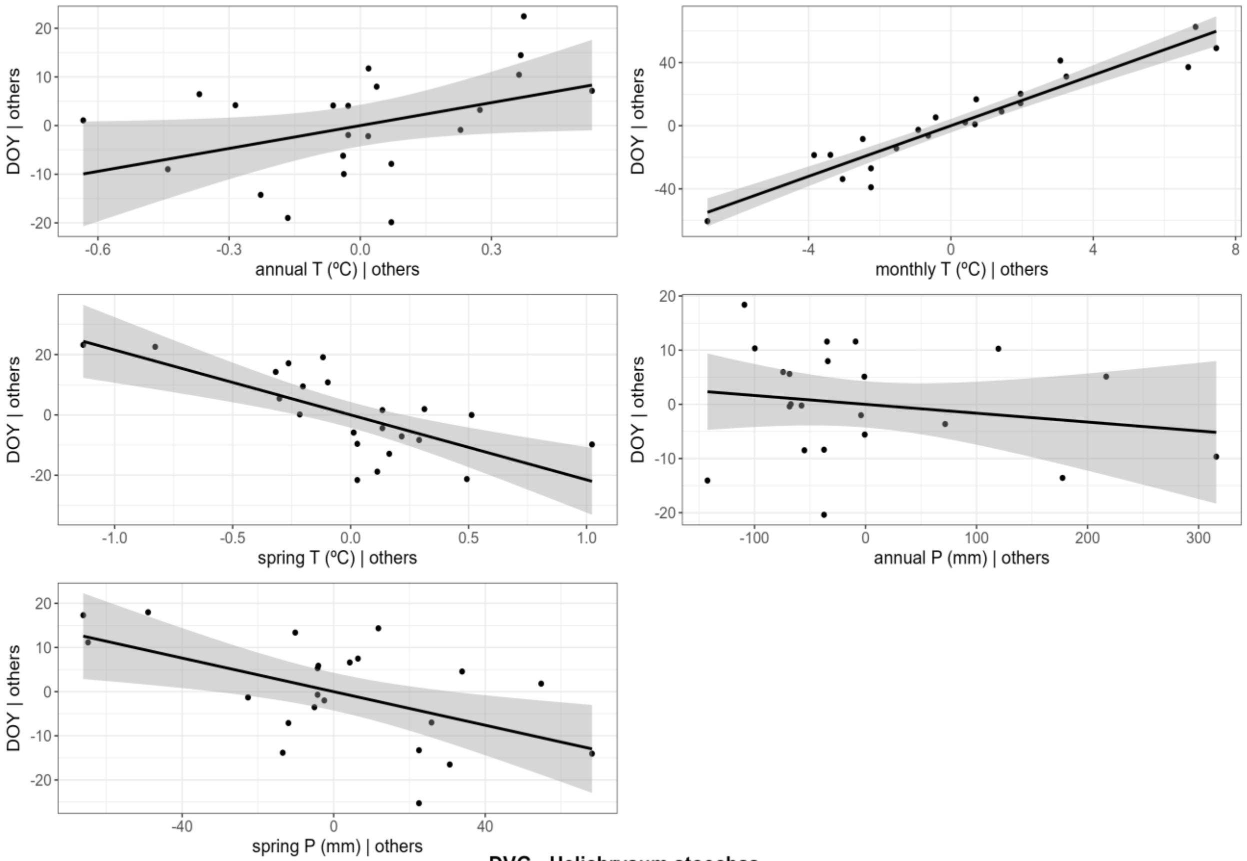

DVG - Helichrysum stoechas

DOY = 72.78 (+15.69*annual T (ºC)) + (+8.02*monthly T (ºC)) + (-21.52*spring T (ºC)) + (-0.02*annual P (mm)) + (-0.19*spring P (mm))

1.55.1. Diagnostics - MLM - DVG - Helichrysum stoechas

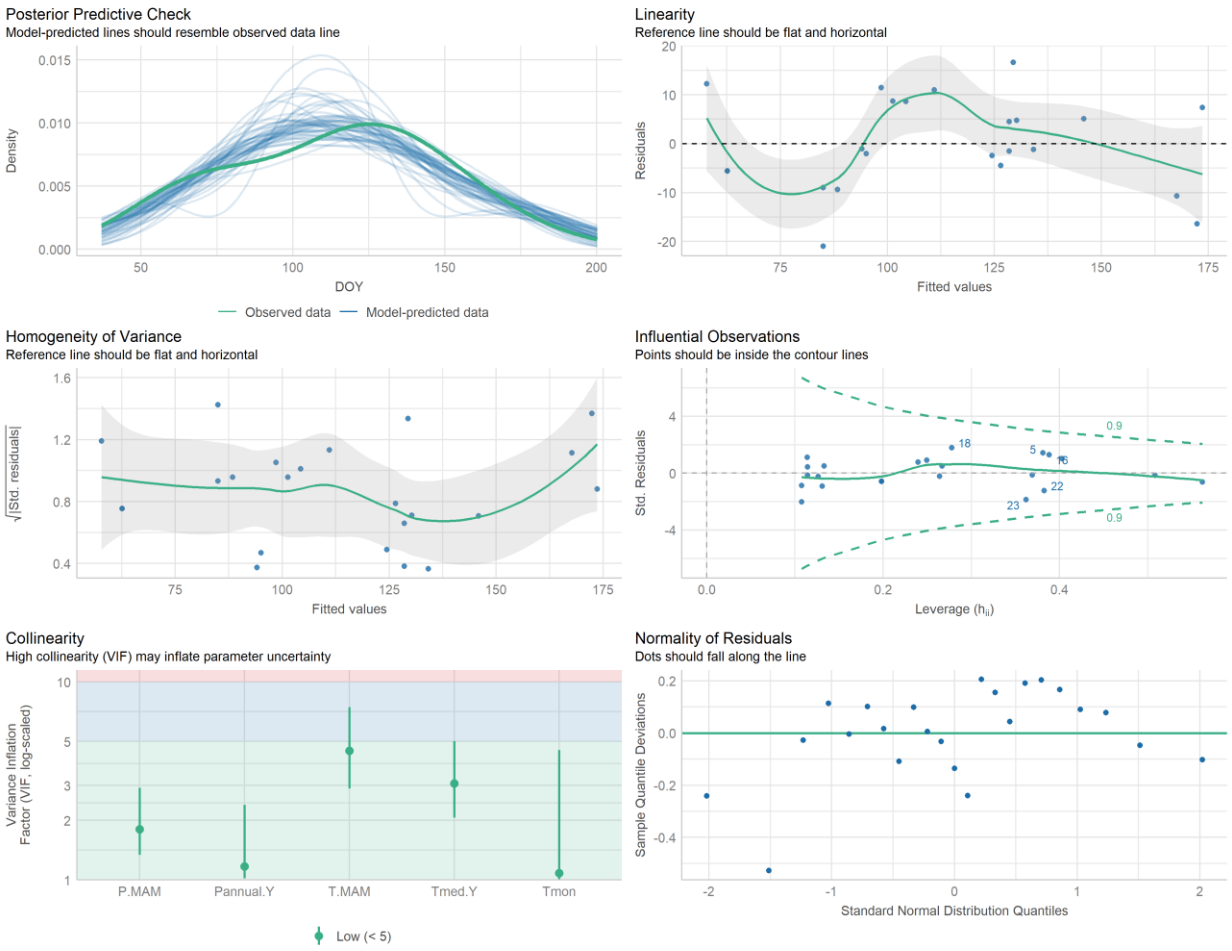

1.56. MLM - FS - Helictotrichon filifolium subsp. arundanum

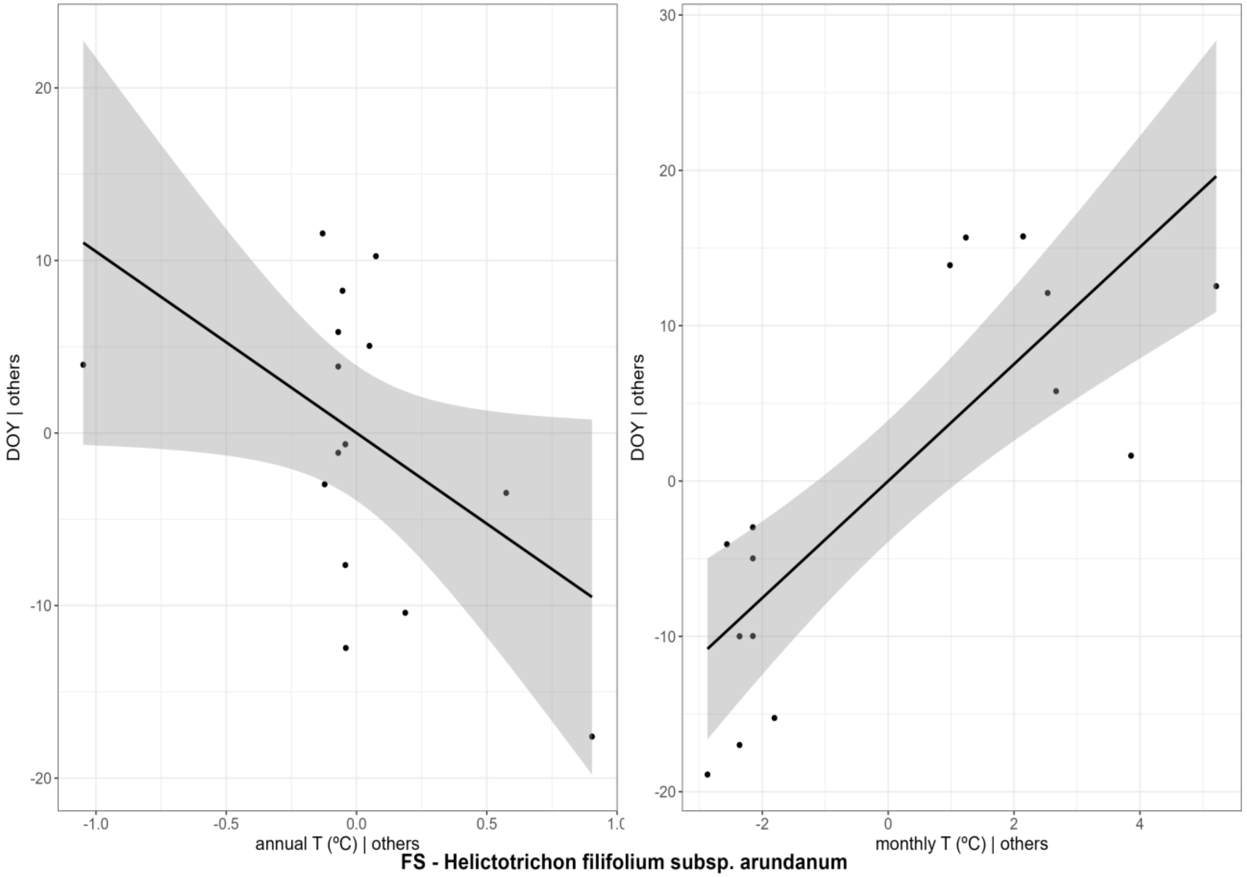

DOY = 256.16 (-10.52*annual T (ºC)) + (+3.76*monthly T (ºC))

1.56.1. Diagnostics - MLM - FS - Helictotrichon filifolium subsp. arundanum

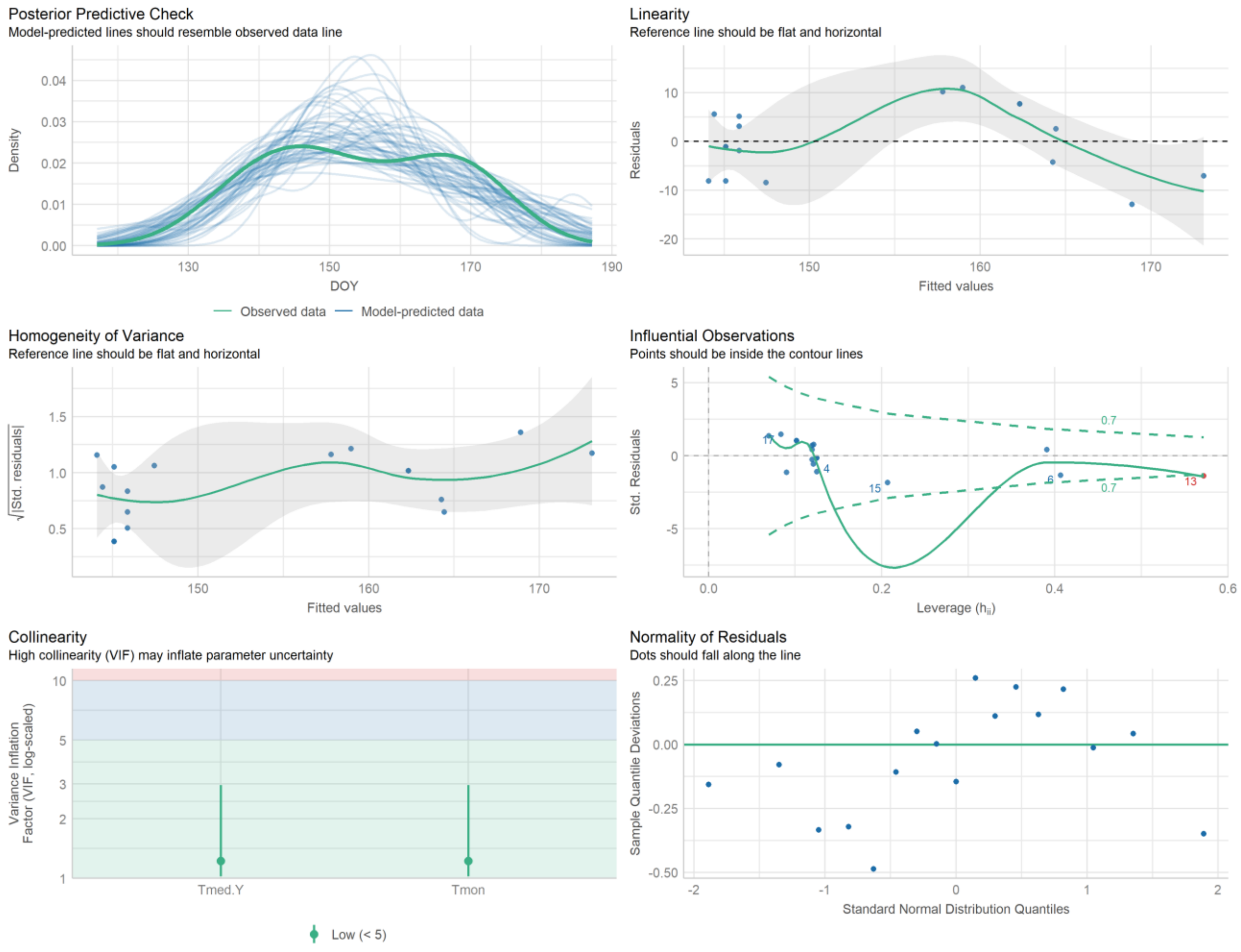

1.57. MLM - FBF - Hormathophylla spinosa

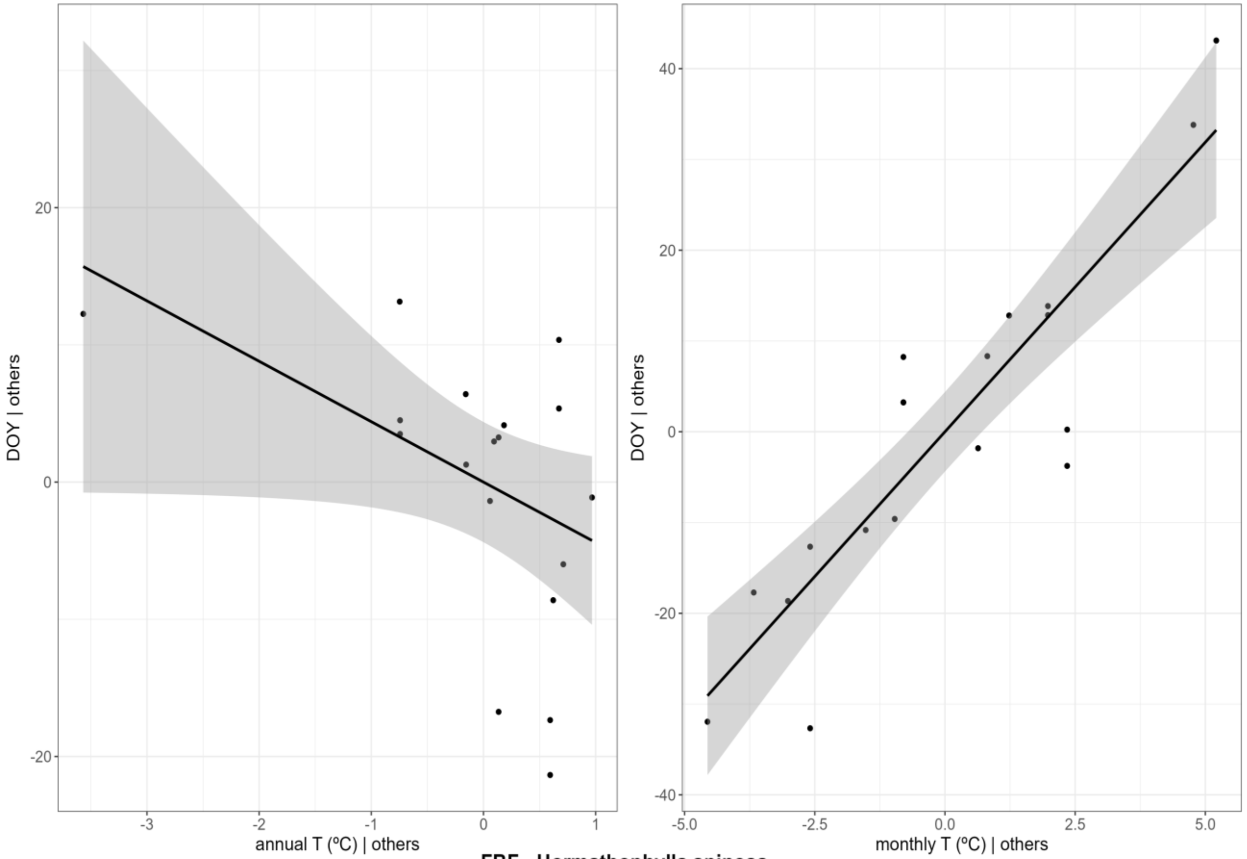

DOY = 101.79 (-4.40*annual T (ºC)) + (+6.38*monthly T (ºC))

1.57.1. Diagnostics - MLM - FBF - Hormathophylla spinosa

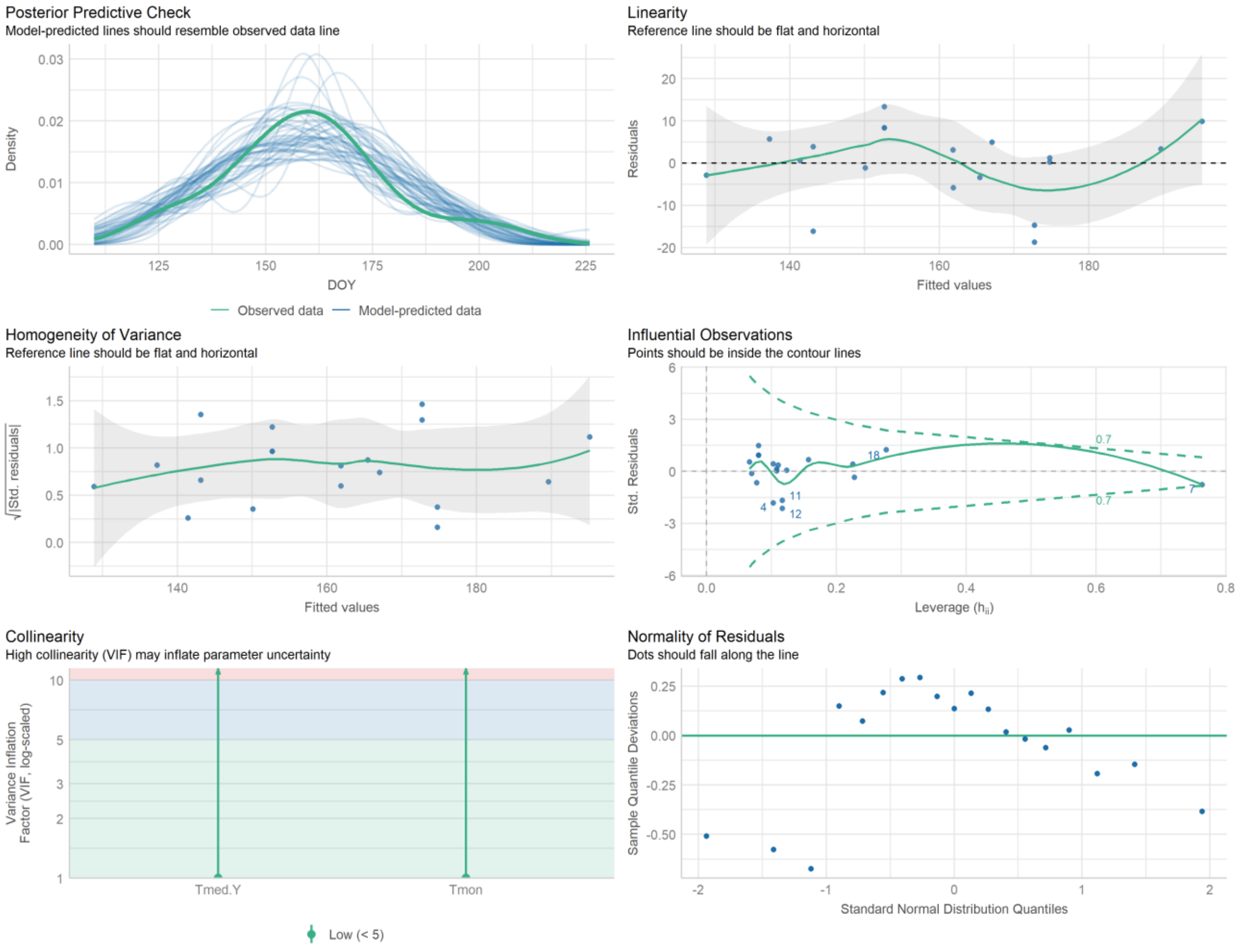

1.58. MLM - F - Hormathophylla spinosa

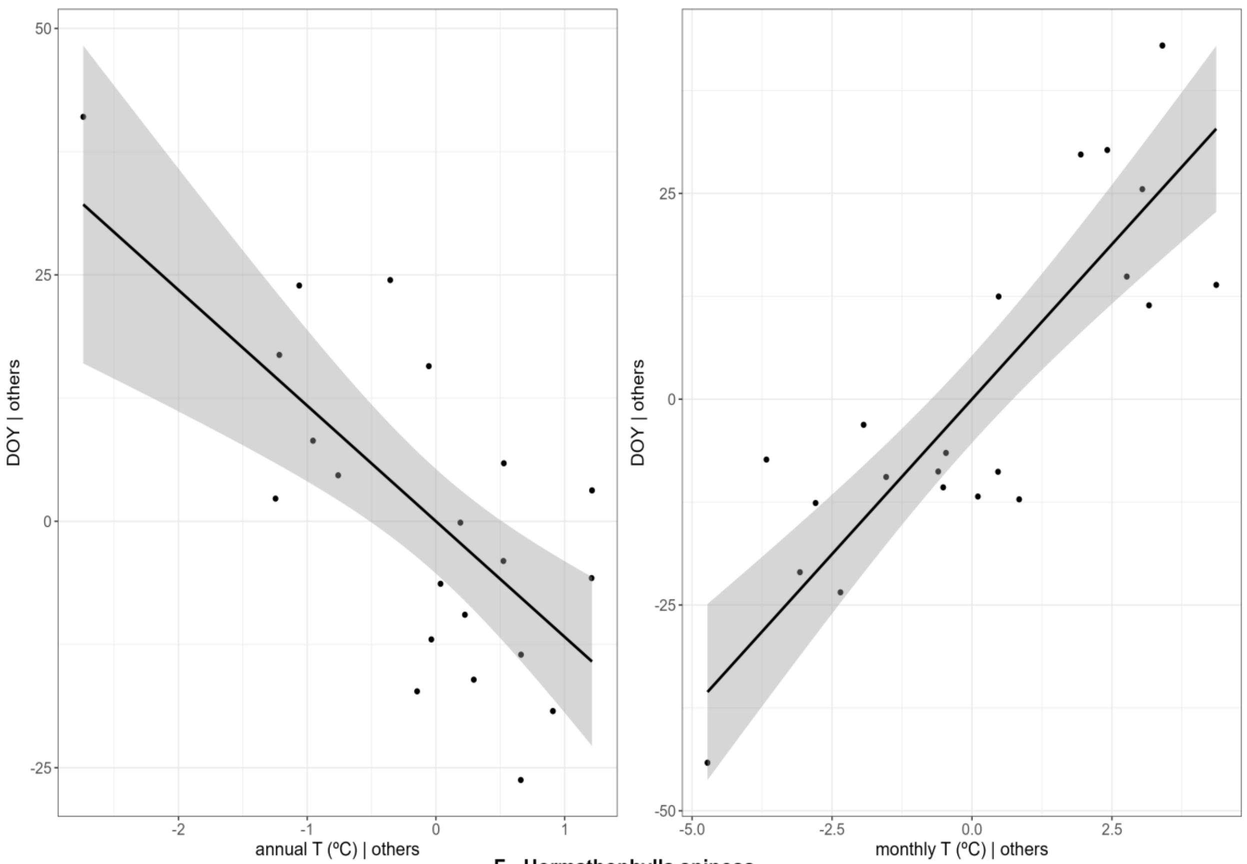

DOY = 202.32 (-11.73*annual T (ºC)) + (+7.53*monthly T (ºC))

1.58.1. Diagnostics - MLM - F - Hormathophylla spinosa

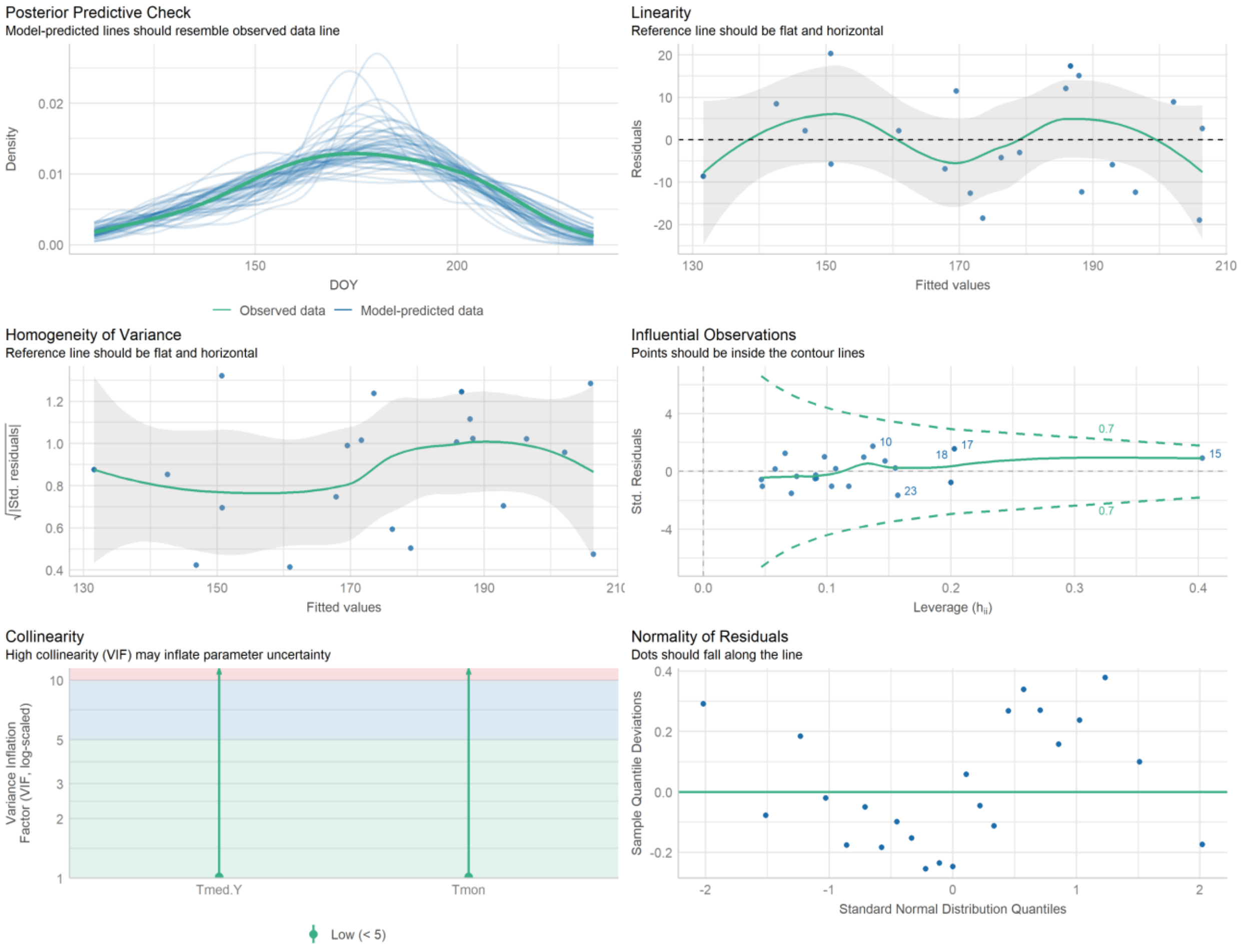

1.59. MLM - FBF - Juniperus oxycedrus

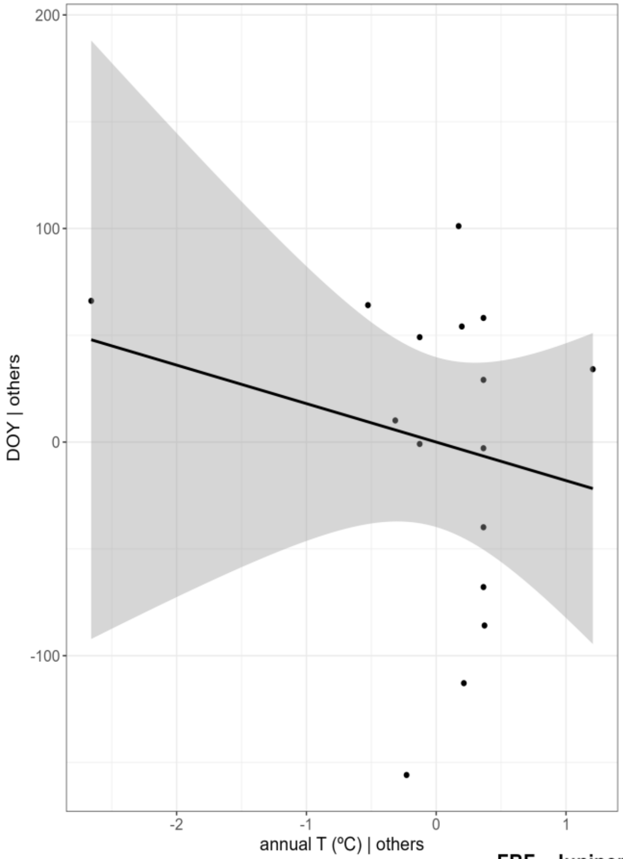

FBF - Juniperus oxycedrus

DOY = 539.65 (-18.02*annual T (ºC))

1.59.1. Diagnostics - MLM - FBF - Juniperus oxycedrus

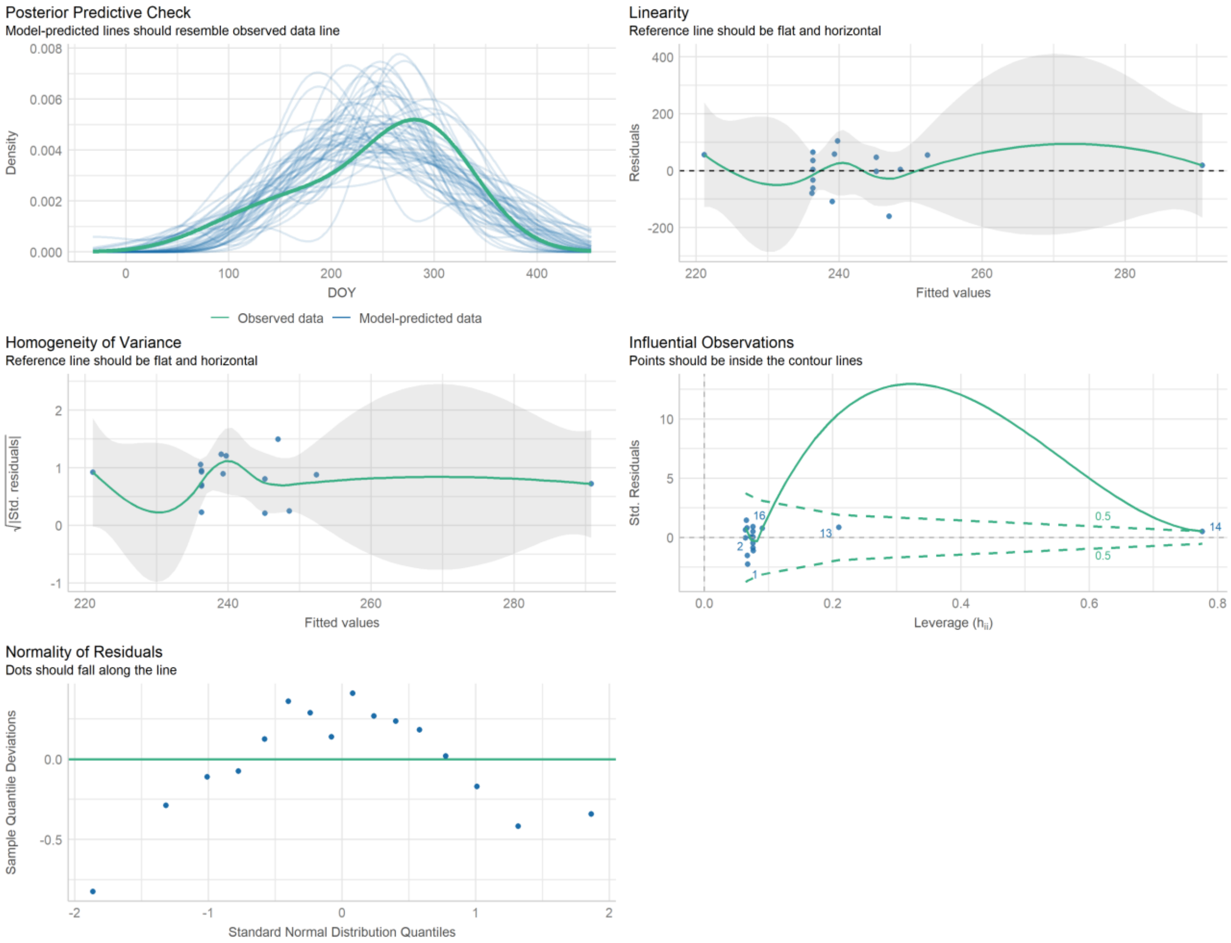

1.60. MLM - F - Juniperus oxycedrus

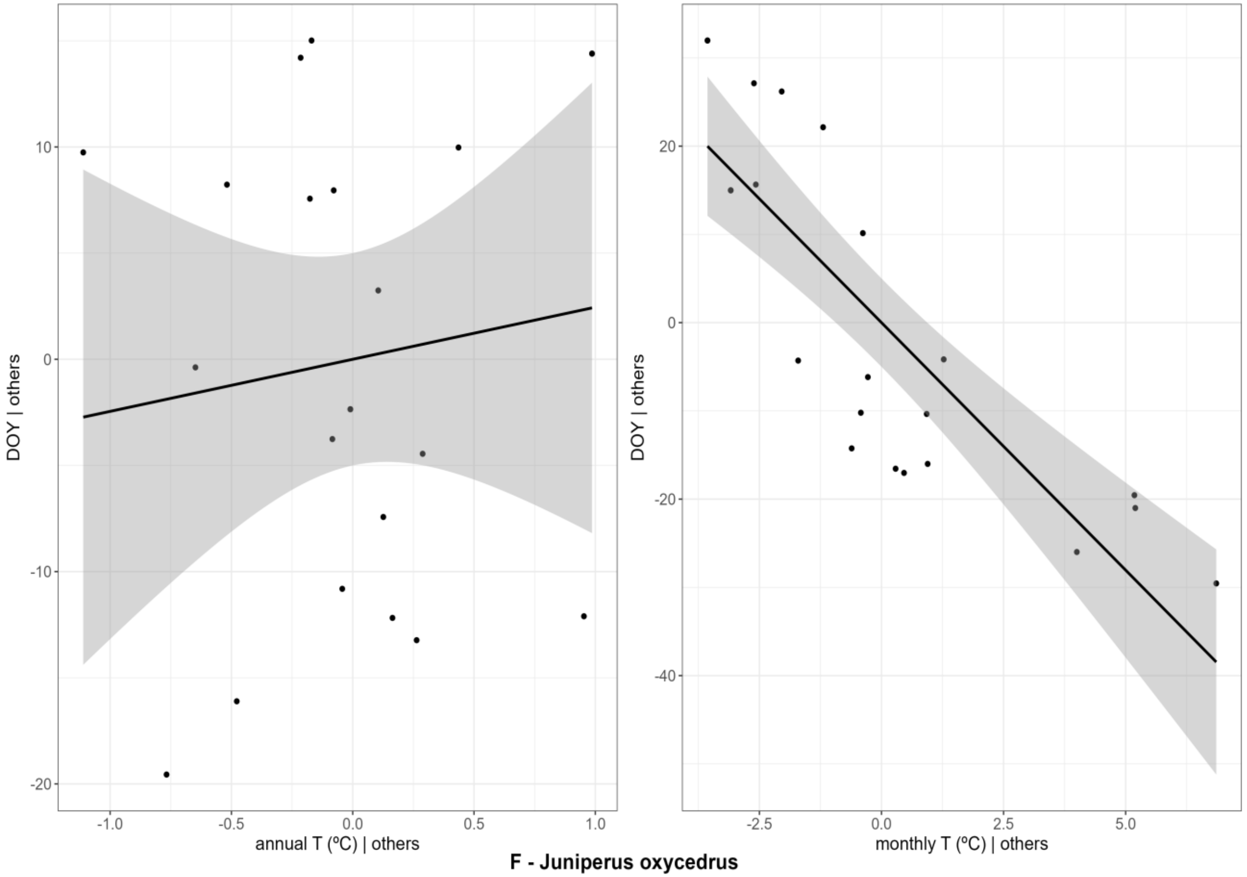

DOY = 353.89 (+2.45*annual T (ºC)) + (-5.61*monthly T (ºC))

1.60.1. Diagnostics - MLM - F - Juniperus oxycedrus

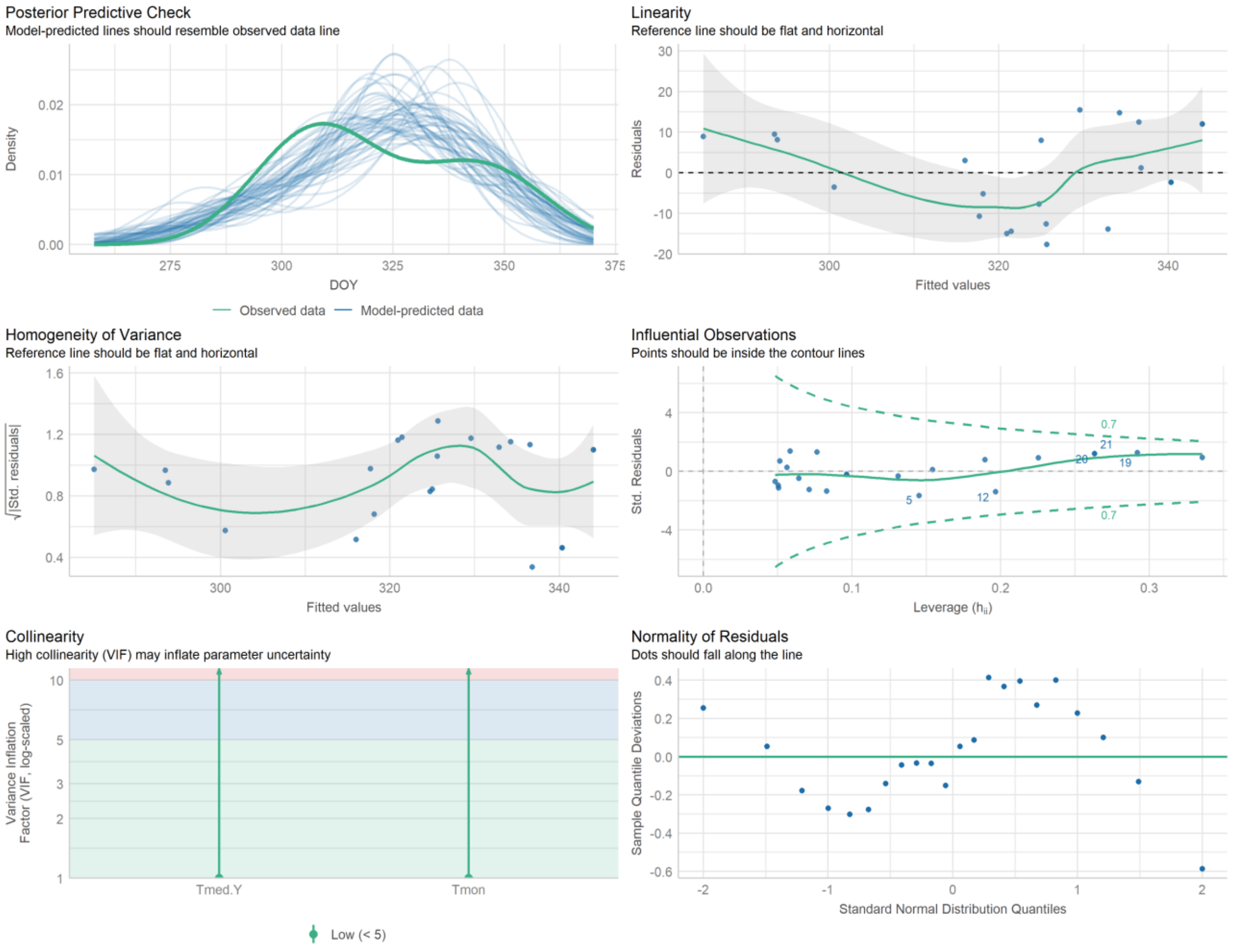

1.61. MLM - DVG - Juniperus oxycedrus

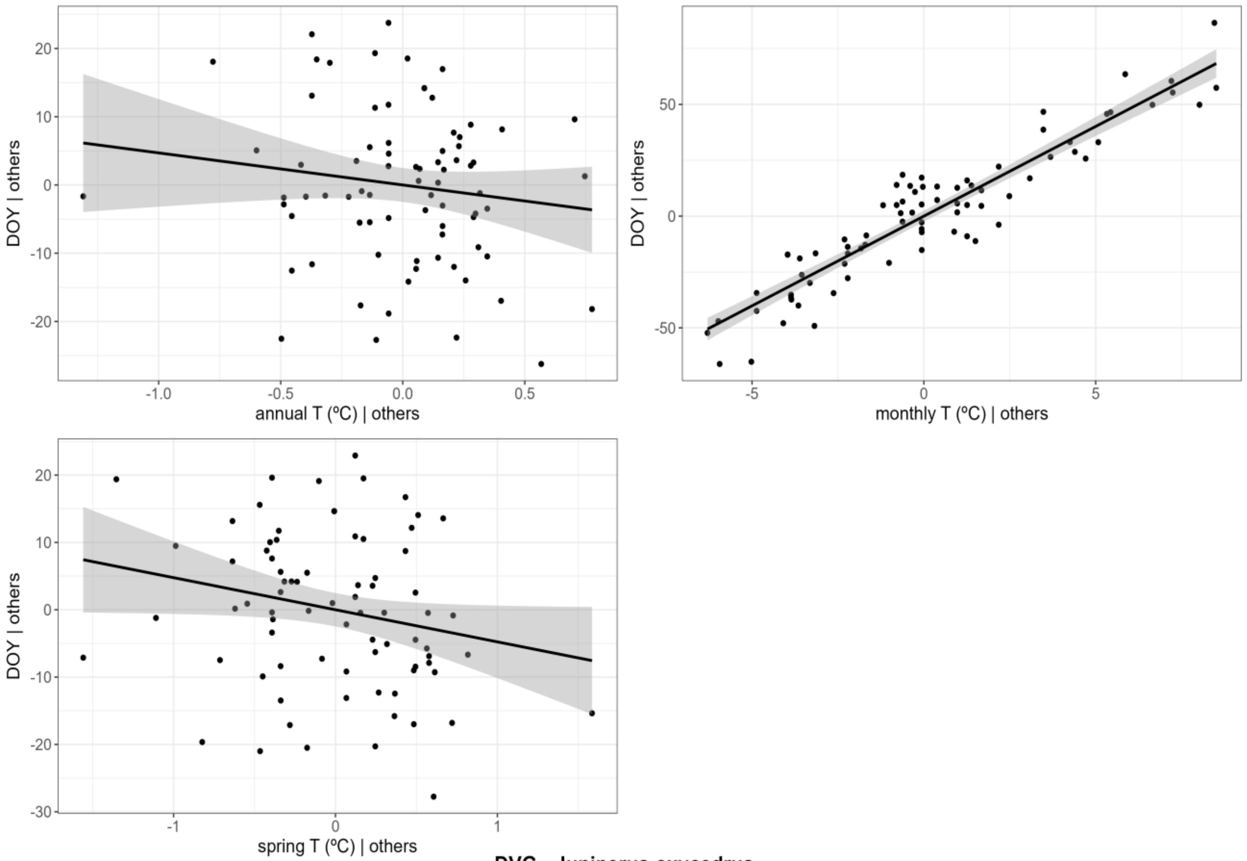

DVG - Juniperus oxycedrus

DOY = 140.99 (-4.70*annual T (ºC)) + (+8.03*monthly T (ºC)) + (-4.77*spring T (ºC))

1.61.1. Diagnostics - MLM - DVG - Juniperus oxycedrus

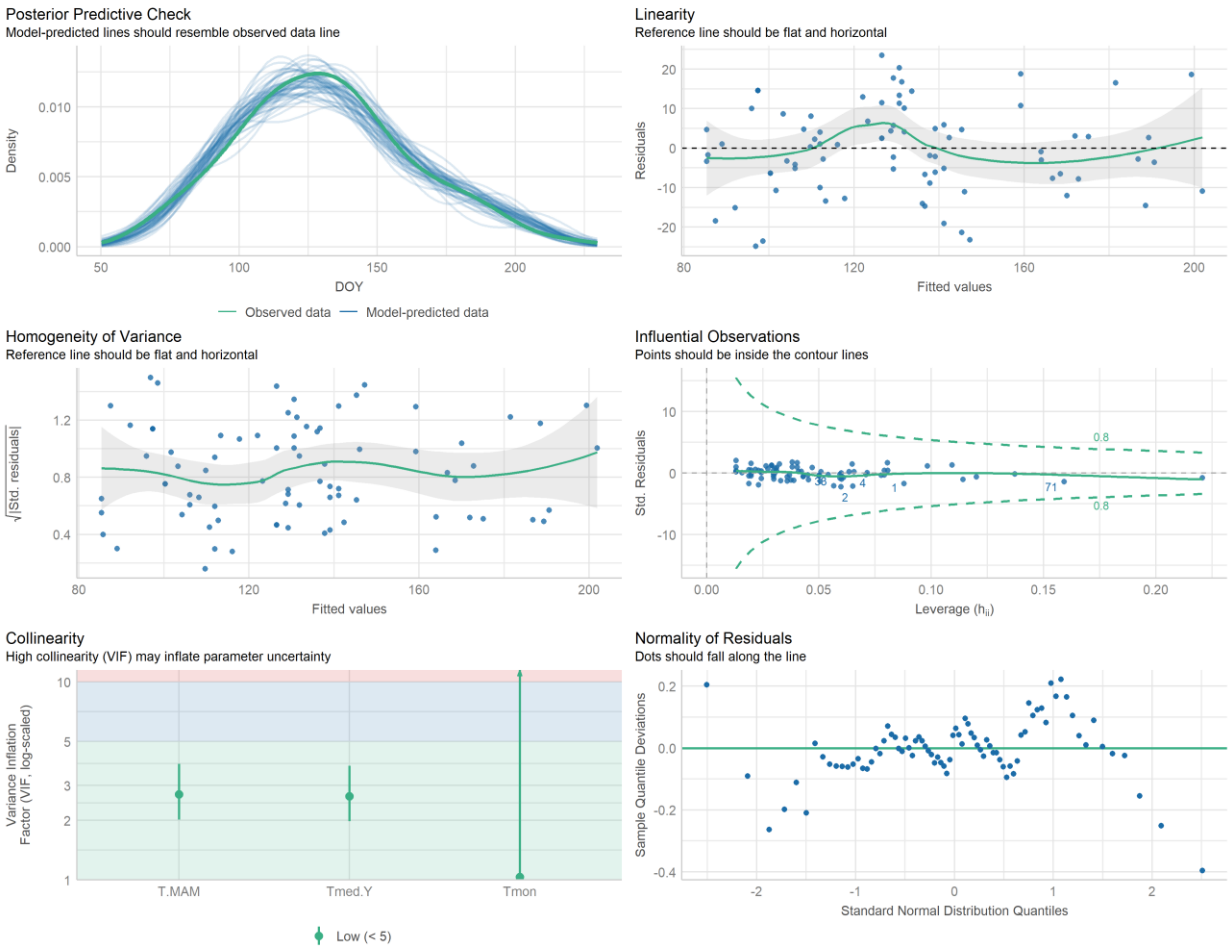

1.62. MLM - FS - Juniperus phoenicea

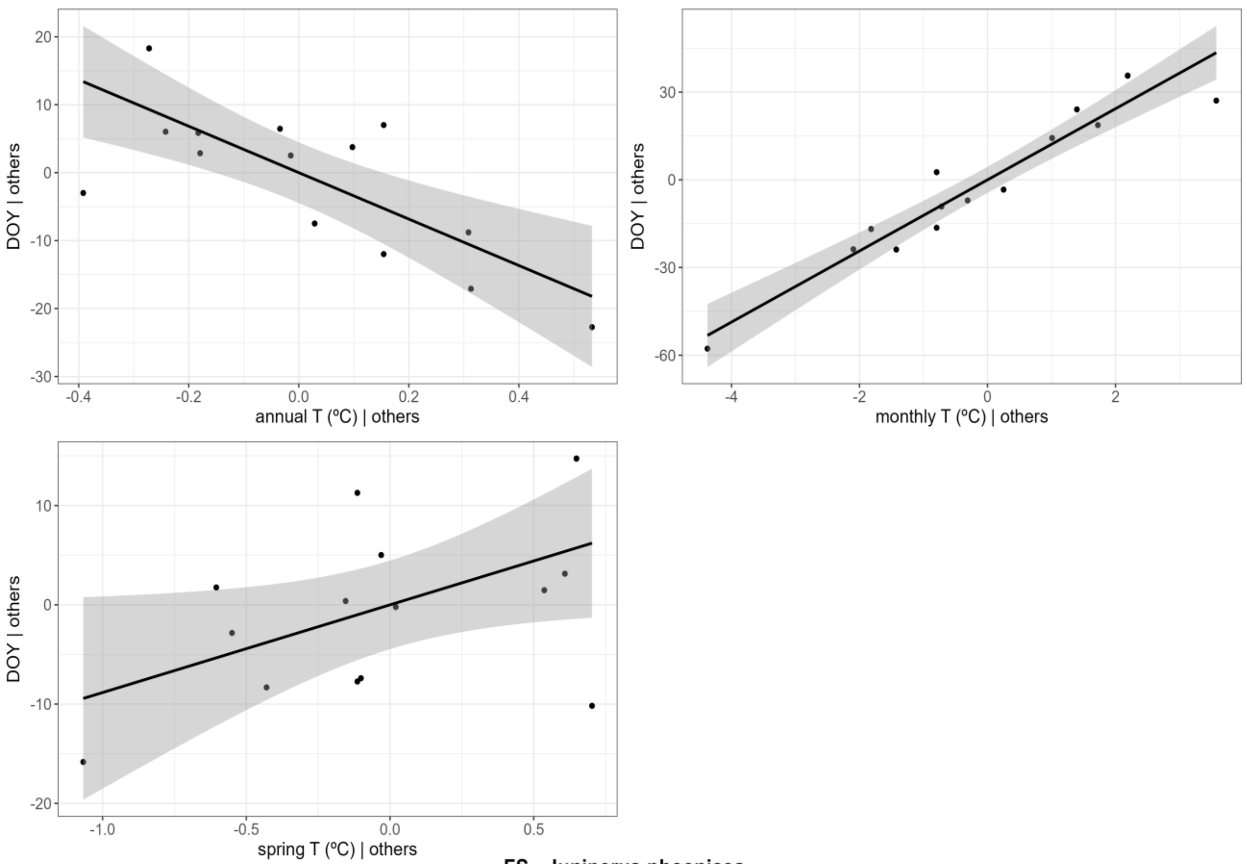

FS - Juniperus phoenicea

DOY = 342.97 (-34.17*annual T (ºC)) + (+12.16*monthly T (ºC)) + (+8.84*spring T (ºC))

1.62.1. Diagnostics - MLM - FS - Juniperus phoenicea

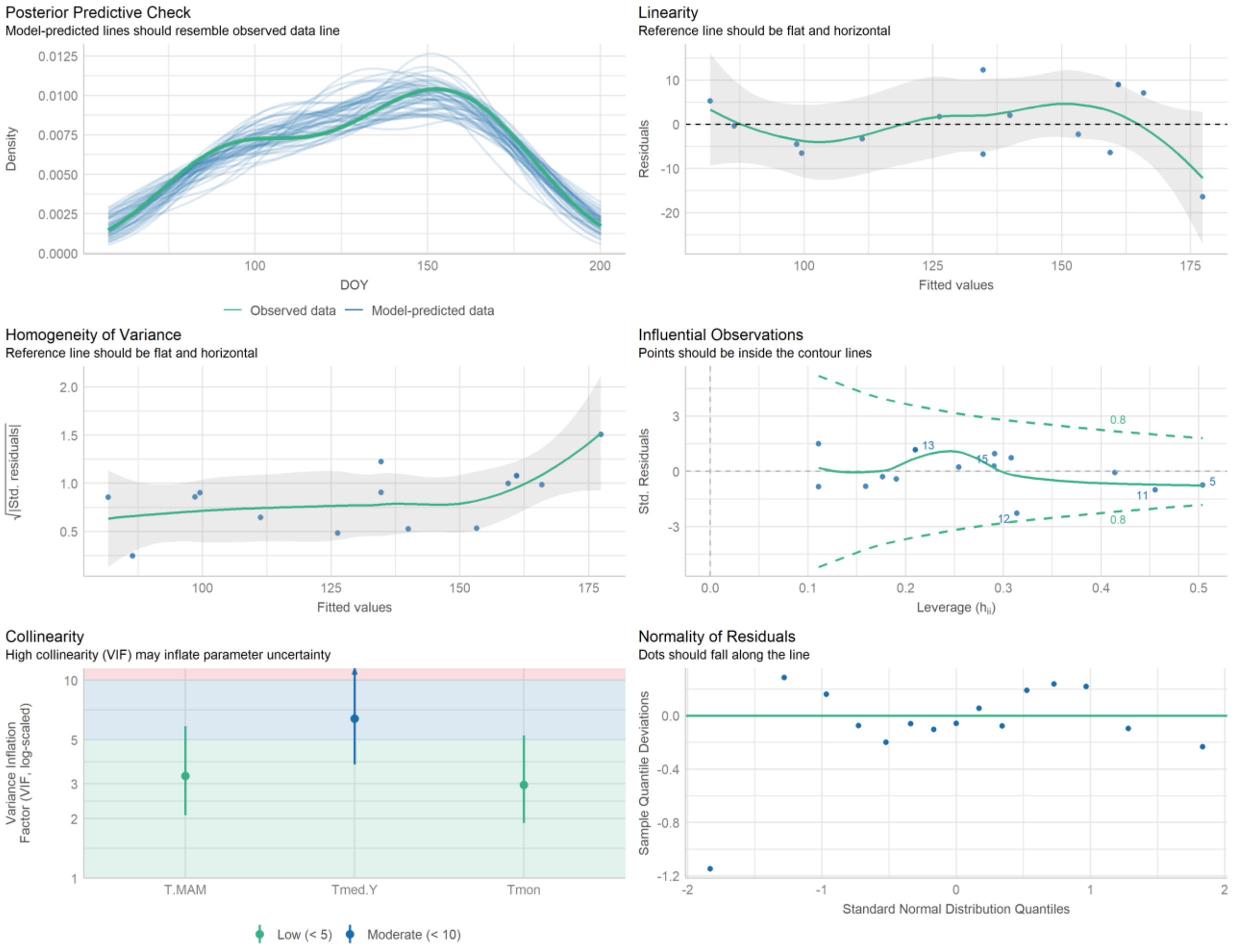

1.63. MLM - DVG - Juniperus sabina

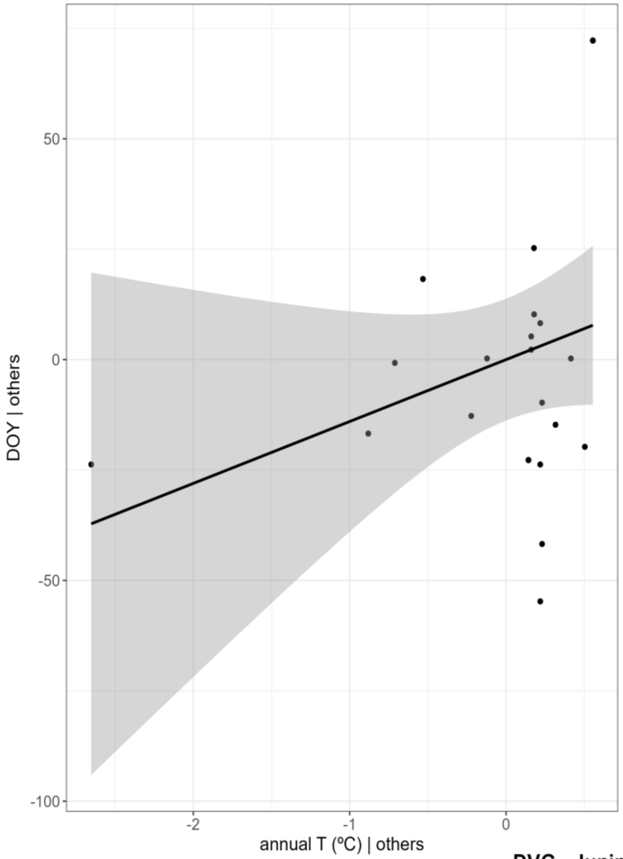

DVG - Juniperus sabina

DOY = -45.98 (+14.02*annual T (ºC))

1.63.1. Diagnostics - MLM - DVG - Juniperus sabina

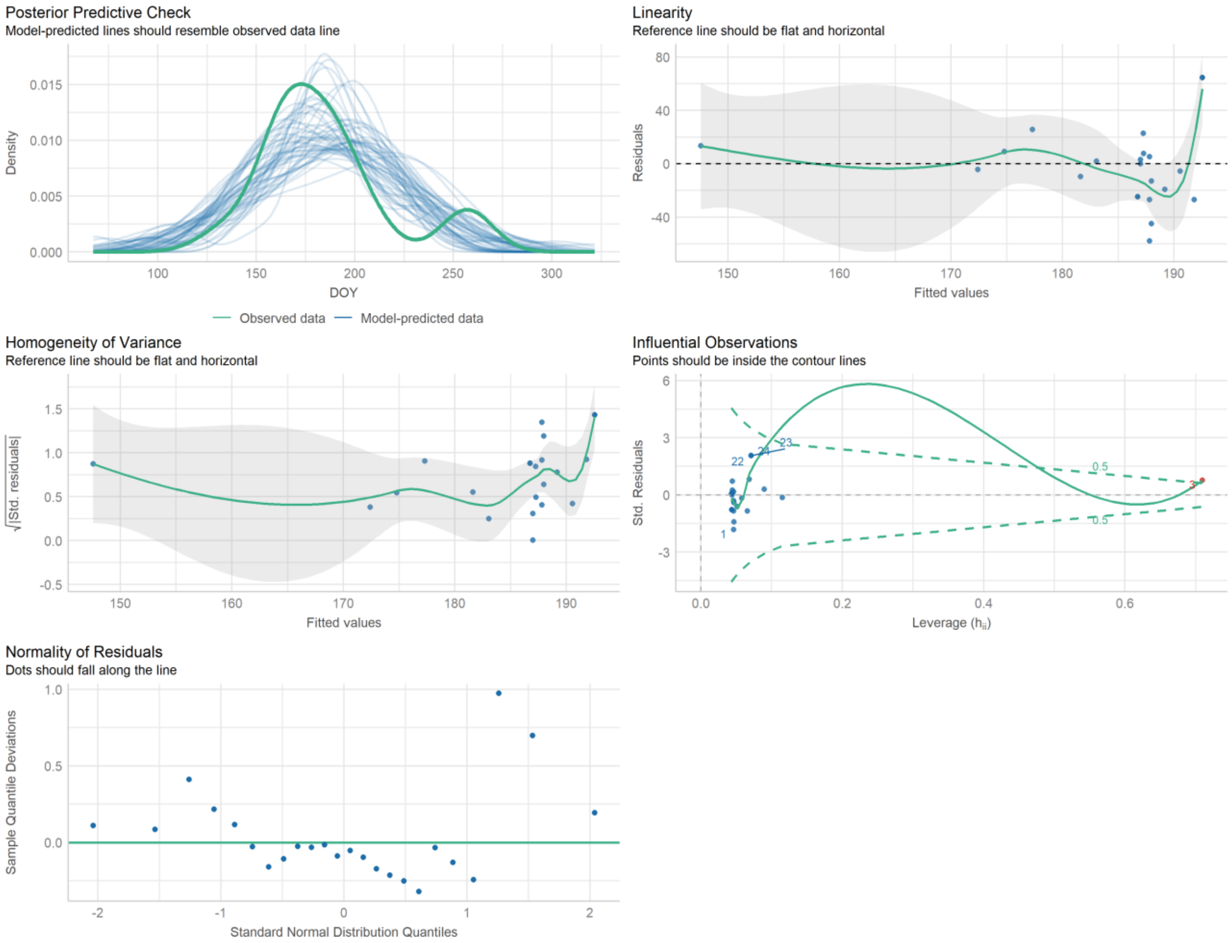

1.64. MLM - DVG - Juniperus turbinata

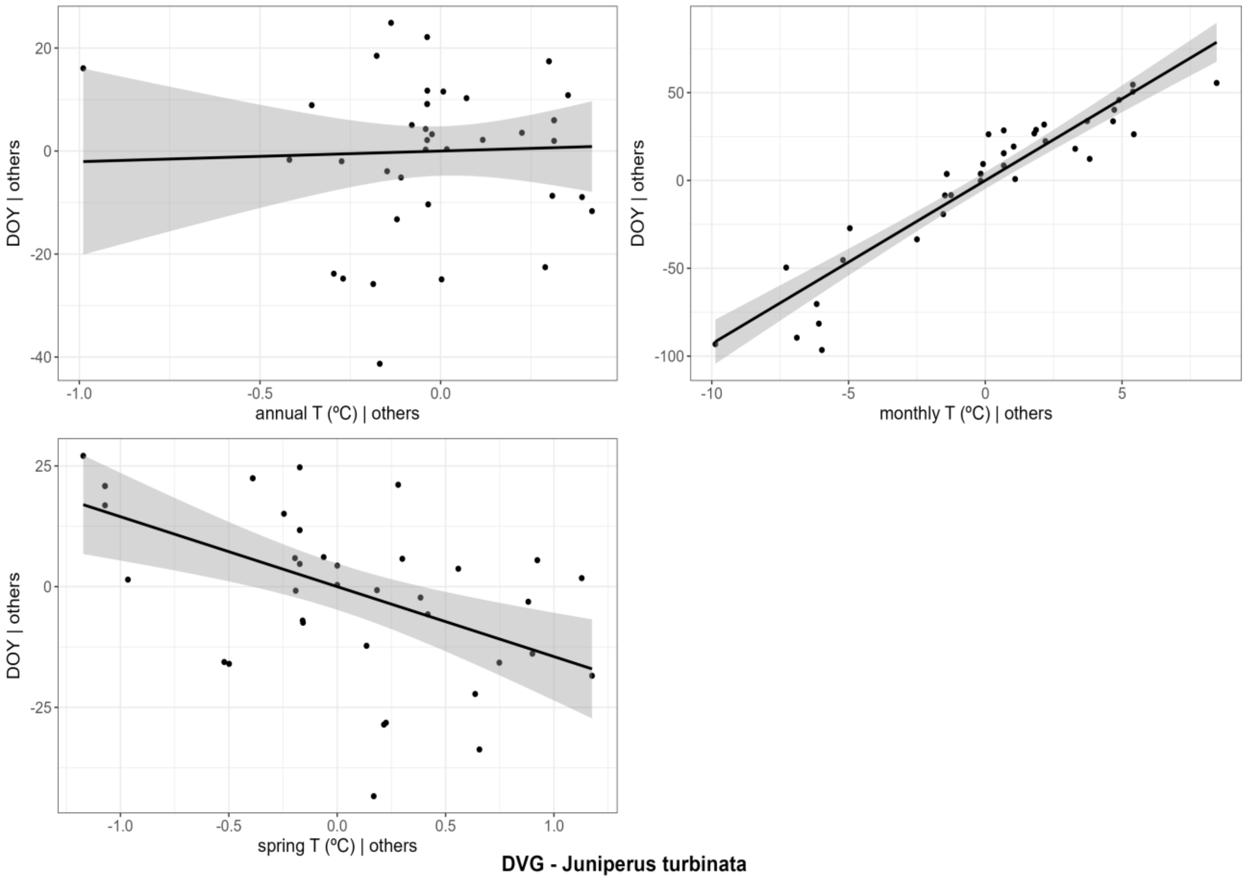

DVG - Juniperus turbinata

DOY = 145.69 (+2.07*annual T (ºC)) + (+9.30*monthly T (ºC)) + (-14.49*spring T (ºC))

1.64.1. Diagnostics - MLM - DVG - Juniperus turbinata

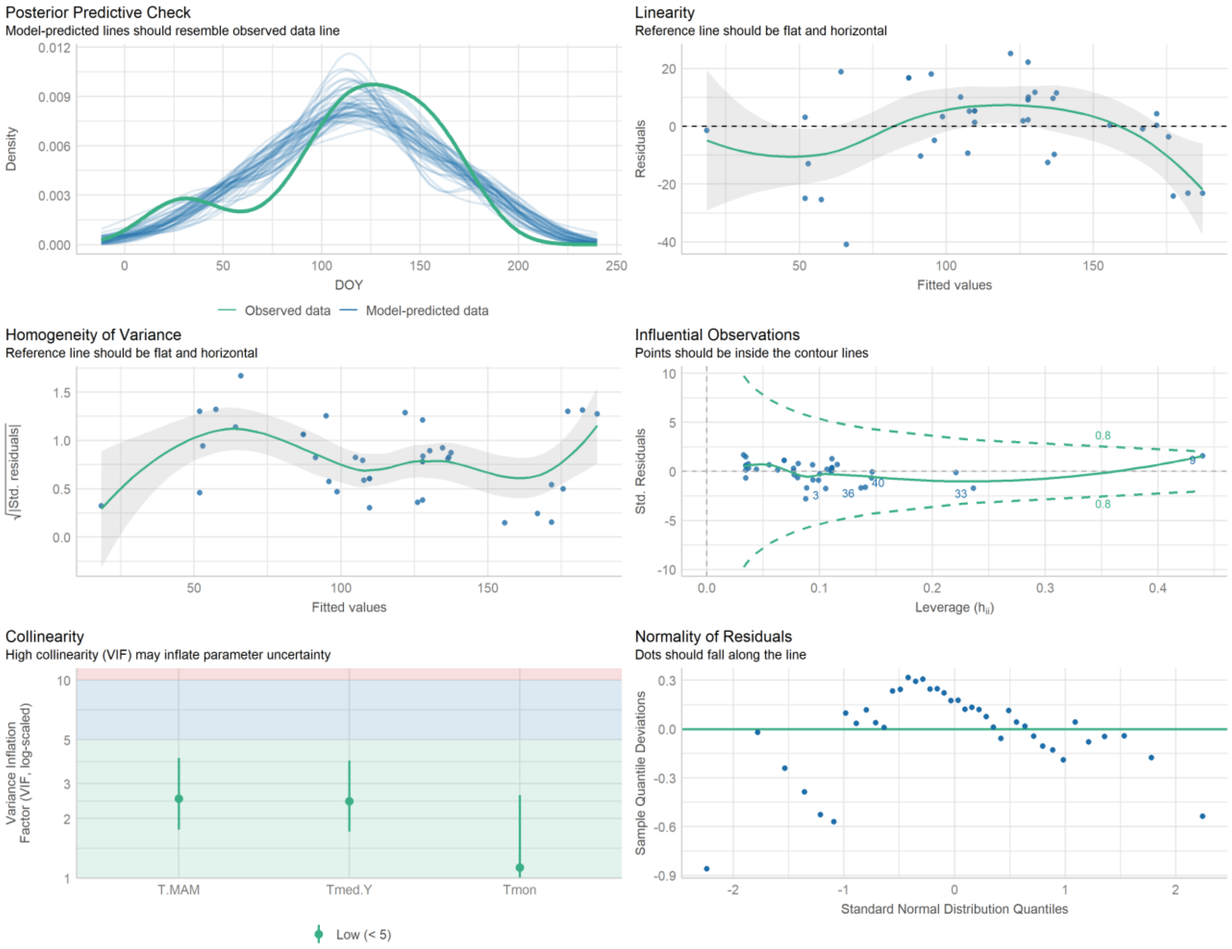

1.65. MLM - F - Klasea baetica

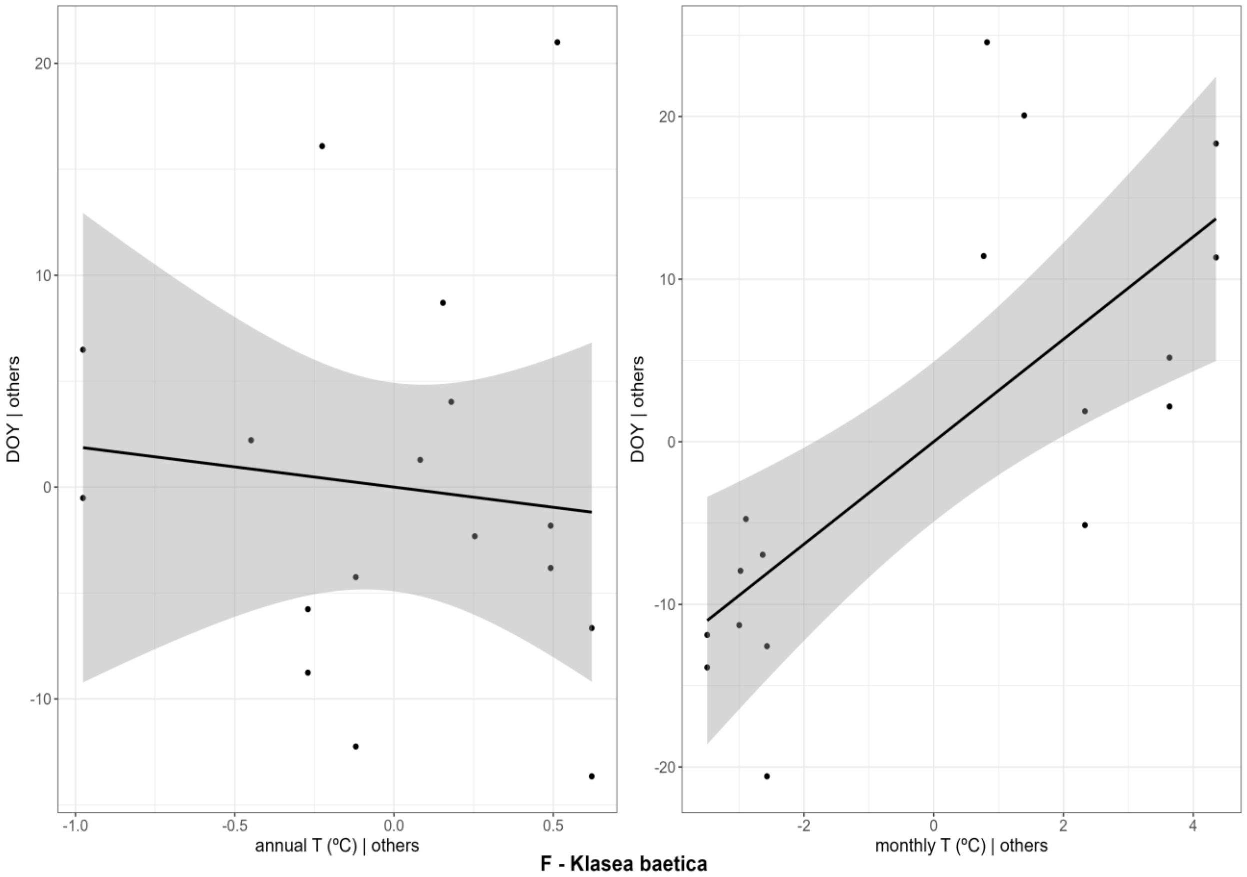

DOY = 120.36 (-1.91*annual T (ºC)) + (+3.15*monthly T (ºC))

1.65.1. Diagnostics - MLM - F - Klasea baetica

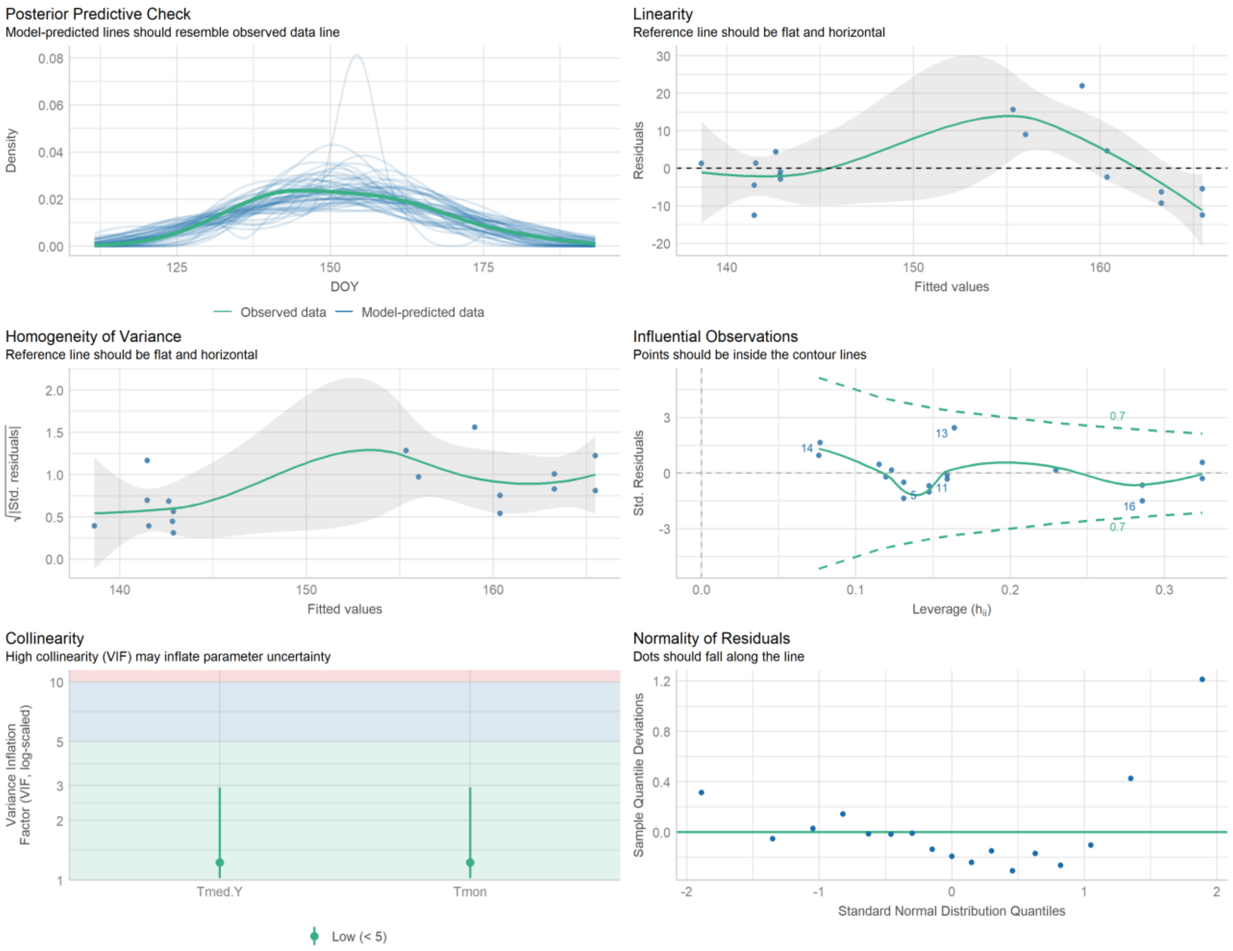

1.66. MLM - FBF - Lavandula lanata

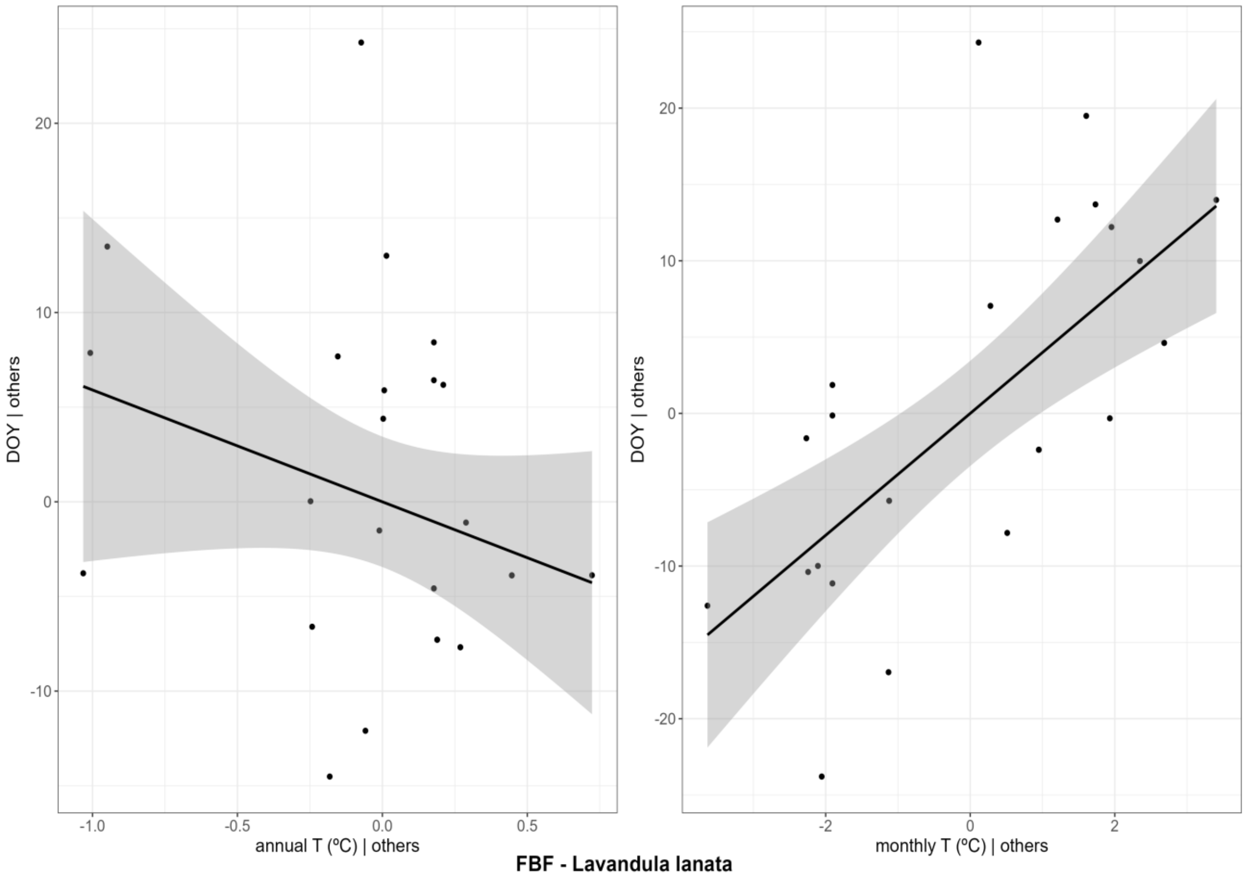

DOY = 182.63 (-5.91*annual T (ºC)) + (+3.99*monthly T (ºC))

1.66.1. Diagnostics - MLM - FBF - Lavandula lanata

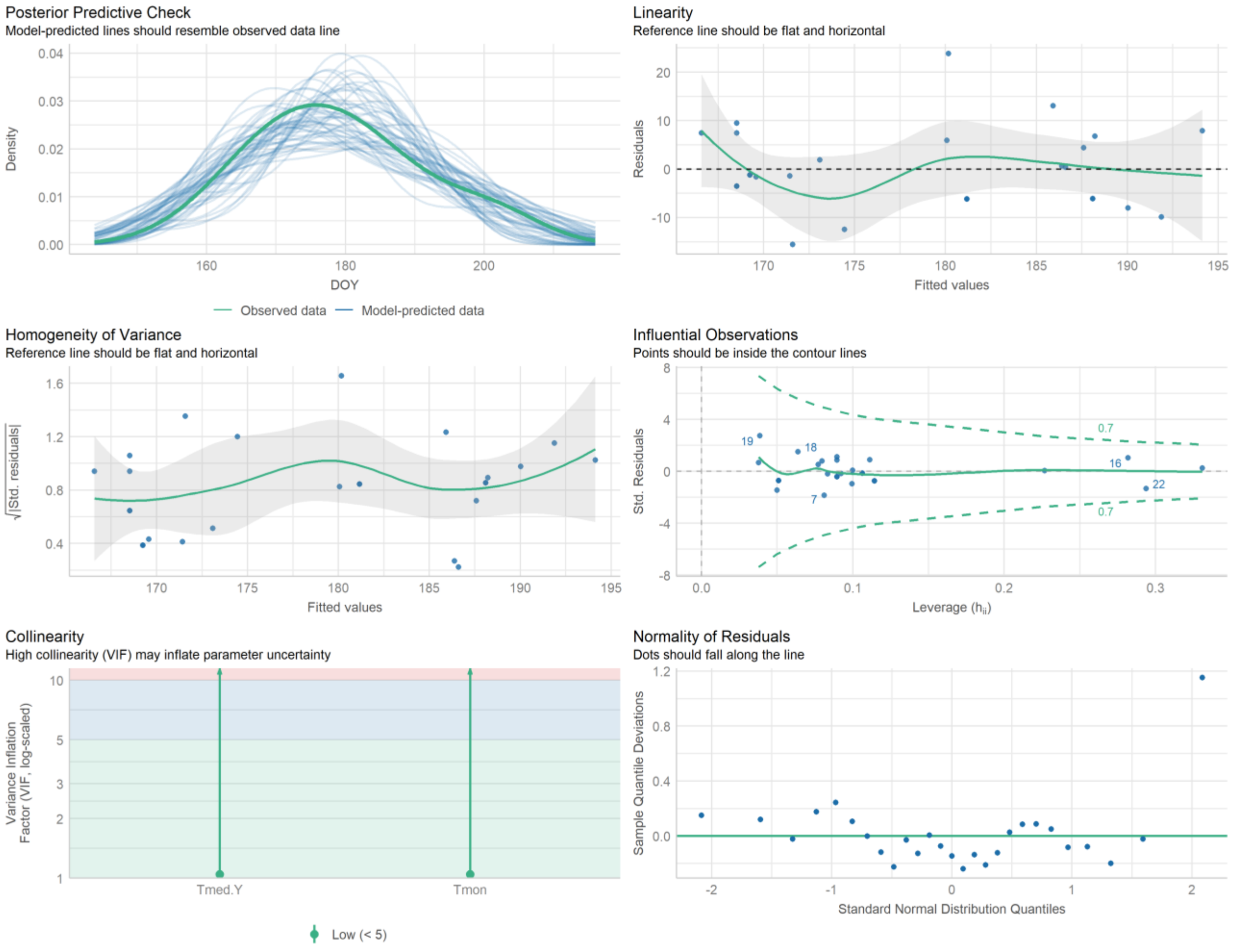

1.67. MLM - DVG - Lavandula lanata

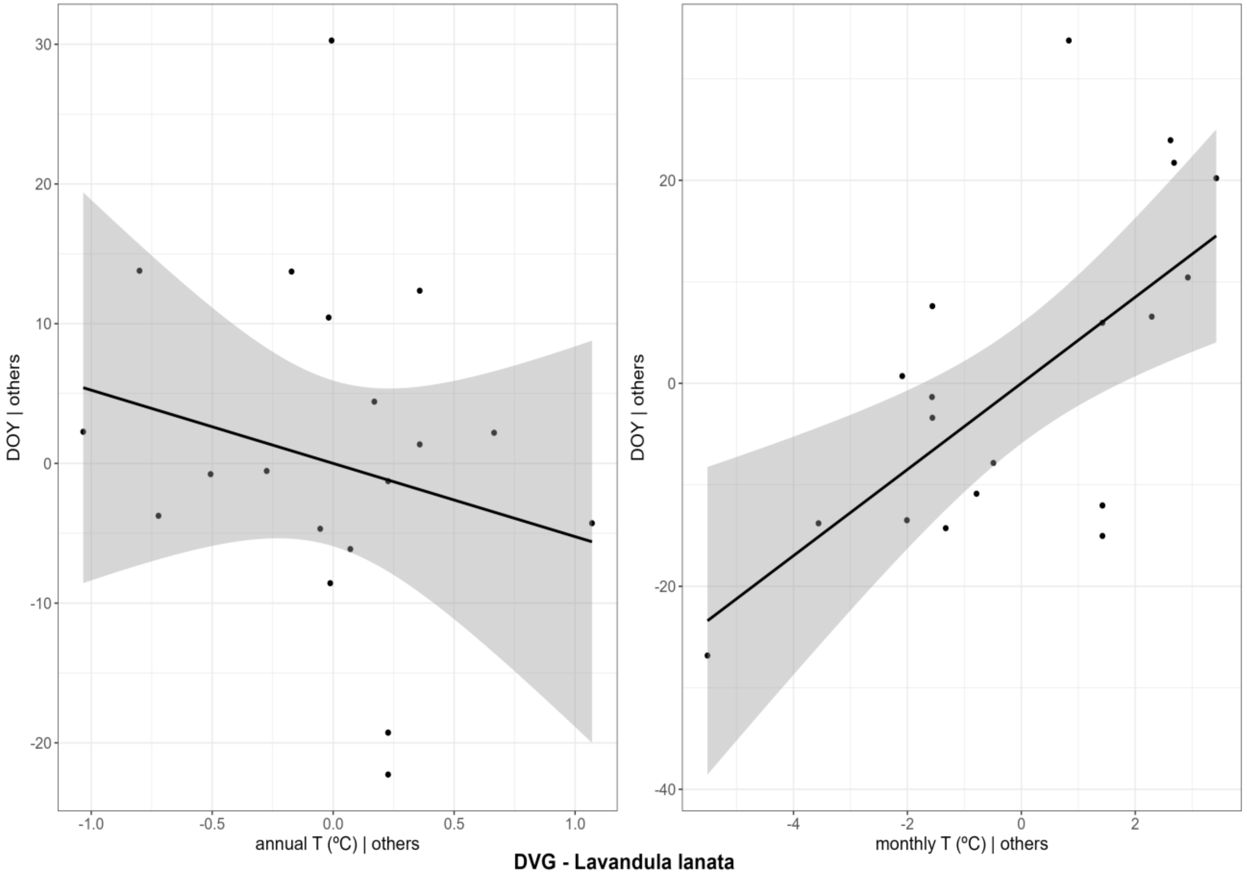

DOY = 159.10 (-5.24*annual T (ºC)) + (+4.25*monthly T (ºC))

1.67.1. Diagnostics - MLM - DVG - Lavandula lanata

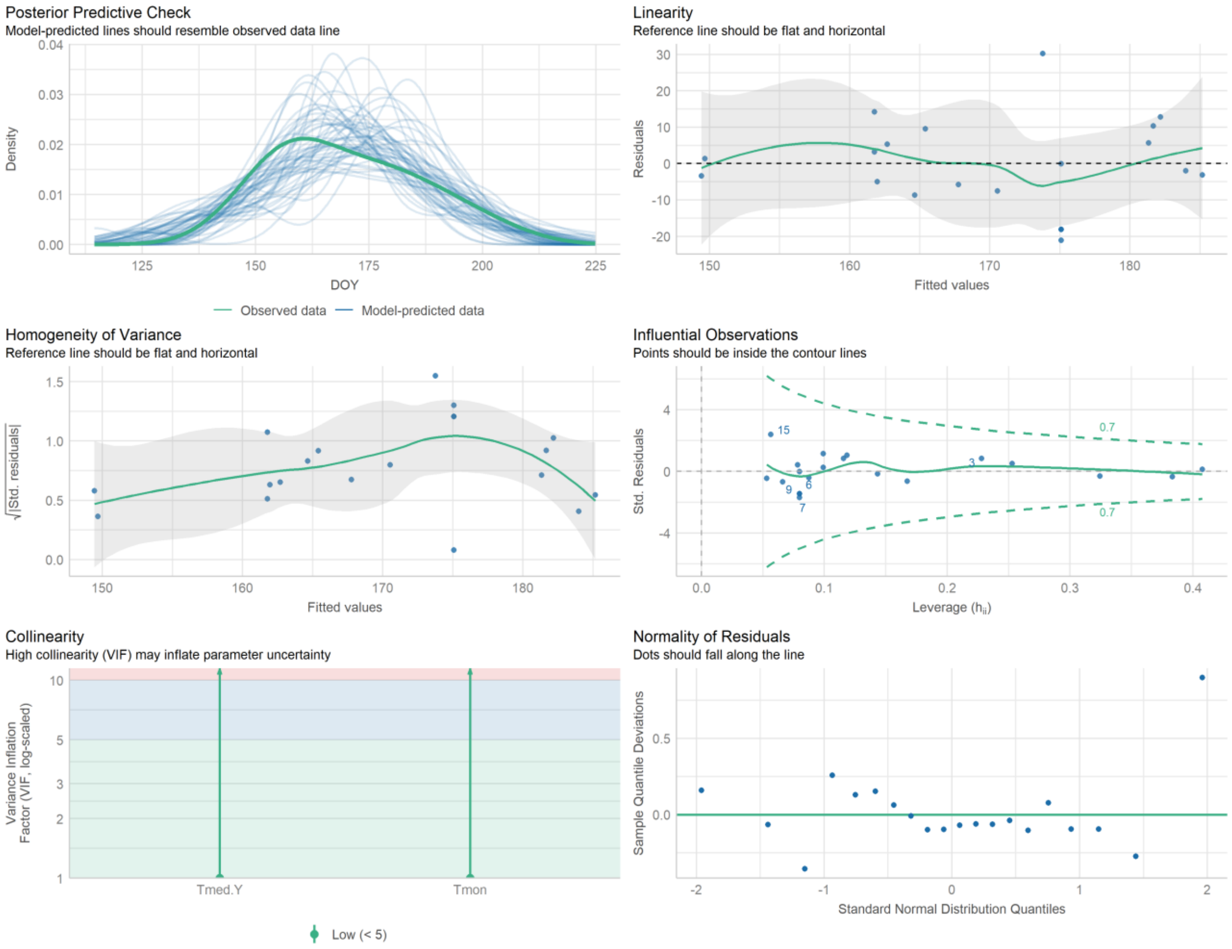

1.68. MLM - FBF - Lavandula stoechas

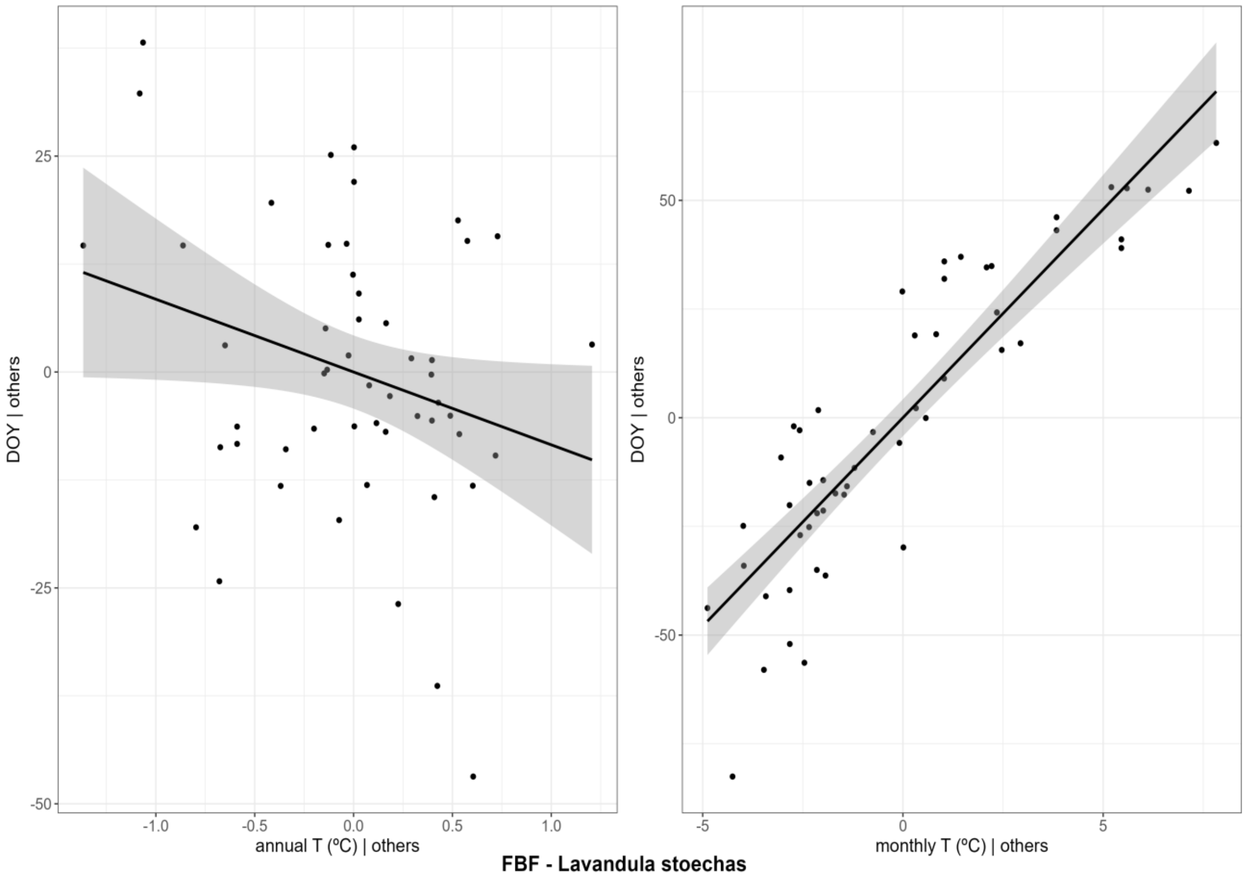

DOY = 100.36 (-8.43*annual T (ºC)) + (+9.59*monthly T (ºC))

1.68.1. Diagnostics - MLM - FBF - Lavandula stoechas

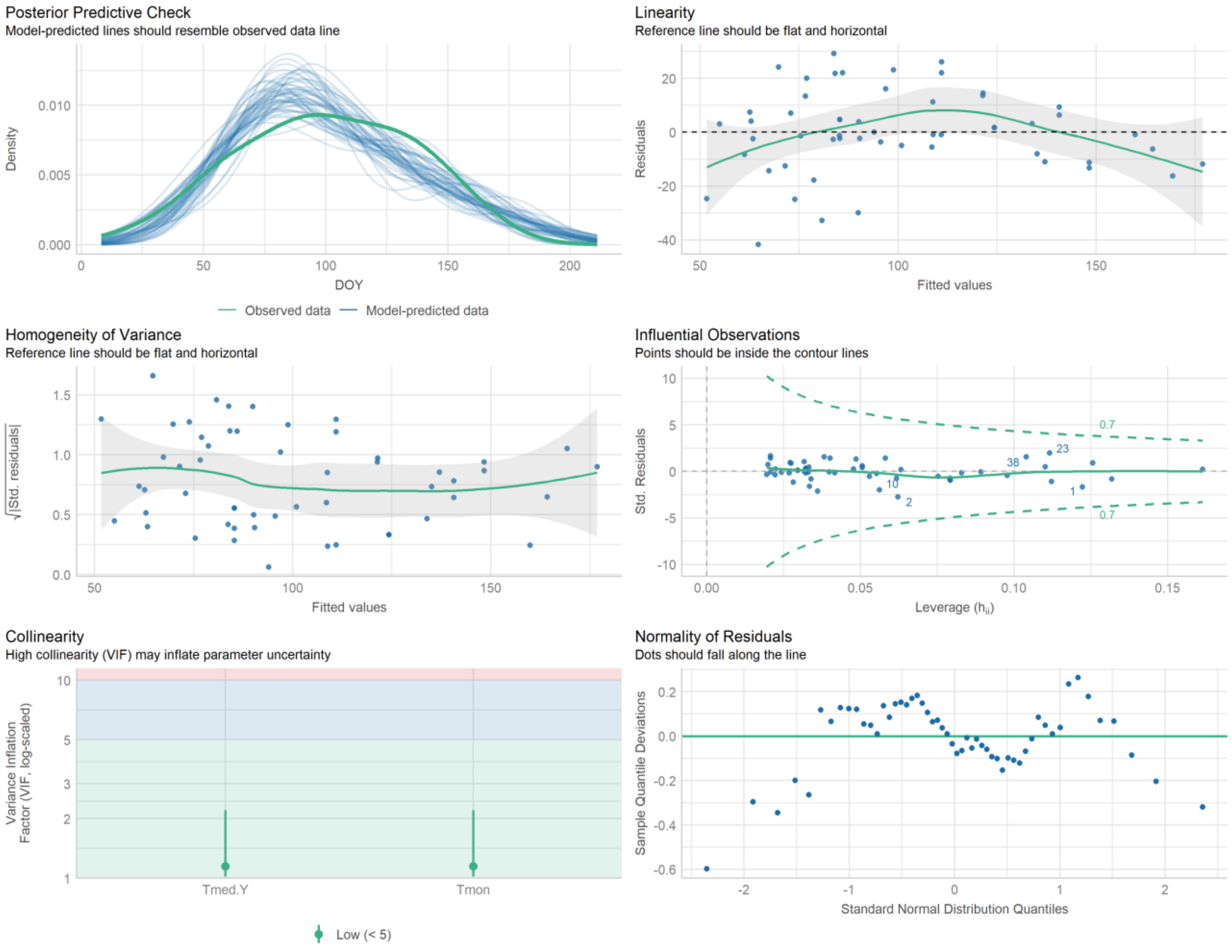

1.69. MLM - F - Leontodon boryi

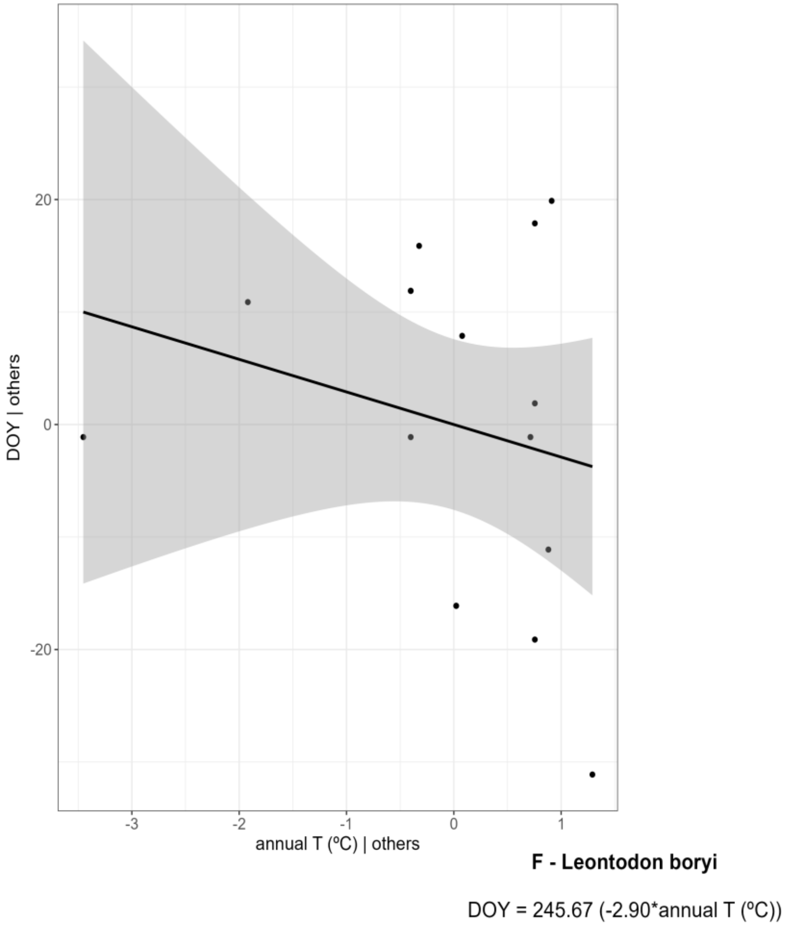

1.69.1. Diagnostics - MLM - F - Leontodon boryi

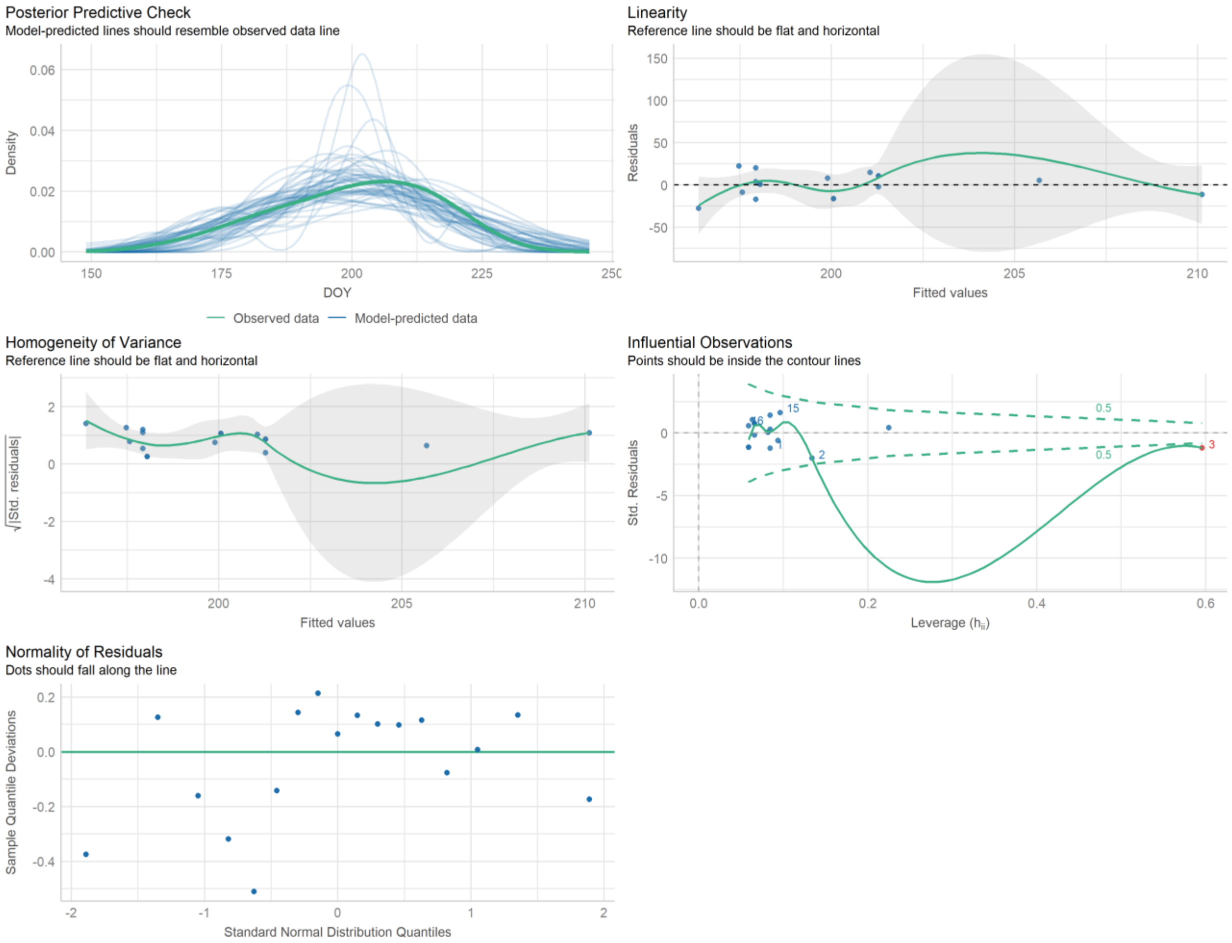

1.70. MLM - F - Linaria saturejoides

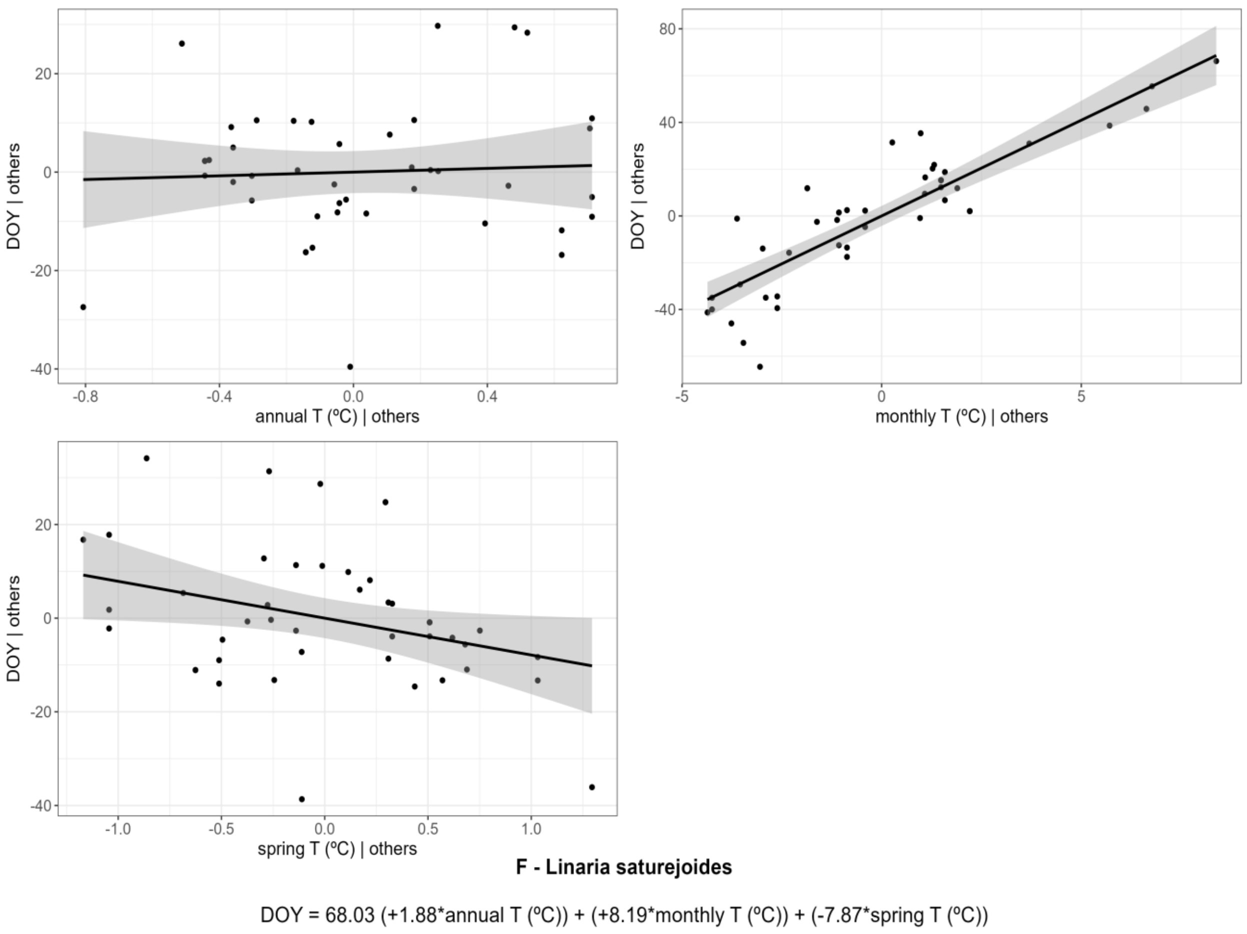

1.70.1. Diagnostics - MLM - F - Linaria saturejoides

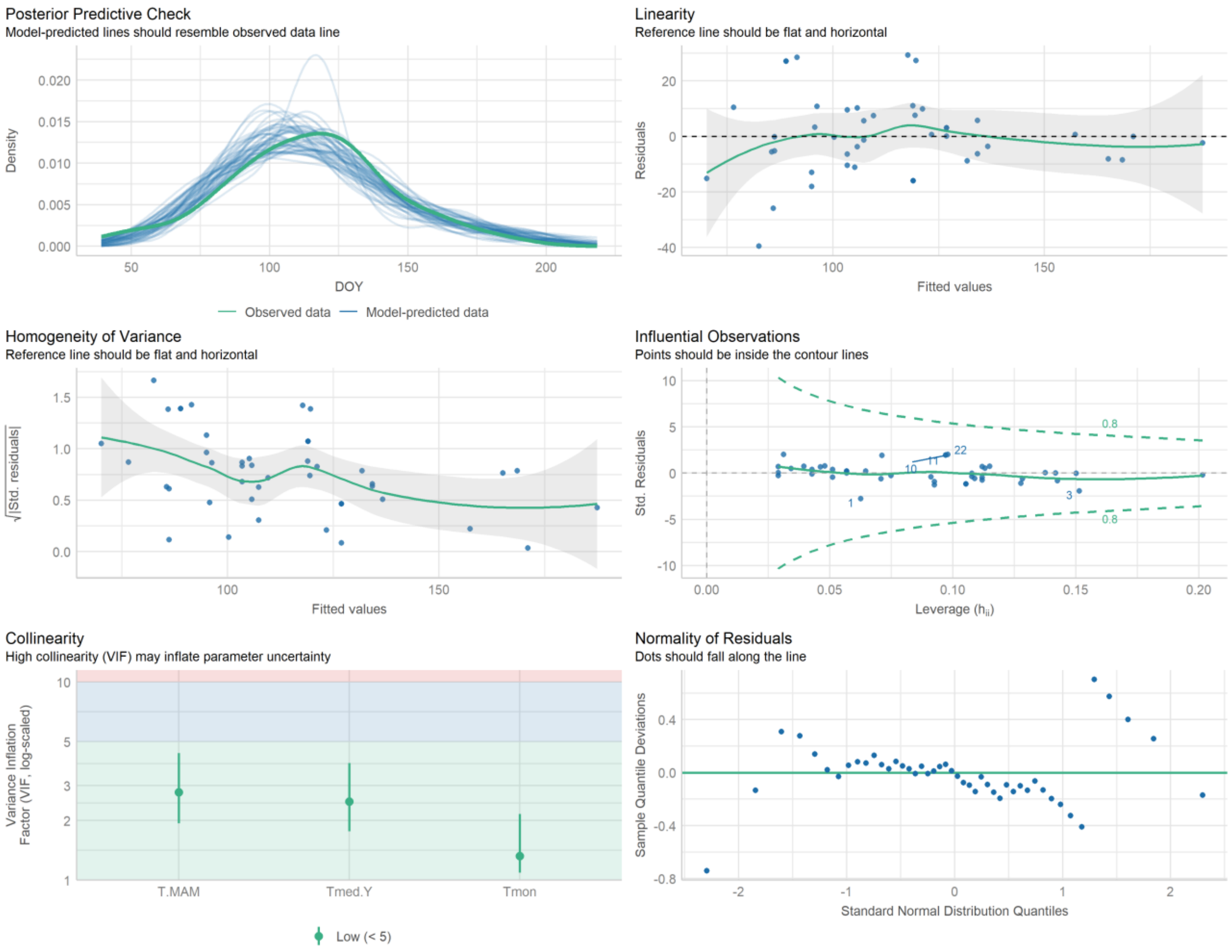

1.71. MLM - F - Lonicera etrusca

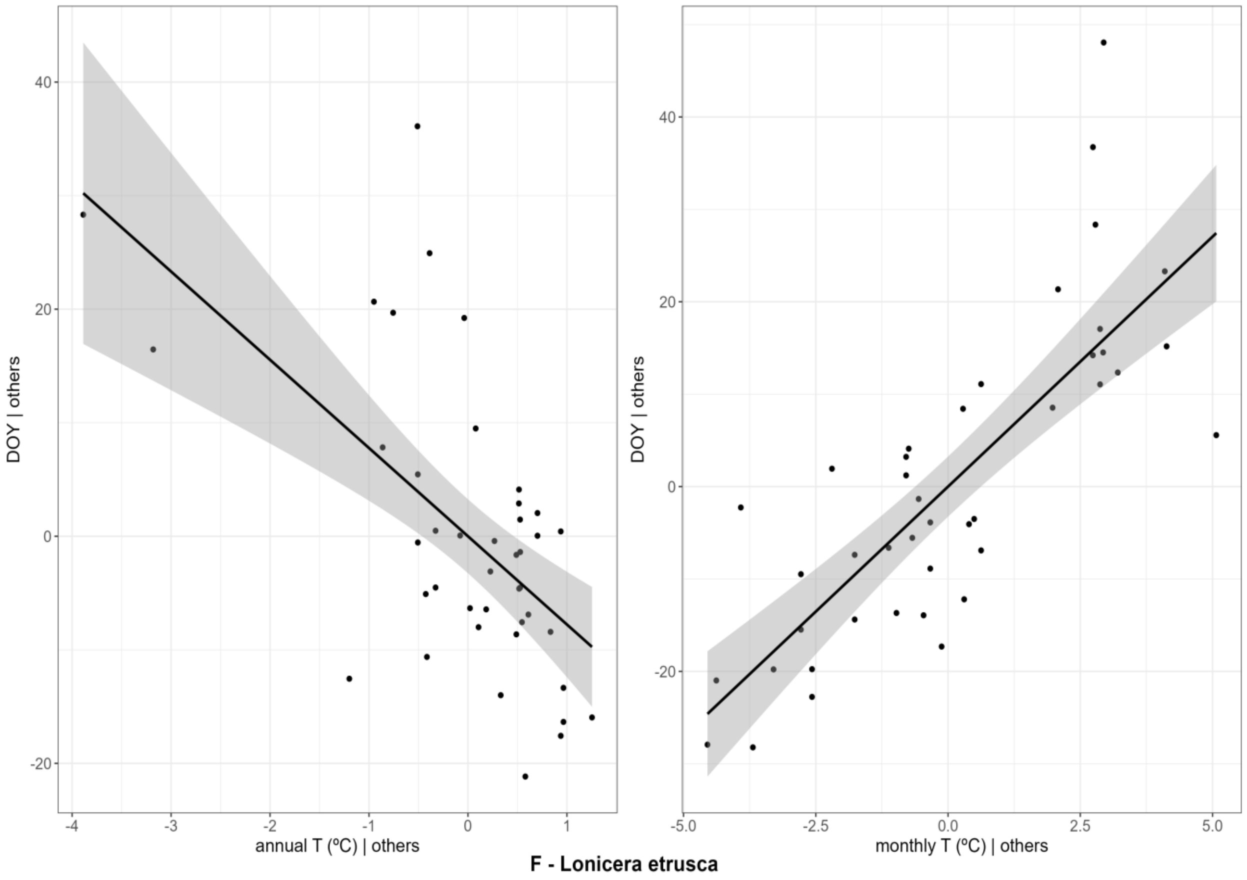

DOY = 179.60 (-7.77*annual T (ºC)) + (+5.41*monthly T (ºC))

1.71.1. Diagnostics - MLM - F - Lonicera etrusca

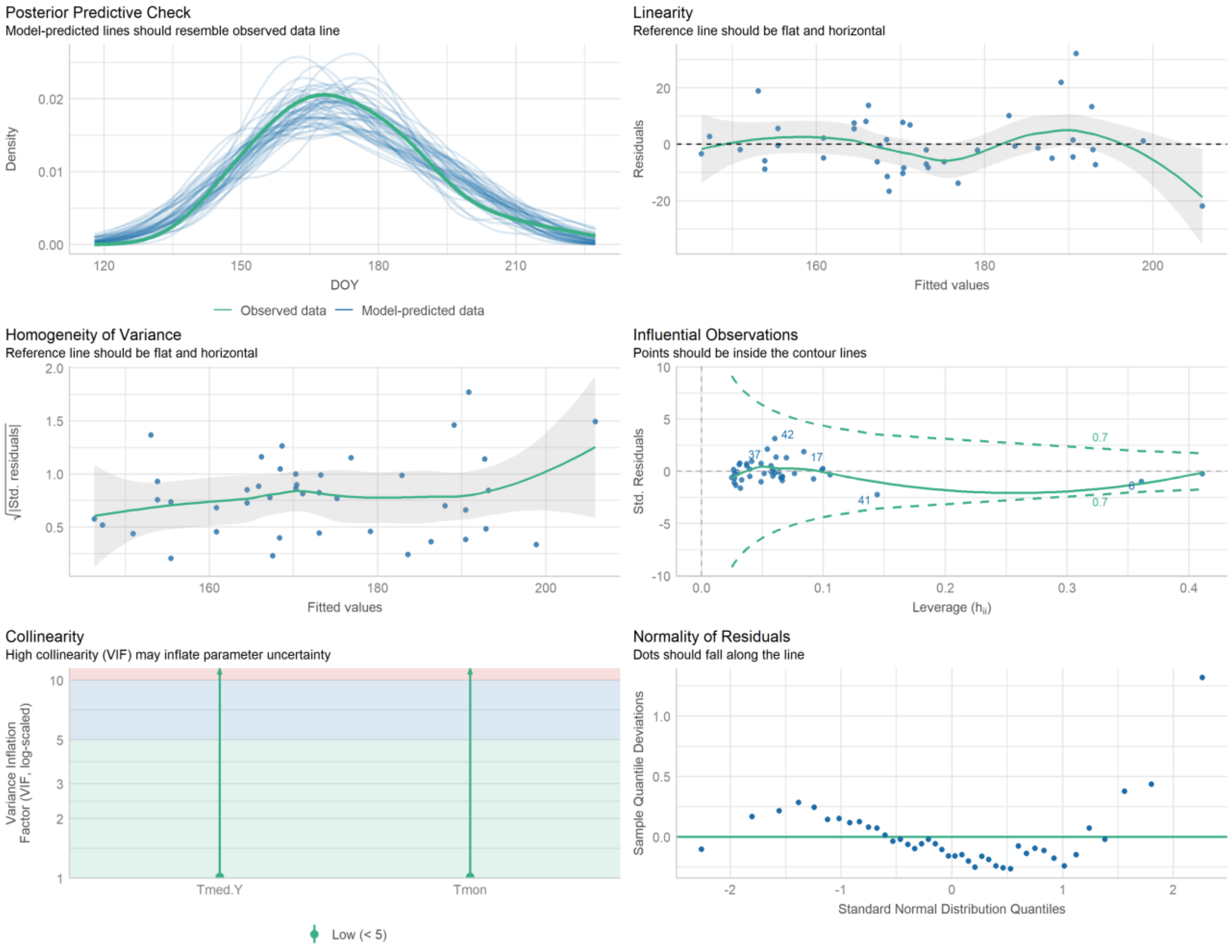

1.72. MLM - FBF - Macrochloa tenacissima

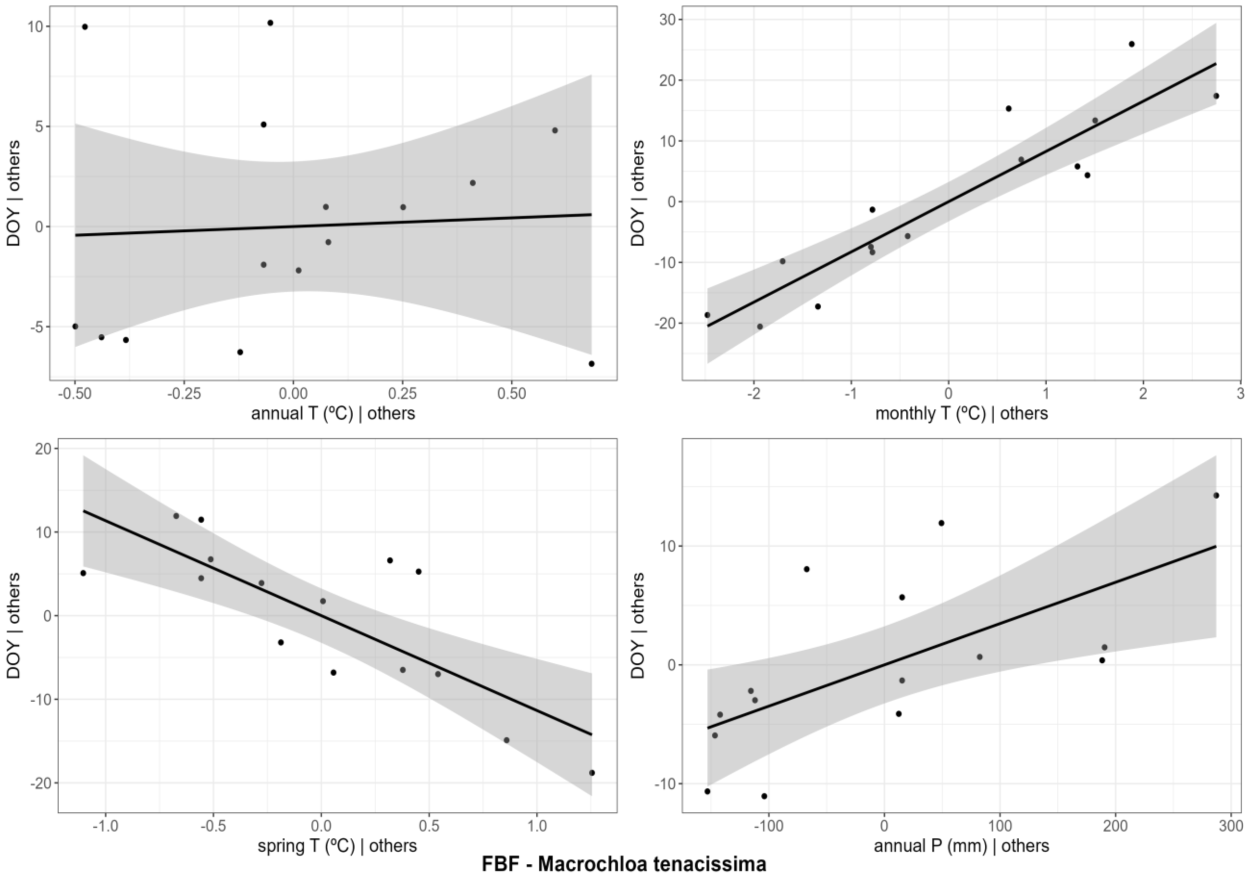

FBF - Macrochloa tenacissima

DOY = 110.43 (+0.87*annual T (ºC)) + (+8.27*monthly T (ºC)) + (-11.35*spring T (ºC)) + (+0.03*annual P (mm))

1.72.1. Diagnostics - MLM - FBF - Macrochloa tenacissima

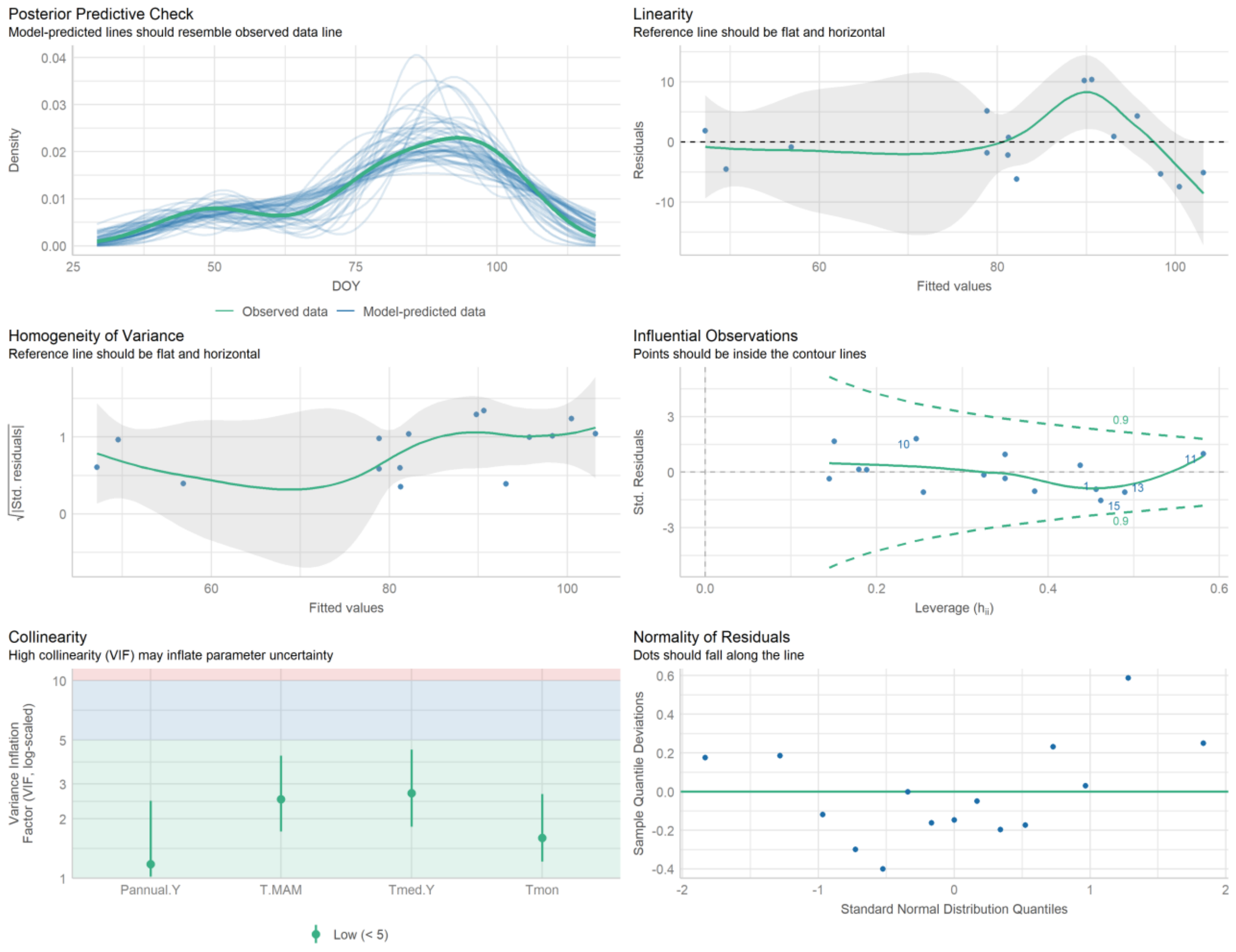

1.73. MLM - F - Macrochloa tenacissima

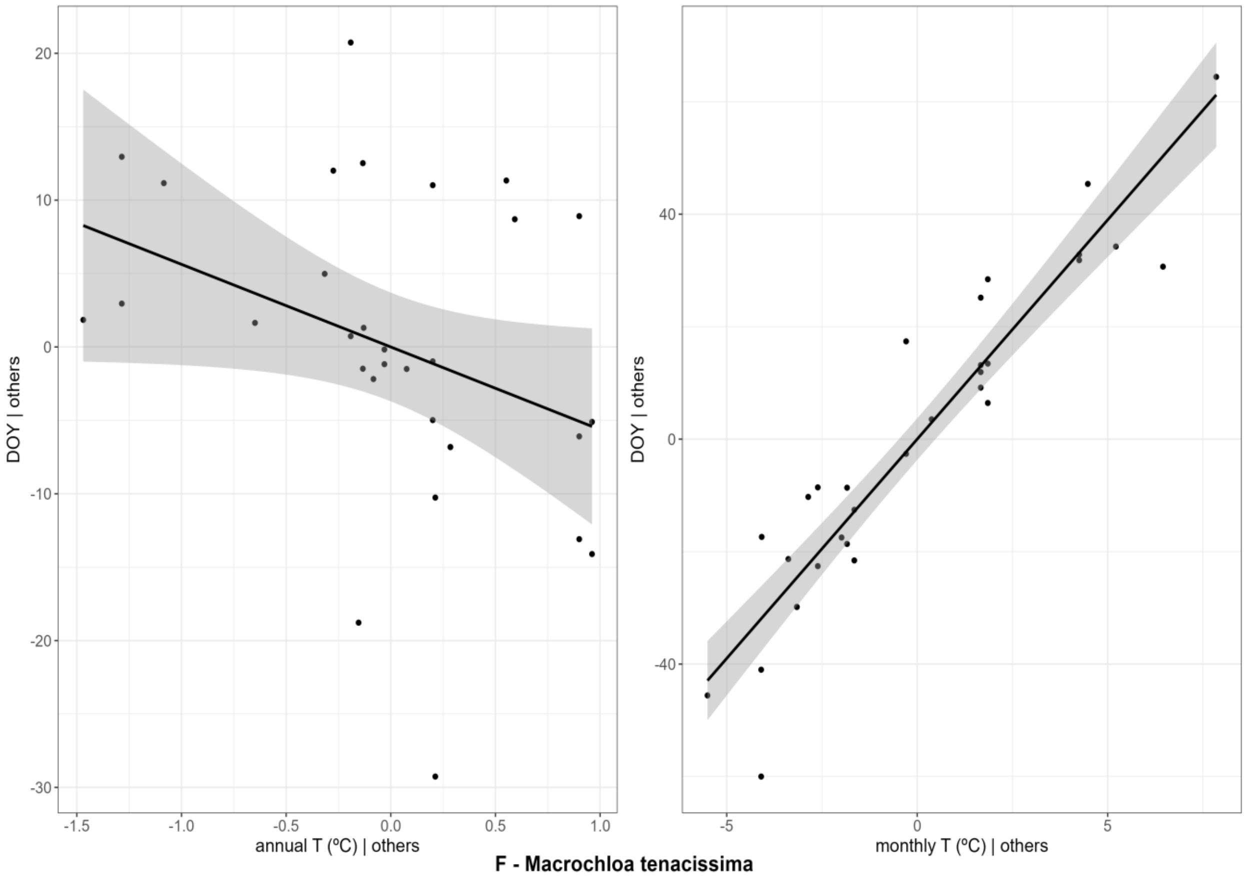

DOY = 83.61 (-5.63*annual T (ºC)) + (+7.80*monthly T (ºC))

1.73.1. Diagnostics - MLM - F - Macrochloa tenacissima

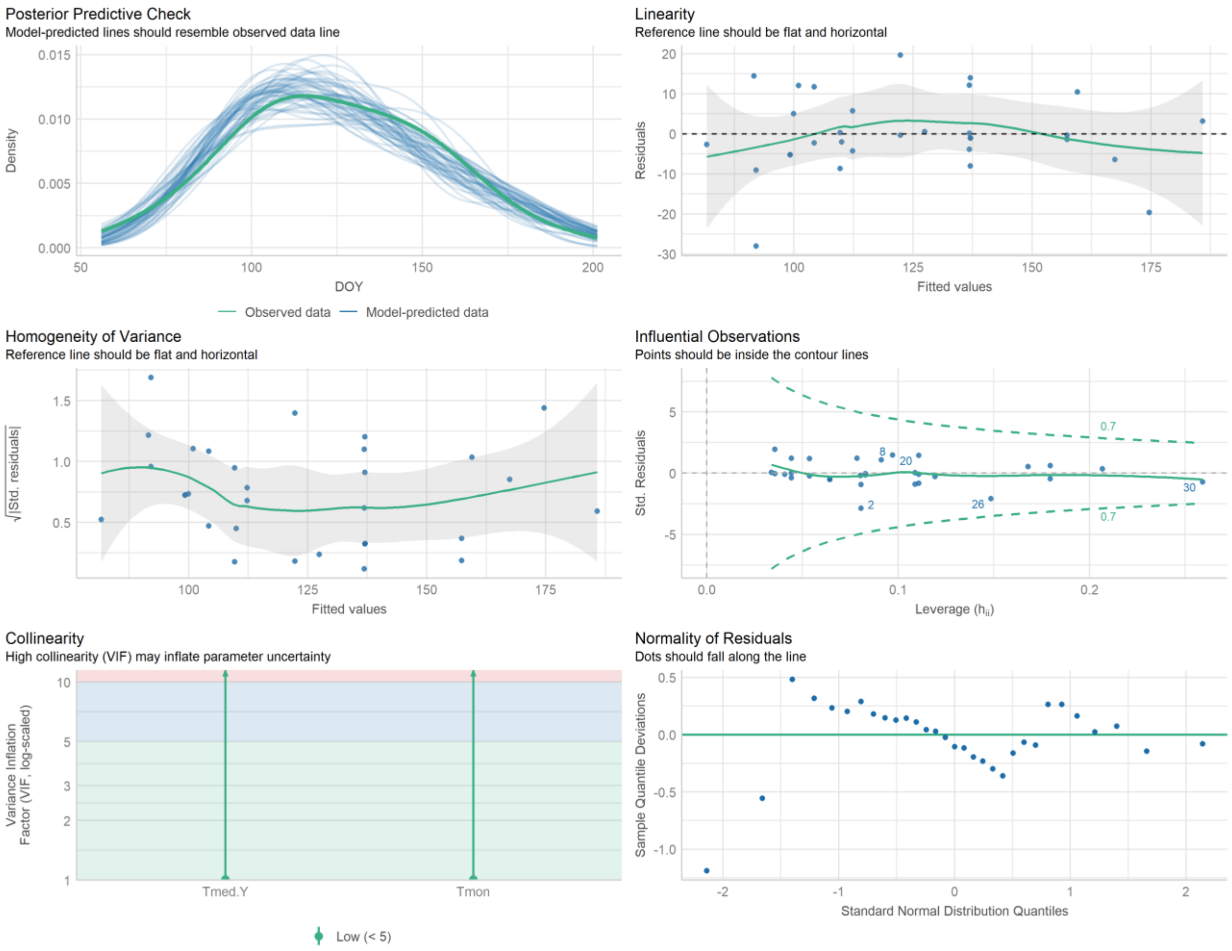

1.74. MLM - F - Myrtus communis

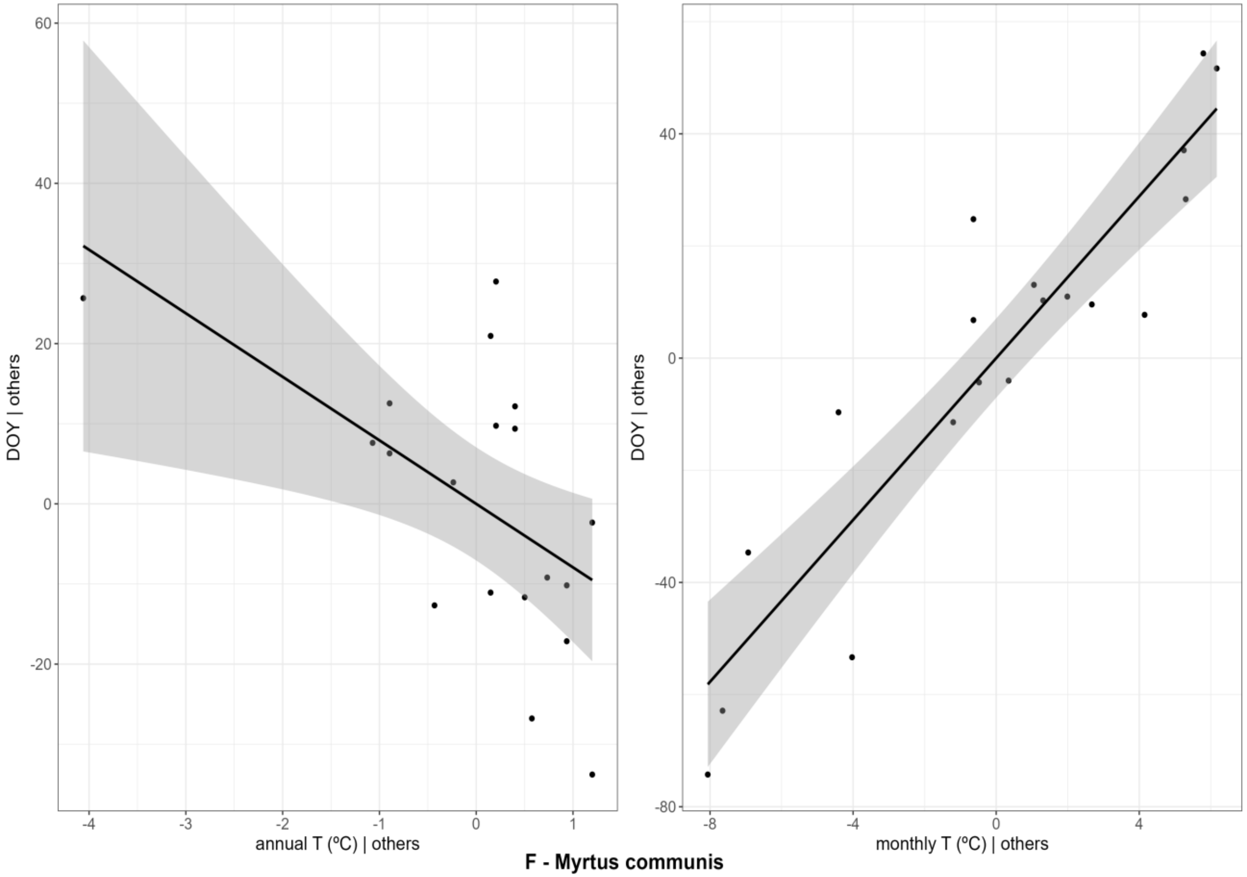

F - Myrtus communis

DOY = 142.26 (-7.93*annual T (ºC)) + (+7.21*monthly T (ºC))

1.74.1. Diagnostics - MLM - F - Myrtus communis

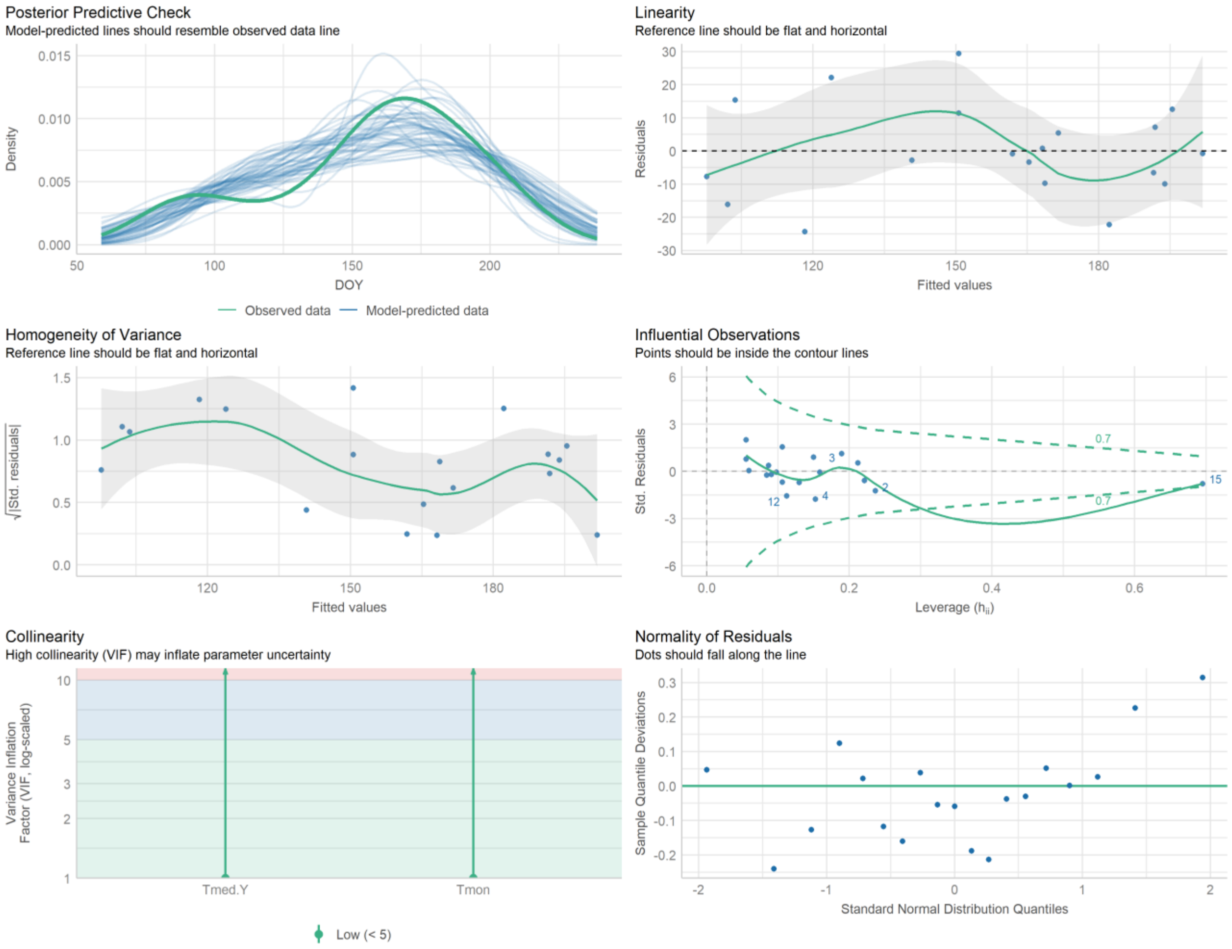

1.75. MLM - DVG - Myrtus communis

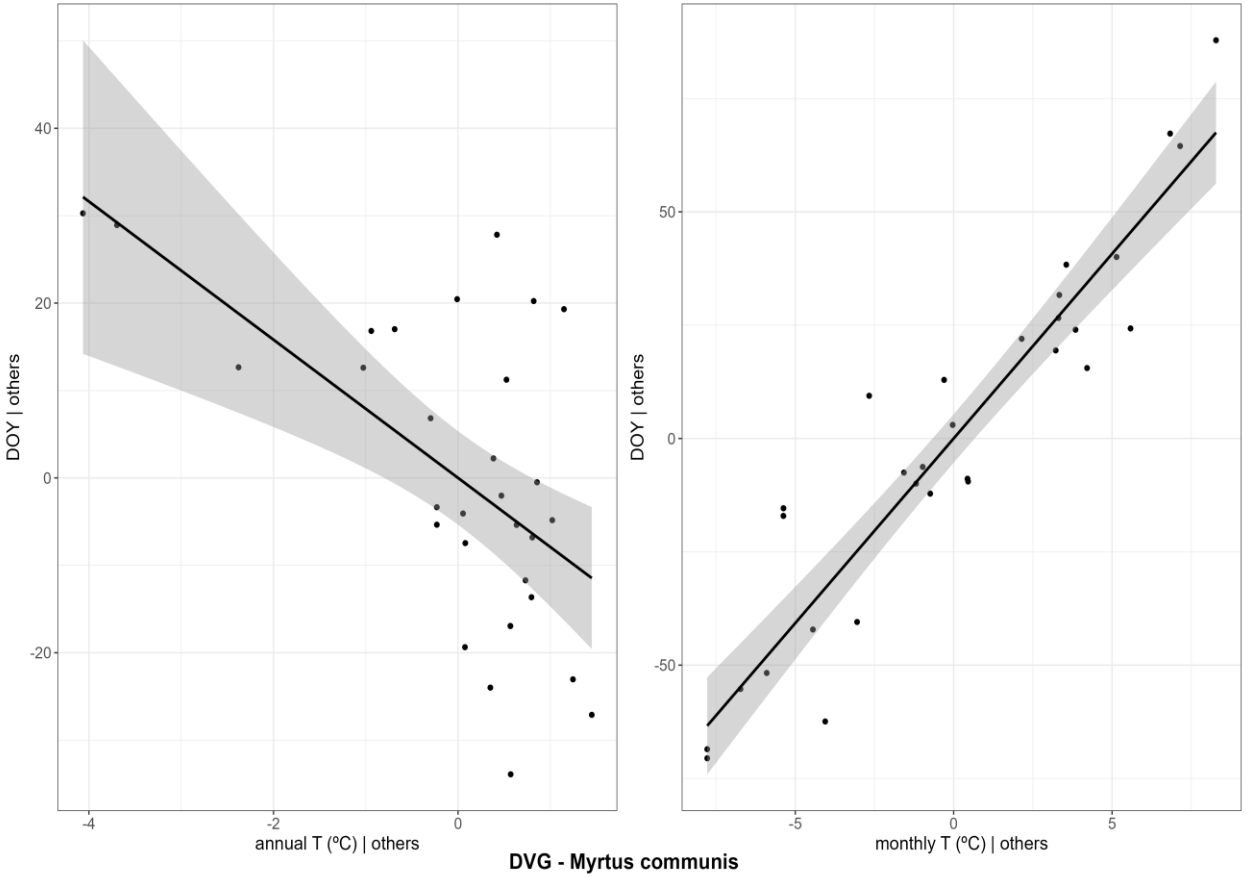

DOY = 118.19 (-7.91*annual T (ºC)) + (+8.15*monthly T (ºC))

1.75.1. Diagnostics - MLM - DVG - Myrtus communis

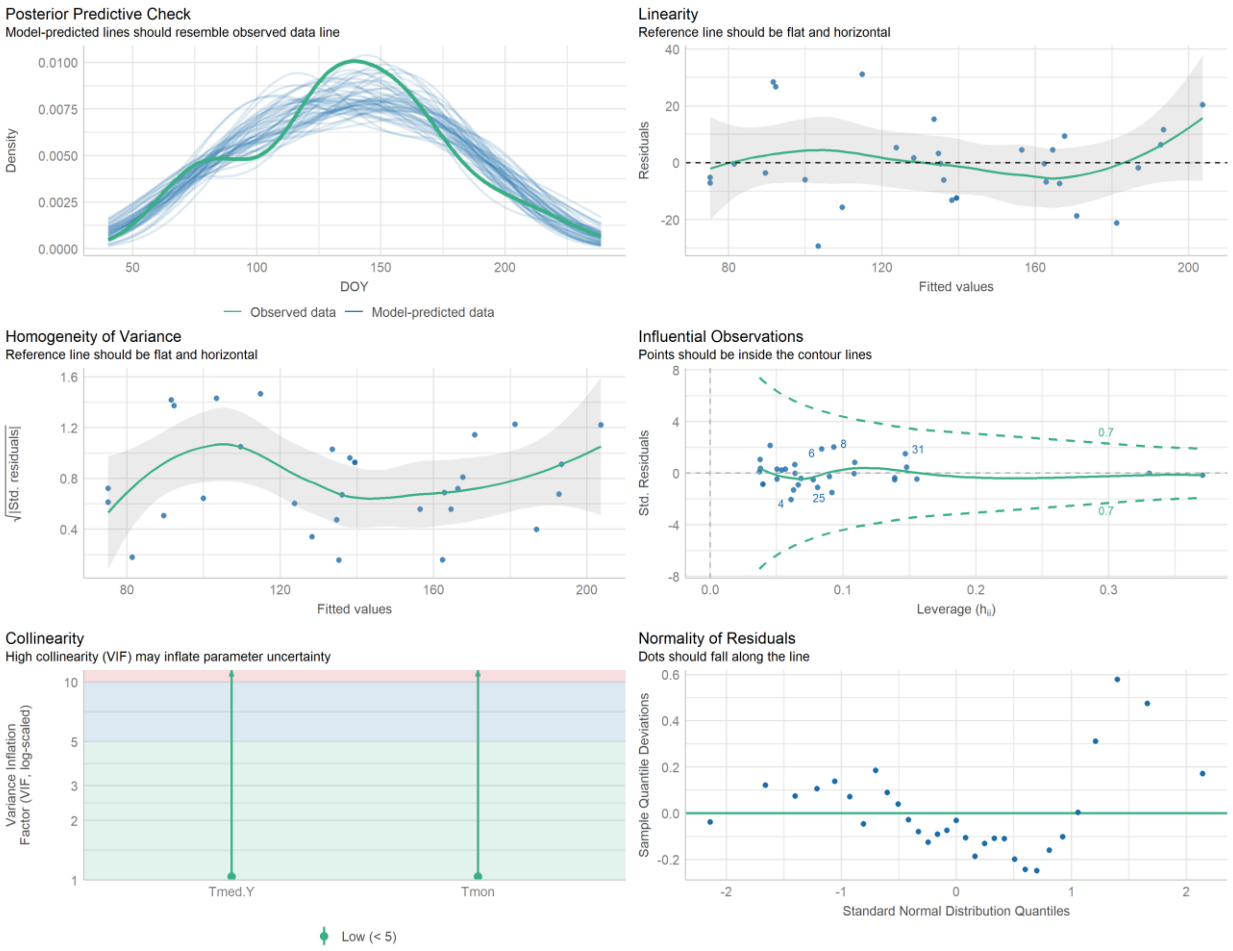

1.76. MLM - F - Nevadensia purpurea

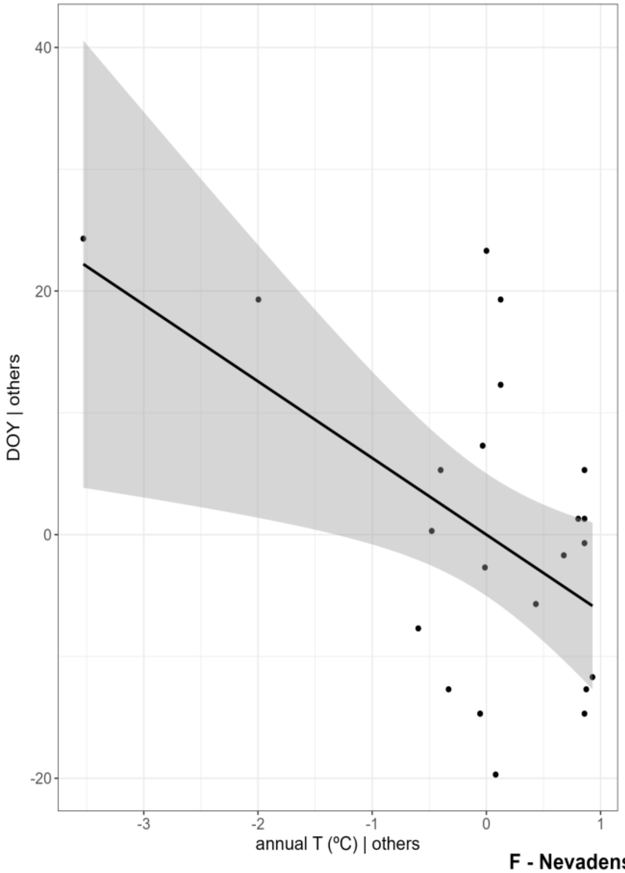

F - Nevadensia purpurea

DOY = 298.15 (-6.29*annual T (ºC))

1.76.1. Diagnostics - MLM - F - Nevadensia purpurea

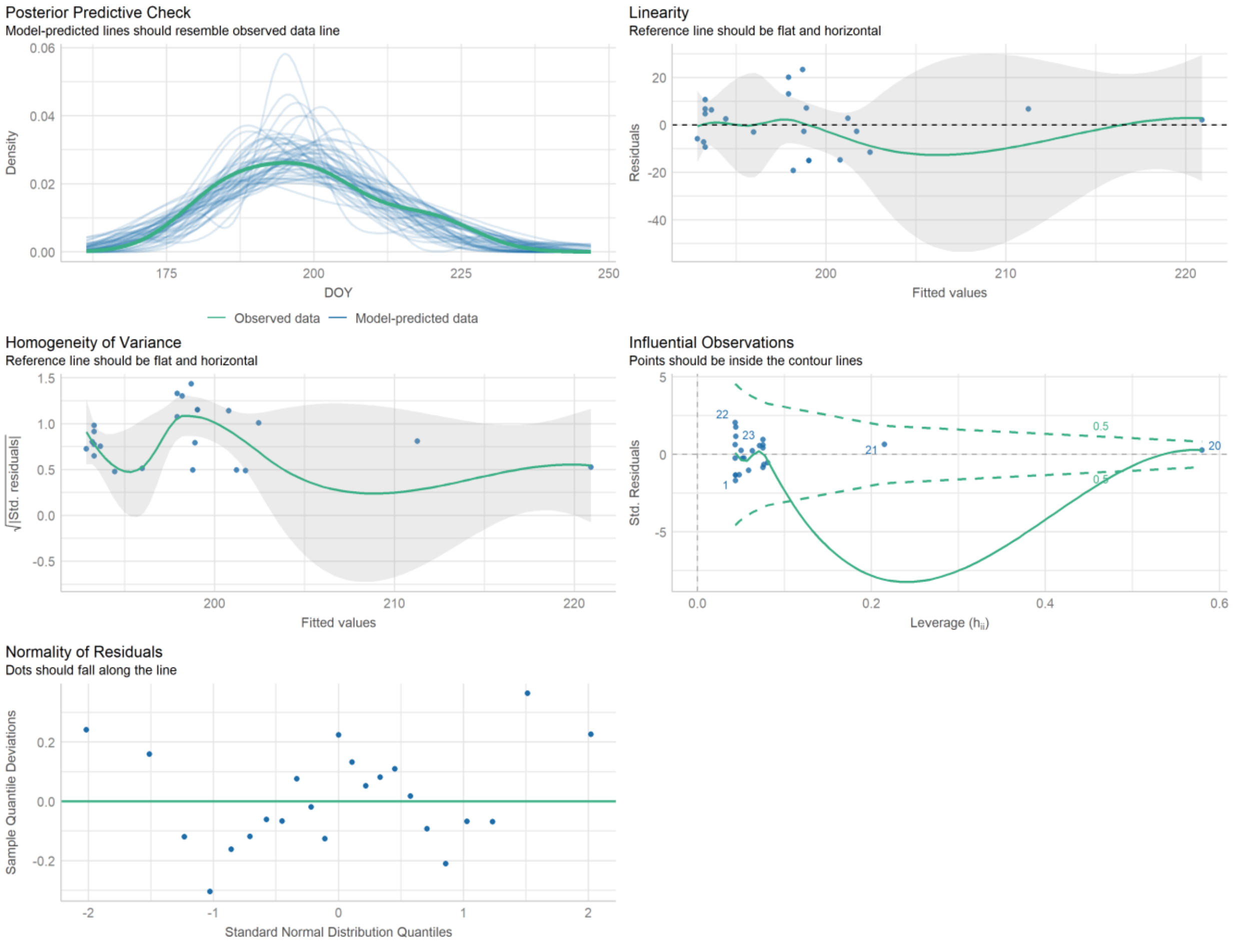

1.77. MLM - FS - Nevadensia purpurea

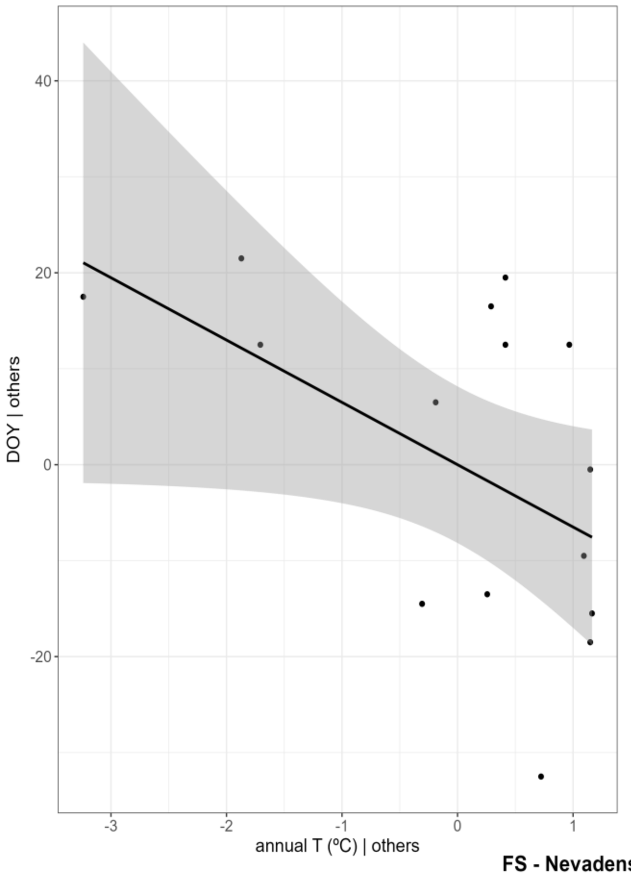

FS - Nevadensia purpurea

DOY = 306.26 (-6.49*annual T (ºC))

1.77.1. Diagnostics - MLM - FS - Nevadensia purpurea

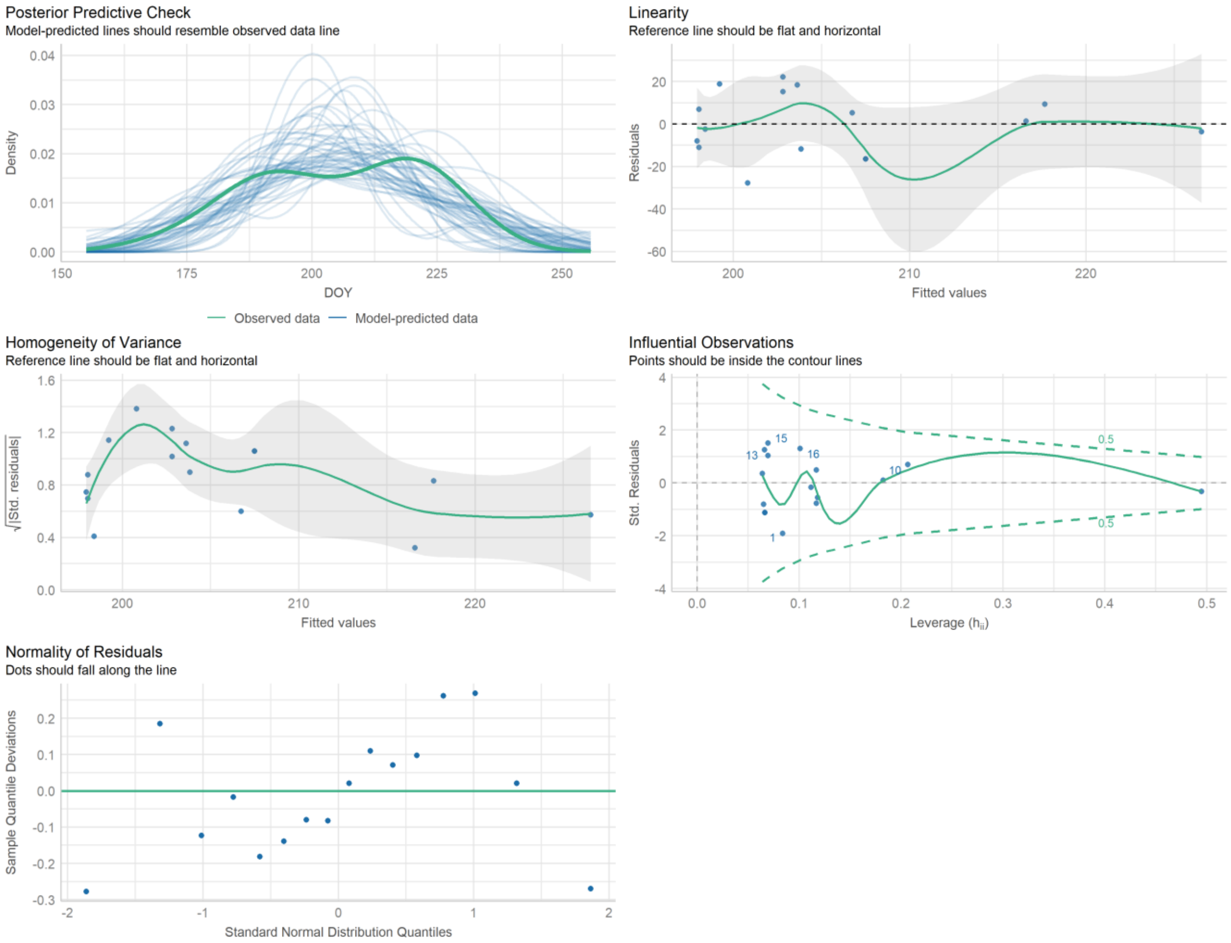

1.78. MLM - FBF - Olea europaea var. sylvestris

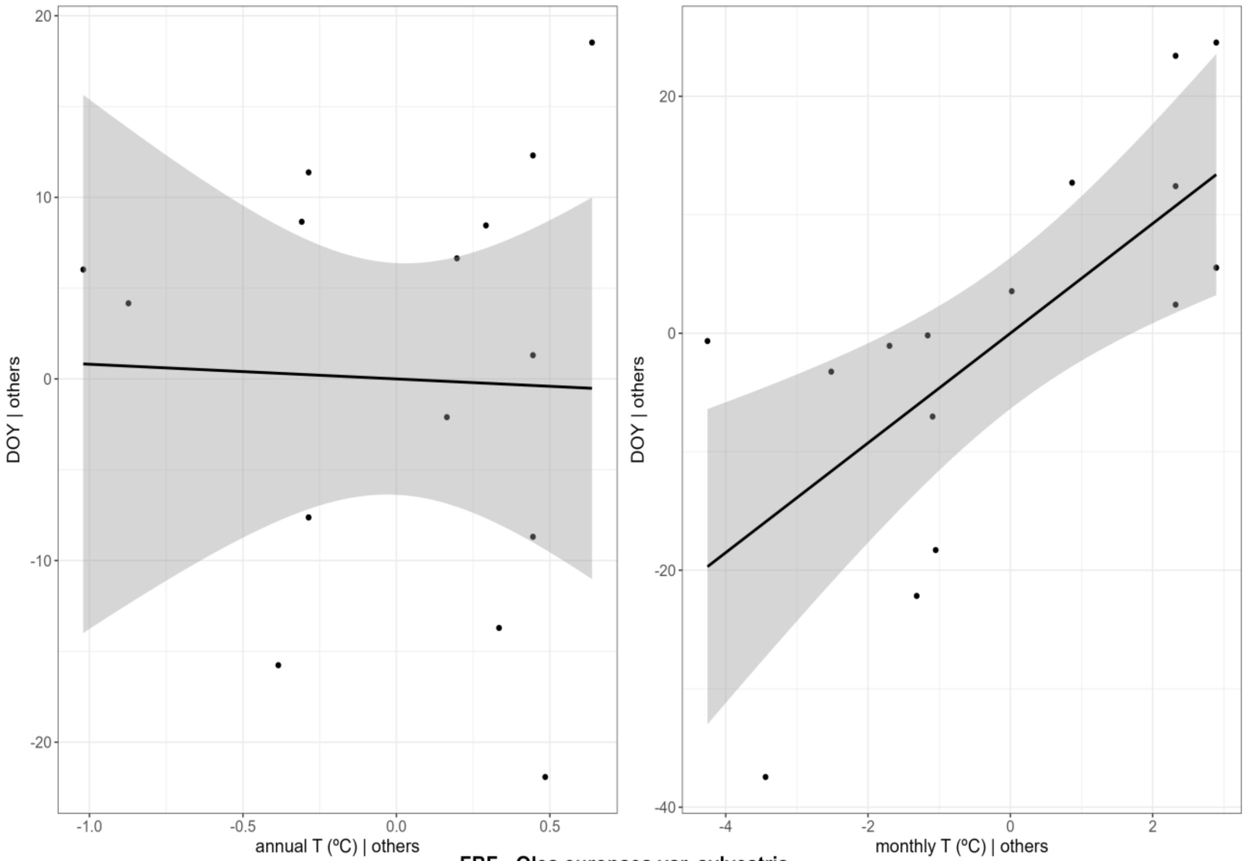

FBF - Olea europaea var. sylvestris

DOY = 56.81 (-0.81*annual T (ºC)) + (+4.63*monthly T (ºC))

1.78.1. Diagnostics - MLM - FBF - Olea europaea var. sylvestris

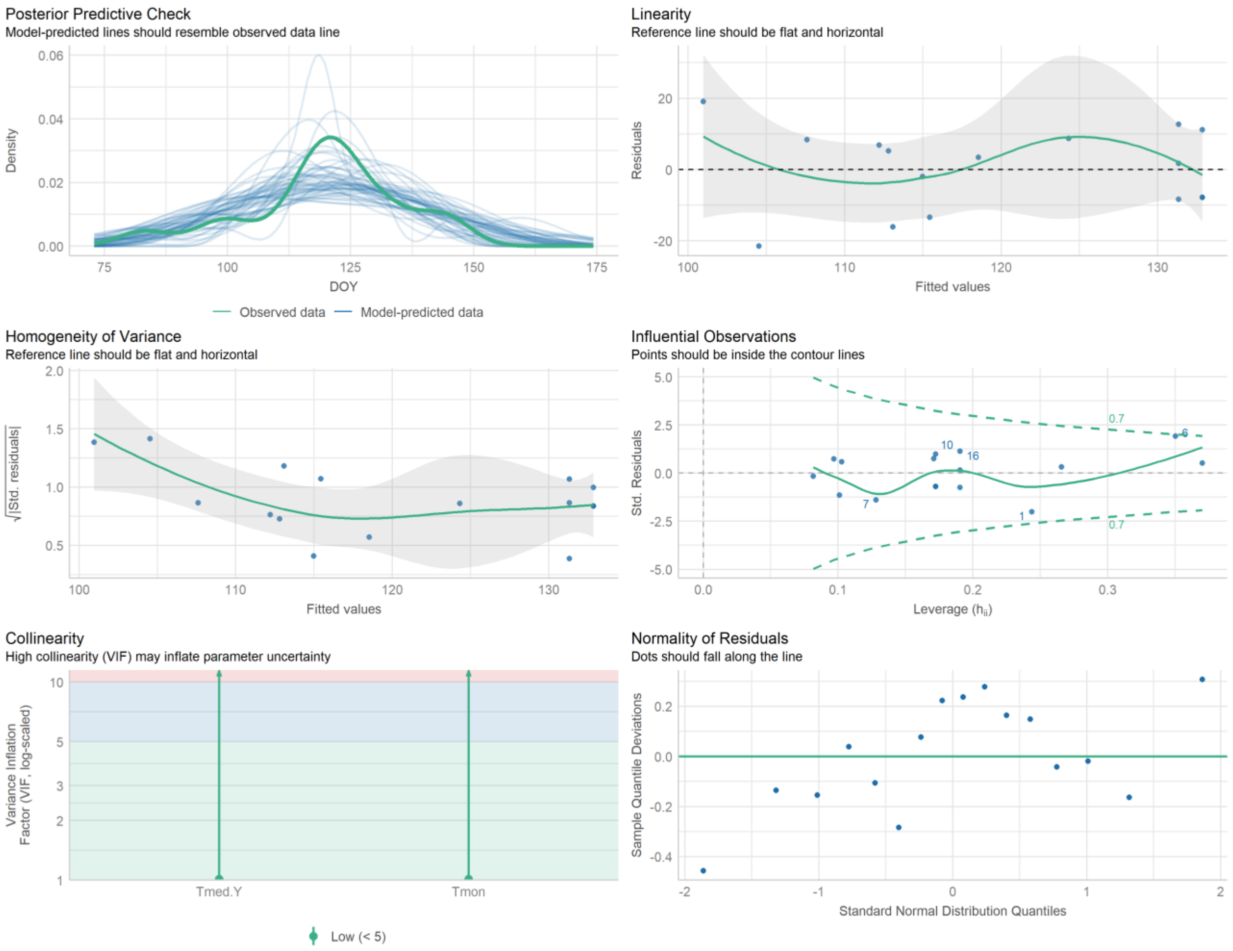

1.79. MLM - DVG - Olea europaea var. sylvestris

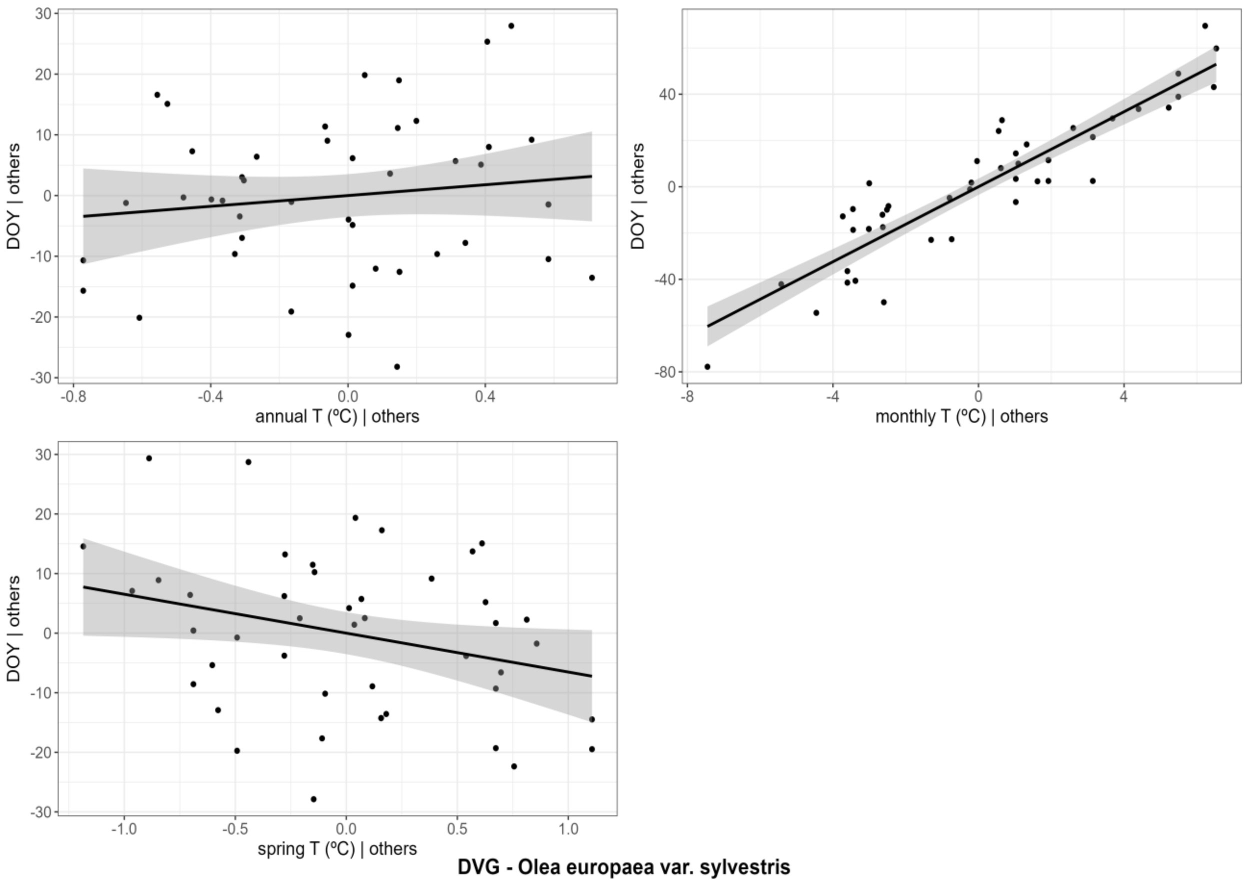

DVG - Olea europaea var. sylvestris

DOY = 11.22 (+4.44*annual T (ºC)) + (+8.10*monthly T (ºC)) + (-6.53*spring T (ºC))

1.79.1. Diagnostics - MLM - DVG - Olea europaea var. sylvestris

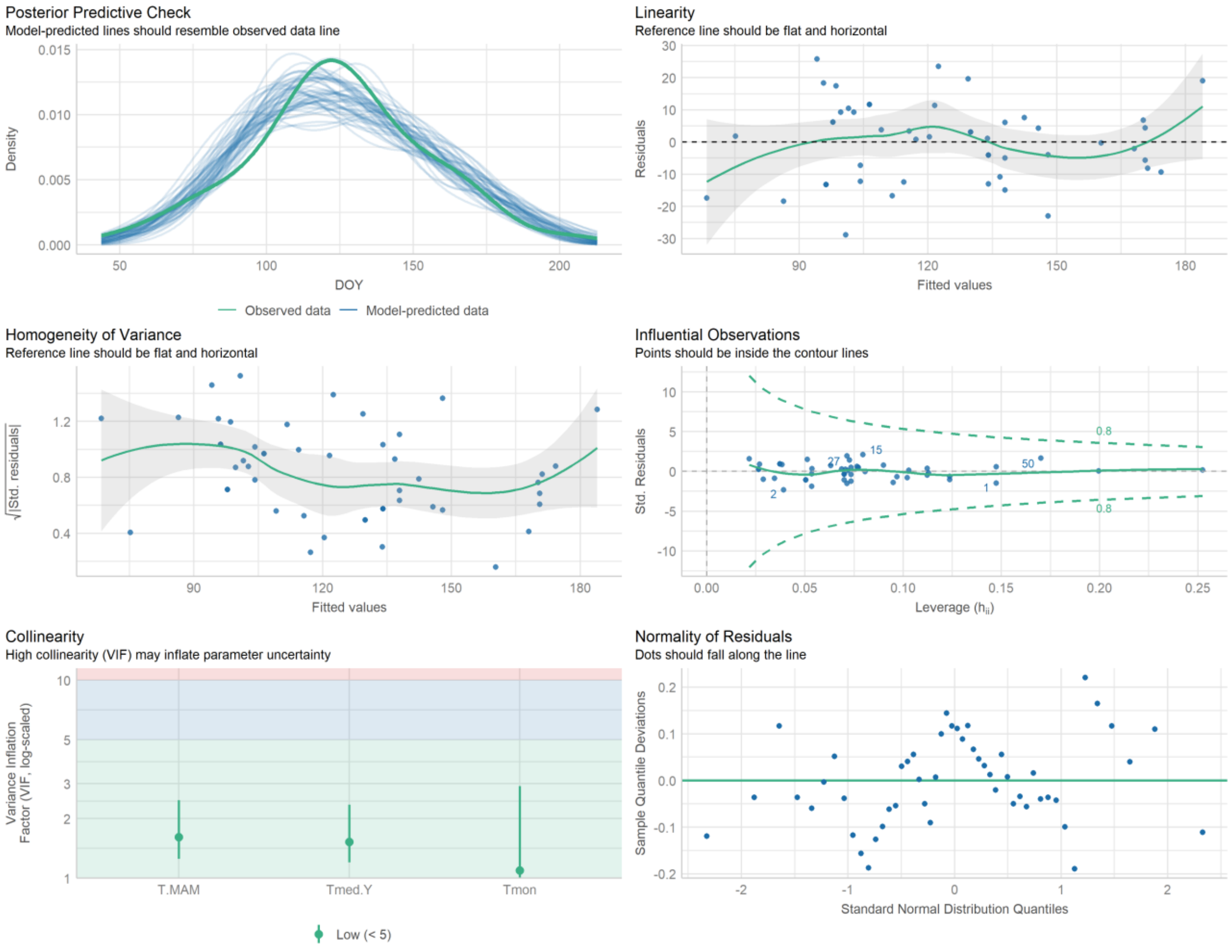

1.80. MLM - FBF - Phillyrea angustifolia

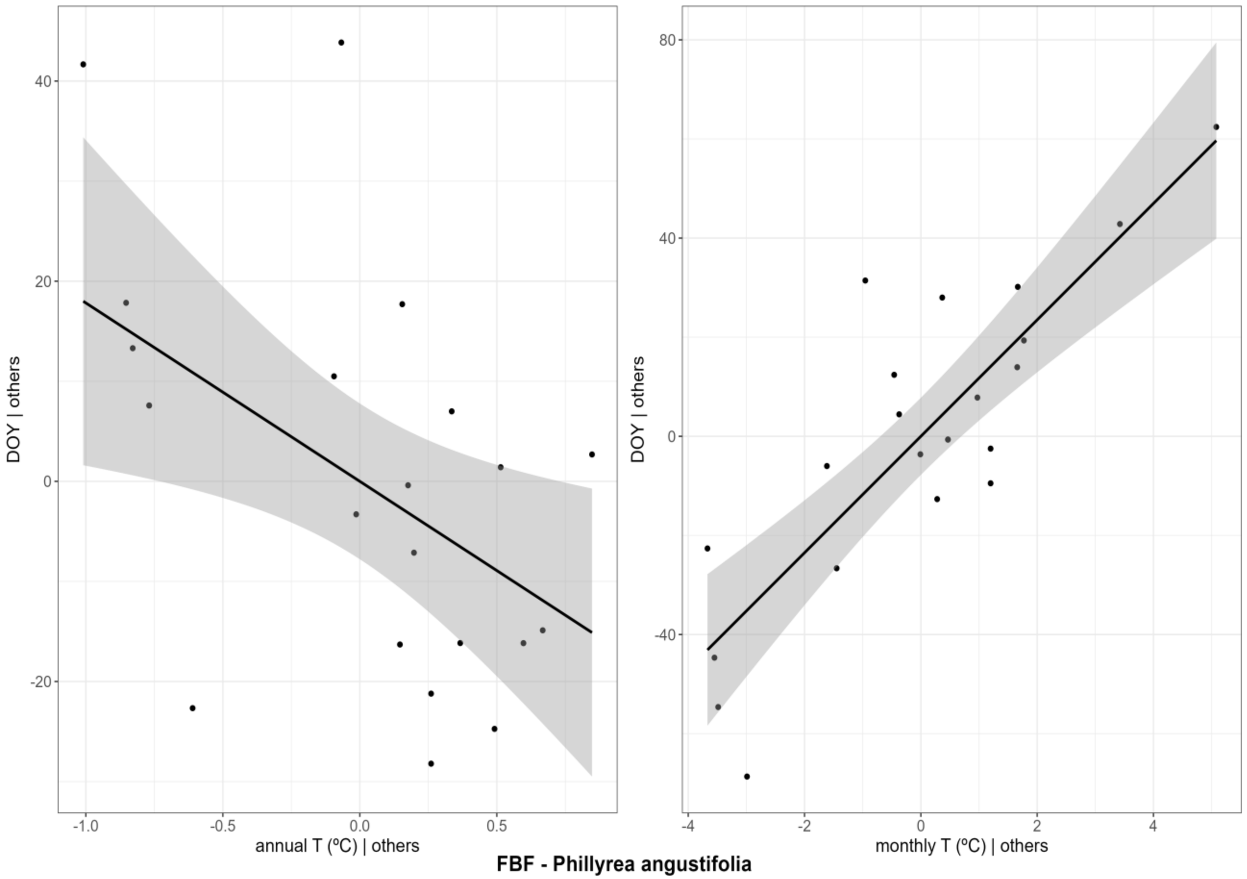

DOY = 224.55 (-17.83*annual T (ºC)) + (+11.75*monthly T (ºC))

1.80.1. Diagnostics - MLM - FBF - Phillyrea angustifolia

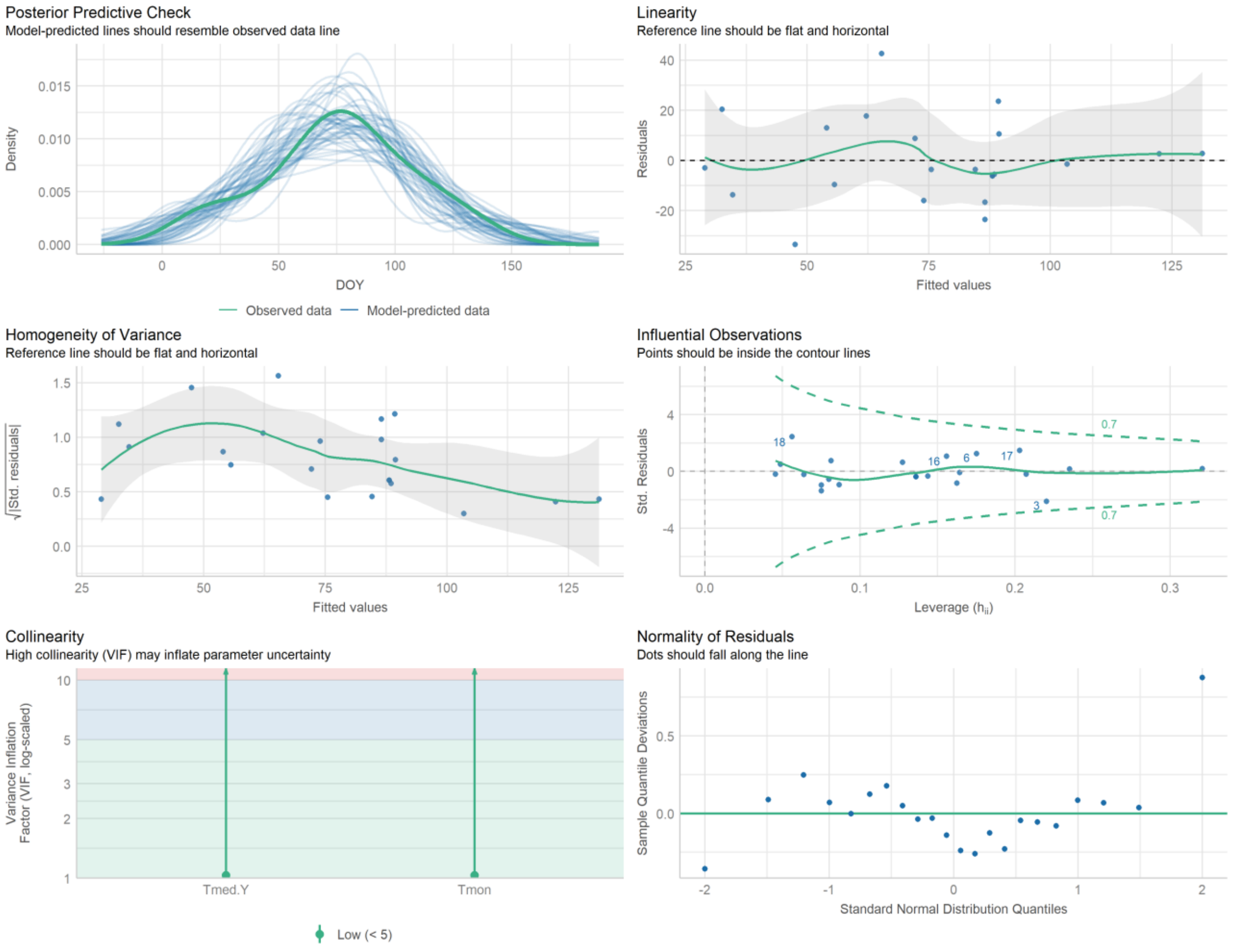

1.81. MLM - F - Phillyrea angustifolia

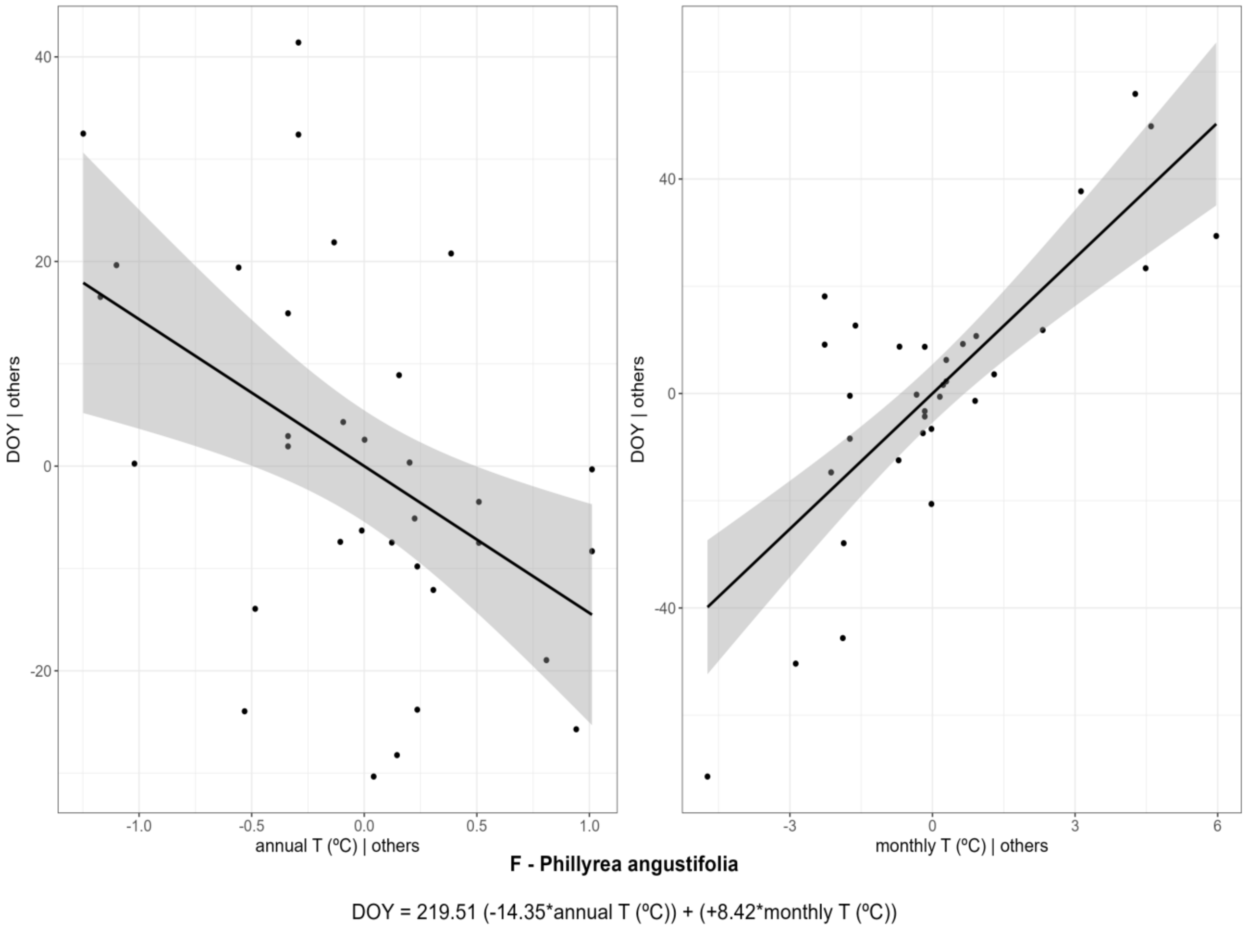

1.81.1. Diagnostics - MLM - F - Phillyrea angustifolia

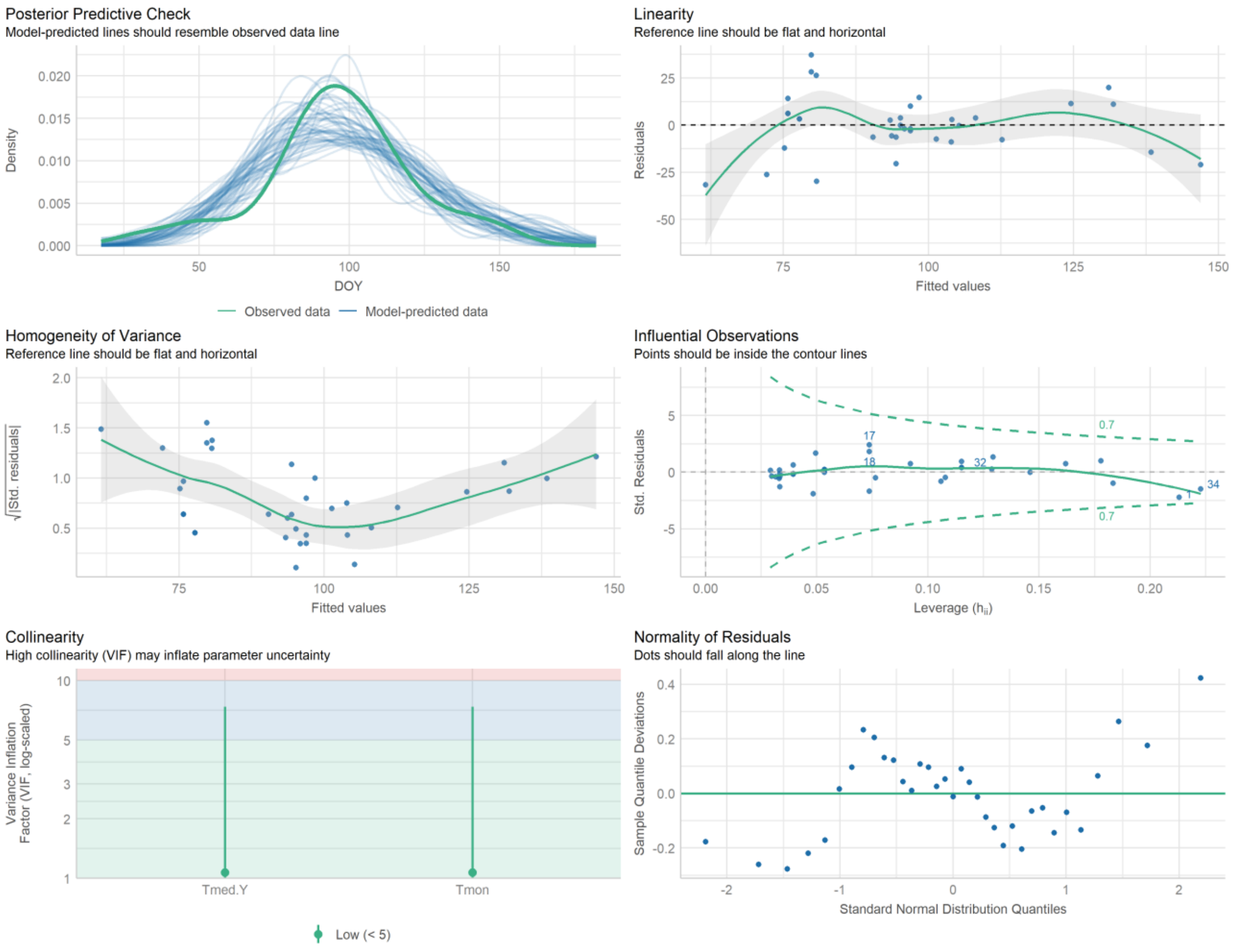

1.82. MLM - DVG - Phillyrea angustifolia

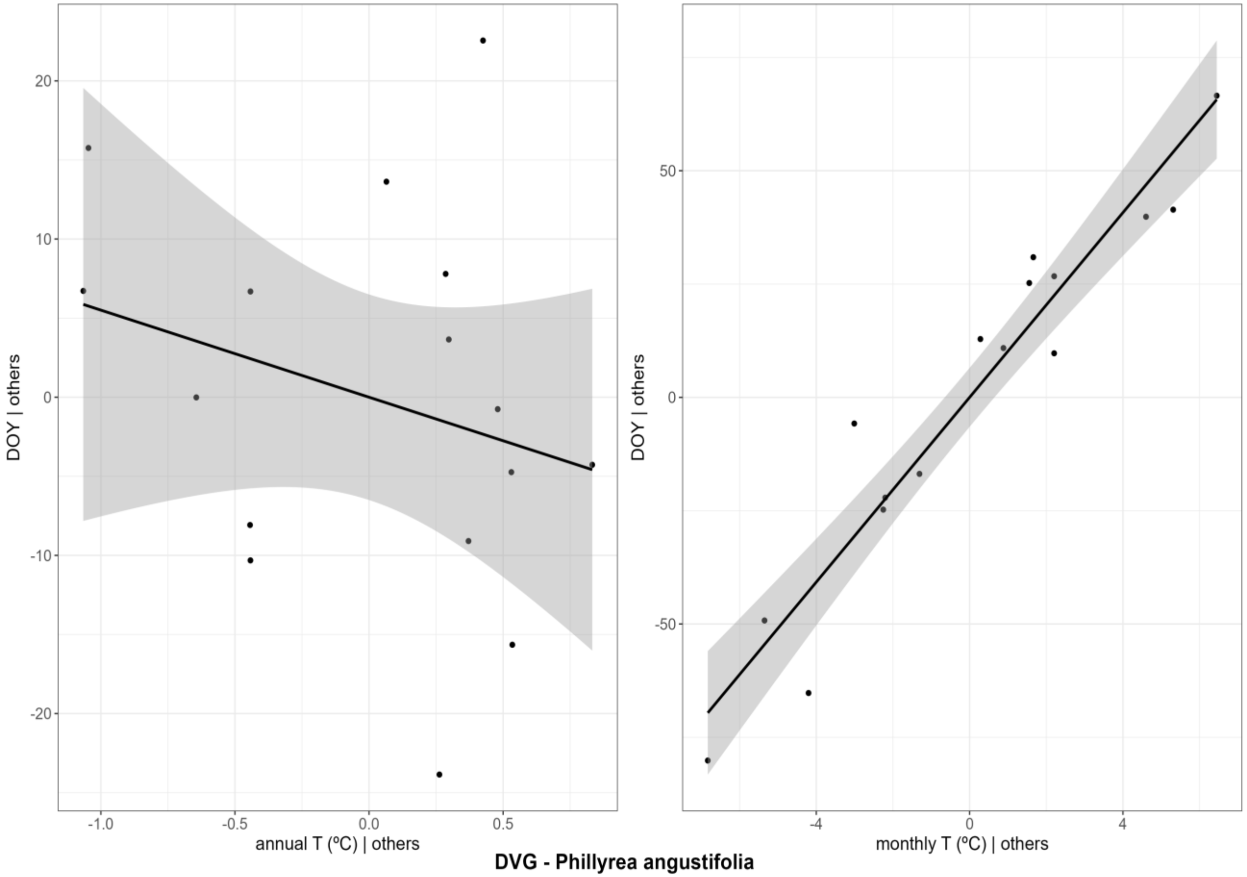

DOY = 44.07 (-5.50*annual T (ºC)) + (+10.19*monthly T (ºC))

1.82.1. Diagnostics - MLM - DVG - Phillyrea angustifolia

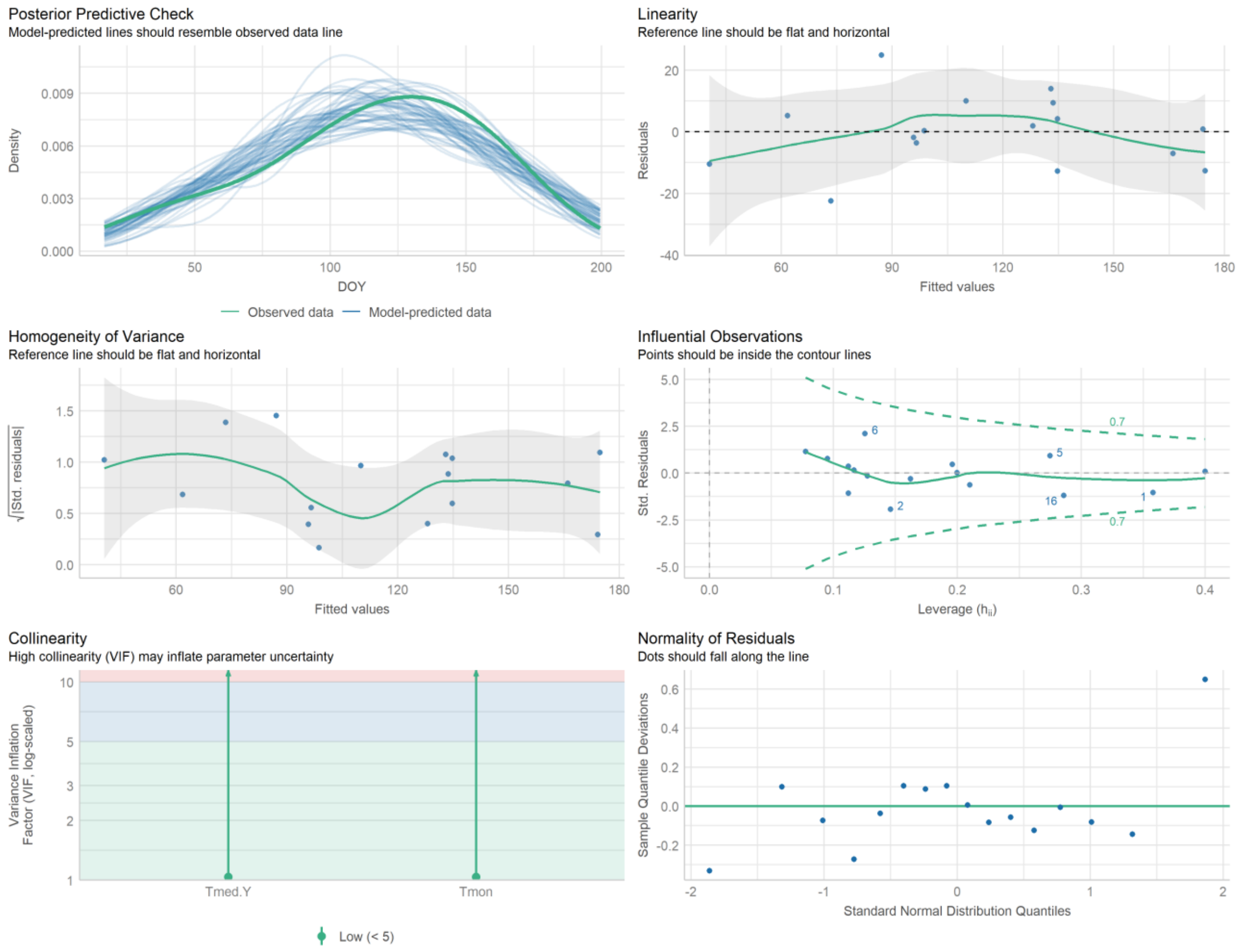

1.83. MLM - FBF - Phlomis crinita subsp. malacitana

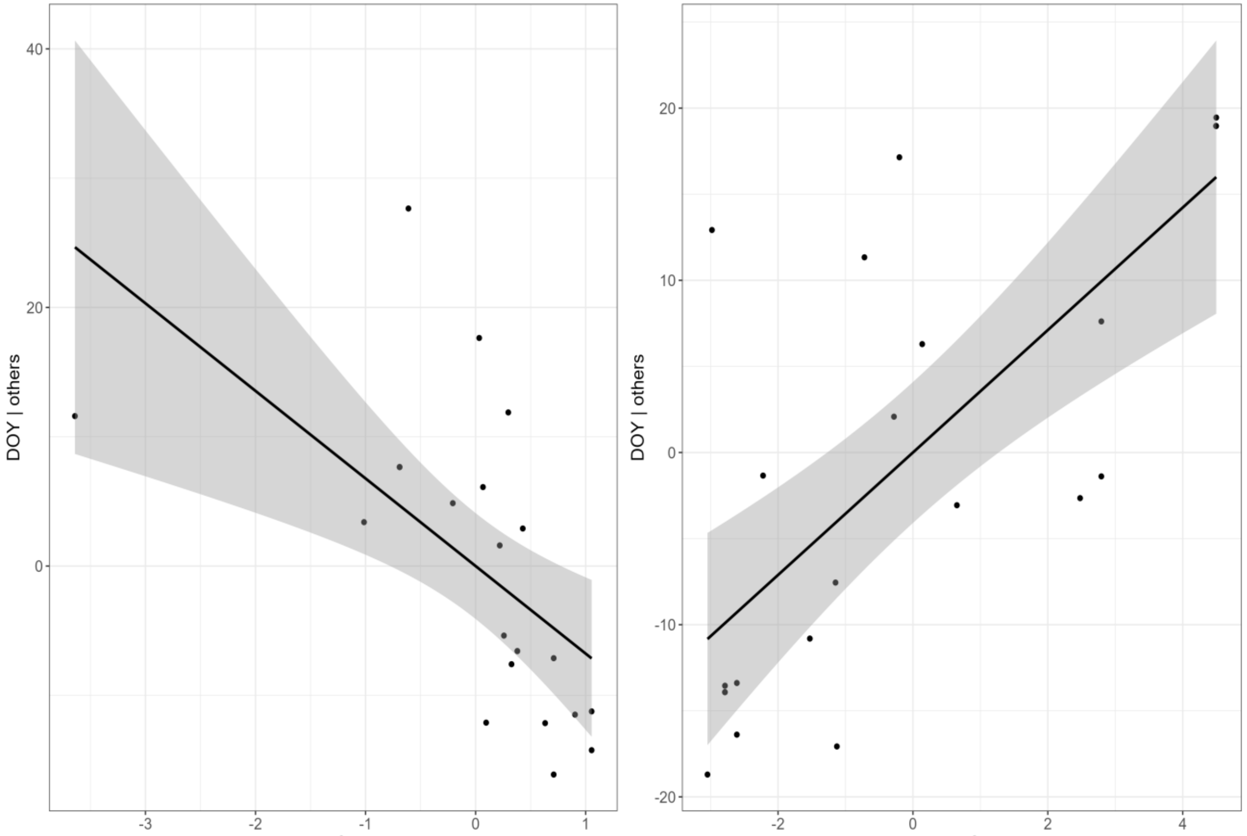

FBF - Phlomis crinita subsp. malacitana

DOY = 200.10 (-6.77*annual T (ºC)) + (+3.56*monthly T (ºC))

1.83.1. Diagnostics - MLM - FBF - Phlomis crinita subsp. malacitana

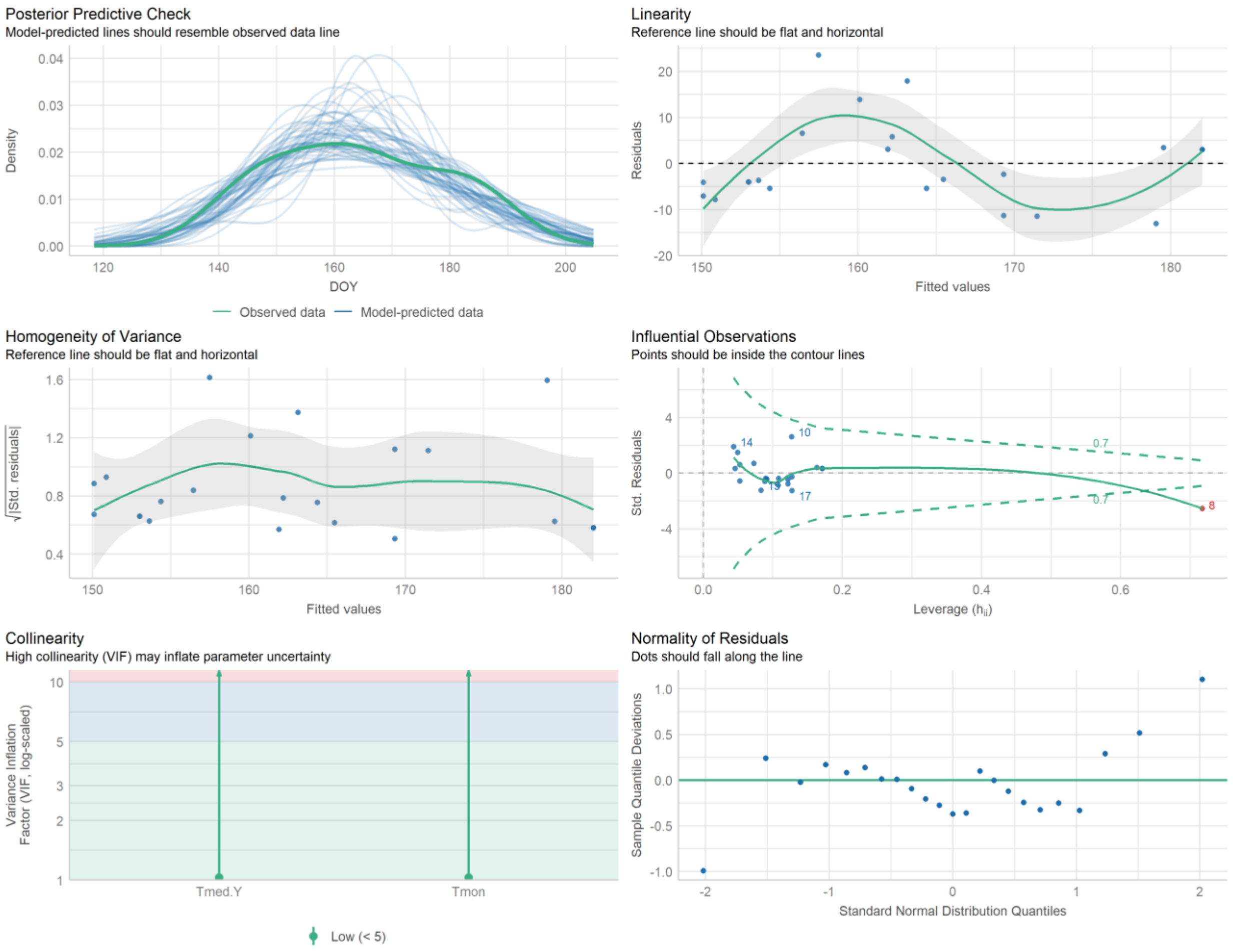

1.84. MLM - F - Phlomis crinita subsp. malacitana

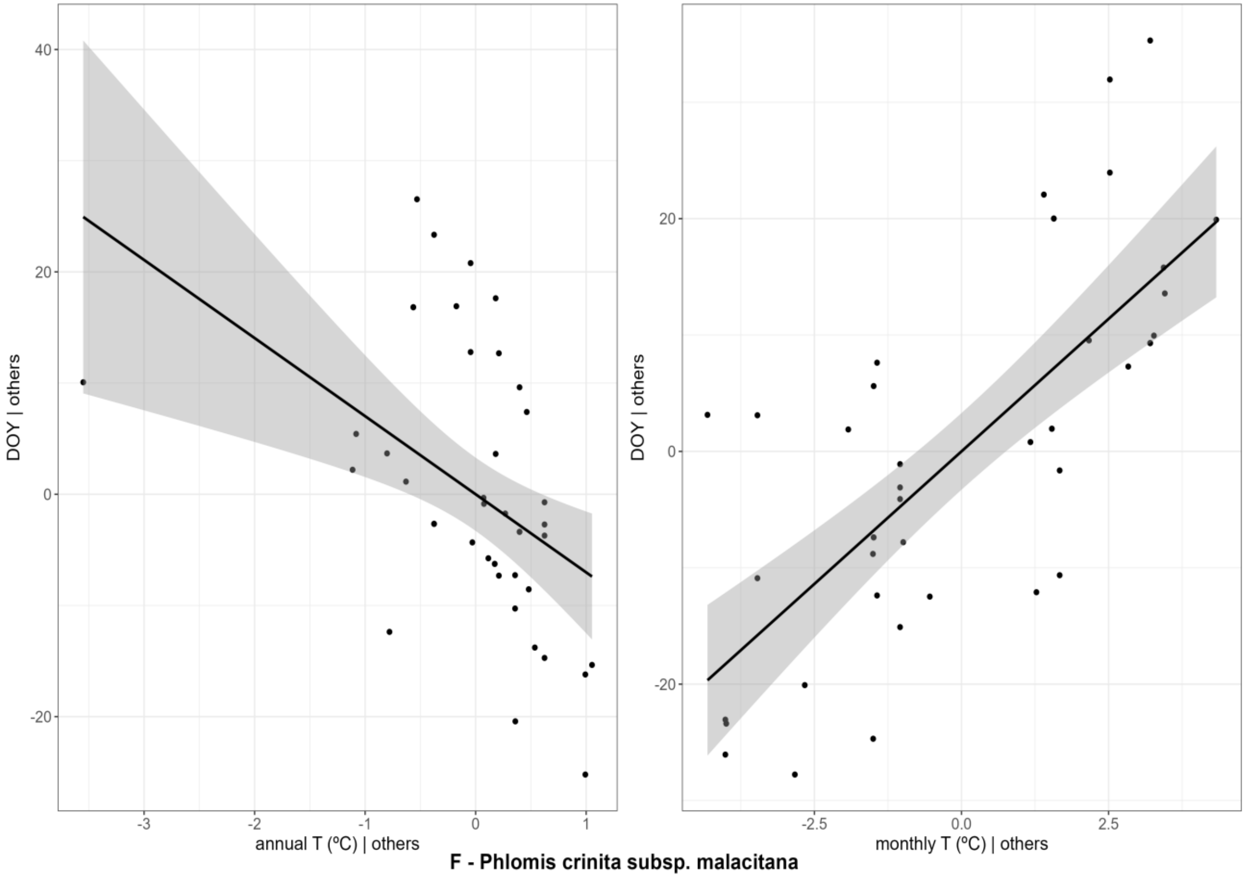

DOY = 186.49 (-7.02*annual T (ºC)) + (+4.55*monthly T (ºC))

1.84.1. Diagnostics - MLM - F - Phlomis crinita subsp. malacitana

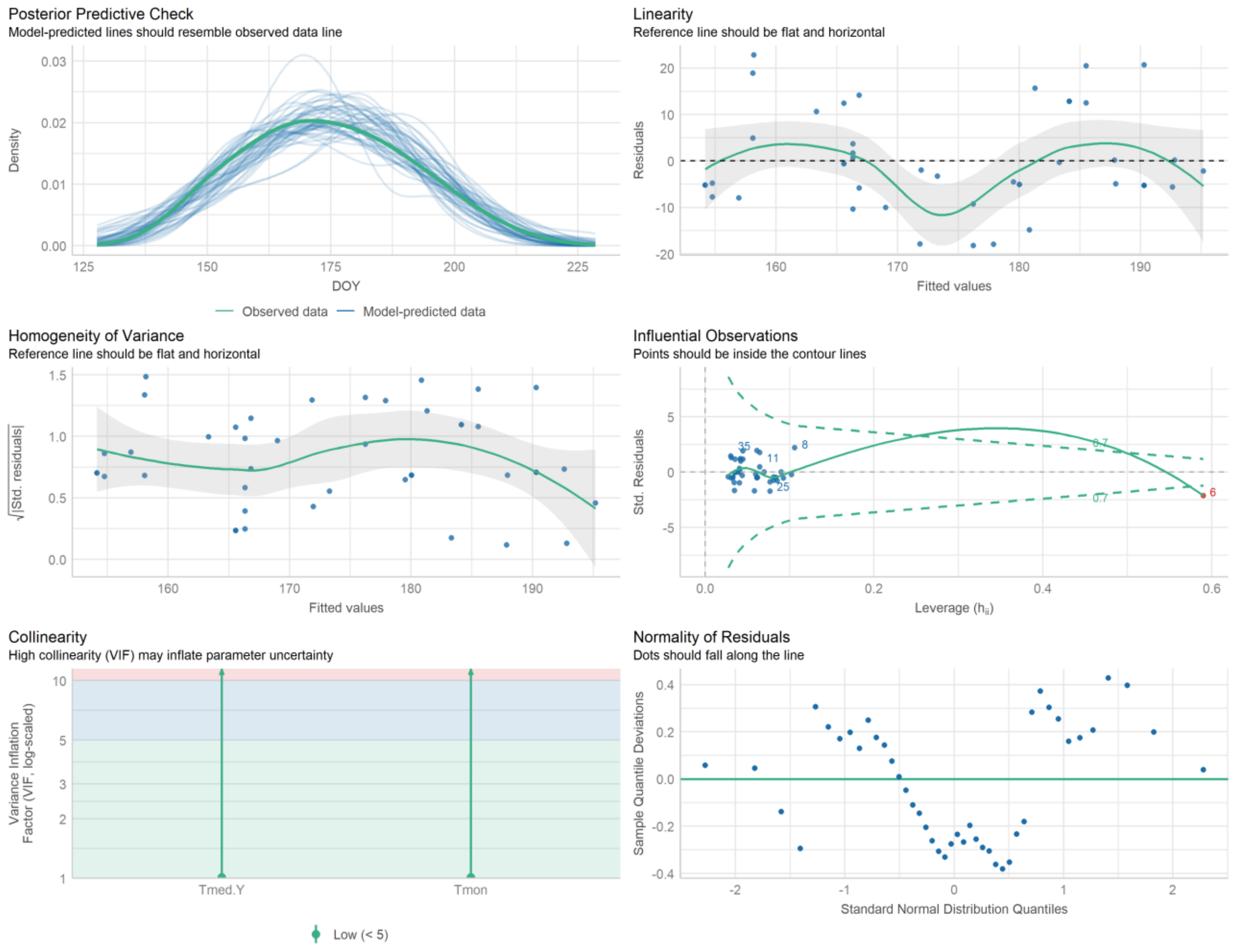

1.85. MLM - FBF - Phlomis purpurea

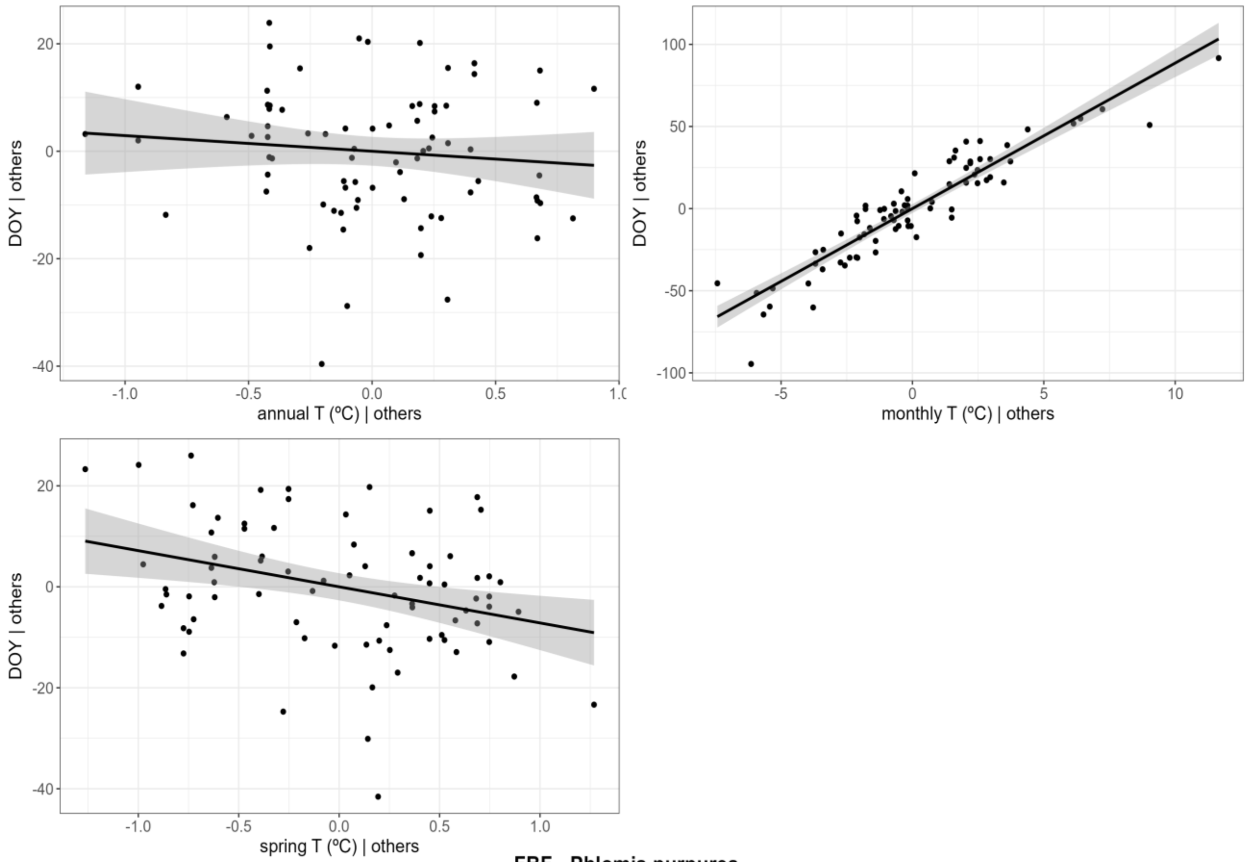

FBF - Phlomis purpurea

DOY = 127.68 (-2.91*annual T (ºC)) + (+8.86*monthly T (ºC)) + (-7.16*spring T (ºC))

1.85.1. Diagnostics - MLM - FBF - Phlomis purpurea

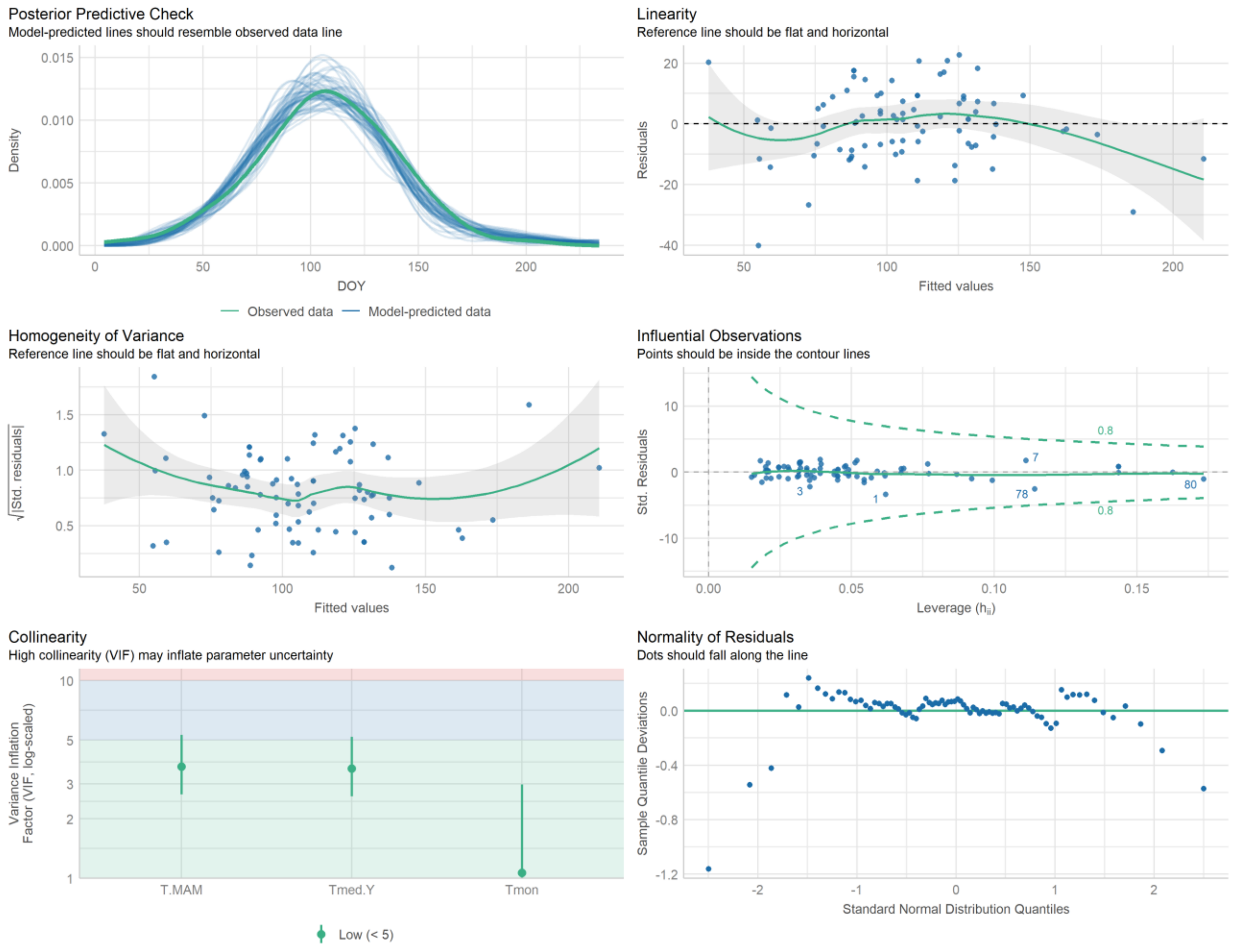

1.86. MLM - F - Phlomis purpurea

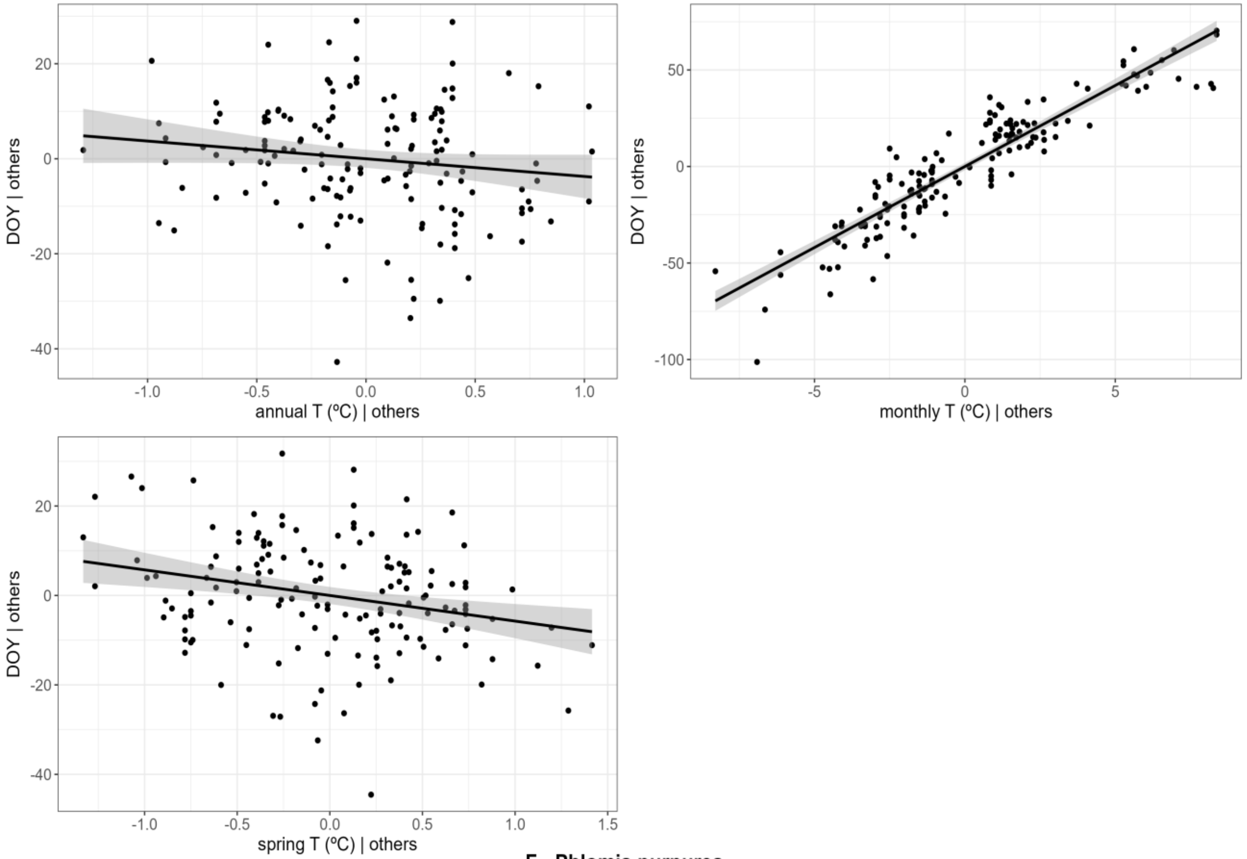

F - Phlomis purpurea

DOY = 127.69 (-3.73*annual T (ºC)) + (+8.39*monthly T (ºC)) + (-5.73*spring T (ºC))

1.86.1. Diagnostics - MLM - F - Phlomis purpurea

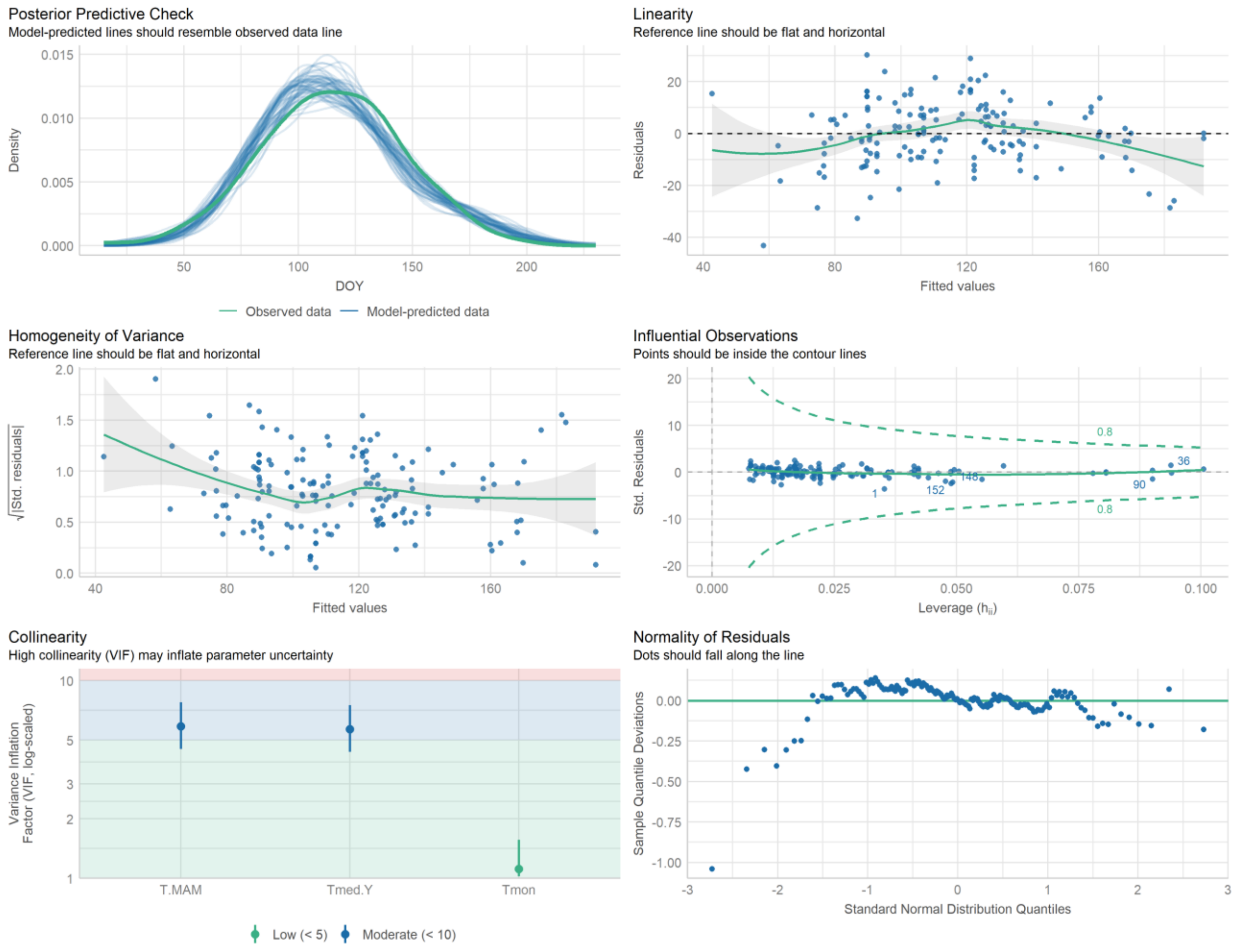

1.87. MLM - DVG - Phlomis purpurea

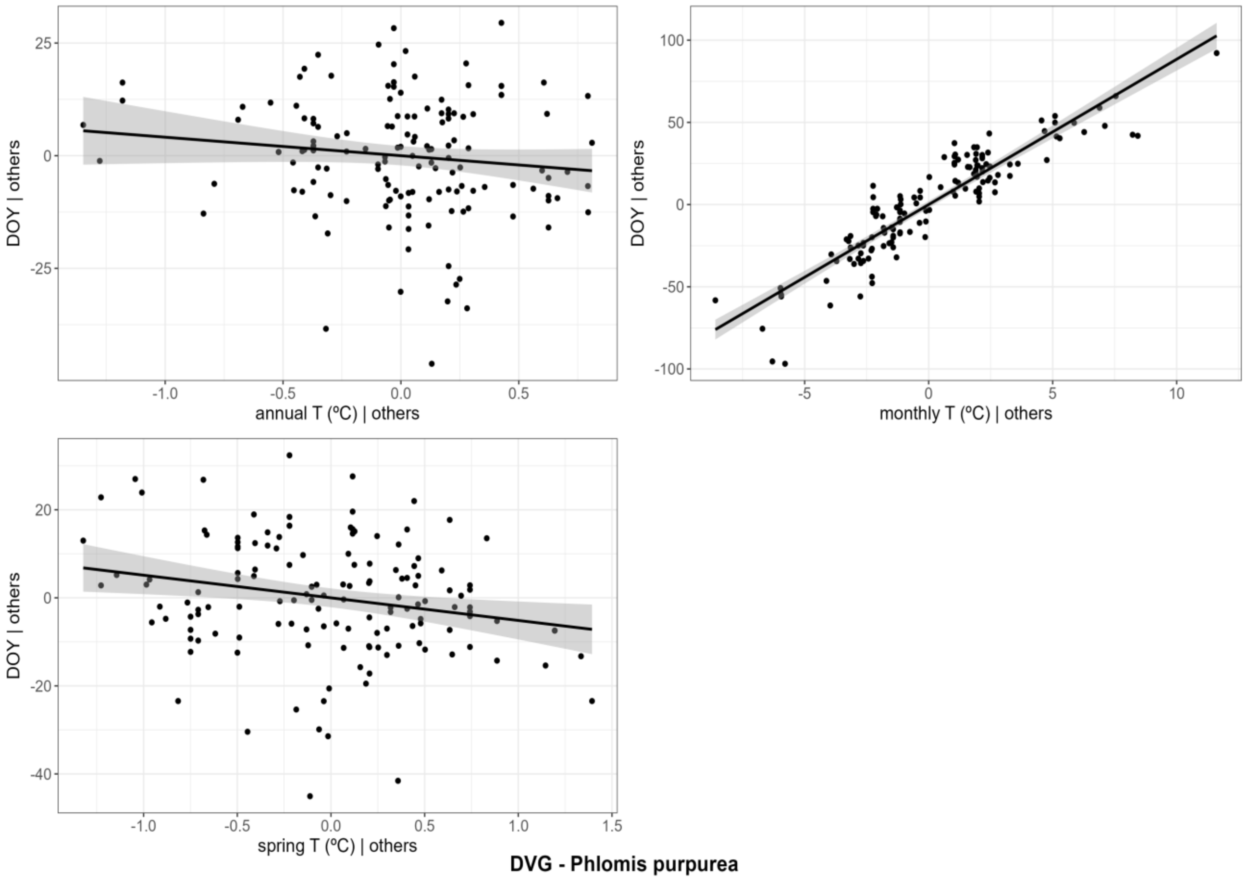

DOY = 118.35 (-4.11*annual T (ºC)) + (+8.84*monthly T (ºC)) + (-5.14*spring T (ºC))

1.87.1. Diagnostics - MLM - DVG - Phlomis purpurea

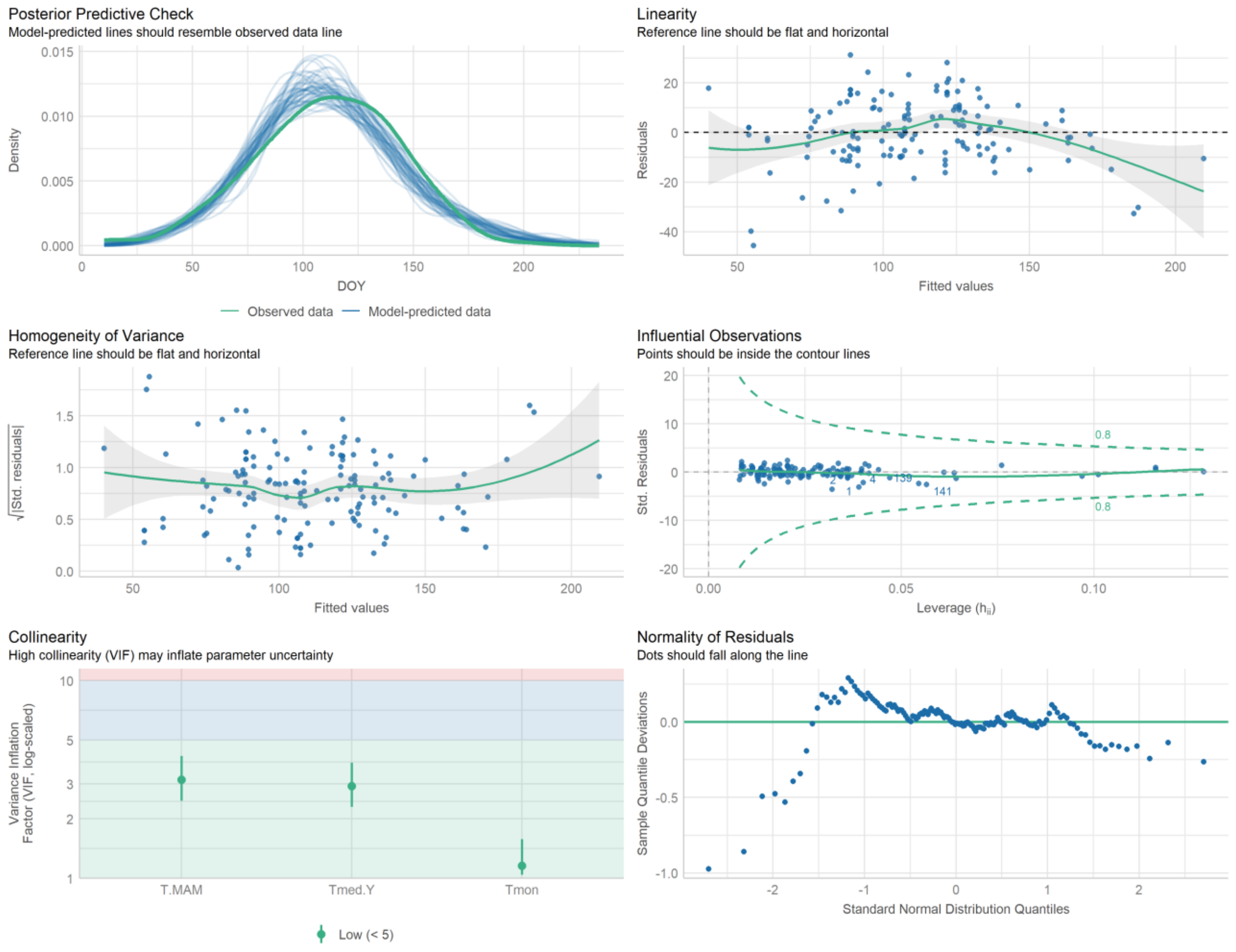

1.88. MLM - F - Pinus pinaster

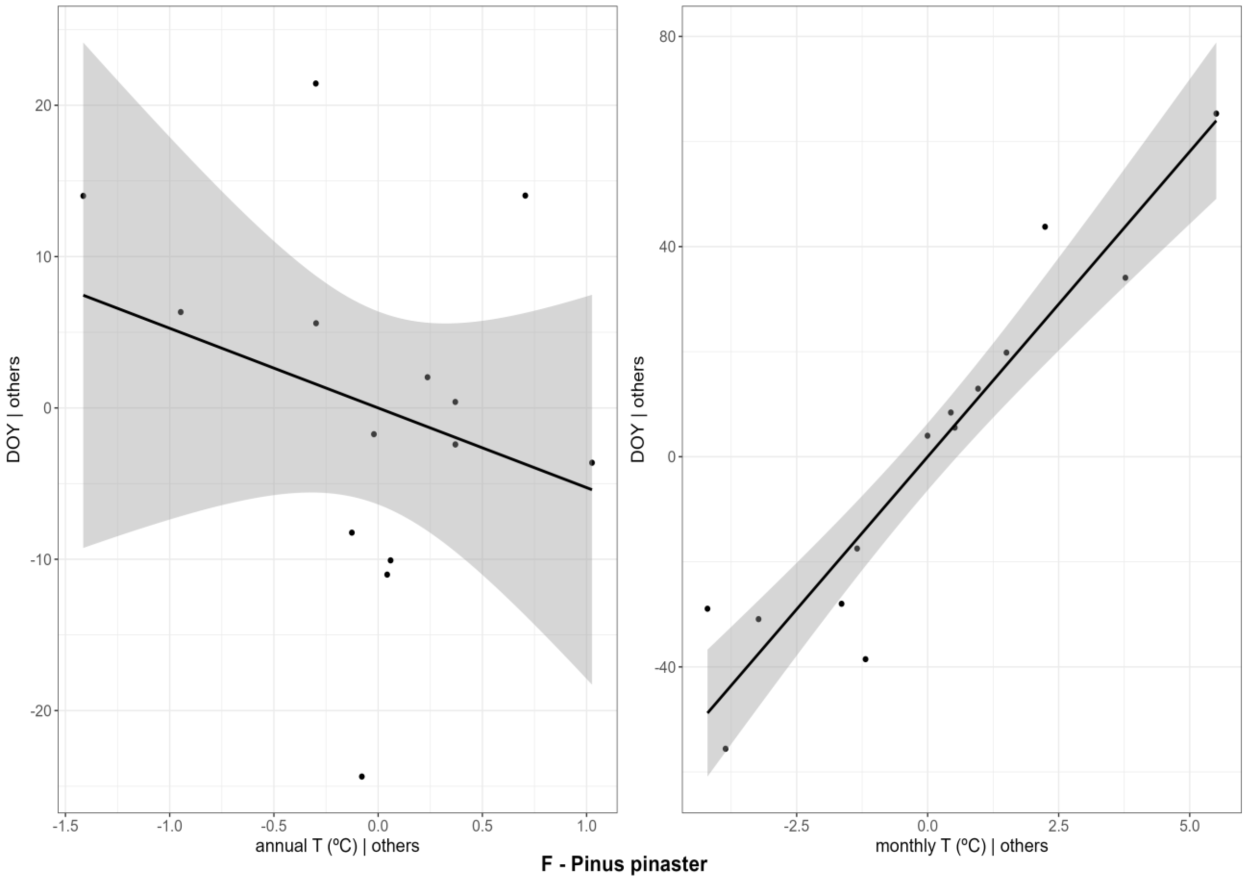

DOY = 21.54 (-5.27*annual T (ºC)) + (+11.62*monthly T (ºC))

1.88.1. Diagnostics - MLM - F - Pinus pinaster

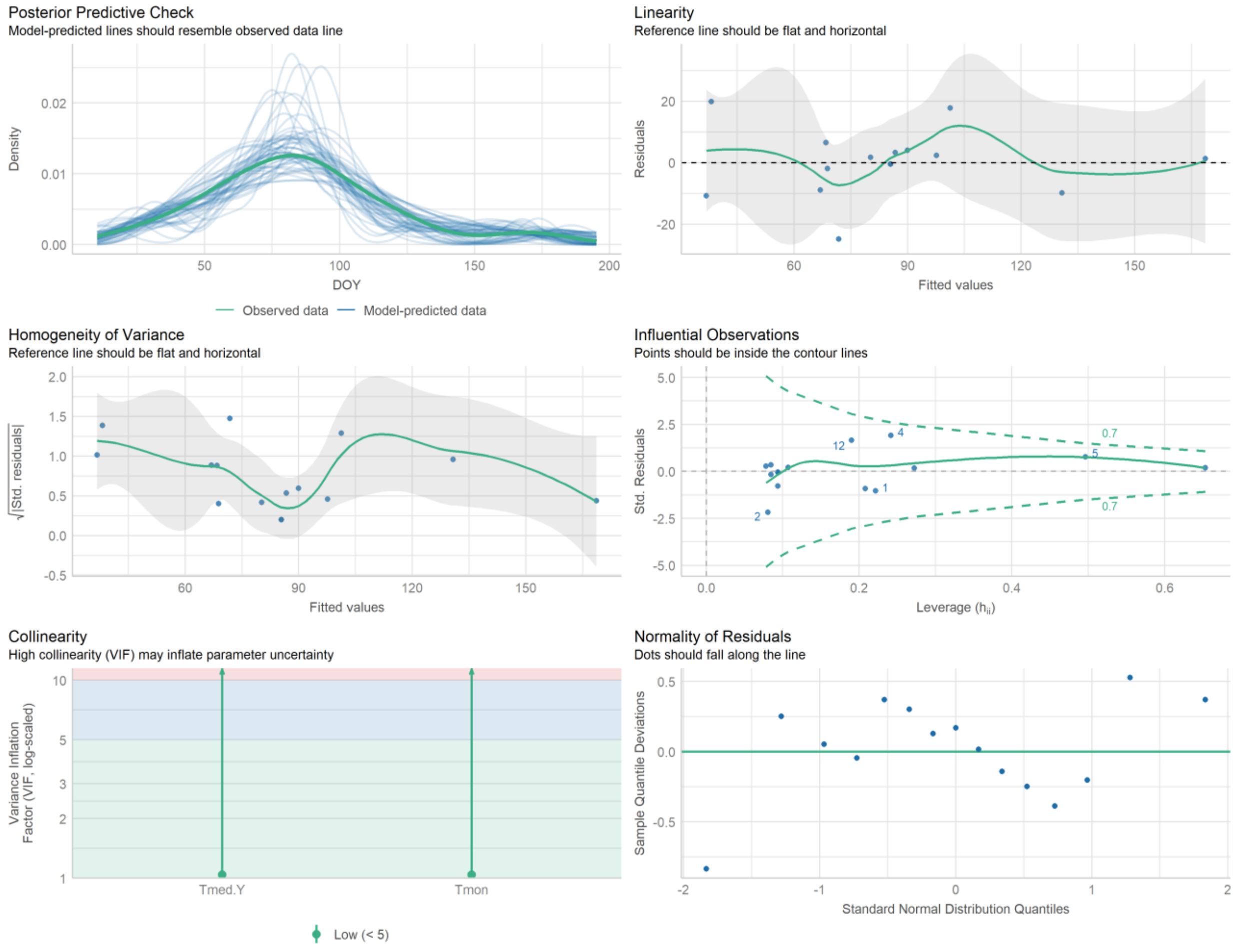

1.89. MLM - F - Pistacia lentiscus

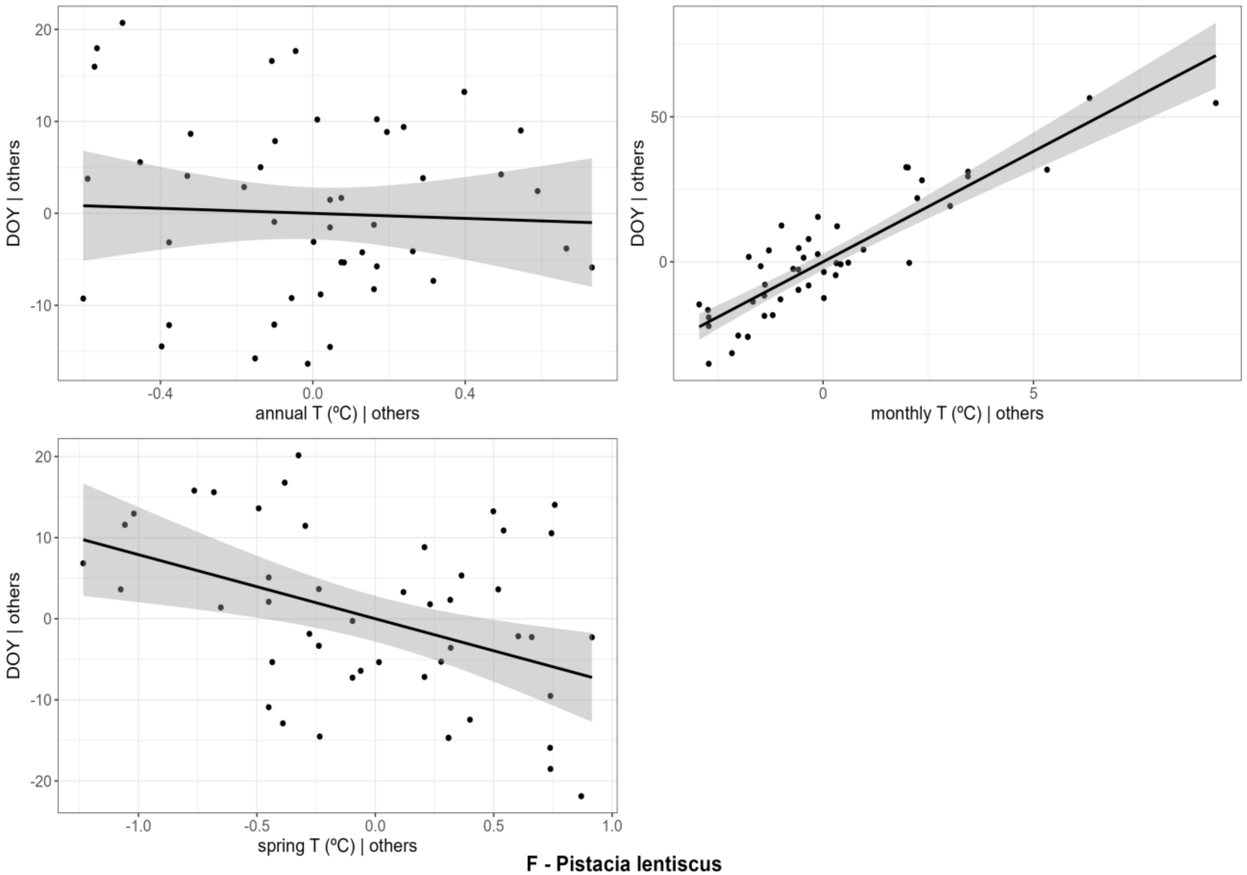

F - Pistacia lentiscus

DOY = 130.85 (-1.37*annual T (ºC)) + (+7.62*monthly T (ºC)) + (-7.90*spring T (ºC))

1.89.1. Diagnostics - MLM - F - Pistacia lentiscus

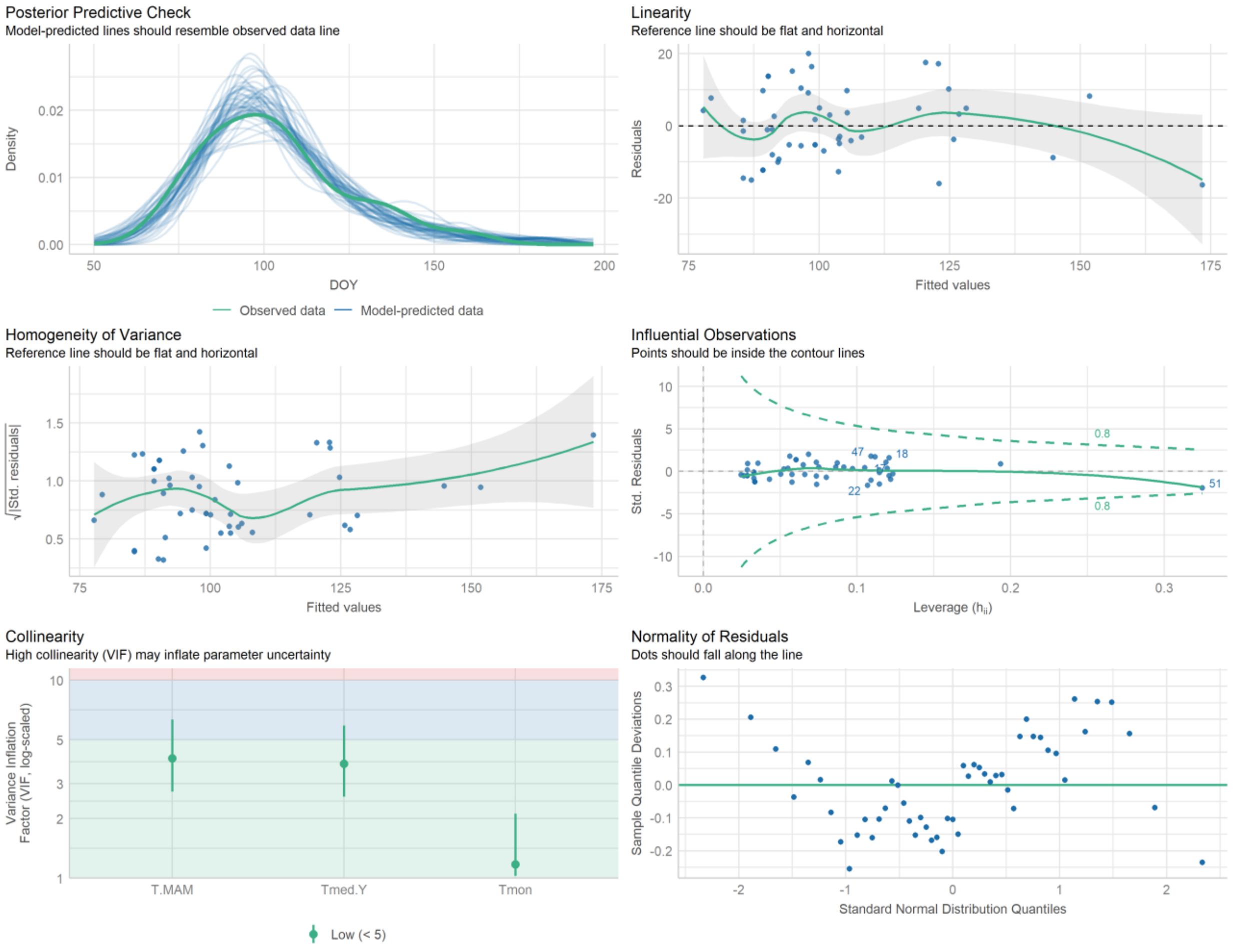

1.90. MLM - FS - Prunus mahaleb

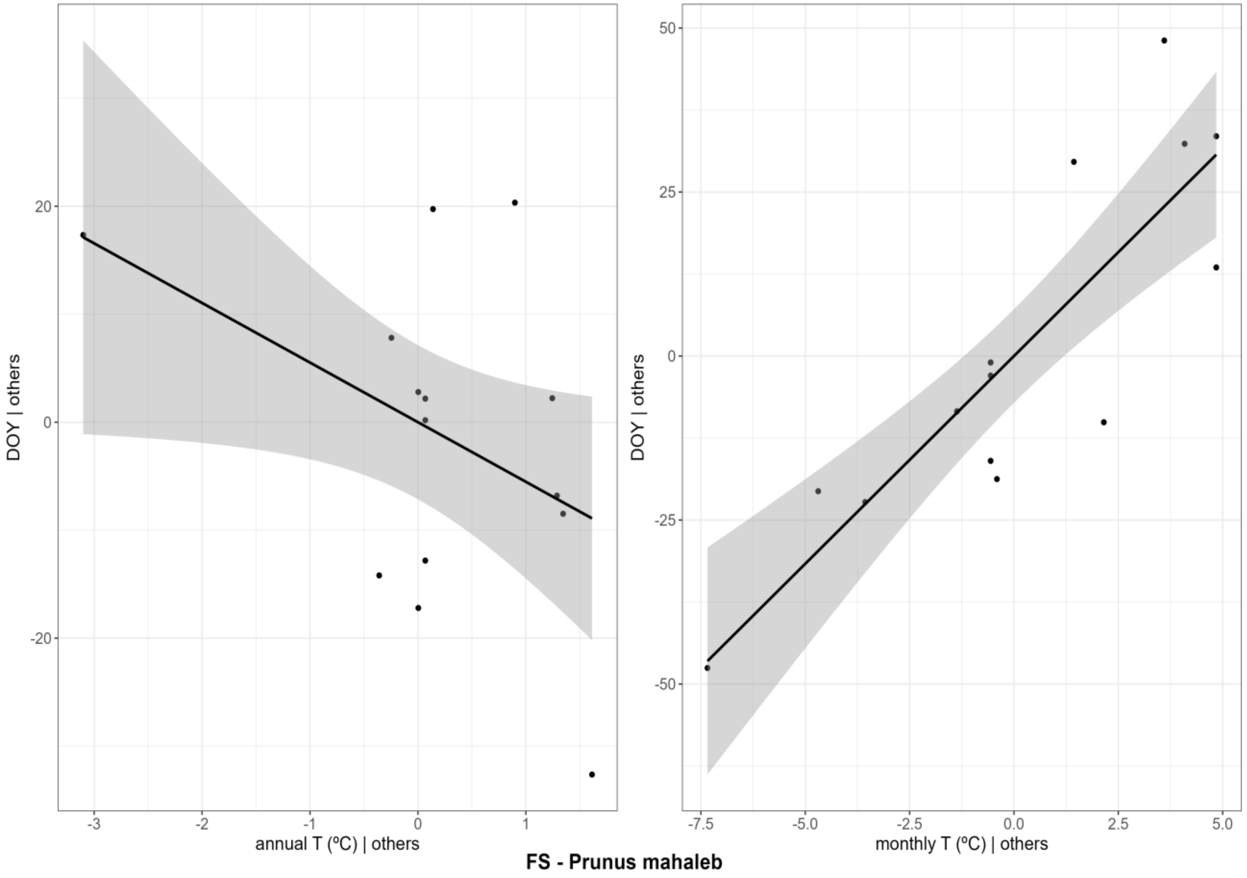

DOY = 127.85 (-5.52*annual T (ºC)) + (+6.33*monthly T (ºC))

1.90.1. Diagnostics - MLM - FS - Prunus mahaleb

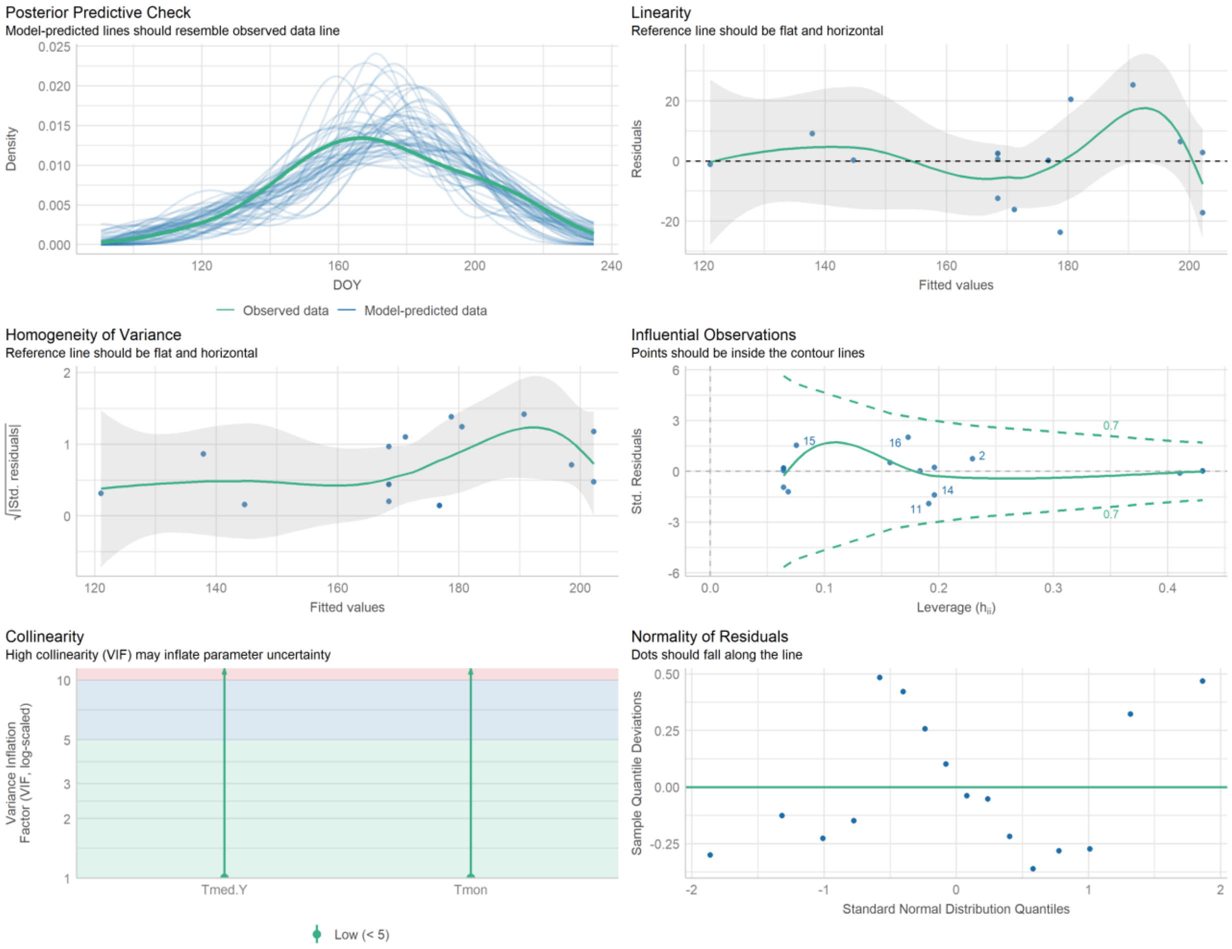

1.91. MLM - DVG - Prunus mahaleb

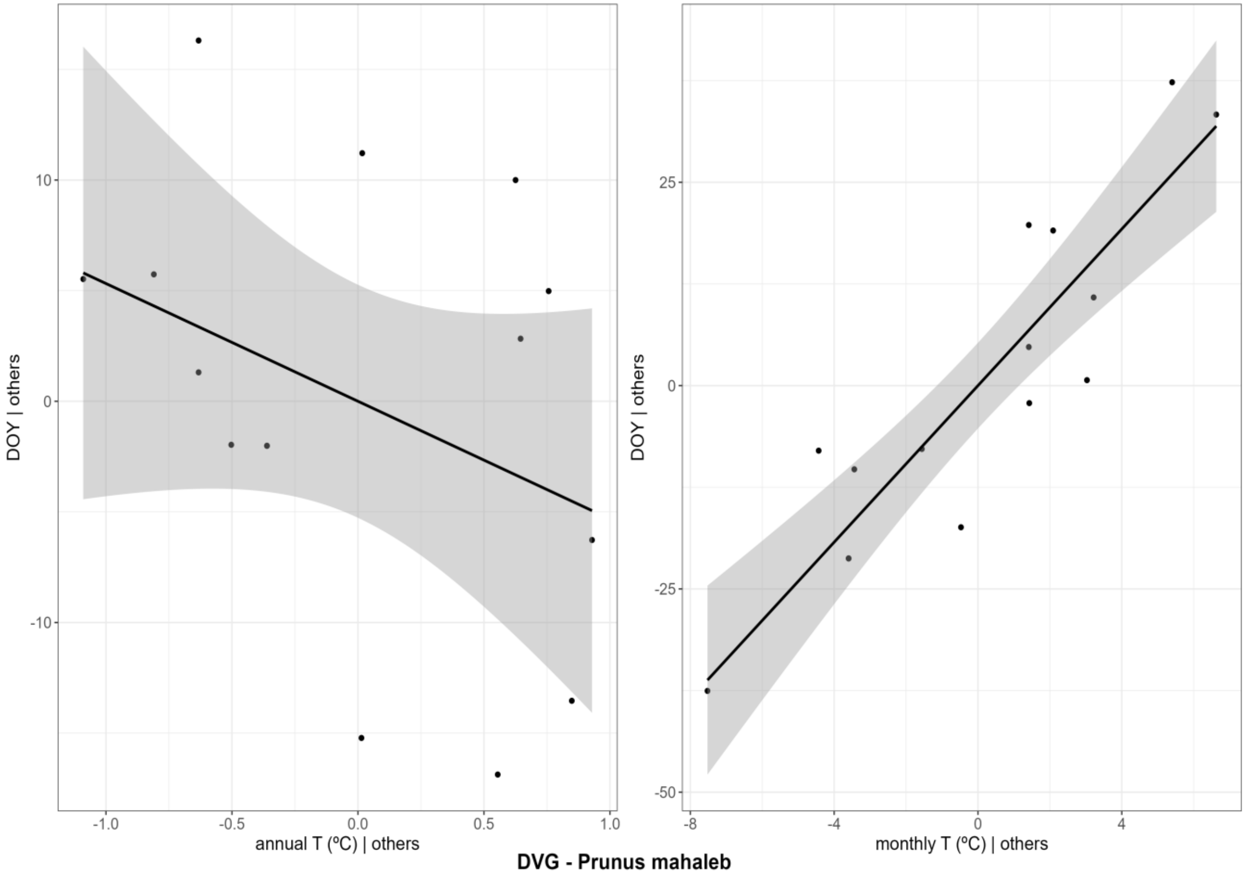

DOY = 144.80 (-5.32*annual T (ºC)) + (+4.81*monthly T (ºC))

1.91.1. Diagnostics - MLM - DVG - Prunus mahaleb

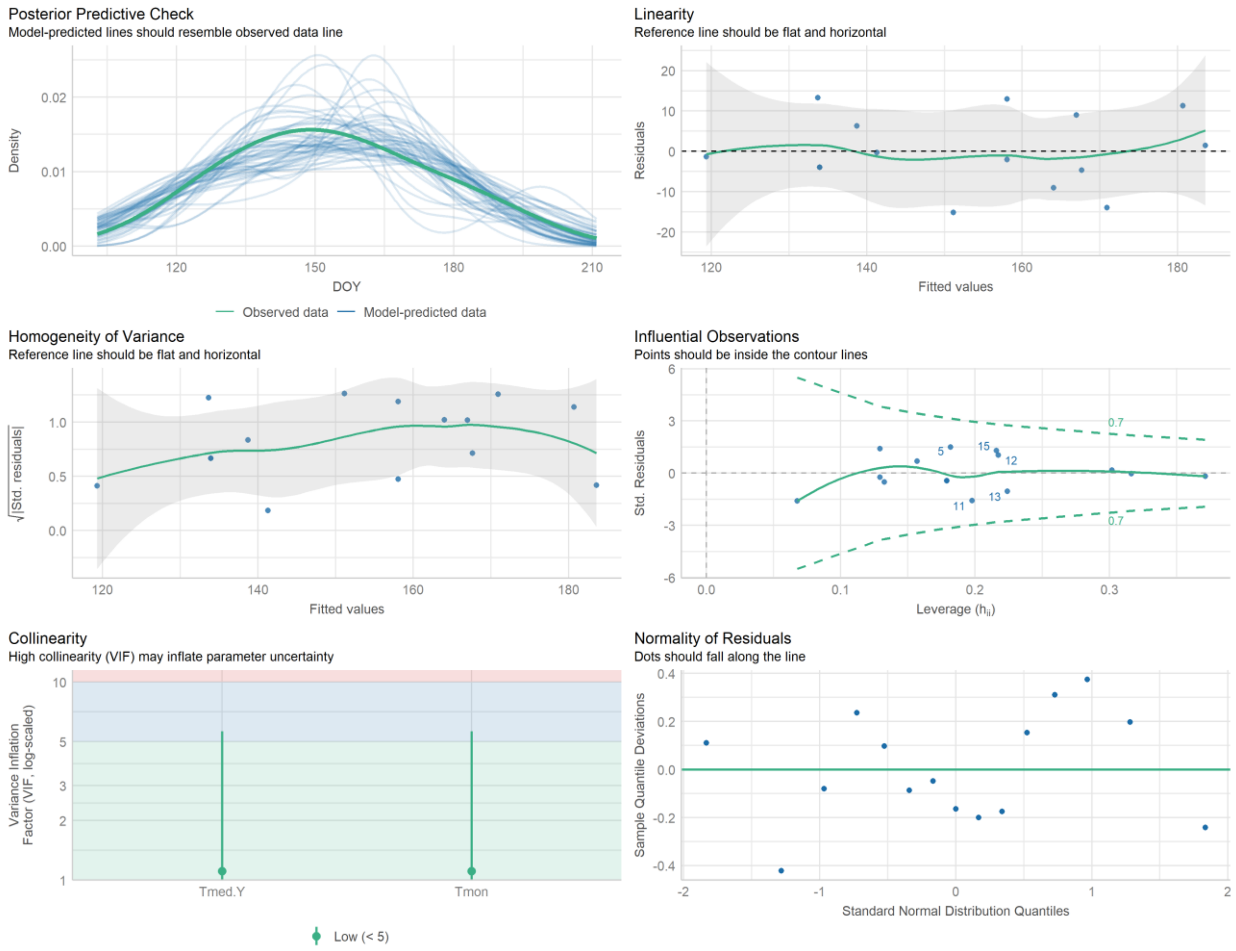

1.92. MLM - F - Prunus prostrata

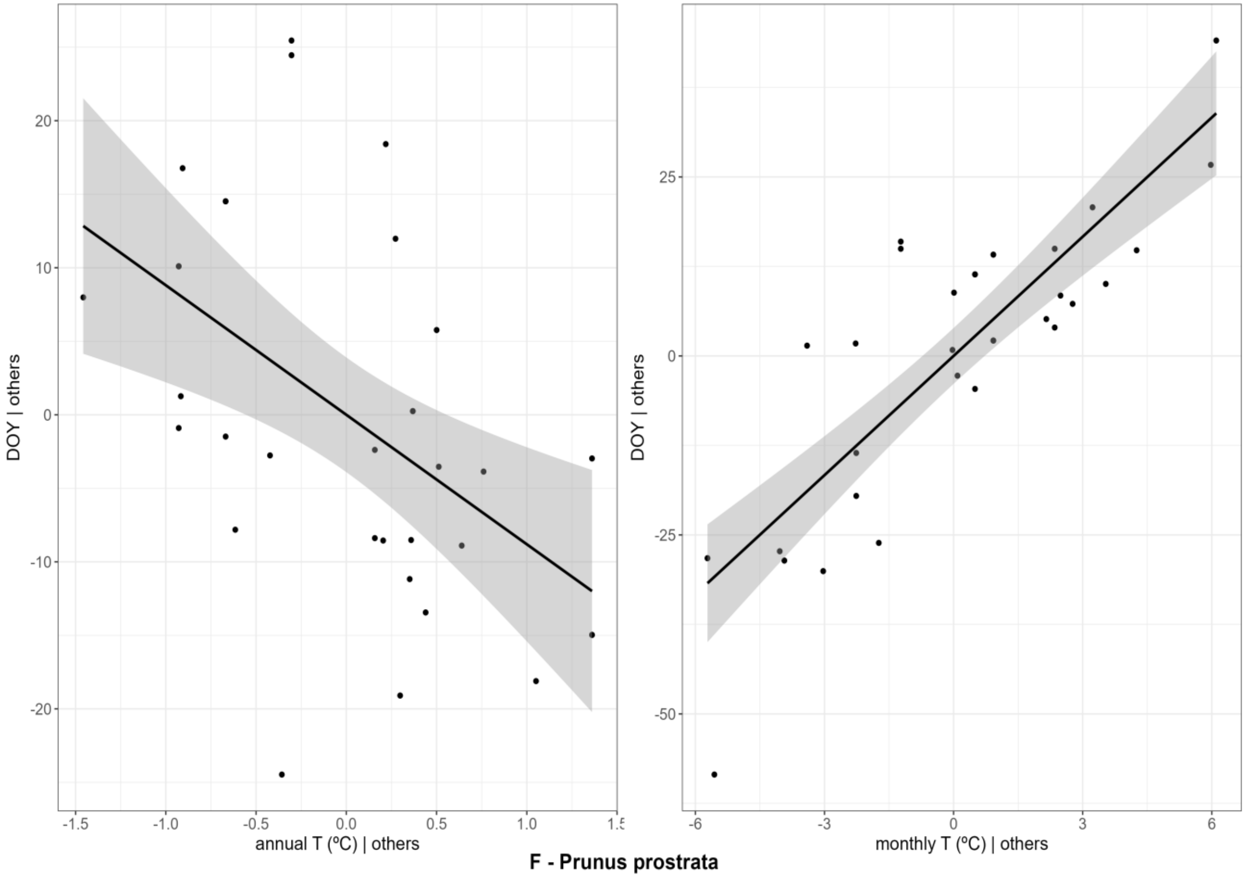

DOY = 188.82 (-8.81*annual T (ºC)) + (+5.55*monthly T (ºC))

1.92.1. Diagnostics - MLM - F - Prunus prostrata

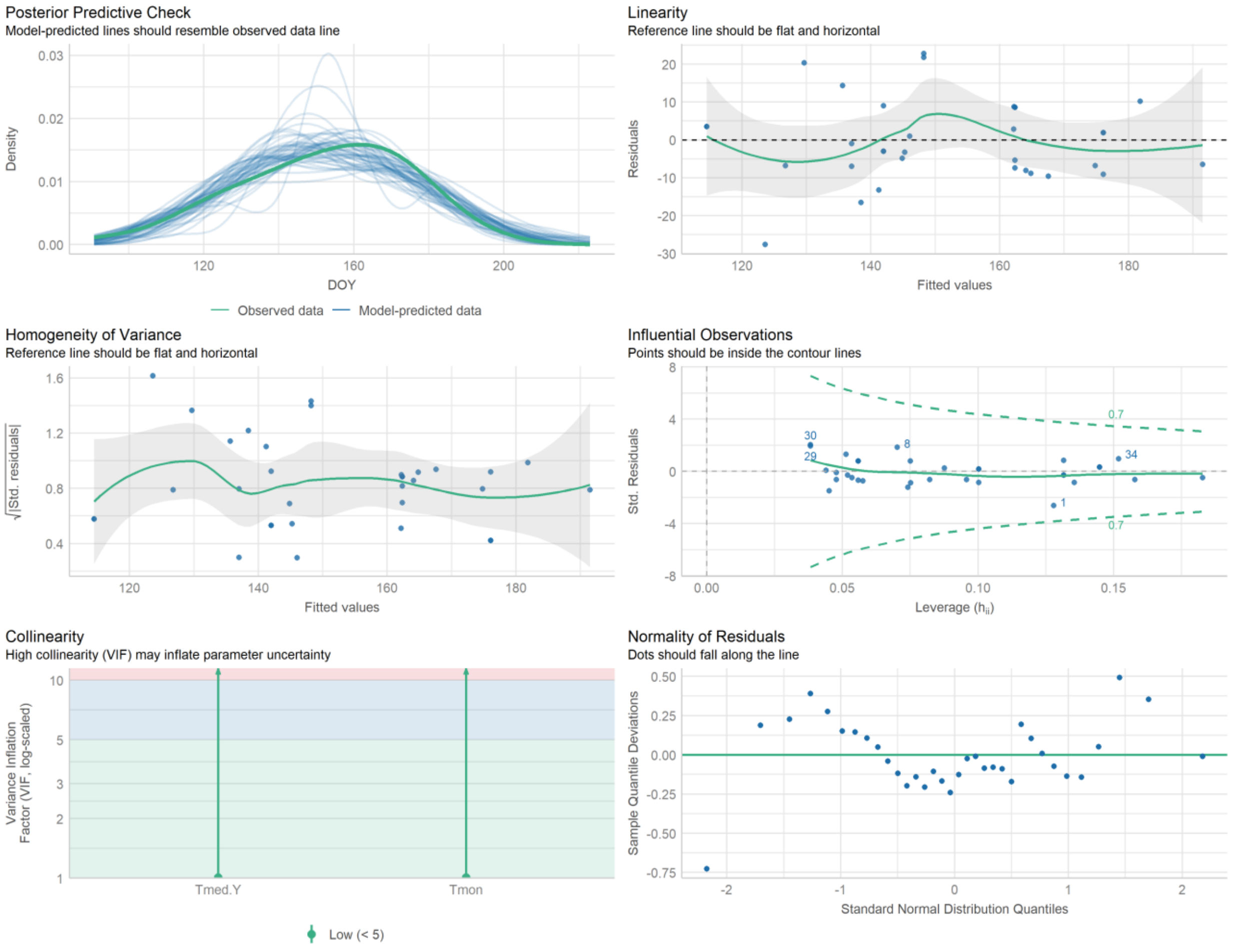

1.93. MLM - FS - Prunus prostrata

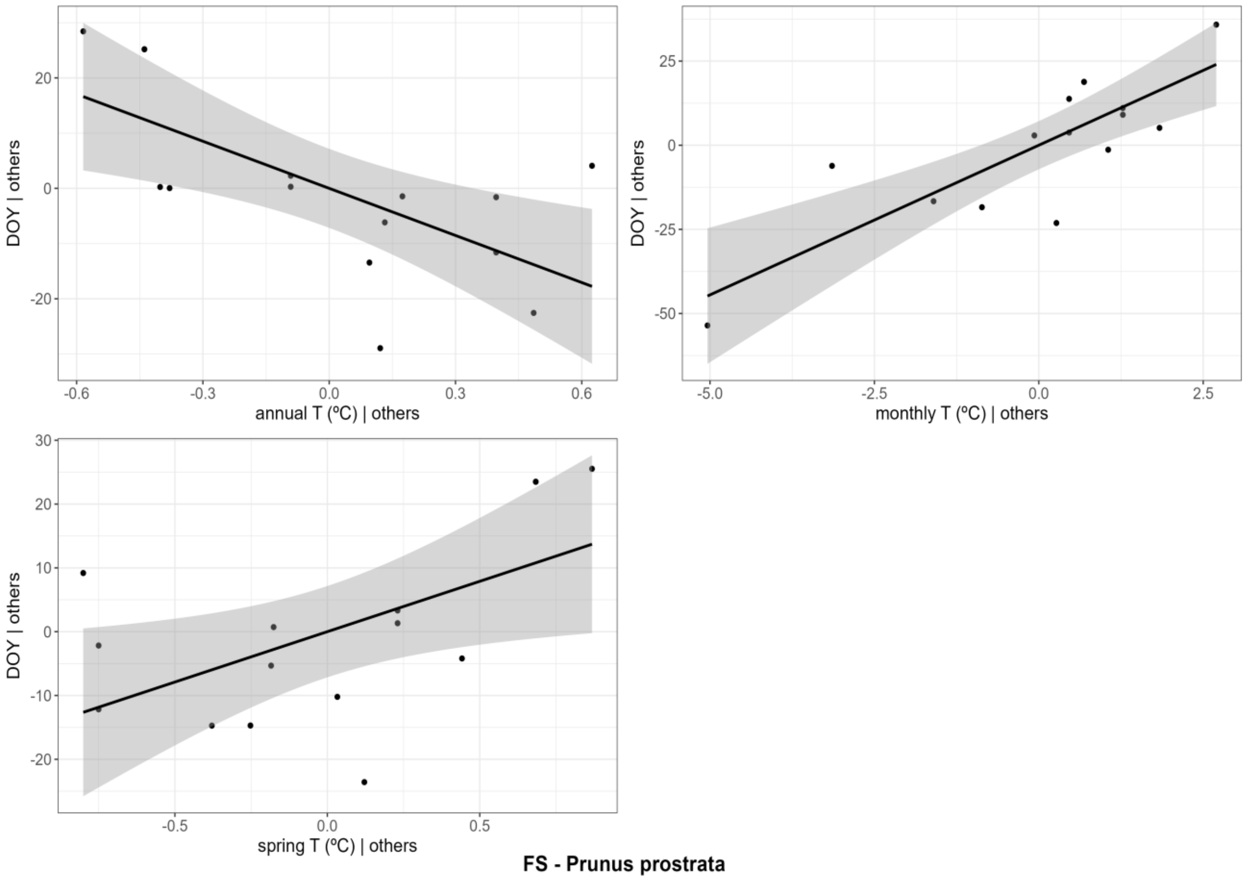

DOY = 206.89 (-28.43*annual T (ºC)) + (+8.89*monthly T (ºC)) + (+15.79*spring T (ºC))

1.93.1. Diagnostics - MLM - FS - Prunus prostrata

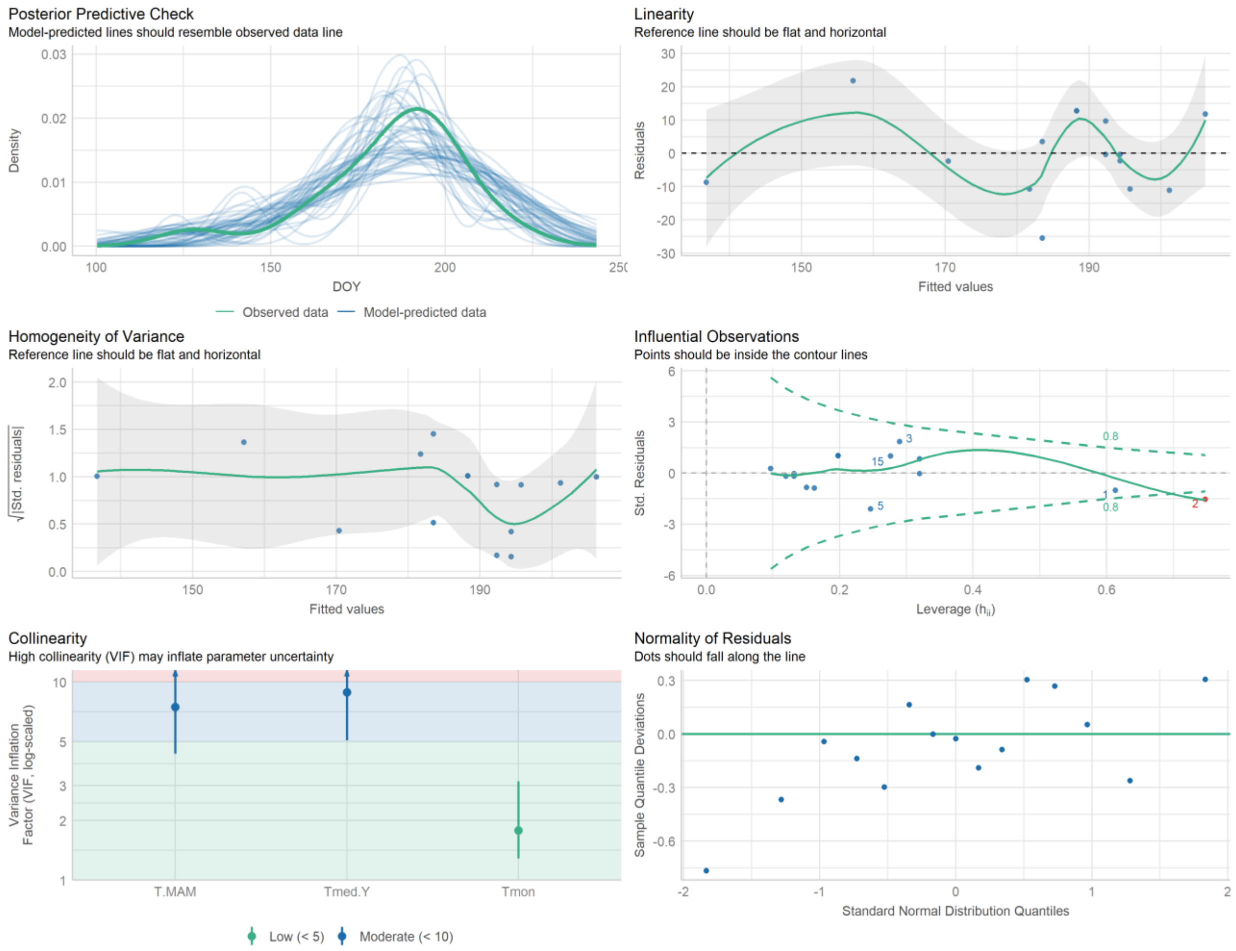

1.94. MLM - F - Quercus coccifera

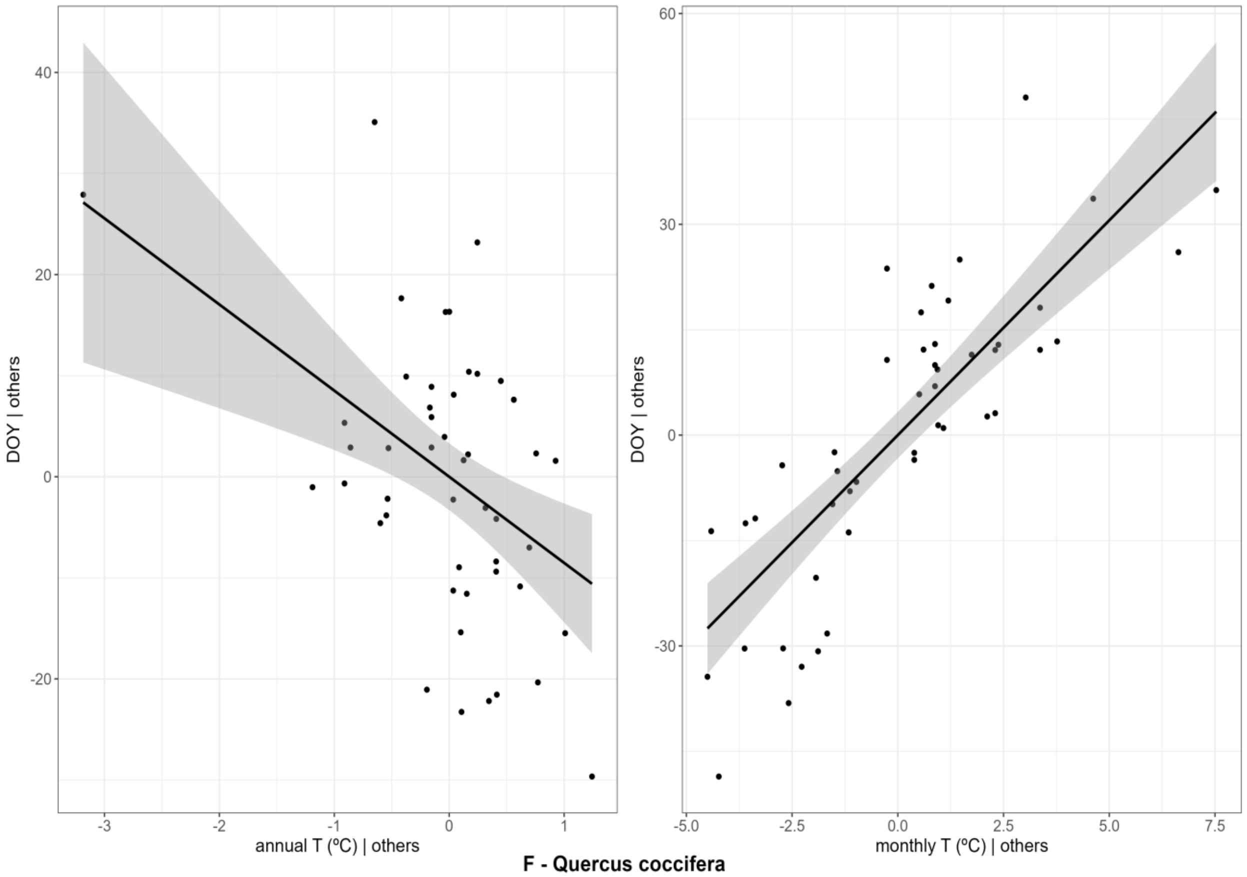

DOY = 165.25 (-8.52*annual T (ºC)) + (+6.11*monthly T (ºC))

1.94.1. Diagnostics - MLM - F - Quercus coccifera

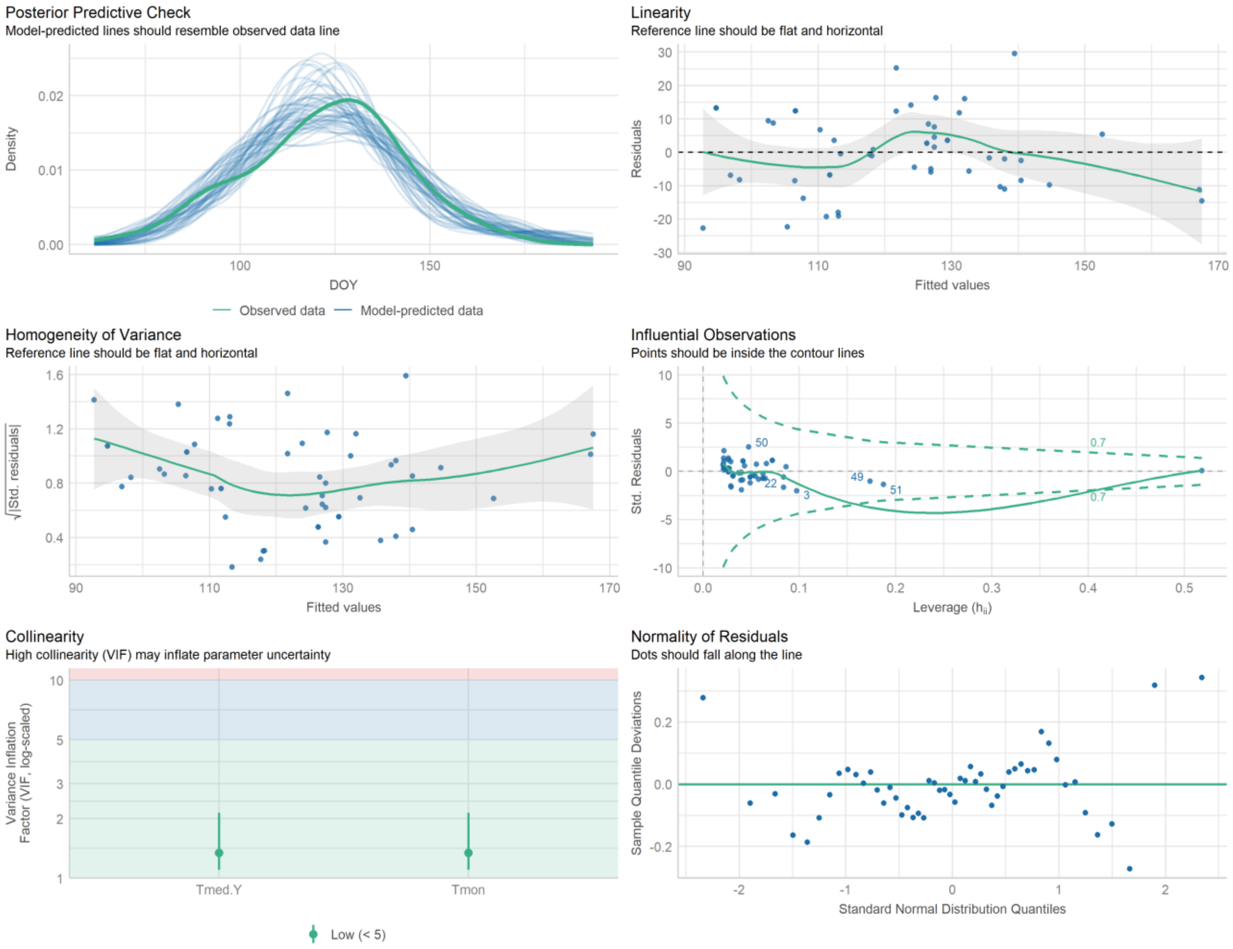

1.95. MLM - DVG - Quercus coccifera

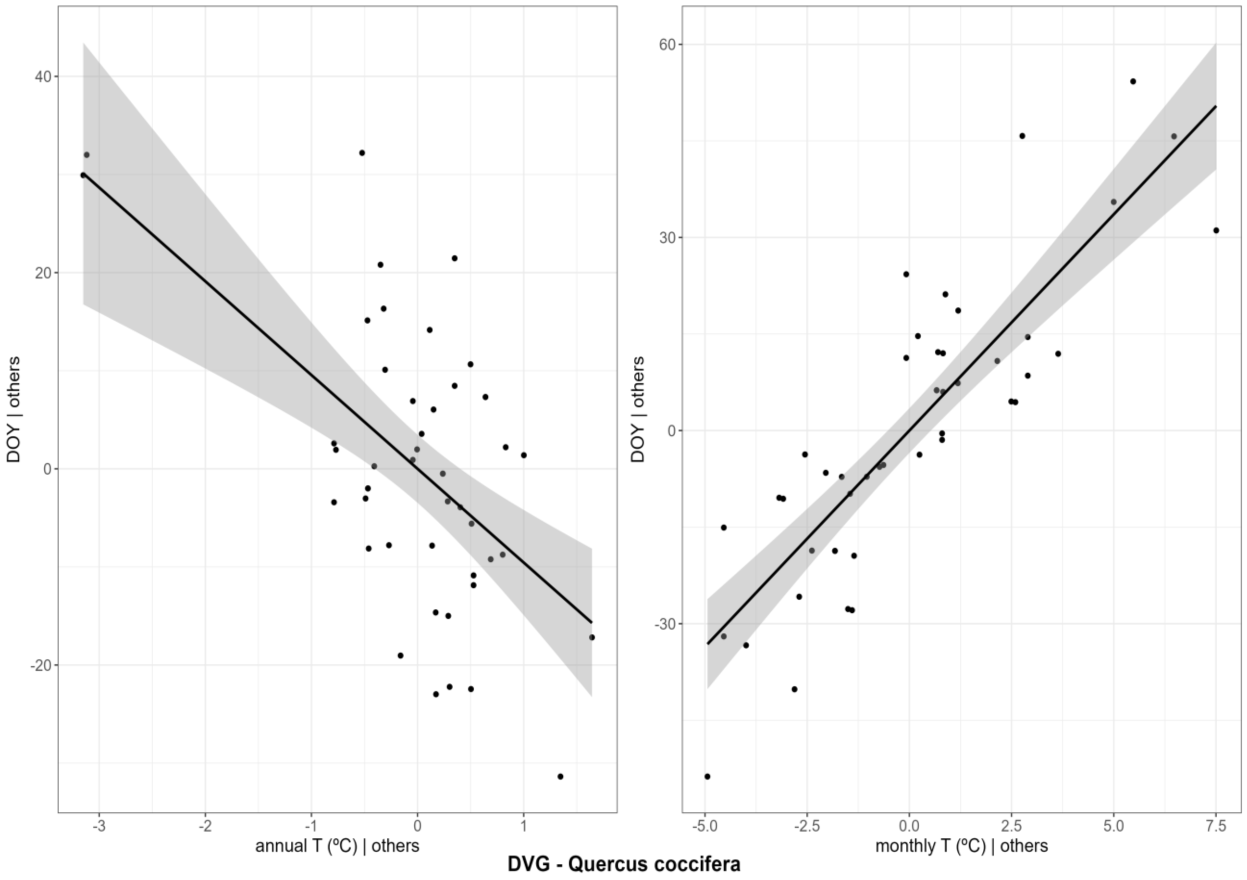

DOY = 173.24 (-9.56*annual T (ºC)) + (+6.72*monthly T (ºC))

1.95.1. Diagnostics - MLM - DVG - Quercus coccifera

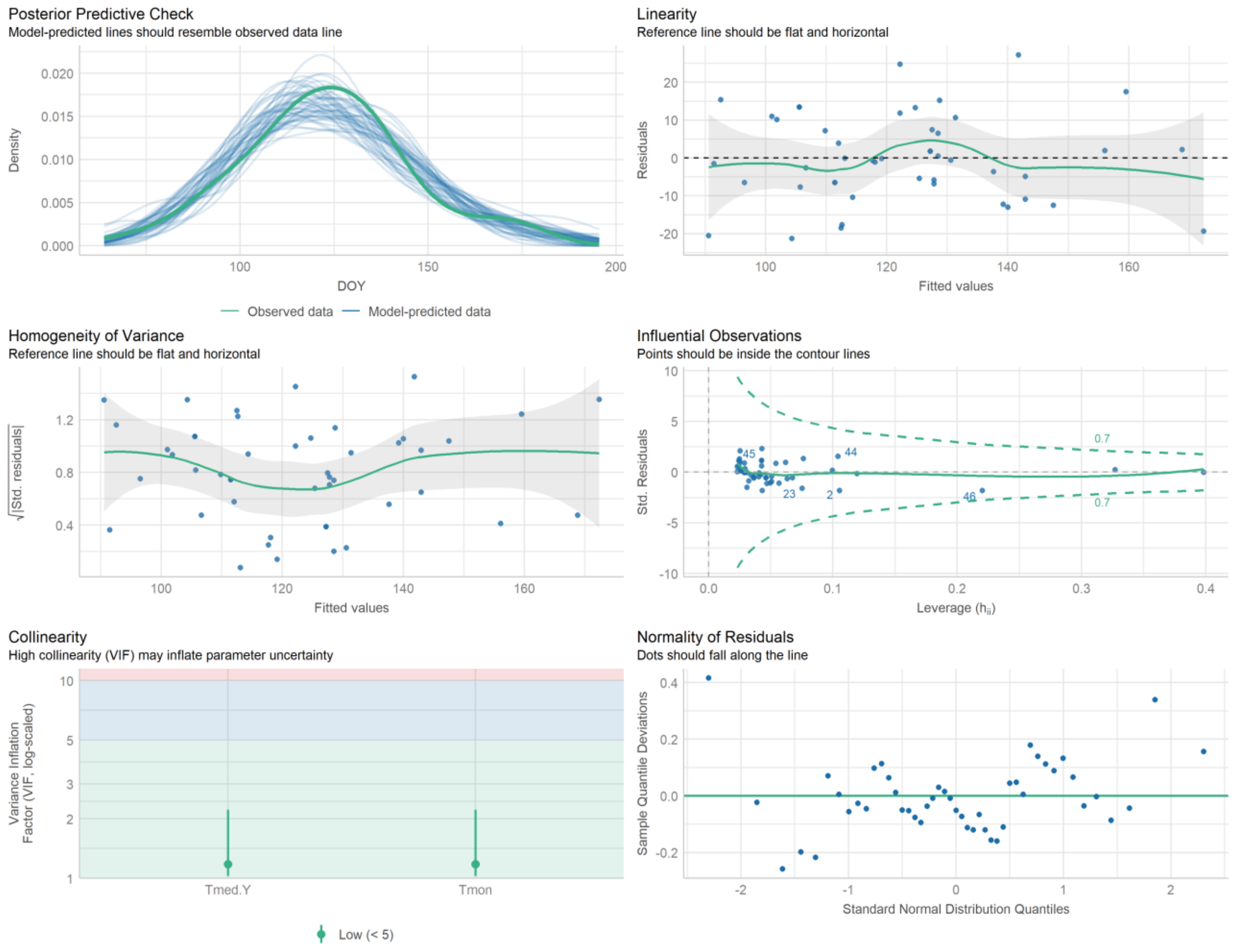

1.96. MLM - F - Quercus faginea

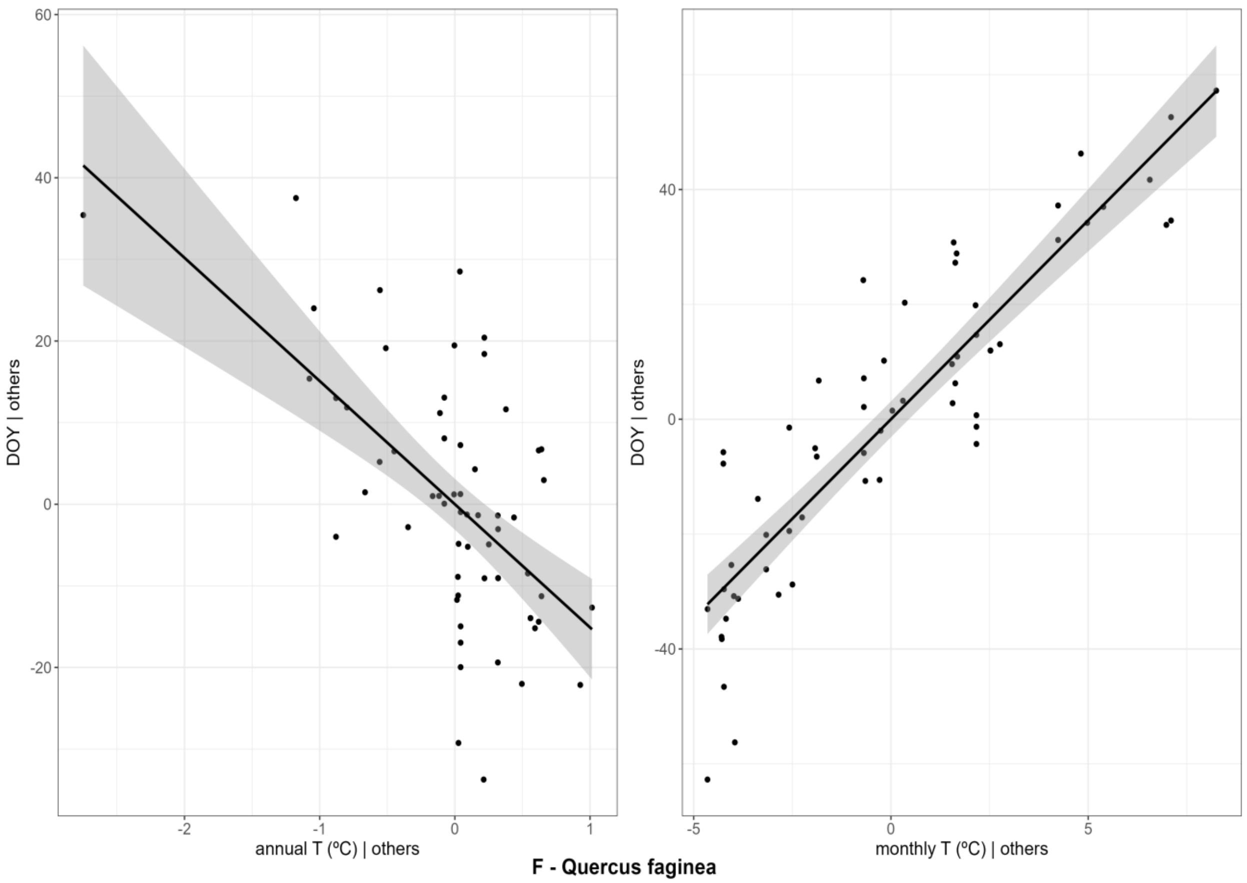

DOY = 255.63 (-15.10*annual T (ºC)) + (+6.93*monthly T (ºC))

1.96.1. Diagnostics - MLM - F - Quercus faginea

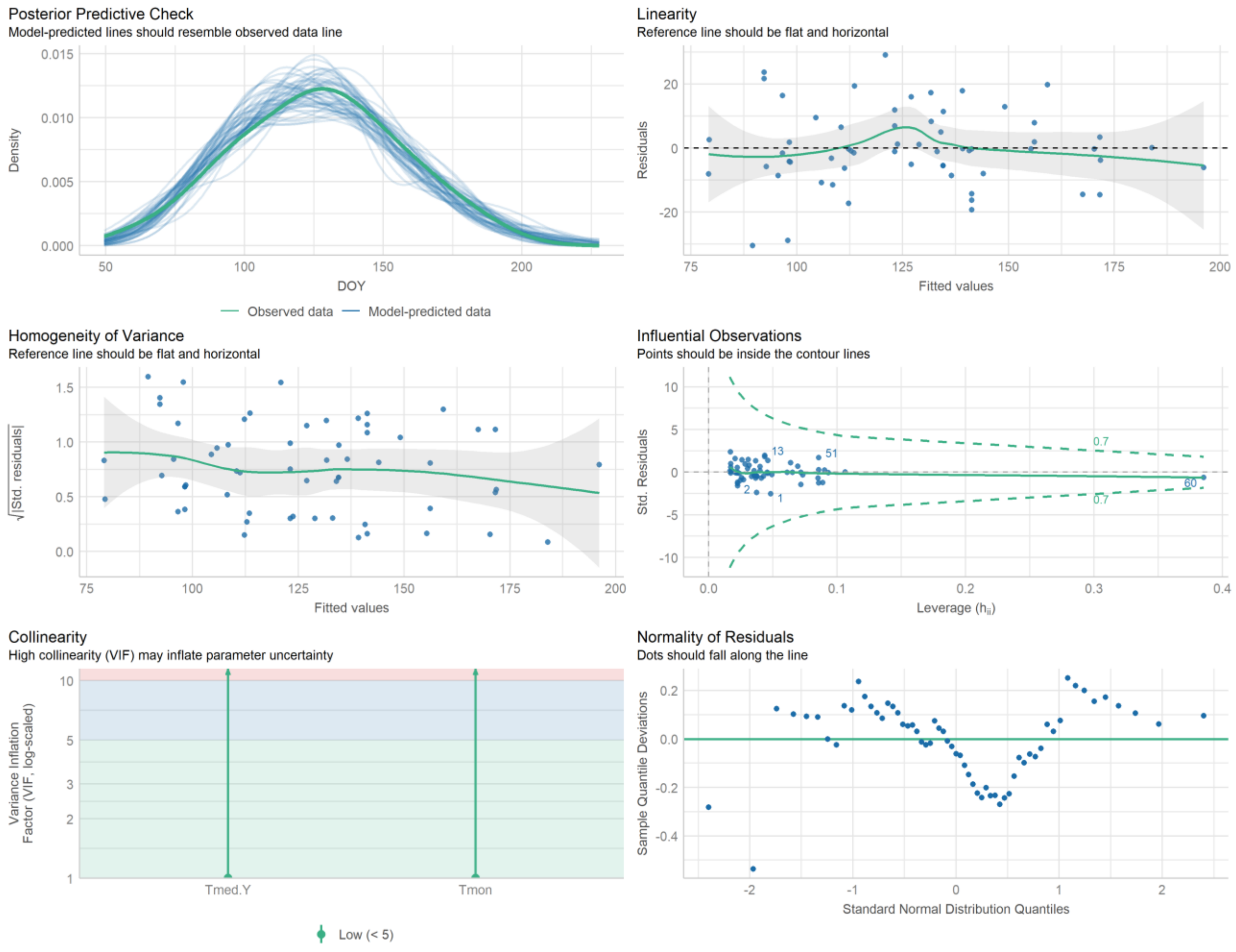

1.97. MLM - DVG - Quercus faginea

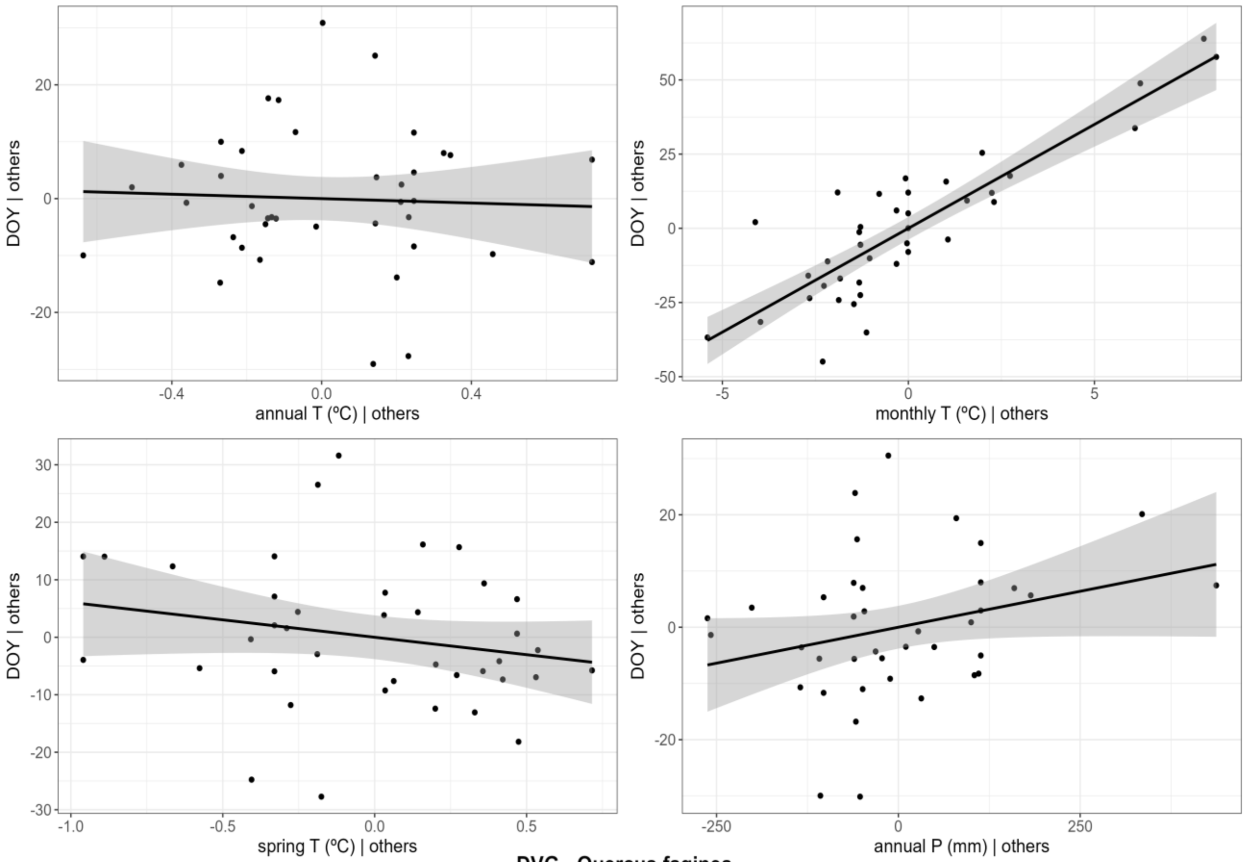

DVG - Quercus faginea

DOY = 109.70 (-1.93*annual T (ºC)) + (+7.00*monthly T (ºC)) + (-6.06*spring T (ºC)) + (+0.03*annual P (mm))

1.97.1. Diagnostics - MLM - DVG - Quercus faginea

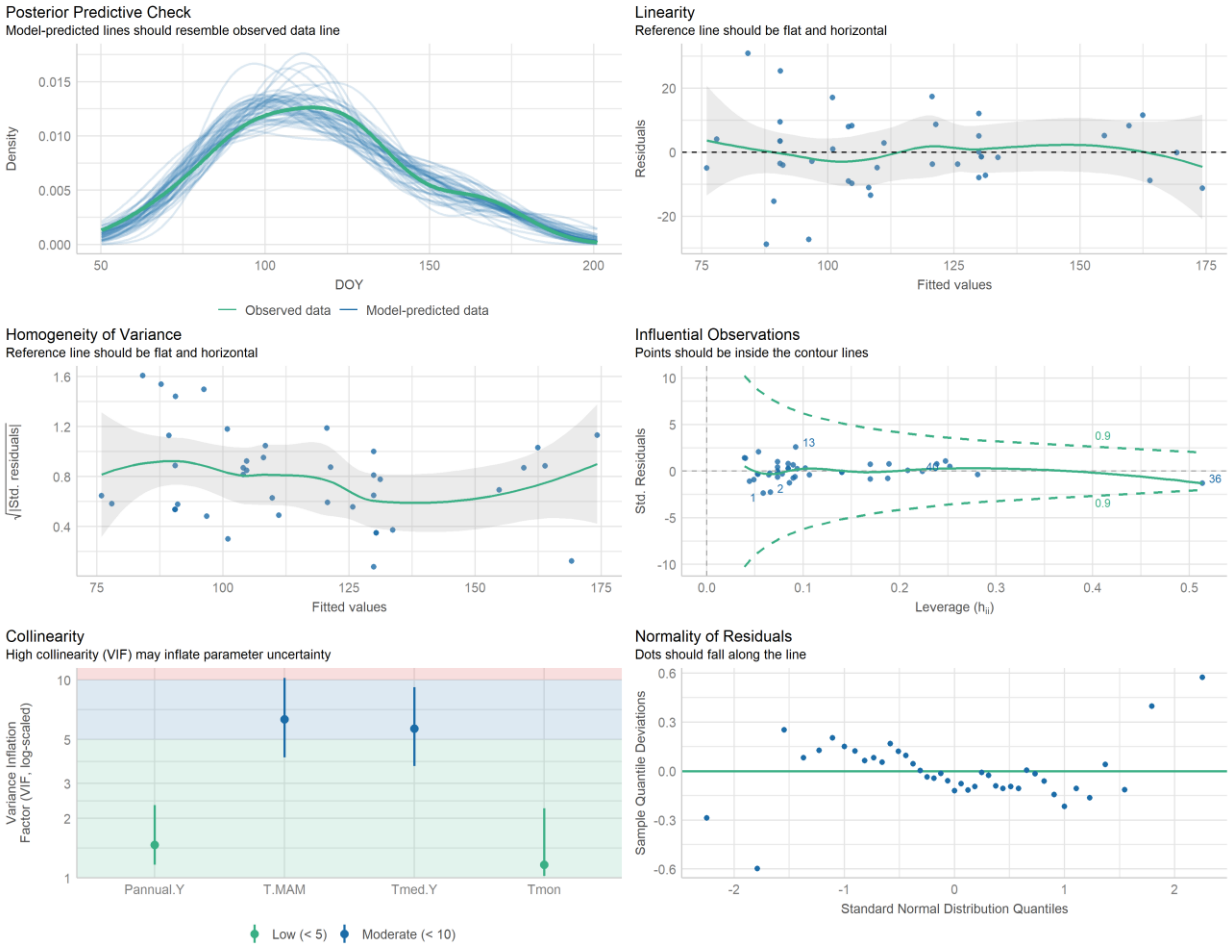

1.98. MLM - F - Quercus faginea subsp. alpestris

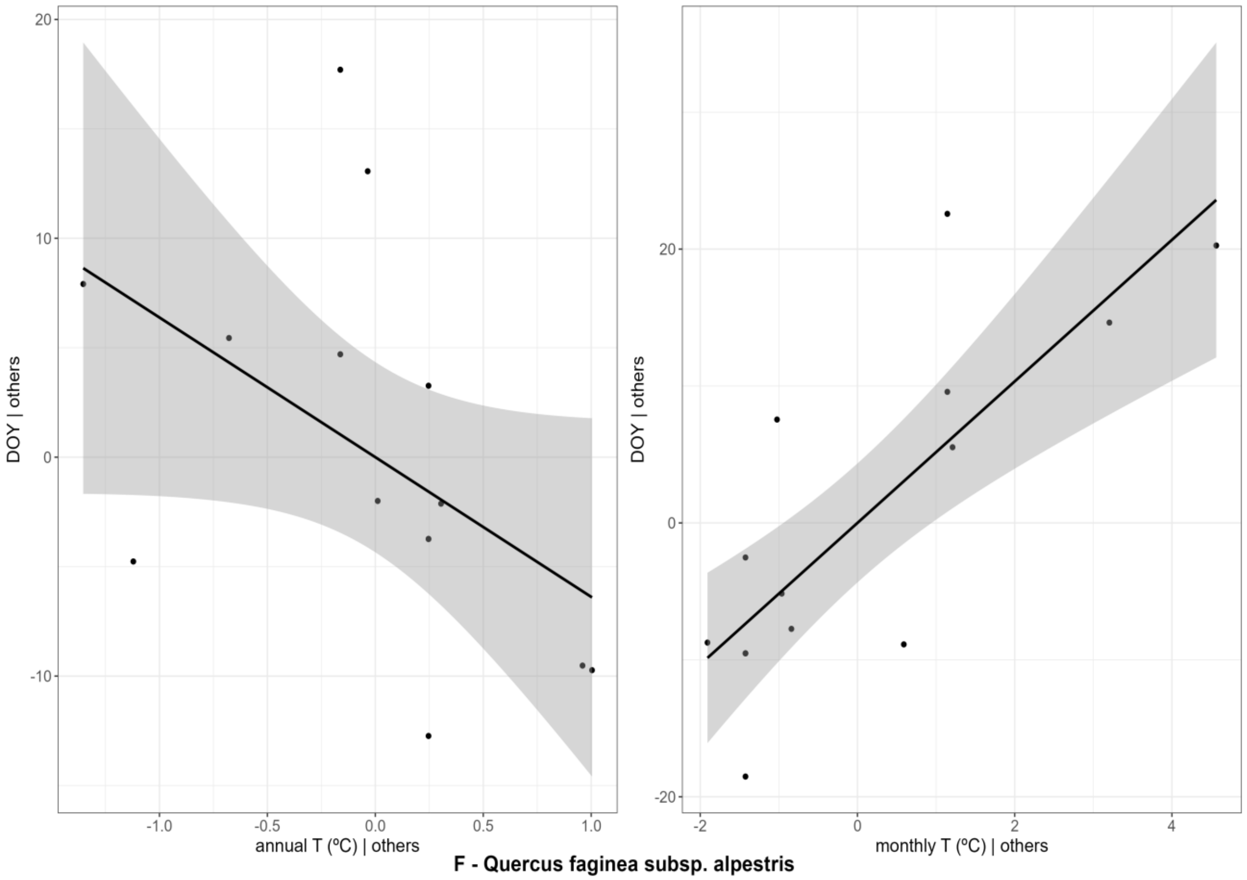

DOY = 153.88 (-6.38*annual T (ºC)) + (+5.17*monthly T (ºC))

1.98.1. Diagnostics - MLM - F - Quercus faginea subsp. alpestris

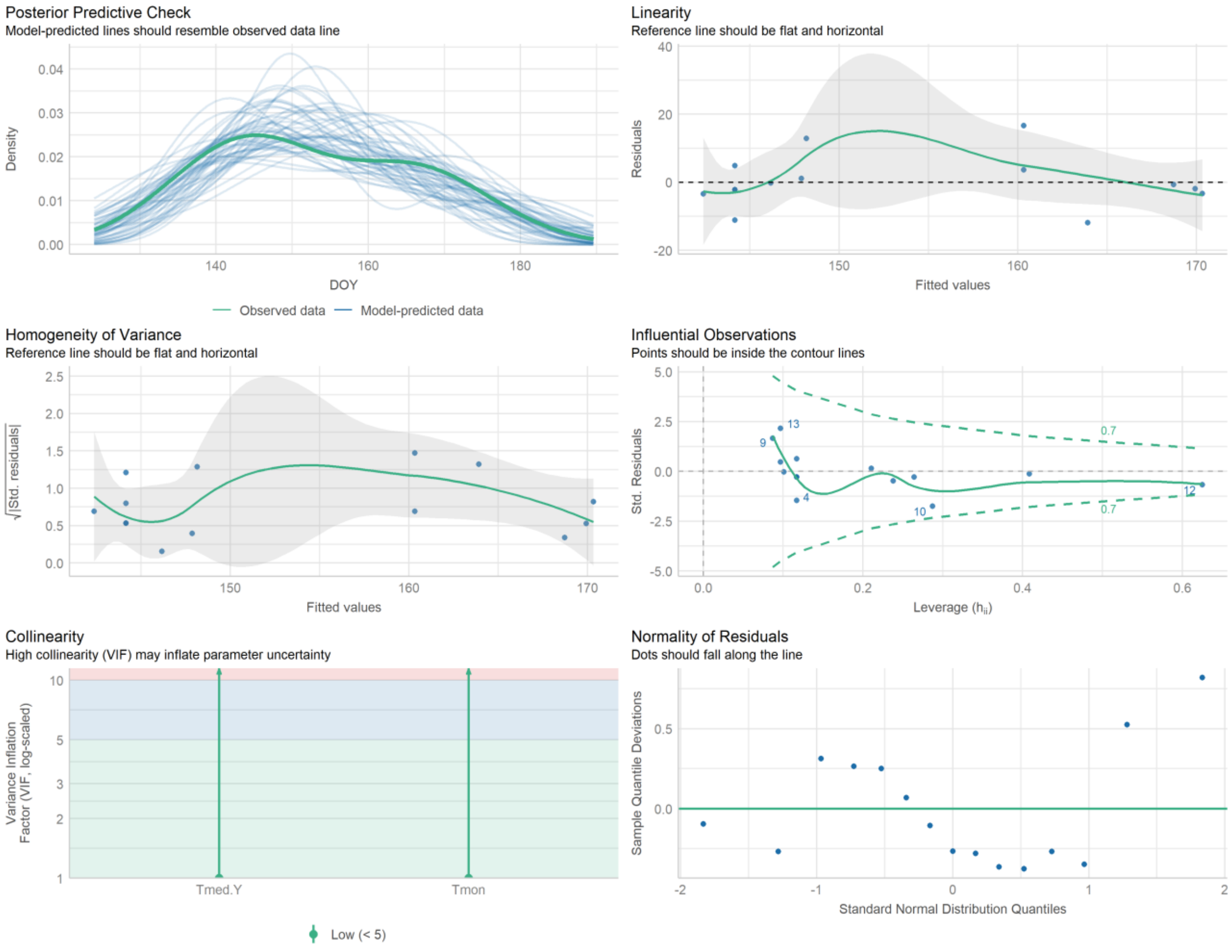

1.99. MLM - F - Quercus rotundifolia

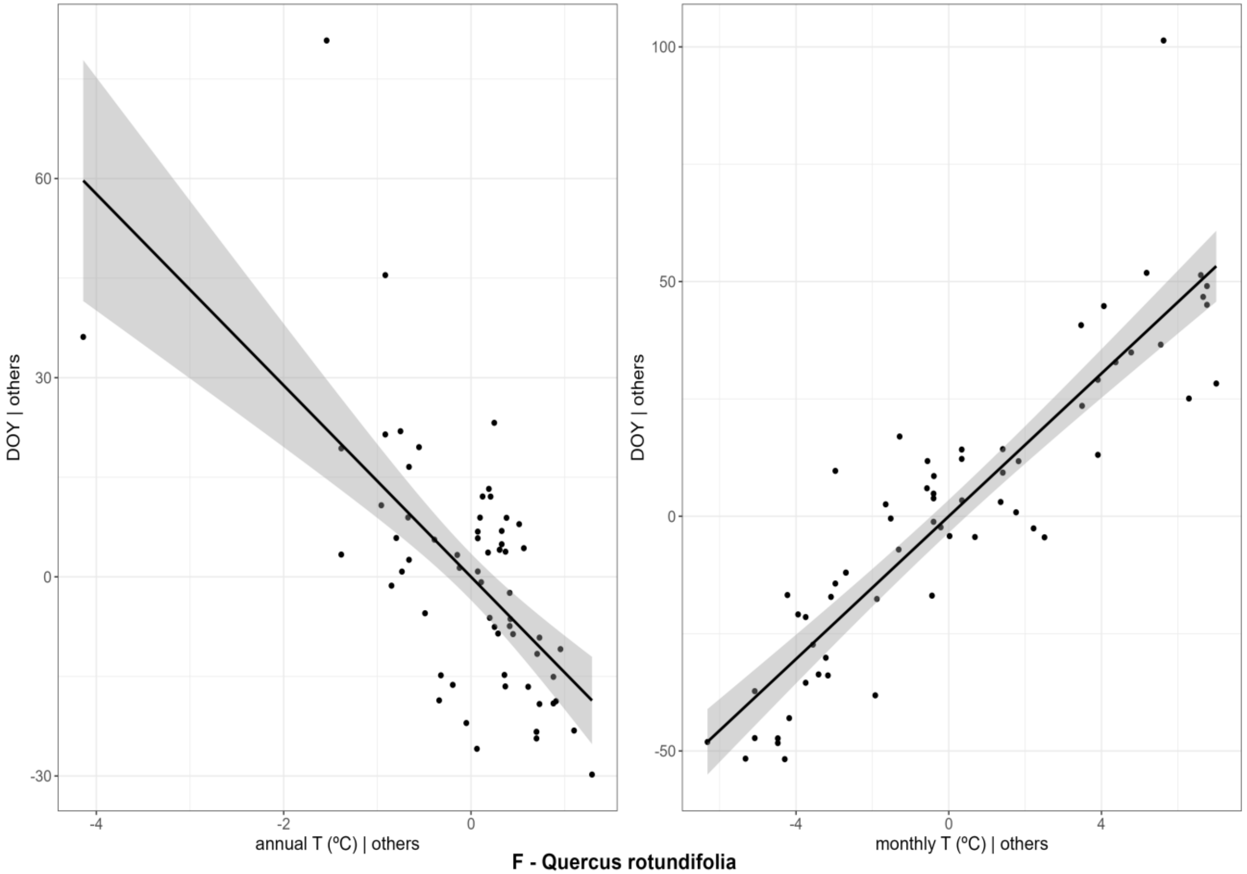

F - Quercus rotundifolia

DOY = 238.10 (-14.42*annual T (ºC)) + (+7.60*monthly T (ºC))

1.99.1. Diagnostics - MLM - F - Quercus rotundifolia

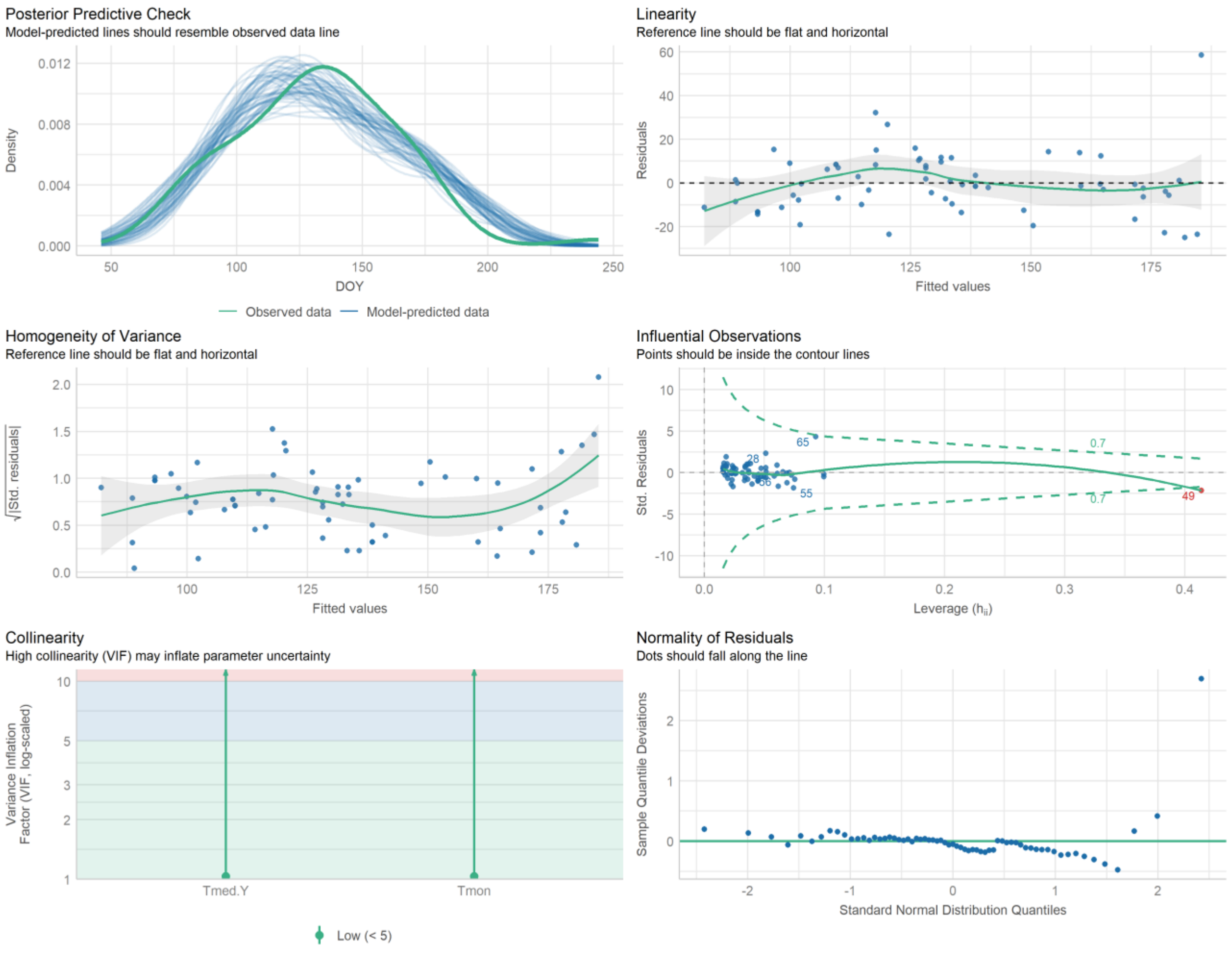

1.100. MLM - DVG - Quercus rotundifolia

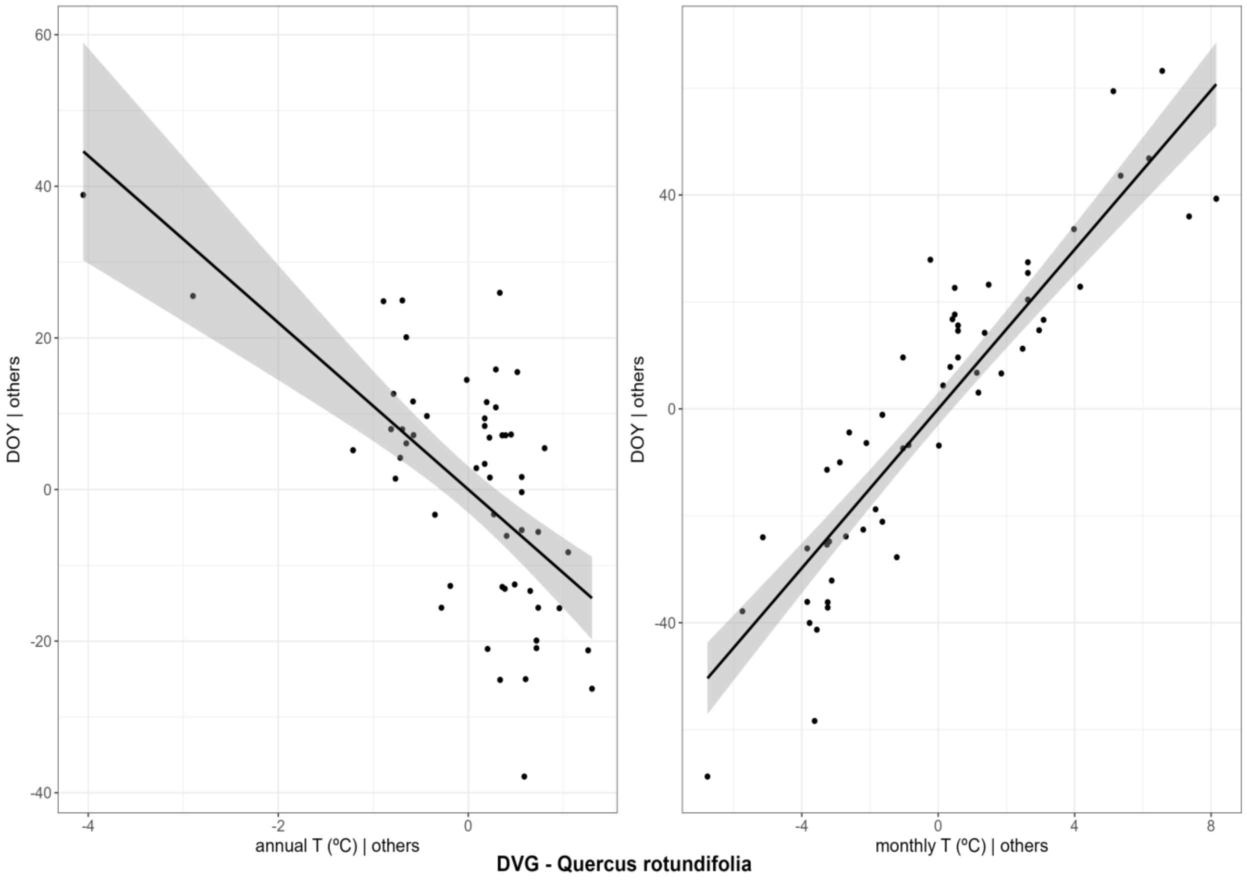

DOY = 180.79 (-11.00*annual T (ºC)) + (+7.45*monthly T (ºC))

1.100.1. Diagnostics - MLM - DVG - Quercus rotundifolia

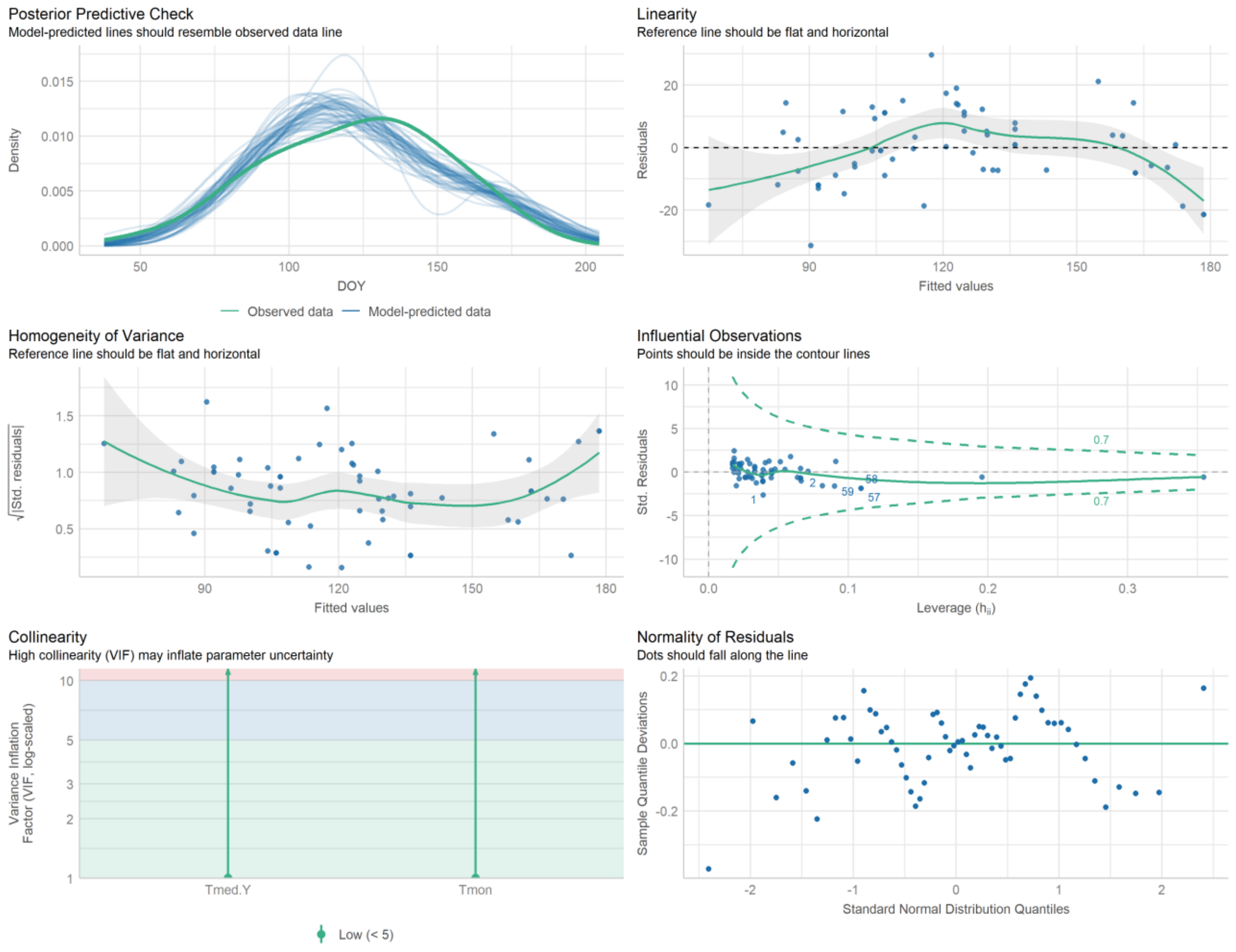

1.101. MLM - F - Quercus suber

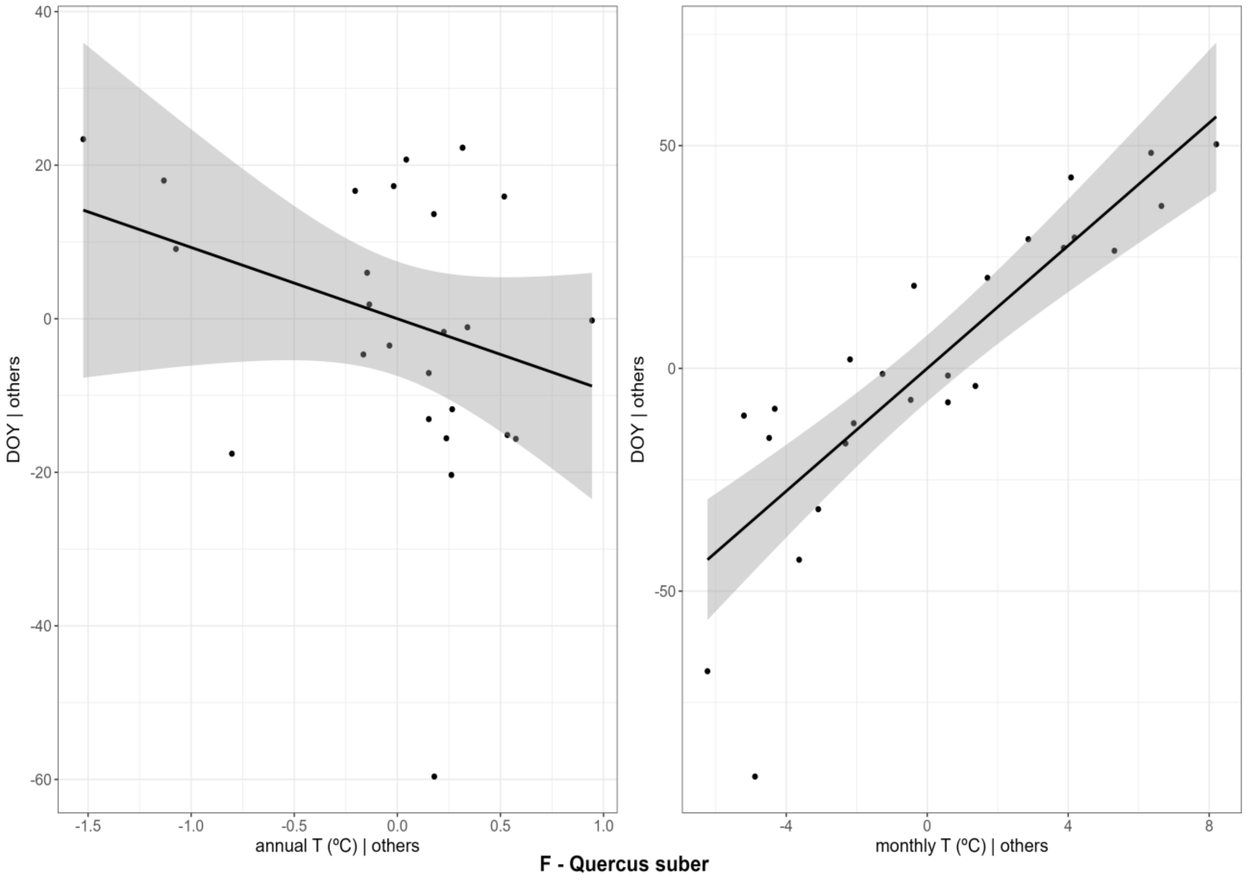

DOY = 166.54 (-9.28*annual T (ºC)) + (+6.89*monthly T (ºC))

1.101.1. Diagnostics - MLM - F - Quercus suber

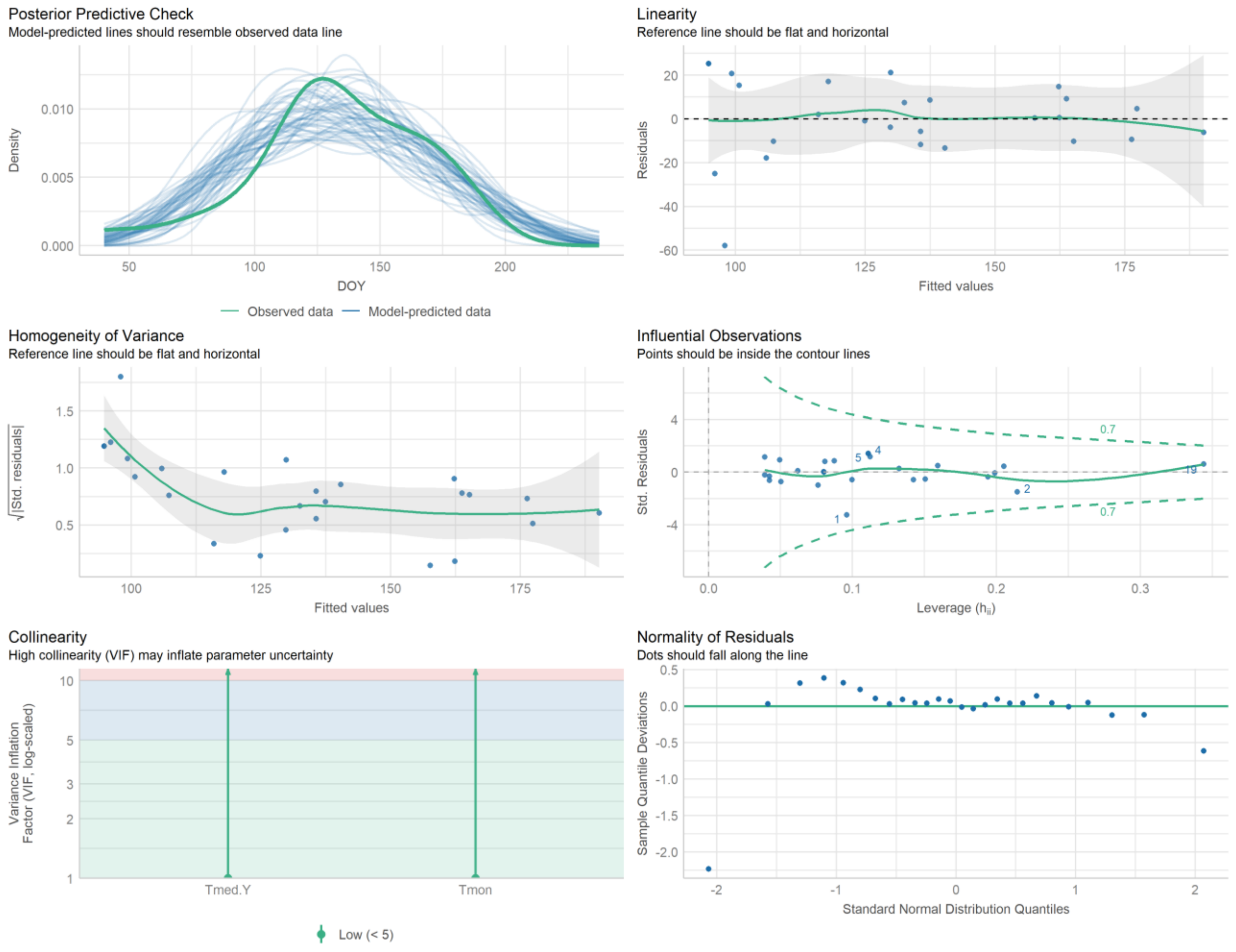

1.102. MLM - DVG - Quercus suber

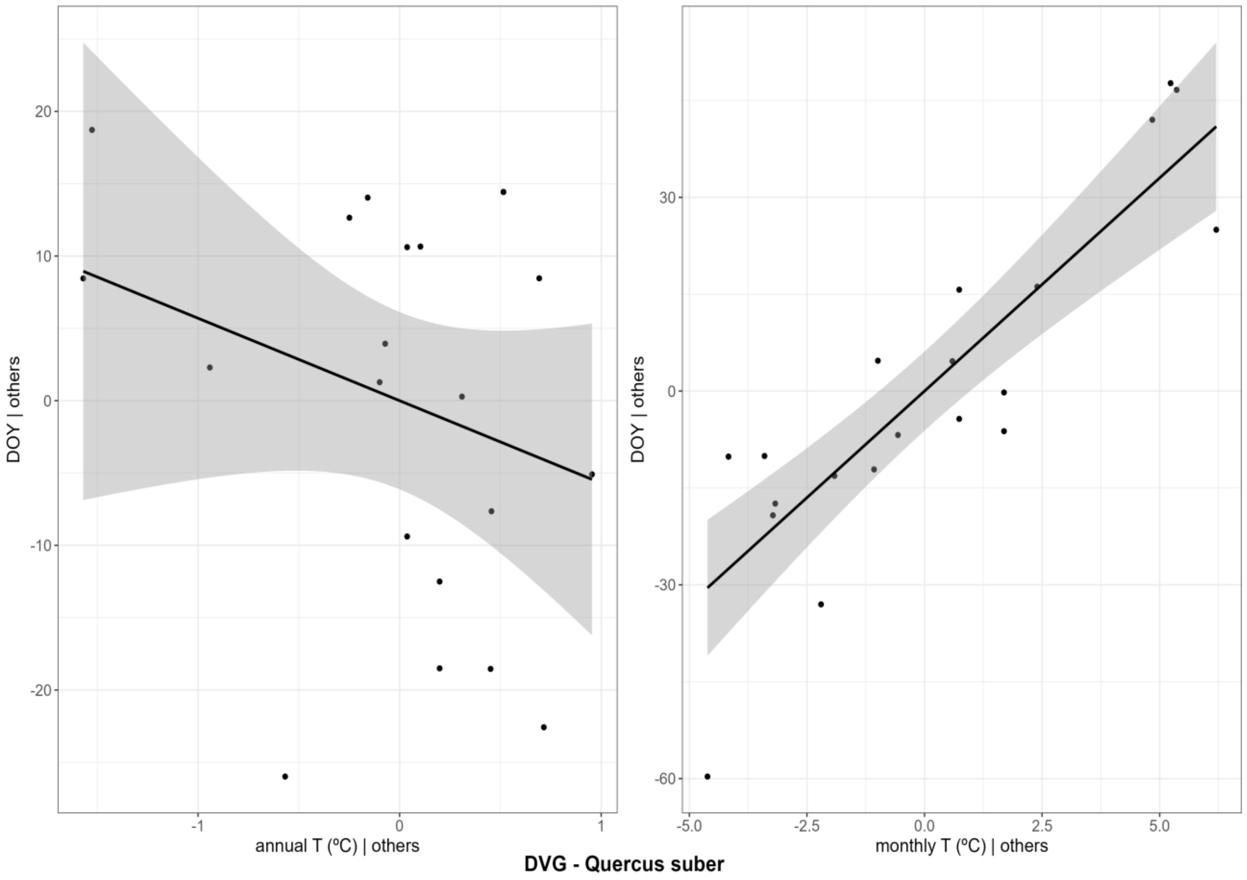

DOY = 116.97 (-5.70*annual T (ºC)) + (+6.60*monthly T (ºC))

1.102.1. Diagnostics - MLM - DVG - Quercus suber

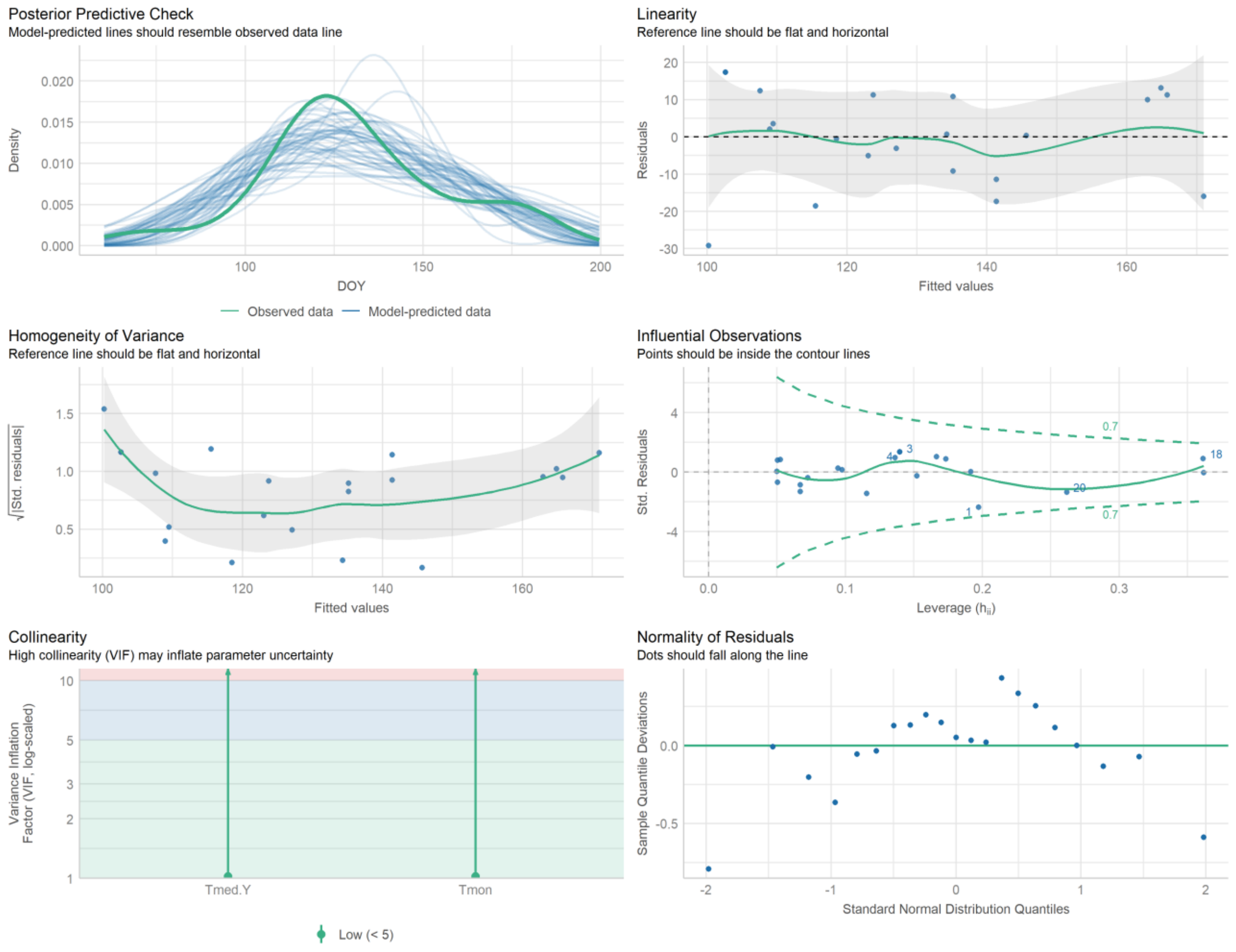

1.103. MLM - F - Retama sphaerocarpa

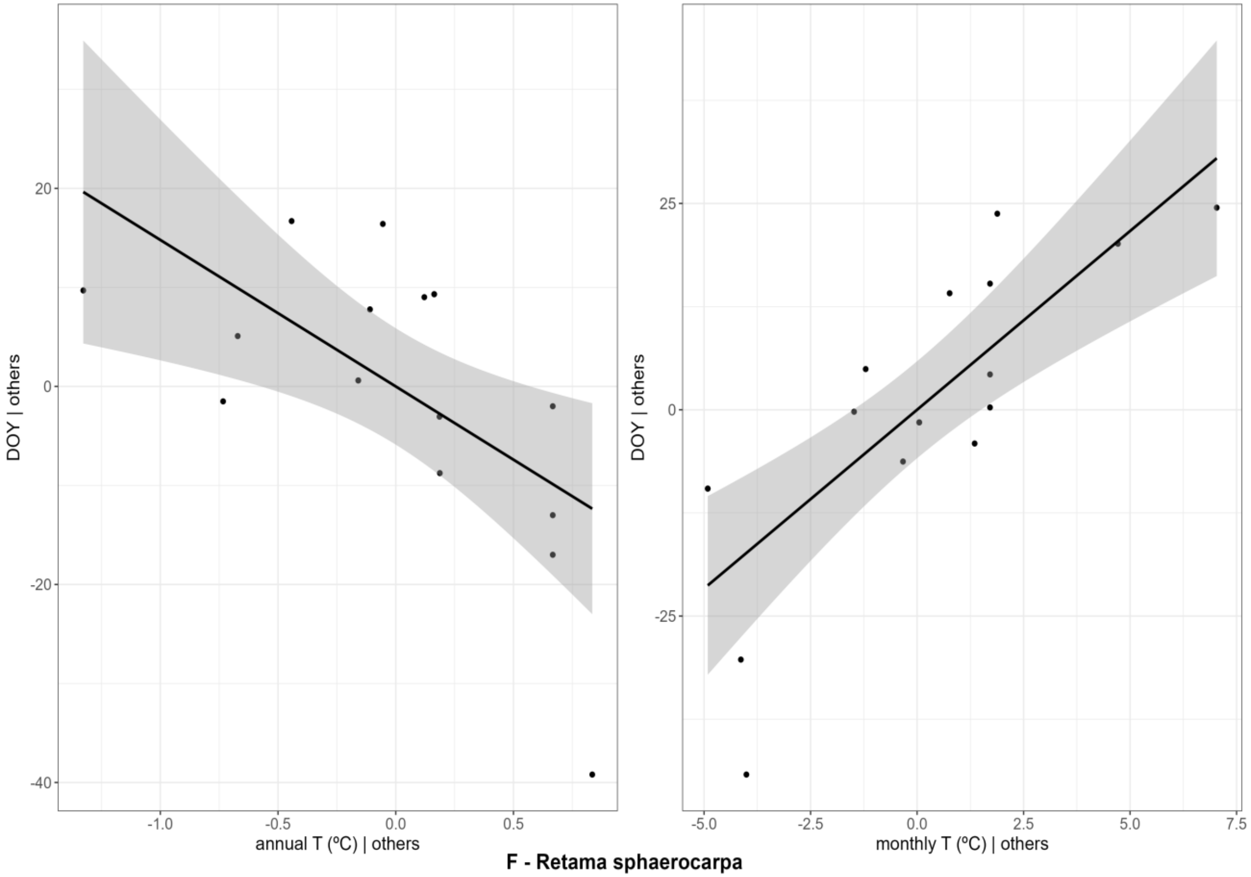

DOY = 305.72 (-14.79*annual T (ºC)) + (+4.33*monthly T (ºC))

1.103.1. Diagnostics - MLM - F - Retama sphaerocarpa

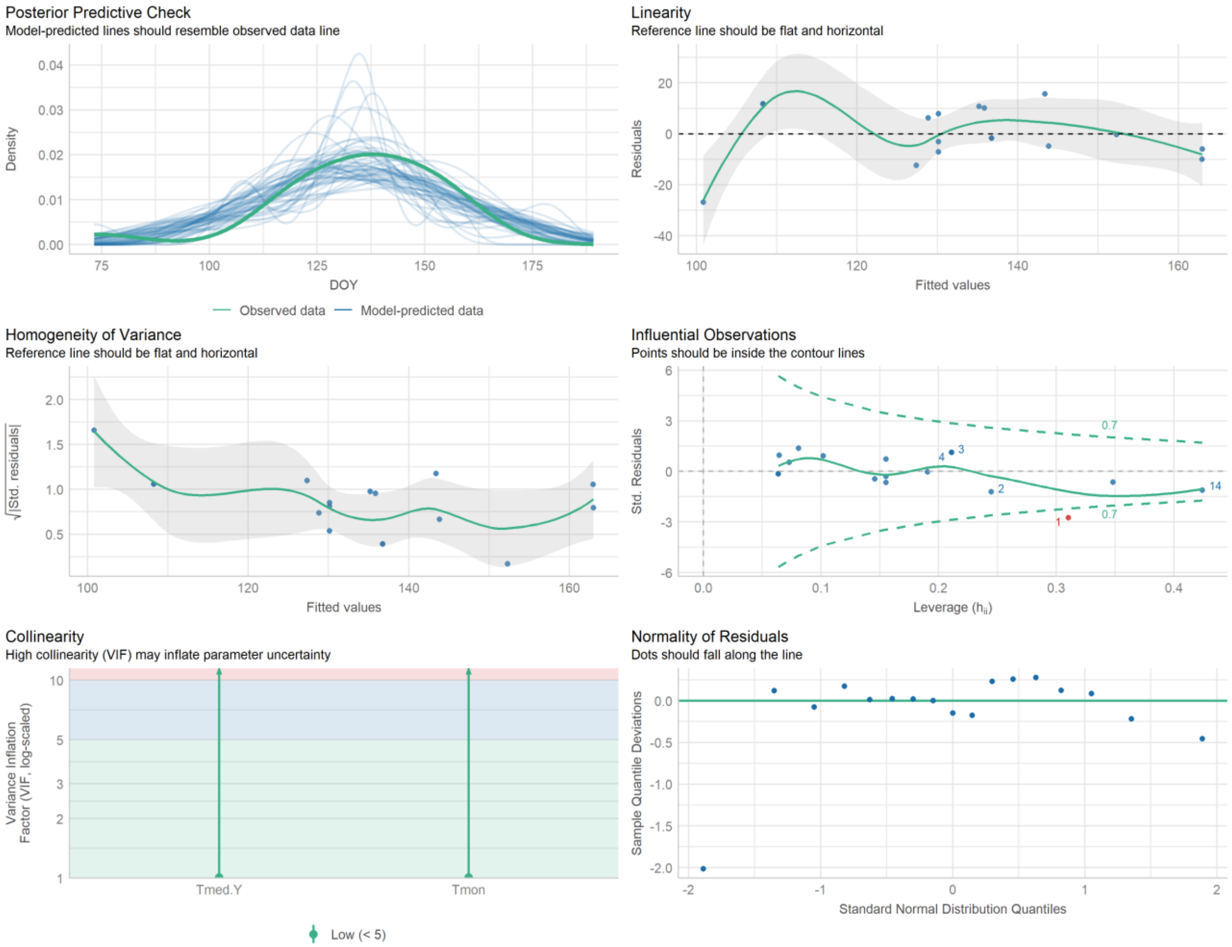

1.104. MLM - FBF - Rhamnus alaternus

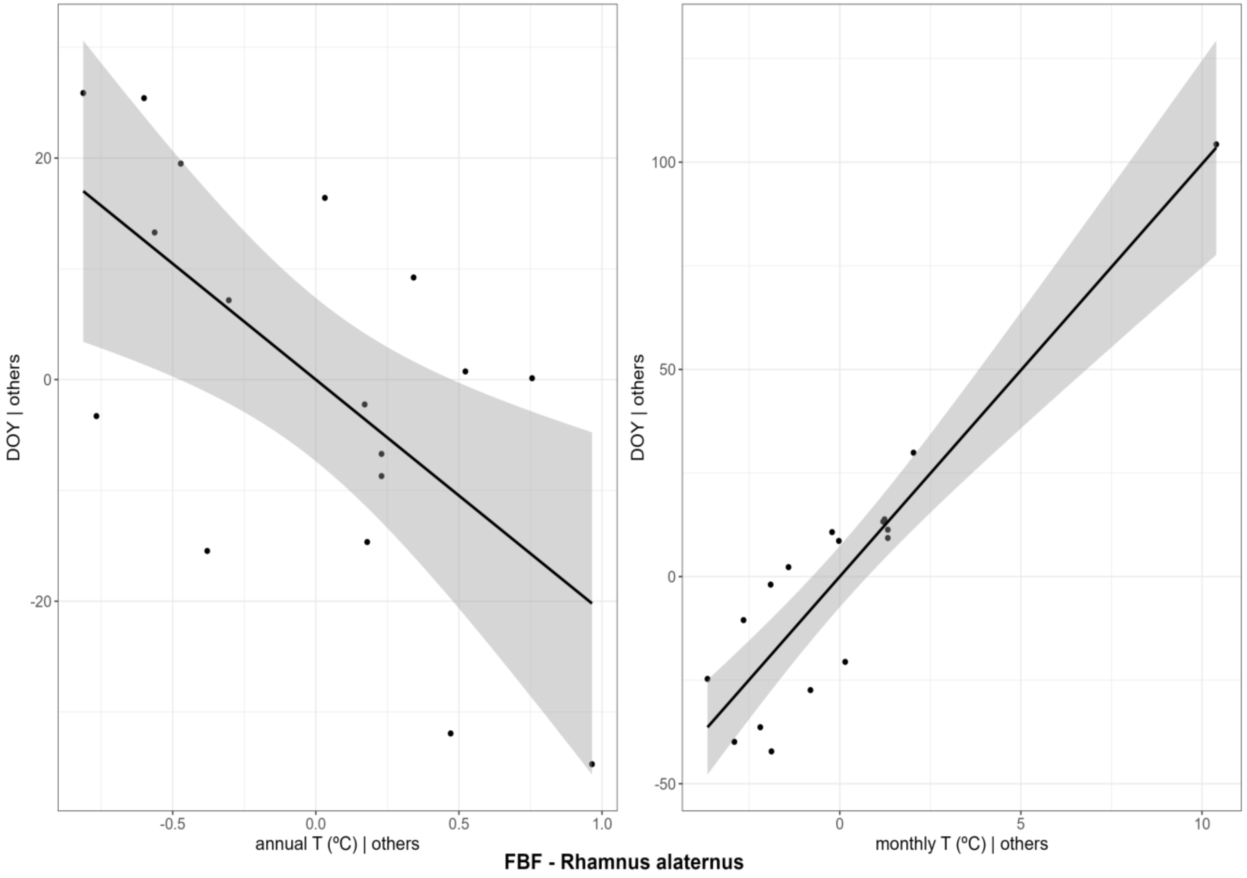

DOY = 287.96 (-20.93*annual T (ºC)) + (+9.96*monthly T (ºC))

1.104.1. Diagnostics - MLM - FBF - Rhamnus alaternus

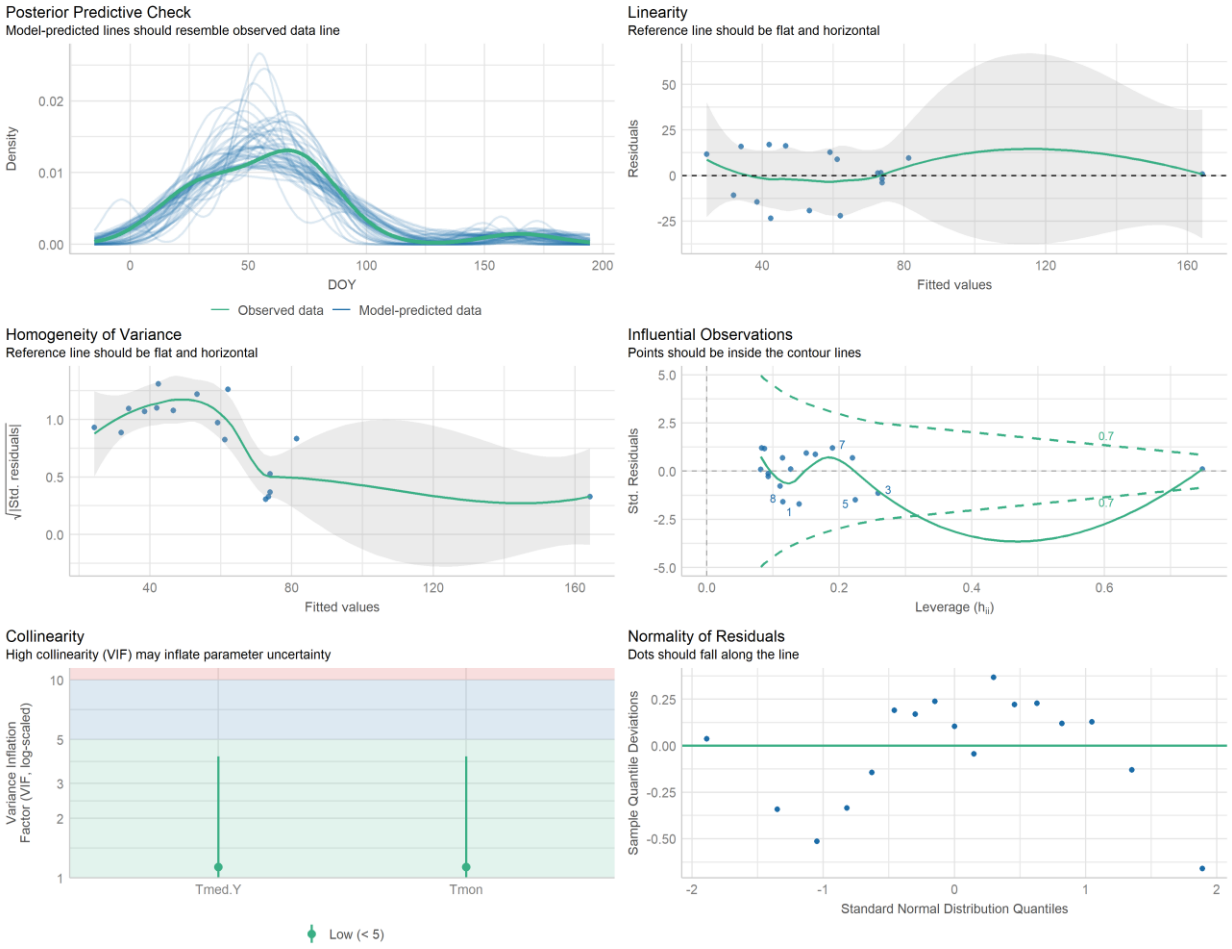

1.105. MLM - F - Rhamnus alaternus

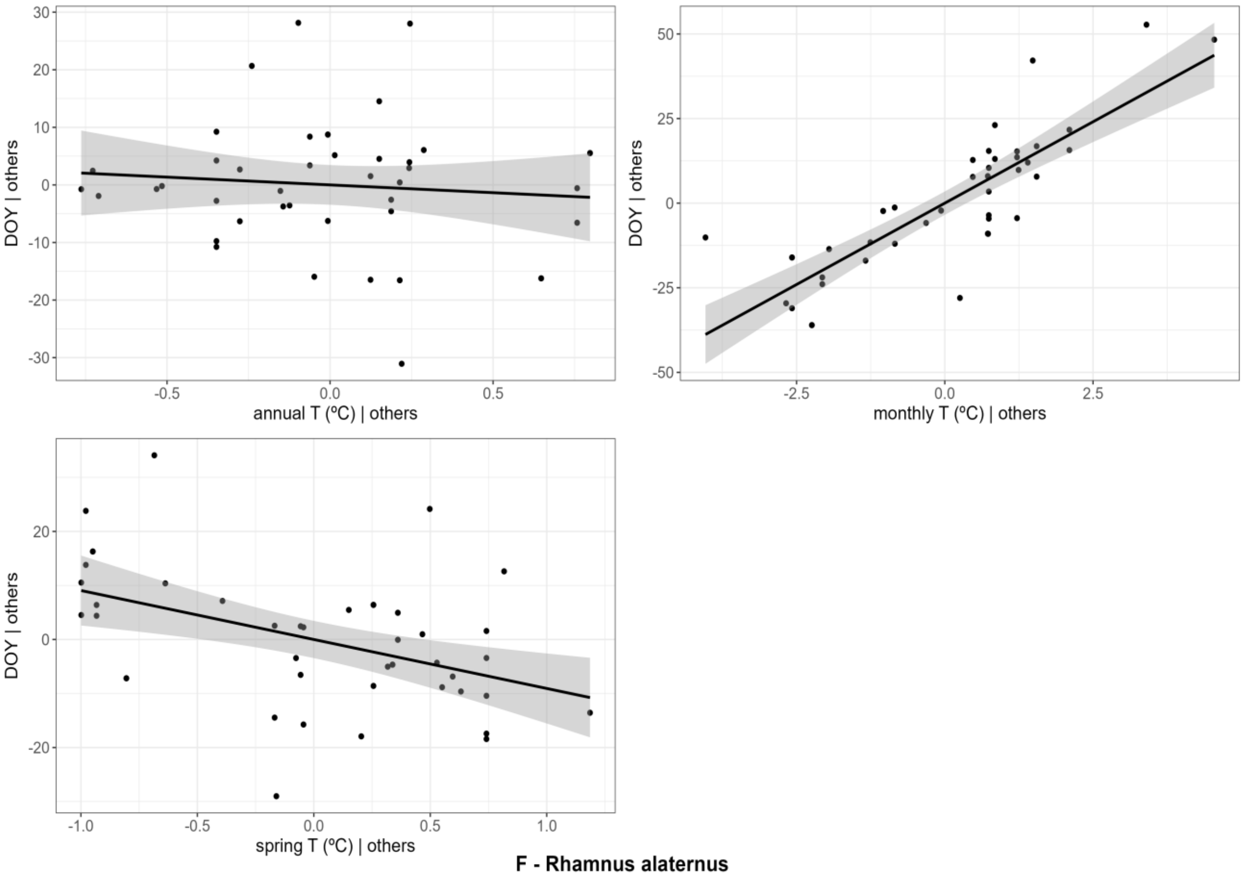

F - Rhamnus alaternus

DOY = 135.20 (-2.70*annual T (ºC)) + (+9.62*monthly T (ºC)) + (-9.07*spring T (ºC))

1.105.1. Diagnostics - MLM - F - Rhamnus alaternus

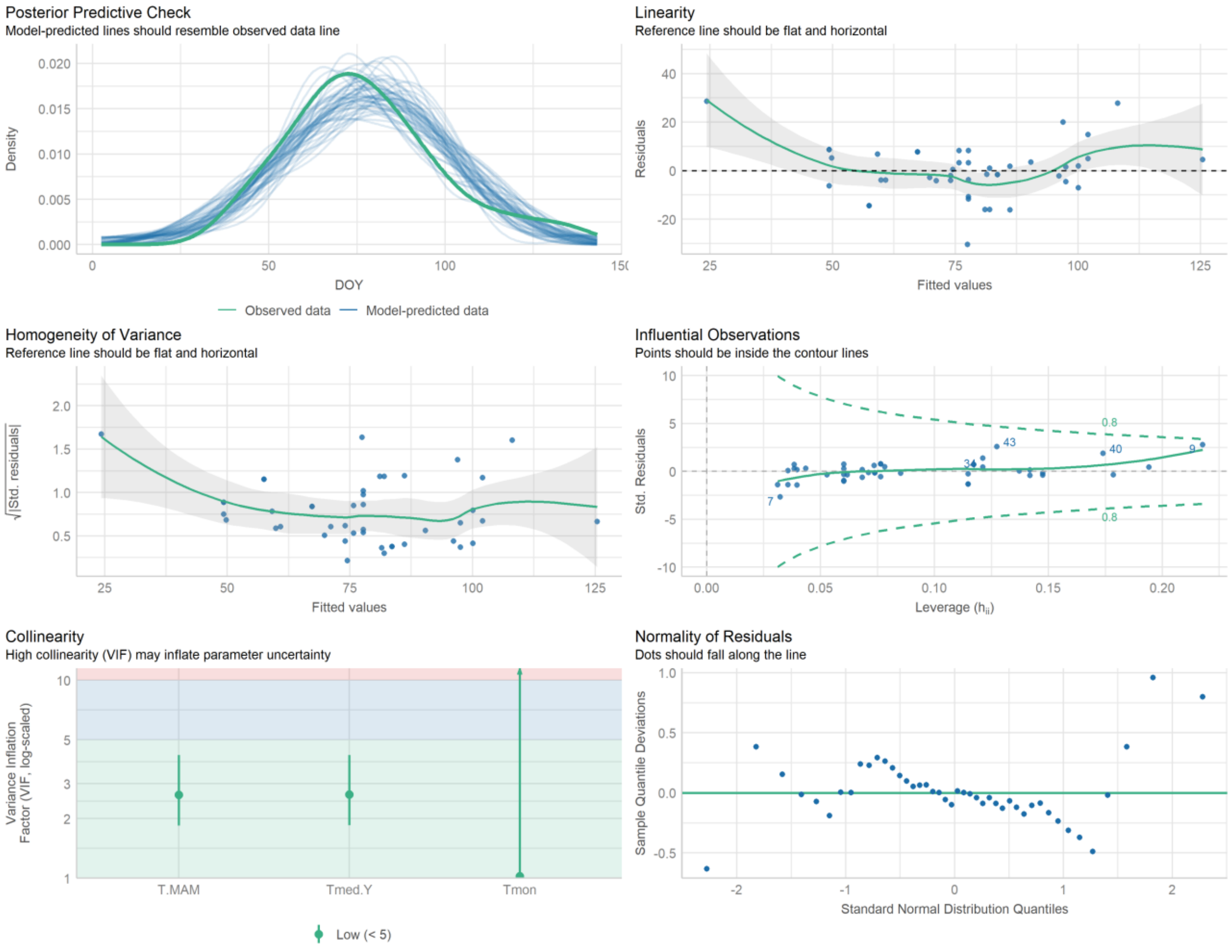

1.106. MLM - FS - Rhamnus alaternus

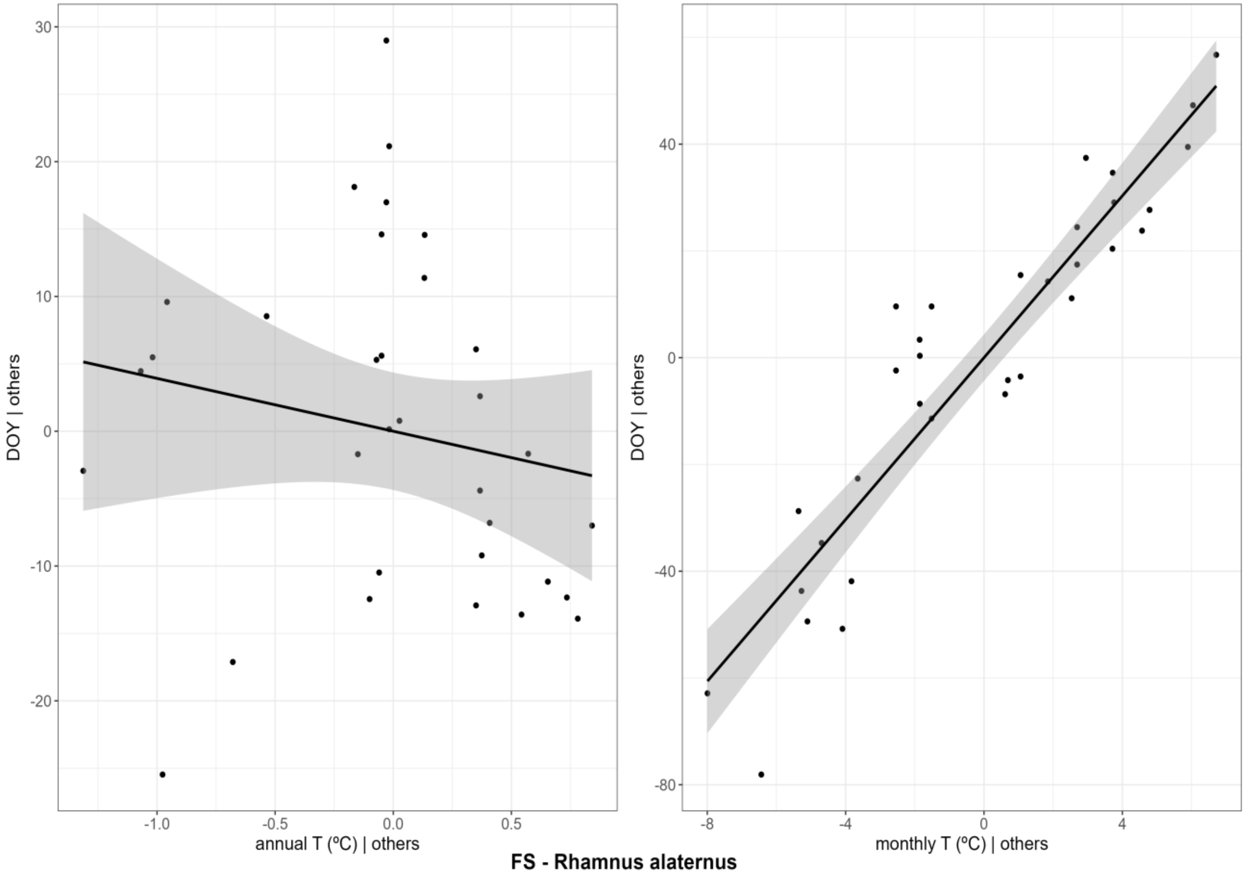

DOY = 61.26 (-3.92*annual T (ºC)) + (+7.58*monthly T (ºC))

1.106.1. Diagnostics - MLM - FS - Rhamnus alaternus

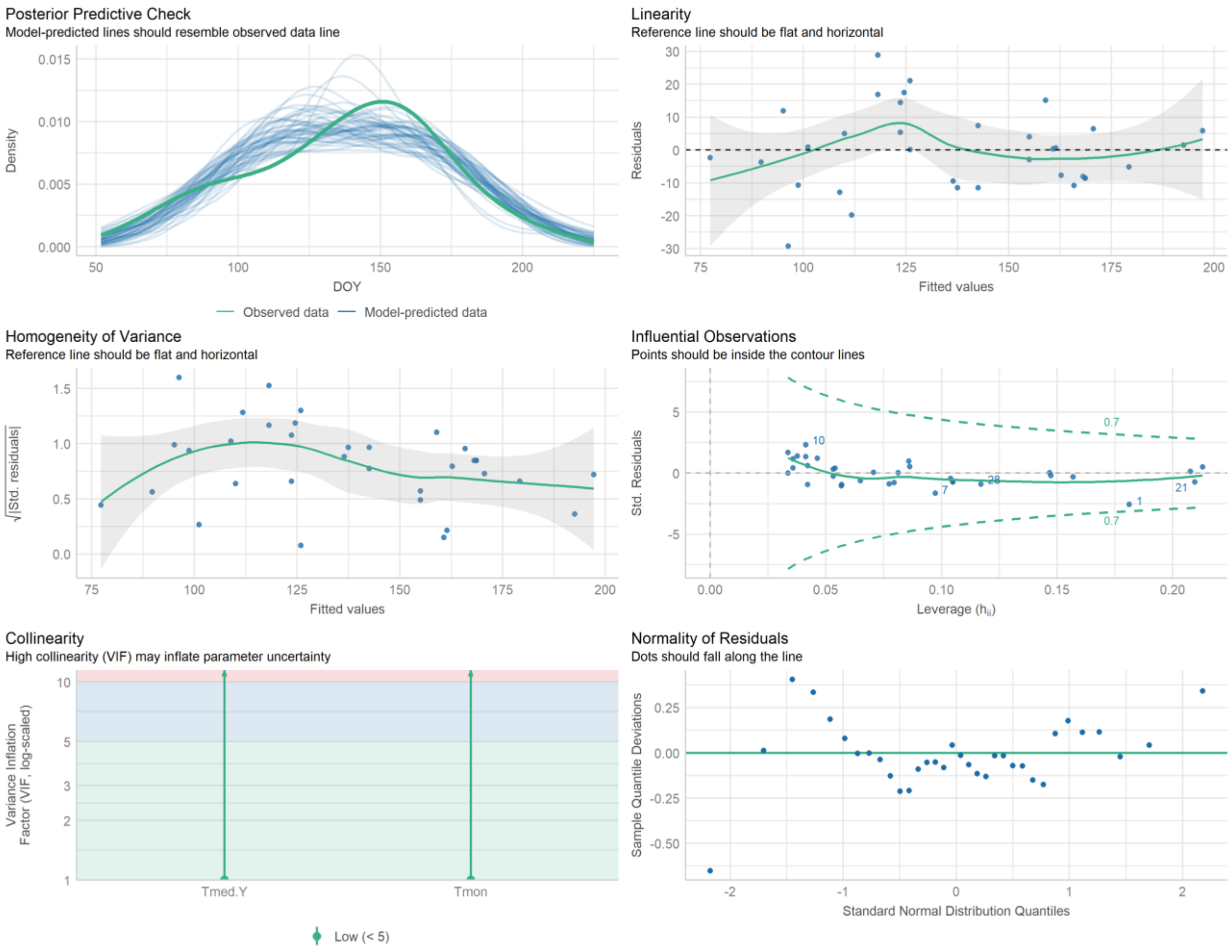

1.107. MLM - DVG - Rhamnus alaternus

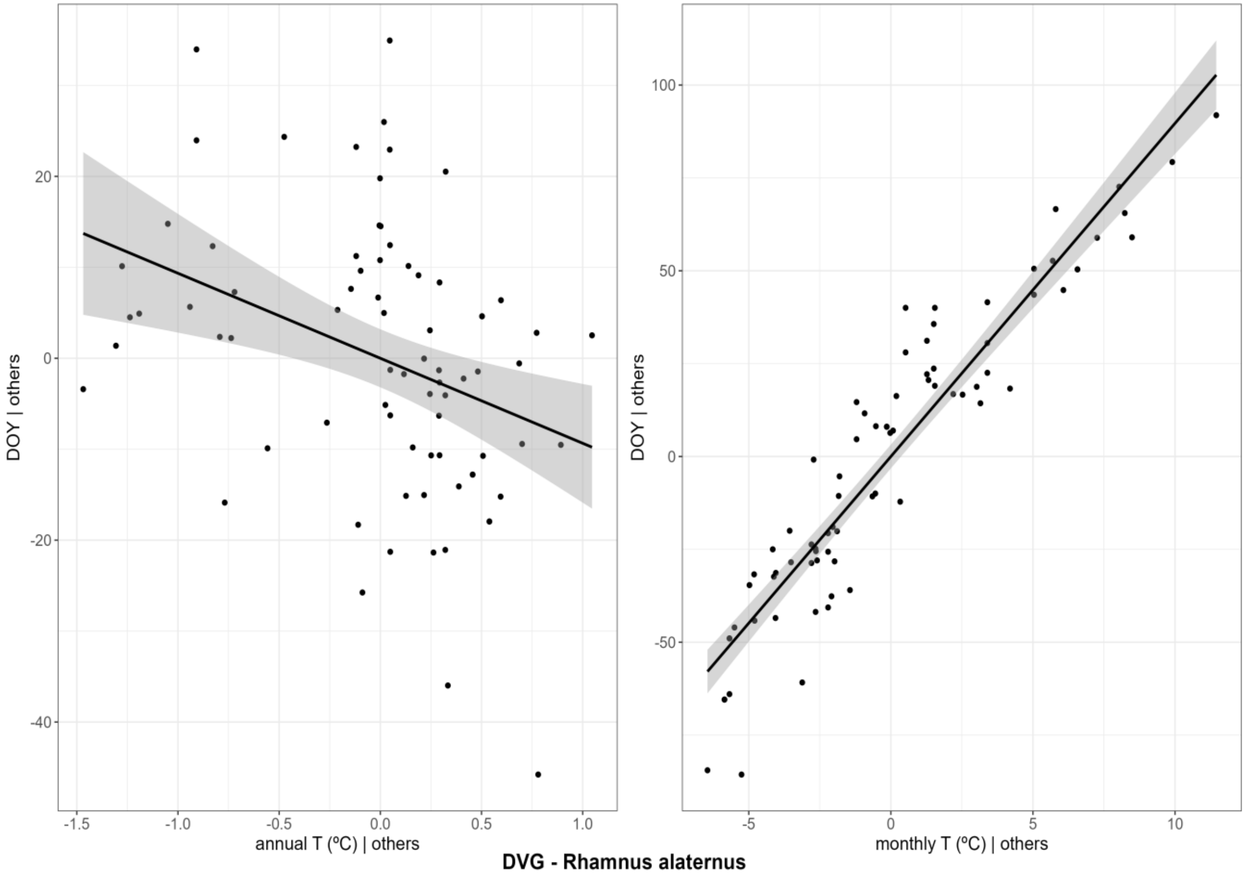

DOY = 122.85 (-9.35*annual T (ºC)) + (+8.97*monthly T (ºC))

1.107.1. Diagnostics - MLM - DVG - Rhamnus alaternus

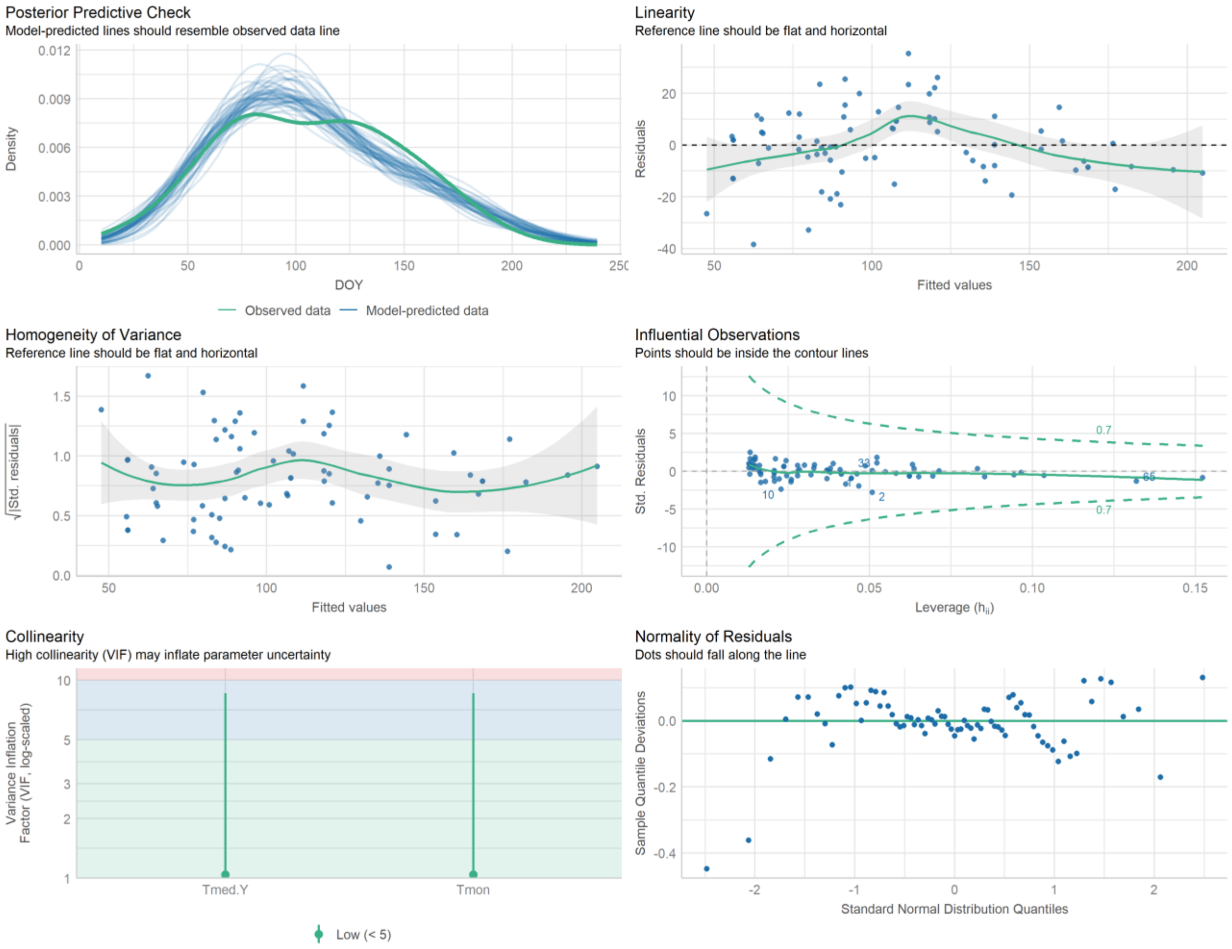

1.108. MLM - DVG - Rhamnus infectoria

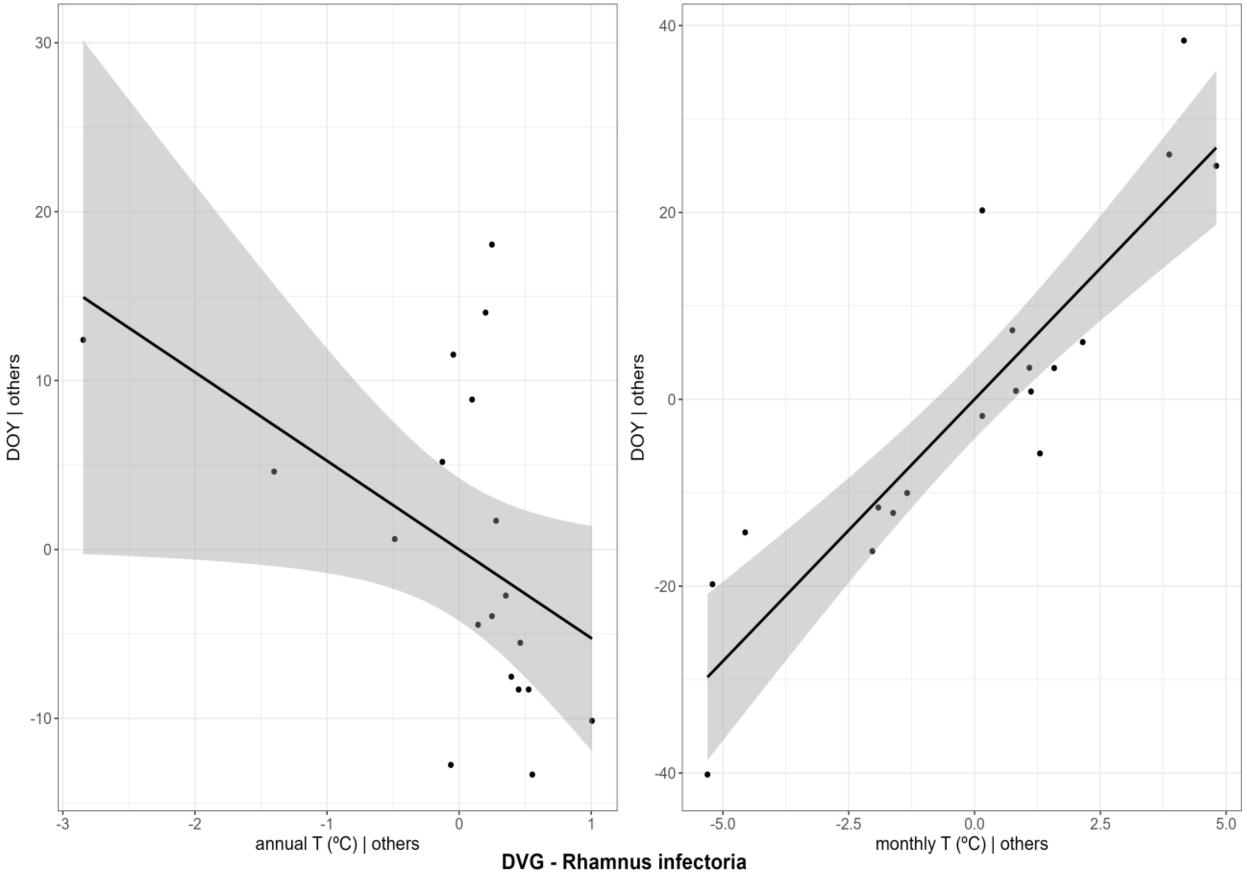

DOY = 126.69 (-5.25*annual T (ºC)) + (+5.61*monthly T (ºC))

1.108.1. Diagnostics - MLM - DVG - Rhamnus infectoria

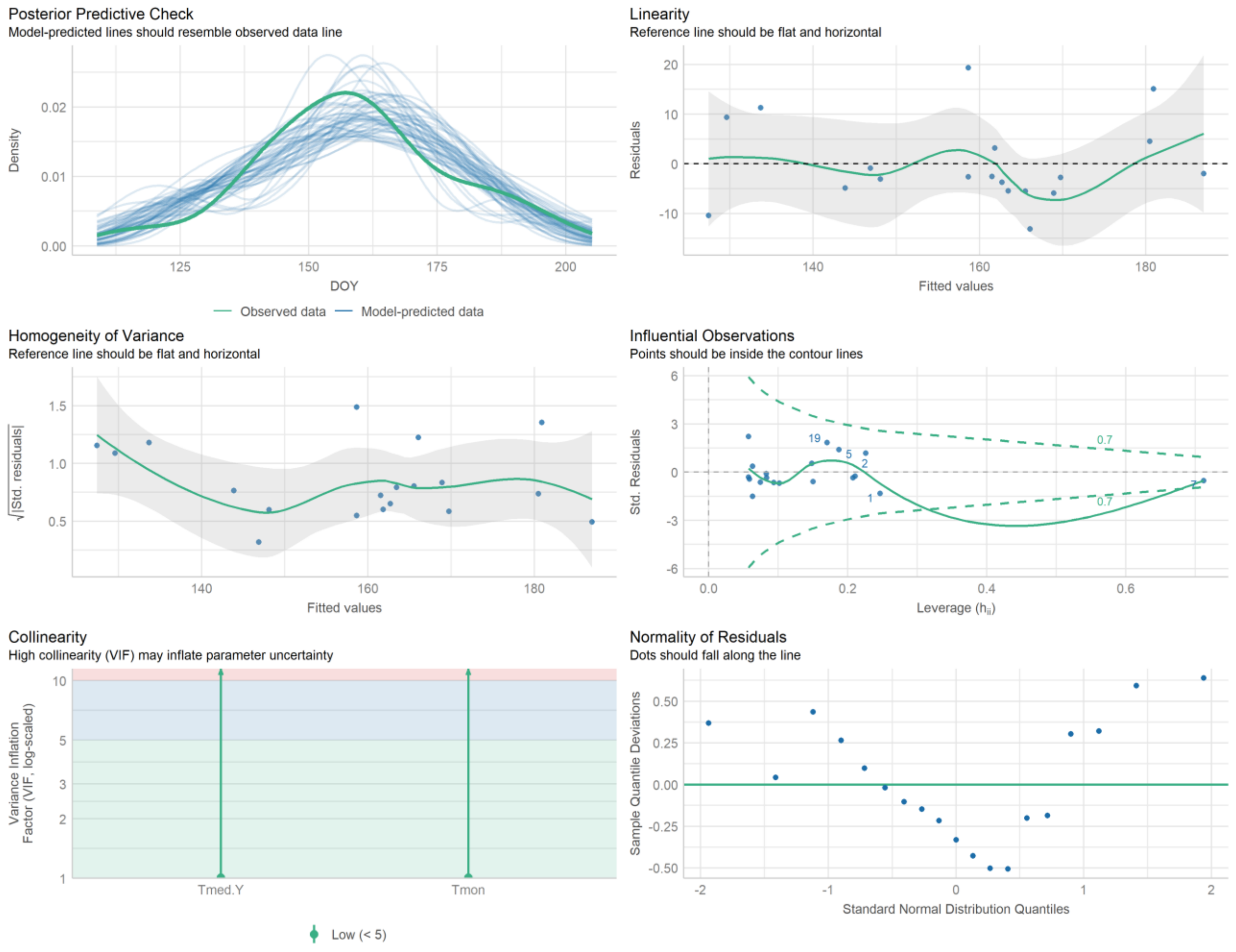

1.109. MLM - FBF - Rubia peregrina

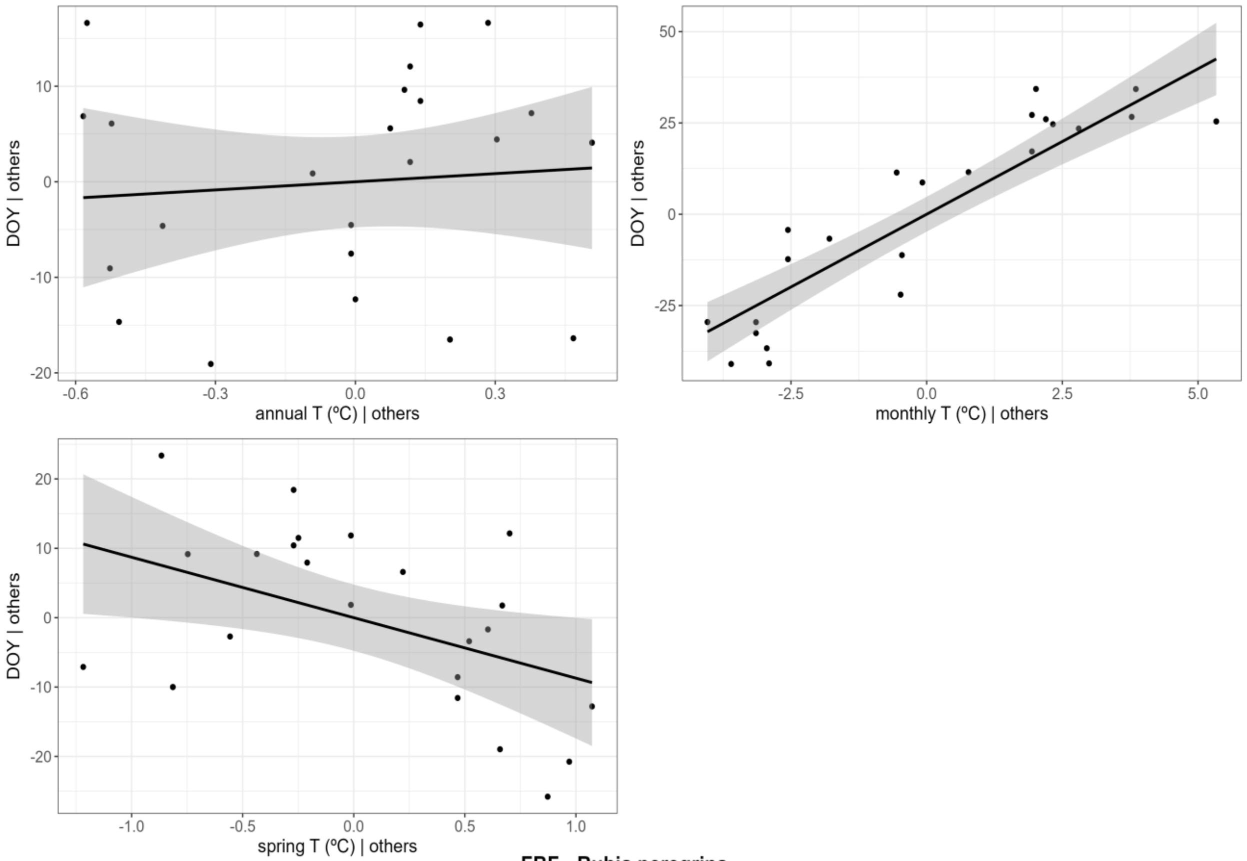

FBF - Rubia peregrina

DOY = 73.68 (+2.85*annual T (ºC)) + (+7.97*monthly T (ºC)) + (-8.72*spring T (ºC))

1.109.1. Diagnostics - MLM - FBF - Rubia peregrina

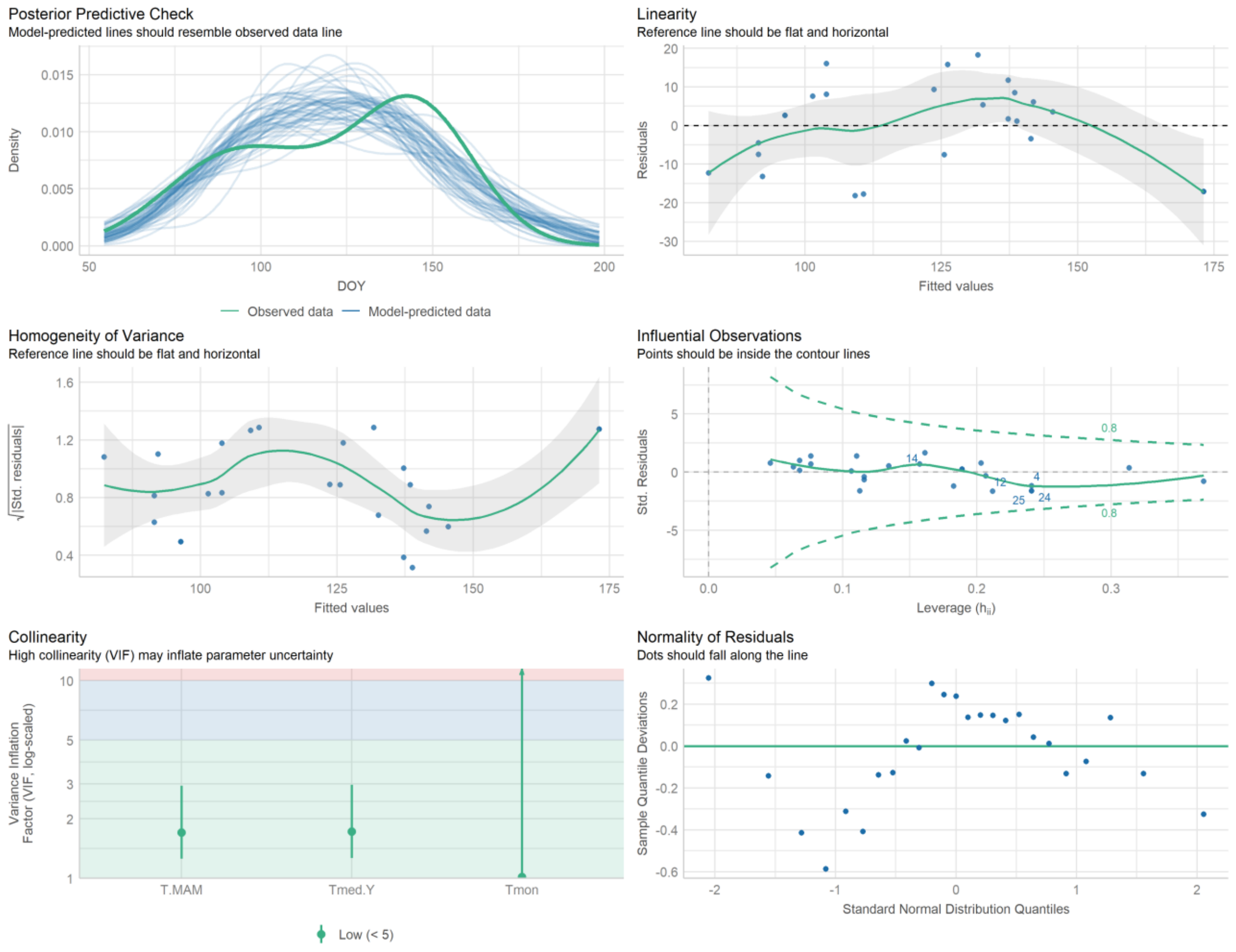

1.110. MLM - F - Rubia peregrina

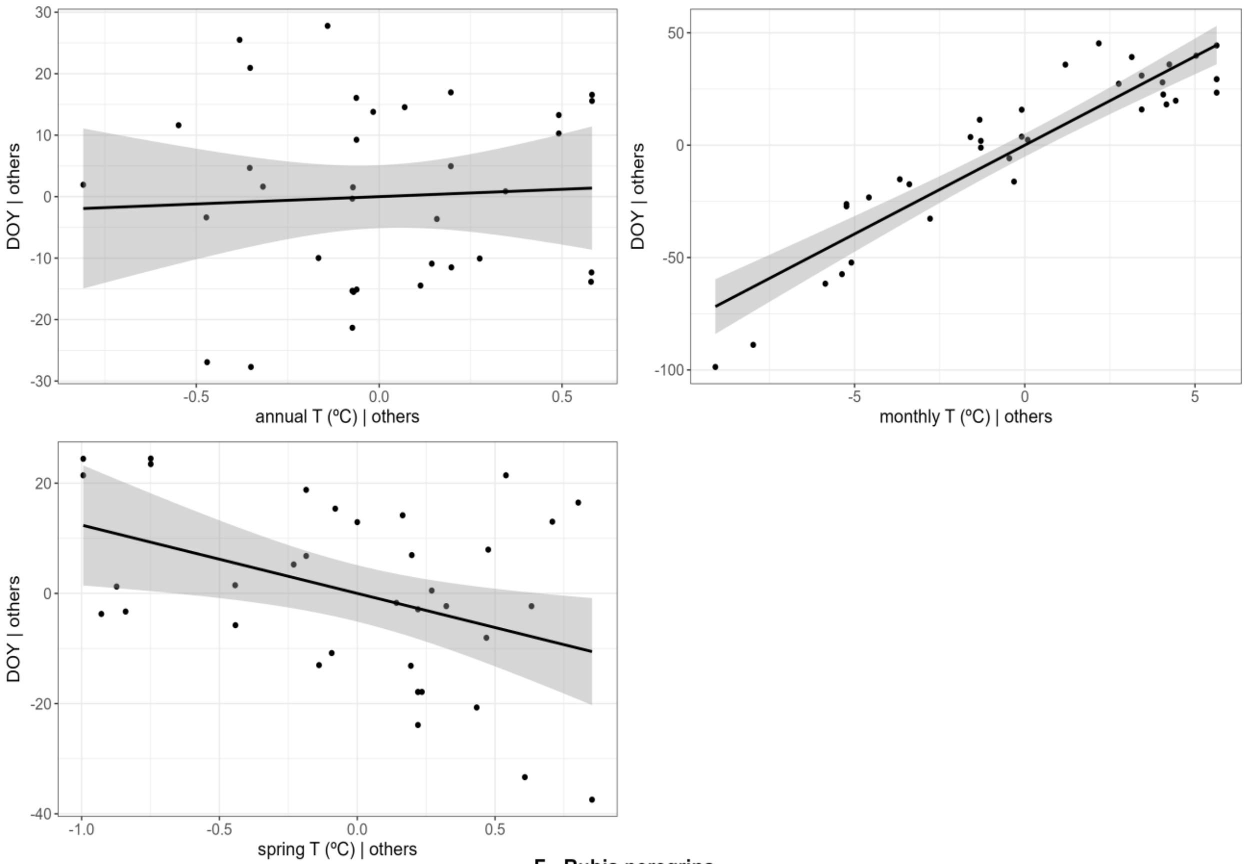

F - Rubia peregrina

DOY = 134.76 (+2.39*annual T (ºC)) + (+7.90*monthly T (ºC)) + (-12.40*spring T (ºC))

1.110.1. Diagnostics - MLM - F - Rubia peregrina

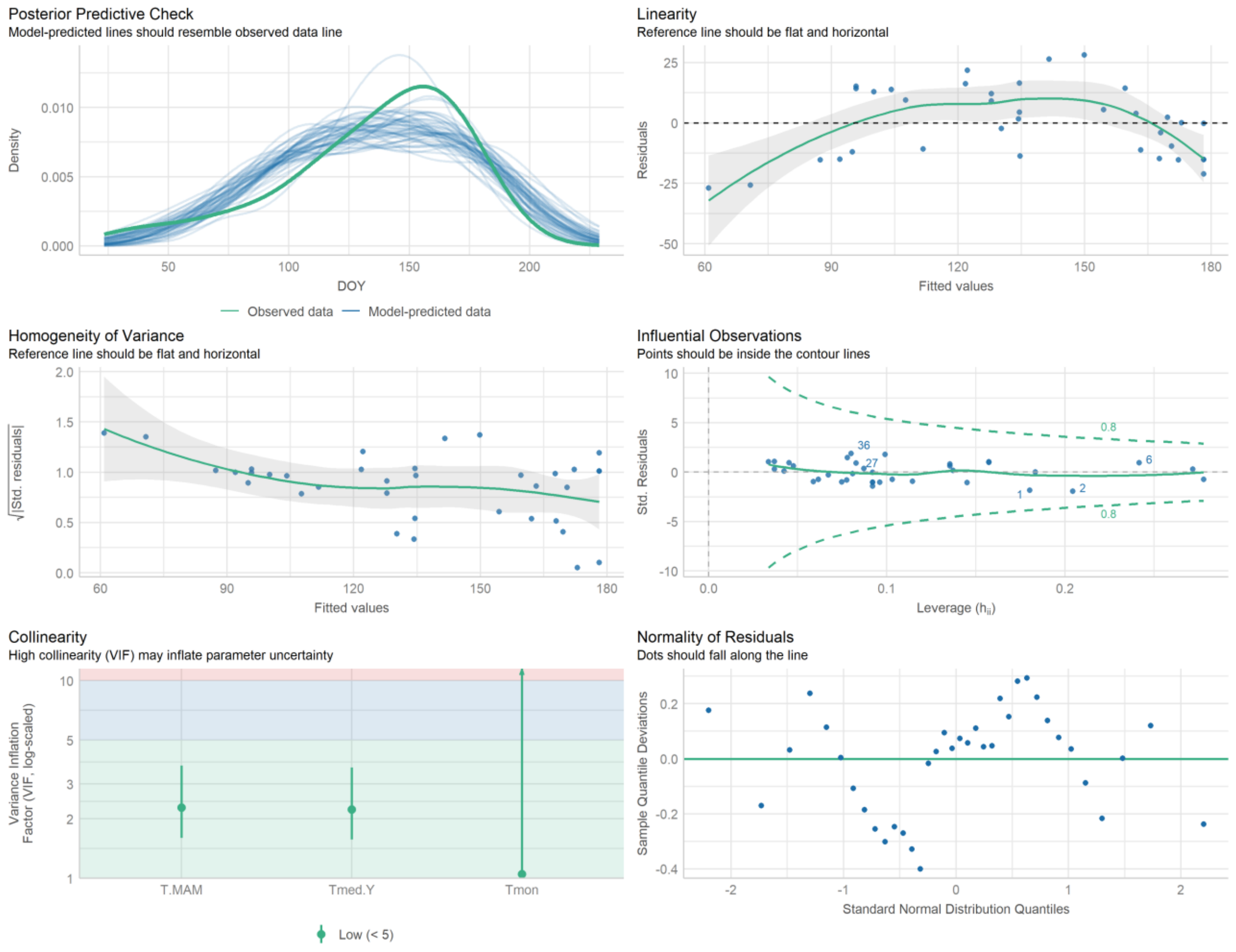

1.111. MLM - DVG - Rubia peregrina

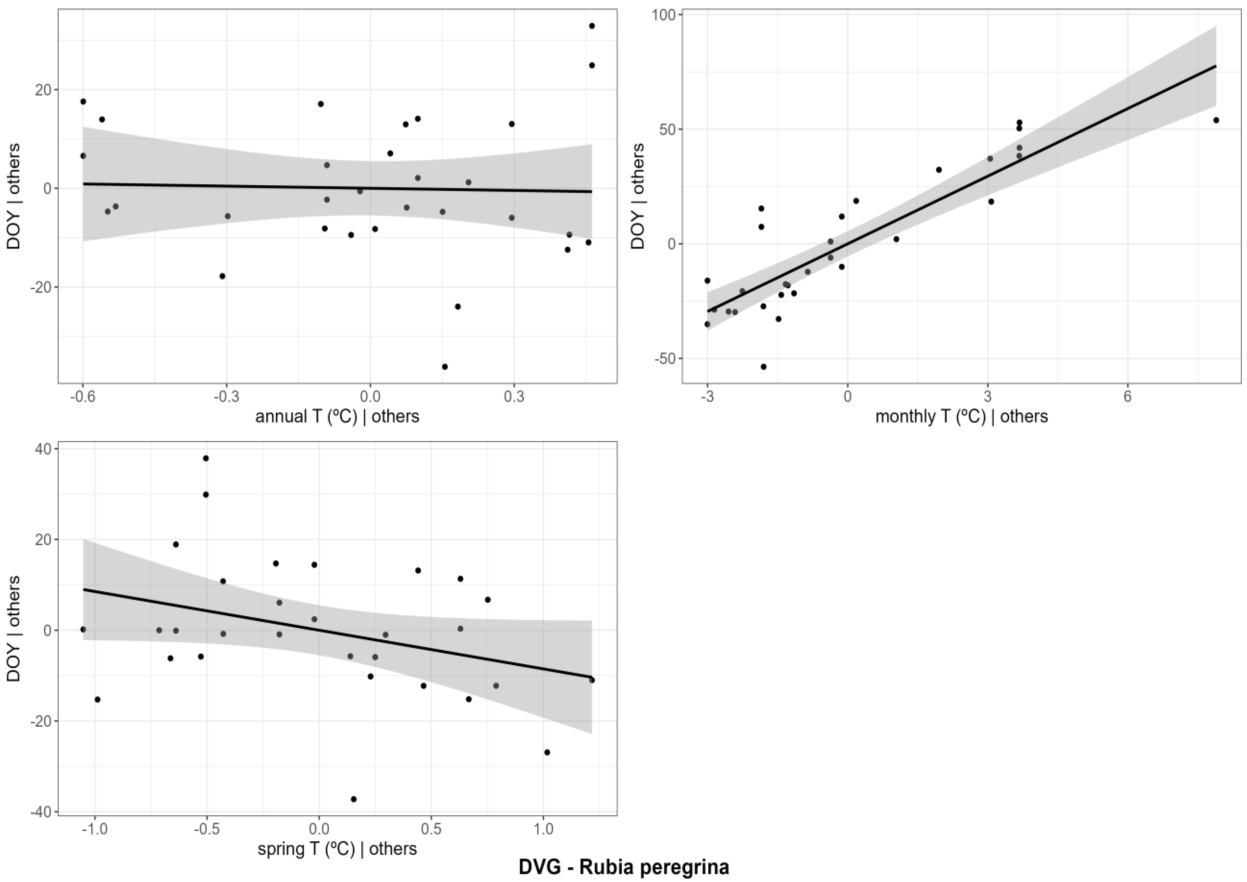

DOY = 108.76 (-1.45*annual T (ºC)) + (+9.84*monthly T (ºC)) + (-8.53*spring T (ºC))

1.111.1. Diagnostics - MLM - DVG - Rubia peregrina

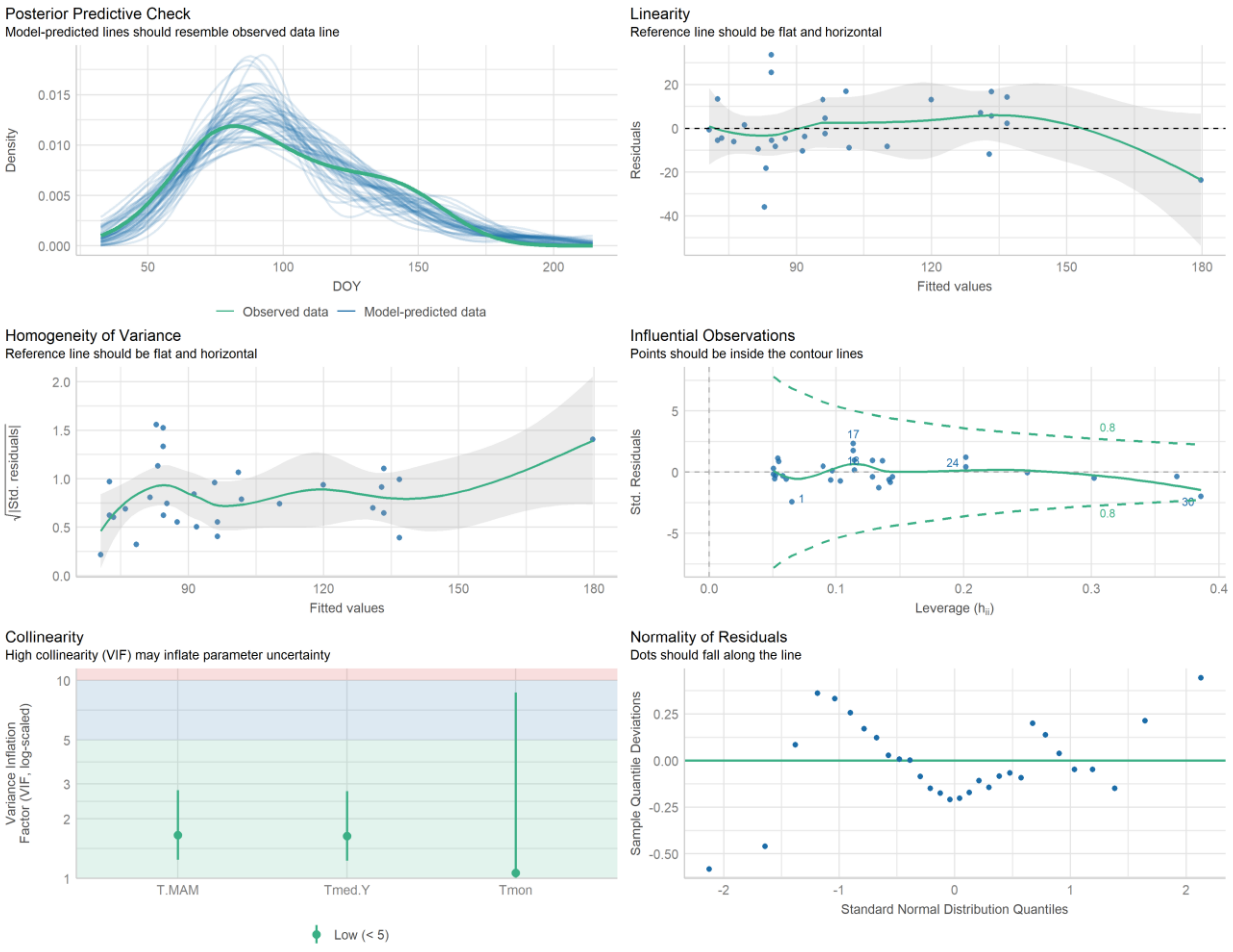

1.112. MLM - FBF - Salvia candelabrum

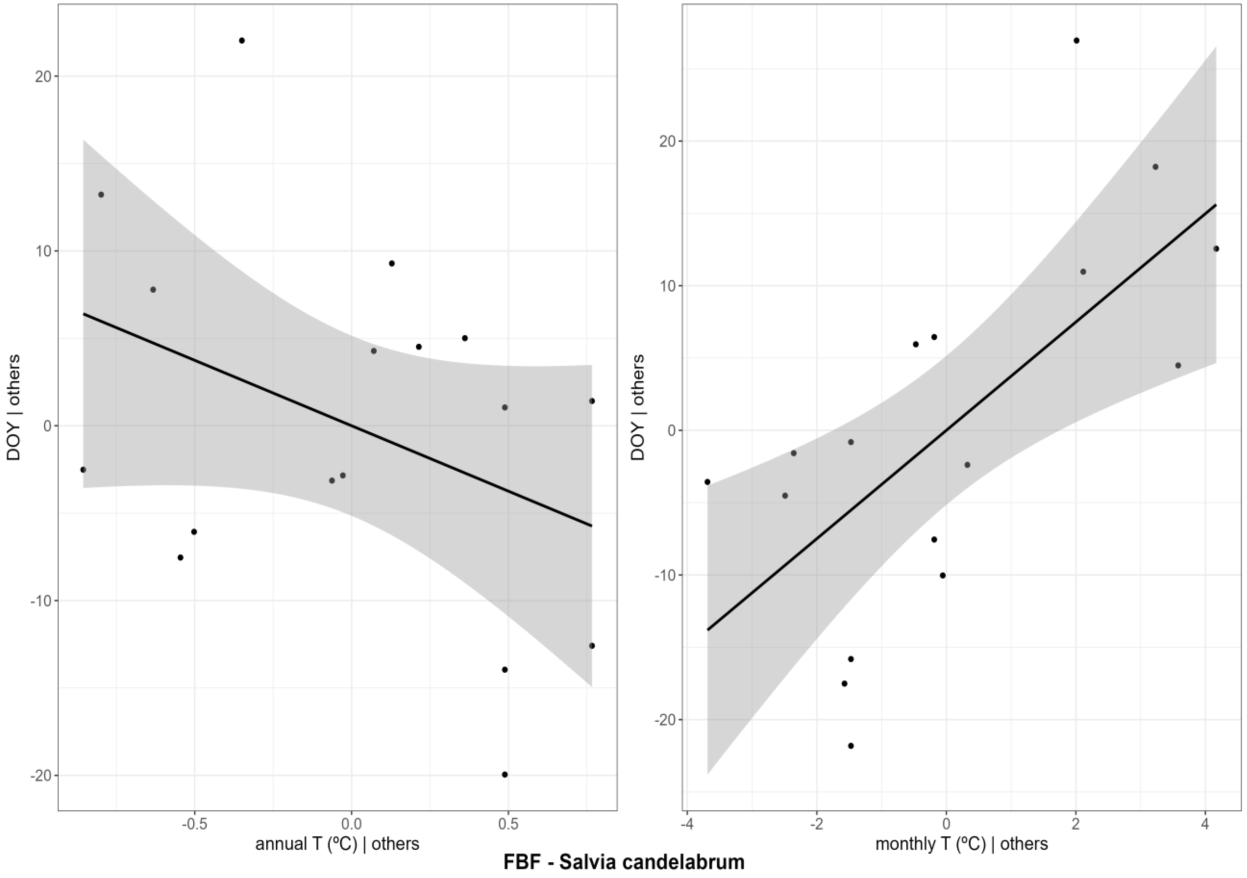

DOY = 196.19 (-7.48*annual T (ºC)) + (+3.75*monthly T (ºC))

1.112.1. Diagnostics - MLM - FBF - Salvia candelabrum

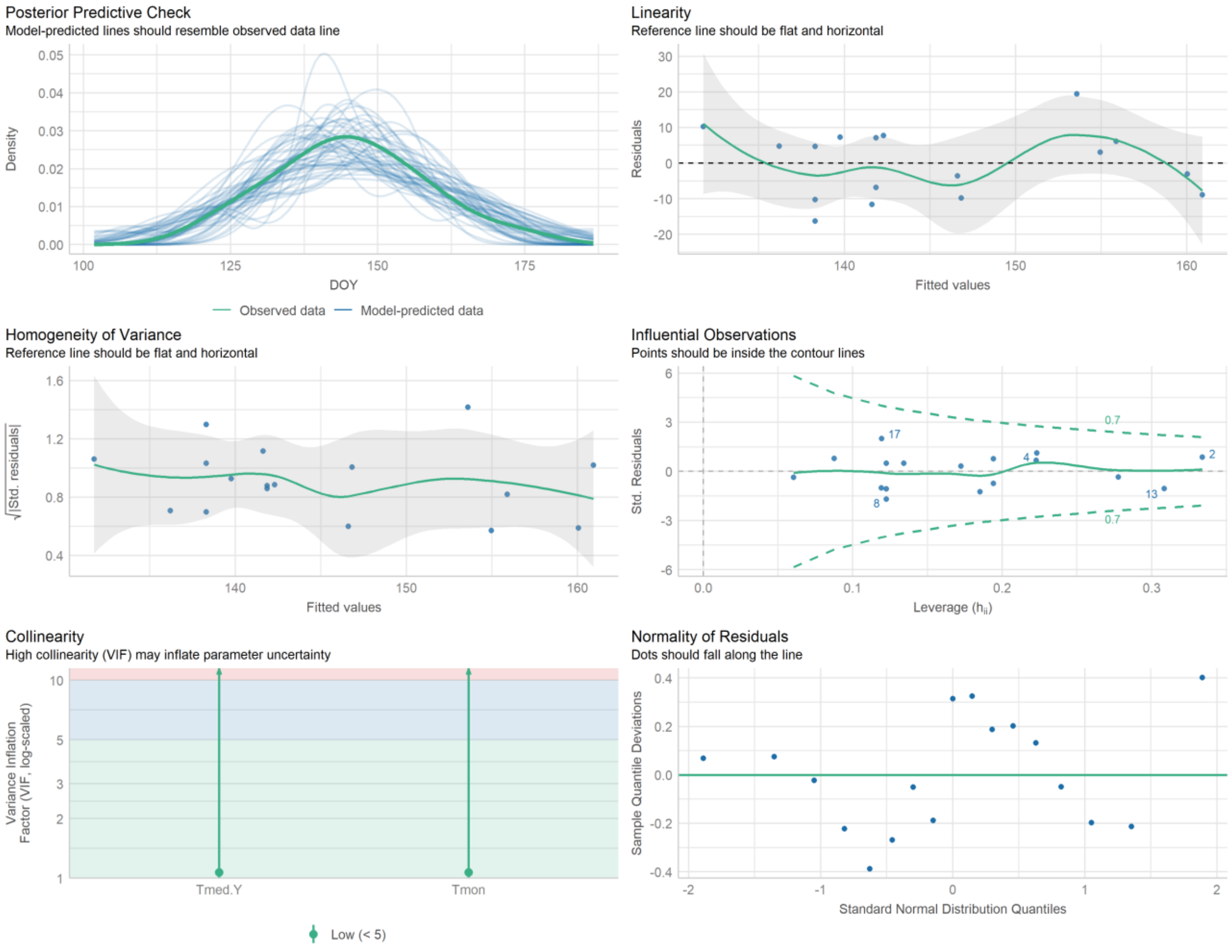

1.113. MLM - F - Salvia candelabrum

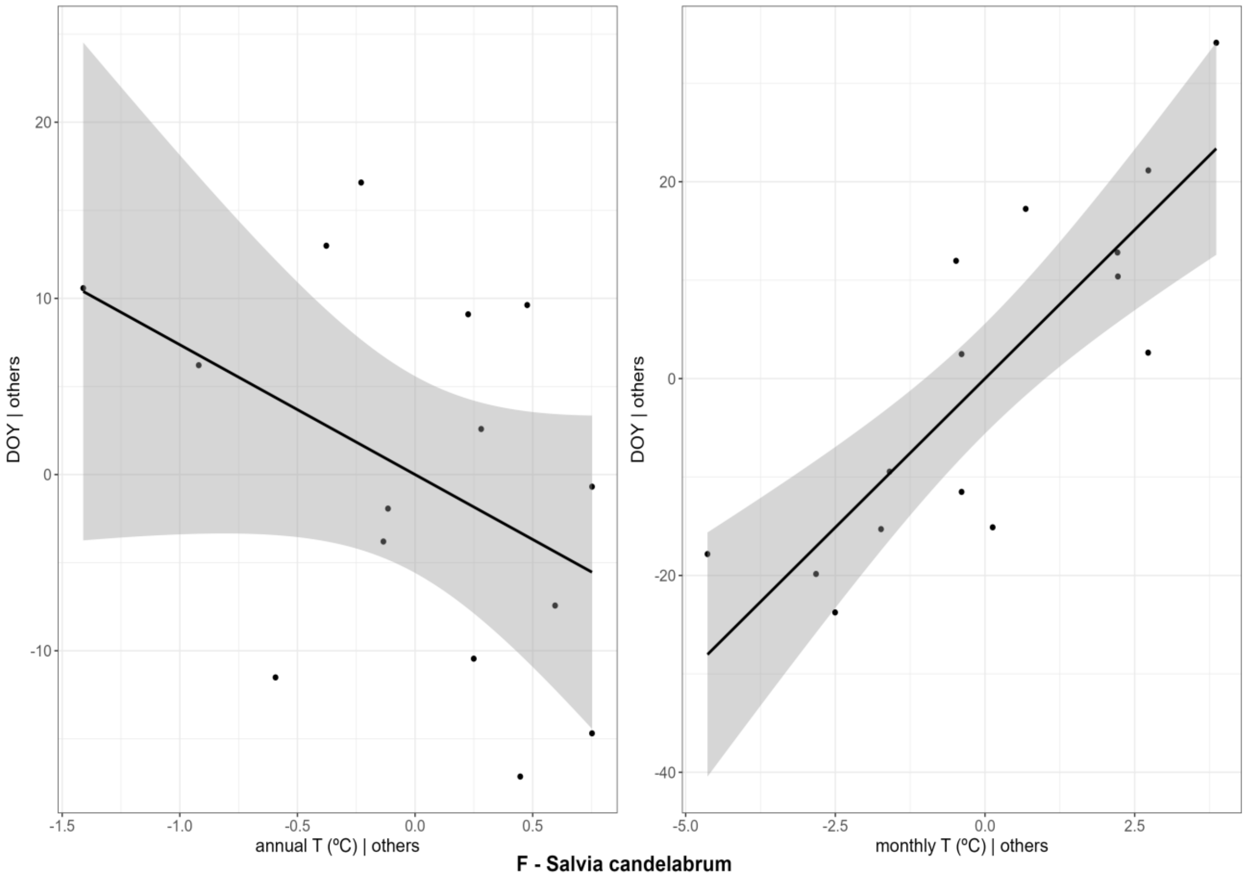

DOY = 148.92 (-7.37*annual T (ºC)) + (+6.05*monthly T (ºC))

1.113.1. Diagnostics - MLM - F - Salvia candelabrum

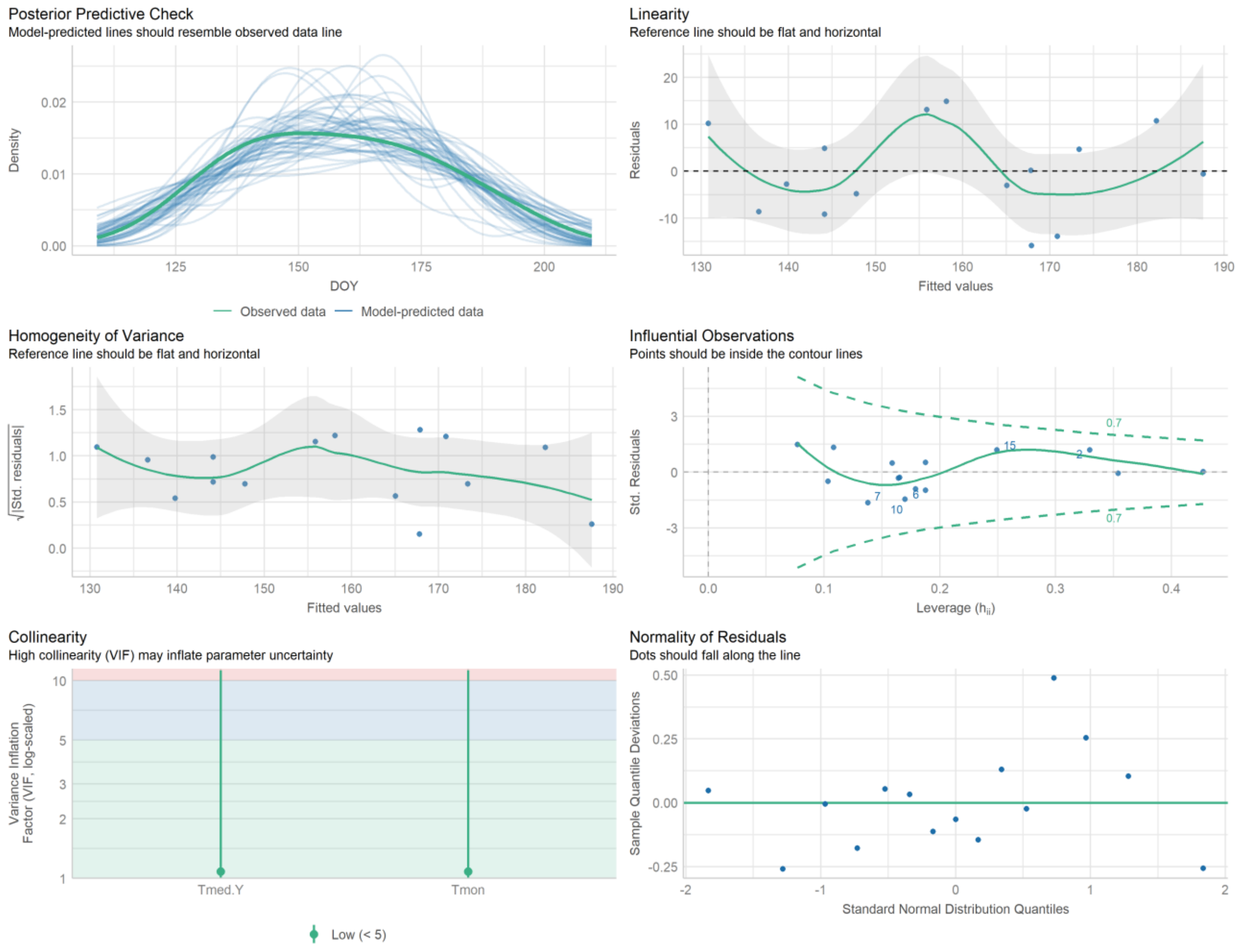

1.114. MLM - FBF - Salvia lavandulifolia

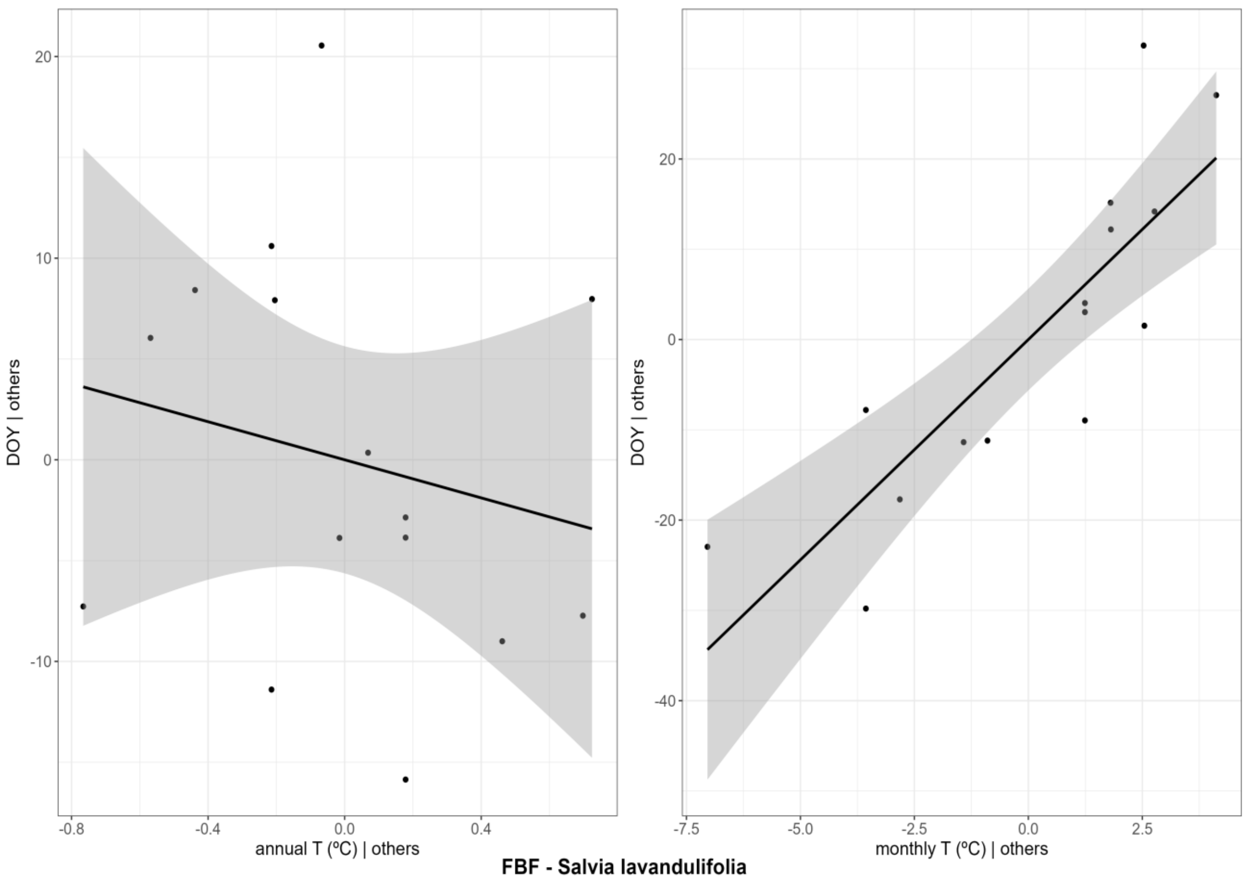

DOY = 140.07 (-4.72*annual T (ºC)) + (+4.88*monthly T (ºC))

1.114.1. Diagnostics - MLM - FBF - Salvia lavandulifolia

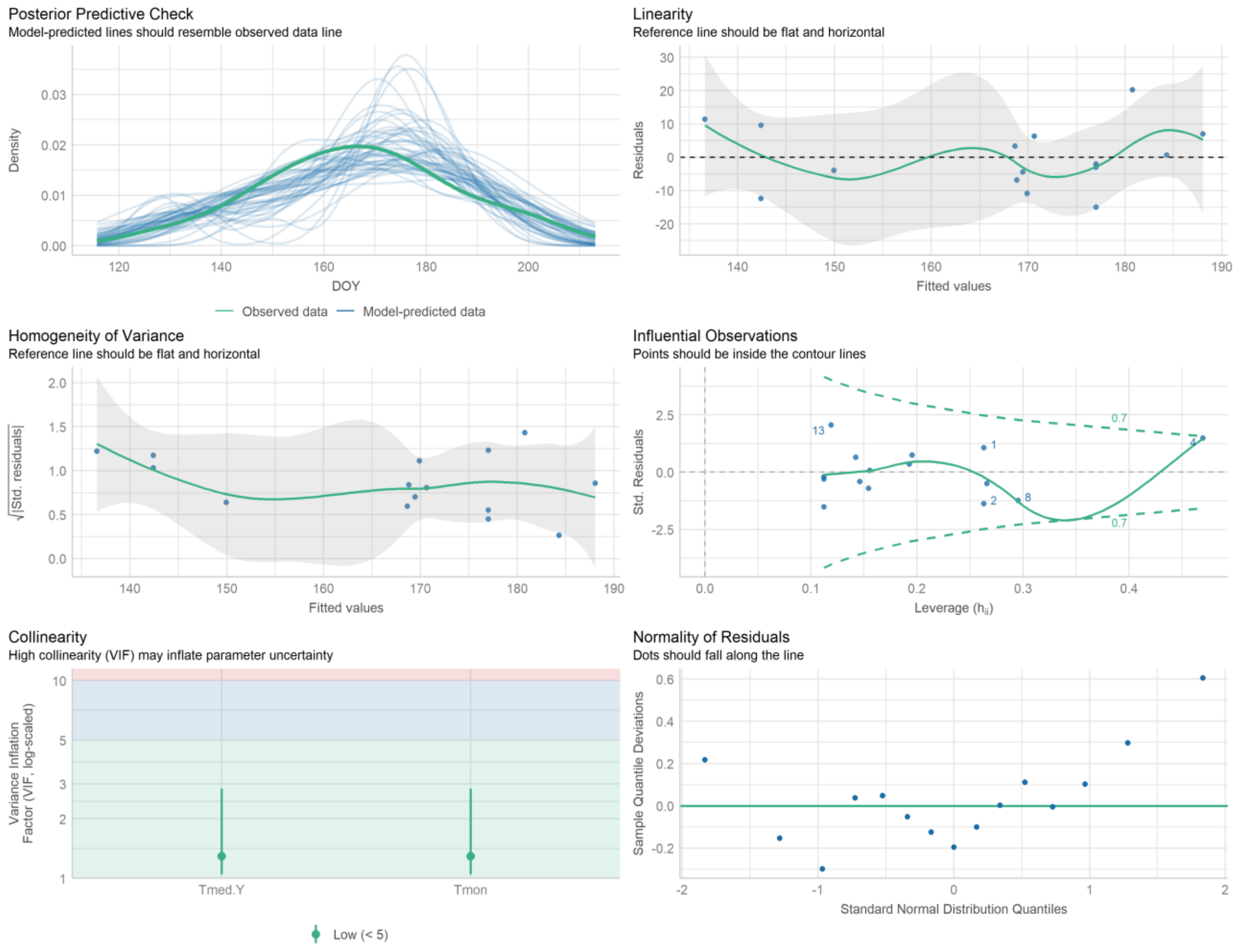

1.115. MLM - F - Salvia lavandulifolia

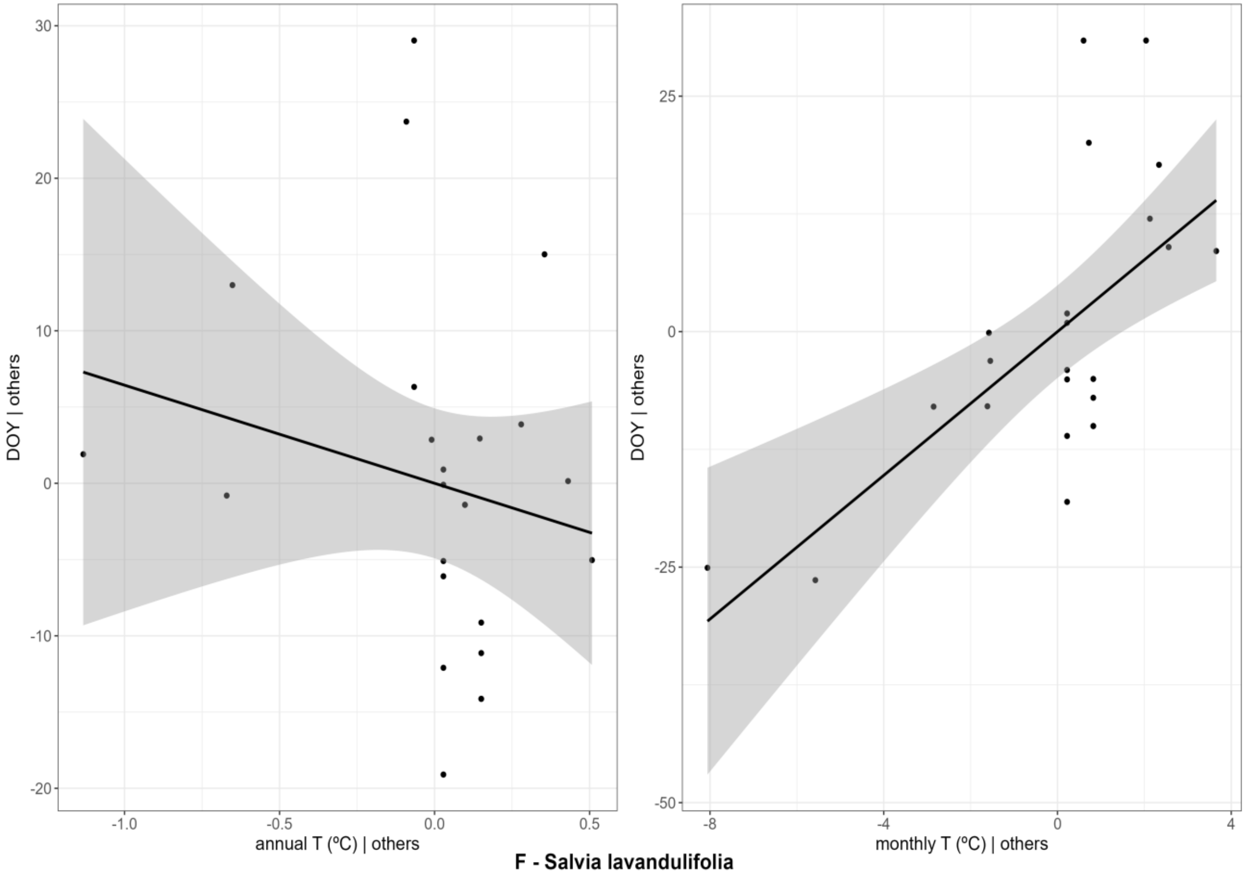

F - Salvia lavandulifolia

DOY = 190.87 (-6.44*annual T (ºC)) + (+3.81*monthly T (ºC))

1.115.1. Diagnostics - MLM - F - Salvia lavandulifolia

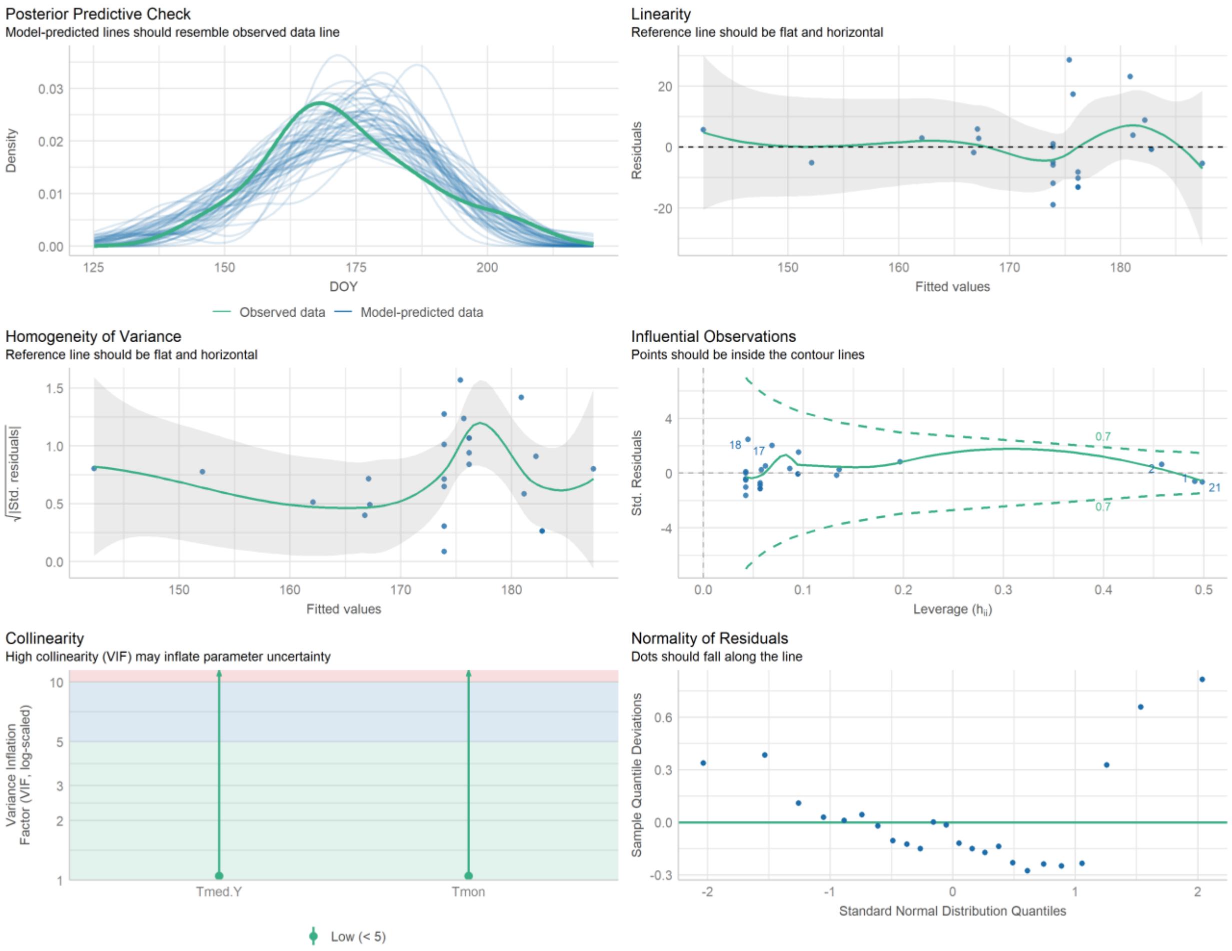

1.116. MLM - FBF - Salvia rosmarinus

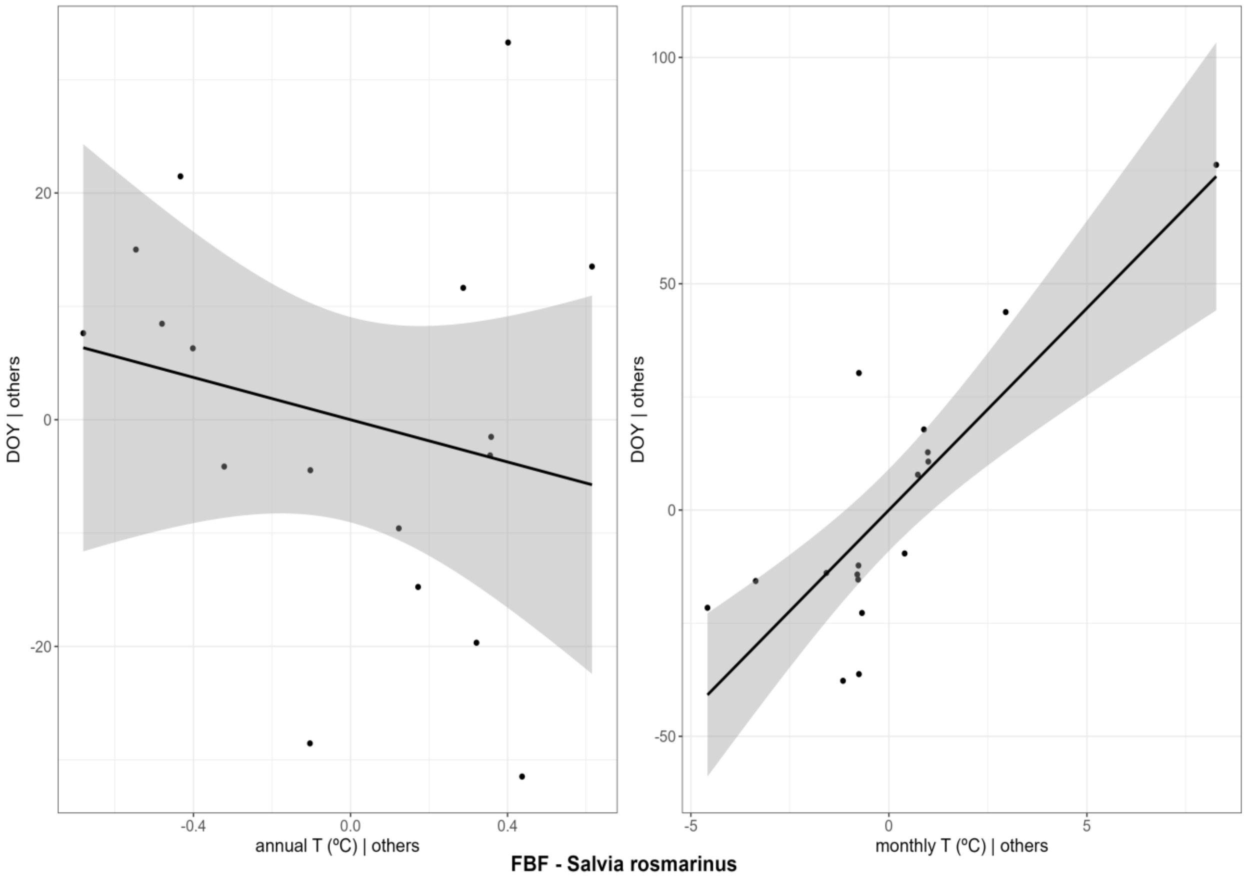

DOY = 118.67 (-9.32*annual T (ºC)) + (+8.92*monthly T (ºC))

1.116.1. Diagnostics - MLM - FBF - Salvia rosmarinus

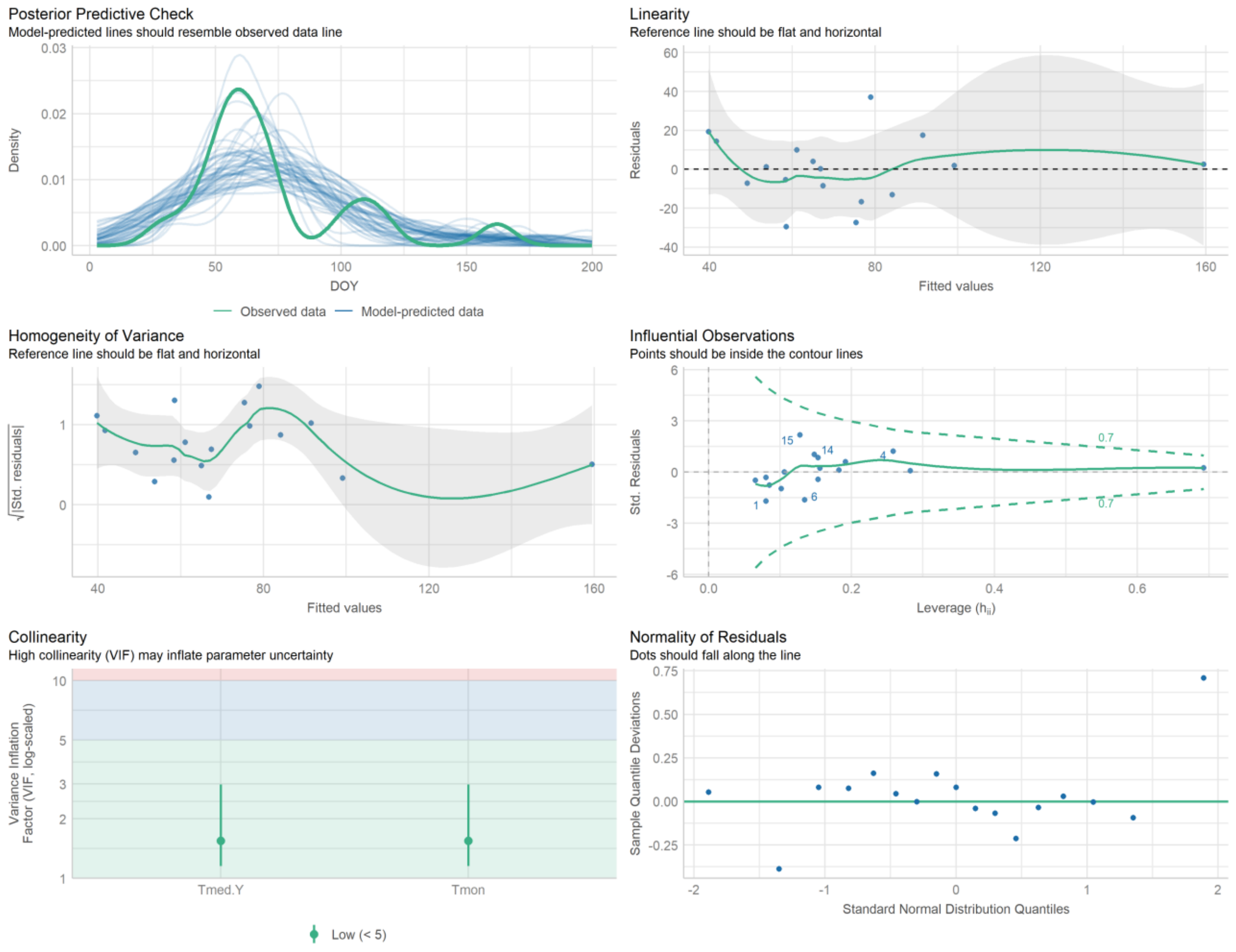

1.117. MLM - F - Saxifraga reuteriana

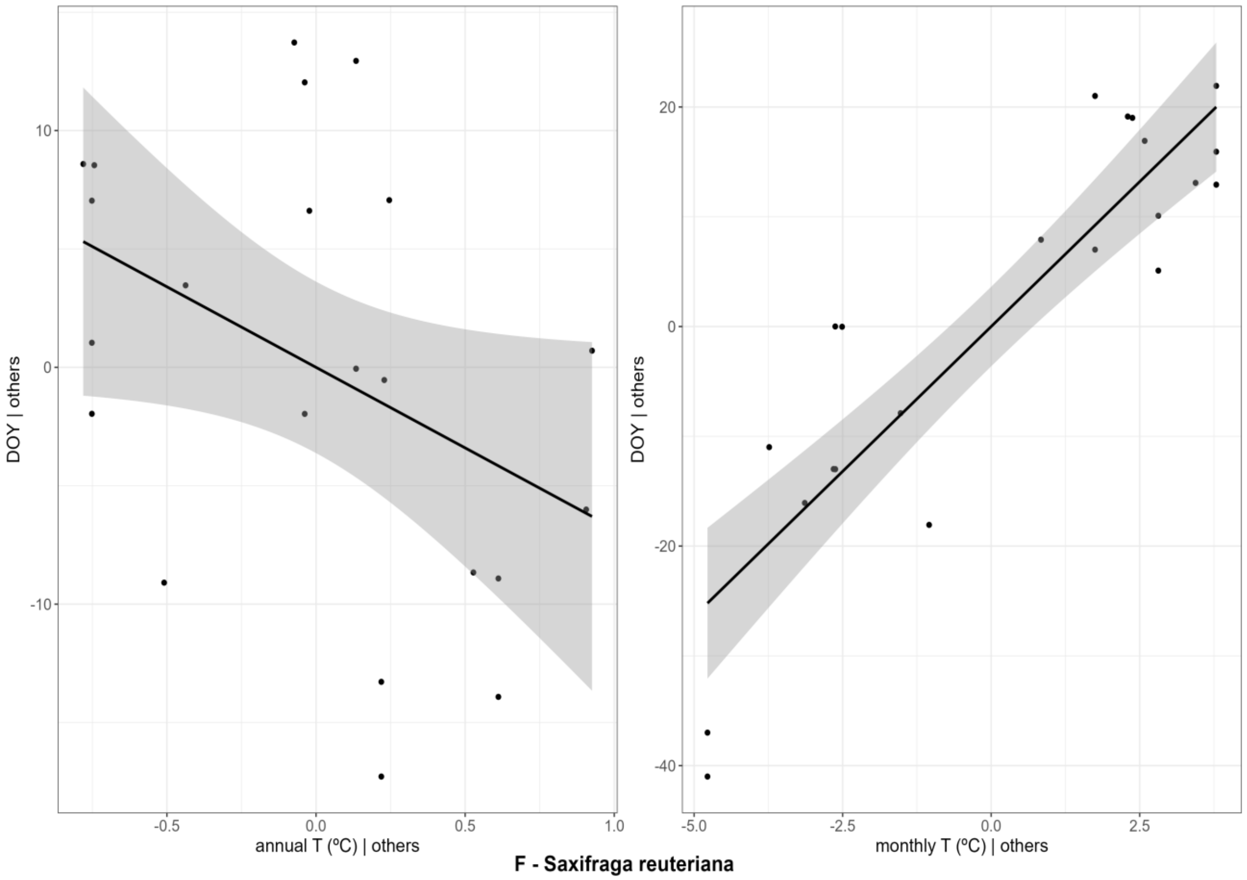

DOY = 160.40 (-6.80*annual T (ºC)) + (+5.28*monthly T (ºC))

1.117.1. Diagnostics - MLM - F - Saxifraga reuteriana

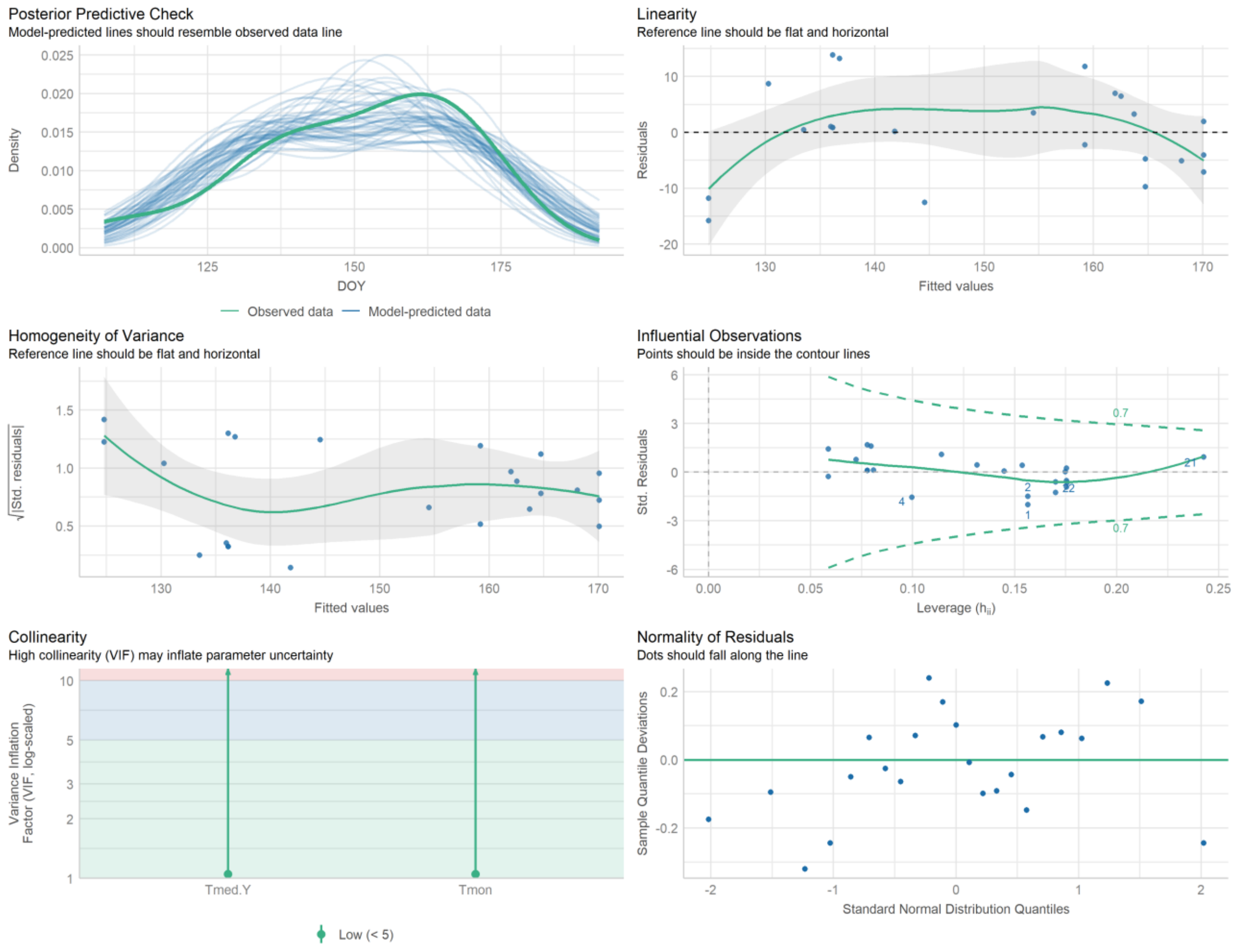

1.118. MLM - F - Sempervivum minutum

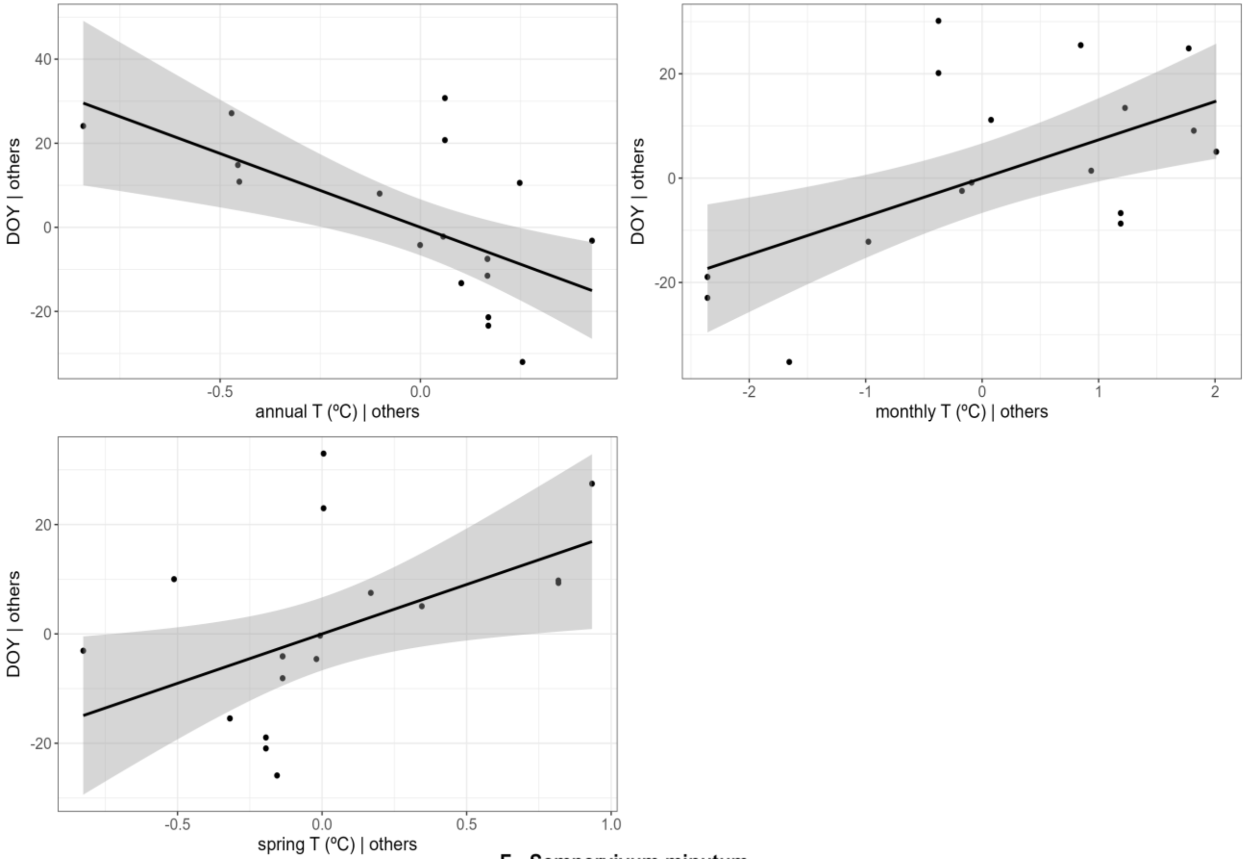

F - Sempervivum minutum

DOY = 335.22 (-35.11*annual T (ºC)) + (+7.34*monthly T (ºC)) + (+18.07*spring T (ºC))

1.118.1. Diagnostics - MLM - F - Sempervivum minutum

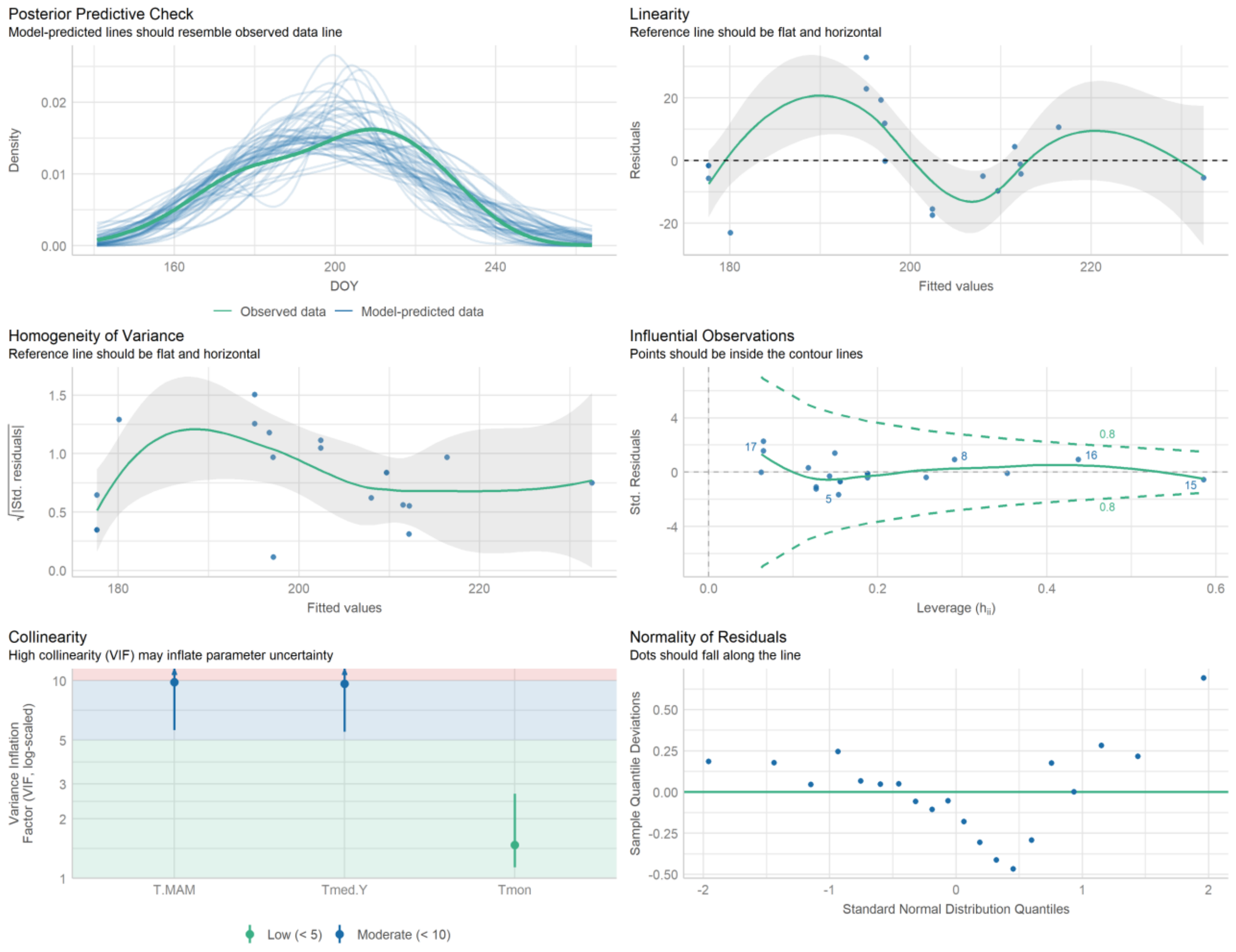

1.119. MLM - FBF - Sideritis glacialis

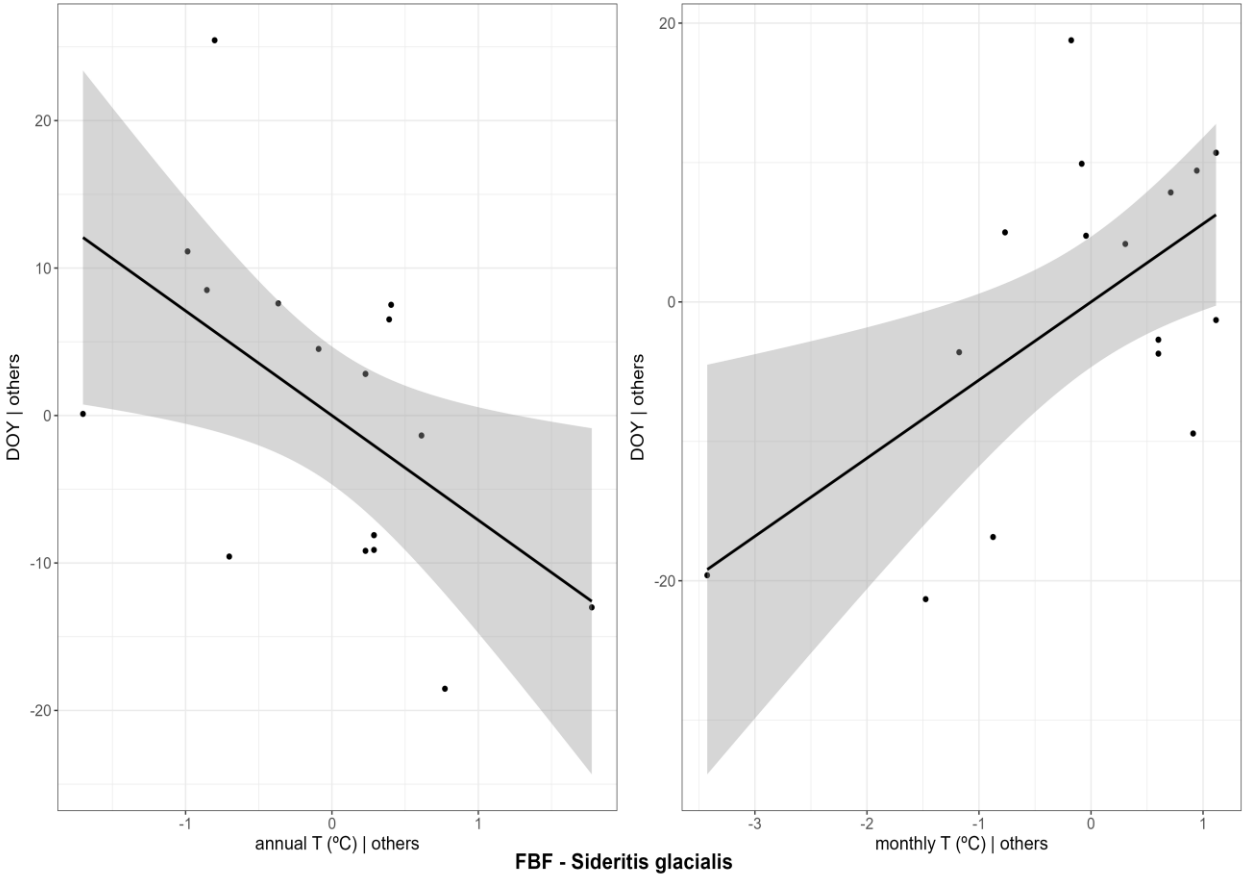

DOY = 167.62 (-7.10*annual T (ºC)) + (+5.60*monthly T (ºC))

1.119.1. Diagnostics - MLM - FBF - Sideritis glacialis

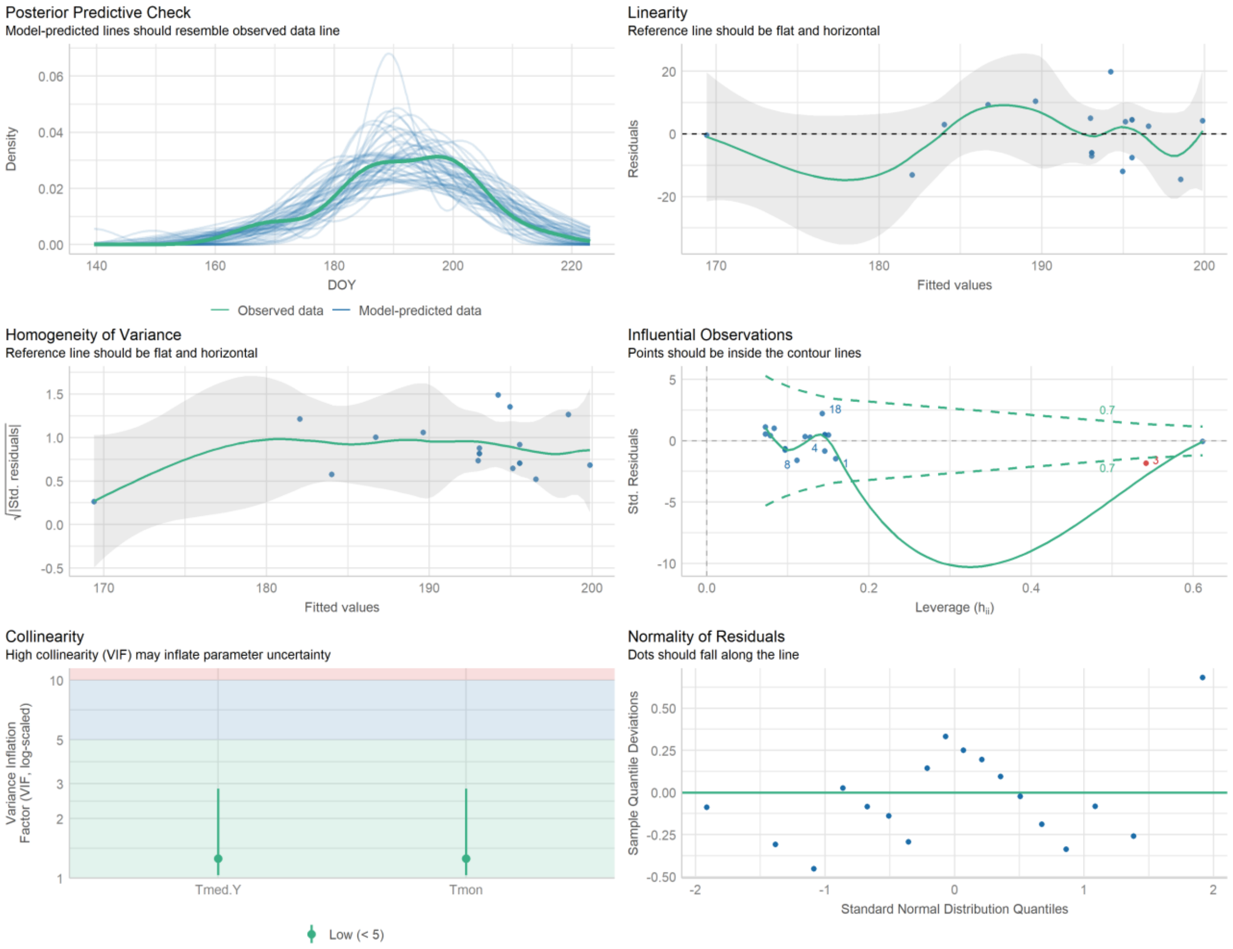

1.120. MLM - F - Sideritis glacialis

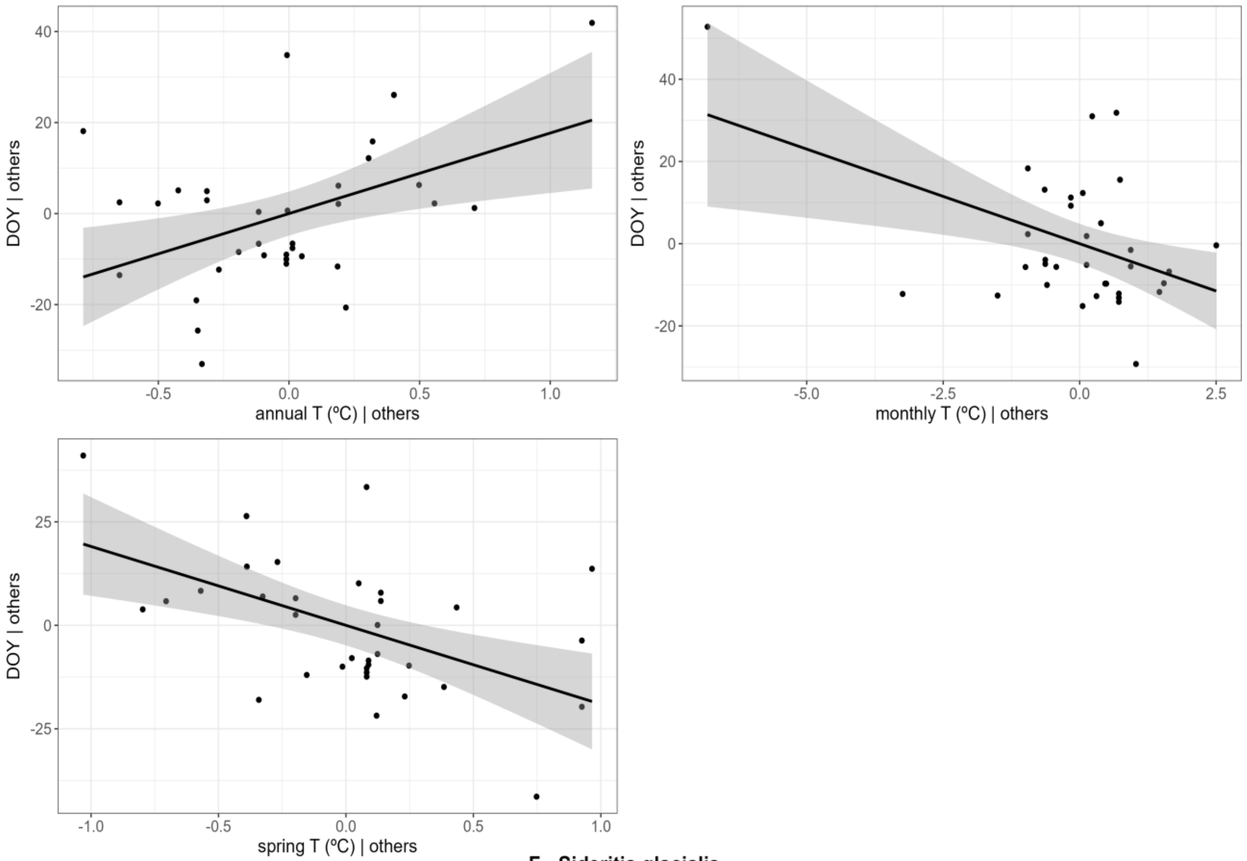

F - Sideritis glacialis

DOY = 300.47 (+17.70*annual T (ºC)) + (-4.60*monthly T (ºC)) + (-19.05*spring T (ºC))

1.120.1. Diagnostics - MLM - F - Sideritis glacialis

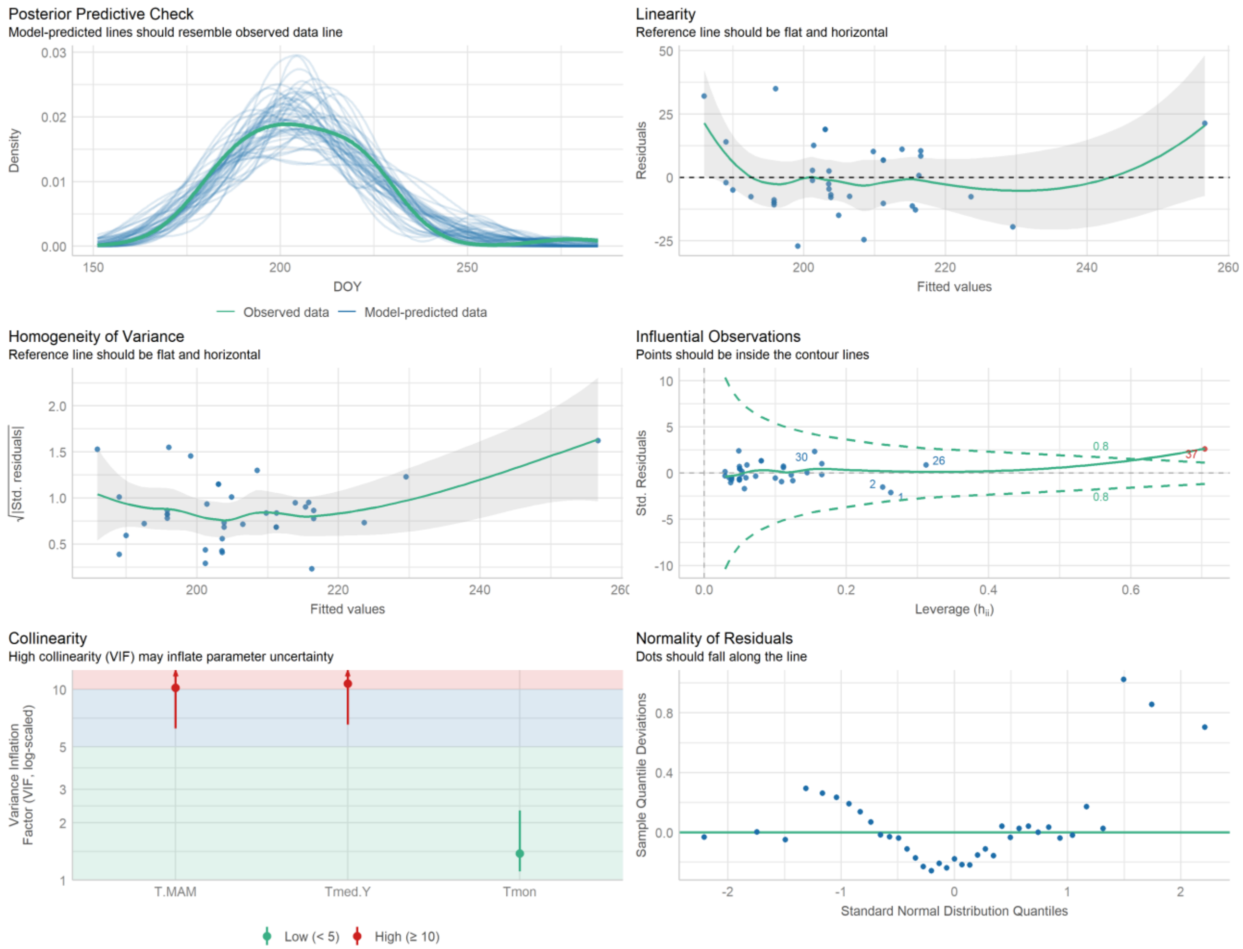

1.121. MLM - DVG - Sideritis glacialis

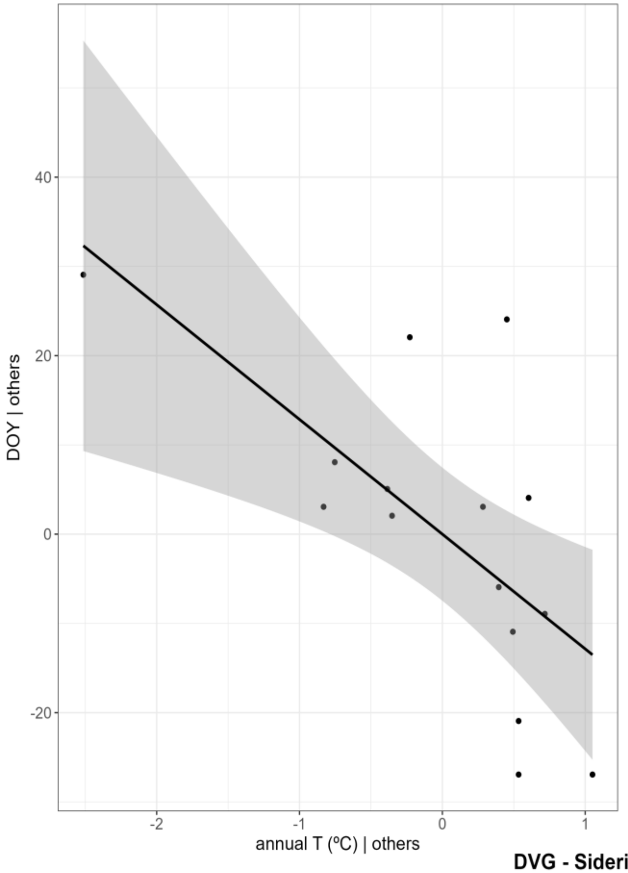

DVG - Sideritis glacialis

DOY = 403.67 (-12.86*annual T (ºC))

1.121.1. Diagnostics - MLM - DVG - Sideritis glacialis

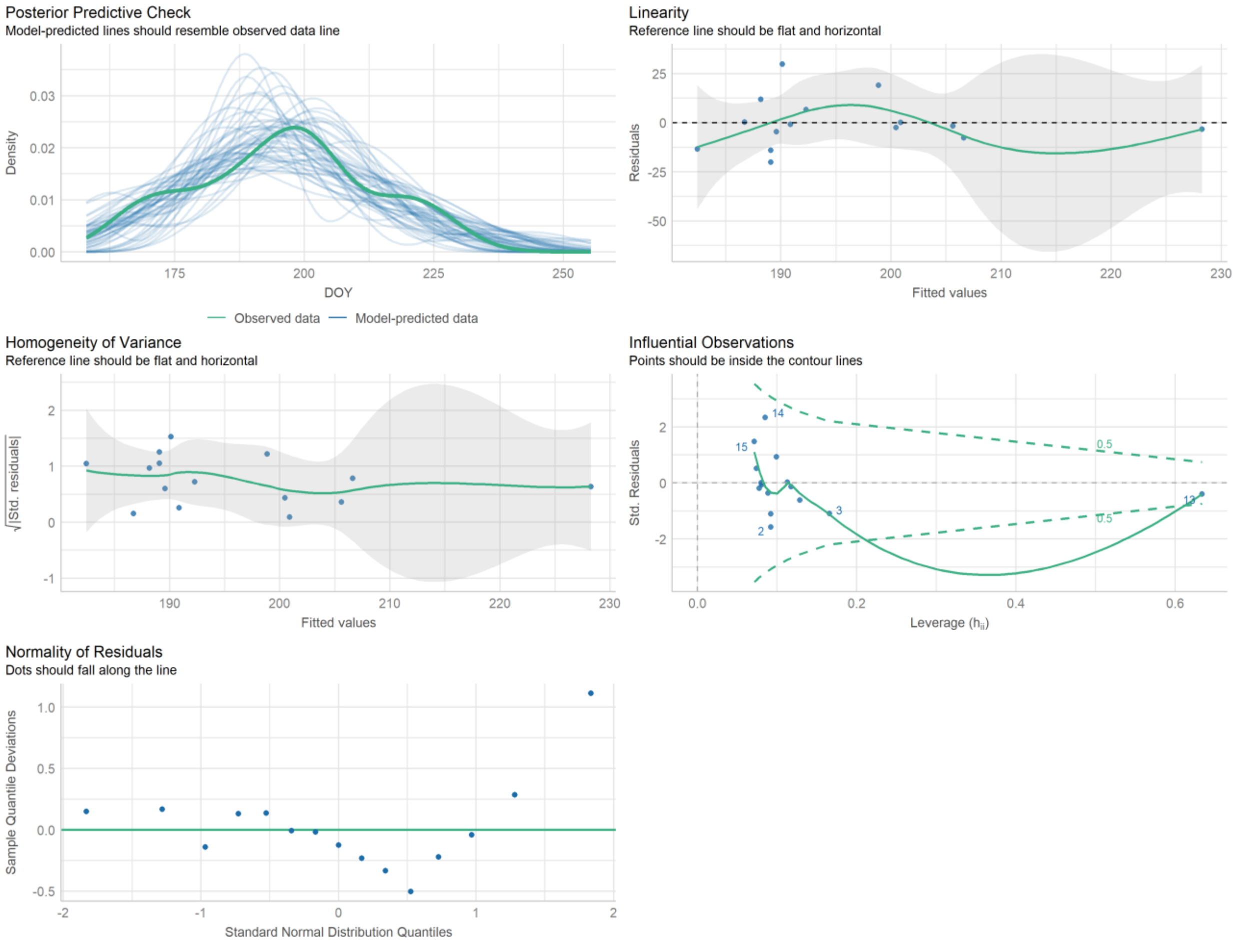

1.122. MLM - DVG - Sideritis incana

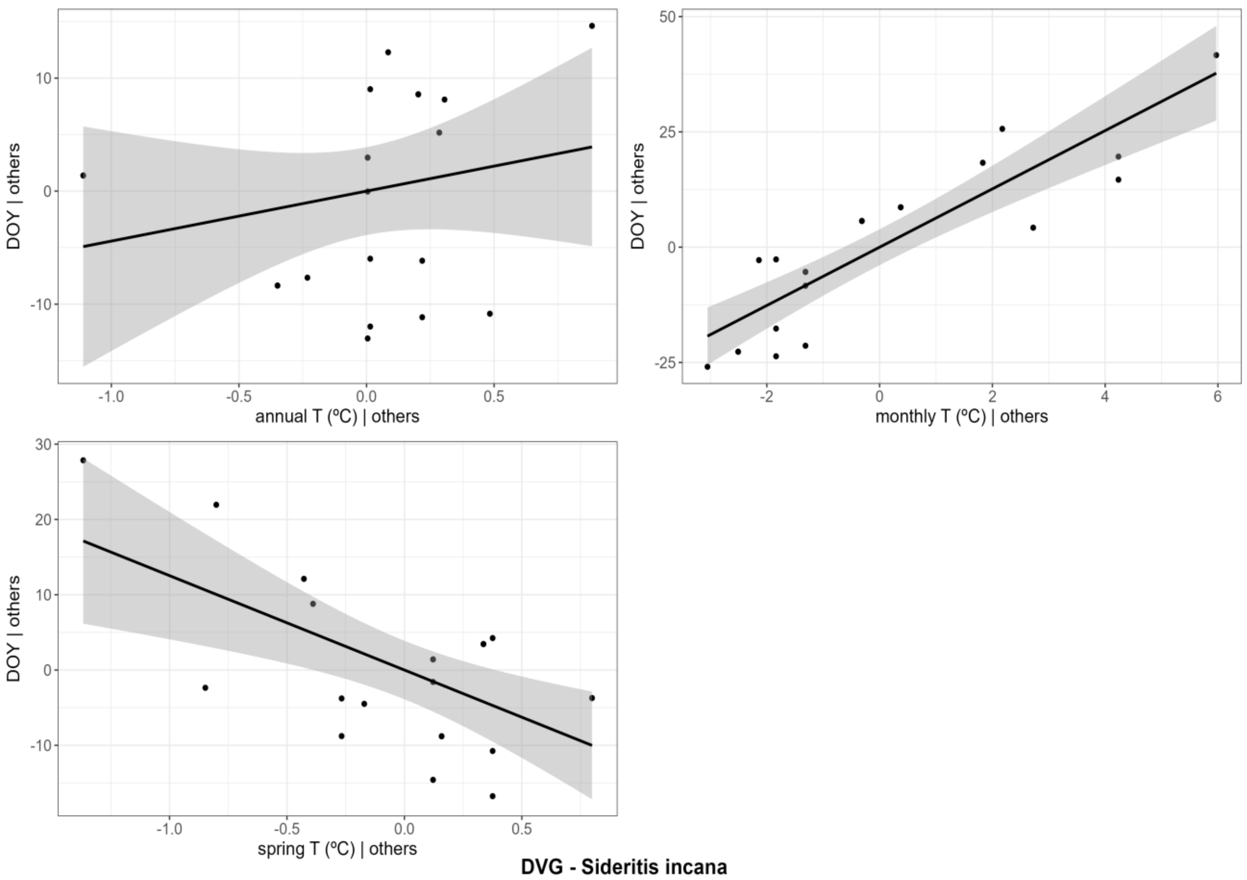

DOY = 138.38 (+4.42*annual T (ºC)) + (+6.32*monthly T (ºC)) + (-12.53*spring T (ºC))

1.122.1. Diagnostics - MLM - DVG - Sideritis incana

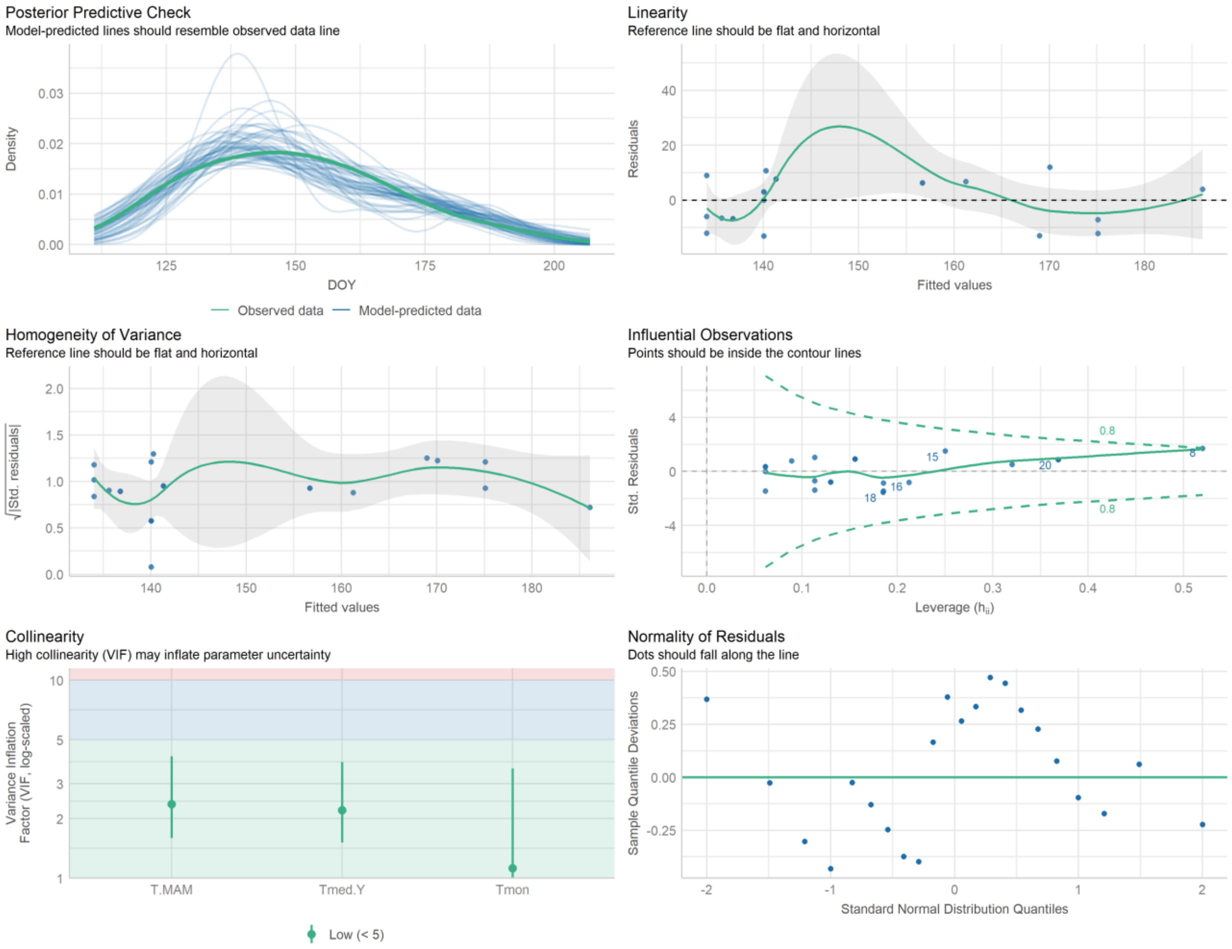

1.123. MLM - FBF - Staehelina baetica

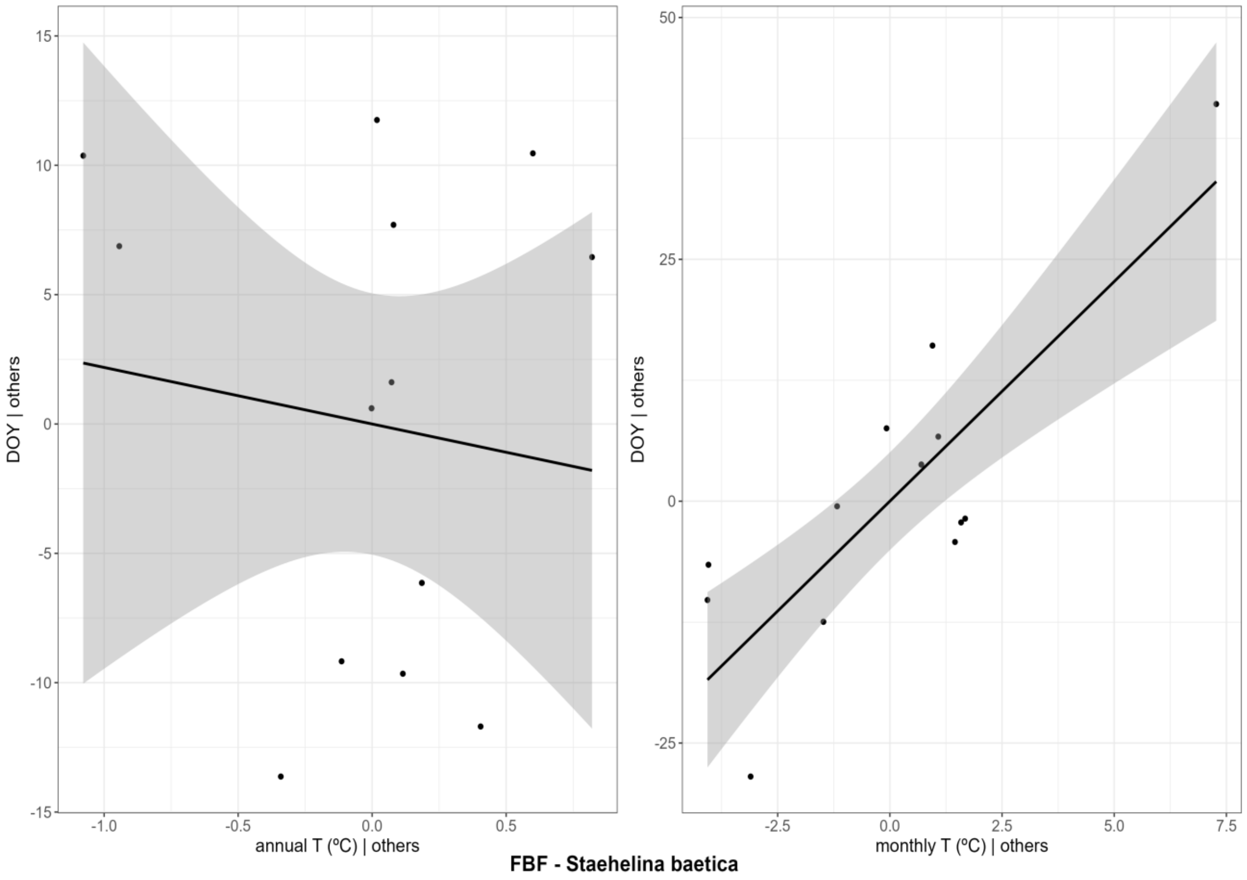

DOY = 88.33 (-2.19*annual T (ºC)) + (+4.54*monthly T (ºC))

1.123.1. Diagnostics - MLM - FBF - Staehelina baetica

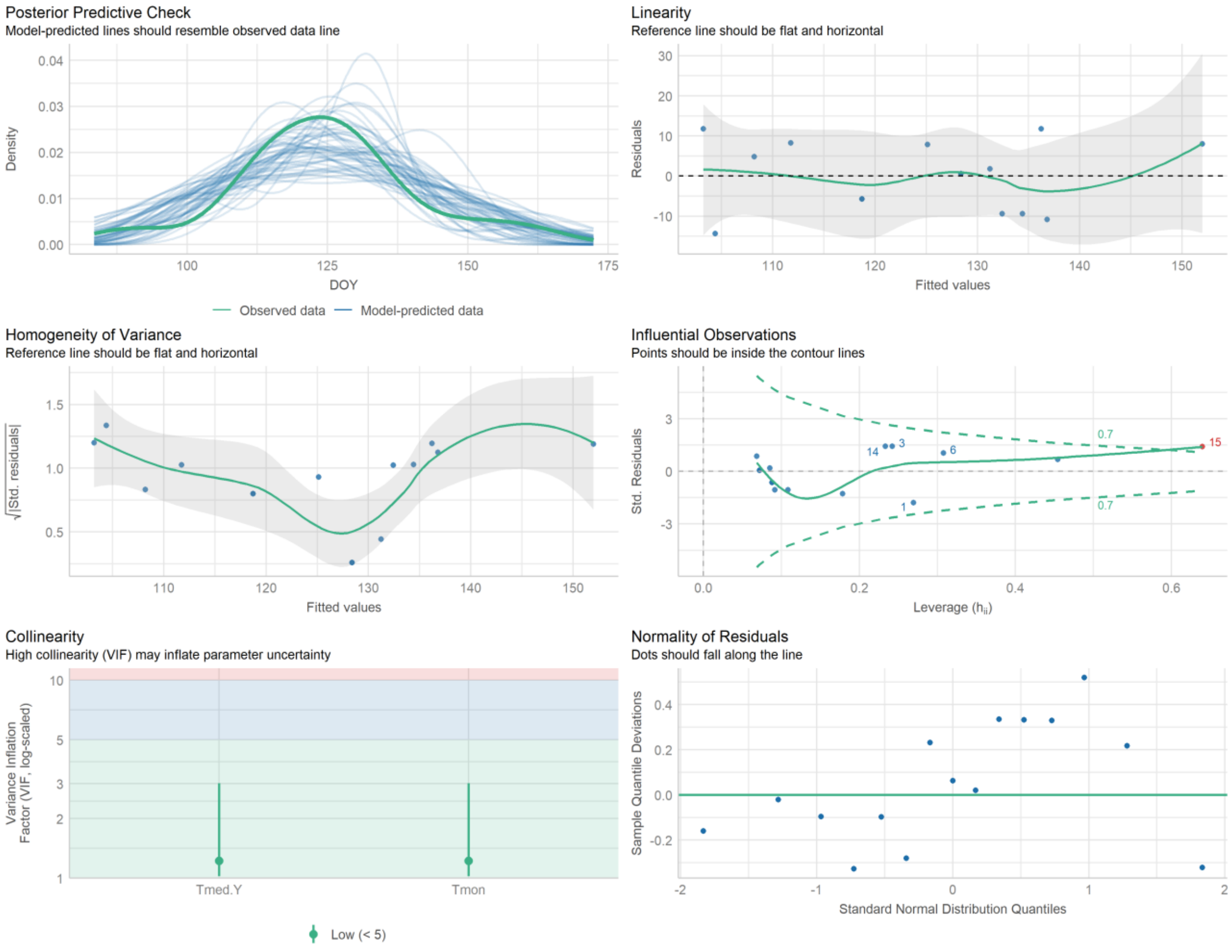

1.124. MLM - F - Staehelina baetica

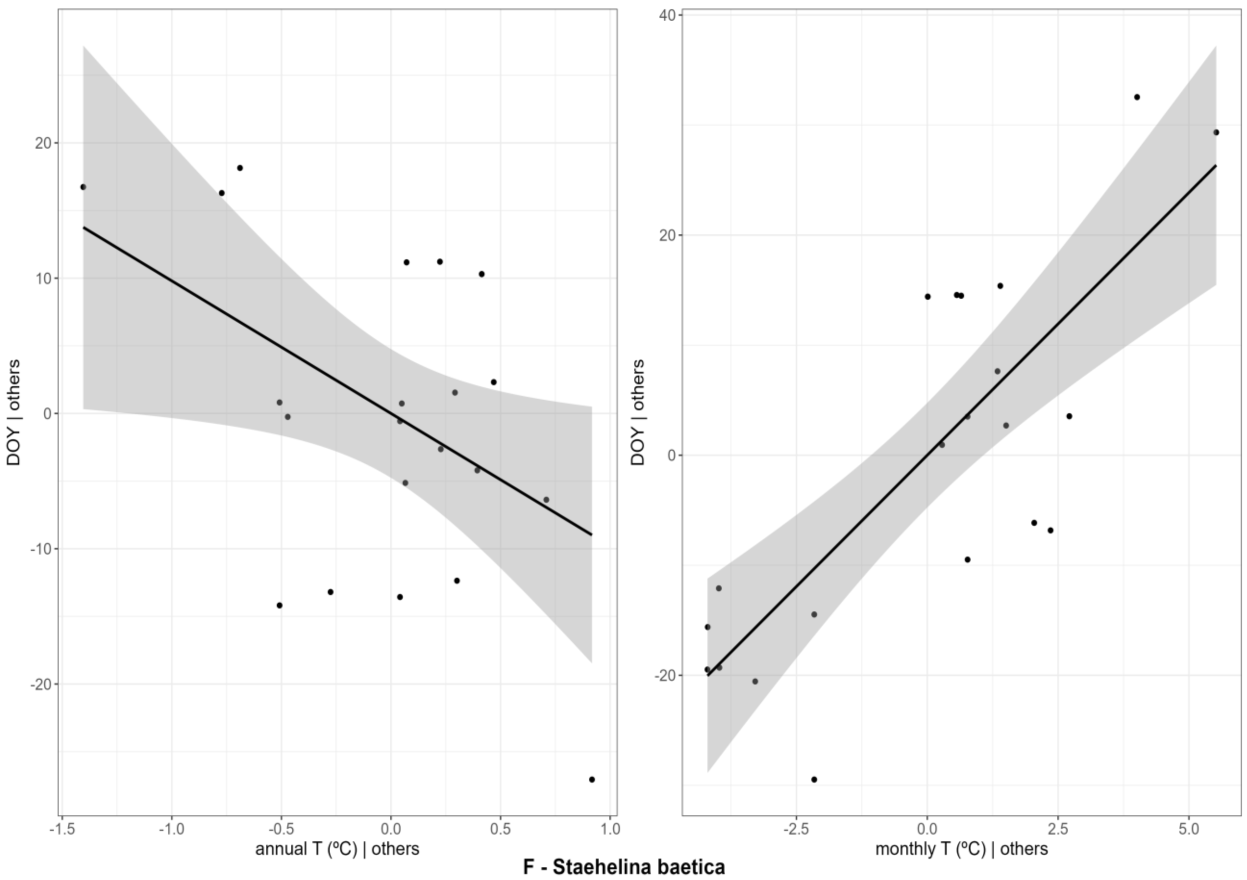

DOY = 224.86 (-9.80*annual T (ºC)) + (+4.77*monthly T (ºC))

1.124.1. Diagnostics - MLM - F - Staehelina baetica

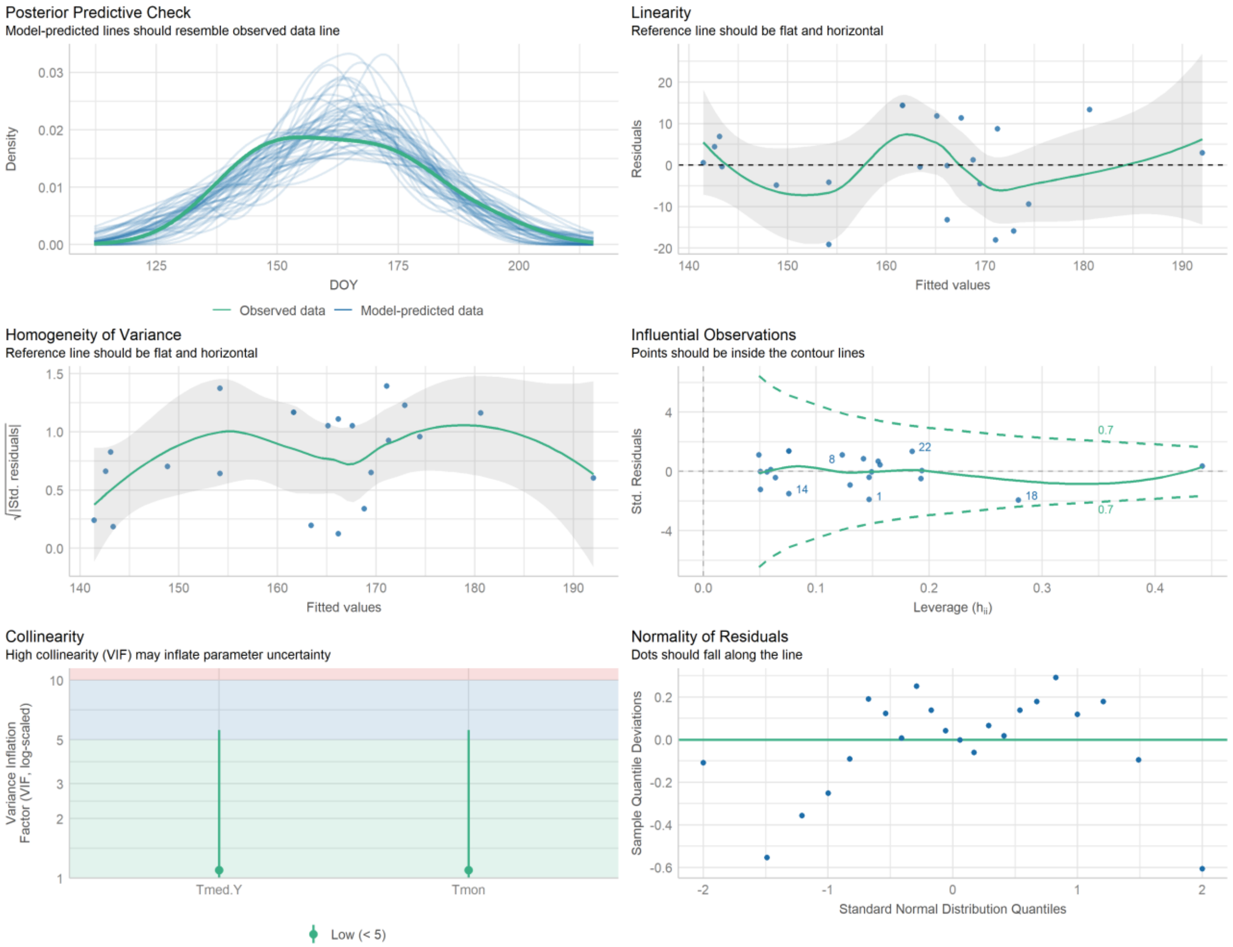

1.125. MLM - FS - Teline linifolia

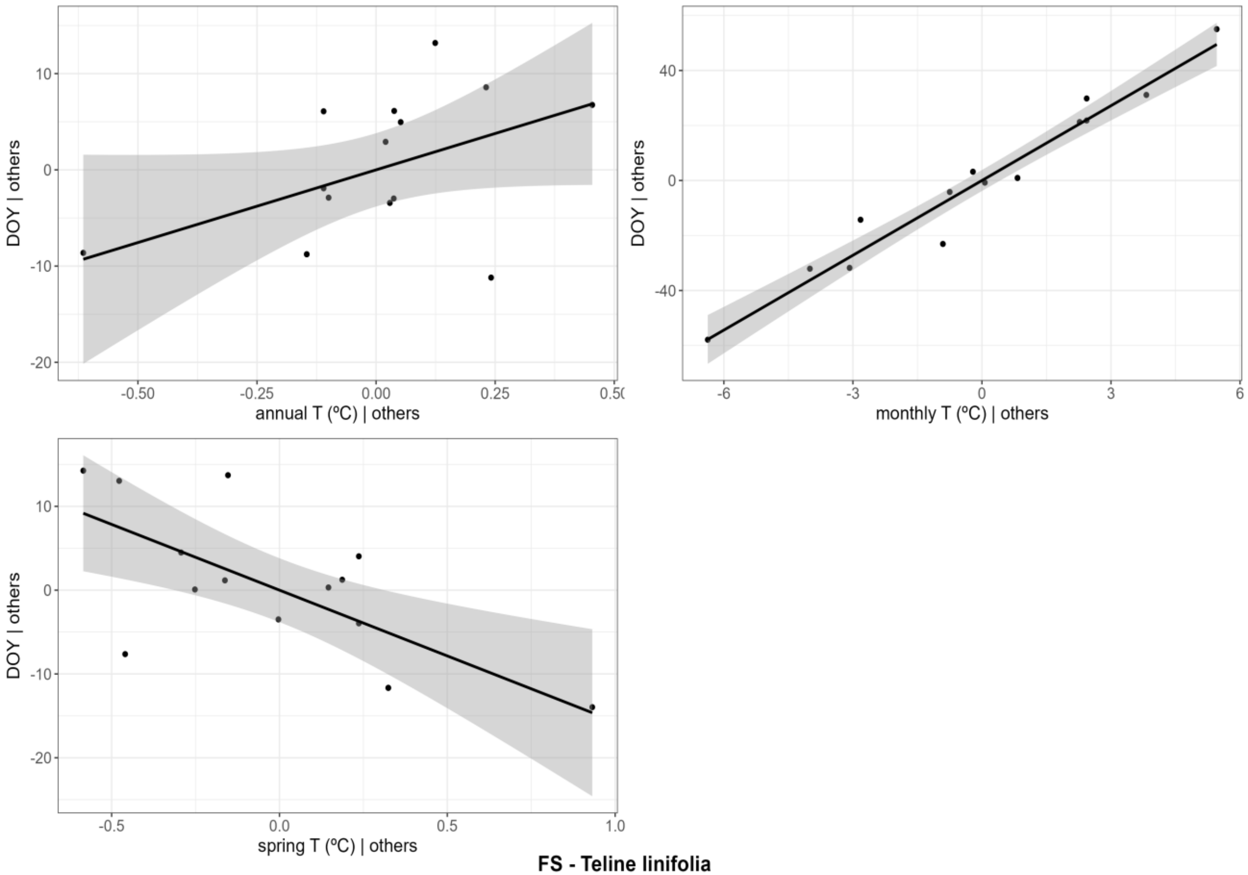

FS - Teline linifolia

DOY = -47.77 (+15.11*annual T (ºC)) + (+9.06*monthly T (ºC)) + (-15.69*spring T (ºC))

1.125.1. Diagnostics - MLM - FS - Teline linifolia

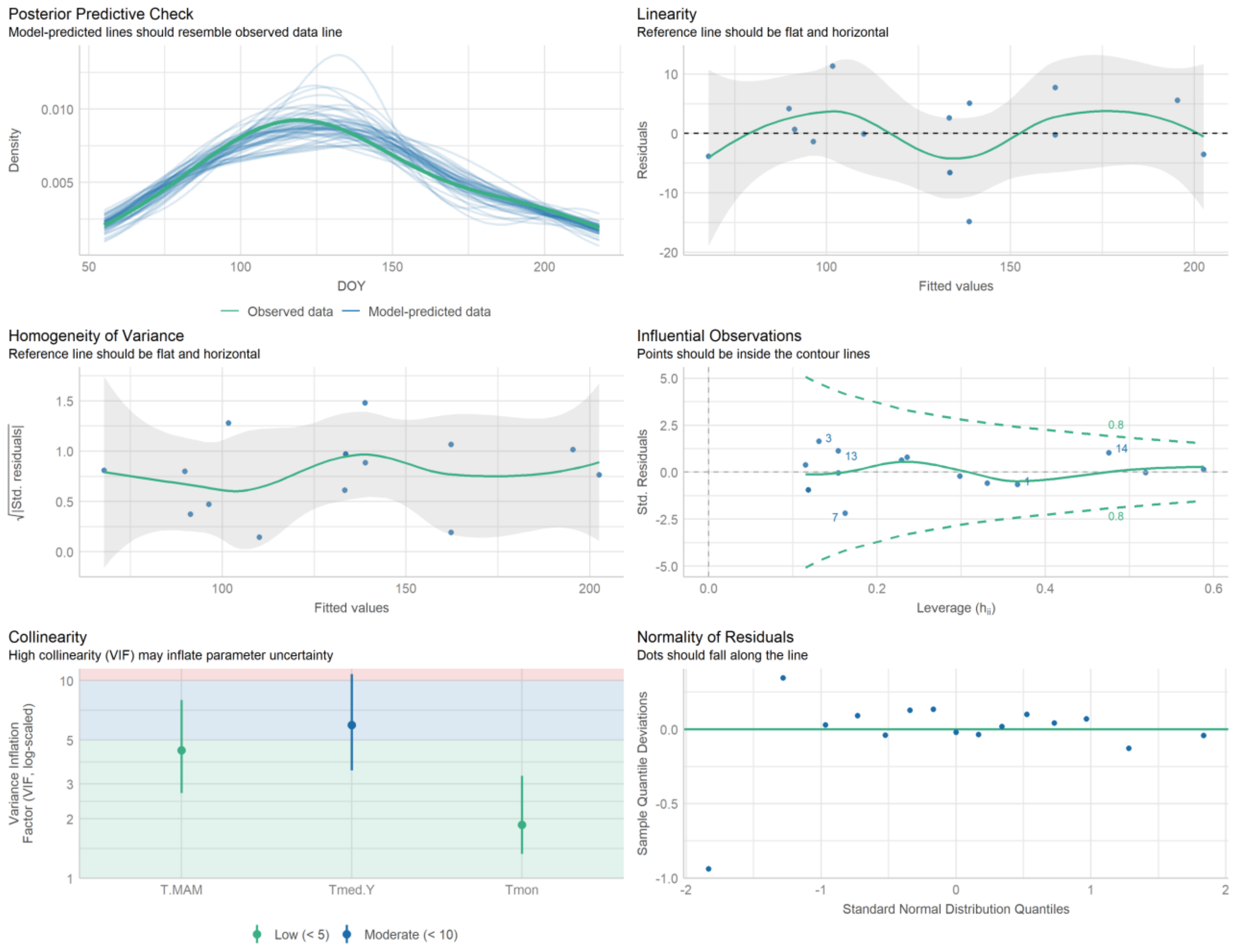

1.126. MLM - F - Thymus longiflorus

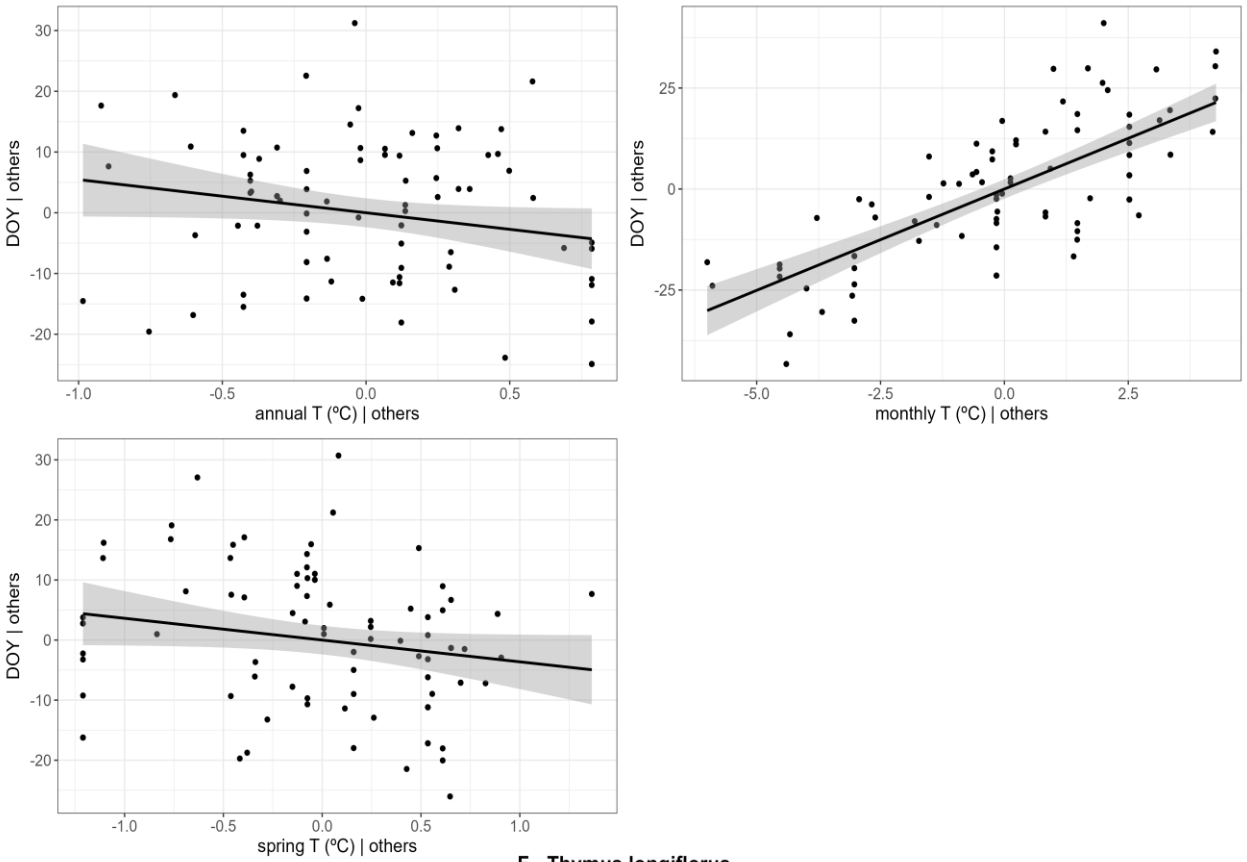

F - Thymus longiflorus

DOY = 196.69 (-5.46*annual T (ºC)) + (+5.02*monthly T (ºC)) + (-3.62*spring T (ºC))

1.126.1. Diagnostics - MLM - F - Thymus longiflorus

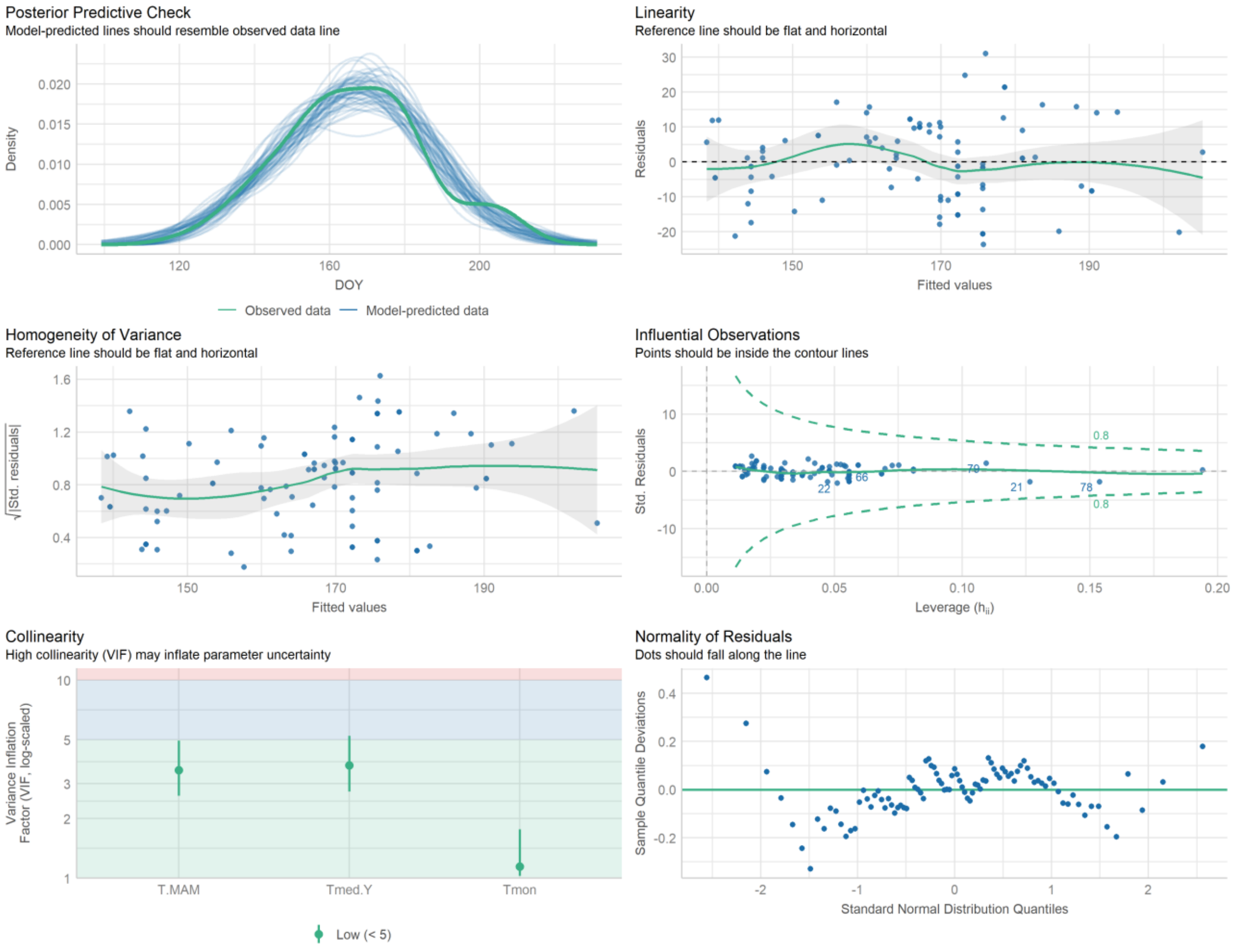

1.127. MLM - FBF - Thymus mastichina

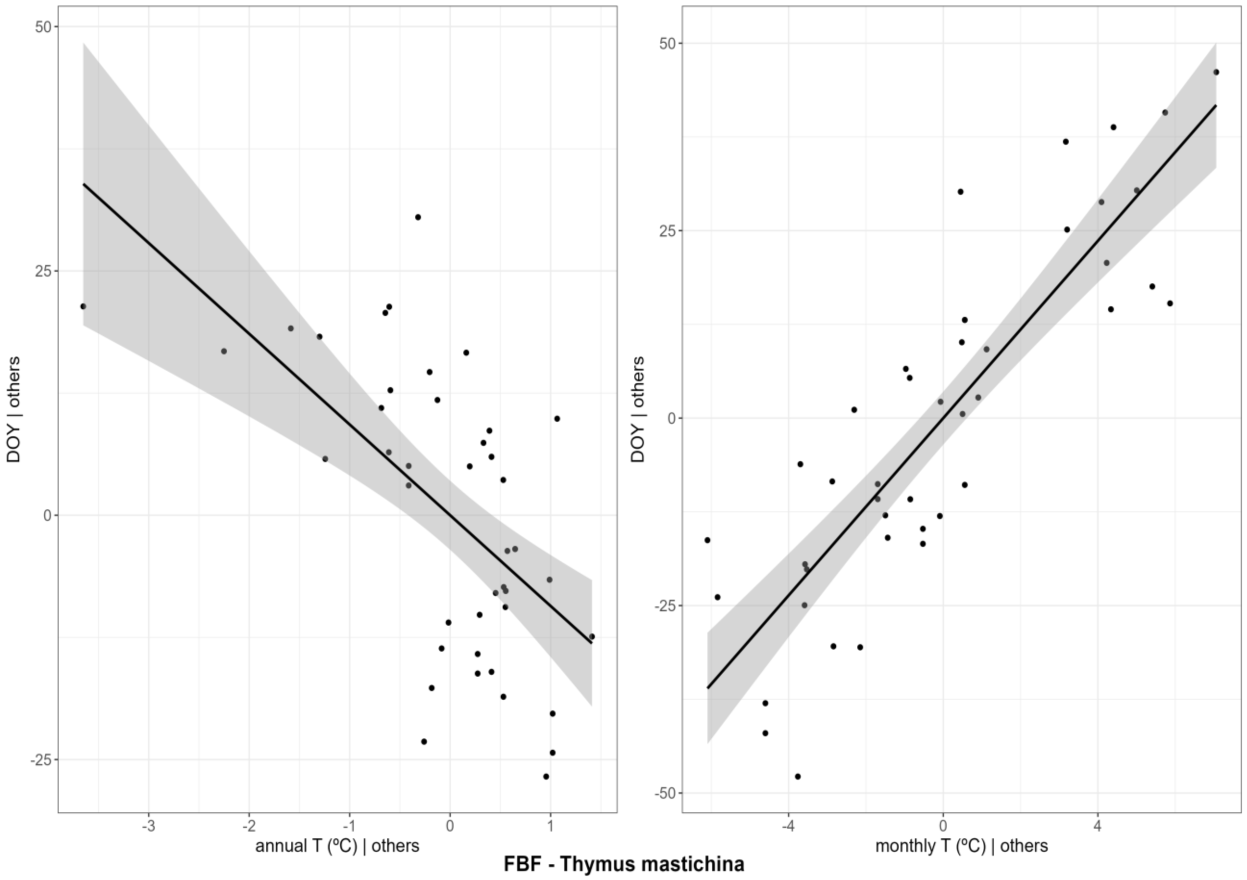

DOY = 184.20 (-9.28*annual T (ºC)) + (+5.91*monthly T (ºC))

1.127.1. Diagnostics - MLM - FBF - Thymus mastichina

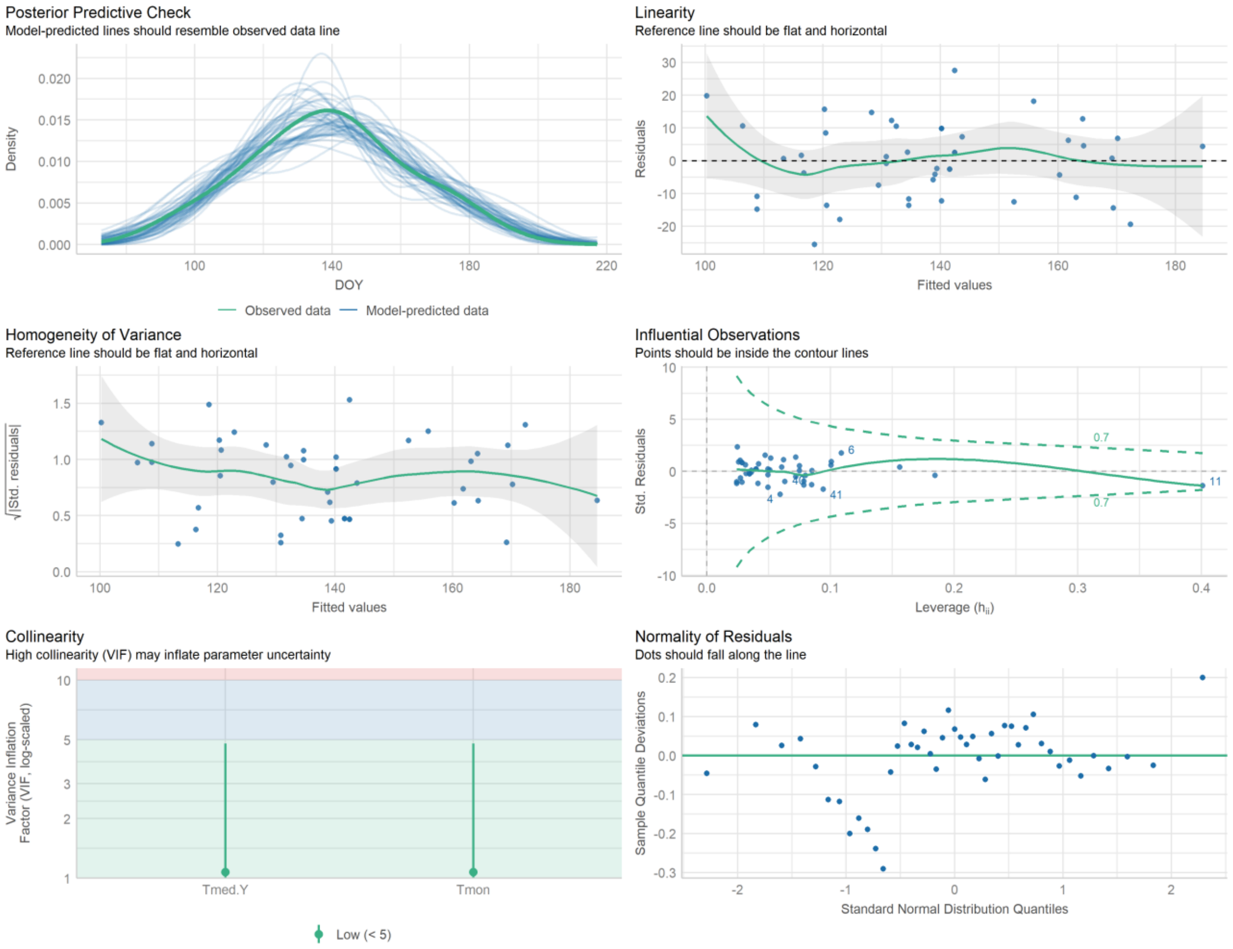

1.128. MLM - F - Thymus mastichina

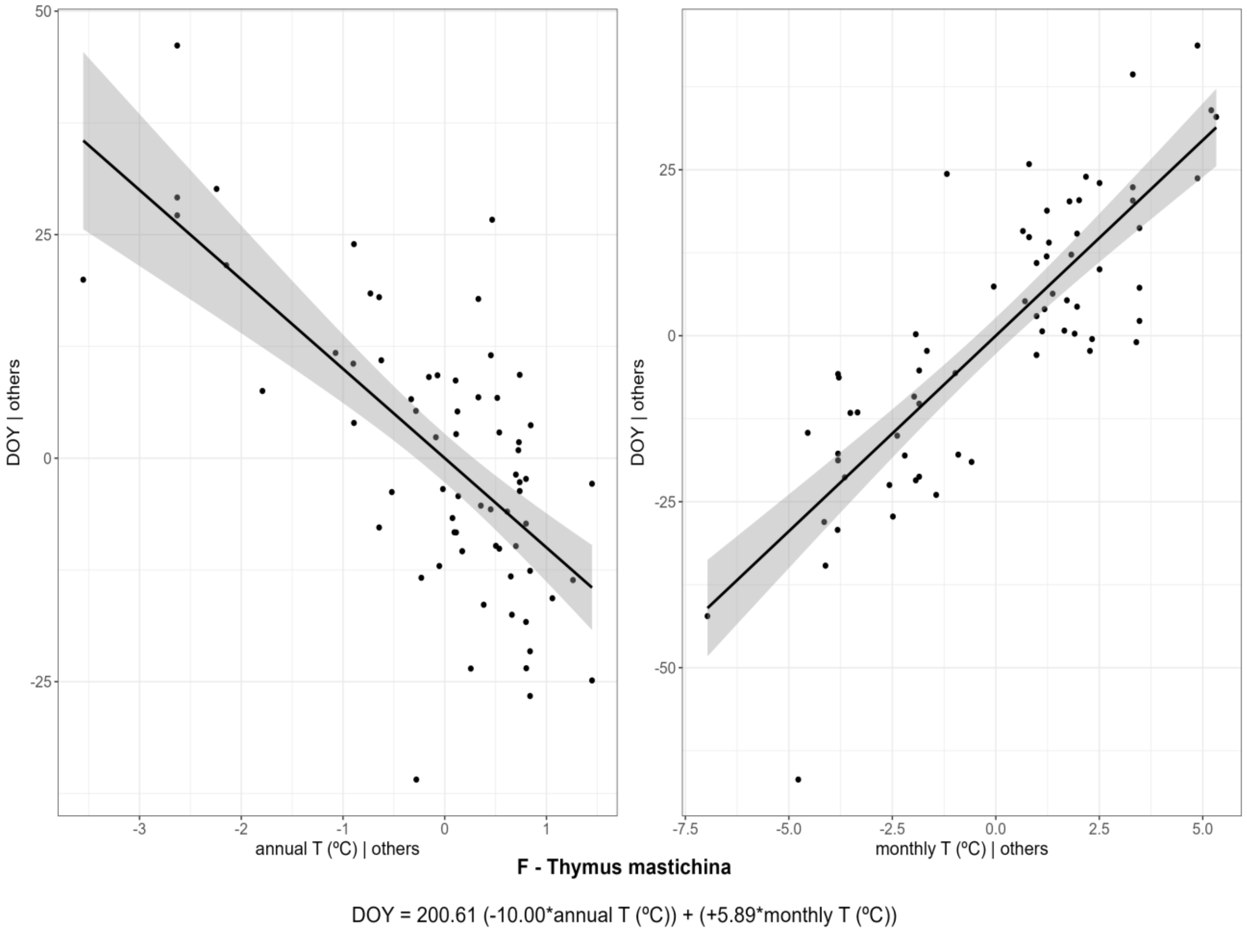

1.128.1. Diagnostics - MLM - F - Thymus mastichina

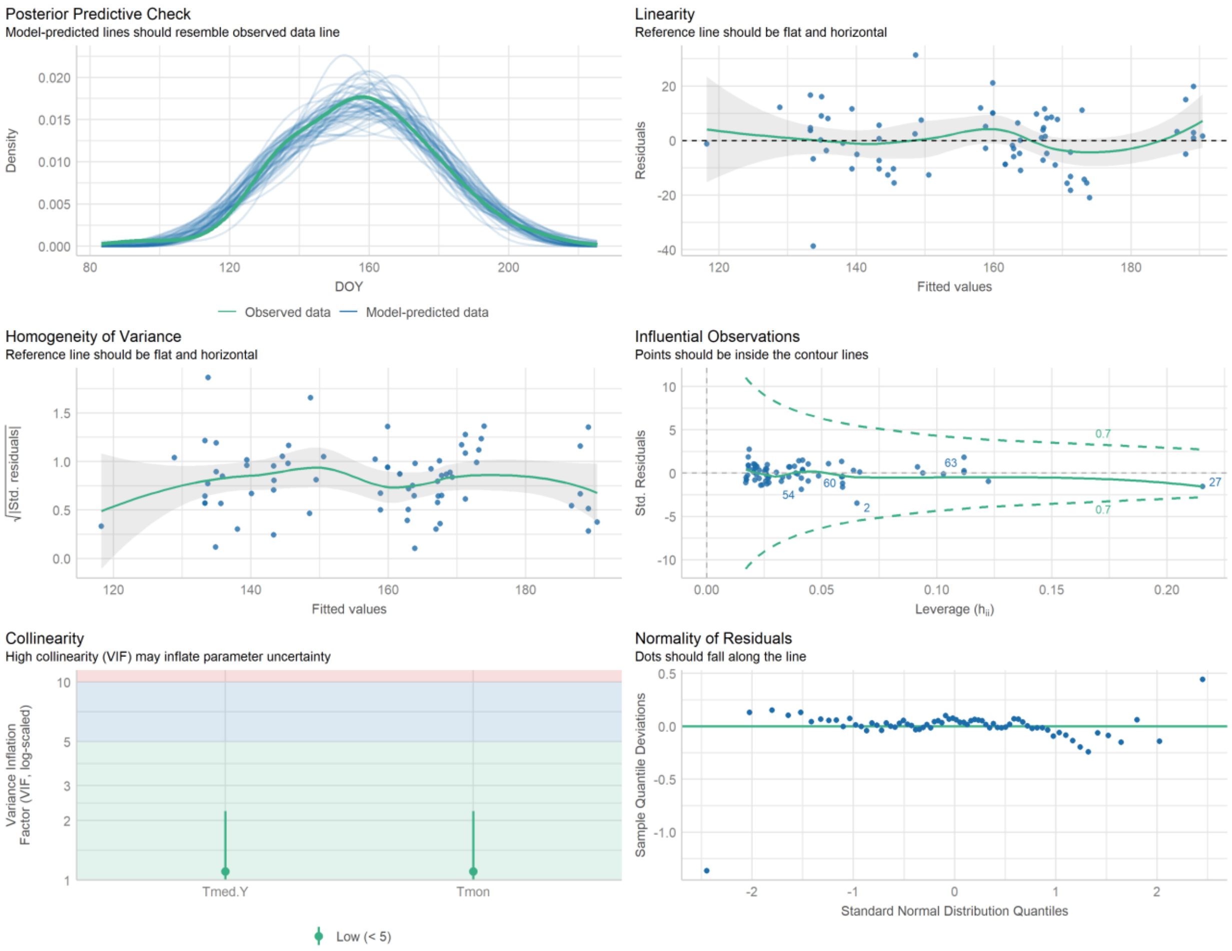

1.129. MLM - FS - Thymus mastichina

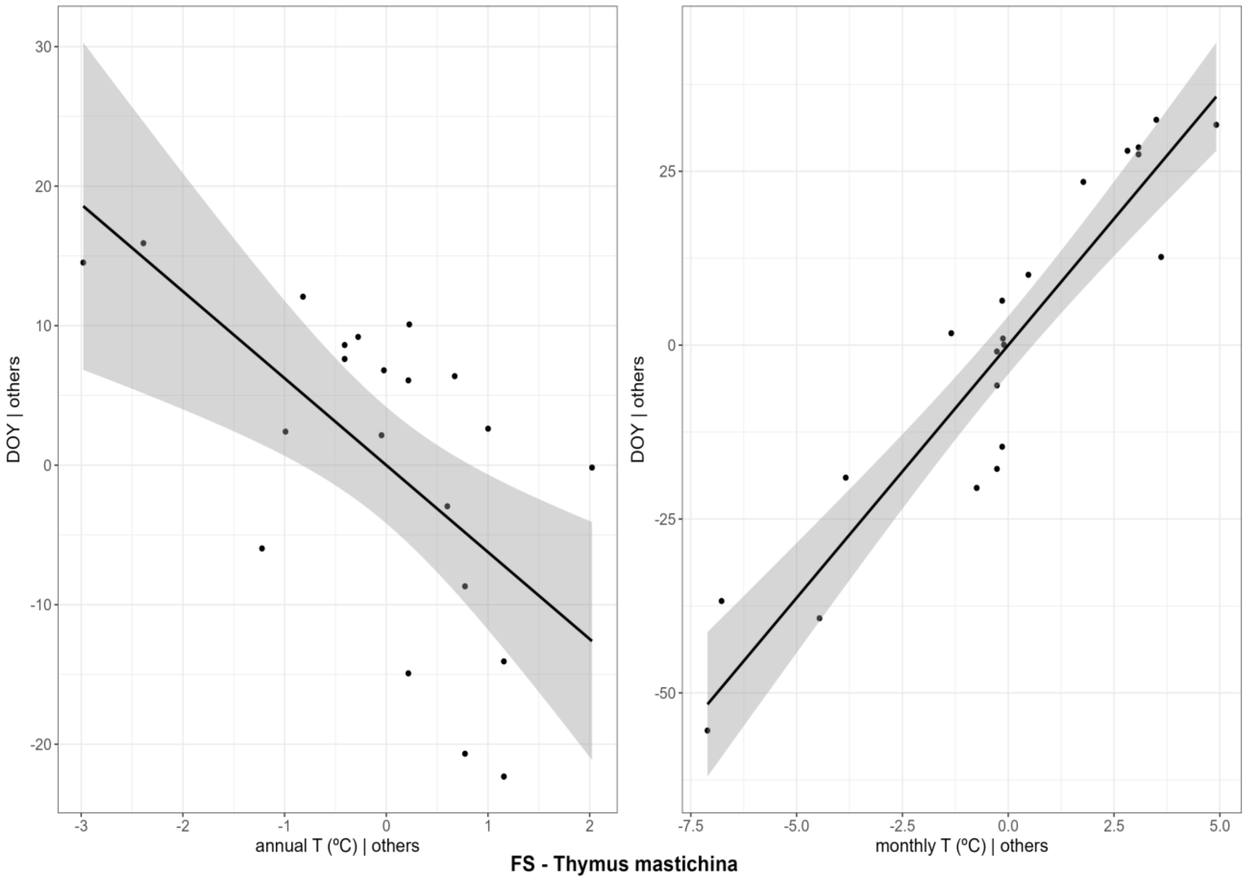

DOY = 115.25 (-6.23*annual T (ºC)) + (+7.27*monthly T (ºC))

1.129.1. Diagnostics - MLM - FS - Thymus mastichina

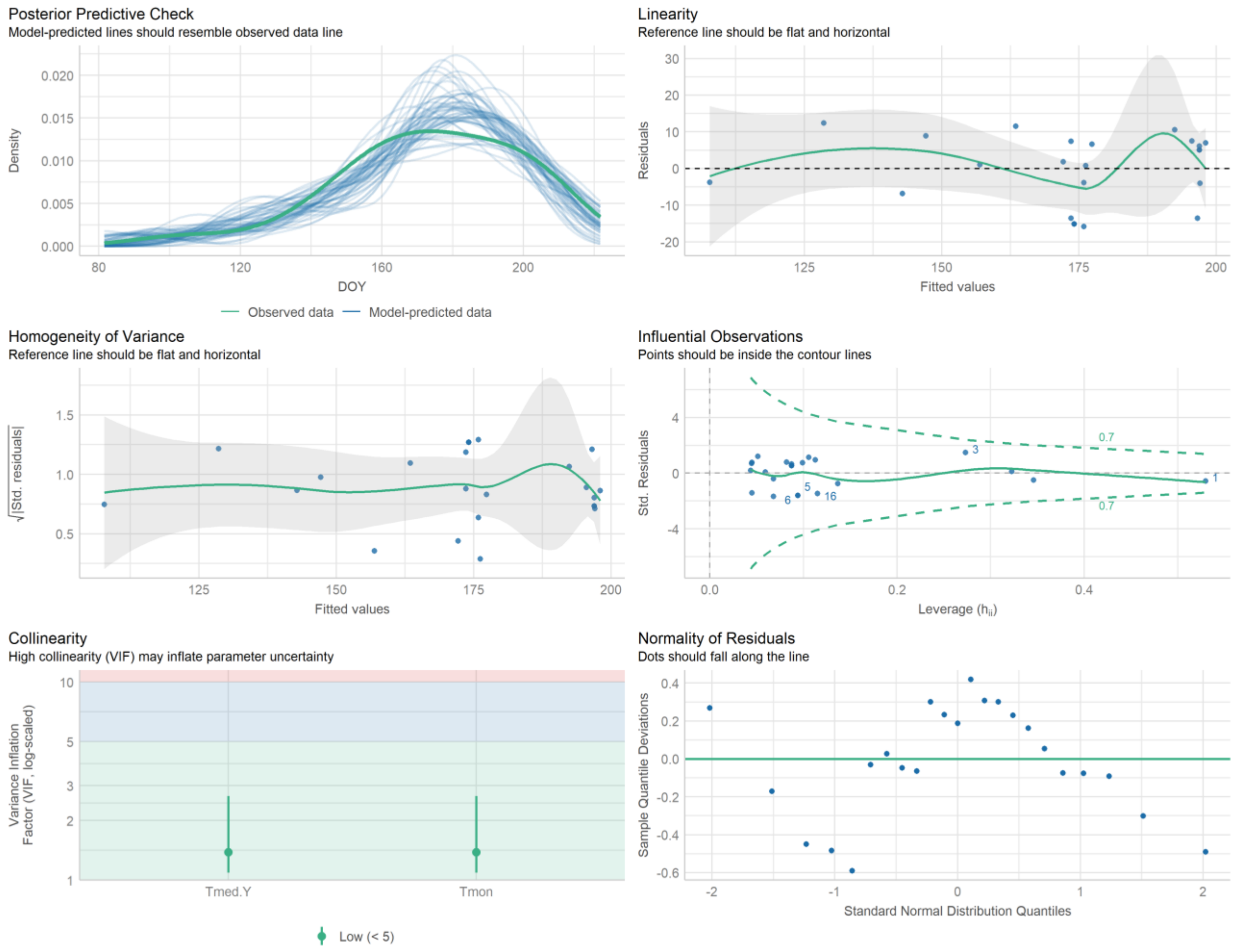

1.130. MLM - DVG - Thymus mastichina

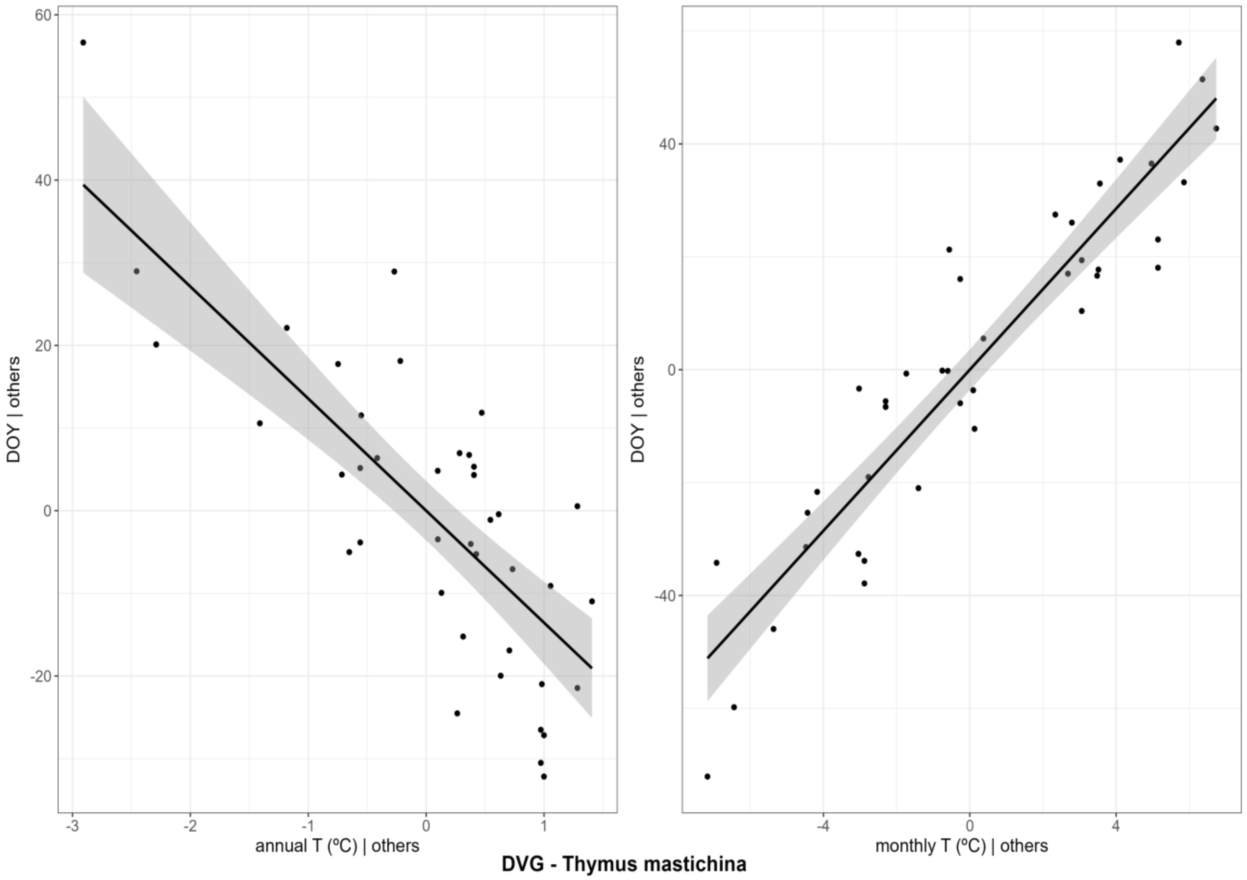

DOY = 232.77 (-13.55*annual T (ºC)) + (+7.13*monthly T (ºC))

1.130.1. Diagnostics - MLM - DVG - Thymus mastichina

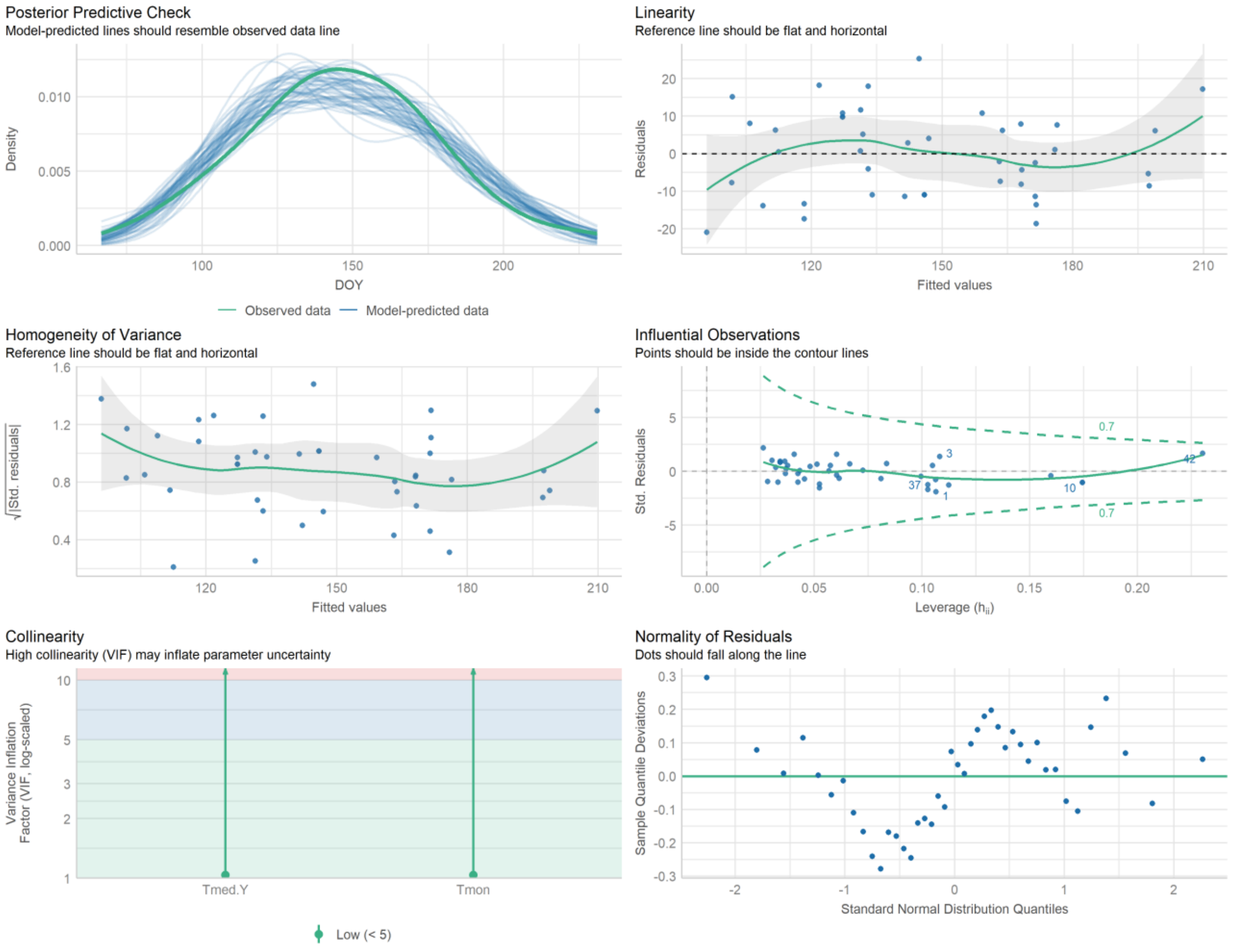

1.131. MLM - FBF - Ulex parviflorus

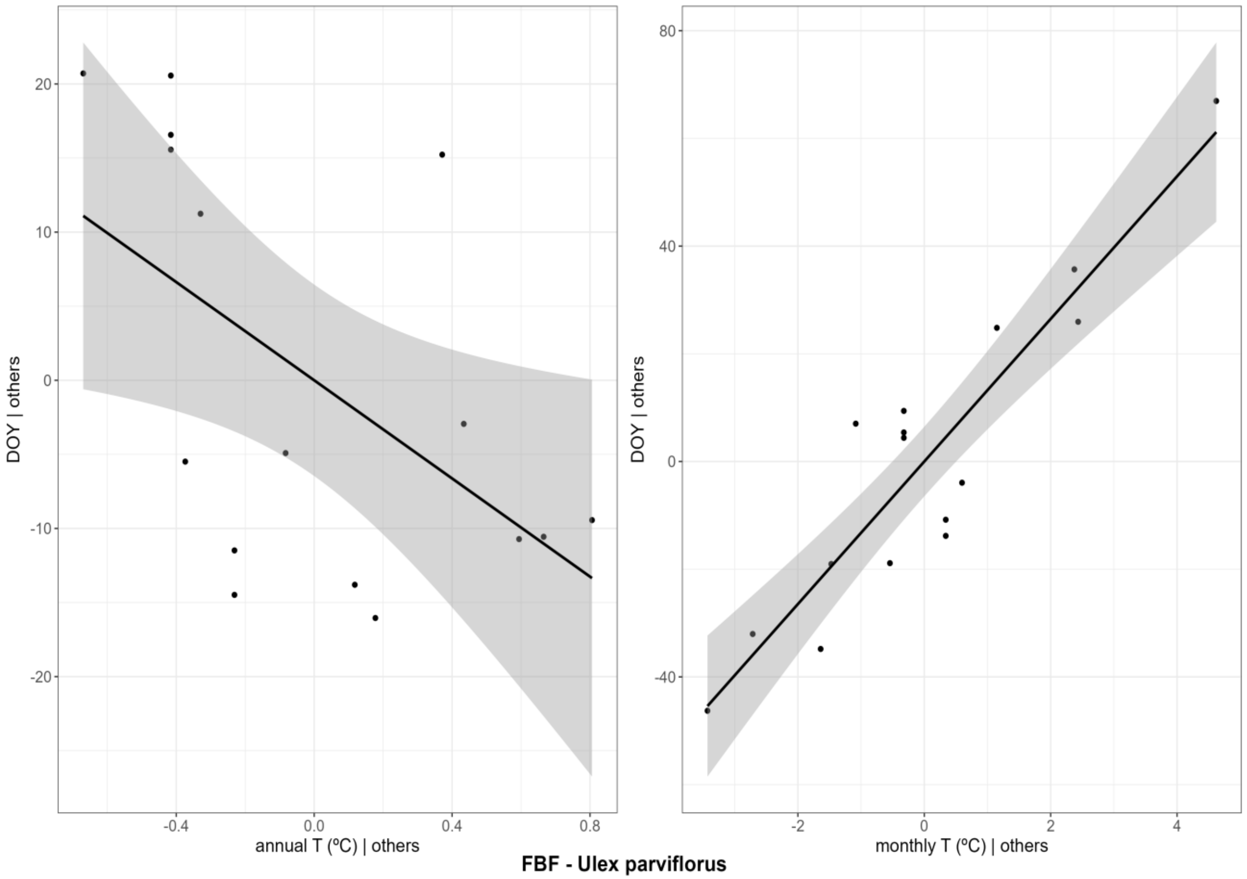

DOY = 179.44 (-16.56*annual T (ºC)) + (+13.24*monthly T (ºC))

1.131.1. Diagnostics - MLM - FBF - Ulex parviflorus

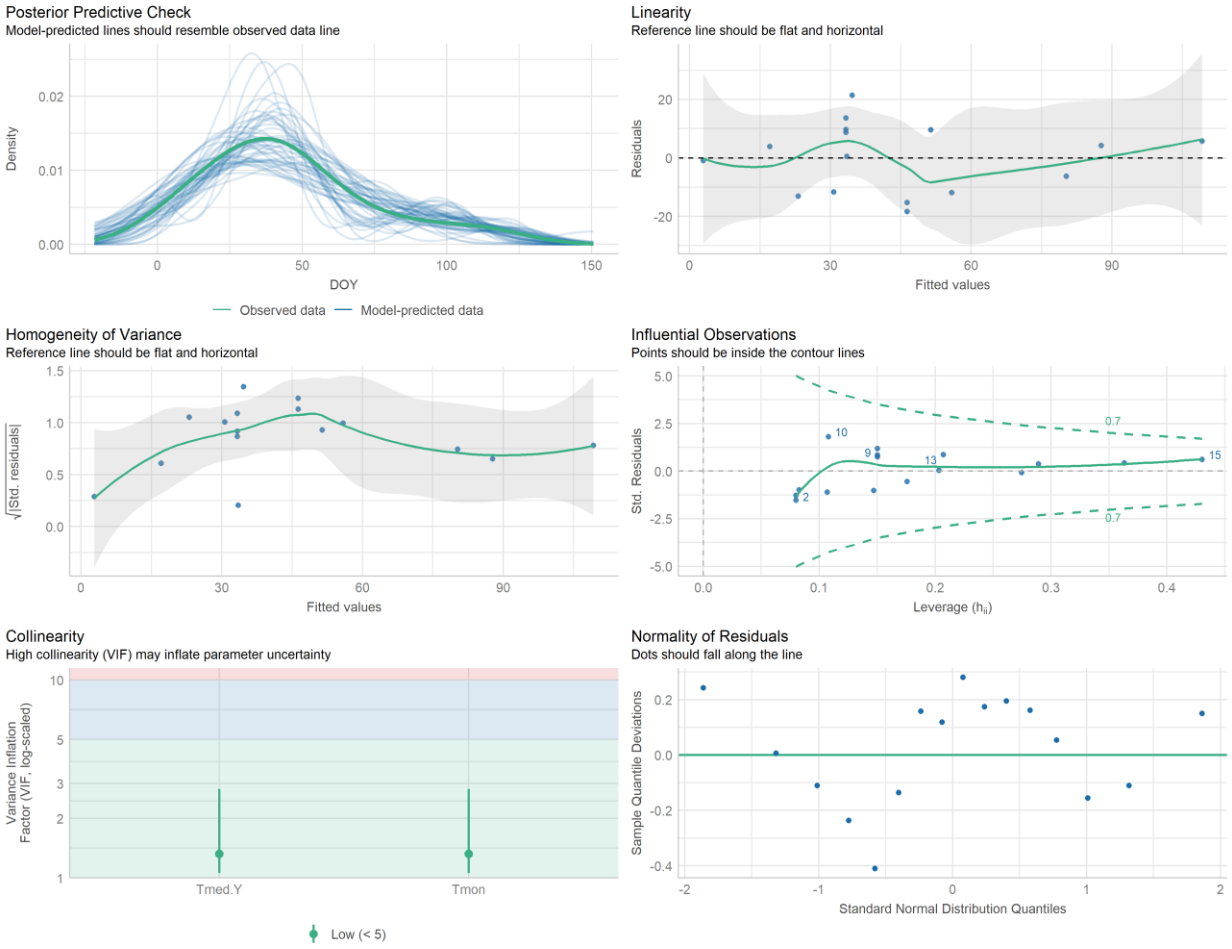

1.132. MLM - F - Ulex parviflorus

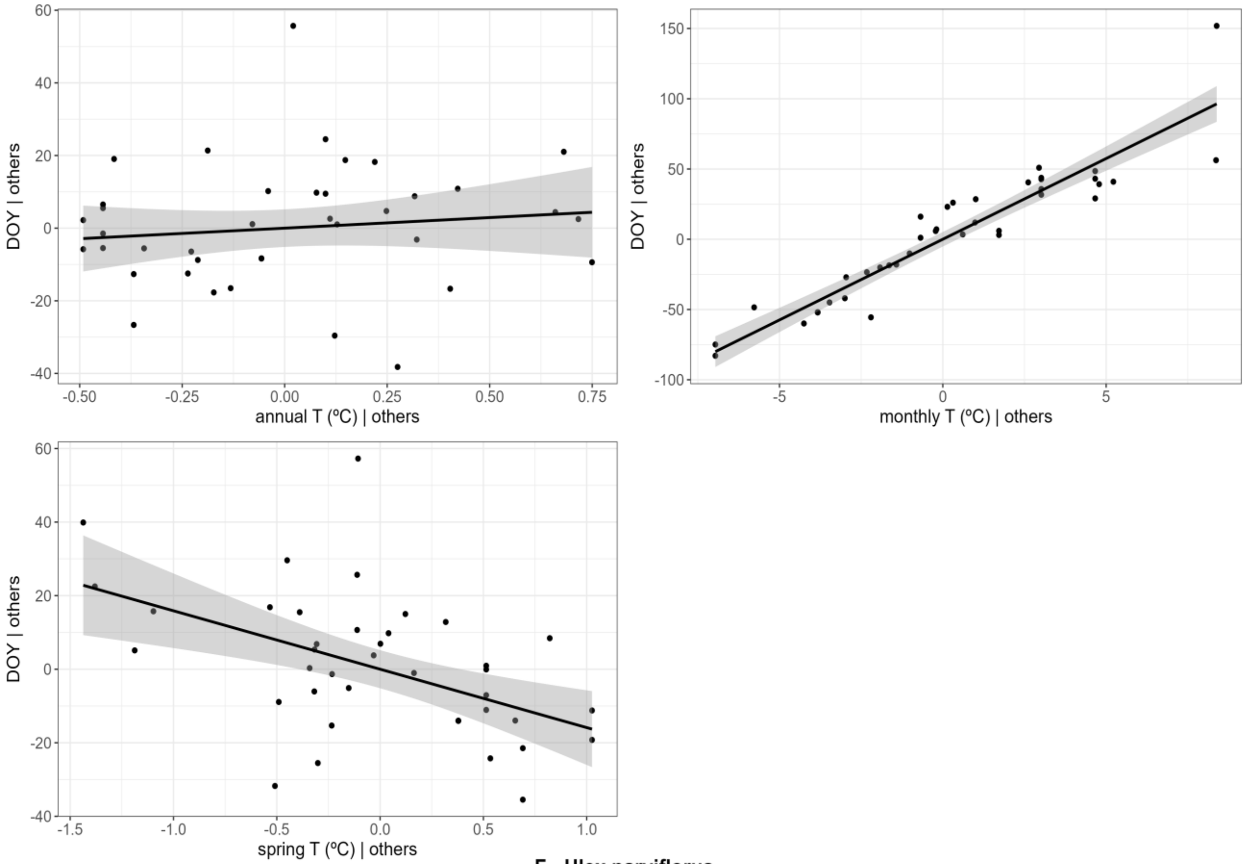

F - Ulex parviflorus

DOY = 75.09 (+5.82*annual T (ºC)) + (+11.49*monthly T (ºC)) + (-15.88*spring T (ºC))

1.132.1. Diagnostics - MLM - F - Ulex parviflorus

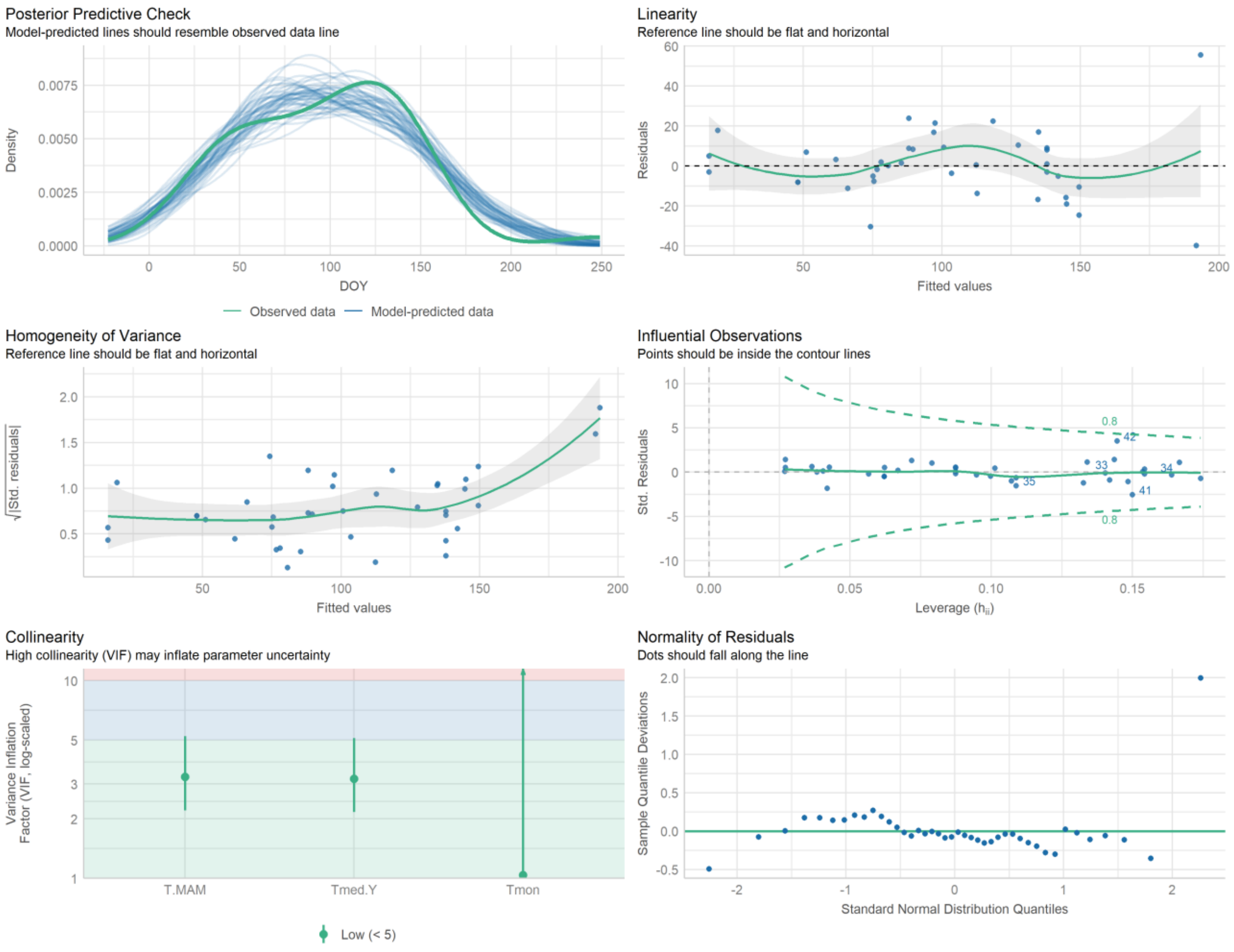

1.133. MLM - DVG - Ulex parviflorus

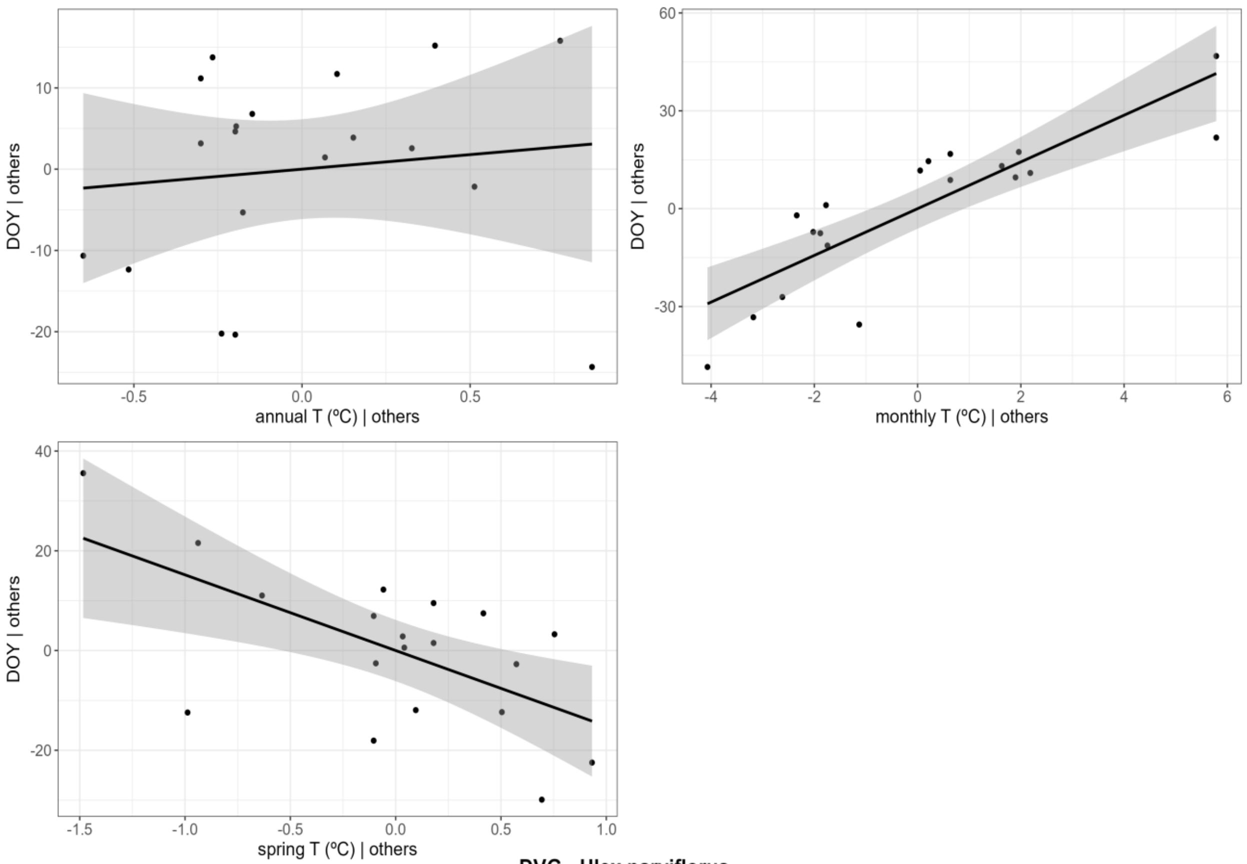

DVG - Ulex parviflorus

DOY = 173.82 (+3.58*annual T (ºC)) + (+7.16*monthly T (ºC)) + (-15.18*spring T (ºC))

1.133.1. Diagnostics - MLM - DVG - Ulex parviflorus

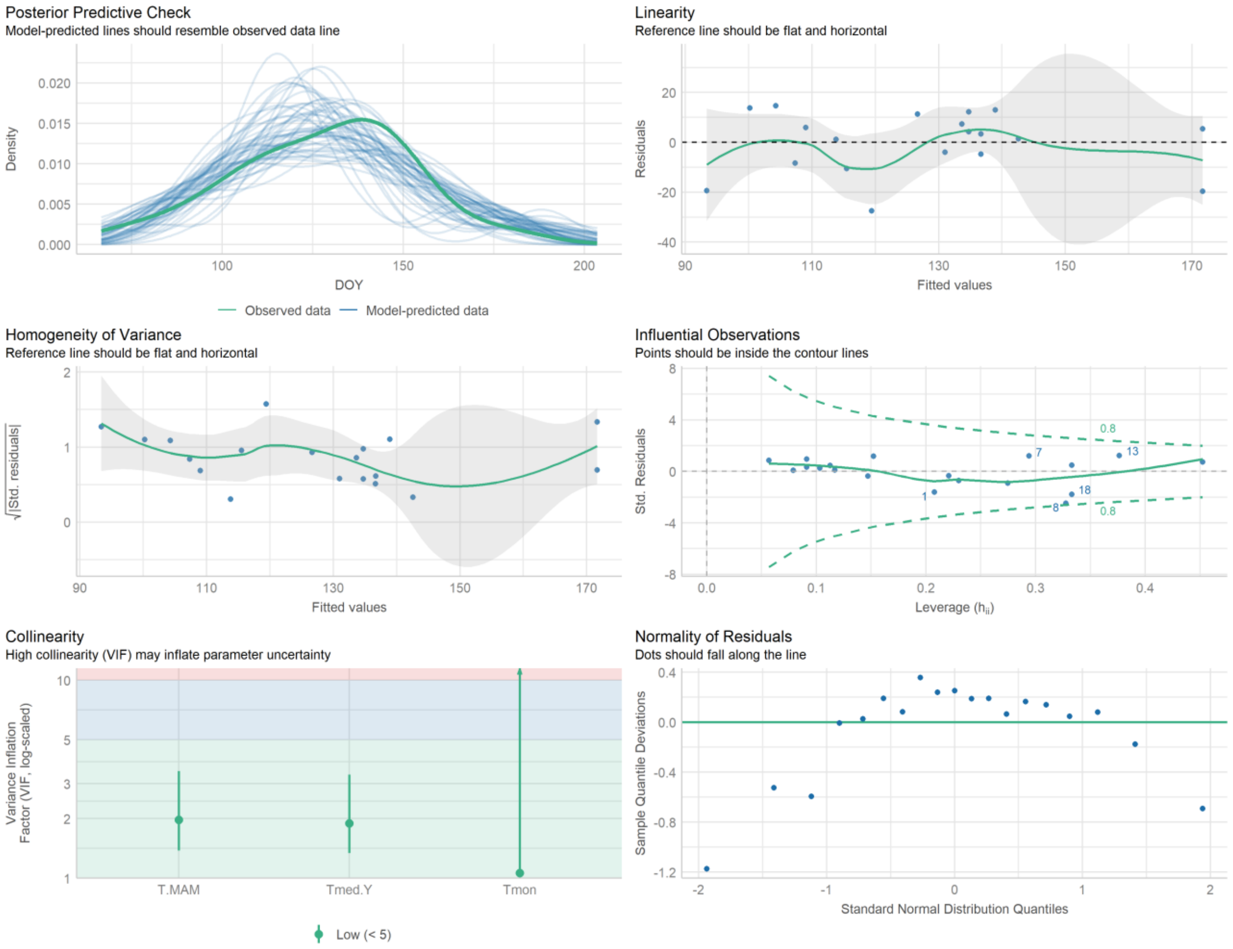

1.134. MLM - F - Viburnum tinus

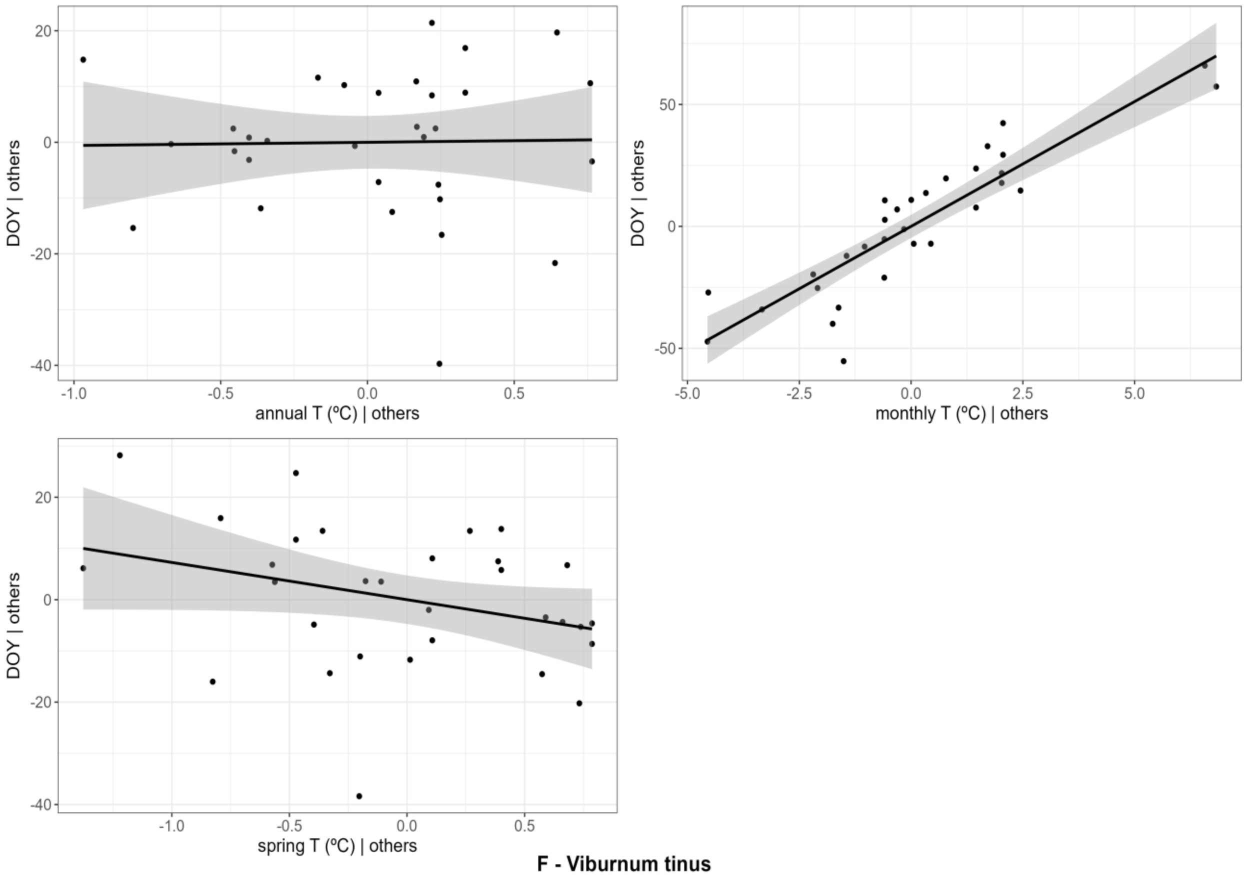

F - Viburnum tinus

DOY = 47.54 (+0.57*annual T (ºC)) + (+10.24*monthly T (ºC)) + (-7.27*spring T (ºC))

1.134.1. Diagnostics - MLM - F - Viburnum tinus

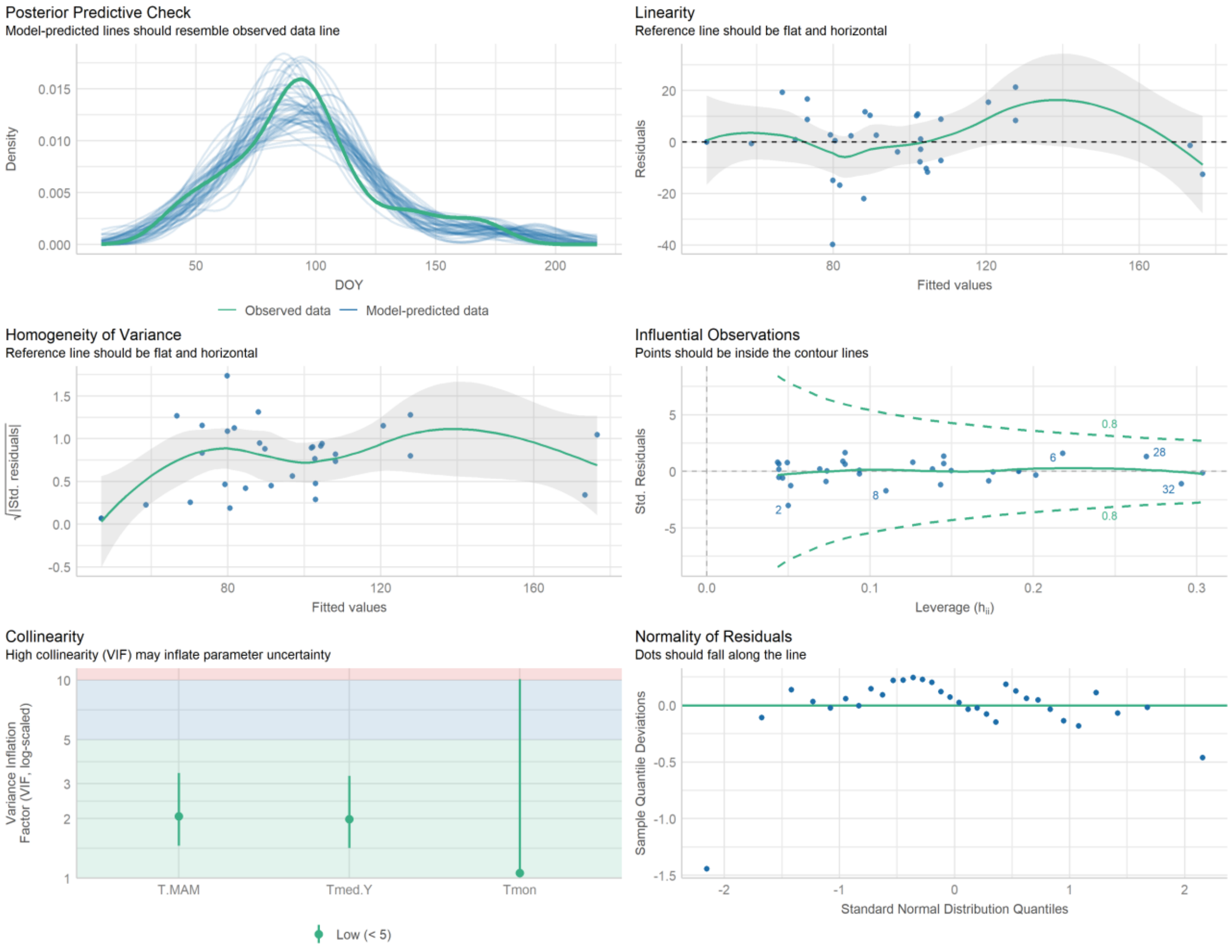

1.135. MLM - DVG - Viburnum tinus

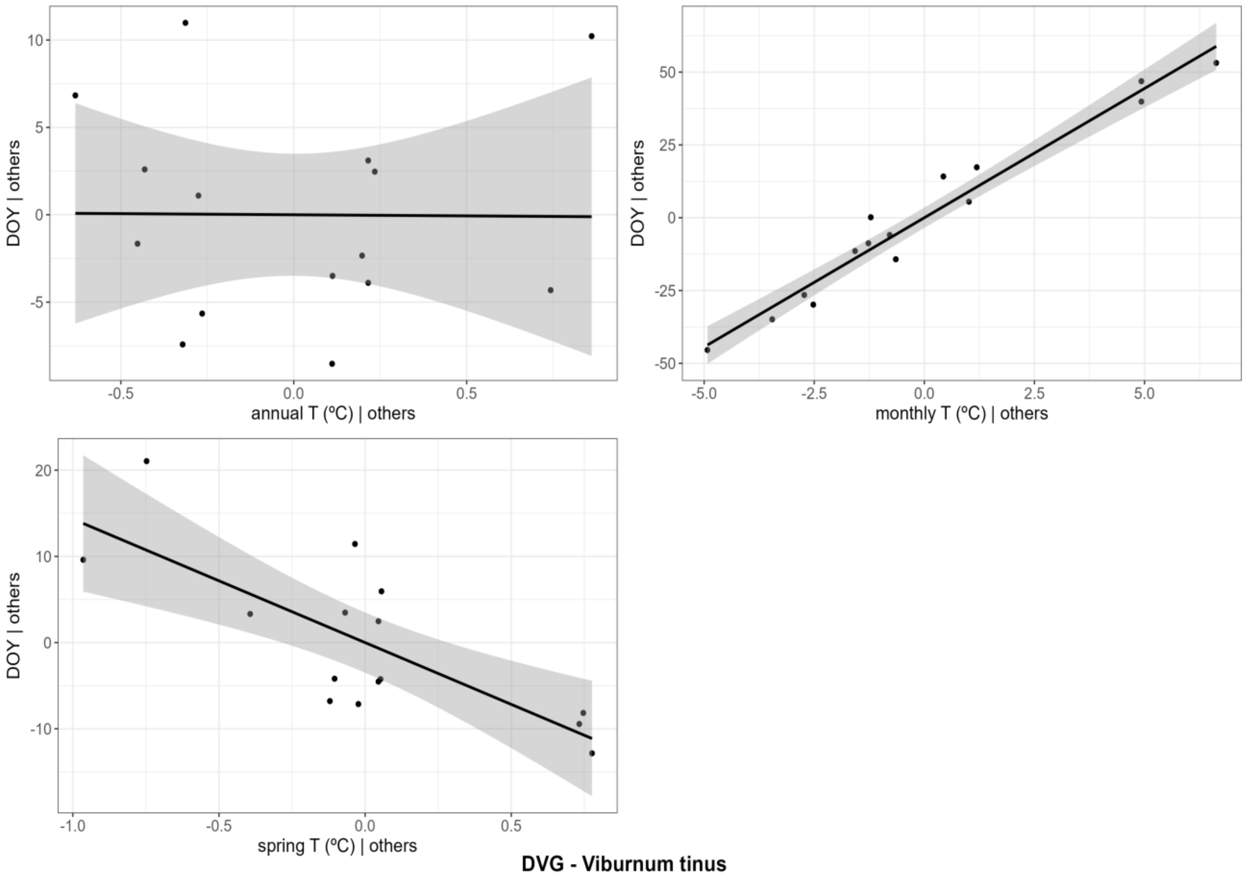

DVG - Viburnum tinus

DOY = 182.62 (-0.13*annual T (ºC)) + (+8.88*monthly T (ºC)) + (-14.33*spring T (ºC))

1.135.1. Diagnostics - MLM - DVG - Viburnum tinus

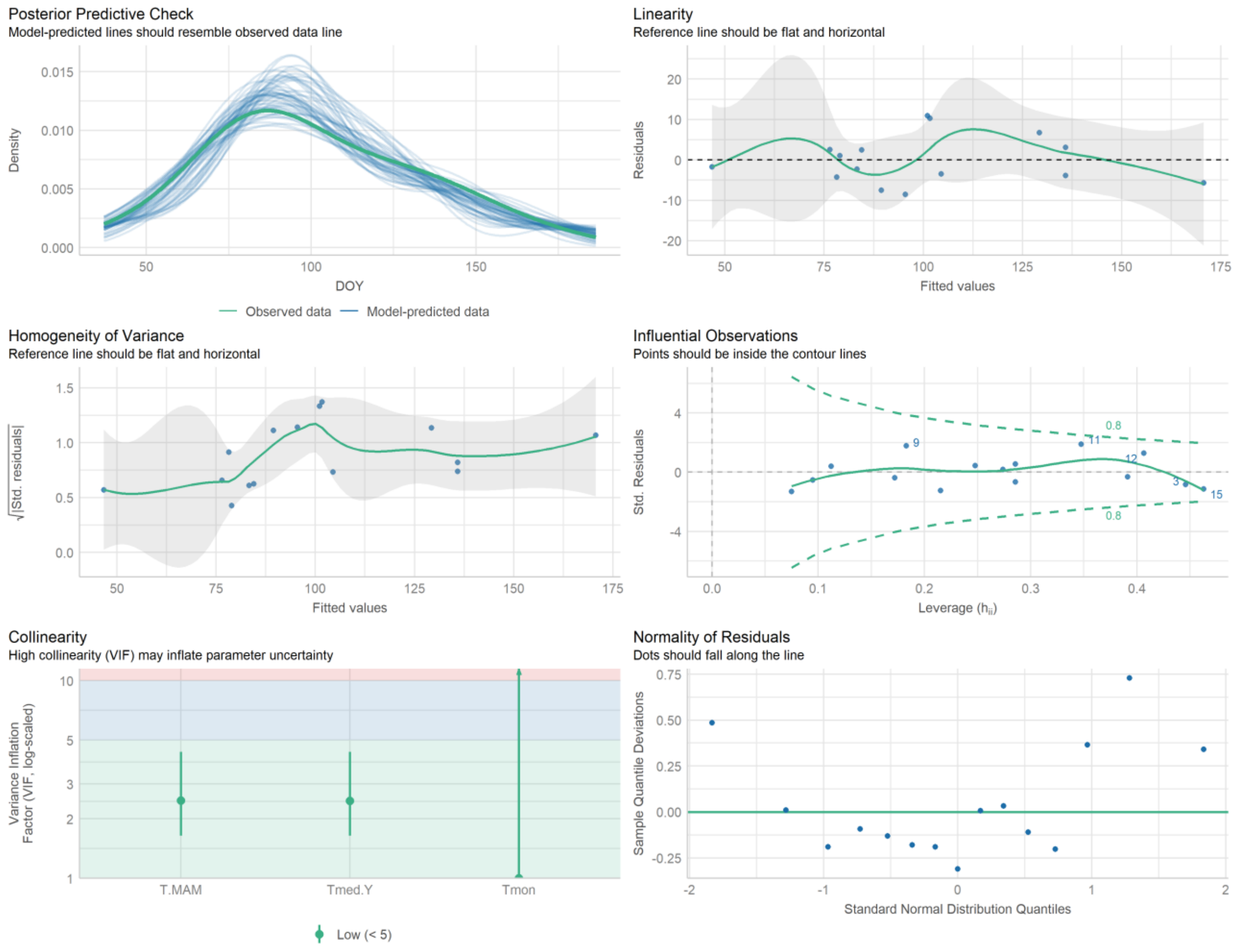

2. GAM Model Results

***In the following section, each taxon with >15 records in a single phenophase, has a figure which represents the generalized additive model trends with the final combination of climate variables for the model with best AIC (GAM), and its corresponding model diagnostic analysis (Diagnostics). FBF: preflowering, F: flowering, FS: fruiting, DVG: growth.**

Table S2. Generalized Additive Model results by taxon and phenophase. Preflowering: FBF, flowering: F, fruiting: FS, growth: DVG. Annual mean temperature: Tmed.Y, monthly temperature: Tmon, spring temperature: T.MAM, total annual precipitation: Pannual_Y, monthly precipitation: Pmon, spring precipitation: P.MAM. BP test: studentized Breusch-Pagan Test.

Phenophase	Taxon	Intercept	Adj. R^2	Dev_Exp	p_value_bp_test	Smooth_Term_Tmed.Y	p_value_Tmed.Y	EDF_Tmed.Y	Smooth_Term_Pannual.Y	p_value_Pannual.Y	EDF_Pannual.Y	Smooth_Term_Tmon	p_value_Tmon	EDF_Tmon	Smooth_Term_Pmon	p_value_Pmon	EDF_Pmon	Smooth_Term_T.MAM	p_value_T.MAM	EDF_T.MAM	Smooth_Term_P.MAM	p_value_P.MAM	EDF_P.MAM
F	*Salvia rosmarinus*	158.440	0.346	43.937	0.059	-19.933	0.016	2.138	-2.435	0.119	0.617	161.940	0.095	0.680	16.325	0.850	0.000						
F	*Lavandula stoechas*	106.465	0.829	83.680	0.018	0.000	0.539	0.000	0.000	0.676	0.000	0.000	0.000	2.561	0.000	0.148	0.523	0.000	0.000	1.238	0.000	0.225	0.306
F	*Thymbra capitata*	172.969	0.831	85.932	0.065	-0.080	0.007	0.911	-0.004	0.415	0.000	0.243	0.000	1.256	-4.494	0.000	3.077						
F	*Abies pinsapo*	139.	0.8	90.668	0.705	-0.296	0.000	1.028	0.041	0.000	1.019	-0.301	0.000	1.172	6.861	0.875	0.000	0.274	0.843	0.000	0.213	0.000	1.007

		105	78																				
F	*Sideritis incana*	148.417	0.719	74.090	0.250	0.000	0.455	0.000	0.000	0.506	0.000	0.000	0.000	1.318	0.000	0.286	0.037	0.000	0.161	0.486	0.000	0.004	0.906
FBF	*Thymbra capitata*	146.333	0.959	96.857	0.123	-0.183	0.001	1.055	0.124	0.814	0.000	0.666	0.000	2.836	-6.555	0.010	0.923						
FS	*Crataegus monogyna*	163.560	0.651	68.995	0.425	0.000	0.498	0.000	0.000	0.181	0.439	0.026	0.000	1.179	0.000	0.235	0.241	0.000	0.040	0.831	0.021	0.382	0.000
FS	*Hormathophylla spinosa*	191.600	0.431	49.509	0.101	0.408	0.004	0.974	-0.204	0.098	0.672	0.030	0.001	1.070	-12.048	0.399	0.000	1.277	0.395	0.000	-0.071	0.464	0.000
FS	*Crepis oporinoides*	208.333	0.448	51.968	0.419	0.048	0.018	0.893				-0.197	0.013	0.930									
DVG	*Thymbra capitata*	153.600	0.986	99.224	0.043	0.848	0.000	1.299	-0.254	0.027	0.871	-0.167	0.000	1.604	-20.445	0.000	3.272	0.653	0.514	0.000	-0.080	0.000	1.182

DVG	*Crataegus monogyna*	139.423	0.961	97.254	0.247	-0.694	0.000	1.847	-1.070	0.007	0.920	0.010	0.000	3.050	-9.022	0.003	0.944	-9.162	0.387	0.000	-0.065	0.088	0.701
DVG	*Crataegus granatensis*	150.909	0.993	99.632	0.470	-3.037	0.000	2.186	-0.482	0.000	1.134	0.181	0.000	3.531	-6.367	0.000	1.378	-10.084	0.000	1.056	0.243	0.037	0.000
DVG	*Prunus prostrata*	167.892	0.841	85.686	0.311	-0.054	0.000	1.150	-0.003	0.567	0.000	0.000	0.000	1.568	-10.078	0.002	0.946	-0.075	0.521	0.000	0.000	0.549	0.000
DVG	*Cotoneaster granatensis*	177.706	0.696	75.787	0.664	-0.006	0.192	0.402	0.015	0.043	0.791	0.048	0.010	0.930	-1.713	0.932	0.000	-0.039	0.022	0.871	-0.095	0.253	0.255

2.1. GAM - F - Abies pinsapo

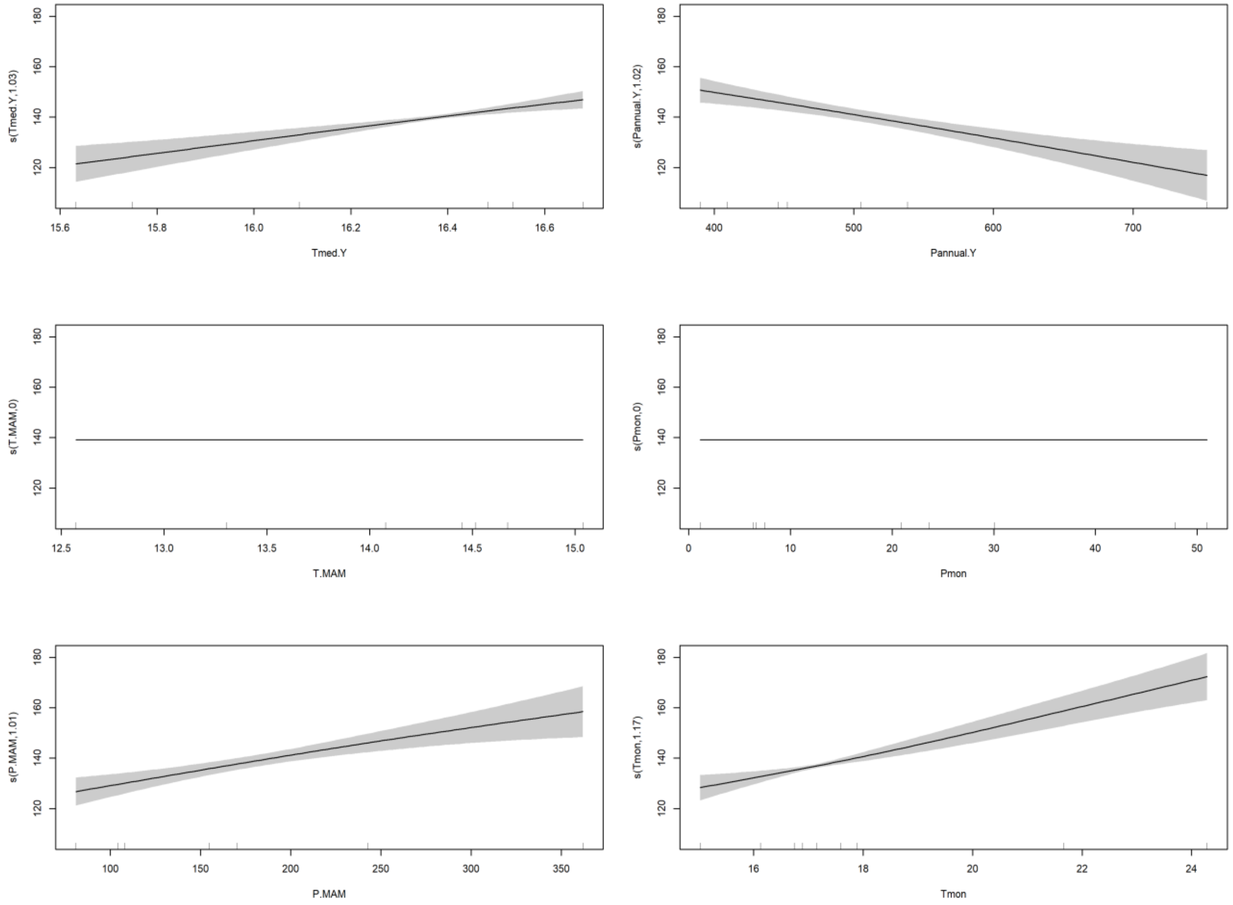

2.1.1. Diagnostics - GAM - F - Abies pinsapo

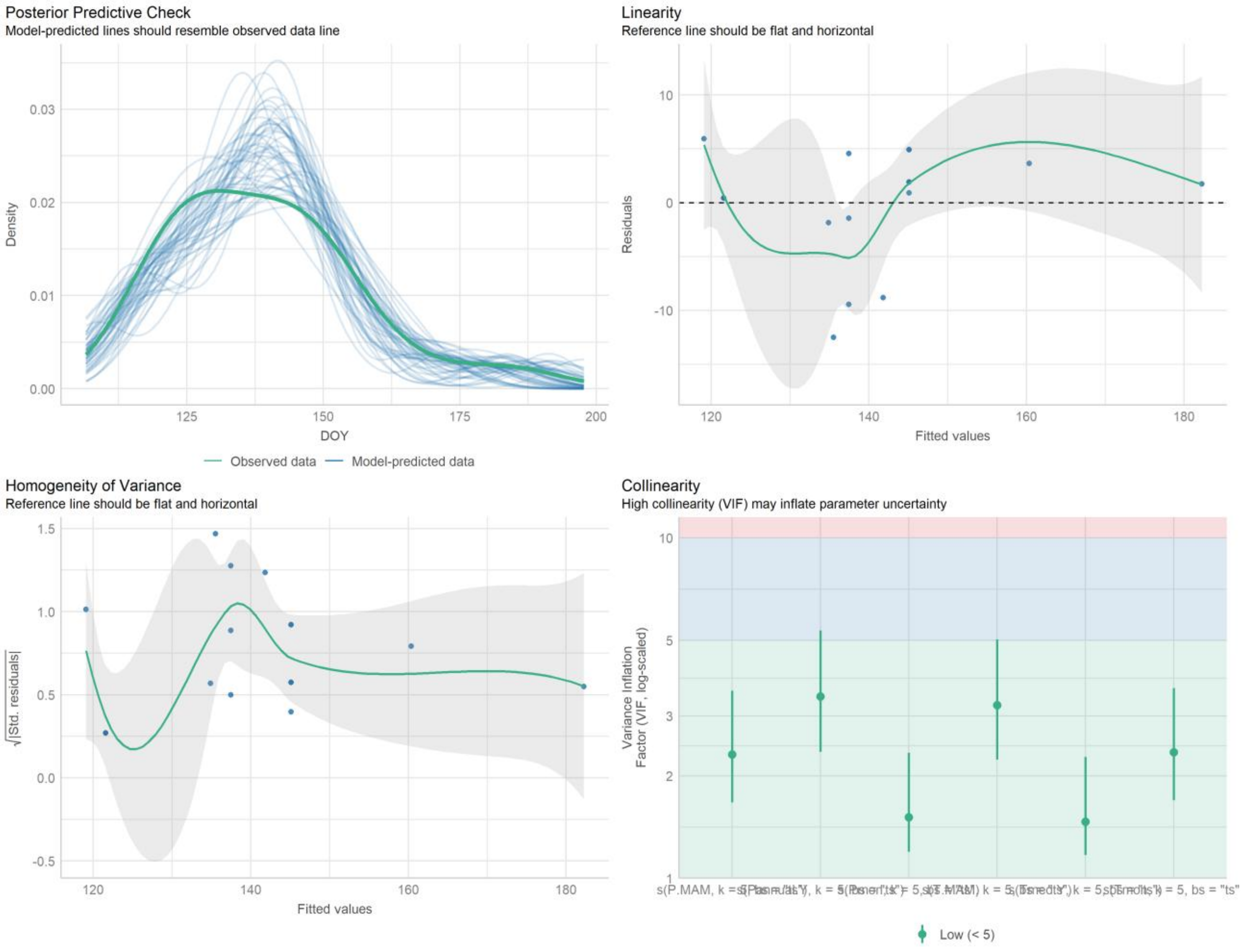

2.2. GAM - DVG - Cotoneaster granatensis

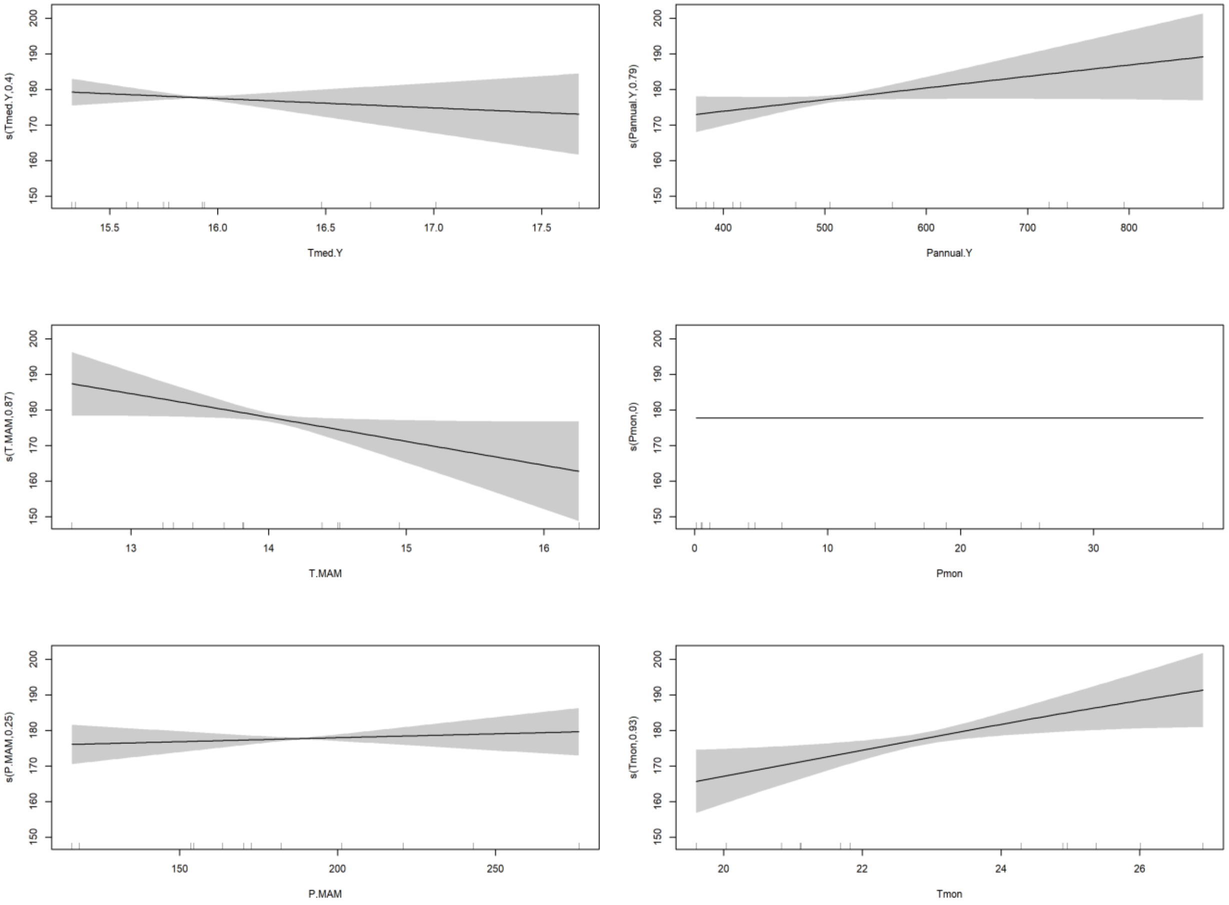

2.2.1. Diagnostics - GAM - DVG - Cotoneaster granatensis

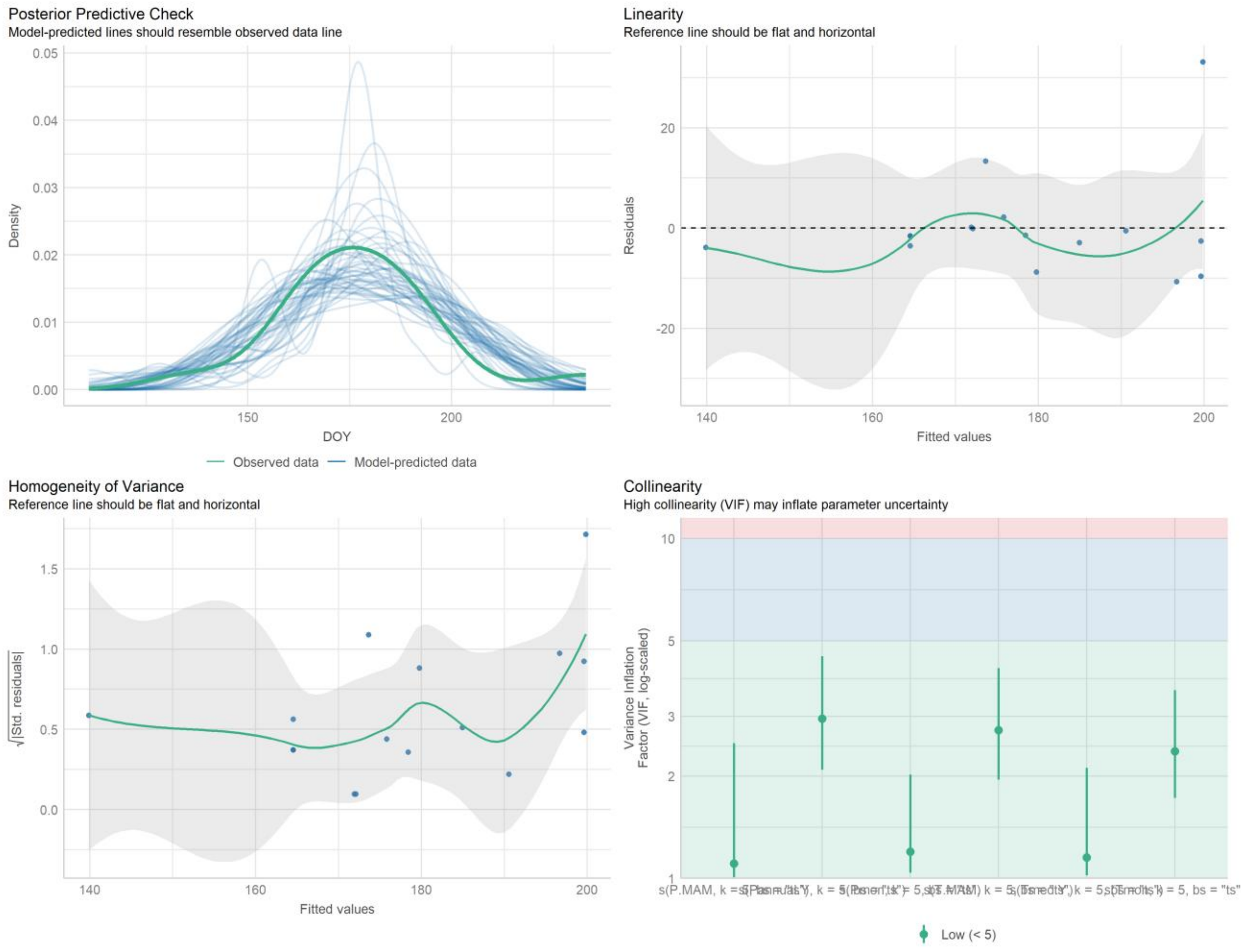

2.3. GAM - DVG - Crataegus granatensis

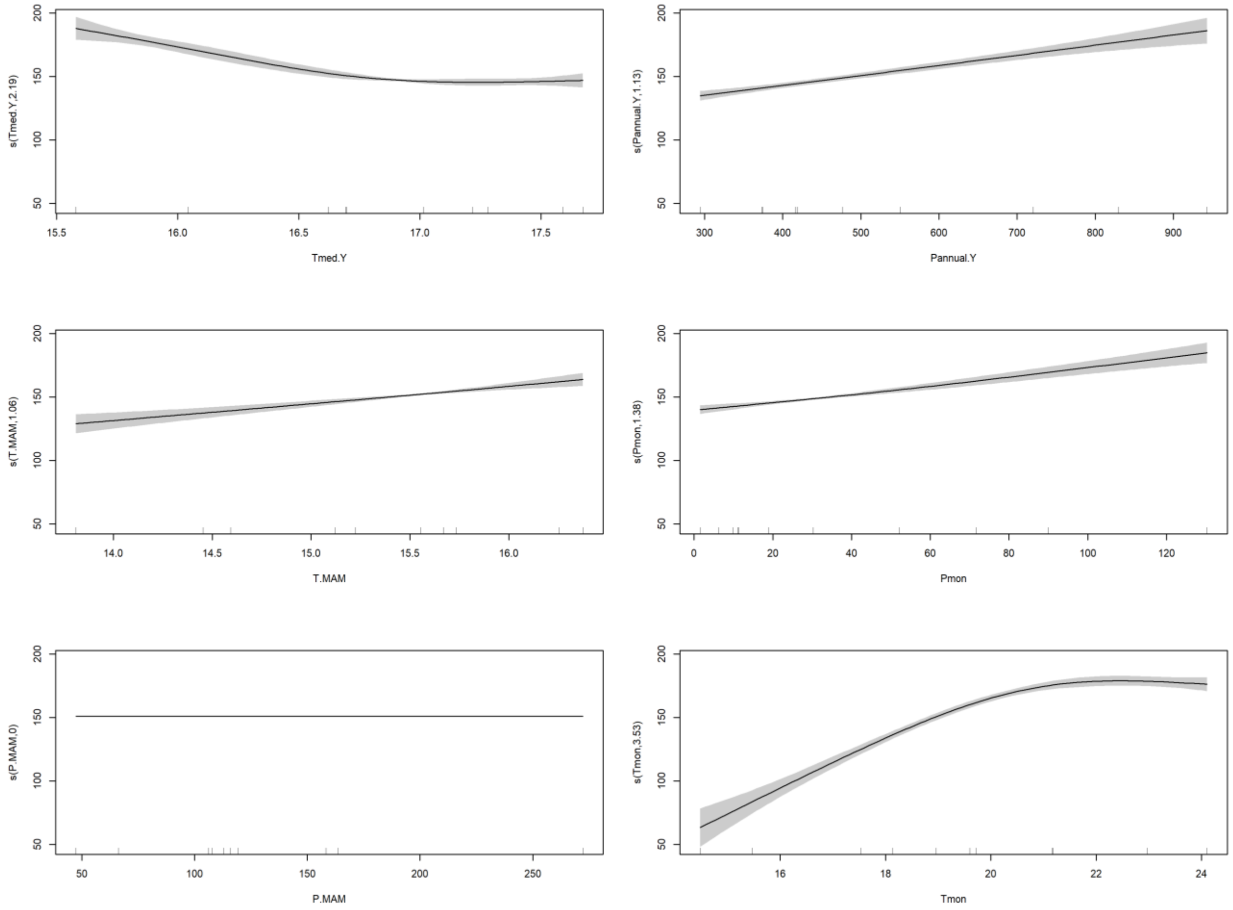

2.3.1. Diagnostics - GAM - DVG - Crataegus granatensis

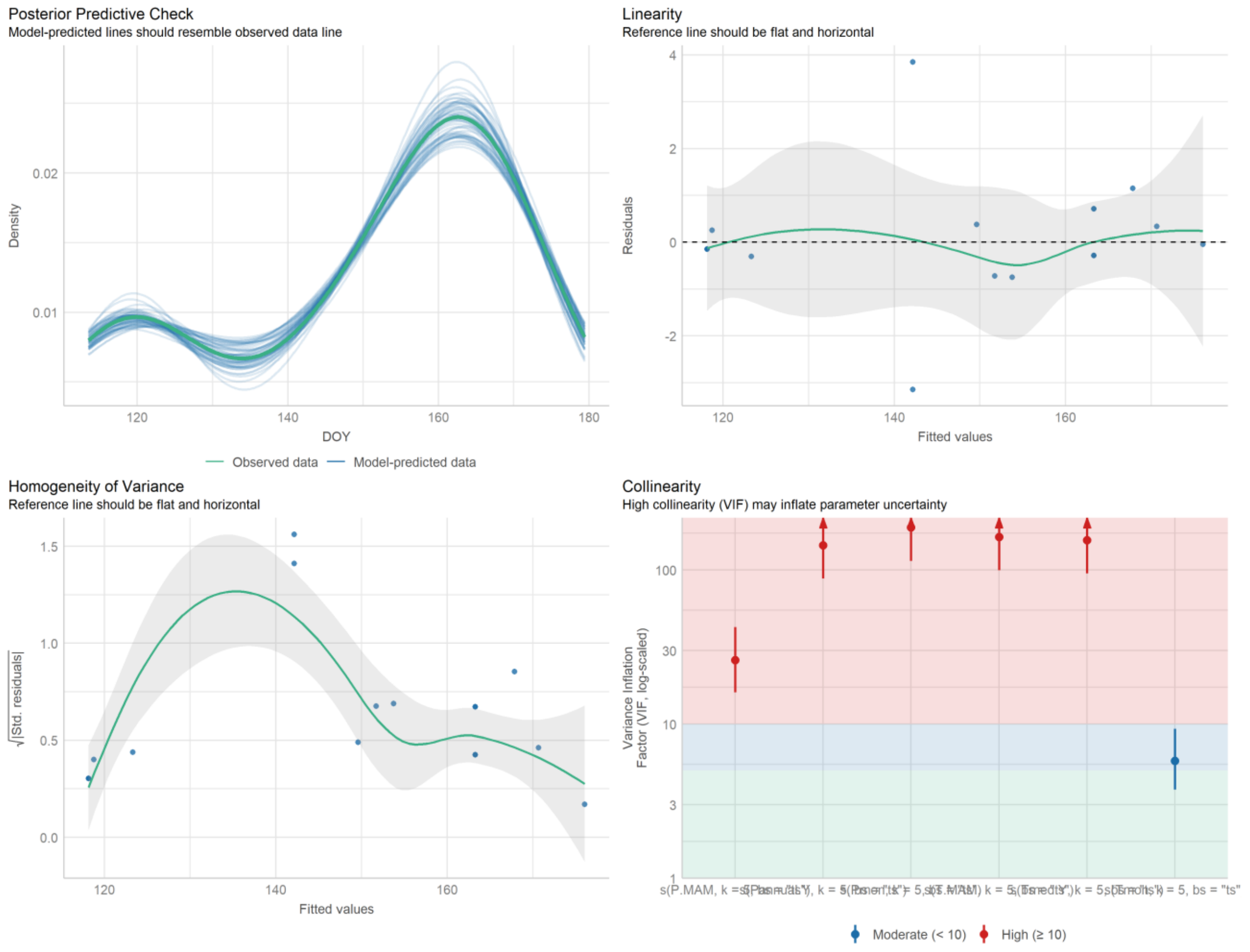

2.4. GAM - FS - Crataegus monogyna

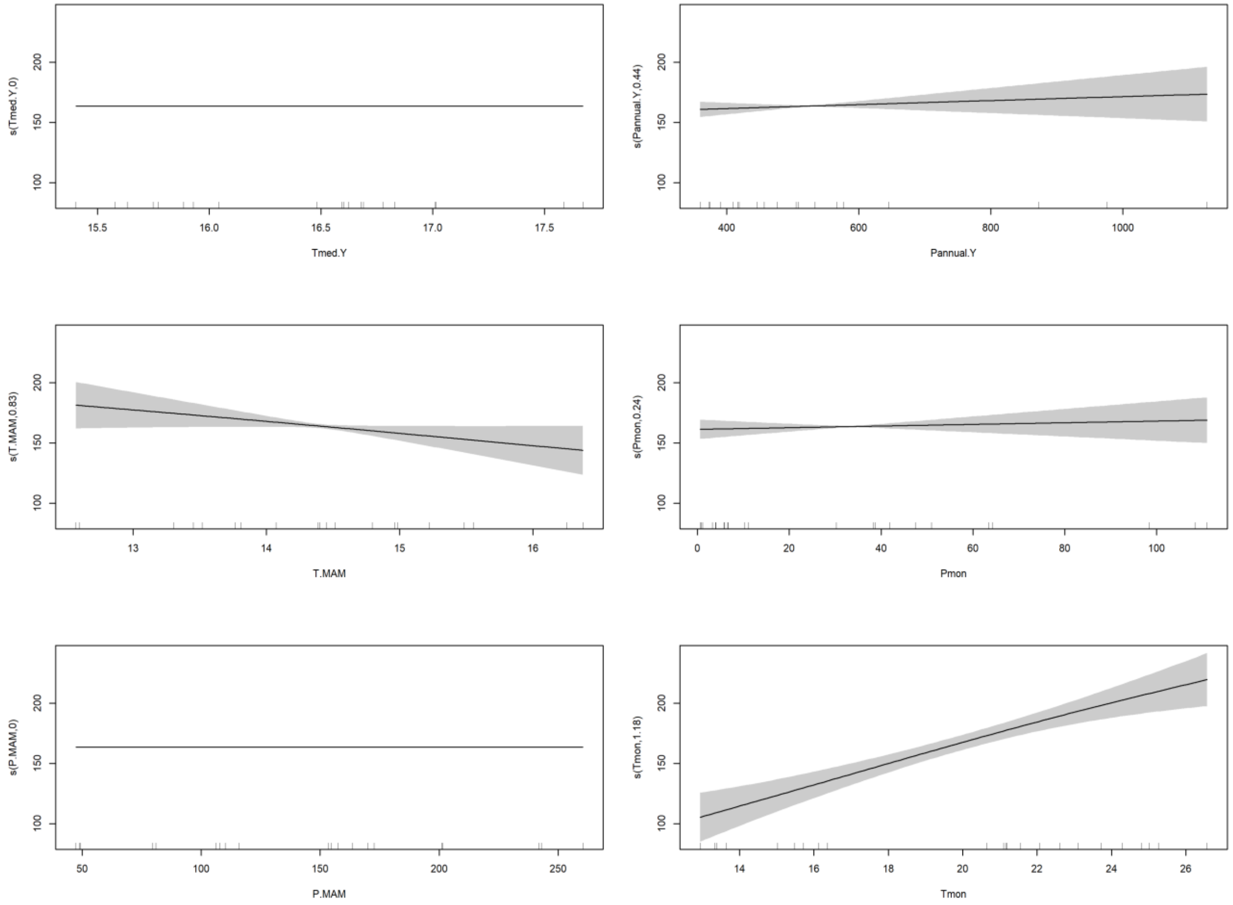

2.4.1. Diagnostics - GAM - FS - Crataegus monogyna

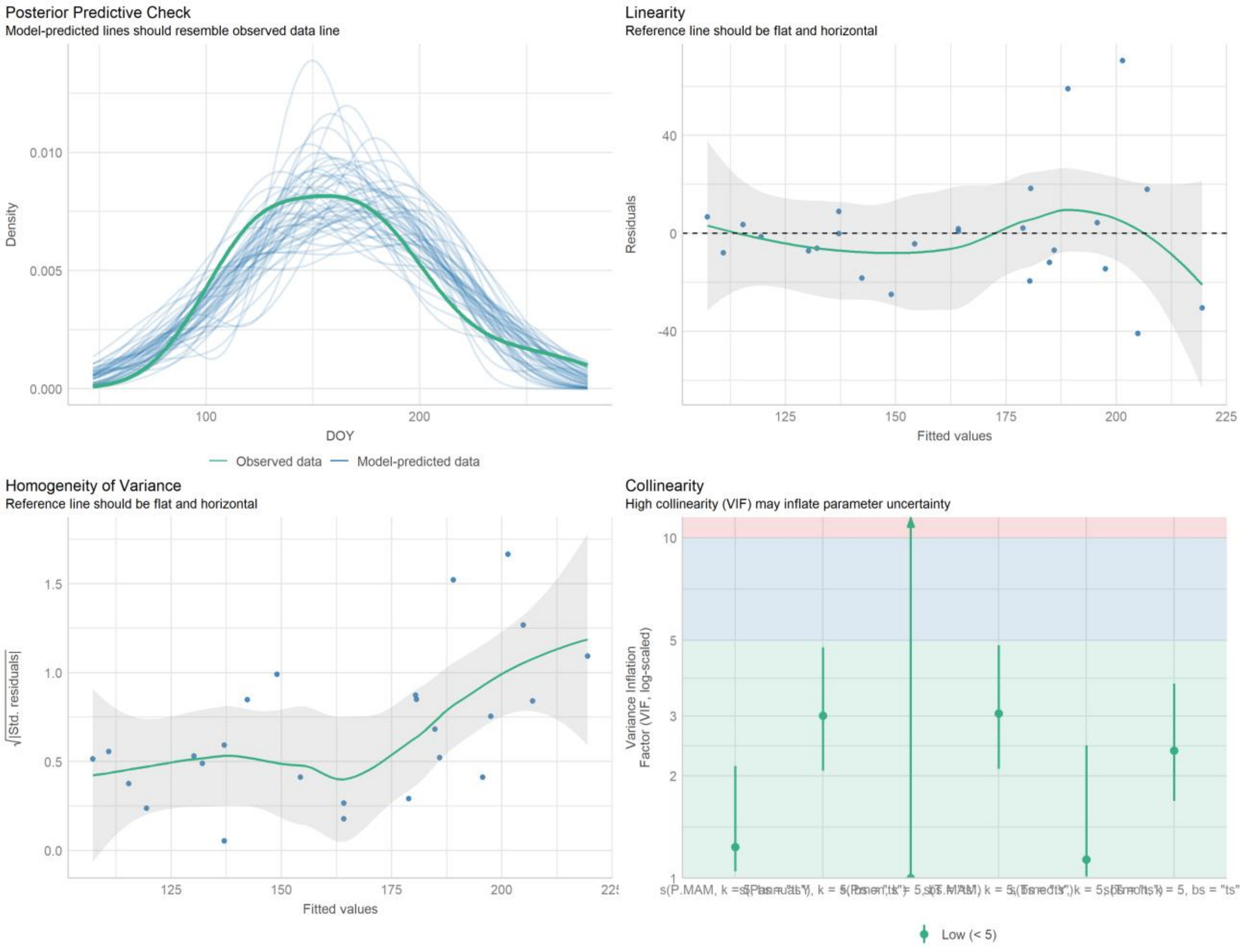

2.5. GAM - DVG - Crataegus monogyna

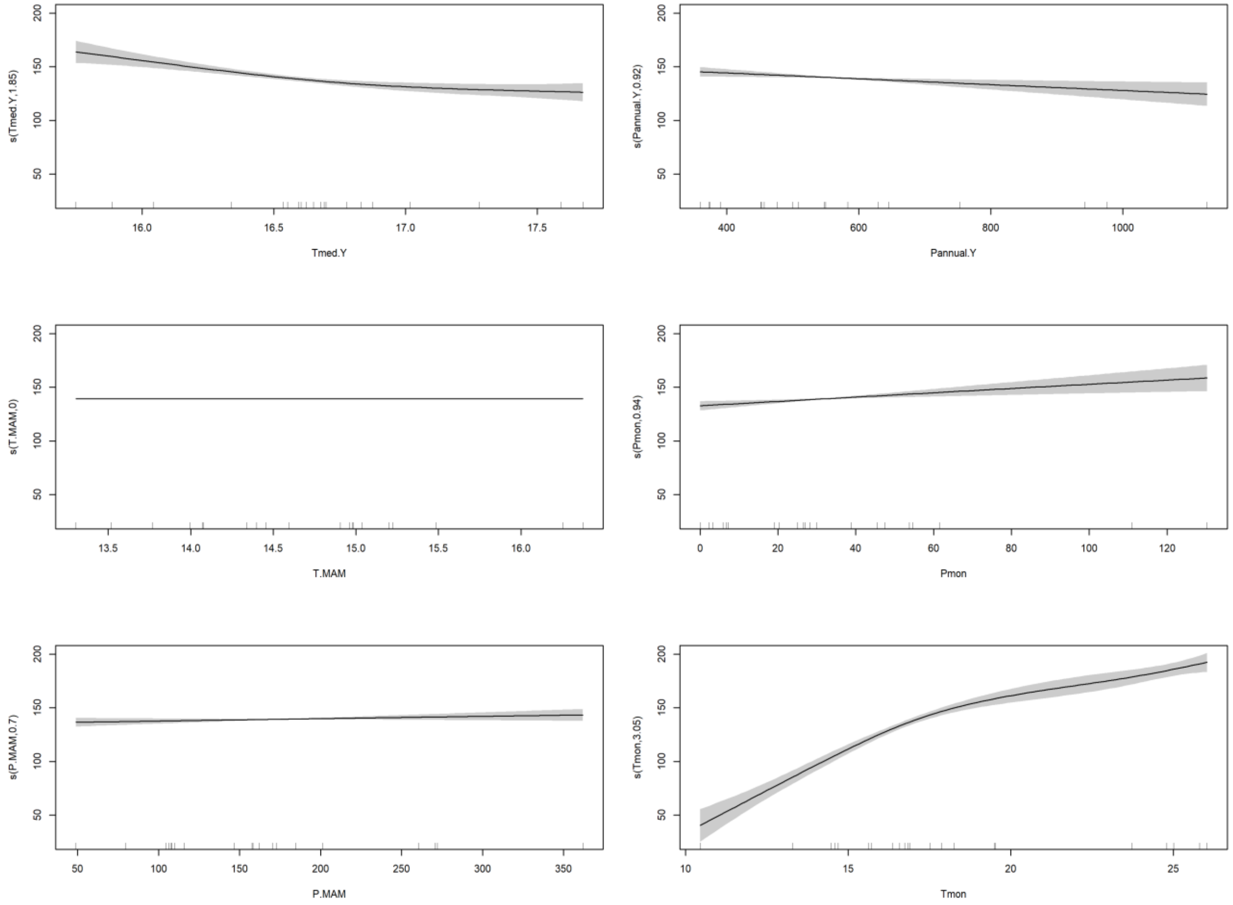

2.5.1. Diagnostics - GAM - DVG - Crataegus monogyna

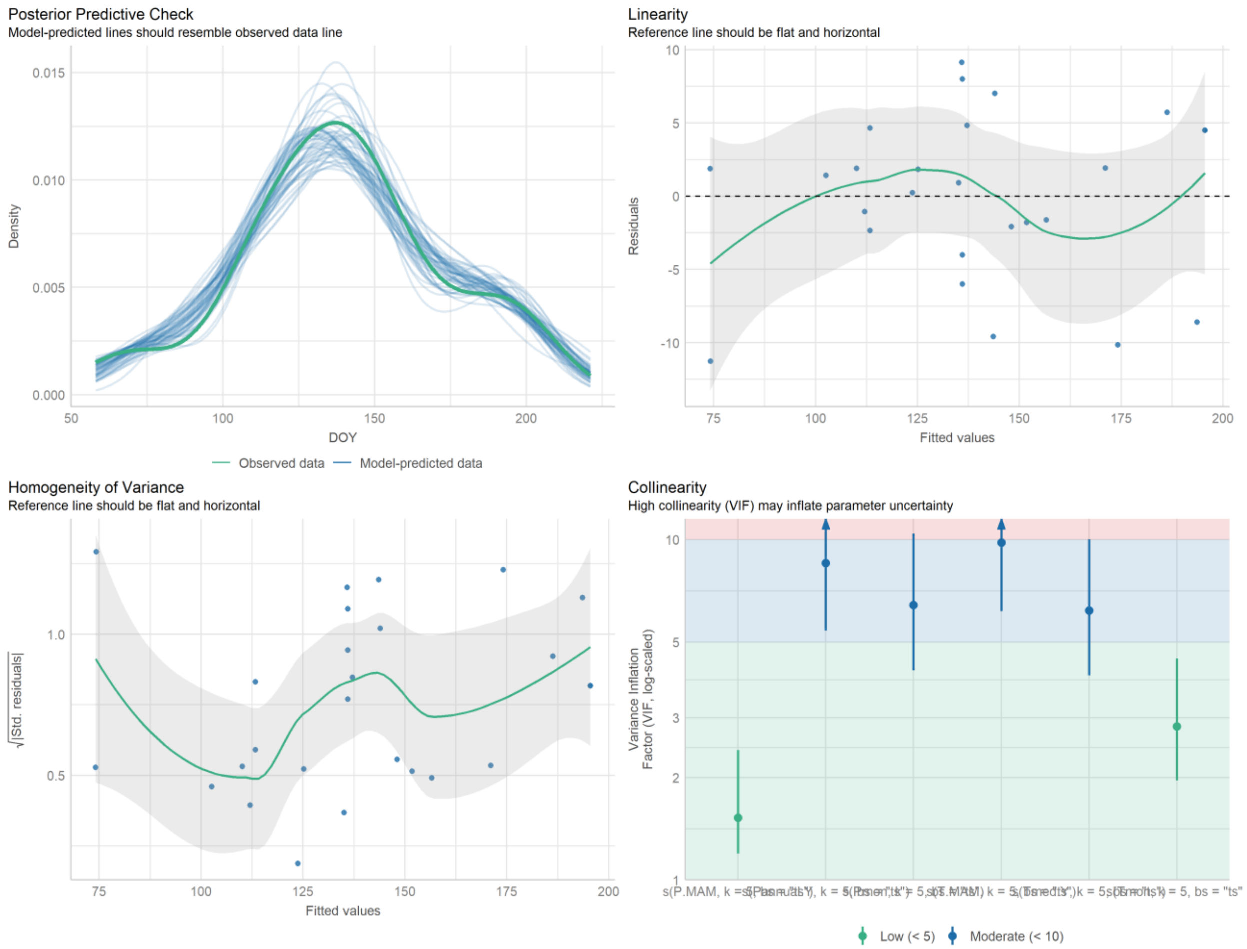

2.6. GAM - FS - Crepis oporinoides

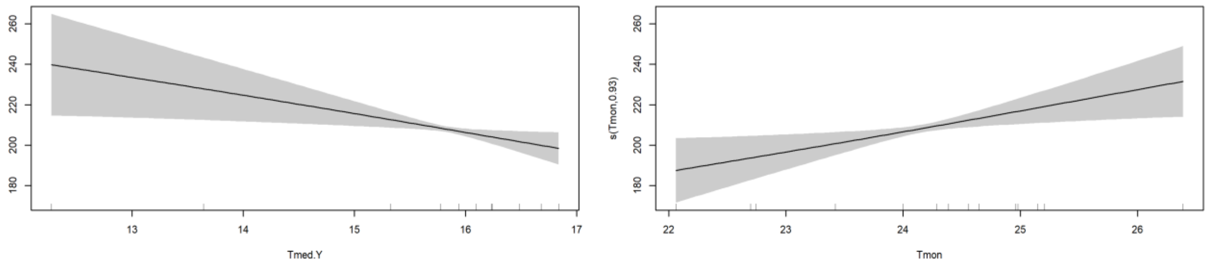

2.6.1. Diagnostics - GAM - FS - Crepis oporinoides

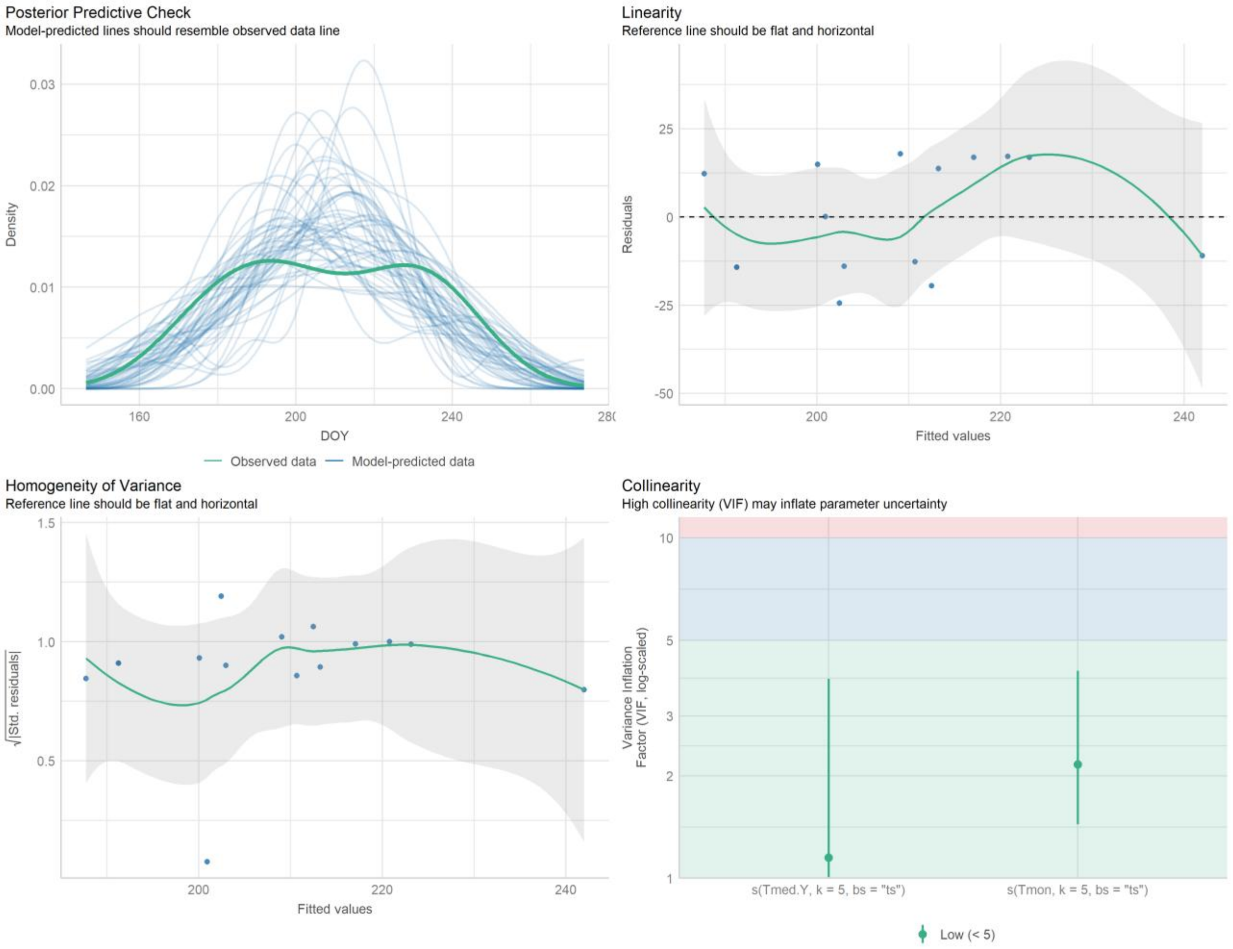

2.7. GAM - FS - Hormathophylla spinosa

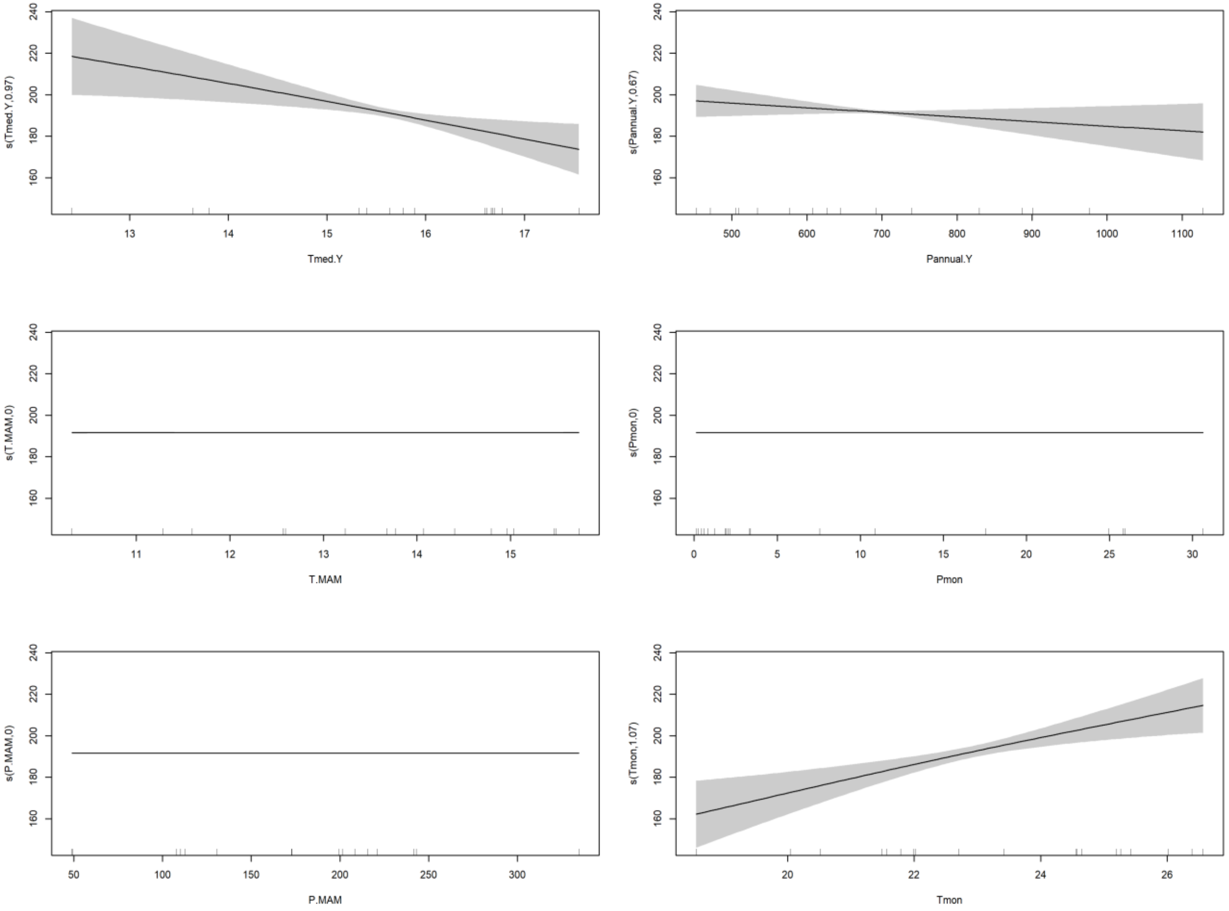

2.7.1. Diagnostics - GAM - FS - Hormathophylla spinosa

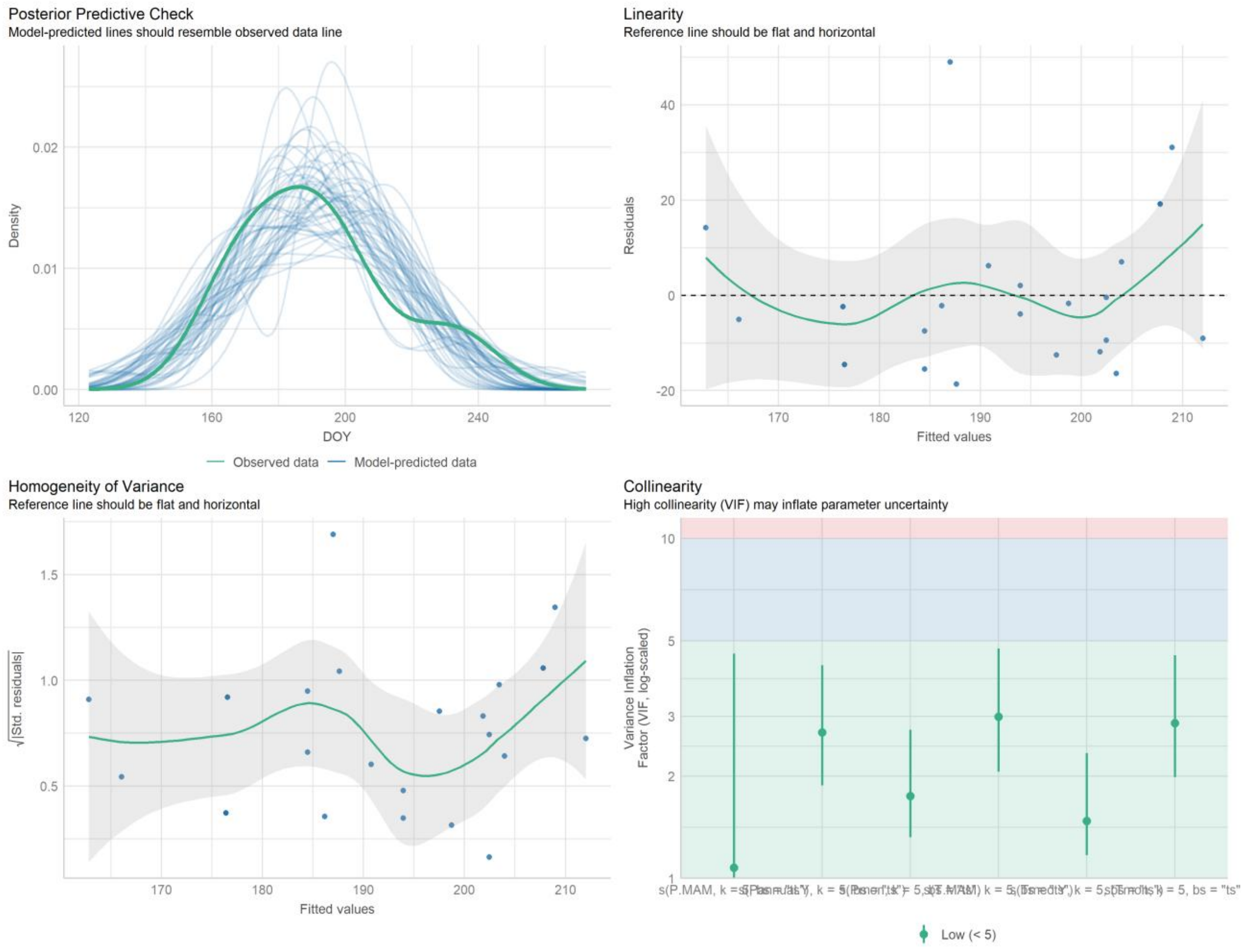

2.8. GAM - F - Lavandula stoechas

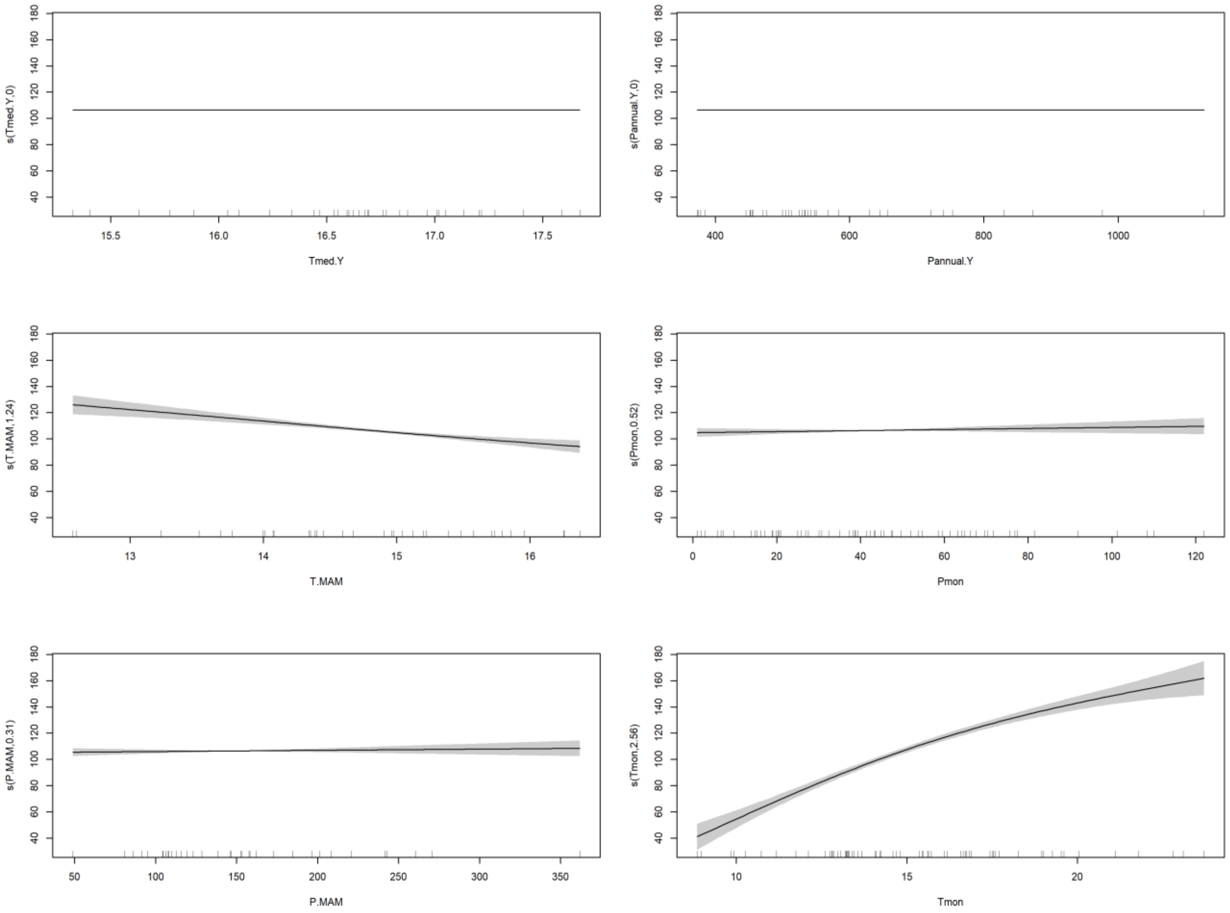

2.8.1. Diagnostics - GAM - F - Lavandula stoechas

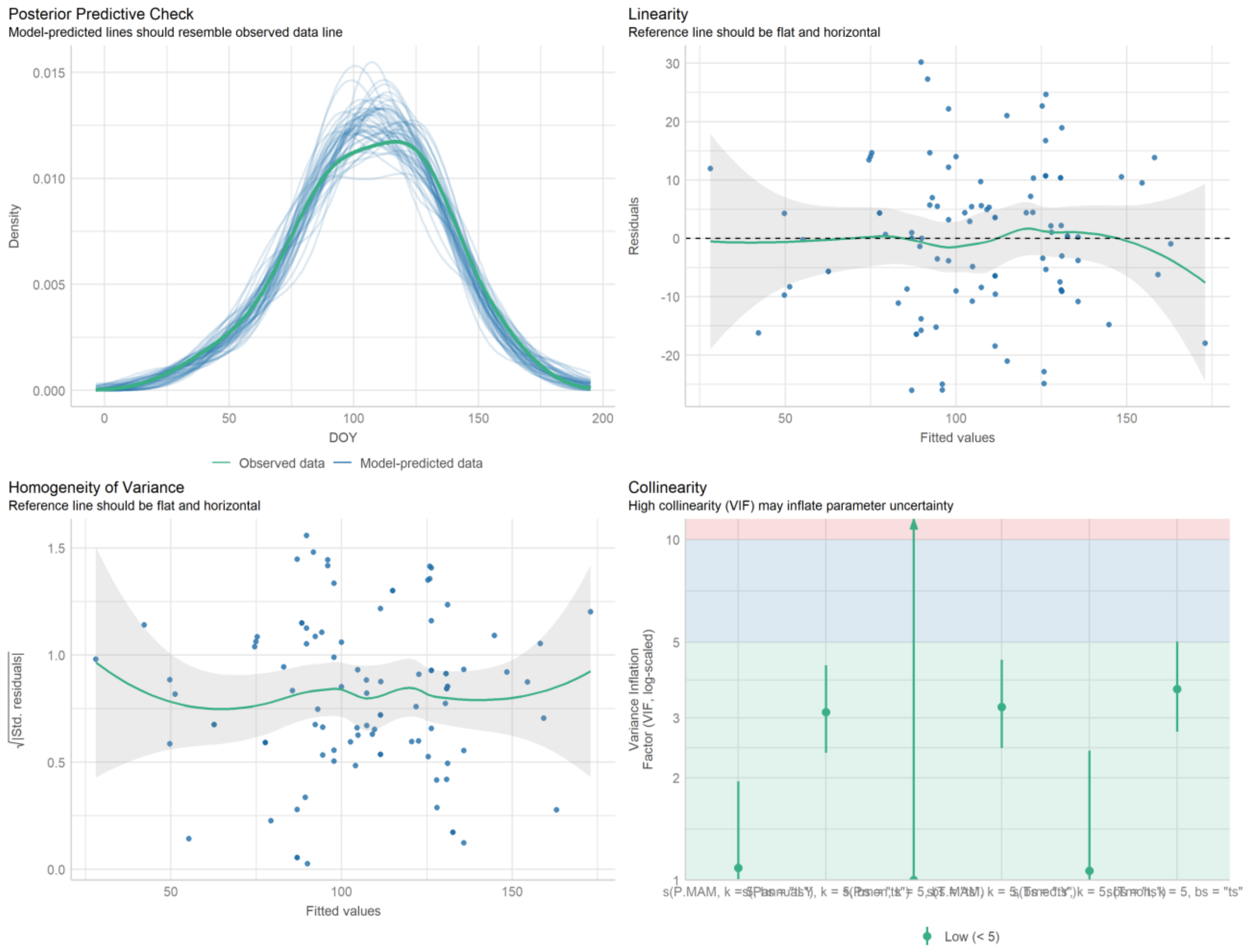

2.9. GAM - DVG - Prunus prostrata

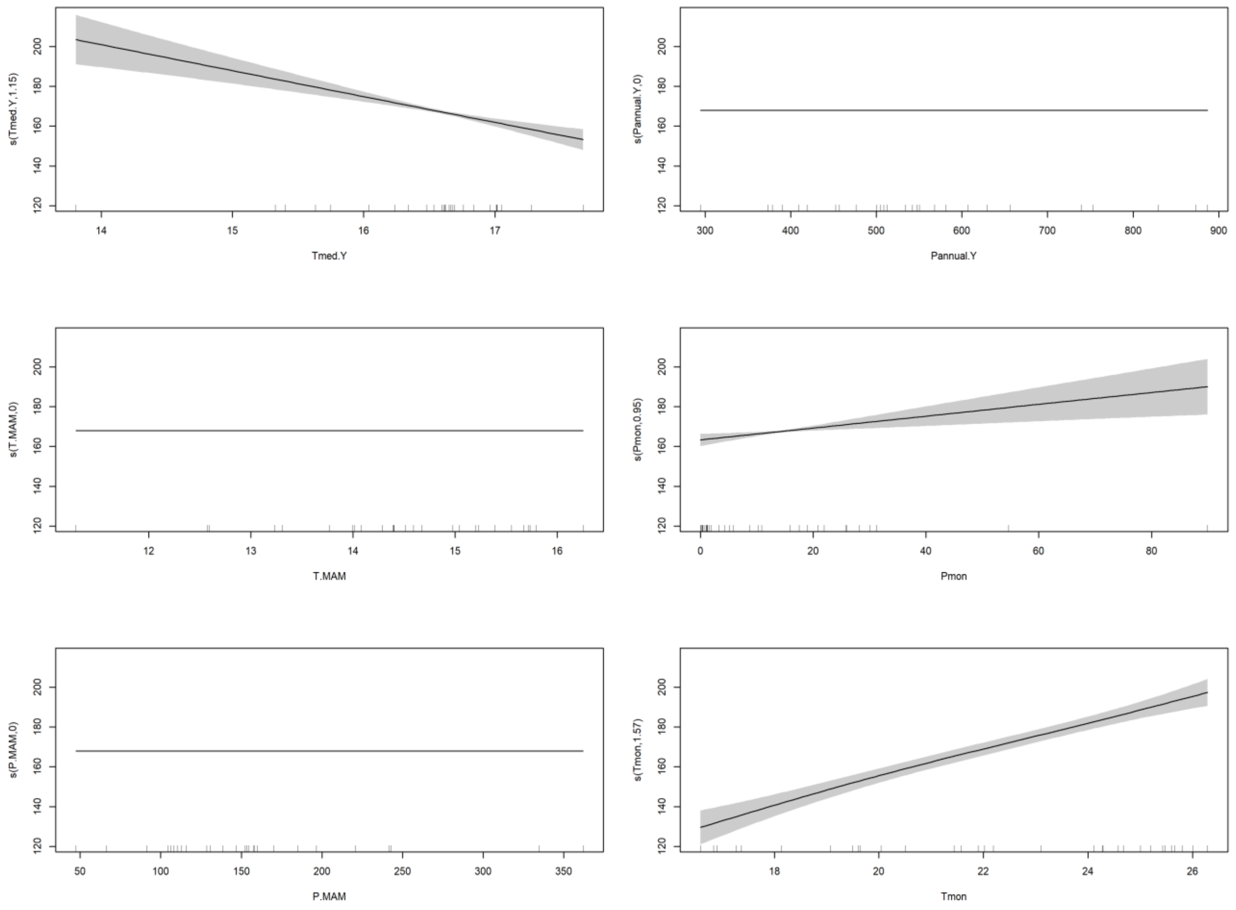

2.9.1. Diagnostics - GAM - DVG - Prunus prostrata

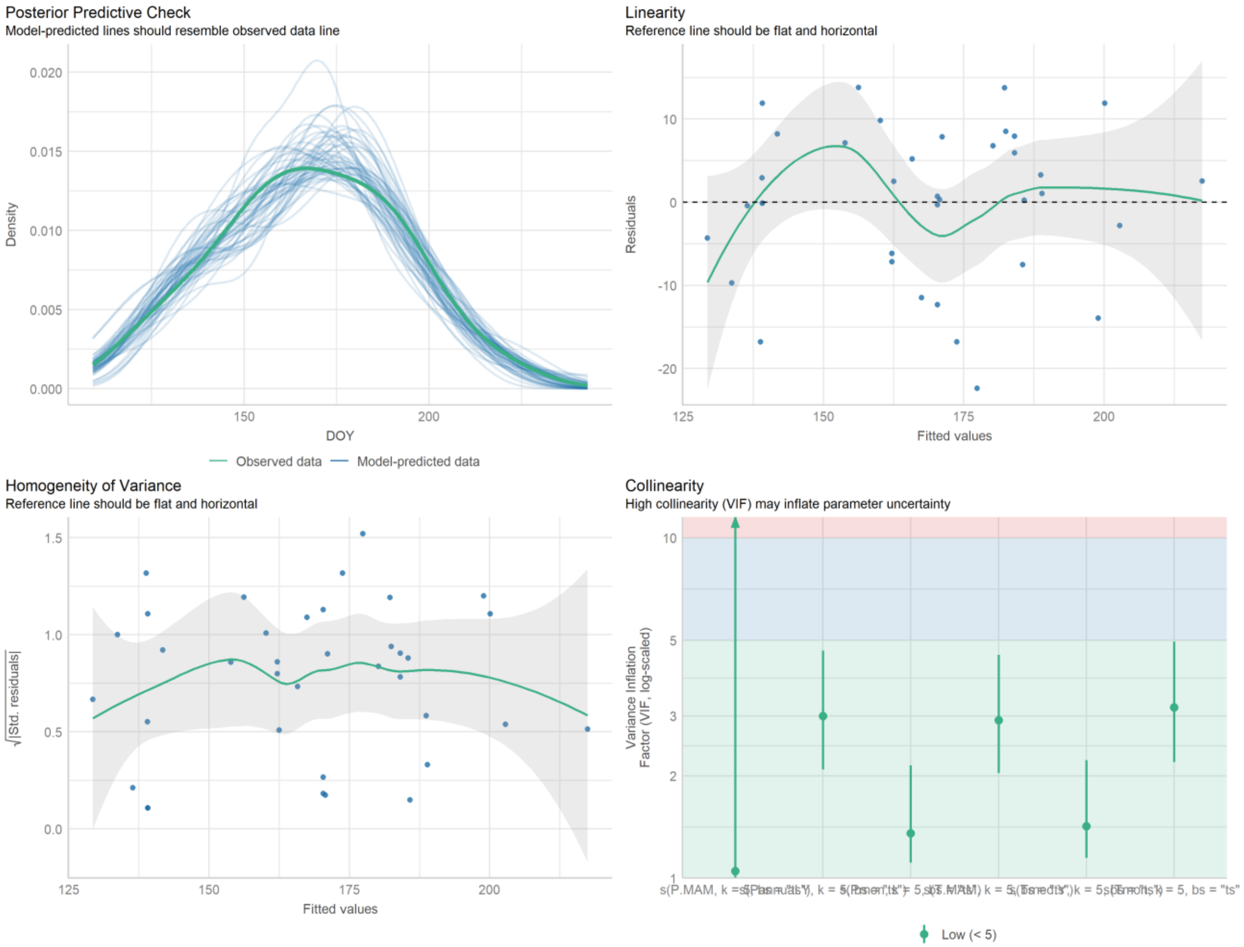

2.10. GAM - F - Salvia rosmarinus

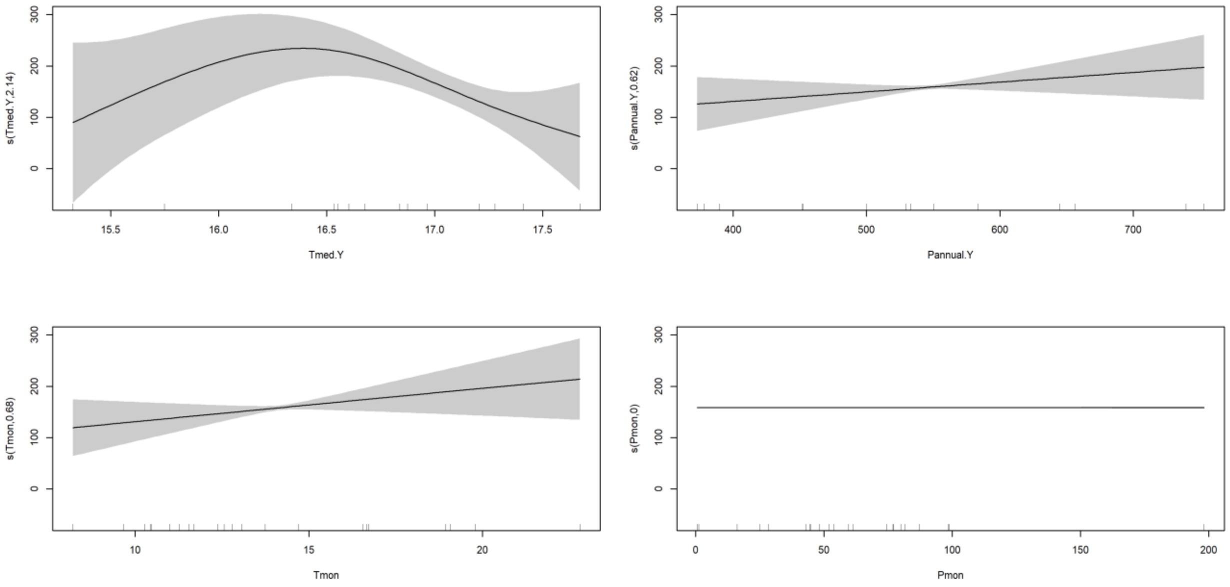

2.10.1. Diagnostics - GAM - F - Salvia rosmarinus

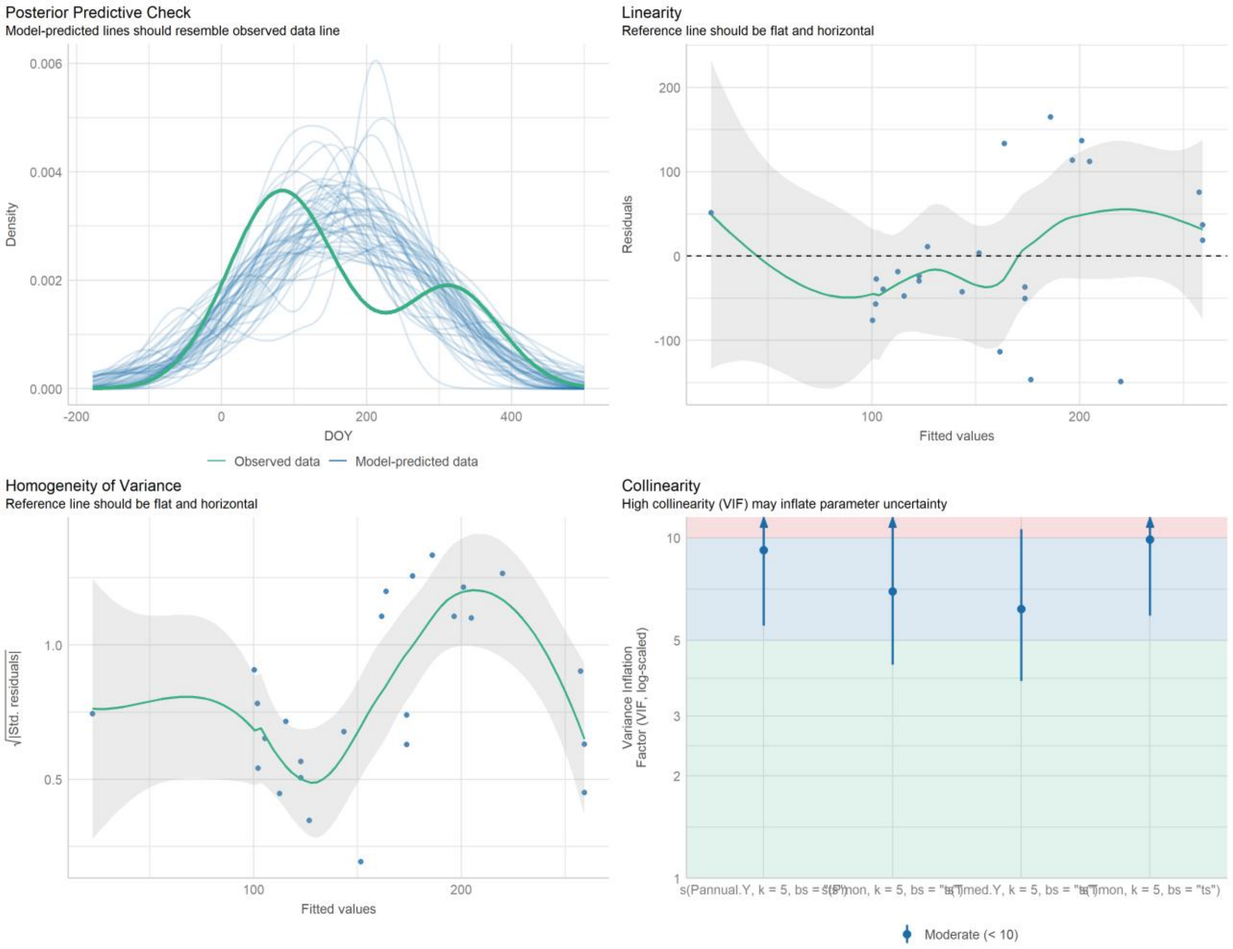

2.11. GAM - F - Sideritis incana

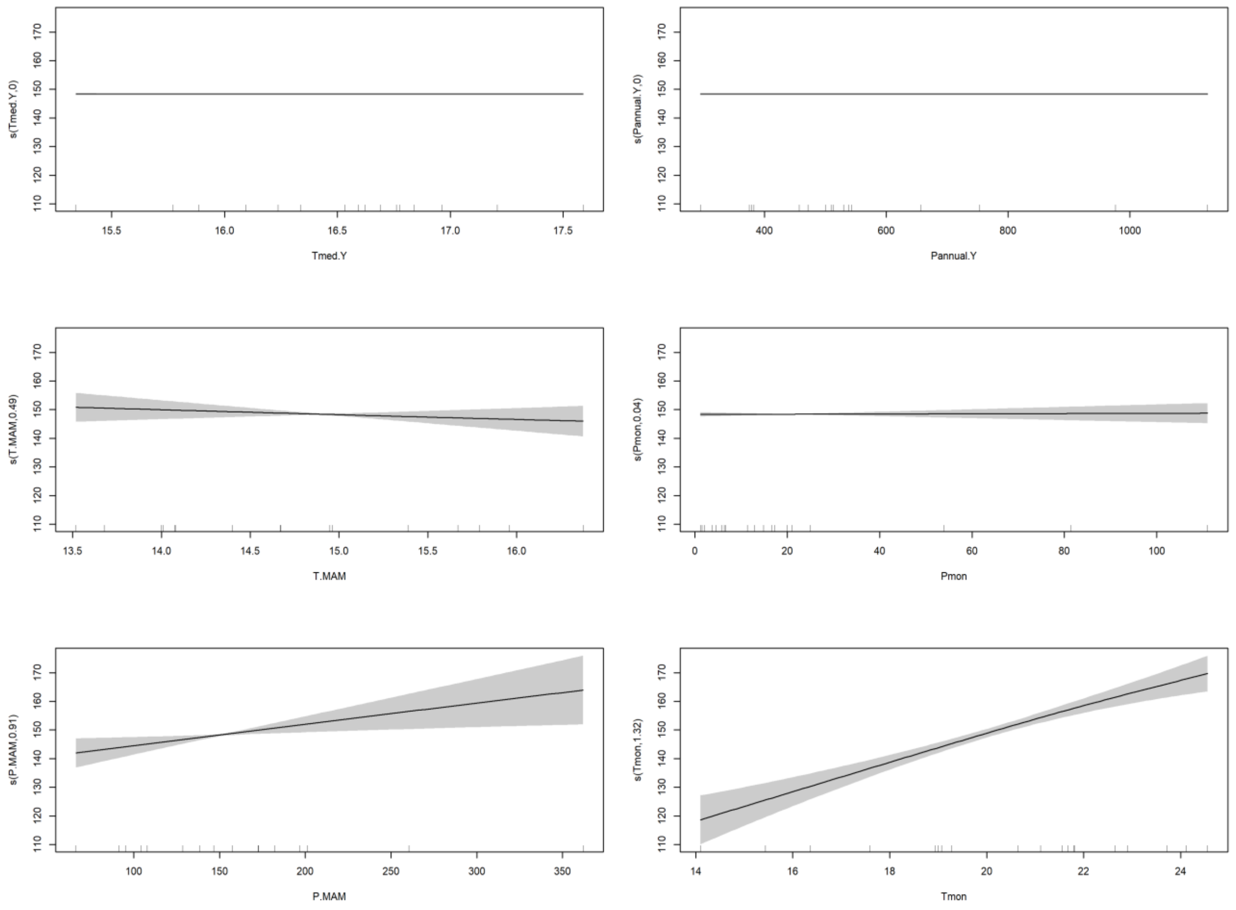

2.11.1. Diagnostics - GAM - F - Sideritis incana

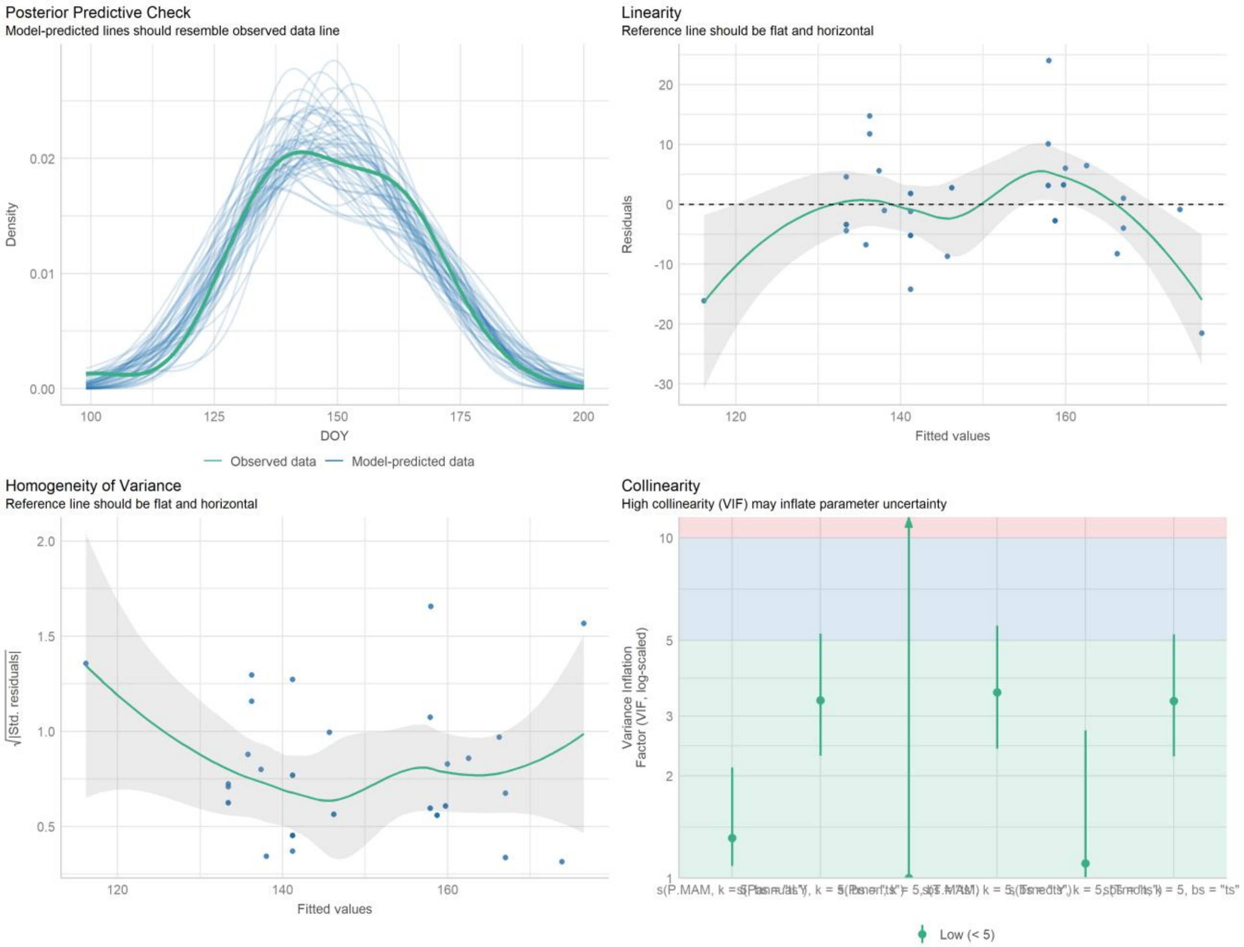

2.12. GAM - FBF - Thymbra capitata

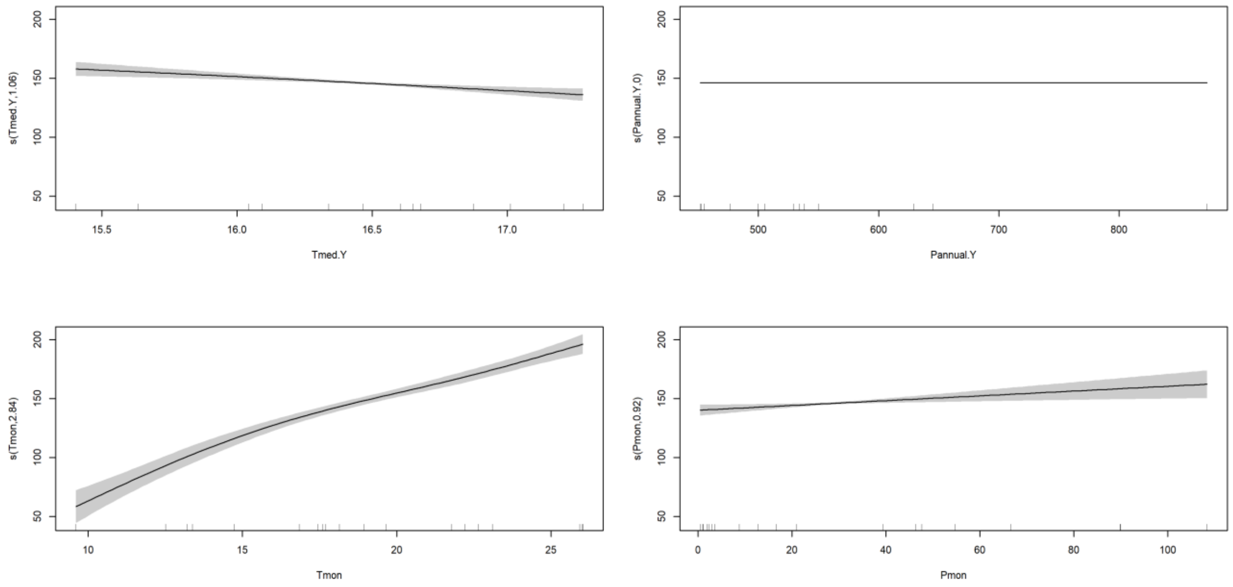

2.12.1. Diagnostics - GAM - FBF - Thymbra capitata

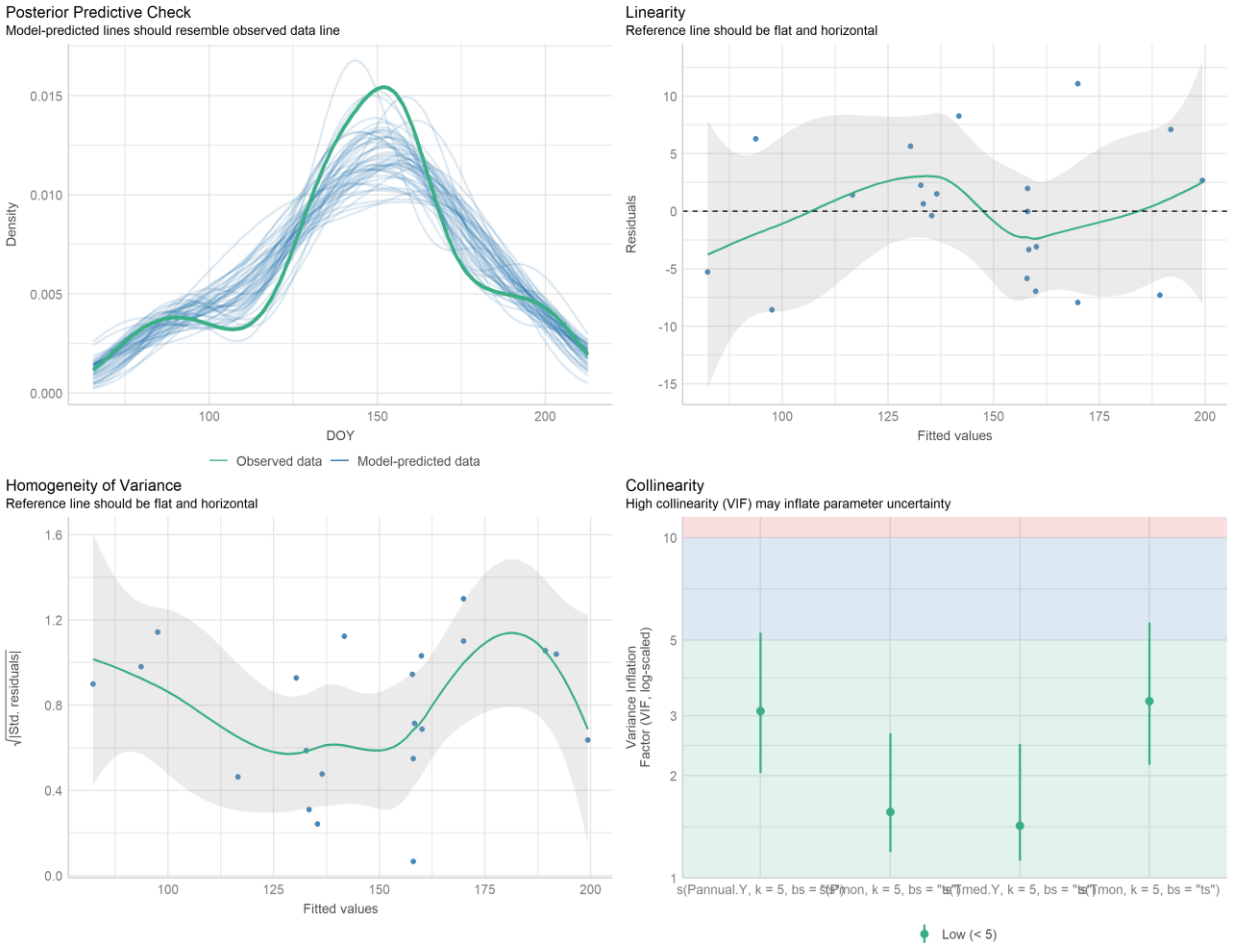

2.13. GAM - F - Thymbra capitata

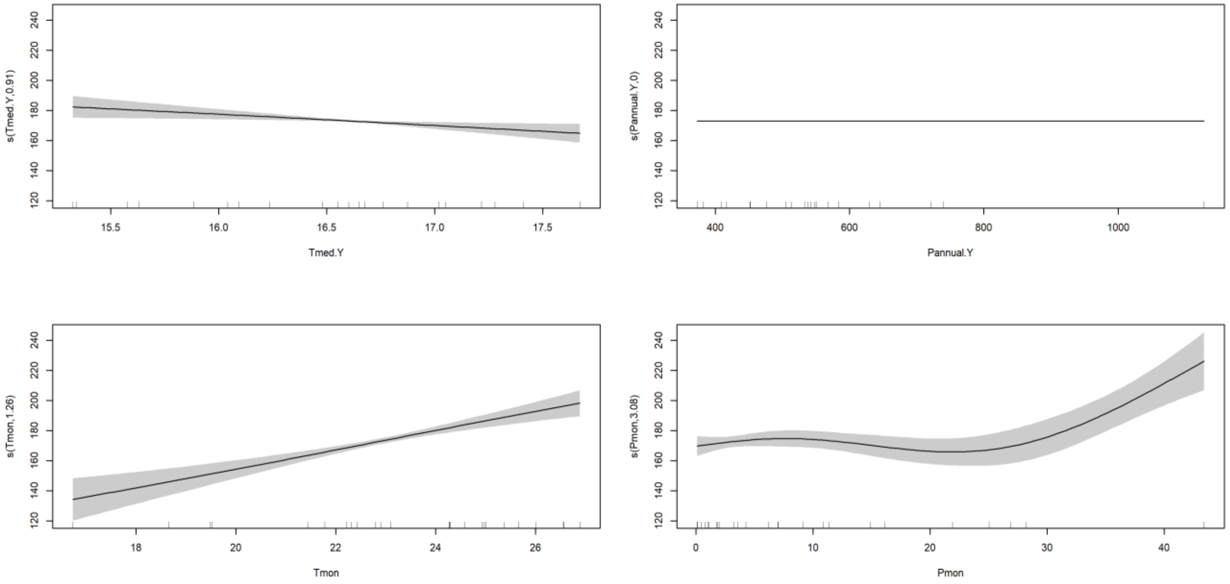

2.13.1. Diagnostics - GAM - F - Thymbra capitata

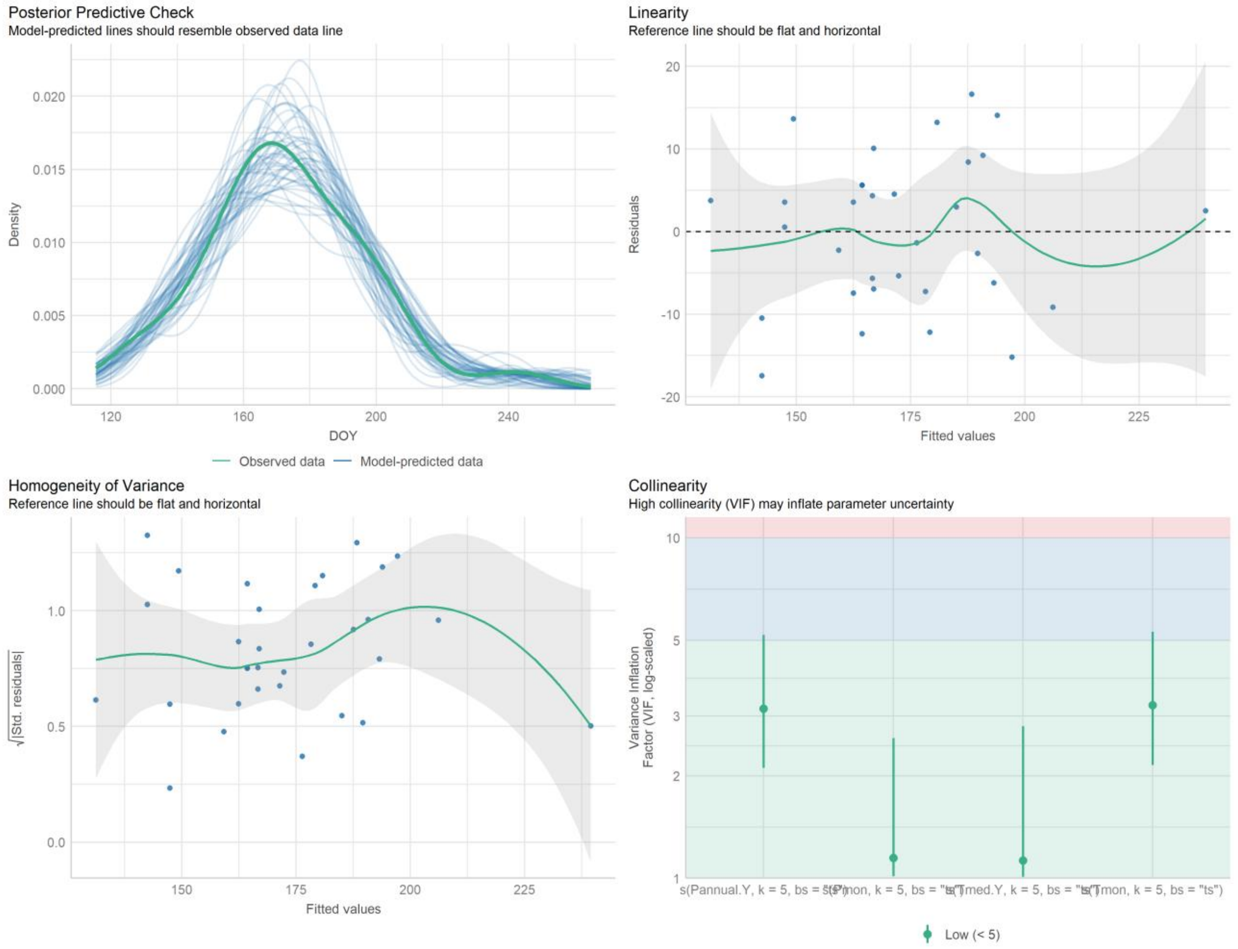

2.14. GAM - DVG - Thymbra capitata

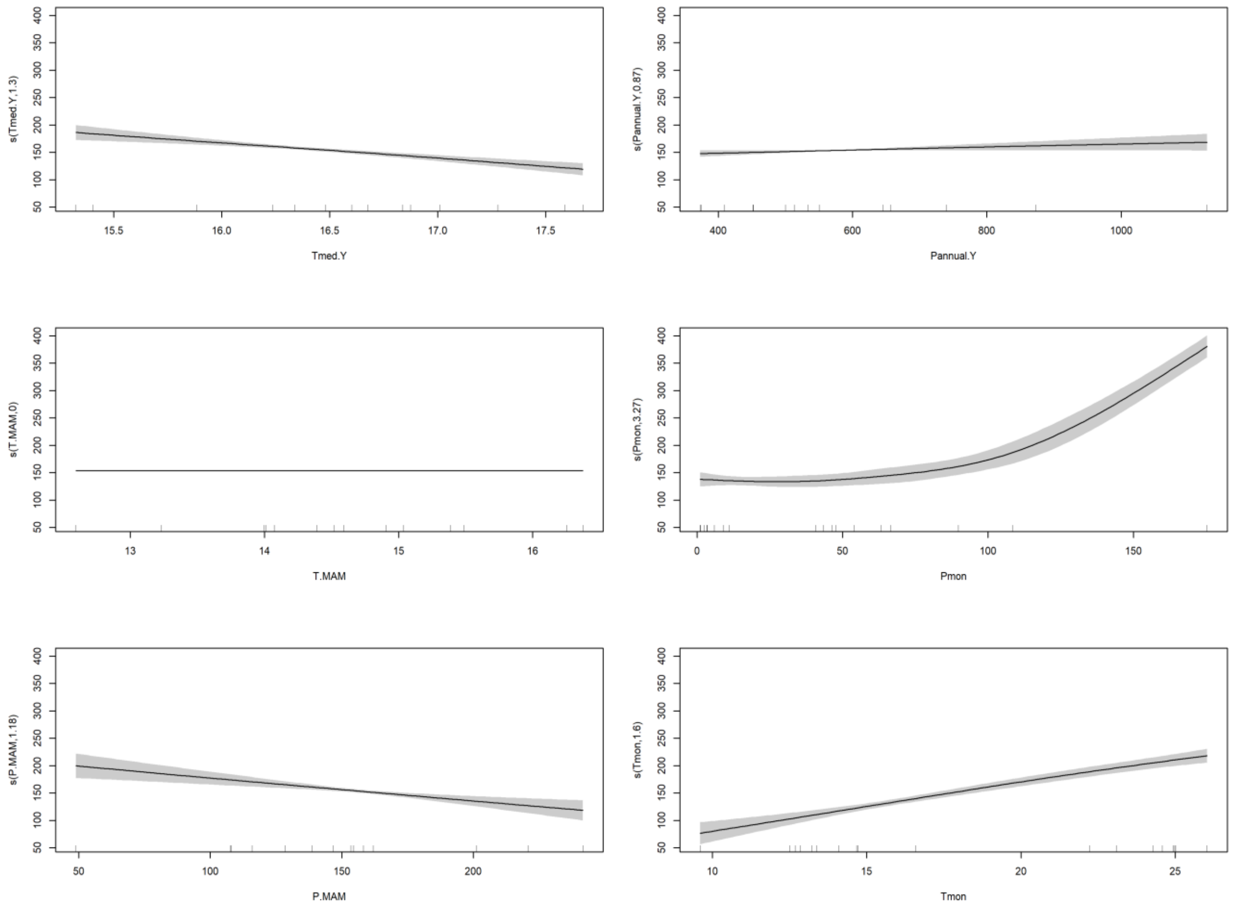

2.14.1. Diagnostics - GAM - DVG - Thymbra capitata

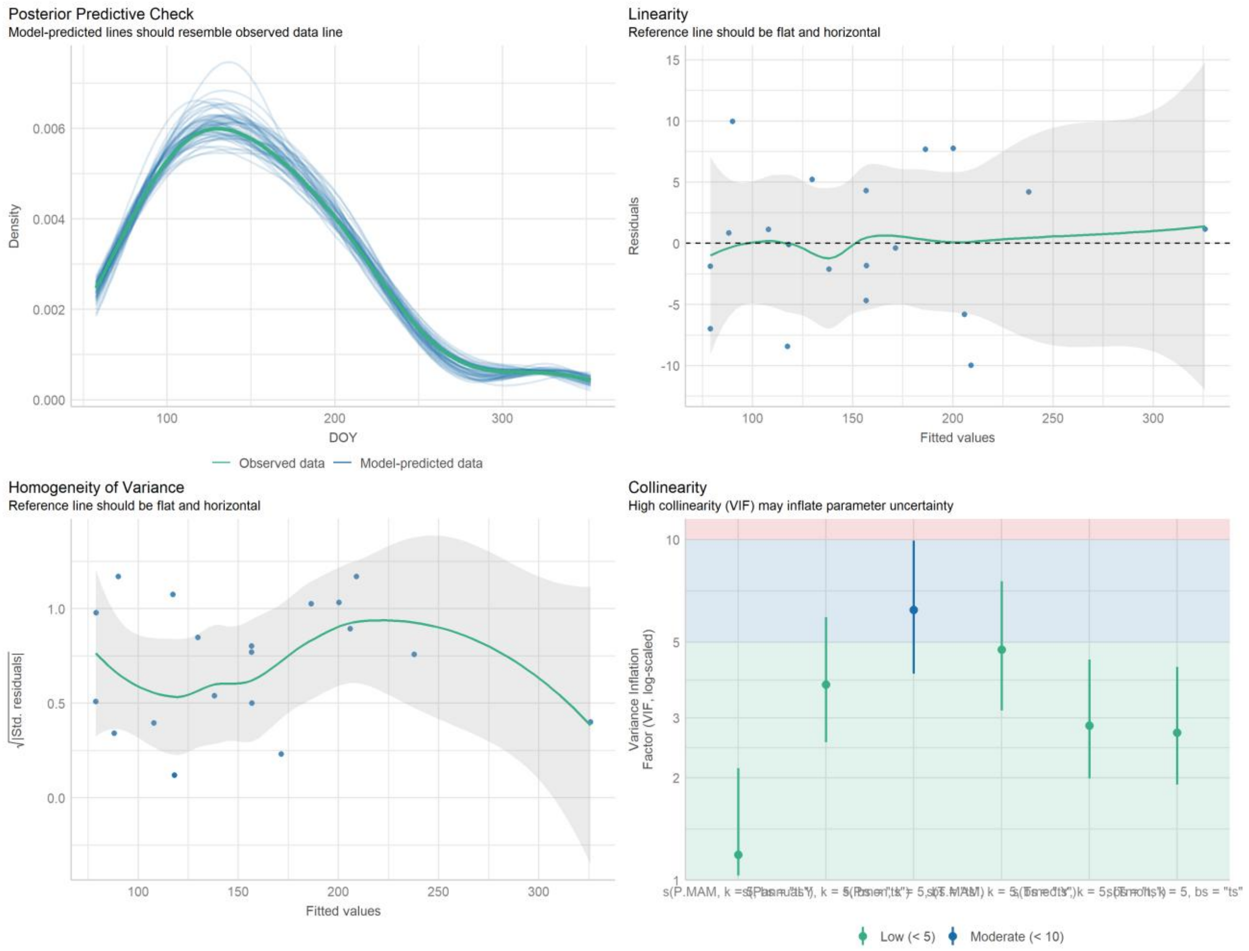

3. AIC Comparison Between Models

Table S3. Akaike Information Criterion analysis on the generalized additive models and generalized linear models by taxon and phenophase.

**Phenofase**	**Taxon**	**AIC_GLM**	**AIC_GAM**
F	*Abies pinsapo*	146.1068	127.78156
DVG	*Cotoneaster granatensis*	141.8300	137.88412
DVG	*Crataegus granatensis*	173.7799	91.94281
DVG	*Crataegus monogyna*	217.4333	184.20513
FS	*Crataegus monogyna*	241.9189	239.72442
FS	*Crepis oporinoides*	133.0984	133.04173
FS	*Hormathophylla spinosa*	220.1933	219.56281
F	*Lavandula stoechas*	849.9680	805.73180
DVG	*Prunus prostrata*	289.5508	280.60135
F	*Salvia rosmarinus*	312.5220	305.28415
F	*Sideritis incana*	275.5389	265.36284
DVG	*Thymbra capitata*	217.7776	146.85351
F	*Thymbra capitata*	268.1236	247.10413
FBF	*Thymbra capitata*	164.9201	147.10563
