# Supplementary material for: Retrospective phenology in western Mediterranean plants: revealing climate change patterns through herbarium specimens
Source: AoB Plants. 2025 Nov 3;17(6):plaf064. doi: 10.1093/aobpla/plaf064 (PMC12611260; doi:10.1093/aobpla/plaf064)
Supplement: plaf064_Supplementary_Data [file plaf064_supplementary_data.zip › Supporting Information - Figures.pdf]

Supporting Information for

Retrospective phenology in western Mediterranean plants: revealing climate change patterns through herbarium specimens

Contents

1. Climatic data
    Figure S1
    Figure S2
    Figure S3
2. Phenological calendars
    Figure S4
    Figure S5
    Figure S6
    Figure S7

1. Climate data: Trends across the Baetic range

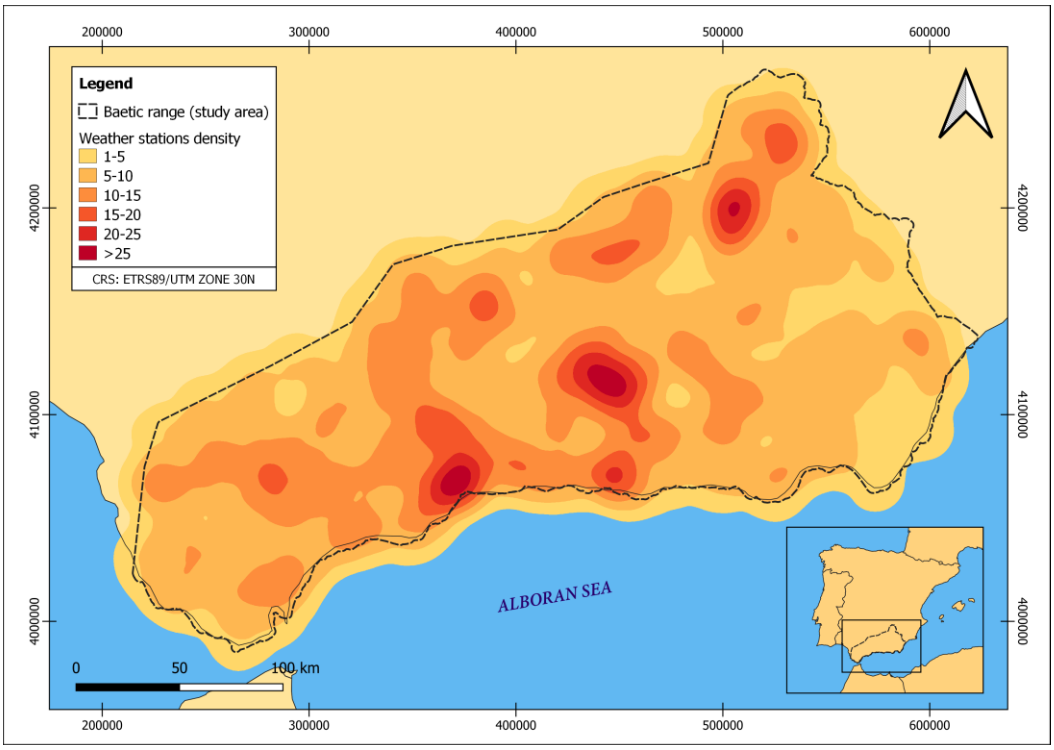

Figure S1. Study area delimitation (dashed line) and map density of the meteorological stations from which the climate data was obtained (CRS: ETRS89/UTM zone 30N).

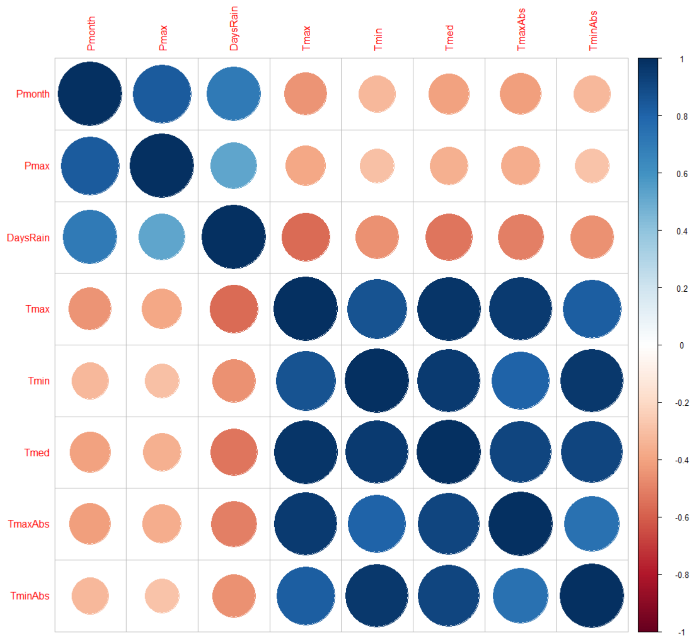

Figure S2. First correlation analysis of the raw data from the initial climatic variables. Pmonth is the monthly precipitation; Pmax is the maximum mm of precipitation of the month; DaysRain is the number of rainy days of the month; Tmax is the average of maximum daily T measured in the month; Tmin is the average of the minimum daily T measured in the month; Tmed is the average temperature of the month; TmaxAbs is the highest T registered in the month; TminAbs is the lowest T registered in the month.

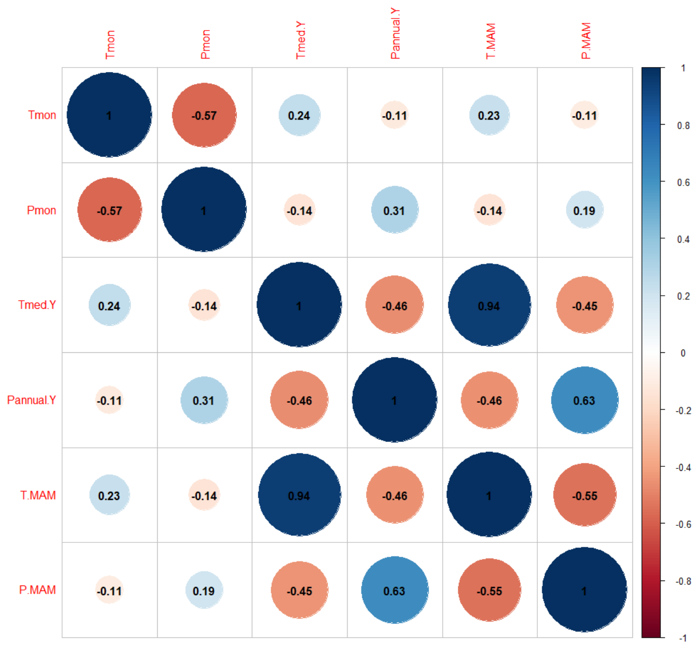

Figure S3. Second correlation analysis of the climatic variables derived from the raw data. P.MAM=spring P (mm); Pannual.Y=annual P (mm); Pmon=monthly P (mm); T.MAM=spring T (°C); Tmed.Y=annual T (°C); Tmon=monthly T (°C).

2. Phenological calendars

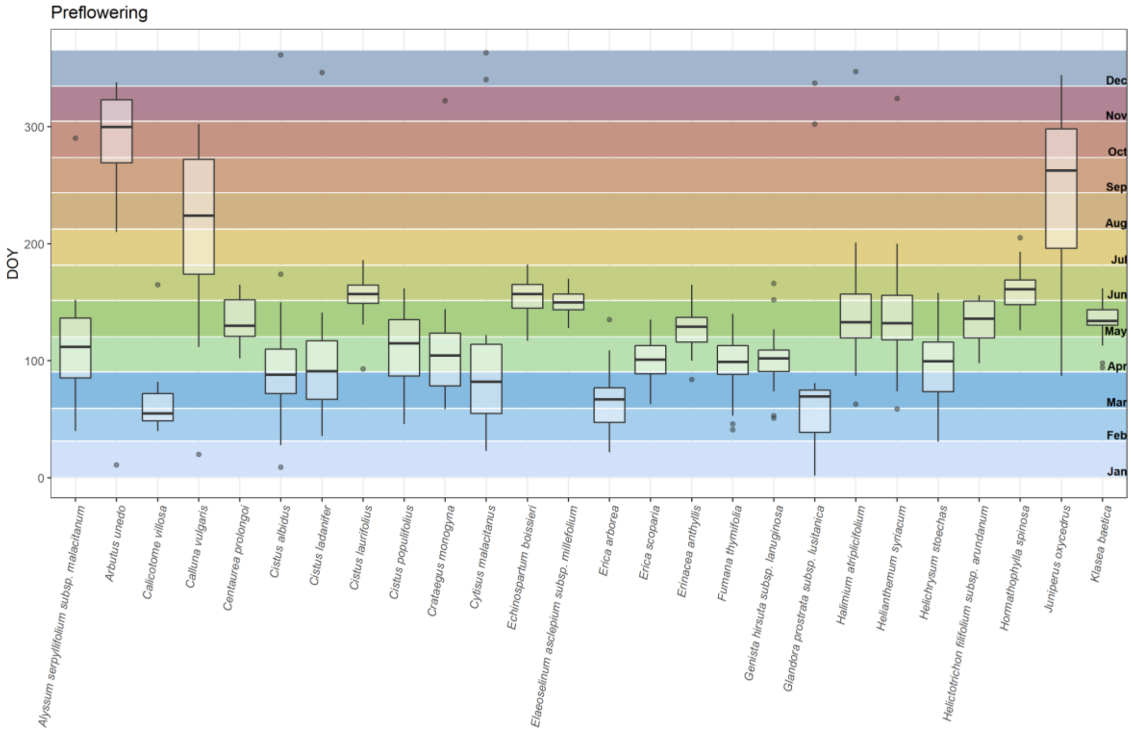

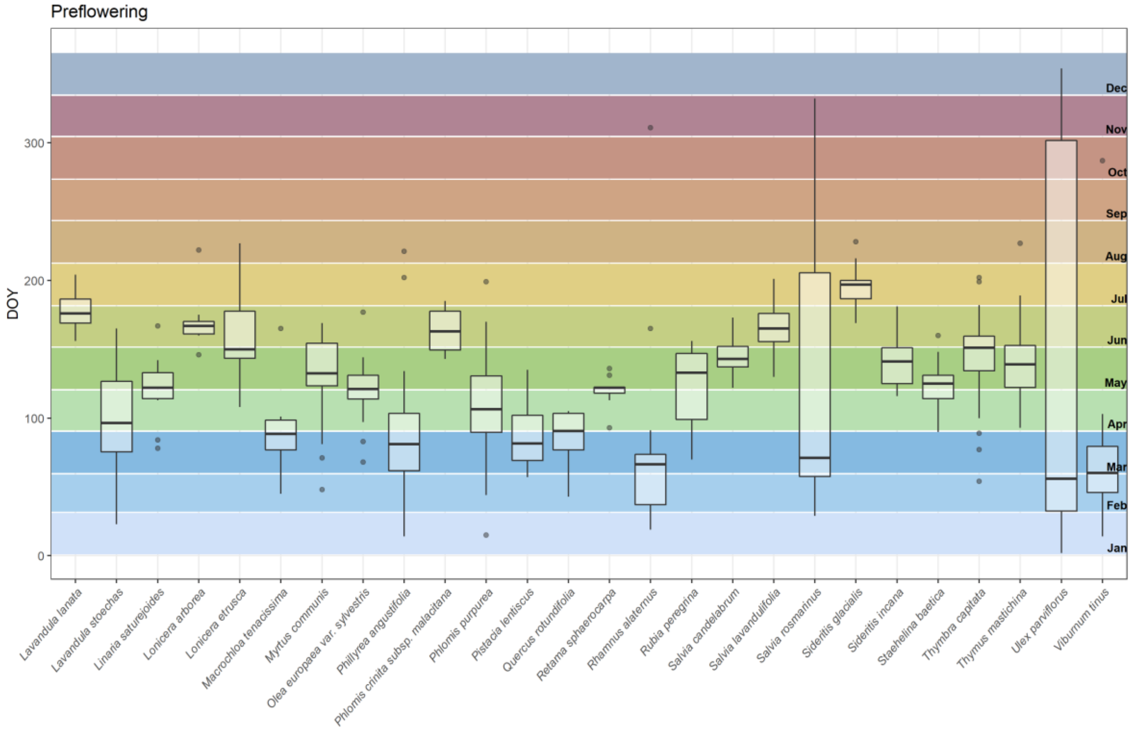

Figure S4. Calendars of the preflowering (FBF) phenophase by taxon, created from the data collected from the preserved herbarium specimen.

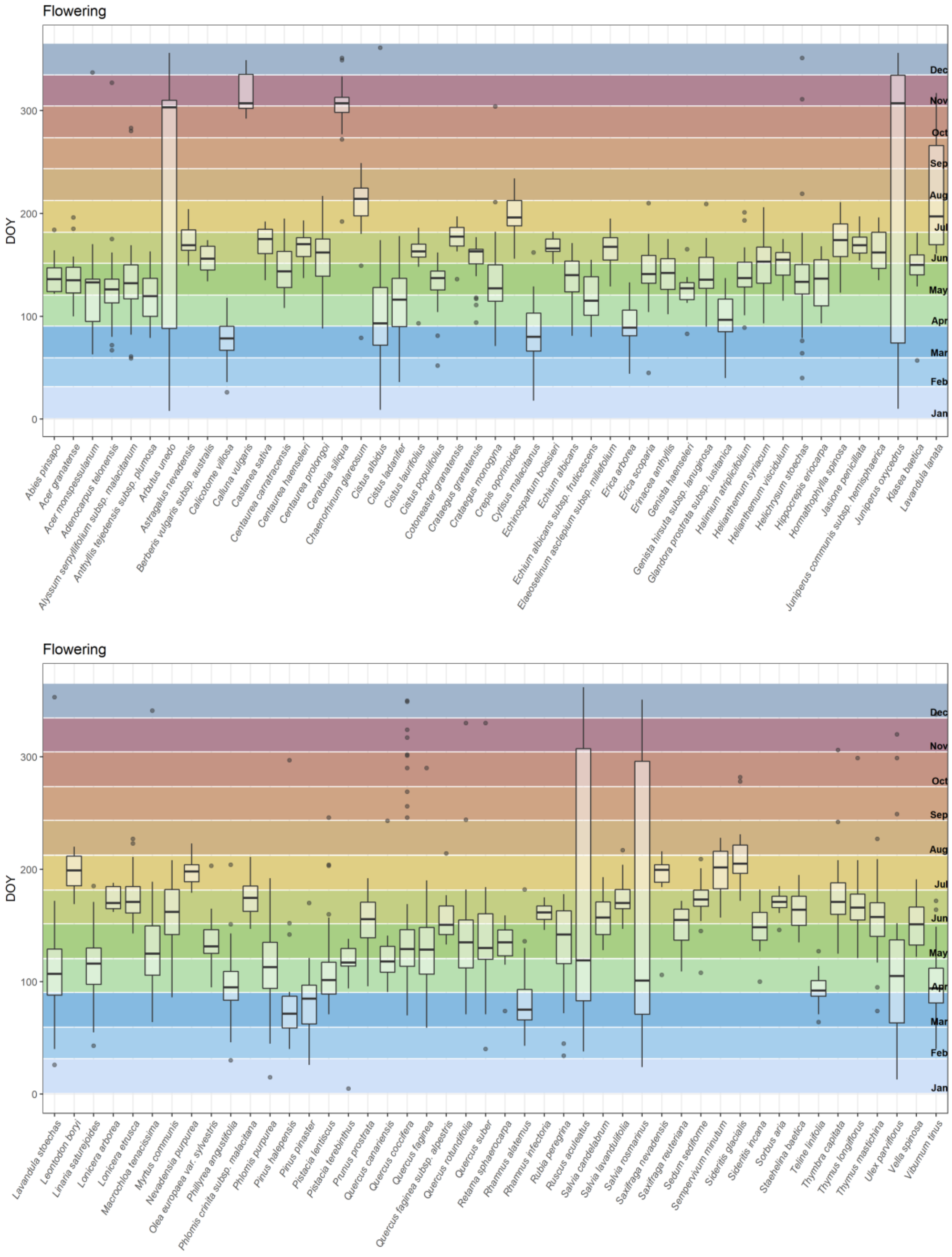

Figure S5. Calendars of the flowering (F) phenophase by taxon, created from the data collected from the preserved herbarium specimen.

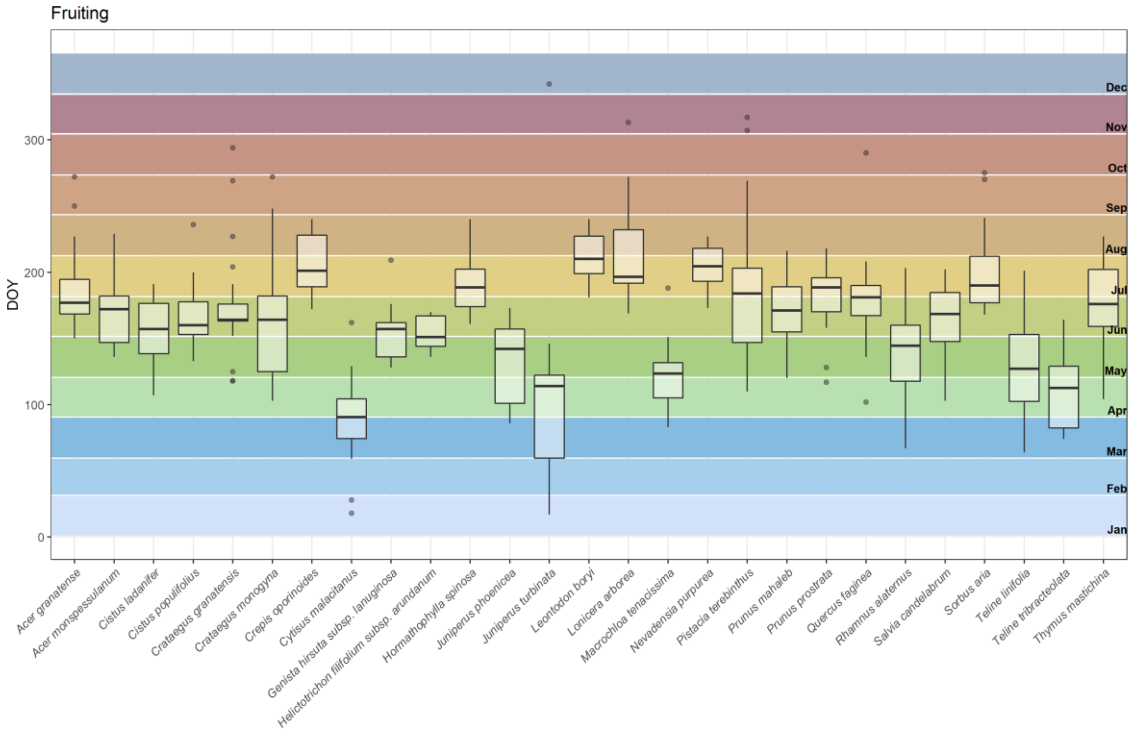

Figure S6. Calendars of the fruiting (FS) phenophase by taxon, created from the data collected from the preserved herbarium specimen.

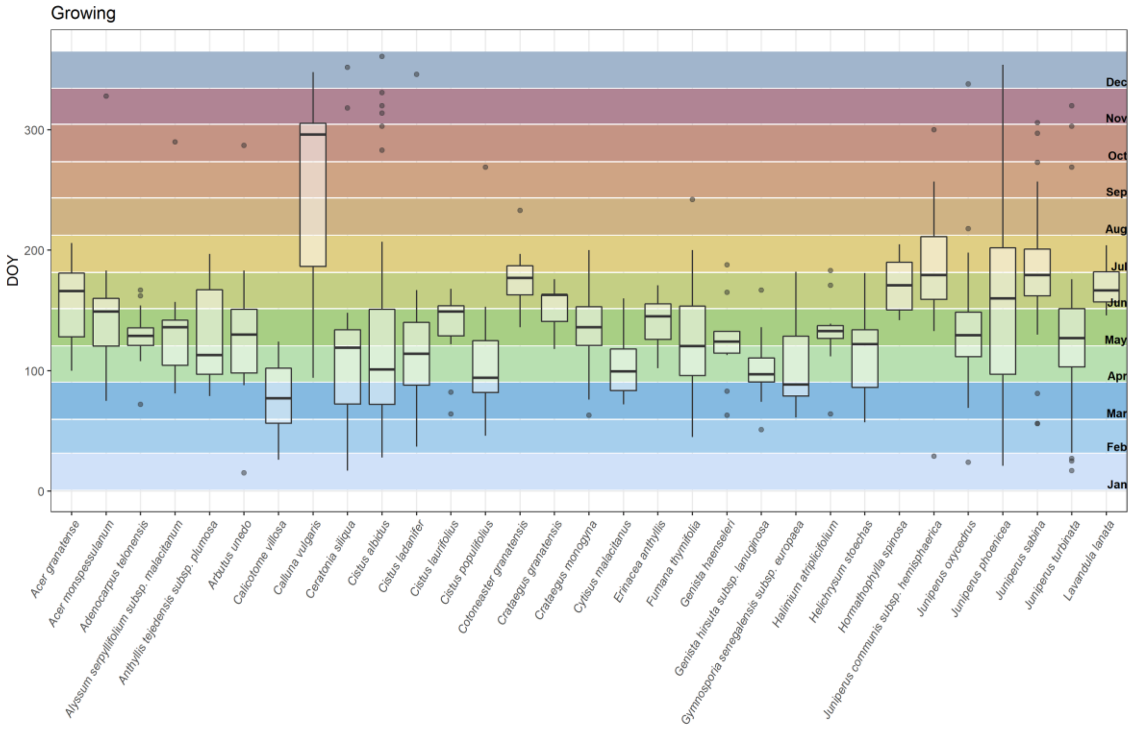

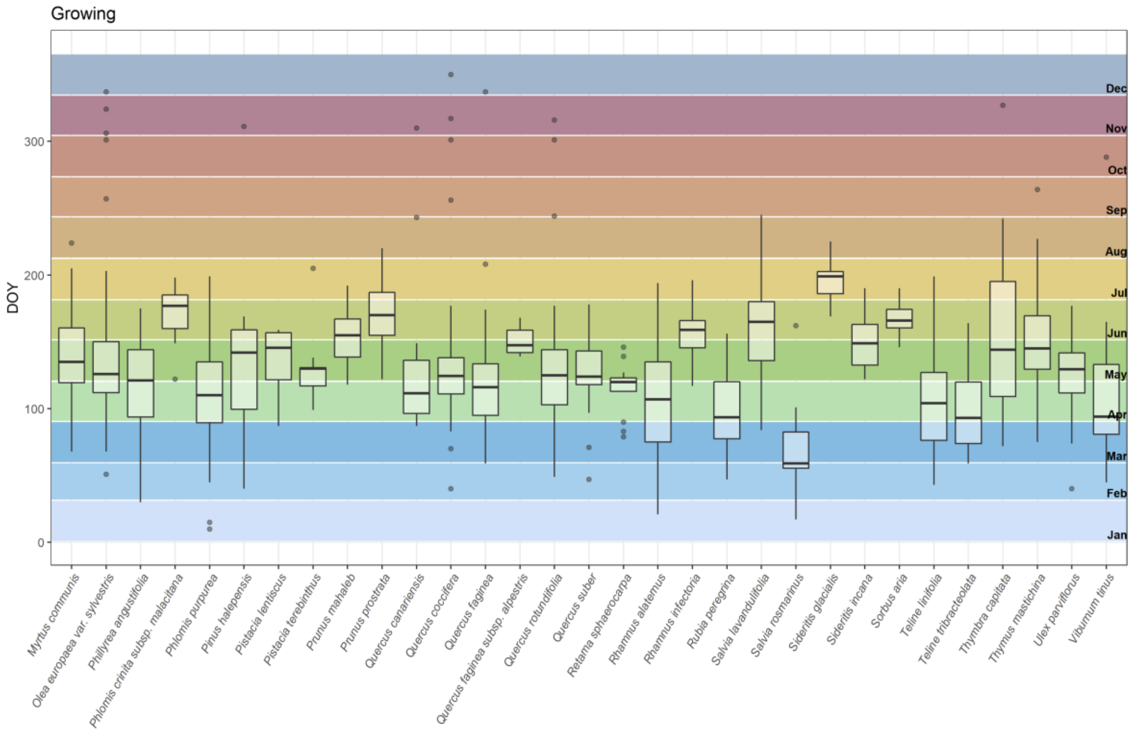

Figure S7. Calendars of the growth (DVG) phenophase by taxon, created from the data collected from the preserved herbarium specimen.
