# Supplementary material for: Retrospective phenology in western Mediterranean plants: revealing climate change patterns through herbarium specimens
Source: AoB Plants. 2025 Nov 3;17(6):plaf064. doi: 10.1093/aobpla/plaf064 (PMC12611260; doi:10.1093/aobpla/plaf064)
Supplement: plaf064_Supplementary_Data [file plaf064_supplementary_data.zip › Supporting Information - Tables.pdf]

Supporting Information for

Retrospective phenology in western Mediterranean plants: revealing climate change patterns through herbarium specimens

Contents

1. Studied taxa
Table S1
Table S2
2. Orophilous and termophilous taxa
Table S3
Table S4

1. Studied taxa

Table S1. List of the selected taxa to study their phenology according to their Habitat of Community Interest and endemicity. Taxa highlighted in yellow had no sufficient data to be analyzed. The family, the Angiosperm Philogeny Group classification (IV), the biological type form (Raunkiær 1934), the Habitat of Community Interest (REDIAM 2020) and the endemic character of each taxon are shown in the table.

	**Taxon**	**Family**	**APGIV**	**BioType**	**Habitat**	**Endemic**	**Narrow endemic**
1	*Abies pinsapo*	Pinaceae	Conifers	Ph	9520	Endemic	Narrow
2	*Acer granatense*	Aceraceae	Rosids	Ph	9240_1		
3	*Acer monspessulanum*	Aceraceae	Rosids	Ph	9240_1		
4	*Adenocarpus telonensis*	Fabaceae	Fabids	Ph	5330_3		
5	*Alyssum serpyllifolium* subsp. *malacitanum*	Brassicaceae	Malvids	Ch	5330_6	Endemic	Narrow
6	*Andryala agardhii*	Asteraceae	Campanulids	Ch	4090	Endemic	
7	*Anthyllis tejedensis subsp. plumosa*	Fabaceae	Fabids	Ch	5330_6	Endemic	Narrow
8	*Arbutus unedo*	Ericaceae	Asterids	Ph	5330_1		
10	*Astragalus nevadensis* subsp. *andres-molinae*	Fabaceae	Fabids	Ch	4090_0	Endemic	Narrow
9	*Astragalus nevadensis subsp. nevadensis*	Fabaceae	Fabids	Ch	4090_0	Endemic	
11	*Berberis vulgaris subsp. australis*	Berberidaceae	Eudicots	Ph	5110_0		
12	*Bupleurum spinosum*	Apiaceae	Campanulids	Ch	4090_0		
13	*Calicotome villosa*	Fabaceae	Fabids	Ph	5330_2		
14	*Calluna vulgaris*	Ericaceae	Asterids	Ch	4030_0		
15	*Castanea sativa*	Fagaceae	Fabids	Ph	9260		
16	*Centaurea carratracensis*	Asteraceae	Campanulids	H	5330_2	Endemic	Narrow
17	*Centaurea haenseleri*	Asteraceae	Campanulids	H	5120	Endemic	Narrow
18	*Centaurea prolongoi*	Asteraceae	Campanulids	H	4090	Endemic	Narrow
19	*Ceratonia siliqua*	Fabaceae	Fabids	Ph	6220_3		
20	*Chaenorhinum glareosum*	Scrophulariaceae	Lamids	H	8220_0	Endemic	Narrow
21	*Chamaerops humilis*	Arecaceae	Commelinids	Ph	5330_2		
22	*Cistus albidus*	Cistaceae	Malvids	Ph	8210		
23	*Cistus ladanifer*	Cistaceae	Malvids	Ph	4030_1		
24	*Cistus laurifolius*	Cistaceae	Malvids	Ph	4030_2		
25	*Cistus populifolius*	Cistaceae	Malvids	Ph	4030_1		
26	*Cotoneaster granatensis*	Rosaceae	Fabids	Ph	5110_1	Endemic	
27	*Crataegus granatensis*	Rosaceae	Fabids	Ph	5110_1		
28	*Crataegus monogyna*	Rosaceae	Fabids	Ph	5110_1		
29	*Crepis oporinoides*	Asteraceae	Campanulids	H	8220_0	Endemic	
30	*Cytisus malacitanus*	Fabaceae	Fabids	Ch	5330_4	Endemic	
31	*Echinospartum boissieri*	Fabaceae	Fabids	Ch	4090_1	Endemic	
32	*Echium albicans* subsp. *albicans*	Boraginaceae	Lamids	H	8130	Endemic	
33	*Echium albicans* subsp. *fruticescens*	Boraginaceae	Lamids	H	8130	Endemic	Narrow
34	*Elaeoselinum asclepium* subsp. *millefolium*	Apiaceae	Campanulids	H	5330_6	Endemic	Narrow

35	*Erica arborea*	Ericaceae	Asterids	Ph	4030_0		
36	*Erica scoparia*	Ericaceae	Asterids	Ph	5330_1		
37	*Erinacea anthyllis*	Fabaceae	Fabids	Ch	4090_0		
38	*Eryngium grosii*	Apiaceae	Campanulids	H	8130	Endemic	Narrow
39	*Euzomodendron bourgaeanum*	Brassicaceae	Malvids	Ch	5330_5	Endemic	Narrow
40	*Fumana thymifolia*	Cistaceae	Malvids	Ch	5330_5		
41	*Genista haenseleri*	Fabaceae	Fabids	Ch	5330_4	Endemic	Narrow
42	*Genista hirsuta* subsp. *lanuginosa*	Fabaceae	Fabids	Ch	5330_6	Endemic	Narrow
43	*Genista hirsuta* subsp. *silicicola*	Fabaceae	Fabids	Ch	5330_6	Endemic	
44	*Genista versicolor*	Fabaceae	Fabids	Ch	5120	Endemic	Narrow
45	*Glandora nítida*	Boraginaceae	Lamids	Ch	8130	Endemic	
46	*Glandora prostrata* subsp. *lusitanica*	Boraginaceae	Lamids	Ch	4030_0		
47	*Gymnosporia senegalensis* subsp. *europaea*	Celastraceae	Rosids	Ph	5220		
48	*Halimium atriplicifolium*	Cistaceae	Malvids	Ph	5330_6		
49	*Helianthemum syriacum*	Cistaceae	Malvids	Ch	6220_1		
50	*Helianthemum viscidulum*	Cistaceae	Malvids	Ch	4090_1	Endemic	Narrow
51	*Helichrysum stoechas*	Asteraceae	Campanulids	Ch	5330_5		
52	*Helictotrichon filifolium* subsp. *arundanum*	Poaceae	Commelinids	Ch	6220_1	Endemic	Narrow
53	*Helictotrichon filifolium* subsp. *filifolium*	Poaceae	Commelinids	Ch	6220_1		
54	*Hippocrepis eriocarpa*	Fabaceae	Fabids	Ch	4090_1	Endemic	Narrow
55	*Hormathophylla spinosa*	Brassicaceae	Malvids	Ch	4090		
56	*Jasione penicillata*	Campanulaceae	Campanulids	Th	6220_3	Endemic	
57	*Juniperus communis* subsp. *hemisphaerica*	Cupressaceae	Conifers	Ph	4060		
58	*Juniperus oxycedrus*	Cupressaceae	Conifers	Ph	5210		
59	*Juniperus phoenicea*	Cupressaceae	Conifers	Ph	5210		
60	*Juniperus sabina*	Cupressaceae	Conifers	Ph	4060		
61	*Juniperus turbinata*	Cupressaceae	Conifers	Ph	2250; 2260_2; 5210		
63	*Klasea baetica* subsp. *alcalae*	Asteraceae	Campanulids	H	5330_6		
62	*Klasea baetica* subsp. *baetica*	Asteraceae	Campanulids	H	5330_6	Endemic	Narrow
64	*Lavandula lanata*	Lamiaceae	Lamids	Ch	4090_1	Endemic	
65	*Lavandula stoechas*	Lamiaceae	Lamids	Ch	4030_2		
66	*Leontodon boryi*	Asteraceae	Campanulids	H	4090_0	Endemic	Narrow
67	*Linaria saturejoides*	Scrophulariaceae	Lamids	Th	8130	Endemic	
68	*Lonicera arborea*	Caprifoliaceae	Campanulids	Ph	5110_1		
69	*Lonicera etrusca*	Caprifoliaceae	Campanulids	Ph	5110_1		
70	*Macrochloa tenacissima*	Poaceae	Commelinids	H	6220_1		
71	*Myrtus communis*	Myrtaceae	Malvids	Ph	5330_2		
72	*Nevadensia purpurea*	Brassicaceae	Malvids	Ch	6160	Endemic	Narrow
73	*Olea europaea* var. *sylvestris*	Oleaceae	Asterids	Ph	9320_0		
74	*Periploca angustifolia*	Apocynaceae	Lamids	Ph	5220		
75	*Phillyrea angustifolia*	Oleaceae	Asterids	Ph	5330_1		
76	*Phlomis crinita* subsp. *malacitana*	Lamiaceae	Lamids	Ch	4090_1	Endemic	
77	*Phlomis purpurea*	Lamiaceae	Lamids	Ch	5330_5		
78	*Pinus halepensis*	Pinaceae	Conifers	Ph	9540		
79	*Pinus nigra* subsp. *salzmannii*	Pinaceae	Conifers	Ph	9530		
80	*Pinus pinaster*	Pinaceae	Conifers	Ph	9540		
81	*Pistacia lentiscus*	Anacardiaceae	Malvids	Ph	5330_2		
82	*Pistacia terebinthus*	Anacardiaceae	Malvids	Ph	9240_0		
83	*Prunus mahaleb*	Rosaceae	Fabids	Ph	5110_1		
84	*Prunus prostrata*	Rosaceae	Fabids	Ch	4060		
85	*Prunus ramburii*	Rosaceae	Fabids	Ph	5110_0; 5110_1	Endemic	
86	*Pterocephalus spathulatus*	Caprifoliaceae	Campanulids	Ch	4090_1	Endemic	
87	*Quercus canariensis*	Fagaceae	Fabids	Ph	9240_0		
88	*Quercus coccifera*	Fagaceae	Fabids	Ph	5330_2		
90	*Quercus faginea* subsp. *alpestris*	Fagaceae	Fabids	Ph	9240_0		
91	*Quercus faginea* subsp. *broteroi*	Fagaceae	Fabids	Ph	9240_0		
89	*Quercus faginea* subsp. *faginea*	Fagaceae	Fabids	Ph	9240_0		
92	*Quercus pyrenaica*	Fagaceae	Fabids	Ph	9230		
93	*Quercus rotundifolia*	Fagaceae	Fabids	Ph	9340		
94	*Quercus suber*	Fagaceae	Fabids	Ph	9330		
95	*Retama sphaerocarpa*	Fabaceae	Fabids	Ph	5330_3		
96	*Rhamnus alaternus*	Rhamnaceae	Fabids	Ph	5330_2		
97	*Rhamnus infectoria*	Rhamnaceae	Fabids	Ph	5110_1		
98	*Rothmaleria granatensis*	Asteraceae	Campanulids	H	4090_1	Endemic	Narrow
99	*Rubia peregrina*	Rubiaceae	Lamids	Ph	5330_1		
100	*Ruscus aculeatus*	Liliaceae	Monocots	G	9260_1		

101	*Salvia candelabrum*	Lamiaceae	Lamids	Ph	5330_2	Endemic	
102	*Salvia granatensis*	Lamiaceae	Lamids	Ch	8210	Endemic	Narrow
103	*Salvia lavandulifolia*	Lamiaceae	Lamids	Ch	4090_1		
104	*Salvia rosmarinus*	Lamiaceae	Lamids	Ph	5330_5		
105	*Sarcocapnos pulcherrima*	Papaveraceae	Eudicots	Ch	8210	Endemic	
106	*Saxifraga nevadensis*	Saxifragaceae	Superrosids	Ch	8220_0	Endemic	Narrow
107	*Saxifraga reuteriana*	Saxifragaceae	Superrosids	Ch	8210	Endemic	
108	*Sedum sediforme*	Crassulaceae	Superrosids	Ch	6220		
109	*Sempervivum minutum*	Crassulaceae	Superrosids	Ch	8220_0	Endemic	
110	*Sideritis glacialis*	Lamiaceae	Lamids	Ch	6160	Endemic	
111	*Sideritis incana*	Lamiaceae	Lamids	Ch	4090_1		
112	*Sorbus aria*	Rosaceae	Fabids	Ph	9240_1		
113	*Staehelina baetica*	Asteraceae	Campanulids	Ch	5330_6	Endemic	Narrow
114	*Teline linifolia*	Fabaceae	Fabids	Ph	5330_1		
115	*Teline tribracteolata*	Fabaceae	Fabids	Ch	8220_0		
116	*Thymbra capitata*	Lamiaceae	Lamids	Ch	5330_5		
117	*Thymelaea tartonraira*	Thymelaeaceae	Malvids	Ch	5330_6	Endemic	
118	*Thymus granatensis*	Lamiaceae	Lamids	Ch	5330_6	Endemic	
119	*Thymus longiflorus*	Lamiaceae	Lamids	Ch	4090_1	Endemic	
120	*Thymus mastichina*	Lamiaceae	Lamids	Ch	4030_2		
121	*Ulex parviflorus*	Fabaceae	Fabids	Ch	5330_4		
122	*Ulex parviflorus* subsp. *rivasgodayanus*	Fabaceae	Fabids	Ph	5330_6		
123	*Vella spinosa*	Brassicaceae	Malvids	Ch	4090_0	Endemic	
124	*Veronica tenuifolia* subsp. *fontqueri*	Scrophulariaceae	Lamids	Ch	4090_0	Endemic	
125	*Viburnum tinus*	Caprifoliaceae	Campanulids	Ph	5330_1		
126	*Vulpia geniculata*	Poaceae	Commelinids	Ch	6220		
127	*Withania frutescens*	Solanaceae	Lamids	Ph	5220		
128	*Ziziphus lotus*	Rhamnaceae	Fabids	Ph	5220		

Table S2. List of taxa with sufficient records to carry out the analyses by phenophase. In preflowering 36 taxa were analyzed, in flowering 67 taxa, in fruiting 19 taxa and in growth 39 taxa. FBF = preflowering; F = flowering; FS = fruiting; DVG = growth.

Taxa analysed in FBF	n	Taxa analysed in F	n	Taxa analysed in FS	n	Taxa analysed in DVG	n
Alyssum serpyllifolium subsp. *malacitanum*	19	*Abies pinsapo*	19	*Acer granatense*	23	*Acer granatense*	21
Centaurea prolongoi	17	*Acer monspessulanum*	15	*Cistus ladanifer*	23	*Adenocarpus telonensis*	19
Cistus albidus	41	*Adenocarpus telonensis*	37	*Cistus populifolius*	18	*Calicotome villosa*	30
Cistus ladanifer	49	*Alyssum serpyllifolium* subsp. *malacitanum*	37	*Crataegus granatensis*	27	*Calluna vulgaris*	18
Cistus laurifolius	20	*Arbutus unedo*	25	*Crataegus monogyna*	27	*Cistus albidus*	41
Cistus populifolius	30	*Calicotome villosa*	28	*Crepis oporinoides*	17	*Cistus ladanifer*	29
Crataegus monogyna	20	*Calluna vulgaris*	19	*Cytisus malacitanus*	16	*Cistus populifolius*	20
Erica arborea	23	*Centaurea carratracensis*	22	*Helictotrichon filifolium* subsp. *arundanum*	17	*Cotoneaster granatensis*	17
Erica scoparia	21	*Centaurea prolongoi*	41	*Hormathophylla spinosa*	28	*Crataegus granatensis*	22
Fumana thymifolia	46	*Ceratonia siliqua*	20	*Juniperus phoenicea*	15	*Crataegus monogyna*	27
Genista hirsuta subsp. *lanuginosa*	24	*Chaenorhinum glareosum*	27	*Juniperus turbinata*	20	*Erinacea anthyllis*	15
Halimium atriplicifolium	59	*Cistus albidus*	61	*Leontodon boryi*	24	*Fumana thymifolia*	16
Helianthemum syriacum	37	*Cistus ladanifer*	54	*Lonicera arborea*	16	*Genista hirsuta* subsp. *lanuginosa*	15

Helichrysum stoechas	18	*Cistus laurifolius*	16	*Nevadensia purpurea*	22	*Helichrysum stoechas*	23
Hormathophylla spinosa	19	*Cistus populifolius*	19	*Prunus mahaleb*	16	*Juniperus communis* subsp. *hemisphaerica*	16
Juniperus oxycedrus	16	*Crataegus granatensis*	29	*Prunus prostrata*	16	*Juniperus oxycedrus*	84
Klasea baetica	15	*Crataegus monogyna*	59	*Rhamnus alaternus*	34	*Juniperus phoenicea*	29
Lavandula lanata	27	*Crepis oporinoides*	15	*Teline linifolia*	15	*Juniperus sabina*	30
Lavandula stoechas	54	*Cytisus malacitanus*	25	*Thymus mastichina*	24	*Juniperus turbinata*	43
Lonicera etrusca	15	*Echium albicans*	56			*Lavandula lanata*	20
Macrochloa tenacissima	16	*Elaeoselinum asclepium* subsp. *millefolium*	20			*Myrtus communis*	31
Olea europaea var. *sylvestris*	18	*Erica arborea*	55			*Olea europaea* var. *sylvestris*	55
Phillyrea angustifolia	24	*Erica scoparia*	25			*Phillyrea angustifolia*	16
Phlomis crinita subsp. *malacitana*	23	*Genista hirsuta* subsp. *lanuginosa*	36			*Phlomis purpurea*	147
Phlomis purpurea	80	*Glandora prostrata subsp. lusitanica*	32			*Prunus mahaleb*	15
Rhamnus alaternus	18	*Halimium atriplicifolium*	20			*Prunus prostrata*	37
Rubia peregrina	25	*Helianthemum syriacum*	24			*Quercus coccifera*	52
Salvia candelabrum	17	*Helichrysum stoechas*	104			*Quercus faginea* subsp. *faginea*	43
Salvia lavandulifolia	15	*Hormathophylla spinosa*	24			*Quercus rotundifolia*	65
Salvia rosmarinus	23	*Juniperus oxycedrus*	32			*Quercus suber*	22
Sideritis glacialis	20	*Klasea baetica*	18			*Rhamnus alaternus*	77
Staehelina baetica	15	*Lavandula stoechas*	102			*Rhamnus infectoria*	19
Thymbra capitata	22	*Leontodon boryi*	18			*Rubia peregrina*	30
Thymus mastichina	46	*Linaria saturejoides*	46			*Sideritis glacialis*	15
Ulex parviflorus	23	*Lonicera etrusca*	43			*Sideritis incana*	22
Viburnum tinus	15	*Macrochloa tenacissima*	32			*Thymbra capitata*	20
		Myrtus communis	19			*Thymus mastichina*	43
		Nevadensia purpurea	27			*Ulex parviflorus*	20
		Phillyrea angustifolia	36			*Viburnum tinus*	16
		Phlomis crinita subsp. *malacitana*	44				
		Phlomis purpurea	161				
		Pinus pinaster	15				
		Pistacia lentiscus	54				
		Pistacia terebinthus	15				
		Prunus prostrata	34				
		Quercus coccifera	62				
		Quercus faginea subsp. *faginea*	62				
		Quercus faginea subsp. *alpestris*	16				
		Quercus rotundifolia	66				
		Quercus suber	27				
		Retama sphaerocarpa	17				
		Rhamnus alaternus	45				
		Rubia peregrina	36				
		Salvia candelabrum	15				
		Salvia lavandulifolia	25				
		Salvia rosmarinus	25				
		Saxifraga reuteriana	23				
		Sempervivum minutum	20				
		Sideritis glacialis	42				
		Sideritis incana	36				
		Staehelina baetica	22				
		Teline linifolia	13				
		Thymbra capitata	33				
		Thymus longiflorus	98				
		Thymus mastichina	72				
		Ulex parviflorus	44				
		Viburnum tinus	33				

2. Orophilous and thermophilous taxa

Table S3. Summary of taxon-level results for orophilous taxa by phenophase. For each taxon × phenophase, YES indicates a significant change ($p < 0.05$) in at least one predictor (Year or a climate variable); NO indicates no significant change despite sufficient data; NA = not available, which indicates insufficient data for the analysis. FBF = pre-flowering bud; F = flowering; FS = fruiting; DVG = vegetative growth. HCI = Habitat of Community Interest associated with the taxon. Endemic and Narrow endemic indicate the endemism status of each taxon.

Taxon	FBF		F		FS		DVG		Any advances	Any delays	Present changes in any of the phenophases and variables	HCI	Endemic	Narrow endemic
	Advances	Delays	Advances	Delays	Advances	Delays	Advances	Delays						
Abies pinsapo	NA	NA	YES	YES	NA	NA	NA	NA	YES	YES	YES	9520	YES	YES
Acer granatense	NA	NA	NO	NO	YES	YES	YES	NO	YES	YES	YES	9240_1	NO	NO
Chaenorhinum glareosum	NA	NA	YES	NO	NA	NA	NA	NA	YES	NO	YES	8130	YES	YES
Cistus laurifolius	YES	NO	NO	NO	NA	NA	NO	NO	YES	NO	YES	4030_2	NO	NO
Cotoneaster granatensis	NA	NA	NO	NO	NA	NA	YES	NO	YES	NO	YES	5110_1	YES	NO
Crataegus granatensis	NA	NA	YES	NO	NO	YES	NO	NO	YES	NO	YES	5110_1	NO	NO
Crataegus monogyna	YES	NO	YES	NO	NO	NO	YES	NO	YES	NO	YES	5110_1	NO	NO
Crepis oporinoides	NA	NA	YES	YES	YES	NO	NA	NA	YES	YES	YES	8220_0	YES	NO
Hormathophylla spinosa	YES	NO	YES	NO	YES	YES	NO	NO	YES	NO	YES	4090	NO	NO
Juniperus sabina	NA	NA	NA	NA	NA	NA	NO	NO	NO	NO	NO	4060	NO	NO
Lavandula stoechas	YES	NO	YES	NO	NA	NA	NA	NA	YES	NO	YES	4030_2	NO	NO
Lonicera etrusca	NO	NO	YES	NO	NA	NA	NA	NA	YES	NO	YES	5110_1	NO	NO
Nevadensia purpurea	NA	NA	YES	NO	YES	NO	NA	NA	YES	NO	YES	6160	YES	YES
Prunus mahaleb	NA	NA	NA	NA	YES	NO	NO	NO	YES	NO	YES	5110_1	NO	NO
Prunus prostrata	NA	NA	YES	NO	YES	YES	YES	YES	YES	YES	YES	4060	NO	NO
Rhamnus infectoria	NA	NA	NO	NO	NA	NA	YES	NO	YES	NO	YES	5110_1	NO	NO
Sempervivum minutum	NA	NA	YES	YES	NA	NA	NA	NA	YES	YES	YES	8220_0	YES	NO
Sideritis glacialis	YES	NO	YES	YES	NA	NA	YES	NO	YES	YES	YES	6160	YES	NO
Thymus mastichina	YES	NO	YES	NO	YES	NO	YES	NO	YES	NO	YES	4030_2	NO	NO

Table S4. Summary of taxon-level results for termophilous taxa by phenophase. For each taxon × phenophase, YES indicates a significant change ($p < 0.05$) in at least one predictor (Year or a climate variable); NO indicates no significant change despite sufficient data; NA = not available, which indicates insufficient data for the analysis. FBF = pre-flowering bud; F = flowering; FS = fruiting; DVG = vegetative growth. HCI = Habitat of Community Interest associated with the taxon. Endemic and Narrow endemic indicate the endemism status of each taxon.

Taxon	FBF		F		FS		DVG		Any advances	Any delays	Present changes in any of the phenophases and variables	HCI	Endemic	Narrow endemic
	Advances	Delays	Advances	Delays	Advances	Delays	Advances	Delays						
Adenocarpus telonensis	NA	NA	YES	NO	NA	NA	NO	NO	YES	NO	YES	5330_3	NO	NO
Alyssum serpyllifolium subsp. malacitanum	YES	YES	YES	NO	NA	NA	NO	NO	YES	YES	YES	5330_6	YES	YES
Arbutus unedo	NA	NA	NO	YES	NA	NA	NO	NO	NO	YES	YES	5330_1	NO	NO
Calicotome villosa	NO	NO	YES	NO	NA	NA	YES	NO	YES	NO	YES	5330_2	NO	NO
Calluna vulgaris	NO	NO	NO	NO	NA	NA	NO	NO	NO	NO	NO	4030_0	NO	NO
Centaurea carratracensis	NO	NO	YES	NO	NA	NA	NA	NA	YES	NO	YES	5330_6	YES	YES
Centaurea prolongoi	NO	NO	YES	NO	NA	NA	NA	NA	YES	NO	YES	5330_6	YES	YES
Ceratonia siliqua	NO	NO	NO	YES	NA	NA	NO	NO	NO	YES	YES	9320_1	NO	NO
Cistus albidus	YES	YES	YES	NO	NA	NA	YES	YES	YES	YES	YES	5330_5	NO	NO
Cistus ladanifer	YES	YES	YES	NO	YES	NO	YES	NO	YES	YES	YES	4030_1	NO	NO
Cistus populifolius	YES	YES	NO	NO	YES	NO	YES	NO	YES	YES	YES	4030_1	NO	NO
Cytisus malacitanus	NA	NA	YES	NO	NO	NO	NO	NO	YES	NO	YES	5330_4	YES	NO
Echium albicans subsp. albicans	NA	NA	YES	NO	NA	NA	NA	NA	YES	NO	YES	8130	YES	NO
Elaeoselinum asclepium subsp. millefolium	NA	NA	YES	NO	NA	NA	NA	NA	YES	NO	YES	5330_6	YES	YES
Erica arborea	YES	NO	YES	NO	NA	NA	NA	NA	YES	NO	YES	4030_0	NO	NO
Erica scoparia	NO	NO	YES	NO	NA	NA	NA	NA	YES	NO	YES	5330_1	NO	NO
Fumana thymifolia	YES	YES	NA	NA	NA	NA	NO	NO	YES	YES	YES	5330_5	NO	NO
Genista hirsuta subsp. lanuginosa	YES	YES	YES	NO	NO	NO	NO	NO	YES	YES	YES	5330_6	YES	YES
Glandora prostrata subsp. lusitanica	NO	NO	YES	NO	NA	NA	NA	NA	YES	NO	YES	4030_0	NO	NO
Halimium atriplicifolium	NO	NO	YES	NO	NA	NA	NA	NA	YES	NO	YES	5330_6	NO	NO
Helianthemum syriacum	NO	NO	NO	NO	NA	NA	NA	NA	NO	NO	NO	6220_1	NO	NO
Helichrysum stoechas	NO	NO	YES	NO	NA	NA	YES	YES	YES	YES	YES	5330_5	NO	NO

Helictotrichon filifolium subsp. arundanum	NO	NO	NA	NA	YES	NO	NA	NA	YES	NO	YES	6220_1	YES	YES
Juniperus oxycedrus	NO	NO	NO	NO	NA	NA	YES	NO	YES	NO	YES	4060	NO	NO
Juniperus turbinata	NA	NA	NA	NA	NO	NO	YES	NO	YES	NO	YES	2250; 2260_2; 5210	NO	NO
Klasea baetica subsp. baetica	NO	NO	NO	YES	NA	NA	NA	NA	NO	YES	YES	5330_6	YES	YES
Linaria saturejoides	NO	NO	YES	NO	NA	NA	NA	NA	YES	NO	YES	8130	YES	NO
Macrochloa tenacissima	YES	NO	YES	NO	NO	NO	NA	NA	YES	NO	YES	6220_1	NO	NO
Myrtus communis	NO	NO	YES	NO	NA	NA	YES	NO	YES	NO	YES	5330_2	NO	NO
Olea europaea var. sylvestris	NO	NO	NA	NA	NA	NA	YES	NO	YES	NO	YES	9320_0	NO	NO
Phillyrea angustifolia	YES	NO	YES	NO	NA	NA	NO	NO	YES	NO	YES	5330_1	NO	NO
Phlomis purpurea	YES	NO	YES	NO	NA	NA	YES	YES	YES	YES	YES	5330_5	NO	NO
Pinus pinaster	NA	NA	NO	NO	NA	NA	NA	NA	NO	NO	NO	9540	NO	NO
Pistacia lentiscus	NO	NO	YES	NO	NA	NA	NO	NO	YES	NO	YES	5330_2	NO	NO
Quercus coccifera	NA	NA	YES	NO	NA	NA	YES	NO	YES	NO	YES	5330_2	NO	NO
Quercus faginea	NA	NA	YES	NO	NA	NA	YES	NO	YES	NO	YES	9240_0	NO	NO
Quercus rotundifolia	NO	NO	YES	NO	NA	NA	YES	NO	YES	NO	YES	9340	NO	NO
Quercus suber	NA	NA	NO	NO	NA	NA	NO	NO	NO	NO	NO	9330	NO	NO
Retama sphaerocarpa	NO	NO	YES	NO	NA	NA	NO	NO	YES	NO	YES	5330_3	NO	NO
Rhamnus alaternus	YES	NO	YES	NO	NO	NO	YES	YES	YES	YES	YES	5330_2	NO	NO
Rubia peregrina	NO	NO	YES	NO	NA	NA	NO	NO	YES	NO	YES	5330_1	NO	NO
Salvia candelabrum	NO	NO	NO	NO	NO	NO	NA	NA	NO	NO	NO	5330_2	YES	NO
Salvia lavandulifolia	NO	NO	NO	NO	NA	NA	NO	NO	NO	NO	NO	4090_1	NO	NO
Salvia rosmarinus	NO	NO	NO	NO	NA	NA	NO	NO	NO	NO	NO	5330_5	NO	NO
Staehelina baetica	NO	NO	YES	NO	NA	NA	NA	NA	YES	NO	YES	5330_6	YES	YES
Teline linifolia	NA	NA	NO	NO	YES	NO	NO	NO	YES	NO	YES	5330_1	NO	NO
Thymbra capitata	YES	NO	YES	NO	NA	NA	YES	NO	YES	NO	YES	5330_5	NO	NO
Ulex parviflorus	YES	NO	YES	NO	NA	NA	YES	NO	YES	NO	YES	5330_4	NO	NO
Viburnum tinus	NO	NO	NO	NO	NA	NA	YES	NO	YES	NO	YES	5330_1	NO	NO

References

Raunkiær C. 1934. *The life forms of plants and statistical plant geography.* London: Oxford University Press.

REDIAM. 2020. *Guía de Identificación de Hábitats de Interés Comunitario en Andalucía.* Consejería de Agricultura, Ganadería, Pesca y Desarrollo Sostenible, Junta de Andalucía.
